# Distinct Metabolic Signals Underlie Clone by Environment Interplay in “Nebbiolo” Grapes Over Ripening

**DOI:** 10.3389/fpls.2019.01575

**Published:** 2019-12-04

**Authors:** Chiara Pagliarani, Paolo Boccacci, Walter Chitarra, Emanuela Cosentino, Marco Sandri, Irene Perrone, Alessia Mori, Danila Cuozzo, Luca Nerva, Marzia Rossato, Paola Zuccolotto, Mario Pezzotti, Massimo Delledonne, Franco Mannini, Ivana Gribaudo, Giorgio Gambino

**Affiliations:** ^1^Institute for Sustainable Plant Protection, National Research Council (IPSP-CNR), Torino, Italy; ^2^Council for Agricultural Research and Economics, Centre of Viticultural and Enology Research (CREA-VE), Conegliano, Italy; ^3^Department of Biotechnology, University of Verona, Verona, Italy; ^4^DMS StatLab, University of Brescia, Brescia, Italy; ^5^Department of Agricultural, Forest and Food Sciences, University of Torino, Grugliasco, Italy; ^6^Big&Open Data Innovation Laboratory, University of Brescia, Brescia, Italy

**Keywords:** candidate gene expression, clones, RNA-seq, secondary metabolism, sugar signalling, *Vitis vinifera*

## Abstract

Several research studies were focused to understand how grapevine cultivars respond to environment; nevertheless, the biological mechanisms tuning this phenomenon need to be further deepened. Particularly, the molecular processes underlying the interplay between clones of the same cultivar and environment were poorly investigated. To address this issue, we analyzed the transcriptome of berries from three "Nebbiolo" clones grown in different vineyards, during two ripening seasons. RNA-sequencing data were implemented with analyses of candidate genes, secondary metabolites, and agronomical parameters. This multidisciplinary approach helped to dissect the complexity of clone × environment interactions, by identifying the molecular responses controlled by genotype, vineyard, phenological phase, or a combination of these factors. Transcripts associated to sugar signalling, anthocyanin biosynthesis, and transport were differently modulated among clones, according to changes in berry agronomical features. Conversely, genes involved in defense response, such as stilbene synthase genes, were significantly affected by vineyard, consistently with stilbenoid accumulation. Thus, besides at the cultivar level, clone-specific molecular responses also contribute to shape the agronomic features of grapes in different environments. This reveals a further level of complexity in the regulation of genotype × environment interactions that has to be considered for orienting viticultural practices aimed at enhancing the quality of grape productions.

## Introduction

Recent climate changes have seriously been challenging agricultural ecosystems across the planet by causing dramatic alterations on the environment where crops are grown (Rosenzweig et al., 2014; Wolkovich et al., 2018). These events negatively impact quality traits of fruit crops, such as wine grape cultivars, that are overall more appreciated for their unique secondary metabolites rather than for high quantitative yields (Ferrandino and Lovisolo, 2014). *Vitis vinifera* is one of the most sensitive species to environmental variations and it shows a broad array of adaptation strategies (Ahuja et al., 2010; Lovisolo et al., 2016), often relying on the genotype of the cultivar and/or rootstock used (Ollat et al., 2015; Chitarra et al., 2017). Another aspect deserving attention is that the majority of grape varieties are characterized by high phenotypic plasticity, a property associated to the capacity of a genotype to respond to different ambient conditions by shaping and/or changing its phenotypical traits (Sultan, 2010). Phenotypic plasticity plays a crucial role in modulating the physiological performances of cultivars across different growing regions, and in determining the agronomic features associated to berry quality. Indeed, the primary and secondary metabolisms governing the final composition of grapes strictly depend on the dynamic cross-talk between vines and surrounding environment (Dai et al., 2011; Hannah et al., 2013; Teixeira et al., 2013; van Leeuwen et al., 2013a; Anesi et al., 2015).

In the last years, more and more researches were addressed to investigate factors controlling berry development and quality (Kuhn et al., 2014). A large set of biochemical and physiological changes take place throughout the distinct phases typically characterizing berry ripening (Coombe and McCarthy, 2000; Conde et al., 2007). Mounting evidence indicates that the progression of berry development is driven by a network of regulatory signals associated to transcriptional reprogramming events (Zenoni et al., 2010; Fasoli et al., 2012), extremely sensitive to external factors (Kuhn et al., 2014; Serrano et al., 2017). In order to optimize sustainability of viticulture production, it is of great interest to deepen how grapevine genotypes and environmental cues interact each other, by identifying the biological processes that most affect resiliency mechanisms and quality yields of grapes (Dûchene et al., 2010). Many studies have explored how grapevine cultivars respond to different growing and climate conditions (Anesi et al., 2015; Ghan et al., 2015; Young et al., 2016; Massonnet et al., 2017; Dal Santo et al., 2018), thus disclosing the high molecular complexity of genotype × environment interactions (Dal Santo et al., 2013; Dal Santo et al., 2016). Nevertheless, other efforts are needed to boost our understanding of how plants can reprogram themselves to survive in a context of high environmental variability (Ahuja et al., 2010).

Existing literature only takes into account responses of either different cultivars within the same growing areas (Degu et al., 2014; Massonnet et al., 2017) or single clone/cultivars among multiple viticultural sites (Cramer et al., 2014; Dal Santo et al., 2016; Paim Pinto et al., 2016). With the exception of works concerning the analysis of clonal variability in terms of berry composition and wine quality (Ferrandino and Guidoni, 2010; van Leeuwen et al., 2013b; Pantelić et al., 2016), little is known on molecular processes tuning the interplay between different clones (vegetatively propagated lines of selected mother plants) of the same cultivar and environment. Further information on this topic would be crucial to decipher plastic responses mediated by epigenetic modifications (Zhang et al., 2013) in turn affecting gene expression (Eichten et al., 2014). Those molecular events are of particular relevance in clonal populations, in which they constitute a strategy of environmental adaptation alternative to genetic recombination (Verhoeven and Preite, 2014; Dodd and Douhovnikoff, 2016). However, the study of epigenetic phenomena underlying the interplay between long-living plants, such as grapevine, and environment is still at the beginning (Fortes and Gallusci, 2017). The analysis of epigenetic variations in *V. vinifera* was applied to the identification of commercial clones (Ocaña et al., 2013) or to the assessment of somaclonal variability induced by *in vitro* propagation (Baránek et al., 2015), but only a few works discussed the relationship between environmental signals and epigenetic regulation (Fabres et al., 2017; Fortes and Gallusci, 2017). First evidence suggests that differences among genotypes, due to distinct DNA methylation patterns (Dal Santo et al., 2018) or small RNA-mediated regulatory cascades (Paim Pinto et al., 2016), can influence transcriptomic plasticity of grapevine cultivars thus contributing to the definition of *terroir* (Xie et al., 2017).

In parallel, other authors have demonstrated that genomic variants (i.e., single-nucleotide variants, small insertions and deletions, inter- and intra-chromosomal translocations and inversions, private genes) associated to clones can contribute to intra-specific variability in grapevine (Carrier et al., 2012; Da Silva et al., 2013; Carbonell-Bejerano et al., 2017; Gambino et al., 2017). In particular, *V. vinifera* cv Nebbiolo, one of the most ancient and prestigious wine grape varieties (Robinson et al., 2012), shows great intra-varietal phenotypic polymorphism (Schneider et al., 2003) associated with genomic differences, as unveiled by a whole genome sequencing analysis conducted on three "Nebbiolo" clones: CVT71, CVT423, CVT185 (Gambino et al., 2017).

Based on the above, it is worth to explore whether the molecular differences existing among clones of the same cultivar may play a role in events of environmental adaptation involving tissue-specific changes in turn affecting fruit quality.

To elucidate this point, we analyzed the molecular and metabolic variability of berries of the three sequenced "Nebbiolo" clones collected at three ripening stages, over two vegetative seasons, in three vineyards of the Langhe territory (Piedmont Region, North-West Italy), a worldwide renowned Italian viticultural site (http://whc.unesco.org/en/list/1390). In order to dissect the whole complexity of the clone × environment (*C x E*) interaction and to identify the hub molecular changes controlled by clone, vineyard, phenological phase, or a combination of them, a multidisciplinary approach was followed. To this aim, transcriptomic data resulting from high throughput sequencing were integrated by: i) analysis of candidate gene expression by real-time PCR; ii) quantification of key secondary metabolite contents; iii) assessment of agronomical parameters; and iv) monitoring of climatic conditions in each growing site during the two consecutive years.

## Material and Methods

### Experimental Field Sites and Plant Material

**Figure 1:**
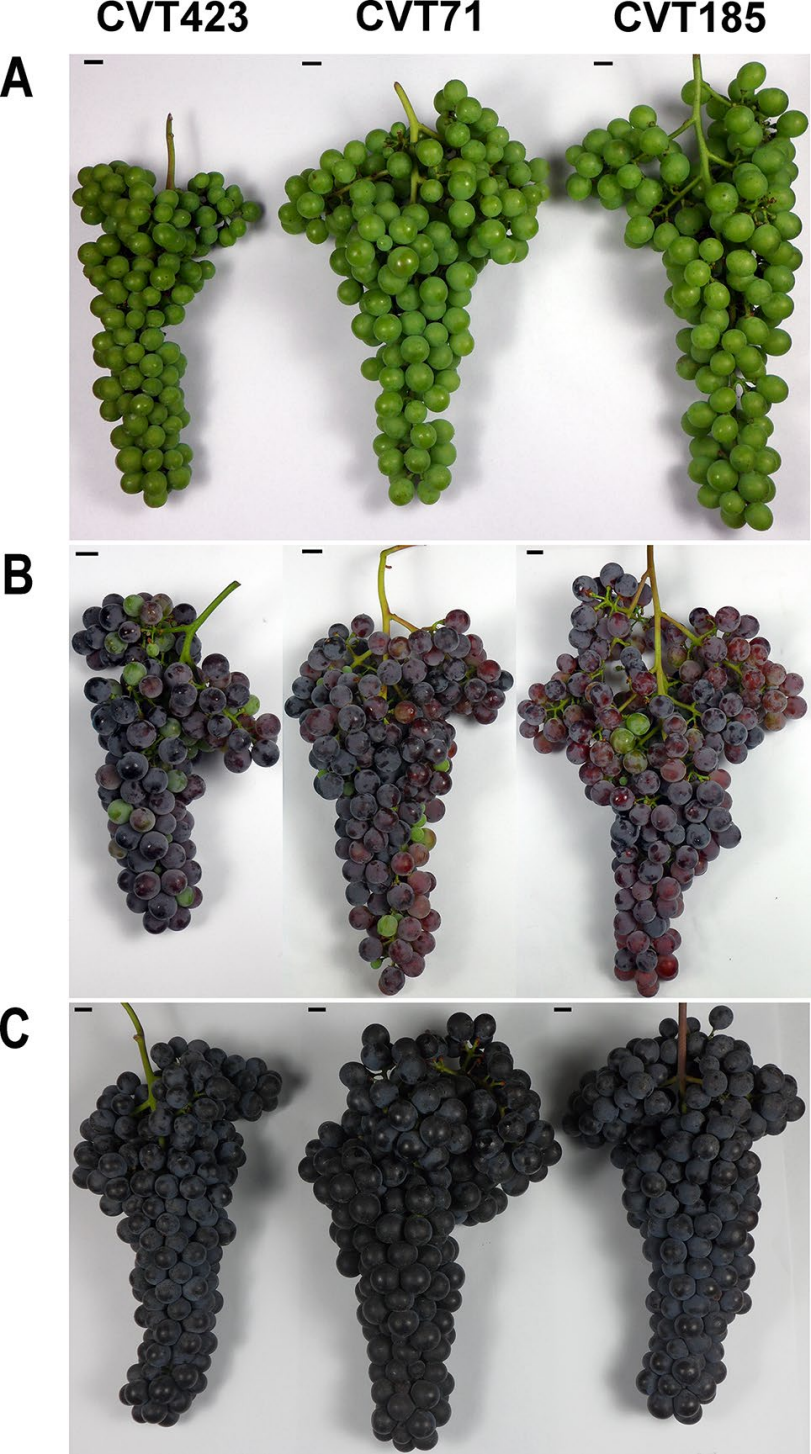
Phenotypic details of the grapes from CVT423, CVT71, and CVT185 "Nebbiolo" clones around pre-véraison **(A)**, véraison **(B)**, and harvest **(C)** time.

Trials were conducted in two consecutive vegetative seasons (2013 and 2014) on plants belonging to three *V. vinifera* "Nebbiolo" clones (CVT71, CVT185, CVT423, **Figure 1** and **Supplementary Table S1**). The grapevines, all grafted onto the Kober 5BB rootstock, were grown in three different vineyards (V1, V2, V3; **Supplementary Table S2**) planted between the years 1999 (V1, V2) and 2000 (V3) at an average density of about 4,500 plants ha^-1^. In all vineyards, the vines were vertically trained and Guyot pruned (11 buds per plant), and planted at a spacing of 0.9 m (within the row) × 2.4 m (between rows) in parallel and contiguous rows.

Before carrying out the experiments, virus detection was conducted by serological (ELISA) and molecular (multiplex RT-PCR, as described by Gambino, 2015) analyses on hundreds of plants in each vineyard. Further tests of molecular diagnosis were performed to detect the phytoplasma-induced diseases *Flavescence Dorée* and *Bois Noir,* according to Marzachì et al. (1999). Plants having a homogeneous virological status (i.e., only infected by the grapevine rupestris stem-pitting associated virus) and free from phytoplasma infection were used.

### Assessment of Agronomical Parameters and Sampling Procedures

The following parameters were monitored over the two vegetative seasons on a total of 21 vines per clone in each vineyard: pruning wood weight, bud-burst index, shoot fertility (number of inflorescences/shoot) at spring, cluster number and weight, yield (kg of grapes per plant) at harvest, and juice composition [total soluble solids (TSS), pH, titratable acidity (TA), content of malic and tartaric acid]. Technological ripening parameters, such as TSS (°Brix), pH, and TA (g L^-1^ tartaric acid), were measured on the musts at the moment of commercial harvest in each vineyard according to the methods proposed by the International Organization of Vine and Wine (O.I.V.-http://oiv.int/en/technical-standards-and-documents/methods-of-analysis). Bulk soil samples were collected after harvesting the grapes at a depth of 30–40 cm under the vine canopy. To avoid differences in soil composition, anomalous areas in terms of appearance were excluded from the sampling procedure as well as those 5 m from the vineyard edges or close to the end of the row. Analyses of soil samples were performed at an external laboratory service. Both collection of soil samples and related chemical analyses were carried out according to official methods as described in the Italian Ministerial Decree D.M 13/09/1999 (https://www.gazzettaufficiale.it/eli/id/1999/10/21/099A8497/sg). Climatic data representative of each vineyard were also collected over the 2 years (**Supplementary Figure S1**).

Samples for molecular (RNAseq and real-time PCR) and metabolic (flavonoid, hormone, and stilbenoid quantification) analyses (see below) were taken from three randomized field plots, each constituted by seven vines per clone, at three stages during ripening: E-L31, E-L35, and E-L38, respectively, referring to berry pea size, véraison, and harvest (TSS values around 24° BRIX), as defined by the modified Eichhorn and Lorenz (E-L) system (Coombe, 1995). The three developmental phases correspond to stages 75, 81, and 89 of the extended BBCH scale by Lorenz et al. (1995). Berries were sampled from the upper, middle, and distal part of the bunch, alternatively from the shaded and from the exposed side of the cluster and from each side of the row, to avoid problems of sun exposition and to guarantee a good repeatability of the measurements. Three biological replicates, each of 200 berries, were collected for each clone in each vineyard at each stage. Fifty berries were randomly chosen among each replicate of 200 berries, frozen in liquid nitrogen and kept at -80°C for molecular, abscisic acid, and stilbenoid analyses (see below). Other 30 berries were randomly taken from each 200 berry-replicate and used for flavonoid quantification and anthocyanin profiling (see below).

### RNA Extraction, Library Preparation, and Sequencing

Total RNA was extracted using the Spectrum™ Plant Total RNA extraction kit (Sigma Aldrich) starting from 100 mg of deseeded berries, and total RNA yield was determined using a NanoDrop spectrophotometer (Thermo Fisher Scientific). Integrity of RNA samples was assessed on an RNA 6000 Nano Labchip using a Bioanalyzer 1000 (Agilent Technologies, Santa Clara, USA) prior to library preparation. Only samples showing a RIN (RNA Integrity Number) value higher than 8 were submitted to sequencing and quantitative expression analyses. The cDNA libraries (81 libraries in total, **Supplementary Table S3**) were prepared from samples collected in 2013 using TruSeq RNA Sample Prep Kit v2 (Illumina, San Diego, USA), starting from 2.5 µg of total RNA and following the manufacturer’s instructions. Libraries were sequenced with an Illumina HiSeq 1000 sequencer (Illumina Inc., San Diego, CA, USA) generating ∼23 million 100 bp single-end reads per sample (**Supplementary Table S3**). Low-quality reads (> 50 bases with quality < 7 or > 10% undetermined bases) were removed and Illumina TruSeq adapter sequences were clipped.

### Elaboration of Sequencing Data

Sequenced reads were aligned against the grape reference genome (PN40024; Jaillon et al., 2007) (V1 12X draft, http://genomes.cribi.unipd.it/) using TopHat v.2.0.14, and an average of 82.3% reads per sample were mapped (**Supplementary Table S3**).

The remaining unmapped reads were grouped for each vineyard and used to detect presence of pathogen sequences (**Supplementary Table S4**). In brief, a subset of 1 million reads were randomly selected from the unmapped reads of each rna-seq sample, thus obtaining for each vineyard a subset of 27 million reads. For each vineyard, the unmapped reads were aligned with HISAT2 (https://ccb.jhu.edu/software/hisat2; Kim et al., 2015) against the reference genomes of the three main fungal pathogens affecting European grapevine (*Botrytis Cinerea*, Van Kan et al., 2017; *Erysiphe Necator*, Jones et al., 2014; *Plasmopara Viticola*, Brilli et al., 2018). In parallel, subsets of unmapped reads were also blasted against the NCBI NR database using diamond aligner (http://ab.inf.uni-tuebingen.de/software/diamond; Buchfink et al., 2015), and the BLASTN outputs were subjected to a taxonomic distribution analysis by means of MEGAN4 (https://uni-tuebingen.de/fakultaeten/mathematisch-naturwissenschaftliche-fakultaet/fachbereiche/informatik/lehrstuehle/algorithms-in-bioinformatics/software/megan6/; Huson et al., 2011).

The normalized averaged expression of each transcript was calculated for each triplicate using Cufflinks v.2.2.0 as FPKM (fragment per kilobase of mapped reads). Transcripts showing no expression (FPKM = 0) overall were discarded as well as those with FPKM > 0 but randomly expressed in only one biological replicate per thesis. After this filtering step, 22,280 expressed transcripts were obtained in total. All transcripts were grouped into functional gene categories (**Table S5**) according to the GO biological process classification and to the VitisNet GO annotations (Grimplet et al., 2009).

A dedicated data mining process, detailed in Dal Santo et al. (2018), was applied to uncover associations between transcript variability and the three variables, the growing site (referred to as vineyard), clone, and ripening stage. After a data-reducing step aimed at trimming genes not associated with the analyzed issues, hierarchical clustering was performed using the R software, and 112 gene clusters were built, accounting for about 80% of the total variance (**Supplementary Table S6**). Then, average cluster profiles were summarized by principal component analysis (PCA). The average expression of the genes belonging to each cluster was used as representative profile for the cluster itself. An index of representativeness, based on the variability of the gene expressions around the profile, was computed in order to measure the degree of representativeness of the average profile. Finally, variable importance measures (VIMs) were calculated by means of a machine learning technique in the class of ensemble learning algorithms, called gradient boosting machine, and the weight of each variable in affecting transcript expression was quantified. To further characterize cluster profiles, VIM values were analyzed with respect to the principal components obtained in the former step. PCA was also applied to average gene expression profiles within each functional gene category, by using the Past software (PAST v.3.20; http://folk.uio.no/ohammer/past). Heat maps of transcriptional profiles associated with specific functional categories were generated with TMeV 4.9 (http://mev.tm4.org), using as input the average expression value (FPKM) of the three biological replicates.

### Real-Time PCR Analysis

Expression changes of target transcripts were profiled by quantitative real-time PCR (RT-qPCR), as reported in Chitarra et al. (2017). Besides helpful for validating RNA-seq results (**Supplementary Figure S2**), this analysis allowed to deepen the influence exerted by the studied factors in regulating specific groups of transcripts.

Both RNA samples from the berries taken in 2013, also used for sequencing process, and RNA samples from the berries taken in 2014 were analyzed. Briefly, RNA samples were treated with DNase (DNase I, Invitrogen; Thermo Fisher Scientific) and first-strand cDNA was synthesized using the High Capacity cDNA Reverse Transcription Kit (Applied Biosystems; Thermo Fisher Scientific). Real-time PCR assays were performed in a CFX Connect Real-Time PCR system (Bio-Rad Laboratories, Hercules, CA, USA), using the SYBR Green (iQTM SYBR Green Supermix; Bio-Rad Laboratories, Hercules, CA, USA) method for quantifying amplification results. Thermal cycling conditions were as follows: an initial denaturation phase at 95°C for 2 min, followed by 40 cycles at 95°C for 15 s and 60°C for 30 s. Specific annealing of primers was inspected on dissociation kinetics performed at the end of each PCR run. Identity of amplicons was checked by sequencing when needed. Transcript expression levels were quantified after normalization to two endogenous genes, *Ubiquitin* (*VvUBI*) and *Actin1* (*VvACT1*), used as internal controls. Gene-specific primers are listed in **Supplementary Table S7**. Three independent biological replicates and three technical replicates were run for each RT-qPCR experiment. Significant differences among samples were analyzed by one-way ANOVA test, using the Tukey’s *HSD post hoc* test for separating means when ANOVA results were significant (*P < 0.05*). Significant differences of pairwise comparisons were assessed by the Student’s *t*-test.

The SPSS statistical software package (SPSS Inc., Cary, NC, USA, v.22) and the GraphPad Prism software (GraphPad Software, La Jolla, CA, USA v.6.01) were used to run the statistical analyses above reported and elaborate figure charts, respectively.

### Analysis of Secondary Metabolites

Concentrations of total flavonoids, total anthocyanins, and anthocyanin profiles were determined on three biological replicates (obtained from the pool of 200 berries per clone) of 30-berry skin each that were further divided into 3 groups of 10 berries used as technical replicates. For each 10-berry sample, the skins were manually separated from pulp and seeds, then immersed in a pH 3.2 ethanol buffer (120 mL L^-1^ ethanol, 5 g L^-1^ tartaric acid, 2 g L^-1^ Na_2_S_2_O_5_, 22 mL L^-1^ NaOH 1 N) and incubated at 30°C for 72 h to allow extraction of skin phenolic compounds, as reported by Ferrandino and Guidoni (2010).

Contents of total flavonoids and total anthocyanins were determined on the grape skin extracts by spectrophotometry, reading the absorbance respectively at 280 and 520 nm, as described in Torchio et al. (2010).

The anthocyanin extractability yield (%) was estimated on a further sample of 50 berries per each clone, randomly selected from the pool of 200 berries (see above). After extraction in the same pH 3.2 buffer, skins were homogenized by means of an Ultraturrax system (IKA Labortechnik, Staufen, Germany) and centrifuged for 5 min at 3,000 g at 20°C. The total content of anthocyanins determined by spectrophotometry on the obtained supernatants was used to evaluate the rate of skin anthocyanin extractability during maceration, as detailed in Rolle et al. (2012).

After a concentration step onto Sep-Pak C18 cartridges (Waters Corporation, Milford, MA, USA) to separate anthocyanins from other polyphenols and sugar contaminants, the berry skin extracts were eluted with methanol and analyzed by liquid chromatography to determine anthocyanin profiles, according to a previously published method (Ferrandino et al., 2017).

Contents of abscisic acid, *trans-*resveratrol, and *ε*-viniferin were quantified by liquid chromatography on three biological replicates per clone starting from 500 mg of powdered berry skins, according to the procedure described in Chitarra et al. (2017).

## Results and Discussion

### Overview of the Hub Molecular Changes Underlying the C x E Interplay

**Figure 2:**
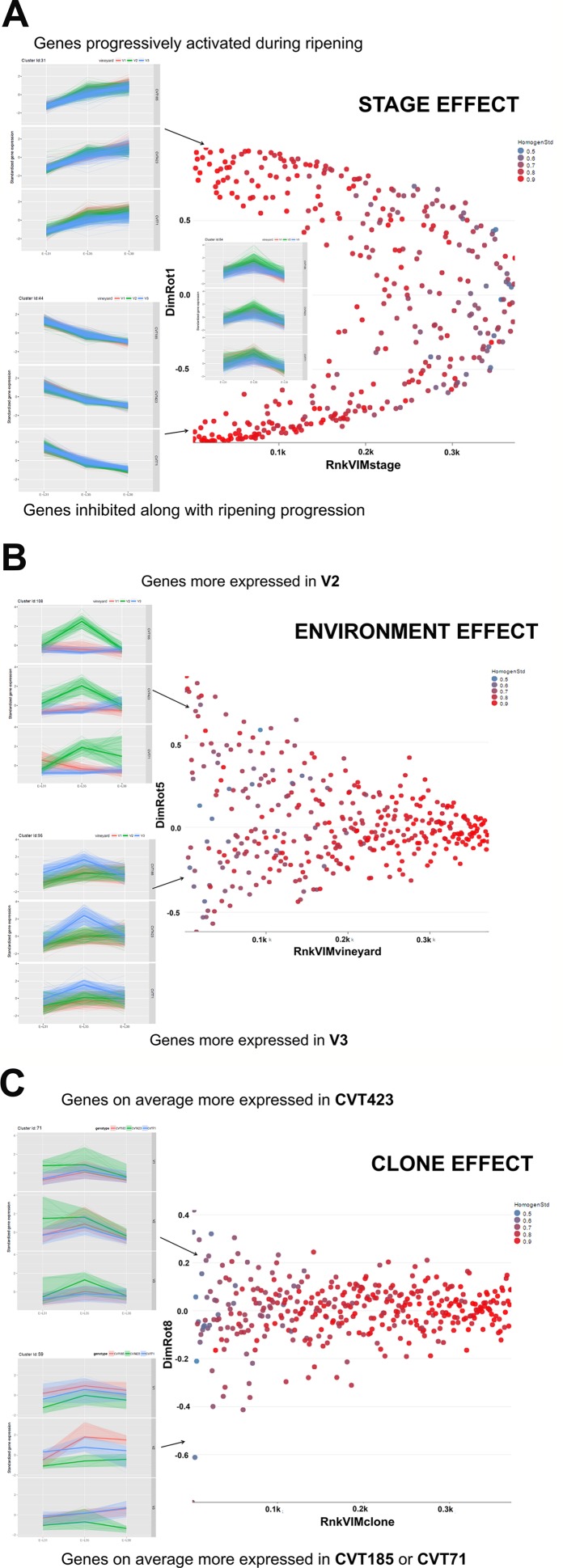
Schematic illustration of the effect exerted by **(A)** Stage, **(B)** Environment, or **(C)** Clone on gene cluster expression. Scatterplots of the 112 clusters were displayed according to the rank in **(A)** VIM Stage, **(B)** VIM Vineyard, and **(C)** VIM Clone (e.g., Rnk_VIM_Stage = rank of clusters according to VIM Stage; low values denote high importance of the stage) and to the loading in the specific rotated principal component (DimRot) (first, fifth, and eighth components for stage, vineyard and clone, respectively). Each dot represents a single cluster. Dots were colored according to the cluster homogeneity index reported on the right at the top of each scatter plot. Examples of clusters are provided within each scatter plot. A complete list of the 112 clusters is reported in **Table S6**.

We first addressed our survey to investigate the whole transcriptome reprogramming events occurring during the first year of trial and associated with the response of "Nebbiolo" clones to either different environments or ripening phase. To this aim, a dedicated data mining statistical approach (Dal Santo et al., 2018) was applied to the elaboration of RNA-seq analysis. This allowed us to provide a comprehensive overview of the variation of the main functional gene classes based on either stage, environment, or clone effect (**Figure 2**).

### Stage Effect

There were classes of genes whose activation or inhibition at a specific phase induced metabolic changes essential for fruit ripening (**Figure 2A**). Transcripts falling into 40 clusters, corresponding approximately to the 50% of the analyzed genes, were significantly modulated according to the phenological phase, independently of clone or environment (**Table S6**). Stage-specific clusters including transcripts related to photosynthesis were progressively down-regulated over the season (e.g., Clusters Id 8, 21, 23, 44; **Supplementary Table S6**). Conversely, at véraison (E-L35) and harvest (E-L38), clusters grouping genes encoding transcription factors and enzymes related to cell cycle and homeostasis, transport, response to endogenous stimulus and carbohydrate metabolism were activated (e.g.,Clusters Id 2, 9, 11, 31, 99; **Supplementary Table S6**). Consistently with studies on other red-grape varieties (Dal Santo et al., 2018), stage-specific modulation of these functional gene categories was of particular interest for investigating the interaction with the biological processes involving the accumulation of compounds (i.e., anthocyanins) that act as markers of ripening evolution (Kuhn et al., 2014; Lecourieux et al., 2014). Although the analysis of stage effect on "Nebbiolo" berry transcriptome revealed transcriptional changes already documented in other cultivars (Deluc et al., 2007; Dal Santo et al., 2013; Castellarin et al., 2016; Dal Santo et al., 2018), these data evidence the effectiveness of the adopted statistical approach, thus making the following considerations on the effects exerted by environment and clone more robust and reliable.

### Environmental Effect

**Figure 3:**
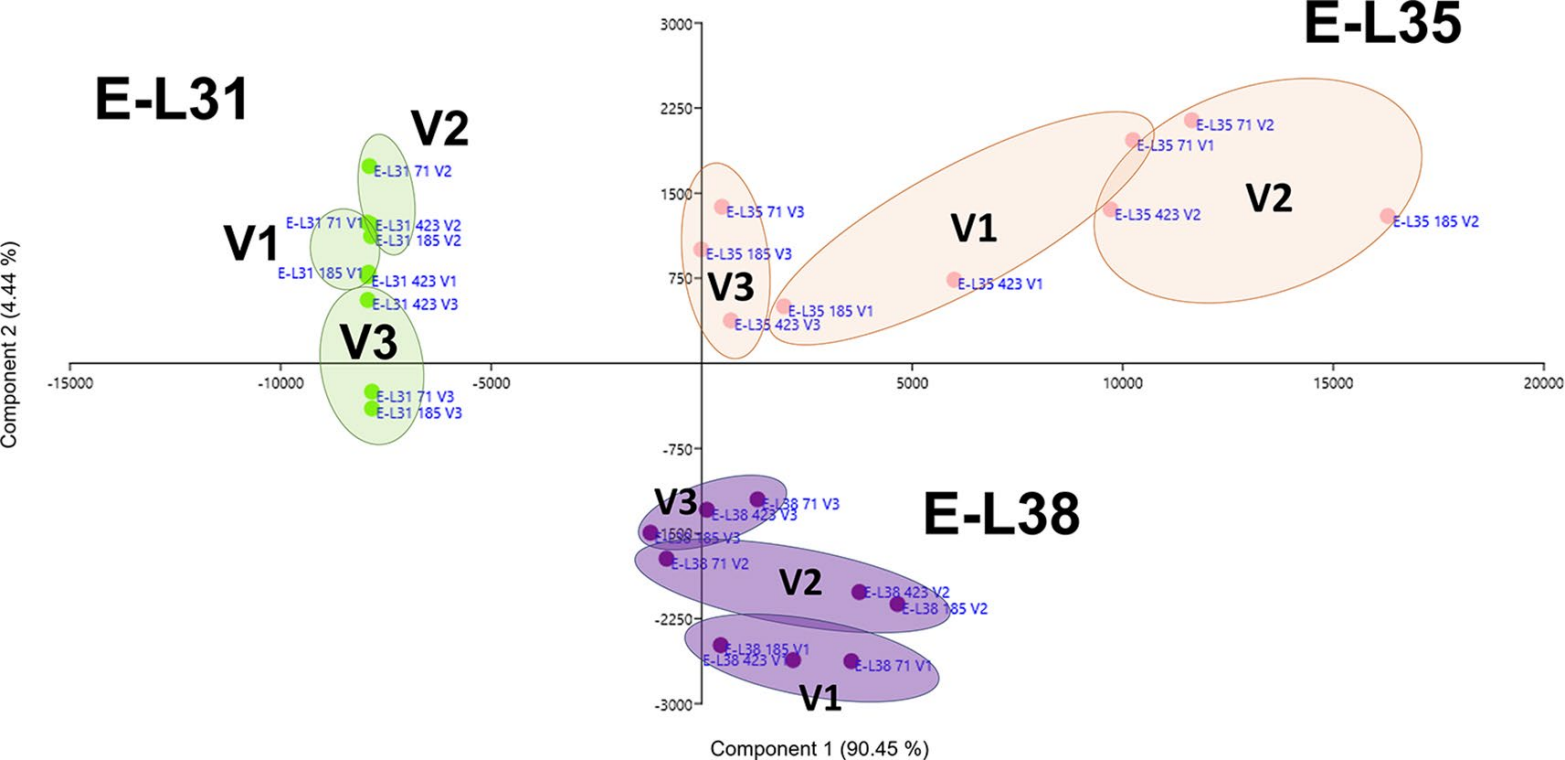
Environmental effect on carbohydrate metabolism. Schematic representation highlighting how the three considered variables, ripening stage (E-L31, E-L35, E-L38), clone (CVT71, CVT423, CVT185), or vineyard (V1, V2, V3), affect the distribution of samples when genes belonging to Carbohydrate metabolic process were analyzed by principal component analysis (PCA). Conversion between the modified E-L scheme and the extended BBCH scheme is as it follows, E-L31 = 75 BBCH; E-L35 = 81 BBCH; E-L38 = 89 BBCH.

**Figure 4:**
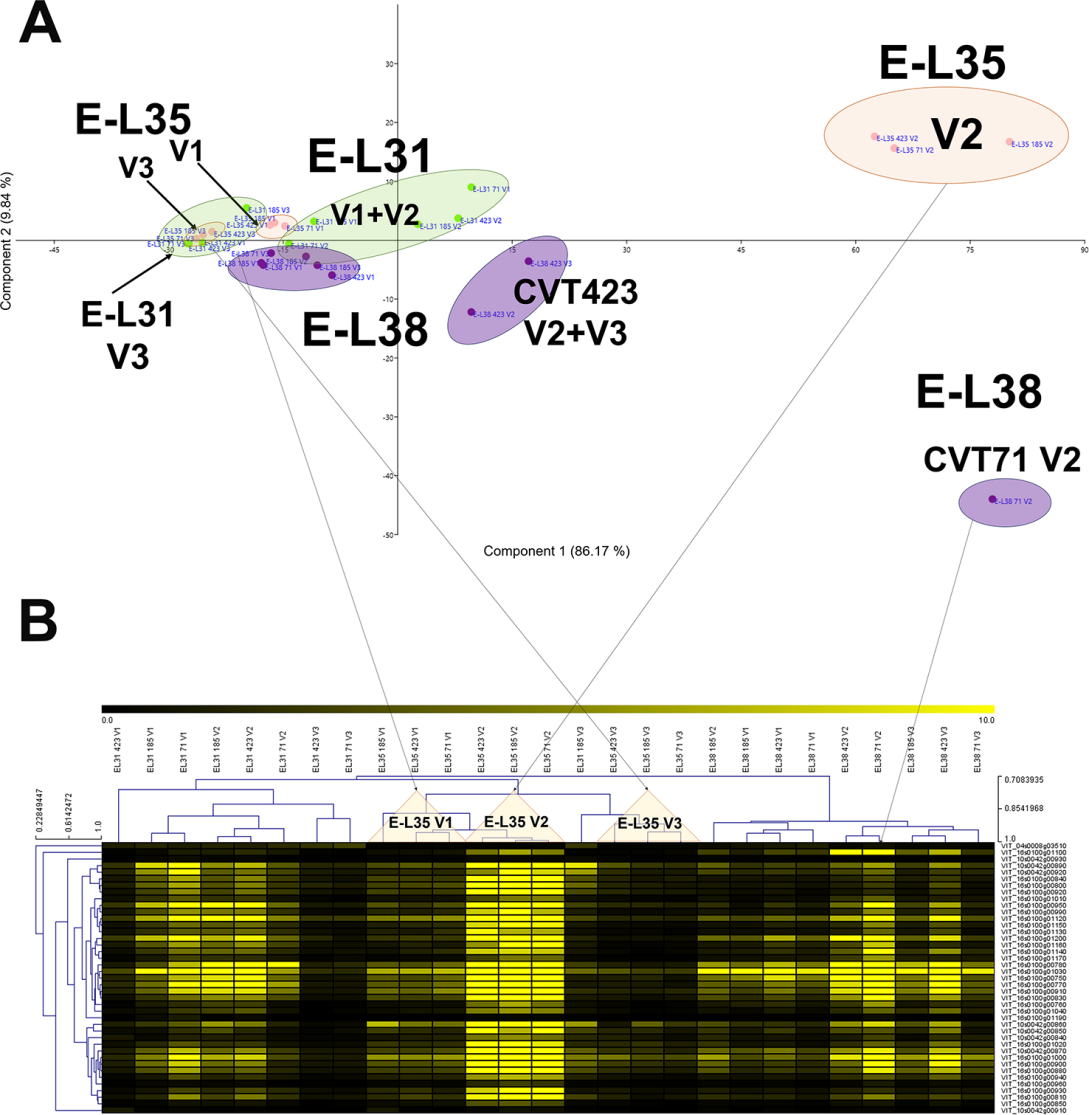
Environmental effect on stilbenoid metabolism. **(A)** PCA and **(B)** MeV heat map of transcripts belonging to the stilbene synthase (*STS*) gene category. Color scale of the heat map chart ranges from black (low expression) to yellow (high expression, FPKM > 10).

More than 20 gene clusters (accounting for about the 12% of all modulated genes) included transcripts significantly affected by the environment (**Figure 2B**). The transcriptional changes associated with primary and secondary metabolism, as well as with the plant stress response, were strongly dependent on the environmental variability, further confirming grapevine as an environmentally sensitive crop (Dai et al., 2011; Dal Santo et al., 2018). Despite transcripts of carbohydrate metabolism fell into clusters mainly controlled by developmental transitions, a "vineyard-specific" distribution was noticed at each stage (**Figure 3**). Additionally, transcripts linked to plant defense, such as pathogenesis related protein genes (PRs) and NBS-LRR genes (clusters Id 63, 89; **Supplementary Table S6**), and production of secondary metabolites, like stilbene synthase genes (cluster Id 108; **Supplementary Table S6**), were all strongly induced in V2 (**Figure 4**). Coherently, V2 samples formed a separated cluster at E-L35 (**Figure 4A**), the same phenological phase at which *STS* genes were most activated (**Figure 4B**). Conversely, the influence of V3 (**Figure 2B**) was ascribed to expression changes related to cell growth and developmental processes (clusters Id 96, 109, 110; **Supplementary Table S6**), particularly at véraison. The environmental component was also important in controlling genes associated to lipid and organic acid metabolism, more evidently at E-L38 and E-L31, respectively (**Supplementary Figures S3A, B**) and hormone metabolism (**Supplementary Figure S3C**). Hormonal cascades are crucial players in berry development (Kuhn et al., 2014), and they are connected with changes in carbohydrate and secondary metabolism as well as with plant defense (Deluc et al., 2007; Ljung et al., 2015; Castellarin et al., 2016). It is conceivable that similar external conditions could affect those molecular networks in a synergistic way at specific developmental steps, thus contributing to adapt agronomic and physiological responses of the clones to different growing sites.

### sClone Effect

As the studied "Nebbiolo" clones are characterized by a few genetic variants (Gambino et al., 2017), accordingly, the genetic component had a lesser impact on transcript regulation than environment and stage (**Figure 2C**): about the 6% of the genes were interested by clone effect, as observed in 12 out of the 112 clusters. The "clone effect" played a role in the modulation of nucleic acid (**Supplementary Figure S4A**) and protein metabolic pathways (**Supplementary Figure S4B**). In the first case, a separation among clones was evident at the onset of ripening (E-L35; **Supplementary Figure S4A**); also for protein metabolism, a distinction among clones was noticed at E-L35 and E-L38, especially for CVT185 and CVT71 (**Supplementary Figure S4B**). Consistently, several genes involved in development and belonging to cluster Id 59 (**Supplementary Table S6**), such as heat shock proteins, ribosomal proteins, zing finger, and GATA-transcription factors and kinases, were more activated in berries from CVT185 and CVT71 than CVT423 (**Figure 2C**). This finding was in accordance with the smaller size and lower yields of CVT423 grapes compared with the other clones (**Table 1**).

Table 1Measurements of agronomical parameters, quantity and quality features of the studied ‘Nebbiolo’ clones (CVT71, CVT423 and CVT185) grown in three different vineyards of the Langhe area (V1, V2, V3) in 2013. Data are means ± standard error (n = 21). Results of ANOVA analysis are detailed below the table.

**Table:** 

Year 2013			CVT71								CVT423							CVT185						
			V1			V2			V3		V1			V2		V3		V1		V2			V3	
Budbreak^a^			4.16 ± 0.08			4.38 ± 0.07			4.55 ± 0.28		3.59 ± 0.12			4.02 ± 0.07		4.51 ± 0.08		4.03 ± 0.05		4.29 ± 0.16			4.84 ± 0.009	
Bud fertility (n° clusters per shoot)			0.98 ± 0.19			1.17 ± 0.08			0.98 ± 0.14		1.12 ± 0.11			1.29 ± 0.07		0.6 ± 0.06		1.03 ± 0.08		0.98 ± 0.09			0.88 ± 0.05	
Yield (kg per plant)			1.9 ± 0.27			1.4 ± 0.13			2.35 ± 0.23		1.54 ± 0.24			1.01 ± 0.08		0.96 ± 0.12		1.99 ± 0.23		1.19 ± 0.16			1.9 ± 0.22	
N° clusters per plant			7.4 ± 0.95			6.7 ± 0.45			6.6 ± 0.62		8 ± 0.90			8.4 ± 0.48		4.7 ± 0.36		8.7 ± 0.74		5.7 ± 0.58			7.1 ± 0.64	
Cluster weight (g)			253 ± 11.79			204 ± 11.29			351 ± 17.89		179 ± 23.35			121 ± 5.58		194 ± 14.42		223 ± 10.71		192 ± 15.70			261 ± 13.08	
Total Soluble Solids (TSS, °Brix)			24 ± 0.18			26 ± 0.16			23.2 ± 0.71		24 ± 0.33			25.4 ± 0.20		25 ± 0.20		24 ± 0.24		25 ± 0.38			24.2 ± 0.46	
pH			2.98 ± 0.03			2.98 ± 0.01			2.95 ± 0.02		2.97 ± 0.01			2.96 ± 0.01		3.03 ± 0.03		2.94 ± 0.02		2.95 ± 0.02			2.97 ± 0.01	
Titratable acidity (TA, g L^-1^)			10.61 ± 0.16			9.48 ± 0.22			8.67 ± 0.23		10.31 ± 0.18			10.36 ± 0.31		8.12 ± 0.14		11.21 ± 0.60		10.85 ± 0.39			9.48 ± 0.11	
Tartaric acid (g L^-1^)			8.9 ± 0.14			9.1 ± 0.22			7.6 ± 0.13		8.5 ± 0.03			9.5 ± 0.13		8.1 ± 0.19		9.2 ± 0.33		9.3 ± 0.20			9.1 ± 0.27	
Malic acid (g L^-1^)			3.7 ± 0.18			2.9 ± 0.08			2.6 ± 0.33		3.5 ± 0.15			2.9 ± 0.32		1.8 ± 0.12		3.8 ± 0.27		3.7 ± 0.27			2.7 ± 0.16	
Total anthocyanins (mg kg^-1^)			749 ± 26.96			707 ± 5.49			657 ± 14.19		662 ± 27.83			669 ± 19.32		662 ± 14.14		795 ± 22.00		783 ± 27.02			787 ± 7.42	
Anthocyanin extractability (%)			61.53 ± 3.02			60.47 ± 3.09			78.78 ± 0.60		66.97 ± 0.34			63.53 ± 2.12		83.29 ± 1.06		74.89 ± 0.93		75.76 ± 0.82			80.85 ± 0.87	
Total flavonoids (mg kg^-1^)			2692 ± 74.5			2882 ± 30.9			3292 ± 29.8		2501 ± 74.1			2752 ± 37.4		3653 ± 270.3		3197 ± 34.5		3334 ± 50.6			4223 ± 24.0	
Pruning wood (kg per plant)			1.21 ± 0.15			0.94 ± 0.07			1.06 ± 0.09		0.69 ± 0.08			0.7 ± 0.04		0.72 ± 0.04		0.73 ± 0.09		1.1 ± 0.07			1.05 ± 0.07	
Ravaz index^b^ (RI, kg)			1.57 ± 0.08			1.49 ± 0.08			2.21 ± 0.16		2.23 ± 0.15			1.44 ± 0.05		1.33 ± 0.10		2.72 ± 0.11		1.08 ± 0.08			1.81 ± 0.09	

a Budbreak index was estimated based on a scale from 1 (dormant bud) to 6 (three unfolded young leaves) according to Baggiolini (1952).

b Yield/pruning weight per plant.

**Table:** 

Source of variation	Budbreak	Bud fertility		Yield	N° clusters/plant		Cluster weight	Total Soluble Solids		pH		Titratable acidity	Tartaric acid		Malic acid		Total anthocyanins		Anthocyanin extractability		Total flavonoids	Pruning wood		Ravaz index
Clone	**	NS		**	NS		**	NS		NS		**	**		**		**		NS		**	**		NS
Vineyard	**	**		**	**		**	**		NS		**	**		**		NS		NS		**	NS		***
Clone x Vineyard	NS	**		NS	**		NS	**		NS		NS	**		NS		NS		NS		NS	**		***

Significance of clone, vineyard, and interaction clone × vineyard effects was tested for P ≤ 0.05 (*), P ≤ 0.01 (**), and P ≤ 0.001 (***); NS, not significant.

These results provide molecular support for the intra-varietal variability previously observed at agronomic and physiological levels only (van Leeuwen et al., 2013b; Pantelić et al., 2016; Arrizabalaga et al., 2018), sustaining that phenomena of phenotypic plasticity can occur not only at the cultivar but also at the clone level.

### Clone × Environment Effect

**Figure 5:**
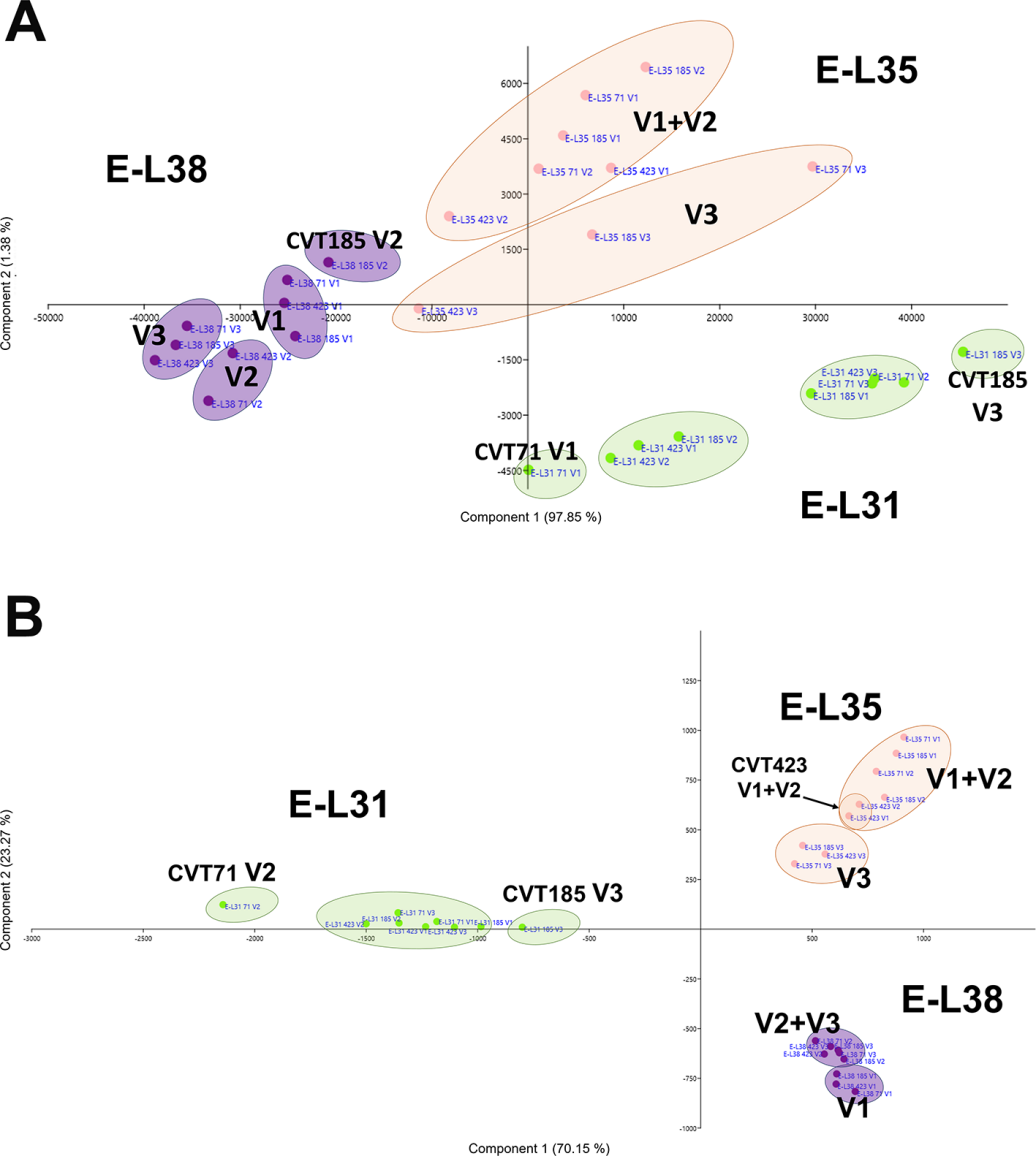
C*****x E*****effect on developmental processes. Schematic representation highlighting how the three considered variables, ripening stage (E-L31, E-L35, E-L38), clone (CVT71, CVT423, CVT185), or vineyard (V1, V2, V3), affect the distribution of samples when genes belonging to **(A)** development and **(B)** cell wall metabolism were analyzed by PCA. Conversion between the modified E-L scheme and the extended BBCH scheme, E-L31 = 75 BBCH; E-L35 = 81 BBCH; E-L38 = 89 BBCH.

**Figure 6:**
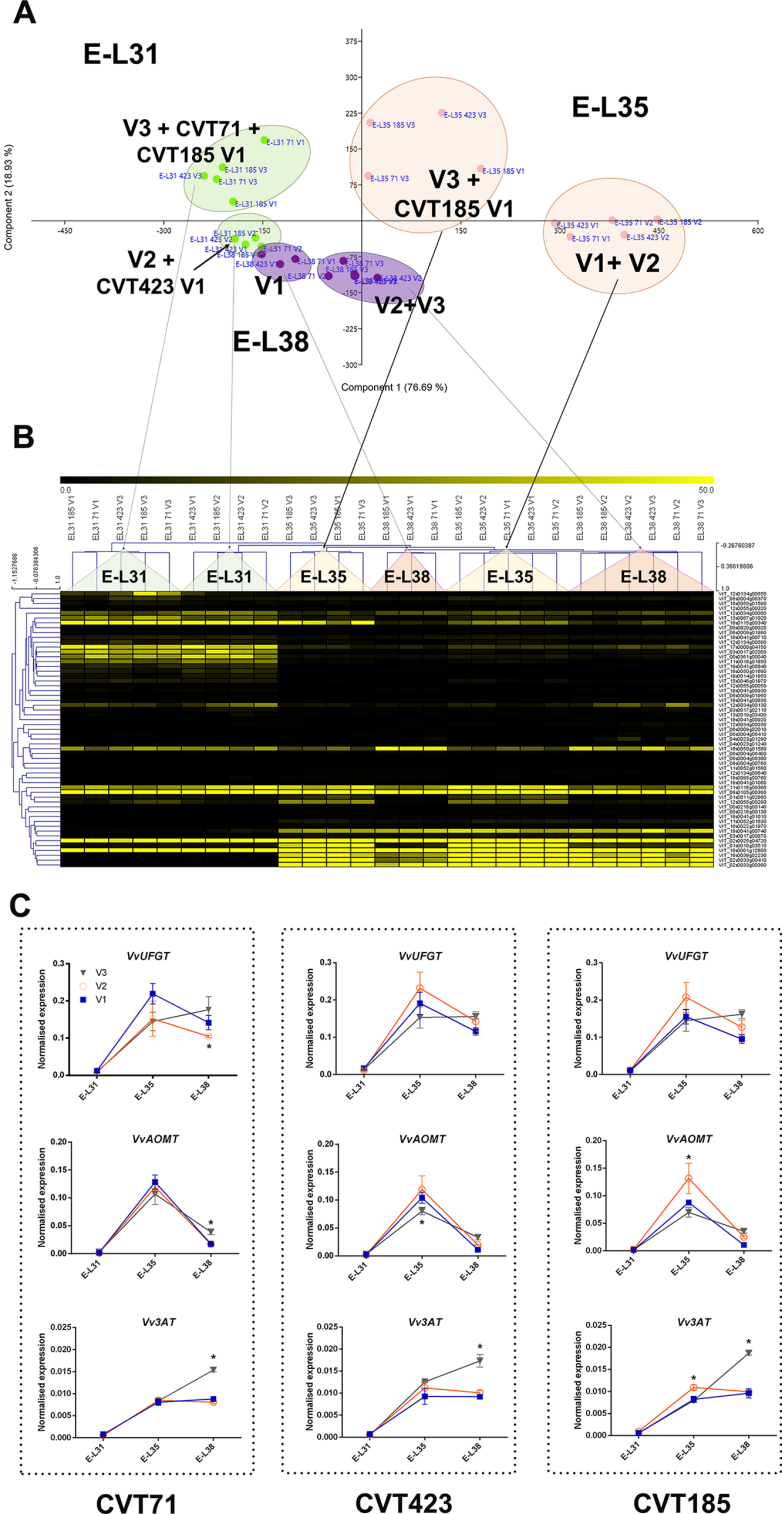
C *x* *E* effect on anthocyanin biosynthesis. **(A)** PCA and **(B)** heat map of transcripts belonging to the category of flavonoid biosynthesis. Color scale of the MeV heat map chart ranges from black (low expression) to yellow (high expression, FPKM > 50). **(C)** RT-qPCR expression profiles of genes encoding the main anthocyanin biosynthetic enzymes *Vv*UFGT (VIT_16s0039g02230), *Vv*AOMT (VIT_01s0010g03510) and *Vv*3AT (VIT_03s0017g00870) analyzed in whole berries collected in three vineyards (V1, V2, V3) from CVT71, CVT423, and CVT185 "Nebbiolo" clones at three ripening stages (E-L31, E-L35, E-L38) in 2013. Asterisk or lower case letters denote significant differences attested by Tukey’s *HSD* test (*P < 0.05*) respectively when values were significant in only one or more samples. Bars represent standard error of the mean (*n = 3*). The E-L31, E-L35, E-L38 stages from the modified E-L scheme respectively correspond to stage 75, 81 and 89 in the extended BBCH scheme.

There were 16 clusters of transcripts (approximately 11% of expressed genes) for which a combined effect of both environment and clone was observed. In the cell growth and development category, a separation of the samples based on the growing site was evident at E-L38 and at E-L35; however, a clone-based distinction was also noticed at E-L31, for CVT71 and CVT185, respectively, in V1 and V3 (**Figure 5A**). The majority of transcripts belonging to cell wall metabolism encoded cellulose synthases, beta-glucanases, polygalacturonases, esterases, and several xyloglucan endotransglucosylases (XET), enzymes all known to exert a role in berry development (Deluc et al., 2007; Grimplet et al., 2017). PCA distribution of this gene class highlighted the separation between CVT185 and CVT71, respectively, in V3 and V2 at the pea size stage (E-L31). CVT423, characterized by significant lower bunch weight and overall lower productivity yields than the two other clones (**Table 1**), was distinguished at E-L35 (**Figure 5B**). A slight effect due to the clone was also observed for genes linked to secondary metabolism at véraison, specifically in V3, as CVT71 and CVT185 clustered separately from CVT423 (**Figure S5**). This was consistent with agronomic data collected in 2013 and reporting the highest amounts of flavonoids in V3, particularly for CVT185 grapes (**Table 1**). A deeper analysis of the flavonoid biosynthetic transcripts (**Figure 6A**), differentially regulated by ripening stage (**Figure 6B**) in concomitance with other functional groups (i.e., sugar and hormone metabolism; Castellarin et al., 2016; Massonnet et al., 2017), strengthened the above results.

All sequencing results discussed above were then used as the source to target the analysis of candidate genes and metabolites involved in the most important biological pathways affected by the studied variables, also highlighting changes between the 2 years of trial. The obtained information was thus integrated with the set of agronomical data collected in parallel over the two vintages.

### A Closer Look on Sugar Metabolism and Transport

Sugar, as well as flavonoid, accumulation represents an upstream trigger for the evolution of berry development (Kuhn et al., 2014; Lecourieux et al., 2014) together with ABA-mediated signalling (Gambetta et al., 2010; Castellarin et al., 2016). A worth noting subject indeed relies on how the accumulation of ABA, sugars, and secondary metabolites changes as a function of plant environmental adaptation (Ferrandino and Lovisolo, 2014). As expected, the transcriptional abundance of sugar transporter genes changed in relation to berry phenology (**Supplementary Figure S6A**). Three hexose transporter (HT) genes were analyzed: *VvHT1* (VIT_00s0181g00010), the first HT member characterized in grapevine and localized at the plasma membrane (Fillion et al., 1999), *VvHT2* (VIT_18s0001g05570) and *VvHT6* (VIT_18s0122g00850), both encoding tonoplast-localized transporters specific of the berry cells (Hayes et al., 2007; Afoufa-Bastien et al., 2010). While *HT1* transcription was more active at pre-véraison, as reported elsewhere (Vignault et al., 2005; Conde et al., 2006), *HT2* and *HT6* were overexpressed at the onset of ripening. Dynamic responses ascribed to *C x E* effect were also observed: in CVT185 grapes from V2, *HT1* was expressed during all developmental stages, without significant variations between 2013 and 2014 (**Supplementary Figures S6B, C**). In 2013, *HT2* was more expressed in CVT71 grapes collected in V3 starting from the onset of ripening (**Supplementary Figure S6B**); and always for this clone, in 2014, the transcription of *HT6* was significantly more induced in V2 at E-L35 (**Supplementary Figure S6C**).

Hexose accumulation is among the primary signals that, concurrently with anthocyanin biosynthesis, switch the berry development towards véraison (Deluc et al., 2009). Interestingly, a sugar-mediated regulation does exist on both structural genes of flavonoid biosynthesis and transcription factors involved in the anthocyanin pathway (Lecourieux et al., 2014). Moreover, ABA metabolic pathways are strictly associated with changes in sugar turnover (Hayes et al., 2010) and anthocyanin production in the berry (Ferrero et al., 2018). Consistently, the ABA biosynthetic gene *VvNCED1* (VIT_19s0093g00550) was overexpressed at the onset of ripening in both the experimental years, in line with the patterns of ABA concentrations (**Figure S7**) and with the transcriptional profiles of *HT2* and *HT6* (**Figures S6B, C**) and of the anthocyanin biosynthetic genes *UFGT* and *AOMT* (**Figures 6C** and **S8**). These findings pointed out to a tight network of sugar and ABA signals that, acting in coordination with changes in secondary metabolism (**Figure S5**) (Gambetta et al., 2010; Speirs et al., 2013), modulate the evolution of berry development promoting the physiological adaptation of “Nebbiolo” clones to the environment.

Further support to this array of molecular changes derived from the analysis of agronomical parameters, such as the content of total soluble solids (TSS), which significantly differed based on environmental conditions and clone (**Tables 1** and **2**). This trend was particularly evident during the first vintage, as TSS values were significantly higher in the berries from V2 than from V1 and V3 (**Table 1**). Despite the viticultural practices were equally managed and clones were all grafted on the same rootstock in all vineyards, the three sites differed in terms of soil composition (**Table S2**). Soil properties, including pH, texture, drainage, and presence of specific microorganism consortia (Belda et al., 2017), can influence water uptake at the root level, triggering specific physiological adaptations (Tramontini et al., 2014; Zerihun et al., 2015). For instance, the soil in V2 was predominantly sandy with very low content of organic matter and limited cation exchange capacity. Conversely, V1 and V3, constituted by prevalently silty and loam soils, respectively, showed good values of organic matter content and cation exchange capacity (**Table S2**). Furthermore, we could not exclude that differences in altitude, row exposition (**Table S2**), meso- and microclimate, or nutrition could have influenced the plant water status and, consequently, the ripening dynamics, with effects on the accumulation of sugars (Brillante et al., 2018). Additionally, to assess whether the V2-dependent increase in TSS values could be supported by alterations of the plant sink-source balance, the ratio between yield and pruning wood (referred to as the Ravaz index, RI) was calculated. Independently of the clone, during the first year, RI values were significantly lower in V2 than in other growing sites (**Table 1**). However, unlike TSS, the RI trend was overall confirmed during the second vintage (**Table 2**). Being the weight of pruning wood not significantly different among the three vineyards in both years, the observed decrease in RI could only rely on the grape yield variability, which was significantly affected by the growing site in both the experimental seasons (**Tables 1** and **2**). Based on these observations and according to previous experimental evidence (Tramontini et al., 2013), the increase in berry sugars associated to V2 grapes in 2013 could most likely result from the interaction of the specific climatic conditions occurring in 2013 during the first period of ripening (**Supplementary Figure S1B**) [i.e., scarce precipitations, higher temperatures, and higher evaporative water demand (corresponding to higher VPD values)] with V2 soil features and row exposition and with rootstock-type (Kober 5BB, poorly tolerant to water deficit; Lovisolo et al., 2016).

Table 2Measurements of agronomical parameters, quantity and quality features of the studied ‘Nebbiolo’ clones (CVT71, CVT423 and CVT185) grown in three different vineyards of the Langhe area (V1, V2, V3) in 2014. Data are means ± standard error (*n = 21*). Results of ANOVA analysis are detailed below the table.

**Table:** 

Year 2014			CVT71								CVT423							CVT185						
			V1			V2		V3			V1			V2		V3		V1		V2				
Budbreak^a^			2.33 ± 0.10			2.82 ± 0.10		2.84 ± 0.11			2.16 ± 0.03			2.59 ± 0.10		2.77 ± 0.10		2.27 ± 0.10		2.92 ± 0.14				
Bud fertility (n° clusters per shoot)			0.72 ± 0.10			2.82 ± 0.10		2.84 ± 0.11			1.07 ± 0.13			2.59 ± 0.10		2.77 ± 0.10		0.69 ± 0.10		2.92 ± 0.14				
Yield (kg per plant)			1.97 ± 0.26			1.10 ± 0.18		2.11 ± 0.26			2.25 ± 0.29			1.04 ± 0.07		1.75 ± 0.13		1.47 ± 0.26		1.47 ± 0.17				
N° clusters per plant			7.24 ± 0.91			4.4 ± 0.67		7.3 ± 0.92			10.87 ± 1.38			5.3 ± 0.44		7.8 ± 0.46		6.50 ± 0.86		5.7 ± 0.69				
Cluster weight (g)			269 ± 17.29			244 ± 15.76		294 ± 10.52			207 ± 11.01			216 ± 11.70		227 ± 10.53		226 ± 8.77		266 ± 14.25				
Total Soluble Solids (TSS, °Brix)			23.08 ± 0.46			24.72 ± 0.21		24.65 ± 0.45			23.27 ± 0.13			24.08 ± 0.16		25.14 ± 0.15		23.04 ± 0.36		22.28 ± 0.6				
pH			2.88 ± 0.03			3.08 ± 0.01		3.05 ± 0.02			2.96 ± 0.03			3.04 ± 0.01		3.07 ± 0.02		2.85 ± 0.02		3.00 ± 0.02				
Titratable acidity (g L^-1^)			13 ± 0.08			10.57 ± 0.2		8.69 ± 0.10			10.89 ± 0.5			10.19 ± 0.26		7.68 ± 0.21		13.56 ± 0.53		11.72 ± 0.25				
Tartaric acid (g L^-1^)			10.45 ± 0.51			9.64 ± 0.12		8.85 ± 0.10			8.68 ± 0.30			8.64 ± 0.10		8.18 ± 0.15		10.54 ± 0.1		8.94 ± 0.14				
Malic acid (g L^-1^)			4.50 ± 0.54			3.28 ± 0.17		1.75 ± 0.28			3.40 ± 0.31			3.56 ± 0.25		0.72 ± 0.10		4.72 ± 0.48		4.92 ± 0.26				
Total anthocyanins (mg kg^-1^)			904 ± 25.98			881 ± 19.27		804 ± 10.17			891 ± 40.92			826 ± 57.05		780 ± 19.50		705 ± 21.93		815 ± 57.09				
Anthocyanin extractability (%)			76 ± 0.67			79 ± 0.58		83 ± 0.33			82 ± 1			79 ± 2.33		82 ± 0.58		88 ± 1.45		70 ± 2.03				
Total flavonoids (mg kg^-1^)			3756 ± 129.62			3958 ± 126.19		3653 ± 89.16			5167 ± 149.16			3937 ± 383.54		3820 ± 46.51		3018 ± 94.82		3452 ± 177.70				
Pruning wood (kg per plant)			1.02 ± 0.13			0.99 ± 0.05		0.75 ± 0.07			0.67 ± 0.09			0.77 ± 0.05		0.61 ± 0.04		0.87 ± 0.08		0.85 ± 0.04				
Ravaz index^b^ (RI, kg)			1.93 ± 0.07			1.11 ± 0.12		2.81 ± 0.13			3.36 ± 0.16			1.35 ± 0.04		2.87 ± 0.05		1.69 ± 0.12		1.73 ± 0.12				

a Budbreak index was estimated based on a scale from 1 (dormant bud) to 6 (three unfolded young leaves) according to Baggiolini (1952).

b Yield/pruning weight per plant.

**Table:** 

Source of variation	Budbreak	Bud fertility		Yield	N° clusters/plant		Cluster weight		Total Soluble Solids	pH		Titratable acidity	Tartaric acid		Malic acid		Total anthocyanins		Anthocyanin extractability		Total flavonoids	Pruning wood		Ravaz index
Clone	**	NS		**	NS		**		NS	**		**	**		**		*		NS		**	**		***
Vineyard	**	**		**	**		**		**	**		**	**		**		NS		NS		NS	NS		***
Clone x Vineyard	NS	**		NS	**		NS		**	NS		NS	**		*		*		NS		**	NS		***

Significance of clone, vineyard, and interaction clone × vineyard effects was tested for P ≤ 0.05 (*), P ≤ 0.01 (**), and P ≤ 0.001 (***); NS, not significant.

Unlike TSS, other technological parameters, such as titratable acidity of musts, showed stable responses between the two seasons, reflecting the major influence of the genotype component. For instance, in both years, once reached the same level of maturity, the berries collected from CVT185 plants showed higher acidity (TA, **Tables 1** and **2**). Accordingly, other authors reported that differences in terms of chemical properties of grapes (including TA), besides being affected by climate (Arrizabalaga et al., 2018), were mainly influenced by the clone genotype (van Leeuwen et al., 2013b).

### Changes in anthocyanin metabolism and transport Underlying the C x E Interplay

Despite the amount of total flavonoids and total anthocyanins was another feature dependent on genetic characteristics (Mattivi et al., 2006; Pinasseau et al., 2017), the obtained results attested that year and field site also concurred to influence them. Accordingly, the analysis of genes controlling the most crucial steps of the anthocyanin biosynthesis (Bogs et al., 2006; Hugheney et al., 2009), namely *VvUFGT* (VIT_16s0039g02230) and *VvAOMT* (VIT_01s0010g03510), evidenced transcriptional changes associated with specific clone-vineyard combinations (**Figure 6C** and **Supplementary Figures S8A–F**). Furthermore, agronomical results showed that in 2013, the grapes harvested in V3 accumulated higher content of total flavonoids than those from other vineyards, and the highest values were observed for CVT185 samples (**Table 1**). On the contrary, in 2014, CVT185 berries had a lower quantity of total flavonoids than CVT71 and CVT423, which showed the highest amount of these compounds in V2 and V1, respectively (**Table 2**). Unlike total flavonoids, total anthocyanins were more abundant in 2014 (**Tables 1** and **2**), characterized by a summer season with overall milder temperatures than 2013 (**Supplementary Figure S1**). Similarly, yields of anthocyanin extractability had a tendency to increase in the second year of trial, with values not significantly different among the clones (**Tables 1** and **2**).

It is well known that weather conditions can negatively affect the pattern of anthocyanin accumulation in grapevine (Bergqvist et al., 2001). In particular, it was pointed out that rainfall events occurring around véraison most predominantly influence the synthesis of anthocyanins and their abundance at harvest (Ferrandino and Guidoni, 2010). Additionally, different studies have demonstrated that high water content has a detrimental effect on the anthocyanin accumulation (Castellarin et al., 2007; Brillante et al., 2017; Mirás-Avalos and Intrigliolo, 2017). Accordingly, although total rainfall was significantly (*P < 0.01*) more abundant over the whole year 2014 than 2013 (1182.86 ± 33.51 mm vs 965.86 ± 35.46 mm, respectively), around véraison (August, 26^th^) in 2013, there were more precipitations (average values: 78.66 ± 9.11 mm) than during véraison (August, 19^th^) in 2014 (average values: 36.80 ± 1.30 mm; **Supplementary Figure S1**). This could represent a reasonable explanation supporting the observed differences in terms of total anthocyanins in the 2 years.

Additionally, although “Nebbiolo” is a peonidin 3-*O*-glucoside prevalent cultivar (Ferrandino et al., 2012), significant differences in terms of percentage of single anthocyanins (**Tables 3** and **4**) were observed among clones in both years of trial. Grapevine intra-specific variability was largely documented in terms of single anthocyanin profiles (Ortega-Regules et al., 2006; González-Neves et al., 2008; Ferrandino et al., 2012), but intra-varietal differences linked with anthocyanin accumulation and proportion do also exist (Arozarena et al., 2002; Arrizabalaga et al., 2018). Consistently, grapes of CVT423 had significantly lower content of malvidin-3-*O*-glucoside and petunidin-3-*O*-glucoside (**Tables 3** and **4**), both tri-substituted anthocyanins, which are more stable forms over wine fermentation and aging (González-Neves et al., 2008; Ferrandino et al., 2012). This finding confirmed that, during ripening, besides accumulation, also partitioning of secondary metabolites could depend on the adaptive response of clones to environmental conditions (Ferrandino and Lovisolo, 2014; Lovisolo et al., 2016). Interestingly, in both years, the variability associated with contents of tri- and di-substituted anthocyanins was also evident in terms of "environmental effect," as both the forms were less accumulated in V2 than in other vineyards (**Table 3**). It was shown that the biosynthesis of tri-hydroxylated anthocyanins is upregulated at the expense of the di-hydroxylated forms upon water deficit (Castellarin et al., 2007). Accordingly, VPD data collected over the 2 years (**Figure S1**) evidenced that the evaporative transpirative demand was always higher in V2 than in the other sites. As the plant water status is easily influenced by the environment/clone characteristics (e.g., interactions among canopy size, climate and soil), differences in the ratio between tri- and di-hydroxylated anthocyanins may vary as a function of the environmental variability existing even within the same vineyard (Brillante et al., 2017).

**Table 3:** Anthocyanin profiles (percent) in the skin of grapes collected at 24°BRIX from the studied ‘Nebbiolo’ clones (CVT71, CVT423 and CVT185) in three different vineyards of the Langhe area (V1, V2, V3) in 2013. Data are means ± standard error (*n = 21*). Results of ANOVA analysis are reported below the table.

Year 2013			CVT71								CVT423								CVT185						
			V1			V2			V3		V1			V2		V3			V1						
Delphinidin-3-*O*-glucoside			4.72 ± 0.33			3.56 ± 0.17			8.08 ± 0.10		4.97 ± 0.03			2.90 ± 0.19		5.39 ± 0.06			6.34 ± 0.42						
Cyanidin-3-*O*-glucoside			15.61 ± 0.95			18.25 ± 0.66			9.08 ± 0.46		18.77 ± 1.29			21.86 ± 0.72		17.18 ± 1.32			16.75 ± 0.83						
Petunidin-3-*O*-glucoside			4.10 ± 0.26			3.19 ± 0.15			6.91 ± 0.16		3.99 ± 0.08			2.48 ± 0.16		4.51 ± 0.15			4.77 ± 0.28						
Peonidin-3-*O*-glucoside			51.96 ± 1.66			55.29 ± 0.99			35.74 ± 0.86		51.22 ± 0.50			56.07 ± 0.43		49.14 ± 0.71			46.93 ± 1.75						
Malvidin-3-*O*-glucoside			18.28 ± 1.91			13.79 ± 1.36			32.66 ± 0.93		14.96 ± 0.70			9.98 ± 0.61		16.20 ± 1.33			19.06 ± 1.05						
Delphinidin-acetylglucoside			0.09 ± 0.01			0.08 ± 0.01			0.23 ± 0.01		0.13 ± 0.00			0.08 ± 0.00		0.17 ± 0.00			0.16 ± 0.01						
Cyanidin-acetylglucoside			0.27 ± 0.02			0.38 ± 0.02			0.25 ± 0.00		0.41 ± 0.02			0.54 ± 0.01		0.52 ± 0.04			0.37 ± 0.02						
Petunidin-acetylglucoside			0.06 ± 0.01			0.04 ± 0.00			0.19 ± 0.01		0.07 ± 0.01			0.03 ± 0.00		0.13 ± 0.01			0.10 ± 0.01						
Peonidin-acetylglucoside			1.14 ± 0.07			1.30 ± 0.04			0.12 ± 0.01		1.26 ± 0.06			1.53 ± 0.06		1.54 ± 0.03			1.14 ± 0.08						
Malvidin-acetylglucoside			0.55 ± 0.05			0.43 ± 0.04			1.20 ± 0.05		0.51 ± 0.04			0.35 ± 0.02		0.67 ± 0.05			0.68 ± 0.04						
Peonidin-caffeoylglucoside			0.11 ± 0.01			0.09 ± 0.00			0.25 ± 0.01		0.14 ± 0.00			0.09 ± 0.00		0.18 ± 0.01			0.17 ± 0.01						
Malvidin-caffeoylglucoside			0.32 ± 0.01			0.30 ± 0.02			0.29 ± 0.01		0.31 ± 0.01			0.29 ± 0.03		0.37 ± 0.02			0.28 ± 0.02						
Dephinidin *p*-coumaroylglucoside			0.14 ± 0.01			0.08 ± 0.01			0.28 ± 0.01		0.12 ± 0.00			0.13 ± 0.01		0.13 ± 0.01			0.13 ± 0.01						
Cyanidin *p*-coumaroylglucoside			0.40 ± 0.02			0.61 ± 0.03			0.38 ± 0.01		0.62 ± 0.05			0.82 ± 0.00		0.73 ± 0.05			0.52 ± 0.03						
Petunidin *p*-coumaroylglucoside			0.09 ± 0.01			0.07 ± 0.00			0.22 ± 0.01		0.01 ± 0.00			0.06 ± 0.00		0.14 ± 0.01			0.13 ± 0.01						
Peonidin *p*-coumaroylglucoside			1.62 ± 0.08			2.09 ± 0.07			1.56 ± 0.01		1.94 ± 0.06			2.42 ± 0.11		2.22 ± 0.02			1.80 ± 0.02						
Malvidin *p*-coumaroylglucoside			0.54 ± 0.06			0.45 ± 0.07			1.57 ± 0.06		0.48 ± 0.03			0.36 ± 0.01		0.79 ± 0.09			0.66 ± 0.02						
Source of variation	Delphinidin-3-*O*-glucoside	Cyanidin-3-*O*-glucoside		Petunidin-3-*O*-glucoside	Peonidin-3-*O*-glucoside		Malvidin3-*O*-glucoside-	Delphinidin-acetylglucoside		Cyanidin- acetylglucoside		Petunidin-acetylglucoside	Peonidin- acetylglucoside		Malvidin- acetylglucoside		Peonidin-caffeoyl-glucoside	Malvidin-caffeoyl-glucoside		Dephinidin *p*-coumaroyl-glucoside	Cyanidin *p*-coumaroyl-glucoside		Petunidin *p*- coumaroyl-glucoside	Peonidin *p*- coumaroyl-glucoside	Malvidin *p*- coumaroyl-glucoside
Clone	***	***		***	***		***	***		***		***	***		***		***	NS		***	***		***	***	***
Vineyard	***	***		***	***		***	***		***		***	***		***		***	*		***	***		***	***	***
Clone x Vineyard	***	NS		***	***		***	***		NS		***	*		***		***	NS		***	NS		***	*	***
Significance of clone, vineyard, and interaction clone × vineyard effects was tested for P ≤ 0.05 (*), P ≤ 0.01 (**), and P ≤ 0.001 (***); NS, not significant.																									

**Table 4:** Anthocyanin profiles (percent) in the skin of grapes collected at 24°BRIX from the studied “Nebbiolo” clones (CVT71, CVT423 and CVT185) in three different vineyards of the Langhe area (V1, V2, V3) in 2014. Data are means ± standard error (*n = 21*). Results of the ANOVA analysis are reported below the table.

Year 2014				CVT71								CVT423							CVT185							
				V1			V2		V3			V1		V2		V3			V1			V2			V3	
Delphinidin-3-*O*-glucoside				3.80 ± 0.10			2.87 ± 0.20		4.47 ± 0.22			3.83 ± 0.23		3.30 ± 0.15		4.10 ± 0.36			4.67 ± 0.30			3.50 ± 0.35			4.53 ± 0.33	
Cyanidin-3-*O*-glucoside				14.73 ± 1.47			13.07 ± 0.82		10.70 ± 0.20			15.83 ± 0.13		20.30 ± 1.19		16.83 ± 0.83			15.70 ± 0.80			16.17 ± 1.31			14.70 ± 0.21	
Petunidin-3-*O*-glucoside				3.43 ± 0.09			3.03 ± 1.12		4.57 ± 0.15			3.63 ± 0.15		3.57 ± 0.09		3.83 ± 0.27			4.03 ± 0.12			3.43 ± 0.18			4.37 ± 0.30	
Peonidin-3-*O*-glucoside				57.60 ± 0.44			61.73 ± 0.09		52.87 ± 1.23			55.63 ± 0.84		57.67 ± 0.61		54.83 ± 1.34			55.10 ± 2.30			55.63 ± 0.55			53.93 ± 1.35	
Malvidin-3-*O*-glucoside				15.50 ± 1.15			14.93 ± 0.30		22.40 ± 0.81			15.97 ± 0.17		12.10 ± 0.70		14.43 ± 1.31			16.13 ± 0.39			15.77 ± 1.23			15.87 ± 0.58	
Delphinidin-acetylglucoside				0.07 ± 0.03			0.03 ± 0.03		0.10 ± 0.00			0.10 ± 0.00		0.10 ± 0.00		0.10 ± 0.00			0.10 ± 0.00			0.10 ± 0.00			0.13 ± 0.03	
Cyanidin-acetylglucoside				0.23 ± 0.03			0.23 ± 0.03		0.10 ± 0.00			0.30 ± 0.00		0.37 ± 0.03		0.30 ± 0.00			0.20 ± 0.10			0.37 ± 0.03			0.40 ± 0.00	
Petunidin-acetylglucoside				0.03 ± 0.03			0.00 ± 0.00		0.10 ± 0.00			0.07 ± 0.03		0.00 ± 0.00		0.07 ± 0.03			0.07 ± 0.03			0.03 ± 0.03			0.10 ± 0.00	
Peonidin-acetylglucoside				0.90 ± 0.00			1.03 ± 0.09		0.83 ± 0.03			1.00 ± 0.06		0.93 ± 0.03		1.00 ± 0.06			0.77 ± 0.23			1.10 ± 0.15			1.40 ± 0.58	
Malvidin-acetylglucoside				0.37 ± 0.03			0.40 ± 0.00		0.53 ± 0.03			0.47 ± 0.03		0.33 ± 0.03		0.47 ± 0.03			0.40 ± 0.15			0.47 ± 0.07			0.70 ± 0.06	
Peonidin-caffeoylglucoside				0.10 ± 0.00			0.10 ± 0.00		0.10 ± 0.00			0.10 ± 0.00		0.00 ± 0.00		0.13 ± 0.03			0.10 ± 0.00			0.10 ± 0.00			0.13 ± 0.03	
Malvidin-caffeoylglucoside				0.30 ± 0.00			0.30 ± 0.15		0.17 ± 0.03			0.43 ± 0.20		0.10 ± 0.00		0.23 ± 0.03			0.27 ± 0.09			0.27 ± 0.03			0.30 ± 0.00	
Dephinidin *p*-coumaroylglucoside				0.10 ± 0.00			0.07 ± 0.07		0.10 ± 0.00			0.13 ± 0.09		0.00 ± 0.00		0.10 ± 0.00			0.10 ± 0.06			0.07 ± 0.03			0.10 ± 0.00	
Cyanidin *p*-coumaroylglucoside				0.40 ± 0.06			0.30 ± 0.06		0.30 ± 0.00			0.40 ± 0.06		0.23 ± 0.03		0.60 ± 0.00			0.37 ± 0.03			0.50 ± 0.00			0.50 ± 0.00	
Petunidin *p*-coumaroylglucoside				0.10 ± 0.00			0.07 ± 0.03		0.10 ± 0.00			0.07 ± 0.03		0.00 ± 0.00		0.10 ± 0.00			0.07 ± 0.03			0.10 ± 0.00			0.10 ± 0.00	
Peonidin *p*-coumaroylglucoside				1.90 ± 0.06			1.47 ± 0.24		1.80 ± 0.06			1.57 ± 0.26		0.77 ± 0.09		2.27 ± 0.03			1.43 ± 0.12			1.93 ± 0.18			2.00 ± 0.10	
Malvidin *p*-coumaroylglucoside				0.53 ± 0.03			0.33 ± 0.07		0.77 ± 0.09			0.47 ± 0.09		0.17 ± 0.03		0.60 ± 0.06			0.50 ± 0.10			0.53 ± 0.09			0.77 ± 0.12	
Source of variation	Delphinidin-3-*O*-glucoside	Cyanidin-3-*O*-glucoside	Petunidin-3-*O*-glucoside		Peonidin-3-*O*-glucoside	Malvidin3-*O*-glucoside-		Delphinidin-acetylglucoside		Cyanidin-acetylglucoside	Petunidin-acetylglucoside		Peonidin- acetylglucoside		Malvidin- acetylglucoside		Peonidin-caffeoyl-glucoside	Malvidin-caffeoyl-glucoside		Dephinidin *p*-coumaroyl-glucoside	Cyanidin *p*-coumaroyl-glucoside		Petunidin *p*- coumaroyl-glucoside	Peonidin *p*- coumaroyl-glucoside		Malvidin *p*- coumaroyl-glucoside
Clone	*	***	NS		NS	***		*		***	NS		NS		NS		NS	NS		NS	**		NS	NS		*
Vineyard	***	*	***		***	***		NS		NS	**		NS		**		**	NS		NS	**		*	***		***
Clone x Vineyard	NS	*	*		NS	***		NS		*	NS		*		NS		**	NS		NS	***		NS	***		NS
Significance of clone, vineyard, and interaction clone × vineyard effects was tested for P ≤ 0.05 (*), P ≤ 0.01 (**), and P ≤ 0.001 (***); NS, not significant.																										

These observations well fit with the analysis of the genes involved in anthocyanin transport: *VvABCC1* (VIT_16s0050g02480), encoding a tonoplast-localized transporter of glucosylated anthocyanins (Francisco et al., 2013), *VvAM1* (VIT_16s0050g00930), encoding a vacuolar ANTHOMATE transporter involved in the delivery of acylated anthocyanins (Gomez et al., 2009), and *VvGST4* (VIT_04s0079g00690), encoding a glutathione S-transferase mediating anthocyanin transport into the vacuole (Ageorges et al., 2006). Indeed, *VvABCC1*expression did not follow the same increasing trend in all the growing sites (**Supplementary Figures S9A–C**). Similarly, *VvAM1* (**Supplementary Figures S9D–F**) and *VvGST4* (**Supplementary Figures S9G–I**) showed transcriptional patterns close to those of the anthocyanin acyltransferase gene *Vv3AT* (**Figure 6C**), involved in the synthesis of acylated anthocyanins (Rinaldo et al., 2015). Given that acylated forms are peculiar of "Nebbiolo" grapes (Ferrandino et al., 2012), this coordination between transcriptional activation of acylated anthocyanin biosynthesis (*Vv3AT*) and transport (*VvAM1*, *VvGST4*) well supported what observed at the metabolite level in terms of anthocyanin partitioning. Moreover, the fact that expression changes of acyltransferase and anthocyanin transporter genes were only partially confirmed in 2014 (**Supplementary Figures S8G–I and Figure S9D**’–I’, respectively), revealed that anthocyanin acylation and transport were influenced by the *C x E* interaction.

### Berry Development and Stress Defense Mechanisms Associated With the C X E Interaction

Considering the differences observed in the two seasons for a number of technological parameters based on geographical site and clone (**Tables 1** and **2**), more attention was paid to analyze genes tied to berry development. Expression levels of transcripts, such as *XET32* (VIT_06s0061g00550), among the most expressed genes of this functional category (**Supplementary Figure S10A**), were highly induced starting from véraison (**Supplementary Figure S10B**). Effect of the *C x E* interplay was found associated to the modulation of cell wall and developmental processes (**Figure 5**), and accordingly, *XET32* was differentially expressed in presence of a specific *C x E* combination (e.g., CVT185 in V3) (**Supplementary Figure S10C**). All these data were in agreement with the high cluster weight and productivity yields of grapes from CVT71 and CVT185 (**Tables 1** and **2**). Moreover, *XET32* followed the same expression profile of *VvNCED1* (**Supplementary Figure S7**) and sugar transporter genes (*VvHT2*, *VvHT6*, **Supplementary Figures S6B, C**). Considering that XET are transcriptionally induced by ABA (Giribaldi et al., 2010) and sugars (Kuhn et al., 2014), these findings outlined how the cross-talk between hormone and sugar signalling cascades may regulate crucial steps of berry development through a direct control of cell wall modifications.

**Figure 7:**
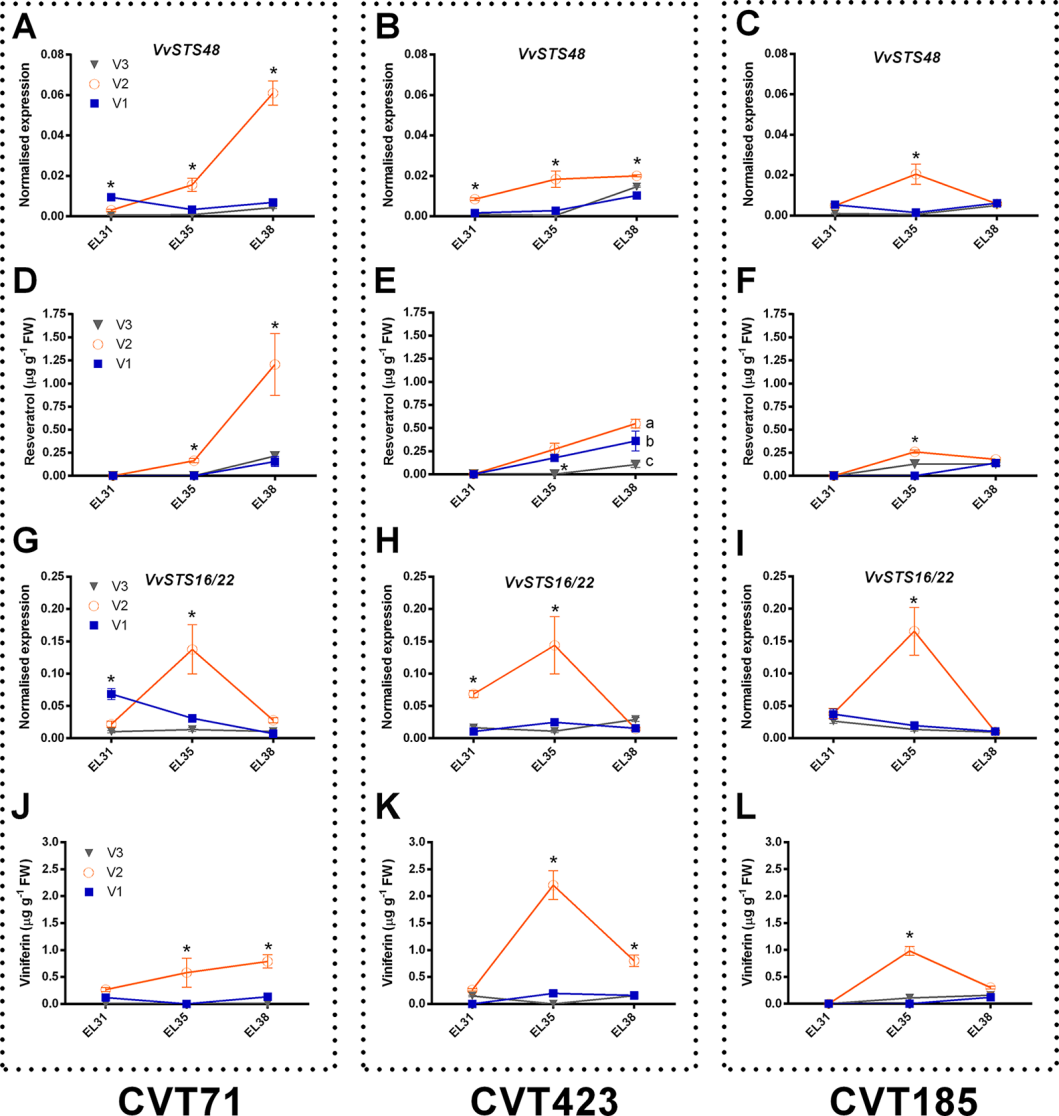
Focus on stilbenoid metabolism. RT-qPCR expression profiles of genes encoding stilbene synthases **(A**-**C)** *Vv*STS48 (VIT_16s0100g01200) and **(G-I)***Vv*STS16/22 (VIT_16s0100g00920) and accumulation patterns of stilbenoid compounds, resveratrol **(D**-**F)**, and viniferin **(J**-**L)** (both expressed as µg g^-1^), analyzed on whole berries collected in three vineyards (V1, V2, V3) from CVT71, CVT423 and CVT185 "Nebbiolo" clones at three ripening stages (E-L31, E-L35, E-L38) during the first year of trial (2013). Asterisk or lower case letters denote significant differences attested by Tukey’s *HSD* test (*P < 0.05*) respectively when values were significant in only one or more samples. Bars represent standard error of the mean (*n = 3*). The E-L31, E-L35, E-L38 stages from the modified E-L scheme respectively correspond to stage 75, 81, and 89 in the extended BBCH scheme.

Within the stress defense category, analysis of genes encoding stilbene synthases (*VvSTS16/22* VIT_16s0100g00920, *VvSTS48* VIT_16s0100g01200), already characterized in grapevine (Parage et al., 2012; Vannozzi et al., 2012), showed induction of these transcripts in the berries from V2 at E-L35 and, only in the case of CVT71, at E-L38 (**Figure 4**). Curiously, transcriptional profiles of *VvSTS48* (**Figures 7A–C**) and *VvSTS16/22* (**Figures 7G–I**) respectively followed accumulation of resveratrol (**Figures 7D–F**) and viniferin (**Figures 7J–L**) quantified on the same samples. Despite little is known about the function of single stilbenoid molecules, these results suggested that diverse stilbene synthase genes could preferentially act on specific branches of the stilbenoid biosynthetic pathway, thus controlling timing and partitioning of phytoalexin synthesis (Duan et al., 2015). Other transcripts falling into the stress response category were primarily affected by V2, at véraison (**Supplementary Figure S11**). This picture of plastic responses of "Nebbiolo" clones to V2 conditions was strengthened by a comparative survey carried out on the subset of unmapped reads remaining from RNA-seq data. Percentages of unmapped reads aligning against the genome of the three main grapevine fungal pathogens (*Botrytis cinerea, Erysiphe necator, Plasmopara viticola*) were similar among the three field sites (**Supplementary Table S4**). It is therefore reasonable that in presence of specific environmental conditions (e.g., V2 soil features, as previously discussed), vines adaptation could result in an overexpression of stress response signals, among which *STS* activation was one of the hub molecular events (Chitarra et al., 2017).

Finally, although expression differences of *STS48* were overall confirmed in 2014 (**Supplementary Figures S12A–C**) together with resveratrol levels (**Supplementary Figures S12D–F**), transcript profiles of *STS16/22* (**Supplementary Figures S12G–I**) and accumulation trends of viniferin (**Supplementary Figures S12J–L**) differed in the 2 years based on environment effect (e.g., *STS16/22* was significantly over-expressed in V3 instead of V2 at E-L35). This represented another example of *C x E* interaction and was in agreement with previous reports attesting that the production of defense compounds can vary in function of grapevine genotype, soil, and climate (Vincenzi et al., 2013).

## Conclusions

Our study provides new insights into the still poorly characterized array of molecular mechanisms underlying the *C x E* interaction in grapevine.

In particular, we demonstrated that even the few genetic differences existing among clones of the same cultivar (i.e., "Nebbiolo") can affect the physiological and agronomical aptitudes of grapevine through fine transcriptional reprogramming events mainly linked to carbohydrate and secondary metabolic changes.

Accordingly, despite transcripts involved in the anthocyanin biosynthesis are prevalently controlled by ripening stage—as well as sugar transport- and ABA biosynthesis-associated genes—the transcriptional regulation of anthocyanin acylation and transport and the related patterns of anthocyanin accumulation and partitioning strictly depend on the clone physiological adaptation to a specific growing site and to climatic variations occurring along the season.

Development and stress defense responses are also processes transcriptionally controlled based on genotype, environment or their combination. For instance, the "V2 environment"-dependent up-regulation of stilbene synthase-encoding genes is most likely the upstream trigger for the increase in resveratrol and viniferin contents at véraison during the first year of trial.

All these findings point to a complex and interactive molecular machinery that contributes to shape the final quality features of "Nebbiolo" grapes at harvest, and that, on a broader scale, can offer valuable support to orient viticultural practices aimed at enhancing the quality of grape productions in light of growing site and clone choice.

## Data Availability Statement

Raw RNA-sequencing data and normalized expression values are available at the Gene Expression Omnibus (GEO) database with accession number GSE116238.

## Author Contributions

CP performed most of the experiments, analyzed and compared all data and wrote the article. PB performed plant material collection, RNA extraction, and complemented the writing. WC carried out the metabolic analyses and complemented the writing. EC, AM, and MR carried out the sequencing analysis and bioinformatic elaboration of data. MS and PZ developed and applied dedicated statistical analyses on sequencing data. IP helped with interpretation of bioinformatics and gene expression data and revised the manuscript. DC, IG, and FM performed sample collection, carried out the agronomical measurements, analyzed the corresponding data, and revised the manuscript. LN helped with the metabolic analyses and analyzed the corresponding data. MP and MD supported sequencing analyses and critically revised the manuscript. GG and IG conceived the project. GG supervised all the experiments and wrote the article. All authors read and approved the manuscript.

## Funding

This work was supported by the Nebbiolo Genomics project, financed by Fondazione Cassa di Risparmio di Cuneo, and a research grant from the Italian Ministry of Education, University and Research (FIR project "The epigenomic plasticity of grapevine in genotype per environment interactions" No. RBFR13GHC5 to I.P).

## Conflict of Interest

The authors declare that the research was conducted in the absence of any commercial or financial relationships that could be construed as a potential conflict of interest.
