# Supplementary material for: Distinct Metabolic Signals Underlie Clone by Environment Interplay in “Nebbiolo” Grapes Over Ripening
Source: Front Plant Sci. 2019 Dec 4;10:1575. doi: 10.3389/fpls.2019.01575 (PMC6904956; doi:10.3389/fpls.2019.01575)

Distinct metabolic signals underlie clone by environment interplay in ‘Nebbiolo’ grapes over ripening

Chiara Pagliarani[1*], Paolo Boccacci[1], Walter Chitarra[1,2], Emanuela Cosentino[3], Marco Sandri[4], Irene Perrone[1], Alessia Mori[3], Danila Cuozzo[1,5], Luca Nerva[1,2], Marzia Rossato[3], Paola Zuccolotto[6], Mario Pezzotti[3], Massimo Delledonne[3], Franco Mannini[1], Ivana Gribaudo[1], Giorgio Gambino[1]

[1]Institute for Sustainable Plant Protection, National Research Council (IPSP-CNR), Torino. Strada delle Cacce 73, 10135 Torino, Italy.

[2]Council for Agricultural Research and Economics Centre of Viticultural and Enology Research (CREA-VE). Viale XXVIII Aprile 26, 31015 Conegliano (Treviso), Italy.

[3]Department of Biotechnology, University of Verona. Strada le Grazie 15, 37134 Verona, Italy

[4]DMS StatLab, University of Brescia, c.da S. Chiara 50, 25122 Brescia, Italy.

[5]Department of Agricultural, Forest and Food Sciences, University of Torino, Largo Braccini 2, 10095 Grugliasco, TO, Italy

[6]Big&Open Data Innovation Laboratory, University of Brescia, c.da S. Chiara 50, 25122 Brescia, Italy.

*Correspondence:
Dr. Chiara Pagliarani
chiara.pagliarani@ipsp.cnr.it

Supplementary material

Table S1. Main agronomic and oenological features of the three studied 'Nebbiolo' clones, as indicated by the selector. The clones were recorded in the Italian National Register of Grape Varieties by the Institute for Sustainable Plant Protection, National Research Council (IPSP-CNR).

Clone ID	Biotype	Origin (Municipality area)	Vigour	Yield	Wine quality
CVT71	Michet	La Morra (CN)	medium-high	medium	high
CVT185	Lampia	Barbaresco (CN)	high	medium	high
CVT423	Picoutener	Pont St. Martin (AO)	low	low	good

Table S2. Soil chemical-physical features of the considered vineyards.

Vineyard ID	Municipality area	Geographic coordinates	Altitude (m a.s.l.)	Row orientation	pH	Soil composition	Organic matter content (g Kg^{-1} soil)	Cation exchange capacity (meq 100 g^{-1} soil)
V1	Monforte d'Alba (CN)	44°59'43.76" N; 7°96'05.80" E	360	south	8.12	sand, 35.6%; silt, 43.7%; clay, 20.6%	12.8	11.6
V2	Monforte d'Alba (CN)	44°60'35.66"N; 7°97'88.11" E	413	south-east	8.29	sand, 65.4%; silt, 25.8%; clay, 8.8%	5.9	7.8
V3	Treiso (CN)	44°68'73.91"N; 8°08'41.82"E	344	south-west	8.18	sand, 50.5%; silt, 33.4%; clay, 16.1%	10.5	9.2

Table S3. Summary statistics of RNA-sequencing data analysis.

Sample description	Clone	Vineyard	Stage	# Total reads count	# Mapped reads count	% mapped (RSEqc)
71-V1-31-rep1	CVT71	V1	E-L31	26881110	22872725	85.09
71-V1-31-rep2	CVT71	V1	E-L31	21709027	18546278	85.43
71-V1-31-rep3	CVT71	V1	E-L31	21969613	18677491	85.02
71-V2-31-rep1	CVT71	V2	E-L31	24799054	20598722	83.06
71-V2-31-rep2	CVT71	V2	E-L31	24164514	20017396	82.84
71-V2-31-rep3	CVT71	V2	E-L31	23560570	20203480	85.75
71-V3-31-rep1	CVT71	V3	E-L31	25251165	21417529	84.82
71-V3-31-rep2	CVT71	V3	E-L31	19153413	16371169	85.47
71-V3-31-rep3	CVT71	V3	E-L31	20682478	17624573	85.21
423-V1-31-rep1	CVT423	V1	E-L31	23544143	20052455	85.17
423-V1-31-rep2	CVT423	V1	E-L31	24568532	21054029	85.70
423-V1-31-rep3	CVT423	V1	E-L31	25730348	22220538	86.36
423-V2-31-rep1	CVT423	V2	E-L31	19331024	16888799	87.37
423-V2-31-rep2	CVT423	V2	E-L31	22488924	19528659	86.84
423-V2-31-rep3	CVT423	V2	E-L31	25530580	21390394	83.78
423-V3-31-rep1	CVT423	V3	E-L31	24532605	20552014	83.77
423-V3-31-rep2	CVT423	V3	E-L31	27376577	23002077	84.02
423-V3-31-rep3	CVT423	V3	E-L31	23801956	20059603	84.28
185-V1-31-rep1	CVT185	V1	E-L31	24125342	20418558	84.64
185-V1-31-rep2	CVT185	V1	E-L31	22912940	19246011	84.00
185-V1-31-rep3	CVT185	V1	E-L31	18480114	15825932	85.64
185-V2-31-rep1	CVT185	V2	E-L31	19523864	16366744	83.83
185-V2-31-rep2	CVT185	V2	E-L31	22105193	19139946	86.59
185-V2-31-rep3	CVT185	V2	E-L31	24803201	21390570	86.24
185-V3-31-rep1	CVT185	V3	E-L31	17754373	15216536	85.71
185-V3-31-rep2	CVT185	V3	E-L31	27534570	23477229	85.26
185-V3-31-rep3	CVT185	V3	E-L31	24353801	20860238	85.65

Table S3. continue.

Sample description	Clone	Vineyard	Stage	# Total reads count	# Mapped reads count	% mapped (RSEqc)
71-V1-35-rep1	CVT71	V1	E-L35	22223035	19001474	85.50
71-V1-35-rep2	CVT71	V1	E-L35	22295683	18252101	81.86
71-V1-35-rep3	CVT71	V1	E-L35	24331322	20093444	82.58
71-V2-35-rep1	CVT71	V2	E-L35	33629305	21867039	65.02
71-V2-35-rep2	CVT71	V2	E-L35	20486614	17832405	87.04
71-V2-35-rep3	CVT71	V2	E-L35	30360951	25435164	83.78
71-V3-35-rep1	CVT71	V3	E-L35	24457044	17448155	71.34
71-V3-35-rep2	CVT71	V3	E-L35	25552273	19841205	77.65
71-V3-35-rep3	CVT71	V3	E-L35	22834546	15163347	66.41
423-V1-35-rep1	CVT423	V1	E-L35	21900018	18923567	86.41
423-V1-35-rep2	CVT423	V1	E-L35	19823900	16332061	82.39
423-V1-35-rep3	CVT423	V1	E-L35	33348356	27112417	81.30
423-V2-35-rep1	CVT423	V2	E-L35	22910086	19111723	83.42
423-V2-35-rep2	CVT423	V2	E-L35	23371676	18817154	80.51
423-V2-35-rep3	CVT423	V2	E-L35	22909223	19370131	84.55
423-V3-35-rep1	CVT423	V3	E-L35	21567004	18181365	84.30
423-V3-35-rep2	CVT423	V3	E-L35	23746035	20375767	85.81
423-V3-35-rep3	CVT423	V3	E-L35	27116508	23041482	84.97
185-V1-35-rep1	CVT185	V1	E-L35	21417248	14702014	68.65
185-V1-35-rep2	CVT185	V1	E-L35	28445849	21231423	74.64
185-V1-35-rep3	CVT185	V1	E-L35	21434917	17707641	82.61
185-V2-35-rep1	CVT185	V2	E-L35	27257851	21807336	80.00
185-V2-35-rep2	CVT185	V2	E-L35	23177647	17810172	76.84
185-V2-35-rep3	CVT185	V2	E-L35	11416930	9416204	82.48
185-V3-35-rep1	CVT185	V3	E-L35	20411482	17522382	85.85
185-V3-35-rep2	CVT185	V3	E-L35	27019798	20913973	77.40
185-V3-35-rep3	CVT185	V3	E-L35	21750560	16511582	75.91

Table S3. continue.

Sample description	Clone	Vineyard	Stage	# Total reads count	# Mapped reads count	% mapped (RSEqc)
71-V1-38-rep1	CVT71	V1	E-L38	30730202	23989126	78.06
71-V1-38-rep2	CVT71	V1	E-L38	25197139	21165935	84.00
71-V1-38-rep3	CVT71	V1	E-L38	22519194	18789430	83.44
71-V2-38-rep1	CVT71	V2	E-L38	22428356	18774058	83.71
71-V2-38-rep2	CVT71	V2	E-L38	33698559	28040384	83.21
71-V2-38-rep3	CVT71	V2	E-L38	24359276	20447610	83.94
71-V3-38-rep1	CVT71	V3	E-L38	26516734	21916410	82.65
71-V3-38-rep2	CVT71	V3	E-L38	19333524	16280801	84.21
71-V3-38-rep3	CVT71	V3	E-L38	24324132	20525407	84.38
423-V1-38-rep1	CVT423	V1	E-L38	20468782	17236645	84.21
423-V1-38-rep2	CVT423	V1	E-L38	19077420	16348003	85.69
423-V1-38-rep3	CVT423	V1	E-L38	18901343	15843518	83.82
423-V2-38-rep1	CVT423	V2	E-L38	18830343	15830752	84.07
423-V2-38-rep2	CVT423	V2	E-L38	18195317	15338173	84.30
423-V2-38-rep3	CVT423	V2	E-L38	16470356	13885880	84.31
423-V3-38-rep1	CVT423	V3	E-L38	25208424	21193048	84.07
423-V3-38-rep2	CVT423	V3	E-L38	24119305	20456013	84.81
423-V3-38-rep3	CVT423	V3	E-L38	24963999	17856337	71.53
185-V1-38-rep1	CVT185	V1	E-L38	24298739	20318169	83.62
185-V1-38-rep2	CVT185	V1	E-L38	20473491	17181741	83.92
185-V1-38-rep3	CVT185	V1	E-L38	18675268	15689564	84.01
185-V2-38-rep1	CVT185	V2	E-L38	18334506	15198558	82.90
185-V2-38-rep2	CVT185	V2	E-L38	16999713	13971889	82.19
185-V2-38-rep3	CVT185	V2	E-L38	19808946	15904729	80.29
185-V3-38-rep1	CVT185	V3	E-L38	20864195	16188423	77.59
185-V3-38-rep2	CVT185	V3	E-L38	25710269	19348751	75.26
185-V3-38-rep3	CVT185	V3	E-L38	25845399	17712791	68.53
			**Average***	**23187874**	**19040427**	**82.34**

***Average values are calculated over the 81 libraries analyzed. Conversion between the modified E-L scheme and the extended BBCH scheme is as it follows: E-L31 = 75 BBCH; E-L35 = 81 BBCH; E-L38 = 89 BBCH.

Table S4. Percentage of RNA-seq unmapped reads aligned against the genome of the three main grapevine fungal pathogens. After mapping the sequencing libraries against the *Vitis vinifera* reference genome (GGDB 12X V1), the remaining unmapped reads were grouped for each vineyard and then used to detect sequences of fungal pathogens. For each vineyard, results related to the presence of the pathogen sequences are shown as percentage of a subset (27M reads) of unmapped reads (the corresponding number of reads is reported in brackets).

	Botrytis cinerea	*Erysiphe necator*	*Plasmopara viticola*
**V1**	1.45% (391524)	1.35% (364533)	0.00% (0)
**V2**	1.37% (369913)	1.26% (340207)	0.00% (0)
**V3**	1.81% (488702)	1.71% (461718)	0.00% (0)

The following supplemental tables are provided separately due to file dimension.

Table S5. Functional categories of genes expressed in 'Nebbiolo' berries collected at three developmental stages (E-L31, E-L35, E-L38) in different vineyards (V1, V2, V3) from three clones (CVT185, CVT423, CVT71). Values (FPKM) are the mean of three biological replicates. Conversion between the modified E-L scheme and the extended BBCH scheme is as it follows: E-L31 = 75 BBCH; E-L35 = 81 BBCH; E-L38 = 89 BBCH.

Table S6. Description of the 112 gene clusters covering the 80% of the explained variability associated to RNA-seq results.

Table S7. Oligonucleotides used in this study for RT-qPCR analysis of target transcripts.

Gene description	Gene ID (VVGDB 12X V1)	Primer	Primer sequence (5'-3')	Product size (bp)	Reference
VvSTS48<br>*Stilbene synthase*	VIT_16s0100g01200	Forward	CTTGAAGGGGGAAAATGCT	160	Vannozzi et al. 2012
		Reverse	TTACTGCATTGAAGGGTAAACC		
VvSTS16/22<br>*Stilbene synthase*	VIT_16s0100g00920	Forward	CTTTTGACCCAATTGGAATCAAC	157	Vannozzi et al. 2012
		Reverse	TGACATGTTCCCATATTCACTTAG		
Vv3AT<br>*Anthocyanin acyltransferase*	VIT_03s0017g00870	Forward	AGTGAGTCGCGAGGATGTGTTGT	155	Rinaldo et al. 2015
		Reverse	TCCAAGCAGGATTTCCCCAACCA		
VvAOMT<br>*Anthocyanin methyltransferase*	VIT_01s0010g03510	Forward	GATGAATGTCCCTGTCGATGAG	119	Hugheney et al. 2009
		Reverse	CAAGAGCTGTTGCCAAGAGA		
VvUFGT<br>*UDP-glucose:flavonoid-3-O-glucosyltransferase*	VIT_16s0039g02230	Forward	AATCTGAGAGCCCTAAGAGA	130	Goto-Yamamoto et al. 2002
		Reverse	GGTGGTACAAGCAACAGTTC		
VvNCED1<br>*9-cis-epoxycarotenoid dioxygenase*	VIT_19s0093g00550	Forward	GGTGGTGAGCCTCTGTTCCT	132	Ferrero et al. 2018
		Reverse	CTGTAAATTCGTGGCGTTCACT		
VvGST4<br>*Glutathione-S-transferase*	VIT_04s0079g00690	Forward	GAGAAGGGCGTGGAGTTTGA	90	This work
		Reverse	CCCAAAAGGCTGTCGAAGGA		
VvAM1<br>*AnthoMATE transporter*	VIT_16s0050g00930	Forward	TGCTTTTGTGATTTTGTTAGAGG	164	Gomez et al. 2009
		Reverse	CCCTTCCCCGATTGAGAGTA		
VvABCC1<br>*ABC transporter*	VIT_16s0050g02480	Forward	AATTCAAAGATTGGAAGC	163	Francisco et al. 2013
		Reverse	GCACTGATTTTGAATAGAA		
VvHT1<br>*Hexose transporter*	VIT_00s0181g00010	Forward	TTCAGGGTTTGCATGGTCCT	83	This work
		Reverse	GTGCAGCAGATCGGATTTCC		
VvHT2<br>*Hexose transporter*	VIT_18s0001g05570	Forward	TACTTTTCGTGCCGGAGACC	83	This work
		Reverse	AAACCTCCGCCAGAACCAAT		
VvHT6<br>*Hexose transporter*	VIT_18s0122g00850	Forward	TTCTTGAAGGTGCCCGAGAC	81	This work
		Reverse	AGTAACCTGCCTTGCTCCAAC		
VvXET32<br>*Xyloglucan-endotransglucosylase*	VIT_06s0061g00550	Forward	CAGGACCCCAAGAGAGACCA	145	This work
		Reverse	ACCCATCACAATGCACTCCA		
VvUBI<br>*Ubiquitin*	VIT_16s0098g01190	Forward	TCTGAGGCTTCGTGGTGGTA	99	Gambino et al 2012
		Reverse	AGGCGTGCATAACATTTGCG		
VvACT<br>*Actin*	VIT_04s0044g00580	Forward	GCCCCTCGTCTGTGACAATG	100	Gambino et al 2012
		Reverse	CCTTGGCCGACCCACAATA		

Fig. S1. Climatic data. Monthly average values of Temperature (T, °C), total rainfall (mm) and Vapour Pressure Deficit (VPD, Pa KPa^{-1}) in 2013 and 2014 in the vineyards of the trial (Langhe, Cuneo Province, North-West Italy). Bars represent standard errors of the mean.

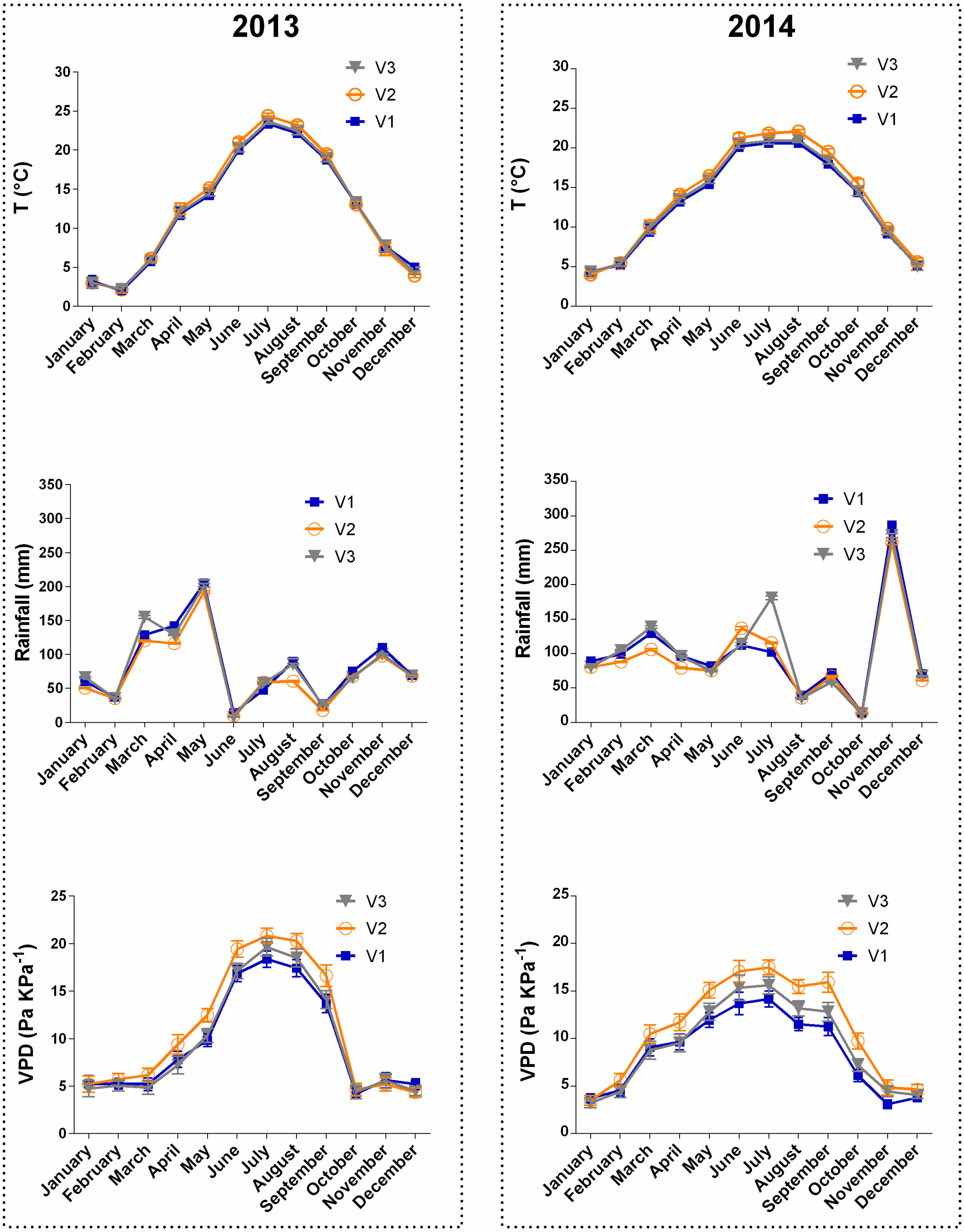

Fig. S2. Correlation between RNA-seq (FPKM) and RT-qPCR results (normalized expression values) obtained from data of the expression analyses performed on transcripts (Table S7) used for validation of RNA-seq results.

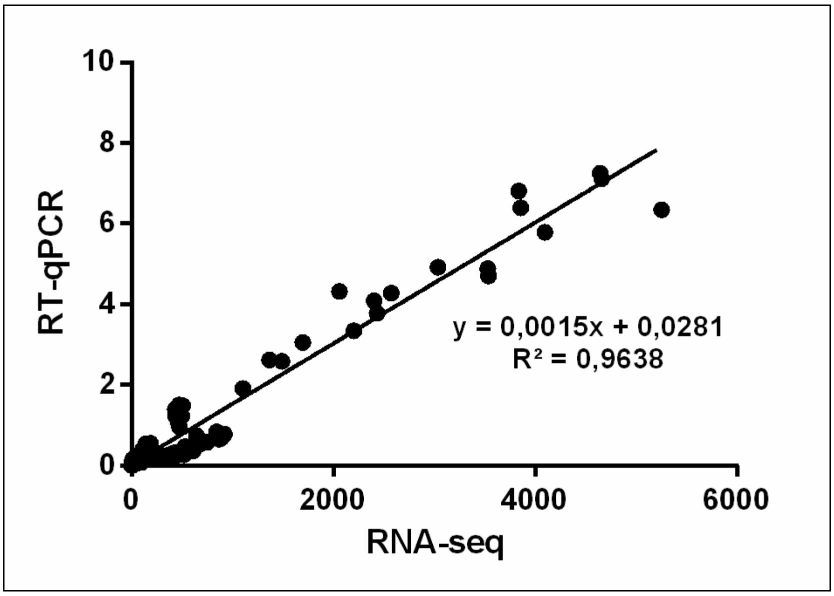

Fig. S3 Environmental effect on lipid, organic acid and hormone metabolism. Schematic representation highlighting how the three considered variables, ripening stage (E-L31, E-L35, E-L38), clone (CVT71, CVT423, CVT185) and vineyard (V1, V2, V3), affect the distribution of samples when genes belonging to A) Lipid metabolic process, B) Organic acid metabolic process and C) Hormone metabolic process were analyzed by Principal Component Analysis (PCA). Conversion between the modified E-L scheme and the extended BBCH scheme is as it follows: E-L31 = 75 BBCH; E-L35 = 81 BBCH; E-L38 = 89 BBCH.

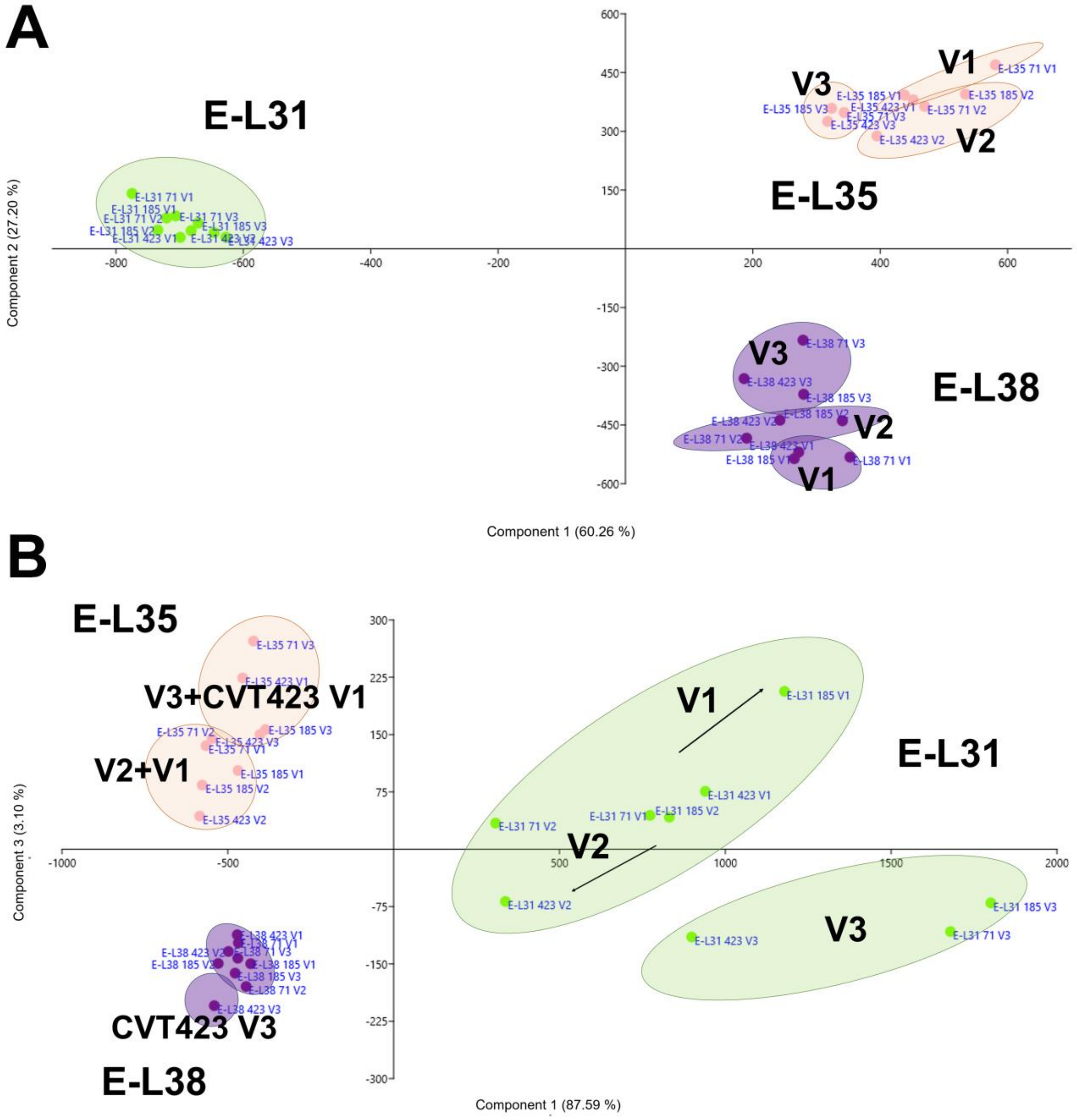

Fig. S3 continue.

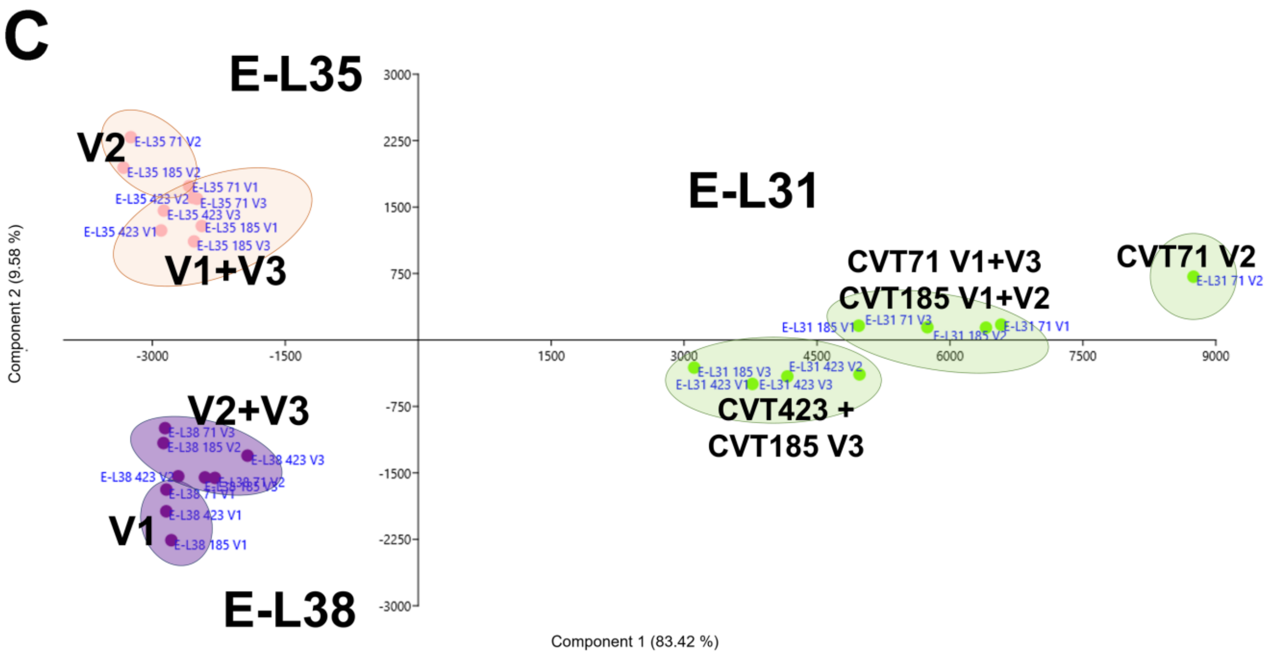

Fig. S4 Clone effect on Nucleic acid and Protein metabolism. Schematic representation highlighting how the three considered variables, ripening stage (E-L31, E-L35, E-L38), clone (CVT71, CVT423, CVT185) and vineyard (V1, V2, V3), affect the distribution of samples when genes belonging to A) Nucleic acid metabolic process and B) Protein metabolic process were analyzed by Principal Component Analysis (PCA). Conversion between the modified E-L scheme and the extended BBCH scheme is as it follows: E-L31 = 75 BBCH; E-L35 = 81 BBCH; E-L38 = 89 BBCH.

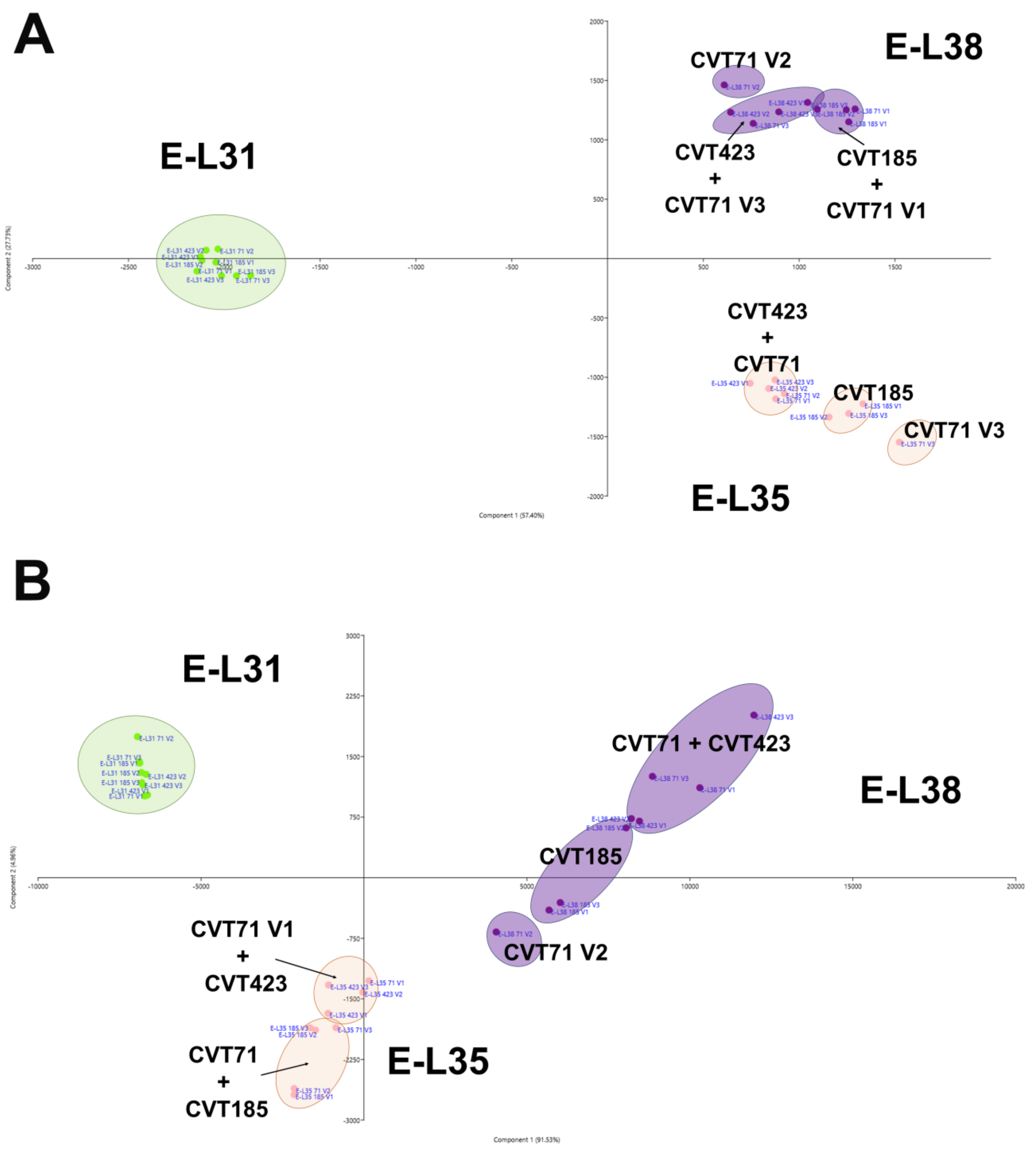

Fig. S5 Environmental effect on secondary metabolism. Schematic representation highlighting how the three considered variables, ripening stage (E-L31, E-L35, E-L38), clone (CVT71, CVT423, CVT185) and vineyard (V1, V2, V3), affect the distribution of samples when genes belonging to Secondary metabolic process were analyzed by Principal Component Analysis (PCA). Conversion between the modified E-L scheme and the extended BBCH scheme is as it follows: E-L31 = 75 BBCH; E-L35 = 81 BBCH; E-L38 = 89 BBCH.

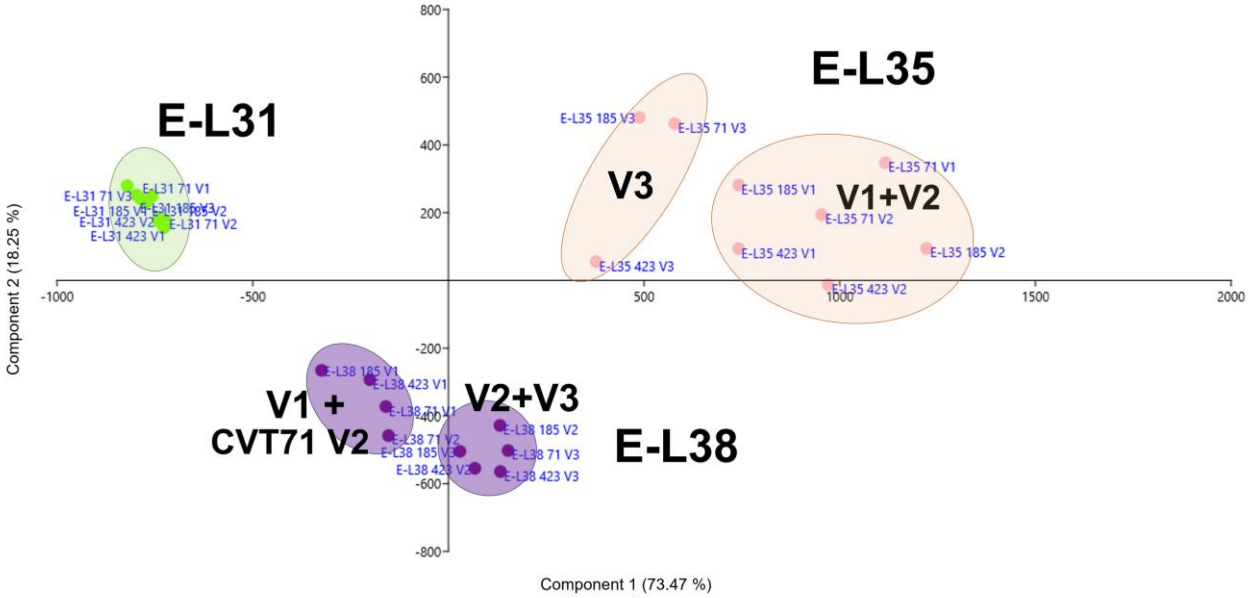

Fig. S6 *C x E* effect on sugar transport. A) PCA analysis and MeV heat map highlighting expression changes occurring in a group of sugar transporter encoding genes in dependence on ripening stage (E-L31, E-L35, E-L38), genotype (CVT71, CVT423, CVT185) and vineyard (V1, V2, V3). Color scale of the heat map chart ranges from black (low expression) to yellow (high expression, FPKM > 100). B-C) Transcriptional trends of a group of hexose transporter genes (*VvHT1*-VIT_00s0181g00010, *VvHT2*-VIT_18s0001g05570, *VvHT6*-VIT_18s0122g00850) in 2013 and 2014 berry samples, respectively. Asterisk or lower case letters denote significant differences attested by Tukey's *HSD* test (*P<0.05*) respectively when values were significant in only one or more samples. Bars represent standard error of the mean (*n=3*). The E-L31, E-L35, E-L38 stages from the modified E-L scheme respectively correspond to stage 75, 81 and 89 in the extended BBCH scheme.

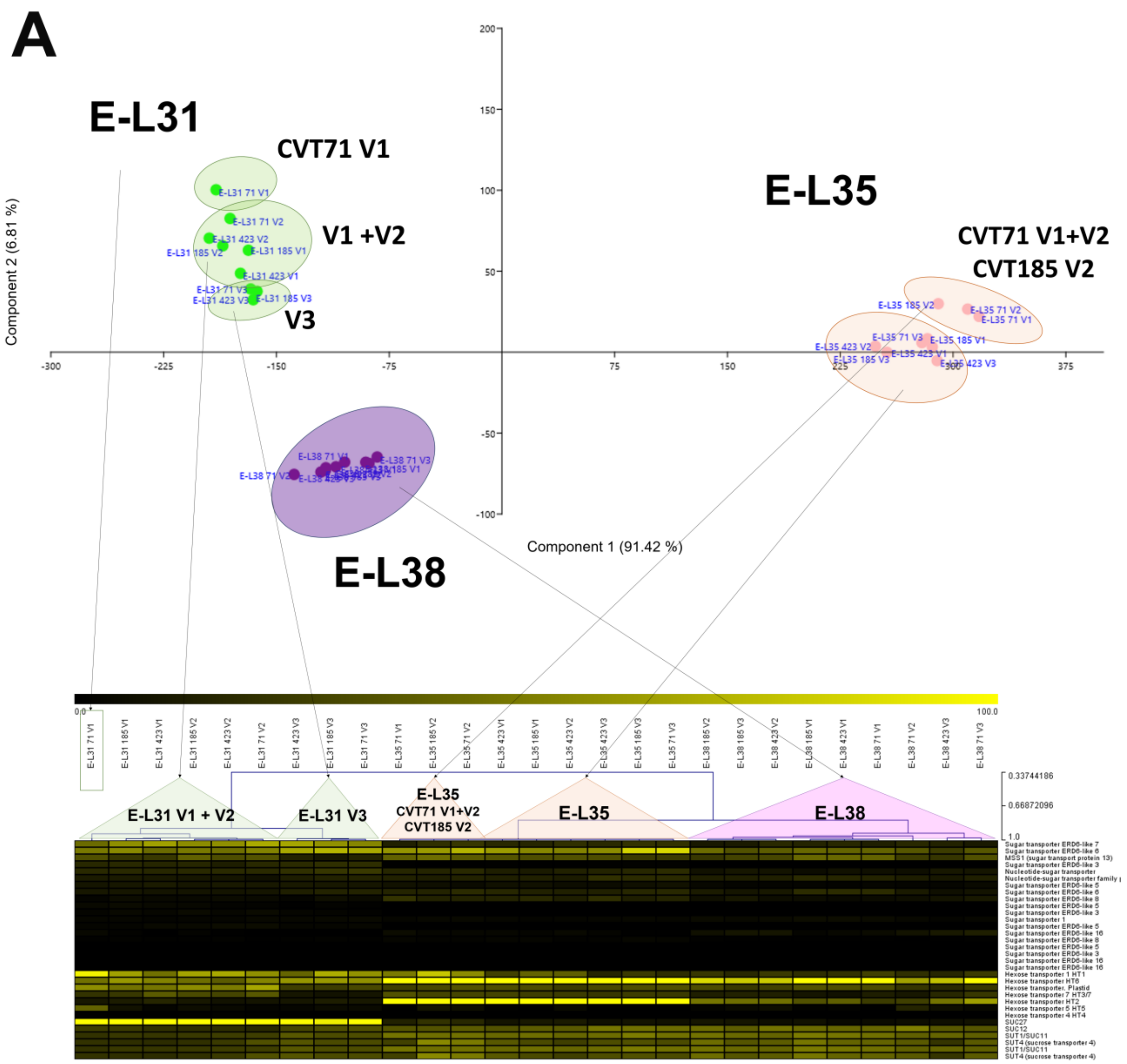

Fig. S6 continue.

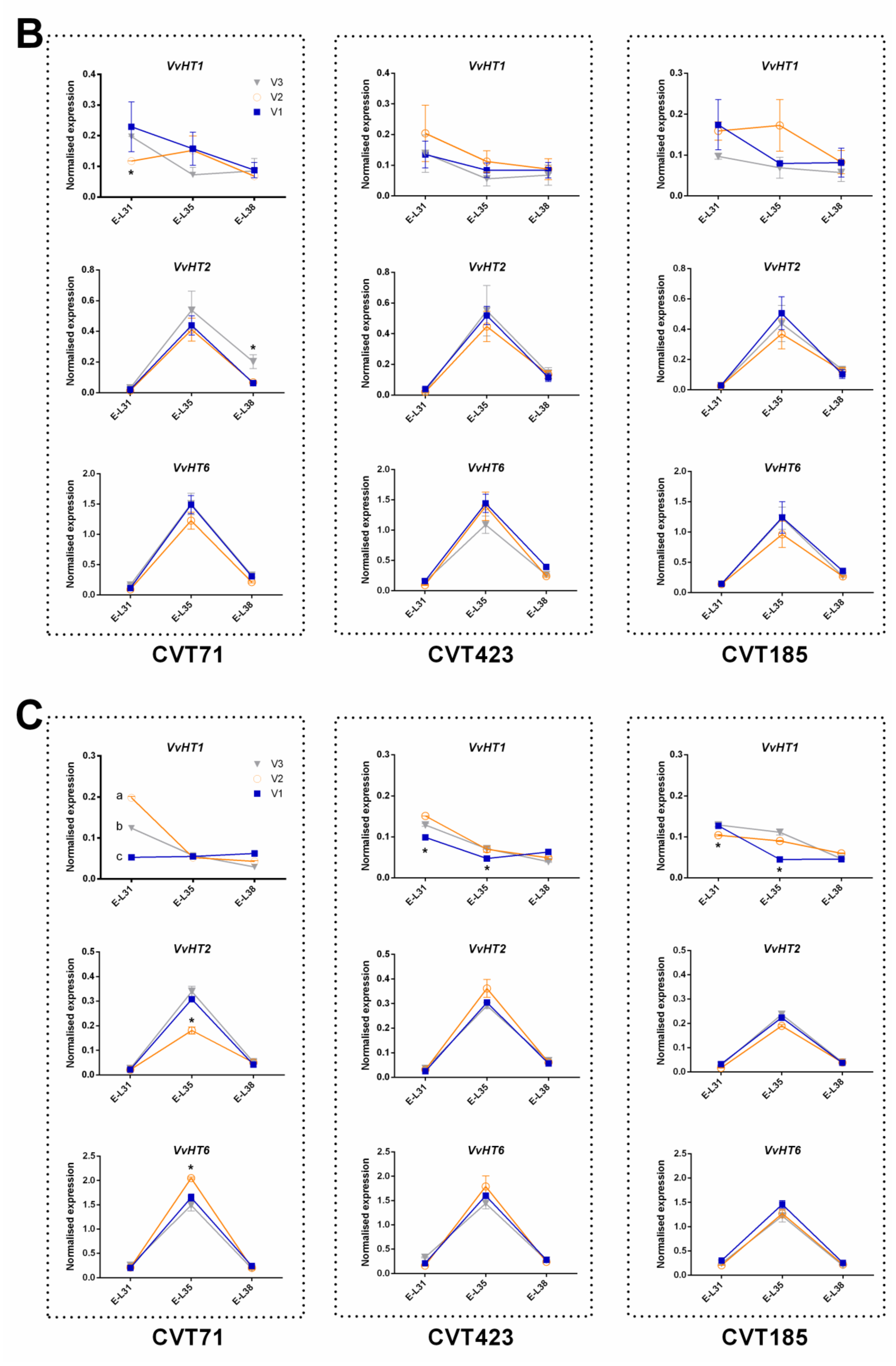

Fig. S7 Focus on abscisic acid (ABA) biosynthesis. RT-qPCR expression profiles of the ABA biosynthetic gene *VvNCED1* (VIT_19s0093g00550) and accumulation pattern of ABA (ng g^{-1}) in berries collected in three vineyards (V1, V2, V3) from CVT71, CVT423 and CVT185 ‘Nebbiolo’ clones at three ripening stages (E-L31, E-L35, E-L38) in 2013 (A) and 2014 (A) years. Asterisk or lower case letters denote significant differences attested by Tukey’s *HSD* test ($P<0.05$) respectively when values were significant in only one or more samples. Bars represent standard error of the mean ($n=3$). The E-L31, E-L35, E-L38 stages from the modified E-L scheme respectively correspond to stage 75, 81 and 89 in the extended BBCH scheme.

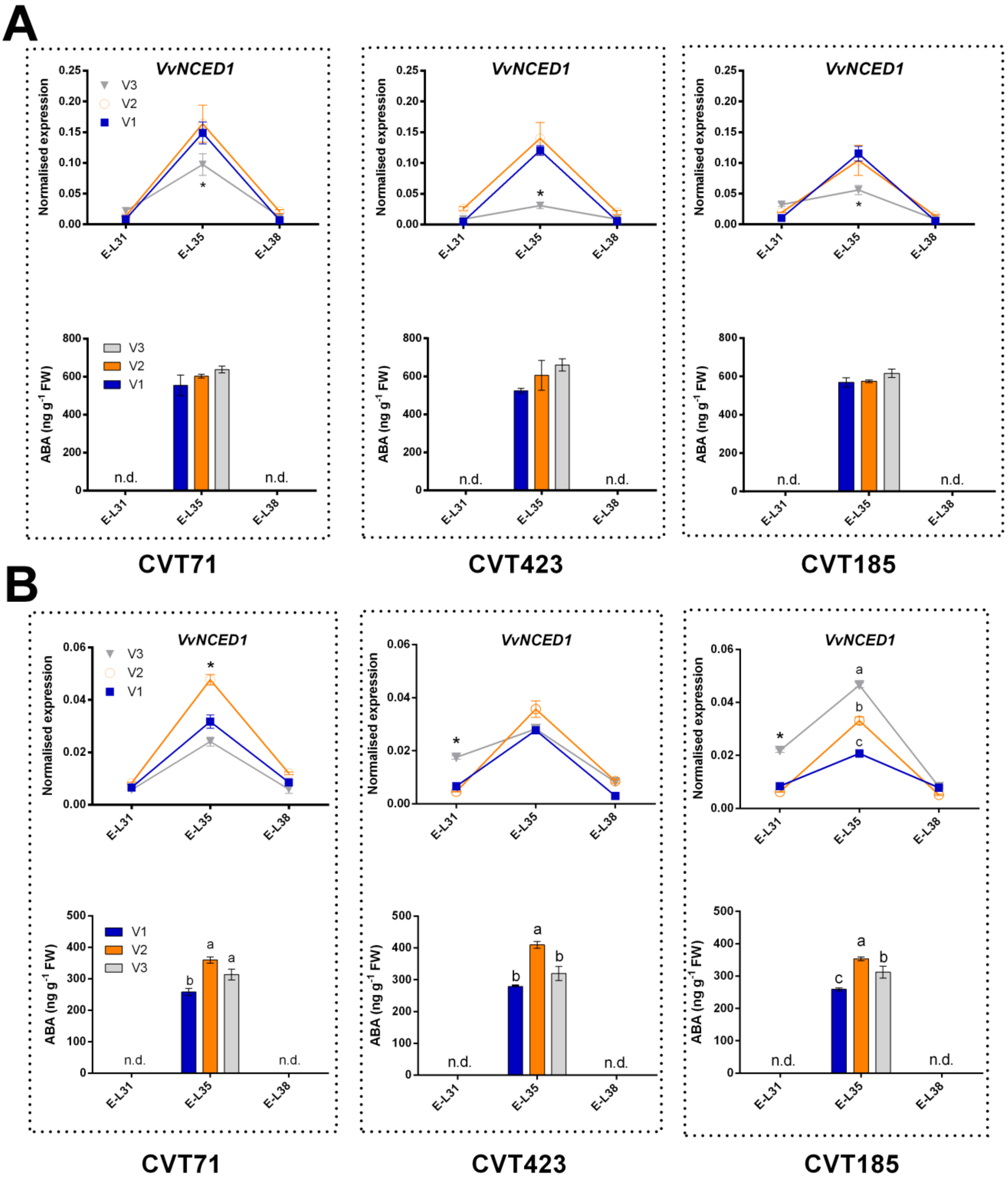

Fig. S8 Focus on anthocyanin metabolism. RT-qPCR expression profiles of genes encoding the main anthocyanin biosynthetic enzymes (A-C) *Vv*UFGT (VIT_16s0039g02230), (D-F) *Vv*AOMT (VIT_01s0010g03510) and (G-I) *Vv*3AT (VIT_03s0017g00870) analyzed in whole berries collected in three vineyards (V1, V2, V3) from CVT71, CVT423 and CVT185 'Nebbiolo' clones at three ripening stages (E-L31, E-L35, E-L38) during the second year of trial (2014). Asterisk or lower case letters denote significant differences attested by Tukey's *HSD* test ($P<0.05$) respectively when values were significant in only one or more samples. Bars represent standard error of the mean ($n=3$). The E-L31, E-L35, E-L38 stages from the modified E-L scheme respectively correspond to stage 75, 81 and 89 in the extended BBCH scheme.

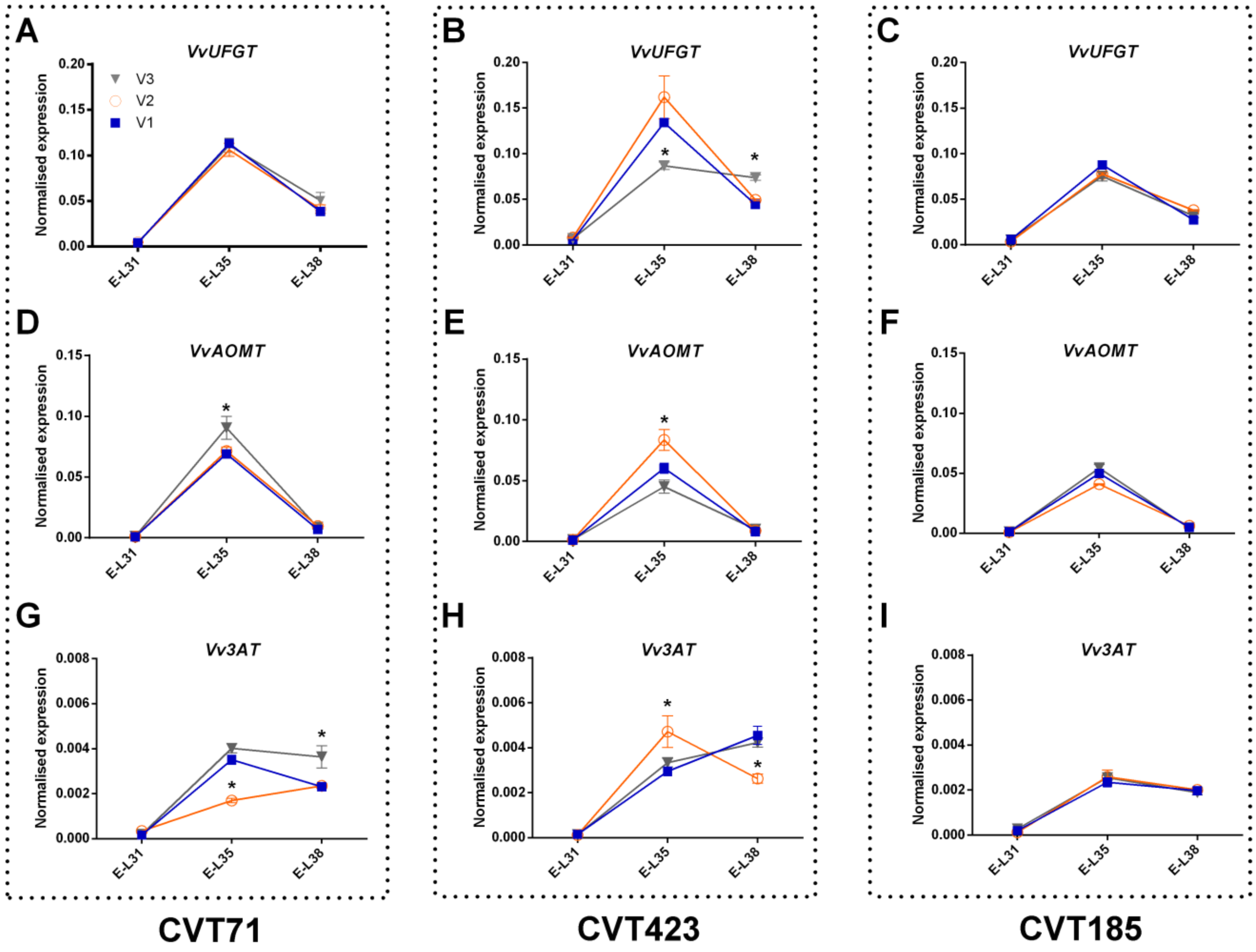

Fig. S9 Focus on anthocyanin transport. Transcriptional levels of genes encoding anthocyanin transporters (A-C and A'-C') *VvABCC1* (VIT_16s0050g02480), (D-F and D'-F') *VvAM1* (VIT_16s0050g00930) and (G-I and G'-I') *VvGST4* (VIT_04s0079g00690) were profiled in berries collected in three vineyards (V1, V2, V3) from CVT71, CVT423 and CVT185 'Nebbiolo' clones at three ripening stages (E-L31, E-L35, E-L38) in 2013 (A-I) and 2014 (A'-I') years. Asterisk or lower case letters denote significant differences attested by Tukey's *HSD* test ($P<0.05$) respectively when values were significant in only one or more samples. Bars represent standard error of the mean ($n=3$). The E-L31, E-L35, E-L38 stages from the modified E-L scheme respectively correspond to stage 75, 81 and 89 in the extended BBCH scheme.

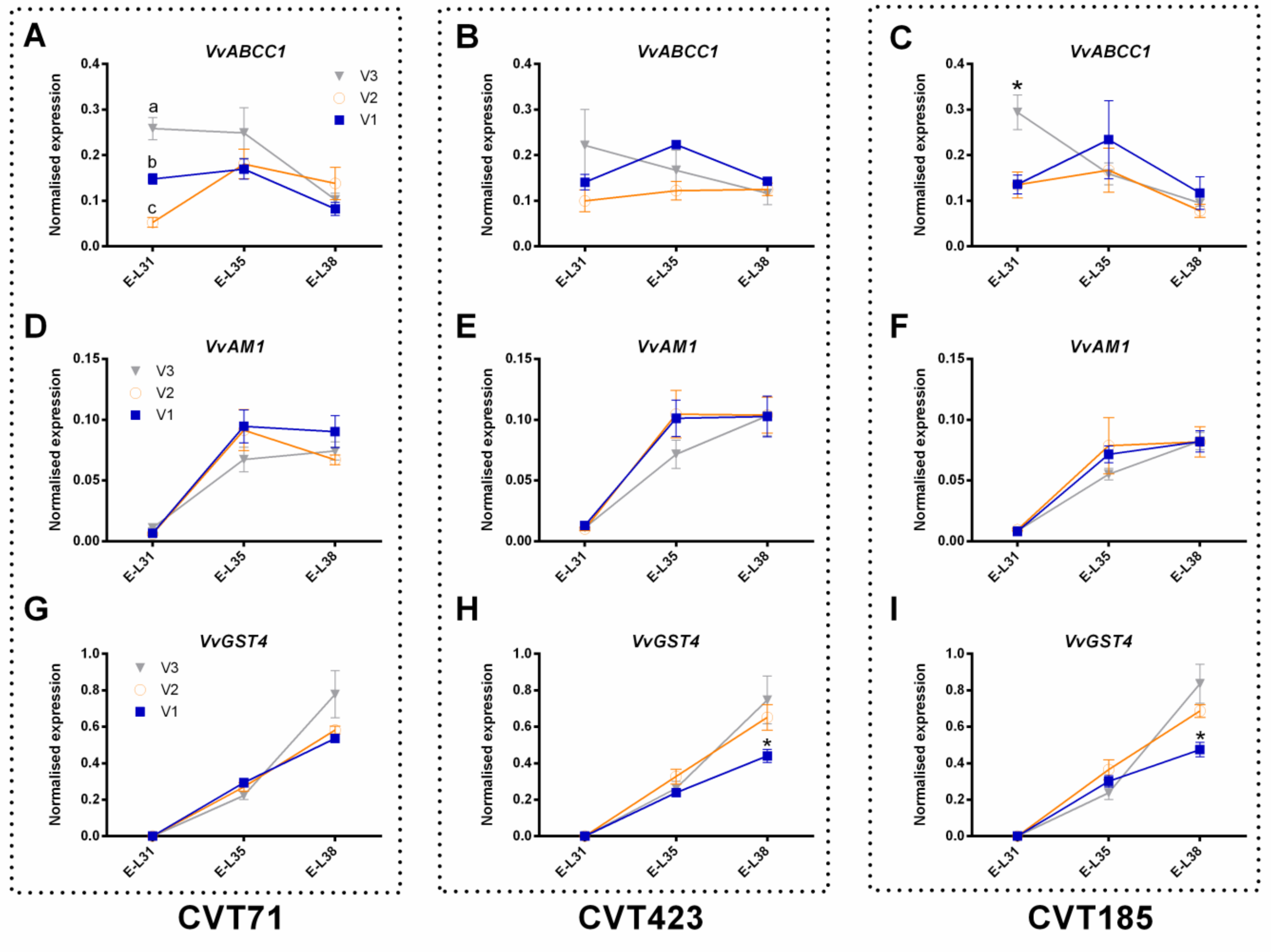

Fig. S9. Continue.

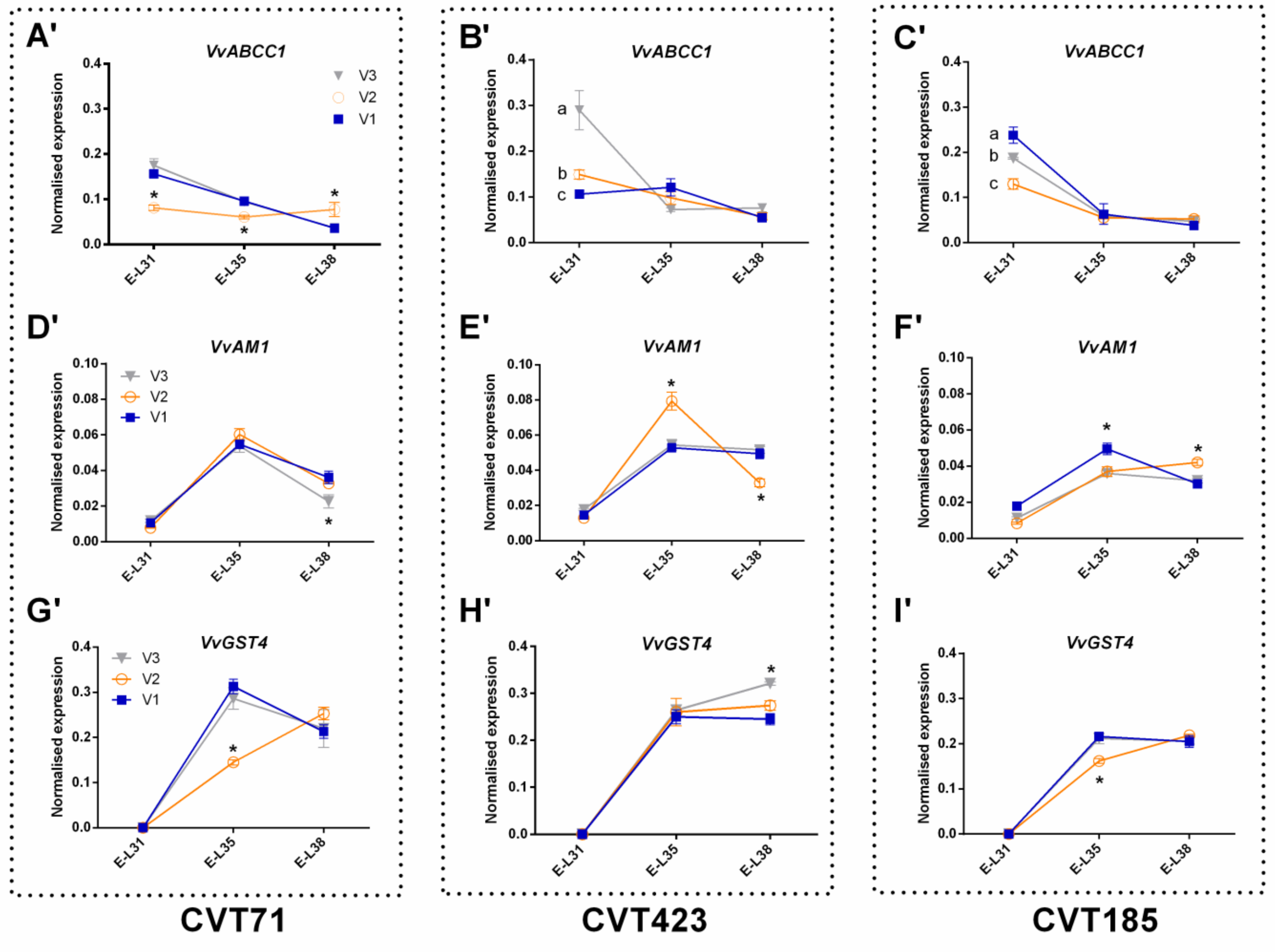

Fig. S10 Focus on cell wall metabolism. A) Heat map displaying the expression changes of transcripts involved in cell wall metabolism. Color scale of the heat map chart ranges from black (low expression) to yellow (high expression, FPKM > 100). RT-qPCR transcriptional profiles of the *VvXET32* (VIT_06s0061g00550) gene (highlighted by the red rectangular in the heat map chart) in berries collected in three vineyards (V1, V2, V3) from CVT71, CVT423 and CVT185 'Nebbiolo' clones at three ripening stages (E-L31, E-L35, E-L38) in 2013 (B) and 2014 (C) years. Asterisk or lower case letters denote significant differences attested by Tukey's *HSD* test (*P<0.05*) respectively when values were significant in only one or more samples. Bars represent standard error of the mean (*n=3*). The E-L31, E-L35, E-L38 stages from the modified E-L scheme respectively correspond to stage 75, 81 and 89 in the extended BBCH scheme.

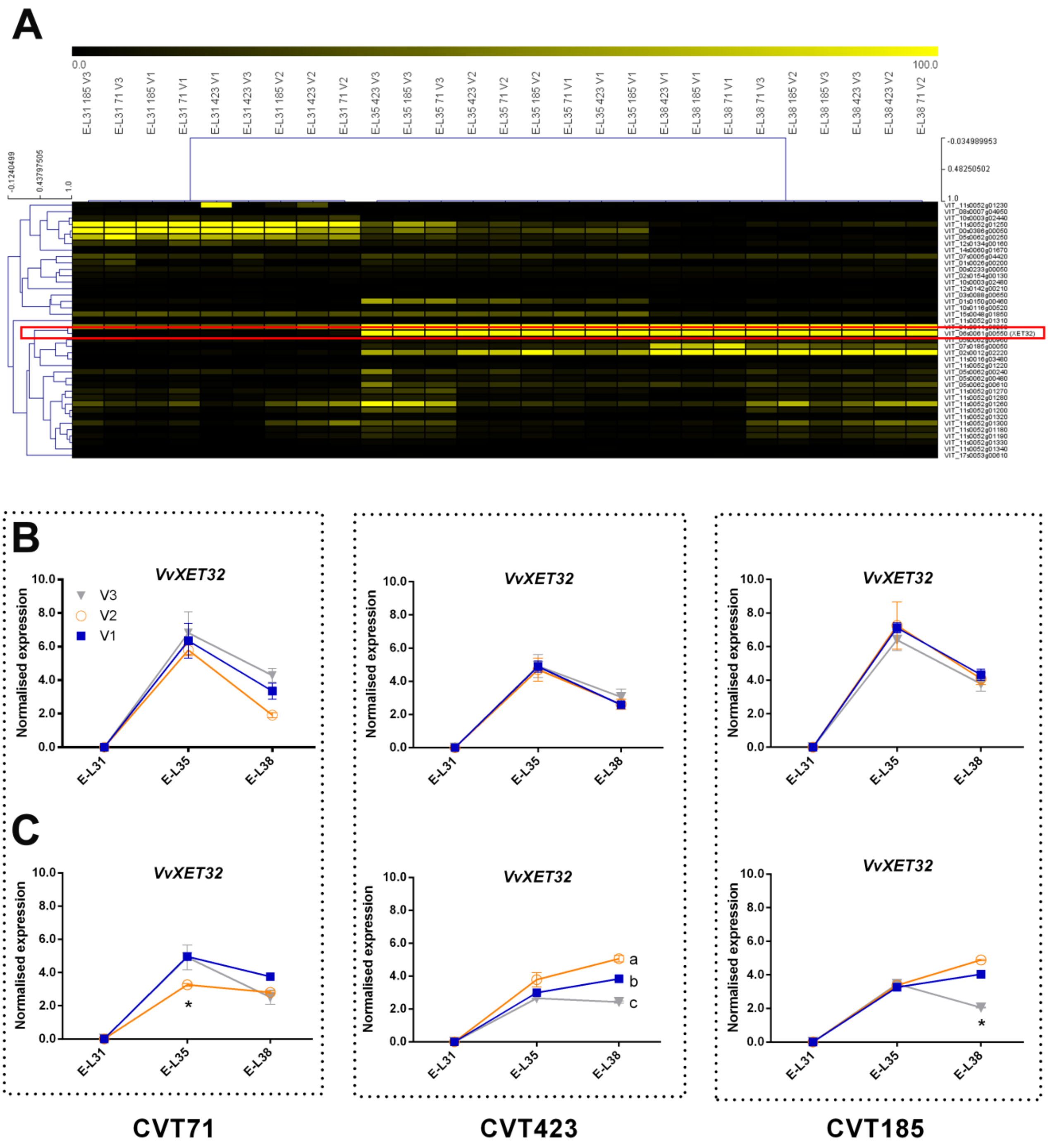

Fig. S11 *C x E* effect on stress response. Schematic representation highlighting how the three considered variables, ripening stage (E-L31, E-L35, E-L38), clone (CVT71, CVT423, CVT185) and vineyard (V1, V2, V3), affect the distribution of samples when genes belonging to Defense and Stress response were analyzed by Principal Component Analysis (PCA). Conversion between the modified E-L scheme and the extended BBCH scheme is as it follows: E-L31 = 75 BBCH; E-L35 = 81 BBCH; E-L38 = 89 BBCH.

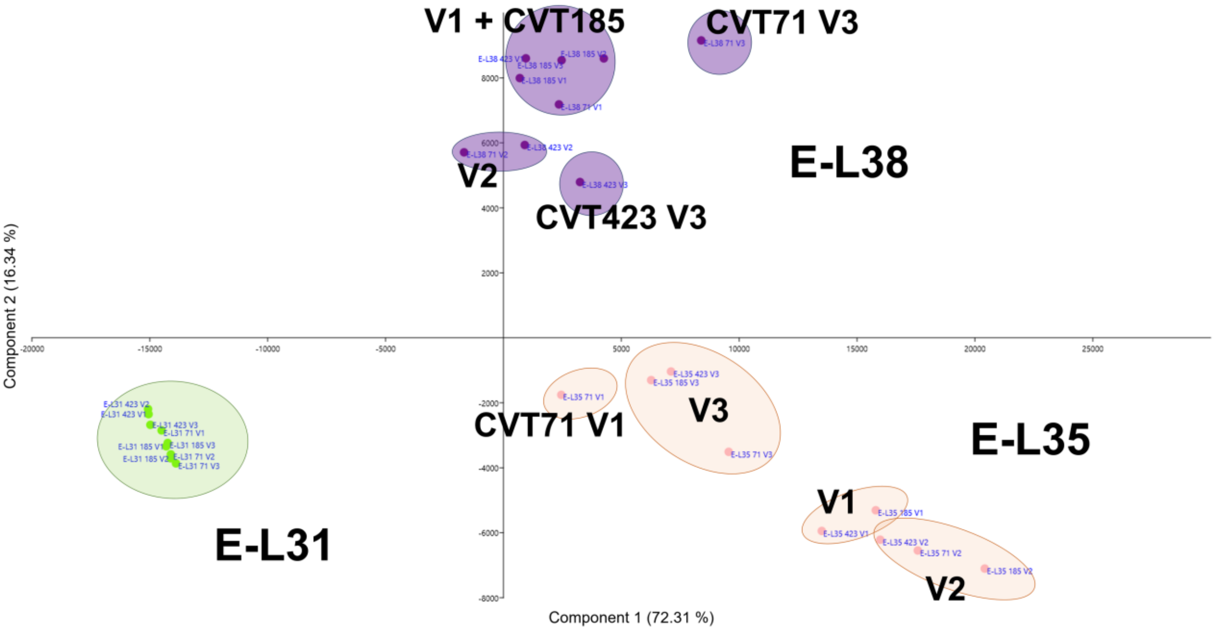

Fig. S12 Focus on stilbenoid metabolism. RT-qPCR expression profiles of genes encoding stilbene synthases (A-C) *Vv*STS48 (VIT_16s0100g01200) and (G-I) *Vv*STS16/22 (VIT_16s0100g00920) and accumulation patterns of the related stilbenoid compounds, resveratrol (D-F) and viniferin (J-L) (both expressed as µg g^{-1}), analyzed on whole berries collected in three vineyards (V1, V2, V3) from CVT71, CVT423 and CVT185 'Nebbiolo' clones at three ripening stages (E-L31, E-L35, E-L38) during the second year of trial (2014). Asterisk or lower case letters denote significant differences attested by Tukey's *HSD* test ($P<0.05$) respectively when values were significant in only one or more samples. Bars represent standard error of the mean ($n=3$). The E-L31, E-L35, E-L38 stages from the modified E-L scheme respectively correspond to stage 75, 81 and 89 in the extended BBCH scheme.

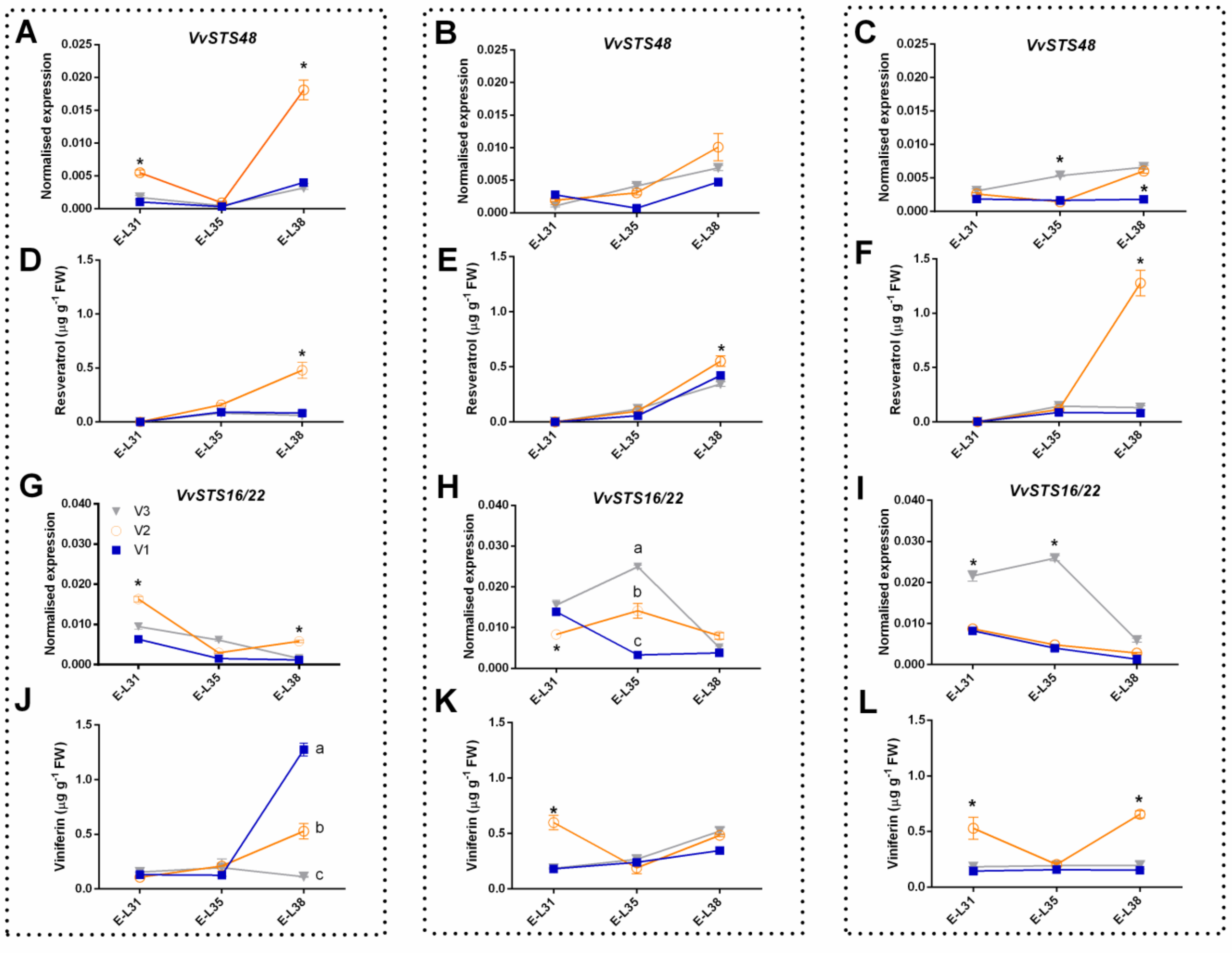

Supplement: Supplementary file 1 [file DataSheet_1.pdf]
