# Supplementary material for: Distinct Metabolic Signals Underlie Clone by Environment Interplay in “Nebbiolo” Grapes Over Ripening
Source: Front Plant Sci. 2019 Dec 4;10:1575. doi: 10.3389/fpls.2019.01575 (PMC6904956; doi:10.3389/fpls.2019.01575)
Supplement: Supplementary file 3 [file DataSheet_3.pdf]

Distinct metabolic signals underlie clone by environment interplay in 'Nebbiolo' grapes over ripening

Chiara Pagliarani[1*], Paolo Boccacci[1], Walter Chitarra[1,2], Emanuela Cosentino[3], Marco Sandri[4], Irene Perrone[1], Alessia Mori[3], Danila Cuozzo[1,5], Luca Nerva[1,2], Marzia Rossato[3], Paola Zuccolotto[6], Mario Pezzotti[3], Massimo Delledonne[3], Franco Mannini[1], Ivana Gribaudo[1], Giorgio Gambino[1]

[1]Institute for Sustainable Plant Protection, National Research Council (IPSP-CNR), Torino. Strada delle Cacce 73, 10135 Torino, Italy.

[2]Council for Agricultural Research and Economics Centre of Viticultural and Enology Research (CREA-VE). Viale XXVIII Aprile 26, 31015 Conegliano (Treviso), Italy.

[3]Department of Biotechnology, University of Verona. Strada le Grazie 15, 37134 Verona, Italy

[4]DMS StatLab, University of Brescia, c.da S. Chiara 50, 25122 Brescia, Italy.

[5]Department of Agricultural, Forest and Food Sciences, University of Torino, Largo Braccini 2, 10095 Grugliasco, TO, Italy

[6]Big&Open Data Innovation Laboratory, University of Brescia, c.da S. Chiara 50, 25122 Brescia, Italy.

*__Correspondence__:

Dr. Chiara Pagliarani

chiara.pagliarani@ipsp.cnr.it

Supplementary Table S6. Detailed description of all the 112 clusters of gene expression defined in this work.

Cluster no. 1

```
## Number of genes in the cluster: 80
## Homogeneity Index:    0.74
## Variable importance for Stage:    Median =  461.5  - Rank =  44
## Variable importance for Clone:    Median =  19.84  - Rank =  49
## Variable importance for Vineyard: Median =  26.45  - Rank =  74
##
## Gene ID               Gene Annotation
## VIT_09s0002g01770  -  N-myristoyl transferase
## VIT_04s0023g03520  -  Cold-shock DNA-binding
## VIT_03s0097g00660  -  Zinc finger (C3HC4-type ring finger)
## VIT_00s0533g00040  -  Cytotoxin precursor (Leucocidin)
## VIT_17s0000g07200  -  Flavonoid 3'-hydroxylase a (F3'Ha) [Vitis vinifera]
## VIT_00s0174g00310  -  Ribonuclease T2
## VIT_09s0002g01050  -  Ring-box protein RBX
## VIT_05s0020g00570  -  Signal recognition particle subunit 19 SRP19
## VIT_08s0007g03890  -  Brix domain-containing protein
## VIT_08s0007g02330  -  Unknown
## VIT_01s0127g00290  -  Unknown protein
## VIT_15s0021g02490  -  Protein transport protein Sec61 subunit beta
## VIT_01s0011g01030  -  H(+)-ATPase 6 AHA6
## VIT_13s0067g02180  -  CTV.12
## VIT_13s0064g00450  -  Cytochrome b5
## VIT_14s0030g01350  -  WD-repeat protein 48
## VIT_10s0003g04580  -  Accelerated cell death 11 (ACD11)
## VIT_16s0039g02660  -  Unknown protein
## VIT_18s0001g00330  -  Unknown protein
## VIT_11s0016g03110  -  Import inner membrane translocase subunit Tim17, Mitochondrial
## VIT_09s0002g05160  -  IAA17
## VIT_17s0000g07210  -  Flavonoid 3'-hydroxylase b (F3'Hb) [Vitis vinifera]
## VIT_11s0037g01420  -  Calcium-dependent protein kinase 13 CPK13
## VIT_08s0040g00400  -  Aquaporin SIP2;1
## VIT_11s0016g05430  -  Unknown protein
## VIT_09s0002g05770  -  Histone H2A variant 3 HTA9
## VIT_18s0001g15050  -  Protein phosphatase 2C
## VIT_17s0000g05930  -  No hit
## VIT_08s0007g01280  -  Substrate carrier, Mitochondrial
## VIT_00s0566g00020  -  Unknown protein
## VIT_03s0088g00230  -  Replication protein A 70 kDa DNA-binding subunit
## VIT_07s0005g04370  -  Unknown protein
## VIT_00s1339g00010  -  Ribonuclease II
## VIT_19s0014g01200  -  No hit
## VIT_01s0011g04830  -  Extensin
## VIT_07s0031g02500  -  RNA polymerase II transcriptional coactivator KIWI
## VIT_17s0000g10050  -  Phosphopantothenoylcysteine decarboxylase
## VIT_16s0039g01640  -  Unknown protein
## VIT_08s0058g00320  -  Ubiquitin activating enzyme E1
## VIT_03s0038g03650  -  Small nuclear ribonucleoprotein D3
## VIT_03s0132g00150  -  GTP cyclohydrolase I
## VIT_07s0104g01780  -  Autophagy 18 ATG18a
## VIT_03s0063g02630  -  Unknown protein
## VIT_01s0127g00350  -  DNA-directed RNA Polymerase II subunit L
## VIT_01s0026g00250  -  Splicing factor
## VIT_02s0025g01460  -  No hit
## VIT_12s0028g02660  -  Acyl-CoA oxidase (ACX3)
```

```
## VIT_00s0184g00030  -  forkhead-associated domain-containing protein
## VIT_19s0014g01870  -  Transcription elongation factor 1
## VIT_00s0120g00010  -  Peroxisomal biogenesis factor 11 (PEX11C)
## VIT_01s0026g00430  -  Cytochrome c
## VIT_02s0025g00690  -  Calcium Dependent Protein Kinase (VvCPK1)
## VIT_14s0171g00080  -  Hcr2-0B
## VIT_08s0007g06550  -  Cation efflux family
## VIT_11s0016g00020  -  Translation initiation factor eIF-5A
## VIT_04s0008g06970  -  DnaJ-like zinc-finger protein
## VIT_00s0273g00020  -  Calcium-dependent protein kinase 13 CPK13
## VIT_06s0004g05830  -  DNA-directed RNA polymerase III subunit C19
## VIT_14s0171g00090  -  Uracil phosphoribosyltransferase.
## VIT_11s0037g00210  -  Endomembrane protein 70, TM4 ;
## VIT_06s0004g02190  -  Armadillo/beta-catenin repeat
## VIT_15s0024g00930  -  VRN1 (reduced vernalization response 1)
## VIT_12s0035g00710  -  Unknown
## VIT_17s0000g06270  -  Malate dehydrogenase precursor
## VIT_13s0019g00340  -  Glutaredoxin
## VIT_12s0142g00090  -  Ribose-phosphate pyrophosphokinase 4
## VIT_15s0021g02620  -  Protein transport protein Sec61 subunit beta
## VIT_11s0016g04590  -  ABC transporter g family pleiotropic drug resistance 12 PDR12
## VIT_07s0191g00130  -  Histone H3.2
## VIT_12s0059g01490  -  Unknown protein
## VIT_08s0007g08610  -  No hit
## VIT_07s0130g00480  -  Unknown protein
## VIT_11s0052g00570  -  Nodulin MtN21
## VIT_10s0116g00770  -  Unknown protein
## VIT_15s0021g01650  -  Zinc finger (Ran-binding)
## VIT_05s0020g00700  -  Squamosa promoter-binding protein (VvSBP5)
## VIT_12s0121g00030  -  PHP domain-containing protein
## VIT_09s0096g00660  -  C2-HC type zinc finger protein C.e-MyT1
## VIT_04s0008g04400  -  No hit
## VIT_00s0531g00030  -  C2 domain containing protein
```

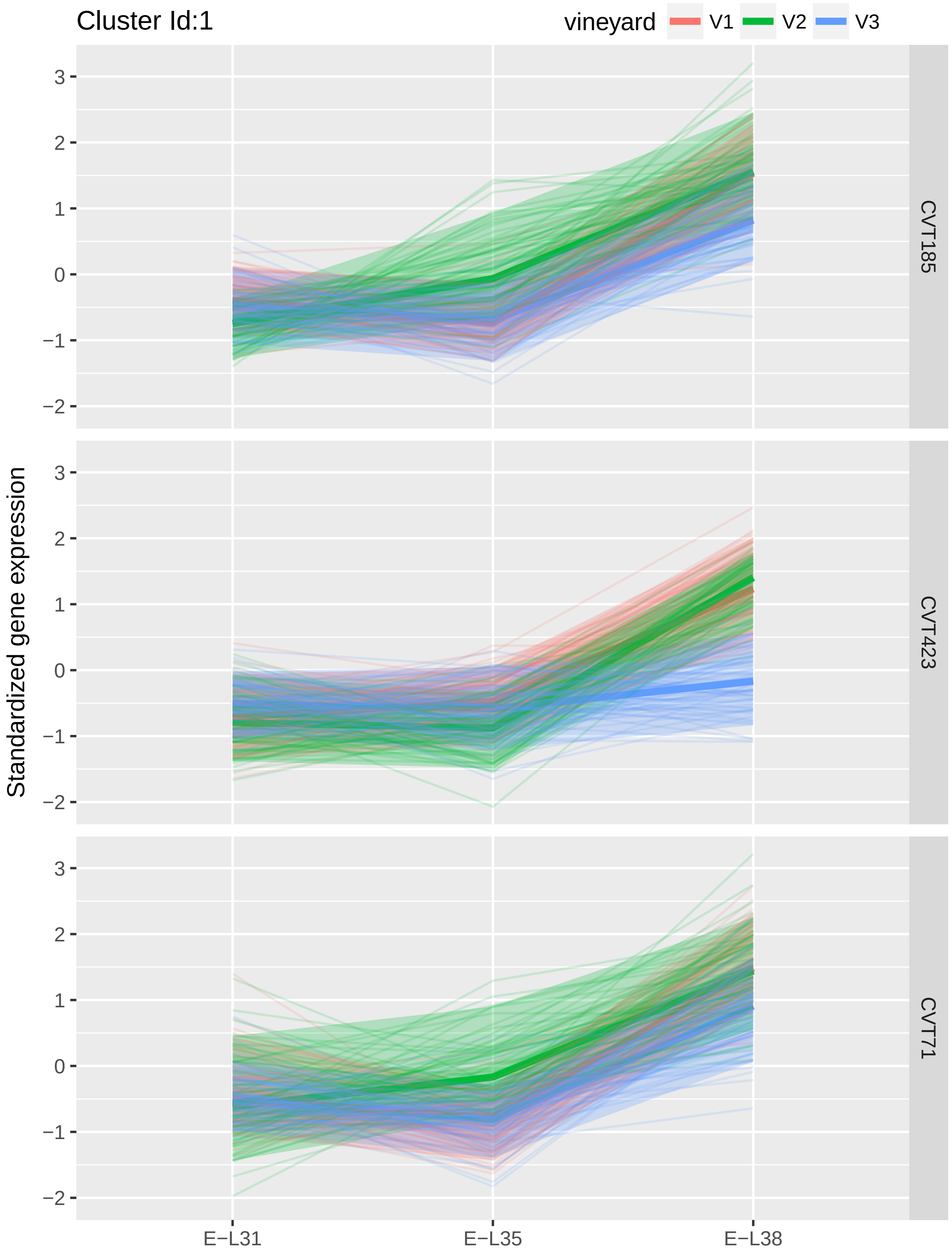

Cluster Id:1
vineyard
V1
V2
V3
Standardized gene expression
3
2
1
0
−1
−2
CVT185
CVT423
CVT71
E−L31
E−L35
E−L38

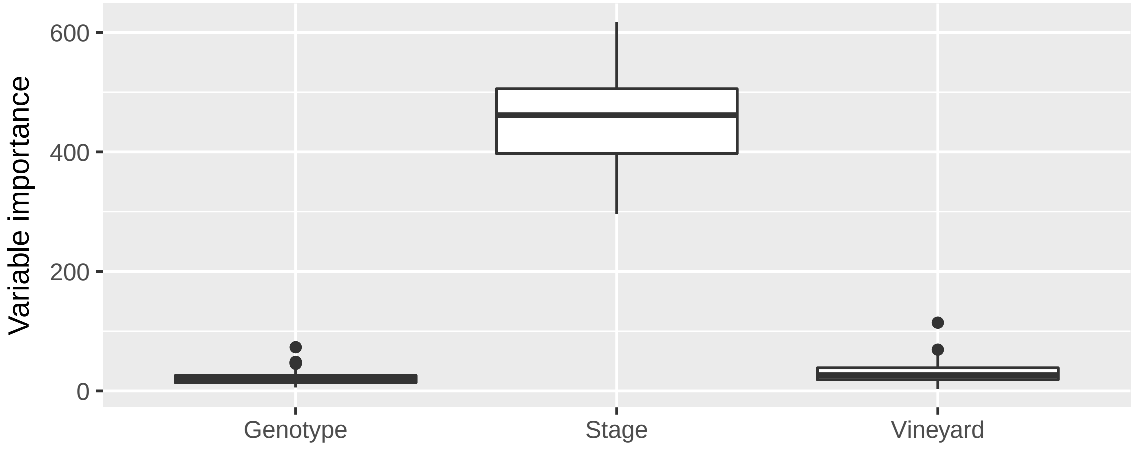

Variable importance
600
400
200
0
Genotype
Stage
Vineyard

Cluster no. 2

```
## Number of genes in the cluster: 241
## Homogeneity Index:    0.84
## Variable importance for Stage:    Median =  619.4  - Rank =  12
## Variable importance for Clone:    Median =  10.07  - Rank =  109
## Variable importance for Vineyard: Median =  12.62  - Rank =  105
##
## Gene ID               Gene Annotation
## VIT_04s0008g01920  -  F-box/LRR-repeat protein 20
## VIT_09s0070g00610  -  Importin beta-3
## VIT_07s0129g00480  -  Molecular chaperone DnaJ
## VIT_10s0116g01690  -  No hit
## VIT_02s0025g00160  -  Unknown
## VIT_05s0094g00860  -  Syntaxin 61
## VIT_03s0063g02560  -  Phenylalanyl-tRNA synthetase alpha chain
## VIT_07s0104g00910  -  Pentatricopeptide (PPR) repeat-containing protein
## VIT_18s0072g01050  -  SNARE associated gtk16 protein
## VIT_09s0018g00660  -  CTV.12
## VIT_13s0067g02210  -  ALPHA-SNAP2 (alpha-soluble nsf attachment protein)
## VIT_08s0007g06290  -  Mitochondrial transcription termination factor-related
## VIT_08s0058g00500  -  Breast cancer susceptibility protein 2b
## VIT_06s0004g06000  -  Rename pre-mRNA-splicing factor RBM22/SLT11
## VIT_02s0087g00100  -  Ankyrin repeat family
## VIT_05s0077g00630  -  No hit
## VIT_01s0010g02690  -  DNA polymerase alpha, subunit B
## VIT_06s0004g04870  -  Unknown protein
## VIT_18s0001g12280  -  Unknown protein
## VIT_08s0007g03880  -  Zinc finger (C2H2 type) family
## VIT_16s0050g02590  -  DnaJ homolog, subfamily B, member 12
## VIT_09s0002g06280  -  Aspartate-glutamate racemase
## VIT_18s0001g13900  -  Unknown protein
## VIT_00s0386g00040  -  Unknown protein
## VIT_17s0000g10100  -  Response regulator ARR11 Type-B
## VIT_11s0016g04300  -  Nudix hydrolase 20, chloroplast precursor (AtNUDT20)
## VIT_01s0011g00910  -  Unknown protein
## VIT_12s0057g01170  -  Pentatricopeptide (PPR) repeat-containing protein
## VIT_01s0010g01710  -  Phytosulfokine receptor
## VIT_18s0001g14750  -  Zinc finger (FYVE type) VPS19
## VIT_18s0001g06970  -  DnaJ homolog, subfamily A, member 3
## VIT_06s0004g06590  -  PRLI-interacting factor N
## VIT_14s0060g02210  -  Translation initiation factor IF-2
## VIT_19s0015g00160  -  Agenet domain-containing protein
## VIT_00s0299g00060  -  MYB divaricata
## VIT_07s0005g03720  -  VPS2.3 SNF7
## VIT_18s0001g05620  -  Carrier protein, Mitochondrial
## VIT_11s0016g04340  -  Unknown protein
## VIT_12s0035g01810  -  Mitotic checkpoint MAD1
## VIT_01s0011g00450  -  Magnesium transporter CorA
## VIT_08s0105g00370  -  myb family
## VIT_04s0008g04340  -  Glycoprotein, Mitochondrial
## VIT_18s0001g15320  -  SEU3B protein
## VIT_01s0011g05830  -  ARR1 typeB
## VIT_06s0004g03440  -  Ulp1 protease
## VIT_00s0181g00090  -  WD-repeat protein
## VIT_13s0047g01040  -  Pentatricopeptide (PPR) repeat-containing protein
```

```
## VIT_01s0026g01240  -  Beta-1 3 galactosyltransferase 5
## VIT_09s0002g04700  -  Transcription factor
## VIT_14s0083g00750  -  Unknown protein
## VIT_15s0048g00930  -  ATP-dependent DNA helicase 2 subunit 2 ku80
## VIT_05s0020g04720  -  Magnesium transporter CorA-like family protein
## VIT_14s0066g01440  -  Homeodomain leucine zipper protein HB-1 (VvATHB-4)
## VIT_11s0149g00150  -  No hit
## VIT_02s0025g00030  -  Zinc finger (C3HC4-type ring finger)
## VIT_06s0004g05990  -  Rename pre-mRNA-splicing factor RBM22/SLT11
## VIT_13s0067g03360  -  Adenylate kinase
## VIT_18s0001g02490  -  PAB3 (poly(A) binding protein 3)
## VIT_03s0091g00740  -  Vacuolar sorting receptor 3
## VIT_18s0001g08850  -  Calcium-transporting ATPase 3, endoplasmic reticulum-type ECA3
## VIT_06s0061g01180  -  PHD finger transcription factor
## VIT_09s0018g02080  -  Coatomer beta subunit
## VIT_13s0019g05390  -  Zinc-binding protein
## VIT_00s0187g00310  -  V-type H+-transporting ATPase subunit H
## VIT_03s0038g04150  -  FAR1-related sequence 9
## VIT_05s0077g01790  -  Triacylglycerol lipase
## VIT_14s0128g00890  -  SNF4 (Sucrose NonFermenting 4)
## VIT_06s0004g00380  -  Unknown protein
## VIT_17s0000g08940  -  FKBP12 interacting protein (FIP37)
## VIT_15s0048g00900  -  Proteasome 20S beta subunit F1 (PBF1)
## VIT_05s0094g00400  -  Signal recognition particle subunit 14 SRP14
## VIT_00s1623g00010  -  Coiled-coil domain-containing protein 94
## VIT_14s0108g01230  -  Snf1 protein kinase (KIN10) (SKIN10)
## VIT_03s0038g01470  -  ER lumen protein retaining receptor
## VIT_04s0008g05840  -  DOMON domain containing protein
## VIT_16s0022g00490  -  Conserved oligomeric Golgi complex 7
## VIT_03s0038g04400  -  Dehydrin (VvDHN4)
## VIT_08s0007g04970  -  Pentatricopeptide (PPR) repeat-containing
## VIT_06s0004g05820  -  Import inner membrane translocase subunit Tim10, Mitochondrial
## VIT_05s0077g01040  -  Peptidylprolyl isomerase domain and WD repeat-containing protein 1
## VIT_08s0007g06540  -  Nudix hydrolase 23, chloroplast precursor (AtNUDT23)
## VIT_17s0000g09290  -  Protein kinase ATN1
## VIT_14s0006g00960  -  DNA-repair protein complementing XP-C cells RAD4
## VIT_06s0080g00230  -  Unknown protein
## VIT_18s0001g03370  -  Ribonuclease II family protein
## VIT_08s0040g00320  -  Alpha-L-arabinofuranosidase
## VIT_16s0098g01760  -  TAP46 (2A phosphatase associated protein of 46 KD)
## VIT_16s0098g00880  -  Unknown protein
## VIT_08s0040g01460  -  Myb TKI1 (TSL-kinase  interacting  protein 1)
## VIT_10s0042g00180  -  Unknown protein
## VIT_01s0010g03650  -  Alkylated DNA repair protein alkB
## VIT_14s0108g01360  -  Unknown protein
## VIT_14s0066g02330  -  Histone deacetylase complex, SIN3 component
## VIT_07s0141g00580  -  Glycosyl transferase family 8 protein
## VIT_14s0066g02030  -  Cation efflux system protein, CDF family
## VIT_08s0040g00500  -  Cupin, RmlC-type
## VIT_08s0007g01980  -  Proteasome 26S regulatory subunit (RPN8)
## VIT_07s0005g00250  -  P23 co-chaperone
## VIT_06s0009g01910  -  Calmodulin-7 (CAM7)
## VIT_17s0000g01430  -  Chlororespiratory reduction 2 (CRR2)
## VIT_09s0018g00650  -  Zinc finger protein 830
## VIT_18s0001g13780  -  Cytochrome P450, family 83, subfamily B, polypeptide 1
```

```
## VIT_03s0038g02420  -  Basic Leucine Zipper Transcription Factor (VvbZIP06)
## VIT_07s0005g01600  -  DEAD (Asp-Glu-Ala-Asp) box polypeptide 42
## VIT_17s0000g03630  -  No hit
## VIT_16s0039g02650  -  Vacuolar protein sorting-associated protein 72
## VIT_08s0040g01700  -  Isocitrate dehydrogenase (NAD+) precursor
## VIT_12s0142g00630  -  Calcineurin-like phosphoesterase family
## VIT_13s0067g01630  -  myb domain protein 65
## VIT_01s0026g02630  -  GTP cyclohydrolase I
## VIT_15s0046g00160  -  WD-40 repeat
## VIT_09s0018g00600  -  Magnesium transporter CorA-like family protein (MRS2-1)
## VIT_00s0299g00030  -  E3 ubiquitin-protein ligase HOS1
## VIT_10s0003g04630  -  SIT4 phosphatase-associated
## VIT_00s0120g00020  -  Importin beta-3
## VIT_04s0008g04550  -  Unknown
## VIT_06s0009g01190  -  RNA-binding protein AKIP1
## VIT_14s0108g00870  -  Malate dehydrogenase precursor
## VIT_12s0035g00400  -  ATP-requiring DNA helicase RecQ
## VIT_18s0075g00200  -  ATP-dependent helicase
## VIT_11s0016g03290  -  SNW domain-containing protein 1 SNW1
## VIT_10s0003g01370  -  Unknown protein
## VIT_01s0011g06190  -  Histidine kinase (AHK4) (WOL)
## VIT_04s0008g05690  -  Meprin and TRAFy domain-containing protein
## VIT_02s0025g02360  -  WNK kinase 6
## VIT_17s0000g07090  -  UDP-glucoronosyl and UDP-glucosyl transferase
## VIT_19s0090g01660  -  RNA-binding protein Musashi
## VIT_11s0016g05820  -  CCR4-NOT transcription complex subunit 10
## VIT_14s0081g00460  -  SAC domain phosphoinositide (3,5)P2 phosphatase
## VIT_18s0001g07510  -  No hit
## VIT_08s0105g00350  -  DnaJ homolog, subfamily B, member 4
## VIT_02s0012g01040  -  NAC domain-containing protein (VvNAC13)
## VIT_18s0001g14860  -  SKP1
## VIT_14s0068g01130  -  Zinc finger (C3HC4-type ring finger)
## VIT_13s0019g05240  -  NAC domain-containing protein (VvNAC20)
## VIT_01s0026g01870  -  Avr9/Cf-9 induced kinase 1
## VIT_07s0005g01960  -  Unknown protein
## VIT_06s0080g01160  -  Aspartic Protease (VvAP14)
## VIT_18s0001g11060  -  Calcineurin phosphoesterase
## VIT_00s0187g00270  -  V-type H+-transporting ATPase subunit H
## VIT_00s0483g00030  -  Isochorismatase hydrolase
## VIT_14s0060g01340  -  SAR1 (secretion-associated ras)
## VIT_06s0004g06420  -  Pentatricopeptide (PPR) repeat-containing protein
## VIT_12s0028g02580  -  Unknown protein
## VIT_09s0002g02130  -  CA-responsive protein
## VIT_08s0007g03460  -  YIP1
## VIT_03s0017g00990  -  DNA topoisomerase 2-associated protein PAT1
## VIT_12s0034g02380  -  Glycine hydroxymethyltransferase
## VIT_10s0003g02880  -  Starch synthase protein
## VIT_09s0054g01780  -  HAC1 (P300/CBP acetyltransferase-related protein 2 gene)
## VIT_07s0130g00510  -  Transcription factor related
## VIT_11s0016g04800  -  No hit
## VIT_19s0014g02770  -  Unknown protein
## VIT_12s0035g02190  -  MLO-like protein 13
## VIT_13s0047g01030  -  LysM receptor-like kinase
## VIT_14s0108g00970  -  Pentatricopeptide (PPR) repeat
```

```
## VIT_05s0077g01870  -  Tetratricopeptide repeat (TPR)-containing
## VIT_11s0103g00660  -  ELM2 domain-containing protein
## VIT_17s0000g03640  -  Proline-rich protein precursor
## VIT_06s0004g00570  -  VvMyb5b
## VIT_04s0008g00730  -  Unknown protein
## VIT_05s0029g01220  -  Unknown protein
## VIT_01s0011g01650  -  FK506-binding protein genes family (VvFKBP17-1)
## VIT_14s0083g01170  -  Mitochondrial substrate carrier family protein
## VIT_15s0024g01470  -  Nuclear matrix protein 1
## VIT_00s0269g00040  -  Unknown
## VIT_15s0021g00980  -  Translation initiation factor eIF-4F
## VIT_08s0056g01110  -  Intron-binding protein aquarius
## VIT_01s0010g00990  -  S-adenosylmethionine decarboxylase proenzyme
## VIT_12s0057g00880  -  TAF15b (TBP-associated factor 15b) zf-ranBP
## VIT_11s0118g00260  -  Multimeric translocon complex in the outer envelope membrane 132 TOC13
## VIT_01s0150g00290  -  NEF1 (no exine formation 1)
## VIT_08s0058g01400  -  Phospholipid-translocating ATPase ALA1
## VIT_04s0023g00280  -  Unknown protein
## VIT_14s0030g01150  -  No hit
## VIT_02s0012g01620  -  RNA recognition motif (RRM)-containing
## VIT_16s0039g01020  -  Unknown protein
## VIT_11s0016g04200  -  MEE55 (maternal effect embryo arrest 55)
## VIT_18s0001g13940  -  Pentatricopeptide (PPR) repeat-containing
## VIT_12s0035g01340  -  Unknown protein
## VIT_06s0004g04920  -  RAB5-interacting protein isoform a
## VIT_08s0007g02870  -  DCP1 (decapping 1)
## VIT_14s0036g00750  -  Protein phosphatase 2 (formerly 2A), regulatory subunit B''
## VIT_05s0020g04710  -  Protein transport protein Sec23A
## VIT_01s0146g00100  -  ACT domain containing protein (ACR4)
## VIT_01s0011g04090  -  Unknown protein
## VIT_14s0066g01530  -  No hit
## VIT_01s0011g01270  -  Unknown protein
## VIT_02s0025g00170  -  No hit
## VIT_07s0205g00150  -  WD40 repeat protein
## VIT_01s0011g04680  -  Glycosyltransferase family 14
## VIT_18s0001g07070  -  Phosphoenolpyruvate carboxylase kinase 2 (PEPKR2)
## VIT_14s0068g00390  -  Esterase PIR7B
## VIT_00s0259g00150  -  5'-AMP-activated protein kinase beta-2 subunit
## VIT_06s0004g08300  -  Zinc finger (C3HC4-type ring finger)
## VIT_13s0067g02000  -  Unknown protein
## VIT_01s0010g02380  -  F-box domain containing protein
## VIT_00s0203g00190  -  Nitric oxide synthase interacting protein
## VIT_01s0026g02170  -  Regulator of chromosome condensation (RCC1)
## VIT_01s0026g02140  -  Regulator of chromosome condensation (RCC1)
## VIT_06s0009g00240  -  OEP80 (outer envelope protein of 80 kda)
## VIT_13s0064g01190  -  RNA recognition motif (RRM)-containing protein
## VIT_05s0020g02230  -  Pto kinase interactor
## VIT_07s0151g00770  -  Kelch repeat-containing F-box protein
## VIT_19s0014g03170  -  WD-40 repeat protein
## VIT_08s0007g04430  -  Low expression of osmotically responsive genes 1
## VIT_00s0804g00010  -  Unknown protein
## VIT_03s0063g02310  -  Acidic leucine-rich nuclear phosphoprotein 32 member B
## VIT_16s0050g02610  -  Nucleotidyltransferase
## VIT_16s0100g00360  -  Per1
```

```
## VIT_05s0020g04650  -  ER degradation enhancer, mannosidase alpha-like 1
## VIT_08s0032g00720  -  Unknown protein
## VIT_02s0012g01770  -  Rad1
## VIT_12s0035g01840  -  CAND1 (Cullin-associated and neddylation dissociated, hemivenata)
## VIT_04s0023g03870  -  Vacuolar protein sorting 35
## VIT_06s0080g00830  -  Unknown protein
## VIT_00s0429g00030  -  SET domain-containing protein (TXR7)
## VIT_09s0054g01600  -  Splicing factor YT521-B
## VIT_08s0007g03350  -  Ubiquinol-cytochrome C reductase UQCRX/QCR9
## VIT_02s0012g00510  -  Golgi transport Got1
## VIT_18s0001g04770  -  Apoptotic chromatin condensation inducer 1 ACIN1
## VIT_02s0087g00650  -  Nod factor
## VIT_19s0015g00680  -  E3 ubiquitin-protein ligase HUWE1
## VIT_18s0122g00500  -  Basic Leucine Zipper Transcription Factor (VvbZIP41)
## VIT_09s0002g03680  -  Casein kinase
## VIT_19s0015g01810  -  Unknown protein
## VIT_08s0007g02130  -  CPL2 (CTD phosphatase-like 2)
## VIT_05s0102g00160  -  Zinc finger (Ran-binding)
## VIT_18s0072g00630  -  TUBBY like protein 1 TLP10
## VIT_04s0008g04370  -  Nucleic acid binding
## VIT_15s0107g00440  -  Enhanced disease resistance 2 (EDR2)
## VIT_04s0044g01940  -  Diacylglycerol O-acyltransferase 1
## VIT_03s0017g00980  -  UDP-arabinose 4-epimerase 3
## VIT_10s0116g01920  -  ABC transporter I member 15
## VIT_19s0090g01540  -  Beta-1,2-xylosyltransferase
## VIT_03s0038g02210  -  Choline transporter
## VIT_06s0004g03800  -  DNA-binding protein
## VIT_01s0026g02230  -  Cullin 3a
## VIT_15s0021g00430  -  Protoheme IX farnesyltransferase
## VIT_00s1364g00010  -  Isochorismatase hydrolase
## VIT_01s0010g00980  -  No hit
```

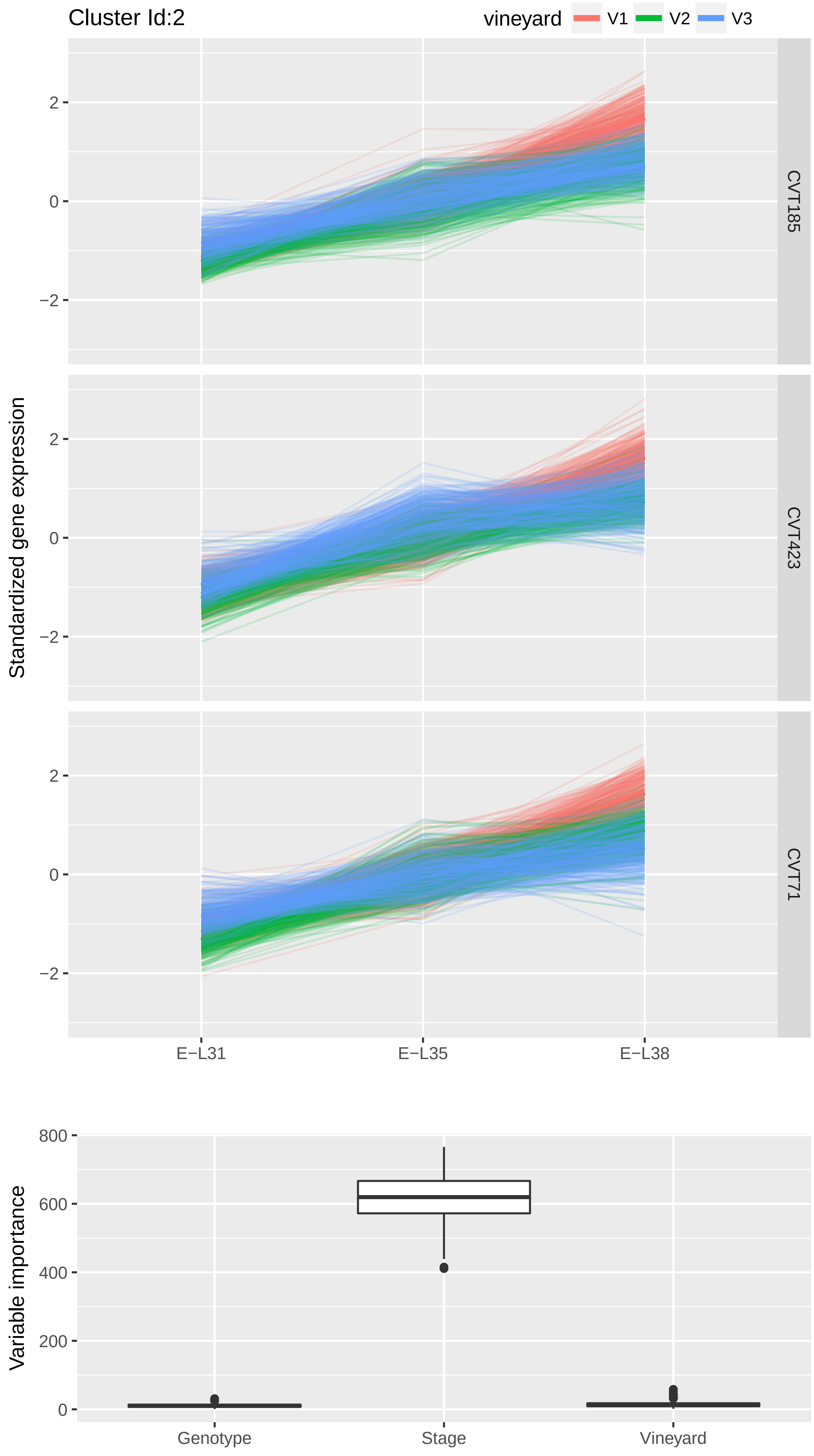
Cluster Id:2
vineyard
V1
V2
V3
Standardized gene expression
2
0
−2
CVT185
CVT423
CVT71
E−L31
E−L35
E−L38
Variable importance
800
600
400
200
0
Genotype
Stage
Vineyard

Cluster no. 3

```
## Number of genes in the cluster: 94
## Homogeneity Index:    0.69
## Variable importance for Stage:    Median =  397.5  - Rank =  55
## Variable importance for Clone:    Median =  18.22  - Rank =  63
## Variable importance for Vineyard: Median =  35.54  - Rank =  58
##
## Gene ID               Gene Annotation
## VIT_14s0030g00180  -  Unknown protein
## VIT_06s0004g01880  -  OEP80 (outer envelope protein of 80 kda)
## VIT_00s0181g00020  -  Armadillo/beta-catenin repeat
## VIT_13s0156g00650  -  Unknown protein
## VIT_15s0046g01070  -  Ubiquitin thiolesterase
## VIT_01s0150g00150  -  Glycyl tRNA synthetase
## VIT_06s0009g02840  -  Flavonoid 3',5'-hydroxylase(F3'5'H)
## VIT_00s0286g00020  -  Curculin (mannose-binding) lectin
## VIT_02s0012g01140  -  Protein kinase
## VIT_18s0117g00190  -  Unknown protein
## VIT_14s0066g00990  -  TRNA methyltransferase complex GCD14 subunit
## VIT_01s0011g01230  -  GTP-binding protein (ERG)
## VIT_06s0009g02810  -  Flavonoid 3',5'-hydroxylase
## VIT_18s0001g11720  -  EMB2756 (embryo defective 2756)
## VIT_18s0122g00990  -  Glutamate receptor Ionotropic
## VIT_05s0124g00230  -  F-box domain containing protein
## VIT_06s0004g07670  -  Unknown protein
## VIT_16s0022g01810  -  AT-hook protein 1
## VIT_01s0026g01000  -  Antiviral helicase SKI2
## VIT_06s0004g00530  -  Origin recognition complex 1b protein
## VIT_19s0027g00340  -  Unknown protein
## VIT_17s0000g07320  -  Unknown protein
## VIT_18s0001g13160  -  Tetrapyrrole methylase
## VIT_13s0019g00890  -  Integral membrane protein, MtN24
## VIT_06s0061g00740  -  Unknown
## VIT_00s0226g00160  -  RabGAP/TBC domain-containing protein
## VIT_17s0000g09860  -  Pentatricopeptide (PPR) repeat-containing protein
## VIT_09s0002g03740  -  Homeobox gene 8
## VIT_02s0025g02900  -  Zinc transporter MTPA2
## VIT_19s0085g00830  -  Ent-kaur-16-ene synthase (VvTPS69)
## VIT_13s0019g00900  -  Integral membrane protein, MtN24
## VIT_18s0001g01590  -  BTB/POZ domain-containing protein
## VIT_18s0001g11850  -  Pentatricopeptide (PPR) repeat-containing protein
## VIT_00s1206g00020  -  WD-40 repeat
## VIT_16s0022g01780  -  Pentatricopeptide (PPR) repeat-containing protein
## VIT_04s0008g05070  -  Pentatricopeptide (PPR) repeat-containing
## VIT_06s0009g01740  -  Geranylgeranyl reductase
## VIT_08s0007g08500  -  Nonsense-mediated mRNA decay NMD3
## VIT_02s0025g01600  -  Harpin-induced 1
## VIT_04s0023g01760  -  Unknown
## VIT_10s0003g00170  -  Dehydration-induced 19
## VIT_08s0007g06960  -  Disease resistance-responsive
## VIT_08s0040g01240  -  basic helix-loop-helix (bHLH) family
## VIT_00s2086g00010  -  1-aminocyclopropane-1-carboxylate oxidase 1
## VIT_04s0079g00290  -  Regulator of chromosome condensation (RCC1)
## VIT_16s0022g01890  -  GTP-binding protein engA
## VIT_11s0016g01830  -  Protein kinase
```

```
## VIT_01s0010g03530  -  Phagocytosis and cell motility protein ELMO1
## VIT_02s0025g02070  -  RWP-RK domain-containing protein
## VIT_18s0001g01530  -  No hit
## VIT_13s0067g01760  -  Nucleoside phosphatase GDA1/CD39
## VIT_02s0234g00030  -  No hit
## VIT_01s0010g03880  -  Unknown protein
## VIT_11s0016g02420  -  UDP-N-acetylglucosamine O-acyltransferase protein
## VIT_06s0004g07920  -  WNK5 (Arabidopsis WNK kinase 5)
## VIT_07s0005g02830  -  DNA-binding protein
## VIT_11s0016g04620  -  PINHEAD (Protein ZWILLE)
## VIT_07s0005g00380  -  Glycosyl hydrolase family 1 protein
## VIT_04s0008g03630  -  Binding
## VIT_04s0023g01160  -  Pentatricopeptide (PPR) repeat-containing
## VIT_12s0035g01820  -  Proton-dependent oligopeptide transport (POT) family protein
## VIT_10s0116g00450  -  Tyrosyl-DNA phosphodiesterase
## VIT_13s0019g03870  -  Substrate carrier , Mitochondrial
## VIT_13s0067g00630  -  Yippee
## VIT_01s0011g00400  -  Nuclear transport factor 2 (NTF2)
## VIT_08s0007g02000  -  Glycerol-3-phosphate acyltransferase 8
## VIT_00s0125g00180  -  Zinc finger (C3HC4-type ring finger)
## VIT_06s0009g03260  -  Unknown protein
## VIT_14s0030g00450  -  Histone H2AXb HTA3
## VIT_19s0085g00010  -  Auxin-regulated protein
## VIT_11s0016g01930  -  Diacylglycerol acyltransferase
## VIT_07s0031g03050  -  GTP-binding protein LepA
## VIT_07s0104g01290  -  TUBBY like protein 3 TLP3
## VIT_06s0004g01610  -  Ethylene-insensitive33 (EIL3)
## VIT_04s0008g03510  -  Lignostilbene-alpha,beta-dioxygenase and related enzymes
## VIT_08s0040g02900  -  Aluminum sensitive 3
## VIT_18s0001g12110  -  No hit
## VIT_07s0005g00960  -  Unknown protein
## VIT_14s0083g01100  -  Alpha-1,4-glucan-protein synthase 1
## VIT_06s0080g00410  -  ferredoxin
## VIT_13s0019g04080  -  Protein transport protein Sec61 subunit beta
## VIT_04s0008g03290  -  Peptidyl-tRNA hydrolase 2
## VIT_09s0002g06010  -  Phospholipase/carboxylesterase
## VIT_18s0164g00070  -  Somatic embryogenesis protein kinase
## VIT_07s0005g00370  -  Glycosyl hydrolase family 1 protein
## VIT_03s0038g04620  -  Isoflavone reductase (synthesis of phytoalexines)
## VIT_14s0030g01700  -  Unknown
## VIT_13s0064g01330  -  Unknown protein
## VIT_01s0011g05390  -  Invertase/pectin methylesterase inhibitor
## VIT_01s0011g03710  -  Thioesterase family
## VIT_05s0077g00530  -  Unknown protein
## VIT_00s0525g00040  -  Pentatricopeptide (PPR) repeat-containing protein
## VIT_03s0091g00960  -  No hit
## VIT_00s0267g00030  -  Unknown
```

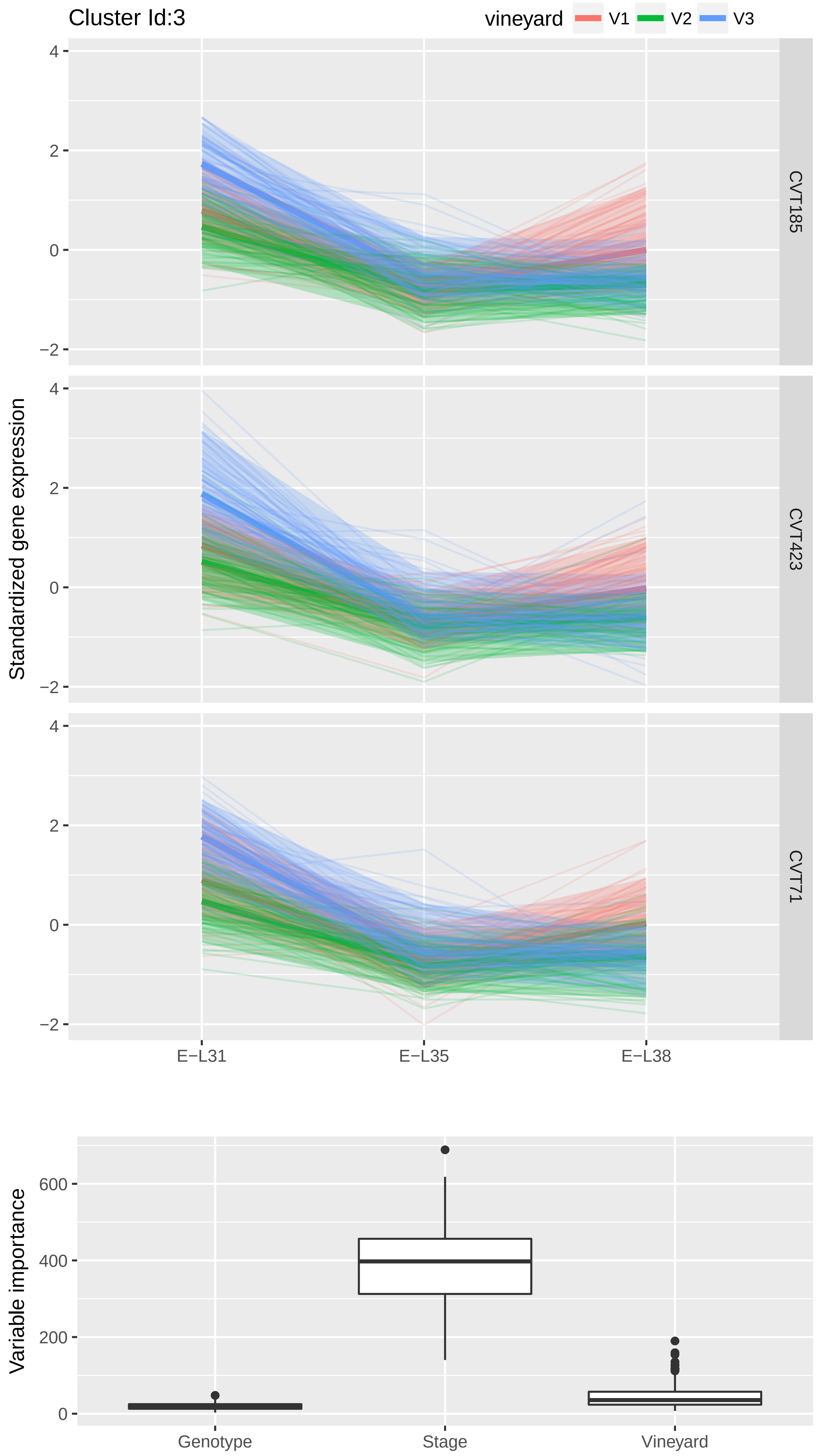

Cluster Id:3
vineyard
V1
V2
V3
Standardized gene expression
CVT185
CVT423
CVT71
4
2
0
−2
E−L31
E−L35
E−L38
Variable importance
600
400
200
0
Genotype
Stage
Vineyard

Cluster no. 4

```
## Number of genes in the cluster: 140
## Homogeneity Index:    0.84
## Variable importance for Stage:    Median =  476.7  - Rank =  41
## Variable importance for Clone:    Median =  16.35  - Rank =  69
## Variable importance for Vineyard: Median =  27.62  - Rank =  70
##
## Gene ID                Gene Annotation
## VIT_18s0072g00310  -  Polypyrimidine tract-binding protein 3
## VIT_09s0002g00430  -  AT-rich interaction region
## VIT_17s0000g05970  -  Ribosomal protein S9 (RPS9C) 40S
## VIT_16s0100g00700  -  TFL1 (Terminal flower 1), VvFLT1C
## VIT_05s0102g01120  -  Basic Leucine Zipper Transcription Factor (VvbZIP16)
## VIT_17s0000g06420  -  Galactosyl transferase GMA12/MNN10
## VIT_06s0004g08140  -  Unknown
## VIT_16s0022g00870  -  Invertase/pectin methylesterase inhibitor
## VIT_15s0021g01940  -  KH domain-containing protein
## VIT_07s0104g00960  -  Histone H2AXb HTA3
## VIT_00s1390g00010  -  DEAD  box  RNA helicase RH16
## VIT_07s0031g02190  -  Exosome complex exonuclease DIS3/RRP44
## VIT_04s0044g00560  -  Histone H3
## VIT_12s0034g02020  -  Chlororespiratory reduction 2 (CRR2)
## VIT_15s0048g01110  -  Ubiquitin related modifier 1
## VIT_08s0056g00560  -  Alphavirus core protein
## VIT_16s0098g01040  -  RNA-binding protein RBP37
## VIT_07s0005g03900  -  Rho GDP-dissociation inhibitor 1
## VIT_12s0142g00170  -  Potassium transporter (KUP7)
## VIT_10s0003g01940  -  RKF1 (receptor-like kinase in flowers 1)
## VIT_09s0002g06060  -  U3 small nucleolar RNA-associated protein 5
## VIT_17s0000g04170  -  DNA mismatch repair protein MSH2 (MSH2)
## VIT_12s0028g01250  -  ATP-dependent Clp protease proteolytic subunit (ClpP4)
## VIT_17s0000g10090  -  Xanthine/uracil/vitamin C permease
## VIT_00s0225g00230  -  Alliin lyase precursor
## VIT_08s0007g05330  -  EDA7 (embryo sac development arrest 7)
## VIT_07s0104g01340  -  Nodulin MtN3 family
## VIT_19s0085g00920  -  Organic cation transport protein OCT2
## VIT_00s0445g00010  -  Strubbelig receptor family 1
## VIT_03s0038g01360  -  Unknown
## VIT_13s0084g00650  -  Protein arginine N-methyltransferase 3
## VIT_02s0241g00010  -  Pentatricopeptide (PPR) repeat-containing protein
## VIT_00s0776g00010  -  Strubbelig receptor family 3
## VIT_07s0005g00770  -  RelA/SpoT protein (RSH1)
## VIT_01s0010g02480  -  Ankyrin repeat
## VIT_05s0077g01420  -  Unknown protein
## VIT_04s0008g05930  -  Zinc finger protein 3
## VIT_14s0060g02360  -  Histone H2A.4 HTA12
## VIT_00s0525g00010  -  DEAD  box  RNA helicase RH16
## VIT_01s0026g02590  -  Calcium-binding protein CML
## VIT_14s0066g01070  -  Peptide deformylase, chloroplast precursor
## VIT_06s0004g01690  -  Histone H3
## VIT_08s0007g00490  -  Transducin protein
## VIT_18s0001g03550  -  Molybdenum cofactor synthesis CNX7/SIR5
## VIT_07s0005g05860  -  Nuclear GTP-binding protein NUG1
## VIT_09s0002g07860  -  Glycerol kinase
## VIT_08s0007g02220  -  Unknown protein
```

```
## VIT_06s0004g00820  -  Ribosomal protein S2; Squalene/phytoene synthase
## VIT_06s0061g00610  -  Unknown protein
## VIT_00s1323g00010  -  Pentatricopeptide repeat-containing protein
## VIT_11s0016g02060  -  RecA
## VIT_04s0008g06760  -  Chlororespiratory reduction 4 CRR4
## VIT_04s0008g02490  -  Adagio protein 1
## VIT_11s0016g00370  -  ARID/BRIGHT DNA-binding domain-containing protein
## VIT_16s0039g01400  -  Unknown protein
## VIT_19s0090g01390  -  Acyltransferase
## VIT_06s0004g08130  -  No hit
## VIT_18s0001g10430  -  Trimethylguanosine synthase
## VIT_13s0019g03970  -  Ribosomal protein P0 (RPP0A) acidic 60S
## VIT_02s0025g05110  -  MATE efflux family protein
## VIT_16s0013g00380  -  Unknown protein
## VIT_04s0008g03660  -  Embryonic flower 1
## VIT_01s0011g03720  -  BEE1 (BR ENHANCED EXPRESSION 1)
## VIT_08s0040g03340  -  PHD finger transcription factor
## VIT_13s0067g03200  -  Proton-dependent oligopeptide transport (POT) family protein
## VIT_19s0014g03740  -  Membrane-associated mannitol-induced
## VIT_10s0003g04370  -  D123
## VIT_00s0198g00040  -  CTV.22
## VIT_16s0013g00600  -  No hit
## VIT_16s0050g00440  -  No hit
## VIT_00s0125g00200  -  No hit
## VIT_03s0038g02140  -  Auxin transporter protein 2
## VIT_00s0868g00010  -  DNA-binding storekeeper protein
## VIT_01s0026g00160  -  No hit
## VIT_08s0007g07610  -  Unknown protein
## VIT_07s0005g01430  -  PROLIFERA protein
## VIT_17s0000g07650  -  Aldehyde Dehydrogenase (VvALDH10A9)
## VIT_05s0102g00740  -  Leucin-rich repeat protein kinase
## VIT_17s0000g09420  -  Unknown
## VIT_00s0291g00100  -  ABC Transporter (VvTAP1 - VvABCB21)
## VIT_13s0019g02000  -  PUMILIO 1 (APUM1)
## VIT_12s0028g00670  -  Zinc knuckle
## VIT_18s0072g00800  -  Histone H3
## VIT_15s0021g02660  -  F-box domain containing protein
## VIT_01s0127g00370  -  Pentatricopeptide (PPR) repeat
## VIT_05s0062g01040  -  Unknown protein
## VIT_00s0441g00010  -  DEAD/DEAH box RNA helicase protein RH16
## VIT_12s0055g00690  -  Pentatricopeptide (PPR) repeat-containing
## VIT_00s2221g00010  -  Unknown
## VIT_18s0117g00370  -  R protein L6
## VIT_07s0005g00500  -  Small nuclear ribonucleoprotein D2
## VIT_14s0066g02600  -  Unknown protein
## VIT_02s0025g00220  -  Chlororespiratory reduction 4 (CRR4)
## VIT_19s0140g00040  -  RKF1 (receptor-like kinase in flowers 1)
## VIT_00s0125g00190  -  Potassium transporter (KUP1)
## VIT_16s0039g01590  -  Ubiquinone biosynthesis methyltransferase COQ5
## VIT_09s0002g01120  -  Exportin PAUSED
## VIT_17s0000g09100  -  Copper amine oxidase
## VIT_18s0117g00380  -  R protein L6
## VIT_15s0046g02850  -  Ankyrin protein kinase
## VIT_07s0255g00050  -  Unknown protein
```

```
## VIT_16s0022g01690  -  Band 7 family
## VIT_07s0104g00820  -  Bystin
## VIT_13s0019g05370  -  Histone H2B
## VIT_18s0001g15330  -  Nodulin MtN3 family
## VIT_19s0015g01060  -  Unknown protein
## VIT_10s0003g02950  -  No hit
## VIT_16s0098g00890  -  Harpin-induced protein
## VIT_19s0015g01050  -  Unknown protein
## VIT_13s0019g04740  -  Pseudouridine synthase
## VIT_08s0040g03300  -  Histone H2A.4 HTA12
## VIT_08s0105g00360  -  PHD finger protein alfin
## VIT_04s0044g01560  -  Protein kinase
## VIT_00s0818g00010  -  Splicing factor 3B subunit 2
## VIT_10s0003g02940  -  basic helix-loop-helix (bHLH) family
## VIT_13s0064g01340  -  Histone H3
## VIT_03s0091g00200  -  DNA repair protein REV1
## VIT_12s0028g02140  -  F-box/LRR-repeat MAX2
## VIT_09s0002g07640  -  Glycerol kinase
## VIT_12s0142g00050  -  PUMILIO 7 (APUM7)
## VIT_00s0173g00140  -  No hit
## VIT_12s0142g00250  -  Potassium transporter (KUP7)
## VIT_09s0002g02390  -  Phospholipase D epsilon
## VIT_17s0000g02250  -  Monooxygenase
## VIT_05s0029g00120  -  No hit
## VIT_05s0020g02070  -  BRCT domain-containing protein
## VIT_18s0075g00110  -  No hit
## VIT_10s0116g00130  -  Auxin-independent growth promoter
## VIT_05s0020g04800  -  Histone H1
## VIT_03s0038g00050  -  BLH6 (BELL1-like homeodomain 5)
## VIT_13s0067g03710  -  Unknown
## VIT_19s0014g00330  -  NAD(H) kinase 3
## VIT_19s0177g00290  -  No hit
## VIT_18s0001g12290  -  Pore protein 24K chain
## VIT_15s0046g02510  -  2-oxo acid dehydrogenase, lipoyl-binding site
## VIT_05s0029g00110  -  Unknown protein
## VIT_10s0116g01500  -  DNA helicase SNF2 domain-containing protein
## VIT_11s0052g00580  -  No hit
## VIT_16s0050g01110  -  Polygalacturonase GH28
## VIT_04s0023g02200  -  S-adenosyl-L-methionine:salicylic acid carboxyl methyltransferase
```

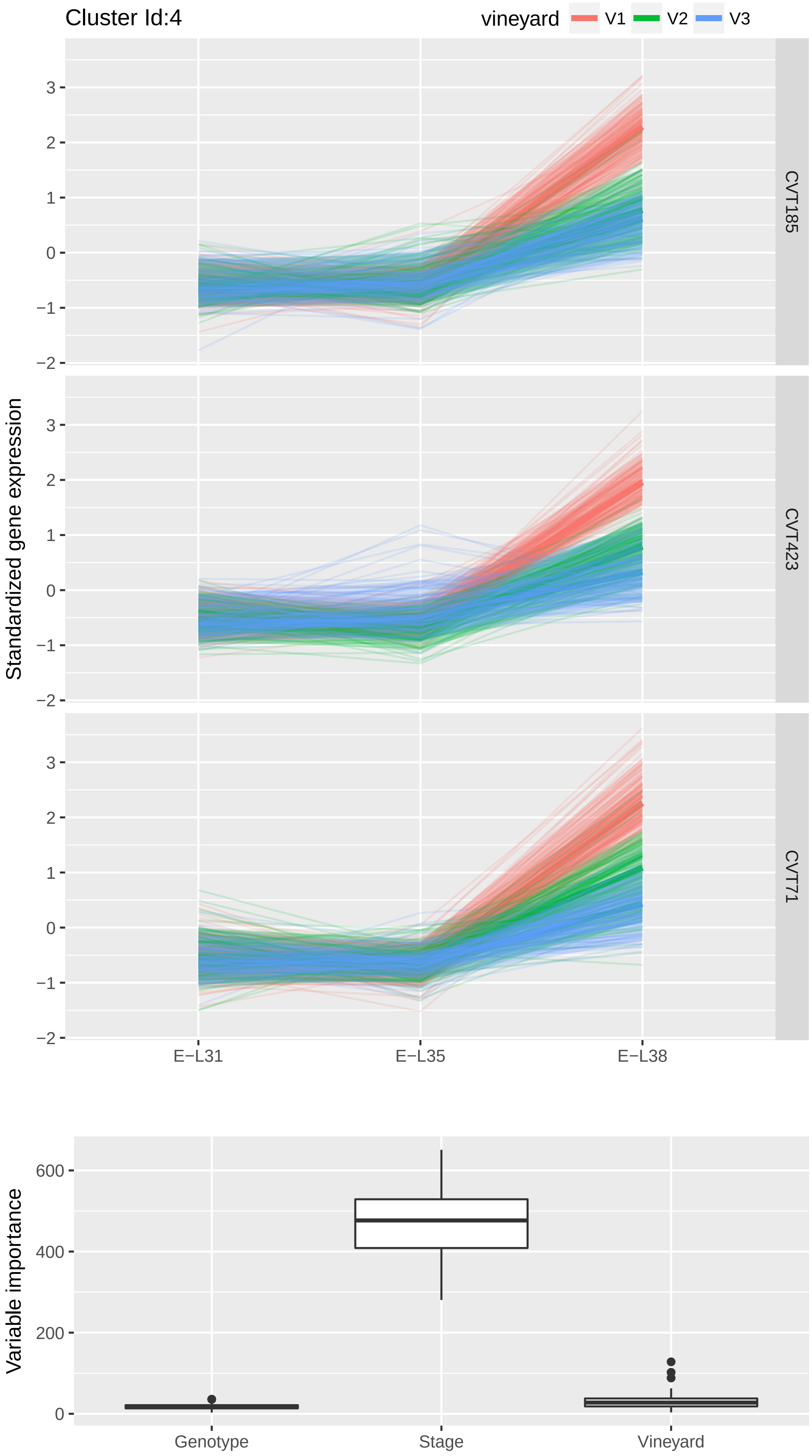

Cluster Id:4
vineyard
V1
V2
V3
CVT185
CVT423
CVT71
Standardized gene expression
3
2
1
0
−1
−2
E−L31
E−L35
E−L38
Variable importance
600
400
200
0
Genotype
Stage
Vineyard

Cluster no. 5

```
## Number of genes in the cluster: 85
## Homogeneity Index:    0.57
## Variable importance for Stage:    Median =  337.4  - Rank =  65
## Variable importance for Clone:    Median =  29.45  - Rank =  9
## Variable importance for Vineyard: Median =  34.23  - Rank =  60
##
## Gene ID               Gene Annotation
## VIT_13s0047g00330  -  FAR1-related sequence 3
## VIT_06s0004g02160  -  Zinc finger (CCCH-type) family protein
## VIT_14s0060g02640  -  Myb family
## VIT_15s0046g01790  -  ARID/BRIGHT DNA-binding domain-containing protein / ELM2
## VIT_08s0007g04380  -  PAB7 (poly(A) binding protein 7)
## VIT_05s0029g01420  -  Ankyrin repeat
## VIT_04s0043g00770  -  Pentatricopeptide (PPR) repeat-containing protein
## VIT_13s0067g03210  -  AarF domain containing kinase 1
## VIT_14s0083g01010  -  Protein disulfide oxidoreductase DSBA oxidoreductase
## VIT_11s0016g02570  -  Phospholipase A2 precursor
## VIT_14s0083g01020  -  Regulator of chromosome condensation (RCC1)
## VIT_03s0038g04210  -  Phototropin-2
## VIT_10s0003g01030  -  Unknown protein
## VIT_09s0002g06590  -  UDP-glucose:flavonoid 7-O-glucosyltransferase
## VIT_14s0066g01210  -  Carbonic anhydrase, chloroplast precursor
## VIT_05s0049g01950  -  Tetratricopeptide helical
## VIT_10s0003g04500  -  Ankyrin
## VIT_12s0057g00260  -  External rotenone-insensitive NADPH dehydrogenase
## VIT_07s0005g00660  -  Late embryogenis abundant protein 5
## VIT_14s0036g00630  -  RNA binding motif protein 42
## VIT_01s0127g00470  -  Galactinol synthase
## VIT_08s0040g01780  -  Lysine decarboxylase
## VIT_04s0023g02810  -  Aldehyde Dehydrogenase (VvALDH3F1)
## VIT_02s0025g04180  -  Unknown protein
## VIT_14s0060g01490  -  DnaJ homolog, subfamily A, member 5
## VIT_03s0038g04730  -  Polyphenol oxidase
## VIT_00s0125g00220  -  Serine/theronine protein kinase 2
## VIT_15s0021g00470  -  Beta-phosphoglucomutase
## VIT_00s0125g00210  -  Serine/theronine protein kinase 2
## VIT_08s0007g04240  -  Late embryogenesis abundant protein
## VIT_02s0012g02050  -  PAP/fibrillin family
## VIT_11s0016g05780  -  No hit
## VIT_00s0710g00010  -  GCR2-like 1 ABA receptor
## VIT_02s0025g00500  -  Unknown protein
## VIT_07s0151g00780  -  Hemolysin A
## VIT_07s0255g00090  -  CYP82C1p
## VIT_01s0010g02440  -  ABC Transporter (VvMRP1 - VvABCC1)
## VIT_18s0001g12710  -  No hit
## VIT_01s0026g02520  -  Ribonuclease T2
## VIT_05s0077g01230  -  SAR1 (secretion-associated ras)
## VIT_14s0006g02510  -  Plastid movement impaired 15 (PMI15)
## VIT_11s0118g00240  -  Purple acid phosphatase 26
## VIT_02s0087g00930  -  (9,10) (9`,10`) cleavage dioxygenase (CCD4) (VvCCD4b)
## VIT_01s0146g00280  -  NAC domain-containing protein (VvNAC01)
## VIT_15s0048g00190  -  SPX (SYG1/Pho81/XPR1) domain-containing protein
## VIT_04s0044g00920  -  Ankyrin
## VIT_08s0058g01020  -  Saposin B domain-containing protein
```

```
## VIT_03s0038g04260  -  Chloride channel protein CLC-e
## VIT_08s0007g00830  -  Lectin protein kinase
## VIT_04s0008g00470  -  Early nodulin Enod93 protein
## VIT_13s0064g00910  -  Unknown
## VIT_00s0309g00090  -  Expansin  (VvEXLB3)
## VIT_00s1455g00010  -  Expansin  (VvEXLB4)
## VIT_08s0007g00820  -  Proteinase inhibitor
## VIT_06s0004g06770  -  No hit
## VIT_00s0705g00010  -  CYP81E1 Isoflavone 2'-hydroxylase
## VIT_09s0002g00730  -  No hit
## VIT_06s0004g04440  -  Osmotin
## VIT_18s0041g00270  -  R protein  PRF  disease resistance protein
## VIT_08s0058g00400  -  No hit
## VIT_12s0059g01470  -  Unknown protein
## VIT_11s0016g04990  -  Unknown protein
## VIT_08s0032g00680  -  SPX (SYG1/Pho81/XPR1) domain-containing protein
## VIT_11s0118g00620  -  Methyltransferase type 11
## VIT_00s0183g00050  -  No hit
## VIT_19s0015g02690  -  Glutathione S-transferase 25 GSTU25
## VIT_11s0016g02200  -  Beta-galactosidase
## VIT_06s0004g06850  -  MAPKKK14
## VIT_01s0146g00530  -  No hit
## VIT_18s0041g00260  -  R protein  PRF  disease resistance protein
## VIT_04s0044g01040  -  basic helix-loop-helix (bHLH) family
## VIT_00s0309g00050  -  Expansin  (VvEXLB2)
## VIT_03s0091g00670  -  Lateral organ boundaries protein 38
## VIT_12s0057g00980  -  Phytochrome defective C (PHYC)
## VIT_12s0035g02100  -  Glutathione S-transferase Z1 GSTZ1
## VIT_01s0010g00450  -  S ribonuclease
## VIT_19s0090g01340  -  No hit
## VIT_17s0000g08520  -  Pantothenate kinase
## VIT_10s0003g01020  -  Unknown protein
## VIT_09s0002g04300  -  Hydroxyphenylpyruvate reductase (HPPR)
## VIT_00s0438g00020  -  ATPase subunit 9
## VIT_07s0031g01940  -  Holocarboxylase synthetase
## VIT_01s0026g01740  -  CBS
## VIT_11s0037g00980  -  Unknown
## VIT_12s0059g01800  -  Unknown protein
```

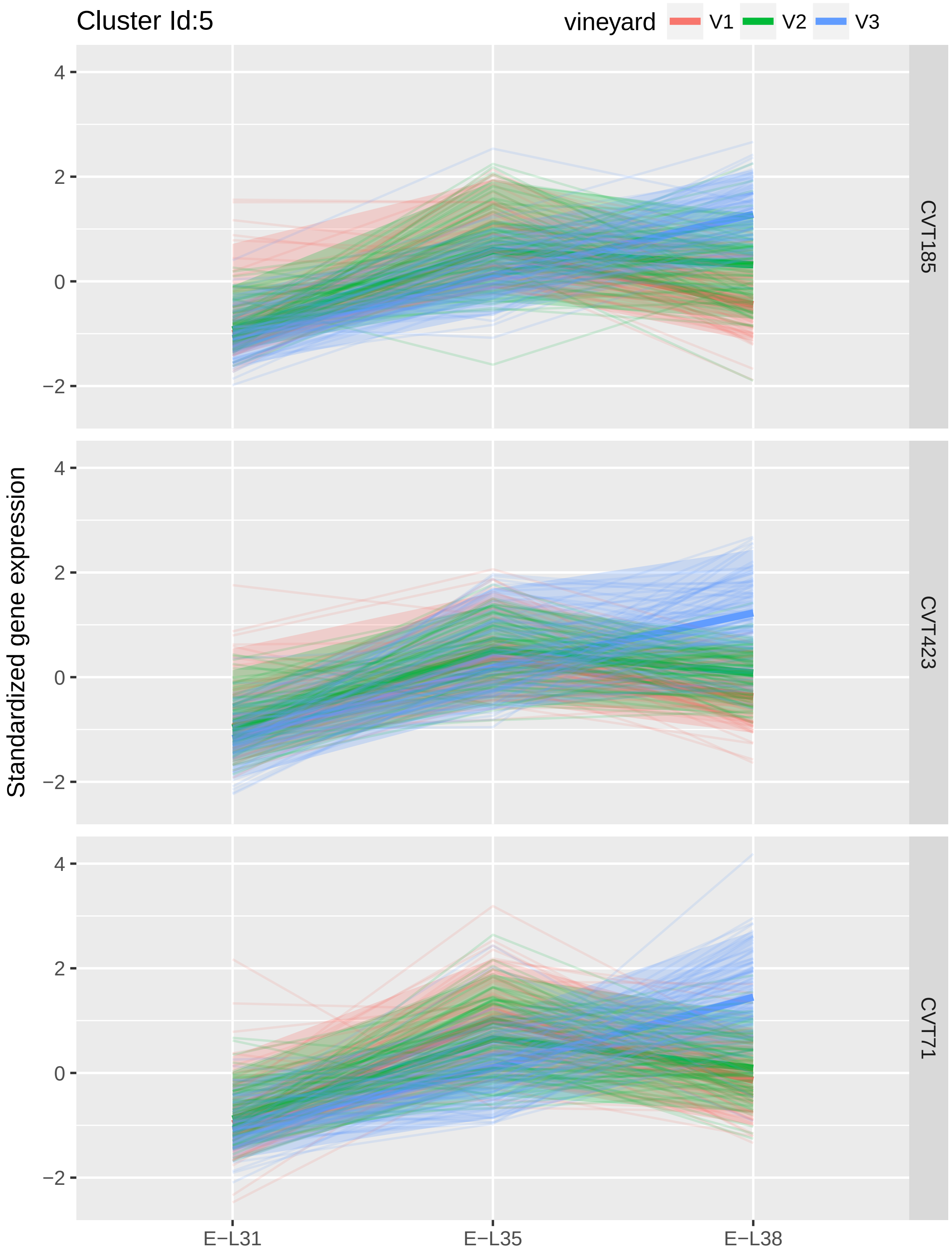

Cluster Id:5
vineyard
V1
V2
V3
Standardized gene expression
CVT185
CVT423
CVT71
4
2
0
−2
E−L31
E−L35
E−L38

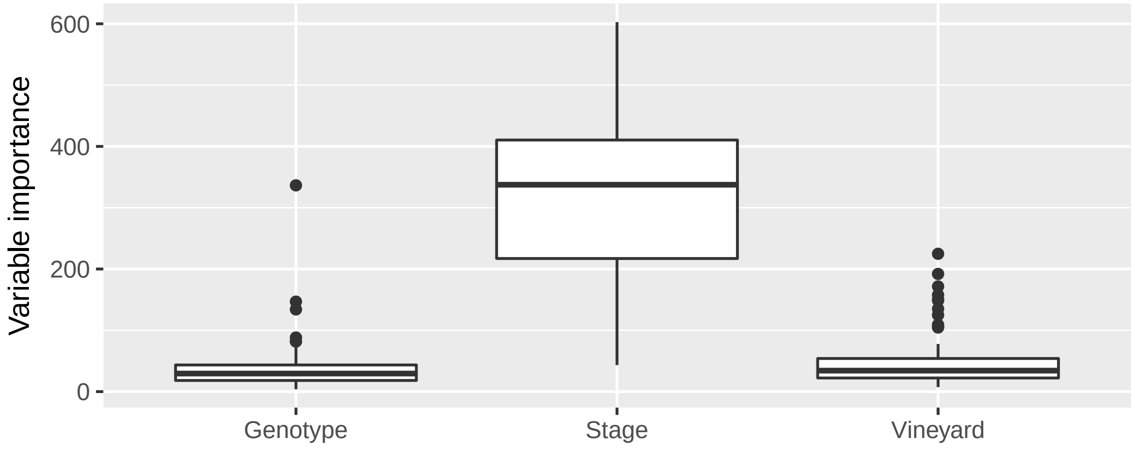

Variable importance
600
400
200
0
Genotype
Stage
Vineyard

Cluster no. 6

```
## Number of genes in the cluster: 179
## Homogeneity Index:     0.8
## Variable importance for Stage:    Median =  625.7  - Rank =  11
## Variable importance for Clone:    Median =  10.16  - Rank =  107
## Variable importance for Vineyard: Median =   14.3  - Rank =  101
##
## Gene ID               Gene Annotation
## VIT_03s0038g00330  -  EXECUTER1 protein, chloroplast precursor
## VIT_17s0000g07550  -  Zinc finger (C3HC4-type ring finger)
## VIT_08s0058g01140  -  Unknown protein
## VIT_07s0005g01220  -  DnaJ homolog, subfamily B, member 11
## VIT_13s0019g04460  -  Phenylalanine ammonia-lyase 2 (PAL2)
## VIT_07s0151g01010  -  Pre-mRNA-splicing factor 18
## VIT_08s0058g01290  -  Protein kinase AtSIK
## VIT_14s0068g01100  -  Unknown protein
## VIT_05s0020g03020  -  ARMADILLO BTB Arabidopsis protein 1 ABAP1
## VIT_07s0005g04220  -  Receptor-like protein kinase
## VIT_00s0203g00210  -  Zinc finger (B-box type)
## VIT_07s0151g00840  -  Late embryogenesis abundant protein D-34 (LEA D-34)
## VIT_03s0063g00680  -  Zinc metalloprotease pitrilysin sub A
## VIT_01s0010g01090  -  Peroxidase ATP23a
## VIT_03s0017g01000  -  Amine oxidase
## VIT_04s0008g05420  -  SPX2 (SYG1/Pho81/XPR1) domain-containing protein SPX2
## VIT_18s0001g09030  -  F-box protein
## VIT_05s0020g01720  -  Zinc finger (C3HC4-type ring finger)
## VIT_16s0100g00510  -  F-box family protein
## VIT_06s0004g07830  -  SOS2 (salt overly sensitive 2)
## VIT_13s0019g05090  -  Stage III sporulation protein AA
## VIT_12s0059g01570  -  AtZW10
## VIT_00s0194g00110  -  4-nitrophenylphosphatase
## VIT_01s0010g03800  -  Unknown protein
## VIT_13s0019g04010  -  myb family transcription factor
## VIT_18s0001g03790  -  Oxysterol binding protein
## VIT_01s0010g01130  -  6-phosphogluconate dehydrogenase
## VIT_19s0014g00850  -  Unknown protein
## VIT_01s0011g04800  -  Quinolinate phosphoribosyl transferase
## VIT_04s0023g01300  -  Aminopeptidase P
## VIT_00s0125g00250  -  Zinc finger (ubiquitin-hydrolase) domain-containing
## VIT_14s0060g01080  -  ferroportin
## VIT_18s0001g05770  -  Protein phosphatase methylesterase 1
## VIT_13s0067g03840  -  Lung seven transmembrane receptor
## VIT_09s0002g01020  -  Cation exchanger (CAX9)
## VIT_00s0805g00020  -  CDKC;1; CDKC;1 (CYCLIN-dependent kinase C;1)
## VIT_10s0071g01080  -  Unknown protein
## VIT_06s0004g02700  -  Unknown protein
## VIT_16s0100g00720  -  Heat shock transcription factor B2A
## VIT_15s0046g00370  -  Unknown protein
## VIT_11s0016g04780  -  Ras GTP-binding protein (RAN2)
## VIT_14s0006g02020  -  NADH dehydrogenase (ubiquinone) Fe-S protein 1
## VIT_17s0053g00620  -  Nascent polypeptide-associated complex subunit beta
## VIT_01s0026g02100  -  Unknown protein
## VIT_01s0011g03660  -  IMP dehydrogenase/GMP reductase
## VIT_05s0077g01830  -  Phospholipase D zeta1
## VIT_01s0010g01230  -  Unknown protein
```

```
## VIT_13s0047g00300  -  No hit
## VIT_18s0075g00520  -  Retrotransposon protein, Unclassified
## VIT_19s0014g01020  -  Protein transport protein Sec61 subunit alpha
## VIT_01s0011g00650  -  Calcineurin phosphoesterase
## VIT_10s0003g04900  -  ARF GTPase activator, ARF-GAP Domain 12
## VIT_14s0108g00450  -  No hit
## VIT_03s0091g00360  -  Seed maturation protein PM23
## VIT_15s0046g00080  -  Yippee
## VIT_05s0062g00890  -  Alpha-glucan water dikinase isoform 3, Chloroplast
## VIT_04s0008g02550  -  4-coumarate-CoA ligase
## VIT_05s0062g00900  -  Alpha-glucan water dikinase isoform 3, Chloroplast
## VIT_04s0008g06800  -  Enhancer of rudimentary protein
## VIT_00s0479g00010  -  Protein kinase 6
## VIT_19s0027g00780  -  NADH dehydrogenase (ubiquinone) flavoprotein 1
## VIT_05s0077g00650  -  Poly(A) polymerase
## VIT_07s0129g00040  -  Peptidyl-prolyl cis-trans isomerase-like 2
## VIT_02s0025g01450  -  Unknown protein
## VIT_14s0030g00400  -  WD40
## VIT_01s0011g01050  -  Vacuolar sorting receptor 6
## VIT_11s0016g03200  -  Gibberellin oxidase
## VIT_14s0060g01250  -  Unknown protein
## VIT_02s0154g00190  -  flavin-containing monooxygenase 3
## VIT_18s0086g00710  -  1-phosphatidylinositol-3-phosphate 5-kinase
## VIT_11s0016g02290  -  Proteasome protein
## VIT_11s0016g04060  -  MUT9-related serine/threonine protein kinase
## VIT_06s0080g01230  -  DnaJ homolog, subfamily A, member 2
## VIT_02s0025g03520  -  Haloacid dehalogenase hydrolase
## VIT_05s0020g02540  -  Endo-1,3;1,4-beta-D-glucanase precursor
## VIT_00s0662g00010  -  Acyl-CoA oxidase (ACX2)
## VIT_02s0025g01710  -  Unknown
## VIT_11s0016g04950  -  NAD+ diphosphatase
## VIT_19s0090g00610  -  DNA-binding bromodomain-containing
## VIT_12s0142g00610  -  Citrate synthase, glyoxysomal precursor
## VIT_10s0003g01840  -  Unknown protein
## VIT_16s0100g00320  -  Zfwd2 protein (ZFWD2)
## VIT_07s0141g00280  -  CDKC;1; CDKC;1 (CYCLIN-dependent kinase C;1)
## VIT_12s0034g01420  -  Hydroxyproline-rich glycoprotein
## VIT_07s0005g03310  -  Cofilin
## VIT_05s0020g03160  -  Transmembrane protein 49
## VIT_01s0026g01010  -  Histone deacetylase complex, SIN3 component
## VIT_05s0020g03860  -  Homocysteine S-methyltransferase 3
## VIT_08s0056g01610  -  No hit
## VIT_08s0056g00200  -  2,3-bisphosphoglycerate-independent phosphoglycerate mutase
## VIT_00s0233g00180  -  flavin-containing monooxygenase 3
## VIT_13s0073g00250  -  Urate oxidase.
## VIT_00s0229g00060  -  Nodulin MtN21 family
## VIT_13s0019g04410  -  Oligosaccharyltransferase complex subunit alpha (ribophorin I)
## VIT_19s0014g04780  -  No hit
## VIT_19s0014g02210  -  Proteasome
## VIT_13s0106g00780  -  Hydrolase, alpha/beta fold
## VIT_04s0008g03860  -  Peroxisomal membrane 22 kDa
## VIT_11s0016g02540  -  Xaa-Pro dipeptidase
## VIT_01s0010g03680  -  Unknown protein
## VIT_02s0012g00930  -  Unknown protein
## VIT_04s0008g01380  -  Unknown protein
```

```
## VIT_06s0080g00630  -  No hit
## VIT_05s0020g04570  -  CBL-interacting protein kinase 7 (CIPK7)
## VIT_00s0598g00010  -  CDP-diacylglycerol synthetase
## VIT_01s0011g04350  -  fructose-bisphosphate aldolase, chloroplast precursor
## VIT_02s0025g03500  -  Unknown protein
## VIT_04s0079g00690  -  Glutathione S-transferase (VvGST4)
## VIT_08s0007g04190  -  ATPP2-A15
## VIT_03s0038g02240  -  Unknown protein
## VIT_19s0015g00450  -  RNA recognition motif (RRM)-containing protein
## VIT_14s0219g00250  -  Ku70-binding
## VIT_11s0016g01700  -  Unknown protein
## VIT_00s0577g00020  -  Phosphoglycerate mutase
## VIT_03s0038g03730  -  Niemann-Pick C1 protein precursor
## VIT_08s0040g03270  -  E3 ubiquitin-protein ligase TRIP12
## VIT_13s0067g03560  -  Unknown protein
## VIT_10s0523g00040  -  Alternative oxidase 4, chloroplastic/chromoplastic
## VIT_07s0005g01090  -  ATP binding protein
## VIT_01s0127g00520  -  Protein disulfide isomerase
## VIT_00s0391g00060  -  Preprotein translocase subunit SecG
## VIT_05s0020g01730  -  Anaphase-promoting complex component APC7
## VIT_15s0046g02640  -  Beta-amylase 4
## VIT_15s0021g02140  -  Zinc finger (C3HC4-type ring finger)
## VIT_18s0001g13930  -  Auxin response factor 5 (Transcription factor MONOPTEROS)
## VIT_19s0093g00160  -  Glutathione S-transferase 8 GSTU19
## VIT_18s0122g01280  -  Niemann-Pick C1 protein precursor
## VIT_13s0084g00040  -  Nuclear transcription factor Y subunit B related
## VIT_18s0001g01510  -  No hit
## VIT_17s0000g08890  -  Cell division control protein 48 homolog E
## VIT_05s0077g00580  -  DnaJ homolog, subfamily C, member 14
## VIT_07s0104g01480  -  Cold-induced thioredoxin domain
## VIT_15s0021g02090  -  DnaJ homolog, subfamily A, member 2
## VIT_05s0020g01500  -  Heat shock factor-binding protein 1
## VIT_18s0076g00330  -  Basic Leucine Zipper Transcription Factor (VvbZIP50)
## VIT_01s0127g00680  -  SRO2 (similar to rcd one 2)
## VIT_05s0020g04520  -  AT rich interactive domain 2 (ARID, RFX-like)
## VIT_02s0012g01910  -  Unknown protein
## VIT_12s0028g01160  -  Transparent testa 12 protein
## VIT_05s0077g00570  -  F-box domain containing protein
## VIT_01s0026g00750  -  Unknown protein
## VIT_00s0984g00010  -  Phosphoglycerate mutase
## VIT_07s0005g05640  -  Unknown protein
## VIT_05s0049g02350  -  HAD superfamily hydrolase
## VIT_03s0038g01240  -  Ubiquinone biosynthesis monooxygenase Coq6
## VIT_14s0006g01030  -  Calmodulin-binding heat-shock protein
## VIT_15s0048g02010  -  Shaggy protein kinase theta
## VIT_06s0004g05490  -  Myosin heavy chain
## VIT_14s0006g02310  -  No hit
## VIT_02s0087g00570  -  Unknown protein
## VIT_06s0009g00900  -  Unknown protein
## VIT_11s0037g01220  -  No hit
## VIT_18s0001g05810  -  Cytochrome c
## VIT_14s0066g02400  -  Mannose-1-phosphate guanyltransferase
## VIT_18s0001g05420  -  UNE2 (unfertilized embryo sac 2); carbohydrate transporter
## VIT_09s0002g06420  -  Lactoylglutathione lyase
## VIT_11s0016g01070  -  Cathepsin H
```

```
## VIT_01s0010g03610  -  RW1
## VIT_16s0022g02340  -  fructokinase-2
## VIT_16s0050g02180  -  Pyruvate kinase isozyme G, chloroplast precursor
## VIT_18s0089g00410  -  Sucrose-phosphate synthase 1
## VIT_15s0046g00150  -  DOF affecting germination 1
## VIT_11s0037g00300  -  Signal recognition particle receptor alpha subunit
## VIT_02s0025g02440  -  GAMM1 protein
## VIT_14s0219g00150  -  Succinate dehydrogenase iron-protein subunit
## VIT_04s0008g03260  -  Thioredoxin domain containing 14
## VIT_05s0049g02320  -  HAD superfamily hydrolase
## VIT_14s0108g00050  -  ERF/AP2 Gene Family (VvAP2-02)
## VIT_18s0075g00340  -  Sucrose-phosphate synthase - like protein
## VIT_01s0011g00760  -  Beta-glucosidase
## VIT_17s0000g05080  -  Cleavage stimulation factor subunit 1
## VIT_03s0017g00460  -  Magnesium-dependent phosphatase 1
## VIT_01s0026g00990  -  Pyruvate dehydrogenase E1 component alpha subunit
## VIT_08s0007g01710  -  E-cadherin binding protein
## VIT_18s0001g03450  -  Glycine-rich protein
## VIT_09s0054g01880  -  Calreticulin interacted protein
## VIT_12s0028g01000  -  Unknown protein
## VIT_04s0008g00540  -  Oleosin OLE-3
## VIT_17s0000g05150  -  Enoyl-CoA hydratase
```

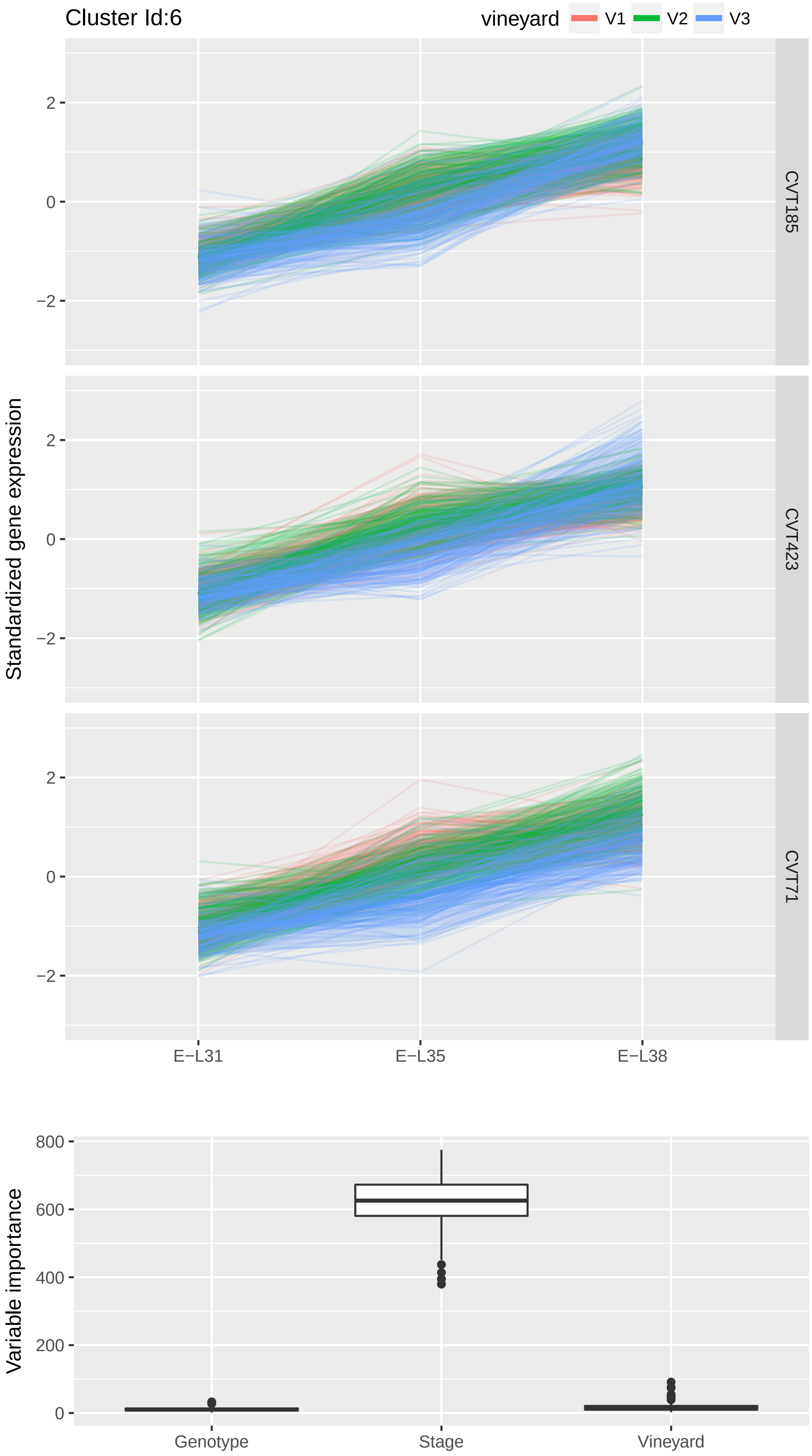
Cluster Id:6
vineyard
V1
V2
V3
Standardized gene expression
CVT185
CVT423
CVT71
2
0
−2
E−L31
E−L35
E−L38
Variable importance
800
600
400
200
0
Genotype
Stage
Vineyard

Cluster no. 7

```
## Number of genes in the cluster: 372
## Homogeneity Index:    0.84
## Variable importance for Stage:    Median =  594.8  - Rank =  15
## Variable importance for Clone:    Median =  14.09  - Rank =  77
## Variable importance for Vineyard: Median =  18.79  - Rank =  85
##
## Gene ID               Gene Annotation
## VIT_08s0056g00190  -  Pyruvate kinase
## VIT_14s0036g00930  -  Polyubiquitin (UBQ4)
## VIT_17s0000g07010  -  5-azacytidine resistance
## VIT_04s0008g00770  -  Glycine hydroxymethyltransferase
## VIT_15s0046g02260  -  Myb family
## VIT_01s0011g00300  -  SIK1 (serine/threonine kinase 1)
## VIT_07s0031g03160  -  U3 small nucleolar ribonucleoprotein protein IMP4
## VIT_12s0057g01300  -  Armadillo/beta-catenin repeat family protein
## VIT_01s0146g00440  -  Unknown protein
## VIT_14s0060g00970  -  Unknown protein
## VIT_07s0104g00590  -  Unknown protein
## VIT_07s0130g00050  -  Zinc finger (C3HC4-type ring finger)
## VIT_14s0060g02400  -  PWWP domain-containing protein
## VIT_00s0174g00190  -  Ribosomal protein L5 60S
## VIT_00s0285g00030  -  ZFWD1 (zinc finger WD40 repeat protein 1)
## VIT_04s0044g01840  -  ARID/BRIGHT DNA-binding domain-containing protein
## VIT_07s0005g04240  -  Import receptor subunit TOM22 homolog 2, Mitochondrial
## VIT_18s0001g03890  -  No hit
## VIT_11s0016g01470  -  Lecithine cholesterol acyltransferase
## VIT_00s0179g00290  -  Peptidyl-prolyl isomerase D (cyclophilin D)
## VIT_01s0026g02210  -  Unknown protein
## VIT_04s0043g00840  -  Armadillo/beta-catenin repeat protein / U-box domain-containing protei
## VIT_14s0066g00390  -  Methyltransferase Rrp8p
## VIT_01s0011g05730  -  Nuclear pore complex protein Nup155 (Nucleoporin 155)
## VIT_13s0175g00150  -  Armadillo repeat-containing protein
## VIT_11s0016g02340  -  Cytidine deaminase
## VIT_18s0001g08900  -  No hit
## VIT_02s0025g01410  -  Acyl-CoA synthetase long-chain member 2
## VIT_17s0000g06340  -  putative MADS-box type delta 2b (VviMADSD2b)
## VIT_19s0177g00100  -  Translation initiation factor eIF-1
## VIT_07s0005g06730  -  PPR2
## VIT_03s0038g04130  -  DEAD box RNA helicase
## VIT_09s0002g05940  -  Ras GTP-binding protein (RAN3)
## VIT_12s0059g00610  -  Auxin-resistance protein AXR1 (AXR1)
## VIT_18s0001g07870  -  Unknown protein
## VIT_17s0000g02400  -  Toc64 (translocon outer membrane complex 64)
## VIT_00s0264g00120  -  Transformer serine/arginine-rich ribonucleoprotein
## VIT_00s0225g00010  -  Unknown protein
## VIT_19s0177g00300  -  Glucose 6 phosphate/phosphate translocator-like protein
## VIT_19s0090g00450  -  Unknown protein
## VIT_18s0001g07630  -  NADPH-cytochrome P450 oxydoreductase isoform 1
## VIT_13s0047g00860  -  Replication protein
## VIT_00s0181g00210  -  Anaphase promoting complex subunit 4
## VIT_00s0370g00030  -  CPL1 (FIERY 2);
## VIT_03s0063g00300  -  4-methyl-5-thiazole monophosphate biosynthesis protein
## VIT_18s0001g11380  -  Seven transmembrane domain protein
## VIT_01s0011g03740  -  Glutaminyl-tRNA synthetase
```

```
## VIT_02s0025g05130  -  High mobility group HMG-I and HMG-Y, DNA-binding
## VIT_00s0857g00010  -  No hit
## VIT_00s2499g00010  -  No hit
## VIT_00s0370g00040  -  CPL1 (FIERY 2);
## VIT_05s0102g00230  -  Protein farnesyltransferase/geranylgeranyltransferase type I alphasubu
## VIT_01s0026g01400  -  Unknown protein
## VIT_07s0151g00760  -  Lipid transfer protein
## VIT_01s0011g01020  -  RPM1 (resistance to p. syringae pv maculicola 1)
## VIT_07s0031g02680  -  HEN3 (HUA enhancer 3) CDKE
## VIT_07s0129g00680  -  Pentatricopeptide (PPR) repeat-containing protein
## VIT_02s0025g01440  -  PHD finger transcription factor
## VIT_18s0072g00840  -  Sensitive to proton rhizotoxicity 1
## VIT_07s0005g00620  -  U3 small nucleolar RNA-associated protein 14
## VIT_06s0004g04240  -  Histone H2B
## VIT_08s0040g00870  -  Basic Leucine Zipper Transcription Factor (VvbZIP24)
## VIT_12s0035g02040  -  No hit
## VIT_06s0004g01810  -  MSF1
## VIT_15s0046g01420  -  Exonuclease RRP41 (RRP41)
## VIT_16s0022g00590  -  Unknown protein
## VIT_12s0028g00560  -  Unknown
## VIT_06s0004g03090  -  Syntaxin of plants SYP7
## VIT_04s0043g00250  -  Nonsense-mediated mRNA decay NMD3
## VIT_05s0020g01280  -  Wound-responsive protein
## VIT_06s0009g02420  -  Dual-specific kinase DSK1
## VIT_07s0005g01100  -  Importin alpha
## VIT_00s0445g00040  -  Unknown
## VIT_00s0880g00020  -  Translation initiation factor eIF-3 subunit 7
## VIT_18s0001g04270  -  Pentatricopeptide (PPR) repeat-containing
## VIT_15s0045g01110  -  Pre-mRNA splicing factor cwc22
## VIT_18s0001g14550  -  Unknown protein
## VIT_19s0014g00150  -  Purine permease 11 PUP11
## VIT_14s0030g00480  -  HAT dimerisation domain-containing protein
## VIT_09s0002g01810  -  Import inner membrane translocase subunit Tim17/Tim22/Tim23
## VIT_08s0007g08400  -  Heme exporter ATP-binding protein CcmA
## VIT_08s0040g02850  -  RSZ33 (Arginine/serine-rich Zinc knuckle-containing protein 33)
## VIT_14s0068g02140  -  SWIB complex BAF60b domain-containing protein
## VIT_14s0066g01660  -  DegP protease
## VIT_09s0002g00910  -  Exocyst subunit EXO70 D1
## VIT_07s0005g00260  -  ABC Transporter (VvMRP21 - VvABCC21)
## VIT_19s0015g00940  -  Glucose-6-phosphate/phosphate-tranlocator
## VIT_11s0149g00180  -  No hit
## VIT_19s0014g02810  -  Nucleotidyltransferase family protein
## VIT_00s0173g00250  -  RKF1 (receptor-like kinase in flowers 1)
## VIT_11s0016g02670  -  Unknown protein
## VIT_07s0005g06490  -  Ribosomal protein L13A (RPL13aD) 60S
## VIT_08s0007g00250  -  WD-40 repeat protein
## VIT_00s0373g00040  -  Malate dehydrogenase Glyoxysomal
## VIT_06s0009g03440  -  Tankyrase 2
## VIT_18s0001g10440  -  THO complex subunit 5
## VIT_06s0004g03320  -  Zinc finger (C3HC4-type ring finger)
## VIT_02s0025g00070  -  Ubiquitin-conjugating enzyme E2 H
## VIT_06s0004g03880  -  Ribonuclease P/MRP protein subunit RPP1
## VIT_08s0007g03400  -  Iron-sulfur cluster assembly complex protein
## VIT_16s0039g00370  -  Coiled-coil domain-containing protein 12
## VIT_09s0002g06550  -  Signal peptidase, endoplasmic reticulum-type
```

```
## VIT_01s0011g05330  -  Clathrin assembly protein 8
## VIT_13s0067g02250  -  Unknown protein
## VIT_06s0004g00680  -  Unknown protein
## VIT_18s0001g13950  -  RNA polymerase Rpa43 subunit
## VIT_13s0047g00320  -  EDA35 (embryo sac development arrest 35)
## VIT_08s0040g02860  -  RSZ33 (Arginine/serine-rich Zinc knuckle-containing protein 33)
## VIT_14s0006g01370  -  VTC2
## VIT_18s0001g04730  -  Kelch repeat-containing F-box protein
## VIT_18s0001g13680  -  Histone H3
## VIT_08s0040g00570  -  Polyglutamine-binding protein 1
## VIT_00s0181g00190  -  D-erythro-sphingosine kinase
## VIT_08s0040g03380  -  Major Facilitator Superfamily
## VIT_17s0000g07260  -  Glucan endo-1,3-beta-glucosidase 3 precursor
## VIT_04s0008g00190  -  ATP-dependent protease La (LON) domain-containing
## VIT_07s0005g00860  -  U3 snoRNP-associated helicase Ecm16
## VIT_06s0080g01270  -  HAT dimerisation domain-containing protein
## VIT_19s0090g01190  -  Chaperone GrpE type 2
## VIT_05s0077g02310  -  PP1/PP2A phosphatases pleiotropic regulator PRL1
## VIT_08s0032g01080  -  Ubiquitin carboxyl-terminal hydrolase 8
## VIT_07s0005g03050  -  Deoxyhypusine hydroxylase
## VIT_06s0004g04340  -  ALY protein
## VIT_18s0001g03430  -  Flavonol synthase
## VIT_06s0004g03300  -  2',3'-cyclic-nucleotide 3'-phosphodiesterase
## VIT_10s0003g03470  -  Multidrug resistance-associated protein 5
## VIT_16s0050g01660  -  UDP-glucoronosyl/UDP-glucosyl transferase UGT88A1
## VIT_04s0008g02460  -  H+-transporting ATPase, Plasma membrane
## VIT_05s0049g00470  -  Electron transfer flavoprotein beta-subunit
## VIT_12s0035g00240  -  DAG protein, chloroplast precursor
## VIT_18s0001g10370  -  S-ribonuclease binding protein SBP1
## VIT_00s1308g00010  -  Unknown
## VIT_06s0009g00340  -  Unknown protein
## VIT_19s0090g00850  -  SWAP (Suppressor-of-White-APricot)
## VIT_19s0014g03680  -  Meiotic recombination 11 MRE11
## VIT_16s0013g00610  -  HAP1/MAGO/MEE63 (maternal effect embryo arrest 63)
## VIT_13s0019g03200  -  myb domain protein 109
## VIT_08s0105g00290  -  Zinc finger (C3HC4-type ring finger)
## VIT_08s0007g07180  -  Translation initiation factor eIF-3 subunit 8
## VIT_19s0085g00530  -  No hit
## VIT_18s0001g11200  -  Unknown
## VIT_07s0151g00400  -  Unknown protein
## VIT_13s0156g00250  -  Cytidine/deoxycytidylate deaminase
## VIT_00s0510g00040  -  Pre-rRNA processing protein ESF1
## VIT_06s0004g07230  -  Indole-3-acetate beta-glucosyltransferase
## VIT_11s0016g03990  -  R protein  PRF  disease resistance protein
## VIT_08s0007g05900  -  GCN5 N-acetyltransferase (GNAT)
## VIT_07s0104g00550  -  GTP binding protein
## VIT_13s0019g00960  -  No hit
## VIT_04s0008g02540  -  Zinc finger (C2H2 type) family
## VIT_18s0001g10390  -  WD-repeat protein 18
## VIT_19s0014g01550  -  DAG protein, chloroplast,
## VIT_04s0008g01230  -  Harpin-induced protein
## VIT_17s0000g00910  -  ATPase domain-containing protein
## VIT_06s0061g00020  -  TTN9 (TITAN9)
## VIT_08s0032g01090  -  Ubiquitin-specific protease 8 (UBP8)
## VIT_07s0191g00060  -  Ribosomal protein S6 (RPS6B) 40S
```

```
## VIT_07s0005g01040  -  Glycosyl transferase family 8 protein
## VIT_06s0004g00450  -  Unknown protein
## VIT_07s0104g00520  -  RANGAP1 (RAN GTPASE activating protein 1)
## VIT_13s0019g00780  -  Histone H4
## VIT_07s0005g00650  -  U2B'' (U2 small nuclear ribonucleoprotein B)
## VIT_19s0027g01950  -  CPL4 (C-Terminal Domain Phosphatase-like 4)
## VIT_11s0016g04530  -  Unknown protein
## VIT_14s0036g00980  -  Transcription initiation factor TFIID subunit 10
## VIT_10s0042g01140  -  Protein-O-fucosyltransferase 1
## VIT_05s0062g00010  -  PUMILIO 5 (APUM5)
## VIT_01s0026g02120  -  Microtubule end binding protein 1 (EB1)
## VIT_08s0007g05450  -  No hit
## VIT_00s0590g00020  -  No hit
## VIT_13s0158g00100  -  putative MADS-box Agamous-like 15a (VviAGL15a)
## VIT_05s0077g00850  -  Ubiquitin-conjugating enzyme E2 O
## VIT_01s0127g00910  -  AERO1 (arabidopsis endoplasmic reticulum oxidoreductins 1)
## VIT_14s0060g02630  -  Unknown protein
## VIT_11s0037g00610  -  Unknown protein
## VIT_06s0004g06250  -  SETH2; transferase, transferring glycosyl groups
## VIT_14s0068g02120  -  CYP94B3
## VIT_06s0004g04380  -  Unknown protein
## VIT_14s0060g02270  -  Translationally-controlled tumor protein
## VIT_03s0097g00530  -  Molecular chaperone DnaK
## VIT_03s0091g01020  -  Unknown protein
## VIT_16s0100g01280  -  Cytochrome b5 domain-containing protein
## VIT_03s0017g01050  -  MBD9 (methyl-CPG-binding Domain 9)
## VIT_02s0025g02040  -  Unknown protein
## VIT_07s0141g00390  -  Glutathione reductase (NADPH)
## VIT_17s0000g06000  -  basic helix-loop-helix (bHLH) family
## VIT_01s0150g00220  -  Unknown protein
## VIT_00s0665g00040  -  RAB GTPase RABH1E
## VIT_08s0040g02300  -  ATP-dependent RNA helicase DDX52
## VIT_02s0025g01640  -  Calcineurin B 4-1
## VIT_12s0057g01340  -  Transposon protein
## VIT_07s0141g00730  -  Histone H1
## VIT_01s0011g01040  -  RPM1 (resistance to p. syringae pv maculicola 1)
## VIT_13s0084g00070  -  CYP72A53v1
## VIT_05s0094g00540  -  Acyl-[acyl-carrier-protein] desaturase
## VIT_00s2034g00010  -  No hit
## VIT_07s0005g00910  -  Zinc finger (MYND type)
## VIT_09s0002g06690  -  DNA-directed RNA polymerase III subunit C34
## VIT_12s0035g01350  -  Cysteinyl-tRNA synthetase
## VIT_00s0415g00040  -  Glycine-rich protein
## VIT_02s0488g00030  -  No hit
## VIT_11s0016g01090  -  Pentatricopeptide (PPR) repeat-containing protein
## VIT_19s0015g01130  -  Unknown protein
## VIT_19s0014g02480  -  Haloacid dehalogenase hydrolase
## VIT_04s0008g02720  -  Myosin heavy chain
## VIT_14s0060g00040  -  Retrotransposon
## VIT_00s1959g00010  -  UDP-N-acetylglucosamine pyrophosphorylase
## VIT_14s0066g01090  -  myb domain protein 24
## VIT_06s0009g01980  -  No hit
## VIT_11s0052g00500  -  Glutaredoxin
## VIT_18s0001g14110  -  Transcription factor E2F/dimerisation partner (TDP) E2F2
## VIT_05s0094g01110  -  Acetolactate synthase small subunit
```

```
## VIT_03s0063g00540  -  Pentatricopeptide (PPR) repeat-containing protein
## VIT_07s0191g00200  -  RNA helicase
## VIT_04s0044g01690  -  Glucose-inhibited division family A
## VIT_01s0011g04130  -  Elongator complex protein 2 (ELP2)
## VIT_13s0019g03920  -  Elongation factor 1-alpha 1
## VIT_11s0052g00170  -  Armadillo-like helical domain-containing
## VIT_07s0005g03730  -  Protein phosphatase 2 (formerly 2A), regulatory subunit B''
## VIT_07s0031g00660  -  Unknown protein
## VIT_07s0104g00160  -  Small nuclear ribonucleoprotein D1
## VIT_08s0007g04870  -  Zinc finger protein ATRZ-1A
## VIT_13s0064g00550  -  R protein MLA10
## VIT_07s0191g00210  -  Helicase domain-containing protein
## VIT_02s0025g00650  -  AAA-type ATPase
## VIT_00s0317g00140  -  Exocyst subunit EXO70 H2
## VIT_08s0007g00970  -  Translation initiation factor eIF-3 subunit 3
## VIT_09s0002g03500  -  No hit
## VIT_04s0023g00140  -  Pelota (PEL1)
## VIT_07s0005g02670  -  3-N-debenzoyl-2-deoxytaxol N-benzoyltransferase
## VIT_07s0005g06480  -  EMB1974 (embryo defective 1974)
## VIT_05s0020g03460  -  XS domain-containing protein
## VIT_19s0015g00830  -  Proliferating cell nuclear protein P120
## VIT_14s0068g00410  -  Transducin family protein / WD-40 repeat
## VIT_00s0198g00030  -  No hit
## VIT_08s0040g00130  -  Copper-binding family protein
## VIT_13s0064g00540  -  R protein MLA10
## VIT_09s0002g04780  -  Sulfate transporter 3.4
## VIT_14s0171g00270  -  COP9 signalosome complex subunit 3
## VIT_18s0041g01940  -  Cation exchanger (CAX10)
## VIT_11s0118g00560  -  Nodulin
## VIT_15s0021g01460  -  RWD domain-containing protein
## VIT_13s0047g01180  -  Zinc-binding protein
## VIT_19s0090g00130  -  Unknown protein
## VIT_00s0125g00270  -  Cyclin-T1-5 (CycT1;5)
## VIT_06s0004g05160  -  Pentatricopeptide (PPR) repeat-containing protein
## VIT_18s0001g02370  -  DNA replication factor C complex subunit 5
## VIT_02s0087g00790  -  F-box domain containing protein
## VIT_08s0007g02320  -  Binding
## VIT_09s0002g01880  -  Unknown protein
## VIT_01s0026g00800  -  Unknown protein
## VIT_02s0025g01420  -  Preprotein translocase YidC subunit
## VIT_18s0001g04540  -  Unknown protein
## VIT_14s0108g01680  -  Sister chromatid cohesion 2
## VIT_14s0068g00710  -  DEAD/DEAH box helicase
## VIT_18s0001g09480  -  PUMILIO 8 (APUM8)
## VIT_19s0015g01710  -  Kinesin family member 22
## VIT_11s0016g03790  -  No hit
## VIT_00s0279g00050  -  Heat shock protein binding
## VIT_15s0048g02350  -  Prohibitin 1
## VIT_03s0038g03750  -  Glycosyl transferase family 8 protein
## VIT_08s0007g00040  -  Histone H4
## VIT_08s0058g00270  -  Importin beta-2
## VIT_19s0085g00730  -  Prohibitin 1
## VIT_14s0083g00670  -  No hit
## VIT_01s0011g01000  -  RPM1 (resistance to p. syringae pv maculicola 1)
```

```
## VIT_04s0023g01890  -  Unknown protein
## VIT_05s0077g01400  -  FAR1-related sequence 11
## VIT_09s0002g07760  -  NADPH quinone oxidoreductase
## VIT_00s1881g00010  -  No hit
## VIT_18s0122g00840  -  EMB2261 (embryo defective 2261)
## VIT_12s0028g02640  -  GTP-binding protein typA/bipA
## VIT_02s0012g00030  -  Unknown
## VIT_13s0067g03530  -  Nitrilase
## VIT_17s0000g03030  -  DEAH (Asp-Glu-Ala-His) box polypeptide 36
## VIT_03s0038g00110  -  Nucleolar protein 4 NOP4
## VIT_11s0016g05040  -  Unknown protein
## VIT_12s0028g03770  -  DNA-directed RNA polymerase II subunit B
## VIT_03s0088g01050  -  Transposon protein, Mutator sub-class
## VIT_05s0102g00250  -  Unknown protein
## VIT_13s0019g00440  -  Auxin-regulated protein
## VIT_07s0095g00700  -  RNA-binding region RNP-1
## VIT_15s0048g01660  -  Unknown protein
## VIT_02s0025g02030  -  No hit
## VIT_14s0128g00360  -  Nucleolar GTPase
## VIT_15s0046g02180  -  Pentatricopeptide (PPR) repeat-containing protein
## VIT_19s0015g01890  -  BRL3 (BRI1-like 3)
## VIT_00s0337g00010  -  Salt tolerance protein 2
## VIT_09s0002g03770  -  Mitotic phosphoprotein N' end (MPPN)
## VIT_00s2317g00010  -  RAB GDP dissociation inhibitor 2 ATGDI2
## VIT_00s0144g00010  -  CCR4-NOT transcription complex, subunit 1
## VIT_04s0008g05770  -  CBL-interacting protein kinase 25 (CIPK25)
## VIT_01s0010g02080  -  Chr019/ETL1 (chromatin remodeling 19)
## VIT_07s0005g03110  -  Tetratricopeptide repeat (TPR)-containing protein
## VIT_04s0159g00040  -  Glutathione transferase microsomal GSTMI
## VIT_10s0116g01560  -  U3 small nucleolar RNA-associated protein 6
## VIT_15s0046g00190  -  Laccase
## VIT_06s0004g01840  -  Cytochrome c-type biogenesis protein
## VIT_14s0060g00570  -  Global transcription factor group C 102
## VIT_06s0004g04650  -  Metallothionein
## VIT_06s0004g00490  -  ERF/AP2 Gene Family (VvERF050)
## VIT_07s0005g02140  -  Unknown protein
## VIT_11s0016g03870  -  Subtilisin
## VIT_08s0040g01230  -  Auxin transport protein (PIN3)
## VIT_04s0008g03040  -  RNA recognition motif (RRM)-containing
## VIT_07s0005g00270  -  Unknown protein
## VIT_04s0069g00800  -  Splicing factor
## VIT_19s0014g02180  -  No hit
## VIT_12s0059g02020  -  Glutamate formiminotransferase
## VIT_07s0005g04630  -  Transducin family protein / WD-40 repeat
## VIT_13s0019g00940  -  SLT1 (sodium- and lithium-tolerant 1)
## VIT_19s0015g01900  -  Dimethylmenaquinone methyltransferase
## VIT_04s0008g04220  -  Serine/threonine-protein kinase AFC3
## VIT_03s0097g00550  -  DNA polymerase V
## VIT_14s0068g01400  -  UPF0497 family
## VIT_13s0064g01350  -  TIF31 protein
## VIT_18s0001g11910  -  1-acyl-sn-glycerol-3-phosphate acyltransferase 4
## VIT_04s0008g06730  -  Import inner membrane translocase subunit Tim17/Tim22/Tim23
## VIT_08s0007g07450  -  Splicing factor YT521-B
## VIT_08s0007g03570  -  Anthocyanin membrane protein 1 (Anm1)
## VIT_12s0059g02440  -  TraB domain-containing protein
```

```
## VIT_02s0025g02160  -  ABC Transporter (VvGCN2 - VvABCF2)
## VIT_19s0014g04790  -  Organic cation/carnitine transporter4
## VIT_14s0066g01710  -  Leaf senescence protein
## VIT_08s0040g03060  -  Serine/threonine Protein kinase BNK1
## VIT_05s0062g01340  -  Unknown protein
## VIT_12s0034g00930  -  Band 7 family
## VIT_08s0040g02160  -  Zinc finger (C3HC4-type ring finger)
## VIT_19s0177g00110  -  Translation initiation factor eIF-1
## VIT_03s0038g02570  -  Ubiquitin-conjugating enzyme E2 O
## VIT_04s0008g03030  -  RNA recognition motif (RRM)-containing
## VIT_16s0115g00120  -  transformation/transcription domain-associated protein
## VIT_14s0171g00150  -  Avr9/Cf-9 rapidly elicited protein 57
## VIT_08s0007g06830  -  Colon cancer-associated protein Mic1 containing protein
## VIT_14s0066g01140  -  Unknown protein
## VIT_04s0008g01840  -  TT2 (transparent testa 2)
## VIT_01s0146g00130  -  GYF domain-containing protein
## VIT_16s0039g00730  -  DNA polymerase delta, subunit D
## VIT_11s0016g02050  -  Nucleosome assembly protein (NAP1)
## VIT_18s0075g00500  -  No hit
## VIT_16s0115g00170  -  Seed maturation protein PM28
## VIT_11s0037g00150  -  WRKY DNA-binding protein 32 (WRKY-14), WRKY Transcription Factor (VvWR
## VIT_09s0002g07400  -  FAR1-related sequence 6
## VIT_14s0006g03050  -  Unknown protein
## VIT_01s0011g06090  -  THO complex subunit 2
## VIT_04s0008g01430  -  APM1 (Aminopeptidase M1)
## VIT_14s0066g01190  -  Unknown protein
## VIT_08s0040g02220  -  DNA-binding protein S1FA
## VIT_13s0067g02650  -  Nucleotidyltransferase family protein
## VIT_18s0001g08370  -  Glycosyl hydrolase family 10 protein
## VIT_17s0000g05390  -  Unknown protein
## VIT_18s0001g14540  -  DEAD-box ATP-dependent RNA helicase 28
## VIT_12s0028g00090  -  Pentatricopeptide (PPR) repeat-containing protein
## VIT_03s0091g00250  -  Unknown
## VIT_12s0059g00260  -  Splicing factor STA1 (stabilized1)
## VIT_12s0055g00360  -  Pinin/SDK/memA/ protein conserved region containing protein
## VIT_10s0071g01040  -  Transcription elongation factor SPT4 1
## VIT_01s0011g03510  -  Protein kinase PKN/PRK1
## VIT_14s0036g00940  -  Transcription initiation factor TFIID subunit 10
## VIT_14s0083g00880  -  Phosphatidylinositol 4-kinase type-II
## VIT_09s0002g01870  -  Unknown protein
## VIT_19s0014g02190  -  Tyrosine aminotransferase
## VIT_09s0002g01190  -  Scarecrow transcription factor 29 (SCL29)
## VIT_17s0000g03540  -  DNA binding
## VIT_00s0220g00080  -  Rad21/Rec8
## VIT_13s0019g04590  -  Arsenite-transporting ATPase (ArsA)
## VIT_17s0000g04230  -  Translation initiation factor eIF-2B delta subunit
## VIT_05s0020g02900  -  Substrate carrier, Mitochondrial
## VIT_09s0002g02330  -  Nucleosome/chromatin assembly factor group A
## VIT_15s0048g00240  -  Phosphatidate cytidylyltransferase
## VIT_16s0050g02670  -  Spermine synthase
## VIT_19s0015g00840  -  Proliferating cell nuclear protein P120
```

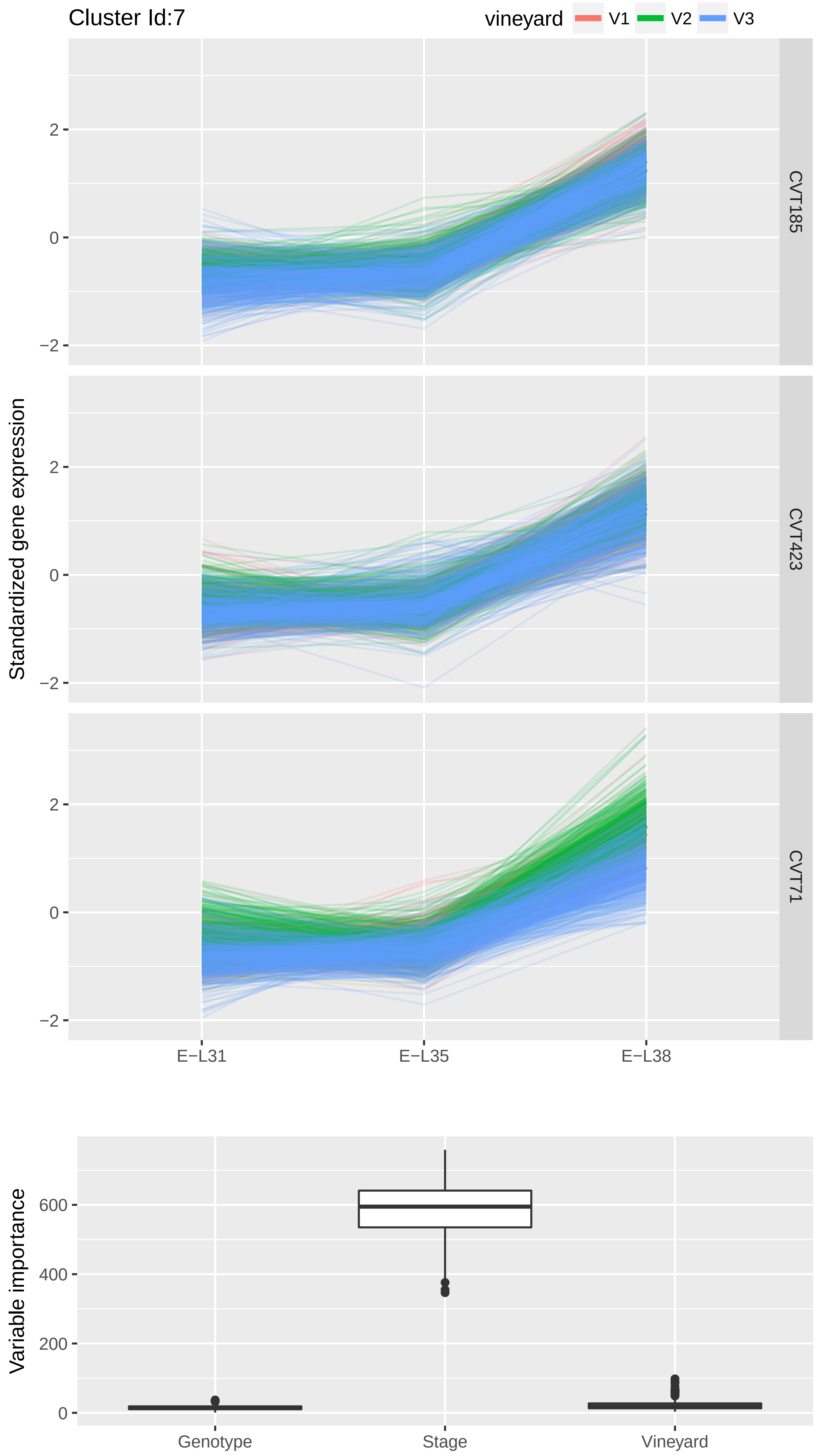

Cluster Id:7
vineyard
V1
V2
V3
Standardized gene expression
CVT185
CVT423
CVT71
2
0
−2
E−L31
E−L35
E−L38
Variable importance
600
400
200
0
Genotype
Stage
Vineyard

Cluster no. 8

```
## Number of genes in the cluster: 391
## Homogeneity Index:    0.86
## Variable importance for Stage:    Median =  569.5  - Rank =  21
## Variable importance for Clone:    Median =  14.92  - Rank =  74
## Variable importance for Vineyard: Median =  18.34  - Rank =  87
##
## Gene ID              Gene Annotation
## VIT_18s0122g00300  -  Unknown protein
## VIT_08s0058g01390  -  WRKY Transcription Factor (VvWRKY25)
## VIT_07s0129g00400  -  Ribonuclease III
## VIT_14s0006g01600  -  Unknown
## VIT_10s0003g01160  -  basic helix-loop-helix (bHLH) family
## VIT_11s0052g00090  -  Accumulation of photosystem one 3 (APO3)
## VIT_18s0122g00770  -  Unknown
## VIT_02s0012g02340  -  CYP76C4
## VIT_07s0104g01730  -  HAK5 (High affinity K+ transporter 5)
## VIT_18s0001g01460  -  No hit
## VIT_07s0005g02210  -  Transducin protein
## VIT_10s0116g00790  -  Pseudouridine synthase
## VIT_18s0001g06760  -  Clathrin assembly protein 16
## VIT_01s0010g01870  -  Salicylic acid-induced fragment 1 protein
## VIT_19s0085g01180  -  HUA1 (enhancer of AG-4 1)
## VIT_14s0060g00050  -  Nucleobase:cation symporter-1, NCS1 family
## VIT_04s0008g00610  -  Zinc finger (CCCH-type) family protein
## VIT_04s0044g01430  -  Unknown protein
## VIT_12s0028g01380  -  No hit
## VIT_09s0002g02450  -  Phosphatase
## VIT_10s0116g00170  -  S-locus lectin protein kinase
## VIT_05s0020g02610  -  Hemolysin
## VIT_16s0013g00890  -  ERF/AP2 Gene Family (VvERF077)
## VIT_06s0004g05000  -  M-phase phosphoprotein 6
## VIT_07s0005g01470  -  Unknown protein
## VIT_03s0038g03200  -  MATE efflux family protein
## VIT_19s0015g00470  -  Threonyl-tRNA synthetase
## VIT_07s0104g00250  -  basic helix-loop-helix (bHLH) family
## VIT_02s0025g03100  -  Cinnamyl alcohol dehydrogenase
## VIT_00s1217g00010  -  3-deoxy-D-arabino-heptulosonate 7-phosphate synthase
## VIT_03s0017g01940  -  Unknown
## VIT_11s0016g03940  -  Heat shock transcription factor C1
## VIT_04s0008g00780  -  Unknown
## VIT_13s0019g05100  -  Amino acid binding protein
## VIT_17s0000g01400  -  Nucleotide pyrophosphatase
## VIT_06s0004g07120  -  Gamma interferon responsive lysosomal thiol reductase family protein
## VIT_08s0007g06150  -  Ribosomal protein L6 (RPL6A) 60S
## VIT_15s0046g02070  -  Zinc finger (C3HC4-type ring finger)
## VIT_17s0000g03790  -  Unknown protein
## VIT_08s0058g00900  -  RAPTOR1B (RAPTOR1)
## VIT_10s0003g01170  -  basic helix-loop-helix (bHLH) family
## VIT_19s0014g05380  -  Monodehydroascorbate reductase
## VIT_13s0019g03520  -  No hit
## VIT_00s0181g00050  -  Unknown protein
## VIT_11s0016g01500  -  PHD finger protein alfin
## VIT_14s0060g01950  -  Pectinesterase family
## VIT_19s0014g01950  -  Anthocyanidin 3-O-glucosyltransferase
```

```
## VIT_13s0019g03940  -  Unknown protein
## VIT_06s0004g00200  -  Splicing factor YT521-B
## VIT_16s0050g01950  -  Disease resistance family protein / LRR family
## VIT_06s0004g01900  -  Homeodomain-like
## VIT_05s0077g00590  -  Ribosomal protein S6, 30S RFC3 (regulator of fatty-acid composition 3)
## VIT_18s0001g07390  -  No hit
## VIT_18s0001g07760  -  Unknown protein
## VIT_18s0089g01030  -  ERF/AP2 Gene Family (VvERF017),Dehydration Responsive Element-Binding
## VIT_13s0064g01790  -  R protein MLA10
## VIT_06s0004g07680  -  Nodulin
## VIT_00s0838g00010  -  Transcriptional corepressor LEUNIG
## VIT_07s0151g00090  -  Quinone-oxidoreductase QR1
## VIT_00s0404g00050  -  fiber protein
## VIT_15s0046g03030  -  Anthranilate phosphoribosyltransferase
## VIT_19s0090g00100  -  Unknown protein
## VIT_07s0031g00750  -  MATE efflux family protein
## VIT_12s0035g02030  -  Unknown protein
## VIT_15s0048g02560  -  Ribonucleoprotein, chloroplast 33 kDa
## VIT_02s0012g00620  -  Phosphoacetylglucosamine mutase
## VIT_04s0008g06410  -  No hit
## VIT_05s0102g00460  -  RPD1 (root primordium defective 1)
## VIT_06s0004g05670  -  Glutathione S-transferase 25 GSTU7
## VIT_18s0001g02520  -  Hypoxanthine phosphoribosyltransferase
## VIT_16s0013g02030  -  Unknown protein
## VIT_06s0004g03600  -  DNA repair protein RAD51 homolog 2
## VIT_00s0592g00020  -  Pentatricopeptide (PPR) repeat-containing protein
## VIT_14s0171g00370  -  Thymidylate kinase
## VIT_04s0008g02210  -  RPD1 (root primordium defective 1)
## VIT_13s0158g00080  -  Serine carboxypeptidase
## VIT_17s0000g07100  -  UDP-glucosyltransferase HRA25
## VIT_11s0016g01720  -  Threonine synthase
## VIT_03s0063g02230  -  Pentatricopeptide (PPR) repeat-containing protein
## VIT_09s0054g00710  -  Ribosomal protein
## VIT_04s0008g01070  -  Calmodulin-binding region IQD6
## VIT_02s0033g00040  -  No hit
## VIT_07s0005g02610  -  Preprotein translocase secA subunit
## VIT_11s0065g00850  -  Unknown protein
## VIT_15s0048g01700  -  CYP86A1
## VIT_09s0054g00720  -  Tetratricopeptide repeat (TPR)-containing
## VIT_00s0153g00050  -  Glutathione S-transferase 8 GSTU8
## VIT_13s0067g02700  -  Ribosomal protein S26 (RPS26C) 40S
## VIT_13s0064g01780  -  Disease resistance protein (NBS-LRR class)
## VIT_08s0040g02330  -  Elongation factor 1-alpha 1
## VIT_01s0026g00600  -  No hit
## VIT_09s0002g02060  -  Unknown protein
## VIT_02s0033g00320  -  PRLI-interacting factor L
## VIT_19s0014g03120  -  flavodoxin family protein
## VIT_04s0008g06870  -  Cytochrome b5 domain-containing protein
## VIT_01s0127g00900  -  No hit
## VIT_07s0005g03840  -  Transcription termination factor mitochondrial mTERF
## VIT_12s0059g00270  -  NADPH-protochlorophyllide oxidoreductase
## VIT_13s0064g00860  -  Nuclear transcription factor Y subunit A-1
## VIT_19s0014g00210  -  Ubiquitin-specific protease 23 (UBP23)
## VIT_09s0002g02490  -  Dof zinc finger protein 1
## VIT_12s0034g00040  -  UDP-glucose glucosyltransferase
```

```
## VIT_15s0048g02370  -  ERF/AP2 Gene Family (VvRAV4)
## VIT_04s0023g01090  -  Unknown
## VIT_18s0089g01170  -  LHCB5 (light harvesting complex of photosystem ii 5)
## VIT_04s0008g04620  -  Expansin family protein (EXPR3)
## VIT_10s0003g01050  -  CHUP1 (chloroplast unusual positioning 1)
## VIT_19s0014g00500  -  Receptor kinase 2
## VIT_01s0011g03460  -  No hit
## VIT_04s0023g00830  -  Calreticulin 3 (CRT3)
## VIT_01s0011g00660  -  Pentatricopeptide (PPR) repeat
## VIT_17s0000g01410  -  Nucleotide pyrophosphatase
## VIT_18s0041g00650  -  Proton-dependent oligopeptide transport (POT) family protein
## VIT_13s0067g03330  -  SNF2 domain-containing protein
## VIT_18s0072g00110  -  Light stress-responsive one-helix protein
## VIT_08s0007g06360  -  Unknown protein
## VIT_06s0004g02930  -  Zinc finger (C3HC4-type ring finger)
## VIT_02s0025g04420  -  MATE efflux family protein
## VIT_18s0001g15700  -  GLTP3 (glycolipid transfer protein 3)
## VIT_00s0220g00110  -  Acetolactate synthase small subunit
## VIT_01s0011g02750  -  Nudix hydrolase 1
## VIT_10s0003g02460  -  SRG1 (senescence-related gene 1) oxidoreductase
## VIT_13s0064g00320  -  R protein  PRF  disease resistance protein
## VIT_05s0077g02150  -  Chalcone reductase
## VIT_07s0104g01710  -  HAK5 (High affinity K+ transporter 5)
## VIT_18s0001g03660  -  Aspartokinase-homoserine dehydrogenase
## VIT_19s0090g01770  -  Transcription termination factor mitochondrial mTERF
## VIT_19s0027g01190  -  Phenylalanyl-tRNA synthetase class IIc
## VIT_06s0004g07330  -  No hit
## VIT_08s0007g02180  -  Signal peptidase I
## VIT_09s0054g01230  -  Beta-amyrin synthase
## VIT_19s0135g00060  -  Glyoxalase II
## VIT_05s0094g00410  -  Unknown protein
## VIT_01s0026g02640  -  UbiE/COQ5 methyltransferase
## VIT_18s0001g00760  -  Plastocyanin, chloroplast precursor
## VIT_13s0067g01080  -  Protein kinase
## VIT_00s0265g00170  -  Lipoxygenase
## VIT_19s0027g01200  -  Phenylalanyl-tRNA synthetase class IIc
## VIT_07s0005g01480  -  PMR5 (powdery mildew resistant 5)
## VIT_18s0001g12940  -  Dihydrofolate reductase-thymidylate synthase
## VIT_18s0001g09070  -  ZCW7 protein
## VIT_00s0684g00030  -  SEN1 (dark inducible 1)
## VIT_08s0058g01430  -  PRC-barrel
## VIT_18s0041g00690  -  basic helix-loop-helix (bHLH) family
## VIT_10s0003g00580  -  ERF/AP2 Gene Family (VvERF075)
## VIT_09s0002g02830  -  Light repressible receptor protein kinase
## VIT_07s0005g03190  -  ERF/AP2 Gene Family (VvERF096)
## VIT_07s0005g03890  -  Transcription termination factor mitochondrial mTERF
## VIT_00s0670g00020  -  GCN5 N-acetyltransferase (GNAT)
## VIT_11s0016g01450  -  Unknown protein
## VIT_04s0008g01100  -  cytochrome P450 (CYP711A1; MAX1) (VvMAX1)
## VIT_15s0046g03380  -  Thiol protease
## VIT_13s0064g01770  -  No hit
## VIT_08s0007g06340  -  Unknown protein
## VIT_17s0000g08920  -  Ribitol dehydrogenase
## VIT_04s0023g00770  -  Unknown protein
```

```
## VIT_07s0255g00040  -  Cell division cycle associated 7 CDCA7
## VIT_02s0025g01090  -  Unknown protein
## VIT_05s0020g01410  -  Ribosome 60S subunit biogenesis protein
## VIT_13s0074g00540  -  Lysine histidine transporter 1
## VIT_16s0098g00990  -  No hit
## VIT_16s0050g01680  -  UDP-glucose: anthocyanidin 5,3-O-glucosyltransferase
## VIT_03s0017g01210  -  Phosphate-induced protein 1
## VIT_12s0028g03200  -  Rooty/superroot1
## VIT_09s0018g01960  -  Unknown protein
## VIT_09s0002g05600  -  ABC transporter g family pleiotropic drug resistance 12 PDR12
## VIT_08s0058g00750  -  Binding
## VIT_02s0033g01270  -  Pentatricopeptide (PPR) repeat-containing protein
## VIT_15s0046g03350  -  Cleavage and polyadenylation specificity factor 5
## VIT_01s0010g03080  -  Unknown
## VIT_05s0094g01340  -  Thiopurine methyltransferase
## VIT_00s0184g00170  -  No hit
## VIT_01s0010g00720  -  Unknown
## VIT_03s0063g00210  -  Receptor protein kinase
## VIT_19s0014g03930  -  TRM5 tRNA methyltransferase 5
## VIT_11s0052g00930  -  Transcription factor, E2F and DP-related E2FR1
## VIT_18s0001g14270  -  Gibberellin-regulated protein 1 (GASA1)
## VIT_03s0063g02360  -  Epicotyl-specific tissue protein
## VIT_07s0031g01890  -  No hit
## VIT_07s0005g01800  -  Agenet domain-containing protein
## VIT_12s0028g02560  -  Sodium/calcium exchanger family protein
## VIT_18s0001g11320  -  Ent-kaurene oxidase
## VIT_05s0062g00780  -  Rho GDP-dissociation inhibitor 2
## VIT_18s0001g11730  -  No hit
## VIT_07s0031g00640  -  Splicing factor, arginine/serine-rich 16
## VIT_10s0003g01980  -  RKF1 (receptor-like kinase in flowers 1)
## VIT_04s0044g01490  -  2-oxoisovalerate dehydrogenase alpha subunit, mitochondrial precursor
## VIT_12s0057g00630  -  LHCB2.1 (Photosystem II light harvesting complex gene 2.1)
## VIT_17s0000g01790  -  4-coumarate-CoA ligase 2
## VIT_10s0003g00090  -  DRM1 dormancy/auxin associated
## VIT_16s0039g01410  -  Tocopherol O-methyltransferase, chloroplast precursor
## VIT_10s0003g01310  -  Unknown protein
## VIT_17s0000g05300  -  Pre-mRNA cleavage complex II protein Clp1
## VIT_11s0052g01140  -  Membrane protein
## VIT_08s0007g01050  -  Aldo-keto reductase
## VIT_09s0018g01680  -  Unknown protein
## VIT_15s0024g01230  -  No hit
## VIT_19s0014g04730  -  Unknown protein
## VIT_01s0011g03470  -  ERF/AP2 Gene Family (VvERF122)
## VIT_05s0077g00560  -  Serine carboxypeptidase S10
## VIT_08s0058g00470  -  Abscisic acid receptor PYL4  RCAR10
## VIT_16s0050g02400  -  ERF/AP2 Gene Family (VvERF054)
## VIT_14s0030g00210  -  Sugar transporter ERD6-like 5
## VIT_14s0060g00640  -  Glucose-6-phosphate dehydrogenase
## VIT_00s0323g00050  -  Invertase/pectin methylesterase inhibitor
## VIT_10s0116g01780  -  Cationic peroxidase
## VIT_03s0038g01520  -  R protein  PRF  disease resistance protein
## VIT_00s0250g00040  -  DNA binding protein, Plastid envelope
## VIT_14s0068g00160  -  No hit
## VIT_04s0023g00700  -  Serine carboxypeptidase S10
## VIT_03s0063g00800  -  Carboxyesterase 12; CXE12
```

```
## VIT_17s0000g07420  -  EDS1 (Enhanced disease susceptibility 1)
## VIT_12s0035g00030  -  Cleavage and polyadenylation specificity factor subunit 3
## VIT_12s0057g00950  -  No hit
## VIT_03s0091g01140  -  Meprin and TRAF homology domain-containing protein
## VIT_01s0010g03380  -  Ribosomal protein L17 (RPL17B) 60S
## VIT_14s0083g00550  -  Pentatricopeptide (PPR) repeat-containing
## VIT_08s0007g07680  -  Aquaporin SIP1;1
## VIT_14s0066g01640  -  Nodulin MtN21 family
## VIT_19s0014g01830  -  Glutamate receptor protein
## VIT_07s0031g01910  -  Unknown protein
## VIT_01s0137g00730  -  Acetylcholinesterase
## VIT_09s0002g02840  -  Light repressible receptor protein kinase
## VIT_13s0064g01150  -  AT hook motif-containing protein
## VIT_01s0011g05610  -  Caleosin
## VIT_00s0823g00020  -  GCN5 N-acetyltransferase (GNAT)
## VIT_19s0027g01220  -  Phenylalanine-tRNA synthetase
## VIT_07s0104g00380  -  Pale cress protein (PAC)
## VIT_03s0038g00400  -  CRS1
## VIT_03s0063g01940  -  Unknown protein
## VIT_13s0019g02050  -  Ribulose bisphosphate carboxylase/oxygenase activase, chloroplast
## VIT_00s0591g00020  -  3-deoxy-D-arabino-heptulosonate 7-phosphate synthase
## VIT_12s0034g01240  -  GNS1/SUR4 membrane
## VIT_16s0050g02240  -  Unknown protein
## VIT_04s0008g05960  -  Unknown protein
## VIT_04s0008g02950  -  DNA topoisomerase I
## VIT_05s0020g02930  -  DNA-directed RNA polymerase III subunit C3
## VIT_09s0002g01590  -  Nuclear transcription factor Y subunit A-8
## VIT_07s0031g00130  -  Kinesin protein FRA1 (fragile fiber 1)
## VIT_14s0030g01750  -  Unknown
## VIT_12s0057g00200  -  Vesicle-associated membrane protein
## VIT_10s0116g00320  -  Unknown
## VIT_03s0038g02720  -  Proteasome 26S regulatory subunit (RPN2)
## VIT_04s0023g00880  -  Unknown
## VIT_08s0007g08920  -  Zeatin O-glucosyltransferase
## VIT_01s0026g02750  -  Carrier protein, Mitochondrial
## VIT_10s0042g00060  -  No hit
## VIT_16s0050g01710  -  Receptor serine/threonine kinase PR5K
## VIT_04s0008g02140  -  EMB2261 (embryo defective 2261)
## VIT_00s0415g00020  -  Endoribonuclease E-like protein
## VIT_08s0058g00640  -  Patellin-5
## VIT_12s0028g02280  -  Calcium-dependent protein kinase 13 CPK13
## VIT_15s0046g01470  -  Unknown protein
## VIT_19s0014g03100  -  flavodoxin family protein
## VIT_07s0104g00110  -  Unknown
## VIT_14s0030g00460  -  Stress regulated protein isoform 3
## VIT_19s0177g00280  -  DNAJ heat shock N-terminal domain-containing
## VIT_11s0016g01480  -  Nuclear transcription factor Y subunit A-3
## VIT_08s0007g00390  -  Eceriferum 3 (CER3)
## VIT_16s0098g01160  -  Subtilisin protease
## VIT_08s0007g04610  -  UGT73C2 (UDP-glucosyl transferase 73C2)
## VIT_13s0156g00010  -  SC35 splicing factor SCL30a, 30a kD
## VIT_16s0050g01980  -  EIX receptor 2
## VIT_07s0141g00920  -  CYP94A1
## VIT_18s0001g00440  -  Cytoplasmic tRNA 2-thiolation protein 1
```

```
## VIT_19s0014g03950  -  R protein  PRF  disease resistance protein
## VIT_17s0000g03680  -  Unknown
## VIT_08s0007g06480  -  Unknown protein
## VIT_19s0090g01040  -  No hit
## VIT_14s0081g00060  -  Photosystem II reaction centre W (PsbW)
## VIT_18s0001g06290  -  Ribosomal protein S6 (RPS6B) 40S
## VIT_00s0225g00150  -  Anthranilate phosphoribosyltransferase
## VIT_09s0002g05350  -  2-oxoglutarate-dependent dioxygenase
## VIT_15s0046g00290  -  Auxin response factor 18
## VIT_14s0006g02680  -  Chloroplastic RNA splicing factor 1
## VIT_18s0001g00710  -  No hit
## VIT_09s0002g05200  -  Phosphoribulokinase/uridine kinase
## VIT_11s0052g01250  -  Xyloglucan endotransglucosylase/hydrolase 23
## VIT_06s0004g06390  -  UDP-glucose glucosyltransferase
## VIT_11s0016g02630  -  Unknown protein
## VIT_09s0002g02880  -  FRK1 (FLG22-induced receptor-like kinase 1)
## VIT_08s0007g03240  -  Carbonic anhydrase precursor
## VIT_12s0028g01710  -  Unknown
## VIT_04s0044g01410  -  Photosystem I reaction center subunit N (PSAN)
## VIT_03s0063g02380  -  Specific tissue protein 2
## VIT_07s0031g02590  -  Beta-galactosidase
## VIT_15s0048g01870  -  Arginine/serine-rich splicing factor RSP41
## VIT_18s0001g08340  -  Unknown
## VIT_01s0026g00610  -  Peroxidase
## VIT_16s0013g00900  -  ERF/AP2 Gene Family (VvERF079)
## VIT_10s0003g04350  -  Photosystem I subunit X (PSAK)
## VIT_03s0038g02730  -  Mutator-like transposase
## VIT_19s0014g05360  -  Replication protein A 70 kDa DNA-binding subunit
## VIT_18s0001g05980  -  Pentatricopeptide (PPR) repeat-containing protein
## VIT_08s0032g00690  -  R,R)-butanediol dehydrogenase
## VIT_04s0023g02350  -  CYP722A1
## VIT_14s0068g01570  -  Glutaredoxin-like
## VIT_00s0160g00210  -  Myosin heavy chain
## VIT_14s0081g00480  -  WDL1 (WVD2-like 1)
## VIT_00s0707g00010  -  Heat shock protein (HSP26.5-P) 26.5 kDa class P
## VIT_04s0008g01790  -  No hit
## VIT_01s0011g03800  -  Protein kinase
## VIT_05s0020g04480  -  Pyruvate kinase
## VIT_16s0098g01080  -  Growth-regulating factor 7
## VIT_06s0009g03770  -  Phycoerythrobilin ferredoxin oxidoreductase
## VIT_17s0000g01360  -  Unknown protein
## VIT_04s0008g01880  -  Cytokinin dehydrogenase 7
## VIT_00s0187g00040  -  Cystatin
## VIT_14s0128g00290  -  Pentatricopeptide (PPR) repeat-containing
## VIT_09s0002g04320  -  Photosystem I reaction center subunit VI PSAH
## VIT_03s0017g01710  -  No hit
## VIT_14s0068g01380  -  Constans-like 14
## VIT_19s0014g03180  -  ERF/AP2 Gene Family (VvERF001),Dehydration Responsive Element-Binding
## VIT_00s0551g00040  -  DNA-directed RNA polymerase III subunit C34
## VIT_01s0011g05960  -  Trehalose-phosphatase
## VIT_07s0005g03170  -  Undecaprenyl pyrophosphate synthetase
## VIT_17s0000g03690  -  Ribulose-1,5-bisphophate carboxylase/oxygenase small subunit
## VIT_18s0072g00340  -  Ribosomal protein L3 (RPL3B) 60S
## VIT_18s0001g00700  -  Mechanosensitive ion channel
## VIT_13s0019g01840  -  Cell division cycle 20-like protein 1
```

```
## VIT_17s0000g07370  -  EDS1 (Enhanced disease susceptibility 1)
## VIT_16s0050g01720  -  Receptor serine/threonine kinase PR5K
## VIT_12s0059g00990  -  Cellulose synthase CSLB04
## VIT_17s0000g06130  -  Glutathione S-transferase 9 GSTU9
## VIT_11s0016g01120  -  Tubulin-tyrosine ligase
## VIT_13s0067g02910  -  Non-specific lipid-transfer protein
## VIT_17s0000g03530  -  Ribosomal protein S7 (RPS7A) 40S
## VIT_04s0043g00600  -  Translational activator
## VIT_14s0068g01020  -  Unknown protein
## VIT_10s0003g00130  -  ERF Domain protein 12
## VIT_00s0487g00030  -  R protein MLA10
## VIT_01s0127g00540  -  Protein disulfide isomerase
## VIT_14s0060g02250  -  Unknown protein
## VIT_14s0030g00260  -  Sugar transporter ERD6-like 3
## VIT_13s0064g00670  -  No hit
## VIT_17s0000g06680  -  Unknown protein
## VIT_00s0125g00280  -  Photosystem I reaction center subunit III (PSAF)
## VIT_18s0001g14650  -  DNA-directed RNA polymerase I subunit A12
## VIT_13s0019g02140  -  Tropinone reductase
## VIT_12s0059g01630  -  Zinc metalloproteinase
## VIT_09s0096g00110  -  Ribonuclease P/MRP protein subunit POP1
## VIT_18s0001g14980  -  3-methyl-2-oxobutanoate dehydrogenase
## VIT_01s0026g01300  -  Unknown protein
## VIT_04s0008g06690  -  Proton gradient regulation 3
## VIT_18s0001g06590  -  Pigment defective 322
## VIT_14s0081g00520  -  ERF12
## VIT_06s0004g06950  -  N-acetyltransferase hookless1 HLS1
## VIT_00s1679g00010  -  Avr9/Cf-9 rapidly elicited protein 75
## VIT_00s0207g00210  -  Photosystem II PsbO protein
## VIT_01s0127g00560  -  Protein disulfide isomerase
## VIT_18s0001g00720  -  Protein kinase
## VIT_02s0087g00720  -  Receptor protein kinase
## VIT_15s0021g00970  -  NAK-type protein kinase
## VIT_08s0058g00910  -  RAPTOR1B (RAPTOR1)
## VIT_03s0063g02340  -  Dehydration-responsive protein (RD22)
## VIT_11s0016g01400  -  PSBX (photosystem II subunit X)
## VIT_04s0008g02640  -  PAP/fibrillin family
## VIT_08s0007g02190  -  LHCB4 chlorophyll A-B binding protein CP29
## VIT_14s0066g01830  -  SAG101 (senescence-associated gene 101)
## VIT_01s0011g06080  -  Lectin protein kinase family
## VIT_12s0028g01080  -  Photosystem II oxygen-evolving complex precursor, 23kda PSBP
## VIT_07s0104g00410  -  Endo-1,3;1,4-beta-D-glucanase precursor
## VIT_04s0023g00410  -  Photosystem I subunit XI (PSAL)
## VIT_05s0020g01240  -  Geranylgeranyl pyrophosphate synthase
## VIT_09s0002g00360  -  Remorin
## VIT_01s0011g01320  -  Cholinephosphate cytidylyltransferase
## VIT_10s0003g04460  -  ABC Transporter (VvMRP19 - VvABCC19)
## VIT_04s0008g03550  -  Aquaporin TIP4;1
## VIT_01s0010g03620  -  LHCA2 (Photosystem I light harvesting complex gene 2)
## VIT_19s0015g01150  -  Kinesin 4 motor protein
## VIT_02s0025g03850  -  YDA (YODA)
## VIT_00s0479g00020  -  basic helix-loop-helix (bHLH) family
## VIT_08s0007g02740  -  IAA-amino acid hydrolase 1 (ILR1)
## VIT_06s0004g04270  -  Histone H2A.4 HTA12
```

```
## VIT_15s0046g00240  -  Lateral organ boundaries protein 1
## VIT_12s0028g02410  -  1-aminocyclopropane-1-carboxylate oxidase
## VIT_07s0141g00570  -  Photosystem II reaction centre W (PsbW)
## VIT_17s0053g00810  -  Cyclic nucleotide-binding transporter 1
## VIT_13s0074g00440  -  Spermidine synthase / putrescine aminopropyltransferase
## VIT_02s0087g00750  -  Proteinase inhibitor I4, serpin
## VIT_17s0000g04320  -  Partner of Nob1
## VIT_00s0317g00130  -  Ribosomal protein L35 (RPL35B) 60S
## VIT_04s0023g01100  -  Calmodulin
## VIT_09s0002g01060  -  Unknown protein
## VIT_00s0904g00010  -  Photosystem II oxygen-evolving enhancer protein PSBQ
## VIT_08s0056g00140  -  Unknown protein
## VIT_15s0107g00180  -  Unknown
## VIT_10s0003g05040  -  3-hydroxyisobutyrate dehydrogenase
## VIT_10s0116g00250  -  Unknown
## VIT_07s0005g04920  -  Photosystem I reaction center subunit IV B (PSAE B)
## VIT_07s0005g02540  -  Protoporphyrinogen oxidase (PPOX)
```

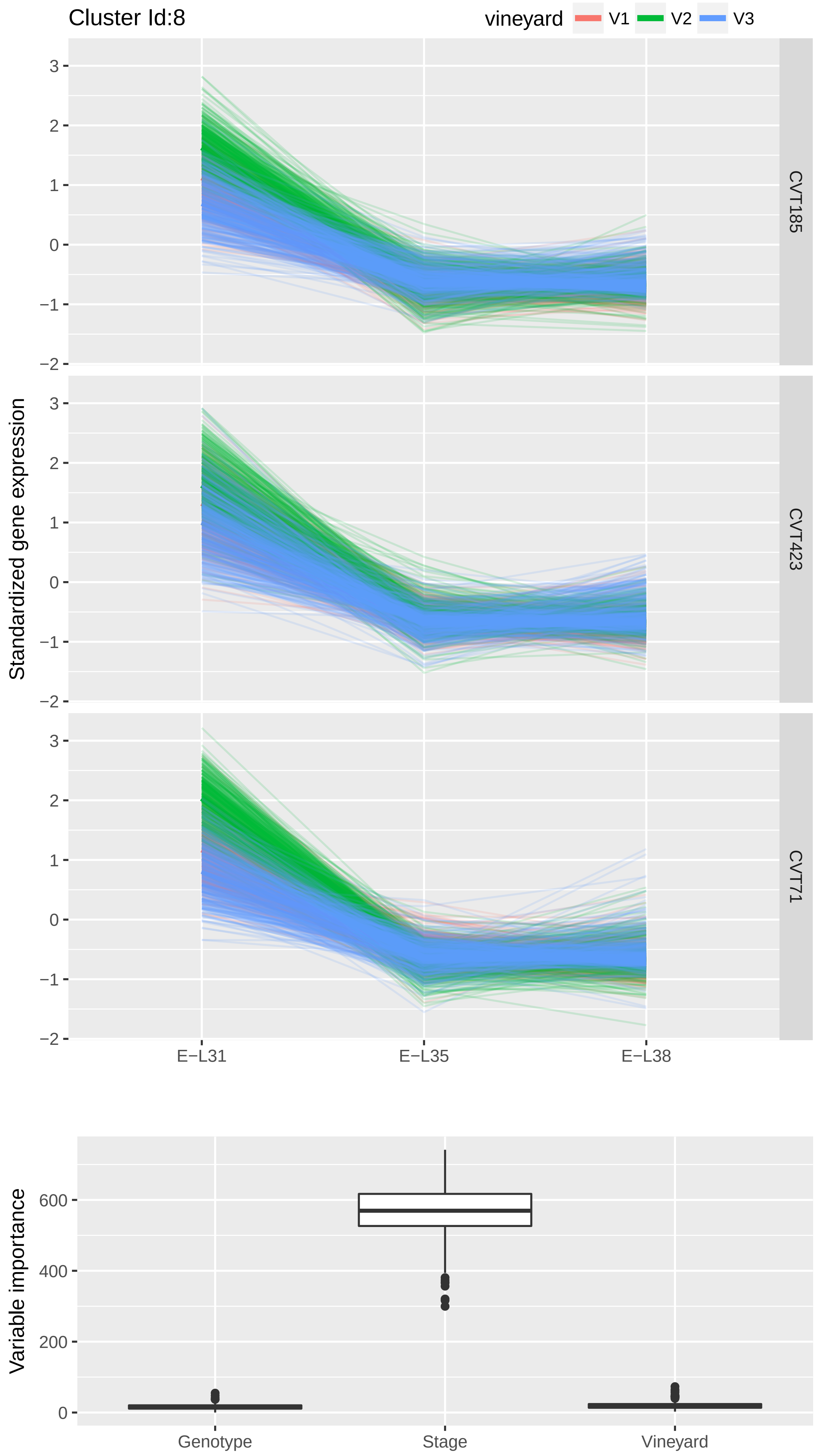

Cluster Id:8
vineyard V1 V2 V3
Standardized gene expression
CVT185
CVT423
CVT71
3
2
1
0
−1
−2
E−L31
E−L35
E−L38
Variable importance
600
400
200
0
Genotype
Stage
Vineyard

Cluster no. 9

```
## Number of genes in the cluster: 253
## Homogeneity Index:     0.9
## Variable importance for Stage:    Median =  658.6  - Rank =  4
## Variable importance for Clone:    Median =  10.19  - Rank =  106
## Variable importance for Vineyard: Median =  12.16  - Rank =  108
##
## Gene ID               Gene Annotation
## VIT_02s0012g00900  -  AT-hook protein 1 (AHP1)
## VIT_13s0067g01110  -  N-(5phosphoribosyl)anthranilate isomerase (PRAI)
## VIT_17s0119g00090  -  Unknown protein
## VIT_05s0094g00760  -  Unknown protein
## VIT_09s0002g07920  -  Vacuolar proton-inorganic pyrophosphatase
## VIT_00s0245g00060  -  No hit
## VIT_02s0025g01350  -  Primase polypeptide 1
## VIT_04s0008g03080  -  Endonuclease V family protein
## VIT_04s0044g01140  -  RNA-binding protein Y14
## VIT_16s0039g01480  -  PHD finger transcription factor
## VIT_02s0234g00100  -  Ubiquitinyl hydrolase 1
## VIT_11s0103g00300  -  Metaxin
## VIT_09s0002g04860  -  GTPase activating protein
## VIT_15s0048g01730  -  Aurora kinase 3
## VIT_18s0001g13790  -  Cytochrome P450, family 83, subfamily B, polypeptide 1
## VIT_12s0059g01310  -  SUM2 (small ubiquitin-like modifier 2)
## VIT_00s0287g00040  -  GATA transcription factor 28
## VIT_05s0077g00950  -  ATP citrate lyase a-subunit
## VIT_19s0090g00230  -  Calcineurin phosphoesterase
## VIT_00s0265g00140  -  No hit
## VIT_07s0130g00230  -  Vesicle transport protein SFT2B
## VIT_02s0241g00080  -  Glycine-rich protein-like
## VIT_13s0019g04170  -  Para-aminobenzoate (PABA) synthase
## VIT_03s0038g00300  -  Minichromosome maintenance protein 4
## VIT_01s0011g04890  -  Unknown protein
## VIT_08s0040g02370  -  DnaJ homolog, subfamily C, member 19
## VIT_03s0038g04360  -  COP9 signalosome subunit 5A
## VIT_14s0030g01600  -  DNA-binding storekeeper protein
## VIT_00s2393g00010  -  GATA transcription factor 28
## VIT_17s0000g08630  -  Unknown protein
## VIT_11s0016g02620  -  Histone acetyltransferase HAG2
## VIT_17s0000g08800  -  No hit
## VIT_08s0058g01410  -  Transcription factor S-II (TFIIS)
## VIT_08s0007g05440  -  Cation exchanger (CAX10)
## VIT_03s0038g00750  -  Ubiquitin fusion degradation protein UFD1
## VIT_12s0035g01410  -  No hit
## VIT_05s0020g04080  -  1,2-dihydroxy-3-keto-5-methylthiopentene dioxygenase 2
## VIT_04s0044g01760  -  Unknown
## VIT_03s0063g00200  -  Histidinol-phosphatase
## VIT_05s0020g04550  -  No hit
## VIT_06s0061g01070  -  Oligopeptidase A
## VIT_15s0046g00050  -  Arginine/serine-rich splicing factor RSP31 (RSP31)
## VIT_12s0178g00200  -  Actin beta/gamma 1
## VIT_18s0001g10480  -  Unknown protein
## VIT_06s0004g00160  -  Pentatricopeptide (PPR) repeat-containing protein
## VIT_10s0003g03960  -  Unknown protein
## VIT_13s0019g05080  -  Zinc finger protein ATRZ-1A
```

```
## VIT_06s0080g00550  -  EIF-4A3
## VIT_02s0154g00610  -  Pex19 protein
## VIT_12s0057g01290  -  Hydroxyproline-rich glycoprotein family protein-like
## VIT_14s0083g00960  -  Auxin transport protein (BIG)
## VIT_00s0181g00100  -  Unknown protein
## VIT_12s0035g01420  -  Zinc finger (C3HC4-type ring finger)
## VIT_14s0083g00950  -  U3 small nucleolar RNA-associated protein IMP3
## VIT_06s0004g06130  -  Thiamine biosynthesis protein ThiC
## VIT_04s0008g03170  -  Ubiquitin-specific protease 26 (UBP26)
## VIT_03s0088g00370  -  Unknown protein
## VIT_00s0125g00310  -  Unknown protein
## VIT_14s0068g00740  -  Avr9/Cf-9 rapidly elicited protein 146
## VIT_13s0067g01910  -  Unknown protein
## VIT_10s0116g00570  -  Nuclear transport factor 2B
## VIT_08s0040g00770  -  Cysteine protease inhibitor
## VIT_10s0116g00550  -  Oligosaccharide transporter OST3/OST6
## VIT_01s0011g03200  -  Transmembrane protein FT27/PFT27
## VIT_09s0002g04450  -  Helicase; 55525-51977
## VIT_06s0004g03390  -  TET3 (tetraspanin3)
## VIT_17s0000g09070  -  Histone deacetylase HDA6
## VIT_16s0098g01200  -  6-phosphogluconolactonase
## VIT_09s0002g08120  -  DEAD-box protein abstrakt
## VIT_08s0217g00030  -  Ran-binding protein 1 RanBP1
## VIT_04s0008g06260  -  Exosome complex component RRP42
## VIT_11s0118g00510  -  Ubiquitin-associated (UBA)/TS-N domain-containing
## VIT_01s0010g02370  -  Signal recognition particle subunit 72 SRP72
## VIT_01s0146g00260  -  Nodulin MtN3
## VIT_06s0004g01820  -  Zf A20 and AN1 domain-containing stress-associated protein 2
## VIT_14s0006g00430  -  Carboxylesterase
## VIT_14s0068g00840  -  RBR1 (retinoblastoma-related 1)
## VIT_07s0255g00160  -  Unknown protein
## VIT_18s0001g15010  -  F-box and leucine-rich repeat protein 1
## VIT_03s0038g02970  -  Ataxia telangiectasia and Rad3 related
## VIT_12s0178g00010  -  PHP domain-containing protein
## VIT_00s0198g00010  -  Unknown
## VIT_08s0007g04980  -  Phosphoadenosine phosphosulfate (PAPS) reductase
## VIT_10s0116g00980  -  Unknown protein
## VIT_06s0004g02910  -  Ruv DNA-helicase
## VIT_05s0094g01470  -  Poly(ADP-ribose) like glycohydrolase
## VIT_09s0002g01510  -  No hit
## VIT_12s0055g00420  -  BZIP transcription factor (VvABF-6), Basic Leucine Zipper Transcriptio
## VIT_14s0060g01360  -  Pentatricopeptide repeat
## VIT_12s0055g00330  -  Unknown protein
## VIT_07s0005g04260  -  Dolichol-phosphate mannosyltransferase
## VIT_11s0016g03000  -  Phosphoinositide 3-kinase regulatory subunit 4
## VIT_17s0000g00660  -  Phosphoglycerate mutase
## VIT_18s0001g11030  -  Binding
## VIT_04s0023g03780  -  Cell cycle checkpoint protein RAD17 (AtRAD17)
## VIT_13s0019g04620  -  OTU cysteine protease
## VIT_12s0028g01820  -  Aspartate transaminase, mitochondrial
## VIT_08s0040g02800  -  Lipin
## VIT_03s0038g03820  -  DNA-binding bromodomain-containing protein
## VIT_08s0007g08160  -  Telomere repeat binding factor Like TRFL10
## VIT_05s0094g00530  -  Calmodulin binding protein
## VIT_14s0060g01880  -  Unknown protein
```

```
## VIT_08s0007g05920  -  UDP-GlcNAc:dolichyl-phosphate GlcNAc-1-phosphate transferase
## VIT_01s0127g00450  -  Iron sulfur cluster assembly protein 1
## VIT_18s0001g14730  -  Glutaredoxin
## VIT_18s0001g07810  -  E3 ubiquitin-protein ligase HUWE1
## VIT_03s0038g02340  -  HECT ubiquitin-protein ligase 3 (KAKTUS protein)
## VIT_03s0063g00270  -  Unknown protein
## VIT_00s0399g00060  -  Alternative oxidase / immutans protein (IM)
## VIT_19s0014g01810  -  Unknown protein
## VIT_09s0002g04830  -  Ran-binding protein 1 RanBP1 domain containing
## VIT_00s2580g00010  -  U4/U6.U5 tri-snRNP-associated protein 2
## VIT_14s0083g00940  -  Auxin-independent growth promoter
## VIT_18s0001g02190  -  Switching protein 3C ATSWI3C
## VIT_02s0025g00680  -  Tiny root hair 1
## VIT_04s0023g01010  -  No hit
## VIT_09s0002g07880  -  Pyrophosphate-energized vacuolar membrane proton pump
## VIT_19s0015g01340  -  Cytochrome oxidase assembly
## VIT_09s0002g02070  -  Protein kinase PKN/PRK1
## VIT_05s0077g01430  -  Importin alpha
## VIT_10s0071g00990  -  Coiled-coil-helix-coiled-coil-helix domain
## VIT_10s0003g02790  -  Double-stranded DNA-binding protein
## VIT_06s0080g00010  -  Unknown protein
## VIT_01s0026g00230  -  U4/U6 small nuclear ribonucleoprotein PRP31
## VIT_15s0046g02840  -  Ankyrin protein kinase
## VIT_17s0000g00340  -  ATP-dependent Clp protease ATP-binding subunit (CD4B)
## VIT_01s0011g00790  -  DnaJ homolog, subfamily C, member 2 gonidia forming protein GlsA
## VIT_08s0007g08730  -  ABC Transporter (VvAOH1 - VvABCA1)
## VIT_08s0040g00610  -  GTB1 (global transcription factor group B1)
## VIT_00s0313g00020  -  Mechanosensitive ion channel MSCS-like 2
## VIT_04s0023g01440  -  SIN
## VIT_08s0032g00840  -  Sucrose-phosphatase.
## VIT_11s0118g00720  -  U5 small nuclear ribonucleoprotein component
## VIT_08s0007g04370  -  Unknown protein
## VIT_07s0129g00610  -  ABI3-interacting protein 2
## VIT_07s0005g04490  -  Transducin protein
## VIT_01s0010g01110  -  Longevity-assurance (LAG1)
## VIT_18s0001g00070  -  Pentatricopeptide (PPR) repeat-containing protein
## VIT_16s0039g02770  -  No hit
## VIT_15s0046g02370  -  Unknown protein
## VIT_10s0003g00510  -  Transcription elongation factor 1
## VIT_03s0063g00490  -  No hit
## VIT_08s0007g06350  -  Emsy N terminus domain-containing protein
## VIT_05s0020g01260  -  Pentatricopeptide (PPR) repeat-containing protein
## VIT_06s0009g01900  -  Ssu72
## VIT_02s0025g04050  -  Rab/Ypt GTPase Ara4-interacting protein
## VIT_15s0021g01160  -  RNA recognition motif (RRM)-containing protein
## VIT_13s0067g03760  -  U4/U6 small nuclear ribonucleoprotein PRP3
## VIT_00s0287g00030  -  No hit
## VIT_12s0057g01000  -  Unknown protein
## VIT_18s0122g00600  -  Unknown protein
## VIT_18s0072g00950  -  Permease nonimprinted in Prader-Willi/Angelman
## VIT_14s0060g01510  -  Importin alpha
## VIT_00s0226g00040  -  Myb family
## VIT_18s0001g00480  -  T-complex protein 1 subunit beta
## VIT_08s0007g07440  -  PUMILIO 12 (APUM12)
## VIT_05s0020g01380  -  Mitochondrial substrate carrier family protein
```

```
## VIT_06s0061g00180  -  WD40
## VIT_02s0025g02990  -  Import inner membrane translocase subunit Tim13, Mitochondrial
## VIT_15s0021g02410  -  Malate dehydrogenase, cytosolic
## VIT_09s0002g00820  -  S-2-hydroxy-acid oxidase, peroxisomal
## VIT_06s0004g05210  -  Transcription initiation factor TFIIA large subunit
## VIT_07s0104g00860  -  WD40
## VIT_05s0020g02020  -  SYD (splayed)
## VIT_03s0038g00610  -  PHD finger transcription factor
## VIT_01s0011g00070  -  Nucleolar complex protein 14 nop14
## VIT_09s0002g03170  -  CHUP1 (chloroplast unusual positioning 1)
## VIT_18s0001g08840  -  Serine carboxypeptidase S10
## VIT_15s0048g00300  -  Phosphotransferase KptA/Tpt1
## VIT_11s0016g00630  -  DEAD-box ATP-dependent RNA helicase 17
## VIT_09s0054g01610  -  Synaptic vesicle 2-related protein
## VIT_17s0053g00120  -  Succinyl-CoA ligase alpha 2 subunit
## VIT_12s0035g01090  -  Regulator of nonsense transcripts 3 UPF3
## VIT_16s0098g01570  -  Acyl-CoA binding protein 2 (ACBP2)
## VIT_01s0127g00460  -  Iron sulfur cluster assembly protein 1
## VIT_14s0171g00460  -  Nucleotide binding nubp1
## VIT_02s0087g00770  -  ABC Transporter (VvRLI1 - VvABCE1)
## VIT_06s0004g01850  -  Cytochrome c-type biogenesis protein
## VIT_17s0000g02710  -  myb domain protein 4R1
## VIT_16s0098g01780  -  Soluble starch synthase 1, chloroplast precursor
## VIT_14s0066g01080  -  ADP-ribosylation factor A1B
## VIT_12s0028g01400  -  DNA-directed RNA polymerase II subunit D
## VIT_11s0052g00590  -  Unknown protein
## VIT_18s0076g00380  -  ABC Transporter (VvGCN5 - VvABCF5)
## VIT_00s0198g00180  -  Unknown
## VIT_18s0001g15400  -  KOW domain-containing transcription factor family protein
## VIT_06s0061g01020  -  Unknown protein
## VIT_14s0060g00310  -  No hit
## VIT_04s0008g04730  -  Unknown protein
## VIT_01s0010g01100  -  Unknown protein
## VIT_08s0007g06850  -  Colon cancer-associated protein Mic1 containing protein
## VIT_18s0001g00750  -  Zinc finger (DNL type)
## VIT_09s0002g05150  -  IAA19
## VIT_18s0001g03580  -  Ubiquitin-fold modifier 1 precursor
## VIT_16s0039g02520  -  Unknown protein
## VIT_15s0048g01890  -  Peroxisomal biogenesis factor 11 (PEX11)
## VIT_04s0023g03620  -  Proteasome 26S regulatory subunit S5A (RPN10)
## VIT_03s0038g04460  -  Myb family
## VIT_18s0001g04040  -  PHD finger transcription factor
## VIT_00s0215g00030  -  Autophagy 4a (APG4a)
## VIT_15s0048g00420  -  Arginase 1
## VIT_08s0105g00460  -  Acyl-CoA oxidase ACX4
## VIT_04s0044g01790  -  Ribosome assembly protein 1
## VIT_13s0019g00880  -  Intron-binding protein aquarius
## VIT_05s0077g02200  -  Unknown protein
## VIT_00s0370g00050  -  Unknown protein
## VIT_07s0005g04950  -  Unknown protein
## VIT_11s0052g00850  -  Replicon protein A (ATRPA2/ROR1/RPA2)
## VIT_18s0001g07830  -  Transcription initiation factor TFIIA gamma chain
## VIT_11s0016g00400  -  Zinc finger (FYVE type)
## VIT_07s0031g02260  -  Unknown protein
## VIT_18s0072g01080  -  DnaJ homolog, subfamily C, member 8
```

```
## VIT_13s0084g00230  -  flavonol sulfotransferase
## VIT_19s0027g00600  -  Unknown protein
## VIT_15s0046g01370  -  Carrier protein, Mitochondrial
## VIT_15s0046g01500  -  Translation initiation factor eIF-1A
## VIT_06s0004g07530  -  U2B'' (U2 small nuclear ribonucleoprotein B)
## VIT_11s0016g02220  -  Transducin family protein / WD-40 repeat
## VIT_03s0038g03240  -  Auxin-independent growth promoter
## VIT_14s0030g01680  -  RNA recognition motif (RRM)-containing protein
## VIT_04s0008g01890  -  F-box family protein (FBL15)
## VIT_11s0052g01480  -  Salt-inducible protein kinase
## VIT_11s0016g01790  -  Unknown protein
## VIT_12s0028g01070  -  WD-repeat protein 26
## VIT_00s0507g00020  -  Membrane bound O-acyl transferase (MBOAT)
## VIT_13s0067g00160  -  NADPH2:quinone reductase
## VIT_06s0004g04230  -  Histone H2B1
## VIT_03s0038g04390  -  Dehydrin 1
## VIT_19s0015g01940  -  Peroxisomal targeting signal 1 receptor; PTS1 receptor; Pex5p
## VIT_13s0019g03560  -  Unknown protein
## VIT_18s0041g01890  -  Unknown protein
## VIT_08s0032g00960  -  Chaperone binding
## VIT_17s0000g02740  -  No hit
## VIT_05s0094g00850  -  Phosphoinositide phosphatase
## VIT_17s0000g01500  -  SWIB complex BAF60b domain-containing protein
## VIT_03s0038g00590  -  Unknown protein
## VIT_04s0023g01000  -  PRLI-interacting factor G-like protein
## VIT_03s0038g04750  -  Universal stress protein (USP) family protein
## VIT_08s0040g02880  -  Unknown protein
## VIT_16s0050g00700  -  Zinc finger (C2H2 type) family
## VIT_01s0026g00740  -  Nucleobase-ascorbate transporter 6 (NAT6)
## VIT_09s0002g00750  -  ABC Transporter (VvMDR9 - VvABCB9)
## VIT_09s0002g01160  -  Transcription initiation factor TFIID subunit D7
## VIT_06s0004g08220  -  Proteasome 26S AAA-ATPase subunit (RPT3)
## VIT_19s0177g00140  -  Thiazole biosynthetic enzyme, chloroplast (ARA6)
## VIT_04s0008g07070  -  Translation initiation factor eIF-3 subunit 9
## VIT_06s0009g02490  -  Urease accessory protein F
## VIT_14s0006g01650  -  Calcium-binding EF-hand
## VIT_18s0001g02090  -  DNA replication factor C complex subunit 4
## VIT_14s0108g00950  -  Unknown protein
## VIT_17s0000g04440  -  TGH (tough)
## VIT_00s0187g00380  -  Phosphatidate cytidylyltransferase
## VIT_06s0004g03970  -  Unknown protein
```

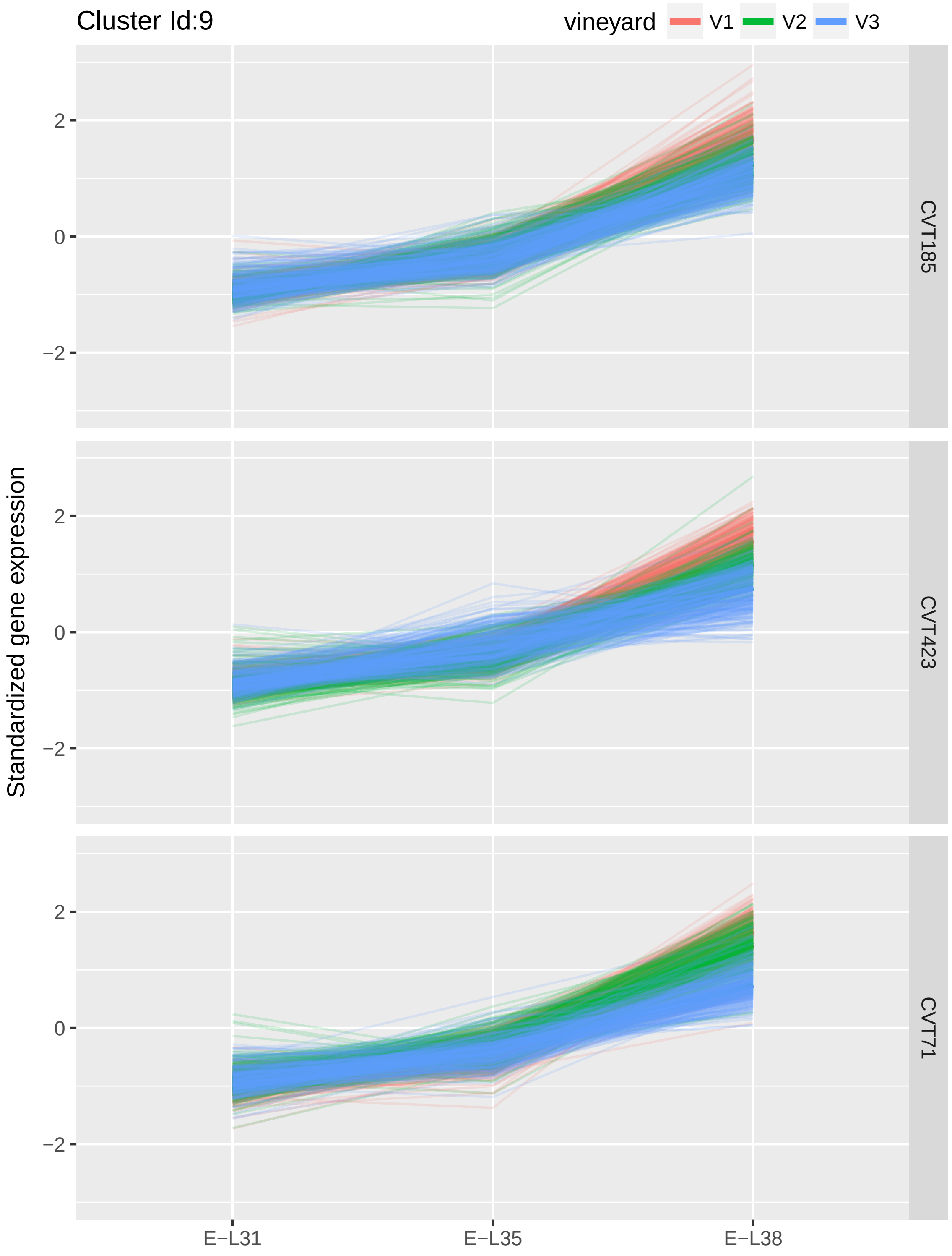

Cluster Id:9
vineyard
V1
V2
V3
Standardized gene expression
CVT185
CVT423
CVT71
2
0
−2
E−L31
E−L35
E−L38

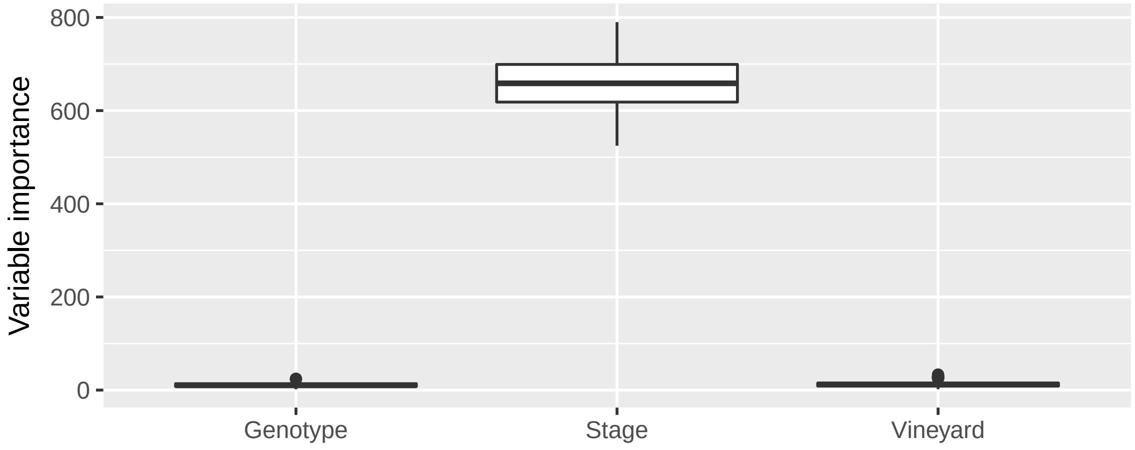

Variable importance
800
600
400
200
0
Genotype
Stage
Vineyard

Cluster no. 10

```
## Number of genes in the cluster: 122
## Homogeneity Index:    0.74
## Variable importance for Stage:    Median =  545.6  - Rank =  24
## Variable importance for Clone:    Median =  13.07  - Rank =  83
## Variable importance for Vineyard: Median =  23.18  - Rank =  80
##
## Gene ID               Gene Annotation
## VIT_07s0031g00630  -  Unknown protein
## VIT_08s0007g06680  -  PMR5 (powdery mildew resistant 5)
## VIT_18s0001g02560  -  Late embryogenesis abundant protein
## VIT_18s0001g07110  -  No hit
## VIT_09s0018g01610  -  2-dehydropantoate 2-reductase
## VIT_04s0008g06700  -  No hit
## VIT_14s0066g00350  -  Glycosyl transferase family 17 beta 1,4 N-acetylglucosaminyltransferas
## VIT_01s0011g06210  -  Constans interacting protein 6
## VIT_08s0007g08220  -  No hit
## VIT_12s0059g02030  -  Unknown
## VIT_08s0007g07140  -  Esterase/lipase/thioesterase family protein
## VIT_05s0102g01110  -  Electron transfer flavoprotein-ubiquinone oxidoreductase,precursor
## VIT_10s0071g01140  -  Unknown protein
## VIT_14s0006g00850  -  Valine--tRNA ligase
## VIT_04s0023g01030  -  LIM domain containing protein
## VIT_14s0060g00100  -  Phytochrome A (PHYA)
## VIT_06s0004g07080  -  Unknown protein
## VIT_06s0004g00460  -  Elongation factor P (EF-P)
## VIT_08s0007g08210  -  Zinc finger (CCCH-type) family protein
## VIT_11s0016g03420  -  Ring-H2 finger protein ATL4J
## VIT_03s0038g04670  -  Isoflavone reductase
## VIT_16s0013g00520  -  RAB GTPase RAB1C
## VIT_16s0100g01220  -  No hit
## VIT_13s0019g00310  -  ABC Transporter (VvMDR8 - VvABCB8)
## VIT_18s0122g01080  -  Calcium ion binding protein
## VIT_01s0026g01060  -  LRR receptor-like serine/threonine-protein kinase
## VIT_06s0004g04970  -  Scarecrow transcription factor 14 (SCL14)
## VIT_05s0051g00420  -  Glycosyltransferase family 14 Beta-1-3-galactosyl-O-glycosyl-glycoprot
## VIT_01s0026g02510  -  Pentatricopeptide (PPR) repeat-containing
## VIT_02s0025g02150  -  Glutamine-dependent NAD(+) synthetase
## VIT_14s0060g01540  -  Syntaxin 16
## VIT_14s0108g01110  -  Cystathionine gamma-synthase isoform 2
## VIT_06s0004g07430  -  Phox (PX) domain-containing protein
## VIT_10s0071g00320  -  GRAM domain-containing protein / ABA-responsive
## VIT_15s0021g01370  -  Unknown protein
## VIT_04s0008g07260  -  EDR2 (enhanced disease resistance 2)
## VIT_19s0090g00580  -  Autophagy-related protein 8C precursor ATG8C
## VIT_19s0015g01790  -  Steroid 22-alpha-hydroxylase
## VIT_02s0087g00600  -  Pentatricopeptide (PPR) repeat-containing protein
## VIT_07s0104g01050  -  Homeobox protein
## VIT_07s0005g05590  -  Unknown
## VIT_01s0011g05500  -  Beta-1,3-glucanase
## VIT_01s0010g00520  -  Unknown protein
## VIT_13s0106g00730  -  YIP1
## VIT_19s0014g01900  -  Protein transport protein Sec24
## VIT_10s0003g04960  -  Myosin heavy chain
## VIT_11s0037g00090  -  Unknown protein
```

```
## VIT_03s0038g03870  -  Calcium ion binding protein
## VIT_17s0000g01320  -  Acetohydroxyacid synthase 3
## VIT_11s0118g00490  -  Octicosapeptide/Phox/Bem1p (PB1) domain-containing protein
## VIT_19s0014g02710  -  Unknown protein
## VIT_09s0002g01430  -  Transcription factor related
## VIT_08s0040g03280  -  HCF152 (high chlorophyll fluorescence 152)
## VIT_01s0010g00530  -  UDP-glucoronosyl and UDP-glucosyl transferase
## VIT_01s0010g01770  -  Zinc finger (CCCH-type) family protein
## VIT_06s0004g02580  -  BLH8 (BEL1-like homeodomain 8)
## VIT_07s0005g00210  -  Thiamin pyrophosphokinase
## VIT_01s0146g00250  -  PBS1 (avrPphB susceptible 1)
## VIT_19s0015g01280  -  myb domain protein 103
## VIT_08s0007g07950  -  Zf A20 and AN1 domain-containing stress-associated protein 2
## VIT_01s0011g06560  -  Potassium transporter (KUP1)
## VIT_07s0151g00830  -  HSP20 chaperone
## VIT_08s0007g06190  -  Tubulin-specific chaperone C
## VIT_03s0091g00580  -  Protein Mpv17
## VIT_05s0020g00490  -  ADP-ribosylation factor A1E
## VIT_11s0016g03810  -  Unknown protein
## VIT_06s0061g00160  -  Unknown protein
## VIT_05s0020g03530  -  Late embryogenesis abundant protein D-34 (LEA D-34)
## VIT_14s0066g01890  -  Sterile alpha motif (SAM) domain-containing
## VIT_17s0000g04030  -  Endonuclease
## VIT_15s0046g01520  -  No hit
## VIT_07s0141g00790  -  Unknown protein
## VIT_13s0067g03440  -  Phosphatidylglycerolphosphate synthase (PGS1)
## VIT_16s0013g01410  -  Serine esterase
## VIT_09s0002g00380  -  1-phosphatidylinositol-3-phosphate 5-kinase
## VIT_15s0024g00850  -  Geranylgeranyl diphosphate synthase
## VIT_18s0001g05730  -  Adenosine 5'-phosphosulfate reductase 3
## VIT_04s0008g03790  -  Myb-related protein 3R-1 (Plant c-MYB-like protein 1) MYB3R1
## VIT_11s0037g00940  -  No hit
## VIT_10s0003g03710  -  Cullin-4
## VIT_14s0030g00440  -  Gibberellin receptor GID1L1
## VIT_07s0031g02570  -  CTV.22
## VIT_15s0048g02000  -  Homeodomain GLABROUS1
## VIT_02s0241g00180  -  UDP-D- glcucuronate  4-epimerase 5 GAE5
## VIT_14s0083g00520  -  Proline oxidase
## VIT_18s0001g03680  -  Protein kinase
## VIT_08s0007g07070  -  DEAD box RNA helicase RH11
## VIT_03s0038g03950  -  Phosphate carrier protein
## VIT_07s0005g02630  -  Protein transport protein SEC23
## VIT_08s0040g03260  -  Nuclear transcription factor Y subunit B-5
## VIT_09s0002g00200  -  Protein-O-fucosyltransferase 2
## VIT_10s0003g01770  -  Heat shock transcription factor A4A
## VIT_08s0007g00080  -  CUE domain containing protein
## VIT_01s0026g01960  -  SUT2 (sucrose transporter 3) (SUT2-4)
## VIT_08s0040g02830  -  Calcium-transporting ATPase 1, endoplasmic reticulum-type ECA1
## VIT_11s0052g00700  -  Serine/theronine protein kinase 2
## VIT_14s0006g02710  -  Unknown
## VIT_05s0062g00130  -  Protein transport protein Sec23A
## VIT_04s0008g03620  -  Catalytic
## VIT_12s0059g01170  -  Protein phosphatase 2 (formerly 2A), regulatory subunit B'
## VIT_11s0037g00950  -  S-adenosylmethionine decarboxylase proenzyme
## VIT_18s0001g07450  -  DnaJ homolog, subfamily B, member 12
```

```
## VIT_15s0045g01250  -  EIF4-gamma/eIF5/eIF2-epsilon domain-containing protein
## VIT_05s0020g02560  -  Ribonucleotide reductase small subunit
## VIT_09s0002g00260  -  Unknown protein
## VIT_17s0000g05120  -  ABC Transporter (VvTAP2 - VvABCB22)
## VIT_05s0029g01060  -  ATELC/ELC
## VIT_09s0002g05490  -  ABC Transporter (VvPDR12 - VvABCG42)
## VIT_08s0007g06380  -  Unknown protein
## VIT_08s0007g06140  -  Anthranilate phosphoribosyltransferase
## VIT_01s0011g04960  -  Unknown protein
## VIT_06s0004g03450  -  V-type H+-transporting ATPase subunit I
## VIT_14s0108g01520  -  Glycosyl transferase family 8 protein
## VIT_05s0020g05060  -  Cellulose synthase CSLG2
## VIT_14s0030g01910  -  Thioredoxin 1
## VIT_17s0000g01280  -  WRKY Transcription Factor (VvWRKY52)
## VIT_06s0004g06550  -  DDB2 (damaged DNA-binding 2)
## VIT_18s0075g00510  -  Calpain alkylated DNA repair protein alkB homolog 6
## VIT_15s0021g02520  -  Unknown protein
## VIT_00s0125g00420  -  Unknown
## VIT_07s0031g02130  -  Serine/threonine-protein kinase SRPK1
## VIT_06s0004g04940  -  Telomere repeat binding factor like TRFL2
```

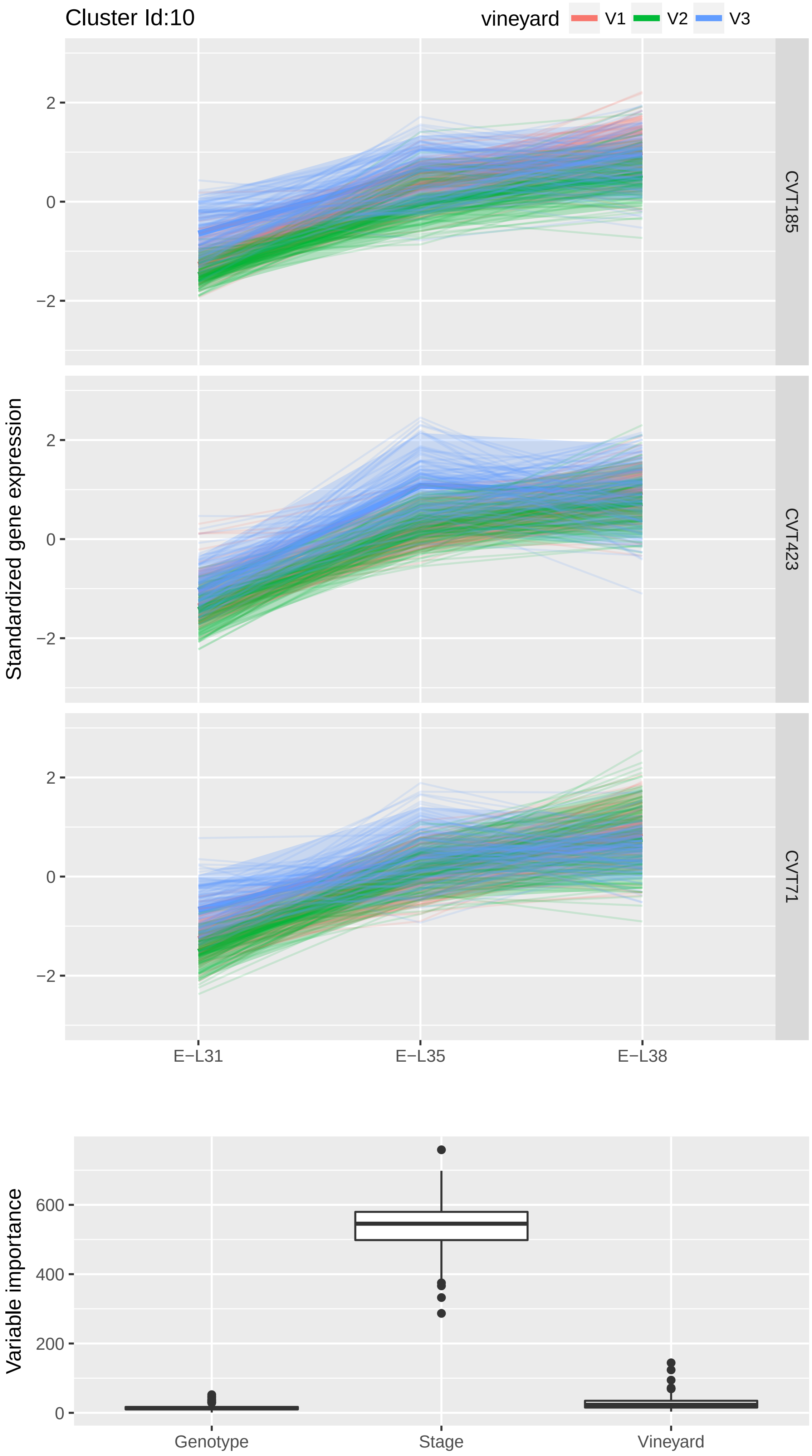

Cluster Id:10
vineyard
V1
V2
V3
Standardized gene expression
2
0
−2
CVT185
CVT423
CVT71
E−L31
E−L35
E−L38
Variable importance
600
400
200
0
Genotype
Stage
Vineyard

Cluster no. 11

```
## Number of genes in the cluster: 291
## Homogeneity Index:    0.81
## Variable importance for Stage:    Median =  275.1  - Rank =  76
## Variable importance for Clone:    Median =  18.86  - Rank =  57
## Variable importance for Vineyard: Median =  22.57  - Rank =  82
##
## Gene ID               Gene Annotation
## VIT_19s0015g02090  -  CYP72A59
## VIT_05s0020g04540  -  Beta-ketoacyl-CoA synthase
## VIT_07s0005g02950  -  AN3 (ANGUSITFOLIA3)
## VIT_08s0058g00450  -  Substrate carrier, Mitochondrial
## VIT_01s0011g02960  -  leucoanthocyanidin reductase 1 (VvLAR1) [Vitis vinifera] GENE ID: 1002
## VIT_11s0016g02350  -  Ubiquinone/menaquinone biosynthesis methyltransferase UbiE
## VIT_18s0089g00340  -  Unknown protein
## VIT_02s0012g01390  -  Unknown
## VIT_03s0038g01180  -  Auxin responsive SAUR protein
## VIT_13s0067g02510  -  Protein phosphatase
## VIT_04s0008g04050  -  RD22
## VIT_16s0050g00710  -  Unknown protein
## VIT_15s0048g02150  -  Methionine aminopeptidase 2B
## VIT_03s0038g02110  -  Co-chaperone-curved DNA binding protein A
## VIT_15s0048g01410  -  NTMC2T4/NTMC2TYPE4; NTMC2T4/NTMC2TYPE4
## VIT_00s0179g00320  -  Unknown protein
## VIT_04s0023g02830  -  forkhead-associated domain-containing protein
## VIT_14s0068g01000  -  Unknown protein
## VIT_05s0049g00780  -  No hit
## VIT_11s0052g01710  -  Xylose isomerase
## VIT_02s0025g04320  -  Thaumatin
## VIT_00s0477g00070  -  Mg2+-importing ATPase (VvPH1)
## VIT_05s0077g01010  -  HCF107 (high chlorophyll fluorescent 107)
## VIT_15s0048g01000  -  2'-hydroxy isoflavone/dihydroflavonol reductase
## VIT_03s0063g02510  -  Protein kinase
## VIT_02s0025g02120  -  1,4-alpha-D-glucan maltohydrolase
## VIT_07s0031g00330  -  MAP3K protein kinase
## VIT_18s0001g01690  -  glutathione transferase 5 GSTF5
## VIT_00s0434g00080  -  Aspartic Protease (VvAP48)
## VIT_10s0116g00540  -  Pentatricopeptide repeat-containing protein
## VIT_08s0007g01530  -  Zinc finger (C3HC4-type ring finger)
## VIT_02s0025g03140  -  Nodulin MtN21 family
## VIT_09s0002g06120  -  Biotin carboxyl carrier protein of acetyl-CoA carboxylase
## VIT_15s0046g02320  -  Unknown protein
## VIT_00s0216g00070  -  Unknown
## VIT_00s0229g00160  -  formyltetrahydrofolate deformylase
## VIT_05s0020g03970  -  Sulfate transporter 3.1 (AST12) (AtST1)
## VIT_19s0090g00210  -  DTW domain-containing protein
## VIT_19s0090g01470  -  Protease inhibitor/seed storage/lipid transfer protein (LTP)
## VIT_19s0014g02620  -  S-receptor kinase
## VIT_19s0090g00380  -  Ubiquitin thioesterase OTU1
## VIT_06s0009g00980  -  CXE carboxylesterase
## VIT_10s0003g00410  -  MLO6 (mildew resistance locus O 6)
## VIT_04s0159g00050  -  Unknown protein
## VIT_01s0011g00410  -  Nuclear transport factor 2 (NTF2)
## VIT_17s0000g02480  -  Calcium-binding protein
## VIT_10s0003g00540  -  Unknown
```

```
## VIT_08s0040g02490  -  RAB GTPase ARA3
## VIT_03s0038g01300  -  Auxin-induced SAUR
## VIT_13s0073g00690  -  Unknown protein
## VIT_01s0011g06010  -  Vacuolar iron transporter 1
## VIT_00s0304g00010  -  Unknown protein
## VIT_16s0050g00090  -  Cytochrome B561
## VIT_08s0007g08050  -  Carboxyl-terminal processing protease
## VIT_14s0083g00180  -  Cytochrome B561
## VIT_04s0008g06790  -  Biopterin transport protein BT1
## VIT_07s0005g03680  -  CCR4-NOT complex subunit CAF16
## VIT_14s0030g00330  -  Sugar transporter ERD6-like 8
## VIT_14s0066g01280  -  Kinesin light chain
## VIT_13s0019g04470  -  Adenosine kinase 2
## VIT_07s0031g02980  -  Oxysterol binding protein
## VIT_00s0524g00030  -  Armadillo/beta-catenin repeat protein / U-box domain-containing protei
## VIT_10s0116g00410  -  Gibberellin 2-beta-dioxygenase 7
## VIT_07s0031g02780  -  Cyclase/dehydrase
## VIT_00s0187g00360  -  Uclacyanin I
## VIT_06s0004g01400  -  S-receptor kinase
## VIT_14s0128g00730  -  No hit
## VIT_04s0044g01860  -  Auxin efflux carrier
## VIT_03s0038g04270  -  Ankyrin repeat
## VIT_05s0094g00770  -  Short-chain dehydrogenase/reductase (SDR)
## VIT_11s0016g05860  -  Unknown protein
## VIT_12s0035g00340  -  AAA-type ATPase
## VIT_08s0007g05750  -  Ankyrin
## VIT_13s0064g00460  -  Unknown protein
## VIT_03s0038g03250  -  Vestitone reductase
## VIT_06s0004g06610  -  Peptidyl-prolyl cis-trans isomerase ROC7 (rotamase CYP 7)
## VIT_04s0008g03600  -  Tocopherol cyclase
## VIT_13s0156g00610  -  S-receptor kinase
## VIT_19s0014g02390  -  No hit
## VIT_18s0001g00490  -  MaoC-like dehydratase domain-containing protein
## VIT_01s0011g04030  -  Reticulon family protein (RTNLB9)
## VIT_04s0008g01860  -  CYP72A58
## VIT_10s0405g00020  -  Mo25
## VIT_17s0000g00030  -  Substrate carrier family mitochondrial
## VIT_08s0056g01160  -  BDG1 (BODYGUARD1) hydrolase
## VIT_05s0020g01850  -  F-box domain containing protein
## VIT_04s0079g00260  -  Unknown protein
## VIT_12s0055g00290  -  Anthocyanidin 3-O-glucosyltransferase
## VIT_01s0010g00330  -  Clavata1 receptor kinase (CLV1)
## VIT_14s0108g00830  -  myb domain protein 94
## VIT_14s0060g01760  -  Unknown protein
## VIT_09s0002g03360  -  Calmodulin binding protein
## VIT_11s0016g05420  -  SOS2 (salt overly sensitive 2)
## VIT_03s0063g01180  -  Gibberellin 20 oxidase 2
## VIT_17s0000g04910  -  L-asparaginase
## VIT_12s0035g02080  -  Unknown protein
## VIT_02s0033g01120  -  Dehydration-responsive protein
## VIT_04s0023g02820  -  putative MADS-box Apetala  3b (VviAP3b)
## VIT_05s0020g01090  -  Basic Leucine Zipper Transcription Factor (VvbZIP15)
## VIT_07s0129g00990  -  Protein kinase
## VIT_05s0020g04450  -  G-protein gamma subunit 1 (AGG1)
## VIT_02s0087g00610  -  Unknown protein
```

```
## VIT_08s0007g08060  -  Zinc finger (HIT type) SEF
## VIT_00s2377g00010  -  Unknown protein
## VIT_11s0149g00060  -  Unknown
## VIT_15s0046g01310  -  Phytochrome A supressor spa1 (SPA1)
## VIT_15s0046g03180  -  Remorin
## VIT_09s0002g05930  -  Unknown protein
## VIT_01s0011g04910  -  Serine carboxypeptidase CPVL precursor
## VIT_00s0144g00040  -  R protein MLA10
## VIT_03s0063g01150  -  Nodulin 1A, Senescence-associated
## VIT_02s0025g04550  -  Glucose-methanol-choline (GMC) oxidoreductase
## VIT_04s0023g00590  -  Regulator of ribonuclease activity a
## VIT_10s0003g05720  -  Protein kinase MK6
## VIT_02s0012g00920  -  Conserved oligomeric complex COG6
## VIT_05s0020g04210  -  Sulfate adenylyltransferase 3
## VIT_06s0004g00260  -  Shoot1 protein
## VIT_01s0137g00180  -  B-keto acyl reductase
## VIT_00s0615g00010  -  Cinnamyl alcohol dehydrogenase
## VIT_11s0037g01400  -  Zinc finger (C3HC4-type ring finger)
## VIT_00s0532g00050  -  Basic helix-loop-helix ILR3
## VIT_01s0011g03910  -  Protein phosphatase 2C
## VIT_11s0016g03120  -  TRNA/rRNA methyltransferase (SpoU)
## VIT_09s0002g03260  -  No hit
## VIT_06s0080g01050  -  Unknown protein
## VIT_19s0090g00430  -  Ubiquitin-like 5 UBL5
## VIT_07s0005g00740  -  Endo-1,4-beta-glucanase
## VIT_07s0031g01360  -  EMB2453
## VIT_17s0000g03280  -  Alcohol dehydrogenase 7
## VIT_10s0003g01360  -  Cysteine protease (PhCAC16.5)
## VIT_17s0000g01250  -  F-box/LRR-repeat protein
## VIT_02s0012g00140  -  Novel plant snare 11
## VIT_07s0104g01800  -  Glutathione S-transferase 13 GSTF13
## VIT_06s0004g08440  -  Unknown protein
## VIT_03s0038g04420  -  DnaJ homolog, subfamily A, member 2
## VIT_19s0090g00200  -  Unknown protein
## VIT_19s0014g03400  -  Receptor protein kinase
## VIT_02s0012g01270  -  Abscisic acid receptor PYL1  RCAR12
## VIT_16s0022g00650  -  No hit
## VIT_00s0225g00090  -  Copper amine oxidase
## VIT_00s1764g00020  -  Leucine-rich repeat transmembrane protein kinase
## VIT_05s0077g00410  -  Pop3 peptide
## VIT_18s0001g00190  -  Cell division protease FtsH
## VIT_00s0357g00100  -  Pollen Ole e 1 allergen and extensin
## VIT_00s0567g00010  -  MAPKKK5
## VIT_00s1682g00010  -  Copper amine oxidase
## VIT_04s0008g02670  -  Cryptochrome DASH
## VIT_01s0010g03460  -  Caffeoyl-CoA O-methyltransferase
## VIT_16s0098g00790  -  Copper-binding family protein
## VIT_05s0062g01360  -  Unknown protein
## VIT_17s0053g00990  -  Expansin  (VvEXPA18)
## VIT_15s0046g03780  -  No hit
## VIT_06s0004g06100  -  MYB divaricata
## VIT_07s0005g00410  -  Glycosyl hydrolase family 1 protein
## VIT_12s0035g01210  -  Cytochrome c
## VIT_18s0001g12510  -  5' nucleotidase
```

```
## VIT_08s0007g08030  -  No hit
## VIT_07s0031g00050  -  Cysteine proteinase
## VIT_01s0011g06580  -  Unknown protein
## VIT_06s0004g01050  -  Calcineurin phosphoesterase
## VIT_18s0001g14810  -  Lipase 3 (EXL3) family II extracellular
## VIT_18s0001g09500  -  CYP81B2v1
## VIT_18s0001g05570  -  Hexose transporter HT2
## VIT_00s0371g00050  -  Mannitol dehydrogenase
## VIT_04s0008g04710  -  Beta-ketoacyl-CoA synthase
## VIT_00s0214g00040  -  Lectin phloem protein 2-like B1 PP2-B1
## VIT_08s0007g04780  -  Aquaporin GAMMA-TIP
## VIT_04s0023g03470  -  DNAJ heat shock N-terminal domain-containing protein
## VIT_07s0191g00070  -  SNF1-related protein kinase 2.7 SNRK2.7 SRK2F
## VIT_00s0186g00090  -  Zinc finger (C3HC4-type ring finger)
## VIT_07s0005g00390  -  Beta Glucosidase 11
## VIT_12s0057g01210  -  Nitrate-responsive NOI protein
## VIT_16s0022g00660  -  Trehalose-6-phosphate phosphatase
## VIT_14s0066g01790  -  GASA like
## VIT_00s0144g00220  -  R protein disease resistance protein
## VIT_08s0007g00440  -  Expansin  (VvEXPA11)
## VIT_12s0028g02130  -  MAP3K delta-1 protein kinase
## VIT_14s0060g00790  -  Galactinol synthase
## VIT_12s0034g01160  -  Blue (type 1) copper domain
## VIT_00s0187g00160  -  Ripening-related protein
## VIT_18s0001g06060  -  UDP-glycosyltransferase 85A1
## VIT_09s0002g05990  -  6-4 photolyase
## VIT_18s0001g14640  -  No hit
## VIT_13s0019g01960  -  Zinc finger (C3HC4-type ring finger)
## VIT_14s0060g00280  -  RARE-cold-inducible 2B
## VIT_14s0006g02880  -  neoxanthin synthase (NSY) (VvNSY1)
## VIT_05s0094g01520  -  Late embryogenesis abundant protein
## VIT_05s0020g02810  -  Unknown protein
## VIT_02s0154g00280  -  Arachidonic acid-induced DEA1
## VIT_03s0091g01050  -  Nucleobase-ascorbate transporter 4 (NAT4)
## VIT_18s0001g06520  -  Isoamylase protein.
## VIT_05s0020g03650  -  Unknown protein
## VIT_00s0199g00270  -  Unknown protein
## VIT_17s0000g06280  -  Geranylgeranyl reductase
## VIT_13s0156g00110  -  Chaperone BCS1 mitochondrial
## VIT_17s0000g00820  -  Nodulin MtN3 family
## VIT_18s0001g02460  -  Unknown protein
## VIT_06s0004g05810  -  TPR-containing protein kinase
## VIT_00s0144g00190  -  R protein MLA10
## VIT_10s0003g05770  -  Short-chain dehydrogenase/reductase (SDR)
## VIT_09s0054g01750  -  Unknown protein
## VIT_02s0012g00280  -  Senescence-associated protein
## VIT_03s0091g00420  -  No hit
## VIT_03s0038g00940  -  Auxin-responsive
## VIT_03s0038g01390  -  Aquaporin TIP3;1
## VIT_00s0322g00010  -  Cyclin-D like protein
## VIT_13s0047g00200  -  Subtilisin
## VIT_01s0010g03910  -  Squamosa promoter-binding protein (VvSBP3)
## VIT_17s0000g05580  -  Isopiperitenol dehydrogenase
## VIT_02s0012g00910  -  Adaptor-related protein complex 2, mu 2 subunit
## VIT_09s0002g00330  -  Pectinesterase PME1
```

```
## VIT_06s0004g03980  -  Ankyrin
## VIT_00s0279g00100  -  No hit
## VIT_14s0030g01930  -  Peptide-N4-(N-acetyl-beta-glucosaminyl)asparagine amidase
## VIT_11s0016g02440  -  HRT domain containing
## VIT_03s0038g03050  -  Geranylgeranyl pyrophosphate synthetase, chloroplast precursor
## VIT_09s0002g04480  -  Kinesin family member 2/24
## VIT_05s0029g00140  -  ERF/AP2 Gene Family (VvERF005),Dehydration Responsive Element-Binding
## VIT_09s0002g04610  -  Unknown protein
## VIT_18s0001g14910  -  Mannitol dehydrogenase
## VIT_00s1194g00010  -  MAPKKK5
## VIT_11s0016g03250  -  Lachrymatory factor synthase
## VIT_05s0094g00950  -  BT3 (BTB and TAZ domain protein 3)
## VIT_12s0028g03720  -  1,3-beta-glucan synthase
## VIT_03s0038g03270  -  Protein kinase
## VIT_19s0014g04660  -  flavodoxin-like quinone reductase 1
## VIT_08s0007g00470  -  Rhomboid ATRBL1
## VIT_07s0005g04790  -  Myosin-like protein XIB
## VIT_19s0085g00650  -  Cation efflux family
## VIT_12s0035g01180  -  Protein kinase CRK1 protein
## VIT_16s0013g00860  -  Embryonic flower 2 (EMF2)
## VIT_19s0090g00560  -  Beta-1,3-galactosyltransferase sqv-2
## VIT_13s0067g02930  -  Expansin  (VvEXPA14)
## VIT_13s0019g00330  -  Aquaporin TIP1;3
## VIT_00s2342g00010  -  PUB9 (plant U- box 9)
## VIT_18s0001g14790  -  Lipase 3 (EXL3) family II extracellular
## VIT_01s0127g00360  -  S-ribonuclease binding protein SBP1
## VIT_00s0189g00080  -  ADP-ribosylation factor
## VIT_17s0000g05570  -  Receptor protein kinase
## VIT_00s1937g00010  -  Amine oxidase
## VIT_02s0025g01360  -  ERF/AP2 Gene Family (VvERF002),Dehydration Responsive Element-Binding
## VIT_18s0001g05990  -  UDP-glycosyltransferase 85A1
## VIT_13s0019g02660  -  Unknown protein
## VIT_04s0008g03570  -  Unknown protein
## VIT_04s0044g00130  -  Unknown protein
## VIT_12s0057g00700  -  Glucan endo-1,3-beta-glucosidase 3 precursor
## VIT_13s0019g00090  -  Unknown
## VIT_19s0014g01050  -  UNE1 (unfertilized embryo sac 1)
## VIT_05s0051g00660  -  Octicosapeptide/Phox/Bem1p; Protein kinase
## VIT_11s0052g00650  -  Peroxidase
## VIT_07s0005g04130  -  Acid phosphatase class B
## VIT_04s0044g01000  -  Pectinesterase family
## VIT_18s0001g04800  -  Hyperosmotically inducible periplasmic protein
## VIT_07s0191g00090  -  14-3-3 protein GF14 omega (GRF2)
## VIT_17s0000g06360  -  Expansin  (VvEXPA17)
## VIT_00s0469g00050  -  Cellulose synthase CSLE1
## VIT_07s0005g02820  -  Cathepsin B cysteine protease
## VIT_05s0020g03550  -  Lactoylglutathione lyase
## VIT_13s0019g03650  -  RARE-cold-inducible 2A
## VIT_14s0068g02190  -  Chloride channel protein B
## VIT_13s0019g04480  -  Adenosine kinase 2 (ADK2)
## VIT_06s0004g04110  -  Unknown protein
## VIT_18s0072g00380  -  Abscisic stress ripening protein 2 (ASR2)
## VIT_13s0064g01260  -  DNA-damage-repair/toleration protein (DRT100)
## VIT_19s0015g02680  -  Glutathione S-transferase 25 GSTU25
## VIT_12s0035g00380  -  AAA-type ATPase
```

```
## VIT_01s0026g00810  -  Leaf senescence protein
## VIT_01s0137g00240  -  Pectate lyase
## VIT_00s0324g00070  -  Cis-zeatin O-beta-D-glucosyltransferase
## VIT_18s0122g00850  -  Tonoplast monosaccharide transporter2
## VIT_14s0060g01430  -  Unknown protein
## VIT_07s0031g00190  -  ERF/AP2 Gene Family (VvERF007),Dehydration Responsive Element-Binding
## VIT_09s0002g03250  -  Profilin 4 (PRO4) (PFN4)
## VIT_04s0008g02800  -  SAUR_D
## VIT_06s0009g01710  -  NADH-cytochrome b5 reductase
## VIT_18s0001g10450  -  ABA-responsive element-binding protein 2 (AREB2), Basic Leucine Zipper
## VIT_08s0007g04850  -  Protein phosphatase 2 (formerly 2A), regulatory subunit B'
## VIT_06s0004g00070  -  Expansin  (VvEXPA5)
## VIT_19s0135g00070  -  Ribosomal protein L18A 60S
## VIT_04s0210g00050  -  Strictosidine synthase
## VIT_03s0063g01870  -  Cold acclimation protein WCOR413 protein beta form
## VIT_05s0094g01210  -  Amine oxidase
## VIT_08s0040g00380  -  Zinc finger (DHHC type) family
## VIT_09s0070g00480  -  LRX2 (leucine-rich repeat/extensin 2)
## VIT_17s0000g01800  -  Unknown protein
## VIT_08s0007g04560  -  PLATZ transcription factor
## VIT_06s0004g01410  -  S-receptor kinase
## VIT_13s0156g00100  -  Unknown protein
## VIT_00s0524g00010  -  Armadillo/beta-catenin repeat protein / U-box domain-containing protei
## VIT_06s0004g01510  -  Lipoxygenase (LOX2) (VvLOXA)
## VIT_17s0000g01160  -  Nodulin
```

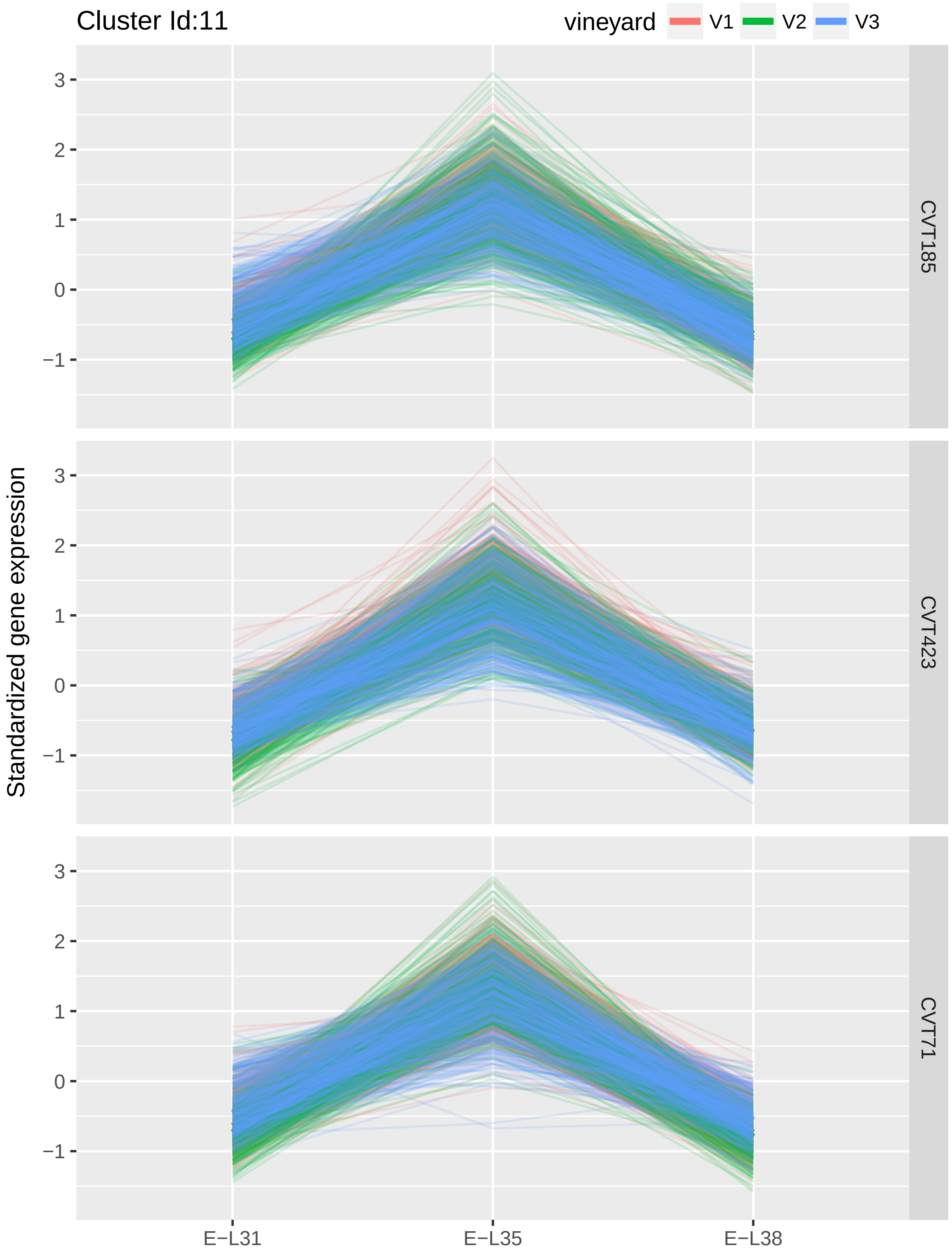
Cluster Id:11
vineyard
V1
V2
V3
Standardized gene expression
3
2
1
0
−1
CVT185
CVT423
CVT71
E−L31
E−L35
E−L38

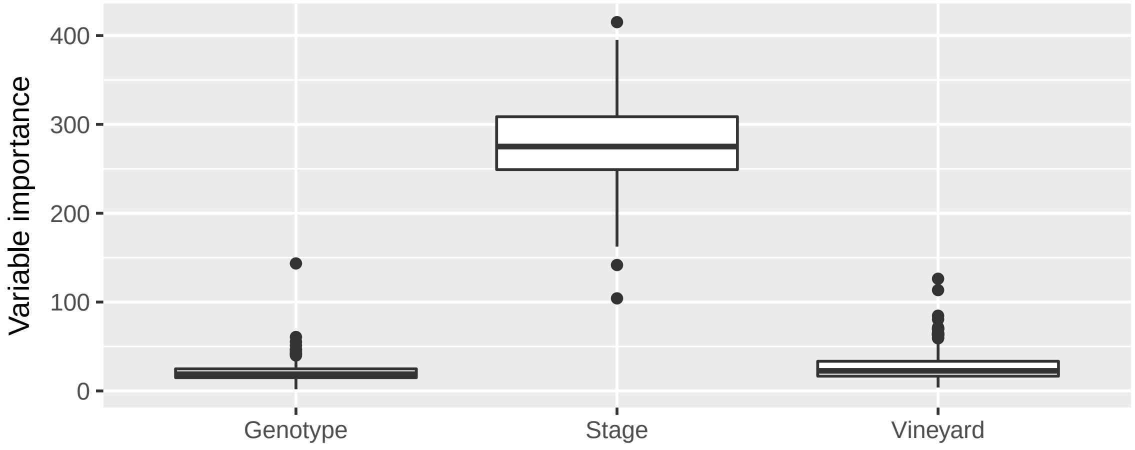
Variable importance
400
300
200
100
0
Genotype
Stage
Vineyard

Cluster no. 12

```
## Number of genes in the cluster: 102
## Homogeneity Index:    0.69
## Variable importance for Stage:    Median =  190.4  - Rank =  92
## Variable importance for Clone:    Median =  25.12  - Rank =  21
## Variable importance for Vineyard: Median =   72.9  - Rank =  26
##
## Gene ID               Gene Annotation
## VIT_11s0016g05250  -  Methyltransferase FkbM family
## VIT_18s0001g02170  -  RAB GTPase LIP1
## VIT_09s0002g00590  -  Phosphoinositide phosphatase
## VIT_00s0580g00020  -  No hit
## VIT_05s0049g01730  -  Unknown protein
## VIT_04s0008g03140  -  Unknown
## VIT_02s0025g00400  -  D111/G-patch domain-containing protein
## VIT_11s0037g00100  -  CCR4-NOT transcription complex subunit 7/8
## VIT_03s0038g04090  -  Guanine nucleotide exchange MIN7
## VIT_19s0014g03640  -  Myosin VII
## VIT_19s0090g01310  -  Transcription initiation factor TFIID subunit D5
## VIT_03s0038g04590  -  F-box domain containing protein
## VIT_02s0025g04610  -  DTDP-D-glucose 4,6-dehydratase
## VIT_14s0060g00370  -  Transposon protein, Mutator sub-class
## VIT_17s0000g04870  -  Microtubule-associated protein MBP2C
## VIT_14s0066g00660  -  No hit
## VIT_16s0022g02060  -  K+ efflux antiporter (KEA5)
## VIT_19s0014g03670  -  Unknown
## VIT_06s0004g05940  -  Uncoupling protein 1
## VIT_04s0008g02970  -  Calmodulin-binding protein
## VIT_01s0011g00780  -  Dehydration-responsive protein
## VIT_05s0020g02420  -  BSD domain-containing protein
## VIT_00s1372g00020  -  RAB GTPase RAB1B
## VIT_07s0141g00370  -  No hit
## VIT_02s0012g01170  -  Pyruvate kinase
## VIT_06s0009g02040  -  Plectin (myosin-like)
## VIT_11s0016g05500  -  Transducin protein
## VIT_06s0004g00600  -  SUB1 (short under blue light 1)
## VIT_11s0052g00670  -  Villin
## VIT_07s0005g06380  -  LUG (LEUNIG)
## VIT_00s0580g00010  -  Cellulose synthase CSLC06
## VIT_12s0035g01080  -  carotenoid isomerase (CISO1) (VvCISO2)
## VIT_08s0007g02290  -  Receptor protein kinase
## VIT_04s0023g01870  -  DNA-3-methyladenine glycosylase 1
## VIT_18s0122g01030  -  Kelch repeat-containing F-box protein
## VIT_11s0016g03830  -  Protein kinase
## VIT_09s0002g05620  -  Unknown protein
## VIT_12s0034g02010  -  Kinetochore protein NUF2
## VIT_16s0050g01180  -  Myb family
## VIT_07s0031g00480  -  Protein kinase family
## VIT_01s0026g01080  -  Serine/threonine protein phosphatase PP2A-1 catalytic subunit (PP2A1)
## VIT_04s0023g01860  -  Sugar transporter ERD6-like 6
## VIT_14s0068g01710  -  Metal-nicotianamine transporter YSL6
## VIT_12s0059g00710  -  Metalloendopeptidase
## VIT_05s0049g01160  -  Zinc finger (C3HC4-type ring finger)
## VIT_07s0031g02090  -  SH3 domain-containing protein
## VIT_00s0524g00020  -  Cellulose synthase CSLC06
```

```
## VIT_00s0360g00020  -  Unknown protein
## VIT_18s0072g00120  -  Light stress-responsive one-helix protein (OHP2)
## VIT_00s0360g00010  -  Unknown protein
## VIT_04s0044g01980  -  Unknown protein
## VIT_18s0001g09150  -  Ca2+-transporting ATPase 2 ACA2
## VIT_08s0007g02510  -  E2F3 (E2F transcription factor-3)
## VIT_03s0091g00720  -  Zinc finger (C3HC4-type ring finger)
## VIT_05s0020g02530  -  Mitogen-activated Protein Kinase (VvMPK4)
## VIT_18s0001g14530  -  Ubiquitin-protein ligase CIP8 (COP1-interacting protein 8)
## VIT_06s0004g04460  -  ALB3 (ALBINO 3)
## VIT_04s0023g01950  -  TUBBY like protein 2 TLP2
## VIT_00s0407g00080  -  Kinesin motor protein
## VIT_04s0008g06520  -  Calcineurin phosphoesterase
## VIT_19s0015g00730  -  Cellulose synthase CSLE1
## VIT_01s0011g01590  -  Ser/Thr protein kinase
## VIT_13s0019g03320  -  Acid phosphatase class B
## VIT_04s0008g06530  -  formin-2
## VIT_12s0055g00450  -  Unknown protein
## VIT_03s0091g00680  -  NPY2 (naked pins in yuc mutants 2)
## VIT_10s0003g03500  -  Katanin p60 catalytic subunit
## VIT_17s0000g00360  -  Dual specificity protein phosphatase (DsPTP1)
## VIT_14s0066g02200  -  Unknown protein
## VIT_08s0032g00800  -  carotenoid isomerase (CISO2) (VvCISO1)
## VIT_15s0046g01640  -  Abl interactor protein 1 (ABIL1)
## VIT_04s0008g05990  -  Threonine synthase, chloroplast precursor (TS)
## VIT_13s0064g00160  -  Unknown protein
## VIT_05s0049g01360  -  HrBP1-1 PAP/fibrillin family
## VIT_01s0011g03790  -  ARG1 (Altered response to gravity 1)
## VIT_13s0019g03410  -  Peroxisomal membrane protein (PMP36)
## VIT_17s0000g08580  -  ApaG domain protein
## VIT_05s0020g01890  -  CRK1 protein
## VIT_11s0016g04420  -  DnaJ homolog, subfamily C, member 13
## VIT_12s0142g00390  -  HAT-like transposase
## VIT_11s0118g00710  -  Glycerol-3-phosphate transporter
## VIT_11s0037g00230  -  Cyclic nucleotide gated channel 5
## VIT_10s0003g04880  -  Ferredoxin:nadp+ Oxidoreductase PETH
## VIT_04s0008g02080  -  GCR1 (G-protein-coupled receptor 1)
## VIT_03s0017g01780  -  far-red elongated HYPOCOTYLS 3
## VIT_02s0025g04540  -  Beta-lactamase, class A
## VIT_17s0000g01780  -  Brain protein 44
## VIT_03s0038g02820  -  Unknown protein
## VIT_12s0142g00070  -  Loricrin
## VIT_10s0116g01640  -  Glucan endo-1,3-beta-glucosidase 3 precursor
## VIT_17s0000g00390  -  Unknown protein
## VIT_07s0031g03190  -  VHS and GAT domain protein
## VIT_01s0011g04610  -  Repressor of RNA polymerase III transcription MAF1
## VIT_18s0001g00910  -  Transportin-2
## VIT_15s0021g00640  -  RAB GTPase RAB6
## VIT_14s0108g01030  -  Polypyrimidine tract-binding protein
## VIT_01s0011g05870  -  RDR1 (RNA-dependent RNA polymerase 1)
## VIT_12s0028g03340  -  Protein binding / zinc ion binding
## VIT_00s0144g00270  -  R protein MLA10
## VIT_00s0561g00030  -  Splicing factor U2AF 65 kDa subunit
## VIT_00s0144g00120  -  R protein MLA10
## VIT_10s0405g00030  -  VTC4 3'(2'),5'-bisphosphate nucleotidase
```

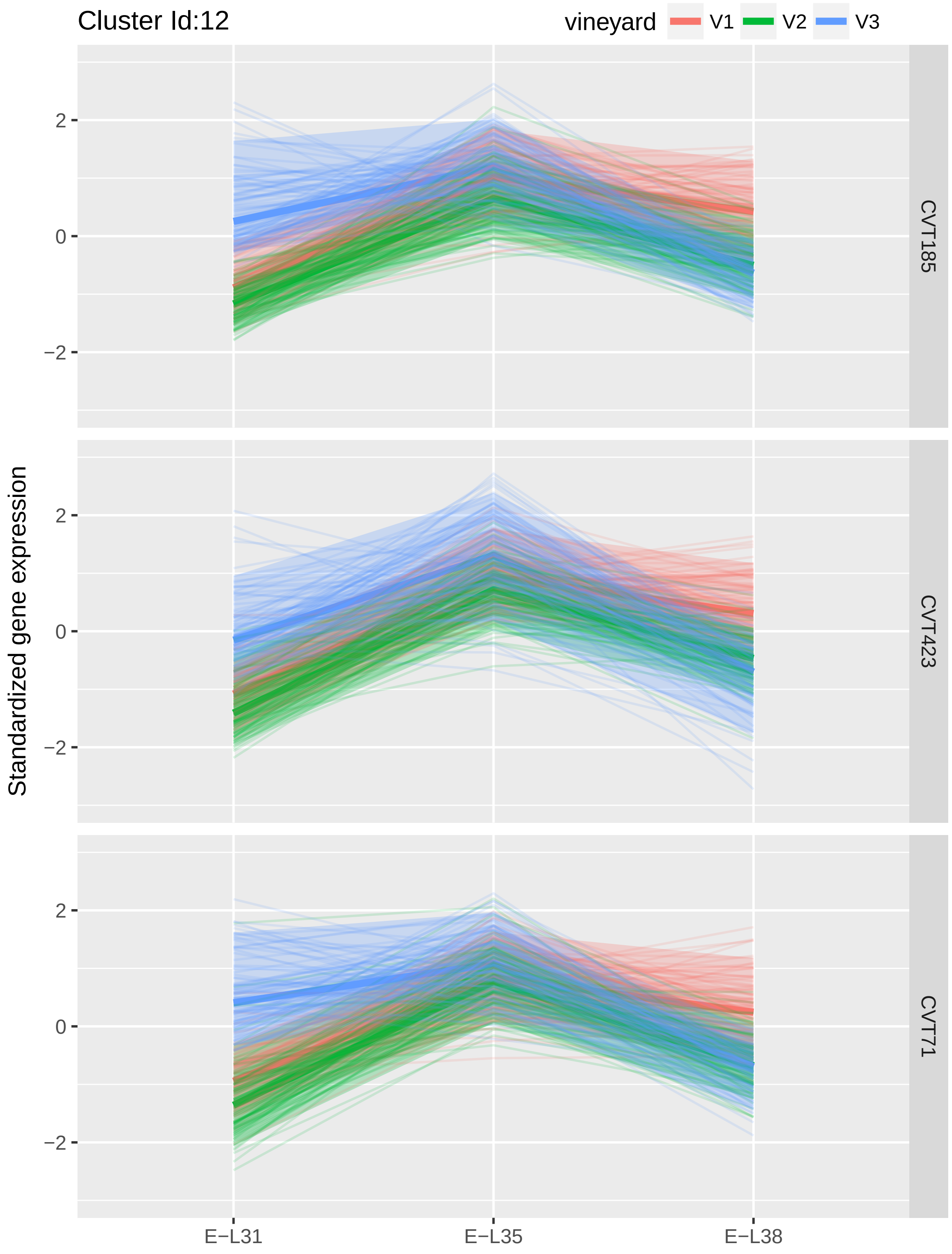

Cluster Id:12
vineyard
V1
V2
V3
Standardized gene expression
2
0
−2
CVT185
CVT423
CVT71
E−L31
E−L35
E−L38

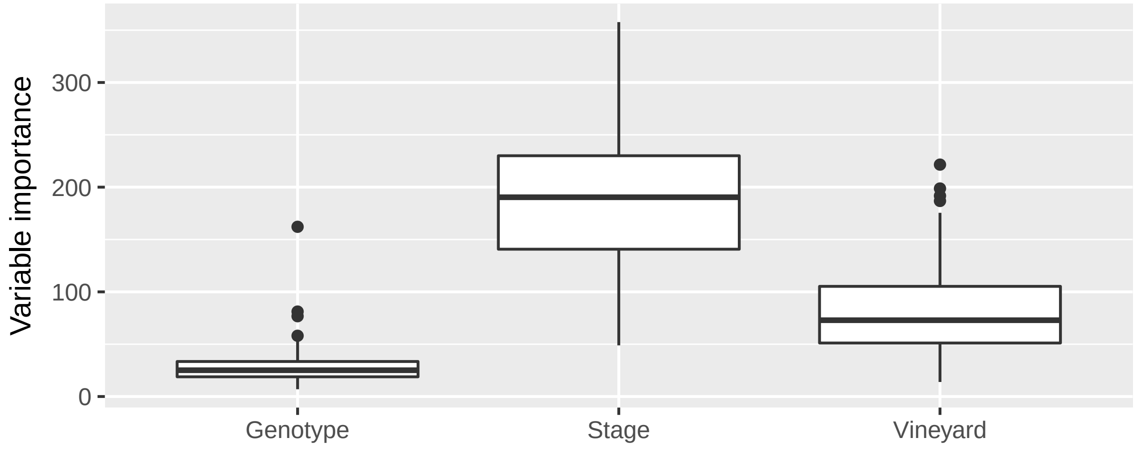

Variable importance
300
200
100
0
Genotype
Stage
Vineyard

Cluster no. 13

```
## Number of genes in the cluster: 310
## Homogeneity Index:    0.86
## Variable importance for Stage:    Median =  593.6  - Rank =  16
## Variable importance for Clone:    Median =  11.11  - Rank =  99
## Variable importance for Vineyard: Median =  15.81  - Rank =  96
##
## Gene ID               Gene Annotation
## VIT_00s0253g00070  -  Unknown
## VIT_06s0004g05100  -  PUMILIO 1 (APUM1)
## VIT_08s0032g00660  -  Sterile alpha motif (SAM) domain-containing
## VIT_13s0064g00720  -  Unknown protein
## VIT_15s0046g00740  -  Tetratricopeptide repeat (TPR)-containing
## VIT_02s0025g02750  -  PHD finger transcription factor
## VIT_17s0000g01390  -  Serine/threonine-protein kinase RIO1
## VIT_19s0090g01700  -  FRIGIDA-like 1
## VIT_05s0124g00580  -  Unknown protein
## VIT_09s0002g02540  -  RWD domain containing 1
## VIT_09s0054g00370  -  Mannosyl-oligosaccharide 1,2-alpha-mannosidase IA
## VIT_19s0093g00480  -  FK506-binding protein genes family (VvFKBP43-2)
## VIT_02s0025g03950  -  DEAD box RNA helicase
## VIT_12s0059g01970  -  Nuclear assembly factor 1
## VIT_19s0085g00610  -  No hit
## VIT_08s0007g06490  -  DNA polymerase delta, subunit B
## VIT_05s0020g01950  -  Unknown
## VIT_19s0014g01170  -  Anaphase-promoting complex component APC6
## VIT_17s0000g09380  -  WNK kinase 3
## VIT_05s0062g01080  -  Translation initiation factor eIF-3 subunit 8
## VIT_09s0002g02530  -  NADP-specific glutamate dehydrogenase
## VIT_01s0244g00120  -  Pentatricopeptide (PPR) repeat-containing protein
## VIT_06s0004g02320  -  Unknown protein
## VIT_02s0154g00460  -  Unknown protein
## VIT_18s0072g00540  -  Unknown
## VIT_18s0001g02040  -  MA3 domain-containing protein
## VIT_04s0023g02750  -  FG-GAP repeat-containing protein
## VIT_12s0028g03140  -  Transducin protein
## VIT_00s0415g00030  -  Molecular chaperone DnaK
## VIT_17s0000g05770  -  Nuclear ribonuclease Z
## VIT_19s0027g01640  -  Unknown protein
## VIT_13s0019g00490  -  PHD zinc finger protein
## VIT_02s0012g02290  -  DNAJ heat shock N-terminal domain-containing protein
## VIT_04s0008g00160  -  Casein kinase
## VIT_18s0001g02100  -  Unknown protein
## VIT_10s0003g03480  -  Multidrug resistance-associated protein 5
## VIT_07s0104g01070  -  Cohesin complex subunit SCC3
## VIT_08s0105g00390  -  Calcium Dependent Protein Kinase (VvCPK10)
## VIT_01s0137g00550  -  CYP71B34
## VIT_11s0037g00390  -  Unknown protein
## VIT_01s0010g03920  -  Unknown protein
## VIT_05s0124g00410  -  UBX domain-containing protein
## VIT_10s0003g04610  -  RNA, U3 small nucleolar interacting protein 2
## VIT_11s0016g03860  -  Raspberry3
## VIT_14s0068g01930  -  Zinc finger (C2H2 type) family
## VIT_04s0069g00410  -  Vacuolar protein-sorting-associated protein 4
## VIT_01s0011g04400  -  Origin recognition complex subunit 4
```

```
## VIT_03s0088g00330  -  F-box protein 7
## VIT_18s0164g00020  -  Transducin protein
## VIT_06s0080g00270  -  Zinc finger (C2H2 type) protein (WIP3)
## VIT_16s0098g00570  -  ABC Transporter (VvMDR19 - VvABCB19)
## VIT_01s0010g03350  -  GTPase EngC
## VIT_12s0059g01430  -  Argonaute (AGO1)
## VIT_19s0015g00700  -  Emp24/gp25L/p24
## VIT_18s0166g00310  -  ABC transporter F member 4
## VIT_02s0025g00810  -  Cation/hydrogen exchanger (CHX18)
## VIT_02s0012g01340  -  Ubiquitin-specific protease 20
## VIT_07s0129g00690  -  HOS15 (High expression of osmotically responsive genes 15)
## VIT_14s0066g01200  -  Protein kinase beta-1 subunit 5'-AMP-activated
## VIT_14s0128g00850  -  Zinc finger (Ran-binding)
## VIT_09s0002g08270  -  Leucine-rich repeat
## VIT_05s0102g00420  -  far-red impaired responsive family protein
## VIT_00s0790g00010  -  Pentatricopeptide (PPR) repeat-containing protein
## VIT_01s0010g02190  -  Lipase GDSL
## VIT_17s0000g04280  -  Zinc-binding protein
## VIT_11s0016g04260  -  Hydrolase, alpha/beta fold
## VIT_06s0004g07190  -  Unknown protein
## VIT_19s0015g01380  -  Enhancer of mRNA-decapping protein 4
## VIT_13s0106g00720  -  Unknown protein
## VIT_12s0057g00610  -  Nucleoporin Nup133
## VIT_02s0012g02300  -  DNAJ heat shock N-terminal domain-containing protein
## VIT_06s0004g05920  -  Proliferating cell nuclear antigen (PCNA)
## VIT_00s0186g00010  -  Hexaprenyldihydroxybenzoate methyltransferase
## VIT_10s0071g00780  -  DNA helicase SNF2 domain-containing protein
## VIT_00s0366g00010  -  No hit
## VIT_07s0185g00170  -  Hexaprenyldihydroxybenzoate methyltransferase
## VIT_08s0040g01390  -  Auxin-responsive family protein
## VIT_01s0137g00260  -  No hit
## VIT_00s0574g00030  -  Zinc finger (C3HC4-type ring finger)
## VIT_07s0185g00050  -  Xyloglucan:xyloglucosyl transferase
## VIT_08s0040g00910  -  CAM  interacting  protein 111
## VIT_19s0014g01790  -  Apoptosis inhibitory 5 (API5)
## VIT_13s0019g03630  -  Unknown
## VIT_00s0184g00040  -  Mini-chromosome maintenance protein MCM6
## VIT_17s0000g03850  -  Aluminum-activated malate transporter 9
## VIT_16s0039g00130  -  CTV.22
## VIT_05s0029g01080  -  Ribosome biogenesis protein Bms1
## VIT_07s0031g01930  -  myb TKI1 (TSL-KINASE INTERACTING PROTEIN 1)
## VIT_17s0000g00230  -  WD-40 repeat
## VIT_11s0016g00530  -  Adrenodoxin ferredoxin 2
## VIT_12s0059g01100  -  PRLI-interacting factor K
## VIT_14s0066g01950  -  Metalloendoproteinase 1 precursor
## VIT_14s0006g00140  -  No hit
## VIT_18s0166g00300  -  Histidinol dehydrogenase, chloroplast precursor
## VIT_05s0094g01560  -  RIO kinase 2
## VIT_05s0020g00780  -  Acidic 82 kDa protein
## VIT_04s0008g03490  -  Ankyrin repeat protein
## VIT_04s0044g01220  -  NAC domain-containing protein (VvNAC48)
## VIT_12s0028g01570  -  Zinc finger (C3HC4-type ring finger)
## VIT_05s0094g01320  -  Ankyrin repeat
## VIT_18s0041g00580  -  Proton-dependent oligopeptide transport (POT) family protein
## VIT_06s0080g00130  -  Homolog of histone chaperone HIRA
```

```
## VIT_11s0016g04130  -  Transcription factor
## VIT_06s0004g04370  -  Histone H4
## VIT_05s0077g01140  -  Basic Leucine Zipper Transcription Factor (VvbZIP14)
## VIT_16s0050g00570  -  Pectinacetylesterase
## VIT_01s0137g00520  -  CYP71B35
## VIT_07s0005g03380  -  Translocase inner membrane subunit 44-2 ATTIM44-2
## VIT_08s0007g08560  -  Cytoplasmic tRNA 2-thiolation protein 2
## VIT_08s0007g08130  -  Adenylosuccinate synthetase (ADSS)
## VIT_18s0001g01930  -  Preprotein translocase secA
## VIT_07s0031g01920  -  Protein kinase CRK1 CRK1 protein(Cdc2-related kinase 1)
## VIT_12s0028g02100  -  Ribosomal protein S5, bacterial and organelle form
## VIT_12s0035g00640  -  Ubiquitin-like domain containing CTD phosphatase 1
## VIT_15s0021g02780  -  Maternal effect embryo arrest 44
## VIT_00s0531g00010  -  HEAT repeat-containing protein
## VIT_03s0017g01290  -  ABC Transporter (VvWBC13 - VvABCG13)
## VIT_19s0015g00640  -  ABA hypersensitive germination (AHG)2
## VIT_04s0008g02620  -  Small nuclear ribonucleoprotein
## VIT_16s0013g01550  -  Multicopy supressor of IRA1  MSI1
## VIT_14s0066g02210  -  Binding
## VIT_11s0016g04940  -  Zinc finger (CCCH-type) family protein
## VIT_19s0085g00510  -  DEAD box RNA helicase (RH10)
## VIT_08s0007g09020  -  DNA helicase SNF2 domain-containing protein
## VIT_18s0001g09660  -  CYP81D2
## VIT_14s0083g00780  -  Anion exchanger adaptor protein Kanadaptin
## VIT_18s0001g03100  -  Transcription initiation factor TFIIH subunit H3
## VIT_07s0095g00690  -  No hit
## VIT_05s0102g00410  -  far-red impaired responsive family protein
## VIT_12s0057g00990  -  Beta 1,2 N-acetylglucosaminyltransferase
## VIT_12s0028g03180  -  DNA-binding protein
## VIT_04s0044g00850  -  WNK6 (Arabidopsis WNK kinase 6)
## VIT_12s0035g01700  -  Glutamine-rich protein23
## VIT_04s0023g00910  -  No hit
## VIT_16s0039g02690  -  Ankyrin repeat
## VIT_01s0150g00590  -  U2 small nuclear ribonucleoprotein A'
## VIT_12s0059g02380  -  Accumulation of photosystem one 4 apo protein 4, mitochondrial
## VIT_15s0048g01270  -  putative MADS-box Agamous-like 6a (VviAGL6a)
## VIT_19s0085g00600  -  Glyceraldehyde-3-phosphate dehydrogenase, cytosolic
## VIT_12s0035g01830  -  Survival of motor neuron-related-splicing factor 30
## VIT_19s0015g01430  -  BTB/POZ domain-containing protein
## VIT_01s0137g00560  -  CYP71B34
## VIT_13s0067g03030  -  Unknown protein
## VIT_18s0001g05600  -  Synaptonemal complex protein 1 (ZYP1A)
## VIT_09s0002g05100  -  DNA repair protein XRCC1
## VIT_15s0021g01910  -  Ribosomal RNA processing protein
## VIT_15s0046g01740  -  Unknown protein
## VIT_11s0052g00190  -  Las1
## VIT_06s0004g03580  -  CCR4-NOT transcription complex, subunit 4
## VIT_08s0007g03320  -  Glucosamine 6-phosphate N-acetyltransferase
## VIT_14s0083g00540  -  Plant adhesion molecule 1 (PAM1)
## VIT_09s0002g01860  -  DNA-directed RNA polymerase
## VIT_18s0122g01060  -  Unknown protein
## VIT_04s0008g00660  -  Early flowering 3
## VIT_11s0016g04550  -  WD-40 repeat protein (LEUNIG)
## VIT_01s0010g01570  -  Pentatricopeptide (PPR) repeat-containing
## VIT_14s0060g01870  -  Unknown protein
```

```
## VIT_10s0003g03050  -  High mobility group HMG1/2
## VIT_08s0105g00280  -  Ribosomal protein S8e
## VIT_06s0004g03890  -  Histone H4
## VIT_01s0146g00240  -  LOI1 (lovastatin insensitive 1)
## VIT_13s0064g00170  -  Ribosomal protein L1
## VIT_00s0231g00070  -  Auxin-responsive protein
## VIT_15s0046g00060  -  Arginine/serine-rich splicing factor RSP31 (RSP31)
## VIT_05s0049g02110  -  Sec20
## VIT_08s0007g07660  -  NAC domain-containing protein (VvNAC60bis)
## VIT_10s0003g01470  -  Auxin-independent growth promoter (axi 1)
## VIT_00s0432g00010  -  No hit
## VIT_04s0069g01100  -  Unknown protein
## VIT_09s0018g01740  -  Unknown
## VIT_06s0004g06870  -  Metallopeptidase M24
## VIT_06s0004g07750  -  TPA: class III peroxidase 40
## VIT_10s0116g00720  -  Pre-mRNA cleavage complex
## VIT_07s0104g01330  -  Unknown protein
## VIT_06s0004g02430  -  SPla/RYanodine receptor (SPRY) domain-containing
## VIT_05s0094g00940  -  U6 snRNA-associated Sm-like protein LSm6
## VIT_11s0052g00180  -  Las1
## VIT_09s0002g02270  -  Unknown protein
## VIT_18s0001g15530  -  Pentatricopeptide (PPR) repeat-containing
## VIT_19s0015g00190  -  Zinc transporter ZIP11
## VIT_02s0025g02790  -  Starch synthase
## VIT_19s0090g01710  -  FRIGIDA-like 2
## VIT_02s0025g03810  -  Potassium channel AKT5
## VIT_08s0007g05320  -  Essential for mitotic growth 1
## VIT_16s0039g02110  -  AAA-type ATPase
## VIT_00s0181g00060  -  Unknown protein
## VIT_08s0056g00210  -  Cyclin-dependent kinase B1;2
## VIT_02s0025g04140  -  DNA replication factor C complex subunit 2
## VIT_05s0029g01070  -  Ribosome biogenesis protein Bms1
## VIT_04s0023g03150  -  Heterogeneous nuclear ribonucleoprotein R
## VIT_17s0000g05240  -  Nuclear transport factor 2 (NTF2)
## VIT_00s0375g00040  -  NAC domain-containing protein (VvNAC03)
## VIT_14s0171g00470  -  No hit
## VIT_01s0011g02770  -  Zinc finger CCHC domain-containing protein 8
## VIT_18s0089g00160  -  1,4-beta-mannan endohydrolase
## VIT_11s0016g03450  -  Unknown protein
## VIT_12s0121g00290  -  Diacylglycerol kinase
## VIT_19s0085g00020  -  Nucleolar complex protein 3 homolog (Protein NOC3 homolog)
## VIT_18s0001g15570  -  Zinc finger (CCCH-type) family protein
## VIT_13s0074g00030  -  Transducin family protein / WD-40 repeat
## VIT_14s0066g02230  -  Binding
## VIT_19s0090g00140  -  5-alpha-taxadienol-10-beta-hydroxylase
## VIT_07s0130g00070  -  Cation-transporting ATPase
## VIT_18s0001g13560  -  Unknown protein
## VIT_11s0052g00600  -  Pentatricopeptide (PPR) repeat-containing protein
## VIT_18s0072g00210  -  Histone-lysine N-methyltransferase ASHH1
## VIT_02s0025g02400  -  N6-adenosine-methyltransferase MT-A70
## VIT_03s0097g00580  -  No hit
## VIT_13s0067g02640  -  Alpha-galactosidase precursor
## VIT_00s0920g00020  -  HEAT repeat-containing protein
## VIT_18s0001g07660  -  Hydroxyproline-rich glycoprotein
```

```
## VIT_14s0066g01230  -  Unknown protein
## VIT_03s0038g04140  -  FAR1-related sequence 5
## VIT_06s0004g07100  -  Translation initiation factor eIF-2 alpha subunit
## VIT_14s0068g00220  -  Proteasome
## VIT_02s0025g02170  -  Trehalase precursor
## VIT_08s0056g01320  -  Exosome complex exonuclease RRP6
## VIT_00s2478g00020  -  Ribosomal protein L12 (gb|SCL121A)
## VIT_11s0065g00660  -  fasciclin arabinogalactan-protein (FLA12)
## VIT_03s0038g04200  -  No hit
## VIT_12s0028g03860  -  Zinc finger (C3HC4-type ring finger) protein (RMA1)
## VIT_05s0102g00300  -  Unknown protein
## VIT_14s0066g02220  -  Binding
## VIT_08s0007g03700  -  Transcription initiation factor TFIID subunit D10
## VIT_13s0156g00360  -  Emsy N terminus domain-containing protein
## VIT_17s0000g08910  -  Unknown protein
## VIT_17s0000g01270  -  SUMO activating enzyme 1B (SAE1B)
## VIT_18s0001g12630  -  Nuclear fusion defective 5 NFD5
## VIT_13s0067g03400  -  Unknown protein
## VIT_05s0077g01180  -  Zinc knuckle
## VIT_10s0003g02860  -  UDP-N-acetylglucosamine transferase subunit ALG14, related
## VIT_07s0104g01280  -  Na+/H+ antiporter
## VIT_12s0059g00800  -  DNA helicase SNF2 domain-containing protein
## VIT_15s0048g00440  -  Unknown protein
## VIT_05s0020g02320  -  Apoptosis inhibitor
## VIT_01s0010g01670  -  Zinc knuckle
## VIT_05s0020g02350  -  SAC3/GANP
## VIT_19s0014g04680  -  Pre-mRNA-processing factor 17
## VIT_16s0098g00580  -  Zinc finger protein UFM
## VIT_18s0001g07200  -  SUPPRESSOR OF ACAULIS 51
## VIT_00s2668g00010  -  F-box family protein
## VIT_16s0050g00600  -  Unknown protein
## VIT_02s0012g00870  -  RNA-binding protein 10
## VIT_05s0029g00340  -  Heat shock transcription factor A5
## VIT_13s0139g00250  -  Translation initiation factor eIF-2B beta subunit
## VIT_12s0134g00470  -  No hit
## VIT_07s0095g00740  -  GPI transamidase component PIG-S
## VIT_04s0023g01540  -  Acidic leucine-rich nuclear phosphoprotein 32 member B
## VIT_07s0031g01880  -  Ubiquitin-specific protease 27
## VIT_19s0090g01430  -  EDM2
## VIT_12s0059g01860  -  Pentatricopeptide (PPR) repeat-containing protein
## VIT_03s0038g02430  -  WD-40 repeat protein (MSI3)
## VIT_09s0002g00620  -  Embryo sac development arrest 25
## VIT_05s0062g01050  -  Unknown protein
## VIT_06s0004g01650  -  Acetyl-CoA carboxylase, biotin carboxylase
## VIT_15s0046g00300  -  Importin-alpha re-exporter
## VIT_18s0001g01800  -  Cleavage stimulation factor 64
## VIT_13s0047g01130  -  Zfwd2 protein (ZFWD2)
## VIT_06s0061g01590  -  Peptidylprolyl isomerase domain and WD repeat-containing protein 1
## VIT_18s0001g01790  -  No hit
## VIT_15s0046g00950  -  Autophagy protein Apg6
## VIT_13s0067g02690  -  N2, N2-dimethylguanosine tRNA methyltransferases-like protein
## VIT_02s0025g01230  -  Amino acid permease
## VIT_18s0001g13770  -  Cytochrome P450, family 83, subfamily B, polypeptide 1
## VIT_00s1188g00010  -  Unknown
## VIT_19s0014g02930  -  Unknown protein
```

```
## VIT_12s0057g00310  -  Gamete expressed1 (GEX1)
## VIT_00s0332g00150  -  No hit
## VIT_17s0000g09890  -  Tetratricopeptide repeat (TPR)-containing
## VIT_16s0022g01760  -  FK506-binding protein genes family (VvFKBP43-1)
## VIT_14s0006g01880  -  ATP-dependent Clp protease proteolytic subunit (ClpP3)
## VIT_17s0000g06870  -  Oxidoreductase, acting on NADH or NADPH
## VIT_06s0061g00690  -  ATP dependent DNA ligase
## VIT_09s0002g03950  -  Unknown protein
## VIT_06s0004g04250  -  Histone H2B
## VIT_02s0154g00390  -  Autophagy 8f (APG8f)
## VIT_00s0662g00020  -  Unknown protein
## VIT_14s0108g00800  -  No hit
## VIT_07s0031g01900  -  Unknown protein
## VIT_19s0014g01800  -  Homogentisate 1,2-dioxygenase
## VIT_13s0019g05360  -  KH domain-containing protein NOVA
## VIT_18s0001g00100  -  Auxin-independent growth promoter
## VIT_08s0217g00090  -  DnaJ homolog, subfamily B, member 9
## VIT_08s0007g08350  -  Cyclin-related
## VIT_05s0020g01940  -  No hit
## VIT_02s0033g01170  -  Replication protein RPA 70kDa subunit
## VIT_09s0002g06030  -  Periodic tryptophan protein 2
## VIT_04s0023g02510  -  Glycosyl transferase family 1 protein
## VIT_16s0039g02410  -  TPL/WSIP1 (WUS- interacting  protein 1)
## VIT_02s0025g03150  -  Nuclear pore complex protein Nup98-Nup96
## VIT_06s0061g00830  -  PHD finger transcription factor
## VIT_12s0059g02530  -  fringe-related protein
## VIT_00s0144g00210  -  CCR4-NOT transcription complex, subunit 1
## VIT_13s0019g01690  -  Ubiquitin-specific protease 24 (UBP24)
## VIT_08s0040g02450  -  Unknown protein
## VIT_12s0121g00280  -  Unknown protein
## VIT_10s0003g04600  -  Cleavage and polyadenylation specificity factor 30 kDa subunit
## VIT_00s0483g00020  -  SGT1
## VIT_01s0137g00430  -  Cellulase
## VIT_13s0019g04860  -  Unknown protein
## VIT_11s0078g00440  -  U4/U6.U5 tri-snRNP-associated protein 1 SART-1
## VIT_18s0072g00220  -  Histone-lysine N-methyltransferase ASHH1
## VIT_11s0016g00250  -  CPL4 (C-Terminal Domain Phosphatase-like 4)
## VIT_01s0011g02820  -  Unknown protein
## VIT_18s0166g00320  -  ABC Transporter (VvGCN1 - VvABCF1)
## VIT_10s0116g00480  -  WNK kinase 6
## VIT_12s0178g00130  -  Unknown protein
## VIT_05s0020g04580  -  Pentatricopeptide (PPR) repeat-containing
## VIT_06s0004g00930  -  Splicing factor I
## VIT_04s0044g00070  -  RAB GDP dissociation inhibitor 1 ATGD1
```

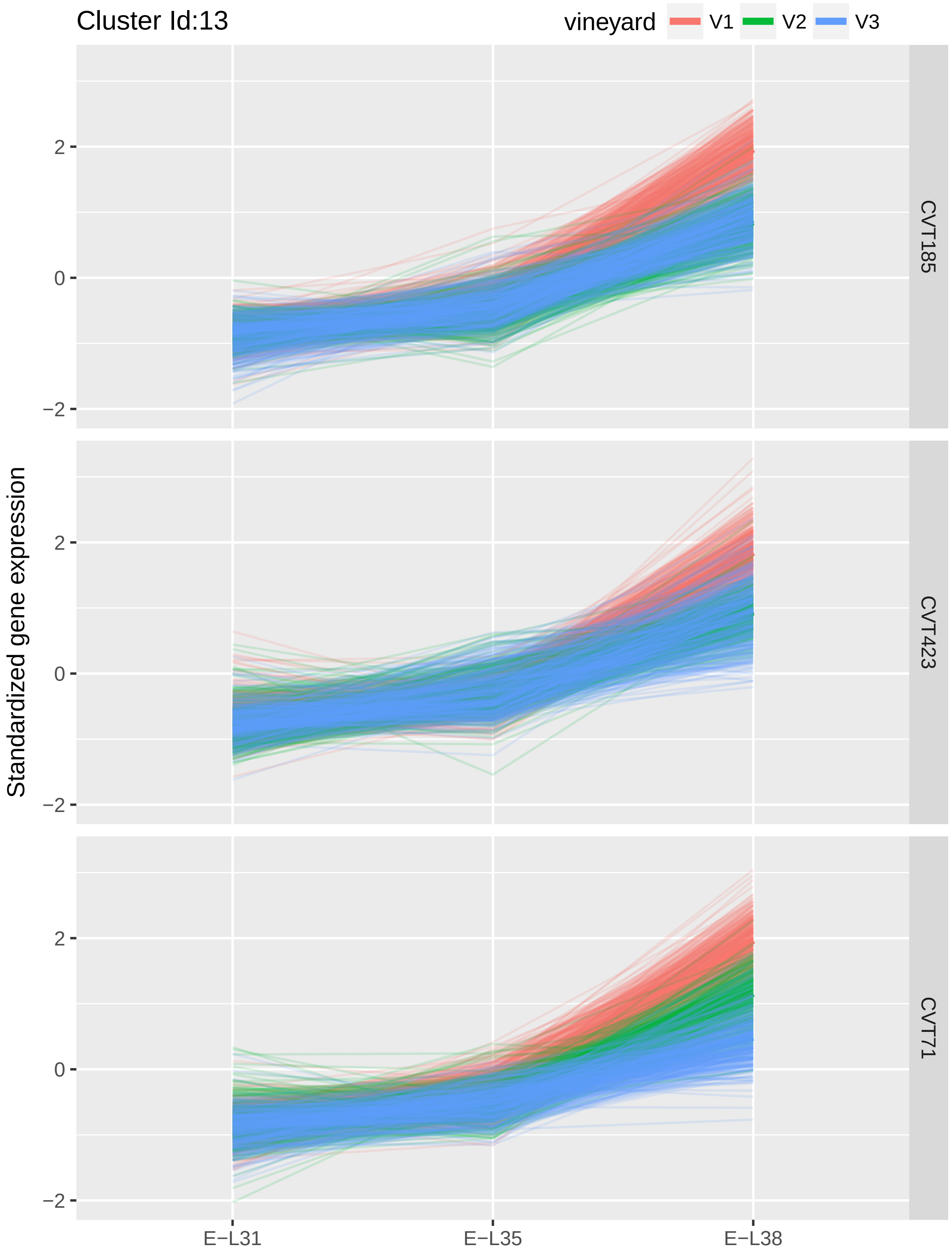

Cluster Id:13
vineyard
V1
V2
V3
Standardized gene expression
CVT185
CVT423
CVT71
2
0
−2
E−L31
E−L35
E−L38

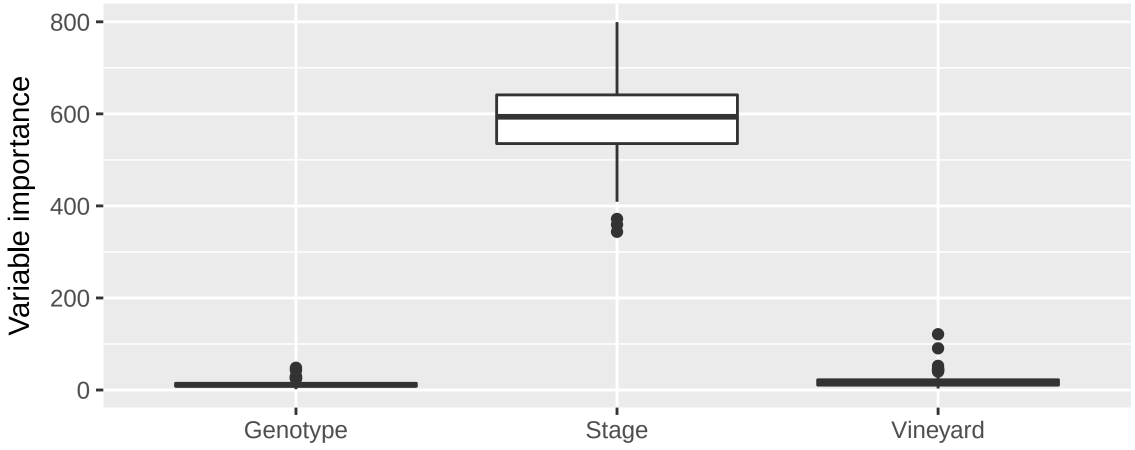

Variable importance
800
600
400
200
0
Genotype
Stage
Vineyard

Cluster no. 14

```
## Number of genes in the cluster: 121
## Homogeneity Index:    0.72
## Variable importance for Stage:    Median =  491.2  - Rank =  35
## Variable importance for Clone:    Median =  12.05  - Rank =  93
## Variable importance for Vineyard: Median =  33.46  - Rank =  61
##
## Gene ID               Gene Annotation
## VIT_18s0001g04980  -  Acetyl-CoA carboxylase 2 (ACC2)
## VIT_10s0003g04540  -  Cationic amino acid transporter 1
## VIT_17s0000g01070  -  Monoglyceride lipase
## VIT_15s0046g02170  -  3-methyl-2-oxobutanoate hydroxy-methyl-transferase
## VIT_04s0023g00430  -  Tyrosine specific protein phosphatase
## VIT_18s0001g10810  -  Carboxylesterase CXE
## VIT_04s0008g01470  -  WRKY Transcription Factor (VvWRKY06)
## VIT_10s0003g02120  -  Lipase GDSL
## VIT_00s1466g00010  -  No hit
## VIT_09s0002g01090  -  flavonoid 3-monooxygenase
## VIT_06s0004g04580  -  Protein kinase family protein
## VIT_14s0036g00460  -  Myb domain protein 102
## VIT_17s0000g09470  -  Nitrate transporter3.1
## VIT_02s0012g00730  -  Purine permease 10 PUP10
## VIT_17s0000g04370  -  Wall-associated kinase
## VIT_18s0001g12750  -  Carbohydrate kinase
## VIT_01s0011g03440  -  DNA mismatch repair protein
## VIT_18s0001g03910  -  Nitrate reductase 2 (NR2)
## VIT_05s0077g01850  -  Glycine-rich protein
## VIT_07s0005g00820  -  ERF/AP2 Gene Family (VvERF057)
## VIT_14s0060g02410  -  Acyl-CoA binding
## VIT_12s0057g00180  -  Wound-induced
## VIT_14s0030g02170  -  Calcium-binding EF hand
## VIT_04s0044g01130  -  Alcohol dehydrogenase 2 [Vitis vinifera]
## VIT_00s0203g00020  -  No hit
## VIT_04s0043g00310  -  Ribulose-phosphate 3-epimerase
## VIT_15s0046g01830  -  Pentatricopeptide (PPR) repeat-containing protein
## VIT_01s0011g03330  -  Unknown protein
## VIT_12s0142g00760  -  Leucine-rich repeat protein kinase
## VIT_01s0026g00570  -  Bet v I allergen
## VIT_17s0000g03720  -  Lateral organ boundaries protein 41
## VIT_04s0069g00920  -  WRKY Transcription Factor (VvWRKY10)
## VIT_12s0142g00780  -  Leucine-rich repeat transmembrane protein kinase
## VIT_04s0008g02860  -  Cation transport protein chaC
## VIT_00s0540g00020  -  Chitinase, class V
## VIT_18s0001g02420  -  Aspartic Protease (VvAP40)
## VIT_12s0057g00060  -  No hit
## VIT_03s0063g02640  -  Beta-ketoacyl-CoA synthase
## VIT_05s0049g00660  -  No hit
## VIT_00s2300g00010  -  Acyltransferase
## VIT_18s0001g08810  -  Unknown protein
## VIT_18s0001g02720  -  fatty acid elongation1 (FAE1)
## VIT_12s0055g01160  -  Brassinosteroid insensitive 1-associated receptor kinase 1
## VIT_07s0031g02390  -  Phytoalexin-deficient 4 protein (PAD4)
## VIT_08s0007g03230  -  Unknown protein
## VIT_01s0010g02740  -  4-coumarate-CoA ligase
## VIT_12s0034g02530  -  R protein disease resistance protein
```

```
## VIT_08s0105g00420  -  Unknown
## VIT_18s0001g06250  -  3-deoxy-D-arabino-heptulosonate 7-phosphate synthase
## VIT_04s0008g02520  -  ATP binding protein
## VIT_19s0015g00130  -  Serine acetyltransferase 3
## VIT_00s0218g00150  -  UDP-rhamnose/rhamnosyltransferase
## VIT_16s0039g02040  -  4-coumarate-CoA ligase 3
## VIT_18s0001g03300  -  1-aminocyclopropane-1-carboxylate synthase
## VIT_16s0013g01920  -  Ser/Thr protein kinase
## VIT_05s0020g04420  -  Calmodulin
## VIT_00s0454g00010  -  Subtilase
## VIT_07s0005g06420  -  Thioredoxin family
## VIT_00s0181g00010  -  Sugar transporter 1 (VvHT8)
## VIT_07s0005g02230  -  S-adenosylmethionine synthetase 1 (SAM1)
## VIT_00s0283g00020  -  Tudor domain protein 4SNc
## VIT_06s0009g03390  -  No hit
## VIT_03s0038g00490  -  GATA transcription factor 12
## VIT_18s0001g00130  -  Unknown protein
## VIT_12s0028g03810  -  DTW domain-containing protein
## VIT_05s0062g00740  -  UDP-glucoronosyl/UDP-glucosyl transferase UGT75C1
## VIT_00s0153g00070  -  Glutathione S-transferase 8 GSTU8
## VIT_05s0020g04460  -  ADP-ribosylation factor C1
## VIT_16s0039g01210  -  Ser/Thr receptor-like kinase1
## VIT_00s2563g00010  -  Glycogenin glucosyltransferase
## VIT_14s0006g02970  -  CYP735A1
## VIT_18s0041g00710  -  UDP-glucose: anthocyanidin 5,3-O-glucosyltransferase
## VIT_08s0040g01580  -  Protein kinase APK1A, chloroplast precursor
## VIT_18s0001g06680  -  far-red impaired responsive family protein
## VIT_03s0017g02170  -  Zinc transporter ZIP5
## VIT_16s0039g00320  -  Alcohol dehydrogenase 1
## VIT_03s0017g02330  -  Coproporphyrinogen III oxidase, chloroplast precursor
## VIT_18s0001g03810  -  Protein kinase
## VIT_04s0008g05500  -  S-receptor kinase
## VIT_03s0038g00230  -  Purple acid phosphatase 2 PAP2
## VIT_03s0088g01290  -  Inorganic pyrophosphatase
## VIT_05s0049g01090  -  Glutathione S-transferase 25 GSTU7
## VIT_15s0046g01190  -  Hydroquinone glucosyltransferase
## VIT_02s0025g03660  -  4-coumarate-CoA ligase
## VIT_08s0007g00510  -  Scarecrow-like transcription factor 9 (SCL9)
## VIT_09s0002g02460  -  Phosphatase
## VIT_13s0019g02040  -  Lectin protein kinase
## VIT_02s0025g04380  -  ATPP2-A13
## VIT_05s0020g02720  -  Aspartic Protease (VvAP11)
## VIT_13s0067g01270  -  Avr9/Cf-9 rapidly elicited protein 284
## VIT_06s0004g06080  -  Proton-dependent oligopeptide transport (POT) family protein
## VIT_09s0018g00240  -  WRKY Transcription Factor (VvWRKY28)
## VIT_01s0011g03820  -  EMB2454 (embryo defective 2454)
## VIT_07s0104g00200  -  Phenazine biosynthesis PhzC/PhzF
## VIT_08s0040g01570  -  Protein kinase APK1A, chloroplast precursor
## VIT_07s0031g00760  -  EF hand
## VIT_06s0004g02830  -  Unknown protein
## VIT_18s0001g14460  -  Unknown protein
## VIT_05s0020g04610  -  SYM10 protein
## VIT_00s0231g00040  -  RPM1-interacting protein 4
## VIT_16s0050g01310  -  C2 domain-containing protein
## VIT_02s0033g00020  -  Unknown protein
```

```
## VIT_19s0014g02630  -  No hit
## VIT_17s0000g07400  -  Disease resistance protein (EDS1)
## VIT_17s0000g01010  -  Lysophospholipase homolog
## VIT_19s0014g02640  -  PPI1 (proton pump interactor 1)
## VIT_18s0001g06550  -  far-red impaired responsive family protein
## VIT_07s0031g00880  -  Nodulation protein
## VIT_16s0050g02740  -  Receptor-like protein kinase
## VIT_06s0004g06710  -  Cys-3-His zinc finger protein
## VIT_05s0049g00610  -  No hit
## VIT_17s0000g07990  -  IS10 transposase
## VIT_09s0002g09280  -  Cysteine peptidase
## VIT_16s0050g01250  -  Heavy-metal-associated domain-containing protein
## VIT_06s0080g00450  -  Calcium-binding protein CML
## VIT_07s0005g02100  -  Shaggy protein kinase alpha (ASK-alpha)
## VIT_17s0000g07560  -  EDS1 (Enhanced disease susceptibility 1)
## VIT_03s0038g00250  -  Purple acid phosphatase 10 ATPAP10/PAP10
## VIT_02s0033g00010  -  Unknown protein
## VIT_05s0049g01120  -  Glutathione S-transferase 25 GSTU7
## VIT_00s0304g00080  -  Trehalose-6-phosphate phosphatase
```

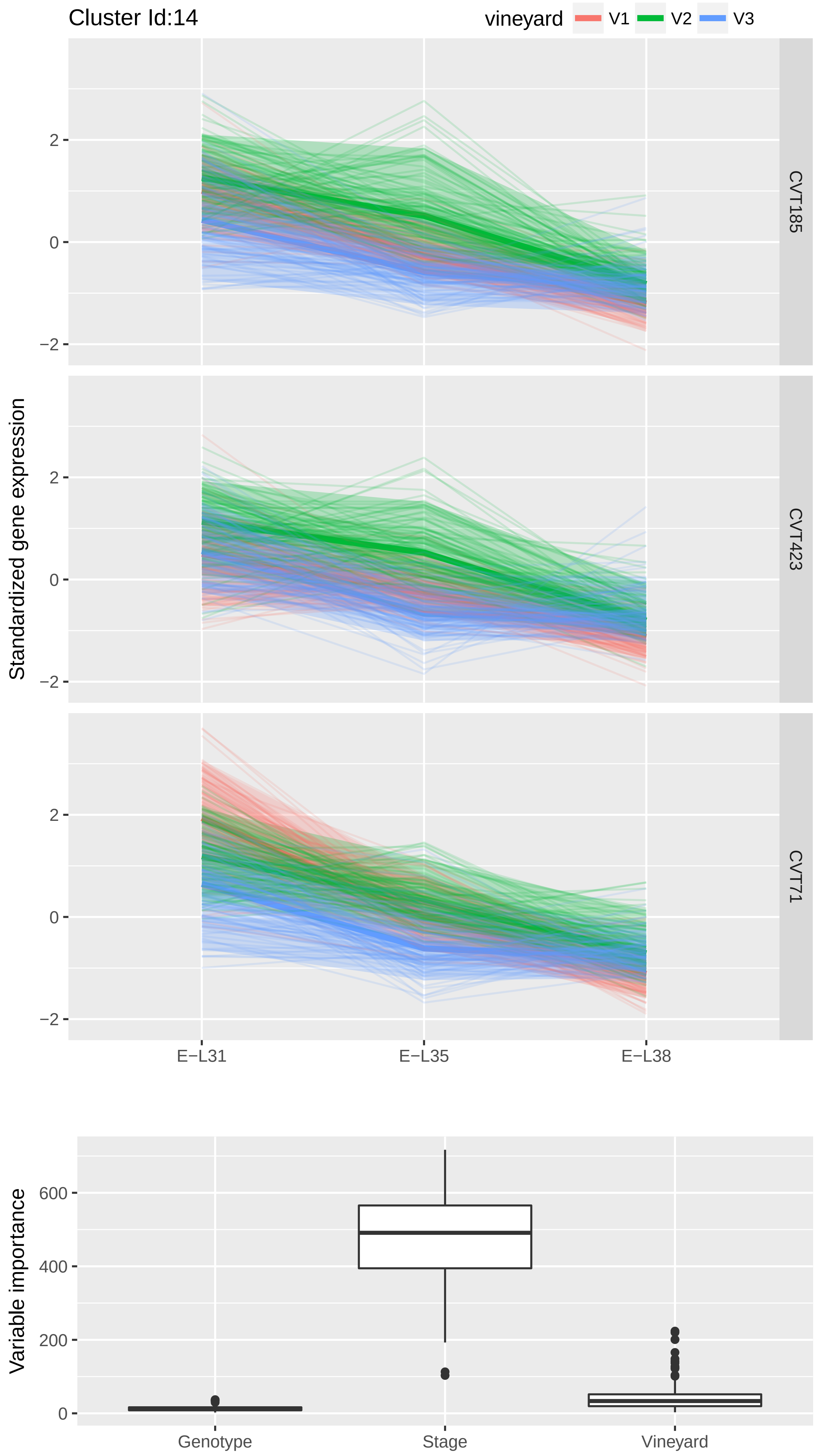

Cluster Id:14
vineyard V1 V2 V3
Standardized gene expression
CVT185
CVT423
CVT71
2
0
−2
E−L31
E−L35
E−L38
Variable importance
600
400
200
0
Genotype
Stage
Vineyard

Cluster no. 15

```
## Number of genes in the cluster: 61
## Homogeneity Index:    0.64
## Variable importance for Stage:    Median =  177.2  - Rank =  93
## Variable importance for Clone:    Median =  20.22  - Rank =  46
## Variable importance for Vineyard: Median =  140.8  - Rank =  8
##
## Gene ID                Gene Annotation
## VIT_13s0019g02650  -  U4/U6 small nuclear ribonucleoprotein SNU13
## VIT_19s0014g00930  -  Unknown protein
## VIT_19s0090g01020  -  Molybdopterin biosynthesis protein CNX5
## VIT_17s0000g08170  -  Chaperonin
## VIT_16s0098g00900  -  Pseudo-response regulator 5 (APRR5)
## VIT_12s0035g01020  -  Ribosomal protein L3B
## VIT_19s0085g00370  -  Phosphoglycerate kinase, cytosolic
## VIT_08s0007g07460  -  Chaperonin
## VIT_14s0068g00240  -  Rough sheath2-interacting KH-domain protein
## VIT_00s0220g00130  -  Allyl alcohol dehydrogenase
## VIT_06s0009g00850  -  Strictosidine synthase
## VIT_19s0014g03420  -  Late embryogenesis abundant protein
## VIT_01s0127g00060  -  Alpha-glucan water dikinase, chloroplast precursor
## VIT_07s0197g00070  -  SWIB complex BAF60b domain-containing protein
## VIT_10s0042g01220  -  Translation initiation factor eIF-2 gamma subunit
## VIT_14s0066g02620  -  RNA 3'-terminal phosphate cyclase-like protein
## VIT_00s0252g00130  -  Calcium-binding protein CML
## VIT_02s0025g01480  -  Translation initiation factor eIF-3 subunit 10
## VIT_02s0012g00050  -  Glycine-rich protein
## VIT_09s0002g04340  -  Glabra2
## VIT_14s0083g00530  -  Cell division protein FtsH
## VIT_17s0000g00160  -  Glycyl-tRNA synthetase 1
## VIT_01s0011g05300  -  Armadillo/beta-catenin repeat
## VIT_02s0012g01010  -  Leucine-rich repeat
## VIT_03s0063g02520  -  PRLI-interacting factor G
## VIT_19s0085g01200  -  CTP synthase
## VIT_01s0150g00190  -  Transaldolase
## VIT_03s0038g04560  -  Ribosomal protein S25 (RPS25E) 40S
## VIT_15s0021g02230  -  T-complex protein 1 alpha subunit
## VIT_17s0000g08410  -  Peroxisomal protein (PEX14)
## VIT_19s0090g01590  -  Import inner membrane translocase subunit Tim17/Tim22/Tim23
## VIT_02s0025g00880  -  BTB/POZ domain-containing protein POB1
## VIT_02s0012g00020  -  Glycine-rich protein
## VIT_07s0005g01560  -  PAPA-1
## VIT_18s0001g12350  -  GYF domain-containing protein
## VIT_08s0007g07590  -  Unknown protein
## VIT_08s0040g00100  -  myb family
## VIT_06s0061g01600  -  Ribosomal protein P2 60s acidic
## VIT_10s0042g00960  -  DNAJ heat shock N-terminal domain-containing protein
## VIT_16s0098g00610  -  Import inner membrane translocase subunit Tim17/Tim22/Tim23
## VIT_07s0005g01570  -  No hit
## VIT_06s0004g07730  -  Inner envelope protein, 110 kD (IEP110) precursor, Chloroplast
## VIT_19s0014g02990  -  Nuclear GTP-binding protein NUG2
## VIT_16s0098g01010  -  Ribosomal protein P1 (RPP1B) acidic 60S
## VIT_07s0141g00710  -  Ribosomal protein S15 (RPS15D) 40S
## VIT_06s0004g00420  -  Calpain-type cysteine protease
## VIT_06s0004g06220  -  Na+/Pi transporter
```

```
## VIT_12s0134g00060  -  Ribosomal protein L2
## VIT_06s0004g01340  -  Esterase/lipase/thioesterase
## VIT_16s0039g00200  -  Ubiquinol-cytochrome c reductase subunit 8
## VIT_11s0103g00250  -  Carboxyl methylase
## VIT_06s0004g08260  -  Ribosomal protein L10 (RPL10B) 60S
## VIT_01s0011g01820  -  Ribosomal protein L27 (RPL27C) 60S
## VIT_00s0160g00150  -  No hit
## VIT_08s0007g08370  -  Ribosomal protein S2 (RPS2D) 40S
## VIT_11s0149g00220  -  Ribosomal protein L39 (RPL39C) 60S
## VIT_18s0001g07080  -  Protein transport protein SFT1
## VIT_12s0035g00960  -  TRNA pseudouridine synthase
## VIT_04s0008g05000  -  Ubiquinol-cytochrome c reductase subunit 7
## VIT_14s0060g02310  -  Ribosomal protein L22-2 (RPL22B) 60S
## VIT_08s0056g00010  -  Seryl-tRNA synthetase
```

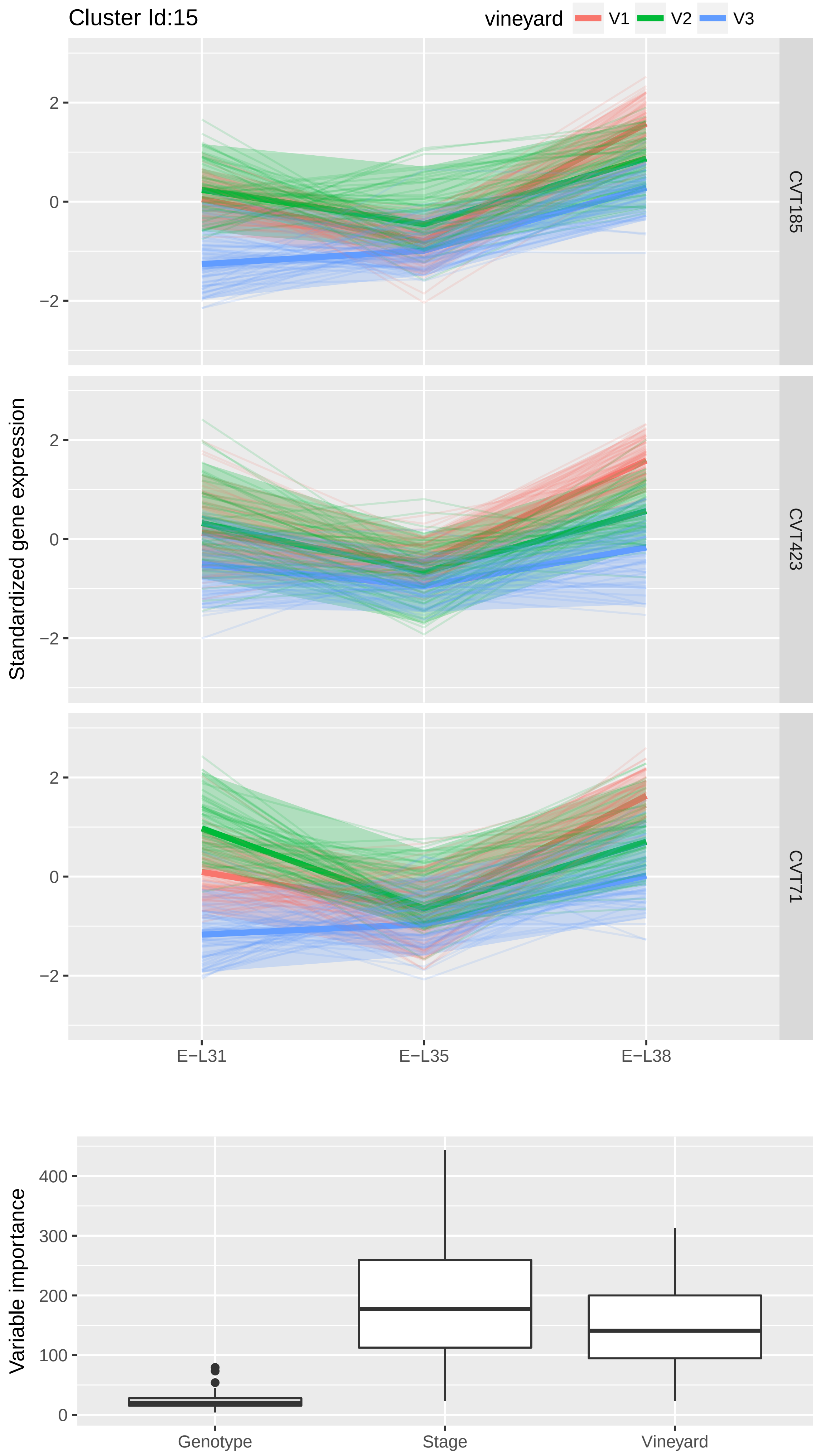

Cluster Id:15
vineyard
V1
V2
V3
Standardized gene expression
CVT185
CVT423
CVT71
2
0
−2
E−L31
E−L35
E−L38
Variable importance
400
300
200
100
0
Genotype
Stage
Vineyard

Cluster no. 16

```
## Number of genes in the cluster: 217
## Homogeneity Index:     0.8
## Variable importance for Stage:    Median =  443.1  - Rank =  50
## Variable importance for Clone:    Median =  13.28  - Rank =  81
## Variable importance for Vineyard: Median =  31.34  - Rank =  64
##
## Gene ID                Gene Annotation
## VIT_07s0205g00030  -  Zinc finger (C3HC4-type ring finger)
## VIT_06s0004g02290  -  Unknown
## VIT_01s0026g00710  -  Cyclin delta-2
## VIT_19s0014g00570  -  Disease resistance protein RFL1
## VIT_17s0000g08690  -  WD-40 repeat
## VIT_00s0227g00190  -  Phosphoglucomutase/phosphomannomutase C terminal
## VIT_07s0005g00890  -  Polygalacturonase GH28
## VIT_14s0030g01540  -  Cysteine synthase
## VIT_04s0008g03840  -  Ankyrin repeat
## VIT_10s0042g01240  -  Transducin protein
## VIT_04s0008g03220  -  Lysophospholipase
## VIT_07s0191g00180  -  Homeobox-leucine zipper protein ATHB-6
## VIT_17s0000g02230  -  TIFY gene family (VvJAZ11)
## VIT_08s0032g01040  -  SYNC1 protein, related
## VIT_11s0016g05090  -  DEAD box RNA helicase
## VIT_09s0002g03410  -  Phytochrome-associated protein 1 (PAP1)
## VIT_03s0038g00830  -  ARGONAUTE7 AGO7
## VIT_18s0001g00690  -  Glutathione S-transferase 8 GSTF8
## VIT_12s0057g01350  -  Constans-like 9
## VIT_09s0002g02080  -  CYP90D2
## VIT_01s0011g02930  -  DNA-binding protein, chloroplast nucleoid
## VIT_00s0160g00160  -  No hit
## VIT_07s0031g02060  -  Cellulose synthase CESA2
## VIT_12s0028g03110  -  Caffeoyl-CoA 3-O-methyltransferase
## VIT_03s0038g02250  -  Pentatricopeptide (PPR) repeat-containing protein
## VIT_12s0034g01330  -  Unknown protein
## VIT_10s0003g03910  -  TCP family transcription factor 24
## VIT_01s0011g05740  -  UDP-3-O-acyl N-acetylglycosamine deacetylase
## VIT_14s0030g01300  -  Unknown
## VIT_19s0015g00340  -  Unknown protein
## VIT_01s0010g03870  -  Unknown protein
## VIT_19s0090g00700  -  No hit
## VIT_11s0016g02640  -  Peptidase M16 inactive domain containing protein
## VIT_10s0003g04990  -  feronia receptor-like kinase
## VIT_08s0007g07110  -  Unknown protein
## VIT_06s0004g04960  -  Scarecrow-like transcription factor 14 SCL14
## VIT_03s0038g03970  -  Unknown protein
## VIT_04s0023g01250  -  Brassinosteroid Signaling positive regulator (BZR1)
## VIT_07s0129g00210  -  BT4 (BTB and TAZ Domain protein 4)
## VIT_05s0062g00840  -  RAB GTPase ARA4
## VIT_04s0008g01700  -  Unknown protein
## VIT_02s0025g01170  -  COP1-interactive protein 1 / CIP1
## VIT_03s0038g04570  -  ADP-glucose pyrophosphorylase large subunit 1
## VIT_07s0005g00060  -  No hit
## VIT_07s0151g00470  -  Leucine-rich repeat family protein
## VIT_12s0134g00650  -  Anthocyanin 5-aromatic acyltransferase
## VIT_00s0233g00050  -  Exostosin (Xyloglucan galactosyltransferase KATAMARI 1)
```

```
## VIT_00s0291g00070  -  Unknown protein
## VIT_04s0023g01550  -  Unknown
## VIT_13s0019g05400  -  RPG related protein 1 RR1
## VIT_08s0007g07340  -  Unknown protein
## VIT_14s0006g02170  -  Jasmonate O-methyltransferase
## VIT_11s0016g05830  -  No hit
## VIT_07s0141g00270  -  Auxin-induced protein 22D
## VIT_11s0052g00040  -  Macrophage migration inhibitory factor
## VIT_16s0022g01870  -  Unknown protein
## VIT_09s0002g01030  -  Subtilisin serine proteinase
## VIT_08s0040g01470  -  Cis-zeatin O-beta-D-glucosyltransferase
## VIT_01s0011g03360  -  Unknown
## VIT_06s0061g00890  -  Carboxypeptidase
## VIT_00s0389g00030  -  CYP72A54
## VIT_18s0089g00040  -  NBS-LRR disease resistance protein
## VIT_17s0000g01970  -  MKK9
## VIT_05s0077g02270  -  Unknown protein
## VIT_08s0007g05550  -  Protein kinase
## VIT_14s0066g00550  -  ABC Transporter (VvTAP4 - VvABCB24)
## VIT_14s0083g01110  -  Brassinosteroid-6-oxidase
## VIT_10s0116g00590  -  Pectinesterase family
## VIT_00s0477g00010  -  No hit
## VIT_04s0023g01920  -  Bg55
## VIT_18s0001g11830  -  Calmodulin
## VIT_02s0012g02540  -  Chlororespiratory reduction 4 (CRR4)
## VIT_12s0057g01070  -  S-adenosyl-L-methionine:salicylic acid carboxyl methyltransferase
## VIT_02s0025g02020  -  Abl interactor 3 (ABIL3)
## VIT_13s0067g00220  -  Aquaporin TMP-C
## VIT_14s0060g01190  -  Nicotianamine synthase
## VIT_10s0003g00230  -  Unknown protein
## VIT_08s0007g00270  -  No hit
## VIT_11s0016g05840  -  Protease inhibitor/seed storage/lipid transfer protein (LTP)
## VIT_17s0000g02440  -  Integral membrane protein
## VIT_15s0024g00050  -  Deoxyribonuclease tatD
## VIT_14s0030g00870  -  CC-NBS-LRR class
## VIT_18s0001g00770  -  Phospholipase D p98
## VIT_14s0006g00920  -  Unknown protein
## VIT_14s0083g00640  -  Constans 2 (COL2)
## VIT_16s0098g01900  -  Pectinesterase family
## VIT_01s0011g03370  -  Agenet domain-containing protein
## VIT_10s0116g00710  -  Tryptophan synthase, beta subunit
## VIT_18s0122g00280  -  Unknown protein
## VIT_08s0007g06700  -  Thioredoxin 5
## VIT_02s0025g02820  -  Membrane-related CP5
## VIT_06s0004g06290  -  Binding
## VIT_18s0001g02220  -  Beta-galactosidase / lactase
## VIT_14s0006g02160  -  SAM dependent carboxyl methyltransferase Methyltransf_7
## VIT_01s0010g01660  -  Receptor protein kinase
## VIT_00s0259g00100  -  Proline-rich family protein
## VIT_01s0011g03050  -  Unknown protein
## VIT_14s0108g00560  -  Ankyrin repeat
## VIT_00s0204g00130  -  Unknown
## VIT_18s0001g13200  -  Cytokinin dehydrogenase 5 precursor
## VIT_18s0122g00290  -  Unknown protein
## VIT_00s0301g00080  -  No hit
```

```
## VIT_02s0033g01000  -  Anthraniloyal-CoA: methanol anthraniloyal transferase
## VIT_18s0041g02080  -  12-oxophytodienoate reductase 2
## VIT_02s0025g00180  -  Bisphosphoglycerate mutase
## VIT_10s0003g03270  -  Inward rectifying potassium channel
## VIT_05s0020g03010  -  Carboxylesterase CXE
## VIT_11s0016g04250  -  HCF101 (high-chlorophyll-fluorescence 101)
## VIT_13s0067g02280  -  basic helix-loop-helix (bHLH) family
## VIT_09s0002g00940  -  Unknown protein
## VIT_15s0024g00060  -  No hit
## VIT_05s0077g02260  -  Nodulin MtN3 family
## VIT_13s0019g00740  -  Amino acid binding protein
## VIT_01s0026g00350  -  basic helix-loop-helix (bHLH) family
## VIT_14s0066g00160  -  Ubiquinone/menaquinone biosynthesis methyltransferase UbiE
## VIT_18s0001g15000  -  ACT domain containing protein (ACR4)
## VIT_11s0016g02900  -  Leucine-rich repeat family protein / extensin
## VIT_13s0106g00550  -  flavonol 3-sulfotransferase
## VIT_00s2594g00010  -  Receptor protein kinase
## VIT_07s0104g00840  -  RKF3 (receptor-like kinase IN in flowers 3)
## VIT_04s0008g01050  -  Unknown
## VIT_05s0077g02010  -  Epoxide hydrolase
## VIT_12s0028g02180  -  Phosphoenolpyruvate carboxylase.
## VIT_08s0056g01290  -  Receptor-like kinase 17
## VIT_18s0001g14030  -  Lysine decarboxylase
## VIT_05s0020g04240  -  Calcium-transporting ATPase 12 ACA12
## VIT_08s0007g02030  -  AUX1 protein
## VIT_18s0001g02000  -  Zinc finger (C2H2 type) family
## VIT_08s0007g05240  -  Phosphatidylinositol-4-phosphate 5-kinase
## VIT_13s0106g00040  -  Ankyrin
## VIT_06s0004g02390  -  Triacylglycerol lipase
## VIT_12s0059g01250  -  Endo-1,4-beta-glucanase
## VIT_07s0005g04380  -  IAA12
## VIT_01s0011g04060  -  Receptor-like kinase RHG1
## VIT_03s0038g01150  -  Auxin-responsive
## VIT_02s0025g02080  -  Nodulin MtN3 family protein
## VIT_05s0020g01100  -  Myb domain protein 61
## VIT_13s0067g02190  -  SAG20 (wound-induced protein 12)
## VIT_19s0085g01130  -  Unknown protein
## VIT_08s0007g05260  -  Glutamate synthase (GLU1), ferredoxin-dependent
## VIT_01s0026g02340  -  Ankyrin protein kinase
## VIT_19s0090g00480  -  ATP synthase gamma chain 1t (ATPC1)
## VIT_13s0074g00420  -  Unknown
## VIT_07s0129g00200  -  Cyclin-D5-1
## VIT_18s0001g10690  -  BKI1 (BRI1 kinase inhibitor 1)
## VIT_16s0050g00170  -  HcrVf3 protein
## VIT_17s0000g05020  -  Squamosa promoter-binding protein 6 (SPL6)
## VIT_01s0011g03550  -  Unknown
## VIT_03s0038g02400  -  Protein kinase
## VIT_05s0020g04220  -  Calcium-transporting ATPase 13 ACA13
## VIT_12s0142g00450  -  Unknown protein
## VIT_11s0037g00960  -  NIK1 (NSP- interacting  kinase 1)
## VIT_02s0025g03450  -  basic helix-loop-helix (VvJAF13)
## VIT_18s0001g15520  -  Leaf senescence protein
## VIT_17s0000g05070  -  Phytochelatin synthetase
## VIT_05s0020g00960  -  No hit
## VIT_00s0291g00080  -  Unknown protein
```

```
## VIT_00s0800g00010  -  Alpha-1,4-glycosyltransferase
## VIT_09s0002g04260  -  Unknown protein
## VIT_10s0003g00040  -  Dof zinc finger protein DOF1.4
## VIT_18s0001g02010  -  No hit
## VIT_00s0275g00060  -  Disease resistance protein (TIR-NBS-LRR class)
## VIT_02s0025g00210  -  fringe protein
## VIT_07s0129g00330  -  Lateral organ boundaries protein 39
## VIT_13s0019g03040  -  Indole-3-acetate beta-glucosyltransferase
## VIT_15s0046g01880  -  Brassinosteroid-responsive ring-H2 (BRH1)
## VIT_00s0420g00040  -  S-locus lectin protein kinase
## VIT_08s0040g02200  -  Peroxidase ATP2a
## VIT_03s0038g04480  -  SEC14 cytosolic factor
## VIT_07s0005g04860  -  Glutaredoxin protein
## VIT_01s0026g01420  -  Wall-associated kinase 4
## VIT_09s0002g03520  -  Arabidopsis histidine phosphotransfer AHP4
## VIT_02s0025g04590  -  TCP family transcription factor TCP9
## VIT_07s0031g00500  -  Subtilase
## VIT_08s0058g00930  -  Alanine--glyoxylate aminotransferase 2 3, mitochondrial
## VIT_04s0023g02230  -  S-adenosyl-L-methionine:salicylic acid carboxyl methyltransferase
## VIT_09s0002g02120  -  Beta-galactosidase
## VIT_14s0030g01360  -  Kinesin motor protein
## VIT_04s0023g02630  -  FAD linked oxidase family protein
## VIT_07s0005g03230  -  ERF/AP2 Gene Family (VvERF099)
## VIT_06s0004g04140  -  myb domain protein 59
## VIT_06s0080g00780  -  NAC domain-containing protein (VvNAC74)
## VIT_11s0016g00870  -  Strictosidine synthase (YLS2)
## VIT_07s0031g00980  -  Aspartate aminotransferase
## VIT_17s0000g05860  -  ABC Transporter (VvATH1 - VvABCA2)
## VIT_05s0020g01110  -  Pectinacetylesterase
## VIT_05s0062g00700  -  UDP-glucose:flavonoid 7-O-glucosyltransferase
## VIT_13s0067g02340  -  Proton-dependent oligopeptide transport (POT) family protein
## VIT_16s0098g01870  -  SOS3 (salt overly sensitive 3)
## VIT_09s0002g04080  -  IAA9
## VIT_13s0064g00890  -  Cellulose synthase CESA3
## VIT_12s0035g00690  -  TCP family transcription factor TCP23
## VIT_02s0033g01020  -  Anthraniloyal-CoA: methanol anthraniloyal transferase
## VIT_00s0259g00070  -  Proline-rich family protein
## VIT_01s0011g05060  -  Major latex-like protein 34
## VIT_00s0587g00030  -  CBS domain-containing protein
## VIT_05s0020g03880  -  TSO1 (chinese for 'ugly')
## VIT_09s0002g08680  -  Unknown
## VIT_11s0016g00190  -  Transcription factor
## VIT_10s0042g00630  -  SET domain-containing protein
## VIT_05s0094g01530  -  ARL (ARGOS)
## VIT_14s0108g01510  -  Membrane-anchored ubiquitin-fold protein 2
## VIT_03s0017g01660  -  Scarecrow transcription factor 23 (SCL23)
## VIT_14s0030g01170  -  CC-NBS-LRR class
## VIT_13s0106g00030  -  Ankyrin repeat
## VIT_08s0007g07920  -  Unknown protein
## VIT_17s0053g00380  -  Metal-nicotianamine transporter YSL7
## VIT_08s0007g06950  -  Disease resistance-responsive
## VIT_08s0007g00020  -  Cation/hydrogen exchanger 20 (CHX20)
## VIT_11s0016g03140  -  ERD7 (EARLY-responsive TO dehydration 7)
## VIT_07s0005g02480  -  Myb domain protein 61
## VIT_05s0094g01570  -  MLO1
```

```
## VIT_18s0117g00260  -  Co-chaperone-curved DNA binding protein A
## VIT_03s0038g02230  -  Subtilisin-like serine protease 2
## VIT_05s0094g01260  -  Allyl alcohol dehydrogenase
## VIT_15s0048g02280  -  NAC domain-containing protein (VvNAC54)
## VIT_09s0070g00210  -  Metal transporter Nramp1
```

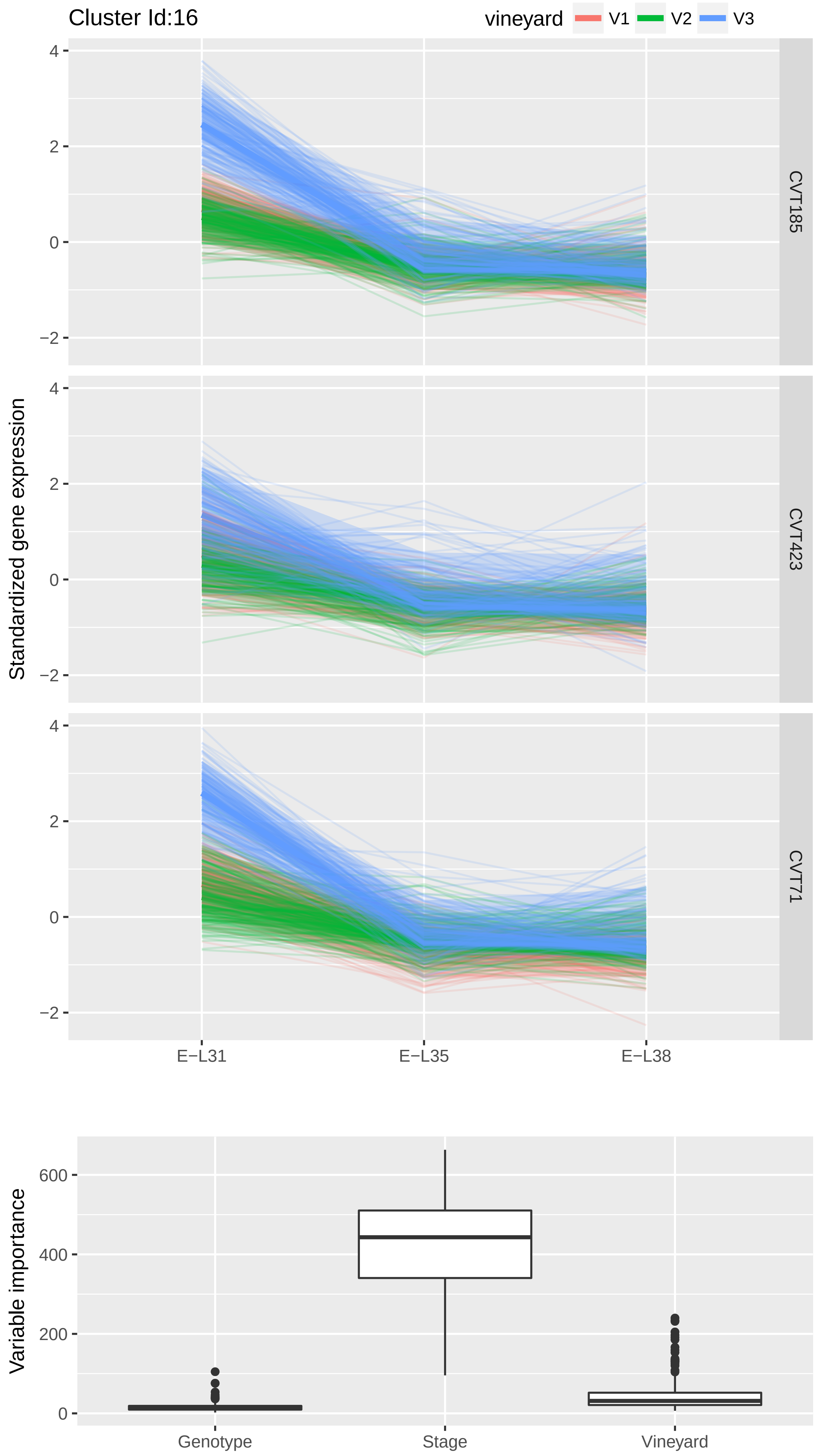

Cluster Id:16
vineyard
V1
V2
V3
CVT185
CVT423
CVT71
Standardized gene expression
4
2
0
−2
E−L31
E−L35
E−L38
Variable importance
600
400
200
0
Genotype
Stage
Vineyard

Cluster no. 17

```
## Number of genes in the cluster: 114
## Homogeneity Index:    0.58
## Variable importance for Stage:    Median =  159.4  - Rank =  97
## Variable importance for Clone:    Median =  22.24  - Rank =  36
## Variable importance for Vineyard: Median =    122  - Rank =  13
##
## Gene ID                Gene Annotation
## VIT_12s0057g00740  -  Pentatricopeptide (PPR) repeat-containing protein
## VIT_03s0038g04370  -  Inositol-1-monophosphatase
## VIT_19s0014g00870  -  Brassinosteroid Signaling positive regulator (BZR1)
## VIT_14s0219g00200  -  Pentatricopeptide (PPR) repeat-containing protein
## VIT_03s0063g00580  -  Rad54
## VIT_06s0009g00770  -  Abscisic acid 8` hydroxylase (CYP707A2) (VvA8H-CYP707A2.6)
## VIT_01s0026g00700  -  RNA recognition motif (RRM)-containing protein
## VIT_19s0014g05150  -  RPS2 (resistant to p. syringae 2)
## VIT_18s0001g13920  -  Unknown protein
## VIT_05s0077g01810  -  Unknown protein
## VIT_11s0016g00350  -  Sec34/COG3
## VIT_03s0038g02930  -  Tetracycline transporter protein
## VIT_18s0086g00210  -  S-locus receptor protein kinase
## VIT_08s0058g01090  -  CBL-interacting protein kinase 14 (CIPK14)
## VIT_13s0019g04100  -  Zinc finger (C3HC4-type ring finger)
## VIT_08s0040g01810  -  Auxin response factor 10
## VIT_04s0043g00790  -  ribulose-1,5 bisphosphate carboxylase oxygenase large subunit N-methyl
## VIT_11s0037g00830  -  Unknown protein
## VIT_01s0026g00200  -  Xyloglucan endotransglucosylase/hydrolase 28
## VIT_11s0149g00030  -  Endomembrane protein 70
## VIT_03s0038g00690  -  Zinc finger (B-box type)
## VIT_07s0005g02300  -  EDA32 (embryo sac development arrest 32)
## VIT_18s0001g03110  -  Unknown protein
## VIT_10s0003g02800  -  KCO6 (Ca2+ activated outward rectifying K+ channel 6)
## VIT_00s0558g00030  -  Pro-X carboxypeptidase Lysosomal
## VIT_13s0084g00100  -  Nodulation protein
## VIT_17s0000g01560  -  Component of oligomeric golgi complex 2
## VIT_19s0090g01150  -  SWIB complex BAF60b domain-containing protein
## VIT_18s0001g05800  -  Dehydration-responsive protein
## VIT_17s0000g05470  -  Nodulin
## VIT_19s0085g00890  -  Myb APL (altered phloem development)
## VIT_11s0052g00920  -  Unknown protein
## VIT_13s0084g00090  -  Nodulin MtN21 family
## VIT_10s0116g00730  -  Monoglyceride lipase
## VIT_03s0038g00170  -  Transducin protein
## VIT_00s0335g00010  -  R protein L6
## VIT_15s0046g00990  -  Unknown protein
## VIT_16s0050g02410  -  Ubiquitin-conjugating enzyme E2 D/E
## VIT_06s0004g08230  -  Root phototropism protein 2
## VIT_00s0160g00310  -  Disease resistance protein (TIR-NBS-LRR class
## VIT_04s0044g00590  -  No hit
## VIT_15s0048g00470  -  Tubulin folding cofactor D
## VIT_01s0010g02760  -  Urease
## VIT_06s0009g00890  -  Ubiquitin-specific protease 8
## VIT_10s0523g00030  -  Photoregulatory zinc-finger protein COP1
## VIT_05s0020g02490  -  Unknown protein
## VIT_06s0004g00190  -  3-methyl-2-oxobutanoate dehydrogenase
```

```
## VIT_00s0455g00020  -  Enhancer of mRNA-decapping protein 4
## VIT_00s0216g00020  -  Glycosyl transferase family 8 protein
## VIT_10s0003g04470  -  ABC Transporter (VvMRP20 - VvABCC20)
## VIT_12s0035g01900  -  Pectinesterase family
## VIT_06s0004g06340  -  flavodoxin-like quinone reductase 1
## VIT_14s0060g00930  -  Importin alpha-1 subunit (IMPA1)
## VIT_00s0225g00130  -  Alanine transaminase.
## VIT_18s0001g07890  -  TRAF-type zinc finger-related
## VIT_03s0038g00410  -  Enhanced disease susceptibility 5 EDS5
## VIT_08s0007g02120  -  Glycoside hydrolase starch-binding domain-containing protein
## VIT_11s0016g04670  -  No hit
## VIT_01s0150g00300  -  Indole-3-acetic acid amido synthetase
## VIT_14s0068g01230  -  fructose-2,6-bisphosphatase
## VIT_18s0001g08310  -  Inositol 1,3,4-trisphosphate 5/6-kinase
## VIT_00s0992g00010  -  Zinc finger (MYND type)
## VIT_04s0023g02690  -  Beta-galactosidase
## VIT_16s0098g01530  -  Unknown protein
## VIT_18s0001g02610  -  Caffeic acid methyltransferase
## VIT_02s0025g02970  -  Flavanone 3-hydroxylase (F3H)
## VIT_19s0027g00130  -  Translation initiation factor IF-2, chloroplast
## VIT_13s0019g05250  -  Malate dehydrogenase [NADP], chloroplast precursor (NADP-MDH)
## VIT_03s0063g00750  -  Carboxylesterase CXE
## VIT_05s0049g01180  -  Ankyrin
## VIT_18s0041g01220  -  GCN5 N-acetyltransferase (GNAT)
## VIT_07s0104g01790  -  Autophagy 18 ATG18d
## VIT_18s0001g14010  -  Sulfate adenylyltransferase
## VIT_13s0067g03410  -  Serine/threonine Protein kinase BNK1
## VIT_17s0053g00210  -  Peptidoglycan-binding LysM domain-containing protein
## VIT_19s0014g00290  -  UDP-glucose 4,6-dehydratase
## VIT_14s0066g01040  -  Cytosine methyltransferase (DRM2)
## VIT_03s0063g02050  -  E3 ubiquitin-protein ligase RNF5
## VIT_06s0061g01310  -  Nucleobase-ascorbate transporter 12 (NAT12)
## VIT_12s0028g03610  -  Cellulase
## VIT_16s0098g01770  -  Vesicle-associated membrane protein 72
## VIT_02s0025g03230  -  Fringe protein
## VIT_02s0025g02640  -  Unknown protein
## VIT_02s0025g02270  -  PRLI-interacting factor G
## VIT_18s0001g06330  -  14-3-3 protein GF14 omega (GRF2)
## VIT_04s0008g01040  -  Lysine decarboxylase
## VIT_09s0002g06320  -  R protein disease resistance protein
## VIT_08s0007g08260  -  Guanylate kinase
## VIT_14s0006g02520  -  HEAT repeat-containing protein
## VIT_04s0023g00380  -  Transmembrane protein FT27/PFT27
## VIT_01s0026g02420  -  Unknown protein
## VIT_06s0061g01120  -  UDP-D-apiose/UDP-D-xylose synthase
## VIT_18s0001g08030  -  Phosphoglycerate mutase
## VIT_18s0001g03960  -  Mekk1
## VIT_08s0040g00630  -  Hydroxyproline-rich glycoprotein
## VIT_07s0141g00770  -  RAB GTPase RABA5E
## VIT_01s0011g04450  -  MATE efflux family protein
## VIT_00s0274g00070  -  basic helix-loop-helix (bHLH) family
## VIT_05s0049g01760  -  Proton gradient regulation 5 (PGR5)
## VIT_08s0040g02180  -  Mlo3
## VIT_11s0016g05380  -  Acidic leucine-rich nuclear phosphoprotein 32 member E
## VIT_15s0046g00660  -  Wax synthase
```

```
## VIT_11s0016g01190  -  Auxin-independent growth promoter
## VIT_01s0026g00270  -  K+ uptake permease 6
## VIT_15s0048g02410  -  Myb CCA1 (circadian clock associated 1)
## VIT_07s0104g00270  -  Isopentenyltransferase 5
## VIT_17s0053g00240  -  Peptidoglycan-binding LysM domain-containing protein
## VIT_08s0007g06740  -  Seven in absentia SINA2
## VIT_07s0005g01180  -  Unknown protein
## VIT_14s0083g00690  -  Glycosyl transferase family 8 protein
## VIT_14s0066g00370  -  Protein kinase ATN1
## VIT_12s0134g00400  -  Zinc finger (B-box type)
## VIT_08s0105g00430  -  Omega-3 fatty acid desaturase, chloroplast precursor
## VIT_01s0011g00690  -  UDP-glucose 6-dehydrogenase
```

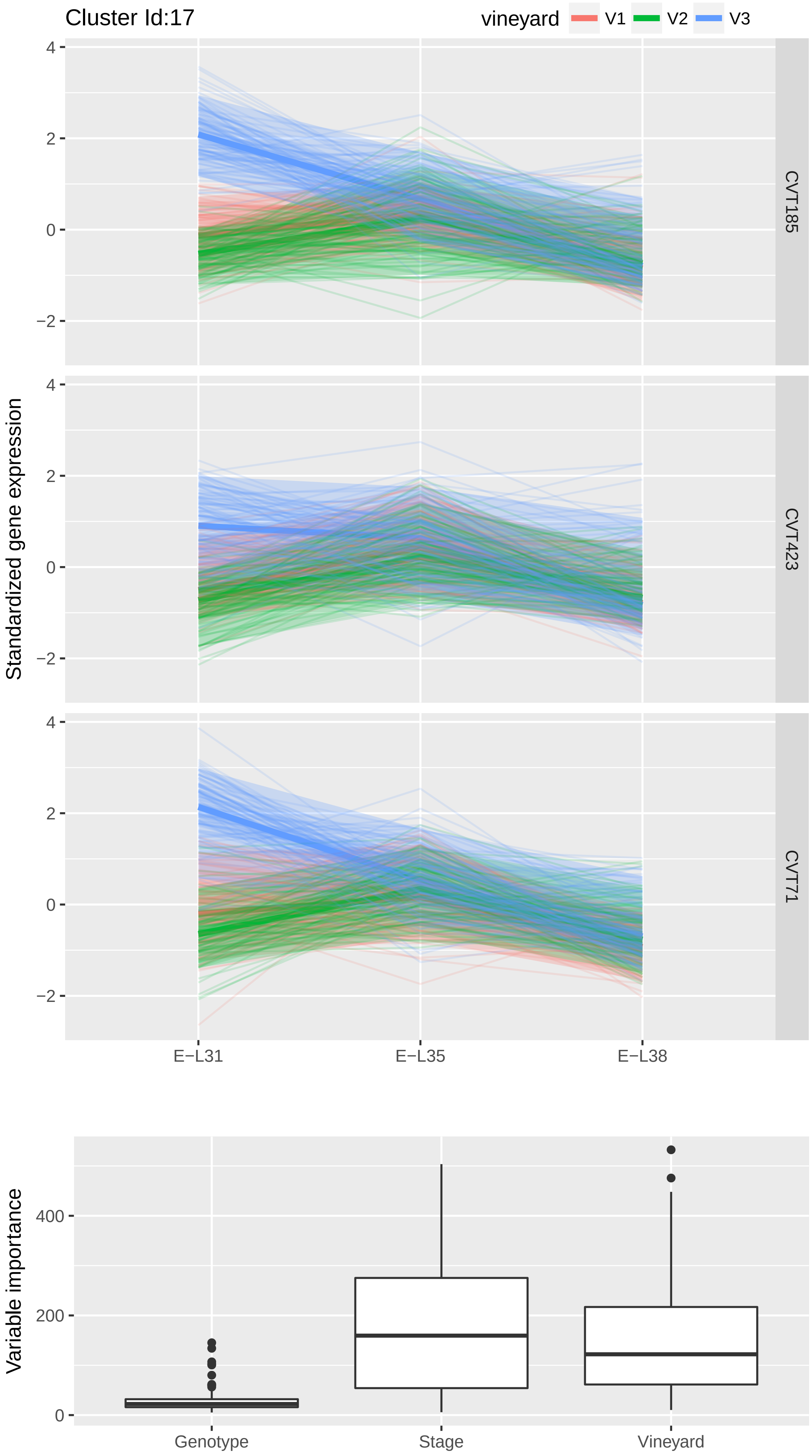

Cluster Id:17
vineyard
V1
V2
V3
Standardized gene expression
CVT185
CVT423
CVT71
4
2
0
−2
E−L31
E−L35
E−L38
Variable importance
400
200
0
Genotype
Stage
Vineyard

Cluster no. 18

```
## Number of genes in the cluster: 92
## Homogeneity Index:    0.45
## Variable importance for Stage:    Median =  175.5  - Rank =  95
## Variable importance for Clone:    Median =  24.64  - Rank =  23
## Variable importance for Vineyard: Median =  61.19  - Rank =  33
##
## Gene ID                Gene Annotation
## VIT_08s0007g07060  -  Unknown protein
## VIT_18s0072g01160  -  Unknown
## VIT_10s0116g00990  -  Pentatricopeptide (PPR) repeat-containing protein
## VIT_07s0104g00370  -  Isoamylase-type starch-debranching enzyme 2
## VIT_08s0040g00060  -  Receptor protein kinase
## VIT_14s0036g00690  -  Pentatricopeptide (PPR) repeat-containing
## VIT_17s0000g04340  -  Unknown protein
## VIT_13s0067g03490  -  ARR9 typeA
## VIT_11s0103g00760  -  CIA2 (chloroplast import apparatus 2)
## VIT_00s0184g00200  -  No hit
## VIT_00s0160g00330  -  TIR-NBS-LRR disease resistance
## VIT_17s0000g00320  -  Auxin response factor 2
## VIT_13s0019g02390  -  Dihydrofolate synthetase
## VIT_00s0184g00120  -  R protein L6
## VIT_03s0038g00580  -  GATA transcription factor 25
## VIT_04s0023g00310  -  CBS domain-containing protein
## VIT_11s0052g01380  -  Calmodulin-binding protein
## VIT_14s0108g01540  -  FAR1-related sequence 5
## VIT_03s0180g00210  -  Myb domain protein R1
## VIT_04s0008g05850  -  CCR4-NOT transcription complex subunit 7/8
## VIT_08s0007g04030  -  Unknown protein
## VIT_07s0005g00510  -  Monoglyceride lipase
## VIT_13s0084g00660  -  Basic Leucine Zipper Transcription Factor (VvbZIP35)
## VIT_00s0299g00100  -  Arabidopsis thaliana homeobox protein 2
## VIT_19s0014g03090  -  CBS domain-containing protein
## VIT_10s0003g02480  -  Xyloglucan endotransglycosylase/hydrolase precursor XTH-21
## VIT_00s0160g00340  -  Unknown
## VIT_07s0129g00030  -  Short-root transcription factor (SHR)
## VIT_00s1375g00010  -  Meiotic recombination 11 MRE11
## VIT_07s0005g04940  -  Unknown protein
## VIT_01s0011g05260  -  DELLA protein GAI1
## VIT_00s0184g00210  -  No hit
## VIT_14s0083g00120  -  Myb domain protein 91
## VIT_03s0063g01730  -  Viral-response family protein-like
## VIT_15s0046g02200  -  SEC10 (exocyst complex component SEC10)
## VIT_12s0034g00660  -  TIR-NBS-LRR-TIR disease resistance protein
## VIT_00s1955g00010  -  No hit
## VIT_12s0034g00770  -  TIR-NBS-LRR-TIR disease resistance protein
## VIT_12s0034g00910  -  R protein L6
## VIT_02s0025g04750  -  Glycerate dehydrogenase
## VIT_07s0005g00220  -  DNA mismatch repair protein PMS2
## VIT_12s0059g02700  -  Pentatricopeptide (PPR) repeat-containing
## VIT_02s0012g01860  -  RNA recognition motif (RRM)-containing protein
## VIT_05s0049g00950  -  Phosphoenolpyruvate carboxylase kinase
## VIT_11s0052g00510  -  Unknown protein
## VIT_10s0003g01390  -  Cupin, RmlC-type
## VIT_12s0142g00310  -  Oxygen evolving enhancer 3 (PsbQ)
```

```
## VIT_00s0686g00010  -  GTP-binding protein engB
## VIT_00s0335g00020  -  TIR-NBS-LRR disease resistance
## VIT_07s0104g01030  -  Serine/threonine-protein phosphatase BSL1
## VIT_10s0116g01260  -  Prefoldin
## VIT_07s0005g03820  -  Acyl-[acyl-carrier-protein] desaturase
## VIT_00s0471g00030  -  GTP-binding protein engB
## VIT_11s0016g02450  -  Serine O-acetyltransferase (SAT-52)
## VIT_12s0059g01260  -  Plastidic glucose transporter 1
## VIT_14s0060g01970  -  F-box domain containing protein
## VIT_05s0094g01390  -  Ion channel OEP37
## VIT_17s0000g10210  -  Unknown protein
## VIT_01s0137g00060  -  Unknown protein
## VIT_08s0058g00550  -  No hit
## VIT_05s0102g01140  -  SKIP16 (SKP1/ASK- interacting  protein 16)
## VIT_08s0040g02970  -  Protein phosphatase 2C
## VIT_18s0001g07730  -  TIFY gene family (VvZML4)
## VIT_08s0007g06400  -  KH domain and CCCH containing protein
## VIT_00s0532g00030  -  Thioredoxin H
## VIT_18s0001g01270  -  Ankyrin
## VIT_18s0001g07920  -  Pentatricopeptide (PPR) repeat-containing
## VIT_15s0048g00950  -  Glutathione S-transferase Z2 GSTZ2
## VIT_13s0019g04000  -  Pseudouridine synthase, RluA
## VIT_06s0004g02770  -  SIT4 phosphatase-associated
## VIT_18s0001g02440  -  Aldehyde Dehydrogenase (VvALDH3H1)
## VIT_00s0415g00060  -  Bromodomain protein
## VIT_04s0008g02380  -  Kelch repeat-containing F-box protein
## VIT_01s0011g04920  -  Mitogen-activated Protein Kinase (VvMPK1)
## VIT_13s0067g00920  -  Nuclear transcription factor, X-box binding 1
## VIT_07s0005g01150  -  CTP synthase
## VIT_07s0031g02700  -  Amidase
## VIT_05s0029g00540  -  HEAT repeat-containing protein
## VIT_00s2785g00010  -  Calmodulin-binding protein
## VIT_09s0002g07520  -  formamidopyrimidine-DNA glycosylase
## VIT_06s0004g03360  -  Carbamoyl phosphate synthase large subunit
## VIT_15s0048g00940  -  ATP-dependent DNA helicase 2 subunit 2
## VIT_08s0040g00420  -  Unknown protein
## VIT_04s0008g04950  -  TIFY gene family (VvTIFY2)
## VIT_12s0028g03790  -  No hit
## VIT_00s0181g00220  -  Calmodulin-binding protein
## VIT_10s0003g01240  -  14-3-3 protein GF14 iota (GRF12)
## VIT_08s0007g05070  -  Zinc finger (C2H2 type) family
## VIT_08s0040g01180  -  No hit
## VIT_09s0002g02320  -  Cell wall apoplastic invertase
## VIT_14s0060g02390  -  SRC2/SRC2 (soybean gene regulated BY cold-2)
## VIT_04s0008g06980  -  Unknown protein
```

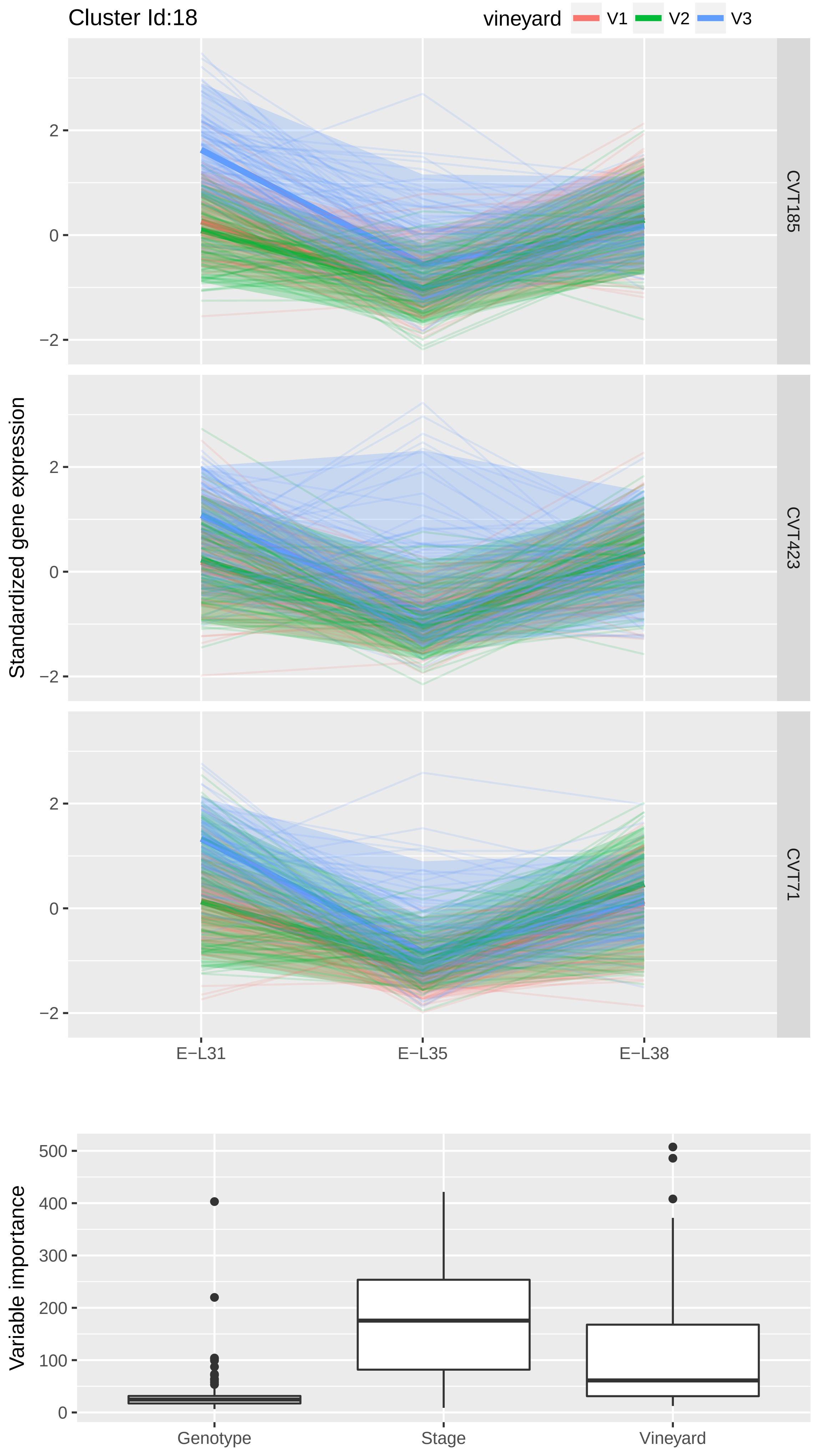

Cluster Id:18
vineyard
V1
V2
V3
Standardized gene expression
CVT185
CVT423
CVT71
2
0
−2
E−L31
E−L35
E−L38
Variable importance
500
400
300
200
100
0
Genotype
Stage
Vineyard

Cluster no. 19

```
## Number of genes in the cluster: 175
## Homogeneity Index:    0.74
## Variable importance for Stage:    Median =  521.7  - Rank =  28
## Variable importance for Clone:    Median =  13.31  - Rank =  80
## Variable importance for Vineyard: Median =  25.42  - Rank =  76
##
## Gene ID               Gene Annotation
## VIT_04s0008g01680  -  EMB2745
## VIT_18s0001g00570  -  Pentatricopeptide (PPR) repeat-containing
## VIT_15s0021g01690  -  Calcium-binding protein
## VIT_12s0055g00970  -  Pentatricopeptide repeat
## VIT_17s0000g06770  -  Pentatricopeptide (PPR) repeat-containing protein
## VIT_04s0023g00300  -  Clavata2 receptor protein (CLV2)
## VIT_14s0068g00760  -  Pentatricopeptide (PPR) repeat-containing
## VIT_17s0000g03060  -  Pentatricopeptide (PPR) repeat-containing protein
## VIT_12s0028g00250  -  Isoprenylcysteine carboxyl methyltransferase
## VIT_11s0016g01170  -  Pentatricopeptide (PPR) repeat-containing
## VIT_15s0048g01220  -  CRS2-associated factor 1
## VIT_06s0004g01710  -  Histone H3
## VIT_07s0191g00120  -  Pentatricopeptide (PPR) repeat-containing protein
## VIT_02s0025g03490  -  Carrier protein, Mitochondrial
## VIT_10s0042g00570  -  Pentatricopeptide (PPR) repeat-containing
## VIT_14s0066g00420  -  Pentatricopeptide (PPR) repeat-containing protein
## VIT_12s0057g00640  -  Pentatricopeptide (PPR) repeat-containing
## VIT_09s0054g01570  -  Strubbelig receptor family 5
## VIT_14s0036g00660  -  Exportin-related
## VIT_14s0066g01750  -  EMB506 ankyrin repeat protein
## VIT_17s0000g06810  -  myb family transcription factor / ELM2 domain-containing
## VIT_12s0059g01950  -  Pentatricopeptide (PPR) repeat-containing protein
## VIT_04s0008g04320  -  EMB2410
## VIT_15s0048g01790  -  Dimethyladenosine transferase
## VIT_03s0017g01800  -  Pentatricopeptide (PPR) repeat-containing protein
## VIT_01s0011g00220  -  WRKY DNA-binding protein 3 (WRKY-17), WRKY Transcription Factor (VvWRK
## VIT_13s0067g00970  -  Unknown
## VIT_13s0019g00590  -  Kinase IkappaB ABA-overly sensitive 1 (ABO1/ELO2)
## VIT_07s0104g01570  -  Metal tolerance protein C1 (AtMTPc1)
## VIT_07s0151g00550  -  BUD32 family protein kinase
## VIT_05s0077g00600  -  Pentatricopeptide (PPR) repeat-containing protein
## VIT_17s0000g07230  -  Activating signal cointegrator 1 complex subunit 3
## VIT_00s0270g00090  -  Pentatricopeptide repeat-containing protein
## VIT_14s0030g00630  -  Ribosomal protein L18 50S
## VIT_02s0012g01730  -  Integrase core domain containing protein
## VIT_00s0227g00030  -  Gag-pol polyprotein
## VIT_08s0007g03070  -  Pentatricopeptide (PPR) repeat-containing protein
## VIT_00s1566g00010  -  UDP-rhamnose/rhamnosyltransferase
## VIT_05s0020g03630  -  Pentatricopeptide (PPR) repeat-containing protein
## VIT_16s0098g00640  -  Thaumatin
## VIT_16s0039g00210  -  Pentatricopeptide (PPR) repeat-containing
## VIT_17s0000g06260  -  Pentatricopeptide (PPR) repeat-containing protein
## VIT_08s0040g03330  -  RNA-binding protein
## VIT_18s0001g01070  -  Pentatricopeptide (PPR) repeat-containing protein
## VIT_14s0060g00320  -  D111/G-patch
## VIT_03s0038g02950  -  Pentatricopeptide (PPR) repeat-containing protein
## VIT_17s0000g06480  -  Pentatricopeptide (PPR) repeat-containing protein
```

```
## VIT_16s0098g01840  -  EMB1796 (embryo defective 1796)
## VIT_08s0007g04280  -  Pentatricopeptide (PPR) repeat-containing
## VIT_04s0008g05110  -  Unknown
## VIT_00s0467g00040  -  No hit
## VIT_01s0011g03630  -  Asp/Glu racemase
## VIT_10s0042g01390  -  Pentatricopeptide (PPR) repeat-containing protein
## VIT_17s0000g02200  -  ABC Transporter (VvWBC6 - VvABCG6)
## VIT_00s1364g00020  -  EMB1674 (embryo defective 1674) kinase interacting family protein
## VIT_05s0020g02950  -  Deoxyribose-phosphate aldolase 2
## VIT_18s0001g14120  -  Translation initiation factor eIF-3 subunit 3
## VIT_09s0002g05690  -  Pentatricopeptide (PPR) repeat-containing
## VIT_04s0044g01180  -  Cell division protease FtsH
## VIT_15s0107g00400  -  Vegetative storage protein
## VIT_15s0046g03020  -  Pentatricopeptide (PPR) repeat-containing protein
## VIT_05s0029g00490  -  Pentatricopeptide (PPR) repeat
## VIT_14s0036g01410  -  Gag-pol polyprotein
## VIT_08s0040g01150  -  Small molecular heat shock protein 10
## VIT_14s0128g00700  -  EMB2261 (embryo defective 2261)
## VIT_15s0021g01680  -  Pentatricopeptide repeat-containing protein
## VIT_01s0011g00230  -  Dihydropterin pyrophosphokinase /dihydropteroate synthase
## VIT_05s0049g00850  -  Retrotransposon protein, Unclassified
## VIT_05s0077g01320  -  Pentatricopeptide (PPR) repeat-containing protein
## VIT_00s0483g00050  -  EMB1674 (embryo defective 1674) kinase interacting family protein
## VIT_19s0014g00940  -  Serine/threonine Protein kinase BNK1
## VIT_07s0104g00290  -  Unknown protein
## VIT_13s0064g00040  -  Unknown protein
## VIT_04s0008g03500  -  Ankyrin
## VIT_19s0015g00780  -  DNA-directed RNA Polymerase III subunit C5
## VIT_13s0158g00430  -  R protein MLA10
## VIT_00s0559g00010  -  Membrane bound O-acyl transferase (MBOAT) family protein
## VIT_00s1360g00010  -  Pentatricopeptide repeat
## VIT_19s0015g00900  -  Pentatricopeptide (PPR) repeat-containing
## VIT_05s0029g01130  -  Pentatricopeptide (PPR) repeat-containing protein
## VIT_13s0019g00220  -  Flowering Locus Y FY
## VIT_00s0264g00130  -  Ankyrin repeat protein, chloroplast precursor (AKRP)
## VIT_09s0070g00450  -  No hit
## VIT_16s0050g00040  -  Pentatricopeptide (PPR) repeat-containing
## VIT_09s0002g01830  -  Tassel serine threonine kinase 1
## VIT_07s0005g00900  -  No hit
## VIT_06s0004g06600  -  Unknown protein
## VIT_04s0069g00420  -  Vacuolar protein sorting 4
## VIT_00s0169g00040  -  CTV.22
## VIT_13s0067g02100  -  Pentatricopeptide (PPR) repeat-containing protein
## VIT_16s0050g00810  -  Ubiquitin-protein ligase
## VIT_00s0174g00290  -  Unknown protein
## VIT_08s0007g05940  -  Unknown
## VIT_10s0003g02700  -  Pre-mRNA-processing factor 39
## VIT_03s0063g01920  -  Pentatricopeptide (PPR) repeat
## VIT_11s0016g02190  -  Unknown protein
## VIT_16s0050g02260  -  Unknown protein
## VIT_14s0068g01660  -  Pentatricopeptide (PPR) repeat-containing protein
## VIT_12s0057g00970  -  Pentatricopeptide (PPR) repeat-containing
## VIT_06s0004g04300  -  Histone H2B
## VIT_12s0055g00400  -  Unknown
```

```
## VIT_05s0029g00210  -  Ubiquitin-specific protease 2 (UBP2)
## VIT_14s0108g01250  -  Unknown
## VIT_00s0525g00030  -  Pentatricopeptide (PPR) repeat-containing
## VIT_17s0000g07530  -  Transportin-SR
## VIT_16s0098g01410  -  Pentatricopeptide (PPR) repeat-containing
## VIT_13s0074g00410  -  Pentatricopeptide (PPR) repeat-containing protein
## VIT_18s0001g14350  -  No hit
## VIT_16s0100g00280  -  EMB2758 (embryo defective 2758)
## VIT_08s0007g03290  -  Pentatricopeptide repeat-containing protein
## VIT_19s0085g01170  -  Seed maturation protein PM41
## VIT_18s0001g00990  -  Calcium Dependent Protein Kinase (VvCPK15)
## VIT_09s0002g06130  -  Zinc finger (CCCH-type) family protein
## VIT_08s0058g00760  -  Pentatricopeptide (PPR) repeat-containing protein
## VIT_09s0054g00550  -  No hit
## VIT_10s0003g01760  -  Pentatricopeptide (PPR) repeat-containing protein
## VIT_18s0001g12460  -  flavodoxin family protein / radical SAM domain-containing protein
## VIT_00s1684g00010  -  No hit
## VIT_19s0015g01700  -  No hit
## VIT_08s0040g02940  -  Pentatricopeptide (PPR) repeat-containing protein
## VIT_08s0007g00190  -  H/ACA ribonucleoprotein complex subunit 4
## VIT_08s0058g00830  -  MEE40 (maternal effect embryo arrest 40)
## VIT_06s0004g04740  -  G protein beta subunit-like
## VIT_04s0008g00170  -  Retrotransposon protein, Unclassified
## VIT_12s0034g01280  -  Exportin1 (XPO1)
## VIT_13s0139g00200  -  Orotate phosphoribosyltransferase
## VIT_15s0021g01700  -  No hit
## VIT_04s0079g00650  -  SET Domain group 40
## VIT_16s0115g00110  -  Unknown
## VIT_05s0020g02920  -  Unknown protein
## VIT_10s0003g00800  -  Unknown
## VIT_15s0021g01720  -  Calcium-binding protein
## VIT_14s0036g00700  -  Protein prenyltransferase alpha subunit
## VIT_06s0061g01090  -  No hit
## VIT_00s0341g00040  -  Exostosin family protein
## VIT_12s0055g01240  -  P-glycoprotein 11
## VIT_12s0059g01120  -  CAF protein
## VIT_02s0012g03200  -  Armadillo/beta-catenin repeat
## VIT_05s0020g03570  -  LOI1 (lovastatin insensitive 1); binding
## VIT_18s0001g02270  -  Unknown
## VIT_11s0052g00550  -  PTAC9 (plastid transcriptionally active9)OSB2
## VIT_06s0004g08120  -  No hit
## VIT_00s0918g00010  -  Pentatricopeptide repeat
## VIT_03s0088g00220  -  Replication factor A 1, rfa1
## VIT_09s0002g09140  -  ERF/AP2 Gene Family (VvERF047)
## VIT_08s0007g04920  -  Zinc finger (C3HC4-type ring finger)
## VIT_06s0009g02430  -  No hit
## VIT_06s0009g01120  -  Pentatricopeptide (PPR) repeat
## VIT_01s0026g00850  -  Zinc finger protein 5
## VIT_18s0089g01260  -  Pentatricopeptide repeat
## VIT_06s0004g08350  -  ABC Transporter (VvATM1 - VvABCB20)
## VIT_09s0002g07340  -  Transposon protein
## VIT_04s0008g01180  -  Esterase
## VIT_07s0005g04650  -  Transducin family protein / WD-40 repeat
## VIT_01s0011g00510  -  Unknown protein
```

```
## VIT_12s0059g00900  -  Pentatricopeptide repeat
## VIT_06s0004g07820  -  Asp/Glu racemase
## VIT_05s0094g01330  -  Inositol polyphosphate 5'-phosphatase
## VIT_08s0007g02560  -  Ovate family protein 13 OFP13
## VIT_12s0059g00790  -  Pentatricopeptide (PPR) repeat-containing
## VIT_01s0011g00900  -  Pentatricopeptide (PPR) repeat-containing protein
## VIT_00s0259g00130  -  Protein kinase beta-1 subunit 5'-AMP-activated
## VIT_18s0001g07670  -  DsRNA-binding protein LH1 HYL1
## VIT_01s0010g02910  -  ATP binding protein
## VIT_13s0019g00760  -  Histone H4
## VIT_19s0014g05020  -  Histone H2A.6 HTA1
## VIT_04s0044g01550  -  Pentatricopeptide repeat
## VIT_00s0207g00300  -  No hit
## VIT_06s0009g01970  -  Light repressible receptor protein kinase
## VIT_14s0171g00480  -  F-box domain containing protein
## VIT_09s0002g09050  -  Glycoprotein, Mitochondrial
## VIT_12s0035g01790  -  U2(RNU2) small nuclear RNA auxiliary factor 1-like 2
## VIT_05s0020g02500  -  Unknown protein
## VIT_00s1711g00020  -  No hit
## VIT_18s0001g10080  -  Unknown protein
```

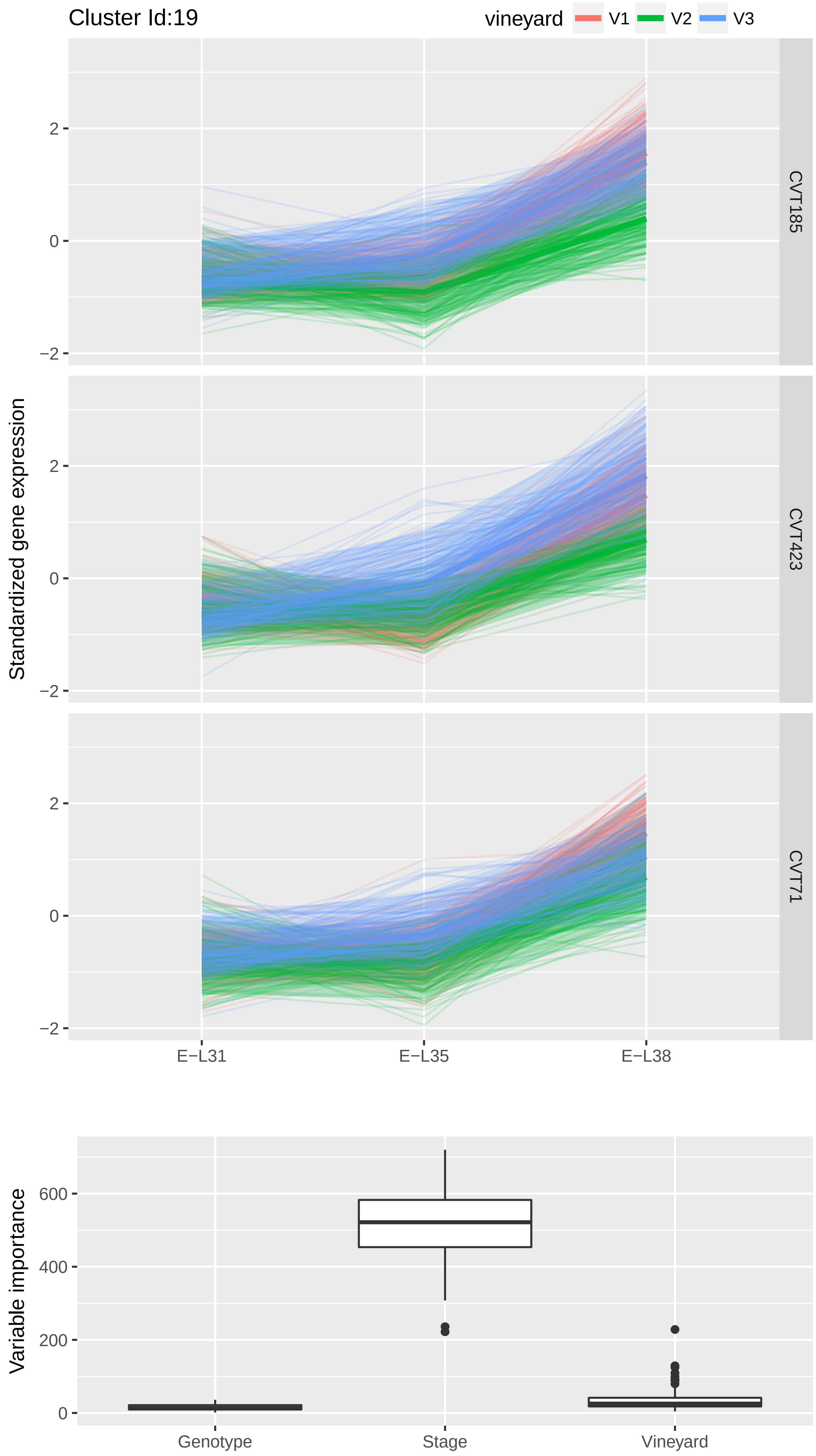

Cluster Id:19
vineyard
V1
V2
V3
CVT185
CVT423
CVT71
Standardized gene expression
2
0
−2
E−L31
E−L35
E−L38
Variable importance
600
400
200
0
Genotype
Stage
Vineyard

Cluster no. 20

```
## Number of genes in the cluster: 111
## Homogeneity Index:    0.62
## Variable importance for Stage:    Median =  355.9  - Rank =  62
## Variable importance for Clone:    Median =  19.57  - Rank =  52
## Variable importance for Vineyard: Median =  40.41  - Rank =  47
##
## Gene ID               Gene Annotation
## VIT_03s0063g01750  -  PI-3-kinase-related kinase SMG-1
## VIT_04s0210g00190  -  Serine O-acetyltransferase, cytosolic
## VIT_14s0108g01550  -  Calmodulin-binding protein
## VIT_05s0020g02910  -  STE11 protein kinase homolog NPK1
## VIT_02s0012g00750  -  Haloacid dehalogenase hydrolase
## VIT_14s0108g01340  -  Pentatricopeptide (PPR) repeat
## VIT_02s0012g01300  -  Unknown
## VIT_17s0000g08950  -  D111/G-patch
## VIT_00s0734g00020  -  GTP-binding protein HflX
## VIT_05s0029g01310  -  Actin related protein 2/3 complex, subunit 4
## VIT_11s0016g00890  -  Unknown protein
## VIT_02s0087g00180  -  Spermidine/putrescine transport system
## VIT_08s0007g03980  -  Pentatricopeptide (PPR) repeat-containing
## VIT_00s0211g00060  -  Metalloendopeptidase
## VIT_19s0014g00980  -  Gamma-glutamyl hydrolase precursor
## VIT_15s0021g01560  -  Leucine carboxyl methyltransferase family protein
## VIT_02s0025g02480  -  Ste20-related protein kinase
## VIT_00s0455g00030  -  EMB2744 (embryo defective 2744)
## VIT_01s0011g01600  -  Exostosin
## VIT_02s0025g00620  -  Ribosomal protein L25
## VIT_03s0038g02160  -  Thaumatin
## VIT_05s0062g00940  -  Trigalactosyldiacylglycerol
## VIT_05s0020g04920  -  fertility restorer
## VIT_08s0007g01990  -  Potassium channel tetramerisation domain-containing protein
## VIT_07s0005g01820  -  Nitrogen fixation NifU
## VIT_14s0066g01390  -  Metal-dependent phosphohydrolase HD domain-containing protein
## VIT_07s0005g02800  -  ADP-glucose pyrophosphorylase large subunit 2
## VIT_15s0024g00080  -  Geranylgeranyl transferase type-2 beta subunit.
## VIT_16s0039g01470  -  Kelch repeat-containing F-box protein
## VIT_13s0147g00220  -  ACT domain containing protein
## VIT_11s0016g05760  -  Unknown protein
## VIT_16s0013g00290  -  Heat shock protein binding
## VIT_16s0039g00160  -  Metallophosphoesterase
## VIT_04s0008g02340  -  Exocyst subunit EXO70 protein
## VIT_01s0244g00060  -  C-Terminal Domain Phosphatase-like 3
## VIT_09s0002g03590  -  ABC transporter G member 21
## VIT_13s0067g00490  -  Protein kinase Xa21
## VIT_14s0083g00200  -  DDT domain-containing protein
## VIT_04s0008g04330  -  EMB2410
## VIT_17s0000g06220  -  Exostosin
## VIT_04s0023g01900  -  AAA-type ATPase
## VIT_11s0037g00730  -  Unknown protein
## VIT_15s0046g01750  -  Golgin candidate 4
## VIT_05s0020g01900  -  No hit
## VIT_19s0015g00740  -  Prefoldin
## VIT_07s0005g01990  -  Pentatricopeptide (PPR) repeat-containing protein
## VIT_08s0007g00460  -  Ribonuclease II family protein
```

```
## VIT_00s0467g00030  -  R protein MLA10
## VIT_11s0206g00060  -  ARR18
## VIT_19s0027g00440  -  DnaJ homolog, subfamily C, member 14
## VIT_13s0067g01130  -  Transducin protein
## VIT_02s0025g02470  -  Unknown protein
## VIT_11s0016g03440  -  Lactoylglutathione lyase
## VIT_05s0020g03150  -  Unknown protein
## VIT_06s0061g00030  -  Hydroxyproline-rich glycoprotein
## VIT_00s0415g00050  -  Bromodomain protein
## VIT_06s0004g00180  -  Transcription initiation factor TFIID-2 (TFIID-2)
## VIT_16s0050g01470  -  Unknown protein
## VIT_09s0002g02350  -  RNA-binding protein Musashi
## VIT_14s0066g01330  -  No hit
## VIT_05s0020g02790  -  Nuclear transcription factor Y subunit C-9
## VIT_14s0083g00740  -  Amino acid permease
## VIT_00s0508g00020  -  Nuclear prelamin A recognition factor; Narf
## VIT_12s0059g01420  -  Photoregulatory zinc-finger protein COP1
## VIT_08s0007g08490  -  Zinc proteinase
## VIT_00s0173g00060  -  No hit
## VIT_01s0026g01070  -  Unknown protein
## VIT_00s0189g00030  -  Phospholipid-transporting ATPase
## VIT_08s0007g04680  -  Expansin  (VvEXPA12)
## VIT_08s0040g00220  -  Exportin 7
## VIT_08s0040g00270  -  Splicing factor 3b, subunit 1
## VIT_05s0020g03090  -  Chloride channel protein (CLC-d)
## VIT_14s0068g01060  -  Unknown
## VIT_11s0016g04410  -  Unknown protein
## VIT_13s0019g04320  -  Homeodomain-leucine zipper protein Revoluta (REV)
## VIT_06s0004g08370  -  Unknown protein
## VIT_01s0011g04320  -  Poly(A) polymerase
## VIT_18s0001g03760  -  Holocarboxylase synthetase 1 (HCS1)
## VIT_08s0007g05120  -  DNA gyrase B subunit
## VIT_00s1864g00010  -  Pentatricopeptide (PPR) repeat-containing protein
## VIT_02s0087g00380  -  No hit
## VIT_02s0087g00440  -  Beta-amylase 8
## VIT_13s0067g01210  -  Anaphase-promoting complex component APC1
## VIT_18s0122g00220  -  Phosphodiesterase/alkaline phosphatase D
## VIT_11s0016g00490  -  CAAX amino terminal protease
## VIT_01s0026g00170  -  Equilibrative nucleoside transporter (ENT1)
## VIT_06s0004g01260  -  U-box protein
## VIT_17s0000g03300  -  Unknown protein
## VIT_17s0000g03430  -  Adaptor-related protein complex 3, delta 1 sub
## VIT_19s0085g00220  -  EMB2758 (embryo defective 2758)
## VIT_04s0044g00300  -  Unknown protein
## VIT_01s0146g00460  -  No hit
## VIT_14s0068g01890  -  Nodulin
## VIT_15s0048g00430  -  Nitroreductase
## VIT_14s0066g00270  -  Dehydration-responsive protein
## VIT_07s0005g01770  -  Pre-mRNA-splicing factor ATP-dependent RNA helicase DHX15/PRP43
## VIT_18s0001g14250  -  Unknown protein
## VIT_08s0007g03410  -  F-box domain containing protein
## VIT_14s0108g01050  -  Pentatricopeptide (PPR) repeat-containing protein
## VIT_11s0016g00900  -  Aldehyde Dehydrogenase (VvALDH7B5)
## VIT_19s0015g00750  -  Sucrase
## VIT_18s0001g03700  -  Unknown protein
```

```
## VIT_08s0040g00230  -  Exportin 7
## VIT_01s0010g02600  -  CDC48C
## VIT_13s0019g02440  -  Mitochondrial substrate carrier family protein
## VIT_12s0028g00520  -  Unknown protein
## VIT_04s0023g00110  -  Alpha-1,4-glucan-protein synthase
## VIT_12s0035g00950  -  Bromodomain
## VIT_17s0000g08930  -  Transducin protein
## VIT_15s0048g02840  -  CRK (CDPK-related kinase)
## VIT_00s0199g00020  -  Thymidylate synthase
```

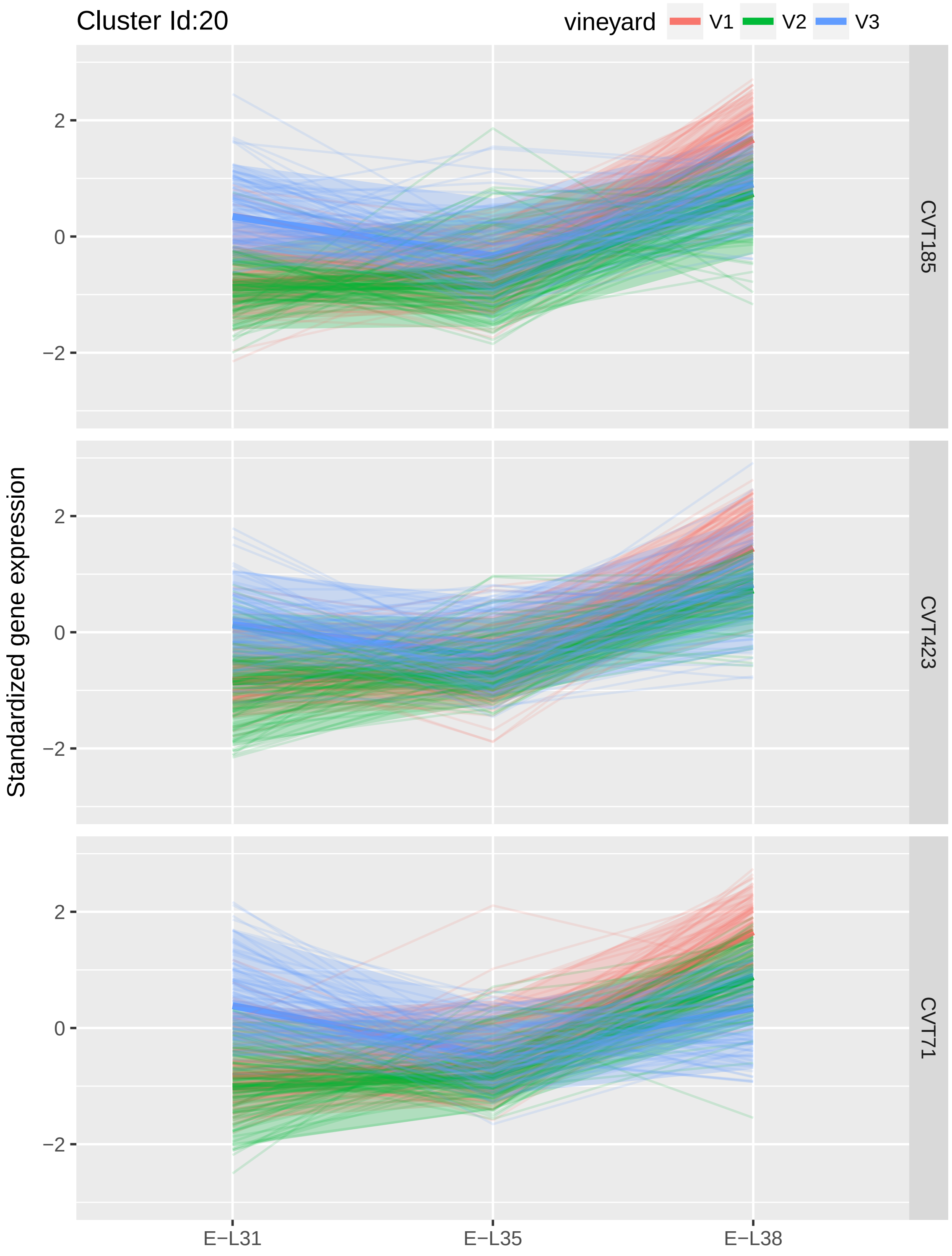
Cluster Id:20
vineyard
V1
V2
V3
Standardized gene expression
CVT185
CVT423
CVT71
2
0
−2
E−L31
E−L35
E−L38

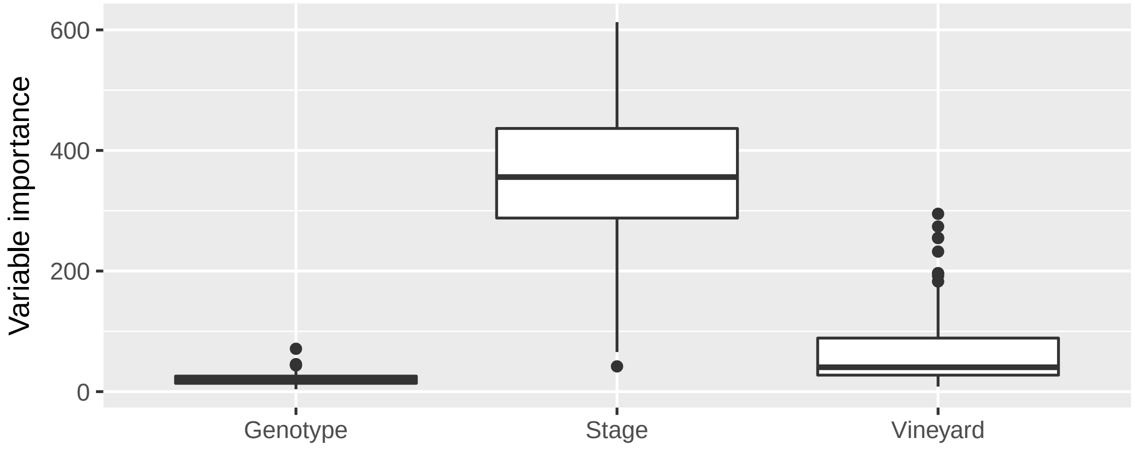
Variable importance
600
400
200
0
Genotype
Stage
Vineyard

Cluster no. 21

```
## Number of genes in the cluster: 227
## Homogeneity Index:    0.76
## Variable importance for Stage:    Median =  582.5  - Rank =  19
## Variable importance for Clone:    Median =  12.02  - Rank =  95
## Variable importance for Vineyard: Median =  16.98  - Rank =  94
##
## Gene ID               Gene Annotation
## VIT_04s0008g03370  -  YebC
## VIT_15s0046g02880  -  Unknown protein
## VIT_13s0067g00900  -  Phosphoinositide-specific phospholipase C
## VIT_11s0016g02010  -  Acid phosphatase survival protein SurE
## VIT_07s0129g01050  -  Myb domain protein 73
## VIT_06s0004g01310  -  Enoyl-[acyl-carrier-protein] reductase [NADH], chloroplast precursor
## VIT_10s0116g00870  -  Malonyl-CoA decarboxylase
## VIT_05s0094g01460  -  Unknown protein
## VIT_02s0154g00200  -  Unknown protein
## VIT_01s0011g05520  -  Unknown protein
## VIT_18s0001g01360  -  Toprim domain-containing protein
## VIT_04s0023g01930  -  basic helix-loop-helix (bHLH) family
## VIT_07s0130g00120  -  Tetratricopeptide repeat (TPR)-containing
## VIT_19s0014g02360  -  Phytosulfokine receptor precursor
## VIT_14s0068g02290  -  Disease resistance protein
## VIT_00s0373g00020  -  HSL2 (HAESA-like 2)
## VIT_19s0014g03140  -  Lanthionine synthetase C
## VIT_16s0098g01670  -  Ribosomal protein L9
## VIT_08s0007g03310  -  Unknown
## VIT_19s0015g00650  -  Sun protein fmu
## VIT_14s0060g00250  -  Unknown
## VIT_14s0128g00510  -  Protein kinase Xa21
## VIT_07s0141g01000  -  No hit
## VIT_05s0102g00100  -  Isoleucyl-tRNA synthetase
## VIT_09s0002g03240  -  Unknown
## VIT_07s0031g02220  -  Unknown protein
## VIT_16s0022g00310  -  EIX receptor 2
## VIT_14s0066g00200  -  Pentatricopeptide (PPR) repeat-containing protein
## VIT_19s0085g00440  -  R protein MLA10
## VIT_05s0020g00860  -  TRNA pseudouridine synthase
## VIT_02s0012g00240  -  Unknown protein
## VIT_10s0071g00930  -  Prolyl oligopeptidase
## VIT_06s0004g02600  -  MOM1 (maintenance of methylation1)
## VIT_12s0034g00920  -  No hit
## VIT_13s0084g00420  -  R protein MLA10
## VIT_14s0108g00150  -  Calmodulin-binding region IQD28
## VIT_14s0171g00190  -  Protoporphyrinogen oxidase
## VIT_10s0071g00610  -  Unknown
## VIT_00s0207g00080  -  UDP-galactose transporter 6
## VIT_07s0005g05030  -  CLIP-associating protein (CLASP)
## VIT_06s0004g07810  -  GTP binding protein EngB
## VIT_08s0040g03130  -  Unknown protein
## VIT_00s1353g00010  -  BAM1 (big apical meristem 1)
## VIT_02s0025g00550  -  Unknown protein
## VIT_12s0059g02460  -  Histone-lysine N-methyltransferase ATXR2
## VIT_18s0041g01340  -  R protein L6
## VIT_17s0053g00890  -  Cyclic nucleotide-binding transporter 1
```

```
## VIT_14s0068g01430  -  Beta-ketoacyl-CoA synthase
## VIT_17s0000g04630  -  Phenylalanyl-tRNA synthetase beta chain
## VIT_00s1308g00020  -  Unknown protein
## VIT_18s0072g00860  -  Photosystem I assembly protein Ycf37
## VIT_13s0019g01470  -  Geranylgeranyltransferase type I beta subunit
## VIT_11s0016g01710  -  Myosin-like protein XIK
## VIT_03s0088g00450  -  FKBP12-rapamycin complex-associated protein
## VIT_02s0012g00250  -  Vacuolar protein sorting 13C protein
## VIT_03s0038g03710  -  RWP-RK domain-containing protein
## VIT_04s0023g00360  -  Unknown protein
## VIT_18s0001g07850  -  Lyase
## VIT_15s0048g00730  -  forkhead-associated domain-containing protein
## VIT_14s0068g01730  -  ABCNAP14
## VIT_09s0002g03930  -  Chaperonin GroEL
## VIT_10s0003g01080  -  Ribosomal protein L34
## VIT_14s0030g01050  -  R protein disease resistance protein
## VIT_01s0244g00140  -  Aspartate kinase
## VIT_09s0002g02340  -  6-phosphogluconate dehydrogenase
## VIT_16s0022g01850  -  No hit
## VIT_15s0048g00720  -  forkhead-associated domain-containing protein
## VIT_12s0035g02120  -  Unknown
## VIT_06s0009g03510  -  Pentatricopeptide (PPR) repeat-containing protein
## VIT_12s0028g01220  -  Zinc finger (C3HC4-type ring finger)
## VIT_06s0009g01840  -  CBL-interacting protein kinase 3 CIPK3
## VIT_08s0032g01170  -  Deoxyribonuclease tatD
## VIT_13s0019g04360  -  GCN5 N-acetyltransferase (GNAT)
## VIT_05s0020g01480  -  Ribosomal protein S21 (RPS21C) 40S
## VIT_03s0063g01100  -  Unknown protein
## VIT_11s0052g00330  -  Choline monooxygenase (CMO)
## VIT_07s0205g00110  -  Glycogen synthase 2
## VIT_18s0089g00570  -  Acetyl-CoA acetyltransferase, cytosolic 2
## VIT_17s0000g00770  -  NAC domain-containing protein (VvNAC07)
## VIT_05s0029g00730  -  Spermine/spermidine synthase
## VIT_13s0047g00720  -  Unknown
## VIT_10s0003g00630  -  Negative regulator of systemic acquired resistance (SNI1)
## VIT_06s0009g01460  -  Homoserine dehydrogenase
## VIT_16s0050g02340  -  Regulator of nonsense transcripts 1
## VIT_14s0030g01710  -  Unknown protein
## VIT_01s0010g00820  -  2-oxoglutarate decarboxylase
## VIT_19s0015g00140  -  Ribosomal protein S10 30S
## VIT_00s0181g00150  -  F-box domain containing protein
## VIT_15s0021g02570  -  CCR4-NOT transcription complex, subunit 1
## VIT_18s0041g02400  -  Aldehyde oxidase
## VIT_03s0038g00840  -  BPC4/BBR/BPC4/BPC4
## VIT_00s0366g00020  -  CRK10 (cysteine-rich RLK10)
## VIT_18s0041g01750  -  RPS4 (resistant to p. syringae 4)
## VIT_03s0017g01920  -  APR9 Tetratricopeptide helical
## VIT_07s0005g03330  -  EMB1879 (embryo defective 1879)
## VIT_18s0041g00490  -  Proton-dependent oligopeptide transport (POT) family protein
## VIT_09s0002g06990  -  Phosphatidic acid phosphatase / PAP2
## VIT_07s0151g00460  -  3-methylcrotonyl-CoA carboxylase
## VIT_15s0024g00860  -  Unknown
## VIT_16s0050g02350  -  Regulator of nonsense transcripts 1
## VIT_04s0008g02650  -  Unknown protein
## VIT_14s0030g01040  -  No hit
```

```
## VIT_18s0001g09650  -  CYP81E1
## VIT_04s0008g00960  -  Calcineurin B protein 10
## VIT_16s0022g01860  -  Cleavage and polyadenylation specificity factor subunit 1
## VIT_06s0004g07020  -  Ethylene overproducer 1 (ETO1)
## VIT_04s0008g00910  -  Histone deacetylase protein (HDA2)
## VIT_06s0004g06560  -  ABC Transporter (VvPDR18 - VvABCG48)
## VIT_14s0068g02020  -  YrdC
## VIT_03s0038g00920  -  Carbohydrate kinase, PfkB
## VIT_07s0031g00260  -  Cyclin delta-3
## VIT_06s0080g00210  -  Histone deacetylase protein (HDA8)
## VIT_06s0061g00600  -  Nodulin family protein
## VIT_13s0084g00360  -  R protein MLA10
## VIT_14s0081g00330  -  Zinc finger, SWIM-type
## VIT_13s0067g00830  -  R protein  PRF  disease resistance protein
## VIT_14s0060g01090  -  LNG1 (LONGIFOLIA1)
## VIT_04s0044g00940  -  ATXR5 (setdomain group 15)
## VIT_02s0012g00270  -  Pleckstriny (PH) domain-containing protein
## VIT_01s0182g00130  -  PHO1-like protein
## VIT_01s0010g02470  -  Pentatricopeptide repeat
## VIT_04s0008g04390  -  No hit
## VIT_18s0001g07190  -  Transducin family protein / WD-40 repeat
## VIT_01s0010g00900  -  Menaquinone biosynthesis protein
## VIT_08s0007g03930  -  Delta14-sterol reductase FK (FACKEL)
## VIT_11s0149g00100  -  DICER-like 4
## VIT_19s0085g00880  -  Aldehyde Dehydrogenase (VvALDH5F1)
## VIT_02s0025g01520  -  Unknown protein
## VIT_13s0067g03120  -  DNA-directed RNA polymerase II subunit A
## VIT_07s0005g05000  -  Leucine-rich repeat
## VIT_13s0019g01620  -  No hit
## VIT_14s0006g01240  -  Protein kinase beta-1 subunit 5'-AMP-activated
## VIT_18s0041g01640  -  Leucine-rich repeat family protein
## VIT_14s0128g00050  -  Thylakoid lumenal 18.3 kDa protein
## VIT_08s0007g03390  -  Cell division protein ftsZ
## VIT_00s0173g00150  -  No hit
## VIT_18s0041g00220  -  TIR-NBS-LRR disease resistance
## VIT_03s0038g00270  -  Root phototropism protein 3 (Non-phototropic hypocotyl protein 3)
## VIT_04s0008g06190  -  Polyprotein
## VIT_11s0037g00240  -  No hit
## VIT_10s0003g01710  -  Ubiquitin domain containing 2
## VIT_10s0003g04940  -  Acyl-CoA synthetases (Acyl-activating enzyme 14)
## VIT_04s0008g02270  -  Hydroxyproline-rich glycoprotein
## VIT_03s0038g00370  -  fructose-1,6-bisphosphatase, chloroplast precursor
## VIT_00s0194g00140  -  Agenet domain-containing protein
## VIT_17s0000g09050  -  No hit
## VIT_04s0069g00930  -  Pentatricopeptide (PPR) repeat-containing
## VIT_14s0068g01110  -  Alpha-mannosidase precursor lysosomal
## VIT_06s0004g01200  -  Beta-1,3-glucan synthase
## VIT_15s0045g01190  -  Retrotransposon protein, Unclassified
## VIT_16s0050g00490  -  Tripeptidyl-peptidase 2
## VIT_09s0002g08290  -  R protein MLA10
## VIT_18s0001g11870  -  No hit
## VIT_09s0054g00340  -  No hit
## VIT_15s0021g01430  -  Glycyl-tRNA synthetase 2, chloroplast/precursor
## VIT_17s0000g04510  -  Acetyl-CoA synthetase
## VIT_01s0010g00870  -  Menaquinone biosynthesis protein
```

```
## VIT_00s0264g00100  -  Phospholipase D delta isoform 1b
## VIT_04s0043g00570  -  Translational activator
## VIT_01s0011g04190  -  Unknown
## VIT_18s0041g02390  -  Abscisic acid 8` hydroxylase (CYP707A2) (VvA8H-CYP707A2.5)
## VIT_08s0007g00110  -  No hit
## VIT_13s0064g01020  -  Unknown protein
## VIT_14s0030g02110  -  Ca2+-transporting ATPase type 2 isoform 8
## VIT_16s0039g00740  -  Histone-lysine N-methyltransferase ASHH3
## VIT_00s0992g00020  -  Heat shock protein (HSP26.5-P) 26.5 kDa class P
## VIT_08s0040g02460  -  Ribosomal protein S15A (RPS15aF) 40S
## VIT_11s0016g05240  -  Nuclear pore complex protein Nup205
## VIT_15s0021g02580  -  CCR4-NOT transcription complex, subunit 1
## VIT_00s0558g00020  -  LINC4 (little nuclei4)
## VIT_00s0324g00010  -  Leucine-rich repeat family protein
## VIT_05s0062g01370  -  Aspartic Protease (VvAP12)
## VIT_14s0128g00300  -  Ctp
## VIT_07s0205g00090  -  Glycogen synthase 2
## VIT_06s0004g02880  -  Receptor-like kinase
## VIT_05s0094g00080  -  Pentatricopeptide repeat
## VIT_13s0019g01640  -  Unknown protein
## VIT_06s0004g00720  -  Glucan endo-1,3-beta-glucosidase 4 precursor
## VIT_15s0046g03550  -  (+)-neomenthol dehydrogenase
## VIT_00s0194g00010  -  Phenylalanine-tRNA ligase
## VIT_04s0023g00470  -  WRKY DNA-binding protein 2 (WRKY-4), WRKY Transcription Factor (VvWRKY
## VIT_09s0002g04670  -  Helicase-related
## VIT_18s0001g13970  -  Myosin-related
## VIT_16s0050g01420  -  Coiled-coil domain-containing protein 111
## VIT_06s0061g01060  -  Magnesium transporter CorA 10
## VIT_03s0091g00120  -  Prp27-like protein
## VIT_07s0104g01510  -  Transcription termination factor mitochondrial mTERF
## VIT_13s0084g00510  -  R protein MLA10
## VIT_13s0047g00990  -  Cinnamyl alcohol dehydrogenase
## VIT_12s0057g00750  -  CF9
## VIT_07s0129g00540  -  Tetratricopeptide repeat (TPR)-containing
## VIT_07s0031g02450  -  Unknown
## VIT_13s0067g02940  -  Amidohydrolase 3
## VIT_04s0008g06180  -  Histone-lysine N-methyltransferase ATX1
## VIT_17s0053g00850  -  Cyclic nucleotide-binding transporter 1
## VIT_06s0061g00570  -  Amino acid permease
## VIT_04s0044g01280  -  Beta-1,3-glucan synthase
## VIT_13s0047g00510  -  DNA gyrase subunit A
## VIT_08s0056g01640  -  3-isopropylmalate dehydratase large subunit 2
## VIT_03s0017g01120  -  Unknown
## VIT_06s0004g06500  -  Villin
## VIT_13s0019g00640  -  THO complex subunit 4
## VIT_05s0020g03130  -  Plastocyanin domain-containing protein
## VIT_01s0137g00510  -  CYP71B10
## VIT_02s0025g00950  -  ABC Transporter (VvMRP13 - VvABCC13)
## VIT_10s0003g04480  -  Unknown protein
## VIT_15s0021g02540  -  CCR4-NOT transcription complex, subunit 1
## VIT_01s0026g00520  -  Nodulin MtN21 family
## VIT_15s0024g00870  -  Unknown
## VIT_13s0019g04530  -  Early flowering 6
## VIT_16s0098g01830  -  Transcription factor jumonji (jmjC) domain-containing protein
## VIT_00s0646g00030  -  Protein serine/threonine kinase
```

```
## VIT_14s0128g00030  -  Unknown protein
## VIT_14s0030g01030  -  Alcohol dehydrogenase
## VIT_08s0040g00600  -  Nascent polypeptide-associated complex NAC
## VIT_11s0052g01570  -  MATE efflux family protein
## VIT_03s0063g00010  -  LRX2 (leucine-rich repeat/extensin 2)
## VIT_09s0002g01440  -  Early flowering 4
## VIT_07s0005g01790  -  Acyl-CoA synthetase long-chain member 2
## VIT_18s0089g00010  -  Disease resistance protein
## VIT_18s0001g11350  -  Unknown protein
## VIT_01s0026g00500  -  Nodulin MtN21 family
## VIT_19s0027g00090  -  AERO1 (arabidopsis endoplasmic reticulum oxidoreductins 1)
## VIT_04s0008g02580  -  V-type H+-transporting ATPase subunit A
## VIT_01s0137g00350  -  ABC Transporter (VvMDR1 - VvABCB1)
## VIT_12s0059g02260  -  Glutamate receptor GLR5
## VIT_07s0005g00040  -  Dephospho-CoA kinase
```

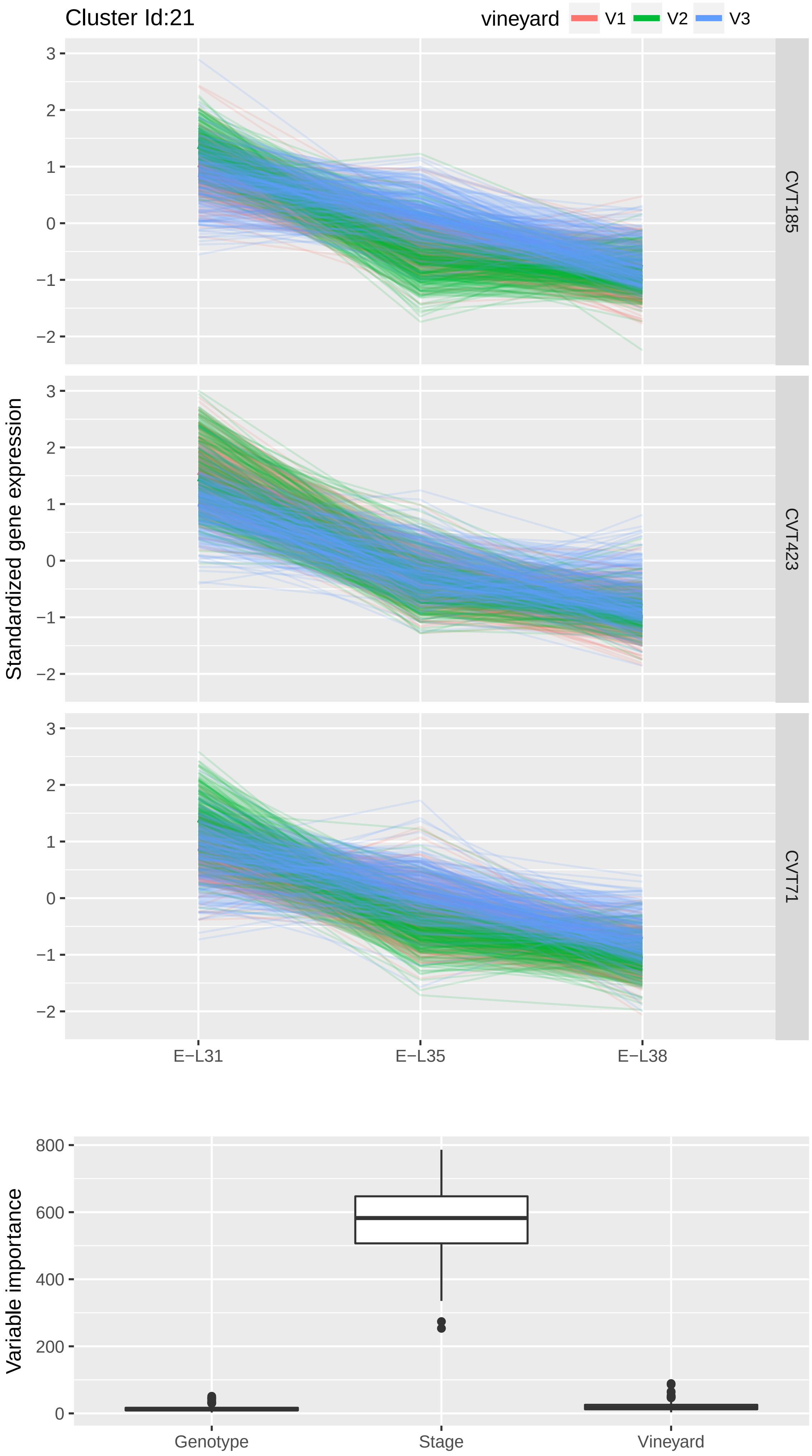

Cluster Id:21
vineyard
V1
V2
V3
CVT185
CVT423
CVT71
Standardized gene expression
3
2
1
0
−1
−2
E−L31
E−L35
E−L38
Variable importance
800
600
400
200
0
Genotype
Stage
Vineyard

Cluster no. 22

```
## Number of genes in the cluster: 226
## Homogeneity Index:    0.71
## Variable importance for Stage:    Median =  388.3  - Rank =  57
## Variable importance for Clone:    Median =  24.06  - Rank =  28
## Variable importance for Vineyard: Median =   40.1  - Rank =  48
##
## Gene ID               Gene Annotation
## VIT_04s0023g03580  -  JR700
## VIT_00s0322g00040  -  RuvB DNA helicase protein
## VIT_08s0007g08940  -  Nuclear pore complex protein Nup93
## VIT_08s0040g03310  -  Unknown protein
## VIT_18s0089g00880  -  CMP/dCMP deaminase, zinc-binding
## VIT_10s0003g02240  -  DNA-binding bromodomain-containing prot
## VIT_00s0218g00070  -  Aldehyde Dehydrogenase (VvALDH6B7)
## VIT_18s0089g01070  -  Vacuolar protein sorting 9 (VPS9)
## VIT_05s0094g00060  -  Mucin
## VIT_03s0132g00290  -  Unknown
## VIT_18s0122g01370  -  Unknown protein
## VIT_12s0059g02300  -  forkhead-associated domain-containing protein
## VIT_06s0004g04470  -  Heat shock protein 70
## VIT_08s0007g03280  -  Phosphoribosylformimino-5-aminoimidazole carboxamide ribotide isomeras
## VIT_19s0015g01090  -  Heat shock protein 81-2 (HSP81-2)
## VIT_18s0089g01060  -  Vacuolar protein sorting 9 (VPS9)
## VIT_14s0066g01740  -  Unknown protein
## VIT_06s0061g01030  -  Aspartic Protease (VvAP13)
## VIT_07s0185g00140  -  Ribosomal protein S21
## VIT_07s0031g01620  -  Developmentally regulated GTP binding protein 1
## VIT_14s0083g00170  -  Translation initiation factor eIF-2B delta subunit
## VIT_18s0001g15280  -  RNA exonuclease 1
## VIT_13s0019g02350  -  Glycoprotein, Mitochondrial
## VIT_05s0020g01060  -  Chromatin structure remodeling complex protein BSH (Protein bushy) (SN
## VIT_17s0000g07000  -  VIP4
## VIT_13s0067g01820  -  Ribosomal protein S4 (RPS4D) 40S
## VIT_18s0001g01940  -  Ripening regulated protein DDTFR8
## VIT_09s0070g00120  -  Phosphatidylinositolglycan class O (PIG-O)
## VIT_05s0094g00800  -  Prp8 binding protein
## VIT_00s0174g00130  -  far-red impaired responsive family protein
## VIT_13s0019g01060  -  SC35 splicing factor, 30 kD (SCL30)
## VIT_09s0018g01140  -  Protein phosphatase 2C
## VIT_10s0003g01570  -  WD-repeat protein 53
## VIT_15s0046g00420  -  EMB1703
## VIT_15s0045g00630  -  Ribosomal protein S15
## VIT_18s0001g01700  -  Histone-lysine N-methyltransferase ASHH2 EFS (early flowering in short
## VIT_00s0173g00260  -  ATP binding
## VIT_00s0174g00200  -  GTPase XAB1
## VIT_00s0271g00080  -  Pentatricopeptide (PPR) repeat membrane-associated salt-inducible prot
## VIT_08s0007g00740  -  Hsp70-interacting protein
## VIT_05s0062g00880  -  Ribosomal protein S11
## VIT_07s0031g00240  -  SEC-C motif-containing protein / OTU cysteine protease
## VIT_19s0085g00350  -  DDT and homeobox transcription factor
## VIT_14s0066g00920  -  Polyribonucleotide nucleotidyltransferase
## VIT_16s0039g00340  -  Harpin-induced protein-related
## VIT_17s0000g01550  -  AAA-type ATPase
## VIT_09s0054g01620  -  myb family
```

```
## VIT_02s0109g00360  -  Translocase of chloroplast 34
## VIT_08s0007g05600  -  Pyrroline-5-carboxylate reductase
## VIT_14s0006g01850  -  Exportin1 (XPO1)
## VIT_18s0001g05630  -  far upstream element-binding protein
## VIT_03s0038g04610  -  FtsJ-like methyltransferase family
## VIT_13s0067g01900  -  ATP-dependent RNA helicase RH18
## VIT_07s0197g00050  -  Tubulin gamma Misato
## VIT_07s0005g01410  -  Dimethyladenosine transferase
## VIT_04s0044g00080  -  Heterogeneous nuclear ribonucleoprotein
## VIT_07s0005g02640  -  Isopenicillin N epimerase
## VIT_05s0020g02940  -  Kinase associated protein phosphatase (KAPP)
## VIT_10s0003g00070  -  Peptidase, trypsin serine and cysteine proteases
## VIT_05s0077g01710  -  U3 small nucleolar RNA-associated protein 5
## VIT_17s0000g01850  -  PDE312/PTAC10 (pigment defective 312)
## VIT_18s0001g05580  -  Unknown protein
## VIT_00s0868g00020  -  Histone H2A.6 HTA1
## VIT_16s0022g01800  -  RRNA-processing protein EBP2
## VIT_14s0171g00430  -  Prohibitin 3
## VIT_03s0088g00990  -  Metal transporter CNNM4 (Cyclin-M4)
## VIT_00s0865g00030  -  Histone H2A.6 HTA1
## VIT_00s0252g00030  -  Unknown protein
## VIT_05s0062g01420  -  Ubiquitin thiolesterase
## VIT_12s0142g00670  -  PMH2 (putative mitochondrial RNA helicase 2)
## VIT_14s0081g00760  -  Activating signal cointegrator
## VIT_15s0048g00360  -  5-enolpyruvylshikimate 3-phosphate synthase
## VIT_11s0016g02270  -  Transducin protein
## VIT_14s0006g00650  -  NET1-associated nuclear protein 1 UTP!/
## VIT_00s1232g00030  -  Pentatricopeptide (PPR) repeat-containing protein
## VIT_04s0008g03800  -  RNA recognition motif (RRM)-containing protein
## VIT_04s0008g05570  -  UDP-glucuronic acid/UDP-N-acetylgalactosamine transporter
## VIT_09s0070g00540  -  Oxidoreductase, zinc-binding dehydrogenase family
## VIT_18s0089g00930  -  SnRK1-interacting protein 1
## VIT_07s0031g03120  -  RNA-binding region RNP-1 (RNA recognition motif)
## VIT_02s0025g02330  -  Zinc finger (HIT type)
## VIT_08s0007g05090  -  DNA-binding protein
## VIT_07s0104g00060  -  XPD/UVH6 (ultraviolet hypersensitive 6)
## VIT_07s0005g02400  -  Unknown protein
## VIT_00s1665g00010  -  Bax inhibitor
## VIT_07s0104g01060  -  SWA1 (slow walker1); nucleotide binding
## VIT_09s0054g00920  -  CGI-144 protein
## VIT_11s0065g00340  -  HUA enhancer 2 (HEN2)
## VIT_00s0207g00240  -  Tetratricopeptide repeat (TPR)-containing
## VIT_14s0083g00790  -  DNA-directed RNA polymerase
## VIT_17s0000g04550  -  Cupin, RmlC-type
## VIT_06s0004g08480  -  DRD1 (defective in rna-directed dna methylation 1)
## VIT_04s0008g05600  -  KEG (keep on going)
## VIT_15s0046g01120  -  Ribosome biogenesis regulatory protein (RRS1)
## VIT_05s0094g00030  -  Unknown protein
## VIT_10s0003g04000  -  ATP-dependent RNA helicase DDX51
## VIT_17s0000g08620  -  U3 small nucleolar RNA-associated protein MPP10
## VIT_17s0000g03020  -  No hit
## VIT_14s0060g02200  -  Phospholipid/glycerol acyltransferase
## VIT_13s0147g00060  -  Ribosomal protein S25 (RPS25E) 40S
## VIT_02s0154g00550  -  DnaJ homolog, subfamily C, member 11
## VIT_18s0001g07480  -  Ulp1 protease
```

```
## VIT_17s0000g01990  -  Pentatricopeptide repeat-containing protein
## VIT_15s0046g01280  -  JAR1-like protein
## VIT_01s0011g00880  -  UvrB/uvrC motif-containing protein
## VIT_03s0038g00640  -  Unknown
## VIT_12s0057g01320  -  Endoribonuclease E
## VIT_13s0067g03550  -  Centromere protein
## VIT_00s0220g00020  -  Pentatricopeptide (PPR) repeat-containing protein
## VIT_00s0956g00030  -  DNA-binding protein
## VIT_18s0001g14900  -  La domain-containing protein
## VIT_11s0037g00630  -  DNA-directed RNA polymerase
## VIT_18s0001g12250  -  COP9 signalosome complex subunit 4
## VIT_14s0060g01740  -  Unknown protein
## VIT_06s0004g04630  -  GTPase ObgE
## VIT_01s0011g02100  -  ATP-dependent Clp protease proteolytic subunit
## VIT_00s0601g00010  -  Pentatricopeptide (PPR) repeat-containing protein
## VIT_04s0008g03940  -  BURP domain-containing protein
## VIT_14s0083g00870  -  Nodulation receptor kinase
## VIT_03s0038g04290  -  Defective IN RNA-directed DNA methylation 1 DRD1
## VIT_02s0025g03690  -  RNA-binding protein NOB1
## VIT_09s0002g00300  -  APUM23 (Arabidopsis PUMILIO 23)
## VIT_13s0147g00200  -  L-aminoadipate-semialdehyde dehydrogenase-phosphopantetheinyltransfera
## VIT_01s0011g01060  -  RNA recognition motif (RRM)-containing protein
## VIT_11s0016g03680  -  Tesmin/TSO1-like CXC domain-containing
## VIT_14s0066g01120  -  PWWP domain-containing protein
## VIT_08s0007g00360  -  myb domain protein 3R-5
## VIT_02s0033g00550  -  No hit
## VIT_02s0025g04220  -  Myb domain protein MYB3R-4
## VIT_09s0054g00830  -  Lipid-binding serum glycoprotein
## VIT_04s0023g01830  -  U3 small nucleolar RNA-associated protein 22
## VIT_18s0001g13100  -  Glycosyl transferase family 1 protein
## VIT_19s0090g01610  -  C2 domain containing protein
## VIT_09s0002g05110  -  EMB2753
## VIT_08s0007g02250  -  Unknown protein
## VIT_12s0057g00900  -  Import inner membrane translocase subunit TIM50
## VIT_09s0002g02750  -  SNF7.1
## VIT_19s0085g01160  -  Unknown protein
## VIT_09s0002g01610  -  Midasin-related
## VIT_08s0056g00470  -  DEAH (Asp-Glu-Ala-His) box polypeptide 8
## VIT_08s0007g00730  -  U3 small nucleolar RNA-associated protein 21
## VIT_08s0007g02900  -  Peptidyl-prolyl cis-trans isomerase-like 1
## VIT_07s0151g00750  -  Rhodanese domain-containing protein
## VIT_18s0001g04420  -  Phosphopantothenate--cysteine ligase
## VIT_07s0104g00390  -  Unknown protein
## VIT_13s0064g00630  -  PHD finger transcription factor
## VIT_05s0020g03830  -  Pentatricopeptide (PPR) repeat-containing
## VIT_04s0023g02590  -  Ribosomal protein L12
## VIT_18s0072g00890  -  Leucine-rich repeat protein kinase
## VIT_12s0028g03440  -  Chromatin remodeling 9
## VIT_02s0025g00890  -  Unknown
## VIT_11s0016g01150  -  OBP3-responsive gene 4 ORG4
## VIT_14s0128g00750  -  Aminopeptidase
## VIT_14s0083g01090  -  N2,N2-dimethylguanosine tRNA methyltransferase
## VIT_11s0016g04450  -  Zinc finger (C3HC4-type ring finger)
## VIT_18s0001g04100  -  Histone acetyl transferase HAM1
## VIT_07s0151g00900  -  Ribosomal protein S24 (RPS24A) 40S
```

```
## VIT_06s0009g02370  -  EMB2261 (embryo defective 2261)
## VIT_05s0020g04260  -  Calcium-transporting ATPase 13 ACA13
## VIT_10s0003g05070  -  HEN1 (HUA enhancer 1)
## VIT_02s0025g02200  -  Methyltransferase type 11
## VIT_17s0000g06950  -  DEAH (Asp-Glu-Ala-His) box polypeptide 16
## VIT_01s0011g04870  -  Homeobox-leucine zipper protein 17 (HB-17)
## VIT_02s0025g03560  -  Transcription factor jumonji (jmjC) DIDO1
## VIT_08s0056g01280  -  MIF4G domain-containing protein
## VIT_19s0085g00130  -  Unknown
## VIT_08s0007g07130  -  Pre-rRNA processing protein RRP5
## VIT_03s0063g00650  -  Unknown protein
## VIT_18s0072g01150  -  Erythroid differentiation factor 1
## VIT_03s0017g02180  -  Pentatricopeptide (PPR) repeat-containing protein
## VIT_03s0038g01950  -  Glycine-rich protein
## VIT_13s0019g03910  -  Unknown
## VIT_18s0001g09690  -  Pentatricopeptide repeat protein
## VIT_14s0108g00990  -  No hit
## VIT_10s0003g02670  -  AML5 (arabidopsis mei2-like protein 5)
## VIT_02s0012g01110  -  Ubiquitin family
## VIT_00s0227g00060  -  WD-40 repeat protein
## VIT_06s0061g00430  -  Ribosomal protein L18
## VIT_19s0027g00550  -  BTB/POZ domain-containing protein
## VIT_07s0005g00470  -  Brix domain-containing protein
## VIT_18s0001g14070  -  Unknown
## VIT_17s0000g09330  -  Pentatricopeptide (PPR) repeat-containing protein
## VIT_16s0022g01260  -  Metalloendopeptidase
## VIT_00s0194g00270  -  RNA-binding protein P67, Chloroplast
## VIT_11s0016g00700  -  Oligouridylate binding protein 1B UBP1B
## VIT_01s0010g00400  -  WD-40 repeat protein
## VIT_06s0004g00790  -  Amino acid permease
## VIT_04s0044g01970  -  Subtilisin protease precursor
## VIT_13s0074g00510  -  ATP-dependent RNA helicase
## VIT_02s0033g01210  -  Cyclops
## VIT_14s0083g01200  -  Nitrilase
## VIT_03s0038g03100  -  Unknown protein
## VIT_07s0031g01750  -  N-acetyltransferase 10
## VIT_17s0000g04780  -  Phosphatidylinositol 4-kinase type-II
## VIT_13s0064g00060  -  R protein MLA10
## VIT_12s0035g00250  -  EMB2758
## VIT_05s0102g00470  -  far-red impaired response 1
## VIT_13s0067g02440  -  Haloacid dehalogenase hydrolase
## VIT_00s0174g00300  -  Intron maturase, type II
## VIT_16s0022g01280  -  Metalloendopeptidase
## VIT_01s0011g02490  -  Cytidine/deoxycytidylate deaminase
## VIT_15s0048g00850  -  Unknown protein
## VIT_13s0019g00610  -  Unknown protein
## VIT_18s0001g10720  -  dihydroflavonal-4-reductase
## VIT_15s0048g02400  -  Serine/threonine-protein kinase
## VIT_04s0008g05810  -  Aldehyde Dehydrogenase (VvALDHI1A3)
## VIT_08s0007g05460  -  Pentatricopeptide (PPR) repeat-containing protein
## VIT_06s0004g03380  -  TRNA-intron endonuclease
## VIT_13s0156g00270  -  Proline-rich family protein
## VIT_15s0048g01140  -  Pex16 shrunken seed protein (SSE1)
## VIT_09s0002g02510  -  Dentin sialophosphoprotein
```

```
## VIT_05s0029g00550  -  HEAT repeat-containing protein
## VIT_09s0070g00550  -  Quinone oxidoreductase
## VIT_01s0011g00770  -  DRL1 (deformed roots and leaves 1)
## VIT_15s0048g02780  -  Unknown protein
## VIT_01s0011g01240  -  Suppressor of FRIGIDA4
## VIT_17s0000g01340  -  Unknown
## VIT_11s0037g01100  -  Mannosyltransferase, putative
## VIT_07s0005g06340  -  Cyclin dependent kinase inhibitor KIP-related protein 3
## VIT_14s0068g00810  -  Helicase
## VIT_02s0012g02710  -  Unknown
## VIT_00s0999g00010  -  DNA-binding storekeeper protein
## VIT_06s0009g02120  -  Nuclear transcription factor Y subunit B related
## VIT_18s0001g05790  -  Unknown protein
## VIT_13s0019g03390  -  Unknown protein
## VIT_17s0000g01350  -  Unknown
```

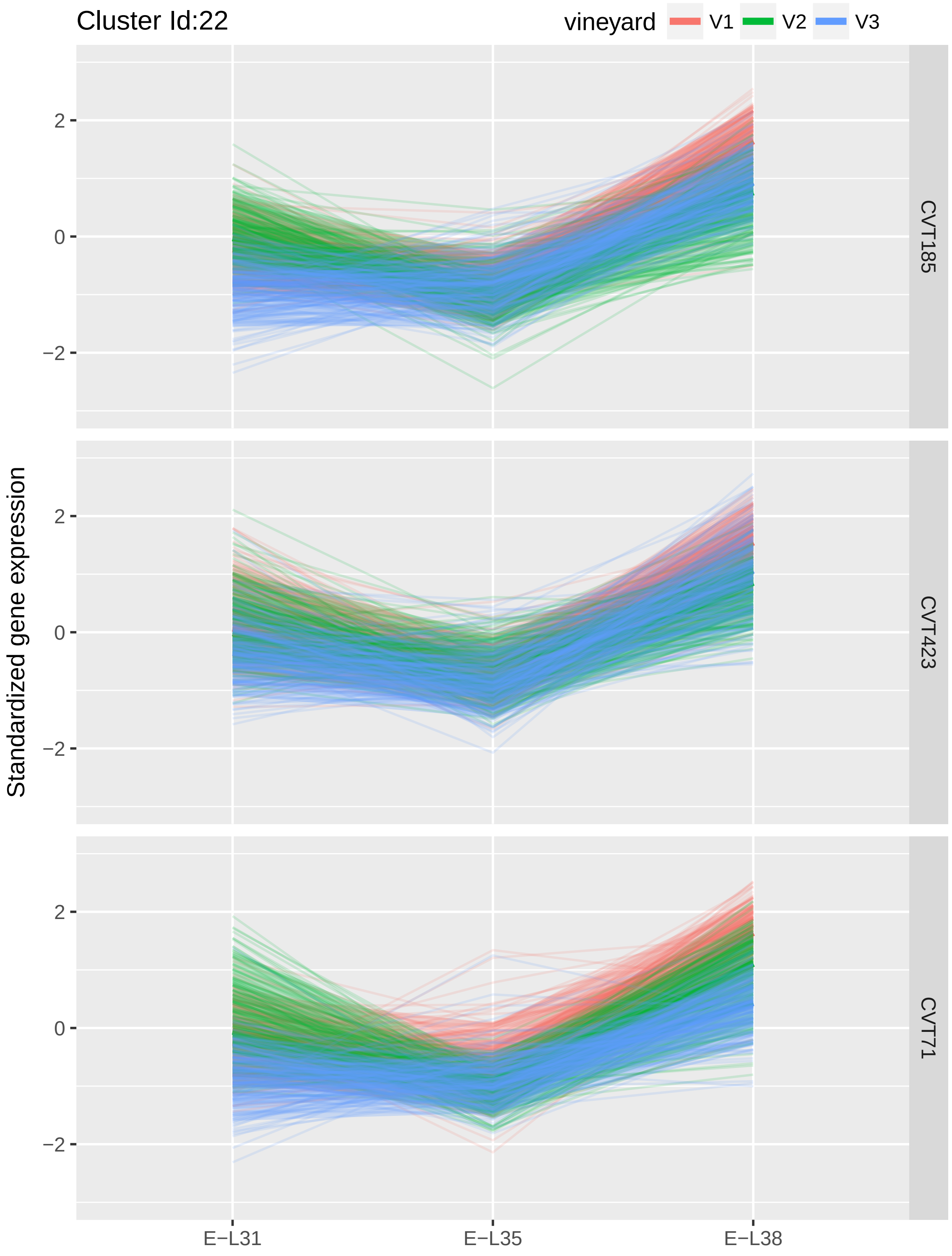
Cluster Id:22
vineyard
V1
V2
V3
Standardized gene expression
CVT185
CVT423
CVT71
2
0
−2
E−L31
E−L35
E−L38

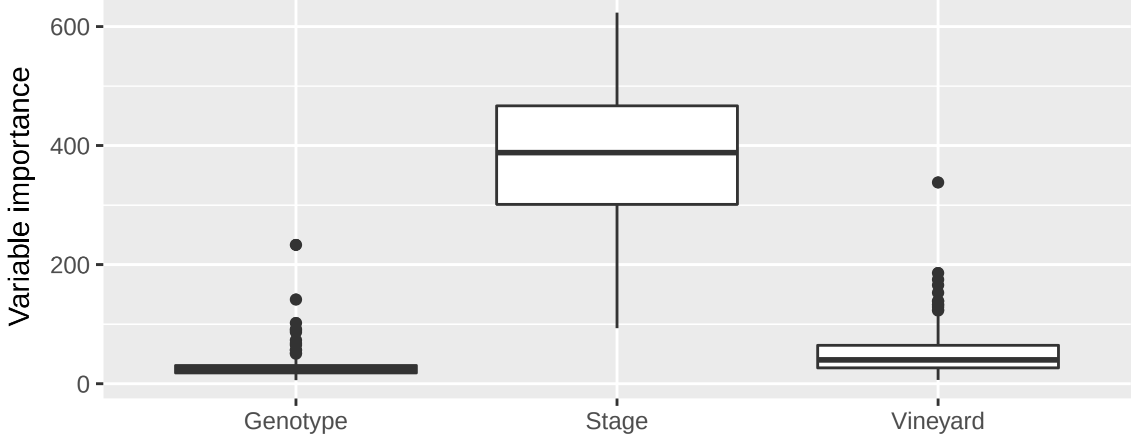
Variable importance
600
400
200
0
Genotype
Stage
Vineyard

Cluster no. 23

```
## Number of genes in the cluster: 254
## Homogeneity Index:    0.89
## Variable importance for Stage:    Median =  608.4  - Rank =  14
## Variable importance for Clone:    Median =  12.34  - Rank =  88
## Variable importance for Vineyard: Median =  14.89  - Rank =  100
##
## Gene ID               Gene Annotation
## VIT_07s0104g00580  -  DNA-directed RNA polymerase III subunit C25
## VIT_18s0001g10130  -  ERF/AP2 Gene Family (VvERF006)
## VIT_14s0066g00980  -  Unknown protein
## VIT_19s0135g00230  -  CYP72A1
## VIT_08s0040g00900  -  myb family
## VIT_13s0067g01350  -  basic helix-loop-helix (bHLH) family
## VIT_13s0019g02460  -  Proton-dependent oligopeptide transport (POT) family protein
## VIT_14s0128g00190  -  Heavy-metal-associated domain-containing protein
## VIT_08s0040g00780  -  P-coumaroyl shikimate 3'-hydroxylase isoform 1
## VIT_02s0087g00470  -  Anthraniloyal-CoA: methanol anthraniloyal transferase
## VIT_03s0091g00450  -  Progesterone 5-beta-reductase
## VIT_08s0040g02600  -  F-box protein
## VIT_16s0050g02460  -  Beta-1,3-galactosyltransferase sqv-2
## VIT_08s0040g01370  -  Heavy-metal-associated domain-containing protein
## VIT_19s0015g01660  -  Phenylalanine-tRNA ligase
## VIT_07s0104g00600  -  Elongation factor Tu
## VIT_08s0007g08380  -  Cellulose synthase CESA3
## VIT_19s0177g00270  -  Molecular chaperone DnaJ
## VIT_03s0088g00050  -  Serine carboxypeptitase 1
## VIT_19s0014g01690  -  Calmodulin-binding region IQD10
## VIT_03s0097g00520  -  Magnesium transporter CorA
## VIT_14s0030g00220  -  Sugar transporter ERD6-like 5
## VIT_04s0044g02010  -  Gibberellin 20 oxidase 2
## VIT_12s0055g00480  -  Amine oxidase
## VIT_05s0077g00110  -  Unknown protein
## VIT_17s0000g04150  -  leucoanthocyanidin reductase 2 (VvLAR2) [Vitis vinifera] GENE ID: 1002
## VIT_02s0012g01350  -  No hit
## VIT_11s0016g02490  -  Ndr family protein
## VIT_08s0007g02920  -  ATAN11 (ANTHOCYANIN11)
## VIT_11s0016g03010  -  Glutamate carboxypeptidase 2
## VIT_07s0031g02910  -  Receptor-like kinase
## VIT_01s0150g00320  -  Nudix hydrolase 13
## VIT_10s0116g00350  -  Unknown protein
## VIT_08s0056g01080  -  MATE efflux family protein
## VIT_03s0091g00210  -  Ethylene-responsive protein
## VIT_04s0008g00420  -  Clavata1 receptor kinase (CLV1)
## VIT_04s0008g03270  -  GATA transcription factor 11
## VIT_09s0002g02220  -  Protein kinase CDG1
## VIT_18s0001g14690  -  Protein kinase
## VIT_02s0025g03170  -  ERF/AP2 Gene Family (VvERF052)
## VIT_13s0073g00140  -  Ovate family protein 13 OFP13
## VIT_01s0026g02270  -  Kinase
## VIT_03s0038g01370  -  ABA-responsive protein (HVA22)HVA22H
## VIT_06s0080g00660  -  No hit
## VIT_12s0028g02520  -  TCP family transcription factor 4
## VIT_04s0069g00960  -  Unknown protein
## VIT_08s0040g01630  -  Translin-associated factor X
```

```
## VIT_12s0035g01910  -  Heat shock protein 18.2 kDa class II
## VIT_05s0049g00090  -  Ethylene receptor (ETR2)
## VIT_00s0772g00010  -  Protein kinase
## VIT_06s0004g01680  -  Unknown protein
## VIT_17s0000g09790  -  BT1 (BTB and TAZ domain protein 1)
## VIT_18s0001g06690  -  Gibberellin 20 oxidase 2
## VIT_16s0013g01990  -  CLL1B clavata1-like receptor S/T protein kinase
## VIT_07s0031g01680  -  CYP86A1
## VIT_18s0001g08650  -  Growth-regulating factor 1
## VIT_05s0020g00930  -  Soluble diacylglycerol acyltransferase
## VIT_01s0011g02000  -  Serine carboxypeptidase S10
## VIT_09s0002g08960  -  EREBP-4
## VIT_18s0001g09400  -  Cytochrome b5 DIF-F (VvCytoB5)
## VIT_03s0063g02620  -  Myb RAD (Transcription factor RAD)
## VIT_06s0004g04080  -  GTP cyclohydrolase II; 3 4-dihydroxy-2-butanone-4-phoshate synthase
## VIT_08s0007g07870  -  basic helix-loop-helix (bHLH) family
## VIT_07s0104g01440  -  Phototropic-responsive NPH3
## VIT_02s0025g01040  -  Receptor protein kinase
## VIT_14s0066g02530  -  Fumarate reductase/succinate dehydrogenase flavoprotein
## VIT_14s0006g02570  -  Protease inhibitor/seed storage/lipid transfer protein (LTP)
## VIT_18s0122g00620  -  Cinnamoyl-CoA reductase
## VIT_11s0206g00120  -  Lipocalin, Temperature-induced
## VIT_19s0014g05080  -  Oxygen evolving enhancer 3 (PsbQ)
## VIT_10s0003g00060  -  L-histidine transmembrane transporter
## VIT_08s0056g01480  -  Cation exchanger, CAX7
## VIT_18s0001g14450  -  Ferredoxin:nadp+ Oxidoreductase PETH
## VIT_03s0038g02510  -  Glyoxylate reductase
## VIT_16s0098g01280  -  Rhodanese domain-containing protein
## VIT_17s0000g00280  -  Magnesium-protoporphyrin O-methyltransferase
## VIT_08s0007g07000  -  Disease resistance-responsive
## VIT_03s0063g02200  -  No hit
## VIT_18s0001g02550  -  Delta-aminolevulinic acid dehydratase, chloroplast precursor
## VIT_15s0048g02680  -  Calmodulin-binding region IQD17
## VIT_19s0014g03650  -  No hit
## VIT_01s0137g00460  -  RGLG2 (ring Domain LIGASE2)
## VIT_01s0011g04340  -  2OG-Fe(II) oxygenase
## VIT_05s0062g00820  -  Unknown protein
## VIT_17s0000g08510  -  PWWP domain-containing protein
## VIT_08s0007g00840  -  Ribulose bisphosphate carboxylase/oxygenase activase, chloroplast
## VIT_00s0194g00160  -  Unknown protein
## VIT_16s0013g00260  -  Superoxide dismutase, Fe-Mn family
## VIT_01s0011g03520  -  Constans-like 16
## VIT_09s0002g05700  -  Phototropic-responsive NPH3
## VIT_08s0040g00850  -  Thylakoid lumenal 20 kDa protein
## VIT_02s0025g02890  -  Ribosomal protein L11
## VIT_16s0022g01740  -  Unknown protein
## VIT_18s0001g14260  -  No hit
## VIT_09s0002g04360  -  Thylakoid membrane phosphoprotein 14 kda, chloroplast precursor
## VIT_18s0122g00700  -  AT-hook protein 1
## VIT_01s0026g01910  -  myb domain protein 88
## VIT_09s0002g02520  -  Ribosomal protein S15A (RPS15aE) 40S
## VIT_03s0088g00290  -  Phytosulfokines PSK2
## VIT_00s0415g00070  -  No hit
## VIT_16s0098g01180  -  Aldo/keto reductase
## VIT_09s0002g00670  -  Auxin responsive SAUR protein
```

```
## VIT_19s0027g00200  -  Photosystem II 10 kDa polypeptide PSBR
## VIT_13s0064g01290  -  basic helix-loop-helix (bHLH) family
## VIT_08s0007g03030  -  Small subunit ribosomal protein S27Ae
## VIT_08s0032g01180  -  CP33 (pigment defective 322)
## VIT_03s0063g01340  -  Gibberellin 20 oxidase 2
## VIT_14s0068g00140  -  No hit
## VIT_05s0020g01330  -  No hit
## VIT_18s0001g15410  -  Alcohol dehydrogenase 1
## VIT_14s0030g01480  -  Cysteine synthase
## VIT_05s0094g00470  -  Zinc finger (C3HC4-type ring finger)
## VIT_14s0066g01590  -  NHL repeat-containing protein
## VIT_05s0094g01160  -  Allyl alcohol dehydrogenase
## VIT_18s0001g04920  -  No hit
## VIT_17s0000g01120  -  Unknown protein
## VIT_00s0225g00070  -  MATE efflux family protein
## VIT_15s0021g02270  -  Unknown protein
## VIT_02s0087g00800  -  Unknown protein
## VIT_07s0005g01750  -  No hit
## VIT_09s0002g08090  -  Oxidoreductase, 2OG-Fe(II) oxygenase
## VIT_12s0055g00590  -  SHR5-receptor-like kinase
## VIT_08s0007g08580  -  AP2-like ethylene-responsive transcription factor
## VIT_05s0094g01350  -  Thiol methyltransferase 1
## VIT_06s0004g05190  -  D-xylose-H+ symporter
## VIT_00s0392g00050  -  Protein-serine/threonine kinase
## VIT_04s0023g01220  -  Dolichyl-diphosphooligosaccharide-protein glycotransferase
## VIT_00s0199g00110  -  Threonine endopeptidase
## VIT_10s0003g05420  -  S-2-hydroxy-acid oxidase, peroxisomal
## VIT_05s0049g01650  -  Cyclopropane fatty acid synthase
## VIT_09s0002g00370  -  Unknown protein
## VIT_10s0042g00210  -  Homeobox protein 23
## VIT_08s0007g06200  -  Rhodanese domain-containing protein
## VIT_00s0415g00080  -  Beta-D-xylosidase
## VIT_03s0091g01010  -  LIM domain protein WLIM1
## VIT_07s0197g00060  -  myb family
## VIT_06s0009g02140  -  Molybdopterin biosynthesis MoaE CNX6
## VIT_18s0157g00090  -  Alliin lyase precursor
## VIT_15s0046g01130  -  Myb Triptychon
## VIT_11s0016g00560  -  Peroxiredoxin bcp
## VIT_07s0104g01220  -  Unknown protein
## VIT_09s0070g00380  -  Unknown protein
## VIT_18s0001g00380  -  Alcohol dehydrogenase
## VIT_02s0012g00450  -  1-aminocyclopropane-1-carboxylate oxidase
## VIT_11s0016g00290  -  Pectinesterase PME3
## VIT_09s0002g06920  -  Unknown protein
## VIT_05s0020g01420  -  FPF1 (flowering promoting factor 1)
## VIT_18s0001g04890  -  Low affinity sulphate transporter
## VIT_01s0010g00940  -  Unknown protein
## VIT_05s0094g01150  -  Allyl alcohol dehydrogenase
## VIT_16s0098g01550  -  No hit
## VIT_07s0095g00750  -  Auxin-independent growth promoter (axi 1)
## VIT_16s0022g02200  -  Subtilase
## VIT_01s0137g00210  -  Photosystem II core complex proteins psbY, chloroplast precursor
## VIT_19s0090g00910  -  Alanine racemase
## VIT_11s0037g00520  -  Translation initiation factor IF-3
## VIT_05s0077g02330  -  Transducin protein
```

```
## VIT_17s0000g06780  -  Dimethylaniline monooxygenase
## VIT_04s0008g01730  -  PSBX (photosystem II subunit X)
## VIT_15s0021g00960  -  1-aminocyclopropane-1-carboxylate oxidase
## VIT_13s0064g01080  -  Thylakoid lumenal 19 kDa protein, chloroplast precursor
## VIT_10s0003g00220  -  Unknown
## VIT_07s0031g00590  -  Triacylglycerol lipase
## VIT_17s0000g07690  -  Transducin family protein / WD-40
## VIT_10s0003g04670  -  Homeobox-leucine zipper transcription factor PHABULOSA
## VIT_14s0128g00860  -  MYB related DIRP family protein
## VIT_01s0011g06590  -  Protease inhibitor/seed storage/lipid transfer protein (LTP)
## VIT_08s0007g02280  -  Phospholipase C
## VIT_17s0000g02470  -  Thaumatin
## VIT_02s0012g00740  -  Dynamin-like protein 2b
## VIT_13s0067g01660  -  Sister chromatid cohesion 1 protein 1 DIF1/SYN1
## VIT_17s0053g00860  -  Cyclic nucleotide-binding transporter 1
## VIT_13s0064g00190  -  RNA polymerase sigma subunit SigD
## VIT_00s0291g00020  -  Unknown protein
## VIT_19s0015g01760  -  Photosystem I reaction center subunit V (PSAG)
## VIT_02s0025g01300  -  Unknown protein
## VIT_03s0091g01290  -  Serine carboxypeptidase S10
## VIT_04s0008g04150  -  RD22
## VIT_11s0037g00570  -  Anthranilate N-benzoyltransferase
## VIT_08s0007g06450  -  Omega-6 fatty acid desaturase, endoplasmic reticulum (FAD2)
## VIT_05s0020g04760  -  DNA-directed RNA polymerase II subunit G
## VIT_12s0028g03910  -  CAAX amino terminal protease
## VIT_17s0000g05650  -  Unknown protein
## VIT_18s0001g02540  -  ARR9 typeA
## VIT_00s0361g00050  -  Protein-serine/threonine kinase
## VIT_03s0088g01190  -  Malate dehydrogenase, glyoxysomal precursor
## VIT_11s0016g00590  -  Invertase/pectin methylesterase inhibitor
## VIT_15s0048g02530  -  Harpin-induced 1
## VIT_01s0011g05660  -  Zinc finger (Ran-binding) family
## VIT_06s0061g00730  -  Aquaporin GAMMA-TIP3/TIP1;3
## VIT_07s0005g00010  -  Glutathione S-transferase 8 GSTF8
## VIT_07s0104g01830  -  Glutathione S-transferase 8 GSTF8
## VIT_18s0089g01210  -  Cupin super
## VIT_04s0008g04250  -  Aspartate aminotransferase
## VIT_05s0049g01320  -  Unknown protein
## VIT_10s0003g01960  -  RKF1 (receptor-like kinase in flowers 1)
## VIT_00s1543g00010  -  Unknown protein
## VIT_12s0059g02480  -  CYCP2;1 (cyclin p2;1)
## VIT_07s0031g02160  -  Protein phosphatase 2C DBP
## VIT_18s0041g00350  -  Dienelactone hydrolase
## VIT_11s0016g02260  -  Unknown
## VIT_15s0048g00370  -  Transketolase, chloroplast precursor
## VIT_02s0012g00990  -  LOL1 (LSD ONE like 1)
## VIT_18s0001g04930  -  Sulfate transporter 1.3
## VIT_18s0076g00250  -  Sucrose-proton symporter 2 SUC2 (SUT2-2)
## VIT_06s0080g00600  -  No hit
## VIT_18s0001g11360  -  VRN1 (reduced vernalization response 1)
## VIT_16s0098g00710  -  Serine protease inhibitor, Kazal-type
## VIT_02s0012g01370  -  No hit
## VIT_15s0048g02500  -  Thylakoid membrane phosphoprotein 14 kda, chloroplast precursor
## VIT_08s0040g03230  -  Unknown protein
## VIT_06s0004g05280  -  Tropinone reductase
```

```
## VIT_12s0034g02180  -  Aminomethyltransferase
## VIT_00s0174g00070  -  Ankyrin repeat protein
## VIT_12s0134g00160  -  Xyloglucan endotransglycosylase/hydrolase 16
## VIT_17s0000g04260  -  Universal stress protein (USP) family protein
## VIT_04s0023g03510  -  Ferredoxin:nadp+ Oxidoreductase PETH
## VIT_05s0020g02180  -  Unknown protein
## VIT_00s0399g00020  -  Protease inhibitor/seed storage/lipid transfer protein (LTP)
## VIT_04s0008g05640  -  Protease inhibitor/seed storage/lipid transfer protein (LTP)
## VIT_14s0083g00650  -  No hit
## VIT_02s0025g04710  -  Unknown protein
## VIT_14s0068g01990  -  Unknown protein
## VIT_08s0056g00330  -  Bromodomain containing
## VIT_04s0008g02200  -  3-beta-hydroxy-delta5-steroid dehydrogenase
## VIT_01s0026g01050  -  Myb family
## VIT_09s0054g01760  -  Unknown protein
## VIT_08s0007g07550  -  GATA transcription factor 11
## VIT_11s0016g01850  -  Anthocyanidin-3-glucoside rhamnosyltransferase
## VIT_13s0064g00830  -  Disease resistance protein RGA2 (RGA2-blb)
## VIT_03s0088g00110  -  Serine carboxypeptidase SCPL17
## VIT_12s0059g02150  -  Aconitate hydratase, cytoplasmic
## VIT_17s0000g02120  -  Unknown protein
## VIT_09s0002g04410  -  No hit
## VIT_04s0023g03710  -  myb domain protein 4 (VvMyb4b)
## VIT_05s0020g00680  -  ABC Transporter (VvPDR17 - VvABCG47)
## VIT_12s0028g03350  -  Squamosa promoter-binding protein (VvSBP11)
## VIT_01s0011g01610  -  Protein kinase C inhibitor
## VIT_09s0002g02110  -  Ribonuclease III
## VIT_01s0026g00640  -  DEAD/DEAH box helicase (RH22)
## VIT_10s0003g02110  -  Lipase GDSL
## VIT_13s0019g04990  -  Nodulin-like protein
## VIT_06s0004g07110  -  Gamma interferon responsive lysosomal thiol reductase family protein
## VIT_10s0003g03850  -  Protease Do-like 5
## VIT_06s0080g00940  -  Nitrilase 4 (NIT4)
## VIT_09s0002g01400  -  myb domain protein 7
## VIT_18s0001g08550  -  Squalene monooxygenase
## VIT_15s0024g00040  -  LHCA3 (Photosystem I light harvesting complex gene 3)
## VIT_15s0024g00660  -  Esterase
## VIT_13s0067g00110  -  CYP72A1
## VIT_04s0043g00380  -  Unknown protein
## VIT_01s0011g05180  -  Bet v I allergen
## VIT_01s0011g00610  -  Unknown protein
## VIT_18s0001g01730  -  Shikimate kinase
```

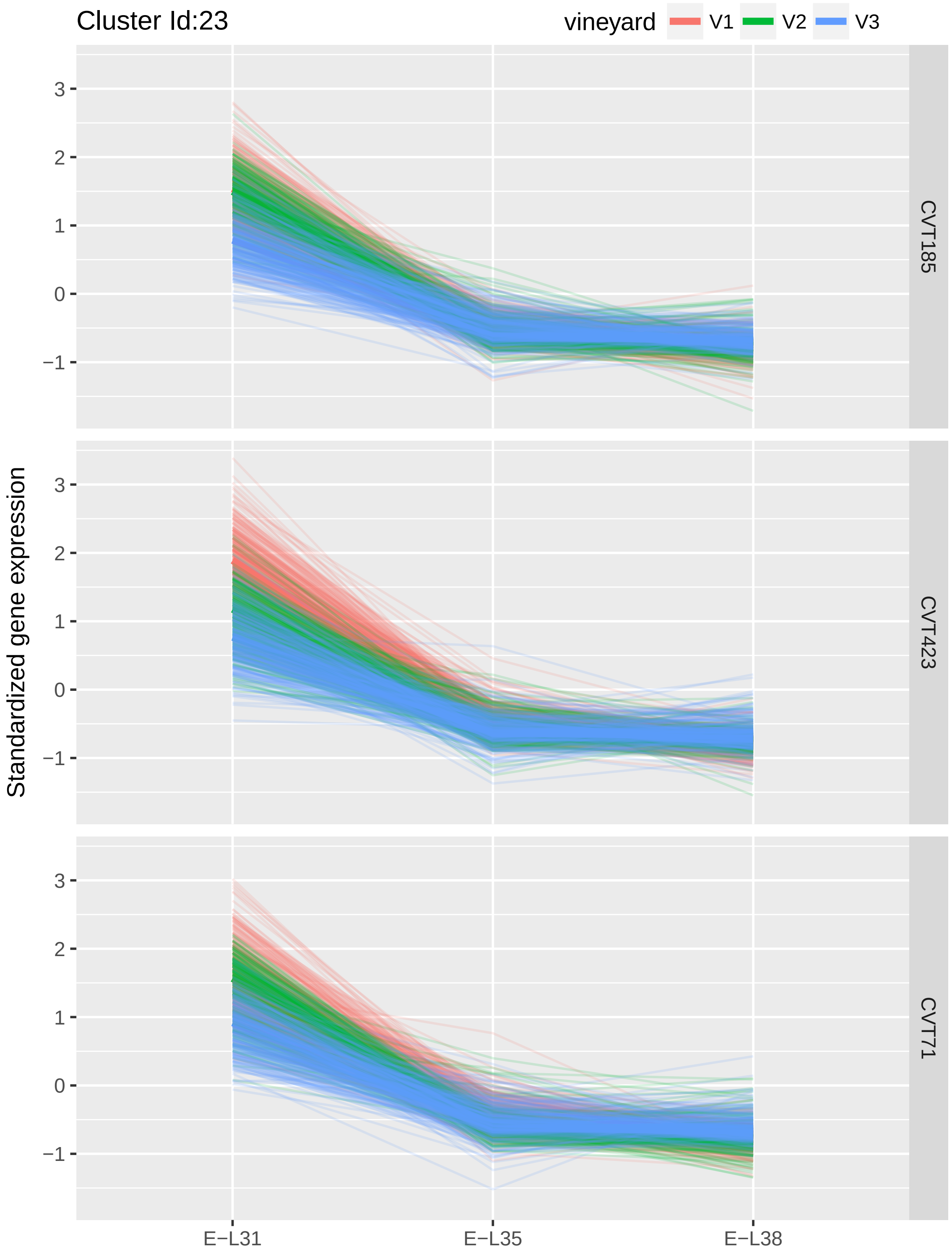

Cluster Id:23
vineyard
V1
V2
V3
Standardized gene expression
CVT185
CVT423
CVT71
3
2
1
0
−1
E−L31
E−L35
E−L38

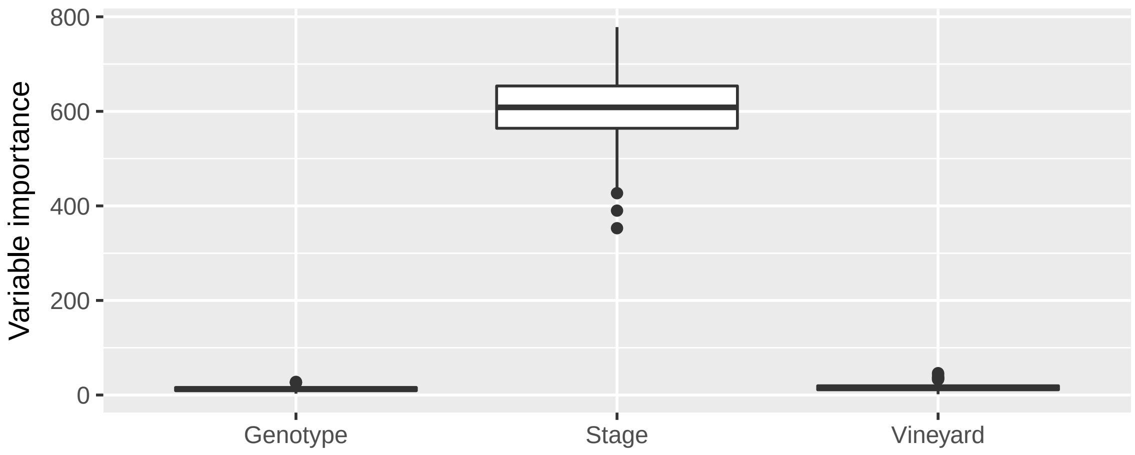

Variable importance
800
600
400
200
0
Genotype
Stage
Vineyard

Cluster no. 24

```
## Number of genes in the cluster: 118
## Homogeneity Index:    0.71
## Variable importance for Stage:    Median =  443.8  - Rank =  49
## Variable importance for Clone:    Median =  23.06  - Rank =  34
## Variable importance for Vineyard: Median =  26.37  - Rank =  75
##
## Gene ID               Gene Annotation
## VIT_19s0177g00320  -  Apolipoprotein D
## VIT_07s0005g04760  -  PfkB-type carbohydrate kinase
## VIT_04s0023g03040  -  Lactoylglutathione lyase
## VIT_00s0233g00190  -  Unknown protein
## VIT_11s0016g02330  -  Prenylated rab acceptor (PRA1)
## VIT_01s0137g00590  -  Pentatricopeptide (PPR) repeat-containing protein
## VIT_06s0009g03740  -  Serine-glyoxylate aminotransferase
## VIT_00s0510g00020  -  Unknown protein
## VIT_04s0210g00140  -  AMP-dependent synthetase and ligase
## VIT_07s0005g06600  -  Unknown protein
## VIT_06s0009g02480  -  myb domain protein 65
## VIT_16s0050g00800  -  Sec-independent protein translocase protein TatA
## VIT_19s0014g00010  -  forkhead-associated domain-containing protein
## VIT_07s0005g02970  -  Myosin-like protein XIC
## VIT_07s0141g00310  -  Phosphoribulokinase/uridine kinase
## VIT_05s0049g02090  -  DNA mismatch repair protein
## VIT_13s0067g03290  -  Ribosomal protein L10
## VIT_18s0001g04700  -  GP5 ubiquitin-like
## VIT_12s0034g01020  -  Ribosomal protein L18
## VIT_04s0023g01610  -  DDM1 (decreased DNA methylation 1)
## VIT_05s0077g01880  -  DAG protein
## VIT_00s0174g00140  -  far-red impaired responsive family protein
## VIT_04s0008g06620  -  Phospholipase D
## VIT_00s0211g00090  -  Serine hydroxymethyltransferase 2
## VIT_03s0017g01010  -  Peptidase C14, caspase catalytic subunit p20
## VIT_19s0027g00080  -  Kinesin motor protein
## VIT_16s0022g00680  -  Multi-copper oxidase (SKU5)
## VIT_19s0085g00490  -  FtsH protease 12
## VIT_05s0049g02130  -  Glutamyl-tRNA(Gln) amidotransferase B
## VIT_02s0012g01020  -  Zinc finger (C3HC4-type ring finger)
## VIT_18s0001g07970  -  Pentatricopeptide (PPR) repeat-containing protein
## VIT_03s0063g02300  -  ATP-dependent Clp protease proteolytic subunit (ClpP)
## VIT_13s0064g00900  -  Unknown protein
## VIT_19s0090g00920  -  Glucan (1,4-alpha-), branching enzyme 1
## VIT_18s0001g11160  -  Unknown protein
## VIT_12s0057g00390  -  Exopolygalacturonase
## VIT_04s0023g00780  -  Phage shock protein A
## VIT_10s0003g00670  -  Apoptosis inhibitory 5 (API5)
## VIT_00s0179g00110  -  Protein phosphatase 2C
## VIT_15s0024g01440  -  Glucose 6 phosphate/phosphate translocator-like protein
## VIT_00s0449g00010  -  Membrane bound O-acyl transferase (MBOAT)
## VIT_05s0020g01670  -  GTP1/OBG
## VIT_09s0070g00100  -  Ribosomal protein S17
## VIT_14s0060g02370  -  DegP protease
## VIT_06s0004g02080  -  TRNA-specific 2-thiouridylase
## VIT_07s0031g01770  -  violaxanthin de-epoxidase (VDE2) (VvVDE2)
## VIT_09s0002g02380  -  Thioredoxin 2
```

```
## VIT_19s0014g02800  -  Unknown protein
## VIT_04s0044g01510  -  Histone deacetylase HDA14
## VIT_04s0210g00110  -  AMP-dependent synthetase and ligase
## VIT_14s0066g00320  -  Pseudouridylate synthase TruB
## VIT_11s0016g01890  -  Telomere repeat binding factor 1
## VIT_09s0018g00680  -  No hit
## VIT_05s0094g01510  -  Unknown protein
## VIT_00s0211g00120  -  Glycine hydroxymethyltransferase
## VIT_18s0001g11640  -  Unknown protein
## VIT_14s0066g01370  -  No hit
## VIT_18s0001g08480  -  EMB1273 [Arabidopsis lyrata subsp. lyrata]
## VIT_02s0087g00460  -  Oxidoreductase, 2OG-Fe(II) oxygenase
## VIT_15s0021g01530  -  VAD1 (vascular associated death1)
## VIT_15s0046g02560  -  basic helix-loop-helix (VvMYCA1)
## VIT_05s0094g00420  -  Endosperm defective 1 EDE1
## VIT_16s0098g01420  -  DOF affecting germination 1
## VIT_07s0104g00130  -  F-box domain containing protein
## VIT_06s0009g02760  -  No hit
## VIT_11s0037g01000  -  Clathrin assembly protein 10
## VIT_00s0204g00020  -  Transcription factor
## VIT_07s0151g01110  -  Ribosome recycling factor, chloroplast precursor
## VIT_11s0016g01160  -  GCN5 N-acetyltransferase (GNAT)
## VIT_15s0046g00980  -  Cyclic nucleotide-regulated ion channel 1
## VIT_00s0333g00020  -  Unknown protein
## VIT_16s0100g00410  -  TraB protein
## VIT_11s0052g00120  -  Purple acid phosphatase 28- ATPAP28/PAP28
## VIT_17s0000g09680  -  RNA-binding protein RBP31
## VIT_17s0000g03110  -  2OG-Fe(II) oxygenase
## VIT_16s0039g01850  -  Syntaxin 7
## VIT_09s0002g01390  -  Autophagy 12a (APG12a)
## VIT_04s0043g00370  -  Ammonium transporter 1;2
## VIT_01s0011g04750  -  Unknown
## VIT_01s0010g01140  -  Unknown
## VIT_08s0007g03270  -  SOUL heme-binding protein
## VIT_07s0104g00810  -  Lipoyltransferase (LIP2p)
## VIT_11s0016g03370  -  Exostosin-like
## VIT_01s0011g01100  -  Pentatricopeptide (PPR) repeat-containing protein
## VIT_11s0037g01280  -  GCN5 N-acetyltransferase (GNAT)
## VIT_05s0102g00030  -  Ankyrin repeat protein family
## VIT_06s0004g01890  -  Cu2+-exporting ATPase HMA5 (heavy metal ATPase 5)
## VIT_18s0001g00610  -  Rho GTPase activator
## VIT_06s0004g06790  -  Gibberellin 2-beta-dioxygenase 7
## VIT_00s0319g00010  -  No hit
## VIT_18s0117g00220  -  Deoxynucleoside kinase
## VIT_16s0050g01510  -  SGT1-2
## VIT_16s0098g01650  -  Protein phosphatase 2C PPH1
## VIT_18s0001g05020  -  Senescence-associated protein
## VIT_15s0021g02370  -  Unknown protein
## VIT_05s0062g01030  -  Unknown protein
## VIT_14s0219g00220  -  CCT motif constans-like
## VIT_13s0064g00790  -  No hit
## VIT_17s0000g06390  -  Pentatricopeptide (PPR) repeat-containing protein
## VIT_19s0014g02350  -  Squamosa promoter-binding protein (VvSBP18)
## VIT_00s0630g00010  -  Lipase class 3
## VIT_14s0083g00240  -  Stress protein
```

```
## VIT_07s0141g00750  -  Triacylglycerol lipase
## VIT_08s0007g03160  -  Signal recognition particle subunit 54 SRP54
## VIT_01s0010g03170  -  No hit
## VIT_10s0116g00330  -  AAA-type ATPase
## VIT_01s0011g05200  -  Phosphoethanolamine N-methyltransferase 1
## VIT_15s0048g01570  -  CYP76C2
## VIT_07s0151g00620  -  Pentatricopeptide (PPR) repeat-containing protein
## VIT_15s0048g01590  -  CYP76B1
## VIT_02s0154g00220  -  Zinc finger (C2H2 type) family
## VIT_01s0010g00580  -  Translin-associated factor X
## VIT_06s0004g07090  -  Unknown protein
## VIT_09s0054g00820  -  Unknown protein
## VIT_17s0000g01110  -  ATFP3 (Arabidopsis thaliana farnesylated protein 3
## VIT_12s0059g01740  -  Phagocytosis and cell motility protein ELMO1
## VIT_05s0020g04630  -  DMI1 protein homologue
## VIT_08s0058g01420  -  Unknown protein
```

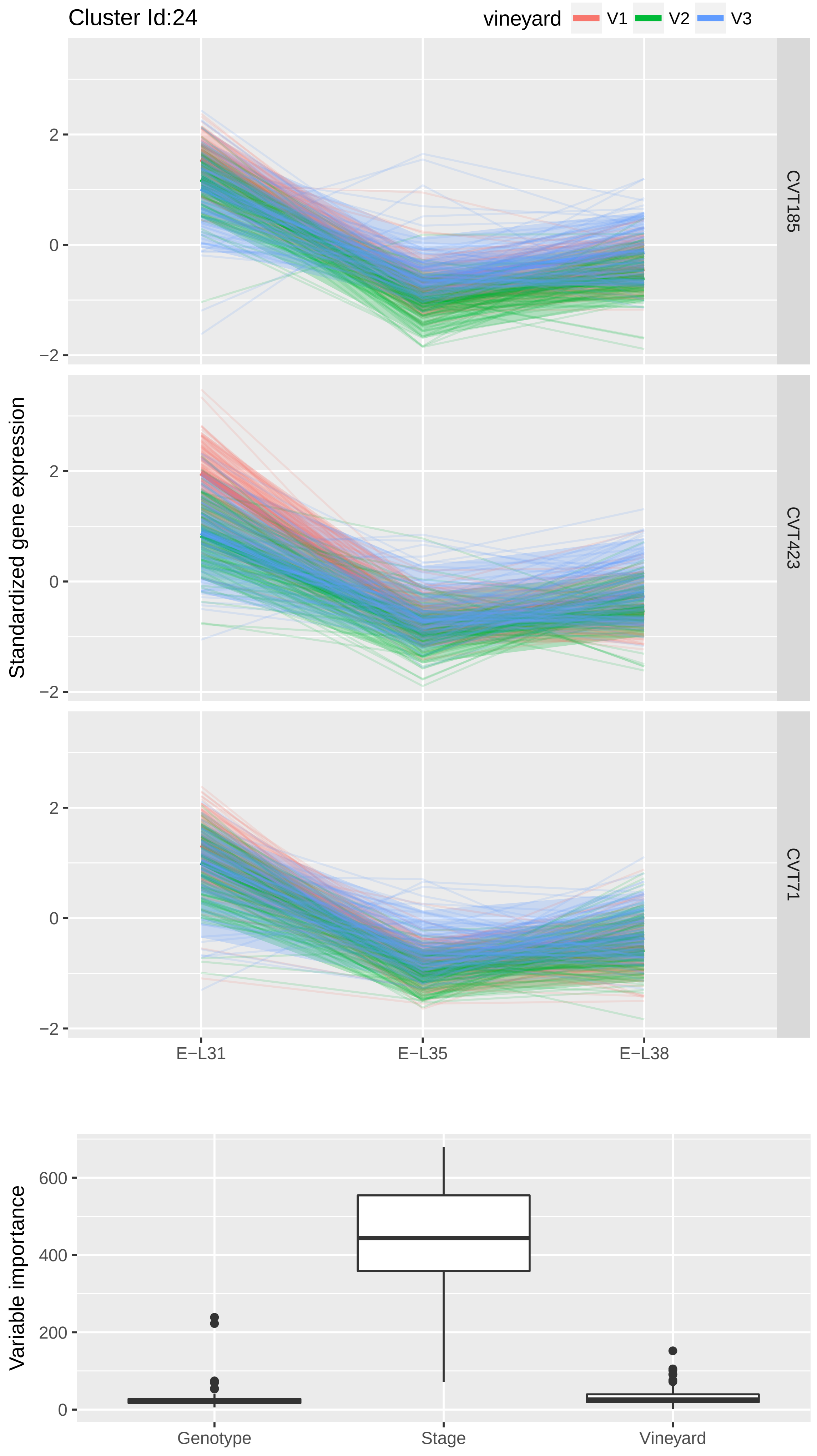

Cluster Id:24
vineyard
V1
V2
V3
Standardized gene expression
2
0
−2
CVT185
CVT423
CVT71
E−L31
E−L35
E−L38
Variable importance
600
400
200
0
Genotype
Stage
Vineyard

Cluster no. 25

```
## Number of genes in the cluster: 165
## Homogeneity Index:     0.8
## Variable importance for Stage:    Median =  501.1  - Rank =  33
## Variable importance for Clone:    Median =  10.48  - Rank =  102
## Variable importance for Vineyard: Median =   32.2  - Rank =  63
##
## Gene ID                 Gene Annotation
## VIT_06s0004g06370  -  UDP-glucose: anthocyanidin 5,3-O-glucosyltransferase
## VIT_03s0088g00200  -  Unknown
## VIT_13s0158g00370  -  RNA recognition motif (RRM)-containing CCCH
## VIT_09s0018g00290  -  Unknown protein
## VIT_07s0031g01470  -  Constitutive expression of PR genes 5 (CPR5)
## VIT_18s0001g11590  -  Dual-specific kinase DSK1
## VIT_13s0019g02380  -  Exostosin
## VIT_06s0004g06140  -  Ribosomal protein L4
## VIT_18s0117g00030  -  No hit
## VIT_19s0085g01190  -  ABC Transporter (VvPMP1 - VvABCD1)
## VIT_05s0094g00920  -  formin protein AHF1
## VIT_18s0001g06500  -  Unknown protein
## VIT_11s0037g00850  -  Tetratricopeptide repeat (TPR)-containing
## VIT_00s0291g00060  -  Inorganic phosphate transporter 2-1, chloroplast precursor
## VIT_04s0079g00680  -  phytoene synthase (PSY) (VvPSY1)
## VIT_07s0005g01590  -  Serine/threonine-specific protein kinase
## VIT_18s0041g01470  -  TIR-NBS-LRR-TIR disease resistance protein
## VIT_13s0047g01200  -  Obtusifoliol 14-demethylase (CYP51)
## VIT_09s0002g05040  -  RPS5 (resistant to p. syringae 5)
## VIT_09s0002g04910  -  R protein  PRF  disease resistance protein
## VIT_02s0012g01870  -  No hit
## VIT_02s0025g00340  -  Monodehydroascorbate reductase, chloroplast precursor
## VIT_08s0007g00940  -  LHW (lonesome highway)
## VIT_09s0002g05870  -  RPS5 (resistant to p. syringae 5)
## VIT_11s0037g00160  -  Protein prenyltransferase, alpha subunit
## VIT_07s0031g02810  -  Tubulin binding
## VIT_14s0030g01760  -  Unknown protein
## VIT_13s0019g03890  -  Calmodulin binding protein
## VIT_11s0016g00160  -  forminy 2 domain-containing protein
## VIT_12s0034g01090  -  Lung seven transmembrane receptor
## VIT_09s0002g05850  -  Disease resistance protein (NBS-LRR class)
## VIT_19s0015g01550  -  Phenylalanine-tRNA ligase
## VIT_09s0002g04880  -  Glycosyl transferase family 1 protein
## VIT_12s0134g00560  -  Unknown protein
## VIT_19s0090g00530  -  Geranyl diphosphate synthase small subunit
## VIT_15s0048g01400  -  Protein kinase
## VIT_10s0003g00050  -  Squamosa promoter-binding protein (VvSBP9)
## VIT_02s0154g00500  -  VAP27-1 (VAMP/synaptobrevin-associated protein 27-1)
## VIT_00s1206g00010  -  Aspartic proteinase nepenthesin-1 precursor
## VIT_18s0001g09450  -  Unknown protein
## VIT_08s0007g07230  -  VvMyb5a
## VIT_09s0018g01780  -  ACT domain repeat 1 (ACR1) uridylyltransferase
## VIT_08s0040g01140  -  Serine carboxypeptidase K10B2.2 precursor
## VIT_18s0117g00050  -  R protein L6
## VIT_06s0004g06110  -  Exo-1,3-beta-glucanase
## VIT_18s0001g13570  -  Calcium ion binding protein
## VIT_10s0071g01160  -  ferredoxin-thioredoxin reductase
```

```
## VIT_09s0002g00920  -  Lung seven transmembrane receptor
## VIT_11s0016g02430  -  Dual-specific kinase DSK1
## VIT_07s0005g06200  -  RPM1 (resistance to p. syringae pv maculicola 1)
## VIT_09s0002g05050  -  RPS5 (resistant to p. syringae 5)
## VIT_17s0000g10260  -  AarF domain containing kinase
## VIT_19s0015g01650  -  Phenylalanine-tRNA ligase
## VIT_05s0020g01310  -  SRG1 (senescence-related gene 1)
## VIT_04s0008g01910  -  ferredoxin-related
## VIT_00s1306g00020  -  No hit
## VIT_17s0000g05550  -  Proton-dependent oligopeptide transport (POT) family protein
## VIT_01s0026g02580  -  Cycling DOF factor 2
## VIT_07s0005g01660  -  Pentatricopeptide (PPR) repeat-containing protein
## VIT_09s0018g00410  -  Cyclic nucleotide gated channel 5
## VIT_14s0083g00420  -  TRN2 (TORNADO 2)
## VIT_13s0158g00300  -  No hit
## VIT_11s0016g04570  -  RKL1 (Receptor-like kinase 1)
## VIT_00s0229g00100  -  Unknown
## VIT_16s0050g01860  -  SLAH3 (SLAC1 Homologue 3)
## VIT_06s0061g00150  -  Zinc finger (CCCH-type) family protein
## VIT_04s0069g01000  -  Drug/metabolite transporter DMT family transporter
## VIT_09s0002g00790  -  Beta-ketoacyl-ACP synthase II
## VIT_09s0002g04890  -  R protein  PRF  disease resistance protein
## VIT_08s0007g06820  -  Kinesin motor protein
## VIT_19s0090g01280  -  Lipid-binding serum glycoprotein family protein
## VIT_08s0007g06170  -  Anthranilate phosphoribosyltransferase
## VIT_01s0011g03000  -  No hit
## VIT_13s0084g00750  -  R protein MLA10
## VIT_06s0061g01370  -  Unknown protein
## VIT_08s0007g03970  -  Peroxiredoxin type 2
## VIT_18s0122g00830  -  PMI1 (plastid movement impaired1)
## VIT_01s0146g00070  -  11-beta-hydroxysteroid dehydrogenase
## VIT_19s0027g01010  -  Unknown protein
## VIT_07s0141g00540  -  Multi-copper oxidase (SKU5)
## VIT_00s1306g00010  -  COP1-interacting protein 7
## VIT_11s0037g00840  -  Tetratricopeptide repeat (TPR)-containing
## VIT_03s0038g02920  -  S-adenosyl-methionine-sterol-C-methyltransferase
## VIT_04s0008g03610  -  Xylan synthase
## VIT_10s0116g01740  -  ATP synthase gamma chain 1t (ATPC1)
## VIT_19s0014g00300  -  Alpha,alpha-trehalose-phosphate synthase
## VIT_00s0734g00030  -  Unknown protein
## VIT_08s0007g03990  -  Cellulose synthase CSLA09
## VIT_01s0026g01790  -  Calmodulin binding protein
## VIT_15s0048g01880  -  Armadillo/beta-catenin repeat
## VIT_05s0020g01970  -  Vesicle-associated membrane protein 27 VAP27-2
## VIT_16s0098g00770  -  Auxin-amidohydrolase precursor
## VIT_09s0002g06300  -  Unknown
## VIT_15s0048g01010  -  2'-hydroxy isoflavone/dihydroflavonol reductase
## VIT_13s0019g03210  -  Lectin-like protein
## VIT_01s0011g06150  -  Receptor protein kinase
## VIT_04s0008g03950  -  RD22
## VIT_12s0142g00340  -  Galacturonosyltransferase 15
## VIT_17s0000g08750  -  ATPase-like domain-containing
## VIT_08s0040g01030  -  Unknown protein
## VIT_18s0001g12000  -  Geranylgeranyl pyrophosphate synthetase 1, chloroplast precursor
## VIT_04s0008g06640  -  Endomembrane protein 70, TM4 ;
```

```
## VIT_03s0017g01810  -  far-red impaired response 1
## VIT_11s0016g04510  -  No hit
## VIT_03s0038g03660  -  Armadillo/beta-catenin repeat protein / U-box domain-containing
## VIT_01s0137g00290  -  Oxysterol binding protein
## VIT_18s0001g02200  -  8-oxoguanine-DNA glycosylase (OGG1)
## VIT_02s0025g01490  -  Proteasome 26S regulatory subunit (RPN8)
## VIT_07s0005g03570  -  Arabinose-5-phosphate isomerase
## VIT_13s0074g00690  -  ABC Transporter (VvPDR26 - VvABCG56)
## VIT_02s0025g00670  -  CAAX amino terminal protease
## VIT_02s0087g00170  -  Leucine-rich repeat transmembrane protein kinase
## VIT_02s0012g00580  -  Ankyrin protein kinase
## VIT_05s0020g04060  -  TIFY gene family (VvPPDI)
## VIT_18s0001g11970  -  Unknown protein
## VIT_00s0194g00210  -  KCO1 (two pore K channel)
## VIT_10s0003g04020  -  CBL- interacting  protein kinase 23 (VvCIPK04)
## VIT_00s0429g00040  -  Aspartic Protease (VvAP47)
## VIT_08s0056g00500  -  Unknown protein
## VIT_17s0000g04810  -  Auxin-independent growth promoter
## VIT_00s0256g00040  -  Ankyrin repeat protein
## VIT_04s0008g04040  -  RD22 [Vitis vinifera]
## VIT_11s0016g03220  -  RNA-directed RNA polymerase
## VIT_14s0036g01060  -  Unknown protein
## VIT_09s0054g01770  -  Oxidoreductase/ transcriptional repressor
## VIT_00s0375g00010  -  COP1-interacting protein 7
## VIT_00s0742g00030  -  Nucleotide-sugar transporter
## VIT_12s0134g00550  -  Equilibrative nucleoside transporter (ENT3)
## VIT_06s0004g00150  -  MYR1 (MYB-related protein 1)
## VIT_00s0174g00170  -  Myb family
## VIT_00s0771g00020  -  Unknown protein
## VIT_19s0015g01600  -  Phenylalanine-tRNA ligase
## VIT_00s0373g00010  -  Tubulin alpha-3 chain
## VIT_18s0001g08780  -  BSD domain-containing protein
## VIT_07s0031g01580  -  SKP1 interacting partner 6 (SKIP6)
## VIT_17s0000g08390  -  1-deoxy-D-xylulose 5-phosphate reductoisomerase, chloroplast
## VIT_06s0004g06520  -  Ammonium transporter 1, member 1 (AMT1.1)
## VIT_00s1322g00010  -  Tubulin alpha-3 chain
## VIT_01s0026g01780  -  Leucine-rich repeat transmembrane
## VIT_05s0077g01450  -  GATA transcription factor 16
## VIT_04s0044g00280  -  Transcription elongation factor
## VIT_05s0077g00830  -  Protein phosphatase 2C
## VIT_14s0068g01300  -  Serine palmitoyltransferase
## VIT_05s0020g04430  -  MPBQ/MSBQ methyltransferase 1
## VIT_08s0007g02800  -  Pro-X carboxypeptidase Lysosomal
## VIT_18s0001g08530  -  Unknown protein
## VIT_04s0023g02010  -  Hydrolase, alpha/beta fold
## VIT_04s0008g06170  -  Unknown protein
## VIT_16s0098g01290  -  Major Facilitator Superfamily protein
## VIT_16s0050g01590  -  UDP-glucose: anthocyanidin 5,3-O-glucosyltransferase
## VIT_09s0002g08420  -  Unknown
## VIT_11s0016g04870  -  Unknown protein
## VIT_17s0000g06230  -  Zinc finger (C3HC4-type ring finger)
## VIT_18s0001g12530  -  Retrotransposon protein, Unclassified
## VIT_06s0080g00750  -  Unknown protein
## VIT_05s0020g02080  -  Zinc finger (C3HC4-type ring finger)
## VIT_06s0004g05780  -  UDP-glucoronosyl and UDP-glucosyl transferase
```

```
## VIT_15s0048g02420  -  No hit
## VIT_00s2015g00020  -  F-box family protein
## VIT_17s0000g03240  -  Inositol polyphosphate multikinase
## VIT_18s0166g00130  -  Substrate carrier, Mitochondrial
## VIT_09s0002g04950  -  RPS5 (resistant to p. syringae 5)
## VIT_03s0063g01910  -  Integral membrane protein
## VIT_10s0116g01760  -  Myb caprice CPC
## VIT_08s0007g08540  -  Mg-chelatase subunit XANTHA-F
```

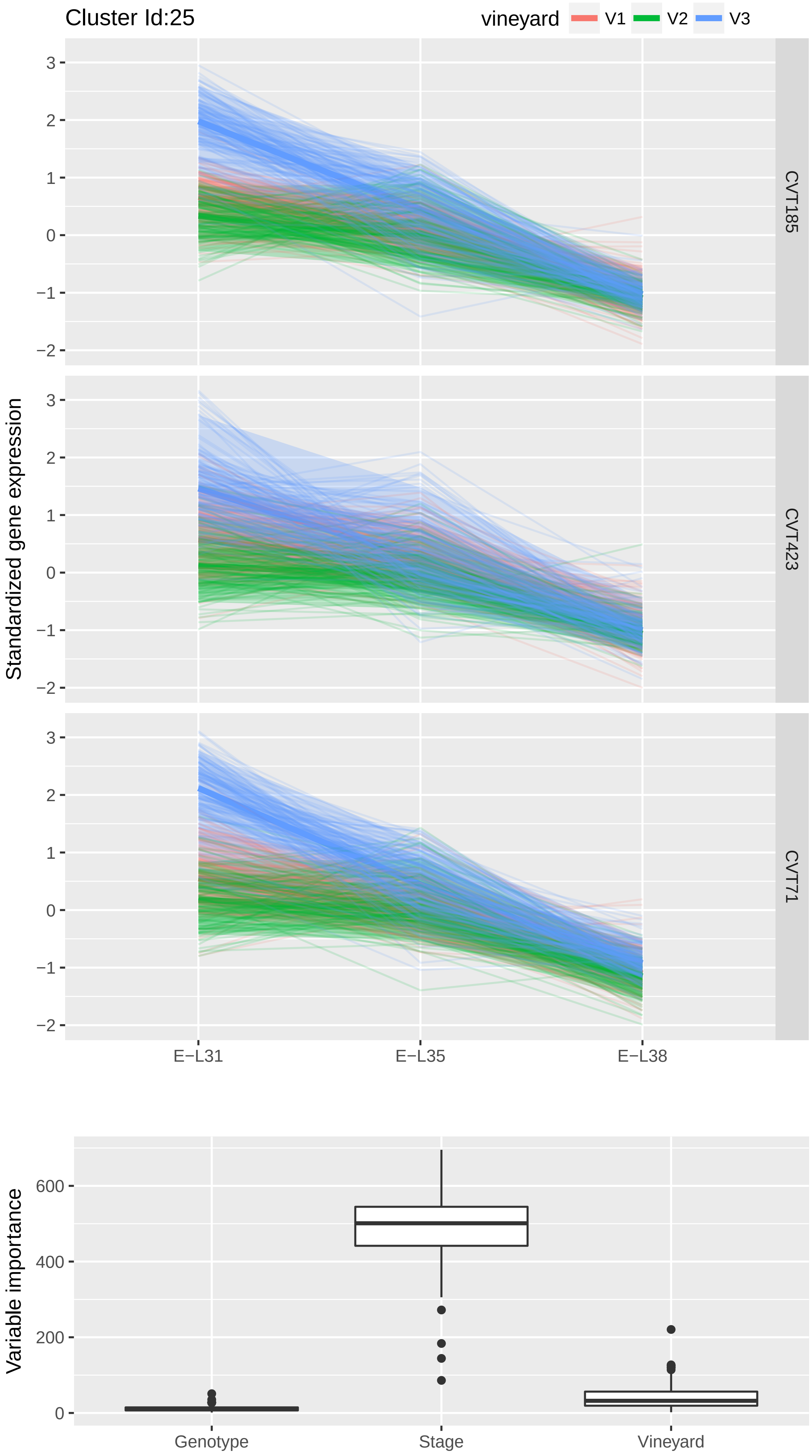

Cluster Id:25
vineyard V1 V2 V3
Standardized gene expression
3
2
1
0
−1
−2
CVT185
CVT423
CVT71
E−L31
E−L35
E−L38
Variable importance
600
400
200
0
Genotype
Stage
Vineyard

Cluster no. 26

```
## Number of genes in the cluster: 204
## Homogeneity Index:    0.66
## Variable importance for Stage:    Median =  212.2  - Rank =  85
## Variable importance for Clone:    Median =   24.1  - Rank =  27
## Variable importance for Vineyard: Median =  52.68  - Rank =  39
##
## Gene ID               Gene Annotation
## VIT_17s0000g02360  -  Receptor protein kinase
## VIT_00s0317g00050  -  Dehydroascorbate reductase
## VIT_06s0004g06010  -  Transformer serine/arginine-rich ribonucleoprotein
## VIT_08s0007g07380  -  Molecular chaperone DnaJ
## VIT_00s0670g00010  -  T-complex protein 1 subunit eta
## VIT_18s0001g14850  -  Unknown protein
## VIT_07s0005g00030  -  Glutathione S-transferase (VvGST2)
## VIT_03s0063g00960  -  Unknown protein
## VIT_01s0011g05050  -  Drug/metabolite transporter DMT family transporter
## VIT_00s0323g00020  -  HSP associated protein
## VIT_13s0064g01360  -  DnaJ homolog, subfamily A, member 3
## VIT_01s0146g00150  -  BCL-2-associated athanogene 5
## VIT_05s0049g01000  -  Unknown protein
## VIT_05s0051g00340  -  Chaperonin GroEL
## VIT_09s0070g00850  -  ATP binding
## VIT_15s0048g00530  -  Auxin-responsive SAUR11
## VIT_16s0013g00350  -  Heat shock protein binding
## VIT_14s0128g00400  -  Ras homolog gene family, member T1
## VIT_05s0049g01520  -  Cyanate hydratase
## VIT_13s0158g00170  -  R protein MLA10
## VIT_06s0004g04060  -  Legumin
## VIT_07s0151g00660  -  Ribosomal protein L24 (At5g23535) 50S
## VIT_18s0001g05220  -  WD-40 repeat
## VIT_04s0210g00200  -  Golgi-localized GRIP domain-containing protein
## VIT_14s0083g00220  -  Zinc finger CCHC domain-containing protein 8
## VIT_12s0035g00770  -  Nitrogen fixation NifU
## VIT_06s0009g03270  -  No hit
## VIT_06s0004g02840  -  Ribosomal protein L12 (RPL12C) 60S
## VIT_12s0057g00670  -  Heat shock protein 90C
## VIT_14s0171g00220  -  Ribosome recycling factor
## VIT_18s0001g12960  -  Wound-responsive protein
## VIT_00s2562g00020  -  Ubiquitin-specific protease 23 (UBP23)
## VIT_18s0001g13310  -  GTP1/OBG family protein
## VIT_05s0020g04620  -  basic helix-loop-helix (bHLH) family
## VIT_07s0005g04290  -  MRNA splicing protein (Prp39)
## VIT_00s0647g00010  -  WD40
## VIT_07s0141g00170  -  Basic Leucine Zipper Transcription Factor (VvbZIP21)
## VIT_12s0142g00200  -  Ribosomal protein L1 60S
## VIT_17s0000g00880  -  Pseudouridine synthase
## VIT_12s0034g01820  -  Pentatricopeptide repeat-containing
## VIT_16s0039g01690  -  TORMOZembryo defective UTP13
## VIT_03s0063g00380  -  Abscisic acid 8` hydroxylase (CYP707A4) (VvA8H-CYP707A4)
## VIT_01s0011g01980  -  fasciclin arabinogalactan-protein (FLA21)
## VIT_00s0264g00030  -  Aconitate hydratase, cytoplasmic
## VIT_12s0057g00380  -  Exopolygalacturonase
## VIT_01s0010g00680  -  Heat shock protein 81-1
## VIT_18s0001g07780  -  ABA-responsive protein (HVA22a)
```

```
## VIT_01s0011g01690  -  Zinc finger (ZPR1-type) family protein
## VIT_15s0021g02450  -  Elongation factor G
## VIT_00s0198g00020  -  ATP binding
## VIT_00s1405g00010  -  WD40
## VIT_19s0014g04710  -  DAG protein, chloroplast precursor
## VIT_08s0105g00550  -  Electron transport SCO1/SenC
## VIT_07s0151g00870  -  PHD finger transcription factor
## VIT_02s0025g01290  -  Esterase
## VIT_04s0023g00170  -  Unknown
## VIT_11s0016g05140  -  U4/U6 small nuclear ribonucleoprotein SNU13
## VIT_05s0020g03600  -  CDPK adapter protein 1
## VIT_07s0130g00160  -  No hit
## VIT_00s0174g00260  -  Ubiquitin-specific protease 23 (UBP23)
## VIT_09s0002g07870  -  Dihydrouridine synthase 2
## VIT_07s0191g00040  -  NOL1/NOP2/sun
## VIT_18s0001g12120  -  Basic Leucine Zipper Transcription Factor (VvbZIP46)
## VIT_08s0007g01670  -  Ribosomal protein L35a (RPL35aC) 60S
## VIT_12s0035g02290  -  Nodulin
## VIT_01s0011g00390  -  Asparagine synthase
## VIT_17s0000g05990  -  ABA-responsive protein (HVA22a)
## VIT_14s0171g00490  -  Glucose-6-phosphate 1-dehydrogenase, cytoplasmic isoform
## VIT_02s0025g04500  -  Ran-binding protein 1 RanBP1 domain containing
## VIT_06s0009g03660  -  Protein kinase
## VIT_18s0122g00320  -  PLATZ transcription factor
## VIT_05s0049g01880  -  Unknown protein
## VIT_12s0034g01000  -  Ribosomal protein P0 acidic
## VIT_17s0000g01640  -  Metallo-beta-lactamase EMB2746
## VIT_05s0077g00740  -  Prefoldin subunit 3
## VIT_10s0003g01920  -  RKF1 (receptor-like kinase in flowers 1)
## VIT_01s0127g00580  -  Subtilisin-like serine protease 2
## VIT_06s0004g00810  -  Unknown protein
## VIT_14s0068g00970  -  RPK1 (receptor-like protein kinase 1)
## VIT_09s0002g02240  -  Chaperonin GroEL
## VIT_19s0027g01920  -  EIF4-gamma/eIF5/eIF2-epsilon domain-containing protein
## VIT_14s0081g00700  -  Peptidyl-prolyl cis-trans isomerase A (cyclophilin A)
## VIT_06s0061g01440  -  Unknown protein
## VIT_05s0094g00170  -  Protein transport protein Sec61 subunit beta
## VIT_05s0062g01200  -  Unknown
## VIT_06s0004g05220  -  Serine/arginine repetitive matrix protein 1
## VIT_01s0011g02060  -  CYP86B1
## VIT_01s0011g01680  -  Chaperonin GroEL
## VIT_13s0084g00600  -  DNA-directed RNA polymerase I subunit A2
## VIT_13s0064g00850  -  Ribosomal protein L30 (RPL30B) 60S
## VIT_18s0076g00100  -  Pentatricopeptide repeat-containing protein
## VIT_17s0000g06240  -  Unknown protein
## VIT_00s0586g00010  -  WD40
## VIT_13s0019g02070  -  Phosphoribosylformylglycinamidine synthase, chloroplast precursor
## VIT_11s0052g00830  -  Vernalization insensitive 3
## VIT_17s0000g09390  -  Ribosomal protein L19
## VIT_11s0016g03530  -  DNA-directed RNA polymerase II subunit E
## VIT_17s0000g00140  -  TBP-binding protein
## VIT_12s0028g00920  -  Glutathione S-transferase 9 GSTF9
## VIT_05s0102g01150  -  F-box domain containing protein
## VIT_01s0010g02410  -  Unknown protein
```

```
## VIT_07s0104g00770  -  Methyl-CpG-binding domain-containing protein
## VIT_09s0002g06460  -  Unknown protein
## VIT_12s0035g01160  -  Leucyl-tRNA synthetase
## VIT_16s0039g02220  -  Exportin1 (XPO1)
## VIT_15s0021g00360  -  Pentatricopeptide repeat-containing protein
## VIT_14s0060g00340  -  Unknown protein
## VIT_11s0016g01380  -  Ribosomal protein S3 (RPS3C) 40S
## VIT_06s0004g08490  -  Defective IN RNA-directed DNA methylation 1 DRD1
## VIT_05s0020g00470  -  DEAD box RNA helicase 1
## VIT_18s0089g00370  -  Carboxyesterase18 CXE18
## VIT_07s0095g00710  -  Bystin
## VIT_17s0000g08020  -  Unknown protein
## VIT_13s0047g00520  -  Unknown protein
## VIT_00s1231g00010  -  Seryl-tRNA synthetase
## VIT_16s0013g01530  -  U3 small nucleolar RNA-associated protein 10
## VIT_08s0217g00020  -  DNA-binding protein GBP16
## VIT_15s0048g00270  -  Scarecrow transcription factor 6 (SCL6)
## VIT_19s0014g03540  -  No hit
## VIT_09s0002g08070  -  Prephenate dehydrogenase
## VIT_08s0040g00210  -  L-2-hydroxyglutarate dehydrogenase
## VIT_05s0020g00940  -  DNA helicase SNF2 domain-containing protein
## VIT_00s0231g00050  -  ATP binding protein
## VIT_18s0122g00880  -  Channel forming outer membrane protein, Chloroplast
## VIT_00s0337g00080  -  Unknown protein
## VIT_16s0050g02580  -  Unknown protein
## VIT_11s0065g01210  -  Zinc finger (C3HC4-type ring finger)
## VIT_18s0001g02690  -  Unknown protein
## VIT_08s0105g00570  -  Adenosine/AMP deaminase active site
## VIT_12s0142g00590  -  R protein L6
## VIT_06s0080g00900  -  Leucine-rich repeat
## VIT_14s0066g01630  -  UVH3 (ultraviolet hypersensitive 3)
## VIT_08s0007g04690  -  U3 small nucleolar RNA-associated protein 12
## VIT_00s0475g00040  -  myb family
## VIT_17s0000g04650  -  Phosphoribulokinase/uridine kinase
## VIT_18s0001g01670  -  Zinc finger (CCCH-type) family protein
## VIT_17s0000g06600  -  No hit
## VIT_04s0008g00760  -  Thioredoxin Trp26
## VIT_05s0020g02570  -  Unknown protein
## VIT_14s0108g01120  -  SPX (SYG1/Pho81/XPR1) domain-containing protein
## VIT_04s0008g04470  -  Zinc finger, SWIM-type
## VIT_15s0048g02770  -  GIF3 (GRF1- interacting  factor 3)
## VIT_15s0046g01840  -  Transmembrane protein
## VIT_06s0004g01630  -  Transcription factor jumonji (jmjC) domain-containing protein
## VIT_09s0070g00660  -  No hit
## VIT_13s0019g00700  -  Unknown protein
## VIT_05s0062g00120  -  Ribosomal protein S8 (RPS8B) 40S
## VIT_17s0000g10180  -  Coproporphyrinogen oxidase
## VIT_07s0005g01530  -  ATP-dependent Clp protease proteolytic subunit (ClpP1)
## VIT_16s0098g01320  -  Armadillo repeat-containing protein
## VIT_05s0020g01290  -  TOC159 (translocon outer membrane complex 159)
## VIT_11s0016g04270  -  Hydrolase, alpha/beta fold
## VIT_18s0001g01560  -  Tetratricopeptide repeat protein 33
## VIT_13s0067g02260  -  Pentatricopeptide (PPR) repeat-containing protein
## VIT_18s0001g07750  -  Sas10/U3 ribonucleoprotein
## VIT_07s0104g01610  -  AAA-type ATPase PEX6
```

```
## VIT_04s0023g02460  -  Transducin protein
## VIT_08s0056g01710  -  Cationic amino acid transporter 2
## VIT_13s0019g00630  -  Unknown protein
## VIT_09s0002g04000  -  Ribonuclease Z
## VIT_07s0141g00530  -  U3 small nucleolar RNA-associated protein 18
## VIT_03s0063g01030  -  Peptidyl-prolyl isomerase D (cyclophilin D)
## VIT_07s0005g04550  -  Brix domain-containing protein
## VIT_06s0004g02990  -  GRIK2 (geminivirus rep  interacting  kinase 2)
## VIT_08s0007g07810  -  basic helix-loop-helix (bHLH) family
## VIT_05s0077g01000  -  ATP binding protein
## VIT_08s0007g08870  -  Unknown
## VIT_11s0016g02700  -  IAA-amino acid hydrolase 2 (ILL2)
## VIT_14s0006g01980  -  Zinc finger (C3HC4-type ring finger)
## VIT_17s0000g07740  -  HhH-GPD base excision DNA repair protein
## VIT_06s0009g01750  -  ATP-dependent RNA helicase DOB1
## VIT_07s0005g04190  -  Histone-lysine N-methyltransferase SUVR4
## VIT_03s0097g00670  -  SEC6
## VIT_09s0070g00440  -  No hit
## VIT_05s0029g01300  -  MAGE (melanoma antigen-encoding gene)
## VIT_13s0019g01070  -  Radical SAM domain-containing protein
## VIT_16s0050g00880  -  RRNA 2'-O-methyltransferase fibrillarin
## VIT_04s0008g00790  -  Ribosomal protein L28 (RPL28C) 60S
## VIT_01s0011g02020  -  2-oxo acid dehydrogenase, lipoyl-binding site
## VIT_08s0040g00310  -  Zinc finger (C3HC4-type ring finger)
## VIT_11s0016g03910  -  Unknown protein
## VIT_17s0000g02030  -  Molecular chaperone DnaJ
## VIT_01s0011g00940  -  No hit
## VIT_15s0048g00610  -  Unknown
## VIT_04s0008g01670  -  PHD finger protein alfin
## VIT_16s0100g00520  -  ATPP2-A13
## VIT_11s0016g05100  -  Unknown protein
## VIT_01s0026g01630  -  Unknown protein
## VIT_14s0066g00140  -  RNA-binding protein RBP31
## VIT_01s0011g05620  -  No hit
## VIT_13s0067g03190  -  SNF2/SWI2 family global transcription factor
## VIT_10s0003g02210  -  Pentatricopeptide (PPR) repeat-containing protein
## VIT_09s0002g04060  -  Transducin protein
## VIT_05s0051g00330  -  F-box domain containing protein
## VIT_07s0005g05670  -  Alba, DNA binding
## VIT_10s0597g00040  -  Pentatricopeptide (PPR) repeat-containing protein
## VIT_12s0034g01030  -  R protein MLA10
## VIT_02s0025g04190  -  DEAD-box ATP dependent DNA helicase
## VIT_01s0010g00690  -  DNA-directed RNA polymerase
## VIT_08s0040g00200  -  FAR1-related sequence 3
## VIT_06s0009g03290  -  Pre-mRNA branch site protein p14
## VIT_18s0001g09050  -  ATP binding domain 1 , member B
## VIT_06s0004g02900  -  Hydroxymethylbutenyl 4-diphosphate synthase
## VIT_13s0074g00590  -  Unknown protein
```

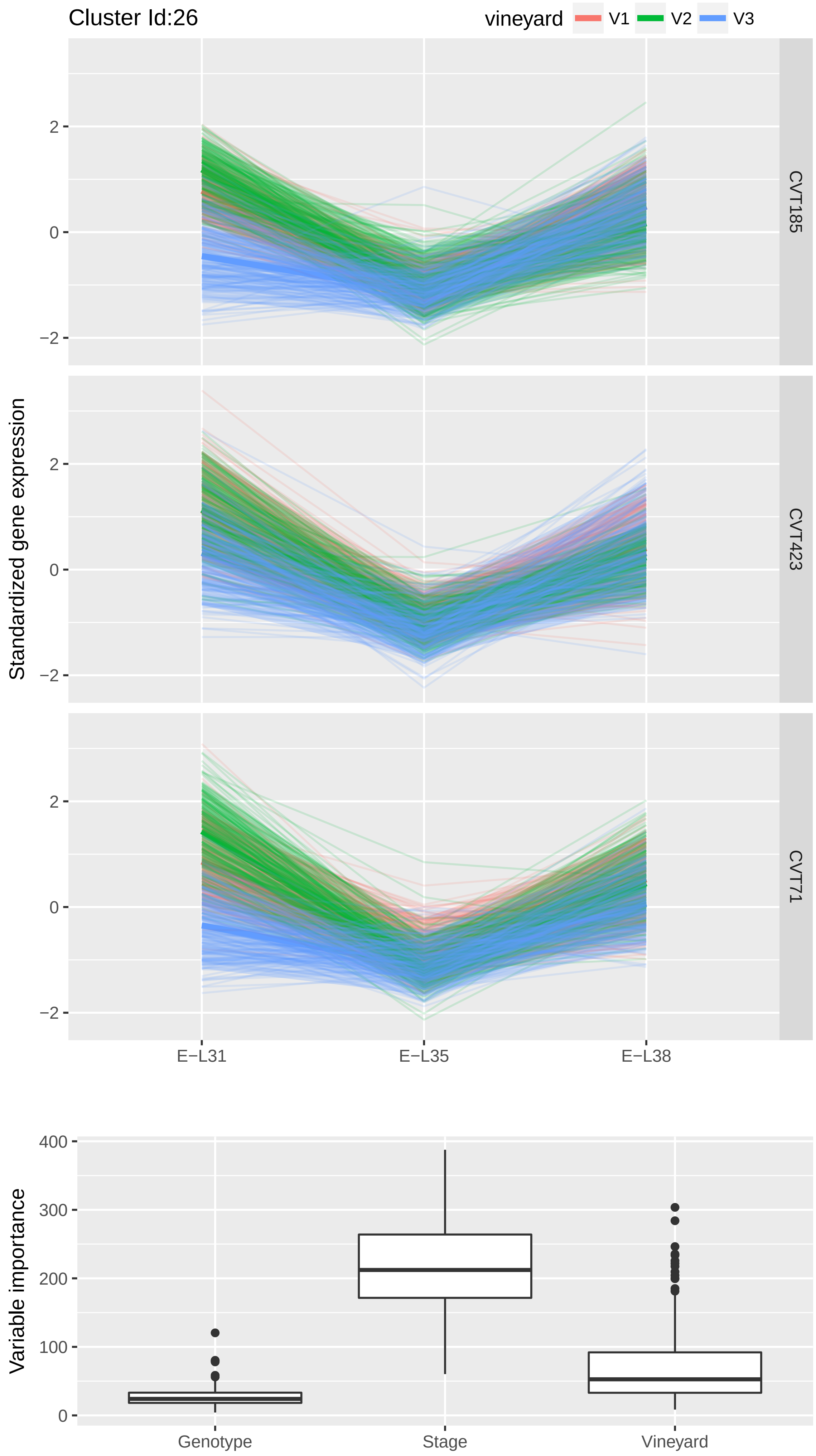

Cluster Id:26
vineyard
V1
V2
V3
CVT185
CVT423
CVT71
Standardized gene expression
2
0
−2
E−L31
E−L35
E−L38
Variable importance
400
300
200
100
0
Genotype
Stage
Vineyard

Cluster no. 27

```
## Number of genes in the cluster: 126
## Homogeneity Index:    0.61
## Variable importance for Stage:    Median =    237  - Rank =  81
## Variable importance for Clone:    Median =  28.08  - Rank =  12
## Variable importance for Vineyard: Median =  39.21  - Rank =  49
##
## Gene ID               Gene Annotation
## VIT_01s0011g06290  -  Purple acid phosphatase 3 ATPAP3/PAP3
## VIT_14s0036g01020  -  Polyubiquitin (UBQ4)
## VIT_00s0187g00010  -  No hit
## VIT_04s0008g06230  -  Ubiquitin family
## VIT_01s0113g00430  -  Mitotic checkpoint protein BUB3
## VIT_02s0033g01400  -  Alternative oxidase 1a, (AOX1A)
## VIT_04s0043g00400  -  Exosome complex component CSL4
## VIT_05s0029g01230  -  Unknown protein
## VIT_16s0100g00340  -  CDPK
## VIT_03s0038g04760  -  basic helix-loop-helix (bHLH) family
## VIT_18s0001g01740  -  Histone-lysine N-methyltransferase ASHH2 EFS (Early flowering in short
## VIT_13s0074g00700  -  ABC Transporter (VvPDR27 - VvABCG57)
## VIT_02s0025g03010  -  Copper chaperone (CCH)
## VIT_03s0097g00140  -  No hit
## VIT_14s0128g00820  -  Polymerase zeta
## VIT_11s0037g00490  -  Translation initiation factor eIF-4F
## VIT_04s0008g06770  -  Translation initiation factor eIF-4F
## VIT_04s0008g06210  -  Nodulin
## VIT_14s0068g01050  -  GCN5 N-acetyltransferase (GNAT)
## VIT_15s0046g02050  -  Unknown protein
## VIT_01s0010g03980  -  Unknown protein
## VIT_04s0044g01800  -  RNA polymerase I specific transcription initiation factor RRN3
## VIT_08s0007g02810  -  ERF/AP2 Gene Family (VvRAV5)
## VIT_12s0034g01410  -  Rhomboid
## VIT_07s0141g00220  -  Unfertilized embryo sac 12 UNE12
## VIT_11s0103g00230  -  Unknown protein
## VIT_14s0030g01520  -  Cysteine synthase [Vitis pseudoreticulata]
## VIT_06s0004g02670  -  Cyclic nucleotide-gated ion channel 15
## VIT_18s0001g02680  -  BTB/POZ domain-containing protein
## VIT_05s0020g00450  -  DNA methyltransferase
## VIT_15s0046g01030  -  Zinc finger (CCCH-type/C3HC4-type ring finger) family protein
## VIT_01s0026g00140  -  RNA-binding protein 25
## VIT_01s0011g04560  -  S locus-linked protein SLL2
## VIT_01s0011g04590  -  Zinc finger (C3HC4-type ring finger)
## VIT_16s0100g00370  -  Valyl-tRNA synthetase
## VIT_11s0065g00950  -  CBL-interacting protein kinase 23
## VIT_12s0059g02650  -  Basic helix-loop-helix ILR3
## VIT_00s0216g00030  -  Nuclear transport factor 2 (NTF2)
## VIT_02s0033g01390  -  No hit
## VIT_03s0017g00620  -  GA 20-oxidase
## VIT_15s0048g00620  -  No hit
## VIT_13s0067g00230  -  Extosin ectopically parting cells
## VIT_14s0036g01160  -  No hit
## VIT_18s0001g12430  -  Unknown protein
## VIT_05s0102g00560  -  Peptidyl-prolyl cis-trans isomerase E
## VIT_07s0005g01170  -  Phosphoribosylformylglycinamidine cyclo-ligase,chloroplast/precursor
## VIT_07s0141g00760  -  Unknown protein
```

```
## VIT_11s0016g00830  -  Ribose isomerase
## VIT_11s0052g00070  -  DNA ligase IV
## VIT_14s0068g02130  -  fidgetin-like 1
## VIT_05s0062g00220  -  Inner membrane import protein Tic22, Chloroplast
## VIT_09s0002g02700  -  basic helix-loop-helix (bHLH) family
## VIT_16s0098g01020  -  Splicing factor, arginine/serine-rich 1/9
## VIT_12s0034g01810  -  2OG-Fe(II) oxygenase
## VIT_03s0017g01480  -  Integral membrane Yip1 family protein
## VIT_04s0008g03330  -  Nucleoporin nup85
## VIT_07s0005g00200  -  AAA-type ATPase
## VIT_05s0020g02620  -  RNA recognition motif (RRM)-containing
## VIT_11s0052g00870  -  IAA33
## VIT_01s0137g00310  -  1-aminocyclopropane-1-carboxylate deaminase
## VIT_00s0515g00030  -  Unknown protein
## VIT_00s0302g00060  -  No hit
## VIT_05s0049g01840  -  Pre-mRNA splicing factor 19
## VIT_09s0002g07130  -  CUE domain containing protein
## VIT_10s0003g03930  -  Inositol transporter 2
## VIT_14s0081g00630  -  Ethylene receptor (EIN4)
## VIT_18s0041g01930  -  Pentatricopeptide (PPR) repeat-containing protein
## VIT_08s0040g02410  -  Ribonuclease P protein subunit POP4
## VIT_17s0000g00600  -  GCN5 N-acetyltransferase (GNAT)
## VIT_18s0041g00050  -  No hit
## VIT_04s0023g01700  -  No hit
## VIT_05s0020g01780  -  Chromatin-remodeling protein 11
## VIT_12s0059g01940  -  Pentatricopeptide (PPR) repeat
## VIT_04s0023g01330  -  Homeobox protein 5 (VvATHB-6)
## VIT_05s0051g00170  -  RRS1 (resistant to ralstonia solanacearum 1)
## VIT_09s0002g04750  -  MAR binding filament-like protein 1 MFP1
## VIT_04s0023g02700  -  Thioredoxin H
## VIT_19s0085g00560  -  Pentatricopeptide (PPR) repeat-containing
## VIT_04s0023g01450  -  Splicing factor U2AF 65 kDa subunit
## VIT_07s0151g00730  -  Unknown
## VIT_18s0001g05590  -  CCR4-NOT transcription complex subunit 6
## VIT_19s0093g00560  -  Ubiquitin-specific protease 25 (UBP25)
## VIT_05s0094g00370  -  Diphthamide synthesis DPH2
## VIT_14s0030g01860  -  Transcription factor
## VIT_12s0055g00380  -  No hit
## VIT_08s0007g01960  -  FtsJ-like methyltransferase
## VIT_03s0038g02490  -  SKP1
## VIT_08s0007g06370  -  Photoperiod independent early flowering1 (PIE1)
## VIT_01s0011g06020  -  Small nuclear ribonucleoprotein D2
## VIT_16s0098g00360  -  CCT motif constans-like
## VIT_16s0050g02560  -  Cyclic nucleotide-regulated ion channel 1
## VIT_18s0001g01770  -  PTAC6 (plastid transcriptionally active6)
## VIT_01s0150g00050  -  GT2-like trihelix DNA-binding protein
## VIT_18s0001g13660  -  EMB2757/TAN (embryo defective 2757)
## VIT_06s0004g04100  -  ABC Transporter (VvMDR13 - VvABCB13)
## VIT_18s0041g02220  -  Unknown protein
## VIT_04s0008g00570  -  Glu-tRNA(Gln) amidotransferase subunit C
## VIT_14s0108g00510  -  Nucleosome assembly protein (NAP-related) NRP2
## VIT_17s0000g06760  -  Unknown protein
## VIT_02s0087g00640  -  Unknown
## VIT_18s0001g06920  -  TRNA-guanine transglycosylase
## VIT_14s0128g00810  -  Polymerase zeta
```

```
## VIT_08s0040g00520  -  Leucine-rich repeat protein kinase
## VIT_08s0056g00810  -  Co-chaperone grpE
## VIT_18s0001g14930  -  Unknown protein
## VIT_08s0007g08090  -  DNA-directed RNA polymerase I subunit A1
## VIT_18s0001g05340  -  Transcription termination factor mitochondrial mTERF
## VIT_08s0040g01600  -  TCP family transcription factor TCP11
## VIT_07s0031g02040  -  Nucleic acid binding
## VIT_08s0007g03130  -  Small nuclear ribonucleoprotein G
## VIT_18s0001g09930  -  Defective chloroplasts and leaves protein / DCL protein
## VIT_09s0054g00750  -  TRNA-splicing endonuclease positive effector SEN1
## VIT_12s0035g00300  -  Carbohydrate transmembrane transporter SFP1
## VIT_05s0062g01210  -  Pentatricopeptide (PPR) repeat-containing protein
## VIT_05s0102g01160  -  FPA (FPA)
## VIT_06s0080g01120  -  Unknown protein
## VIT_16s0050g02250  -  Pentatricopeptide (PPR) repeat
## VIT_09s0002g04160  -  Thioesterase family
## VIT_04s0008g01000  -  EIF-4A3
## VIT_00s0174g00320  -  Unknown protein
## VIT_15s0024g01510  -  Pentatricopeptide (PPR) repeat-containing protein
## VIT_03s0088g00500  -  TRNA synthetase class II (D, K and N)
## VIT_05s0049g01300  -  No hit
## VIT_16s0039g02270  -  Unknown protein
## VIT_03s0038g02070  -  TOC132
## VIT_09s0002g08110  -  Unknown
```

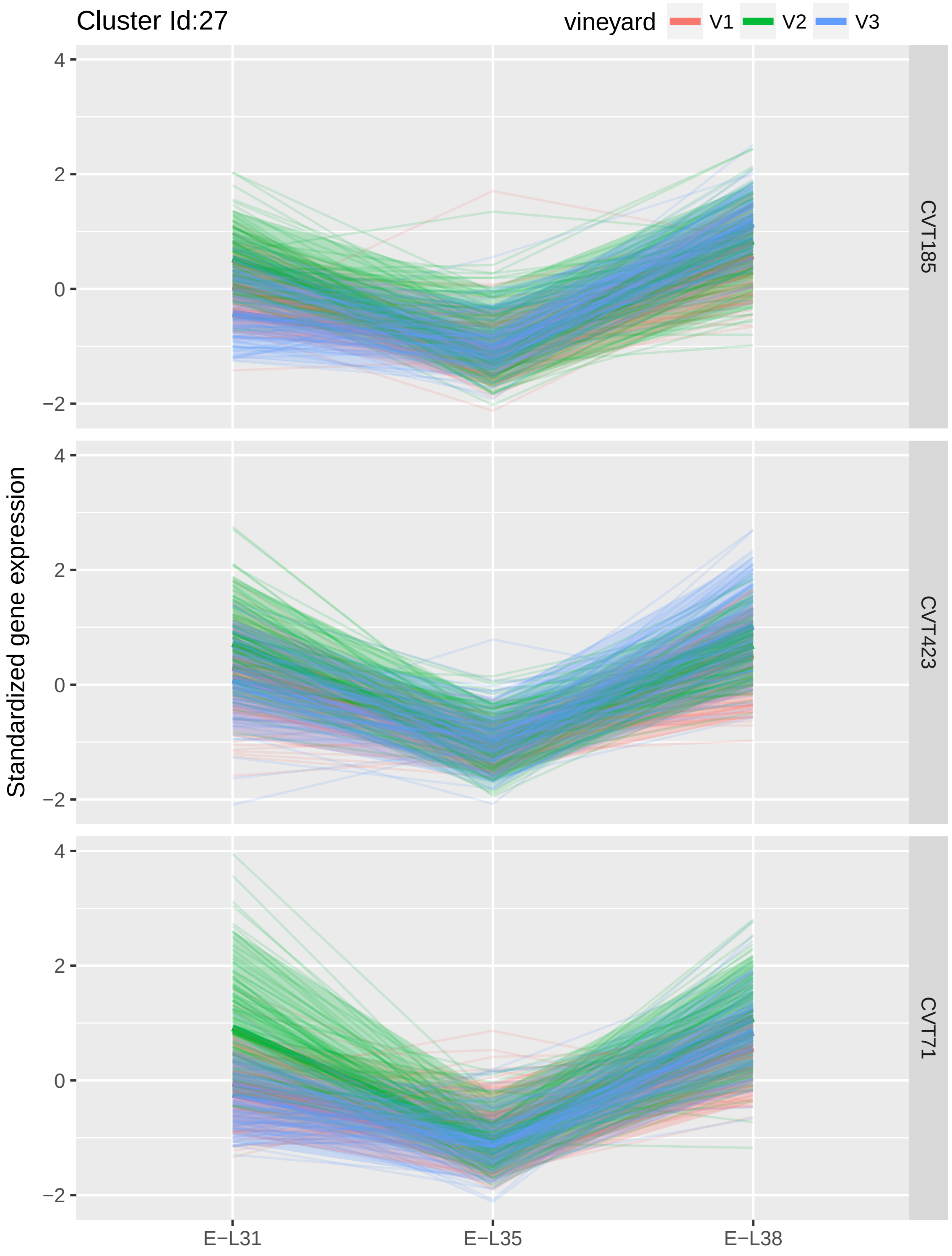
Cluster Id:27
vineyard
V1
V2
V3
Standardized gene expression
CVT185
CVT423
CVT71
4
2
0
−2
E−L31
E−L35
E−L38

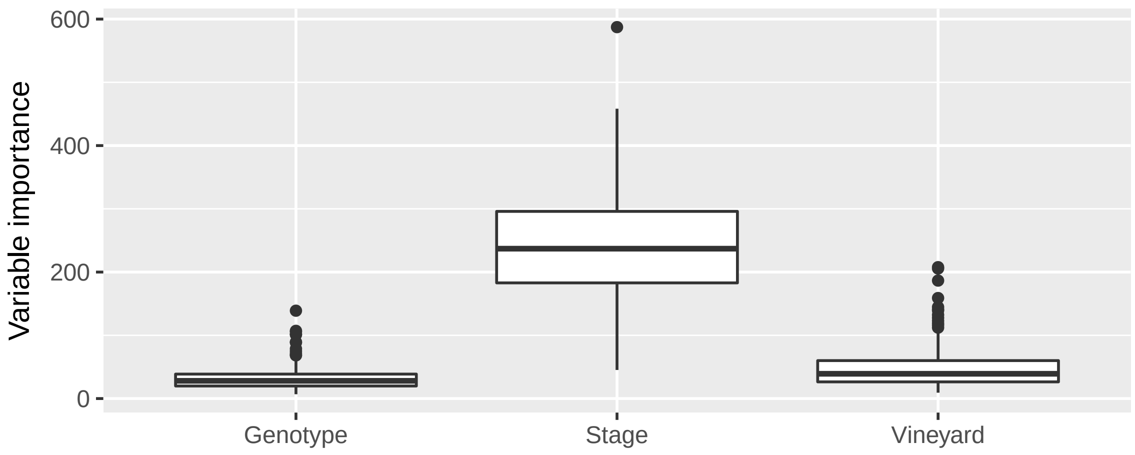
Variable importance
600
400
200
0
Genotype
Stage
Vineyard

Cluster no. 28

```
## Number of genes in the cluster: 183
## Homogeneity Index:    0.86
## Variable importance for Stage:    Median =    665  - Rank =  2
## Variable importance for Clone:    Median =   9.67  - Rank =  111
## Variable importance for Vineyard: Median =  13.69  - Rank =  103
##
## Gene ID               Gene Annotation
## VIT_02s0236g00150  -  calcineurin B 1 (VvCBL01-Like)
## VIT_07s0104g01150  -  RNA-binding protein
## VIT_00s0225g00100  -  Armadillo
## VIT_08s0007g01720  -  Reticulon family protein (RTNLB12)
## VIT_07s0005g06210  -  RPM1 (resistance to p. syringae pv maculicola 1)
## VIT_19s0014g05110  -  Unknown
## VIT_12s0028g01100  -  Aldo/keto reductase
## VIT_16s0115g00340  -  UDP-glucose: anthocyanidin 5,3-O-glucosyltransferase
## VIT_06s0004g04810  -  Protein kinase APK1A
## VIT_00s0259g00160  -  Acyl carrier protein, mitochondrial 3
## VIT_18s0122g00960  -  Glyceraldehyde-3-phosphate dehydrogenase B, chloroplast precursor
## VIT_02s0025g02130  -  Unknown
## VIT_07s0005g04710  -  Protein kinase
## VIT_02s0236g00140  -  calcineurin B 1 (VvCBL01)
## VIT_19s0090g01780  -  Zinc finger (CCCH-type) family protein
## VIT_16s0039g02530  -  No hit
## VIT_09s0018g01870  -  D-3-phosphoglycerate dehydrogenase, chloroplast precursor (3-PGDH)
## VIT_12s0142g00570  -  R protein disease resistance protein
## VIT_08s0007g05040  -  Unknown protein
## VIT_04s0008g00450  -  Haloacid dehalogenase hydrolase
## VIT_13s0073g00160  -  Unknown protein
## VIT_09s0002g01360  -  No hit
## VIT_13s0019g04870  -  Unknown protein
## VIT_13s0067g00080  -  COP1-interacting protein 7
## VIT_12s0134g00200  -  Methyltransferase
## VIT_12s0142g00220  -  Haloacid dehalogenase hydrolase
## VIT_09s0002g01650  -  5'-nucleotidase surE
## VIT_07s0005g04400  -  Photosystem II reaction centre W (PsbW)
## VIT_01s0010g02670  -  3-oxoacyl-[acyl-carrier-protein] reductase, chloroplast
## VIT_09s0002g06390  -  Peptide deformylase
## VIT_16s0013g01510  -  WD-repeat protein 8
## VIT_04s0044g00420  -  MTN21
## VIT_08s0007g08470  -  24-sterol C-methyltransferase
## VIT_07s0141g00200  -  Ribosomal protein L29 50S
## VIT_13s0084g00630  -  Unknown protein
## VIT_11s0037g00740  -  Serine carboxypeptidase S10
## VIT_06s0004g05900  -  Phosphopyruvate hydratase.
## VIT_05s0020g02970  -  RANGAP2 (RAN GTPASE activating protein 2)
## VIT_01s0026g00590  -  GCN5 N-acetyltransferase (GNAT)
## VIT_00s0174g00330  -  formin protein AHF1
## VIT_12s0057g00890  -  Glycosyl hydrolase family 17 protein
## VIT_07s0005g03960  -  Peptide transporter protein 3
## VIT_02s0012g01050  -  ABC protein 6 non-intrinsic
## VIT_15s0021g00530  -  Unknown protein
## VIT_12s0059g01050  -  No hit
## VIT_18s0001g01210  -  Senescence-inducible chloroplast stay-green protein 2
## VIT_14s0036g00480  -  Tyrosyl-tRNA synthetase
```

```
## VIT_15s0048g02190  -  PAP/fibrillin family
## VIT_02s0025g03060  -  Unknown protein
## VIT_18s0001g00090  -  Seed maturation protein PM23
## VIT_08s0007g02970  -  Unknown protein
## VIT_07s0104g00540  -  CYCP1;1; CYCP1;1
## VIT_07s0005g01210  -  VvMybF1
## VIT_00s0227g00200  -  formin protein AHF1
## VIT_13s0067g03420  -  RabGAP/TBC domain-containing protein
## VIT_11s0016g04850  -  Carboxylic ester hydrolase
## VIT_18s0001g04680  -  RPG related protein 1 RR1
## VIT_07s0031g02560  -  UVB-resistance protein UVR8
## VIT_11s0016g05400  -  Unknown protein
## VIT_08s0040g00390  -  Magnesium-protoporphyrin IX monomethyl ester [oxidative] cyclase
## VIT_07s0031g00320  -  Curly leaf PHCLF1
## VIT_11s0016g04500  -  Protein phosphatase 2 (formerly 2A), regulatory subunit B'
## VIT_00s1326g00010  -  Kinesin motor protein
## VIT_16s0022g01820  -  Microtubule associated protein (MAP65/ASE1) pleiade
## VIT_04s0044g01850  -  Auxin efflux carrier
## VIT_03s0038g00440  -  Unknown protein
## VIT_07s0129g00730  -  CYP81E1 Isoflavone 2'-hydroxylase
## VIT_15s0046g00220  -  Kinesin light chain
## VIT_12s0055g00490  -  Accumulation and replication of chloroplast 5
## VIT_01s0011g00550  -  Hydrolase, alpha/beta fold family
## VIT_05s0020g01210  -  No hit
## VIT_01s0010g02170  -  CAAX amino terminal protease
## VIT_18s0001g12020  -  Brassinosteroid Signaling positive regulator (BZR1)
## VIT_04s0044g00010  -  Unknown
## VIT_17s0000g05180  -  Unknown
## VIT_05s0020g01200  -  Kinesin family member 2/24
## VIT_00s0375g00050  -  No hit
## VIT_02s0012g00460  -  Glycine-rich protein
## VIT_04s0008g05120  -  Phospholipase/carboxylesterase
## VIT_17s0053g00090  -  Accumulation and replication of chloroplasts 6H
## VIT_14s0066g00220  -  Elongation factor EF-Tu RABE1B
## VIT_11s0103g00120  -  Phenylalanyl-tRNA synthetase
## VIT_00s0199g00260  -  GLABRA2 expression modulator
## VIT_05s0020g03170  -  Lipoxygenase
## VIT_09s0002g00150  -  Strubbelig receptor family 6
## VIT_10s0116g01540  -  Aminomethyltransferase
## VIT_07s0191g00030  -  RAB GTPase RABA4A
## VIT_18s0001g12660  -  TUBBY like protein 6 TLP6
## VIT_08s0007g04530  -  carotene hydroxylase (CYP97C1; LUT1) (VvLUT1)
## VIT_06s0004g01600  -  No hit
## VIT_19s0014g05210  -  Fertilization-independent endosperm development protein
## VIT_08s0032g01110  -  Axial regulator YABBY2
## VIT_08s0007g03920  -  DEMETER protein (DME)
## VIT_04s0008g01330  -  RTE1 (reversion-to-ethylene sensitivity1)
## VIT_04s0044g00360  -  MTN21
## VIT_01s0026g01120  -  R protein L6
## VIT_06s0004g03690  -  Glutathione S-transferase GSTO1
## VIT_15s0024g01650  -  Glutathione S-transferase 8 GSTU8
## VIT_05s0020g02640  -  Unknown protein
## VIT_10s0003g02710  -  Cold acclimation protein COR413-TM1
## VIT_11s0037g00750  -  CDK-activating kinase assembly factor MAT1
```

```
## VIT_06s0080g00350  -  Myo-inositol-1-phosphate synthase
## VIT_01s0011g04280  -  Receptor-like kinase ARK1AS
## VIT_12s0034g01080  -  3-isopropylmalate dehydrogenase 2, chloroplast precursor
## VIT_18s0001g02740  -  Photosystem II 22 kDa protein PSBS
## VIT_13s0067g02450  -  Uroporphyrinogen decarboxylase
## VIT_04s0008g00870  -  Phosphoethanolamine/phosphocholine phosphatase
## VIT_18s0001g05270  -  ERGIC and golgi 3
## VIT_13s0019g03960  -  Alpha-taxilin
## VIT_14s0006g01900  -  Unknown protein
## VIT_08s0007g05710  -  L-Galactono-1,4-lactone dehydrogenase
## VIT_07s0141g00630  -  Prenylated rab acceptor (PRA1)
## VIT_02s0025g01370  -  Unknown protein
## VIT_02s0025g03250  -  Protein kinase
## VIT_03s0017g00940  -  Cytochrome B6-F complex iron-sulfur subunit, PETC
## VIT_08s0032g00780  -  Calcium Dependent Protein Kinase (VvCPK8)
## VIT_02s0025g00510  -  Respiratory burst oxidase protein F (RBOHF)
## VIT_00s0615g00030  -  Mannitol dehydrogenase (ELI3-1)
## VIT_08s0056g01700  -  Cationic amino acid transporter 2
## VIT_19s0014g03190  -  Unknown protein
## VIT_14s0083g00080  -  Unknown protein
## VIT_00s2249g00010  -  GLABRA2 expression modulator
## VIT_14s0068g02160  -  Ribosomal protein S21
## VIT_17s0000g06320  -  Endonuclease
## VIT_15s0046g02030  -  Protein kinase
## VIT_19s0085g00840  -  SAM-dependent methyltransferase
## VIT_16s0039g02540  -  Rubredoxin
## VIT_04s0008g03560  -  Lactoylglutathione lyase
## VIT_01s0010g01730  -  Aldose reductase
## VIT_14s0030g01340  -  Beta-ketoacyl-CoA synthase
## VIT_11s0052g01510  -  MATE efflux family protein
## VIT_12s0028g01330  -  Glu-tRNA(Gln) amidotransferase subunit A
## VIT_06s0004g06660  -  Phosphoinositide-specific phospholipase C
## VIT_00s2038g00010  -  Tobamovirus multiplication 1
## VIT_07s0130g00250  -  4,5-DOPA dioxygenase extradiol
## VIT_10s0042g00170  -  TCP family transcription factor 7
## VIT_05s0124g00530  -  Ankyrin protein kinase
## VIT_08s0007g02270  -  LMBR1 integral membrane
## VIT_00s2525g00010  -  Unknown
## VIT_14s0066g01050  -  Glycogen synthase kinase 3 beta
## VIT_05s0124g00450  -  Unknown protein
## VIT_11s0016g01330  -  Unknown protein
## VIT_11s0052g01560  -  MATE efflux family protein
## VIT_05s0020g02300  -  TRNA/rRNA methyltransferase (SpoU)
## VIT_06s0009g00210  -  Calmodulin-binding region IQ5
## VIT_07s0005g04730  -  No hit
## VIT_19s0027g00380  -  Amine oxidase
## VIT_07s0031g03210  -  Protein kinase SPK-3 ASK1 (SnRK-7)
## VIT_10s0003g00860  -  Calmodulin-binding region IQD9
## VIT_13s0064g00680  -  Retroelement pol polyprotein
## VIT_01s0244g00070  -  Unknown protein
## VIT_15s0048g00750  -  Dihydrodipicolinate synthase
## VIT_01s0010g01720  -  Aldose reductase
## VIT_03s0038g04470  -  SEC14 cytosolic factor
## VIT_01s0146g00120  -  Amidohydrolase
## VIT_14s0006g01410  -  fructokinase-2
```

```
## VIT_18s0001g07150  -  Serine/threonine-protein kinase
## VIT_18s0001g10760  -  Strictosidine synthase
## VIT_01s0011g03560  -  fiber protein Fb34
## VIT_01s0011g01630  -  Protein NAP1 (Nck-associated protein 1)
## VIT_00s0759g00010  -  Porphobilinogen deaminase, chloroplast precursor
## VIT_02s0012g01800  -  Shikimate kinase
## VIT_17s0000g01650  -  Cyclopropyl isomerase
## VIT_01s0127g00720  -  Carbonic anhydrase, beta
## VIT_14s0128g00710  -  Mannosylglycoprotein endo-beta-mannosidase
## VIT_10s0116g00400  -  SEC14 cytosolic factor
## VIT_11s0016g00960  -  Subtilase
## VIT_10s0003g00120  -  Leucine-rich repeat family protein (LeSPL-CNR ortholog)
## VIT_01s0026g01540  -  Unknown protein
## VIT_00s1667g00010  -  LEM3 (ligand-effect modulator 3) family protein
## VIT_11s0037g00340  -  Homoserine kinase (HSK)
## VIT_00s0762g00040  -  S-locus lectin protein kinase
## VIT_17s0000g02570  -  Mitogen-activated Protein Kinase (VvMPK10)
## VIT_04s0008g02150  -  DCL3 (DICER-like 3)
## VIT_15s0046g03700  -  3-beta hydroxysteroid dehydrogenase
## VIT_09s0002g01040  -  Subtilase
## VIT_01s0011g02710  -  No hit
## VIT_07s0141g00590  -  Magnesium transporter CorA-like family protein (MRS2-2)
## VIT_02s0025g02550  -  Late embryogenesis abundant protein
## VIT_01s0137g00650  -  Unknown protein
## VIT_02s0012g00360  -  1-aminocyclopropane-1-carboxylate oxidase
## VIT_16s0050g01090  -  ß-carotene hydroxylase (BCH2) (VvBCH2)
## VIT_08s0007g00290  -  Pectinacetylesterase
```

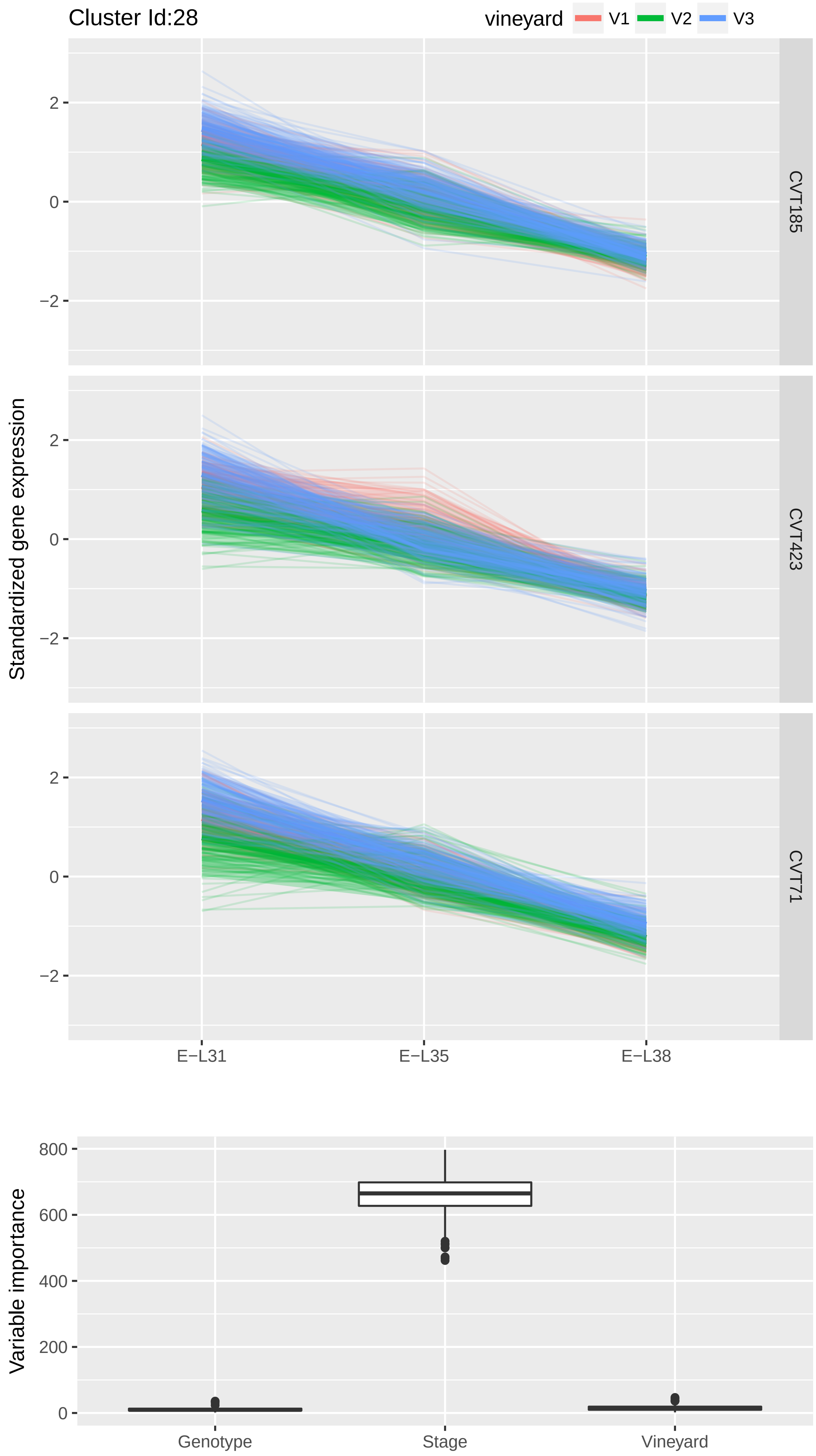

Cluster Id:28
vineyard
V1
V2
V3
Standardized gene expression
CVT185
CVT423
CVT71
2
0
−2
E−L31
E−L35
E−L38
Variable importance
800
600
400
200
0
Genotype
Stage
Vineyard

Cluster no. 29

```
## Number of genes in the cluster: 141
## Homogeneity Index:    0.46
## Variable importance for Stage:    Median =  133.2  - Rank =  101
## Variable importance for Clone:    Median =  19.35  - Rank =  55
## Variable importance for Vineyard: Median =  137.2  - Rank =  10
##
## Gene ID               Gene Annotation
## VIT_06s0004g03040  -  Transducin protein
## VIT_01s0011g05970  -  Heat stress transcription factor A-8
## VIT_09s0002g00340  -  Alanine aminotransferase 2
## VIT_15s0048g00350  -  3-phosphoshikimate 1-carboxyvinyltransferase, chloroplast precursor
## VIT_01s0026g02540  -  Zinc finger (C3HC4-type ring finger)
## VIT_04s0008g02700  -  TRNA (guanine-N(1)-)-methyltransferase
## VIT_03s0038g04500  -  Bifunctional dihydrofolate reductase-thymidylate synthase 1
## VIT_12s0057g00070  -  Wound-induced
## VIT_12s0057g00090  -  Wound-induced
## VIT_05s0077g00820  -  Unknown protein
## VIT_08s0007g03870  -  Phytosulfokines PSK1
## VIT_08s0007g04170  -  Pyruvate kinase, cytosolic isozyme
## VIT_17s0000g00300  -  Signal recognition particle subunit 68 SRP68
## VIT_18s0001g15660  -  Pathogen-related
## VIT_08s0056g01150  -  NADH-cytochrome b5 reductase
## VIT_03s0017g01700  -  Glycine cleavage system H protein, mitochondrial
## VIT_01s0026g02660  -  Negatively light-regulated protein
## VIT_19s0090g00400  -  Zinc finger (C3HC4-type ring finger)
## VIT_05s0049g00010  -  Cellulose synthase CSLG2
## VIT_18s0001g07470  -  Ulp1 protease
## VIT_01s0010g02460  -  Glyceraldehyde-3-phosphate dehydrogenase, cytosolic 3
## VIT_12s0057g00080  -  Wound-induced
## VIT_06s0080g00400  -  Ribosomal protein L10A (RPL10aA) 60S
## VIT_09s0002g05730  -  Esterase
## VIT_18s0001g07840  -  Unknown protein
## VIT_06s0004g03550  -  L-ascorbate peroxidase 1, cytosolic (APX1)
## VIT_04s0023g03600  -  SEN1 (dark inducible 1)
## VIT_00s0682g00020  -  RuvB DNA helicase protein
## VIT_07s0005g00360  -  Glycosyl hydrolase family 1
## VIT_03s0088g01070  -  Mutator-like transposase
## VIT_18s0001g02470  -  L-ascorbate peroxidase, thylakoid-bound (tAPX)
## VIT_18s0001g08160  -  No hit
## VIT_12s0035g02070  -  Cinnamoyl-CoA reductase
## VIT_15s0024g01170  -  No hit
## VIT_11s0078g00260  -  Unknown protein
## VIT_11s0016g01820  -  Lactoylglutathione lyase
## VIT_11s0016g02380  -  1-aminocyclopropane-1-carboxylate oxidase 2
## VIT_11s0016g00840  -  Ribosomal protein S29 (RPS29C) 40S
## VIT_00s0194g00130  -  Transcriptional adaptor (ADA2b)PROPORZ1
## VIT_05s0020g02400  -  F-box protein (FBL4)
## VIT_07s0005g00350  -  No hit
## VIT_02s0025g00530  -  Unknown
## VIT_08s0007g04310  -  Threonine dehydratase/deaminase
## VIT_02s0033g01160  -  Unknown protein
## VIT_11s0037g00450  -  No hit
## VIT_05s0094g00500  -  C2 domain-containing protein
## VIT_01s0127g00490  -  Pantothenate kinase 2
```

```
## VIT_14s0066g02340  -  Zinc finger (C3HC4-type ring finger)
## VIT_14s0083g00970  -  Ribosomal protein L7A (RPL7aB) 60S
## VIT_07s0005g03660  -  Non-intrinsic ABC protein 4
## VIT_17s0000g06370  -  Thioredoxin 2
## VIT_04s0008g00510  -  Derlin-1
## VIT_19s0014g03310  -  No hit
## VIT_13s0074g00010  -  Protein phosphate transporter traffic facilitator 1 (PHF-1)
## VIT_18s0122g00510  -  6-phosphofructokinase, pyrophosphate dependent
## VIT_12s0057g00170  -  Wound-induced
## VIT_10s0003g03130  -  Ribosomal protein L18A (RPL18aB) 60S
## VIT_00s0583g00040  -  Zinc finger (C3HC4-type ring finger)
## VIT_03s0091g00310  -  Indole-3-acetic acid-amido synthetase GH3.8
## VIT_14s0060g01200  -  Unknown protein
## VIT_16s0039g01670  -  Cinnamoyl-CoA reductase
## VIT_16s0039g02500  -  Shikimate kinase
## VIT_12s0057g00150  -  Wound-induced
## VIT_03s0063g00640  -  Transcriptional factor B3
## VIT_13s0074g00660  -  ABC Transporter (VvPDR24 - VvABCG54)
## VIT_00s0214g00160  -  Lectin, phloem-specific
## VIT_06s0009g01130  -  Molybdopterin biosynthesis protein CNX1
## VIT_04s0023g03590  -  Unknown
## VIT_00s0179g00090  -  TIFY gene family (VvPPD2)
## VIT_01s0011g04510  -  No hit
## VIT_18s0089g00450  -  R protein L6
## VIT_09s0002g03380  -  Poly(A) polymerase
## VIT_10s0003g03950  -  AERO1 (arabidopsis endoplasmic reticulum oxidoreductins 1)
## VIT_12s0057g00140  -  Wound-induced
## VIT_18s0001g01830  -  ATP-NAD kinase
## VIT_14s0171g00320  -  Kelch repeat-containing protein
## VIT_01s0011g01160  -  Haloacid dehalogenase hydrolase
## VIT_12s0028g01410  -  Heat shock transcription factor A4A
## VIT_06s0004g03660  -  Pseudo-response regulator 7 (APRR7)
## VIT_18s0001g03390  -  S-receptor kinase
## VIT_08s0007g05890  -  GTP binding
## VIT_02s0025g02490  -  Unknown protein
## VIT_14s0060g01990  -  ATPP2-B15 (Phloem protein 2-B15)
## VIT_04s0008g01660  -  Carrier protein, Mitochondrial
## VIT_08s0056g00790  -  Phagocytosis and cell motility protein ELMO1
## VIT_14s0030g01650  -  Unknown protein
## VIT_01s0011g01170  -  No hit
## VIT_06s0004g03650  -  Pseudo-response regulator 7 (APRR7)
## VIT_12s0057g00110  -  Wound-induced
## VIT_15s0048g02860  -  Auxin-responsive SAUR29
## VIT_12s0057g00120  -  Wound-induced
## VIT_13s0019g02430  -  LIM domain containing protein
## VIT_02s0025g01070  -  CHCH domain containing protein
## VIT_07s0031g01170  -  Aspartic Protease (VvAP17)
## VIT_04s0008g00520  -  6-phosphofructokinase
## VIT_16s0098g00550  -  DNA binding
## VIT_15s0048g02260  -  Calcium-binding EF hand
## VIT_14s0068g01500  -  Glycerophosphoryl diester phosphodiesterase
## VIT_08s0056g01300  -  Cupin, RmlC-type
## VIT_08s0058g01000  -  Aspartate aminotransferase
## VIT_18s0001g00950  -  Prolyl 4-hydroxylase alpha-2 subunit precursor
```

```
## VIT_14s0066g02170  -  Prolyl 4-hydroxylase
## VIT_08s0056g01660  -  Histone-lysine N-methyltransferase SUVR3
## VIT_03s0097g00470  -  ATHVA22A (Arabidopsis thaliana HVA22 homologue A)
## VIT_17s0000g04160  -  Zinc finger (C3HC4-type ring finger)
## VIT_08s0040g01760  -  Ribonucleoprotein (CP-RBP31)
## VIT_08s0007g07540  -  HNH endonuclease domain-containing protein
## VIT_04s0079g00810  -  Cold induced protein
## VIT_18s0089g00460  -  TIR-NBS-TIR type disease resistance protein
## VIT_13s0067g01840  -  ferritin
## VIT_16s0100g01260  -  Methyl-CpG-binding domain 13 MBD13
## VIT_19s0015g01000  -  Zinc finger (C3HC4-type ring finger)
## VIT_18s0001g11040  -  Heme oxygenase 2
## VIT_11s0016g03210  -  NADP-dependent malic enzyme
## VIT_11s0037g00080  -  Ribosomal protein S12 (RPS12C) 40S
## VIT_08s0105g00220  -  PRLI-interacting factor G-like protein
## VIT_12s0057g00100  -  Wound-responsive
## VIT_17s0000g05010  -  Unknown
## VIT_01s0010g02050  -  UBX domain-containing protein
## VIT_14s0060g02380  -  Autophagy 18 ATG18d
## VIT_12s0035g01760  -  DNA (cytosine-5)-methyltransferase AthI
## VIT_11s0016g00690  -  Senescence-associated protein
## VIT_04s0008g04720  -  Endonuclease/exonuclease/phosphatase family protein
## VIT_08s0007g01290  -  BEL1-like homeodomain protein 9 LSN (LARSON)
## VIT_10s0042g00100  -  Superoxide dismutase, Fe-Mn family
## VIT_06s0004g06530  -  Zinc finger (C3HC4-type ring finger)
## VIT_18s0072g00060  -  Ribosomal protein S18 (RPS18C) 40S
## VIT_19s0085g00390  -  Unknown protein
## VIT_14s0108g01080  -  myb domain protein 106
## VIT_18s0001g14440  -  Molecular chaperone DnaJ
## VIT_12s0057g00190  -  Unknown
## VIT_03s0017g01240  -  2-nitropropane dioxygenase
## VIT_09s0054g00450  -  HEN4 (HUA enhancer 4)
## VIT_05s0077g02080  -  Branched chain alpha-keto acid dehydrogenase E2 subunit (din3)
## VIT_14s0108g00090  -  Cupin, RmlC-type
## VIT_06s0061g00710  -  Zinc finger (C3HC4-type ring finger)
## VIT_09s0002g09110  -  No hit
## VIT_08s0007g04180  -  Retrotransposon protein, Ty1-copia subclass
## VIT_19s0014g04590  -  S-locus protein kinase
## VIT_12s0035g01770  -  DNA (cytosine-5)-methyltransferase AthI
## VIT_04s0044g00970  -  Unknown protein
```

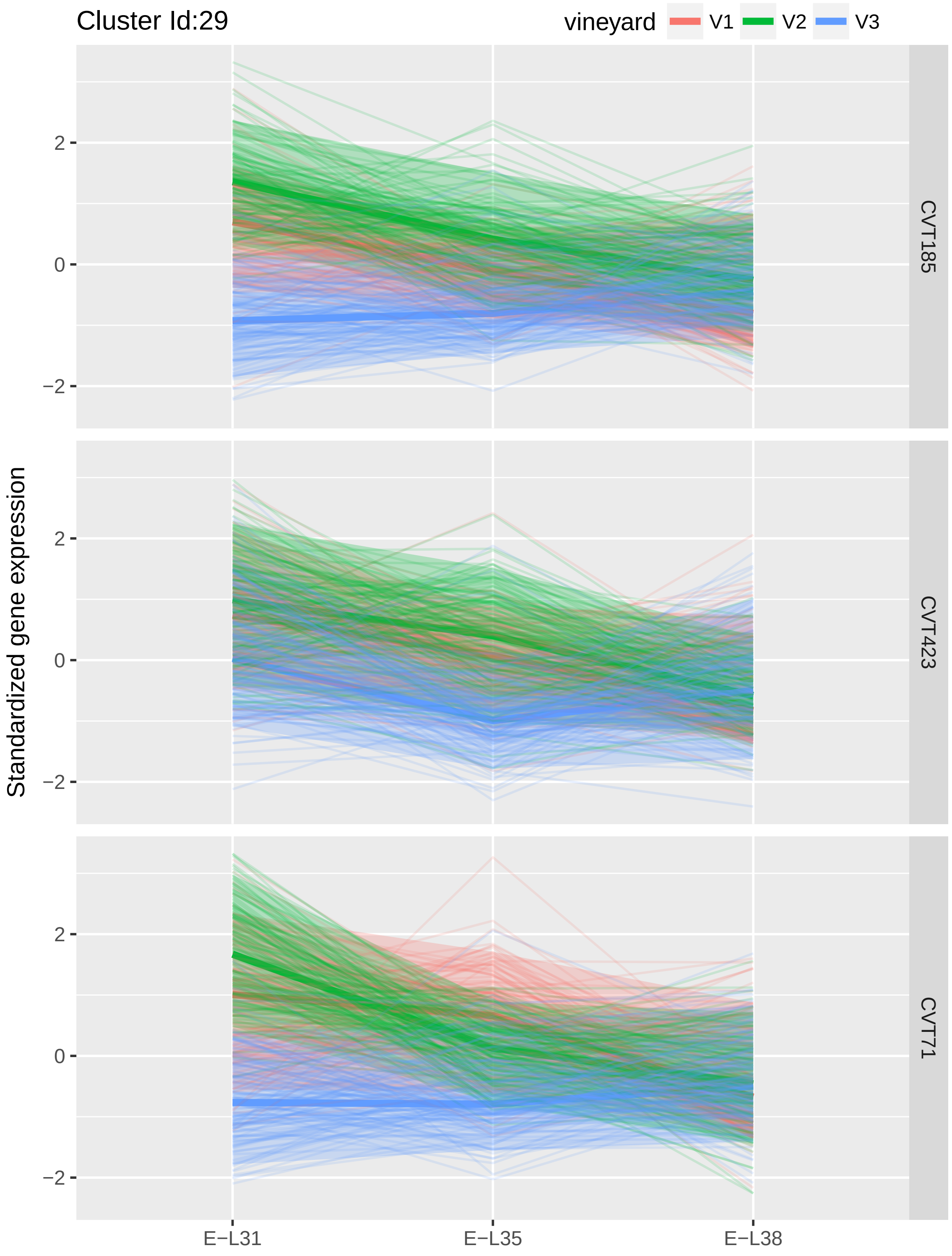
Cluster Id:29
vineyard
V1
V2
V3
Standardized gene expression
CVT185
CVT423
CVT71
2
0
−2
E−L31
E−L35
E−L38

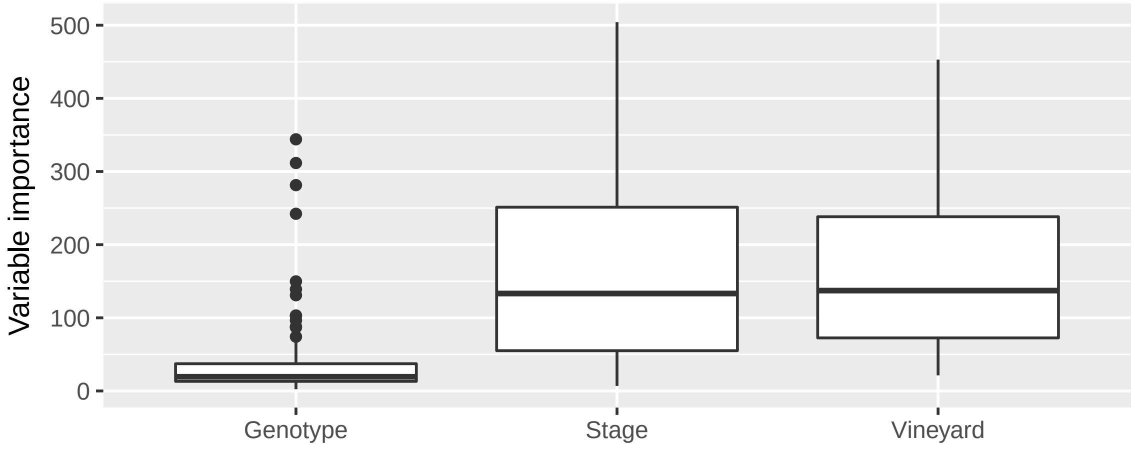
Variable importance
500
400
300
200
100
0
Genotype
Stage
Vineyard

Cluster no. 30

```
## Number of genes in the cluster: 85
## Homogeneity Index:    0.49
## Variable importance for Stage:    Median =  156.9  - Rank =  98
## Variable importance for Clone:    Median =  28.23  - Rank =  10
## Variable importance for Vineyard: Median =  91.59  - Rank =  18
##
## Gene ID               Gene Annotation
## VIT_09s0054g01030  -  Transcription termination factor mitochondrial mTERF
## VIT_05s0049g01930  -  RNA-binding protein cp29
## VIT_08s0007g02610  -  TLD domain-containing protein KIAA1609
## VIT_07s0005g04300  -  Nuclear transport factor 2 (NTF2)
## VIT_16s0098g01690  -  Unknown
## VIT_08s0007g04490  -  PHD finger transcription factor
## VIT_02s0012g01780  -  WD-repeat protein 74
## VIT_18s0001g04970  -  TRNA modification GTPase trmE
## VIT_01s0010g02810  -  Unknown protein
## VIT_03s0091g00440  -  Pentatricopeptide (PPR) repeat-containing protein
## VIT_09s0002g04430  -  Phox (PX) domain-containing protein
## VIT_05s0029g00650  -  Zinc finger (C3HC4-type ring finger)
## VIT_14s0068g01590  -  Geranylgeranylated protein ATGP4
## VIT_13s0019g04720  -  Vacuolar protein sorting 33
## VIT_17s0000g02420  -  Auxin transport protein (PIN3)
## VIT_01s0026g01860  -  MAIGO 1 MAG1
## VIT_13s0067g03620  -  SET domain-containing protein
## VIT_01s0010g01590  -  Unknown protein
## VIT_05s0094g00890  -  Haloacid dehalogenase hydrolase
## VIT_06s0004g01860  -  Zinc transporter ZIP3
## VIT_11s0037g00190  -  Endonuclease/exonuclease/phosphatase, related
## VIT_06s0004g00080  -  EMB1011 (embryo defective 1011)
## VIT_10s0003g02190  -  Nodulin MtN3 family
## VIT_02s0025g01990  -  Zinc finger (C3HC4-type ring finger)
## VIT_18s0001g09320  -  Casein kinase II subunit alpha-2
## VIT_03s0091g00860  -  DNA polymerase kappa subunit
## VIT_02s0025g01980  -  Cellulose synthase CSLG3
## VIT_14s0108g00580  -  DegP protease
## VIT_13s0019g00810  -  Methionyl-tRNA synthetase (AtcpMetRS)
## VIT_13s0156g00350  -  zeaxanthin epoxidase (ZEP; ABA1) (VvZEP2)
## VIT_08s0105g00510  -  Unknown protein
## VIT_19s0027g00700  -  Iojap protein
## VIT_19s0177g00220  -  Electron transfer flavoprotein alpha subunit
## VIT_14s0060g01120  -  Pentatricopeptide (PPR) repeat
## VIT_14s0006g02230  -  Unknown protein
## VIT_06s0004g07420  -  Unknown protein
## VIT_04s0044g00550  -  Carbamoyl phosphate synthetase small subunit
## VIT_12s0059g01930  -  Secretory carrier membrane protein (SCAMP1)
## VIT_06s0004g03460  -  Transducin protein
## VIT_04s0008g02440  -  Unknown protein
## VIT_15s0048g02200  -  Caffeoyl-CoA O-methyltransferase
## VIT_17s0000g09670  -  Stachyose synthase precursor
## VIT_01s0026g01720  -  ATWHY1/PTAC1 (A. thaliana WHIRLY 1)
## VIT_10s0116g01630  -  Endonuclease/exonuclease/phosphatase family
## VIT_12s0142g00120  -  Nuclear movement protein
## VIT_02s0025g01110  -  Phytochrome A supressor spa1 (SPA1)
## VIT_10s0003g01230  -  Serine esterase
```

```
## VIT_05s0020g01800  -  Zinc finger (C3HC4-type ring finger)
## VIT_06s0004g04950  -  Scarecrow-like transcription factor 14 SCL14
## VIT_10s0003g01110  -  Unknown protein
## VIT_19s0014g01190  -  Alpha-1,3-mannosyltransferase ALG2
## VIT_17s0000g01720  -  Nucleolar protein 58
## VIT_04s0008g01410  -  Ran-binding protein 1 RanBP1
## VIT_12s0059g01920  -  Strubbelig-receptor family 3
## VIT_17s0000g07580  -  ARR6 typeA
## VIT_07s0104g00320  -  Glutathione peroxidase 4
## VIT_09s0054g01360  -  Cycloartenol synthase
## VIT_18s0001g02860  -  Binding
## VIT_00s0179g00100  -  Ethylene overproducer 1 (ETO1)
## VIT_17s0000g01530  -  Unknown protein
## VIT_01s0011g04970  -  SEC12-like protein 2 STL2P
## VIT_01s0026g00650  -  DNA polymerase lambda subunit
## VIT_17s0000g05630  -  Homeodomain leucine zipper protein HB-1
## VIT_03s0038g04690  -  Isoflavone reductase protein 6
## VIT_16s0039g02170  -  D-xylose-proton symporter-like 2
## VIT_10s0003g01150  -  Autophagy 7 (APG7)
## VIT_17s0000g02540  -  CTR1 kinase kinase kinase
## VIT_01s0011g00840  -  Ankyrin
## VIT_07s0129g00060  -  Adenylylsulfate kinase
## VIT_11s0016g03410  -  Unknown protein
## VIT_17s0000g03590  -  Unknown protein
## VIT_04s0008g05470  -  EIN3-binding F-box protein 2
## VIT_03s0038g02390  -  Ubiquitin-conjugating enzyme E2 D/E UBC28
## VIT_08s0007g00330  -  Metallothionein
## VIT_06s0004g03180  -  Zinc finger (FYVE type)
## VIT_11s0016g05410  -  EIN3-binding F-box protein 1
## VIT_00s0187g00210  -  Rho GDP-dissociation inhibitor 2
## VIT_13s0067g02960  -  BTI2 (VIRB2- interacting  protein 2)
## VIT_01s0026g01250  -  Unknown protein
## VIT_02s0012g00560  -  Tobamovirus multiplication 2A TOM2A
## VIT_00s0317g00060  -  Unknown protein
## VIT_05s0020g04590  -  Acylamino-acid-releasing enzyme
## VIT_10s0116g01210  -  Aspartic Protease (VvAP27)
## VIT_18s0001g02830  -  Binding
## VIT_16s0098g01430  -  3'-5' exonuclease domain-containing protein
```

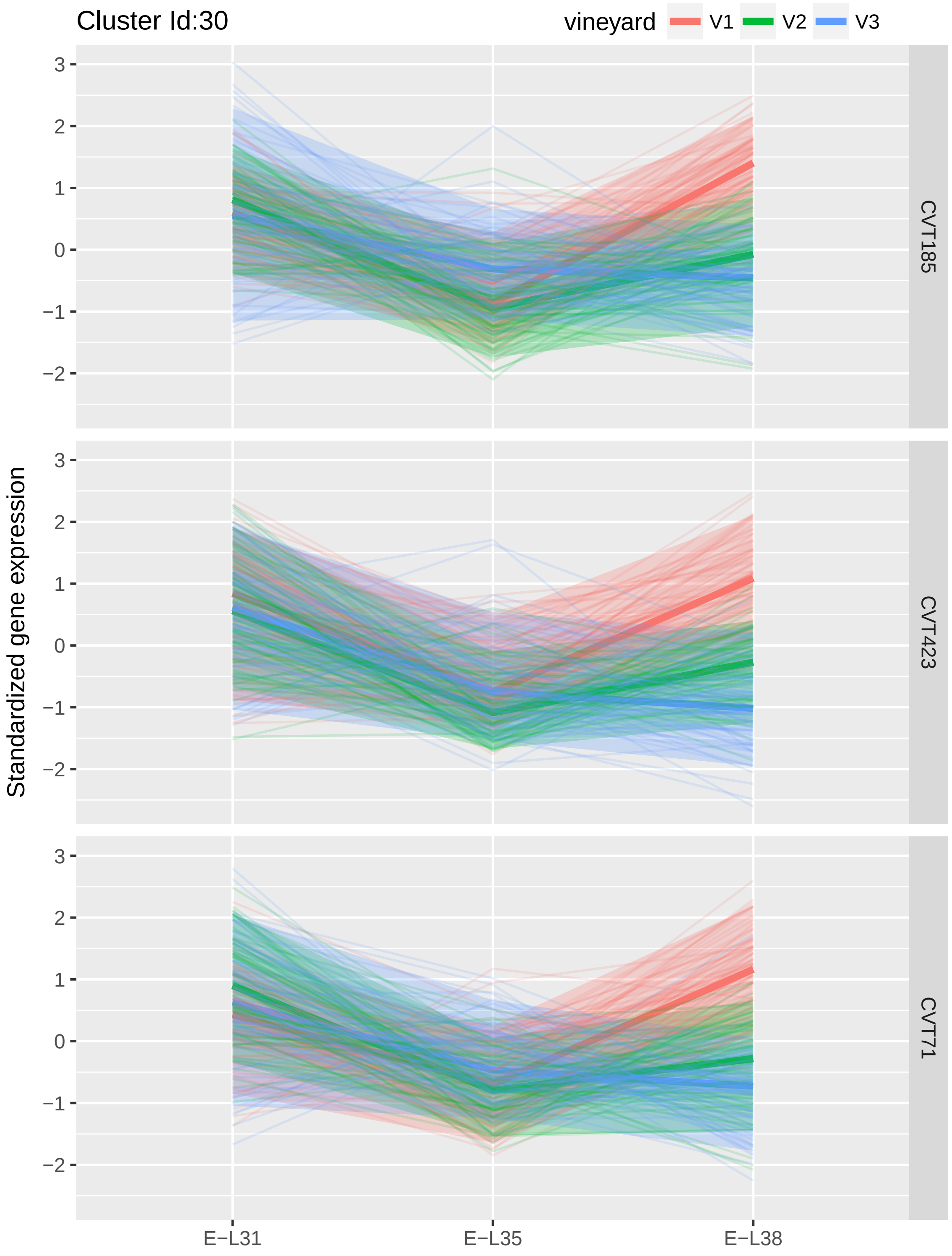

Cluster Id:30
vineyard
V1
V2
V3
Standardized gene expression
CVT185
CVT423
CVT71
E−L31
E−L35
E−L38
3
2
1
0
−1
−2

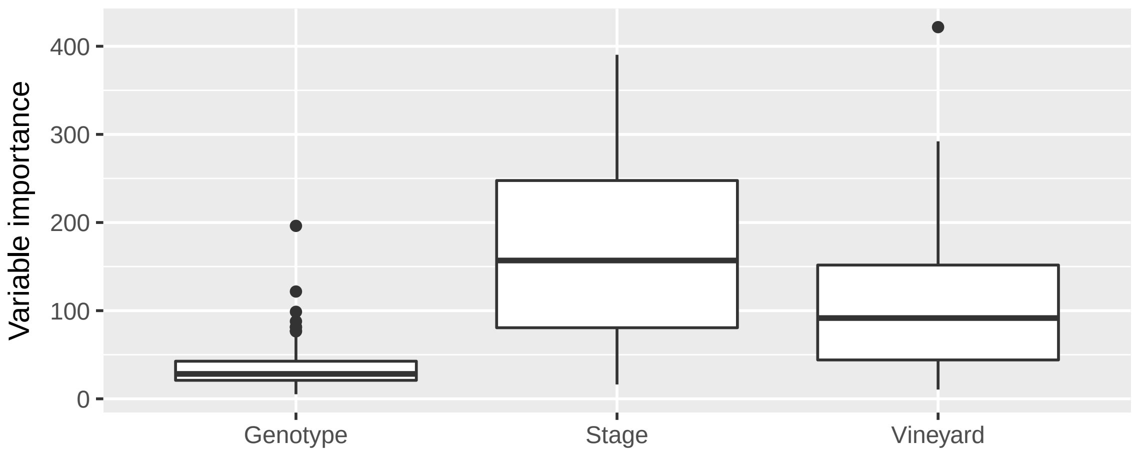

Variable importance
400
300
200
100
0
Genotype
Stage
Vineyard

Cluster no. 31

```
## Number of genes in the cluster: 272
## Homogeneity Index:    0.82
## Variable importance for Stage:    Median =  632.8  - Rank =  9
## Variable importance for Clone:    Median =  12.05  - Rank =  92
## Variable importance for Vineyard: Median =  15.22  - Rank =  99
##
## Gene ID              Gene Annotation
## VIT_16s0039g01560  -  PLATZ transcription factor
## VIT_03s0063g00170  -  basic helix-loop-helix (bHLH) family
## VIT_05s0020g00820  -  RelA/SpoT protein (RSH4)
## VIT_19s0014g02160  -  Drought induced 19 protein
## VIT_03s0180g00260  -  Cinnamyl alcohol dehydrogenase
## VIT_16s0039g01840  -  Heat shock transcription factor protein 3 (HSF3)
## VIT_03s0038g03900  -  Amino acid permease
## VIT_18s0001g07280  -  Glucose-6-phosphate isomerase
## VIT_09s0002g06660  -  DEAD  box  RNA helicase RH15
## VIT_13s0067g03880  -  Ankyrin repeat
## VIT_17s0000g01680  -  Adenylate kinase family
## VIT_14s0171g00520  -  Unknown protein
## VIT_02s0025g01720  -  GDA2
## VIT_00s0220g00120  -  Protein kinase
## VIT_16s0039g02680  -  Unknown protein
## VIT_12s0028g03580  -  Lectin-receptor like protein kinase 3
## VIT_10s0003g05840  -  Unknown protein
## VIT_16s0050g01060  -  Unknown protein
## VIT_07s0031g00460  -  Nicotinate phosphoribosyltransferase
## VIT_10s0116g00210  -  UBX domain-containing protein 1
## VIT_05s0077g01190  -  Outer envelope protein 16
## VIT_12s0055g00840  -  Sucrose-6-phosphate phosphatase
## VIT_08s0032g00730  -  F-box and JmjC domain protein
## VIT_09s0002g01490  -  Developmentally regulated G-protein 1
## VIT_13s0084g00580  -  Histone mRNA exonuclease 1
## VIT_09s0002g07300  -  Phox
## VIT_18s0001g12380  -  Continuous vascular ring (COV1)
## VIT_08s0058g00330  -  Unknown
## VIT_17s0000g04730  -  Zinc finger (C3HC4-type ring finger)
## VIT_07s0191g00170  -  LMBR1 integral membrane
## VIT_16s0050g00910  -  MATE efflux family protein (VvAnthoMATE2)
## VIT_17s0000g04450  -  Phospholipid-transporting ATPase
## VIT_08s0007g03960  -  Phosphopyruvate hydratase.
## VIT_00s0333g00030  -  Coiled-coil domain-containing protein 94
## VIT_08s0058g00440  -  ferritin
## VIT_07s0141g00230  -  Ubiquitin-specific protease 3 (UBP3)
## VIT_01s0150g00090  -  AML5 (arabidopsis mei2-like protein 5)
## VIT_08s0007g07300  -  Protein disulfide isomerase (PDIL) protein
## VIT_08s0007g01430  -  Universal stress protein (USP) family protein
## VIT_01s0026g02720  -  ATP/GTP-binding protein
## VIT_19s0014g02760  -  Ubiquitin-conjugating enzyme E2 O
## VIT_00s0404g00060  -  Pre-mRNA-splicing factor SYF1
## VIT_19s0085g00040  -  Related to Ubiquitin 1 (RUB1)
## VIT_18s0001g00560  -  Alpha-amylase / 1,4-alpha-D-glucan glucanohydrolase
## VIT_05s0077g02140  -  fatty acid multifunctional protein (MFP2)
## VIT_18s0122g01220  -  DNA-binding bromodomain-containing protein
## VIT_01s0137g00500  -  Unknown protein
```

```
## VIT_00s0187g00150  -  E3 ubiquitin-protein ligase HUWE1
## VIT_16s0050g00930  -  anthocyanin permease (VvAnthoMATE1 - VvAM1)
## VIT_02s0025g03600  -  Phospholipid hydroperoxide glutathione peroxidase
## VIT_02s0012g02090  -  Unknown protein
## VIT_14s0036g01380  -  PHR1 (phosphate starvation response 1)
## VIT_15s0021g02050  -  PRIB5 protein
## VIT_15s0046g01290  -  Carrier protein, Mitochondrial
## VIT_01s0011g04490  -  ATP synthase beta chain 2, mitochondrial
## VIT_09s0096g00090  -  Hypoxia up-regulated 1 HSP70
## VIT_09s0070g00130  -  Ribosomal protein L7 (RPL7A) 60S
## VIT_08s0007g06240  -  CDK5RAP3
## VIT_14s0006g01870  -  E3 ubiquitin-protein ligase SIAH2
## VIT_08s0040g00430  -  COP9 signalosome complex subunit 2
## VIT_05s0077g01350  -  Mov34 STAM-binding protein
## VIT_10s0003g01850  -  RKF1 (receptor-like kinase in flowers 1)
## VIT_07s0005g02960  -  Kelch repeat-containing F-box protein
## VIT_09s0070g00320  -  Male germ cell-associated kinase
## VIT_01s0011g04000  -  Unknown protein
## VIT_17s0053g00320  -  Zinc finger (C3HC4-type ring finger)
## VIT_08s0058g01360  -  Vacuolar sorting receptor 1
## VIT_18s0001g14310  -  flavanone-3-hydroxylase  2 (F3H2) [Vitis vinifera]
## VIT_13s0084g00680  -  Ring E3 ligase (keep on going)
## VIT_02s0087g00340  -  NFD4 (nuclear fusion defective 4)
## VIT_16s0050g00950  -  Scarecrow-like transcription factor 8 (SCL8)
## VIT_15s0021g02730  -  SEC16 homolog B
## VIT_01s0010g03730  -  F-box domain containing protein
## VIT_18s0001g04190  -  Co-chaperone grpE
## VIT_13s0019g05110  -  Amino acid binding protein
## VIT_14s0006g00830  -  Unknown protein
## VIT_07s0005g01730  -  flowering time control protein FCA
## VIT_05s0020g03720  -  RNA pol II accessory factor Cdc73
## VIT_08s0058g01230  -  Non-specific lipid-transfer protein
## VIT_05s0124g00550  -  Unknown protein
## VIT_14s0171g00330  -  Ubiquinol-cytochrome c reductase cytochrome c1
## VIT_02s0087g00410  -  Unknown protein
## VIT_04s0008g04690  -  Proteasome 26S AAA-ATPase subunit (RPT6a)
## VIT_08s0040g00410  -  Proteasome 20S alpha subunit G (PAG1) (PRC8)
## VIT_05s0020g02150  -  NADH dehydrogenase (ubiquinone) 1 alpha subcomplex 8
## VIT_05s0029g00010  -  Type 1 membrane protein
## VIT_08s0058g00410  -  ferritin 1 (FER1)
## VIT_18s0001g05720  -  14-3-3 protein GF14 epsilon (GRF10)
## VIT_18s0001g02510  -  Protein disulfide-isomerase A1
## VIT_12s0055g00780  -  VPS20.2 SNF7
## VIT_17s0000g09990  -  Zinc finger (CCCH-type) family protein
## VIT_06s0009g03530  -  Homeodomain protein 14
## VIT_06s0061g00540  -  VPS24.1 SNF7
## VIT_05s0102g00130  -  Unknown protein
## VIT_14s0083g00450  -  Protein kinase PKN/PRK1, effector
## VIT_15s0046g00020  -  Ubiquitin-protein ligase
## VIT_02s0025g04660  -  Senescence-inducible chloroplast stay-green protein 1
## VIT_03s0063g01780  -  Extra-large G-protein (XLG1)
## VIT_10s0003g01000  -  Malate dehydrogenase
## VIT_05s0020g04900  -  N-carbamyl-L-amino acid amidohydrolase
## VIT_08s0007g01540  -  MYB1
## VIT_14s0066g02440  -  Glycosyl transferase family 14 protein
```

```
## VIT_06s0004g00100  -  Unknown protein
## VIT_17s0000g04540  -  Enolase
## VIT_19s0014g03410  -  Late embryogenesis abundant protein
## VIT_10s0042g01130  -  Zinc finger (C3HC4-type ring finger)
## VIT_16s0013g01540  -  Ubiquinol-cytochrome C chaperone
## VIT_06s0004g05560  -  Invertase/pectin methylesterase inhibitor
## VIT_04s0023g03920  -  Zinc finger (FYVE type)
## VIT_03s0038g00030  -  DRD1 (defective in rna-directed dna methylation 1)
## VIT_10s0003g02450  -  flavonol synthase
## VIT_16s0050g00580  -  Signal peptide peptidase
## VIT_14s0083g00160  -  Proteasome maturation factor UMP1
## VIT_07s0005g04560  -  Unknown protein
## VIT_13s0019g00840  -  UDP-glucuronate decarboxylase.
## VIT_18s0117g00130  -  Pi starvation-induced protein
## VIT_03s0063g00480  -  DEM2
## VIT_04s0023g00080  -  carotene hydroxylase  (CYP97A3; LUT5) (VvLUT5)
## VIT_01s0011g02300  -  Unknown protein
## VIT_08s0056g00250  -  Harpin-induced 1
## VIT_01s0011g00540  -  SPFH domain-containing protein 2 precursor band 7 family
## VIT_06s0004g06700  -  ATP-dependent Clp protease ClpB protein
## VIT_05s0094g01400  -  La domain-containing protein
## VIT_09s0002g01800  -  Dihydrolipoamide S-acetyltransferase
## VIT_01s0011g02810  -  Nuclear matrix constituent protein 1
## VIT_19s0015g01830  -  Unknown protein
## VIT_17s0000g01240  -  Gamma carbonic anhydrase-like 2 (gamma CAL2)
## VIT_04s0210g00220  -  Ubiquilin-1
## VIT_13s0156g00640  -  AP2-associated kinase
## VIT_08s0040g01910  -  V-type H+-transporting ATPase subunit G
## VIT_14s0068g01560  -  Unknown protein
## VIT_08s0007g01340  -  Proteasome inhibitor subunit 1 (PI31)
## VIT_14s0060g01170  -  Cytochrome c oxidase subunit Vc
## VIT_17s0000g05510  -  Pentatricopeptide (PPR) repeat-containing protein
## VIT_08s0058g00430  -  ferritin
## VIT_14s0171g00340  -  F-box family protein
## VIT_08s0007g02820  -  Unknown protein
## VIT_10s0003g01530  -  Unknown protein
## VIT_12s0028g00410  -  Protein phosphatase 2C
## VIT_02s0025g00780  -  Cation/hydrogen exchanger (CHX18)
## VIT_03s0038g03630  -  F-box domain containing protein
## VIT_14s0066g00800  -  Proline-rich family protein
## VIT_05s0020g04150  -  Emp24/gp25L/p24
## VIT_16s0039g02250  -  Cytochrome c oxidase subunit XI assembly protein
## VIT_17s0000g06250  -  RWP-RK domain-containing protein
## VIT_08s0040g01290  -  F-box family protein
## VIT_12s0028g02620  -  Protein kinase C substrate 80K-H
## VIT_01s0150g00110  -  Unknown protein
## VIT_08s0007g02590  -  Unknown
## VIT_19s0015g01160  -  SEC14 cytosolic factor
## VIT_04s0008g04970  -  1-deoxy-D-xylulose-5-phosphate synthase, chloroplast precursor
## VIT_10s0003g01420  -  CBL-interacting protein kinase 19 CIPK19
## VIT_05s0124g00520  -  No hit
## VIT_02s0033g01100  -  No hit
## VIT_18s0001g08890  -  Haemolysin-III related
## VIT_16s0013g00620  -  Methionine sulfoxide reductase A
## VIT_02s0087g00880  -  Nodulin
```

```
## VIT_06s0004g05140  -  DnaJ homolog, subfamily B, member 9
## VIT_04s0008g01400  -  Protein kinase MK6
## VIT_18s0001g10820  -  Proteasome 26S regulatory subunit (RPN11)
## VIT_02s0012g00850  -  Splicing factor PWI domain-containing protein
## VIT_15s0021g00920  -  ATP synthase delta' chain, mitochondrial
## VIT_13s0019g01900  -  FK506-binding protein genes family (VvFKBP20-1)
## VIT_14s0066g01720  -  Bromo-adjacenty (BAH) domain-containing protein
## VIT_08s0040g00120  -  DnaJ homolog, subfamily B, member 13
## VIT_05s0094g00120  -  DNA-directed RNA polymerase II subunit H
## VIT_04s0008g05030  -  Ras GTP-binding protein (RAN3)
## VIT_00s0229g00110  -  Agenet domain-containing protein
## VIT_11s0065g00450  -  Adhesion regulating molecule 1 Adrm1 protein
## VIT_06s0004g03140  -  Isovaleryl-CoA-dehydrogenase (IVD)
## VIT_12s0059g01680  -  Lipid-binding serum glycoprotein family protein
## VIT_05s0020g01430  -  Vesicle-associated membrane protein 72
## VIT_08s0007g03610  -  Monodehydroascorbate reductase
## VIT_14s0108g01650  -  Peptidase beta subunit, Mitochondrial
## VIT_04s0008g04280  -  Zinc finger (C3HC4-type ring finger)
## VIT_14s0219g00190  -  Steroid nuclear receptor, ligand-binding
## VIT_06s0004g01090  -  Protein kinase
## VIT_11s0016g04390  -  Unknown protein
## VIT_02s0087g01020  -  CRK10 (cysteine-rich RLK10)
## VIT_05s0077g02040  -  fumarylacetoacetate hydrolase
## VIT_09s0002g05800  -  Proteasome 26S AAA-ATPase subunit (RPT6a)
## VIT_14s0219g00110  -  Unknown protein
## VIT_12s0034g01440  -  Emp24/gp25L/p24
## VIT_05s0029g01140  -  Sucrose-phosphate synthase
## VIT_05s0051g00350  -  PHD finger transcription factor
## VIT_12s0028g03600  -  Unknown protein
## VIT_03s0038g00680  -  Exostosin
## VIT_02s0033g01110  -  Male germ cell-associated kinase
## VIT_13s0106g00790  -  Mevalonate diphosphate decarboxylase (MVD1)
## VIT_19s0014g05190  -  Cellulase precursor
## VIT_00s0207g00100  -  Metallophosphoesterase
## VIT_11s0065g00160  -  Splicing factor YT521-B
## VIT_01s0011g01540  -  Unknown protein
## VIT_14s0081g00090  -  Prenylated rab acceptor (PRA1)
## VIT_06s0004g02980  -  Kelch repeat-containing F-box protein
## VIT_14s0006g01990  -  Translation initiation factor eIF-5
## VIT_08s0105g00270  -  NC domain-containing protein
## VIT_04s0023g00840  -  Mitochondrial Acyl carrier protein 2
## VIT_03s0063g02410  -  Switching protein 3D ATSWI3D
## VIT_13s0019g03420  -  C2 domain-containing protein
## VIT_18s0075g00350  -  Sucrose-phosphate synthase isoform C
## VIT_14s0030g01660  -  Serine/threonine kinase WNK1
## VIT_01s0026g01650  -  Porin II, 36kDa
## VIT_05s0029g01340  -  Auxin-independent growth promoter (axi 1)
## VIT_18s0001g09570  -  Chalcone reductase
## VIT_12s0028g01050  -  Helicase in vascular tissue and TAPETUM
## VIT_14s0060g02280  -  Zinc finger (C3HC4-type ring finger)
## VIT_09s0002g02430  -  ABC Transporter (VvMRP8 - VvABCC8)
## VIT_04s0008g04290  -  Transcription elongation factor S-II
## VIT_06s0004g06510  -  Phosphoesterase
## VIT_07s0031g00220  -  ERF/AP2 Gene Family (VvAP2-13)
## VIT_10s0071g00980  -  Unknown protein
```

```
## VIT_09s0018g01390  -  STE20/SPS1 proline-alanine-rich protein kinase
## VIT_13s0019g01670  -  Telomeric DNA binding protein 1
## VIT_08s0007g07160  -  Transcriptional co-activator
## VIT_18s0001g08290  -  Poly(ADP-ribose) polymerase
## VIT_13s0019g03380  -  Coronatine-insensitive protein 1
## VIT_16s0013g01930  -  Acid phosphatase
## VIT_18s0086g00680  -  No hit
## VIT_13s0019g04670  -  Zinc finger (DHHC type) family
## VIT_19s0015g01400  -  DNA damage-inducible protein 1
## VIT_02s0033g01070  -  Anthraniloyal-CoA: methanol anthraniloyal transferase
## VIT_08s0007g08310  -  Alkaline alpha galactosidase
## VIT_03s0063g01900  -  Import receptor subunit TOM6, Mitochondrial
## VIT_09s0018g01370  -  STE20/SPS1 proline-alanine-rich protein kinase
## VIT_12s0057g00230  -  Pep3/Vps18/deep orange
## VIT_07s0005g04970  -  NADH dehydrogenase
## VIT_00s0179g00140  -  Protein phosphatase 2C / PP2C
## VIT_03s0038g03680  -  Ubiquilin-2
## VIT_18s0001g09170  -  Interferon developmental regulator
## VIT_06s0004g05890  -  Ceramidase
## VIT_01s0113g00560  -  Unknown protein
## VIT_13s0019g01720  -  Abscisic acid insensitive 8
## VIT_06s0080g00540  -  Unknown protein
## VIT_02s0025g00900  -  ABC Transporter (VvMRP11 - VvABCC11)
## VIT_09s0018g01910  -  Unknown
## VIT_01s0146g00270  -  Delta3,5-delta2,4-dienoyl-CoA isomerase
## VIT_04s0008g01340  -  Unknown protein
## VIT_05s0029g01090  -  Alpha 1,3-glucosidase
## VIT_07s0205g00070  -  Phosphoenolpyruvate carboxykinase
## VIT_17s0000g03040  -  Chaperonin
## VIT_17s0000g02130  -  Transposase, IS4
## VIT_08s0056g01600  -  No hit
## VIT_00s0445g00020  -  Lipoic acid synthase
## VIT_09s0002g00350  -  Proteasome 20S alpha subunit E2 (PAE2)
## VIT_02s0025g03510  -  Bromo-adjacenty (BAH) domain-containing protein
## VIT_07s0031g01970  -  Proteasome 26S regulatory subunit (RPN5)
## VIT_01s0011g04520  -  Programmed cell death 6-interacting protein
## VIT_02s0087g00560  -  Unknown protein
## VIT_06s0061g00410  -  ABC Transporter (VvGCN4 - VvABCF4)
## VIT_04s0008g04600  -  Expansin family protein (EXPR3)
## VIT_07s0141g00520  -  Serine carboxypeptidase 1 precursor
## VIT_01s0010g02900  -  Proteasome 20S beta subunit E1 (PBE1) (PRCE)
## VIT_13s0067g02990  -  Superoxide dismutase, Fe-Mn family
## VIT_05s0020g04160  -  Stem-specific protein TSJT1
## VIT_18s0001g01640  -  UDP-sugar pyrophospharylase
## VIT_14s0006g00990  -  DNA repair protein Rad4 family
## VIT_10s0003g01860  -  RKF1 (receptor-like kinase in flowers 1)
## VIT_11s0118g00820  -  D111/G-patch domain-containing protein
## VIT_05s0094g00900  -  Mitogen-activated Protein Kinase (VvMPK5)
## VIT_00s0850g00010  -  Unknown protein
## VIT_15s0024g00280  -  Na+/H+ antiporter, isoform 6
## VIT_09s0002g00980  -  Proteasome 20S alpha subunit A1
## VIT_04s0023g00860  -  Short-chain dehydrogenase/reductase
## VIT_00s0187g00220  -  Partial optokinetic response b
## VIT_05s0020g00380  -  Dihydrolipoyl dehydrogenase
```

```
## VIT_05s0020g00790  -  HAT dimerisation domain-containing protein
## VIT_05s0062g01180  -  Importin beta-2
## VIT_11s0037g00990  -  ATPase AFG1
## VIT_05s0051g00640  -  Purple acid phosphatase 23- ATPAP23/PAP23
## VIT_06s0080g00860  -  DCP1 (decapping 1)
## VIT_03s0038g00880  -  Amidophosphoribosyltransferase 2
```

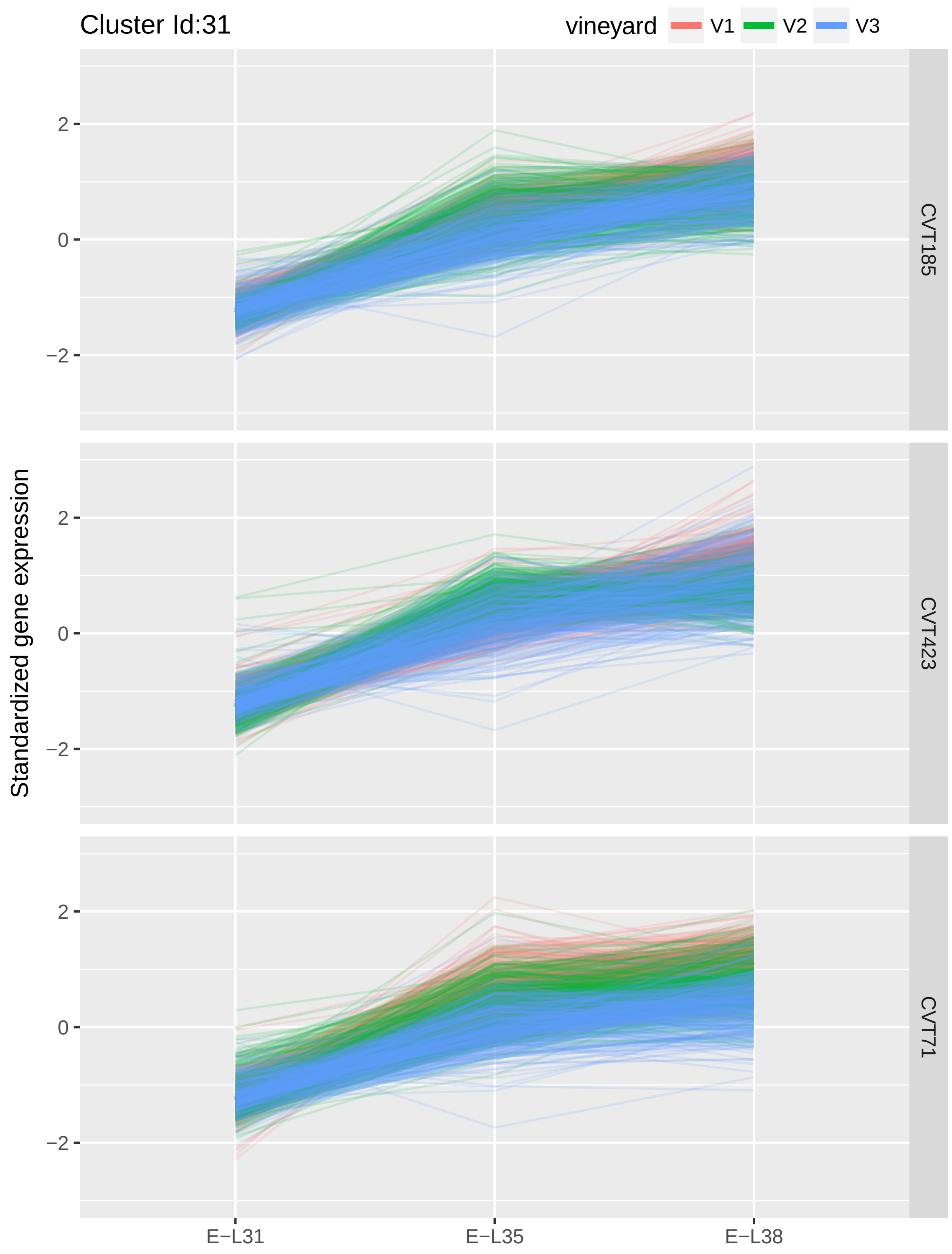

Cluster Id:31
vineyard
V1
V2
V3
Standardized gene expression
CVT185
CVT423
CVT71
2
0
−2
E−L31
E−L35
E−L38

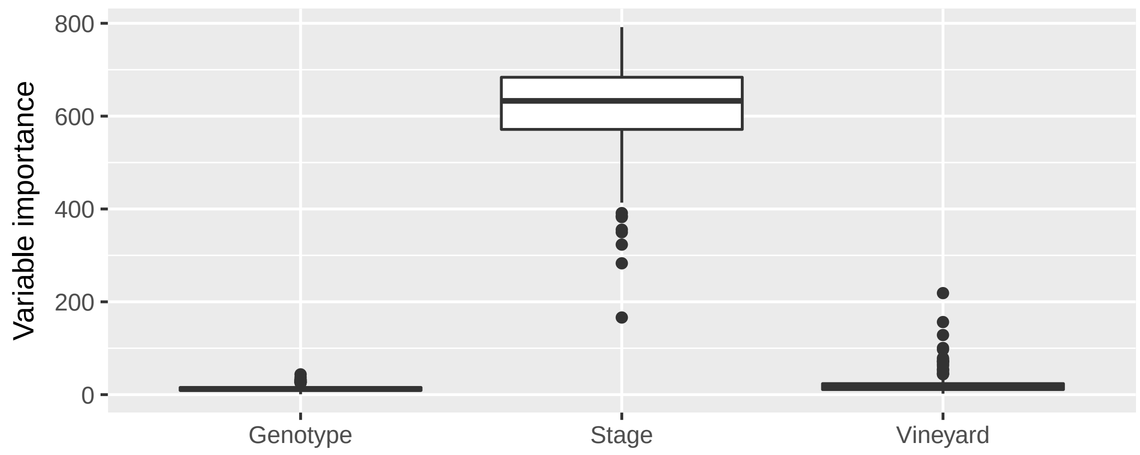

Variable importance
800
600
400
200
0
Genotype
Stage
Vineyard

Cluster no. 32

```
## Number of genes in the cluster: 59
## Homogeneity Index:    0.85
## Variable importance for Stage:    Median =  278.8  - Rank =  74
## Variable importance for Clone:    Median =  20.51  - Rank =  44
## Variable importance for Vineyard: Median =  41.12  - Rank =  46
##
## Gene ID               Gene Annotation
## VIT_18s0122g00860  -  Unknown protein
## VIT_14s0108g01500  -  APG6/CLPB-P/CLPB3
## VIT_18s0041g01230  -  Heat shock protein 70
## VIT_06s0004g00240  -  Chaperonin GroEL
## VIT_19s0014g03470  -  Unknown protein
## VIT_16s0022g00510  -  Heat shock 22 kDa protein
## VIT_12s0028g03470  -  Haloacid dehalogenase hydrolase
## VIT_13s0019g01030  -  Binding
## VIT_06s0061g00270  -  Chaperonin GroEL
## VIT_09s0002g08100  -  Calcineurin
## VIT_11s0016g04080  -  Multiprotein-bridging factor 1c MBF1C
## VIT_12s0055g00900  -  RAE1 RNA export 1 homolog
## VIT_13s0019g02850  -  Heat shock protein 17.6 kDa class I
## VIT_11s0037g00510  -  Heat shock protein 70
## VIT_04s0008g05870  -  CLPB-M/CLPB4/HSP98.7
## VIT_04s0008g01590  -  Heat shock protein 17.6 kDa class II
## VIT_04s0008g01110  -  Heat shock transcription factor A6B
## VIT_16s0050g01150  -  Heat shock protein 90-1
## VIT_01s0010g02290  -  Heat shock protein 26a, chloroplast
## VIT_08s0007g06710  -  Aha1 domain-containing protein
## VIT_13s0019g02770  -  Heat shock protein 17.6 kDa class I
## VIT_05s0020g03330  -  Heat shock protein 70
## VIT_17s0000g07190  -  Heat shock protein 101
## VIT_00s0179g00150  -  Heat shock transcription factor A6B
## VIT_06s0080g00870  -  Coiled-coil domain-containing protein 94
## VIT_07s0005g01980  -  Glycosyl transferase family 8 protein
## VIT_01s0011g04990  -  Chaperonin
## VIT_13s0019g03160  -  Heat shock protein 17.6 kDa class I
## VIT_13s0047g00110  -  Ripening regulated protein DDTFR8
## VIT_00s0194g00190  -  No hit
## VIT_19s0014g02100  -  Unknown protein
## VIT_13s0019g02740  -  Heat shock protein 17.6 kDa class I
## VIT_13s0019g02840  -  Heat shock protein 18.2 kDa class I
## VIT_06s0004g05770  -  Heat shock protein 17.6 kDa class I
## VIT_05s0102g00620  -  No hit
## VIT_13s0019g02780  -  Heat shock protein 17.6 kDa class I
## VIT_05s0102g00600  -  No hit
## VIT_08s0058g00210  -  Chaperone
## VIT_18s0041g00280  -  T-complex protein 11
## VIT_04s0008g01490  -  Heat shock protein 17.6 kDa class II
## VIT_13s0019g03000  -  Heat shock protein 17.6 kDa class I
## VIT_13s0019g03090  -  Heat shock protein class I (HSP17.8-CI)
## VIT_02s0025g04060  -  Rab/Ypt GTPase Ara4-interacting protein
## VIT_03s0088g00320  -  Peptidase M50
## VIT_16s0050g02220  -  Acidic endochitinase (CHIB1)
## VIT_07s0005g01970  -  Galactinol synthase
## VIT_04s0043g00700  -  PTAC3 (plastid transcriptionally active3)
```

```
## VIT_13s0019g00860  -  Small heat-shock protein HSP17.5 Cytosolic class I
## VIT_13s0019g02930  -  Heat shock protein 17.6 kDa class I
## VIT_04s0008g04740  -  GCN5 N-acetyltransferase (GNAT)
## VIT_00s0189g00050  -  No hit
## VIT_18s0089g01270  -  Heat shock protein precursor 22.0 kDa class IV
## VIT_05s0077g01920  -  No hit
## VIT_17s0000g08090  -  No hit
## VIT_07s0031g02750  -  CBS domain containing protein
## VIT_17s0000g04010  -  Actin-related protein 4
## VIT_12s0035g02150  -  ferric reduction oxidase 7 FRO7
## VIT_19s0085g01050  -  Heat shock protein 17.6 kDa class I
## VIT_16s0100g01300  -  Chromo; Helix-hairpin-helix motif, class 2
```

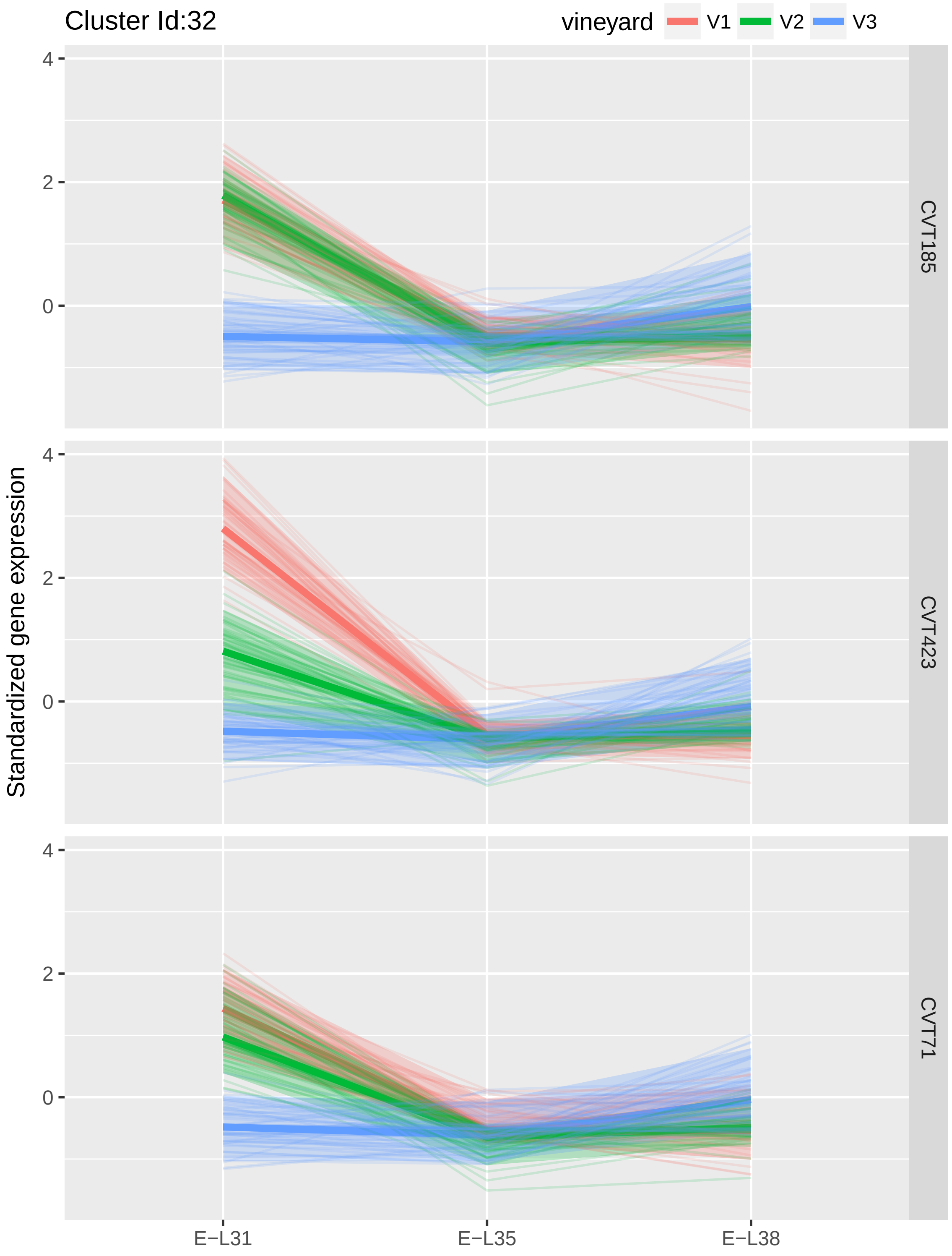
Cluster Id:32
vineyard
V1
V2
V3
Standardized gene expression
4
2
0
CVT185
CVT423
CVT71
E−L31
E−L35
E−L38

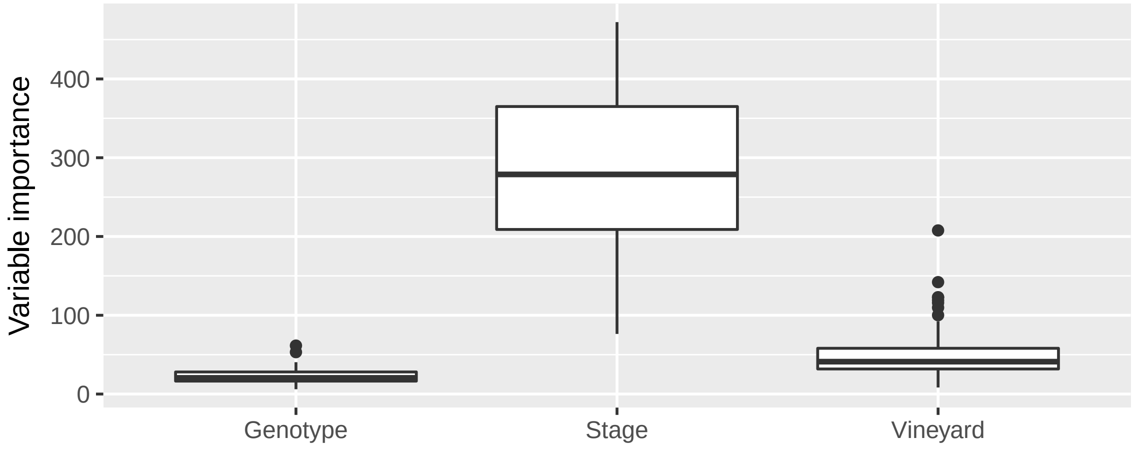
Variable importance
400
300
200
100
0
Genotype
Stage
Vineyard

Cluster no. 33

```
## Number of genes in the cluster: 175
## Homogeneity Index:    0.69
## Variable importance for Stage:    Median =  375.3  - Rank =  61
## Variable importance for Clone:    Median =  24.84  - Rank =  22
## Variable importance for Vineyard: Median =  39.06  - Rank =  50
##
## Gene ID               Gene Annotation
## VIT_01s0011g05010  -  Phytanoyl-CoA dioxygenase (PhyH)
## VIT_05s0077g01200  -  Unknown protein
## VIT_03s0017g01640  -  Cysteine--tRNA ligase.
## VIT_04s0023g03130  -  Histone H1
## VIT_01s0146g00520  -  Sterile alpha motif (SAM) domain-containing
## VIT_02s0025g05050  -  Zf A20 and AN1 domain-containing stress-associated protein 1
## VIT_07s0104g00240  -  Unknown protein
## VIT_09s0018g01660  -  Biopterin transport-related protein BT1
## VIT_12s0057g01500  -  Calcium exchanger 5
## VIT_14s0128g00370  -  Seryl-tRNA synthetase
## VIT_00s0186g00020  -  SUM2 (small ubiquitin-like modifier 2)
## VIT_06s0009g01570  -  C2H2 zinc-finger protein SERRATE (SE)
## VIT_00s0361g00080  -  Translation initiation factor eIF-4B
## VIT_00s0260g00070  -  FK506-binding protein genes family (VvFKBPa)
## VIT_13s0019g04070  -  MLO1
## VIT_07s0005g02770  -  DNAJ heat shock N-terminal domain-containing
## VIT_18s0001g15670  -  Cytoplasm protein
## VIT_07s0130g00370  -  Smr protein/MutS2 C-terminal
## VIT_02s0033g01190  -  Unknown protein
## VIT_18s0072g01170  -  Phosphoribosylanthranilate transferase
## VIT_02s0025g05080  -  Zf A20 and AN1 domain-containing stress-associated protein 1
## VIT_01s0011g02950  -  Zinc finger (C3HC4-type ring finger)
## VIT_19s0015g01040  -  No hit
## VIT_17s0000g01540  -  Elongation factor 1-beta
## VIT_05s0020g04980  -  Meprin and TRAFy domain-containing protein
## VIT_01s0026g00920  -  Unknown protein
## VIT_15s0021g02240  -  3-hydroxyisobutyryl-CoA hydrolase
## VIT_02s0236g00120  -  Unknown
## VIT_00s0769g00010  -  FK506-binding protein genes family (VvFKBP62)
## VIT_15s0021g01840  -  Cationic amino acid transporter 9
## VIT_07s0031g03040  -  Calmodulin-binding protein
## VIT_11s0016g05750  -  HAT transposase
## VIT_12s0035g01130  -  Elongation factor 1-gamma
## VIT_06s0004g02220  -  Heterogeneous nuclear ribonucleoprotein
## VIT_10s0003g03510  -  HSP20 chaperone
## VIT_19s0027g00760  -  Ribosome assembly protein 1
## VIT_01s0026g02110  -  Ribosomal protein S9 (RPS9C) 40S
## VIT_16s0100g00300  -  Sorbitol dehydrogenase
## VIT_18s0041g00420  -  Patellin-5
## VIT_07s0031g00530  -  MAPKKK21
## VIT_08s0007g04710  -  Splicing factor 3A subunit 2
## VIT_08s0040g03390  -  Unknown
## VIT_14s0081g00240  -  Phosphoribosylaminoimidazole carboxylase
## VIT_01s0011g04050  -  Nucleotidyltransferase
## VIT_18s0001g01780  -  Wound-responsive protein
## VIT_01s0010g01160  -  Zinc finger (DHHC type)
## VIT_03s0038g00620  -  Zinc knuckle
```

```
## VIT_05s0077g00710  -  flowering time control protein FCA
## VIT_01s0011g05630  -  Ubiquitin-conjugating enzyme E2 variant 1
## VIT_19s0027g01940  -  No hit
## VIT_12s0028g01040  -  Protein kinase
## VIT_14s0006g00660  -  No hit
## VIT_00s2837g00010  -  Histone H3
## VIT_00s0187g00250  -  Unknown
## VIT_03s0091g00820  -  Radical SAM domain-containing protein
## VIT_01s0150g00600  -  Nucleotide exchange factor SIL1
## VIT_11s0016g02460  -  Pentatricopeptide (PPR) repeat-containing protein
## VIT_19s0015g00920  -  Heterogeneous nuclear ribonucleoprotein A1/A3
## VIT_12s0034g00480  -  No hit
## VIT_05s0102g00380  -  Unknown
## VIT_11s0118g00680  -  Ribosomal protein S11 (RPS11A) 40S
## VIT_13s0064g00010  -  F-box family protein / WD-40 repeat family protein
## VIT_08s0056g00270  -  Unknown protein
## VIT_11s0037g00430  -  F-box protein (FBL19)
## VIT_03s0063g00940  -  CRK (CDPK-related kinase)
## VIT_00s0958g00020  -  FK506-binding protein genes family (VvFKBPb)
## VIT_02s0154g00560  -  No hit
## VIT_18s0001g14330  -  No hit
## VIT_03s0017g02160  -  Suppressor of FRIGIDA4
## VIT_13s0019g03660  -  Unknown protein
## VIT_04s0023g01750  -  TVLP1
## VIT_09s0002g07820  -  Quinone oxidoreductase
## VIT_17s0000g09110  -  Ribosomal protein L27A (RPL27aC) 60S
## VIT_11s0037g01020  -  Tetratricopeptide repeat (TPR)-containing
## VIT_13s0019g03540  -  Regulator of nonsense transcripts 2 UPF2
## VIT_00s0207g00060  -  Unknown protein
## VIT_19s0015g00500  -  1,4-alpha-D-glucan maltohydrolase
## VIT_15s0024g00700  -  Lysyl-tRNA synthetase
## VIT_00s0179g00190  -  Transcription factor jumonji (jmjC) domain-containing protein
## VIT_08s0040g01960  -  Transducin protein
## VIT_16s0039g00690  -  D111/G-patch domain-containing protein
## VIT_18s0001g08140  -  Enhanced silencing phenotype 4 ESP4 symplekin
## VIT_13s0064g00730  -  Actin-related protein 7
## VIT_06s0009g01030  -  BTB-POZ and math Domain 4 ATBPM4
## VIT_19s0015g00870  -  Oligopeptide transporter 1
## VIT_13s0067g03060  -  Methionyl-tRNA synthetase
## VIT_17s0000g10190  -  Histone acetyltransferase ELP3
## VIT_06s0004g06030  -  Calcium/calmodulin-regulated receptor kinase
## VIT_04s0008g00820  -  KH domain-containing protein
## VIT_19s0085g00520  -  Nuclear transcription factor Y subunit C-2
## VIT_14s0068g00340  -  Transcription initiation factor TFIID subunit 8
## VIT_06s0004g06070  -  Unknown protein
## VIT_14s0060g01150  -  Serine/threonine protein phosphatase
## VIT_03s0063g02460  -  Transcription termination factor mitochondrial mTERF
## VIT_10s0003g03780  -  Unknown protein
## VIT_17s0000g09690  -  L-isoaspartate-O-methyl transferase
## VIT_00s0187g00200  -  Unknown protein
## VIT_14s0128g00840  -  Polymerase zeta
## VIT_01s0011g04360  -  DEAH (Asp-Glu-Ala-His) box polypeptide 36
## VIT_14s0081g00500  -  Histone H1
## VIT_19s0014g00350  -  Zinc finger (B-box type)
## VIT_08s0007g04160  -  Uridylate kinase
```

```
## VIT_05s0094g00550  -  Stearoyl-acyl-[acyl-carrier-protein] desaturase
## VIT_03s0088g00390  -  DnaJ homolog, subfamily C, member 17
## VIT_00s0780g00010  -  6-phosphofructokinase 2
## VIT_07s0031g03030  -  Calmodulin-binding protein
## VIT_08s0040g01850  -  Unknown protein
## VIT_18s0001g07060  -  Phosphoribosylaminoimidazole-succinocarboxamide synthase.
## VIT_02s0234g00120  -  FUS5
## VIT_19s0014g01590  -  Unknown protein
## VIT_18s0001g12160  -  Unknown protein
## VIT_17s0000g00990  -  Switching protein 3A ATSWI3A
## VIT_17s0119g00330  -  Unknown protein
## VIT_10s0003g02610  -  Unknown protein
## VIT_04s0023g02610  -  Epoxide hydrolase 2
## VIT_04s0023g01690  -  No hit
## VIT_06s0004g00710  -  Pre-mRNA splicing factor cwc22
## VIT_07s0005g03510  -  SAP protein BP-73 E1-E2_ATPase
## VIT_06s0004g04090  -  Ribosomal protein L23A (RPL23aA) 60S
## VIT_05s0020g03500  -  Prefoldin KE2
## VIT_13s0064g00870  -  FRA10AC1
## VIT_08s0058g01300  -  High-level expression of sugar-inducible like 1
## VIT_09s0054g00610  -  F-box domain containing protein
## VIT_17s0000g07600  -  Unknown protein
## VIT_11s0065g00380  -  Pentatricopeptide (PPR) repeat-containing protein
## VIT_07s0031g02850  -  UVB-resistance protein UVR8
## VIT_04s0008g01760  -  PEX10 (peroxin 10)
## VIT_18s0001g07770  -  Unknown protein
## VIT_07s0031g02860  -  Elongation factor EF-2
## VIT_11s0016g02130  -  CwfJ protein
## VIT_16s0115g00220  -  Myosin-like protein XIA
## VIT_19s0085g01060  -  Polypeptide N-acetylglucosaminyltransferase
## VIT_19s0014g04770  -  RNA recognition motif (RRM)-containing protein
## VIT_18s0117g00230  -  Unknown protein
## VIT_03s0038g01820  -  Translocon-associated protein alpha TRAP complex
## VIT_04s0008g06810  -  U2-associated protein SR140
## VIT_18s0001g10300  -  basic helix-loop-helix (bHLH) family
## VIT_01s0010g02420  -  DNA-binding protein GT-1-related
## VIT_10s0042g01060  -  Unknown protein
## VIT_13s0019g00850  -  U3 small nucleolar ribonucleoprotein protein IMP4
## VIT_05s0029g00440  -  GRP2 (cold, circadian rhythm, and RNA binding 2)
## VIT_17s0000g03460  -  Unknown protein
## VIT_04s0023g03770  -  DNA binding
## VIT_08s0056g01250  -  Transcription factor IIB (TFIIB) family protein
## VIT_16s0050g00790  -  Hydroxyproline-rich glycoprotein
## VIT_00s0179g00180  -  Transcription factor jumonji (jmjC) domain-containing protein
## VIT_16s0039g01550  -  Unknown protein
## VIT_08s0056g01500  -  Unknown protein
## VIT_05s0020g03070  -  Cyclin-T1-4
## VIT_05s0049g01960  -  C2 domain containing protein
## VIT_06s0004g02460  -  Myb family
## VIT_06s0080g00440  -  Ribosomal protein S9
## VIT_04s0023g01400  -  5-azacytidine resistance
## VIT_19s0014g02260  -  Unknown protein
## VIT_06s0004g03640  -  Trans-2-enoyl-CoA reductase
## VIT_07s0031g01210  -  Apoptotic chromatin condensation inducer 1
```

```
## VIT_00s0672g00010  -  Translation initiation factor eIF-2B gamma subunit
## VIT_04s0008g03890  -  GTP binding protein EngB
## VIT_17s0119g00310  -  Unknown protein
## VIT_19s0085g01080  -  Polypeptide N-acetylglucosaminyltransferase
## VIT_18s0001g00640  -  Pentatricopeptide (PPR) repeat-containing protein
## VIT_17s0000g09220  -  MAK16 protein
## VIT_10s0003g00810  -  DEAH (Asp-Glu-Ala-His) box polypeptide 35
## VIT_01s0010g02570  -  EMB2421 (embryo defective 2421) monooxygenase
## VIT_16s0039g01430  -  ATORC3/ORC3 (Origin recognition complex protein 3)
## VIT_00s0279g00040  -  Heat shock protein binding
## VIT_09s0002g07620  -  NADPH quinone oxidoreductase
## VIT_17s0000g06030  -  Nucleosome assembly protein (NAP-related) NRP2
## VIT_14s0060g02290  -  Splicing factor, arginine/serine-rich 1/9
## VIT_14s0083g00730  -  Unknown protein
## VIT_05s0094g00440  -  Sas10/U3 ribonucleoprotein (Utp)
## VIT_01s0137g00580  -  Serine/threonine-protein kinase AFC2
## VIT_13s0074g00210  -  Pyruvate kinase
## VIT_04s0023g01710  -  No hit
## VIT_13s0067g03170  -  Transcription elongation factor S-II
```

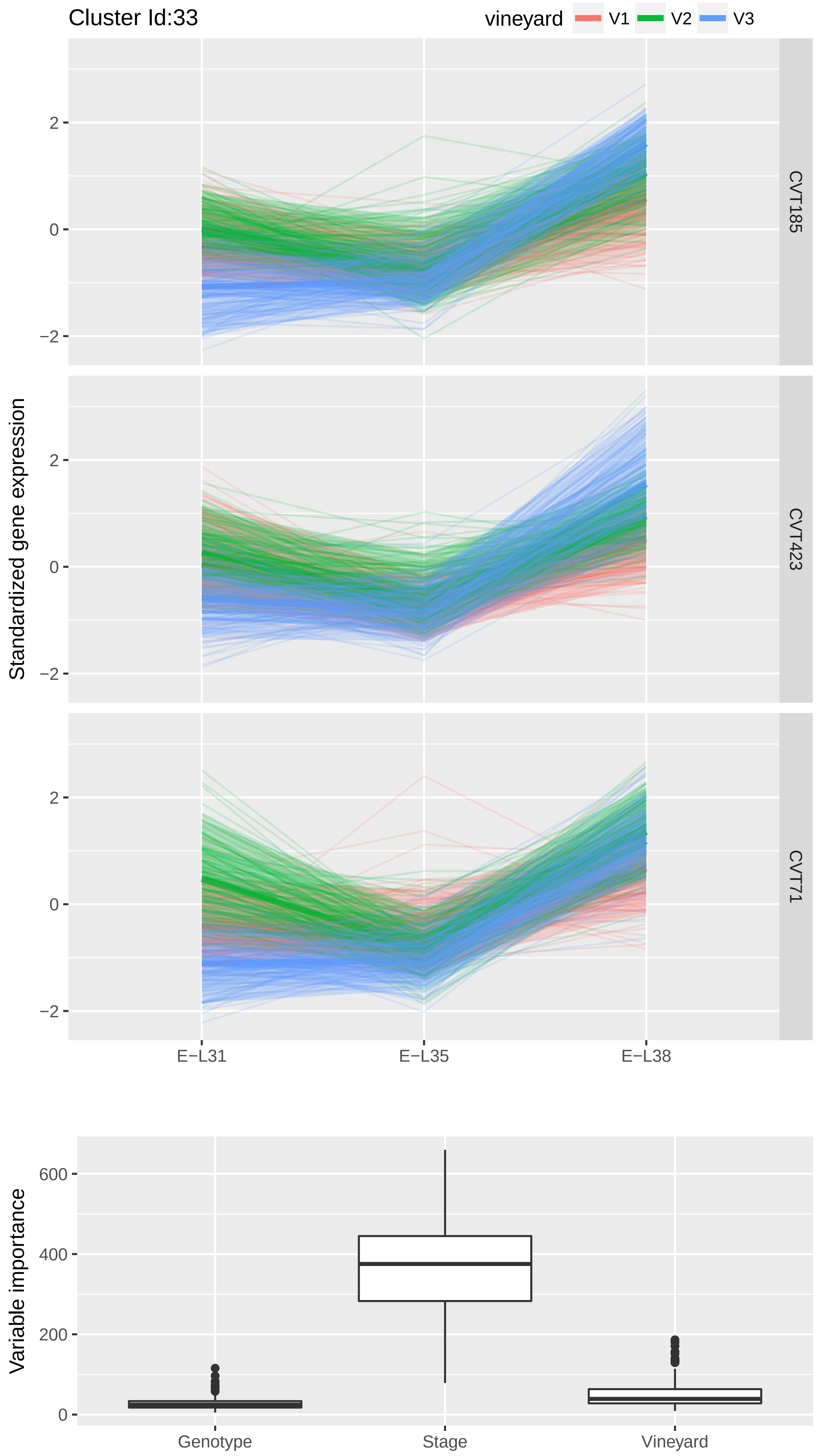
Cluster Id:33
vineyard
V1
V2
V3
Standardized gene expression
CVT185
CVT423
CVT71
2
0
−2
E−L31
E−L35
E−L38
Variable importance
600
400
200
0
Genotype
Stage
Vineyard

Cluster no. 34

```
## Number of genes in the cluster: 425
## Homogeneity Index:    0.81
## Variable importance for Stage:    Median =  650.2  - Rank =  5
## Variable importance for Clone:    Median =  11.24  - Rank =  97
## Variable importance for Vineyard: Median =   12.3  - Rank =  107
##
## Gene ID               Gene Annotation
## VIT_18s0001g03900  -  R protein L6
## VIT_09s0002g06330  -  Partner of SLD five, PSF1
## VIT_09s0070g00600  -  Peroxisomal biogenesis factor 11 (PEX11)
## VIT_19s0085g00050  -  myb domain protein 58
## VIT_14s0006g00250  -  Cysteine-rich repeat secretory protein 60
## VIT_12s0059g02370  -  Translation initiation factor eIF-1
## VIT_00s1232g00020  -  Ribosomal protein L12
## VIT_07s0141g00810  -  Zinc finger, SWIM-type
## VIT_08s0056g01170  -  Zinc finger protein VAR3, chloroplastic
## VIT_11s0016g04180  -  Coatomer protein complex, subunit beta 2
## VIT_03s0097g00590  -  Import receptor subunit TOM40, Mitochondrial
## VIT_18s0001g13890  -  Unknown protein
## VIT_13s0084g00700  -  Unknown protein
## VIT_14s0060g00590  -  Unknown protein
## VIT_07s0031g01240  -  Unknown protein
## VIT_13s0067g02890  -  Unknown protein
## VIT_01s0137g00680  -  No hit
## VIT_16s0039g01030  -  Cell division cycle 20, cofactor of APC complex
## VIT_00s0434g00020  -  Protein phosphatase 2C
## VIT_10s0003g03330  -  Receptor-like serine-threonine protein kinase
## VIT_07s0104g01600  -  MORC family CW-type zinc finger 4
## VIT_14s0066g02380  -  Protein transport SEC13
## VIT_08s0007g06790  -  fb27
## VIT_02s0241g00060  -  Unknown protein
## VIT_05s0102g00690  -  far-red impaired responsive family protein
## VIT_14s0081g00490  -  Deoxyribodipyrimidine photolyase
## VIT_02s0025g04630  -  Homeodomain transcription factor (KNAT7)
## VIT_04s0008g05620  -  Adenine phosphoribosyltransferase
## VIT_19s0015g01690  -  Cysteine desulfuration protein SufE
## VIT_16s0039g01810  -  GCN5 N-acetyltransferase (GNAT)
## VIT_15s0048g02290  -  NAC domain-containing protein (VvNAC54)
## VIT_19s0090g01550  -  Mo25
## VIT_00s1045g00030  -  Unknown
## VIT_04s0008g02980  -  Lupeol synthase
## VIT_08s0040g00360  -  SUVH3 (SU(VAR)3-9 homolog 3)
## VIT_06s0004g06690  -  Ribosomal protein S18 30S
## VIT_04s0008g03720  -  Vacuolar sorting-associated protein (Vps27)
## VIT_05s0102g00290  -  Unknown protein
## VIT_19s0090g00690  -  Unknown protein
## VIT_13s0073g00580  -  Small nuclear ribonucleoprotein B and B'
## VIT_14s0030g01260  -  CRK1 protein
## VIT_08s0032g01210  -  Mal d 1-associated protein
## VIT_12s0028g02510  -  DNA-binding bromodomain-containing protein
## VIT_07s0005g06680  -  DNA binding
## VIT_04s0008g02630  -  Unknown protein
## VIT_12s0055g00740  -  fertility restorer salt-inducible
## VIT_13s0101g00090  -  Unknown protein
```

```
## VIT_19s0090g00590  -  myb domain protein 101
## VIT_18s0001g09490  -  Metal transporter Nramp2
## VIT_18s0001g15020  -  DnaJ homolog, subfamily A, member 4
## VIT_06s0004g05880  -  ABI3-interacting protein 3 [Callitropsis nootkatensis]
## VIT_05s0049g01770  -  Translation initiation factor eIF-4E
## VIT_13s0019g02030  -  HAT dimerisation domain-containing protein
## VIT_00s0379g00060  -  SAP domain-containing protein
## VIT_16s0039g02450  -  PWWP domain-containing protein
## VIT_00s0391g00050  -  Unknown protein
## VIT_00s1351g00010  -  Polyadenylate-binding protein 2
## VIT_00s0199g00170  -  Cysteine desulfurase
## VIT_18s0072g00100  -  Unknown protein
## VIT_02s0025g04850  -  CYP76B1
## VIT_01s0137g00370  -  No hit
## VIT_13s0019g04920  -  Protein phosphatase 2 (formerly 2A), regulatory subunit B'
## VIT_07s0005g01630  -  Pentatricopeptide (PPR) repeat-containing protein
## VIT_17s0000g07350  -  Exocyst subunit EXO70 E2
## VIT_16s0039g01900  -  Myb KAN2 (KANADI 2)
## VIT_17s0000g02760  -  Peroxin-3 (PEX3)
## VIT_05s0020g04120  -  DnaJ homolog, subfamily A, member 1
## VIT_11s0016g04820  -  Agenet domain-containing protein
## VIT_07s0005g03800  -  Unknown protein
## VIT_13s0047g01240  -  CTV.12
## VIT_12s0059g02770  -  Shoot gravitropism 7
## VIT_10s0003g03860  -  KH domain-containing protein
## VIT_07s0005g02190  -  Zinc finger (C2H2 type) family
## VIT_12s0034g01950  -  Legumin
## VIT_07s0129g01020  -  MRG
## VIT_13s0067g03150  -  Rhodanese like protein
## VIT_11s0016g05580  -  Methyl-CpG-binding domain-containing protein
## VIT_10s0003g00290  -  Homeobox-1
## VIT_18s0001g04440  -  Co-chaperone protein DnaJ
## VIT_19s0090g00080  -  Ubiquitin-conjugating enzyme E2 variant
## VIT_03s0063g02000  -  IBR domain-containing protein
## VIT_03s0132g00310  -  Myosin-like protein XIK
## VIT_13s0067g00710  -  Nascent polypeptide associated complex alpha chain
## VIT_07s0255g00010  -  Hydroxyproline-rich glycoprotein family protein
## VIT_08s0007g08720  -  Pentatricopeptide (PPR) repeat-containing protein
## VIT_11s0065g01010  -  Methyl-CpG-binding domain 2 MBD02
## VIT_15s0021g02650  -  Lipase GDSL
## VIT_05s0102g00760  -  Unknown protein
## VIT_06s0061g01410  -  TRNA isopentenyltransferase
## VIT_06s0004g04540  -  Zinc finger (DHHC type) family
## VIT_13s0067g03350  -  Ribosomal protein L4/L1 (RPL4A) 60S
## VIT_08s0058g01050  -  Unknown protein
## VIT_19s0014g02870  -  Cellulase
## VIT_18s0001g07520  -  Mechanosensitive ion channel MSCS-like 2
## VIT_07s0005g01000  -  Syntaxin 16
## VIT_08s0007g05080  -  Unknown protein
## VIT_09s0002g03990  -  Unknown protein
## VIT_00s0598g00020  -  Alkaline phytoceramidase
## VIT_05s0020g03870  -  MLK/Raf-related protein kinase 1
## VIT_09s0054g01820  -  Indeterminate(ID)-domain 2
## VIT_06s0004g01830  -  NIMA-related protein kinase
## VIT_15s0046g00940  -  Ubiquitin family
```

```
## VIT_03s0088g01000  -  Unknown protein
## VIT_18s0001g10560  -  Myosin heavy chain
## VIT_02s0025g00490  -  Unknown protein
## VIT_15s0021g01810  -  Armadillo/beta-catenin repeat family protein
## VIT_12s0035g01980  -  PHD finger protein alfin
## VIT_06s0009g03120  -  Cytochrome P450, family 79, subfamily A, polypeptide 2
## VIT_08s0040g00620  -  Unknown protein
## VIT_16s0039g02760  -  RNA polymerase II mediator complex subunit SOH1
## VIT_12s0034g00960  -  Unknown
## VIT_14s0030g01250  -  HASTY
## VIT_19s0027g01670  -  Cyclin-T1-4
## VIT_10s0003g04680  -  Unknown protein
## VIT_09s0002g08670  -  Acetylornithine aminotransferase
## VIT_06s0004g04730  -  CAX  interacting  protein 4
## VIT_12s0034g01970  -  Cupin
## VIT_13s0019g01270  -  Phytoene synthase
## VIT_18s0001g08540  -  Molecular chaperone DnaJ
## VIT_11s0016g05120  -  DnaJ homolog, subfamily B, member 12
## VIT_17s0000g03250  -  Unknown protein
## VIT_03s0038g01810  -  XAP5
## VIT_06s0004g01210  -  PRLI-interacting factor A
## VIT_03s0180g00010  -  Cysteine proteinase
## VIT_07s0104g00560  -  U6 snRNA-associated Sm-like protein LSm2
## VIT_07s0031g02350  -  Heat shock protein binding
## VIT_07s0005g03010  -  Triacylglycerol lipase
## VIT_19s0014g02250  -  Zinc-binding protein
## VIT_03s0097g00560  -  No hit
## VIT_11s0103g00530  -  Oxysterol binding protein
## VIT_11s0016g02830  -  Gamma-glutamyltranspeptidase
## VIT_15s0046g02500  -  Unknown protein
## VIT_12s0057g01110  -  Nudix hydrolase 2
## VIT_18s0001g09060  -  Unknown protein
## VIT_04s0008g03750  -  Translation initiation factor eIF-3 subunit 6
## VIT_14s0108g01140  -  Emp24/gp25L/p24 family protein
## VIT_05s0102g00480  -  Pre-mRNA-splicing helicase
## VIT_09s0054g01810  -  ATP-dependent RNA helicase DDX23/PRP28
## VIT_05s0124g00510  -  Oxalyl-CoA decarboxylase [Vitis vinifera]
## VIT_19s0027g00790  -  Transglycosylase SLT domain containing protein
## VIT_15s0048g00710  -  Zinc finger (DHHC type)
## VIT_08s0007g09030  -  DnaJ homolog, subfamily A, member 5
## VIT_05s0049g01040  -  Pre-mRNA-processing factor SLU7
## VIT_09s0002g02610  -  Lectin jacalin
## VIT_13s0067g01780  -  Histidine acid phosphatase
## VIT_13s0139g00170  -  Orotidine-5'-phosphate decarboxylase.
## VIT_19s0015g01950  -  COP9 signalosome complex subunit 6a
## VIT_06s0080g00880  -  Nicalin precursor
## VIT_04s0069g00870  -  Myosin-related
## VIT_16s0039g00880  -  CYP89H3
## VIT_18s0001g05110  -  DNA2-NAM7 helicase
## VIT_11s0052g00030  -  PEP (pepper)
## VIT_07s0191g00110  -  Unknown protein
## VIT_00s1221g00010  -  Unknown protein
## VIT_00s1428g00020  -  No hit
## VIT_03s0063g00080  -  E3 ubiquitin-protein ligase MARCH6
## VIT_09s0002g00830  -  Unknown protein
```

```
## VIT_11s0016g00640  -  Auxin response factor 11 ARF19
## VIT_06s0009g01140  -  Amino acid permease
## VIT_16s0013g02150  -  No hit
## VIT_03s0038g00420  -  HEN4 (HUA enhancer 4)
## VIT_06s0004g00580  -  Hydrolase, alpha/beta fold family protein
## VIT_05s0124g00030  -  Epsin protein
## VIT_10s0003g03110  -  Cytochrome B561
## VIT_06s0004g04260  -  RNA-binding protein
## VIT_11s0016g01750  -  Unknown protein
## VIT_13s0158g00230  -  IMP dehydrogenase/GMP reductase
## VIT_14s0060g00910  -  Metallopeptidase M24
## VIT_00s0261g00010  -  Unknown protein
## VIT_17s0000g06820  -  Zinc finger (C2H2 type) family
## VIT_00s0317g00150  -  Pectate lyase
## VIT_04s0023g01470  -  Splicing factor 3B subunit 4
## VIT_01s0146g00140  -  GYF domain-containing protein
## VIT_04s0069g00760  -  Ubiquitin-specific protease 15
## VIT_00s0349g00060  -  La domain-containing protein
## VIT_05s0102g01050  -  No hit
## VIT_04s0044g00610  -  Unknown protein
## VIT_01s0011g04230  -  Ubiquitin family
## VIT_11s0016g05800  -  Unknown
## VIT_09s0002g07020  -  TPR4/WSIP2 (topless-related 4)
## VIT_01s0011g02760  -  No hit
## VIT_04s0008g03060  -  Transducin protein
## VIT_04s0008g05330  -  DEAD  box  RNA helicase RH15
## VIT_02s0012g01750  -  NHL8 (NDR1/HIN1-like 8)
## VIT_17s0000g00080  -  Pentatricopeptide (PPR) repeat-containing protein
## VIT_17s0000g10250  -  Proteasome 26S regulatory subunit (RPN7)
## VIT_02s0012g00630  -  Myb family
## VIT_14s0006g00420  -  Unknown protein
## VIT_14s0060g01520  -  GATA transcription factor 16
## VIT_18s0001g06050  -  Phosphotyrosyl phosphatase activator PTPA (GB:X73478)
## VIT_12s0028g02020  -  Multiprotein-bridging factor 1a MBF1A
## VIT_06s0004g07350  -  Substrate carrier, Mitochondrial
## VIT_08s0007g00050  -  EMB1674 (embryo defective 1674) kinase interacting family protein
## VIT_07s0151g01030  -  GCN5 N-acetyltransferase (GNAT)
## VIT_17s0000g05450  -  ELF5 (early flowering 5)
## VIT_06s0004g04390  -  UDP-glucuronic acid decarboxylase 1
## VIT_12s0059g01550  -  Triacylglycerol/steryl ester lipase
## VIT_11s0016g03670  -  Zinc finger (Ran-binding)
## VIT_04s0008g04790  -  ABC Transporter (VvPDR29 - VvABCG59)
## VIT_13s0064g00920  -  Zinc finger (CCCH-type) family protein
## VIT_07s0031g00730  -  Unknown protein
## VIT_08s0007g04400  -  Low expression of osmotically responsive genes 1
## VIT_14s0083g00300  -  RNA polymerase sigma factor
## VIT_04s0008g04700  -  Histone H2A variant 3 HTA9
## VIT_18s0041g00730  -  Peptide chain release factor eRF subunit 1
## VIT_17s0000g05430  -  Aminoacyl-tRNA synthetase
## VIT_05s0020g02280  -  Unknown protein
## VIT_13s0064g01210  -  Zf A20 and AN1 domain-containing stress-associated protein 2
## VIT_17s0000g02860  -  G1 to S phase transition protein
## VIT_18s0001g12240  -  Peptide chain release factor eRF subunit 1
## VIT_00s0218g00030  -  ELO1 (ELONGATA 1)
## VIT_19s0027g00310  -  Pentatricopeptide (PPR) repeat-containing
```

```
## VIT_05s0124g00010  -  Epsin-like protein
## VIT_05s0094g00450  -  Ribosomal protein L51/S25/CI-B8
## VIT_04s0008g01130  -  Solute carrier family 25
## VIT_07s0005g00170  -  Rubber elongation factor (REF)
## VIT_18s0089g00620  -  Glycerophosphoryl diester phosphodiesterase
## VIT_07s0255g00130  -  Unknown protein
## VIT_12s0028g02170  -  ATP synthase protein I
## VIT_04s0008g01170  -  FtsH protease that is localized to the mitochondrion
## VIT_18s0001g13650  -  Amine oxidase
## VIT_00s0207g00050  -  No hit
## VIT_19s0085g00990  -  Unknown protein
## VIT_13s0064g00640  -  PHD finger transcription factor
## VIT_07s0104g00870  -  WD40
## VIT_17s0000g09750  -  Transducin protein
## VIT_17s0000g02310  -  Zinc finger (Ran-binding)
## VIT_00s0199g00130  -  Cysteine desulfurase
## VIT_16s0050g00970  -  Unknown
## VIT_18s0001g05740  -  DNA-binding protein
## VIT_02s0087g00830  -  CBF1 interacting corepressor CIR
## VIT_12s0028g01990  -  Unknown protein
## VIT_01s0011g00580  -  Pentatricopeptide (PPR) repeat-containing protein
## VIT_08s0058g00620  -  Kelch repeat-containing protein
## VIT_19s0015g00930  -  Hydroxyproline-rich glycoprotein
## VIT_19s0015g02590  -  Glutathione S-transferase 25 GSTU25
## VIT_08s0105g00440  -  Selenoprotein
## VIT_10s0003g03060  -  High mobility group HMG1/2
## VIT_12s0035g01890  -  RNA recognition motif (RRM)-containing
## VIT_02s0025g04860  -  CYP76B1
## VIT_11s0052g00080  -  Unknown protein
## VIT_03s0091g00700  -  ATP-dependent Clp protease proteolytic subunit (ClpR1) (nClpP5)
## VIT_01s0150g00040  -  Unknown protein
## VIT_15s0048g01340  -  Nucleotidyltransferase
## VIT_17s0000g08880  -  Transcription factor jumonji, jmjC
## VIT_12s0035g00040  -  Splicing factor 3B subunit 3
## VIT_04s0008g01310  -  MAP3K delta-1 protein kinase
## VIT_05s0049g01380  -  MIF4G domain-containing protein
## VIT_14s0108g01070  -  NAC domain-containing protein (VvNAC11)
## VIT_12s0034g00950  -  Kinetochore protein
## VIT_13s0019g01290  -  Crossover junction endonuclease MUS81
## VIT_14s0060g02260  -  Proteasome 26S AAA-ATPase subunit (RPT5a)
## VIT_09s0002g01640  -  KOW domain-containing transcription factor
## VIT_10s0071g00970  -  D111/G-patch domain-containing protein
## VIT_01s0011g02130  -  Peptidoglycan-binding LysM domain-containing protein
## VIT_09s0018g00610  -  Unknown protein
## VIT_02s0025g02530  -  RabGAP/TBC domain-containing protein
## VIT_18s0072g01010  -  Peptide chain release factor eRF subunit 1
## VIT_11s0037g00130  -  fip1 motif-containing protein
## VIT_19s0090g00060  -  Biopterin transport-related protein BT1
## VIT_07s0151g00500  -  MLK/Raf-related protein kinase 1
## VIT_03s0038g01070  -  Pentatricopeptide (PPR) repeat-containing protein
## VIT_19s0014g03430  -  Amine oxidase  LDL2
## VIT_06s0009g03750  -  CHR5 (chromatin remodeling 5)
## VIT_07s0104g01170  -  U1 small nuclear ribonucleoprotein C
## VIT_05s0020g02850  -  Peroxiredoxin-5
## VIT_07s0005g02730  -  Myb Radialis
```

```
## VIT_14s0066g00890  -  Cytochrome c oxidase subunit Vc
## VIT_18s0076g00310  -  Translation initiation factor eIF-5B
## VIT_04s0008g05820  -  Stromal cell-derived factor 2 protein precursor (SDF2 protein)
## VIT_01s0127g00660  -  Pentatricopeptide (PPR) repeat-containing protein
## VIT_02s0025g02760  -  Ubiquitin thiolesterase 4
## VIT_00s2750g00010  -  Phosphatidate cytidylyltransferase
## VIT_05s0077g00460  -  Zinc knuckle
## VIT_11s0016g05890  -  BSD domain-containing protein
## VIT_14s0083g00590  -  Ribosomal RNA-processing protein 7
## VIT_02s0109g00310  -  flavonoid 3-monooxygenase
## VIT_08s0007g02230  -  Brittle-1, chloroplast precursor
## VIT_07s0005g00230  -  U6 snRNA-associated Sm-like protein LSm4
## VIT_08s0007g01580  -  Unknown protein
## VIT_17s0000g02730  -  myb domain protein 4R1
## VIT_08s0040g02440  -  PWWP domain-containing protein
## VIT_08s0056g01310  -  MEMB11 (Golgi SNARE protein membrin 11)
## VIT_00s0245g00070  -  Heptaprenyl diphosphate synthase component II
## VIT_17s0000g00860  -  Unknown protein
## VIT_04s0008g07000  -  Zinc finger (CCCH-type) family protein
## VIT_06s0004g08180  -  Translation initiation factor eIF-2 beta subunit
## VIT_07s0031g00230  -  E3 ubiquitin-protein ligase MARCH6
## VIT_18s0001g04880  -  Unknown protein
## VIT_10s0003g02740  -  TA9
## VIT_14s0083g00440  -  PHD finger transcription factor
## VIT_05s0094g01370  -  Translation initiation factor eIF-1A
## VIT_06s0004g05150  -  TOC75 (translocon outer membrane complex 75)
## VIT_06s0080g00850  -  Cysteine peptidase
## VIT_08s0007g07430  -  Galactose-1-phosphate uridyl transferase
## VIT_04s0008g03880  -  Adaptor-related protein complex 4, beta 1
## VIT_09s0002g00170  -  Proteasome 26S AAA-ATPase subunit (RPT1a)
## VIT_11s0037g00050  -  Unknown protein
## VIT_02s0087g00950  -  Pentatricopeptide repeat
## VIT_18s0001g09310  -  Transcription initiation factor TFIID subunit 1-A
## VIT_17s0053g00360  -  Pentatricopeptide (PPR) repeat
## VIT_01s0010g03760  -  DNAJ heat shock N-terminal domain-containing protein
## VIT_00s0456g00020  -  S-locus lectin protein kinase
## VIT_11s0016g03550  -  NADP adrenodoxin-like ferredoxin reductase
## VIT_13s0067g03660  -  Unknown protein
## VIT_13s0047g00310  -  C2H2 zinc-finger protein SERRATE (SE)
## VIT_12s0059g01140  -  Unknown protein
## VIT_04s0008g06590  -  Kinetochore protein NUF2
## VIT_15s0021g01730  -  Unknown protein
## VIT_19s0015g00790  -  Importin beta-3
## VIT_01s0010g02150  -  Calcium-dependent protein kinase-related
## VIT_08s0007g06720  -  DNA-3-methyladenine glycosylase (MAG)
## VIT_09s0002g01740  -  Casein kinase 1
## VIT_01s0011g02910  -  Coiled-coil domain containing 130
## VIT_08s0007g05930  -  U4/U6 small nuclear ribonucleoprotein PRP4
## VIT_01s0011g03670  -  Bifunctional nuclease
## VIT_03s0063g00630  -  Transcriptional factor B3
## VIT_10s0071g00740  -  No hit
## VIT_04s0008g03020  -  Serine/threonine/tyrosine kinase DSK2
## VIT_06s0009g00270  -  DnaJ homolog, subfamily C, member 8
## VIT_03s0017g01340  -  Synaptotagmin binding, cytoplasmic RNA interacting
## VIT_09s0002g02550  -  ATP binding / DNA binding
```

```
## VIT_18s0001g13640  -  Amine oxidase
## VIT_14s0006g02240  -  2-hydroxyacyl-CoA lyase 1
## VIT_05s0029g01350  -  RabGAP/TBC domain-containing protein
## VIT_07s0031g00090  -  TRNA-dihydrouridine synthase B
## VIT_07s0031g01200  -  RNase L inhibitor protein
## VIT_04s0008g05040  -  Agenet domain-containing protein
## VIT_14s0006g01020  -  THO complex subunit 4
## VIT_07s0104g01590  -  Cullin-1
## VIT_11s0052g00730  -  Adagio protein 1
## VIT_07s0005g03100  -  Autophagy 9
## VIT_08s0032g00460  -  No hit
## VIT_03s0038g04070  -  Vacuolar protein sorting 9 (VPS9)
## VIT_00s0587g00040  -  Unknown protein
## VIT_11s0052g01370  -  Micro-fibrillar-associated 1, C-terminal
## VIT_11s0016g03980  -  Unknown protein
## VIT_03s0038g02300  -  Proteasome 20S alpha subunit D2 (PAD2) (PRS1) (PRC6)
## VIT_02s0241g00090  -  Translation release factor
## VIT_11s0016g03260  -  Anaphase-promoting complex component APC3
## VIT_18s0001g00860  -  DNA-directed RNA polymerase II subunit A
## VIT_15s0048g01290  -  Sin3 associated polypeptide p18
## VIT_17s0000g06960  -  UDP-glucose 6-dehydrogenase
## VIT_00s0873g00020  -  NADH dehydrogenase subunit 3
## VIT_18s0001g07820  -  No hit
## VIT_17s0000g00400  -  Phosphate carrier protein
## VIT_19s0085g00200  -  Epstein-Barr virus EBNA-1
## VIT_06s0061g00060  -  THUMP domain-containing protein 1
## VIT_04s0008g05780  -  Transcription initiation factor TFIID subunit 5
## VIT_14s0081g00440  -  FLK (flowering locus KH domain)
## VIT_11s0016g01390  -  Replication protein A 70 kDa DNA-binding subunit
## VIT_13s0067g02310  -  Unknown
## VIT_04s0008g02310  -  ABI3-interacting protein 2, AIP2
## VIT_16s0098g01110  -  GIF2 (GRF1- interacting  factor 2)
## VIT_01s0026g00040  -  Pre-mRNA-processing factor 17
## VIT_06s0080g01150  -  Electron transport complex protein RnfC
## VIT_12s0028g01200  -  Zinc finger (C3HC4-type ring finger)
## VIT_09s0070g00590  -  Dihydrolipoyl dehydrogenase
## VIT_07s0005g02700  -  KCO5 (Ca2+ activated outward rectifying K+ channel 5)
## VIT_10s0003g00270  -  Homeobox-1
## VIT_02s0109g00250  -  4-coumarate-CoA ligase
## VIT_15s0021g00330  -  Proton-dependent oligopeptide transport (POT) family protein
## VIT_00s0873g00010  -  Ribosomal protein L2
## VIT_06s0004g03370  -  Unknown protein
## VIT_16s0022g02230  -  Leucine-rich repeat receptor protein kinase EXS
## VIT_08s0007g03010  -  Hydrolase, alpha/beta fold
## VIT_05s0062g01060  -  WD-40 repeat
## VIT_16s0013g02160  -  No hit
## VIT_11s0078g00250  -  Phosphoethanolamine cytidylyltransferase
## VIT_12s0059g01290  -  Unknown protein
## VIT_06s0009g01180  -  UBP1 interacting protein 2a (UBA2a)
## VIT_12s0055g00770  -  SWAP (Suppressor-of-White-APricot)
## VIT_13s0019g05210  -  RNA recognition motif (RRM)-containing protein
## VIT_05s0124g00060  -  Pentatricopeptide (PPR) repeat-containing
## VIT_19s0090g01350  -  Aspartyl protease
## VIT_11s0016g05600  -  Zinc finger (ubiquitin-hydrolase) domain-containing
## VIT_00s0285g00050  -  No hit
```

```
## VIT_02s0025g01180  -  Carrier protein, Mitochondrial
## VIT_03s0091g00590  -  Unknown protein
## VIT_05s0102g00700  -  Group II intron splicing factor CRS1-like
## VIT_19s0015g00610  -  Unknown protein
## VIT_09s0002g06040  -  Monothiol glutaredoxin-S15 (AtGrxS15)
## VIT_13s0064g00360  -  Notchless-like protein
## VIT_18s0089g00030  -  Protein kinase
## VIT_18s0001g02160  -  Switching protein 3C ATSWI3C
## VIT_13s0067g01320  -  Alpha-1,2-mannosyltransferase
## VIT_01s0011g01480  -  CHR4/MI-2-like (chromatin remodeling 4)
## VIT_05s0020g01520  -  Bud site selection protein 31
## VIT_03s0038g03830  -  DNA-binding bromodomain-containing protein
## VIT_13s0067g03800  -  Cytochrome c oxidase subunit VIb
## VIT_18s0001g13210  -  Lectin jacalin
## VIT_16s0050g02620  -  Abscisic acid receptor PYL8  RCAR3
## VIT_02s0012g00500  -  Invertase/pectin methylesterase inhibitor
## VIT_14s0066g01920  -  Cellular repressor of E1A-stimulated genes (CREG)
## VIT_13s0067g00670  -  Nuclear pore complex protein Nup88
## VIT_18s0001g13250  -  Thioredoxin H-type 1
## VIT_18s0001g13710  -  BPC6/BBR/BPC6/BPC6
## VIT_01s0011g02260  -  Metal-nicotianamine transporter YSL7
## VIT_08s0007g06530  -  DnaJ homolog, subfamily B, member 6
## VIT_00s2262g00010  -  Low expression of osmotically responsive genes 1
## VIT_09s0002g07290  -  Signal recognition particle subunit 54 SRP54
## VIT_05s0094g01070  -  Leucine zipper-EF-hand containing transmembrane protein 1
## VIT_05s0051g00730  -  Zinc finger (C3HC4-type ring finger)
## VIT_08s0007g05390  -  ARR3 typeA
## VIT_04s0023g00850  -  Ribosomal protein L25
## VIT_02s0033g01330  -  Acyl-CoA binding protein
## VIT_17s0000g01740  -  Aspartyl aminopeptidase
## VIT_00s1466g00020  -  Unknown protein
## VIT_05s0062g00980  -  Aldo/keto reductase AKR
## VIT_09s0018g01710  -  RNA recognition motif (RRM)-containing protein
## VIT_14s0066g00260  -  SURF2 (surfeit 2)
## VIT_00s0179g00210  -  WD-40 repeat / beige
## VIT_14s0006g02280  -  Cyclin-dependent kinase D1;2
## VIT_04s0023g02110  -  Unknown protein
## VIT_15s0021g02720  -  Zinc finger (C2H2 type) family
## VIT_10s0003g00350  -  NAC domain-containing protein (VvNAC37)
## VIT_12s0178g00150  -  Unknown protein
## VIT_18s0001g08680  -  Pre-mRNA-processing factor 40
## VIT_12s0059g00660  -  Autophagy 8d (APG8d)
## VIT_16s0013g02130  -  Protein kinase
## VIT_03s0017g01790  -  Ribosomal protein L20
## VIT_09s0002g05250  -  Aminotransferase class I and II
## VIT_16s0039g01660  -  Unknown protein
## VIT_19s0014g02370  -  3-hydroxybutyryl-CoA dehydrogenase
## VIT_09s0018g01820  -  MKK5
```

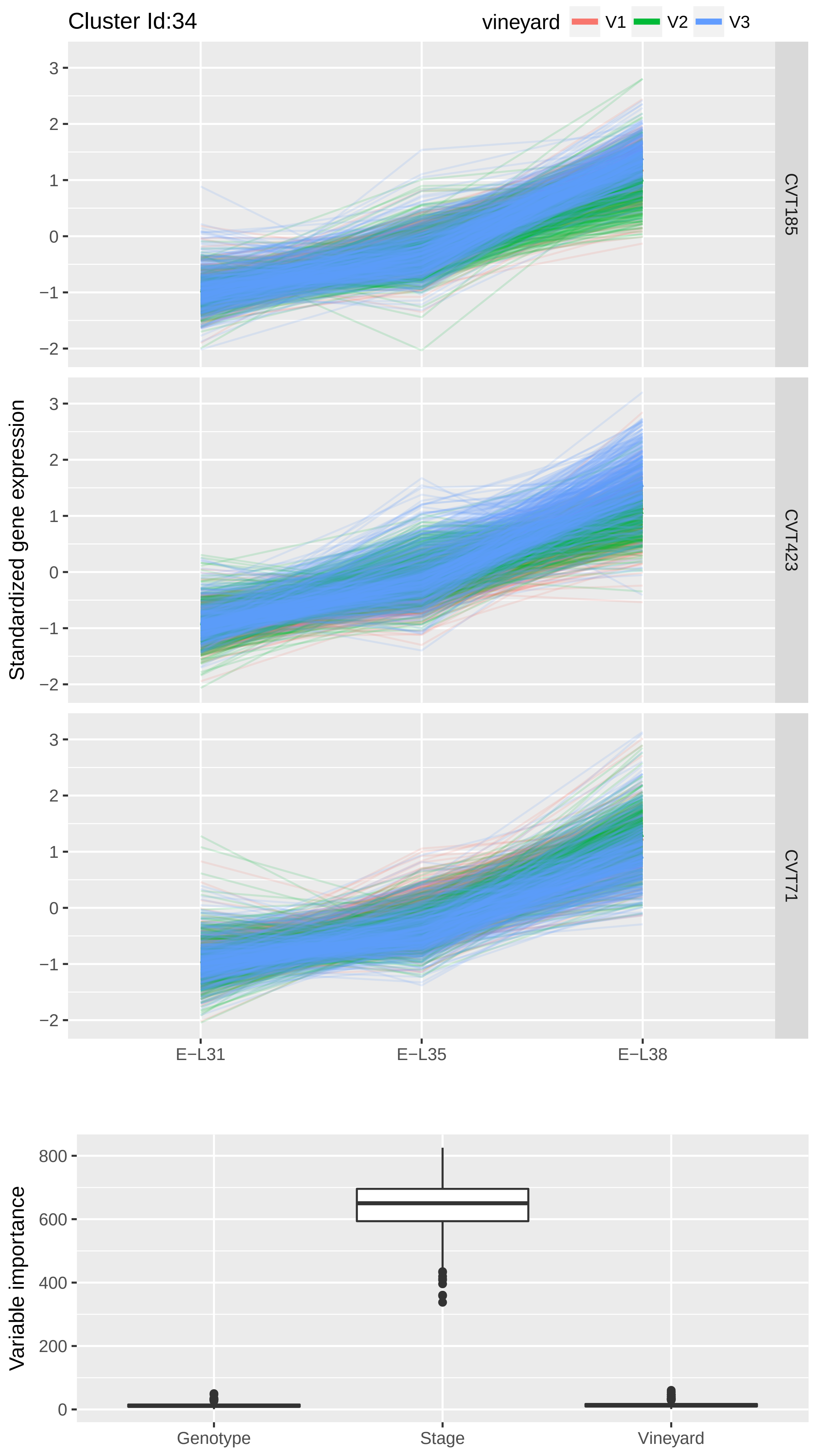

Cluster Id:34
vineyard V1 V2 V3
Standardized gene expression
CVT185
CVT423
CVT71
3
2
1
0
−1
−2
E−L31
E−L35
E−L38
Variable importance
800
600
400
200
0
Genotype
Stage
Vineyard

Cluster no. 35

```
## Number of genes in the cluster: 60
## Homogeneity Index:    0.53
## Variable importance for Stage:    Median =  122.5  - Rank =  103
## Variable importance for Clone:    Median =  33.08  - Rank =  6
## Variable importance for Vineyard: Median =  62.35  - Rank =  30
##
## Gene ID              Gene Annotation
## VIT_10s0405g00050  -  Inositol transporter 4
## VIT_09s0002g02440  -  ABC Transporter (VvMRP9 - VvABCC9)
## VIT_01s0011g03780  -  Zinc finger protein 6
## VIT_17s0000g07620  -  Pentatricopeptide (PPR) repeat-containing protein
## VIT_14s0030g01000  -  No hit
## VIT_00s0179g00220  -  Pentatricopeptide repeat
## VIT_12s0035g00680  -  ACR4 (Arabidopsis CRINKLY4)
## VIT_02s0025g04570  -  Unknown protein
## VIT_07s0130g00180  -  Pentatricopeptide (PPR) repeat-containing
## VIT_08s0007g00230  -  No hit
## VIT_04s0008g02450  -  Unknown protein
## VIT_16s0039g02630  -  Unknown protein
## VIT_17s0000g01620  -  Uridine/cytidine kinase 1
## VIT_01s0026g02130  -  Pentatricopeptide (PPR) repeat-containing protein
## VIT_06s0004g06240  -  Zinc finger (C2H2 type) family
## VIT_18s0001g03130  -  Unknown protein
## VIT_12s0028g02610  -  Tetratricopeptide helical
## VIT_11s0016g03500  -  Unknown protein
## VIT_15s0021g02690  -  Unfertilized embryo sac 10 UNE10
## VIT_18s0001g14880  -  Unknown protein
## VIT_19s0090g01690  -  WNK3 (Arabidopsis WNK kinase 3)
## VIT_01s0010g02230  -  Response regulator ARR11 Type-B
## VIT_15s0048g02220  -  Unknown protein
## VIT_11s0016g02410  -  MYB divaricata
## VIT_12s0057g01540  -  Regulator of chromosome condensation (RCC1)
## VIT_10s0003g05590  -  Toprim domain-containing protein
## VIT_09s0002g01580  -  Mitogen-activated protein kinase organizer 1
## VIT_00s0323g00080  -  Elongation factor G, chloroplast precursor
## VIT_10s0092g00520  -  Leucine-rich repeat family
## VIT_05s0049g01970  -  IAA13
## VIT_18s0001g08830  -  FK506-binding protein genes family (VvFKBP65)
## VIT_18s0001g07250  -  Pentatricopeptide (PPR) repeat-containing protein
## VIT_04s0008g01500  -  Heat shock protein 17.6 kDa class II
## VIT_00s0181g00040  -  TIR1 (transport inhibitor response 1)
## VIT_02s0025g00280  -  Heat shock protein 90-1
## VIT_07s0130g00390  -  DNA (cytosine-5)-methyltransferase
## VIT_16s0050g01430  -  Ornithine cyclodeaminase
## VIT_00s0181g00030  -  No hit
## VIT_00s0742g00020  -  Ribosomal protein L23
## VIT_12s0028g01980  -  Ribosomal protein L1
## VIT_01s0010g03290  -  Beta 1,3-glycosyltransferase I
## VIT_04s0008g01580  -  Heat shock protein 17.6 kDa class II
## VIT_04s0008g01510  -  Heat shock protein 17.6 kDa class II
## VIT_19s0015g00260  -  Import inner membrane translocase subunit TIM50
## VIT_19s0027g00210  -  DNA repair protein recA, chloroplast precursor
## VIT_06s0004g06320  -  Anthranilate synthase component I-1, chloroplast precursor
## VIT_02s0154g00480  -  Heat shock protein MTSHP
```

```
## VIT_01s0011g00920  -  Pentatricopeptide repeat
## VIT_07s0104g01760  -  Unknown
## VIT_19s0015g00460  -  TRNA-splicing endonuclease positive effector
## VIT_04s0008g01550  -  Small molecular heat shock protein 17.5
## VIT_04s0008g01570  -  Heat shock protein 17.6 kDa class II
## VIT_02s0154g00490  -  Heat shock 22 kDa protein mitochondrial
## VIT_01s0011g00240  -  Fructokinase-2
## VIT_16s0022g02140  -  CYP704A2
## VIT_14s0060g02340  -  Heat shock protein 70
## VIT_14s0081g00680  -  Unknown protein
## VIT_11s0016g02150  -  Protein kinase CDG1
## VIT_19s0014g03480  -  Unknown protein
## VIT_18s0001g09710  -  MRG
```

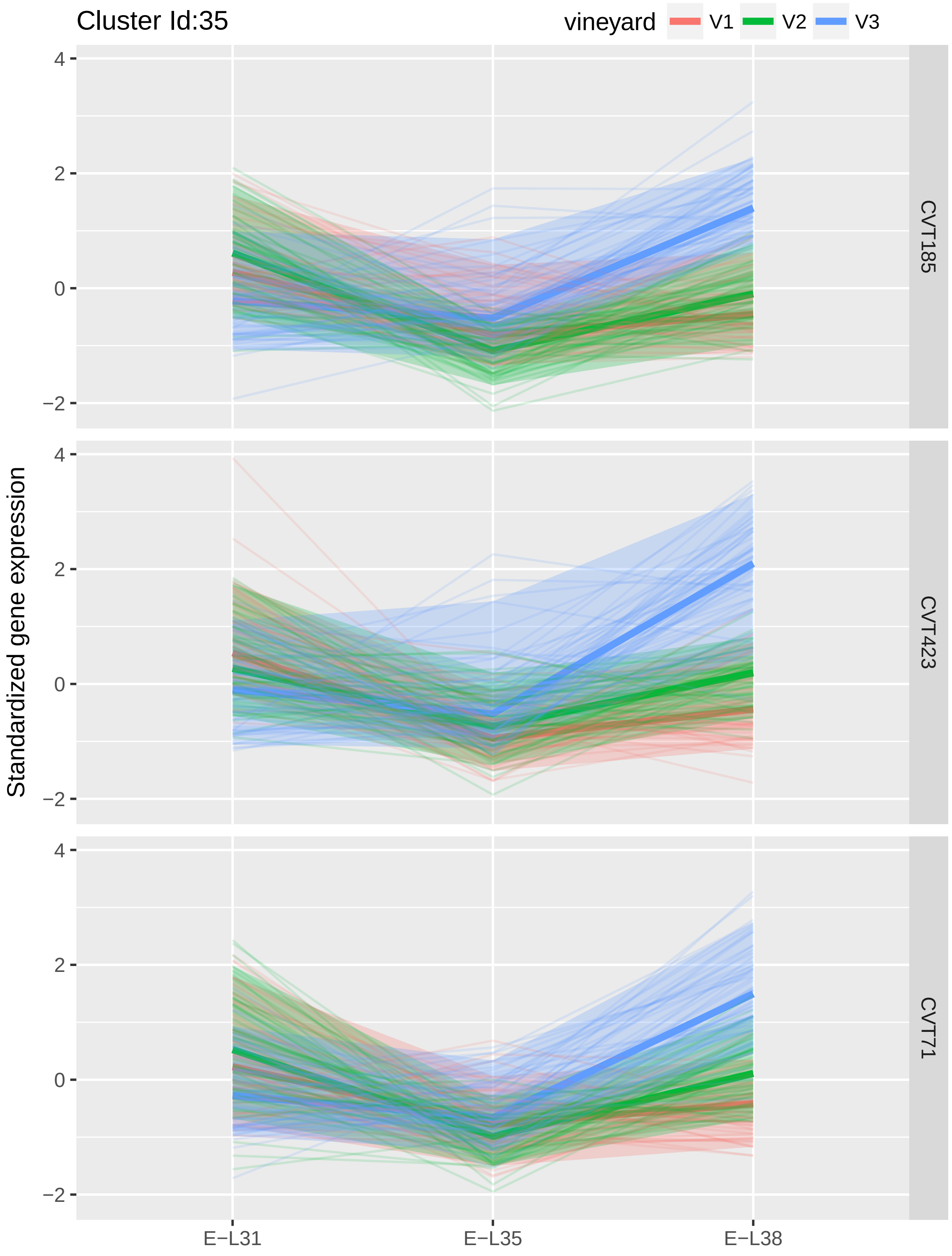
Cluster Id:35
vineyard
V1
V2
V3
Standardized gene expression
CVT185
CVT423
CVT71
4
2
0
−2
E−L31
E−L35
E−L38

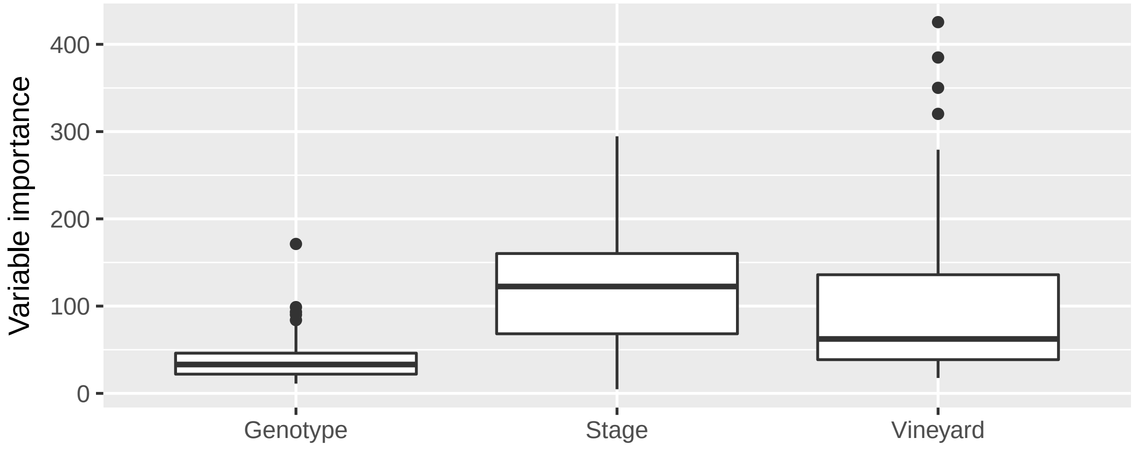
Variable importance
400
300
200
100
0
Genotype
Stage
Vineyard

Cluster no. 36

```
## Number of genes in the cluster: 37
## Homogeneity Index:    0.58
## Variable importance for Stage:    Median =    121  - Rank =  104
## Variable importance for Clone:    Median =  20.81  - Rank =  42
## Variable importance for Vineyard: Median =    120  - Rank =  14
##
## Gene ID               Gene Annotation
## VIT_19s0014g04150  -  S-receptor protein kinase
## VIT_11s0016g01060  -  Avr9/Cf-9 induced kinase 1
## VIT_15s0048g02270  -  No apical meristem cup-shaped cotyledon2
## VIT_00s0179g00250  -  No hit
## VIT_17s0000g03370  -  Calmodulin-binding protein
## VIT_06s0004g07490  -  Unknown protein
## VIT_02s0025g01430  -  Zinc finger (C3HC4-type ring finger) family protein ATL1E
## VIT_17s0000g04360  -  Wall-associated kinase 2 (WAK2)
## VIT_18s0001g09850  -  Myb domain protein R1
## VIT_08s0040g02730  -  Exocyst subunit EXO70 H4
## VIT_00s0179g00260  -  Calcium-transporting ATPase 12 ACA12
## VIT_14s0030g01890  -  Unknown
## VIT_13s0047g00230  -  Pectinesterase family
## VIT_01s0011g05440  -  Unknown protein
## VIT_13s0074g00580  -  Unknown protein
## VIT_05s0062g00790  -  NSL1 (necrotic spotted lesions 1)
## VIT_05s0020g04300  -  Ca2+-ATPase 13 ACA13, plasma membrane
## VIT_14s0068g01800  -  putative MADS-box Flowering Locus C 2 (VviFLC2)
## VIT_01s0011g01810  -  No hit
## VIT_07s0031g00350  -  caffeoyl-CoA O-methyltransferase 1 (CCoAOMT1)
## VIT_05s0049g00210  -  Lesion inducing protein
## VIT_07s0104g01130  -  No hit
## VIT_09s0070g00490  -  Protein phosphatase 2C
## VIT_04s0023g01720  -  SEC14 cytosolic factor, putative
## VIT_17s0000g05530  -  DnaJ homolog, subfamily B, member 4
## VIT_00s0590g00010  -  Cytochrome c biogenesis protein CcmE
## VIT_00s2648g00010  -  FAD-dependent pyridine nucleotide-disulphide oxidoreductase
## VIT_02s0154g00370  -  YbaK/prolyl-tRNA synthetase associated region
## VIT_08s0007g08360  -  Zf AN1 and C2H2 domain-containing stress-associated protein 11
## VIT_00s2376g00010  -  NADH dehydrogenase, putative
## VIT_15s0046g01870  -  Proline-rich family protein
## VIT_12s0028g02440  -  Unknown protein
## VIT_01s0127g00500  -  OBP32pep
## VIT_08s0105g00400  -  GT-1-like transcription factor
## VIT_12s0059g01080  -  Acid phosphatase/vanadium-dependent haloperoxidase
## VIT_18s0001g04600  -  Glutaredoxin
## VIT_08s0007g04860  -  Nodulin family protein
```

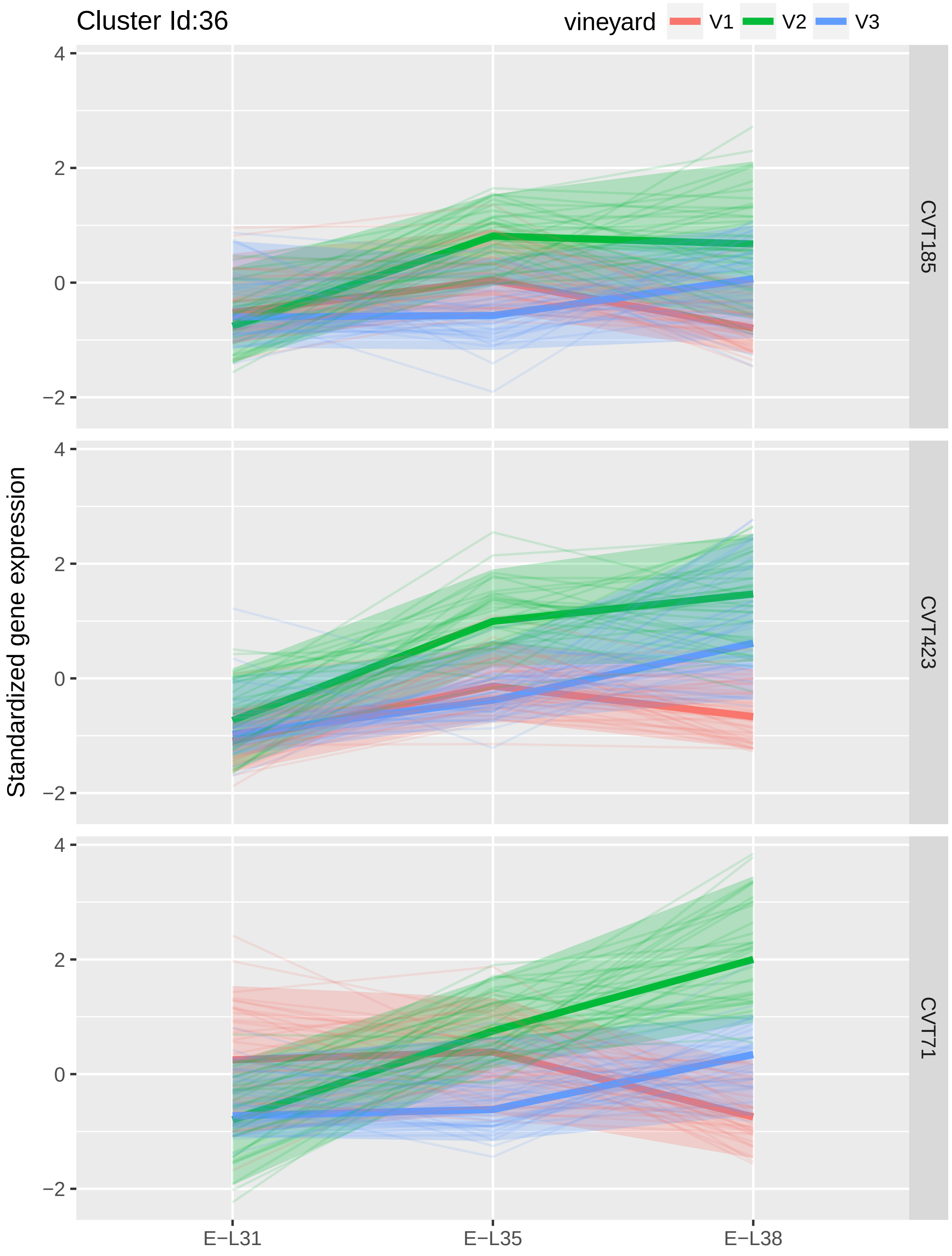

Cluster Id:36
vineyard
V1
V2
V3
Standardized gene expression
CVT185
CVT423
CVT71
4
2
0
−2
E−L31
E−L35
E−L38

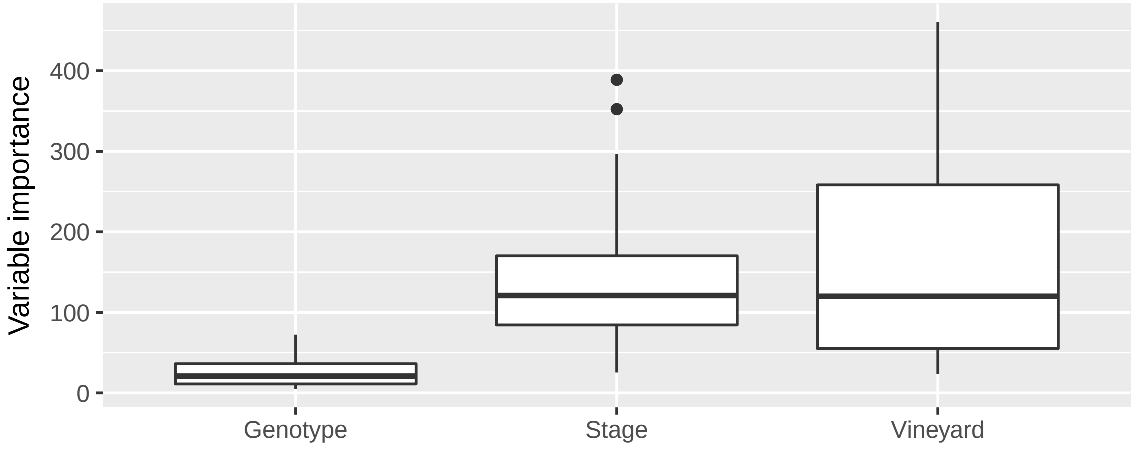

Variable importance
400
300
200
100
0
Genotype
Stage
Vineyard

Cluster no. 37

```
## Number of genes in the cluster: 159
## Homogeneity Index:    0.71
## Variable importance for Stage:    Median =  515.3  - Rank =  31
## Variable importance for Clone:    Median =   15.8  - Rank =  70
## Variable importance for Vineyard: Median =  24.62  - Rank =  77
##
## Gene ID               Gene Annotation
## VIT_08s0007g04910  -  Dual specificity phosphatase Cdc25
## VIT_03s0038g03800  -  Protein kinase APK1B
## VIT_07s0129g00360  -  Peroxidase 73
## VIT_03s0038g00500  -  ferulate 5-hydroxylase
## VIT_18s0001g13500  -  forkhead-associated domain-containing protein
## VIT_19s0085g00630  -  Defective in cullin neddylation DCN1 protein 2
## VIT_18s0075g00120  -  Alpha-1,4-fucosyltransferase
## VIT_19s0015g02410  -  Receptor protein kinase
## VIT_00s0187g00130  -  Tonoplast dicarboxylate transporter
## VIT_03s0038g04160  -  DC1 domain-containing protein
## VIT_15s0021g00890  -  Ring-H2 zinc finger protein ATL4
## VIT_08s0007g08170  -  Unknown protein
## VIT_13s0156g00120  -  EF hand
## VIT_12s0059g01560  -  Protein disulfide-isomerase A6
## VIT_09s0070g00140  -  CBL-interacting protein kinase 6 (CIPK6)
## VIT_03s0038g04550  -  Unknown
## VIT_19s0085g00820  -  IMP dehydrogenase
## VIT_02s0012g02520  -  Vacuolar protein sorting 25
## VIT_00s0214g00060  -  F-box protein PP2-B2
## VIT_02s0025g05070  -  Microtubule motor PAK (phosphatidic acid kinase) KHC
## VIT_06s0004g02400  -  Unknown protein
## VIT_03s0063g01770  -  No hit
## VIT_12s0035g00180  -  Leucine-rich repeat protein kinase
## VIT_04s0008g01950  -  YGGT
## VIT_07s0005g00340  -  Phytochrome A-associated F-box protein
## VIT_17s0000g03670  -  Unknown protein
## VIT_13s0047g00210  -  flavonol synthase
## VIT_19s0014g03320  -  Peroxisomal membrane protein
## VIT_13s0074g00400  -  PTL (PETAL LOSS)
## VIT_12s0059g01460  -  ERF/AP2 Gene Family (VvERF066)
## VIT_14s0068g00130  -  feronia receptor-like kinase
## VIT_02s0025g05060  -  Microtubule motor PAK (phosphatidic acid kinase) KHC
## VIT_14s0068g00720  -  Tudor domain protein 4SNc
## VIT_18s0086g00690  -  Exostosin
## VIT_01s0010g01390  -  Phototropic-responsive NPH3
## VIT_15s0046g02410  -  Aquaporin TMP-C
## VIT_00s2188g00010  -  Tonoplast dicarboxylate transporter
## VIT_04s0008g06300  -  Leucine-rich repeat family
## VIT_07s0031g00450  -  Basic helix-loop-helix protein SPATULA
## VIT_05s0020g02340  -  Methionine sulfoxide reductase
## VIT_18s0001g00600  -  F-box family protein
## VIT_00s0338g00030  -  Cellular retinaldehyde-binding/triple function, C-terminal
## VIT_14s0219g00170  -  Zinc finger (CCCH-type) family protein
## VIT_10s0003g01600  -  WRKY Transcription Factor (VvWRKY30)
## VIT_00s0353g00060  -  ARK3 (Arabidopsis Receptor Kinase 3)
## VIT_06s0004g02520  -  Telomere repeat binding factor like TRFL6
## VIT_02s0154g00040  -  Thylakoid lumenal protein
```

```
## VIT_17s0000g03450  -  No hit
## VIT_16s0013g00070  -  Unknown
## VIT_04s0023g01360  -  Basic Leucine Zipper Transcription Factor (VvbZIP12)
## VIT_06s0009g02790  -  Dihydropyrimidinase
## VIT_14s0066g01900  -  ferredoxin thioredoxin reductase catalytic beta chain
## VIT_17s0000g02700  -  Alpha-dioxygenase 2
## VIT_03s0063g02490  -  Glucan endo-1,3-beta-glucosidase 7 precursor
## VIT_07s0005g00450  -  Unknown protein
## VIT_08s0058g00280  -  Lectin protein kinase
## VIT_14s0006g03040  -  DNA repair protein RAD23
## VIT_00s0245g00050  -  Oleoyl-acyl carrier protein thioesterase, chloroplast precursor
## VIT_03s0063g01880  -  Acyl-CoA synthetase long-chain member 2
## VIT_15s0046g02010  -  Mitogen-activated Protein Kinase (VvMPK9)
## VIT_01s0011g04290  -  Receptor serine/threonine kinase PR5K
## VIT_08s0007g05720  -  No hit
## VIT_17s0000g02290  -  GTPase activating protein
## VIT_09s0018g01220  -  Zinc finger (C3HC4-type ring finger)
## VIT_00s0187g00120  -  Cystatin
## VIT_14s0066g01570  -  Unknown protein
## VIT_05s0029g00690  -  RPM1-interacting protein 4
## VIT_06s0004g08170  -  CENP-E like kinetochore protein
## VIT_05s0049g00410  -  1-aminocyclopropane-1-carboxylate oxidase
## VIT_06s0061g00450  -  4-coumarate-CoA ligase
## VIT_02s0236g00040  -  Peptidyl-prolyl cis-trans isomerase, cyclophilin type
## VIT_16s0098g01700  -  Epoxide hydrolase
## VIT_05s0077g01060  -  Aldehyde Dehydrogenase (VvALDH22A1)
## VIT_12s0059g02290  -  Unknown protein
## VIT_18s0072g00770  -  fructose-1,6-bisphosphatase, cytosolic
## VIT_19s0015g00550  -  Nudix hydrolase 10
## VIT_01s0146g00450  -  Transport inhibitor response 1 protein
## VIT_11s0065g00040  -  CYP706A12
## VIT_06s0004g06980  -  Phytol kinase 2, chloroplast precursor
## VIT_07s0005g00730  -  Pectinesterase family
## VIT_11s0016g00470  -  Sucrose synthase
## VIT_19s0015g02500  -  CYP72A1
## VIT_14s0006g02080  -  S locus-linked protein SLL2
## VIT_08s0007g04790  -  Zinc finger (C3HC4-type ring finger)
## VIT_19s0014g03610  -  Unknown
## VIT_09s0070g00520  -  Ubiquitin-specific protease 3 (UBP3)
## VIT_12s0059g00300  -  TAFII59 (TATA box associated factor II 59)
## VIT_17s0000g09640  -  RGLG2 (ring Domain LIGASE2)
## VIT_15s0021g01350  -  Transducin family protein / WD-40 repeat
## VIT_06s0004g07370  -  Unknown protein
## VIT_04s0008g06850  -  Biopterin transport-related protein BT1
## VIT_13s0064g00050  -  Glutamine-fructose-6-phosphate transaminase 2
## VIT_09s0002g06760  -  Phospholipase D alpha 1 precursor (PLD 1) (Choline phosphatase 1)
## VIT_05s0020g03620  -  Unknown protein
## VIT_12s0035g01040  -  NSL1 (necrotic spotted lesions 1)
## VIT_02s0087g00740  -  Unknown protein
## VIT_02s0012g02780  -  Cytochrome b5 domain-containing protein
## VIT_07s0005g05850  -  Unknown protein
## VIT_14s0219g00070  -  Zinc finger (C2H2 type) family
## VIT_08s0007g03670  -  Lipase GDSL
## VIT_03s0063g02350  -  Organ-specific protein S2
## VIT_07s0005g01390  -  Calreticulin 2 (CRT2)
```

```
## VIT_14s0036g00370  -  Serine/threonine-protein phosphatase PP1
## VIT_12s0034g00210  -  Mekk1
## VIT_00s0227g00170  -  Syntaxin 52
## VIT_00s0227g00070  -  Sialidase BNR/Asp-box repeat family protein
## VIT_01s0182g00020  -  Ankyrin repeat family protein
## VIT_18s0041g00190  -  R protein L6
## VIT_06s0004g04160  -  Trp repressor/replication initiator
## VIT_01s0011g04300  -  Receptor serine/threonine kinase PR5K
## VIT_13s0047g00350  -  Unknown
## VIT_17s0000g07520  -  Ribosomal-protein S6 kinase p70
## VIT_02s0012g02160  -  Glycerol-3-phosphate acyltransferase
## VIT_17s0000g01190  -  Phosphatidylglycerol specific phospholipase C
## VIT_00s0218g00110  -  1-deoxy-D-xylulose-5-phosphate synthase, chloroplast precursor
## VIT_13s0019g04240  -  Protein disulfide-isomerase A1
## VIT_04s0008g06830  -  Biopterin transport-related protein BT1
## VIT_12s0059g00890  -  Dienelactone hydrolase
## VIT_14s0066g01580  -  K+ uptake permease 6
## VIT_07s0031g01660  -  CYP86A2
## VIT_07s0151g00960  -  WD repeat domain 5
## VIT_08s0007g00120  -  Ubiquitin-conjugating enzyme 13 (UBC13), E2
## VIT_02s0012g02530  -  Phosphomevalonate kinase
## VIT_07s0130g00150  -  Thioesterase family
## VIT_02s0025g03470  -  Tyrosyl-tRNA synthetase
## VIT_17s0000g09230  -  Leucine-rich repeat family protein
## VIT_13s0101g00520  -  Unknown protein
## VIT_00s0179g00300  -  MPBQ/MSBQ methyltransferase 2
## VIT_01s0150g00270  -  Unknown protein
## VIT_10s0003g03090  -  3-beta hydroxysteroid dehydrogenase
## VIT_03s0038g04710  -  Isoflavone reductase (synthesis of phytoalexines)
## VIT_18s0089g01340  -  Class I peptide chain release factor; Helix-turn-helix, Fis-type
## VIT_00s0194g00060  -  No hit
## VIT_11s0052g00620  -  AHA2 (Arabidopsis H(+)-ATPase 2)
## VIT_04s0008g06890  -  Solute carrier family 35
## VIT_14s0060g00380  -  PHD finger transcription factor
## VIT_02s0012g00570  -  Pseudo-response regulator 2 (APRR2) (TOC2)
## VIT_02s0154g00530  -  Histidine triad nucleotide binding protein 3
## VIT_11s0016g02680  -  Asparaginyl-tRNA synthetase, cytoplasmic 3
## VIT_12s0059g02750  -  Eukaryotic translation initiation factor-related
## VIT_07s0130g00350  -  Serine C-palmitoyltransferase (LCB2)
## VIT_02s0025g02500  -  Metal-nicotianamine transporter YSL1
## VIT_08s0040g03400  -  Short-chain dehydrogenase/reductase
## VIT_06s0080g01080  -  Ubiquitin-conjugating enzyme E2 variant
## VIT_03s0038g00650  -  Coenzyme Q10 homolog B
## VIT_17s0000g00290  -  Alpha-mannosidase precursor lysosomal
## VIT_01s0146g00020  -  Unknown protein
## VIT_13s0067g03890  -  Beta-ketoacyl-CoA synthase
## VIT_17s0000g05700  -  Phosphoribosylanthranilate transferase
## VIT_03s0038g01510  -  Unknown protein
## VIT_17s0000g09840  -  Adenosylhomocysteinase 1
## VIT_13s0047g00540  -  Cinnamyl alcohol dehydrogenase
## VIT_17s0000g02970  -  MATE efflux family protein
## VIT_09s0002g03140  -  Lipase GDSL
## VIT_03s0038g00800  -  NADP-dependent D-sorbitol-6-phosphate dehydrogenase
## VIT_14s0066g01320  -  formate dehydrogenase (FDH)
```

```
## VIT_03s0091g00850  -  Unknown protein
## VIT_08s0007g03200  -  RNA-binding region RNP-1
## VIT_14s0068g00100  -  feronia receptor-like kinase
```

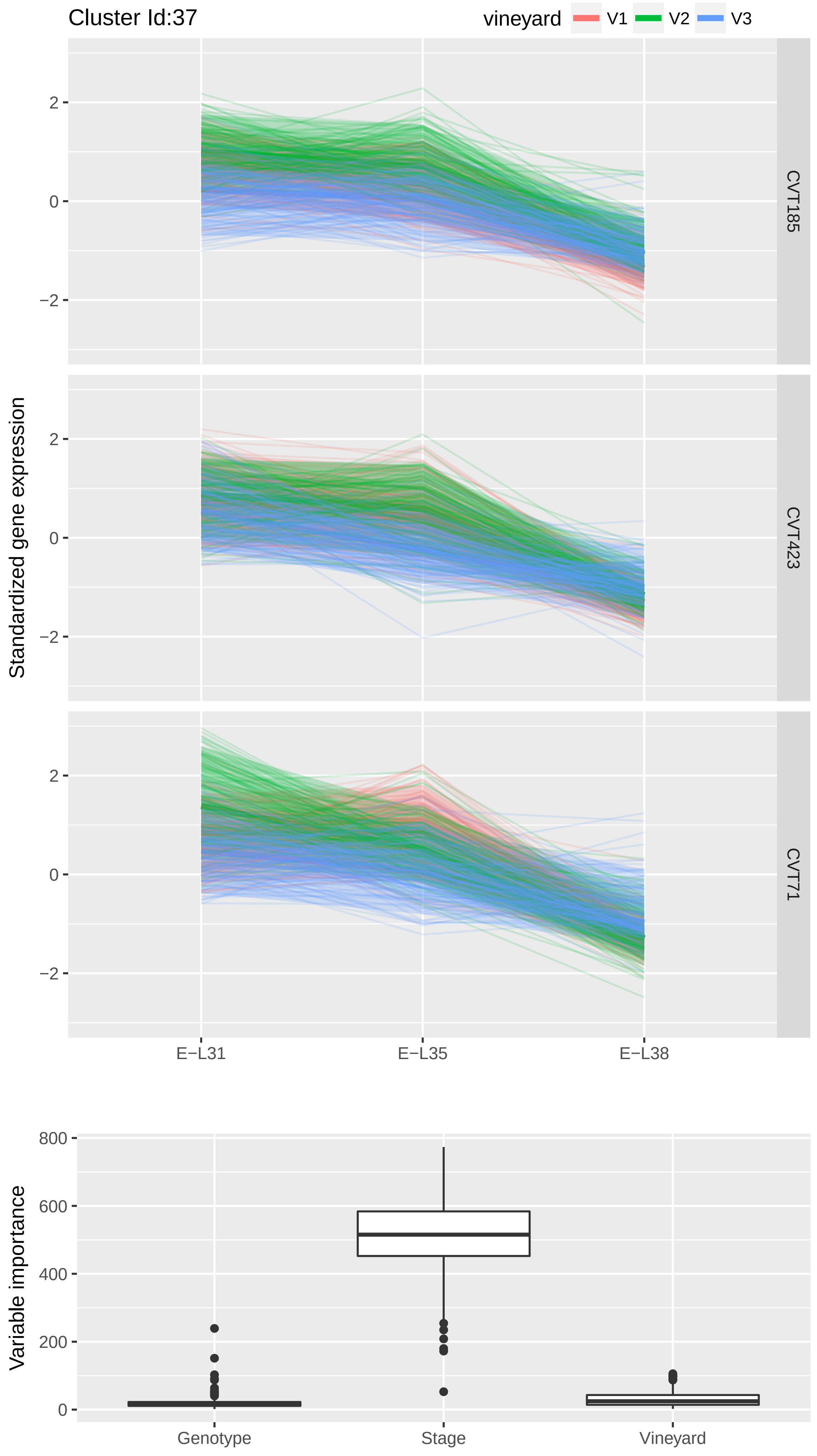

Cluster Id:37
vineyard
V1
V2
V3
Standardized gene expression
CVT185
CVT423
CVT71
2
0
−2
E−L31
E−L35
E−L38
Variable importance
800
600
400
200
0
Genotype
Stage
Vineyard

Cluster no. 38

```
## Number of genes in the cluster: 78
## Homogeneity Index:    0.66
## Variable importance for Stage:    Median =  422.8  - Rank =  53
## Variable importance for Clone:    Median =  18.35  - Rank =  62
## Variable importance for Vineyard: Median =  18.09  - Rank =  88
##
## Gene ID               Gene Annotation
## VIT_18s0001g09220  -  Adenine phosphoribosyltransferase
## VIT_05s0077g01650  -  s5_Pathogenesis protein 10 [Vitis vinifera]
## VIT_18s0001g07500  -  Mechanosensitive ion channel MSCS-like 2
## VIT_05s0077g01090  -  RKF2 (receptor-like serine/threonine kinase 2)
## VIT_01s0146g00400  -  Humj1
## VIT_06s0009g02100  -  Nucleolar complex protein 2 homolog (Protein NOC2 homolog)
## VIT_18s0072g00330  -  Nucleic acid binding
## VIT_14s0108g00630  -  Amino acid permease
## VIT_05s0049g00460  -  Basic helix-loop-helix ILR3
## VIT_07s0104g00650  -  No hit
## VIT_03s0038g04060  -  Expansin  (VvEXPA3)
## VIT_11s0016g02310  -  Thioredoxin 2
## VIT_04s0023g01120  -  Galacturonosyltransferase
## VIT_04s0044g00050  -  Protein kinase APK1B
## VIT_05s0020g02200  -  Inosine-uridine preferring nucleoside hydrolase
## VIT_07s0005g05780  -  Pantoate--beta-alanine ligase
## VIT_02s0012g00400  -  1-aminocyclopropane-1-carboxylate oxidase
## VIT_00s2539g00010  -  Protein kinase APK1B
## VIT_07s0031g01980  -  ERF/AP2 Gene Family (VvERF113)
## VIT_07s0104g00850  -  Pto kinase interactor
## VIT_19s0014g02850  -  Dynein light subunit lc6, flagellar outer arm
## VIT_17s0000g04880  -  Dirigent protein
## VIT_08s0058g01100  -  Armadillo/beta-catenin repeat / U-box domain-containing protein
## VIT_18s0001g12520  -  ATP binding
## VIT_18s0001g05710  -  Hydrolase, alpha/beta fold
## VIT_05s0094g00360  -  Chitinase class IV
## VIT_07s0005g04620  -  Unknown
## VIT_18s0001g06310  -  SnRK2-8
## VIT_08s0007g01930  -  Anthranilate N-benzoyltransferase
## VIT_00s0372g00040  -  Linalool synthase (VvTPS57), (3S)- Linalool syn
## VIT_10s0003g02910  -  FLS2 (flagellin-sensitive 2)
## VIT_07s0005g01450  -  Basic Leucine Zipper Transcription Factor (VvbZIP22)
## VIT_08s0040g01070  -  Serine carboxypeptidase S10
## VIT_11s0016g00520  -  Auxin-responsive SAUR38
## VIT_11s0118g00520  -  Unknown
## VIT_19s0014g03790  -  Diacylglycerol kinase
## VIT_10s0003g00550  -  RAB GTPase RABA1F
## VIT_18s0072g00260  -  Ethylene-responsive transcription factor related to APETALA2 6
## VIT_14s0068g01190  -  Aspartate kinase
## VIT_05s0077g01150  -  Beta-1,3-glucanase
## VIT_11s0149g00280  -  Chitinase A
## VIT_06s0080g01260  -  Receptor-like kinase
## VIT_03s0038g00700  -  Kinesin motor protein
## VIT_01s0011g04480  -  Histone chaperone ASF1B (Anti-silencing 1b)
## VIT_08s0040g01130  -  Serine carboxypeptidase II
## VIT_06s0004g04170  -  Methyl-CpG-binding domain-containing protein
## VIT_16s0039g01420  -  Unknown protein
```

```
## VIT_13s0019g05150  -  Unknown protein
## VIT_01s0150g00440  -  Respiratory burst oxidase protein D (RBOHD)
## VIT_13s0019g04550  -  Protein kinase
## VIT_10s0003g04090  -  Remorin
## VIT_08s0040g01040  -  Serine carboxypeptidase II
## VIT_19s0027g00390  -  Protein kinase
## VIT_00s0238g00040  -  R protein disease resistance protein
## VIT_07s0104g00950  -  Ankyrin repeat protein E4_8
## VIT_18s0001g01980  -  Auxilin
## VIT_08s0007g07560  -  Glucan endo-1,3-beta-glucosidase 5 precursor
## VIT_01s0146g00480  -  TIFY gene family (VvJAZ2)
## VIT_02s0012g01630  -  Transmembrane protein 41B
## VIT_04s0023g02420  -  Mitogen-activated Protein Kinase (VvMPK3)
## VIT_00s0218g00220  -  UDP-glucoronosyl/UDP-glucosyl transferase
## VIT_07s0141g01060  -  basic helix-loop-helix (bHLH) family
## VIT_13s0019g03550  -  ERF/AP2 Gene Family (VvAP2-11)
## VIT_03s0038g03400  -  Endochitinase 1, basic
## VIT_03s0063g02330  -  No hit
## VIT_00s0271g00060  -  Linalool synthase (VvTPS56), (E)- Nerolidol syn
## VIT_18s0001g06090  -  Cis-zeatin O-beta-D-glucosyltransferase
## VIT_01s0010g02890  -  Armadillo/beta-catenin repeat protein / U-box domain-containing
## VIT_18s0001g06460  -  Queuine tRNA-ribosyltransferase
## VIT_14s0108g00540  -  Phosphofructokinase
## VIT_19s0014g04930  -  Germacrene-D synthase (VvTPS28)
## VIT_11s0118g00530  -  Unknown
## VIT_18s0001g14410  -  Unknown protein
## VIT_06s0080g00790  -  MYB divaricata
## VIT_03s0063g01310  -  Oxidoreductase, 2OG-Fe(II) oxygenase family
## VIT_00s0179g00350  -  Pyruvate kinase isozyme A, chloroplast precursor
## VIT_00s0353g00050  -  Serine/threonine kinase BRLK
## VIT_02s0012g00390  -  Norcoclaurine synthase
```

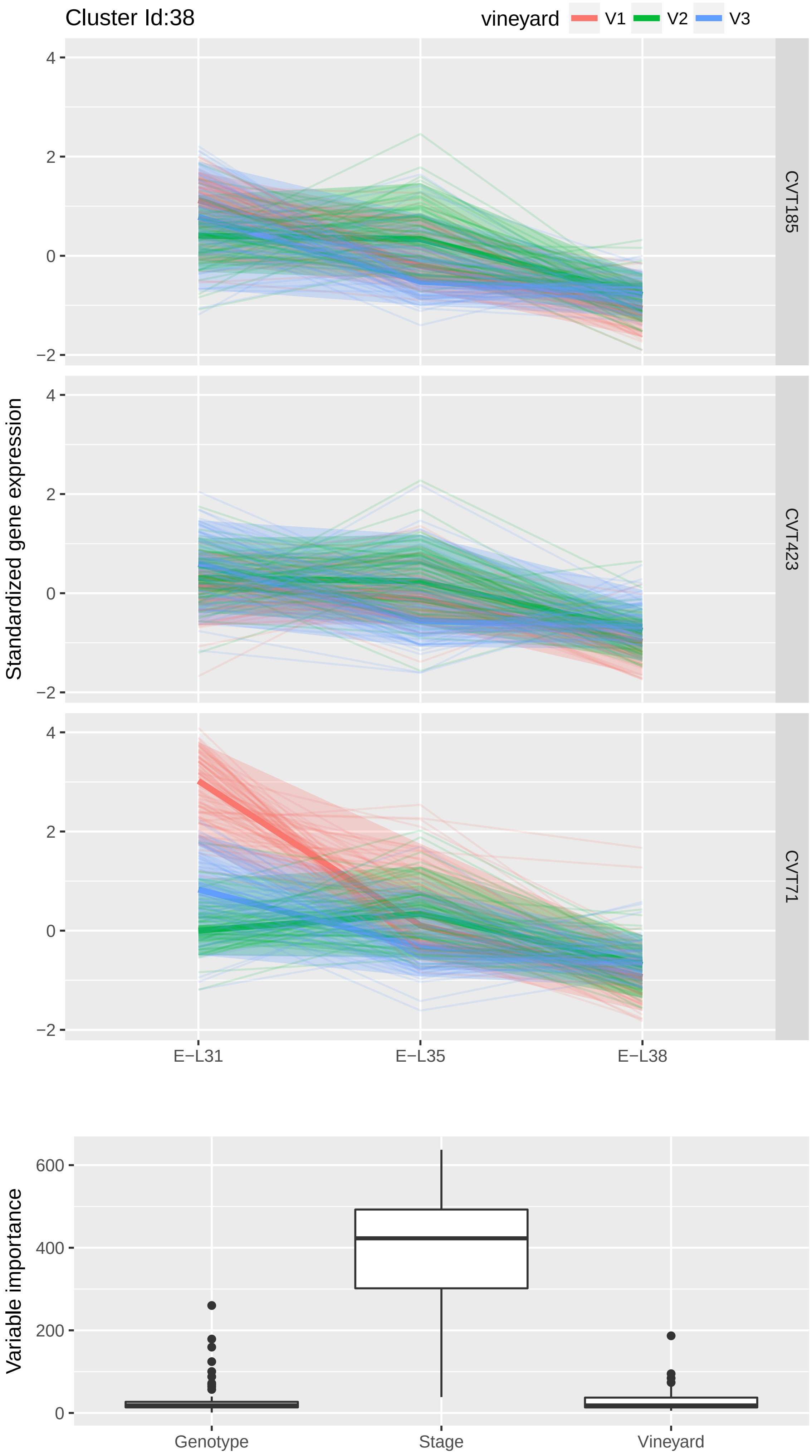

Cluster Id:38
vineyard
V1
V2
V3
Standardized gene expression
CVT185
CVT423
CVT71
E−L31
E−L35
E−L38
4
2
0
−2
Variable importance
600
400
200
0
Genotype
Stage
Vineyard

Cluster no. 39

```
## Number of genes in the cluster: 94
## Homogeneity Index:    0.72
## Variable importance for Stage:    Median =    584  - Rank =  18
## Variable importance for Clone:    Median =  12.32  - Rank =  89
## Variable importance for Vineyard: Median =  15.35  - Rank =  98
##
## Gene ID              Gene Annotation
## VIT_01s0011g03010  -  Serine/threonine-protein kinase SNT7, chloroplast precursor
## VIT_08s0056g00700  -  La domain-containing protein
## VIT_03s0038g02670  -  Unknown protein
## VIT_13s0074g00240  -  Uridylate kinase
## VIT_06s0061g00080  -  Saccharopine dehydrogenase
## VIT_01s0011g02530  -  Carboxylesterase
## VIT_11s0052g00680  -  Unknown protein
## VIT_15s0024g00690  -  Unknown protein
## VIT_16s0050g00630  -  Prolyl 4-hydroxylase
## VIT_17s0000g00130  -  SEC14 cytosolic factor
## VIT_13s0139g00360  -  Unknown protein
## VIT_15s0046g00510  -  Wax synthase
## VIT_17s0000g06830  -  Zinc finger (C2H2 type) family
## VIT_14s0108g01380  -  TRNA/rRNA methyltransferase (SpoU)
## VIT_00s0533g00050  -  Extensin
## VIT_11s0037g00030  -  Unknown protein
## VIT_01s0010g01460  -  Pentatricopeptide (PPR) repeat-containing protein
## VIT_18s0072g00440  -  UDP-glucose:sterol glucosyltransferase
## VIT_07s0005g01520  -  WRKY Transcription Factor (VvWRKY18)
## VIT_12s0035g01750  -  Unknown protein
## VIT_12s0028g01740  -  DNAJ heat shock N-terminal domain-containing protein
## VIT_01s0011g04720  -  Multi copper oxidase type 1
## VIT_08s0007g04800  -  Carbonic anhydrase precursor
## VIT_13s0019g02720  -  Guanylyl cyclase (GC1)
## VIT_12s0028g02370  -  Methyltransferase type 11
## VIT_10s0071g00660  -  MYB divaricata
## VIT_07s0031g00620  -  zeaxanthin epoxidase (ZEP; ABA1) (VvZEP1)
## VIT_07s0031g00680  -  Red chlorophyll catabolite reductase (accelerated cell death 2)
## VIT_12s0059g02110  -  Galacturonosyltransferase 10
## VIT_00s0207g00250  -  No hit
## VIT_05s0077g01080  -  PAPA-1-like family protein
## VIT_01s0011g04740  -  LPR1 (Low Phosphate Root1) multicopper oxidase
## VIT_06s0004g00990  -  Dirigent protein
## VIT_11s0016g05570  -  Thylakoid lumenal 16.5 kDa protein, chloroplast precursor
## VIT_15s0048g01930  -  ATPP2-A13
## VIT_03s0038g01760  -  Disease resistance protein (CC-NBS class)
## VIT_17s0000g07720  -  Sterile alpha motif (SAM) domain-containing protein
## VIT_15s0048g00220  -  Membrane-associated mannitol-induced
## VIT_19s0014g00400  -  Ribosomal protein L13
## VIT_14s0083g00770  -  Phytoene desaturase
## VIT_07s0129g00470  -  Unknown protein
## VIT_16s0039g00240  -  U6 snRNA-associated Sm-like protein LSm8
## VIT_04s0008g05440  -  Ethylene-responsive transcription factor SHINE 3
## VIT_04s0044g00630  -  MAP/ERK kinase kinase 3
## VIT_04s0023g00720  -  Unknown protein
## VIT_00s0199g00160  -  Heat shock protein binding
## VIT_13s0064g01800  -  R protein MLA10
```

```
## VIT_04s0044g00740  -  Unknown protein
## VIT_06s0009g02550  -  Chaperonin
## VIT_00s0227g00140  -  MLO1
## VIT_15s0021g01760  -  Palmitoyl-protein thioesterase 1 precursor
## VIT_09s0054g01220  -  Cycloartenol synthase
## VIT_11s0016g01880  -  lycopene epsilon-cyclase (LECY) (VvLECY1)
## VIT_08s0007g08080  -  G protein beta subunit-like
## VIT_03s0063g01260  -  Nodulin 1A, Senescence-associated
## VIT_08s0007g07630  -  Tryptophan/tyrosine permease family
## VIT_02s0025g02370  -  Yippee
## VIT_08s0007g05980  -  Calmodulin binding protein
## VIT_18s0001g01600  -  Ankyrin
## VIT_17s0000g07640  -  Mannose-1-phosphate guanyltransferase
## VIT_14s0066g00340  -  L-fucokinase
## VIT_00s0179g00360  -  Unknown protein
## VIT_12s0134g00590  -  Anthocyanidin 3-O-glucoside-6''-O-malonyltransferase
## VIT_01s0150g00210  -  CYP51A
## VIT_07s0005g01190  -  Unknown protein
## VIT_14s0171g00130  -  Ribulose-phosphate 3-epimerase
## VIT_14s0060g02050  -  Ribosomal protein L22-2 (RPL22B) 60S
## VIT_18s0001g12550  -  Beta-ketoacyl-CoA synthase
## VIT_10s0003g04560  -  Auxin-Induced in Root cultures 9 AIR9
## VIT_00s0983g00010  -  Abhydrolase domain-containing protein
## VIT_01s0011g06320  -  Cyclin J18
## VIT_10s0003g00490  -  Unknown protein
## VIT_14s0068g02040  -  Gda-1
## VIT_19s0090g00410  -  Calcium Dependent Protein Kinase (VvCPK17)
## VIT_14s0083g00290  -  No hit
## VIT_02s0012g01840  -  Phosphatidylinositol glycan, class X
## VIT_12s0134g00630  -  Quercetin 3-O-glucoside-6''-O-malonyltransferase
## VIT_14s0060g01270  -  Pseudouridine synthase
## VIT_04s0023g00680  -  5-amino-6-(5-phosphoribosylamino)uracil reductase
## VIT_03s0038g02170  -  Thaumatin
## VIT_03s0038g02560  -  Unknown protein
## VIT_02s0012g01180  -  Unknown protein
## VIT_19s0015g00230  -  Zinc transporter ZIP11
## VIT_12s0142g00700  -  MEKK1
## VIT_15s0024g01600  -  No hit
## VIT_13s0067g01890  -  Glycosyl hydrolase family 85
## VIT_02s0025g00390  -  Unknown protein
## VIT_09s0002g04020  -  Aquaporin DELTA-TIP
## VIT_19s0014g02340  -  Protein phosphatase 2C
## VIT_13s0019g05000  -  Cytokinesis Sec1 protein (KEULE)
## VIT_13s0019g04520  -  Early flowering 6
## VIT_09s0070g00060  -  CCR4-NOT transcription complex subunit 7/8
## VIT_15s0046g03670  -  NADP-dependent malic enzyme
## VIT_15s0048g00560  -  UNE1 (unfertilized embryo sac 1)
```

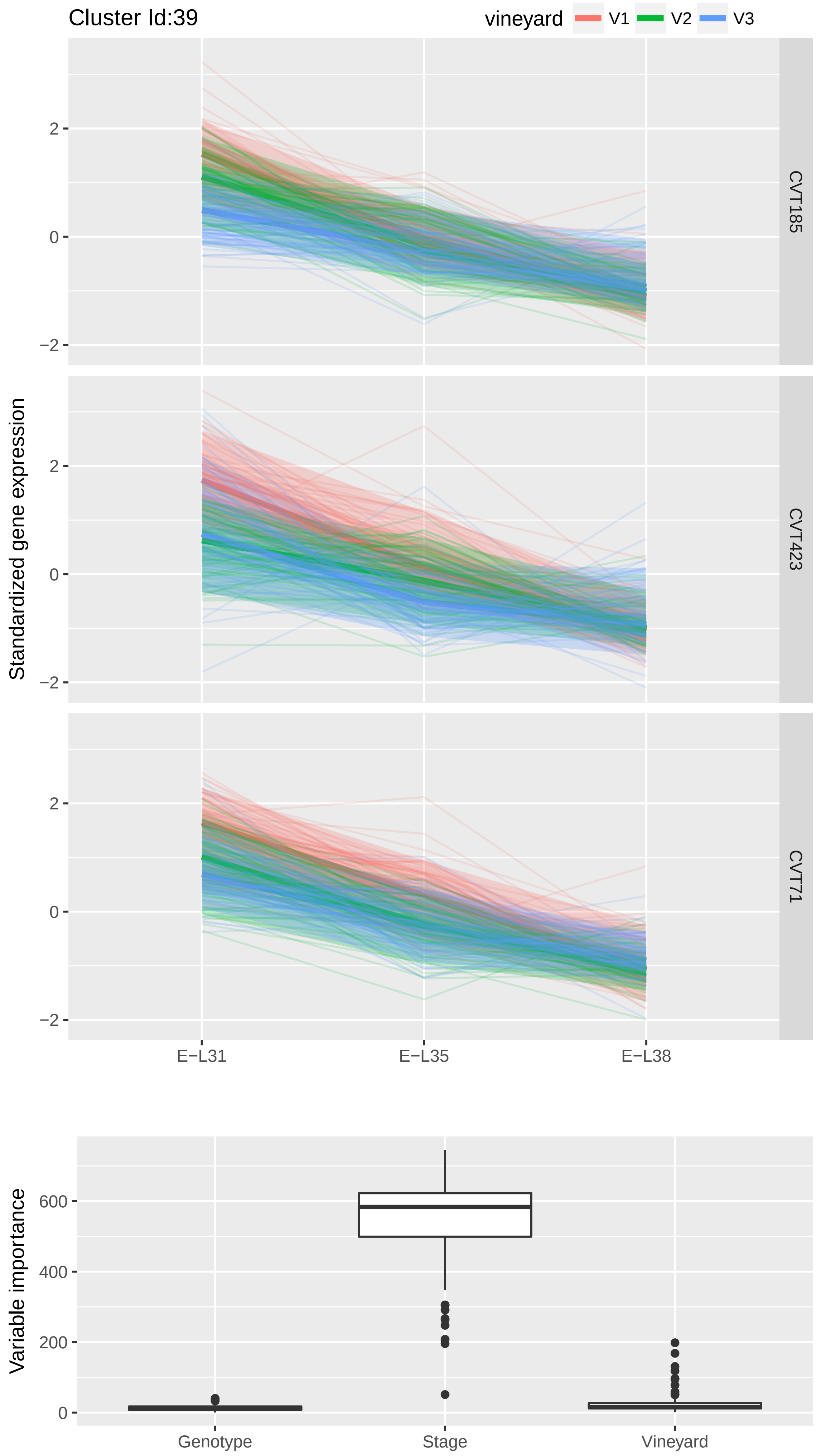

Cluster Id:39
vineyard
V1
V2
V3
CVT185
CVT423
CVT71
Standardized gene expression
2
0
−2
E−L31
E−L35
E−L38
Variable importance
600
400
200
0
Genotype
Stage
Vineyard

Cluster no. 40

```
## Number of genes in the cluster: 130
## Homogeneity Index:     0.6
## Variable importance for Stage:    Median =  209.9  - Rank =  86
## Variable importance for Clone:    Median =  24.37  - Rank =  25
## Variable importance for Vineyard: Median =  57.43  - Rank =  36
##
## Gene ID               Gene Annotation
## VIT_01s0011g03730  -  (myb domain protein 62
## VIT_07s0141g00290  -  IAA16
## VIT_18s0072g01030  -  Calcium-dependent protein kinase (VvCPK16)
## VIT_19s0027g00450  -  Pentatricopeptide (PPR) repeat-containing protein
## VIT_00s0181g00080  -  F-box domain containing protein
## VIT_18s0001g06450  -  No hit
## VIT_05s0020g00920  -  Hydroxyproline-rich glycoprotein
## VIT_05s0020g03480  -  Ribosomal protein L18P/L5E
## VIT_08s0040g00590  -  Zinc finger (C3HC4-type ring finger)
## VIT_02s0025g03330  -  H(+)-ATPase 4 AHA4
## VIT_13s0019g01130  -  DC1 domain-containing protein
## VIT_01s0137g00360  -  Transducin family protein / WD-40 repeat
## VIT_05s0102g00390  -  far-red impaired responsive family protein
## VIT_15s0046g02620  -  Beta-amylase 9 BMY9
## VIT_08s0007g08120  -  Exostosin
## VIT_03s0017g01830  -  FAR1-related sequence 2
## VIT_06s0009g02830  -  Flavonoid 3',5'-hydroxylase
## VIT_02s0025g04980  -  1-aminocyclopropane-1-carboxylate synthase
## VIT_00s0282g00040  -  Major facilitator superfamily protein (MFS) Spinster
## VIT_04s0023g02130  -  1,4-beta-mannan endohydrolase
## VIT_00s0181g00120  -  WD-repeat protein
## VIT_13s0019g03020  -  Glucosyltransferase-2
## VIT_07s0141g00180  -  POR (PORCINO)
## VIT_14s0060g01710  -  Ribosomal protein L18
## VIT_06s0080g00580  -  Rac GTPase activating protein 1
## VIT_05s0077g01470  -  Phytochelatin synthetase
## VIT_02s0087g00360  -  Pentatricopeptide (PPR) repeat-containing protein
## VIT_05s0020g01710  -  Tonneau 2
## VIT_01s0026g01430  -  Unknown protein
## VIT_05s0029g00180  -  ER lumen retaining receptor (HDEL receptor)
## VIT_07s0130g00080  -  3-phosphoinositide-dependent protein kinase-1
## VIT_14s0006g01570  -  DegP protease
## VIT_01s0150g00570  -  NADH dehydrogenase (ubiquinone) 1 alpha subcomplex 2
## VIT_15s0048g01740  -  Growth-regulating factor 9
## VIT_05s0020g00830  -  CBL-interacting protein kinase 1 (CIPK1)
## VIT_14s0108g01280  -  WRKY DNA-binding protein 4  (WRKY-18), WRKY Transcription Factor (VvW
## VIT_06s0004g07390  -  Calcineurin phosphoesterase
## VIT_05s0077g02120  -  Unknown protein
## VIT_19s0090g00300  -  Unknown protein
## VIT_03s0017g00270  -  Carbonic anhydrase, chloroplast precursor
## VIT_12s0059g01870  -  GH3-like protein
## VIT_09s0002g06830  -  Curculin (mannose-binding) lectin
## VIT_13s0019g00020  -  Cation efflux MTP11
## VIT_03s0038g04280  -  Protein BCCIP homolog
## VIT_12s0028g03020  -  Unknown
## VIT_10s0003g03250  -  Unknown protein
## VIT_01s0011g04650  -  Unknown protein
```

```
## VIT_07s0095g00660  -  Curculin (mannose-binding) lectin
## VIT_10s0116g01650  -  5'-adenylylsulfate reductase (APR1)
## VIT_01s0011g01520  -  Peroxisomal 2,4-dienoyl-CoA reductase
## VIT_11s0149g00270  -  Chitinase-3 protein 1 precursor
## VIT_14s0006g01290  -  myb domain protein 113 (VvMYBA6)
## VIT_04s0023g02430  -  Basic Leucine Zipper Transcription Factor (VvbZIP13)
## VIT_12s0028g02120  -  No hit
## VIT_00s0259g00110  -  ATFP3 (Arabidopsis thaliana farnesylated protein 3
## VIT_16s0050g02390  -  Plastid division protein PDV1
## VIT_01s0150g00060  -  SOUL heme-binding
## VIT_18s0001g11950  -  Unknown protein
## VIT_12s0059g01340  -  Armadillo/beta-catenin repeat
## VIT_03s0063g02600  -  Leucine-rich repeat transmembrane protein kinase
## VIT_19s0014g05140  -  R protein disease resistance protein
## VIT_18s0001g13090  -  Proteasome 26S AAA-ATPase subunit (RPT4)
## VIT_06s0080g01100  -  Unknown protein
## VIT_01s0137g00810  -  Acetylglucosaminyltransferase
## VIT_06s0004g02740  -  GATA transcription factor 10
## VIT_07s0005g01080  -  Unknown protein
## VIT_11s0016g04480  -  Acyl ACP-thioesterase
## VIT_07s0005g02000  -  ADP-ribosylation factor A1B
## VIT_02s0025g02430  -  Carbon-carbon lyase
## VIT_04s0023g03090  -  No hit
## VIT_18s0001g05690  -  Protein phosphatase 2C
## VIT_04s0008g04020  -  RD22 [Vitis vinifera]
## VIT_18s0122g00950  -  Unknown protein
## VIT_03s0097g00510  -  Oligopeptide transporter OPT4
## VIT_18s0001g08200  -  MATE efflux family protein ZF14
## VIT_18s0001g08510  -  Lipase family
## VIT_01s0011g03160  -  PFT1 (phytochrome and flowering time 1) MED25
## VIT_00s0349g00020  -  Unknown protein
## VIT_11s0016g02480  -  2,3-diketo-5-methylthio-1-phosphopentane phosphatase
## VIT_06s0004g07970  -  Expansin  (VvEXPA7)
## VIT_05s0077g00430  -  Galactinol synthase
## VIT_12s0028g03650  -  Two-pore calcium channel (TPC1)
## VIT_08s0032g00620  -  Ribosomal protein L22
## VIT_04s0008g07400  -  NAD dependent epimerase/dehydratase
## VIT_13s0019g04450  -  ATSYTA/NTMC2T1.1/NTMC2TYPE1.1/SYTA
## VIT_13s0064g01270  -  TRNA/rRNA methyltransferase (SpoU)
## VIT_04s0044g01500  -  NAC domain-containing protein (VvNAC47)
## VIT_01s0010g02220  -  Zinc finger (C3HC4-type ring finger)
## VIT_04s0008g06050  -  Phytosulfokine receptor precursor
## VIT_13s0067g03180  -  Unknown protein
## VIT_18s0001g14780  -  Lipase 3 (EXL3) family II extracellular
## VIT_19s0014g00070  -  Solanesyl diphosphate synthase
## VIT_13s0019g03760  -  Lateral organ boundaries protein 11
## VIT_13s0064g00480  -  Beta-primeverosidase
## VIT_01s0137g00230  -  Regulator of chromosome condensation (RCC1)
## VIT_03s0038g02980  -  Mechanosensitive ion channel
## VIT_00s1764g00010  -  1-aminocyclopropane-1-carboxylate synthase
## VIT_15s0046g00490  -  Wax synthase
## VIT_07s0005g03610  -  NAC domain-containing protein (VvNAC56)
## VIT_07s0104g00640  -  No hit
## VIT_08s0007g07580  -  MYR1 (MYB-related protein 1)
```

```
## VIT_13s0084g00800  -  Transcription factor
## VIT_01s0011g02200  -  Glutamine synthetase
## VIT_10s0071g00500  -  GRAM domain-containing protein / ABA-responsive
## VIT_10s0071g00590  -  GRAM domain-containing protein / ABA-responsive
## VIT_19s0014g02590  -  (-)-germacrene D synthase (VvTPS24), Selina-411-diene/Interme deol sy[illegible]
## VIT_18s0001g09900  -  No hit
## VIT_13s0073g00050  -  Cationic amino acid transporter 6
## VIT_18s0001g08500  -  Lipase family
## VIT_18s0122g00360  -  DC1 domain-containing protein
## VIT_18s0089g00860  -  Ring-H2 finger protein ATL4E
## VIT_05s0049g00750  -  No hit
## VIT_11s0065g00170  -  Squamosa promoter-binding protein (VvSBP10)
## VIT_14s0006g02600  -  Wall-associated kinase 4
## VIT_03s0038g02190  -  Nodulin
## VIT_02s0025g01190  -  Extra-large G-protein (XLG1)
## VIT_15s0046g03600  -  (+)-neomenthol dehydrogenase
## VIT_10s0071g00430  -  GRAM domain-containing protein / ABA-responsive
## VIT_14s0060g00900  -  Unknown protein
## VIT_04s0023g03550  -  Thaumatin
## VIT_14s0030g00340  -  Sugar transporter ERD6-like 8
## VIT_15s0046g00480  -  Wax synthase
## VIT_09s0002g02500  -  Ubiquitin-specific protease 6 (UBP6)
## VIT_10s0042g01360  -  Unknown protein
## VIT_05s0049g00690  -  No hit
## VIT_08s0007g02960  -  VPS5A subunit of retromer complex
## VIT_10s0071g00390  -  GRAM domain-containing protein / ABA-responsive
## VIT_05s0049g00680  -  No hit
## VIT_04s0008g05270  -  ATPase AFG1
## VIT_04s0023g03540  -  Thaumatin SCUTL1
```

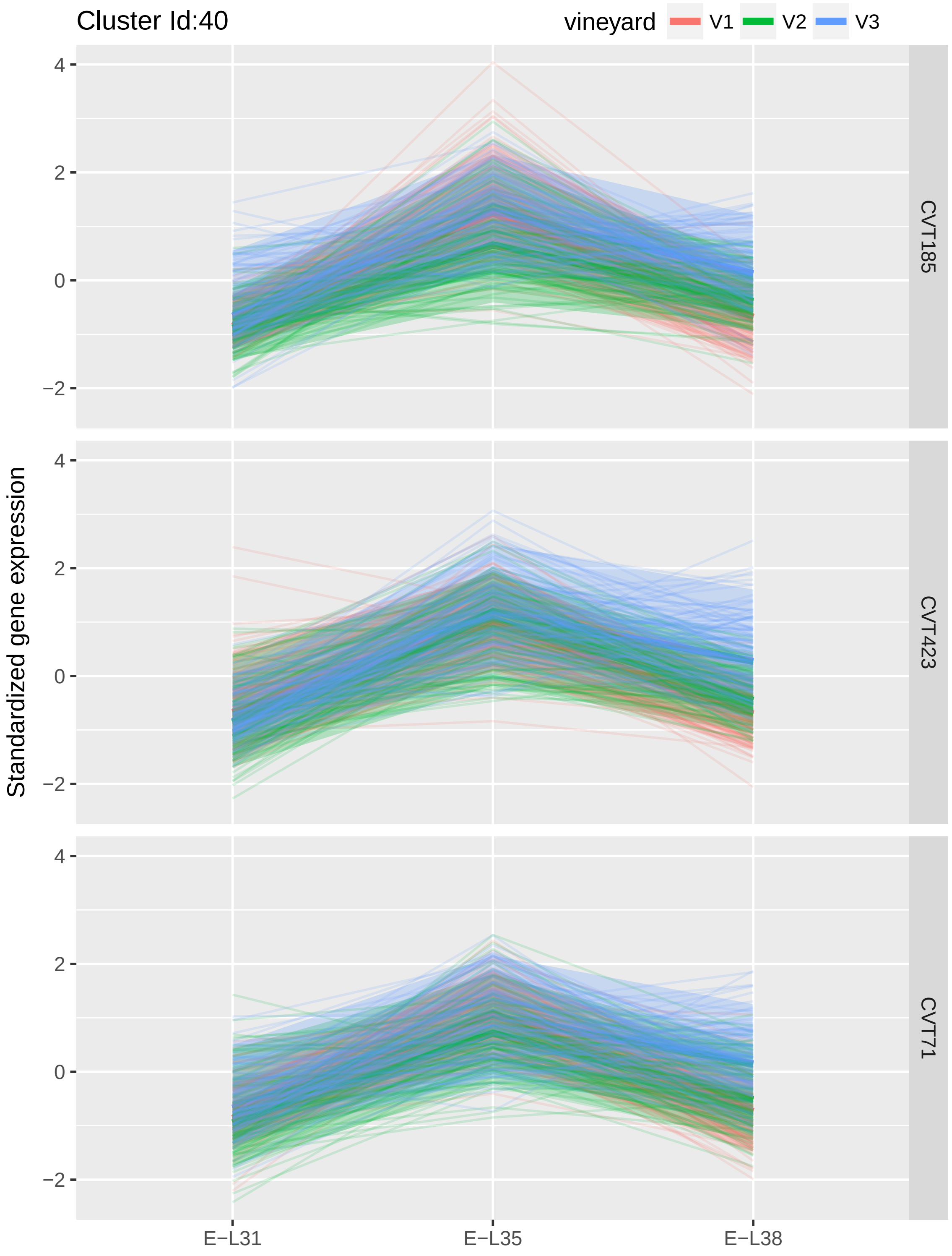

Cluster Id:40
vineyard
V1
V2
V3
Standardized gene expression
CVT185
CVT423
CVT71
4
2
0
−2
E−L31
E−L35
E−L38

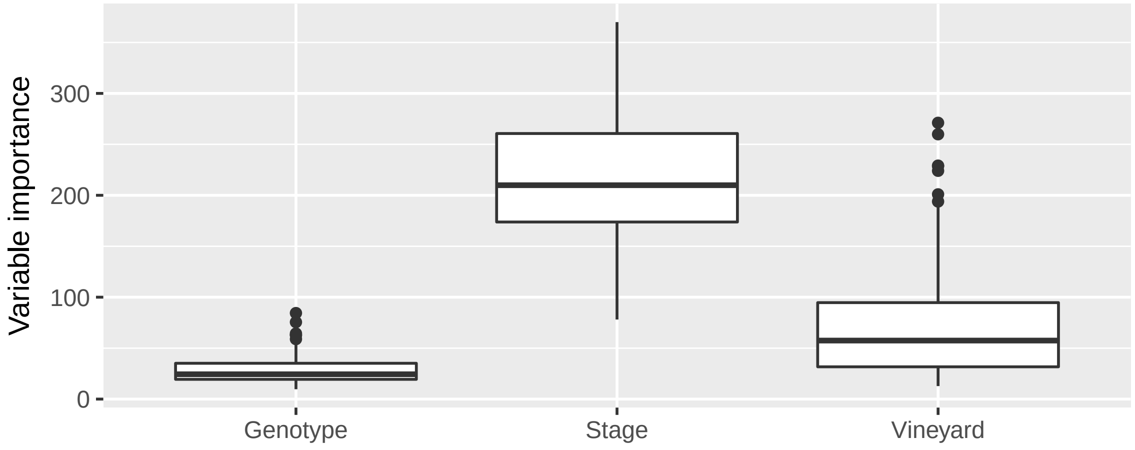

Variable importance
300
200
100
0
Genotype
Stage
Vineyard

Cluster no. 41

```
## Number of genes in the cluster: 129
## Homogeneity Index:    0.81
## Variable importance for Stage:    Median =  507.8  - Rank =  32
## Variable importance for Clone:    Median =  13.15  - Rank =  82
## Variable importance for Vineyard: Median =  24.55  - Rank =  78
##
## Gene ID               Gene Annotation
## VIT_18s0001g11740  -  Ring zinc finger ariadne protein ARI2
## VIT_00s0181g00130  -  Riboflavin kinase
## VIT_14s0006g03200  -  Chaperonin
## VIT_02s0025g04780  -  Subtilisin stomatal density and distribution
## VIT_07s0031g01330  -  Alanine:glyoxylate aminotransferase 2
## VIT_18s0001g15630  -  MAP kinase activating protein
## VIT_01s0026g00240  -  Spermidine synthase
## VIT_06s0061g01160  -  Unknown protein
## VIT_02s0025g03790  -  ATP-dependent RNA helicase DDX6/DHH1
## VIT_17s0000g02430  -  Phosphoethanolamine N-methyltransferase
## VIT_05s0020g02160  -  Squamosa promoter-binding protein (VvSBP6)
## VIT_04s0023g03820  -  3-dehydroquinate synthase
## VIT_07s0031g03090  -  Avr9 elicitor response protein
## VIT_03s0038g04680  -  Isoflavone reductase Bet v 6.0101
## VIT_16s0039g02260  -  RelA/SpoT protein (RSH3)
## VIT_02s0087g00400  -  Pectinacetylesterase
## VIT_03s0017g01760  -  Gamma-aminobutyrate transaminase subunit isozyme 1
## VIT_12s0057g01270  -  Acyl-CoA synthetase long-chain member 8
## VIT_19s0027g00470  -  SNARE-associated protein
## VIT_16s0098g01640  -  No hit
## VIT_18s0001g01580  -  PHD finger transcription factor
## VIT_07s0005g01910  -  Myb family transcription factor DNA methyltransferase 1 associated
## VIT_11s0016g05700  -  RabGAP/TBC domain-containing protein
## VIT_11s0037g00250  -  1-acyl-sn-glycerol-3-phosphate acyltransferase 1, chloroplast precurso
## VIT_06s0004g01770  -  Acetyl-CoA carboxylase, biotin carboxylase
## VIT_01s0026g02490  -  Apyrase
## VIT_08s0040g02810  -  Lung seven transmembrane receptor 1
## VIT_14s0030g01440  -  EDR1 (Enhanced disease resistance 1)
## VIT_13s0158g00160  -  Unknown protein
## VIT_14s0171g00140  -  Lys Motif-Type Receptor-Like Kinase LYK10
## VIT_11s0016g03320  -  Oligosaccharyl transferase STT3 subunit
## VIT_03s0091g00270  -  TIP41
## VIT_07s0005g01460  -  Dipeptidyl aminopeptidases/acylaminoacyl-peptidases
## VIT_12s0028g00830  -  Pleckstriny (PH) domain-containing protein
## VIT_05s0020g01610  -  MYB related DIRP family protein
## VIT_00s0287g00050  -  Unknown protein
## VIT_05s0020g00370  -  No hit
## VIT_12s0059g02050  -  Unknown protein
## VIT_07s0005g00310  -  Zinc finger (CCCH type) helicase family protein
## VIT_00s0463g00010  -  WRKY DNA-binding protein 20  (WRKY-6), WRKY Transcription Factor (VvWR
## VIT_15s0046g03720  -  Unknown
## VIT_06s0004g03190  -  Proteasome 20S beta subunit G1 (PBG1) (PRCH)
## VIT_19s0014g01860  -  RNA exonuclease 4
## VIT_07s0005g02420  -  Haloacid dehalogenase hydrolase
## VIT_11s0016g00970  -  Ring-box protein RBX
## VIT_14s0060g00580  -  FACT complex subunit SPT16
## VIT_09s0002g05750  -  Bromo adjacent region
```

```
## VIT_15s0048g02490  -  Catechol O-methyltransferase
## VIT_06s0061g01050  -  Protein kinase SRPK1
## VIT_06s0004g00250  -  PUMILIO 12 (APUM12)
## VIT_01s0137g00620  -  Auxin-independent growth promoter
## VIT_03s0038g04700  -  Isoflavone reductase
## VIT_10s0003g05000  -  VTC2 (vitamin C defective)
## VIT_14s0128g00250  -  Nuclear transcription factor Y subunit C-9
## VIT_19s0093g00470  -  Transcription initiation factor TFIID subunit 11
## VIT_15s0046g00460  -  Phosphate/phosphoenolpyruvate translocator protein
## VIT_11s0103g00580  -  Unknown protein
## VIT_01s0011g00570  -  Glucan endo-1,3-beta-glucosidase 3 precursor
## VIT_10s0092g00120  -  Gamma-soluble NSF attachment protein (GSNAP)
## VIT_05s0077g01700  -  Copper-binding family protein
## VIT_07s0031g01280  -  Zinc finger (C3HC4-type ring finger)
## VIT_19s0027g00810  -  Per1
## VIT_05s0049g01830  -  CCT motif constans-like
## VIT_01s0026g00320  -  NHL repeat-containing protein
## VIT_15s0021g01580  -  COP9 signalosome complex subunit 7
## VIT_15s0046g01020  -  Nucleotidyltransferase
## VIT_19s0014g00020  -  ATP-dependent Clp protease ATP-binding subunit (ClpA)
## VIT_17s0000g02870  -  S-adenosyl-L-methionine:salicylic acid carboxyl methyltransferase
## VIT_10s0003g00500  -  NAC domain-containing protein (VvNAC38)
## VIT_14s0030g01820  -  Tetratricopeptide repeat domain 35
## VIT_08s0056g00440  -  Zinc finger (C3HC4-type ring finger)
## VIT_02s0012g00480  -  Regulator of nonsense transcripts 1
## VIT_07s0005g03640  -  Asparagine N-glycosyltransferase
## VIT_08s0058g00340  -  Unknown protein
## VIT_18s0001g03070  -  SPla/RYanodine receptor (SPRY) domain-containing protein
## VIT_00s0252g00010  -  RecA
## VIT_04s0023g00670  -  EIF4-gamma/eIF5/eIF2-epsilon domain-containing protein
## VIT_04s0044g01090  -  Chromatin assembly factor-2 (FASCIATA2) (FAS2)
## VIT_04s0023g03900  -  Zinc finger (FYVE type)
## VIT_04s0044g01770  -  Serine/threonine protein phosphatase (PP7)
## VIT_14s0060g00480  -  S-adenosylmethionine synthetase 1 (SAM1)
## VIT_06s0004g08050  -  Telomere repeat binding factor like TRFL7
## VIT_15s0048g02480  -  Caffeate 3-O-methyltransferase 1
## VIT_00s0199g00030  -  Ubiquitin-specific protease 16
## VIT_14s0083g00580  -  S-adenosyl-L-methionine decarboxylase
## VIT_15s0021g02700  -  Expansin  (VvEXPB4)
## VIT_10s0042g01200  -  ARGONAUTE 2 (AGO2)
## VIT_17s0000g03650  -  Unknown protein
## VIT_16s0039g01610  -  V-type H+-transporting ATPase 54 kD subunit
## VIT_13s0084g00150  -  Choline kinase
## VIT_13s0073g00310  -  Pyridine nucleotide-disulphide oxidoreductase
## VIT_05s0077g00860  -  3-hydroxybutyryl-CoA dehydratase
## VIT_07s0031g00840  -  Unknown protein
## VIT_16s0013g01680  -  L-asparaginase 3 precursor
## VIT_07s0104g00080  -  Unknown protein
## VIT_17s0000g04140  -  Unknown protein
## VIT_05s0049g01470  -  Unknown protein
## VIT_14s0068g01310  -  No hit
## VIT_14s0066g02390  -  Golgi alpha-mannosidase II
## VIT_14s0108g01580  -  myb family transcription factor / ELM2 domain-containing
## VIT_02s0012g02790  -  Secretory carrier membrane protein (SCAMP4)
## VIT_00s0215g00040  -  Proteasome 26S regulatory subunit (RPN2)
```

```
## VIT_10s0003g03280  -  Alpha-mannosidase
## VIT_12s0028g03360  -  Serine/threonine-protein kinase bub1,checkpoint-associated
## VIT_14s0060g00420  -  Pyruvate dehydrogenase kinase
## VIT_18s0001g12230  -  Unknown protein
## VIT_00s0252g00070  -  Translation initiation factor eIF-3 subunit 5
## VIT_02s0087g00780  -  No hit
## VIT_09s0002g08930  -  Calcium ion binding
## VIT_08s0032g00600  -  Ubiquitin-specific protease 12 (UBP12)
## VIT_09s0002g00280  -  Ubiquitin-conjugating enzyme E2 J2
## VIT_09s0054g00700  -  Vacuolar-pyrophosphatase like protein 1 AVPL1
## VIT_18s0166g00180  -  Zinc finger protein ATRZ-1A
## VIT_10s0003g05010  -  No hit
## VIT_18s0001g15370  -  Exostosin family protein
## VIT_07s0031g03130  -  PHD finger transcription factor
## VIT_16s0039g02200  -  E3 ubiquitin-protein ligase synoviolin
## VIT_03s0063g02060  -  Phospholipase A-2-activating protein
## VIT_00s1406g00010  -  Katanin p80 (WD repeat containing) subunit B 1
## VIT_07s0031g00820  -  Methionine aminopeptidase 1B, chloroplast precursor
## VIT_11s0016g00850  -  RecQl3 (Recq 3); ATP binding / ATP-dependent helicase
## VIT_14s0068g00780  -  Nematode chemoreceptor
## VIT_00s0201g00070  -  HhH-GPD base excision DNA repair family protein
## VIT_17s0000g01420  -  Snurportin-1 (RNA U transporter 1)
## VIT_06s0009g00250  -  Large subunit GTPase 1
## VIT_08s0007g08480  -  Zinc proteinase
## VIT_13s0067g00280  -  Syntaxin 5
## VIT_10s0003g01120  -  No hit
## VIT_12s0028g02670  -  NAC domain-containing protein (VvNAC35)
```

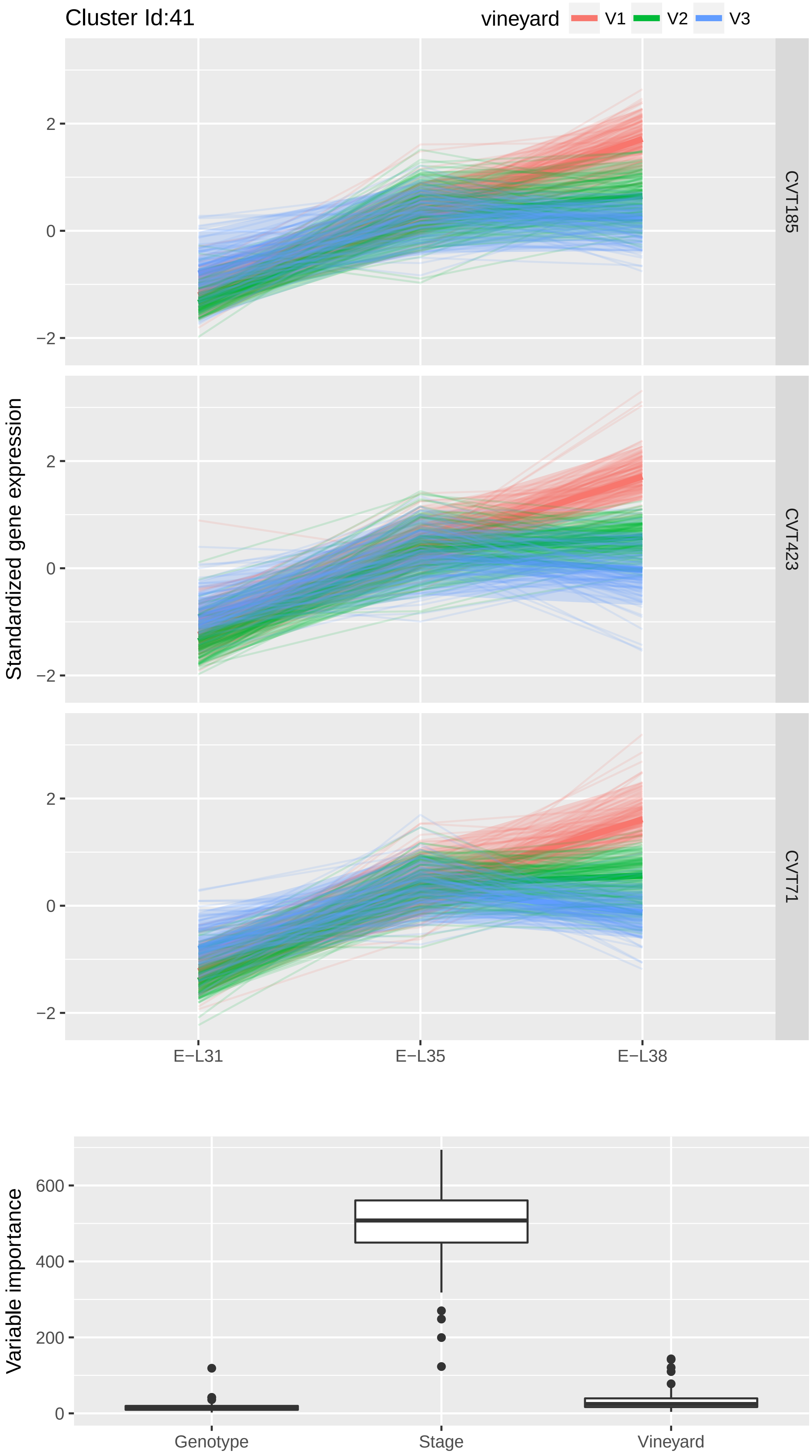

Cluster Id:41
vineyard
V1
V2
V3
Standardized gene expression
CVT185
CVT423
CVT71
2
0
−2
E−L31
E−L35
E−L38
Variable importance
600
400
200
0
Genotype
Stage
Vineyard

Cluster no. 42

```
## Number of genes in the cluster: 293
## Homogeneity Index:    0.88
## Variable importance for Stage:    Median =  637.7  - Rank =  8
## Variable importance for Clone:    Median =  10.65  - Rank =  101
## Variable importance for Vineyard: Median =  11.81  - Rank =  111
##
## Gene ID               Gene Annotation
## VIT_16s0050g00900  -  Anthocyanin permease (VvAnthoMATE3 - VvAM3)
## VIT_18s0001g10100  -  Ubiquitin-conjugating enzyme E2 I
## VIT_05s0077g00510  -  Beta-fructofuranosidase
## VIT_18s0041g00480  -  Proton-dependent oligopeptide transport (POT) family protein
## VIT_01s0011g04160  -  Phosphatidic acid phosphatase alpha
## VIT_08s0007g01350  -  No hit
## VIT_12s0028g03830  -  Thioredoxin M-type 2
## VIT_13s0019g00250  -  Ribosomal protein L23A (RPL23aA) 60S
## VIT_12s0059g02360  -  Myb CAPRICE (VvCPC1)
## VIT_19s0093g00350  -  Glutathione S-transferase 25 GSTU25
## VIT_05s0049g00400  -  Desacetoxyvindoline 4-hydroxylase
## VIT_13s0067g02220  -  Amino acid permease
## VIT_17s0000g09170  -  Receptor lectin kinase
## VIT_12s0057g01250  -  ribulose-1,5 bisphosphate carboxylase oxygenase large subunit N-methyl
## VIT_00s0203g00040  -  Vesicle-associated membrane protein
## VIT_08s0032g00560  -  No hit
## VIT_09s0002g02640  -  Ribosomal protein S30 (RPS30C) 40S
## VIT_01s0011g01460  -  AICARFT/IMPCHase bienzyme; MGS
## VIT_08s0007g08990  -  Unknown protein
## VIT_16s0098g00940  -  Plastid-specific 30S ribosomal protein 1
## VIT_16s0098g00200  -  Receptor serine/threonine kinase PR5K
## VIT_11s0016g01730  -  VIP3 (vernalization independence 3)
## VIT_06s0061g00240  -  ABC transporter G member 7
## VIT_03s0038g03060  -  Transposase
## VIT_01s0026g00540  -  Nodulin MtN21 family
## VIT_14s0171g00120  -  Unknown protein
## VIT_13s0147g00160  -  Aluminium-tolerance ALMT1
## VIT_17s0053g00840  -  Cyclic nucleotide-binding transporter 1
## VIT_01s0011g02070  -  Metal-dependent phosphohydrolase HD domain-containing protein
## VIT_14s0108g01330  -  Chorismate mutase, chloroplast (CM1)
## VIT_13s0158g00440  -  Disease resistance protein
## VIT_13s0067g02400  -  Myosin heavy chain
## VIT_00s0259g00140  -  Oligopeptide transporter OPT3
## VIT_13s0019g04680  -  Ras-related protein Rab-5B
## VIT_08s0058g00040  -  Verticillium wilt disease resistance protein Ve2
## VIT_13s0067g01640  -  Lectin-like receptor kinase 7;2
## VIT_18s0122g00430  -  Photosystem II light harvesting complex gene 2.3
## VIT_17s0000g02650  -  myb domain protein 7
## VIT_04s0023g02070  -  Trichohyalin AUL1 (auxin-like 1 protein)
## VIT_02s0025g02510  -  Metal-nicotianamine transporter YSL1
## VIT_12s0059g01670  -  Nuclear ribonuclease Z
## VIT_06s0009g03600  -  Wall-associated receptor kinase-like 14
## VIT_05s0020g04190  -  PINHEAD (Protein ZWILLE)
## VIT_00s0259g00120  -  Oligopeptide transporter OPT3
## VIT_07s0151g00440  -  AP2 domain containing protein
## VIT_02s0025g00310  -  Ribosomal protein S10 (RPS10C) 40S
## VIT_08s0056g00750  -  far-red impaired responsive protein
```

```
## VIT_19s0015g01010  -  Farnesyl diphosphate synthase
## VIT_04s0008g02000  -  Unknown protein
## VIT_01s0026g02680  -  Oxygen evolving enhancer 3 (PsbQ)
## VIT_14s0060g02670  -  No hit
## VIT_11s0206g00110  -  Serine carboxypeptidase S28
## VIT_08s0040g03150  -  L-ascorbate peroxidase 1, cytosolic (APX1)
## VIT_03s0180g00070  -  Cyclase
## VIT_17s0000g08010  -  Trehalose 6-phosphate synthase
## VIT_13s0156g00540  -  Disease resistance protein (CC-NBS-LRR class)
## VIT_06s0061g00800  -  Unknown protein
## VIT_17s0000g10070  -  Zinc finger (C3HC4-type ring finger)
## VIT_04s0044g01270  -  Calcineurin phosphoesterase
## VIT_18s0001g08170  -  Inorganic pyrophosphatase
## VIT_11s0206g00150  -  Unknown protein
## VIT_06s0061g00480  -  Alpha-L-fucosidase 1 precursor
## VIT_18s0001g12200  -  CYP721A1
## VIT_06s0009g01150  -  La domain-containing protein
## VIT_19s0014g02320  -  Unknown protein
## VIT_03s0038g00450  -  Squalene synthase
## VIT_14s0068g01120  -  Alpha-mannosidase precursor lysosomal
## VIT_14s0006g01620  -  myb domain protein 4 (VvMybC2-L3)
## VIT_14s0066g01650  -  Nodulin MtN21 family
## VIT_05s0049g00310  -  E8 protein
## VIT_14s0068g00500  -  Indole-3-acetate beta-glucosyltransferase
## VIT_02s0154g00060  -  Thylakoid lumenal protein
## VIT_15s0048g02090  -  Thioesterase family
## VIT_05s0020g00480  -  Mitochondrial substrate carrier family protein
## VIT_14s0030g00640  -  DnaJ homolog, subfamily B, member 12
## VIT_18s0089g01020  -  Ethylene-responsive transcription factor
## VIT_06s0004g05740  -  IMP dehydrogenase/GMP reductase
## VIT_19s0015g00660  -  E3 ubiquitin-protein ligase HUWE1
## VIT_03s0038g02320  -  L-ascorbate peroxidase.
## VIT_15s0046g01400  -  Ubiquitin-conjugating enzyme E2 D/E
## VIT_17s0119g00050  -  UBQ5 (ubiquitin 5)
## VIT_17s0000g05820  -  Ubiquitin-conjugating enzyme E2 A
## VIT_08s0007g03860  -  S-receptor kinase
## VIT_18s0072g00790  -  RPD1 (root primordium defective 1)
## VIT_08s0032g00190  -  F-box family protein
## VIT_04s0023g01820  -  ADP-ribosylation factor
## VIT_17s0000g07970  -  RDR2 (RNA-dependent RNA polymerase 2)
## VIT_14s0030g00140  -  CC-NBS-LRR class
## VIT_05s0020g04850  -  H1flk
## VIT_09s0002g02980  -  FRK1 (FLG22-induced receptor-like kinase 1)
## VIT_11s0016g01130  -  MTD1
## VIT_11s0118g00800  -  KNAT2 (knotted1-like homeobox gene 3)
## VIT_13s0156g00020  -  SC35 splicing factor SCL30a, 30a kD
## VIT_14s0006g03260  -  Rhomboid
## VIT_15s0046g01080  -  Glycosyl hydrolase family 18
## VIT_14s0066g00500  -  Nuclear pore complex protein Nup210
## VIT_02s0033g00190  -  DNA replication licensing factor MCM2
## VIT_01s0011g01800  -  Inositol monophosphatase family protein
## VIT_13s0074g00620  -  Kelch repeat-containing protein
## VIT_17s0000g03800  -  Unknown protein
## VIT_17s0000g00900  -  Glutamate 1-semialdehyde aminotransferase
## VIT_06s0004g00940  -  Carbamoyl-phosphate synthase, large subunit
```

```
## VIT_03s0088g01250  -  D-threo-aldose 1-dehydrogenase
## VIT_09s0002g04440  -  Ribosomal protein L37 (RPL37C) 60S
## VIT_02s0025g04840  -  Nudix hydrolase 14
## VIT_02s0025g02880  -  Subtilase
## VIT_19s0090g00810  -  Imidazole glycerol phosphate synthase hisHF, chloroplast
## VIT_07s0141g00610  -  Zinc finger (C3HC4-type ring finger)
## VIT_04s0023g00160  -  Haloacid dehalogenase hydrolase
## VIT_04s0008g05190  -  Overexpressor of cationic peroxidase 3 (OCP3)
## VIT_14s0006g02810  -  Unknown protein
## VIT_02s0025g00300  -  No hit
## VIT_08s0007g05650  -  Unknown protein
## VIT_06s0004g05640  -  Pentatricopeptide (PPR) repeat-containing protein
## VIT_01s0011g03680  -  ER6 protein universal stress protein (USP) family
## VIT_17s0000g05890  -  Protein kinase APK1B
## VIT_08s0058g00390  -  Ribosomal protein S4 (RPS4D) 40S
## VIT_06s0004g05950  -  Unknown protein
## VIT_19s0014g03160  -  NADPH:protochlorophyllide oxidoreductase
## VIT_18s0041g00230  -  TIR-NBS-LRR disease resistance
## VIT_15s0048g01810  -  fructosamine kinase
## VIT_11s0037g00330  -  Unknown protein
## VIT_17s0000g00980  -  Ribosomal protein L7A (RPL7aB) 60S
## VIT_01s0011g04270  -  Receptor kinase homolog LRK10
## VIT_02s0154g00520  -  Aspartyl protease
## VIT_08s0040g01610  -  2-phosphoglycerate kinase
## VIT_03s0038g04180  -  Pigment defective 191 (PDE191)
## VIT_11s0052g01660  -  Serine esterase
## VIT_17s0000g08670  -  Glutaredoxin
## VIT_03s0038g03180  -  flavin-containing monooxygenase 1
## VIT_08s0040g00710  -  3'-5' exonuclease domain-containing protein
## VIT_00s0203g00060  -  3'(2'),5'-bisphosphate nucleotidase
## VIT_17s0000g10280  -  Nucleotide diphosphate kinase II, chloroplast (NDPK2)
## VIT_06s0061g01480  -  ABC Transporter (VvPDR21 - VvABCG51)
## VIT_06s0061g00670  -  Salutaridine reductase
## VIT_14s0108g00350  -  3-dehydroquinate synthase
## VIT_12s0057g01260  -  Unknown protein
## VIT_13s0067g01450  -  Lateral organ boundaries domain 21
## VIT_19s0090g01460  -  SWIB complex BAF60b domain-containing protein
## VIT_14s0030g01970  -  Auxin-induced protein PCNT115
## VIT_01s0011g06200  -  Zinc finger protein 4
## VIT_02s0033g01370  -  Sodium:solute symporter family protein
## VIT_19s0014g04970  -  Ribosomal protein L24, chloroplast (CL24) 50S
## VIT_12s0134g00510  -  Cytochrome B6-F complex iron-sulfur subunit, PETC
## VIT_04s0044g00190  -  Mannitol dehydrogenase
## VIT_16s0022g00630  -  ABC transporter E member 1
## VIT_11s0016g04840  -  Avr9/Cf-9 rapidly elicited protein 11
## VIT_07s0129g00420  -  Unknown protein
## VIT_15s0046g01480  -  Unknown protein
## VIT_00s0203g00140  -  Octicosapeptide/Phox/Bem1p (PB1) domain-containing protein
## VIT_13s0084g00880  -  NADPH:quinone oxidoreductase
## VIT_07s0141g00260  -  Unknown protein
## VIT_08s0040g00070  -  Argonaute
## VIT_08s0058g00610  -  Binding
## VIT_05s0020g01630  -  Transcription termination factor mitochondrial mTERF
## VIT_15s0046g02690  -  Ribosomal protein L36 (RPL36C) 60S
## VIT_18s0001g03880  -  Polcalcin
```

```
## VIT_08s0007g05350  -  Haloacid dehalogenase hydrolase
## VIT_13s0067g00360  -  No hit
## VIT_19s0090g01820  -  CBS domain-containing protein
## VIT_18s0001g07650  -  VIN3
## VIT_10s0003g00300  -  Acclimation of photosynthesis to environment
## VIT_05s0029g00020  -  Ribosomal protein L13A (RPL13aD) 60S
## VIT_11s0103g00220  -  Ribosomal protein P3 (RPP3B) acidic 60S
## VIT_11s0016g00390  -  Zinc finger (C3HC4-type ring finger)
## VIT_10s0003g02660  -  RRNA processing protein RimM
## VIT_07s0104g00210  -  Phenazine biosynthesis PhzC/PhzF protein
## VIT_12s0035g01950  -  4-diphosphocytidyl-2-C-methyl-D-erythritol synthase
## VIT_11s0016g02810  -  Glycine-rich protein
## VIT_07s0031g00780  -  Transcriptional factor B3
## VIT_18s0041g00290  -  Zinc finger (C3HC4-type ring finger)
## VIT_02s0025g02830  -  Beta-1,3-galactosyltransferase sqv-2
## VIT_00s0199g00150  -  Threonine endopeptidase
## VIT_19s0014g03360  -  Unknown protein
## VIT_14s0128g00880  -  MYB related DIRP family protein
## VIT_15s0046g02910  -  Ribosomal protein L21, chloroplast / CL21 (RPL21) 50S
## VIT_05s0049g01280  -  COP1-interacting protein 7
## VIT_16s0013g00650  -  Histone-lysine N-methyltransferase, H3 lysine-9 specific SUVH5
## VIT_03s0017g01330  -  1,4-dihydroxy-2-naphthoate octaprenyltransferase
## VIT_16s0098g00190  -  Receptor kinase homolog LRK10
## VIT_05s0077g01730  -  Unknown
## VIT_16s0050g01230  -  No hit
## VIT_00s0203g00150  -  Octicosapeptide/Phox/Bem1p (PB1) domain-containing protein
## VIT_02s0154g00050  -  Glycosyl hydrolase family 5
## VIT_06s0009g02450  -  Integrase
## VIT_12s0142g00690  -  No hit
## VIT_02s0012g01420  -  Pigment defective 322
## VIT_16s0100g01210  -  6-phospho 3-hexuloisomerase
## VIT_13s0019g01660  -  Dehydration-induced protein (ERD15)
## VIT_12s0059g00290  -  Calmodulin-binding region IQD33
## VIT_02s0012g00550  -  Inositol polyphosphate 5-phosphatase II
## VIT_13s0019g02330  -  GDP-mannose pyrophosphorylase (GMP1)
## VIT_15s0046g01850  -  Syntaxin of plants SYP7
## VIT_04s0044g00020  -  Catalase [Vitis vinifera]
## VIT_05s0094g00780  -  Cysteine synthase
## VIT_01s0011g02330  -  Unknown protein
## VIT_00s0698g00010  -  Catalase
## VIT_17s0000g06980  -  Ribosomal protein S19 (RPS19C) 40S
## VIT_08s0007g07880  -  Polygalacturonase GH28
## VIT_14s0066g02250  -  Leucine-rich repeat transmembrane protein kinase
## VIT_09s0002g04130  -  Proton-dependent oligopeptide transport (POT) family protein
## VIT_05s0124g00400  -  2-isopropylmalate synthase B
## VIT_00s0338g00020  -  Unknown protein
## VIT_04s0008g07340  -  Constans-like 4
## VIT_03s0063g00610  -  ARP protein (REF)
## VIT_04s0023g00870  -  Peptide chain release factor
## VIT_13s0147g00210  -  Unknown protein
## VIT_08s0007g00920  -  Tropinone reductase
## VIT_17s0000g05410  -  Galactosyltransferase
## VIT_06s0004g05850  -  Zinc finger (C3HC4-type ring finger)
## VIT_05s0049g01500  -  DAG protein, chloroplast precursor
## VIT_01s0011g05690  -  SET domain-containing protein
```

```
## VIT_07s0005g00690  -  Ribosomal protein L18 (RPL18B) 60S
## VIT_06s0080g00320  -  Esterase/lipase/thioesterase
## VIT_18s0001g10640  -  No hit
## VIT_02s0025g05010  -  Ycf49
## VIT_06s0004g06990  -  Phytol kinase 2, chloroplast precursor
## VIT_05s0020g00870  -  UbiE/COQ5 methyltransferase
## VIT_00s0181g00170  -  F-box family protein
## VIT_05s0102g00590  -  No hit
## VIT_14s0068g01070  -  SUVH4 (SU(VAR)3-9 homolog 4)
## VIT_03s0091g00050  -  Triacylglycerol/steryl ester hydrolase
## VIT_13s0019g02320  -  No hit
## VIT_17s0000g08540  -  Lipase GDSL
## VIT_14s0068g01090  -  SUVH4 (SU(VAR)3-9 homolog 4)
## VIT_06s0004g05430  -  Tropinone reductase
## VIT_06s0004g05680  -  Glutathione S-transferase 25 GSTU7
## VIT_02s0025g01270  -  Unknown protein
## VIT_13s0019g04050  -  TET6 (tetraspanin6)
## VIT_00s0207g00110  -  Antigen receptor
## VIT_15s0046g03570  -  (+)-neomenthol dehydrogenase
## VIT_00s0370g00070  -  Epoxide hydrolase
## VIT_19s0085g00860  -  Aldehyde Dehydrogenase (VvALDH5F3)
## VIT_16s0050g01990  -  Unknown
## VIT_09s0002g01600  -  PHD finger protein alfin
## VIT_00s0252g00020  -  Unknown
## VIT_15s0021g01640  -  Unknown protein
## VIT_04s0008g04000  -  Dehydration-responsive protein (RD22)
## VIT_17s0000g00040  -  Pentatricopeptide (PPR) repeat-containing
## VIT_05s0029g00680  -  Beta-D-galactosidase
## VIT_11s0065g00470  -  Protein kinase APK1B
## VIT_04s0008g03730  -  4-amino-4-deoxychorismate lyase
## VIT_07s0104g01470  -  Peptide chain release factor 1
## VIT_18s0001g08420  -  Unknown
## VIT_12s0055g01130  -  BTH-induced protein phosphatase 1
## VIT_02s0234g00080  -  Enoyl-CoA hydratase/isomerase
## VIT_02s0025g04470  -  No hit
## VIT_14s0066g00540  -  Nuclear pore complex protein Nup210
## VIT_19s0014g02890  -  Thioredoxin M-type 2
## VIT_17s0000g03620  -  Chaperonin 21, Chloroplast
## VIT_19s0014g00750  -  Leucine-rich repeat protein kinase
## VIT_16s0022g00760  -  Zinc knuckle
## VIT_14s0068g01140  -  DnaJ homolog, subfamily B, member 12
## VIT_05s0124g00390  -  2-isopropylmalate synthase B
## VIT_18s0001g13410  -  V-type H+-transporting ATPase subunit I
## VIT_07s0104g01400  -  Glutaredoxin
## VIT_07s0005g02450  -  Unknown protein
## VIT_12s0035g00760  -  ATP phosphoribosyl transferase
## VIT_07s0031g01230  -  Ribosomal protein L30 (RPL30A) 60S
## VIT_14s0030g00670  -  Dehydroquinate dehydratase
## VIT_19s0090g00630  -  Tetraacyldisaccharide 4'-kinase
## VIT_01s0010g03370  -  Ribosomal protein L17-2 60S
## VIT_16s0022g01460  -  Ribosomal protein S20 (RPS20A) 40S
## VIT_16s0050g02630  -  FtsH protease that is localized to the chloroplast
## VIT_05s0094g00150  -  Membrane-associated 30 kDa protein, chloroplast precursor (M30)
## VIT_14s0066g00490  -  Nuclear pore complex protein Nup210
```

```
## VIT_06s0080g00570  -  Unknown protein
## VIT_18s0001g07430  -  Unknown
## VIT_10s0003g04040  -  Mitochondrial transcription termination factor
## VIT_15s0046g00120  -  Unknown protein
## VIT_17s0000g00520  -  Forever young oxidoreductase
## VIT_13s0067g00180  -  Hydroxyacylglutathione hydrolase
## VIT_14s0066g01820  -  SAG101 (senescence-associated gene 101)
## VIT_00s0181g00160  -  F-box family protein
## VIT_19s0015g02910  -  CYP72A1
## VIT_17s0000g03270  -  CYP77A5P
## VIT_15s0046g02160  -  Proline iminopeptidase
## VIT_05s0020g04600  -  Metallothionein
## VIT_14s0066g02370  -  Transcription factor E2Fe DEL1
## VIT_04s0008g04380  -  Vernalization insensitive 3
## VIT_07s0031g02620  -  Aspartic Protease (VvAP18)
## VIT_00s2015g00010  -  No hit
## VIT_18s0001g07690  -  CTR1-like protein kinase
## VIT_08s0007g07280  -  Superoxide dismutase [Cu-Zn] 2
## VIT_01s0011g04840  -  No hit
## VIT_00s0665g00030  -  Unknown protein
## VIT_11s0016g04230  -  Unknown protein
## VIT_00s0282g00010  -  Methionine sulfoxide reductase
## VIT_14s0036g00470  -  Unknown protein
## VIT_13s0158g00120  -  Protein kinase
## VIT_08s0058g00490  -  Unknown protein
## VIT_01s0127g00420  -  Naphthoate synthase
## VIT_00s0566g00010  -  Unknown protein
```

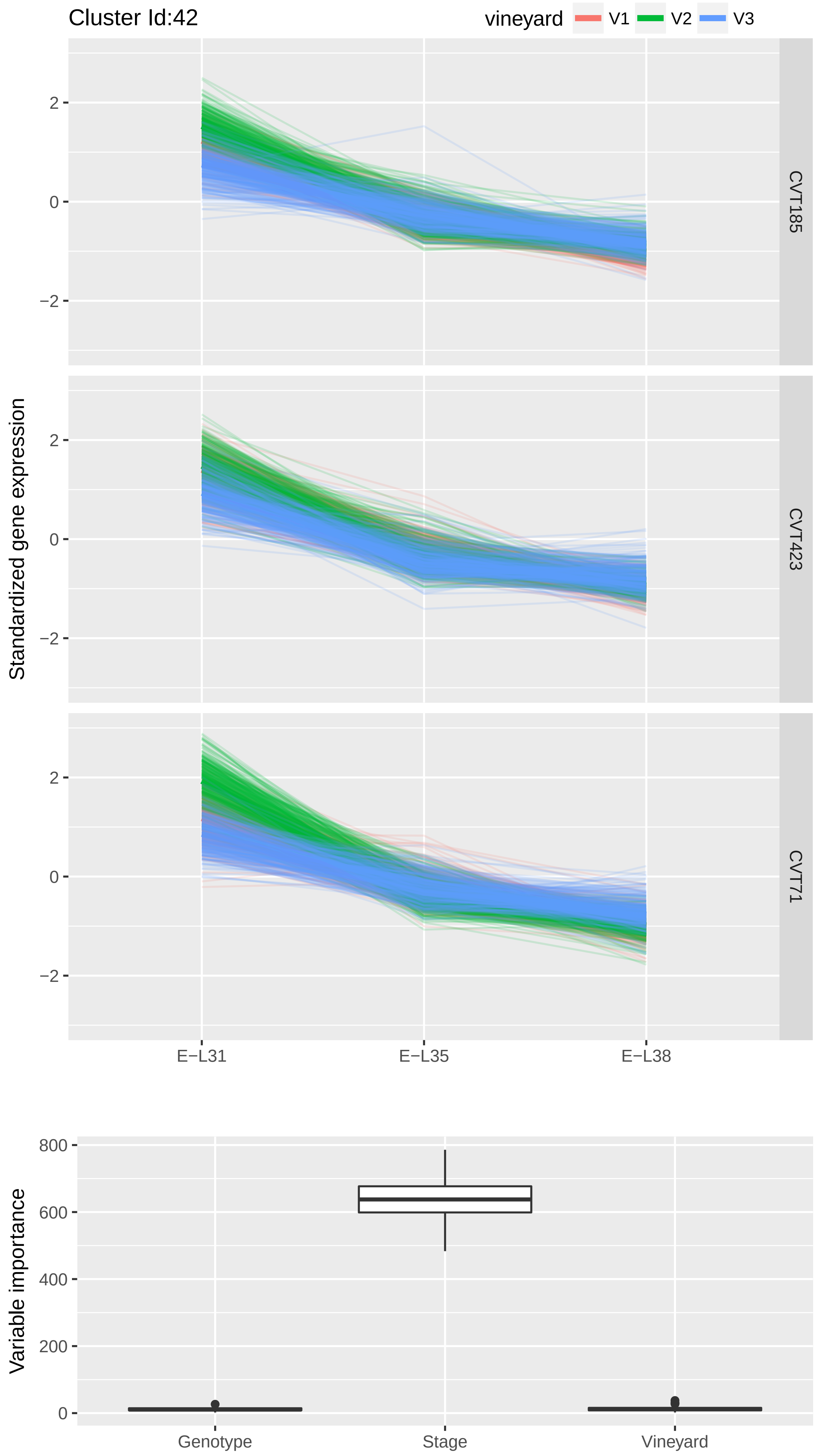

Cluster Id:42
vineyard
V1
V2
V3
CVT185
CVT423
CVT71
Standardized gene expression
2
0
−2
E−L31
E−L35
E−L38
Variable importance
800
600
400
200
0
Genotype
Stage
Vineyard

Cluster no. 43

```
## Number of genes in the cluster: 158
## Homogeneity Index:     0.8
## Variable importance for Stage:    Median =  462.5  - Rank =  43
## Variable importance for Clone:    Median =  17.49  - Rank =  64
## Variable importance for Vineyard: Median =  23.39  - Rank =  79
##
## Gene ID               Gene Annotation
## VIT_02s0241g00100  -  Unknown protein
## VIT_01s0127g00250  -  Kelch repeat-containing F-box protein
## VIT_00s0480g00030  -  Polyphenol oxidase
## VIT_08s0007g02850  -  F-box family protein
## VIT_00s0480g00040  -  Polyphenol oxidase II, chloroplast precursor
## VIT_11s0016g01970  -  Calmodulin-binding region IQD6
## VIT_19s0093g00320  -  Glutathione S-transferase (VvGST1)
## VIT_11s0016g05540  -  Dicyanin
## VIT_09s0054g00560  -  No hit
## VIT_14s0066g01810  -  SAG101 (senescence-associated gene 101)
## VIT_18s0072g00160  -  Peroxidase 12
## VIT_05s0049g01290  -  Protein phosphatase 2C
## VIT_08s0007g01560  -  Photoassimilate-responsive protein
## VIT_04s0043g00220  -  Amine oxidase
## VIT_00s0347g00060  -  S-locus lectin protein kinase
## VIT_03s0017g00650  -  GTP cyclohydrolase II
## VIT_04s0069g00970  -  WRKY Transcription Factor (VvWRKY11)
## VIT_10s0071g00150  -  R protein disease resistance protein
## VIT_19s0090g00840  -  WRKY Transcription Factor (VvWRKY55)
## VIT_19s0014g01530  -  Protein kinase
## VIT_08s0007g03480  -  No hit
## VIT_08s0007g03900  -  Heat stress transcription factor A3
## VIT_12s0059g01380  -  1-aminocyclopropane-1-carboxylate oxidase 1
## VIT_12s0121g00300  -  Brassinosteroid insensitive 1-associated receptor kinase 1
## VIT_12s0134g00480  -  Myb domain protein 14
## VIT_02s0025g02660  -  Transmembrane receptor
## VIT_18s0075g00280  -  Branched-chain amino acid aminotransferase
## VIT_08s0105g00470  -  PHD finger-like domain-containing protein 5A
## VIT_18s0001g11130  -  Calmodulin-binding protein AR781
## VIT_04s0079g00790  -  Acyl-CoA synthetases (Acyl-activating enzyme 11)
## VIT_04s0008g07120  -  Chloroplast nucleoid DNA binding protein
## VIT_09s0002g05610  -  Unknown
## VIT_09s0002g02620  -  Lectin jacalin
## VIT_07s0130g00220  -  Peroxidase ATP32, class III
## VIT_00s0780g00030  -  No hit
## VIT_16s0050g00400  -  Photoassimilate-responsive protein PAR-1a
## VIT_06s0004g02410  -  ATP-NAD kinase
## VIT_16s0039g01060  -  CYP89A2
## VIT_16s0039g01070  -  Glutathione S-transferase 25 GSTU7
## VIT_12s0034g02220  -  RKF3 (receptor-like kinase IN in flowers 3)
## VIT_15s0024g00410  -  Lectin
## VIT_01s0010g00760  -  Unknown
## VIT_17s0000g03510  -  Serine/threonine kinase BRLK
## VIT_06s0061g00680  -  Unknown protein
## VIT_14s0128g00110  -  basic helix-loop-helix (bHLH) family
## VIT_18s0001g06890  -  Peroxidase GvPx2b, class III [Vitis vinifera]
## VIT_19s0090g01070  -  Glucan endo-1,3-beta-glucosidase 7 precursor
```

```
## VIT_04s0008g07220  -  Chloroplast nucleoid DNA binding protein
## VIT_07s0031g02610  -  NAC domain-containing protein (VvNAC39)
## VIT_09s0002g05320  -  2-oxoglutarate-dependent dioxygenase
## VIT_06s0004g05240  -  Ethylene receptor (ETR2)
## VIT_04s0023g01630  -  Steroid 22-alpha-hydroxylase (CYP90B1) (DWF4)
## VIT_01s0011g02630  -  No hit
## VIT_04s0008g07130  -  Aspartic Protease (VvAP7)
## VIT_01s0026g01640  -  Band 7 family
## VIT_15s0046g03430  -  Metal ion binding
## VIT_11s0103g00520  -  Unknown
## VIT_17s0000g03980  -  Peptidyl-tRNA hydrolase
## VIT_07s0005g00950  -  Carrier protein, Mitochondrial
## VIT_18s0001g08040  -  basic helix-loop-helix (bHLH) family
## VIT_05s0094g01080  -  Ankyrin protein kinase
## VIT_03s0038g03220  -  Chitin elicitor-binding CEBIP LysM domain-containing
## VIT_07s0005g00560  -  Ribosomal protein L22 (RPL22C) 60S
## VIT_11s0016g03020  -  Pectinesterase family
## VIT_06s0080g00910  -  F-box family protein
## VIT_19s0014g00830  -  RKF1 (receptor-like kinase in flowers 1)
## VIT_16s0100g00670  -  Homeodomain GLABROUS1
## VIT_18s0001g10260  -  Zinc finger (C3HC4-type ring finger)
## VIT_18s0001g12450  -  No hit
## VIT_03s0017g02260  -  Protease inhibitor/seed storage/lipid transfer protein (LTP)
## VIT_15s0046g03500  -  Metal ion binding
## VIT_05s0102g00710  -  fructokinase-1
## VIT_07s0129g00280  -  Unknown protein
## VIT_04s0023g03440  -  AP endonuclease 2
## VIT_04s0008g07140  -  Chloroplast nucleoid DNA binding protein
## VIT_19s0014g00160  -  LHCII type I CAB-1
## VIT_11s0037g00890  -  Glutamine amidotransferase class-I domain-containing protein
## VIT_08s0040g02100  -  Zinc finger (C2H2 type) JACKDAW
## VIT_06s0004g03930  -  Leucine-rich repeat protein kinase
## VIT_18s0001g11310  -  OBF binding protein 1
## VIT_07s0141g00680  -  WRKY Transcription Factor (VvWRKY17)
## VIT_07s0141g00360  -  Lipase class 3
## VIT_12s0059g01090  -  Early-responsive to dehydration
## VIT_02s0236g00100  -  Unknown
## VIT_19s0027g00010  -  Leucine-rich repeat family protein
## VIT_01s0011g00680  -  Ribosomal protein S19 (RPS19A) 40S
## VIT_11s0016g02800  -  Myo-inositol oxygenase
## VIT_13s0064g01370  -  Polygalacturonase inhibiting protein 1 PGIP1
## VIT_03s0017g00670  -  Zinc finger (C3HC4-type ring finger)
## VIT_04s0008g07230  -  No hit
## VIT_10s0003g00980  -  Unknown protein
## VIT_10s0071g00130  -  R protein disease resistance protein
## VIT_02s0154g00350  -  L-lactate dehydrogenase A
## VIT_04s0023g01640  -  Steroid 22-alpha-hydroxylase (CYP90B1) (DWF4)
## VIT_08s0007g08410  -  4,5-DOPA dioxygenase extradiol
## VIT_10s0003g01830  -  Aquaporin NIP1;2
## VIT_17s0000g03960  -  Fw2.2 ORFX
## VIT_10s0003g05740  -  WRKY Transcription Factor (VvWRKY32)
## VIT_11s0052g01650  -  Pathogenesis-related protein 1 precursor (PRP 1)
## VIT_18s0001g10670  -  EF hand
## VIT_11s0016g05720  -  Protein kinase APK1B
## VIT_04s0069g00780  -  Cellulose synthase CSLC05
```

```
## VIT_09s0002g04560  -  Calmodulin-binding protein
## VIT_11s0118g00540  -  Calcium-binding EF hand
## VIT_02s0025g02540  -  Unknown protein
## VIT_05s0020g04470  -  ADP-ribosylation factor C1
## VIT_14s0068g02240  -  HcrVf1 protein
## VIT_16s0098g00220  -  Membrane located receptor
## VIT_11s0016g01520  -  Phenylalanine ammonia-lyase
## VIT_18s0001g08760  -  No hit
## VIT_07s0031g02340  -  Amino acid permease 2
## VIT_12s0028g03550  -  basic helix-loop-helix (bHLH) family
## VIT_04s0044g01880  -  Auxin Efflux Carrier
## VIT_08s0007g04390  -  Aspartic Protease (VvAP25)
## VIT_03s0038g00430  -  Enhanced disease susceptibility 5 EDS5
## VIT_19s0140g00120  -  Gibberellin 2-beta-dioxygenase 1
## VIT_05s0049g00790  -  No hit
## VIT_11s0037g00320  -  Serine carboxypeptidase S10
## VIT_04s0008g03820  -  Membrane protein
## VIT_15s0046g03420  -  Unknown
## VIT_07s0129g00240  -  C2H2 zinc finger protein AZF1
## VIT_16s0098g00210  -  Receptor serine/threonine kinase
## VIT_15s0045g01370  -  Unknown
## VIT_05s0049g00810  -  No hit
## VIT_11s0016g04490  -  IAA16
## VIT_02s0154g00320  -  Protease inhibitor/seed storage/lipid transfer protein (LTP)
## VIT_18s0001g05890  -  Dehydration Responsive Element-Binding Transcription Factor (VvDREB32)
## VIT_05s0020g01620  -  Transcription termination factor mitochondrial mTERF
## VIT_10s0003g01890  -  RKF1 (receptor-like kinase in flowers 1)
## VIT_18s0001g06790  -  Protein TRANSPARENT TESTA 12 (DDTFR18)
## VIT_10s0071g00190  -  R protein disease resistance protein
## VIT_10s0003g02440  -  Xyloglucan endotransglucosylase/hydrolase 23
## VIT_13s0019g01020  -  Ring-H2 finger protein ATL3A
## VIT_05s0049g01420  -  Peroxisomal membrane protein PMP22
## VIT_07s0005g02220  -  LHCII type I CAB-1
## VIT_07s0031g00160  -  Ubiquitin-conjugating enzyme E2 I
## VIT_11s0016g04930  -  Binding / zinc ion binding
## VIT_13s0019g02530  -  Subtilisin protease C1
## VIT_07s0005g02040  -  Alpha-L-fucosidase 2 precursor
## VIT_16s0050g01240  -  Heavy-metal-associated domain-containing protein
## VIT_02s0154g00170  -  flavin-containing monooxygenase 3
## VIT_00s0181g00180  -  LHCB3 (light-harvesting chlorophyll binding protein 3)
## VIT_13s0064g00410  -  R protein MLA10
## VIT_16s0050g01840  -  Nucleoside triphosphatase
## VIT_14s0066g00600  -  Unknown protein
## VIT_12s0055g01150  -  Brassinosteroid insensitive 1-associated receptor kinase 1
## VIT_01s0011g03070  -  ERF/AP2 Gene Family (VvRAV1)
## VIT_18s0001g08410  -  Homeobox-leucine zipper protein 22
## VIT_00s1463g00010  -  Protease Do-like 5
## VIT_15s0046g02190  -  WRKY Transcription Factor (VvWRKY49)
## VIT_01s0026g00630  -  N-hydroxythioamide S-beta-glucosyltransferase
## VIT_16s0100g00290  -  L-idonate dehydrogenase
## VIT_07s0141g00690  -  ERF/AP2 Gene Family (VvERF065)
## VIT_04s0043g00850  -  Unknown protein
## VIT_06s0004g01180  -  Peroxidase
## VIT_01s0011g02620  -  No hit
## VIT_00s0379g00070  -  CDPK protein kinase
```

```
## VIT_08s0058g00980  -  Cationic peroxidase
```

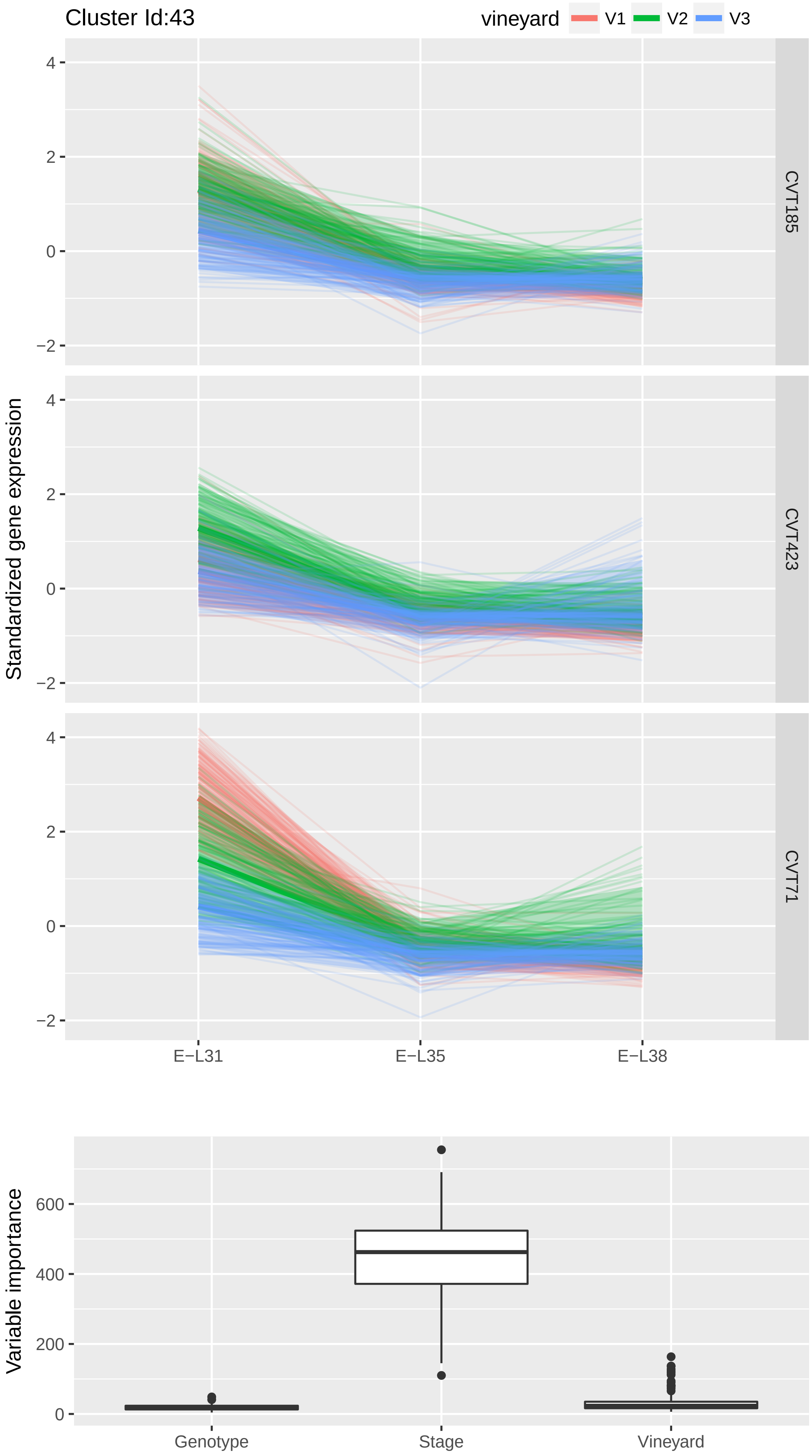

Cluster Id:43
vineyard
V1
V2
V3
Standardized gene expression
CVT185
CVT423
CVT71
E−L31
E−L35
E−L38
4
2
0
−2
Variable importance
600
400
200
0
Genotype
Stage
Vineyard

Cluster no. 44

```
## Number of genes in the cluster: 265
## Homogeneity Index:     0.9
## Variable importance for Stage:    Median =  680.5  - Rank =  1
## Variable importance for Clone:    Median =  10.06  - Rank =  110
## Variable importance for Vineyard: Median =   11.2  - Rank =  112
##
## Gene ID                Gene Annotation
## VIT_13s0139g00140  -  No hit
## VIT_03s0063g00550  -  Fw2.2 ORFX
## VIT_18s0001g13360  -  Nodulin MtN21 family
## VIT_18s0001g10460  -  Defective chloroplasts and leaves protein / DCL protein
## VIT_17s0000g09270  -  MATE efflux family protein
## VIT_00s0207g00270  -  Thioredoxin family
## VIT_18s0089g00970  -  No hit
## VIT_09s0002g04330  -  Ribosomal protein S17 30S
## VIT_12s0142g00400  -  Copper-transporting ATPase PAA1
## VIT_08s0040g00300  -  No hit
## VIT_19s0014g01290  -  Phosphoesterase
## VIT_15s0046g00410  -  Seven in absentia SINA1p
## VIT_14s0066g02040  -  Chaperone BCS1 mitochondrial
## VIT_07s0005g00840  -  Ribosomal protein L19 (RPL19B) 60S
## VIT_08s0007g00140  -  SLT1 (sodium- and lithium-tolerant 1)
## VIT_07s0129g00290  -  formamidase, putative / formamide amidohydrolase, putative
## VIT_04s0069g00260  -  Glutamate receptor 2.8
## VIT_01s0127g00130  -  Cysteine-rich receptor-like protein kinase 3
## VIT_03s0063g00030  -  Unknown protein
## VIT_06s0004g01540  -  Oxidoreductase
## VIT_13s0106g00740  -  Bile acid:sodium symporter
## VIT_04s0008g01190  -  UDP-sulfoquinovose synthase SQD1
## VIT_00s0181g00230  -  LSD ONE like 2
## VIT_05s0020g03490  -  ferredoxin
## VIT_01s0150g00520  -  Ribosomal protein L21 (RPL21E) 60S
## VIT_18s0001g10800  -  Carboxylesterase CXE
## VIT_14s0006g03060  -  Ribosomal protein S3, Chloroplast 30S
## VIT_11s0052g00420  -  Allyl alcohol dehydrogenase
## VIT_06s0004g00890  -  Unknown protein
## VIT_02s0025g02000  -  Ribosomal protein S6
## VIT_13s0064g00600  -  Cyclin-related
## VIT_06s0009g00810  -  Periplasmic beta-glucosidase precursor
## VIT_04s0069g00270  -  Glutamate receptor 2.8
## VIT_12s0028g00510  -  Lipase class 3
## VIT_01s0026g02190  -  ABA-responsive protein (HVA22c)
## VIT_05s0020g00400  -  Galactosyltransferase family protein
## VIT_08s0056g00150  -  Protein kinase APK1B
## VIT_05s0077g01170  -  DNA-directed RNA polymerase II subunit I
## VIT_13s0019g03190  -  Zinc finger (C3HC4-type ring finger)
## VIT_05s0020g01400  -  SRC2/SRC2 (soybean gene regulated BY cold-2)
## VIT_19s0014g05400  -  C-myc binding protein
## VIT_04s0023g02120  -  GTP-binding protein era
## VIT_01s0026g01930  -  Unknown protein
## VIT_00s2642g00010  -  H(+)-ATPase 4 AHA4
## VIT_13s0019g02230  -  PAP/25A associated domain containing protein
## VIT_01s0011g03590  -  Ribosomal protein L15, chloroplast (CL15) 50S
## VIT_17s0000g05840  -  Calmodulin binding IQD31 (IQ-domain 31)
```

```
## VIT_03s0091g00430  -  SWIB complex BAF60b domain-containing protein
## VIT_12s0035g00270  -  ferredoxin, chloroplast (PETF)
## VIT_03s0180g00030  -  Calcium-binding protein
## VIT_15s0021g00880  -  ferredoxin-related
## VIT_11s0016g01230  -  12-oxophytodienoate reductase 3
## VIT_08s0105g00590  -  Chlorophyll synthetase
## VIT_00s0616g00020  -  Ribosomal protein L19
## VIT_18s0122g00680  -  Oxygen-evolving complex PsbP
## VIT_13s0019g00260  -  Photosystem II reaction center PsbP
## VIT_13s0064g00260  -  No hit
## VIT_08s0007g04510  -  RPG related protein 1 RR1
## VIT_11s0037g00140  -  ABC transporter I member 1
## VIT_09s0002g07500  -  Hydrolase, alpha/beta fold
## VIT_05s0136g00290  -  VAMP726 (VESICLE-associated membrane protein)
## VIT_08s0058g00460  -  Unknown protein
## VIT_12s0057g01240  -  Indole-3-glycerol phosphate synthase
## VIT_17s0053g00460  -  Pinoresinol-lariciresinol reductase
## VIT_17s0000g09620  -  Rhodanese domain-containing protein
## VIT_06s0061g00620  -  Short-chain dehydrogenase carbonyl reductase 3
## VIT_05s0049g01110  -  Aldo-keto reductase
## VIT_02s0033g01260  -  No hit
## VIT_06s0061g00460  -  Lactoylglutathione lyase
## VIT_06s0009g00970  -  Thylakoid lumenal P17.1 protein
## VIT_05s0020g01930  -  TUBBY like protein 3 TLP3
## VIT_15s0046g03340  -  Acetyl co-enzyme A carboxylase carboxyltransferase alpha subunit
## VIT_14s0066g01780  -  Heavy-metal-associated domain-containing protein
## VIT_14s0068g01950  -  Ribosomal protein L27, chloroplast (RPL27) 50S
## VIT_18s0001g14360  -  Tubulin beta-1 chain
## VIT_06s0004g04280  -  Cofilin
## VIT_06s0004g04510  -  Heat shock protein 70
## VIT_18s0001g05950  -  Cis-zeatin O-beta-D-glucosyltransferase
## VIT_13s0067g01930  -  Unknown protein
## VIT_03s0110g00350  -  Cinnamyl alcohol dehydrogenase
## VIT_14s0068g00250  -  Exocyst subunit EXO70 E1
## VIT_08s0040g00460  -  Ubiquitin-conjugating enzyme E2 S
## VIT_00s0965g00010  -  Magnesium-chelatase subunit chlD, chloroplast precursor
## VIT_13s0064g01430  -  Pigment defective 322
## VIT_07s0104g00420  -  Endo-1,3;1,4-beta-D-glucanase precursor
## VIT_05s0020g04130  -  Perakine reductase aldo/keto reductase
## VIT_08s0056g00570  -  Serine/threonine protein kinase activity
## VIT_13s0139g00130  -  R protein MLA10
## VIT_11s0016g03360  -  Lipase-related
## VIT_01s0010g01170  -  YGGT
## VIT_15s0046g02130  -  EMB3001 (embryo defective 3001)
## VIT_04s0008g05240  -  Brittle 1 protein
## VIT_06s0004g05110  -  Unknown
## VIT_11s0016g00120  -  Omega-6 fatty acid desaturase, chloroplast (FAD6) (FADC)
## VIT_18s0001g00290  -  OPCL1 (OPC-8:0 COA ligase1)
## VIT_14s0108g01320  -  Plastocyanin domain-containing protein
## VIT_12s0059g02250  -  Ribosomal protein L6
## VIT_11s0052g01540  -  MATE efflux family protein
## VIT_06s0061g01490  -  ABC Transporter (VvPDR20 - VvABCG50)
## VIT_14s0108g00460  -  Ribosomal protein S9 (RPS9C) 40S
## VIT_18s0001g05850  -  Dehydration Responsive Element-Binding Transcription Factor (VvDREB31)
## VIT_10s0003g00890  -  Magnesium-chelatase subunit chlI, chloroplast precursor
```

```
## VIT_01s0026g00730  -  Unknown protein
## VIT_13s0067g00610  -  Yippee
## VIT_02s0025g01050  -  Ubiquitin-conjugating enzyme E2 D/E
## VIT_15s0107g00300  -  Unknown
## VIT_05s0029g01410  -  No hit
## VIT_01s0011g06330  -  Microtubule associated protein (MAP65-6)
## VIT_09s0002g00970  -  Ribosomal protein S29 (RPS29C) 40S
## VIT_18s0001g10070  -  Receptor kinase
## VIT_08s0007g08680  -  Unknown protein
## VIT_00s0732g00010  -  Homeobox-leucine zipper protein 22
## VIT_16s0100g00310  -  Unknown protein
## VIT_18s0001g05040  -  Pollen Ole e 1 allergen and extensin
## VIT_16s0039g00990  -  Glutathione S-transferase 8 GSTU8
## VIT_08s0032g00910  -  Fasciclin domain-containing protein FLA17
## VIT_07s0031g00610  -  Remorin
## VIT_11s0016g05460  -  BRI1-KD interacting protein
## VIT_17s0000g01170  -  Thylakoid lumenal 17.9 kDa protein, chloroplast
## VIT_01s0026g01290  -  Glycosyl transferase family 14 protein
## VIT_02s0025g00230  -  Unknown
## VIT_05s0020g03180  -  Photosystem I reaction center subunit II, chloroplast precursor
## VIT_01s0150g00280  -  Unknown protein
## VIT_17s0119g00110  -  GRAM domain-containing protein / ABA-responsive
## VIT_08s0007g06890  -  Ribosomal protein L21 (RPL21A) 60S
## VIT_06s0061g00010  -  Magnesium-chelatase subunit chlD, chloroplast precursor
## VIT_18s0041g00670  -  Proton-dependent oligopeptide transport (POT) family protein
## VIT_07s0129g01090  -  ADP-ribosylation factor
## VIT_13s0067g02860  -  LRP1 (lateral root primordium 1)
## VIT_04s0008g01770  -  Molecular chaperone DnaJ
## VIT_12s0034g02200  -  RKF3 (receptor-like kinase IN in flowers 3)
## VIT_07s0129g00560  -  No hit
## VIT_05s0094g00820  -  Unknown protein
## VIT_07s0031g00080  -  WRKY Transcription Factor (VvWRKY21)
## VIT_03s0038g03840  -  Cuticular water permeability
## VIT_13s0019g05380  -  Unknown protein
## VIT_01s0026g01570  -  Nitrate transporter 1:2
## VIT_05s0094g01100  -  Ribosomal protein L2
## VIT_11s0016g05520  -  Plastocyanin domain-containing protein
## VIT_12s0034g01180  -  PAP/fibrillin family
## VIT_11s0016g01770  -  MKK2
## VIT_14s0030g02180  -  GDP-mannose 3,5-epimerase 1
## VIT_14s0006g02830  -  Ribosomal protein S7 (RPS7A) 40S
## VIT_18s0001g14230  -  Hexokinase
## VIT_01s0150g00130  -  Unknown protein
## VIT_14s0108g01300  -  Division regulator MinE, Plastid
## VIT_19s0014g03960  -  Zinc finger (B-box type)
## VIT_13s0156g00060  -  Serine/threonine-protein phosphatase PP2A catalytic subunit
## VIT_07s0151g00950  -  Unknown protein
## VIT_06s0009g02020  -  Tubulin beta-4 chain
## VIT_17s0000g07060  -  UDP-glucosyltransferase HRA25
## VIT_19s0085g00290  -  RRNA methylase
## VIT_18s0001g04790  -  Aspartic Protease (VvAP42)
## VIT_18s0001g05960  -  Nuclear transport factor 2 (NTF2)
## VIT_07s0104g00490  -  Unknown
## VIT_11s0052g00410  -  Allyl alcohol dehydrogenase
## VIT_05s0020g01140  -  Ribosomal protein L18
```

```
## VIT_06s0061g00330  -  Unknown protein
## VIT_17s0000g09370  -  Elongation factor EF-Tu RABE1B
## VIT_09s0002g01750  -  Unknown protein
## VIT_15s0045g00310  -  Cyclin dependent kinase A CDKA1
## VIT_18s0001g13230  -  Beta-galactosidase BG1 [Vitis vinifera]
## VIT_18s0001g11370  -  Cytochrome b6f complex subunit (petM)
## VIT_16s0050g00380  -  Tubulin beta-1
## VIT_06s0004g00620  -  Accelerated cell death 1 ACD1
## VIT_03s0180g00110  -  Stress enhanced protein 1 (SEP1)
## VIT_07s0031g00650  -  Defective chloroplasts and leaves protein / DCL protein
## VIT_08s0007g02490  -  2-cys peroxiredoxin BAS1, chloroplast precursor
## VIT_09s0018g00400  -  Unknown protein
## VIT_14s0068g00680  -  Glyceraldehyde-3-phosphate dehydrogenase A, chloroplast precursor
## VIT_14s0171g00300  -  4-coumarate-CoA ligase
## VIT_02s0012g01850  -  Syntaxin 7
## VIT_03s0063g02020  -  Tic Complex Tic62 Subunit
## VIT_19s0014g02470  -  Unknown protein
## VIT_12s0028g03850  -  Thioredoxin M-type 2
## VIT_05s0020g00970  -  Thioesterase family
## VIT_03s0063g01690  -  flavonoid 3-monooxygenase
## VIT_02s0012g00980  -  ferredoxin-6, chloroplast precursor
## VIT_07s0129g00090  -  Ribosomal protein L26 (RPL26A) 60S
## VIT_12s0035g01150  -  RAB GTPase FP8
## VIT_04s0044g00150  -  Wuschel-related homeobox 13
## VIT_06s0004g05180  -  Ribulose bisphosphate carboxylase/oxygenase activase, chloroplast
## VIT_12s0028g00740  -  Unknown protein
## VIT_05s0077g01840  -  Ribosomal protein L19 (RPL19A) 60S
## VIT_19s0015g00690  -  Nitrate-responsive NOI protein
## VIT_02s0025g00130  -  Hydroxyproline-rich glycoprotein
## VIT_04s0044g01020  -  Pectinesterase family
## VIT_16s0115g00080  -  Ribosomal protein L35
## VIT_18s0001g09530  -  Zinc finger (C3HC4-type ring finger)
## VIT_13s0073g00120  -  Ribosomal protein S17 (RPS17A) 40S
## VIT_16s0039g00970  -  Glutathione S-transferase 8 GSTU8
## VIT_03s0091g00870  -  Adenylylsulfate kinase 1 (AKN1)
## VIT_12s0059g01180  -  Bromo-adjacenty (BAH) domain-containing protein
## VIT_16s0098g00730  -  Ribosomal protein L9 (RPL90A/C) 60S
## VIT_03s0063g01110  -  N-acetyl-gamma-glutamyl-phosphate reductase, chloroplast precursor
## VIT_08s0007g00780  -  One helix protein (OHP)
## VIT_01s0011g02720  -  No hit
## VIT_06s0004g06170  -  Thylakoid soluble phosphoprotein
## VIT_00s0347g00110  -  S-locus lectin protein kinase
## VIT_16s0050g01700  -  Receptor serine/threonine kinase PR5K
## VIT_00s0229g00180  -  Phosphatidylserine decarboxylase
## VIT_12s0034g01860  -  Phosphogluconate dehydrogenase
## VIT_18s0001g05530  -  Annexin 1 (ANN1)
## VIT_18s0122g01140  -  Wuschel-related homeobox 13
## VIT_15s0024g01630  -  Glutathione S-transferase 8 GSTU8
## VIT_16s0050g01000  -  Unknown protein
## VIT_09s0002g04570  -  Unknown
## VIT_15s0048g02080  -  Signal recognition particle receptor protein, chloroplast (FTSY)
## VIT_10s0003g02780  -  Unknown protein
## VIT_04s0044g01010  -  Pectinesterase family
## VIT_13s0067g02600  -  No hit
## VIT_04s0044g01310  -  NADH-plastoquinone oxidoreductase subunit o
```

```
## VIT_08s0007g07360  -  No hit
## VIT_11s0016g05900  -  Clathrin assembly protein 10
## VIT_18s0001g07640  -  Eceriferum 2 (CER2)
## VIT_17s0053g00750  -  Unknown protein
## VIT_11s0016g05290  -  Bundle-sheath defective protein 2 family / bsd2 family
## VIT_19s0177g00340  -  Unknown protein
## VIT_16s0050g01220  -  Heavy-metal-associated domain-containing protein
## VIT_12s0035g00910  -  Bile acid sodium symporter
## VIT_13s0019g04300  -  Ribosomal protein L12 (RPL12A) 60S
## VIT_15s0021g00130  -  RPP13 (recognition of peronospora parasitica 13)
## VIT_16s0039g00530  -  BolA
## VIT_09s0002g06560  -  Thiol-disulphide oxidoreductase DCC
## VIT_12s0028g00810  -  Unknown protein
## VIT_03s0063g01560  -  CYP82C1p
## VIT_05s0062g01320  -  Chitinase
## VIT_16s0098g01720  -  Abl interactor 2 (ABIL2)
## VIT_05s0020g01180  -  Proteasome 26S AAA-ATPase subunit (RPT5a)
## VIT_18s0072g01040  -  Invertase, neutral/alkaline
## VIT_07s0151g00930  -  Unknown protein
## VIT_12s0028g03380  -  ADP,ATP carrier protein
## VIT_06s0004g05230  -  Plastid transcriptionally active18
## VIT_09s0002g08470  -  NADP-dependent oxidoreductase
## VIT_18s0001g11770  -  F-box domain containing protein
## VIT_04s0008g05020  -  Ras GTP-binding protein (RAN3)
## VIT_08s0007g02760  -  IAA-amino acid hydrolase 1 (ILR1)
## VIT_12s0028g02540  -  Glucan endo-1,3-beta-glucosidase 1 precursor
## VIT_11s0016g00130  -  Omega-6 fatty acid desaturase, chloroplast (FAD6) (FADC)
## VIT_18s0164g00030  -  ATP synthase delta chain, chloroplast precursor
## VIT_19s0085g00260  -  3-mercaptopyruvate sulfurtransferase.
## VIT_11s0016g05270  -  Bundle-sheath defective protein 2
## VIT_02s0025g02450  -  Unknown protein
## VIT_16s0115g00310  -  Ribosomal protein L35
## VIT_08s0032g00470  -  Periplasmic beta-glucosidase precursor
## VIT_16s0098g01540  -  Thylakoid lumenal 17.4 kDa protein, chloroplast
## VIT_18s0001g06370  -  L-ascorbate peroxidase, chloroplast
## VIT_00s0780g00020  -  Glucan endo-1,3-beta-glucosidase 5 precursor
## VIT_13s0064g01240  -  Ribosomal protein L31 (RPL31C) 60S
## VIT_11s0016g01800  -  Protein kinase MK6
## VIT_01s0010g03890  -  Unknown
## VIT_04s0079g00420  -  Expansin  (VvEXPA4)
## VIT_06s0004g08380  -  Unknown protein
## VIT_08s0007g00420  -  Protein kinase APK1B
## VIT_13s0019g02550  -  Subtilisin protease C1
## VIT_08s0007g04340  -  FK506-binding protein genes family (VvFKBP16-4)
## VIT_08s0105g00310  -  Rhodanese domain-containing protein
## VIT_05s0049g01480  -  Ribosomal protein L28, chloroplast (CL28) 50S
## VIT_19s0090g01320  -  Cell division protein FtsZ
## VIT_05s0051g00860  -  PTAC5 (plastid transcriptionally active5)
## VIT_07s0104g00790  -  Endonuclease
## VIT_05s0049g00420  -  E8 protein
## VIT_12s0142g00420  -  Copper-transporting ATPase PAA1
## VIT_06s0004g04130  -  Protein tyrosine phosphatase protein (PAS2)
## VIT_05s0020g00730  -  Receptor-like protein kinase
```

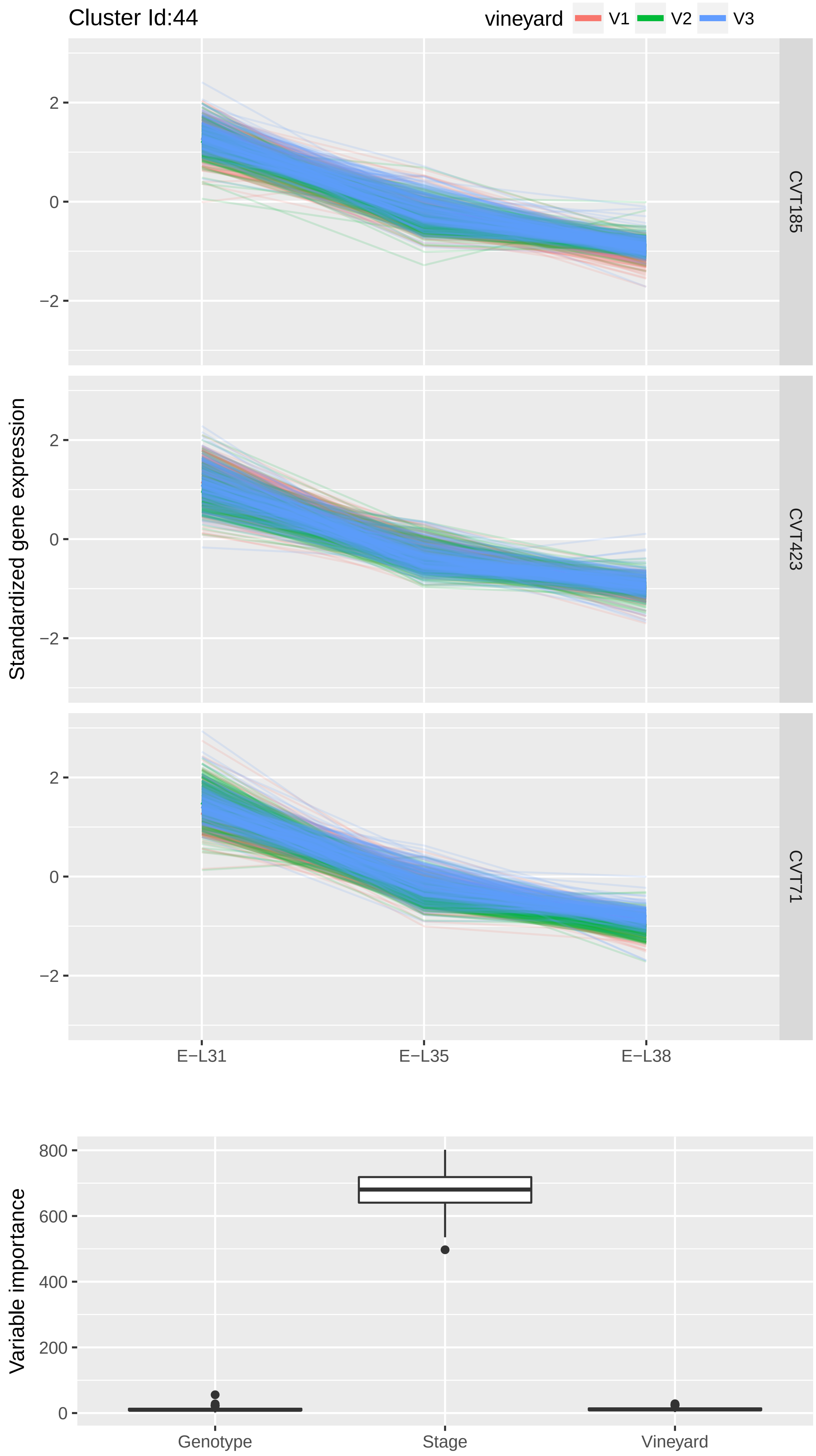

Cluster Id:44
vineyard
V1
V2
V3
Standardized gene expression
CVT185
CVT423
CVT71
2
0
−2
E−L31
E−L35
E−L38
Variable importance
800
600
400
200
0
Genotype
Stage
Vineyard

Cluster no. 45

```
## Number of genes in the cluster: 82
## Homogeneity Index:    0.67
## Variable importance for Stage:    Median =  452.2  - Rank =  46
## Variable importance for Clone:    Median =  12.35  - Rank =  87
## Variable importance for Vineyard: Median =  43.19  - Rank =  43
##
## Gene ID               Gene Annotation
## VIT_08s0007g03830  -  fructose-bisphosphate aldolase cytoplasmic isozyme
## VIT_16s0013g00450  -  No hit
## VIT_19s0085g00360  -  No hit
## VIT_13s0074g00570  -  Gamma-aminobutyric acid transporter
## VIT_01s0010g03600  -  Rubber elongation factor (REF)
## VIT_11s0065g01090  -  DEAD  box  RNA helicase RH10
## VIT_10s0116g00360  -  Pyrophosphate-dependent phosphofructokinase beta subunit
## VIT_11s0052g01220  -  Xyloglucan endotransglycosylase 6
## VIT_09s0002g06970  -  Palmitoyl-monogalactosyldiacylglycerol delta-7 desaturase, chloroplast
## VIT_01s0011g03420  -  Unknown protein
## VIT_19s0015g00020  -  ABC Transporter (VvMRP3 - VvABCC3)
## VIT_01s0011g02280  -  Aminoacyl-tRNA synthetase
## VIT_03s0063g01790  -  Transducin protein
## VIT_16s0022g01770  -  Phosphopyruvate hydratase.
## VIT_11s0065g00370  -  Senescence-associated protein
## VIT_08s0058g00200  -  Transcription factor
## VIT_17s0000g04750  -  UDP-glycosyltransferase 89B2
## VIT_04s0044g00710  -  UTP--glucose-1-phosphate uridylyltransferase
## VIT_06s0061g01210  -  Lysine histidine transporter 1
## VIT_07s0031g00670  -  Heat shock transcription factor 4
## VIT_17s0000g08490  -  Exosome complex component RRP40
## VIT_10s0003g01790  -  BES1/BZR1 homolog protein 4
## VIT_19s0015g00050  -  ABC Transporter (VvMRP5 - VvABCC5)
## VIT_02s0025g02920  -  caffeic acid 3-O-methyltransferase
## VIT_07s0151g00790  -  Transducin family protein / WD-40 repeat
## VIT_16s0013g00480  -  Unknown protein
## VIT_01s0011g02480  -  Phospholipid-transporting ATPase
## VIT_07s0151g00260  -  Quinone-oxidoreductase, Chloroplast
## VIT_07s0031g00490  -  MAP3K protein kinase
## VIT_00s0508g00030  -  Membrane protein
## VIT_14s0068g02150  -  Kelch repeat-containing F-box family protein
## VIT_09s0002g04190  -  Cytochrome c biogenesis protein
## VIT_09s0054g01680  -  No hit
## VIT_18s0001g15270  -  SCL1 (scarecrow-like 1)
## VIT_19s0014g02860  -  Cellulase
## VIT_18s0076g00280  -  Protein tyrosine phosphatase
## VIT_16s0013g00390  -  No hit
## VIT_18s0001g14420  -  Unknown
## VIT_05s0049g01200  -  S-locus lectin protein kinase
## VIT_19s0015g01190  -  Ubiquitin-conjugating enzyme E2 D/E
## VIT_03s0097g00240  -  No hit
## VIT_04s0008g06540  -  Translation initiation factor eIF-5A
## VIT_07s0005g00290  -  Universal stress protein (USP) family protein
## VIT_18s0001g11900  -  GNS1/SUR4 membrane
## VIT_14s0108g00890  -  Beta-1,3-galactosyltransferase
## VIT_03s0091g00470  -  Protein phosphatase 2C
## VIT_11s0016g04920  -  Early nodulin 93
```

```
## VIT_14s0128g00120  -  Ring-H2 finger protein ATL3B
## VIT_13s0084g00620  -  Peptidyl-prolyl isomerase H (cyclophilin H)
## VIT_08s0007g08860  -  Transcription initiation factor TFIIB
## VIT_08s0007g07640  -  NAC domain-containing protein (VvNAC61)
## VIT_13s0019g01810  -  Scarecrow transcription factor 14 (SCL14)
## VIT_03s0091g00480  -  Zinc finger (C3HC4-type ring finger)
## VIT_03s0063g02100  -  Dolichyl-diphosphooligosaccharide-protein glycosyltransferase 48kDa
## VIT_12s0035g00660  -  Zinc finger (C2H2 type) family
## VIT_07s0005g02660  -  ABC Transporter (VvMDR14 - VvABCB14)
## VIT_15s0046g01510  -  BTB/POZ domain-containing protein
## VIT_07s0005g00160  -  Alkylated DNA repair protein
## VIT_08s0007g00200  -  Ankyrin repeat
## VIT_18s0001g03690  -  Unknown protein
## VIT_11s0016g05080  -  Unknown protein
## VIT_02s0025g04560  -  Copper amine oxidase
## VIT_10s0003g02990  -  Signal peptidase I
## VIT_15s0046g01270  -  Unknown protein
## VIT_10s0116g01000  -  Alanine racemase
## VIT_12s0059g00810  -  B-cell receptor-associated protein 31
## VIT_16s0098g01150  -  Auxin-responsive SAUR29
## VIT_08s0007g04470  -  Vacuolar protein sorting 37C
## VIT_00s0531g00020  -  No hit
## VIT_09s0002g00990  -  Triacylglycerol lipase
## VIT_19s0014g02880  -  Glycosyl hydrolase family 5 protein
## VIT_00s0186g00080  -  SUM2 (small ubiquitin-like modifier 2)
## VIT_17s0000g06860  -  SNAP25ous protein SNAP30
## VIT_12s0055g01140  -  Lipid transfer protein
## VIT_05s0049g02240  -  AWPM-19
## VIT_05s0049g01660  -  RNA-binding protein Musashi
## VIT_08s0007g07310  -  No hit
## VIT_14s0068g01840  -  Unknown protein
## VIT_05s0077g00130  -  No hit
## VIT_12s0028g03620  -  Unknown protein
## VIT_00s0181g00250  -  Diacylglycerol kinase
## VIT_14s0006g00100  -  Potassium channel SKOR
```

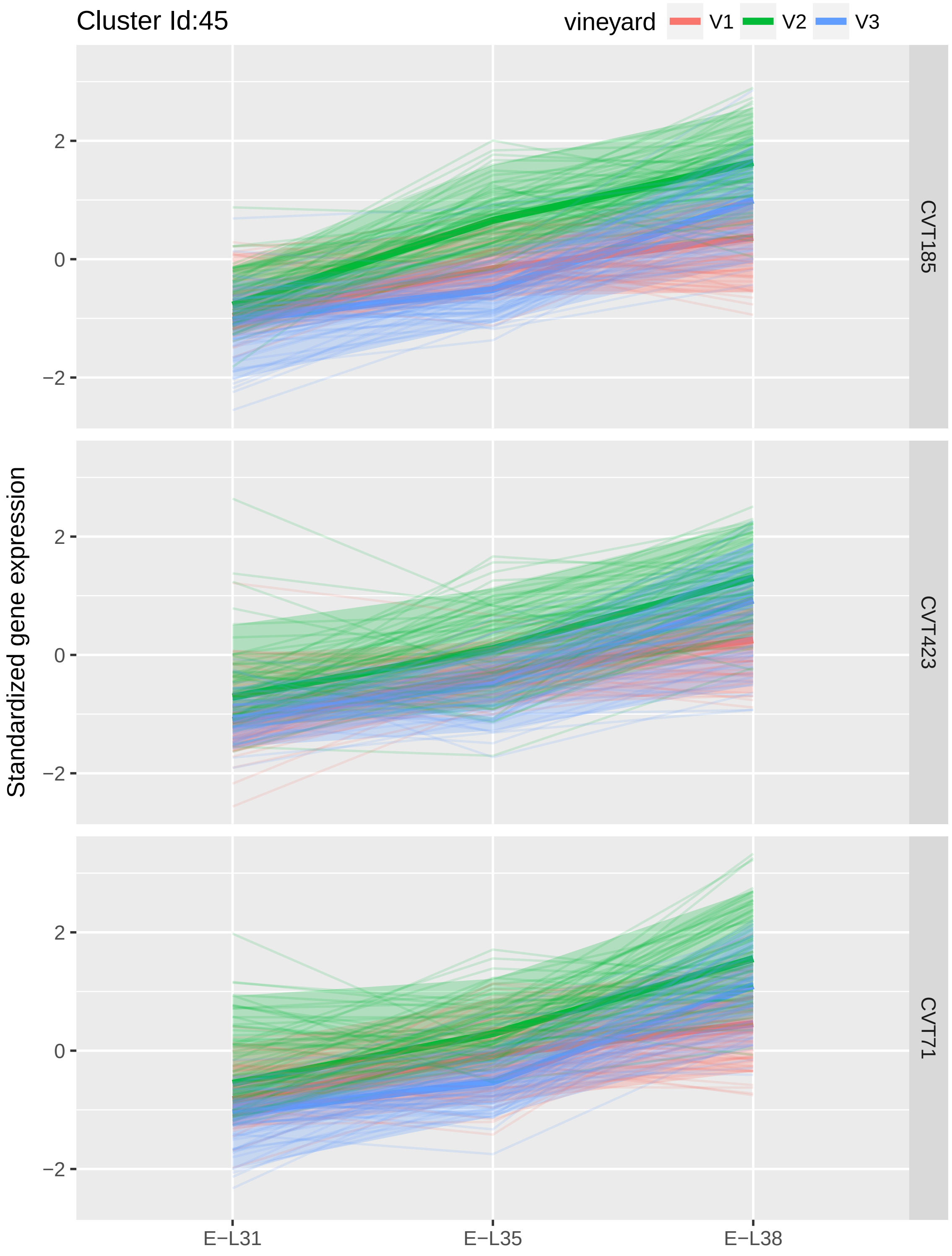

Cluster Id:45
vineyard
V1
V2
V3
Standardized gene expression
2
0
−2
CVT185
CVT423
CVT71
E−L31
E−L35
E−L38

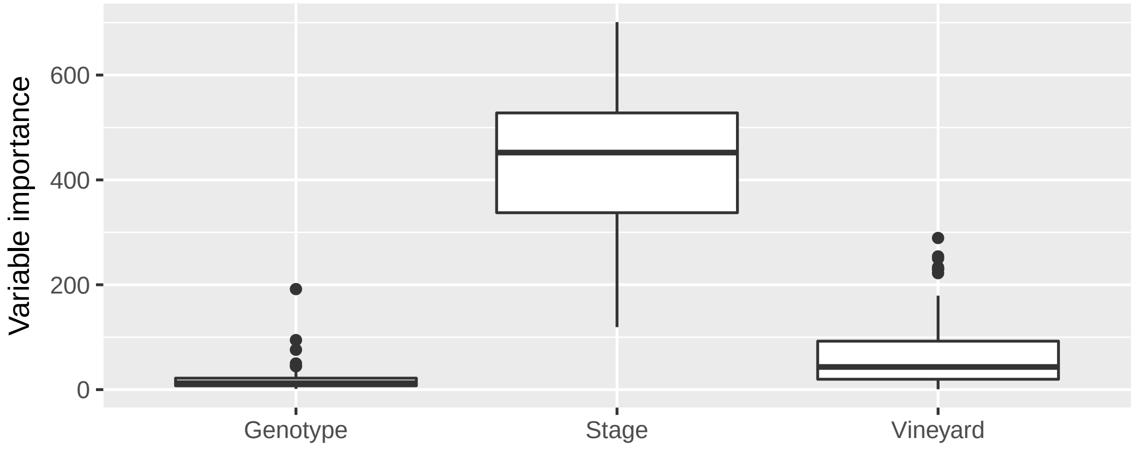

Variable importance
600
400
200
0
Genotype
Stage
Vineyard

Cluster no. 46

```
## Number of genes in the cluster: 132
## Homogeneity Index:    0.79
## Variable importance for Stage:    Median =  535.6  - Rank =  26
## Variable importance for Clone:    Median =  16.45  - Rank =  68
## Variable importance for Vineyard: Median =  17.39  - Rank =  91
##
## Gene ID               Gene Annotation
## VIT_01s0011g05470  -  PGPS/D12
## VIT_06s0004g07870  -  CBL-interacting protein kinase 16
## VIT_09s0070g00570  -  No hit
## VIT_13s0067g00020  -  Tetratricopeptide repeat (TPR)-containing SET domain protein
## VIT_19s0014g05430  -  NDR1 (non race-specific disease resistance)
## VIT_15s0021g00520  -  Transposon protein
## VIT_08s0007g08520  -  Unknown protein
## VIT_07s0197g00140  -  R protein disease resistance protein
## VIT_06s0004g03540  -  Mitogen-activated Protein Kinase (VvMPK6)
## VIT_13s0067g00060  -  Tetratricopeptide repeat (TPR)-containing SET domain protein
## VIT_06s0061g01300  -  Prephenate dehydratase
## VIT_09s0018g01190  -  Anthranilate N-benzoyltransferase
## VIT_01s0026g01550  -  Homeodomain leucine zipper protein HB-1
## VIT_14s0128g00690  -  Germin protein 3
## VIT_06s0009g00440  -  LHW (lonesome highway)
## VIT_15s0046g00320  -  Myc2 bHLH protein
## VIT_08s0007g06060  -  Beta 1-3 glucanase
## VIT_11s0052g00300  -  BRI1-KD interacting protein 132
## VIT_18s0001g02380  -  Kinesin protein (MKRP1)
## VIT_10s0003g01580  -  Unknown protein
## VIT_18s0001g10120  -  SPla/RYanodine receptor (SPRY) domain-containing protein
## VIT_02s0234g00130  -  Ethylene responsive element binding factor 1
## VIT_06s0061g01250  -  Unknown protein
## VIT_00s0411g00020  -  Ankyrin repeat protein
## VIT_09s0002g01520  -  Unknown protein
## VIT_13s0067g03780  -  Leucine-rich repeat protein kinase
## VIT_00s0515g00020  -  R protein MLA10
## VIT_09s0070g00160  -  CBL-interacting protein kinase 6 (VvCIPK02)
## VIT_14s0081g00730  -  Ethylene response factor ERF1
## VIT_01s0127g00210  -  KNAT2 (knotted1-like homeobox gene 6)
## VIT_01s0011g06060  -  PLATZ transcription factor
## VIT_01s0127g00480  -  Unknown protein
## VIT_18s0001g01500  -  No hit
## VIT_05s0102g01070  -  No hit
## VIT_01s0011g03430  -  No hit
## VIT_10s0092g00640  -  Unknown protein
## VIT_14s0068g01390  -  Unknown protein
## VIT_18s0072g00150  -  Unknown protein
## VIT_13s0067g03260  -  Pheophorbidase
## VIT_05s0124g00240  -  basic helix-loop-helix (bHLH) family
## VIT_14s0030g01560  -  Cysteine synthase
## VIT_12s0142g00330  -  Copper-transporting ATPase PAA1
## VIT_11s0118g00470  -  TIP growth defective 1
## VIT_05s0020g04670  -  No hit
## VIT_19s0014g04720  -  Unknown protein
## VIT_18s0001g06230  -  No hit
## VIT_11s0118g00730  -  No hit
```

```
## VIT_05s0062g00640  -  UDP-glucose:flavonoid 7-O-glucosyltransferase
## VIT_00s0181g00260  -  High-level expression of sugar-inducible like 1
## VIT_01s0011g05680  -  Unknown protein
## VIT_18s0001g13130  -  BEL1-like homeodomain 10
## VIT_14s0006g03210  -  Unknown
## VIT_16s0022g00860  -  Invertase/pectin methylesterase inhibitor
## VIT_09s0002g00700  -  Dormancy/auxin associated protein
## VIT_02s0025g00750  -  Pinoresinol forming dirigent protein
## VIT_16s0039g00570  -  10-deacetylbaccatin III 10-O-acetyltransferase
## VIT_19s0014g00090  -  Glucan endo-1,3-beta-glucosidase 4 precursor
## VIT_05s0020g04690  -  IAA7
## VIT_15s0046g00310  -  ERF/AP2 Gene Family (VvERF015),Dehydration Responsive Element-Binding
## VIT_19s0014g04670  -  basic helix-loop-helix (bHLH) family
## VIT_07s0129g00460  -  Prolyl 4-hydroxylase
## VIT_18s0001g11170  -  Myb domain protein 73
## VIT_18s0089g00270  -  R protein L6
## VIT_00s2032g00010  -  No hit
## VIT_13s0019g04440  -  Zinc finger (C3HC4-type ring finger)
## VIT_00s0207g00180  -  Unknown protein
## VIT_00s0256g00110  -  Ankyrin repeat protein
## VIT_19s0177g00030  -  Gibberellin 2-beta-dioxygenase 7
## VIT_03s0063g01580  -  CYP82C2
## VIT_01s0127g00870  -  Polygalacturonase JP630
## VIT_13s0019g00010  -  Sulfate transporter 4.2
## VIT_19s0014g04310  -  S-locus protein kinase
## VIT_09s0002g00570  -  Lipase GDSL 1
## VIT_15s0046g01970  -  Anthocyanidine rhamnosyl-transferase
## VIT_11s0016g03590  -  Transducin protein
## VIT_08s0040g00960  -  CXE carboxylesterase
## VIT_05s0020g04560  -  Armadillo/beta-catenin repeat protein / U-box domain-containing protei
## VIT_18s0001g01300  -  Wall-associated receptor kinase 5
## VIT_07s0031g02550  -  ABC Transporter (VvWBC17 - VvABCG17)
## VIT_18s0001g11240  -  MAPKKK5 (Mitogen-activated protein kinase kinase kinase 5)
## VIT_19s0014g03370  -  Methyltransferase type 11
## VIT_07s0129g00660  -  Indole-3-acetic acid-amido synthetase GH3.2
## VIT_00s0404g00100  -  Syntaxin of plants 124
## VIT_14s0083g00850  -  Lipase GDSL 7
## VIT_13s0019g00750  -  Annexin ANN4
## VIT_01s0011g02780  -  Unknown protein
## VIT_10s0003g04270  -  BCL2 binding anthogene
## VIT_03s0063g00180  -  U-box domain-containing protein
## VIT_02s0025g00360  -  1-aminocyclopropane-1-carboxylate synthase
## VIT_02s0012g01320  -  MYC jasmonic acid 3
## VIT_13s0019g01710  -  Scarecrow transcription factor 14 (SCL14)
## VIT_14s0006g00300  -  Nudix hydrolase 18
## VIT_03s0038g04220  -  Dihydroflavonol-4-reductase
## VIT_17s0000g05000  -  putative MADS-box sepallata 2 (VviSEP2)
## VIT_01s0010g03560  -  CYP78A5
## VIT_06s0004g04210  -  No hit
## VIT_05s0020g04680  -  Auxin-induced protein 22D
## VIT_05s0094g01120  -  NADP-dependent oxidoreductase
## VIT_11s0016g00730  -  LHCA6 (Photosystem I light harvesting complex gene 6)
## VIT_06s0004g02730  -  Unknown protein
## VIT_19s0140g00140  -  GA 20-oxidase 2
## VIT_03s0038g03040  -  Protein kinase
```

```
## VIT_16s0022g01430  -  Phosphate-induced protein 1
## VIT_14s0068g00260  -  Serine carboxypeptidase S10
## VIT_00s0802g00010  -  No hit
## VIT_03s0063g01530  -  CYP82C4
## VIT_18s0001g01310  -  Wall-associated receptor kinase 5
## VIT_13s0019g04500  -  Transketolase
## VIT_14s0066g00680  -  Ramosa 2
## VIT_18s0001g13420  -  SHI-related sequence 5
## VIT_04s0044g01670  -  Ankyrin
## VIT_17s0000g05600  -  (-)-isopiperitenol dehydrogenase
## VIT_13s0019g01080  -  Protein kinase APK1B
## VIT_11s0052g00100  -  basic helix-loop-helix (bHLH) family
## VIT_00s0375g00030  -  Alpha-galactosidase
## VIT_02s0025g01240  -  UDP-glucuronosyl/UDP-glucosyltransferase
## VIT_05s0020g02880  -  ADP-glucose pyrophosphorylase
## VIT_15s0048g02650  -  Unknown protein
## VIT_10s0116g00190  -  Homeobox protein shoot MERISTEMLESS (STM)
## VIT_02s0012g00310  -  Lon protease
## VIT_05s0094g01410  -  Inositol 1,3,4-trisphosphate 5/6-kinase
## VIT_05s0062g01260  -  No hit
## VIT_09s0002g06820  -  Equilibrative nucleoside transporter ENT8
## VIT_12s0059g01190  -  Homeobox protein shoot MERISTEMLESS (STM)
## VIT_00s0256g00050  -  Unknown
## VIT_06s0004g04640  -  Metallothionein 2b
## VIT_06s0009g00310  -  Unknown protein
## VIT_05s0062g01430  -  Glycosyl hydrolase family 17 protein
## VIT_17s0000g01260  -  Squamosa promoter-binding protein (VvSBP15)
## VIT_11s0016g05230  -  Allyl alcohol dehydrogenase
## VIT_00s1328g00020  -  Phytosulfokines PSK4
## VIT_07s0005g02070  -  No hit
```

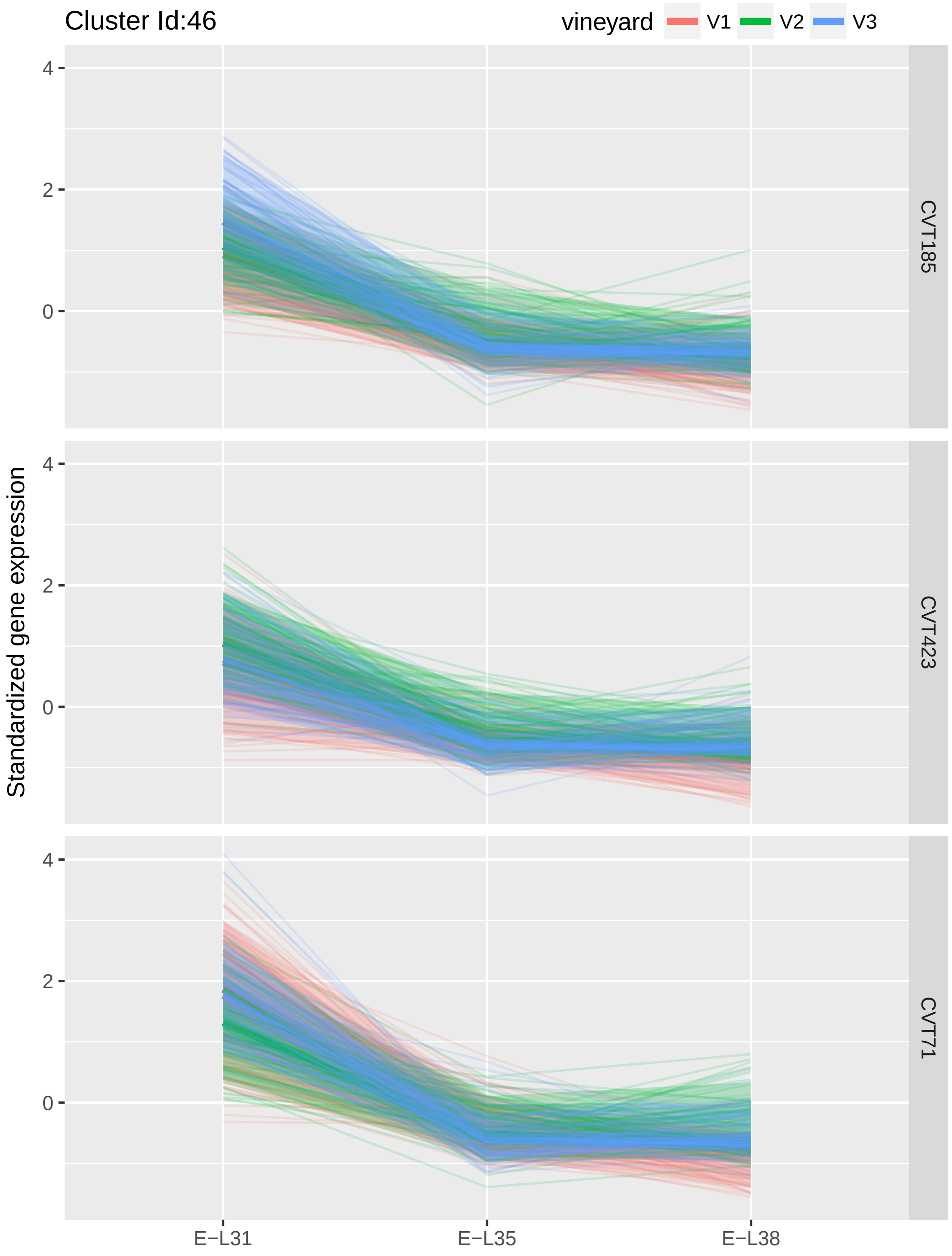

Cluster Id:46
vineyard
V1
V2
V3
Standardized gene expression
CVT185
CVT423
CVT71
4
2
0
E−L31
E−L35
E−L38

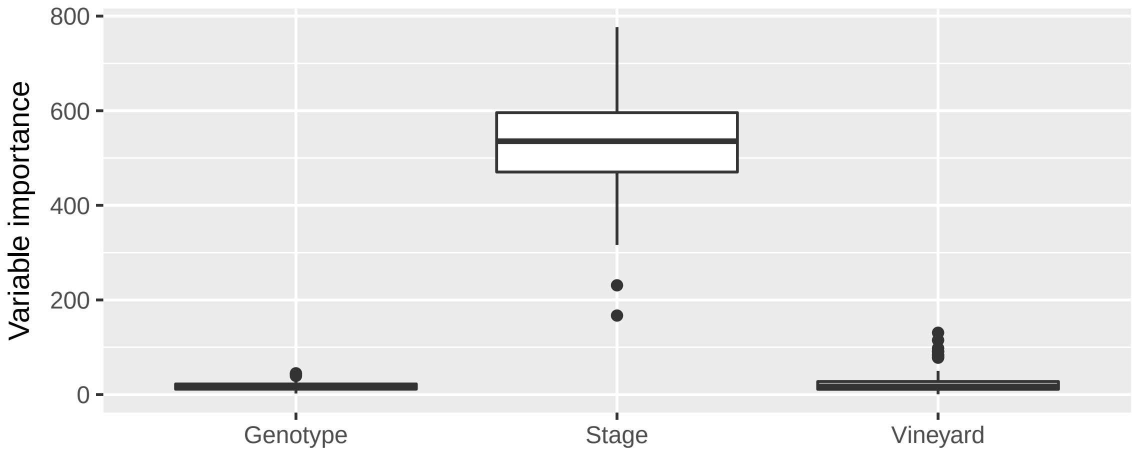

Variable importance
800
600
400
200
0
Genotype
Stage
Vineyard

Cluster no. 47

```
## Number of genes in the cluster: 229
## Homogeneity Index:    0.83
## Variable importance for Stage:    Median =  588.1  - Rank =  17
## Variable importance for Clone:    Median =  10.28  - Rank =  105
## Variable importance for Vineyard: Median =  20.36  - Rank =  84
##
## Gene ID              Gene Annotation
## VIT_12s0055g00750  -  Pentatricopeptide (PPR) repeat-containing protein
## VIT_19s0015g01910  -  F-box family protein
## VIT_13s0019g01050  -  R protein MLA10
## VIT_06s0004g07050  -  Ubiquitin family
## VIT_01s0026g02250  -  Haloacid dehalogenase hydrolase
## VIT_09s0054g01870  -  Transmembrane receptor
## VIT_17s0000g01870  -  Indeterminate(ID)-domain 16
## VIT_13s0019g01770  -  Pentatricopeptide repeat
## VIT_07s0031g01850  -  BRI1 protein
## VIT_01s0011g05840  -  No hit
## VIT_00s0335g00090  -  R protein MLA10
## VIT_16s0098g00160  -  Receptor serine/threonine kinase
## VIT_03s0091g00410  -  Unknown protein
## VIT_00s0238g00130  -  RPS4 (resistant to p. syringae 4)
## VIT_07s0031g01410  -  Somatic embryogenesis receptor kinase 1 SERK1
## VIT_16s0050g00240  -  UDP-glycosyltransferase 88A4
## VIT_09s0002g02680  -  Early flowering 3
## VIT_18s0001g06820  -  MATE efflux family protein ripening responsive
## VIT_13s0019g02990  -  Cis-zeatin O-beta-D-glucosyltransferase
## VIT_09s0018g01950  -  Cellulose synthase CSLC05
## VIT_09s0002g01910  -  Myosin-like protein XIK
## VIT_12s0057g01550  -  Unknown protein
## VIT_00s0324g00110  -  Haloacid dehalogenase hydrolase
## VIT_01s0010g01480  -  AGD4 (ARF-GAP domain 4)
## VIT_03s0038g03980  -  Unknown protein
## VIT_09s0018g00310  -  ABC Transporter (VvMRP7 - VvABCC7)
## VIT_06s0080g00930  -  Unknown protein
## VIT_00s1349g00010  -  Cellulose synthase CSLE1
## VIT_00s0184g00010  -  AP2 domain-containing transcription factor family
## VIT_05s0020g01440  -  Unknown protein
## VIT_08s0007g01490  -  Unknown protein
## VIT_04s0023g02780  -  Short internodes
## VIT_04s0069g00770  -  UDP-glucuronic acid/UDP-N-acetylgalactosamine transporter
## VIT_06s0009g01110  -  Cation exchanger (CAX7)
## VIT_01s0010g03310  -  Unknown protein
## VIT_12s0059g00960  -  Cellulose synthase CSLB04
## VIT_01s0026g02200  -  TCP family transcription factor TCP15
## VIT_08s0007g00610  -  Unknown
## VIT_13s0067g01950  -  Chromatin remodeling 42
## VIT_14s0083g00400  -  Leaf senescence protein
## VIT_10s0116g00280  -  UDP-3-O-[3-hydroxymyristoyl] glucosamine N-acyltransferase
## VIT_08s0056g01000  -  MATE efflux family protein
## VIT_06s0004g02050  -  Origin recognition complex subunit 1 protein
## VIT_09s0002g02230  -  Harpin-induced 1
## VIT_14s0081g00010  -  IAA16
## VIT_09s0018g00520  -  Plastocyanin domain-containing protein
## VIT_02s0154g00250  -  Oxysterol binding protein
```

```
## VIT_15s0021g01920  -  Mitochondrial transcription termination factor mTERF
## VIT_00s0222g00100  -  CC-NBS-LRR class
## VIT_01s0010g03710  -  Squamosa promoter-binding protein (VvSBP2)
## VIT_14s0068g00940  -  Receptor protein kinase
## VIT_08s0007g00210  -  Unknown protein
## VIT_18s0117g00040  -  No hit
## VIT_03s0038g02440  -  Heavy-metal-associated domain-containing protein
## VIT_08s0040g03370  -  Pentatricopeptide (PPR) repeat-containing
## VIT_19s0027g00500  -  GTP-binding protein YchF
## VIT_17s0000g07890  -  Rac-like GTP-binding protein ARAC10 (GTPase protein ROP10)
## VIT_17s0000g06880  -  Heparanase protein 2 precursor
## VIT_00s0211g00040  -  Nodulation receptor kinase
## VIT_01s0010g02090  -  Indeterminate(ID)-domain 5
## VIT_19s0014g05160  -  RPS2 (resistant to p. syringae 2)
## VIT_05s0020g03000  -  2-Hydroxyisoflavanone dehydratase
## VIT_06s0009g02670  -  Unknown
## VIT_13s0067g03610  -  No hit
## VIT_12s0059g00200  -  Lanthionine synthetase C
## VIT_18s0001g03670  -  Zinc finger (C2H2 type) family
## VIT_02s0012g01450  -  Basic helix-loop-helix BHLH071
## VIT_16s0050g00760  -  Short-chain dehydrogenase/reductase (SDR)
## VIT_17s0000g04490  -  Extracellular Ca2+ sensing receptor
## VIT_01s0010g00750  -  Unknown protein
## VIT_01s0010g03780  -  Histidine kinase (AHK3)
## VIT_07s0005g06180  -  RPM1 (resistance to p. syringae pv maculicola 1)
## VIT_18s0001g14100  -  ABA-responsive protein (HVA22)HVA22H
## VIT_18s0001g05160  -  Glycosyl hydrolase family 3 protein
## VIT_13s0019g01610  -  XH/XS domain-containing protein
## VIT_12s0028g01790  -  FAD-linked oxidoreductase 1
## VIT_10s0003g02730  -  D1 protease precursor
## VIT_01s0026g00330  -  NHL repeat-containing protein
## VIT_13s0019g03450  -  Unknown protein
## VIT_05s0020g04860  -  Zinc knuckle
## VIT_05s0077g01750  -  SAG101 (senescence-associated gene 101)
## VIT_18s0001g12220  -  Indeterminate(ID)-domain 2
## VIT_09s0002g03880  -  Cell division protein FtsH
## VIT_08s0040g03010  -  Pigment defective 149
## VIT_02s0154g00080  -  Multi-copper oxidase (SKU5)
## VIT_08s0007g05180  -  Unknown
## VIT_14s0068g00030  -  feronia receptor-like kinase
## VIT_07s0141g00470  -  RNA polymerase Rpb7 N-terminal domain-containing
## VIT_09s0002g04250  -  Protein disulfide-isomerase A1
## VIT_14s0128g00320  -  Amidase
## VIT_02s0234g00020  -  Embryonic flower 2 (EMF2)
## VIT_00s0211g00030  -  Symbiosis receptor kinase
## VIT_09s0002g01450  -  Unknown protein
## VIT_04s0023g00790  -  Protein translocase Tic20
## VIT_15s0048g02240  -  Acid phosphatase PAP14
## VIT_13s0074g00130  -  R protein MLA10
## VIT_04s0044g00730  -  S-receptor kinase
## VIT_13s0084g00110  -  ABC transporter I member 8
## VIT_14s0036g01350  -  R protein disease resistance protein
## VIT_08s0007g07290  -  Translation initiation factor eIF-4E
## VIT_09s0018g01670  -  Aspartic Protease (VvAP26)
## VIT_08s0007g03910  -  CTR1 serine/threonine protein kinase
```

```
## VIT_17s0000g03990  -  Aspartyl protease
## VIT_18s0001g12970  -  Ethylene-responsive protein
## VIT_12s0028g02160  -  Ribulose bisphosphate carboxylase, large chain
## VIT_12s0057g01020  -  fasciclin-like arabinogalactan protein FLA2
## VIT_12s0059g01660  -  3-oxoacyl-[acyl-carrier-protein] synthase II
## VIT_15s0046g01010  -  DEAD box RNA helicase (RH26)
## VIT_06s0009g01670  -  forkhead-associated domain-containing protein
## VIT_08s0007g01610  -  KOW domain-containing transcription factor family protein
## VIT_19s0014g00950  -  DNA polymerase I
## VIT_07s0031g02290  -  Phosphate carrier protein
## VIT_02s0025g04010  -  flavonoid 3-monooxygenase
## VIT_12s0035g01680  -  CC-NBS-LRR class
## VIT_13s0019g00470  -  No hit
## VIT_08s0007g00760  -  Thaumatin
## VIT_08s0040g01800  -  BTB/POZ; NPH3
## VIT_00s0215g00110  -  Sphingosine kinase
## VIT_15s0045g00500  -  DNA mismatch repair protein Mlh1
## VIT_02s0025g04200  -  Unknown
## VIT_18s0001g09910  -  L-asparaginase
## VIT_00s0397g00010  -  HcrVf1 protein
## VIT_05s0029g00260  -  DNA repair protein RAD50
## VIT_01s0011g05480  -  Dynein light subunit lc6, flagellar outer arm
## VIT_19s0014g02610  -  RNA-binding S4 domain-containing protein
## VIT_05s0020g01600  -  Unknown protein
## VIT_05s0102g00750  -  Zinc finger (B-box type)
## VIT_05s0020g04870  -  Membrane related protein CP5
## VIT_06s0009g02660  -  Unknown protein
## VIT_02s0025g01200  -  Leaf senescence protein
## VIT_05s0020g03760  -  RNA helicase SDE3 (SDE3)
## VIT_19s0014g05180  -  RPS2 (resistant to p. syringae 2)
## VIT_00s0583g00030  -  Sphingolipid delta 4 desaturase DES-1
## VIT_16s0050g02420  -  Myosin heavy chain
## VIT_13s0074g00730  -  PID (PINOID)
## VIT_08s0056g01120  -  MATE efflux family protein
## VIT_19s0014g03350  -  Peptidyl-prolyl cis-trans isomerase TLP38, chloroplast
## VIT_12s0059g00540  -  EDM2
## VIT_17s0000g08330  -  Ataxia-telangiectasia mutated protein (Atm)
## VIT_07s0141g00500  -  V-type H+-transporting ATPase 21kDa proteolipid subunit
## VIT_01s0026g02260  -  Unknown protein
## VIT_06s0004g01380  -  S-receptor kinase
## VIT_00s0283g00070  -  Short-chain dehydrogenase/reductase (SDR)
## VIT_08s0007g05570  -  Embryo-abundant protein
## VIT_08s0007g02090  -  No hit
## VIT_09s0002g05070  -  RPS5 (resistant to p. syringae 5)
## VIT_18s0117g00080  -  R protein L6
## VIT_04s0008g05430  -  RDR6 (RNA-dependent RNA polymerase 6)
## VIT_07s0005g05010  -  CLIP-associating protein (CLASP)
## VIT_09s0096g00010  -  Disease resistance protein (NBS-LRR class)
## VIT_02s0234g00050  -  Embryonic flower 2
## VIT_00s0256g00030  -  Ankyrin repeat protein
## VIT_08s0007g01300  -  Glyoxal oxidase
## VIT_18s0001g14300  -  Cytomatrix protein
## VIT_18s0041g01350  -  Receptor-like protein kinase HAIKU2
## VIT_01s0011g00060  -  No hit
## VIT_08s0007g06250  -  Strubbelig receptor family 2
```

```
## VIT_10s0003g00420  -  Auxin response factor 3
## VIT_08s0007g01760  -  Steroid 5 alpha reductase DET2
## VIT_08s0007g02020  -  Proton-dependent oligopeptide transport (POT) family protein
## VIT_11s0016g03380  -  Exostosin-like
## VIT_15s0048g00790  -  TCH2 (touch 2)
## VIT_10s0116g01700  -  Autophagy 8c (APG8c)
## VIT_02s0025g03240  -  Protein kinase
## VIT_01s0150g00100  -  Ubiquitin thioesterase protein OTUB1
## VIT_17s0000g05350  -  5-formyltetrahydrofolate cycloligase
## VIT_06s0004g01870  -  No transmitting tract
## VIT_03s0063g02110  -  RUB1-conjugating enzyme
## VIT_05s0020g01650  -  Zinc finger (CCCH-type) family protein
## VIT_06s0004g01530  -  DNA topoisomerase 6 subunit B (TOP6B)
## VIT_00s0471g00010  -  U-box domain-containing protein
## VIT_02s0488g00010  -  H(+)-ATPase 4 AHA4
## VIT_12s0028g03750  -  Zinc finger (C3HC4-type ring finger)
## VIT_11s0016g02400  -  K+ efflux antiporter (KEA4)
## VIT_13s0047g00250  -  EIN3
## VIT_07s0031g02480  -  Beta-galactosidase
## VIT_04s0008g05540  -  Clathrin assembly protein 2
## VIT_09s0018g02110  -  Unknown protein
## VIT_01s0011g02740  -  Phosphoenolpyruvate carboxylase
## VIT_18s0001g03710  -  R protein L6
## VIT_12s0059g00530  -  EDM2
## VIT_04s0008g03760  -  Unknown protein
## VIT_16s0013g00240  -  Unknown protein
## VIT_03s0180g00280  -  Indole-3-acetate beta-glucosyltransferase
## VIT_04s0008g02750  -  Basic Leucine Zipper Transcription Factor (VvbZIP09)
## VIT_14s0068g00330  -  PTF1 (plastid transcription factor 1) TCP13
## VIT_05s0077g01720  -  SAG101 (senescence-associated gene 101)
## VIT_17s0000g05040  -  No hit
## VIT_14s0068g01620  -  Pollen proteins Ole e I
## VIT_07s0031g00210  -  PAB8 (poly(A) binding protein 8)
## VIT_13s0067g03600  -  RSZ33 (Arginine/serine-rich Zinc knuckle-containing protein 33)
## VIT_08s0056g00600  -  No hit
## VIT_02s0025g04020  -  S-N-methylcoclaurine 3'-hydroxylase
## VIT_16s0039g00310  -  Unknown protein
## VIT_14s0030g01370  -  Methyltransferase type 12
## VIT_12s0035g00470  -  No hit
## VIT_14s0171g00170  -  Unknown protein
## VIT_06s0004g02650  -  No hit
## VIT_08s0040g00890  -  Single-stranded nucleic acid binding R3H
## VIT_16s0098g01170  -  Homeobox-leucine zipper protein HB-12 (VvATHB-10)
## VIT_06s0061g00790  -  Pheophorbide a oxygenase
## VIT_10s0042g01250  -  NPR1-like protein 3
## VIT_00s0397g00050  -  Disease resistance family protein
## VIT_14s0108g01420  -  DEFENSE NO death 1
## VIT_07s0129g00870  -  Protein kinase family
## VIT_04s0008g03210  -  Lachrymatory factor synthase
## VIT_07s0141g00670  -  No hit
## VIT_04s0008g05220  -  Cellulose synthase CESA1
## VIT_08s0058g00940  -  2OG-Fe(II) oxygenase
## VIT_18s0001g06720  -  Rieske [2Fe-2S] domain
## VIT_11s0103g00400  -  XH/XS domain-containing protein
## VIT_08s0007g05270  -  No hit
```

```
## VIT_05s0051g00010  -  Beta-amylase 1
## VIT_09s0002g03340  -  Unknown
## VIT_02s0154g00240  -  Oxysterol binding protein
## VIT_00s0400g00020  -  HcrVf1 protein
## VIT_01s0010g02360  -  Thimet oligopeptidase
## VIT_18s0001g08010  -  Octicosapeptide/Phox/Bem1p (PB1) domain-containing protein
## VIT_18s0001g07360  -  ER6 protein universal stress protein (USP) family
## VIT_03s0038g01350  -  Glutamine amidotransferase class-I
## VIT_07s0104g01460  -  Zinc finger (CCCH-type) family protein
## VIT_13s0067g01690  -  Ankyrin
## VIT_02s0025g05140  -  ATP-dependent Clp protease proteolytic subunit (ClpP2)
## VIT_08s0007g04630  -  HEAT repeat-containing protein
## VIT_15s0021g00940  -  Senescence-associated protein
## VIT_18s0001g08970  -  No hit
## VIT_17s0000g07840  -  Unknown protein
## VIT_09s0002g02630  -  IAA-amino acid hydrolase 3
## VIT_15s0024g00980  -  Amidase
```

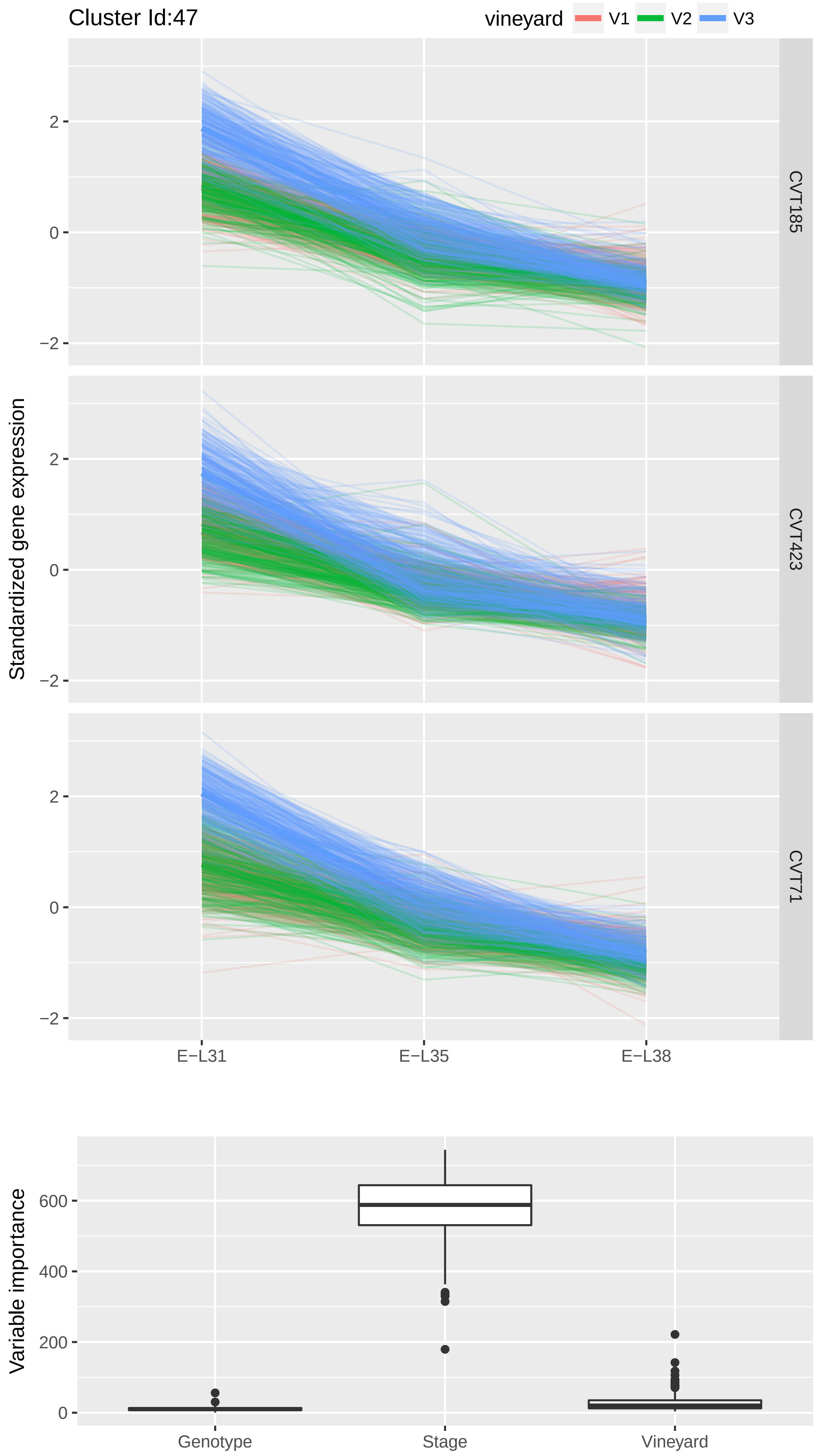
Cluster Id:47
vineyard
V1
V2
V3
CVT185
CVT423
CVT71
Standardized gene expression
2
0
−2
E−L31
E−L35
E−L38
Variable importance
600
400
200
0
Genotype
Stage
Vineyard

Cluster no. 48

```
## Number of genes in the cluster: 271
## Homogeneity Index:    0.87
## Variable importance for Stage:    Median =  663.2  - Rank =  3
## Variable importance for Clone:    Median =  10.14  - Rank =  108
## Variable importance for Vineyard: Median =  12.02  - Rank =  109
##
## Gene ID               Gene Annotation
## VIT_10s0003g00260  -  DnaJ homolog, subfamily B, member 4
## VIT_08s0056g00120  -  Ethanolamine-phosphate cytidylyltransferase
## VIT_18s0001g05130  -  Unknown protein
## VIT_18s0001g13070  -  NAD-dependent epimerase/dehydratase
## VIT_01s0011g06620  -  Peptide transporter PTR2-B
## VIT_09s0002g01480  -  Telomere repeat binding factor like TRFL6
## VIT_14s0128g00480  -  Translation initiation factor eIF-3 subunit 1
## VIT_07s0005g03490  -  VHS domain-containing protein
## VIT_16s0098g01370  -  RNA recognition motif (RRM)-containing protein
## VIT_11s0016g01110  -  Unknown protein
## VIT_16s0039g01520  -  Molecular chaperone DnaJ
## VIT_09s0002g04290  -  Hydroxyphenylpyruvate reductase (HPPR)
## VIT_13s0019g05130  -  Serine carboxypeptidase III
## VIT_00s0347g00030  -  Zinc finger (B-box type)
## VIT_17s0000g05110  -  CYP78A4
## VIT_08s0056g00620  -  V-type H+-transporting ATPase subunit E
## VIT_18s0041g00360  -  Unknown protein
## VIT_02s0025g02340  -  UBX domain-containing protein
## VIT_18s0001g15290  -  Ribosome maturation protein SDO1
## VIT_02s0025g02010  -  Proteasome 26S regulatory subunit (RPN12)
## VIT_01s0150g00640  -  Unknown protein
## VIT_03s0038g00790  -  V-type H+-transporting ATPase 16kDa proteolipid subunit
## VIT_07s0104g00970  -  Histone H2AXb HTA3
## VIT_03s0038g02580  -  Unknown protein
## VIT_10s0116g00030  -  Pentatricopeptide (PPR) repeat membrane-associated salt-inducible prot
## VIT_17s0119g00070  -  Unknown protein
## VIT_13s0156g00180  -  Citrate synthase 4
## VIT_15s0048g00960  -  Transducin protein
## VIT_18s0001g02070  -  Unknown protein
## VIT_12s0057g01200  -  Acetyl-CoA acetyltransferase, cytosolic 1
## VIT_04s0008g06880  -  ER membrane DUF1077
## VIT_10s0003g02200  -  Protein kinase PKN/PRK1
## VIT_12s0034g02170  -  SAG18 (Senescence associated gene 18)
## VIT_05s0094g00490  -  Selenoprotein
## VIT_12s0035g02110  -  Glutathione S-transferase Z1 GSTZ1
## VIT_10s0116g01480  -  2-oxoglutarate dehydrogenase E2 subunit
## VIT_04s0023g01380  -  Scarecrow-like
## VIT_07s0104g00440  -  Endo-1,3;1,4-beta-D-glucanase precursor
## VIT_04s0023g03120  -  Histone H3
## VIT_17s0000g01200  -  CBS domain containing protein
## VIT_09s0018g01420  -  Serine incorporator 3
## VIT_06s0004g03990  -  Nudix hydrolase 9
## VIT_13s0074g00370  -  Zinc finger (C3HC4-type ring finger)
## VIT_14s0068g02030  -  Import receptor subunit TOM20-3, Mitochondrial
## VIT_04s0044g01070  -  Leaf senescence related protein-like
## VIT_11s0016g00780  -  Unknown protein
## VIT_11s0016g01920  -  EIF-4A3
```

```
## VIT_05s0102g01130  -  Unknown protein
## VIT_15s0048g02210  -  Unknown protein
## VIT_14s0068g02060  -  Unknown protein
## VIT_08s0058g01330  -  Unknown
## VIT_02s0012g01240  -  PHD finger transcription factor
## VIT_11s0118g00230  -  ATSYTE/NTMC2T2.1/NTMC2TYPE2.1/SYTE
## VIT_07s0191g00250  -  Exo-1,3-beta-glucanase
## VIT_13s0019g03220  -  Proline transporter 1 (ProT1)
## VIT_05s0051g00740  -  Zinc finger (C3HC4-type ring finger)
## VIT_01s0127g00270  -  Signal recognition particle receptor beta subunit
## VIT_05s0051g00750  -  Unknown
## VIT_02s0012g02660  -  Proteasome 26S regulatory subunit (RPN9)
## VIT_18s0072g00640  -  No hit
## VIT_17s0000g09280  -  Ubiquitin-associated (UBA)
## VIT_05s0077g00690  -  Galactokinase (GAL1)
## VIT_01s0146g00310  -  Pyruvate dehydrogenase E1 component subunit beta
## VIT_03s0017g00640  -  Oxoglutarate/malate translocator DIT2.1
## VIT_01s0011g00700  -  myb family transcription factor / ELM2 domain-containing
## VIT_17s0000g07870  -  Ribosomal protein L29
## VIT_15s0048g01720  -  fidgetin-like 1
## VIT_09s0018g01940  -  Pyruvate dehydrogenase E1 component alpha subunit
## VIT_18s0001g11940  -  Metalloendopeptidase
## VIT_16s0050g02450  -  Proline-rich cell wall protein
## VIT_18s0122g01410  -  Zinc carboxypeptidase A
## VIT_00s0268g00020  -  Phytase
## VIT_10s0116g01730  -  Soluble starch synthase 3, chloroplast precursor
## VIT_15s0046g01550  -  C2 domain-containing protein
## VIT_11s0016g03390  -  CRS2-associated factor 1
## VIT_00s0265g00080  -  Ribosomal protein L21
## VIT_10s0042g00800  -  Proteasome 26S regulatory subunit (RPN2)
## VIT_09s0002g05590  -  ABC Transporter (VvPDR16 - VvABCG46)
## VIT_18s0001g13990  -  No hit
## VIT_10s0003g00530  -  28 kDa heat- and acid-stable phosphoprotein
## VIT_08s0056g00710  -  Protein kinase C inhibitor
## VIT_11s0016g04730  -  Zinc finger (C3HC4-type ring finger) family protein
## VIT_05s0020g02820  -  Ubiquitin activating enzyme E1c (ECR1)
## VIT_12s0059g01400  -  Tyrosine specific protein phosphatase
## VIT_04s0008g06490  -  Unknown protein
## VIT_06s0009g02230  -  Cysteine protease inhibitor
## VIT_01s0010g03300  -  Protein processing in endoplasmic reticulum
## VIT_06s0009g00260  -  BSU1-like protein 3 BSL3
## VIT_15s0024g01750  -  Protein kinase
## VIT_01s0010g01120  -  Longevity-assurance (LAG1)
## VIT_17s0000g03730  -  NADH dehydrogenase (ubiquinone) 1 beta subcomplex 10
## VIT_07s0151g00690  -  Proteasome 20S beta subunit D (PBD1) (PRGB)
## VIT_07s0104g01080  -  NPL4
## VIT_08s0007g04900  -  Unknown protein
## VIT_06s0009g03520  -  Signal peptide peptidase
## VIT_00s0203g00100  -  AarF domain-containing kinase ABC1
## VIT_09s0002g00680  -  Auxin-induced protein
## VIT_11s0016g03890  -  Short-chain dehydrogenase/reductase (SDR)
## VIT_08s0040g02090  -  DnaJ homolog, subfamily C, member 3
## VIT_18s0001g09670  -  WPP2
## VIT_00s0203g00200  -  Universal stress protein (USP) family protein
## VIT_14s0083g00320  -  Cinnamoyl-CoA reductase
```

```
## VIT_14s0066g00790  -  RNA binding motif protein 26
## VIT_07s0005g02430  -  AarF domain containing kinase
## VIT_04s0008g03740  -  Unknown protein
## VIT_07s0129g00020  -  Glycosyl hydrolase family 43 protein
## VIT_05s0094g01420  -  Inosine-uridine preferring nucleoside hydrolase
## VIT_18s0001g02260  -  Proteasome 20S beta subunit C1 (PBC1) (PRCT)
## VIT_12s0059g01600  -  Lipid-A-disaccharide synthase
## VIT_03s0063g02550  -  NADH dehydrogenase (ubiquinone) Fe-S protein 8
## VIT_05s0094g01540  -  Unknown protein
## VIT_11s0016g05020  -  IMP dehydrogenase/GMP reductase
## VIT_10s0042g00070  -  Proteasome 20S alpha subunit C (PAC1) (PRC9)
## VIT_08s0056g01240  -  No hit
## VIT_01s0011g06630  -  Translation initiation factor eIF-3 subunit 2
## VIT_04s0043g00280  -  No hit
## VIT_02s0025g03320  -  CYP86A2
## VIT_00s0360g00040  -  No hit
## VIT_07s0141g00820  -  No hit
## VIT_12s0035g01850  -  MATE antiporter/ drug transporter
## VIT_18s0001g03270  -  Zinc finger (C3HC4-type ring finger)
## VIT_14s0068g01650  -  GYF domain-containing protein
## VIT_05s0049g00280  -  1-aminocyclopropane-1-carboxylate oxidase homolog 1
## VIT_08s0032g00090  -  replication factor C subunit 3/5
## VIT_05s0029g01180  -  Unknown
## VIT_11s0052g00720  -  Adagio protein 1
## VIT_01s0113g00540  -  Vesicle transport v-SNARE 13
## VIT_06s0009g03650  -  Permease nonimprinted in Prader-Willi/Angelman
## VIT_11s0016g05390  -  Ribosomal protein L36
## VIT_19s0027g00220  -  Unknown protein
## VIT_11s0016g00340  -  DNA repair protein RAD51
## VIT_06s0009g01890  -  Oligoribonuclease
## VIT_17s0000g05320  -  IFA binding protein
## VIT_13s0064g01450  -  Electron carrier/ protein disulfide oxidoreductase
## VIT_05s0029g01380  -  UV radiation resistance associated
## VIT_00s0187g00170  -  Partial optokinetic response b
## VIT_05s0077g01510  -  Unknown protein
## VIT_13s0019g00460  -  Unknown protein
## VIT_12s0134g00410  -  frataxin protein
## VIT_18s0001g03990  -  Stearoyl-ACP desaturase
## VIT_14s0108g00040  -  Structure-specific recognition protein 1 SSRP1
## VIT_17s0000g09030  -  Disease resistance protein (NBS-LRR class)
## VIT_19s0090g00340  -  Heat shock protein 91
## VIT_01s0011g00530  -  Ureide permease 2 (AtUPS2)
## VIT_06s0004g07060  -  Ubiquitin activating enzyme E1
## VIT_04s0043g00710  -  Hypoxia up-regulated 1 HSP70
## VIT_09s0002g04110  -  Peroxisome assembly protein 2 (Peroxin-2) (PEX2)
## VIT_17s0000g07540  -  paired amphipathic helix protein Sin3a
## VIT_14s0030g00690  -  ABI3-interacting protein 2
## VIT_19s0014g01410  -  Alpha-1,2-mannosyltransferase ALG9
## VIT_18s0001g02650  -  V-type H+-transporting ATPase 16kDa proteolipid subunit
## VIT_11s0118g00830  -  D111/G-patch domain-containing protein
## VIT_03s0038g02640  -  Translation initiation factor IF-3
## VIT_18s0001g04340  -  Glycine hydroxymethyltransferase
## VIT_00s0360g00050  -  Cyclin-related
## VIT_09s0002g04590  -  Unknown protein
## VIT_00s1488g00020  -  Glycogen (starch) synthase
```

```
## VIT_12s0178g00050  -  Unknown protein
## VIT_10s0003g04530  -  Cation:chloride symporter
## VIT_00s0184g00020  -  Import inner membrane translocase subunit Tim17/Tim22/Tim23
## VIT_07s0005g03790  -  Succinyl-CoA ligase beta-chain
## VIT_19s0014g01650  -  Global transcription factor group E 4
## VIT_11s0016g03780  -  Continuous vascular ring (COV1)
## VIT_14s0171g00260  -  Phosducin
## VIT_01s0011g01960  -  Proteasome 20S alpha subunit B
## VIT_04s0044g01720  -  Unknown protein
## VIT_11s0016g01990  -  Regulatory protein NPR1 (Nonexpresser of PR genes 1)
## VIT_04s0008g05800  -  HEAT repeat-containing protein
## VIT_05s0077g00910  -  DNA-binding enhancer protein
## VIT_01s0011g04210  -  Amino acid permease
## VIT_13s0064g01050  -  ADP,ATP carrier protein 1
## VIT_15s0046g02040  -  CaaX processing zinc-metallo endoprotease
## VIT_00s0214g00120  -  F-box family protein
## VIT_08s0007g00180  -  OBP3 (OBF-binding protein 3)
## VIT_06s0004g00870  -  Unknown protein
## VIT_07s0141g00830  -  2-keto-3-deoxygluconokinase pfkB-type carbohydrate kinase
## VIT_03s0038g00280  -  Cysteine proteinase
## VIT_04s0008g04990  -  Potassium channel (VvK1.2)
## VIT_08s0040g02520  -  Inositol-1,4,5-trisphosphate 5-phosphatase CVP2, type I
## VIT_11s0016g05180  -  Nitrate transporter
## VIT_08s0007g03360  -  EMB1345 (embryo defective 1345)
## VIT_13s0047g01070  -  Calcium-binding EF-hand
## VIT_17s0000g08610  -  Phox (PX) domain-containing protein
## VIT_01s0011g02980  -  ILP1 (Increased level of polyploidy1-1d)
## VIT_08s0040g01480  -  Cell division cycle protein 48
## VIT_11s0078g00460  -  PUMILIO 5 (APUM5)
## VIT_05s0102g01190  -  Calreticulin-3 precursor
## VIT_02s0025g00140  -  Ring-H2 zinc finger protein RHA1b
## VIT_04s0008g02290  -  ABI3-interacting protein 2
## VIT_17s0000g05230  -  Unknown protein
## VIT_11s0118g00500  -  Protein OS-9
## VIT_09s0002g05570  -  ABC Transporter (VvPDR15 - VvABCG45)
## VIT_06s0004g06150  -  C2 domain-containing protein
## VIT_08s0040g00510  -  ZIFL2 (zinc induced facilitator-like 2)
## VIT_17s0000g08810  -  Unknown protein
## VIT_01s0026g02730  -  Anaphase-promoting complex component APC2
## VIT_07s0104g00690  -  GPI transamidase component Gpi16 subunit
## VIT_01s0011g02970  -  ILP1 (Increased level of polyploidy1-1d)
## VIT_13s0047g00170  -  Ring finger protein
## VIT_16s0098g01660  -  KH domain-containing protein
## VIT_09s0002g05790  -  Serine-threonine kinase receptor-associated protein STRAP
## VIT_18s0001g04010  -  Salt tolerant protein
## VIT_12s0057g00920  -  Transcriptional adapter ADA2a
## VIT_00s0207g00090  -  Ring zinc finger ariadne protein ARI5
## VIT_03s0038g03670  -  MAP kinase activating protein
## VIT_19s0090g01370  -  No hit
## VIT_12s0121g00240  -  MuDR transposase; Zinc finger, SWIM-type; Malate synthase-like
## VIT_12s0034g02550  -  Magnesium transporter CorA
## VIT_18s0001g00890  -  Pentatricopeptide (PPR) repeat-containing
## VIT_18s0122g01110  -  Phosphatidylinositolglycan class O (PIG-O)
## VIT_12s0035g00810  -  Transmembrane protein 18
## VIT_11s0103g00270  -  NADH dehydrogenase (ubiquinone) 1 alpha subcomplex 1
```

```
## VIT_14s0030g00020  -  Unknown
## VIT_09s0002g01560  -  Ataxin-2 N-terminal region
## VIT_18s0001g09990  -  Cysteine protease CP1
## VIT_18s0001g00050  -  Dolichyl-phosphate beta-D-mannosyltransferase
## VIT_04s0043g00760  -  UDP-N-acetylglucosamine--peptide N-acetylglucosaminyltransferase SEC
## VIT_11s0016g05730  -  CUE domain containing protein
## VIT_08s0040g02530  -  No hit
## VIT_08s0007g07900  -  Unknown protein
## VIT_08s0058g00880  -  Class E vacuolar protein-sorting machinery protein HSE1
## VIT_19s0093g00580  -  Ethylene receptor 1 (ETR1)
## VIT_15s0021g01330  -  Nucleoside diphosphate kinase 1 (NDK1)
## VIT_17s0000g07610  -  B12D protein
## VIT_13s0106g00620  -  Nitrogen fixation NifU
## VIT_18s0001g09180  -  CUE domain containing protein
## VIT_18s0001g08560  -  Unknown protein
## VIT_17s0000g02000  -  IRE (incomplete root hair elongation)
## VIT_07s0104g00230  -  IWS1 C-terminus
## VIT_13s0067g01510  -  Outer membrane OMP85
## VIT_14s0108g00110  -  Serine carboxypeptidase S10
## VIT_07s0205g00080  -  Unknown protein
## VIT_12s0028g00440  -  Protein phosphatase 2C
## VIT_00s2466g00010  -  Exo-1,3-beta-glucanase
## VIT_12s0059g02520  -  fringe
## VIT_06s0080g00800  -  Vesicle-associated membrane protein 714
## VIT_15s0048g01980  -  COP9 signalosome complex subunit 1
## VIT_04s0023g03190  -  Peptidyl-prolyl cis-trans isomerase NIMA-interacting 1
## VIT_17s0000g04410  -  TGH (tough); RNA binding
## VIT_10s0003g00210  -  Unknown
## VIT_01s0010g02030  -  Gamma-thionin precursor
## VIT_07s0104g00280  -  No hit
## VIT_07s0031g01810  -  Unknown protein
## VIT_02s0087g00680  -  Unknown protein
## VIT_13s0019g03270  -  Phosphate translocator protein2, Plastidic
## VIT_04s0079g00760  -  Ras-related protein Rab-5A
## VIT_04s0023g03020  -  Serine carboxypeptidase S28
## VIT_08s0040g01280  -  Ubiquitin-associated (UBA)
## VIT_08s0007g06660  -  E3 ubiquitin-protein ligase RNF5
## VIT_03s0038g03120  -  Isocitrate dehydrogenase subunit 1
## VIT_19s0090g01240  -  Hydroxyproline-rich glycoprotein VTA1
## VIT_06s0004g03150  -  Unknown protein
## VIT_01s0011g00820  -  Remorin
## VIT_01s0150g00160  -  Ubiquinol-cytochrome C reductase iron-sulfur subunit, mitochondrial
## VIT_09s0002g02940  -  Myo-inositol oxygenase 1
## VIT_10s0003g02720  -  Beta-1,3 glucanase
## VIT_14s0068g01850  -  No hit
## VIT_19s0014g05200  -  Unknown protein
## VIT_13s0067g02140  -  Heavy-metal-associated domain-containing protein
## VIT_03s0038g03610  -  Unknown protein
## VIT_05s0029g00470  -  Unknown protein
## VIT_02s0025g01560  -  UDP-glucose 4-epimerase GEPI48
## VIT_11s0016g00770  -  Actin beta/gamma 1
## VIT_00s0227g00130  -  A/G-specific adenine glycosylase
## VIT_14s0006g02000  -  No hit
## VIT_13s0019g00510  -  2-oxoglutarate dehydrogenase E1 component
## VIT_01s0011g05370  -  Phosphoglucomutase, cytoplasmic
```

```
## VIT_14s0030g00620  -  Unknown protein
## VIT_02s0025g00640  -  BTI2 (VIRB2- interacting  protein 2)
## VIT_13s0019g04330  -  Unknown protein
## VIT_09s0002g04870  -  Glycine-rich protein-like
```

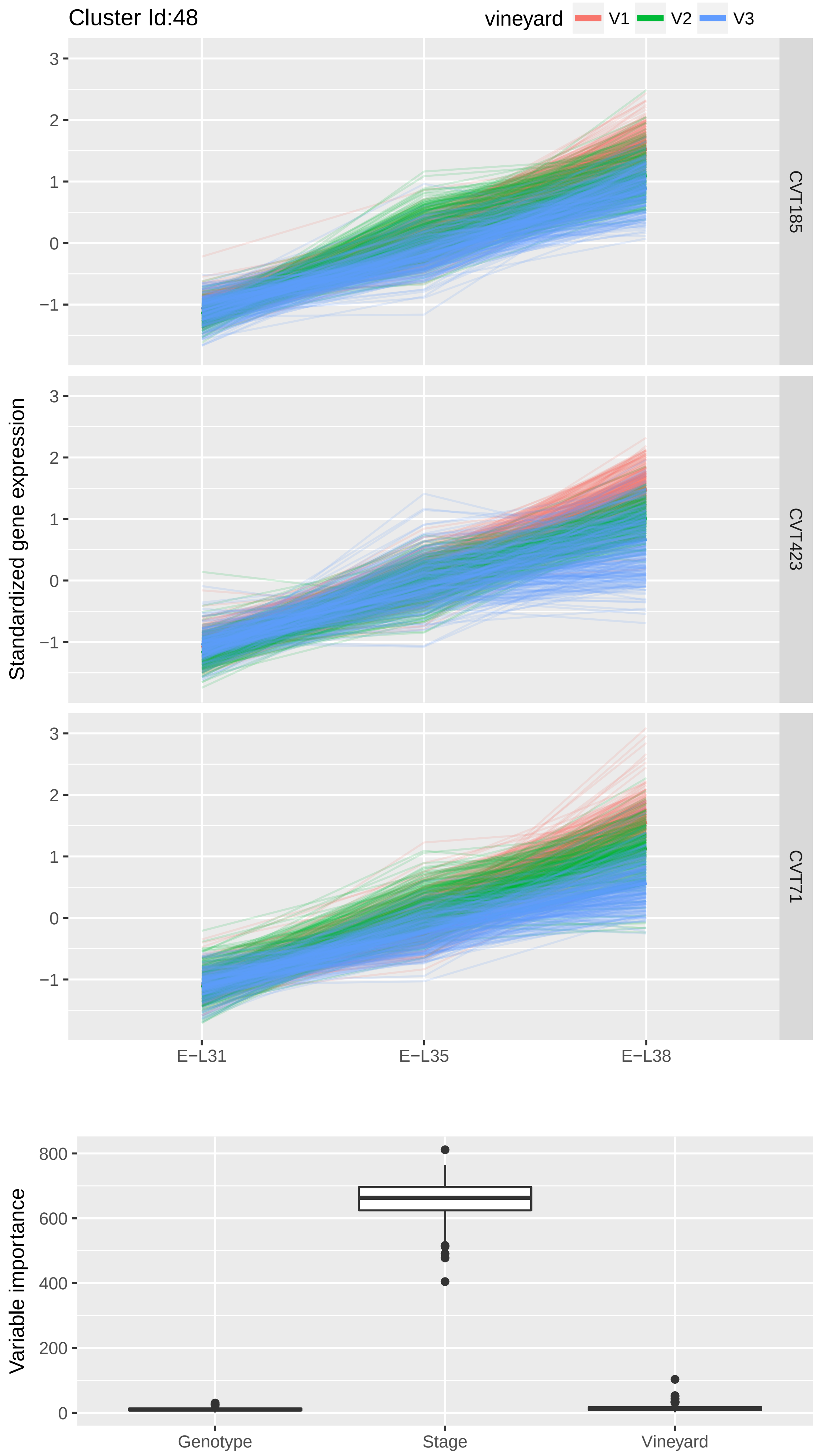

Cluster Id:48
vineyard
V1
V2
V3
Standardized gene expression
CVT185
CVT423
CVT71
3
2
1
0
−1
E−L31
E−L35
E−L38
Variable importance
800
600
400
200
0
Genotype
Stage
Vineyard

Cluster no. 49

```
## Number of genes in the cluster: 107
## Homogeneity Index:    0.79
## Variable importance for Stage:    Median =  384.3  - Rank =  60
## Variable importance for Clone:    Median =  14.63  - Rank =  75
## Variable importance for Vineyard: Median =  91.04  - Rank =  20
##
## Gene ID                Gene Annotation
## VIT_06s0004g02750  -  Auxin response factor 16
## VIT_07s0005g02130  -  TolB protein-related
## VIT_07s0031g03220  -  Patellin-1
## VIT_00s0184g00050  -  B-cell receptor-associated protein 31
## VIT_02s0025g03420  -  Unknown protein
## VIT_10s0092g00510  -  CYP71D10p
## VIT_14s0006g02760  -  Unknown protein
## VIT_07s0129g00220  -  Actin binding formin homology 2
## VIT_08s0007g08530  -  Unknown protein
## VIT_14s0066g02260  -  Unknown protein
## VIT_15s0046g01770  -  D-erythro-sphingosine kinase
## VIT_13s0158g00200  -  R protein MLA10
## VIT_17s0000g05200  -  NLI interacting factor (NIF) family protein
## VIT_10s0003g01610  -  Ras protein Rab11B
## VIT_15s0024g01530  -  No hit
## VIT_04s0023g00350  -  Unknown
## VIT_17s0000g03210  -  Zinc finger (C3HC4-type ring finger)
## VIT_15s0046g01380  -  Unknown
## VIT_12s0059g00250  -  Polyol transporter 5
## VIT_14s0108g01590  -  UDP-glucose 6-dehydrogenase
## VIT_12s0028g02650  -  RAB GTPase RABA1F
## VIT_08s0007g05520  -  Pentatricopeptide (PPR) repeat
## VIT_06s0004g04890  -  Rhomboid ATRBL1
## VIT_18s0001g12490  -  O-methyltransferase
## VIT_13s0158g00190  -  R protein MLA10
## VIT_14s0108g00380  -  Protein kinase APK1A
## VIT_04s0023g03340  -  Adaptor-related protein complex 2, sigma 1 sub
## VIT_17s0000g02850  -  No hit
## VIT_03s0091g01100  -  Unknown protein
## VIT_03s0038g00350  -  Kinesin protein (MKRP2)
## VIT_07s0005g06220  -  RPM1 (resistance to p. syringae pv maculicola 1)
## VIT_03s0091g00240  -  Haloacid dehalogenase hydrolase
## VIT_14s0030g00160  -  Bisphosphoglycerate mutase
## VIT_18s0001g02700  -  Chlorophyll a oxygenase (CAO)
## VIT_04s0023g03480  -  Armadillo/beta-catenin repeat
## VIT_10s0003g01620  -  Ras protein Rab11B
## VIT_14s0108g01600  -  UDP-glucose 6-dehydrogenase
## VIT_07s0031g01960  -  Zinc finger (DHHC type)
## VIT_11s0016g03820  -  Signal peptidase I
## VIT_14s0066g01850  -  Peroxidase
## VIT_17s0000g05640  -  Nitrate transporter 1:2
## VIT_13s0067g01150  -  Unknown protein
## VIT_14s0108g01610  -  UDP-glucose 6-dehydrogenase
## VIT_04s0044g01320  -  ABCNAP13
## VIT_04s0008g03180  -  Unknown protein
## VIT_00s0532g00040  -  No hit
## VIT_12s0035g02180  -  Endo-1,4-beta-glucanase
```

```
## VIT_07s0031g01700  -  RAB GTPase LIP1
## VIT_17s0000g07310  -  3-phosphoserine phosphatase, chloroplast precursor
## VIT_15s0048g02700  -  RNA recognition motif (RRM)-containing protein
## VIT_01s0026g01140  -  ZCW32 (BIGPETAL, BIGPETALUB)
## VIT_03s0063g02120  -  Minor allergen
## VIT_11s0016g01200  -  Mob1/phocein
## VIT_01s0010g00510  -  Unknown protein
## VIT_18s0122g00120  -  Cellulose synthase CESA2
## VIT_01s0026g00960  -  Unknown protein
## VIT_18s0001g10310  -  Protein kinase family
## VIT_00s0455g00040  -  Glycosyl transferase family 8 protein
## VIT_09s0002g01730  -  Phospholipid-transporting ATPase
## VIT_00s0802g00020  -  Unknown protein
## VIT_18s0122g01040  -  Ubiquitin-protein ligase
## VIT_03s0038g03960  -  Calcium-dependent protein kinase (VvCPK2)
## VIT_00s0475g00010  -  Glycosyl transferase family 8 protein
## VIT_14s0108g01350  -  Unknown protein
## VIT_00s0199g00310  -  No hit
## VIT_14s0066g00780  -  Disease resistance protein (NBS-LRR class)
## VIT_02s0012g00710  -  RAB GTPase RAB6
## VIT_18s0001g00110  -  Unknown protein
## VIT_18s0001g08250  -  Tubulin alpha-3 chain
## VIT_01s0026g02550  -  MYR1 (MYB-related protein 1)
## VIT_06s0004g07520  -  F-box family protein
## VIT_03s0038g02200  -  Peptidyl-prolyl cis-trans isomerase A (cyclophilin A)
## VIT_08s0007g02470  -  Aspartic Protease (VvAP24)
## VIT_12s0028g01670  -  Trehalose-phosphatase
## VIT_06s0061g00810  -  Cyclin-dependent kinase regulatory subunit CKS2
## VIT_14s0108g00400  -  Glutaredoxin family
## VIT_01s0010g01380  -  Ankyrin repeat
## VIT_19s0090g01490  -  Glycerophosphoryl diester phosphodiesterase
## VIT_01s0011g05560  -  TIFY gene family (VvJAZI)
## VIT_04s0008g03250  -  Homeobox gene 8
## VIT_10s0116g01850  -  Unknown protein
## VIT_14s0060g00460  -  Receptor-like protein kinase
## VIT_18s0001g10040  -  LRX1 (leucine-rich repeat/extensin 1)
## VIT_18s0122g00140  -  Lipid-A-disaccharide synthase (LpxB)
## VIT_11s0052g01700  -  CBL-interacting protein kinase 21 (CIPK21)
## VIT_11s0206g00090  -  Calmodulin-binding protein
## VIT_19s0014g00140  -  Transcription factor related
## VIT_13s0175g00090  -  No hit
## VIT_11s0052g00800  -  Protein kinase family
## VIT_15s0048g01300  -  Glycosyl transferase family 8 protein
## VIT_04s0023g03860  -  Rac-like GTP-binding protein ARAC1 precursor (GTPase protein ROP3)
## VIT_13s0067g02900  -  Basic Leucine Zipper Transcription Factor (VvbZIP33)
## VIT_04s0023g02000  -  Integral membrane protein-like
## VIT_18s0041g01570  -  R protein L6
## VIT_14s0006g02800  -  Unknown protein
## VIT_07s0205g00140  -  Kinesin motor protein
## VIT_17s0000g00210  -  Copine BON1 (BONZAI 1)
## VIT_15s0046g01350  -  Tubulin gamma-2 chain
## VIT_18s0086g00230  -  Unknown protein
## VIT_11s0206g00070  -  Calmodulin-binding protein
## VIT_01s0011g00110  -  putative MADS-box sepallata 4 (VviSEP4)
## VIT_00s0301g00020  -  Unknown protein
```

```
## VIT_09s0002g04810  -  Coatomer protein complex, subunit beta 2 (beta prime)
## VIT_11s0206g00080  -  Calmodulin-binding protein
## VIT_10s0071g01150  -  Katanin p80 (WD repeat containing) subunit B 1
## VIT_07s0141g00600  -  Unknown protein
## VIT_13s0074g00450  -  DEMETER protein (DME)
```

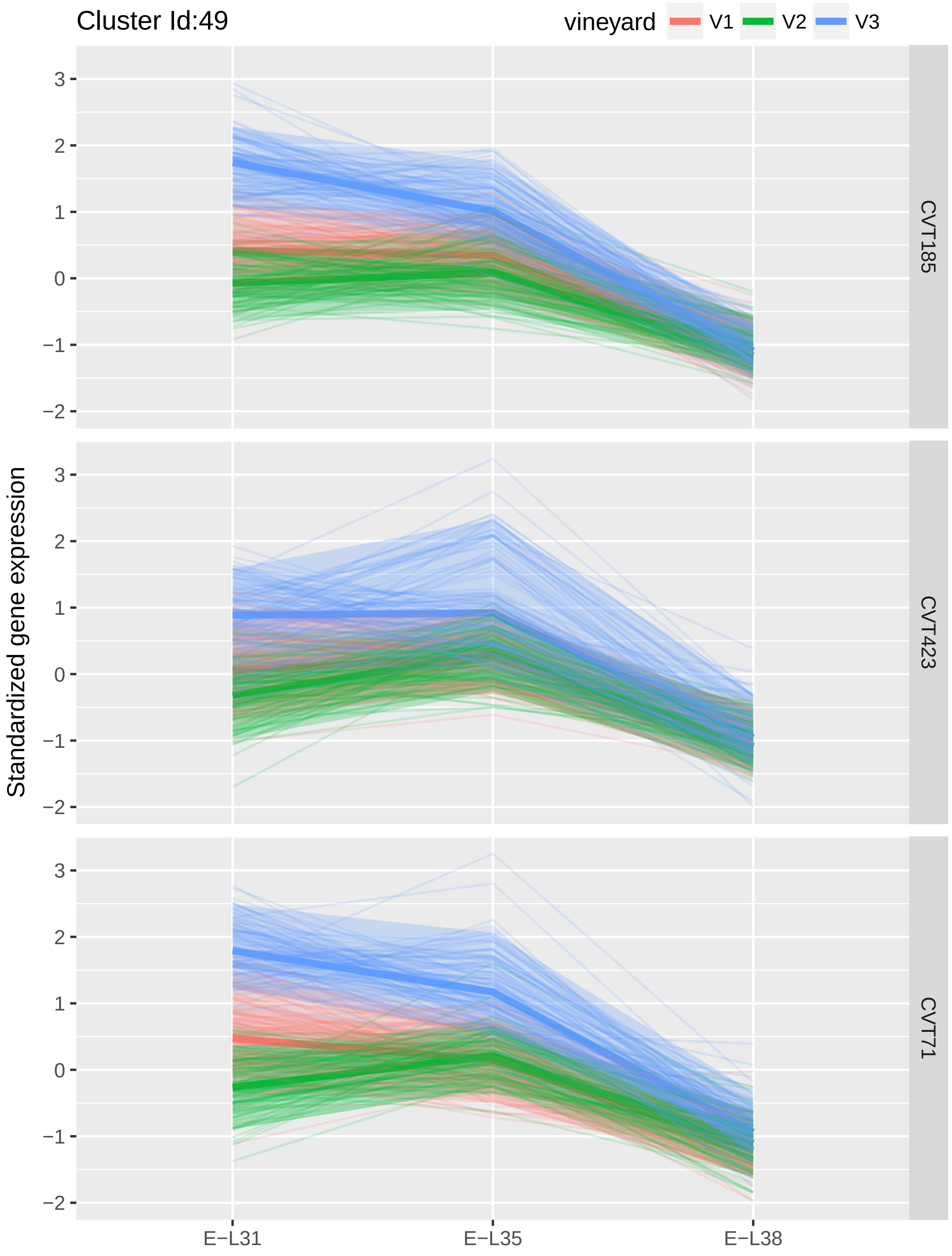
Cluster Id:49
vineyard
V1
V2
V3
Standardized gene expression
CVT185
CVT423
CVT71
3
2
1
0
−1
−2
E−L31
E−L35
E−L38

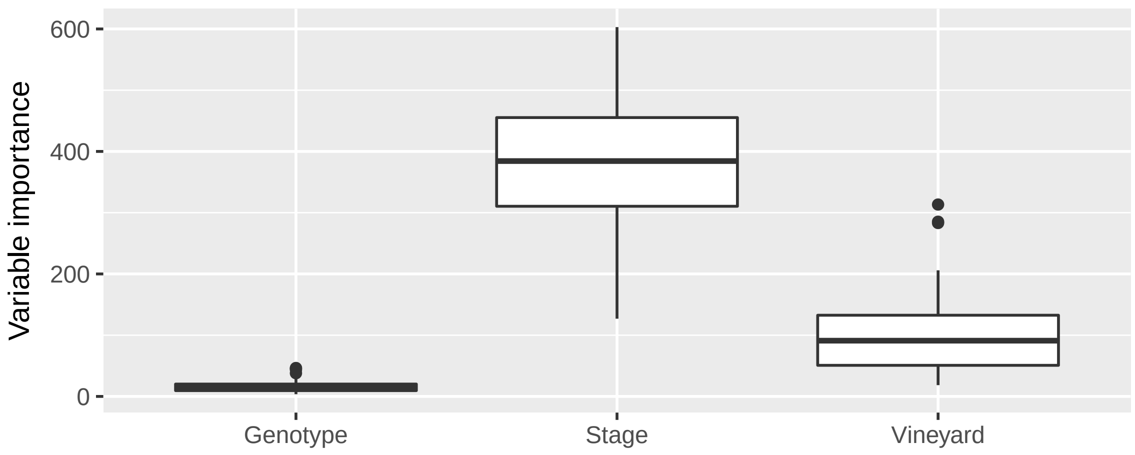
Variable importance
600
400
200
0
Genotype
Stage
Vineyard

Cluster no. 50

```
## Number of genes in the cluster: 196
## Homogeneity Index:    0.75
## Variable importance for Stage:    Median =  448.8  - Rank =  48
## Variable importance for Clone:    Median =  23.66  - Rank =  29
## Variable importance for Vineyard: Median =  30.68  - Rank =  66
##
## Gene ID               Gene Annotation
## VIT_03s0038g04190  -  Pentatricopeptide (PPR) repeat-containing protein
## VIT_18s0001g00060  -  1,4-alpha-glucan branching enzyme, chloroplast precursor
## VIT_13s0019g04310  -  DCP2 (decapping 2)
## VIT_00s0341g00020  -  Transposon protein, CACTA, En/Spm sub-class
## VIT_00s0820g00010  -  Unknown protein
## VIT_08s0007g02210  -  Zinc finger (C3HC4-type ring finger)
## VIT_18s0001g08860  -  U3 small nucleolar RNA-associated protein 4
## VIT_16s0022g02010  -  1-aminocyclopropane-1-carboxylate synthase
## VIT_08s0007g03770  -  Unknown protein
## VIT_05s0020g01340  -  Cullin-1
## VIT_16s0050g02430  -  Receptor protein kinase
## VIT_18s0001g10730  -  Unknown protein
## VIT_10s0042g00540  -  Catalase; Tetratricopeptide helical
## VIT_05s0077g01770  -  PUMILIO 24 (APUM24)
## VIT_12s0034g01370  -  Unknown protein
## VIT_00s1847g00010  -  Adenine phosphoribosyltransferase
## VIT_19s0090g00310  -  AT hook motif-containing protein
## VIT_12s0035g01860  -  ATP-dependent Clp protease ATP-binding subunit ClpX1 (CLPX)
## VIT_14s0066g02550  -  H(+)-ATPase 4 AHA4
## VIT_16s0022g00320  -  Nitric-oxide synthase 1 (AtNOS1)
## VIT_16s0050g01600  -  UDP-glycosyltransferase 88A4
## VIT_03s0091g00560  -  Leucine-rich repeat protein kinase
## VIT_19s0015g00980  -  Protein CWC15
## VIT_19s0090g00420  -  Translation initiation factor eIF-1
## VIT_03s0088g01060  -  Aminotransferase AGD2
## VIT_02s0025g03210  -  Unknown protein
## VIT_04s0023g02620  -  Ubiquitin-conjugating enzyme E2 W
## VIT_13s0019g01450  -  Unknown protein
## VIT_18s0001g13300  -  Unknown
## VIT_08s0040g02740  -  Pectate lyase
## VIT_19s0014g01300  -  Unknown protein
## VIT_01s0011g03290  -  Aluminum-activated malate transporter 9
## VIT_04s0023g02990  -  Cell growth defect factor 1
## VIT_18s0001g02050  -  TolB protein-related
## VIT_02s0025g01500  -  Nucleoside diphosphate kinase 3, (NDK3)
## VIT_19s0014g02490  -  VQ motif-containing protein
## VIT_00s0204g00110  -  TRNA/rRNA methyltransferase (SpoU)
## VIT_01s0137g00190  -  CIP4 (COP1-interacting protein 4)
## VIT_10s0071g01010  -  Unknown protein
## VIT_07s0005g00320  -  Splicing factor, arginine/serine-rich 1/9
## VIT_11s0016g05320  -  Peroxidase
## VIT_06s0009g02520  -  Replication factor C subunit 1
## VIT_01s0011g02340  -  Importin
## VIT_05s0020g00170  -  Zinc knuckle
## VIT_08s0007g07530  -  3-oxoacyl-[acyl-carrier-protein] reductase, chloroplast
## VIT_04s0008g02810  -  Chaperone protein dnaJ
## VIT_08s0007g03590  -  LRR and BTB/POZ domain-containing protein FBL11
```

```
## VIT_14s0066g00830  -  Sirohydrochlorin ferrochelatase
## VIT_00s0199g00240  -  Pyrophosphate-fructose-6-phosphate 1-phosphotransferase
## VIT_07s0130g00420  -  Unknown protein
## VIT_16s0013g02120  -  Pentatricopeptide (PPR) repeat-containing protein
## VIT_19s0015g01920  -  Protein phosphatase 2C
## VIT_17s0000g03610  -  EMB1865
## VIT_03s0091g00080  -  Methylthioribose kinase
## VIT_17s0000g03840  -  Agenet domain-containing protein
## VIT_12s0059g02140  -  F-box family protein
## VIT_14s0006g02490  -  ATP binding protein
## VIT_18s0001g04380  -  Unknown protein
## VIT_14s0030g01810  -  Ribosomal protein S3A (RPS3aB) 40S
## VIT_08s0007g03580  -  LRR and BTB/POZ domain-containing protein FBL11
## VIT_14s0006g02480  -  Phosphatidic acid phosphatase alpha
## VIT_12s0034g01070  -  R protein MLA10
## VIT_05s0020g00310  -  Zinc knuckle
## VIT_08s0040g00530  -  Peroxisomal membrane protein 2
## VIT_07s0104g01630  -  Myb family
## VIT_16s0039g00940  -  DNA binding
## VIT_17s0000g02070  -  Deoxyhypusine synthase
## VIT_01s0011g04020  -  Unknown protein
## VIT_12s0055g00370  -  Harpin-induced protein
## VIT_15s0048g02180  -  Unknown protein
## VIT_07s0095g00170  -  Lysine histidine transporter 1
## VIT_05s0020g02090  -  Unknown
## VIT_04s0023g01210  -  Geranylgeranyl pyrophosphate synthetase 1, chloroplast precursor
## VIT_13s0019g00720  -  Zinc knuckle
## VIT_17s0000g03710  -  Unknown protein
## VIT_14s0068g00910  -  Histidine biosynthesis bifunctional protein
## VIT_14s0030g01270  -  Nucleolar protein 56
## VIT_14s0060g02440  -  Indeterminate(ID)-domain 2
## VIT_13s0019g01220  -  Scarecrow transcription factor 3 (SCL3)
## VIT_17s0000g07330  -  GR-RBP3 (glycine-rich RNA-binding protein 3)
## VIT_14s0068g01970  -  Unknown protein
## VIT_08s0056g00980  -  F-box domain containing protein
## VIT_12s0059g02270  -  Glutamate receptor protein GLR3.4b
## VIT_12s0034g01770  -  Unknown
## VIT_11s0016g01370  -  Early flowering 4
## VIT_00s2483g00010  -  Protein kinase
## VIT_09s0002g02670  -  RNA methyltransferase
## VIT_03s0038g03920  -  Glutamate receptor 2 GLUR2
## VIT_08s0007g08890  -  Cis-zeatin O-beta-D-glucosyltransferase
## VIT_07s0005g02790  -  RPK1 (receptor-like protein kinase 1)
## VIT_03s0038g02130  -  Cold shock protein-1
## VIT_17s0000g05050  -  COBRA-like protein 4
## VIT_01s0026g00860  -  Sterile alpha motif (SAM) domain-containing
## VIT_07s0104g00630  -  Zinc finger (C3HC4-type ring finger)
## VIT_06s0004g03430  -  Prephenate dehydratase
## VIT_16s0022g02320  -  Phosphoglucomutase chloroplast precursor
## VIT_08s0056g00800  -  myb domain protein 60 (VvMYB60)
## VIT_03s0063g02610  -  GRP7 (cold, circadian rhythm, and RNA binding 2)
## VIT_16s0098g00970  -  Subtilisin serine protease
## VIT_05s0049g00100  -  DNA-binding protein
## VIT_19s0014g01660  -  Stage III sporulation protein AA
## VIT_10s0003g03290  -  Intron maturase, type II
```

```
## VIT_18s0001g07440  -  Unknown protein
## VIT_11s0016g04960  -  Aspartate-glutamate racemase
## VIT_18s0001g07980  -  CBL-interacting protein kinase 8 (CIPK8)
## VIT_00s0199g00220  -  Phosphofructokinase
## VIT_12s0034g00060  -  UDP-glucose glucosyltransferase
## VIT_00s0299g00080  -  Unknown
## VIT_19s0014g05230  -  Pentatricopeptide (PPR) repeat-containing protein
## VIT_13s0067g01100  -  RPM1 (resistance to p. syringae pv maculicola 1)
## VIT_13s0067g01310  -  R protein disease resistance protein
## VIT_00s0467g00020  -  TIR-NBS-LRR disease resistance
## VIT_15s0046g01050  -  Abscisic acid receptor PYL9  RCAR1
## VIT_16s0098g01390  -  Yippee
## VIT_06s0061g01240  -  Histone deacetylase (HD2A)
## VIT_08s0007g01740  -  Cysteine peptidase
## VIT_10s0003g01130  -  Armadillo/beta-catenin repeat
## VIT_03s0063g01060  -  myb family
## VIT_14s0108g00730  -  5'-aminoimidazole ribonucleotide synthetase
## VIT_12s0028g03420  -  Ribonuclease P/MRP protein subunit POP1
## VIT_05s0020g00290  -  Zinc knuckle
## VIT_00s0684g00010  -  Protein kinase
## VIT_12s0059g01160  -  Unknown protein
## VIT_19s0014g00530  -  Receptor kinase 2
## VIT_07s0129g01000  -  F-box family protein
## VIT_12s0034g01470  -  RPM1 (resistance to p. syringae pv maculicola 1)
## VIT_08s0040g03170  -  No hit
## VIT_11s0016g05510  -  Unknown protein
## VIT_07s0141g00210  -  Tryptophan synthase, alpha subunit
## VIT_00s0184g00180  -  CRR2 (chlororespiratory reduction 2)
## VIT_03s0063g00780  -  CXE carboxylesterase
## VIT_11s0016g02320  -  Unknown protein
## VIT_02s0025g03410  -  MurE pigment defective embryo (PDE316)
## VIT_13s0067g02950  -  Appr-1-p processing enzyme
## VIT_06s0009g03610  -  EMB2739
## VIT_14s0081g00200  -  AN3 (ANGUSITFOLIA3)
## VIT_05s0020g01750  -  Glutaredoxin
## VIT_01s0011g05860  -  Aspartate-semialdehyde dehydrogenase
## VIT_09s0054g00760  -  TRNA-splicing endonuclease positive effector SEN1
## VIT_00s1291g00020  -  Binding
## VIT_11s0016g01340  -  Unknown protein
## VIT_18s0001g08980  -  Beta-1,4-mannosyltransferase
## VIT_12s0035g01800  -  Auxin response factor 1
## VIT_09s0002g01760  -  EIF-4A3
## VIT_17s0053g01010  -  Transcription initiation factor TFIIB
## VIT_16s0013g01120  -  ERF/AP2 Gene Family (VvERF092)
## VIT_17s0000g07440  -  Replication protein A 70 kDa DNA-binding subunit
## VIT_08s0007g06220  -  No hit
## VIT_04s0008g01250  -  Unknown
## VIT_05s0020g00040  -  Unknown protein
## VIT_06s0009g00960  -  Ubiquitin-specific protease 12 (UBP12)
## VIT_05s0029g00240  -  Pentatricopeptide (PPR) repeat-containing
## VIT_09s0002g03780  -  Integral membrane family protein UPF0497
## VIT_12s0028g00450  -  Carrier protein, Mitochondrial
## VIT_08s0007g07240  -  Armadillo helical
## VIT_10s0003g02430  -  flavonol synthase
## VIT_00s1190g00010  -  Protein kinase
```

```
## VIT_08s0056g00820  -  Cysteine synthase, chloroplast precursor
## VIT_16s0050g01170  -  Unknown
## VIT_02s0025g04090  -  GCS1/HAP2 (generative cell-specific 1)
## VIT_13s0156g00220  -  PRLI-interacting factor A
## VIT_00s0657g00010  -  Branched-chain amino acid aminotransferase
## VIT_13s0019g00370  -  Myosin-like protein XIK
## VIT_11s0016g02500  -  Pentatricopeptide (PPR) repeat-containing protein
## VIT_15s0046g03390  -  K+ efflux antiporter (KEA1)
## VIT_06s0004g03000  -  SNO glutamine amidotransferase
## VIT_16s0039g00920  -  CYP89A28
## VIT_13s0067g00150  -  Hydrolase, alpha/beta fold
## VIT_01s0026g00080  -  Metal-dependent phosphohydrolase HD domain-containing protein
## VIT_01s0011g03930  -  No hit
## VIT_02s0154g00540  -  Protein arginine N-methyltransferase
## VIT_04s0069g00250  -  Unknown
## VIT_11s0016g04830  -  Protein arginine N-methyltransferase
## VIT_09s0002g03190  -  DNA repair protein
## VIT_14s0030g00930  -  Laccase
## VIT_02s0025g00850  -  Lactoylglutathione lyase
## VIT_14s0060g01930  -  Ribosomal protein L18 60S
## VIT_17s0000g09760  -  ABC Transporter (VvMDR2 - VvABCB2)
## VIT_06s0004g00800  -  Amino acid permease
## VIT_06s0004g00430  -  Unknown protein
## VIT_08s0040g02380  -  Hydrolase, alpha/beta fold
## VIT_00s0211g00010  -  Endoribonuclease E-like protein
## VIT_04s0008g03680  -  Unknown protein
## VIT_03s0091g00100  -  Methylthioribose kinase
## VIT_00s0558g00010  -  Branched-chain-amino-acid aminotransferase 3, chloroplastic
## VIT_06s0004g01720  -  Unknown protein
## VIT_18s0001g07410  -  basic helix-loop-helix (bHLH) family
## VIT_09s0002g02150  -  FVE gene
## VIT_13s0064g00180  -  Structural maintenance of chromosomes (SMC) family protein (MSS2)
## VIT_00s0184g00230  -  No hit
## VIT_14s0068g00830  -  2,3,4,5-tetrahydropyridine-2,6-dicarboxylate N-succinyltransferase
## VIT_08s0007g07410  -  H/ACA ribonucleoprotein complex subunit 1 protein 1
## VIT_05s0094g00510  -  Unknown protein
## VIT_07s0031g01320  -  Basic Leucine Zipper Transcription Factor (VvbZIP23)
## VIT_00s1430g00010  -  Protein kinase
## VIT_18s0001g13750  -  Regulator of chromosome condensation (RCC1)
```

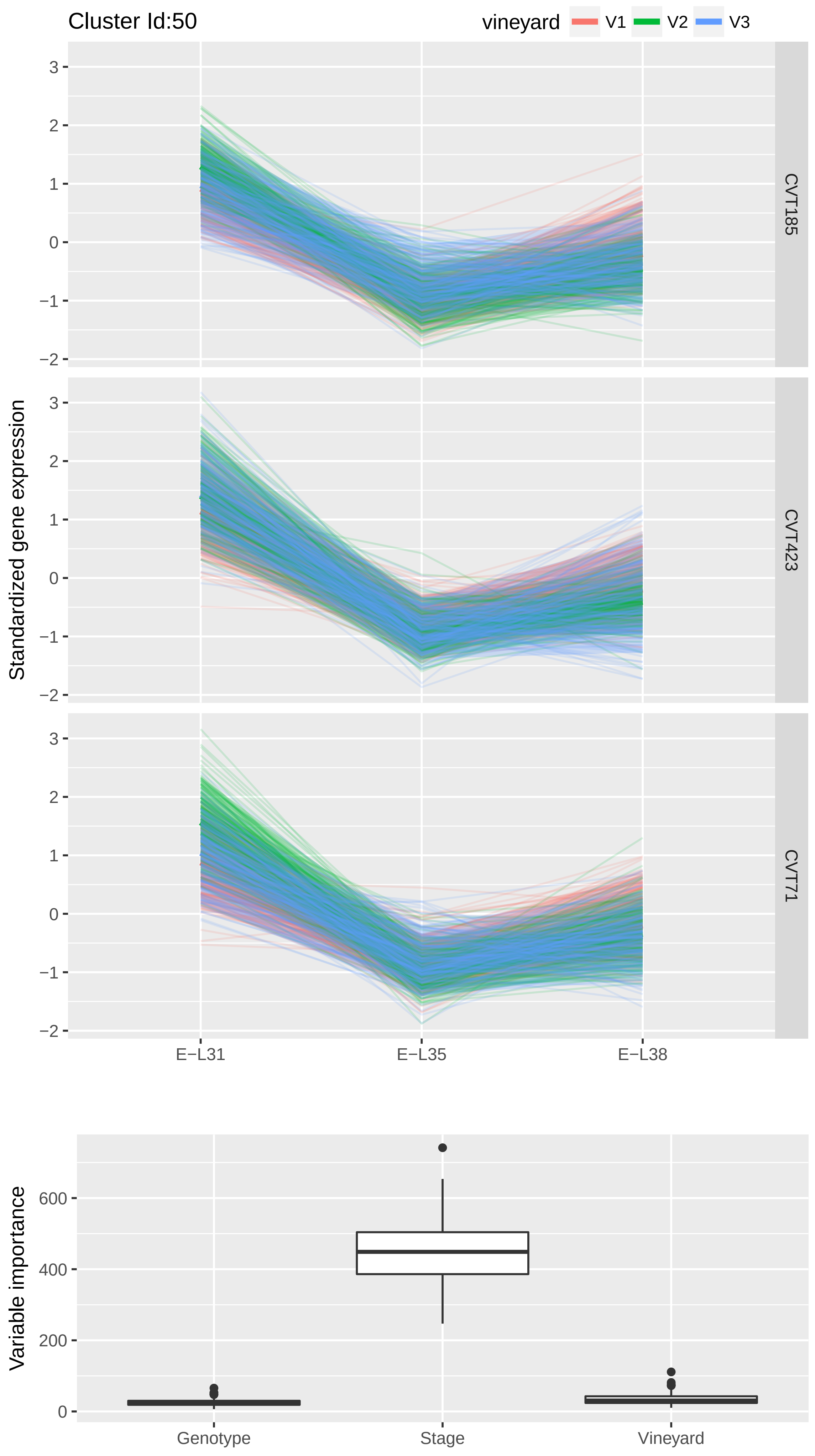
Cluster Id:50
vineyard
V1
V2
V3
Standardized gene expression
CVT185
CVT423
CVT71
3
2
1
0
−1
−2
E−L31
E−L35
E−L38
Variable importance
600
400
200
0
Genotype
Stage
Vineyard

Cluster no. 51

```
## Number of genes in the cluster: 356
## Homogeneity Index:     0.9
## Variable importance for Stage:    Median =  618.5  - Rank =  13
## Variable importance for Clone:    Median =  12.25  - Rank =  91
## Variable importance for Vineyard: Median =  15.54  - Rank =  97
##
## Gene ID               Gene Annotation
## VIT_11s0016g00260  -  Tac7077
## VIT_08s0007g05780  -  Intron maturase, type II
## VIT_04s0008g03240  -  BAM2 (big apical meristem 2)
## VIT_07s0151g00810  -  Unknown protein
## VIT_15s0048g01840  -  Zinc finger (C3HC4-type ring finger)
## VIT_02s0025g03700  -  ATP binding protein
## VIT_04s0023g03290  -  Unknown protein
## VIT_11s0065g00310  -  Auxin response factor 2
## VIT_01s0010g02590  -  ATP-dependent DNA helicase RecQ
## VIT_06s0004g07010  -  Ubiquitin family
## VIT_18s0001g04470  -  Basic Leucine Zipper Transcription Factor (VvbZIP42)
## VIT_17s0000g09900  -  Unknown protein
## VIT_09s0002g07060  -  Receptor protein kinase PERK1
## VIT_04s0023g02560  -  Unknown protein
## VIT_16s0098g01050  -  RNA binding / nucleic acid binding / nucleotide binding [Arabidopsis t
## VIT_12s0035g01520  -  Disease resistance protein RPS2
## VIT_08s0040g01510  -  No hit
## VIT_06s0009g03780  -  Diphthine synthase
## VIT_00s0194g00330  -  Proline-rich family protein
## VIT_07s0104g00480  -  Dynamin
## VIT_14s0083g00410  -  Unknown
## VIT_18s0001g02570  -  IAA-amino acid hydrolase 6
## VIT_05s0094g00930  -  Phosphoglucomutase/phosphomannomutase C terminal
## VIT_03s0038g02050  -  Dirigent protein pDIR7
## VIT_18s0001g09890  -  D-3-phosphoglycerate dehydrogenase
## VIT_01s0127g00850  -  Polygalacturonase BURP
## VIT_18s0001g01660  -  NADH dehydrogenase I subunit M
## VIT_06s0004g02640  -  Receptor protein kinase PERK1
## VIT_07s0005g03910  -  Phosphatidylcholine-sterol O-acyltransferase
## VIT_03s0017g02300  -  Unknown protein
## VIT_07s0151g00860  -  Hydrolase, alpha/beta fold
## VIT_00s0194g00080  -  Phototropic-responsive NPH3
## VIT_17s0000g03890  -  Peptidyl-prolyl isomerase C
## VIT_18s0001g01680  -  Protein kinase
## VIT_05s0094g01020  -  Indole-3-acetate beta-glucosyltransferase
## VIT_11s0016g01940  -  Unknown protein
## VIT_07s0129g00380  -  Phototropic-responsive NPH3
## VIT_14s0030g00410  -  Arabidopsis histidine phosphotransfer AHP1
## VIT_17s0000g06020  -  TCP family transcription factor TCP14
## VIT_06s0080g00420  -  Glucan endo-1,3-beta-glucosidase 7 precursor
## VIT_10s0003g00960  -  Carboxylic ester hydrolase
## VIT_18s0001g05930  -  Pentatricopeptide (PPR) repeat-containing protein
## VIT_15s0048g01750  -  fasciclin arabinogalactan-protein (FLA8)
## VIT_18s0001g08610  -  ERF/AP2 Gene Family (VvAP2-19)
## VIT_17s0000g09910  -  Haloacid dehalogenase hydrolase
## VIT_00s0230g00030  -  No hit
## VIT_15s0048g00550  -  Abnormal floral organs
```

```
## VIT_10s0116g01830  -  Unknown protein
## VIT_17s0000g01840  -  3-oxoacyl-[acyl-carrier-protein] reductase, chloroplast
## VIT_17s0000g02830  -  R protein MLA10
## VIT_12s0134g00260  -  Receptor-like protein kinase ARK1
## VIT_07s0031g02180  -  Protein kinase IRE1
## VIT_18s0157g00180  -  S-adenosylmethionine carrier 1
## VIT_06s0080g00590  -  Harpin-induced protein
## VIT_02s0033g00300  -  myb family
## VIT_00s0304g00020  -  UNE2 (unfertilized embryo sac 2)
## VIT_18s0089g00910  -  Auxin response factor 1
## VIT_01s0011g03180  -  Lysine and histidine specific transporter
## VIT_18s0001g08080  -  No hit
## VIT_14s0083g01210  -  feronia receptor-like kinase
## VIT_15s0046g02000  -  Polygalacturonase GH28
## VIT_13s0067g03590  -  R protein MLA10
## VIT_13s0019g02450  -  BEL1-like homeodomain protein 9 LSN (LARSON)
## VIT_07s0031g02030  -  Polypyrimidine tract-binding protein 3
## VIT_18s0001g06900  -  Unknown protein
## VIT_02s0025g00200  -  BEL1 (BELL 1)
## VIT_09s0054g00430  -  Unknown protein
## VIT_13s0106g00070  -  Ankyrin repeat protein
## VIT_04s0008g05590  -  F-type H+-transporting ATPase b chain CfoII
## VIT_05s0020g02210  -  Histidine-containing phosphotransfer protein
## VIT_08s0007g02770  -  IAA-amino acid hydrolase 1 (ILR1)
## VIT_16s0050g02530  -  Myb Triptychon
## VIT_14s0108g00840  -  Unknown protein
## VIT_02s0241g00050  -  Unknown protein
## VIT_11s0118g00780  -  Zinc finger (C3HC4-type ring finger)
## VIT_12s0035g00900  -  TIFY gene family (VvJAZI 0)
## VIT_07s0005g02470  -  Cellulose synthase CESA3
## VIT_13s0106g00230  -  Ankyrin
## VIT_08s0007g00980  -  Integral membrane family protein UPF0497
## VIT_15s0046g03240  -  Unknown protein
## VIT_11s0118g00610  -  Methyltransferase type 11
## VIT_05s0020g02690  -  Copper-binding family protein
## VIT_08s0007g06050  -  No hit
## VIT_18s0076g00210  -  Alpha/beta hydrolase fold
## VIT_13s0019g02220  -  No hit
## VIT_06s0061g00770  -  Ribosomal protein L9, chloroplast (CL9) 50S
## VIT_05s0077g01240  -  Calmodulin-binding transcription activator 6
## VIT_13s0074g00040  -  Phytol kinase 2, chloroplast precursor
## VIT_14s0068g00040  -  No hit
## VIT_08s0007g07400  -  Glutamate dehydrogenase 1
## VIT_01s0011g00930  -  FK506-binding protein genes family (VvFKBP13)
## VIT_05s0094g00100  -  FK506-binding protein genes family (VvFKBP16-3)
## VIT_01s0137g00170  -  B-keto acyl reductase
## VIT_10s0003g00380  -  Homeobox-leucine zipper protein HB52
## VIT_18s0001g10420  -  Remorin
## VIT_00s0238g00020  -  Unknown protein
## VIT_05s0029g00080  -  SERK family receptor-like protein kinase
## VIT_16s0098g01520  -  WD-40 repeat
## VIT_18s0001g11750  -  Ribosomal protein S9
## VIT_13s0019g00240  -  Glycosyltransferase family 14 Beta-1-3-galactosyl-O-glycosyl-glycoprot
## VIT_13s0047g01190  -  NADH:cytochrome b5 reductase
## VIT_18s0041g00390  -  Cysteine protease inhibitor
```

```
## VIT_02s0033g00290  -  Pinoresinol-lariciresinol reductase
## VIT_07s0031g02170  -  Glutamate receptor GLR3.6
## VIT_11s0052g01120  -  Zinc finger protein 2
## VIT_11s0016g03540  -  Auxin-responsive protein IAA27
## VIT_03s0167g00220  -  MEK1
## VIT_00s2077g00020  -  Alkaline alpha galactosidase II
## VIT_02s0025g00290  -  Pentatricopeptide (PPR) repeat-containing protein
## VIT_12s0059g01790  -  Caffeic acid O-methyltransferase (VvOMT1)
## VIT_04s0008g05100  -  Cytochrome c oxidase subunit Vb
## VIT_15s0046g03770  -  Anaphase-promoting complex component APC11
## VIT_03s0063g01050  -  RNA recognition motif (RRM)-containing protein
## VIT_12s0035g01250  -  Disease resistance protein (NBS-LRR class)
## VIT_19s0015g00530  -  fasciclin arabinogalactan-protein (FLA1)
## VIT_00s0211g00130  -  Unknown
## VIT_13s0139g00340  -  R protein MLA10
## VIT_18s0001g09390  -  Protein phosphatase 2C
## VIT_08s0040g00160  -  Unknown protein
## VIT_01s0150g00480  -  Protein binding protein
## VIT_11s0016g01430  -  Zinc finger (C3HC4-type ring finger)
## VIT_02s0012g01120  -  Glycine cleavage system H protein, mitochondrial
## VIT_06s0004g07880  -  Allergen
## VIT_14s0108g01660  -  Biotin carboxyl carrier protein of acetyl-CoA carboxylase
## VIT_13s0019g00320  -  Photosystem II protein P PsbP
## VIT_10s0003g00400  -  No hit
## VIT_14s0083g00110  -  Allene oxide cyclase (jasmonates from fatty acids)
## VIT_11s0016g00300  -  Pectinesterase family
## VIT_17s0000g03010  -  Phosphatidyl serine synthase
## VIT_01s0011g02860  -  CHUP1 (chloroplast unusual positioning 1)
## VIT_03s0091g01240  -  Serine carboxypeptidase S10 / Anthocyanin Acyl-transferase
## VIT_18s0001g06220  -  F-box protein (FBW2)
## VIT_18s0001g05910  -  Cis-zeatin O-beta-D-glucosyltransferase
## VIT_16s0050g00670  -  Kinesin motor
## VIT_12s0028g03100  -  GPRI1 (GOLDEN2 1)
## VIT_18s0001g15360  -  Thylakoid lumenal 29.8 kDa protein
## VIT_14s0060g00680  -  Lipase GDSL
## VIT_00s0202g00040  -  Ankyrin repeat protein
## VIT_18s0001g03610  -  Auxin-independent growth promoter
## VIT_11s0016g01300  -  Myb protein (VvMYBPAR)
## VIT_00s0361g00090  -  Phototropic-responsive NPH3 protein
## VIT_07s0130g00060  -  Unknown protein
## VIT_01s0026g00490  -  Nodulin
## VIT_00s0391g00020  -  Protein cbbY
## VIT_16s0039g01450  -  Growth-regulating factor 5
## VIT_00s1314g00010  -  basic helix-loop-helix (bHLH) family
## VIT_08s0007g07470  -  Galactose mutarotase
## VIT_05s0049g01870  -  Import receptor subunit TOM22 homolog 2, Mitochondrial
## VIT_17s0000g03910  -  flavonoid 3-monooxygenase
## VIT_12s0057g00600  -  Unknown protein
## VIT_15s0021g01850  -  FK506-binding protein genes family (VvFKBP20-2)
## VIT_06s0009g01380  -  Ethylene-insensitive 3 (EIN3)
## VIT_06s0061g01040  -  Argonaute protein
## VIT_01s0011g01120  -  R protein MLA10
## VIT_13s0064g01870  -  R protein MLA10
## VIT_03s0180g00090  -  Receptor kinase
## VIT_15s0048g02910  -  Unknown
```

```
## VIT_11s0016g05340  -  ERF/AP2 Gene Family (VvERF043)
## VIT_04s0023g03010  -  fructose-bisphosphate aldolase, chloroplast precursor
## VIT_11s0016g04810  -  BAS1 (PHYB activation tagged suppressor 1)
## VIT_18s0001g13010  -  Mitogen-activated Protein Kinase (VvMPK11)
## VIT_14s0030g01870  -  NIMA protein kinase
## VIT_12s0035g00970  -  Evolutionarily conserved C-terminal region 11 ECT11
## VIT_18s0001g11090  -  Uroporphyrinogen-III synthase
## VIT_04s0023g03650  -  Galactose mutarotase-like
## VIT_19s0027g01890  -  Amino acid permease
## VIT_11s0149g00040  -  Adenylate kinase
## VIT_18s0001g13040  -  Basic Leucine Zipper Transcription Factor (VvbZIP47)
## VIT_03s0091g00500  -  Unknown protein
## VIT_12s0028g01550  -  Peroxisomal membrane 22 kDa
## VIT_00s0211g00170  -  Unknown
## VIT_11s0078g00310  -  Isoamylase-type starch-debranching enzyme 1
## VIT_09s0002g07220  -  Unknown
## VIT_11s0016g00100  -  Adapter protein SPIKE1 (SPK1)
## VIT_05s0051g00910  -  Unknown protein
## VIT_01s0010g01210  -  Uracil-DNA glycosylase
## VIT_13s0019g00730  -  Annexin ANN3
## VIT_10s0071g01100  -  Alpha-galactosidase precursor
## VIT_03s0063g01480  -  CYP82C4
## VIT_18s0001g13020  -  No hit
## VIT_07s0005g06300  -  Phototropic-responsive NPH3
## VIT_06s0004g03130  -  Auxin response factor 4
## VIT_18s0001g05440  -  Methyltransferase type 11
## VIT_01s0011g00460  -  Unknown protein
## VIT_08s0007g00410  -  Myb domain protein 91
## VIT_06s0004g03210  -  Unknown
## VIT_14s0030g01790  -  Ndr family protein
## VIT_05s0077g00670  -  Unknown protein
## VIT_04s0008g01740  -  Spermine synthase ACAULIS5
## VIT_02s0025g00990  -  ABC Transporter (VvMRP16 - VvABCC16)
## VIT_07s0031g02140  -  Amino acid permease
## VIT_14s0068g01960  -  Acetolactate synthase III, chloroplast precursor
## VIT_00s0253g00140  -  Polyneuridine-aldehyde esterase precursor
## VIT_14s0081g00560  -  WRKY Transcription Factor (VvWRKY42)
## VIT_08s0058g01070  -  Xanthine/uracil permease
## VIT_15s0048g00090  -  No hit
## VIT_09s0002g03840  -  RPS5 (resistant to p. syringae 5)
## VIT_09s0002g00450  -  Subtilase
## VIT_07s0005g02120  -  Cyclase/dehydrase
## VIT_05s0051g00070  -  Coenzyme F420 hydrogenase/dehydrogenase beta subunit
## VIT_04s0008g01870  -  ATMYB66/WER/WER1 (WEREWOLF 1)
## VIT_13s0320g00060  -  Carboxyl-terminal peptidase
## VIT_04s0008g06130  -  KNAT2 (knotted1-like homeobox gene 3)
## VIT_16s0022g00670  -  Vacuolar invertase 1, GIN1
## VIT_15s0046g01990  -  UDP-glucosyltransferase
## VIT_04s0008g01900  -  Unknown protein
## VIT_05s0049g02080  -  Calcium ion binding protein
## VIT_14s0030g02240  -  Carbon-sulfur lyase
## VIT_08s0040g02770  -  Unknown protein
## VIT_09s0002g07890  -  R protein disease resistance protein
## VIT_08s0007g07200  -  Tocotrienol/plastoquinone prenyltransferase
```

```
## VIT_18s0041g00210  -  TIR-NBS-LRR disease resistance
## VIT_10s0003g00250  -  Glucan phosphorylase
## VIT_05s0062g00430  -  UDP-glucose:flavonoid 7-O-glucosyltransferase
## VIT_03s0063g02010  -  Protease
## VIT_02s0033g00230  -  Unknown protein
## VIT_18s0001g03850  -  Ribosomal protein S10 30S
## VIT_02s0109g00260  -  Retrotransposon gag protein
## VIT_08s0007g06270  -  Squamosa promoter-binding protein (VvSBP8)
## VIT_03s0038g03810  -  Serine/threonine Protein kinase BNK1
## VIT_04s0044g00530  -  PAP/fibrillin family
## VIT_12s0057g00400  -  Threonyl-tRNA synthetase / threonine--tRNA ligase
## VIT_15s0048g00770  -  Kinesin motor KHC
## VIT_01s0011g02030  -  Serine carboxypeptidase S10
## VIT_03s0091g00510  -  Unknown
## VIT_00s0271g00100  -  Unknown
## VIT_01s0011g04080  -  Zinc finger (C3HC4-type ring finger)
## VIT_18s0001g00820  -  Thioredoxin-like protein CDSP32
## VIT_13s0019g01950  -  R protein MLA10
## VIT_13s0019g03350  -  Sedoheptulose-1,7-bisphosphatase (SBPase), Chloroplast
## VIT_13s0067g01830  -  Steroid 5alpha-reductase
## VIT_09s0002g04010  -  Ferredoxin
## VIT_07s0129g01070  -  Leucine-rich repeat protein kinase
## VIT_05s0020g03040  -  Unknown
## VIT_02s0025g00190  -  No hit
## VIT_13s0106g00060  -  Ankyrin repeat
## VIT_00s0193g00110  -  Ankyrin repeat protein
## VIT_12s0055g00620  -  Glutamate synthase (NADH), chloroplast
## VIT_07s0031g00920  -  Inositol-3-phosphate synthase
## VIT_17s0000g01490  -  CYP94B3
## VIT_18s0001g01860  -  Preprotein translocase secA
## VIT_19s0014g03850  -  Cytochrome B6-F complex iron-sulfur subunit, PETC
## VIT_19s0090g01000  -  VTC2 (vitamin C defective)
## VIT_04s0008g05340  -  Bundle-sheath defective protein 2
## VIT_10s0092g00370  -  CYP71D7
## VIT_08s0007g05530  -  Glutaredoxin
## VIT_17s0000g05250  -  Unknown protein
## VIT_08s0007g03820  -  PTL (PETAL LOSS)
## VIT_11s0037g00020  -  No hit
## VIT_16s0022g01390  -  flavonoid 3-monooxygenase
## VIT_18s0001g10550  -  LHCA5 (Photosystem I light harvesting complex gene 5)
## VIT_07s0005g02440  -  Pectinacetylesterase
## VIT_14s0219g00290  -  Esterase/lipase/thioesterase
## VIT_04s0008g05520  -  Unknown protein
## VIT_19s0177g00210  -  Protein disulfide isomerase (PDIL) protein
## VIT_17s0000g06010  -  Unknown protein
## VIT_17s0000g04210  -  Oleosin OLE-5
## VIT_05s0049g01130  -  Aldo/keto reductase
## VIT_07s0095g00760  -  Methionyl-tRNA synthetase
## VIT_18s0041g00370  -  Double strand break repair protein (XRCC4)
## VIT_06s0004g03020  -  Beta-galactosidase
## VIT_13s0073g00550  -  Beta-D-glucosidase
## VIT_08s0007g03250  -  Unknown protein
## VIT_15s0021g02210  -  Unknown protein
## VIT_17s0000g00110  -  Rubredoxin
## VIT_05s0020g04020  -  Unknown protein
```

```
## VIT_00s0878g00020  -  Stachyose synthase precursor
## VIT_18s0041g00550  -  Proton-dependent oligopeptide transport (POT) family protein
## VIT_14s0068g00280  -  Unknown protein
## VIT_13s0073g00530  -  Beta-D-glucosidase
## VIT_07s0005g02590  -  Polygalacturonase GH28
## VIT_06s0004g03590  -  TOE1 (target of eat1 1)  related to apetala2 7
## VIT_06s0004g03070  -  Zinc finger (C2H2 type) JACKDAW
## VIT_03s0038g00670  -  fructose-bisphosphate aldolase, chloroplast precursor
## VIT_06s0004g04320  -  Alcohol dehydrogenase 3
## VIT_05s0020g03440  -  Photosystem II 11 kDa protein PSB27
## VIT_02s0025g02840  -  Ankyrin 3, epithelial isoform a
## VIT_01s0011g00590  -  Acclimation of photosynthesis to environment
## VIT_07s0005g00940  -  Kinesin motor protein
## VIT_13s0067g03640  -  R protein MLA10
## VIT_14s0108g01440  -  Unknown protein
## VIT_16s0022g02370  -  Unknown protein
## VIT_07s0129g00320  -  formamidase, putative / formamide amidohydrolase, putative
## VIT_06s0004g01390  -  S-receptor kinase
## VIT_05s0062g00630  -  UDP-glucose transferase (UGT75B2)
## VIT_01s0127g00740  -  Quinone oxidoreductase, chloroplast precursor
## VIT_17s0000g05460  -  Nodulin
## VIT_18s0001g10500  -  Abscisic acid 8` hydroxylase (CYP707A2) (VvA8H-CYP707A2.3)
## VIT_07s0005g06160  -  No hit
## VIT_12s0028g01350  -  RKF1 (receptor-like kinase in flowers 1)
## VIT_04s0008g03050  -  Acetylglutamate kinase
## VIT_11s0103g00210  -  F-box family protein
## VIT_11s0065g00570  -  Glutamate carboxypeptidase (AMP1)
## VIT_08s0058g01010  -  Unknown
## VIT_04s0044g00880  -  WD40
## VIT_02s0109g00080  -  Phosphoribulokinase
## VIT_10s0042g00640  -  Cinnamoyl-CoA reductase
## VIT_04s0023g01150  -  Unknown protein
## VIT_16s0013g00220  -  Metacaspase AtMCP1b
## VIT_01s0137g00820  -  Phototropic-responsive NPH3
## VIT_04s0023g01140  -  GNS1/SUR4 membrane protein
## VIT_00s0193g00160  -  Ankyrin
## VIT_08s0007g02170  -  Yippee
## VIT_19s0093g00510  -  S-2-hydroxy-acid oxidase, peroxisomal
## VIT_10s0003g03830  -  S-2-hydroxy-acid oxidase, peroxisomal
## VIT_16s0039g01770  -  Major pollen allergen Car b 1 isoforms 1A and 1B
## VIT_12s0059g02320  -  Unknown protein
## VIT_01s0010g02750  -  Lipoxygenase
## VIT_08s0007g01570  -  fructose 1,6-bisphosphatase
## VIT_07s0129g00910  -  Protein kinase
## VIT_07s0104g00510  -  MRNA-binding protein precursor
## VIT_14s0060g02430  -  Photosystem II oxygen evolving complex protein PsbP
## VIT_08s0007g03630  -  Calmodulin binding protein
## VIT_18s0122g00980  -  Glucan endo-1,3-beta-glucosidase 7 precursor
## VIT_17s0000g00270  -  GT2-like trihelix DNA-binding protein
## VIT_03s0017g00560  -  Disease resistance RPP13 protein 4
## VIT_03s0038g04110  -  AT-hook DNA-binding protein
## VIT_05s0094g01000  -  Indole-3-acetate beta-glucosyltransferase
## VIT_03s0063g00770  -  Carboxyesterase 12; CXE12
## VIT_13s0139g00320  -  R protein MLA10
## VIT_14s0108g00940  -  Glycogen synthase
```

```
## VIT_05s0102g00190  -  No hit
## VIT_14s0060g00990  -  Unknown
## VIT_04s0008g01720  -  Mechanosensitive ion channel
## VIT_15s0021g01150  -  Calcium-dependent protein kinase-related
## VIT_14s0006g02900  -  Monoglyceride lipase
## VIT_01s0010g00590  -  Short-chain dehydrogenase/reductase SDR
## VIT_03s0091g00490  -  Unknown protein
## VIT_00s0373g00030  -  HSL2 (HAESA-like 2)
## VIT_17s0000g00930  -  Unknown protein
## VIT_18s0001g13030  -  No hit
## VIT_11s0016g01020  -  flavonoid 3-monooxygenase
## VIT_08s0040g02650  -  Unknown protein
## VIT_06s0004g07220  -  Indole-3-acetate beta-glucosyltransferase
## VIT_19s0014g03240  -  Beta-mannosidase 4
## VIT_14s0060g00850  -  Thylakoid lumenal 21.5 kDa protein, chloroplast precursor
## VIT_00s0193g00080  -  Ankyrin
## VIT_13s0019g01880  -  R protein MLA10
## VIT_00s0187g00050  -  No hit
## VIT_11s0065g00990  -  Replication protein A 70 kDa DNA-binding subunit
## VIT_00s0286g00140  -  Serine/threonine-protein kinase receptor ARK3
## VIT_11s0078g00380  -  Isoamylase isoform 1
## VIT_19s0085g00380  -  Phosphoglycerate kinase
## VIT_07s0141g00330  -  F-box protein (FBL17)
## VIT_09s0002g00550  -  Lipase GDSL 1
## VIT_00s2269g00010  -  Armadillo/beta-catenin repeat
## VIT_08s0007g05540  -  DNA-directed RNA polymerase II subunit E
## VIT_05s0049g00390  -  E8 protein
## VIT_06s0004g04980  -  Scarecrow transcription factor 14 (SCL14)
## VIT_05s0062g00520  -  UDP-glucose:flavonoid 7-O-glucosyltransferase
## VIT_02s0087g00840  -  ABC Transporter (VvWBC29 - VvABCG29)
## VIT_00s1203g00010  -  Auxin response factor ARF6
## VIT_03s0038g00470  -  Subtilase family protein
## VIT_10s0092g00240  -  Alpha-glucosidase 1 (AGLU1)
## VIT_04s0008g03200  -  Lachrymatory factor synthase
## VIT_05s0062g00250  -  Xyloglucan endotransglucosylase/hydrolase 15
```

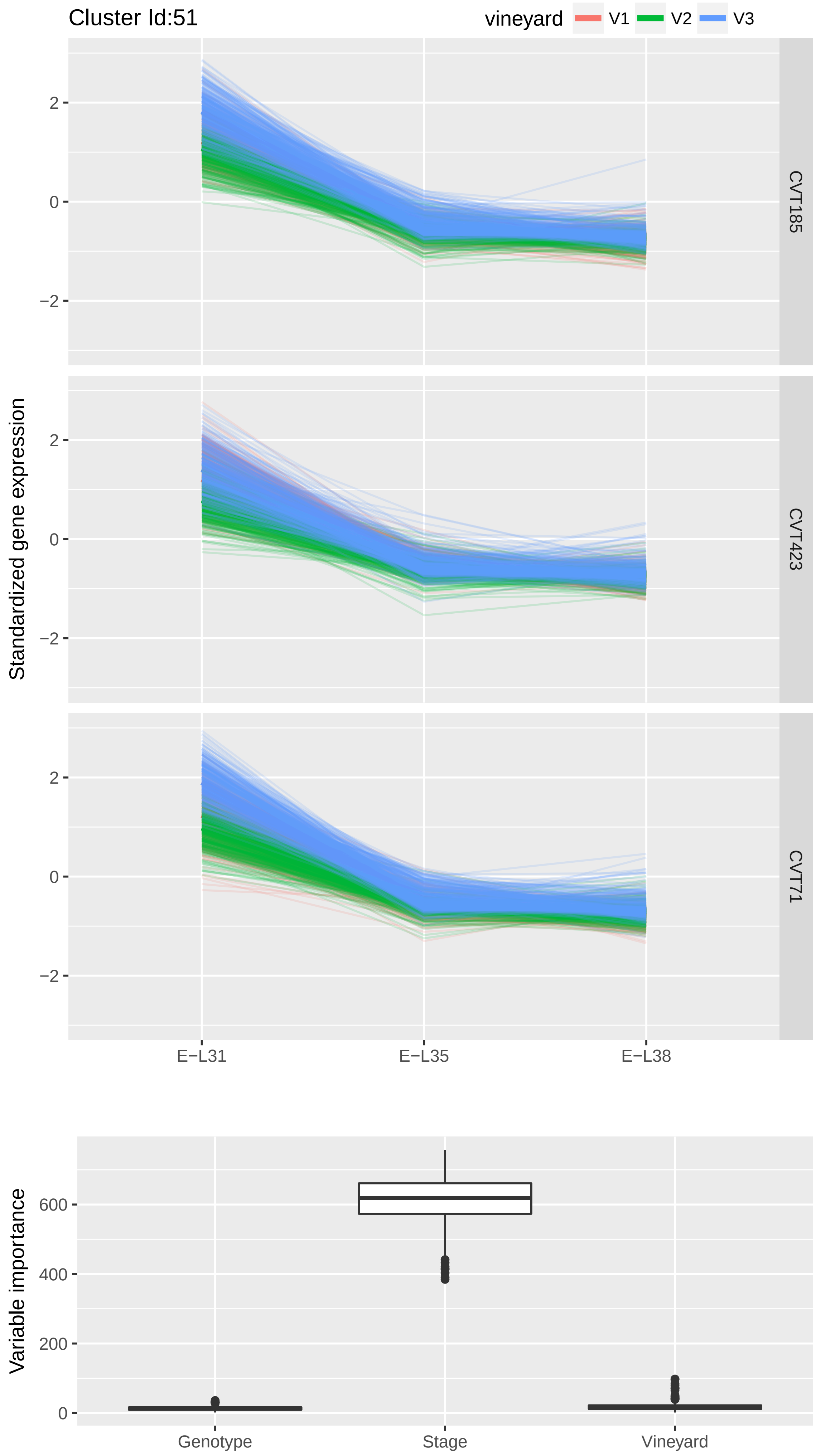

Cluster Id:51
vineyard
V1
V2
V3
Standardized gene expression
2
0
−2
CVT185
CVT423
CVT71
E−L31
E−L35
E−L38
Variable importance
600
400
200
0
Genotype
Stage
Vineyard

Cluster no. 52

```
## Number of genes in the cluster: 129
## Homogeneity Index:    0.48
## Variable importance for Stage:    Median =  276.1  - Rank =  75
## Variable importance for Clone:    Median =  21.48  - Rank =  39
## Variable importance for Vineyard: Median =  61.94  - Rank =  31
##
## Gene ID               Gene Annotation
## VIT_06s0004g01120  -  YqeH GTP binding
## VIT_08s0007g04960  -  Unknown protein
## VIT_13s0175g00100  -  Sad1/unc-84 protein
## VIT_19s0014g02920  -  Pentatricopeptide (PPR) repeat-containing
## VIT_04s0008g04780  -  Protein phosphatase 2C DBP
## VIT_04s0008g02280  -  Unknown protein
## VIT_17s0000g00090  -  Crumpled leaf
## VIT_17s0000g06510  -  Exonuclease
## VIT_07s0005g04180  -  Glutathione S-transferase
## VIT_13s0019g03290  -  Unknown protein
## VIT_09s0070g00030  -  Stromal cell-derived factor 2 protein precursor (SDF2 protein)
## VIT_01s0150g00260  -  Zinc finger (C3HC4-type ring finger)
## VIT_04s0008g04190  -  Bromo adjacent region
## VIT_14s0068g01670  -  ACT domain-containing protein (ACR6)
## VIT_09s0002g01690  -  Unknown protein
## VIT_19s0090g01300  -  Syntaxin 52
## VIT_05s0020g02450  -  FAR1-related sequence 7
## VIT_14s0171g00230  -  Transcription termination factor mitochondrial mTERF
## VIT_09s0002g07210  -  Molecular chaperone DnaJ
## VIT_11s0037g00200  -  Unknown
## VIT_18s0001g12720  -  Endoplasmatic reticulum retrieval protein 1B
## VIT_18s0001g12080  -  Phospholipid/glycerol acyltransferase
## VIT_04s0043g00870  -  Unknown protein
## VIT_02s0012g01920  -  RCD1 (radical-induced cell death1)
## VIT_09s0002g00930  -  Small nuclear ribonucleoprotein LSM1
## VIT_05s0094g01380  -  Unknown protein
## VIT_06s0080g00030  -  Aldo/keto reductase
## VIT_12s0059g00760  -  Adenosine 5' phosphosulfate reductase
## VIT_18s0041g01840  -  Unknown
## VIT_02s0025g00440  -  Transducin protein
## VIT_05s0102g00370  -  Receptor protein kinase (ACR4)
## VIT_06s0061g01530  -  Protein tyrosine phosphatase
## VIT_14s0066g01310  -  CAAX farnesyltransferase beta subunit
## VIT_07s0005g03040  -  Poly(ADP)-ribose polymerase
## VIT_07s0141g00130  -  Protein phosphatase 2C
## VIT_10s0116g00920  -  CERK1 (chitin elicitor receptor kinase 1)
## VIT_10s0003g04120  -  Galactosyltransferase
## VIT_09s0002g04120  -  Ethylene-responsive protein
## VIT_12s0028g02500  -  Unknown protein
## VIT_03s0091g00660  -  Unknown protein
## VIT_18s0001g08260  -  Xylulose kinase
## VIT_05s0102g00240  -  Endonuclease III homologue
## VIT_18s0001g05680  -  Cryptochrome 1
## VIT_15s0046g02900  -  Protein kinase
## VIT_02s0025g03550  -  Protein kinase
## VIT_05s0020g01810  -  Adenyl cyclase
## VIT_01s0011g02270  -  MAP3K epsilon protein kinase
```

```
## VIT_02s0025g01660  -  Seven in absentia SINA5
## VIT_10s0071g00210  -  Myosin VA (heavy chain 12, myoxin)
## VIT_15s0048g01990  -  Methyltransferase dehydration-responsive
## VIT_11s0065g00940  -  Preprotein translocase secY subunit, chloroplast (CpSecY)
## VIT_01s0011g02850  -  Unknown protein
## VIT_03s0180g00200  -  Limonoid UDP-glucosyltransferase (VvGT2)
## VIT_00s0301g00110  -  Bifunctional nuclease
## VIT_15s0107g00550  -  Tetratricopeptide repeat domain male sterility MS5
## VIT_17s0000g06640  -  formin-2
## VIT_07s0129g01040  -  Copper-exporting ATPase HMA1 (Heavy metal ATPase 1)
## VIT_04s0008g03460  -  Histidine kinase 1 AHK3
## VIT_04s0023g00030  -  CHR4/MI-2-like (chromatin remodeling 4)
## VIT_00s0220g00180  -  Glycosyl hydrolase family 20 protein
## VIT_16s0050g02640  -  Polypyrimidine tract-binding protein 2
## VIT_17s0000g03760  -  Endonuclease/exonuclease/phosphatase family
## VIT_04s0008g03010  -  Aspartyl-tRNA synthetase
## VIT_08s0040g00760  -  Protein phosphatase 2C
## VIT_09s0002g04070  -  Unknown protein
## VIT_05s0020g04510  -  GDP-mannose 3,5-epimerase 1
## VIT_14s0068g00990  -  No hit
## VIT_02s0033g00120  -  Pleckstriny (PH) domain-containing protein
## VIT_13s0074g00100  -  3-phosphoinositide-dependent protein kinase
## VIT_00s0340g00040  -  Beta-hexosaminidase beta chain precursor
## VIT_19s0140g00200  -  Ubiquitin-specific protease 15 (UBP15)
## VIT_10s0071g00940  -  Oligopeptidase B
## VIT_14s0006g01420  -  Ser/Thr receptor kinase
## VIT_06s0004g06620  -  Zinc finger (C3HC4-type ring finger)
## VIT_07s0141g00040  -  Protein phosphatase 2C
## VIT_04s0008g02590  -  Selenoprotein
## VIT_08s0007g02240  -  Calcium/proton exchanger CAX3
## VIT_12s0035g00880  -  Lactoylglutathione lyase
## VIT_12s0034g02140  -  S3 self-incompatibility locus
## VIT_17s0000g00610  -  Ubiquitin-conjugating enzyme 26 UBC27
## VIT_12s0028g03120  -  No hit
## VIT_05s0020g00750  -  Novel plant snare 13
## VIT_18s0001g01620  -  AarF domain containing kinase 1
## VIT_17s0000g09780  -  Methionine sulfoxide reductase A
## VIT_06s0004g08160  -  DEAD box RNA helicase (RH20)
## VIT_02s0033g00240  -  Glucosyltransferase twi1
## VIT_14s0036g01450  -  Unknown protein
## VIT_07s0205g00120  -  Major facilitator superfamily protein (MFS) Spinster
## VIT_18s0001g12780  -  Ornithine carbamoyltransferase
## VIT_14s0066g01030  -  Tubulin gamma complex component 2
## VIT_14s0066g00210  -  Unknown protein
## VIT_05s0094g01550  -  RAB GDP dissociation inhibitor 1 ATGD1
## VIT_03s0038g02770  -  Programmed cell death 6
## VIT_00s0499g00040  -  Chromatin assembly factor-1 (FASCIATA1) (FAS1)
## VIT_05s0077g01160  -  Transducin family protein / WD-40
## VIT_14s0083g00260  -  No hit
## VIT_13s0158g00380  -  Basic Leucine Zipper Transcription Factor (VvbZIP36)
## VIT_13s0101g00410  -  Vesicle-associated membrane protein 72
## VIT_10s0003g00110  -  FES1 (frigida-essential 1)
## VIT_07s0151g00650  -  ABI3-interacting protein 2
## VIT_05s0049g01860  -  Receptor-like protein kinase
## VIT_19s0014g00560  -  Exostosin
```

```
## VIT_12s0028g00350  -  T-complex protein 1 epsilon subunit
## VIT_14s0108g01040  -  KH domain-containing protein
## VIT_03s0038g02380  -  RAC G-protein Rac1
## VIT_01s0011g05700  -  Ankyrin repeat
## VIT_08s0058g00530  -  Unknown protein
## VIT_05s0020g01550  -  Isoleucyl-tRNA synthetase
## VIT_04s0044g01680  -  Chromatin remodeling 24
## VIT_10s0071g00270  -  Abscisic acid-insensitive protein 3 (ABI3)
## VIT_07s0005g05790  -  VAMP726 (VESICLE-associated membrane protein)
## VIT_18s0089g01000  -  F-box family protein
## VIT_00s0187g00240  -  SWIB complex BAF60b domain-containing protein
## VIT_12s0057g01390  -  Unknown protein
## VIT_01s0150g00200  -  FK506-binding protein genes family (VvFKBP19)
## VIT_04s0008g00750  -  Unknown protein
## VIT_14s0083g01050  -  putative MADS-box sepallata 1 (VviSEP1)
## VIT_19s0014g02310  -  MAP kinase (Map 4 kinase alpha2)
## VIT_03s0017g00610  -  Disease resistance protein (CC-NBS-LRR class)
## VIT_02s0025g04150  -  Zinc finger (C3HC4-type ring finger)
## VIT_00s0338g00010  -  F-box family protein
## VIT_00s0253g00080  -  Methyl jasmonate esterase
## VIT_06s0004g06920  -  fructose-6-phosphate-2-kinase
## VIT_04s0008g04520  -  Zinc finger, FYVE domain containing 26
## VIT_11s0016g01260  -  Early flowering 8 (ELF8)
## VIT_18s0001g07590  -  Argininosuccinate lyase
## VIT_00s0187g00190  -  SWIB complex BAF60b domain-containing protein
## VIT_05s0094g01620  -  Multisynthetase complex auxiliary component p43
## VIT_16s0100g01230  -  Unknown protein
```

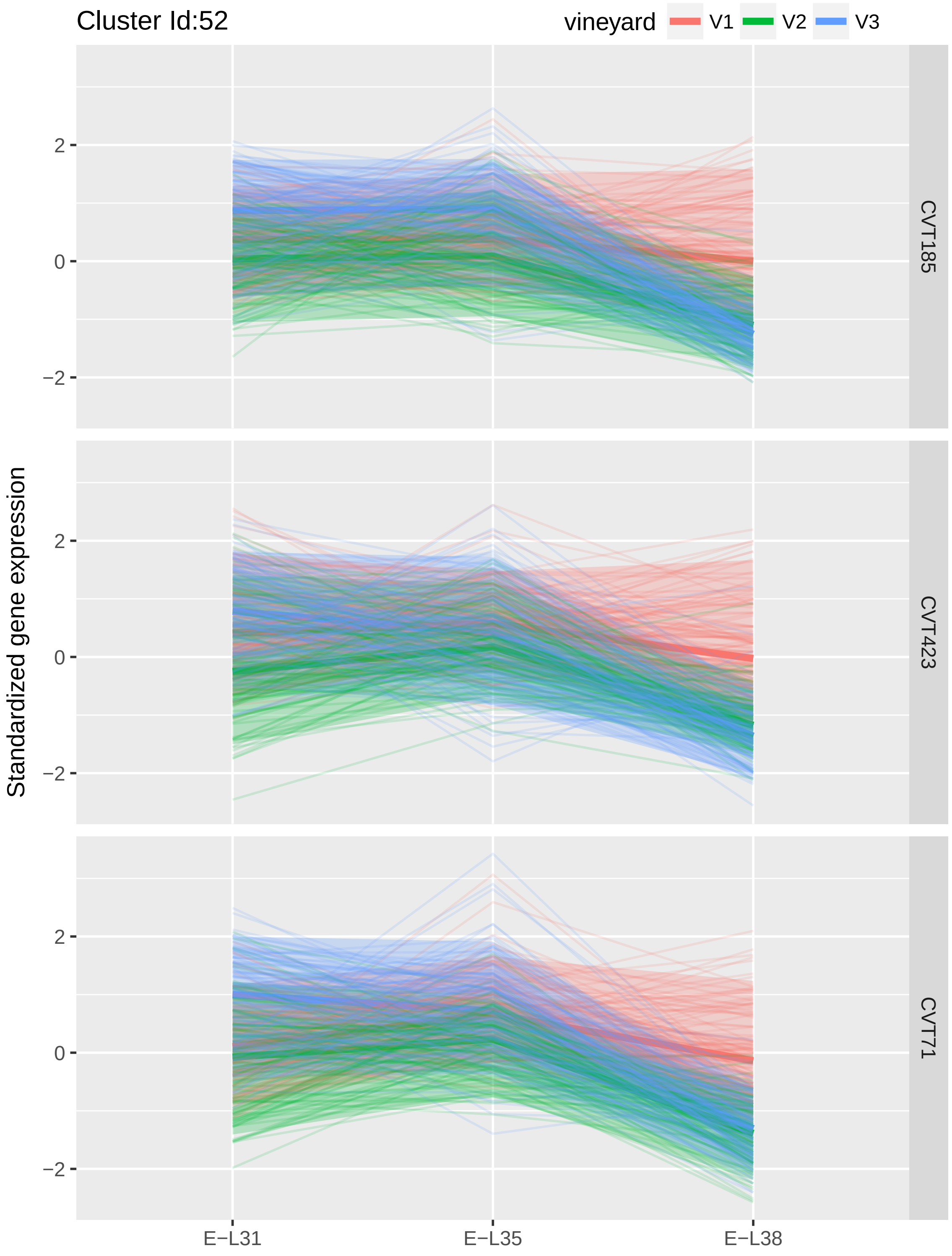

Cluster Id:52
vineyard
V1
V2
V3
Standardized gene expression
2
0
−2
CVT185
CVT423
CVT71
E−L31
E−L35
E−L38

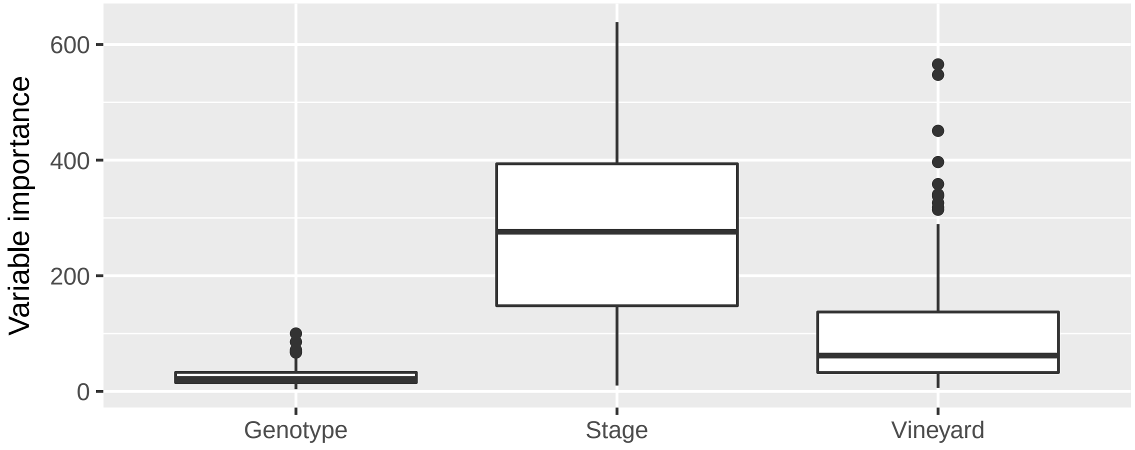

Variable importance
600
400
200
0
Genotype
Stage
Vineyard

Cluster no. 53

```
## Number of genes in the cluster: 70
## Homogeneity Index:    0.67
## Variable importance for Stage:    Median =  293.2  - Rank =  72
## Variable importance for Clone:    Median =  28.18  - Rank =  11
## Variable importance for Vineyard: Median =  38.03  - Rank =  51
##
## Gene ID              Gene Annotation
## VIT_06s0004g07440  -  Exocyst subunit EXO70 family protein B1
## VIT_13s0156g00340  -  DNA-binding bromodomain-containing protein
## VIT_15s0046g02240  -  Unknown protein
## VIT_04s0023g01790  -  phytoene dehydrogenase-related (PDH) (VvPDH2)
## VIT_02s0025g02050  -  Unknown
## VIT_07s0129g00860  -  CYP81E1 Isoflavone 2'-hydroxylase
## VIT_16s0098g01890  -  Phosphatidylinositol 3- and 4-kinase / ubiquitin
## VIT_17s0000g04970  -  ADP, ATP carrier protein
## VIT_10s0003g03980  -  LEM3 (ligand-effect modulator 3) family protein
## VIT_04s0008g00860  -  Ndr family protein
## VIT_18s0001g08150  -  Xylan synthase
## VIT_06s0009g02340  -  Tryptophan/tyrosine permease family
## VIT_00s0187g00280  -  Cellulase
## VIT_18s0122g00090  -  Transporter-related
## VIT_18s0072g00080  -  Glucose-6-phosphate/phosphate translocator
## VIT_16s0050g02480  -  ABC transporter C member 15 (VvABCC1)
## VIT_16s0039g02740  -  NADH glutamate dehydrogenase [Vitis vinifera]
## VIT_18s0001g00120  -  Transducin protein
## VIT_00s0404g00020  -  MAPK activating protein C22orf5
## VIT_01s0146g00060  -  Glucose-6-phosphate 1-dehydrogenase 2, chloroplast precursor
## VIT_18s0001g02970  -  Glucose-6-phosphate/phosphate translocator related
## VIT_00s0525g00050  -  Metal transporter CNNM4 (Cyclin-M4)
## VIT_05s0020g03610  -  Unknown protein
## VIT_17s0000g08790  -  EMB2759 (embryo defective 2759)
## VIT_12s0034g02510  -  Unknown
## VIT_18s0001g03230  -  Aminoacylase/ metallopeptidase
## VIT_13s0064g01230  -  U6 snRNA-associated Sm-like protein LSm7
## VIT_14s0060g01010  -  basic helix-loop-helix (bHLH) family
## VIT_04s0008g04270  -  Protein phosphatase 2 (formerly 2A), regulatory subunit B'
## VIT_08s0056g00640  -  Transposon protein, CACTA, En/Spm sub-class
## VIT_04s0008g06920  -  1,2-diacylglycerol 3-beta-galactosyltransferase
## VIT_16s0098g01620  -  Synbindin TRAPPC1
## VIT_06s0004g02820  -  Sand family
## VIT_12s0028g00470  -  Component of oligomeric golgi complex 5
## VIT_07s0129g00170  -  Porin
## VIT_05s0020g00390  -  Unknown protein
## VIT_11s0016g00440  -  Phosphoinositide phosphatase
## VIT_03s0063g00060  -  IMP dehydrogenase
## VIT_18s0001g10750  -  Unknown protein
## VIT_05s0094g01630  -  Apyrase (APY1)
## VIT_11s0118g00810  -  Peptidyl-prolyl cis-trans isomerase
## VIT_18s0001g12950  -  SEC14 cytosolic factor
## VIT_11s0118g00410  -  ANTR2 (anion transporter 2
## VIT_01s0011g01570  -  Coatomer subunit delta
## VIT_01s0011g03580  -  Unknown protein
## VIT_04s0023g00370  -  Zinc finger CCCH domain-containing protein ZFN1
## VIT_10s0116g00760  -  Glucose-6-phosphate/phosphate translocator
```

```
## VIT_01s0011g06540  -  Endomembrane protein 70
## VIT_07s0104g00570  -  BTB-POZ and math Domain 2 ATBPM2
## VIT_04s0008g02320  -  Coatomer subunit beta
## VIT_14s0060g01750  -  Serine/threonine-protein phosphatase PP1
## VIT_07s0129g00180  -  Ca2+-ATPase 2 ACA2, plasma membrane
## VIT_06s0080g00510  -  Sec23/sec24 transport family
## VIT_18s0072g00460  -  Transcription elongation factor
## VIT_17s0000g03090  -  R protein disease resistance protein
## VIT_02s0025g04410  -  Beta-ketoacyl-CoA synthase
## VIT_00s0264g00060  -  Unknown
## VIT_05s0020g01910  -  1,4-alpha-D-glucan maltohydrolase
## VIT_06s0004g04200  -  RAB GTPase ARA3
## VIT_08s0007g02040  -  No hit
## VIT_03s0063g02450  -  Coatomer subunit gamma
## VIT_01s0011g00050  -  Heat shock protein binding
## VIT_17s0000g07960  -  Ubiquitin-conjugating enzyme E2 H
## VIT_00s0340g00080  -  Component of oligomeric golgi complex 1
## VIT_07s0129g00110  -  Ca2+-ATPase 2 ACA2, plasma membrane
## VIT_02s0025g01020  -  Basic Leucine Zipper Transcription Factor (VvbZIP03)
## VIT_03s0091g01040  -  No hit
## VIT_18s0001g07600  -  Unknown protein
## VIT_16s0100g00640  -  Rhomboid ATRBL2
## VIT_17s0000g01380  -  Ubiquitin-activating enzyme E1 domain-containing protein 1
```

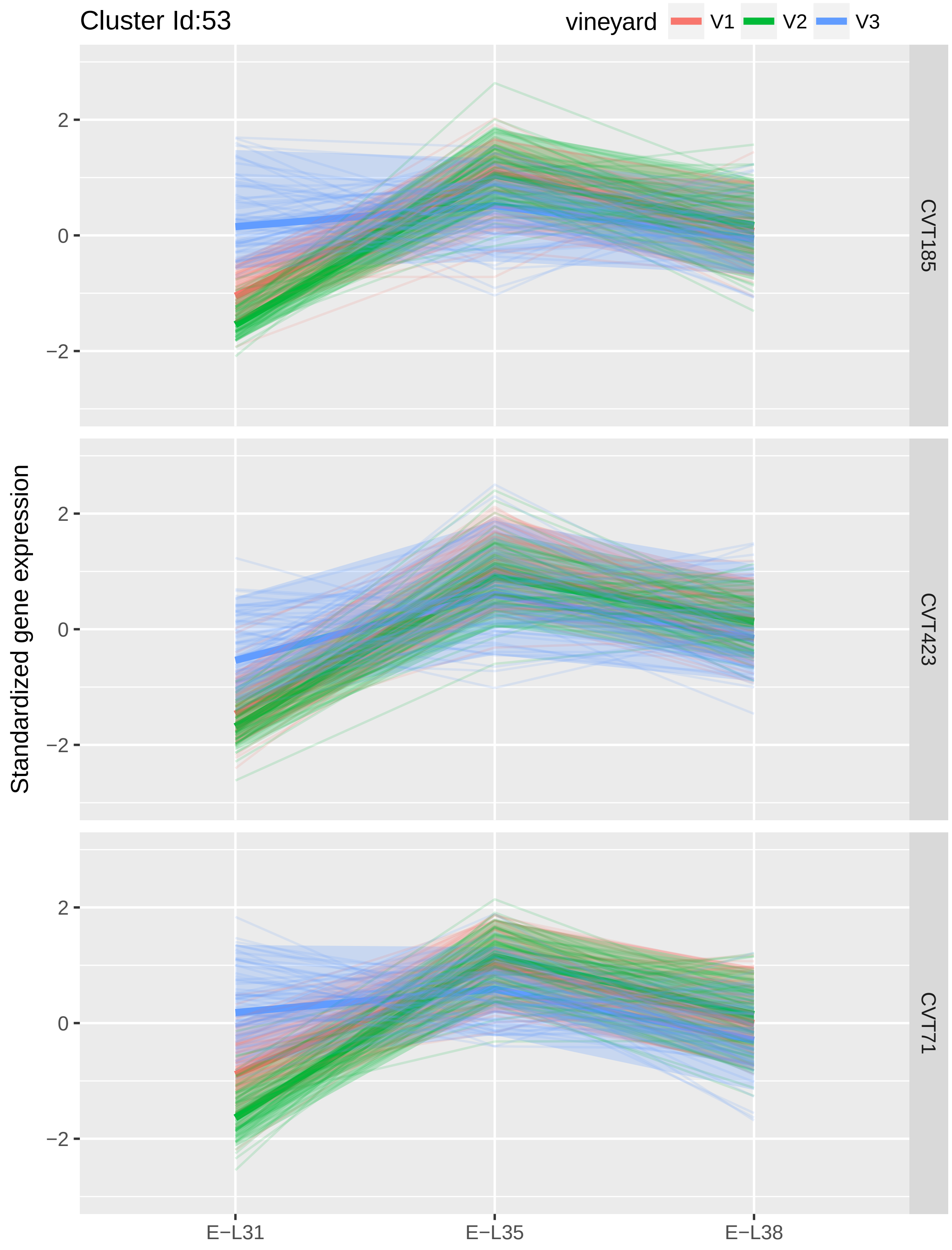
Cluster Id:53
vineyard
V1
V2
V3
Standardized gene expression
2
0
−2
CVT185
CVT423
CVT71
E−L31
E−L35
E−L38

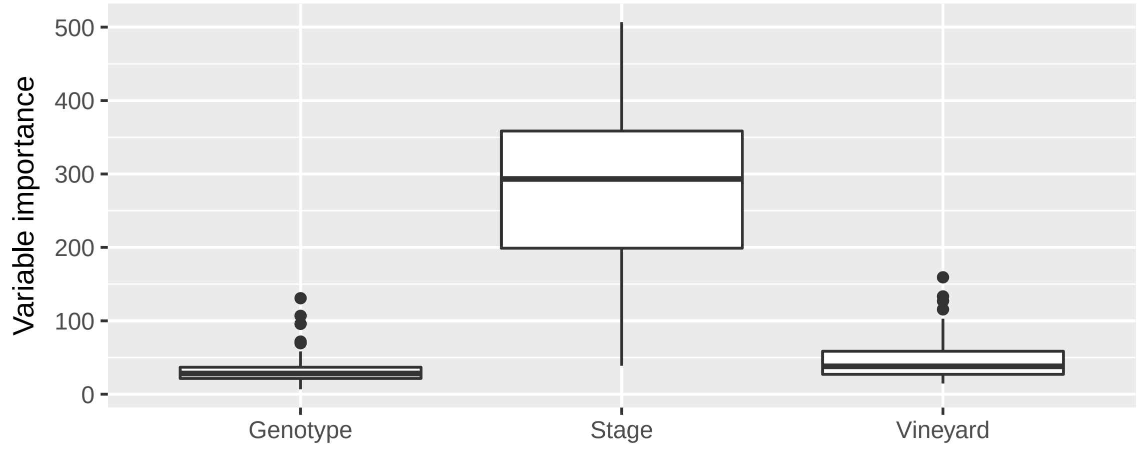
Variable importance
500
400
300
200
100
0
Genotype
Stage
Vineyard

Cluster no. 54

```
## Number of genes in the cluster: 202
## Homogeneity Index:    0.63
## Variable importance for Stage:    Median =  249.5  - Rank =  79
## Variable importance for Clone:    Median =  27.15  - Rank =  14
## Variable importance for Vineyard: Median =  42.82  - Rank =  44
##
## Gene ID               Gene Annotation
## VIT_11s0037g01130  -  TET8 (tetraspanin8)
## VIT_19s0085g00500  -  Peroxisomal 2,4-dienoyl-CoA reductase
## VIT_14s0030g00650  -  Shikimate dehydrogenase
## VIT_03s0063g01960  -  Non-symbiotic hemoglobin class 1
## VIT_07s0005g03350  -  Malate dehydrogenase, cytosolic
## VIT_05s0094g00200  -  Chitinase class IV
## VIT_09s0002g04170  -  Acyl-CoA synthetases (Acyl-activating enzyme 13)
## VIT_02s0025g04490  -  UDP-galactose transporter 2 ATUTR2/UTR2
## VIT_15s0046g02110  -  Late embryogenesis abundant protein Lea14-A
## VIT_19s0085g01000  -  RGLG2 (ring Domain LIGASE2)
## VIT_18s0001g09940  -  VFB1 (VIER F- box  proteinE 1)
## VIT_05s0049g00940  -  Inorganic phosphate transporter 1-4
## VIT_07s0005g01670  -  No hit
## VIT_18s0001g05380  -  TTL1 (tetratricopetide-repeat thioredoxin-like 1)
## VIT_08s0007g03440  -  Hydrolase, alpha/beta fold
## VIT_14s0068g01340  -  No hit
## VIT_04s0008g07110  -  Aspartic Protease (VvAP6)
## VIT_11s0016g02850  -  Phosphofructokinase
## VIT_18s0001g11250  -  R protein MLA10
## VIT_18s0001g10180  -  N-hydroxycinnamoyl/benzoyltransferase 3
## VIT_08s0007g06030  -  Beta 1-3 glucanase [Vitis vinifera]
## VIT_17s0000g05380  -  Dynein light chain LC8-type
## VIT_15s0024g00800  -  No hit
## VIT_13s0067g01970  -  Laccase
## VIT_11s0037g00070  -  Aldehyde Dehydrogenase (VvALDHI1B1)
## VIT_11s0118g00360  -  Leucoanthocyanidin dioxygenase
## VIT_13s0064g01220  -  Zf A20 and AN1 domain-containing stress-associated protein 5
## VIT_08s0007g02600  -  Unknown protein
## VIT_13s0067g03220  -  Unknown protein
## VIT_16s0098g01850  -  Seven in absentia SINA1
## VIT_14s0036g00420  -  Phosphoenolpyruvate carboxylase kinase
## VIT_12s0057g00520  -  Serine/threonine-protein kinase WNK3
## VIT_18s0089g01010  -  Nitrate transporter NTL1
## VIT_08s0007g04570  -  UGT73D1 (UDP-glucosyl transferase 73D1); UDP-glycosyltransferase
## VIT_17s0000g09650  -  Glyoxal oxidase
## VIT_08s0007g04460  -  Unknown protein
## VIT_05s0049g00180  -  IBR10 (indole-3-butyric acid response 10); catalytic/ dodecenoyl-CoA d
## VIT_12s0034g01140  -  Plastocyanin domain-containing protein
## VIT_03s0017g01220  -  Polyadenylate-binding protein 2
## VIT_16s0050g00870  -  Acyltransferase
## VIT_05s0094g01590  -  Kelch repeat-containing F-box protein
## VIT_13s0074g00150  -  Dihydrolipoamide S-acetyltransferase precursor
## VIT_08s0007g03260  -  Unknown protein
## VIT_13s0067g03100  -  Lung seven transmembrane receptor
## VIT_01s0011g05670  -  Nudix hydrolase 18
## VIT_03s0038g03930  -  No hit
## VIT_01s0011g04310  -  Zinc finger (C3HC4-type ring finger)
```

```
## VIT_08s0007g04840  -  Unknown protein
## VIT_17s0000g05960  -  Pectinesterase family
## VIT_11s0016g04720  -  Blight-associated protein p12 precursor
## VIT_03s0017g00830  -  2OG-Fe(II) oxygenase
## VIT_16s0022g00940  -  Pectinesterase PME3
## VIT_01s0011g04780  -  Unknown protein
## VIT_08s0007g05990  -  Beta 1-3 glucanase
## VIT_06s0009g03190  -  Unknown protein
## VIT_07s0151g00200  -  Quinone-oxidoreductase, Chloroplast
## VIT_03s0063g00400  -  Alpha-amylase / 1,4-alpha-D-glucan glucanohydrolase
## VIT_07s0289g00050  -  UDP-glucose:salicylic acid glucosyltransferase
## VIT_07s0031g01250  -  1-acylglycerone phosphate reductase
## VIT_05s0077g00030  -  Ammonium transporter 1, member 1 (AMT1.1)
## VIT_14s0066g01290  -  Exonuclease
## VIT_19s0085g00270  -  Unknown protein
## VIT_12s0059g00870  -  MAPKKK15
## VIT_15s0046g02230  -  Heavy-metal-associated domain-containing protein
## VIT_11s0016g00710  -  TIFY gene family (VvJAZ9)
## VIT_09s0002g04210  -  Receptor protein kinase PERK1
## VIT_12s0028g00710  -  4-hydroxyphenylpyruvate dioxygenase
## VIT_16s0050g00850  -  C2 domain-containing protein
## VIT_04s0023g00690  -  Protein phosphatase 2C
## VIT_00s0301g00030  -  Secretory carrier membrane protein (SCAMP3)
## VIT_18s0001g07090  -  Unknown protein
## VIT_11s0016g03740  -  Rho guanyl-nucleotide exchange factor ROPGEF8
## VIT_00s1338g00020  -  Protein transport protein Sec61 subunit alpha
## VIT_14s0060g01470  -  Calmodulin-binding protein
## VIT_06s0004g02560  -  Kiwellin Ripening-related protein grip22
## VIT_09s0002g06880  -  Beta-1,3-glucanase
## VIT_08s0007g00720  -  Ring-H2 finger C2A
## VIT_13s0019g04710  -  PUB17 (plant U- box 17)
## VIT_07s0005g03300  -  Inorganic phosphate transporter 1-4
## VIT_13s0019g02180  -  Tropinone reductase
## VIT_09s0002g03450  -  Taurine dioxygenase
## VIT_01s0010g03790  -  Nuclear transport factor 2B
## VIT_05s0049g00570  -  Unknown
## VIT_18s0001g09330  -  Unknown protein
## VIT_17s0000g05880  -  Calcineurin phosphoesterase
## VIT_18s0001g03250  -  Unknown protein
## VIT_04s0079g00600  -  Glutamate decarboxylase 1
## VIT_04s0079g00800  -  DJ-1 protein / protease
## VIT_05s0049g02210  -  Unknown protein
## VIT_00s0286g00130  -  S-locus lectin protein kinase
## VIT_06s0004g07850  -  Unknown protein
## VIT_10s0003g05630  -  Coatomer subunit epsilon
## VIT_01s0026g00280  -  Trehalose 6-phosphate synthase
## VIT_09s0002g06400  -  No hit
## VIT_13s0019g02360  -  Aldehyde Dehydrogenase (VvALDHI8B1)
## VIT_01s0011g06000  -  Vacuolar iron transporter 1
## VIT_02s0025g04350  -  C2 domain protein
## VIT_19s0014g01710  -  Sodium/calcium exchanger family protein
## VIT_00s0229g00190  -  Inositol 2-dehydrogenase like protein
## VIT_06s0004g03310  -  Hydrolase, alpha/beta fold
## VIT_04s0008g02760  -  Unknown protein
## VIT_06s0061g00590  -  Nodulin family protein
```

```
## VIT_11s0016g01870  -  Dihydrolipoamide S-acetyltransferase
## VIT_06s0004g05650  -  Unknown protein
## VIT_13s0047g00010  -  ZIFL2 (zinc induced facilitator-like 1)
## VIT_00s1491g00020  -  Lil3 protein
## VIT_00s0187g00300  -  fumarylacetoacetase
## VIT_10s0003g03730  -  Kelch repeat-containing F-box protein
## VIT_00s0203g00070  -  Myb domain protein 102
## VIT_01s0011g04760  -  myb domain protein 4 (VvMybC2-L1)
## VIT_06s0004g04530  -  Armadillo/beta-catenin repeat protein/U-box domain-containing
## VIT_06s0080g00250  -  Sialyltransferase
## VIT_04s0023g01180  -  Unknown protein
## VIT_07s0005g03360  -  Malate dehydrogenase, cytosolic
## VIT_06s0004g04660  -  No hit
## VIT_05s0020g01830  -  UPF0497 family
## VIT_06s0004g06350  -  Pleckstriny (PH) domain-containing protein
## VIT_19s0138g00140  -  ATSYTE/NTMC2T6.1/NTMC2TYPE6.1/SYTE
## VIT_19s0140g00210  -  SOUL heme-binding
## VIT_19s0177g00200  -  Plastocyanin domain
## VIT_04s0008g01390  -  RNA recognition motif (RRM)-containing protein
## VIT_00s0510g00050  -  Aspartic Protease (VvAP50)
## VIT_05s0077g01940  -  Acyl-CoA dehydrogenase (ibr3)
## VIT_14s0030g01590  -  Rubredoxin
## VIT_16s0098g01880  -  Dipeptidyl peptidase 8
## VIT_01s0026g02710  -  NAC domain-containing protein (VvNAC26)
## VIT_18s0001g09460  -  GASA5
## VIT_02s0236g00070  -  Unknown
## VIT_18s0001g00230  -  Unknown protein
## VIT_19s0014g03800  -  RANGAP2 (RAN GTPASE activating protein 2)
## VIT_02s0236g00130  -  SNF1-related protein kinase SRK2F (SnRK-5)
## VIT_00s0304g00070  -  Lipid-associated family protein
## VIT_08s0040g01060  -  Serine carboxypeptidase S10
## VIT_17s0053g00700  -  Sucrose synthase 2
## VIT_18s0122g01170  -  12-oxophytodienoate reductase 2
## VIT_06s0004g06970  -  Unknown protein
## VIT_08s0058g00870  -  Unknown protein
## VIT_03s0088g01260  -  TIP growth defective 1
## VIT_04s0023g00730  -  Aspartic Protease (VvAP9)
## VIT_01s0011g04950  -  Diadenosine 5',5'''-P1,P4-tetraphosphate hydrolase
## VIT_04s0044g00310  -  Nodulin MtN21 family
## VIT_14s0128g00720  -  Acyl-CoA synthetase long-chain member 7
## VIT_13s0019g04660  -  Amino acid permease
## VIT_12s0028g03310  -  Protein phosphatase 2C
## VIT_13s0047g00030  -  ZIFL1 (Zinc induced facilitator 1)
## VIT_09s0018g00620  -  Co-chaperone-curved DNA binding protein A
## VIT_17s0000g05030  -  Alpha-1,4-glucan-protein synthase 1
## VIT_00s1737g00010  -  Zinc finger (DHHC type)
## VIT_12s0057g00010  -  Diacylglycerol kinase
## VIT_05s0049g00740  -  No hit
## VIT_13s0019g04600  -  ABC Transporter (VvWBC15 - VvABCG15)
## VIT_09s0002g00740  -  COP1-interacting protein
## VIT_06s0009g00680  -  Pyridine nucleotide-disulphide oxidoreductase
## VIT_07s0104g00090  -  basic helix-loop-helix (VvMYC1)
## VIT_17s0000g06050  -  Ankyrin protein kinase
## VIT_09s0002g08430  -  Nodulin
## VIT_02s0025g03190  -  2-oxoglutarate-dependent dioxygenase
```

```
## VIT_06s0009g01090  -  Heat shock  HSP20 family protein
## VIT_17s0000g05720  -  No hit
## VIT_13s0067g01550  -  MRH1 (morphogenesis of root hair 1)
## VIT_10s0071g00330  -  GRAM domain-containing protein / ABA-responsive
## VIT_18s0072g01220  -  ABC Transporter (VvWBC28 - VvABCG28)
## VIT_00s2536g00010  -  Aspartic proteinase nepenthesin-1 precursor
## VIT_03s0132g00040  -  ATHVA22A (Arabidopsis thaliana HVA22 homologue A)
## VIT_11s0016g02940  -  Profilin 1
## VIT_06s0009g02780  -  Histone mRNA exonuclease 1
## VIT_06s0004g05660  -  Glycosyl transferase family 1 protein
## VIT_03s0132g00090  -  ATHVA22A (Arabidopsis thaliana HVA22 homologue A)
## VIT_06s0004g05380  -  Tropinone reductase
## VIT_12s0028g01190  -  ATPase, BadF/BadG/BcrA/BcrD-type
## VIT_12s0028g02380  -  Unknown protein
## VIT_00s0207g00200  -  Integral membrane protein, putative
## VIT_08s0007g02580  -  Ubiquitin-conjugating enzyme E2 variant
## VIT_13s0047g00040  -  ZIFL1 (Zinc induced facilitator 1)
## VIT_03s0132g00070  -  ATHVA22A (Arabidopsis thaliana HVA22 homologue A)
## VIT_06s0004g03770  -  Embryo-specific protein
## VIT_14s0068g00230  -  Spiral 1 like 2
## VIT_00s0204g00170  -  Lil3 protein
## VIT_14s0066g00900  -  Cysteine-rich receptor-like protein kinase 42
## VIT_08s0058g00680  -  Integral membrane protein, putative
## VIT_11s0016g05060  -  Choline/ethanolamine kinase
## VIT_09s0018g01650  -  ERF/AP2 Gene Family (VvAP2-06)
## VIT_18s0001g13180  -  Ubiquitin-conjugating enzyme E2 W
## VIT_18s0157g00190  -  Choline kinase
## VIT_06s0004g08110  -  No hit
## VIT_05s0102g00660  -  No hit
## VIT_16s0039g02000  -  Peptidyl-tRNA hydrolase 2
## VIT_17s0000g03230  -  Alpha-mannosidase
## VIT_18s0001g08120  -  Ubiquitin-like (UBL) post-translational modifier
## VIT_10s0003g01810  -  No hit
## VIT_14s0066g00120  -  Unknown protein
## VIT_10s0003g00390  -  Glutaredoxin
## VIT_03s0038g01650  -  ABA-responsive protein (HVA22a)
## VIT_19s0090g00660  -  Lipase GDSL
## VIT_07s0151g00490  -  NADH dehydrogenase (ubiquinone) Fe-S protein 4
## VIT_11s0016g05070  -  Choline/ethanolamine kinase
## VIT_01s0011g01990  -  Glycine decarboxylase
## VIT_07s0031g01530  -  Stress enhanced protein 2 (SEP2)
## VIT_07s0129g00520  -  Protein phosphatase 2C
## VIT_14s0030g01230  -  No hit
## VIT_10s0092g00230  -  Lesion simulating disease
## VIT_14s0030g01220  -  No hit
```

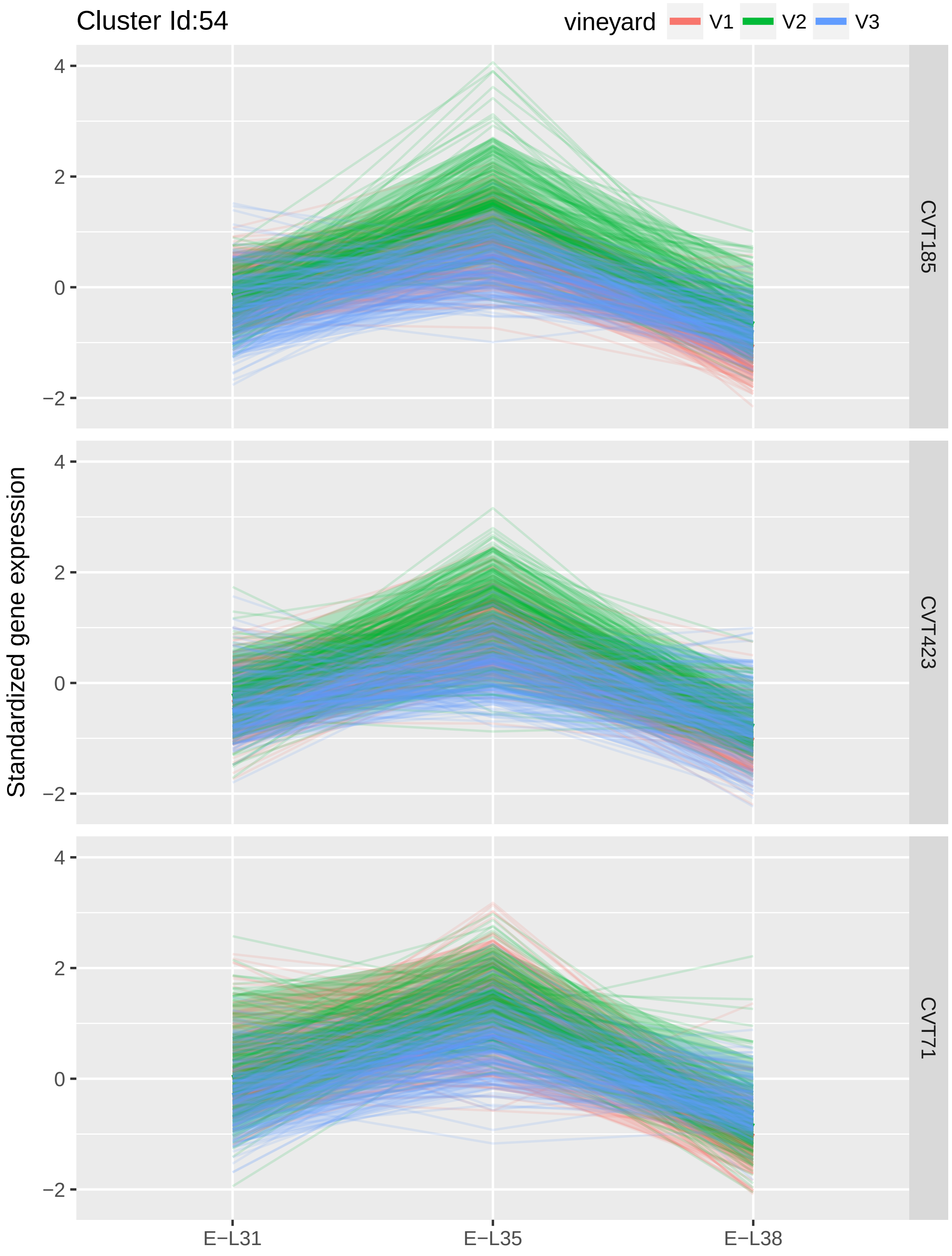

Cluster Id:54
vineyard
V1
V2
V3
Standardized gene expression
CVT185
CVT423
CVT71
4
2
0
−2
E−L31
E−L35
E−L38

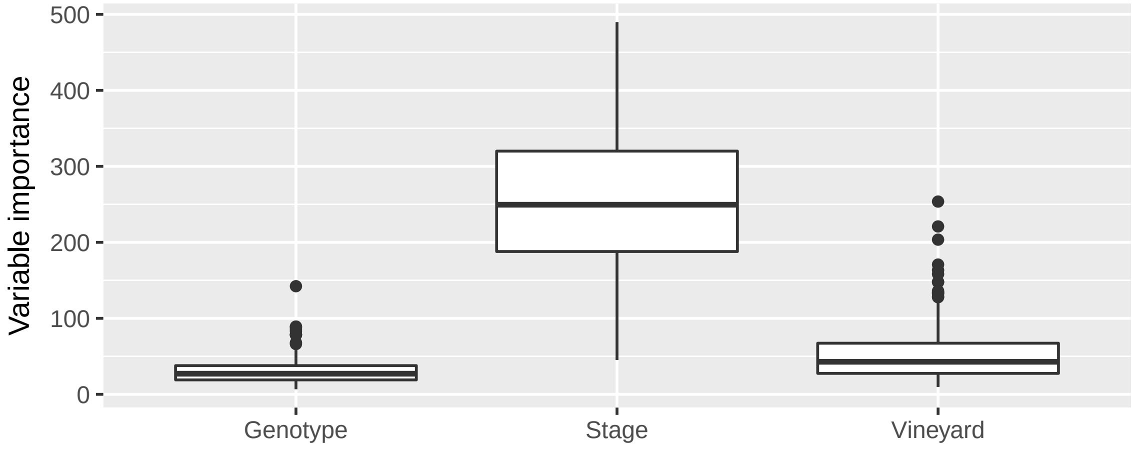

Variable importance
500
400
300
200
100
0
Genotype
Stage
Vineyard

Cluster no. 55

```
## Number of genes in the cluster: 57
## Homogeneity Index:    0.34
## Variable importance for Stage:    Median =  45.21  - Rank =  108
## Variable importance for Clone:    Median =  54.72  - Rank =  1
## Variable importance for Vineyard: Median =  80.76  - Rank =  21
##
## Gene ID               Gene Annotation
## VIT_07s0005g00680  -  Exocyst complex subunit Sec15B
## VIT_08s0032g00860  -  Unknown protein
## VIT_19s0014g00220  -  Mitogen-activated Protein Kinase (VvMPK12)
## VIT_07s0005g04420  -  Exostosin (Xyloglucan galactosyltransferase KATAMARI 1)
## VIT_06s0004g02810  -  DsRNA-binding protein 2 (DRB2)
## VIT_02s0025g02240  -  WD-40 repeat
## VIT_06s0009g00230  -  Unknown protein
## VIT_08s0007g08000  -  No hit
## VIT_06s0004g06750  -  Armadillo/beta-catenin repeat
## VIT_19s0090g00600  -  No hit
## VIT_18s0001g10470  -  Protein kinase MK6
## VIT_13s0064g00390  -  Polyamine oxidase
## VIT_01s0011g05280  -  Unknown protein
## VIT_01s0026g01880  -  Protein kinase APK1B
## VIT_08s0007g07990  -  No hit
## VIT_14s0006g01400  -  Calmodulin
## VIT_02s0025g04160  -  Unknown protein
## VIT_14s0060g02140  -  Rubber elongation factor (REF)
## VIT_15s0046g00750  -  Root hair initiation protein root hairless 1 (RHL1)
## VIT_05s0077g01500  -  Drought induced 19 protein
## VIT_18s0001g13600  -  No hit
## VIT_01s0011g01730  -  ATPP2-A9 (Phloem protein 2-A9)
## VIT_19s0015g00320  -  KCO1 (two pore K channel)
## VIT_06s0004g06640  -  PDX1 (pyridoxine biosynthesis 1.3)
## VIT_10s0003g03790  -  Jasmonate ZIM domain-containing protein (VvJAZ5)
## VIT_16s0050g02190  -  Unknown
## VIT_18s0001g13610  -  Abhydrolase domain-containing protein 5
## VIT_05s0020g01040  -  Unknown protein
## VIT_00s0187g00320  -  Cellulase
## VIT_01s0011g03110  -  myb family
## VIT_06s0004g06650  -  1-phosphatidylinositol-4,5-bisphosphate phosphodiesterase
## VIT_11s0103g00570  -  Microtubule-associated protein Rab GTPase
## VIT_01s0026g00820  -  IAA-amino acid hydrolase 1 (ILR1)
## VIT_11s0206g00130  -  VAMP-like protein YKT61
## VIT_01s0026g01890  -  UDP-galactose transporter 3
## VIT_16s0050g01870  -  CAAX amino terminal protease
## VIT_18s0001g11980  -  NAC domain-containing protein (VvNAC09)
## VIT_01s0026g01190  -  CW14
## VIT_00s0527g00020  -  VQ motif-containing protein
## VIT_00s0301g00090  -  NDA2 (alternative NAD(P)H dehydrogenase 2)
## VIT_01s0026g01450  -  DnaJ homolog, subfamily B, member 4
## VIT_04s0023g02950  -  Zinc finger (CCCH-type) family protein
## VIT_15s0021g00480  -  Dynamin-like protein 2b
## VIT_08s0058g00780  -  DISTORTED3/SCAR2
## VIT_18s0001g11890  -  E3 ubiquitin-protein ligase RNF5
## VIT_15s0046g02330  -  Unknown protein
## VIT_12s0034g02360  -  SEC6
```

```
## VIT_13s0158g00340  -  Glycerol-3-phosphate dehydrogenase
## VIT_01s0011g04860  -  Auxin transport protein (PIN3)
## VIT_10s0003g03800  -  Jasmonate ZIM domain-containing protein (VvJAZ6)
## VIT_10s0003g03300  -  Unknown protein
## VIT_16s0022g02080  -  Exostosin family protein
## VIT_03s0063g02420  -  Ras-related protein Rab-7A
## VIT_19s0177g00170  -  RabGAP/TBC domain-containing protein
## VIT_18s0001g09380  -  Syntaxin 6
## VIT_08s0007g07600  -  Pyruvate kinase, cytosolic isozyme
## VIT_13s0019g03990  -  Unknown protein
```

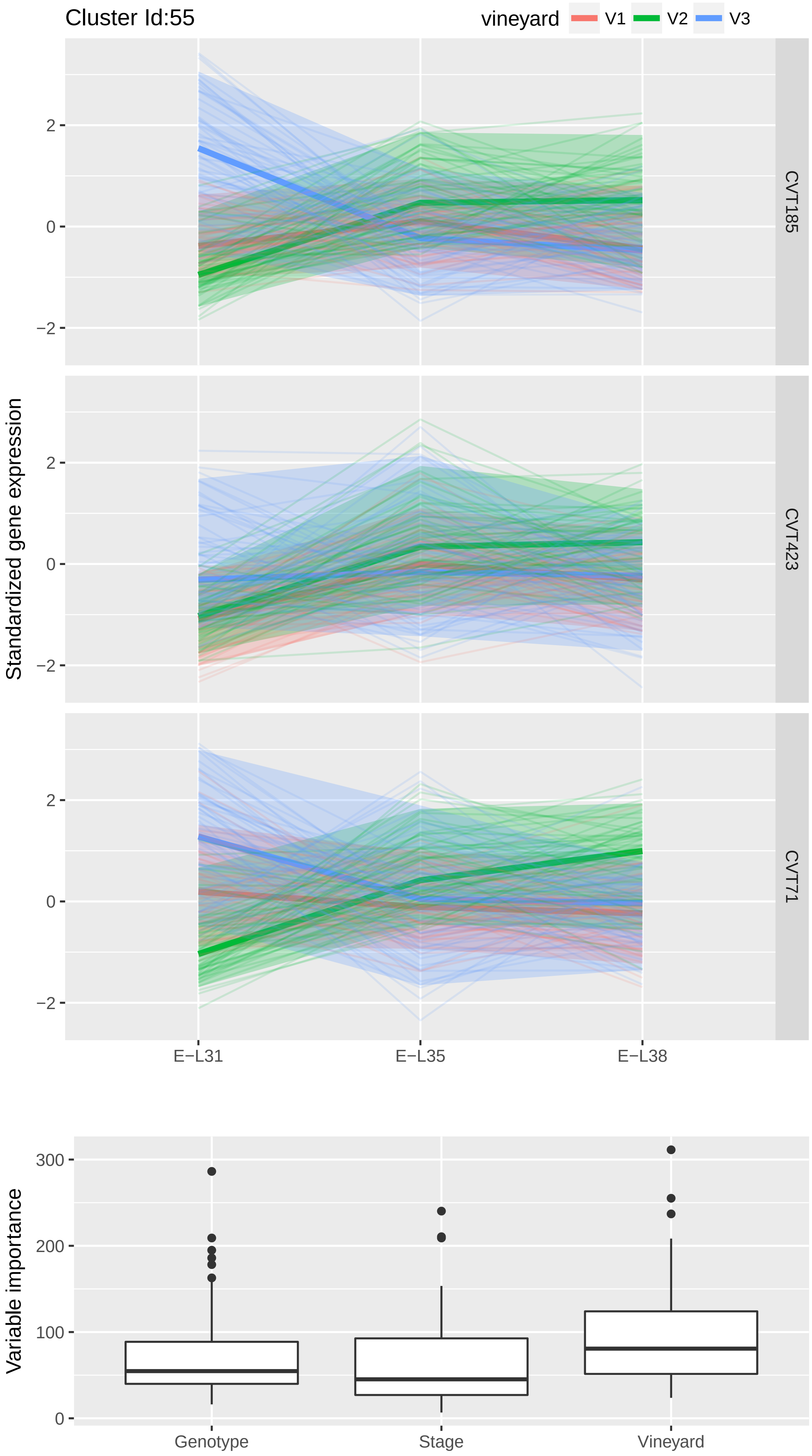

Cluster Id:55
vineyard
V1
V2
V3
Standardized gene expression
CVT185
CVT423
CVT71
2
0
−2
E−L31
E−L35
E−L38
Variable importance
300
200
100
0
Genotype
Stage
Vineyard

Cluster no. 56

```
## Number of genes in the cluster: 82
## Homogeneity Index:    0.71
## Variable importance for Stage:    Median =  519.6  - Rank =  30
## Variable importance for Clone:    Median =  17.36  - Rank =  65
## Variable importance for Vineyard: Median =   16.7  - Rank =  95
##
## Gene ID               Gene Annotation
## VIT_01s0146g00380  -  LITAF-domain-containing protein
## VIT_05s0020g03340  -  Unknown protein
## VIT_16s0098g01190  -  Ubiquitin A-52 residue ribosomal protein fusion product 1
## VIT_04s0023g00740  -  Thioredoxin F-type 2 (Trx-F2)
## VIT_07s0130g00290  -  Transposase, IS4
## VIT_13s0019g01750  -  Unknown protein
## VIT_08s0032g00310  -  Ubiquitin-conjugating enzyme E2 A
## VIT_04s0008g03990  -  Unknown
## VIT_16s0022g01590  -  Ribosomal protein S13 (RPS13A) 40S
## VIT_05s0102g01220  -  Ribosomal protein L38 (RPL38B) 60S
## VIT_10s0003g01300  -  Transcription factor
## VIT_05s0020g03920  -  Stress-induced-phosphoprotein 1
## VIT_00s0187g00340  -  fumarylacetoacetase
## VIT_04s0023g00930  -  Ribosomal protein L38 (RPL38B) 60S
## VIT_05s0094g01480  -  Dihydrodipicolinate reductase
## VIT_02s0025g05090  -  Microtubule motor PAK (phosphatidic acid kinase) KHC
## VIT_01s0026g01520  -  Guanine nucleotide exchange factor protein
## VIT_01s0026g01530  -  Homeobox protein 54
## VIT_00s1664g00010  -  TRNA/rRNA methyltransferase (SpoU)
## VIT_05s0062g01130  -  Aromatic and neutral transporter 1
## VIT_03s0088g01040  -  Aminotransferase AGD2
## VIT_05s0020g03450  -  Ribosomal protein S18 (RPS18C) 40S
## VIT_19s0090g00050  -  Pollen Ole e 1 allergen and extensin
## VIT_14s0066g00410  -  Aldose 1-epimerase
## VIT_05s0077g02290  -  Ribosomal protein L22-2 (RPL22B) 60S
## VIT_00s0301g00050  -  3'(2') 5'-bisphosphate nucleotidase
## VIT_19s0027g01380  -  Phenylalanine-tRNA synthetase
## VIT_05s0020g02990  -  Ribosomal protein S16 (RPS16C) 40S
## VIT_06s0004g07130  -  Cinnamoyl-CoA reductase
## VIT_04s0023g02800  -  Glutaredoxin
## VIT_12s0028g01090  -  Reticulon family protein (RTNLB5)
## VIT_18s0001g07860  -  TRAF-type zinc finger-related
## VIT_06s0004g00750  -  Acyl-CoA synthetases (Acyl-activating enzyme 18)
## VIT_13s0067g00270  -  Ribosomal protein P0 (RPP0A) acidic 60S
## VIT_09s0002g00470  -  ERF/AP2 Gene Family (VvERF059)
## VIT_03s0038g04240  -  Histone deacetylase
## VIT_17s0000g00780  -  Import inner membrane translocase subunit Tim8, Mitochondrial
## VIT_03s0017g00540  -  Subtilase
## VIT_04s0043g00440  -  Ribosomal protein S5 (RPS5B) 40S
## VIT_01s0150g00610  -  SnRK1-interacting protein 1
## VIT_18s0001g08750  -  Unknown
## VIT_14s0060g02110  -  Ribosomal protein S21 (RPS21C) 40S
## VIT_05s0077g02070  -  Ribosomal protein L18 (RPL18B) 60S
## VIT_04s0023g03210  -  Ribosomal protein L8 (RPL8C) 60S
## VIT_17s0000g00870  -  1-aminocyclopropane-1-carboxylate deaminase
## VIT_08s0007g03000  -  Chaperone protein dnaJ-related
## VIT_18s0122g00810  -  No hit
```

```
## VIT_11s0052g00540  -  ABC protein 3 ATNAP3 non-intrinsic
## VIT_14s0128g00490  -  DnaJ homolog, subfamily B, member 5
## VIT_19s0014g04760  -  Ribosomal protein L8 (RPL8C) 60S
## VIT_14s0128g00760  -  Epoxide hydrolase
## VIT_11s0206g00100  -  Unknown protein
## VIT_04s0008g06390  -  TPR1 (topless-related 1)
## VIT_06s0009g02350  -  Zinc finger (C3HC4-type ring finger)
## VIT_09s0070g00270  -  Ribosomal protein S17
## VIT_03s0038g04080  -  Glutamyl-tRNA synthetase
## VIT_14s0030g00030  -  Glutathione synthetase, chloroplast precursor
## VIT_17s0000g07340  -  Presenilin
## VIT_06s0004g00400  -  Lysyl-tRNA synthetase
## VIT_19s0015g01410  -  No hit
## VIT_06s0004g04800  -  Triosephosphate isomerase, cytosolic
## VIT_14s0066g02410  -  Aldehyde Dehydrogenase (VvALDH10B1)
## VIT_14s0036g00840  -  Endoribonuclease L-PSP
## VIT_07s0005g01400  -  Unknown protein
## VIT_14s0060g00630  -  Pyrrolidone-carboxylate peptidase
## VIT_04s0043g00140  -  Cadmium-induced protein AS8
## VIT_04s0008g06400  -  TPR1 (topless-related 1)
## VIT_04s0008g06380  -  TPR1 (topless-related 1)
## VIT_00s0299g00010  -  Acyl-CoA oxidase (ACX5)
## VIT_16s0022g01420  -  Unknown protein
## VIT_08s0040g02750  -  Ankyrin repeat
## VIT_06s0009g02410  -  Elongation factor 1-beta 1
## VIT_05s0049g01790  -  Ribosomal protein L23 (RPL23C) 60S
## VIT_14s0068g01440  -  Beta-ketoacyl-CoA synthase
## VIT_16s0022g01840  -  Clp amino terminal domain-containing protein
## VIT_07s0129g00160  -  Ribosomal protein L26 (RPL26A) 60S
## VIT_06s0080g00680  -  No hit
## VIT_07s0005g04700  -  Dihydroneopterin aldolase
## VIT_01s0011g04170  -  Phosphatidic acid phosphatase alpha
## VIT_11s0016g02170  -  FVE gene
## VIT_11s0016g04630  -  GAI protein.
## VIT_08s0007g07520  -  3-oxoacyl-[acyl-carrier-protein] reductase, chloroplast
```

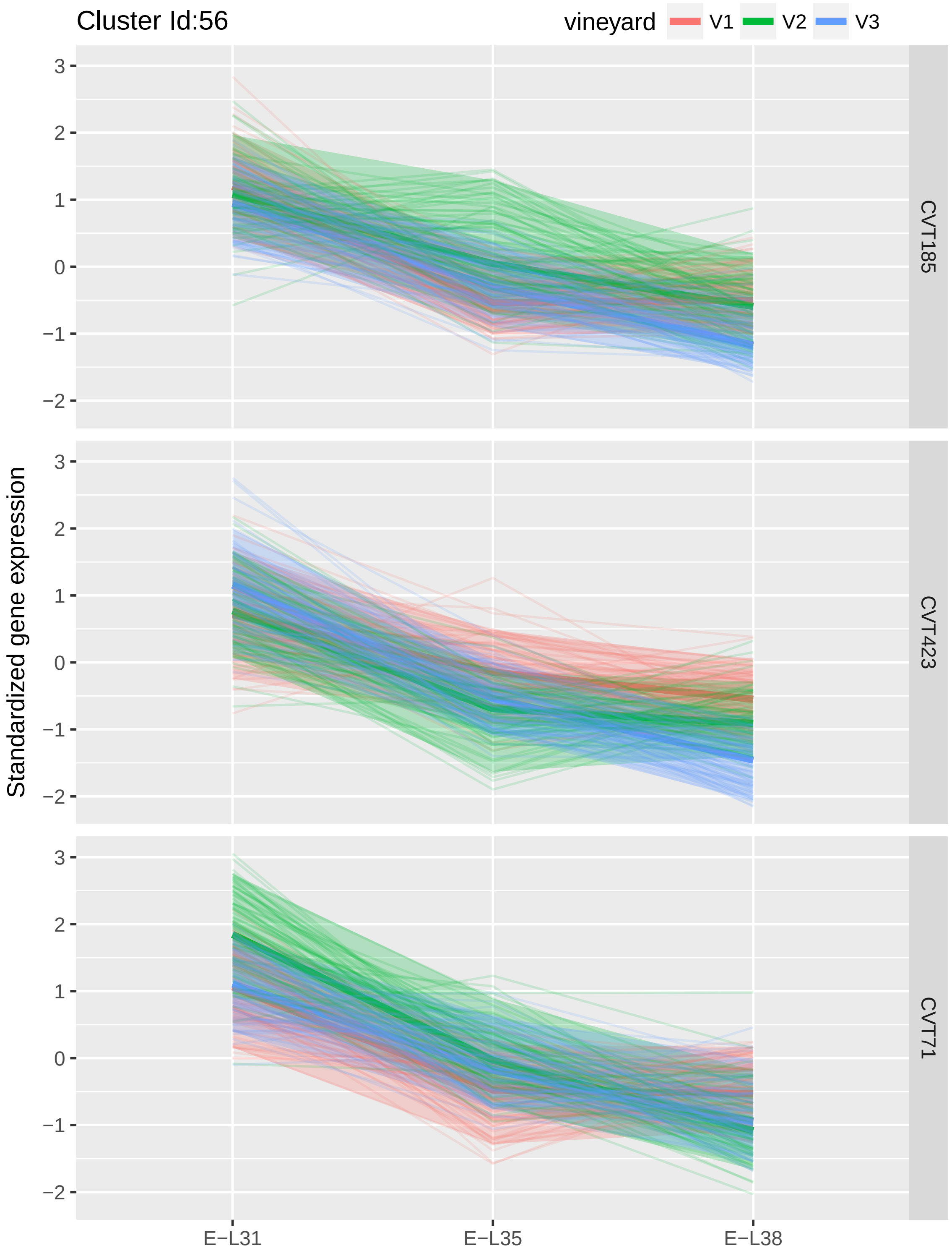

Cluster Id:56
vineyard
V1
V2
V3
Standardized gene expression
CVT185
CVT423
CVT71
3
2
1
0
−1
−2
E−L31
E−L35
E−L38

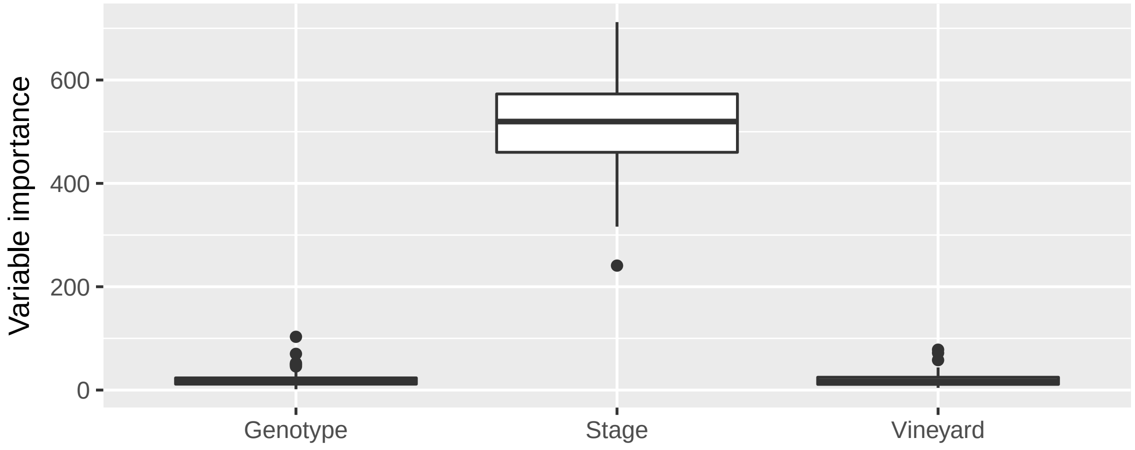

Variable importance
600
400
200
0
Genotype
Stage
Vineyard

Cluster no. 57

```
## Number of genes in the cluster: 134
## Homogeneity Index:    0.66
## Variable importance for Stage:    Median =  290.5  - Rank =  73
## Variable importance for Clone:    Median =  26.84  - Rank =  18
## Variable importance for Vineyard: Median =  41.91  - Rank =  45
##
## Gene ID               Gene Annotation
## VIT_09s0002g00690  -  DnaJ homolog, subfamily B, member 6
## VIT_10s0003g04650  -  Nuclear transcription factor Y subunit A-7
## VIT_01s0010g00740  -  basic helix-loop-helix (bHLH) family
## VIT_13s0139g00260  -  SKP1 interacting partner 5 (SKIP5)
## VIT_10s0003g00370  -  Guanylate-binding
## VIT_09s0002g08660  -  F-box protein (FBL14)
## VIT_05s0077g00940  -  Phytochrome B
## VIT_09s0002g03670  -  No hit
## VIT_09s0002g06510  -  Auxin-independent growth promoter axi1
## VIT_04s0008g03120  -  Protein arginine N-methyltransferase 5
## VIT_08s0058g00850  -  Gag-pol polyprotein
## VIT_00s0341g00060  -  Unknown protein
## VIT_07s0104g00900  -  Transducin LEC14B
## VIT_05s0094g00750  -  Stearoyl-CoA 9-desaturase
## VIT_07s0129g00140  -  Adenylylsulfate kinase
## VIT_06s0004g00700  -  Transcription initiation factor TFIID subunit D6
## VIT_04s0008g02840  -  Avirulence induced gene (AIG)
## VIT_18s0122g00260  -  Superkiller protein 3
## VIT_18s0001g07710  -  WD-40 repeat / beige
## VIT_08s0007g07350  -  Pyridoxamine 5'-phosphate oxidase
## VIT_02s0025g00270  -  Mitogen-activated Protein Kinase (VvMPK2)
## VIT_06s0004g06860  -  Clathrin heavy chain
## VIT_02s0012g00590  -  Unknown protein
## VIT_06s0004g07660  -  Alanine--glyoxylate aminotransferase 2 2, mitochondrial
## VIT_13s0067g03730  -  Unknown
## VIT_13s0067g00390  -  Metalloendopeptidase
## VIT_07s0104g01410  -  OTU cysteine protease
## VIT_18s0089g00610  -  Cytoplasmic membrane protein
## VIT_12s0035g01230  -  MEK kinase
## VIT_12s0028g00300  -  Amidase
## VIT_17s0000g07700  -  Glucose inhibited division protein B
## VIT_08s0058g01350  -  Non-green plastid inner envelope membrane protein
## VIT_01s0011g05360  -  High mobility group protein HMGI/Y-1
## VIT_08s0040g00670  -  4-alpha-glucanotransferase
## VIT_06s0004g00090  -  EMB1011 (embryo defective 1011)
## VIT_03s0063g01460  -  Cytochrome b6f complex subunit (petM)
## VIT_06s0004g05120  -  ARR1 typeB
## VIT_05s0020g01570  -  MORC family CW-type zinc finger 4
## VIT_00s0341g00050  -  ATMYB66/WER/WER1 (WEREWOLF 1)
## VIT_17s0000g04130  -  MYB divaricata
## VIT_07s0031g03150  -  S-glutathione dehydrogenase/class III alcohol dehydrogenase
## VIT_14s0068g01010  -  Unknown protein
## VIT_14s0068g01160  -  Cytokinin-repressed protein CR9
## VIT_07s0104g01550  -  Transcription termination factor mitochondrial mTERF
## VIT_00s1569g00020  -  Calcineurin B protein (VvCBL03)
## VIT_08s0032g00110  -  RNA recognition motif (RRM)-containing protein
## VIT_08s0007g07940  -  No hit
```

```
## VIT_03s0038g04100  -  3-hydroxy-3-methylglutaryl-coenzyme A reductase 3
## VIT_15s0048g02740  -  CBL-interacting protein kinase 9 (CIPK9)
## VIT_05s0049g02310  -  Biopterin transport-related protein BT1
## VIT_04s0008g04460  -  Molybdate transporter 1
## VIT_06s0061g01190  -  SWI/SNF matrix-associated regulator of chromatin sbfamily A mber 3 2
## VIT_03s0038g04000  -  Cysteine endopeptidase, papain-type (XCP1)
## VIT_00s0225g00110  -  Sel1 domain protein repeat-containing protein
## VIT_07s0255g00060  -  Prolyl oligopeptidase family
## VIT_14s0030g02200  -  Basic Leucine Zipper Transcription Factor (VvbZIP38)
## VIT_12s0034g01480  -  Disease resistance protein (NBS-LRR class) Rps1-k-2
## VIT_19s0014g00970  -  Unknown protein
## VIT_05s0094g01200  -  flavonoid 3'-hydroxylase cytochrome P450
## VIT_02s0025g03630  -  Cu2+-exporting ATPase HMA5 (heavy metal ATPase 5)
## VIT_16s0098g00670  -  Ribonuclease P/MRP protein subunit POP5
## VIT_10s0116g00840  -  Unknown
## VIT_12s0035g01240  -  No hit
## VIT_02s0033g00540  -  No hit
## VIT_00s0189g00090  -  Unknown
## VIT_06s0004g07170  -  Protein kinase PKN/PRK1
## VIT_08s0007g02310  -  Binding
## VIT_08s0007g05400  -  Unknown protein
## VIT_18s0041g01280  -  Adenylate kinase, chloroplast
## VIT_19s0014g03920  -  Ubiquitin-specific protease 14 (UBP14)
## VIT_07s0005g01230  -  Transcription initiation factor TFIID subunit D11
## VIT_04s0008g00900  -  MYB divaricata
## VIT_16s0050g01580  -  UDP-glucose: anthocyanidin 5,3-O-glucosyltransferase
## VIT_04s0023g03280  -  Zinc finger (Ran-binding)
## VIT_11s0052g00640  -  Nitrogen fixation NifU
## VIT_02s0012g00080  -  Casein kinase II subunit beta-4
## VIT_09s0002g03970  -  Unknown protein
## VIT_05s0020g00410  -  C2 domain-containing protein
## VIT_19s0090g00270  -  Unknown protein
## VIT_17s0000g05310  -  Pre-mRNA cleavage complex II protein Clp1
## VIT_19s0027g01680  -  Tetratricopeptide helical
## VIT_01s0026g00190  -  Armadillo/beta-catenin repeat
## VIT_18s0001g05100  -  No hit
## VIT_07s0255g00120  -  Unknown protein
## VIT_14s0068g00980  -  Unknown protein
## VIT_09s0054g00600  -  Pyridoxamine 5-phosphate oxidase
## VIT_03s0088g00300  -  Unknown
## VIT_15s0048g02380  -  DCL1 (DICER 1)
## VIT_00s1506g00010  -  RAB GTPase RAB2
## VIT_11s0052g00140  -  Mannosyl-oligosaccharide 1,2-alpha-mannosidase
## VIT_17s0000g07250  -  Accumulation of photosystem one 3 (APO3)
## VIT_13s0156g00210  -  Callose synthase
## VIT_15s0046g02650  -  Calmodulin-binding heat shock protein
## VIT_00s0274g00040  -  Pentatricopeptide (PPR) repeat membrane-associated salt-inducible prot
## VIT_14s0066g00170  -  CYP724B1
## VIT_19s0015g00250  -  Zinc transporter ZIP11
## VIT_00s1682g00020  -  flavonoid 3'-hydroxylase cytochrome P450
## VIT_08s0105g00450  -  Transducin protein
## VIT_15s0024g00350  -  BTAF1 RNA polymerase II, B-TFIID transcription factor-associated
## VIT_02s0025g04100  -  CCR4-NOT transcription complex subunit 2
## VIT_14s0066g01550  -  Aldehyde Dehydrogenase (VvALDH2B9)
## VIT_01s0026g02470  -  Zinc finger (C3HC4-type ring finger)
```

```
## VIT_14s0219g00140  -  ATP synthase mitochondrial F1 complex assembly factor 2
## VIT_00s1351g00020  -  No hit
## VIT_11s0052g00150  -  Mannosyl-oligosaccharide 1,2-alpha-mannosidase
## VIT_18s0001g13730  -  BPC6/BBR/BPC6/BPC6
## VIT_07s0104g00880  -  Arabidopsis histidine phosphotransfer AHP1
## VIT_17s0000g04900  -  ATP-dependent RNA helicase
## VIT_04s0008g06630  -  Phototropic-responsive NPH3
## VIT_14s0068g02070  -  Peptidase M48
## VIT_04s0023g01770  -  Adaptor-related protein complex 3, sigma 2 sub
## VIT_00s0455g00010  -  RAB GTPase RAB2
## VIT_06s0004g00500  -  Armadillo helical
## VIT_11s0016g05490  -  Actin 6 ARP6
## VIT_14s0030g01530  -  SF21
## VIT_12s0034g01050  -  No hit
## VIT_03s0038g02940  -  Transducin protein
## VIT_04s0023g01940  -  Anaphase-promoting complex, subunit 10 / APC10
## VIT_08s0007g05170  -  Basic Leucine Zipper Transcription Factor (VvbZIP27)
## VIT_03s0063g00600  -  Autophagy-related protein 2
## VIT_11s0016g00070  -  Zinc finger (C3HC4-type ring finger)
## VIT_09s0002g08160  -  R protein MLA10
## VIT_04s0008g03960  -  DNA polymerase theta subunit
## VIT_03s0063g01390  -  Phox (PX) domain-containing protein
## VIT_15s0046g03730  -  Digalactosyldiacylglycerol synthase
## VIT_01s0011g01620  -  ParB nuclease domain-containing protein
## VIT_07s0031g02300  -  Translation initiation factor eIF-6
## VIT_03s0063g00590  -  No hit
## VIT_13s0019g00910  -  Ubiquitin-conjugating enzyme 13 (UBC13), E2
## VIT_09s0002g04100  -  Vacuolar sorting protein 9
## VIT_18s0001g01090  -  Nucleic acid binding
## VIT_13s0067g01470  -  Unknown protein
## VIT_18s0001g14020  -  5'-3' exoribonuclease 2
## VIT_11s0016g03150  -  TRNA a64-2'-o-ribosylphosphate transferase
```

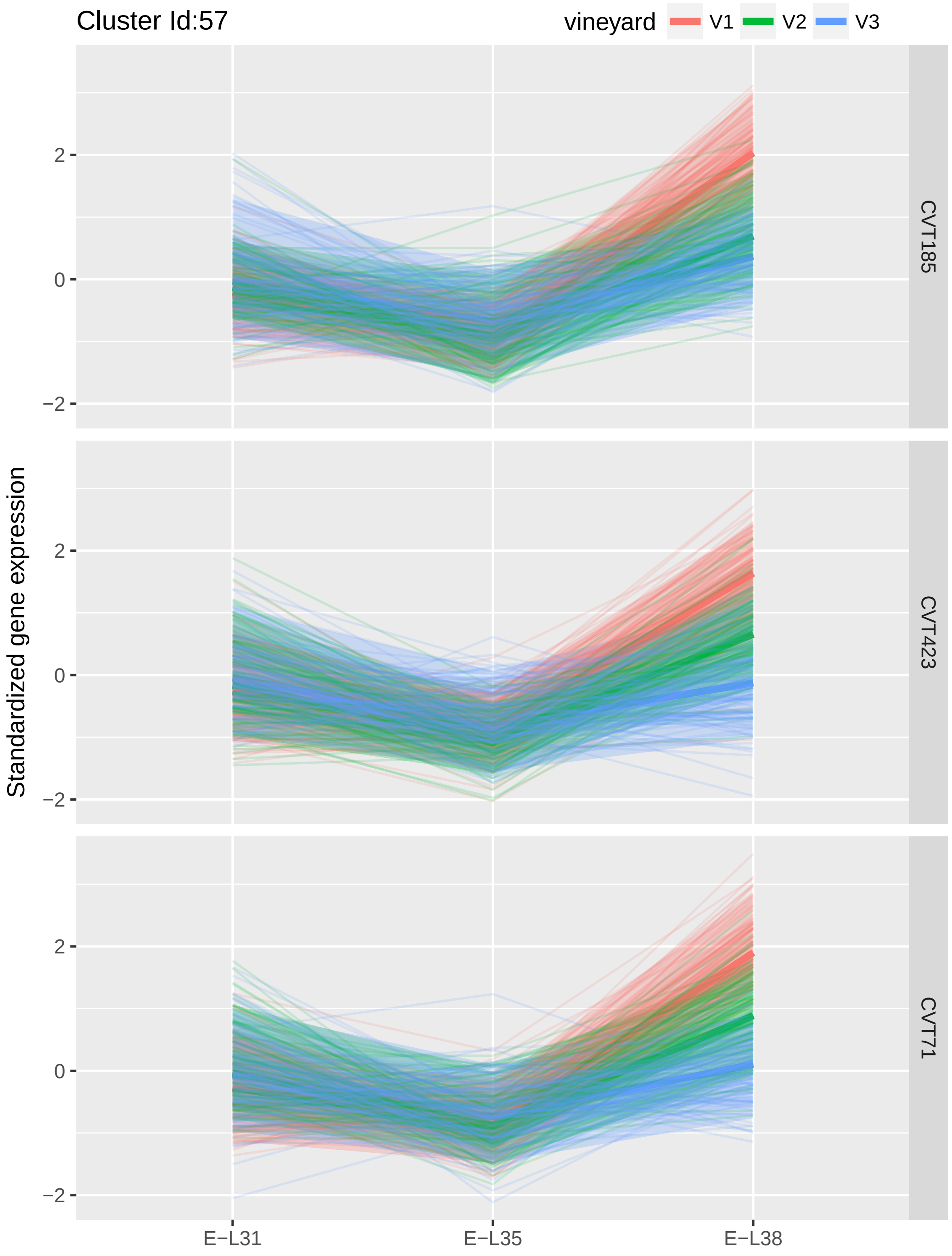

Cluster Id:57
vineyard
V1
V2
V3
CVT185
CVT423
CVT71
Standardized gene expression
2
0
−2
E−L31
E−L35
E−L38

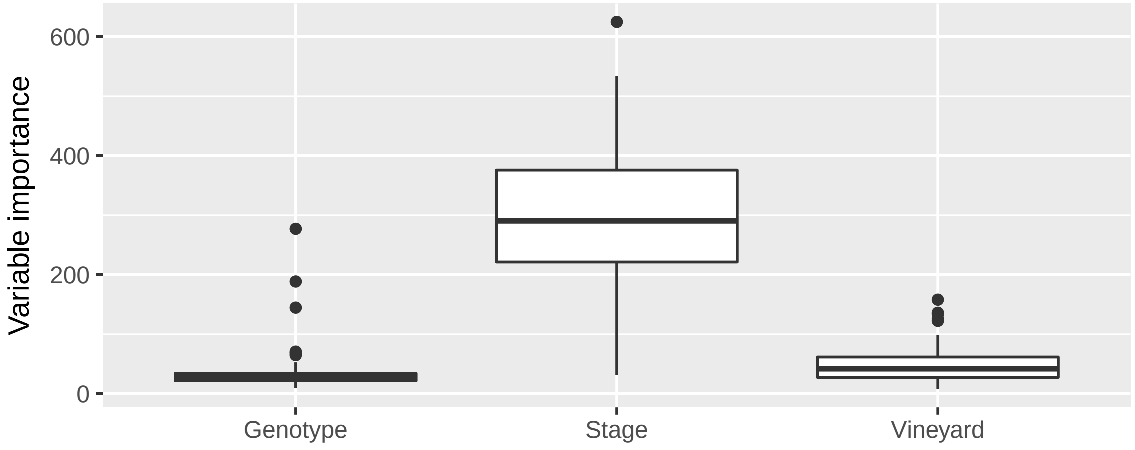

Variable importance
600
400
200
0
Genotype
Stage
Vineyard

Cluster no. 58

```
## Number of genes in the cluster: 199
## Homogeneity Index:    0.81
## Variable importance for Stage:    Median =  481.1  - Rank =  40
## Variable importance for Clone:    Median =  12.04  - Rank =  94
## Variable importance for Vineyard: Median =   26.9  - Rank =  72
##
## Gene ID               Gene Annotation
## VIT_15s0021g00320  -  No hit
## VIT_04s0044g01110  -  Alcohol dehydrogenase 6
## VIT_18s0001g15450  -  Alcohol dehydrogenase 3
## VIT_17s0000g06460  -  Zinc finger (C3HC4-type ring finger)
## VIT_10s0003g00990  -  Pyruvate decarboxylase isozyme 2
## VIT_11s0118g00390  -  Oxidoreductase, 2OG-Fe(II) oxygenase
## VIT_18s0001g02710  -  Unknown protein
## VIT_19s0014g03130  -  Stem-specific protein TSJT1
## VIT_14s0006g02720  -  Hexose transporter, Plastid
## VIT_18s0041g02020  -  12-oxophytodienoate reductase 2
## VIT_15s0021g01000  -  Ribosomal protein L37a (RPL37aB) 60S
## VIT_00s0762g00030  -  S-locus lectin protein kinase
## VIT_05s0102g00400  -  far-red impaired responsive family protein
## VIT_00s0762g00020  -  S-locus lectin protein kinase
## VIT_15s0046g01660  -  Unknown protein
## VIT_03s0063g01760  -  No hit
## VIT_02s0025g03460  -  Eukaryotic translation initiation factor
## VIT_01s0011g01360  -  Copper-transporting ATPase RAN1
## VIT_07s0104g00800  -  Indole-3-acetic acid-amido synthetase GH3.6
## VIT_08s0056g00550  -  ZIP family transporter
## VIT_11s0065g00730  -  F-box protein
## VIT_00s0370g00090  -  Vernalization independence 5 (VIP5)
## VIT_06s0004g08200  -  Ubiquitin-conjugating enzyme E2 D/E
## VIT_01s0010g00600  -  Phosphatidylinositol 4-kinase type-II
## VIT_13s0019g01090  -  Triosephosphate isomerase, cytosolic
## VIT_13s0047g01160  -  Ribosomal protein L13 (RPL13D) 60S
## VIT_00s0347g00080  -  Curculin (mannose-binding) lectin
## VIT_07s0031g01600  -  Shikimate kinase
## VIT_17s0000g05780  -  Unknown protein
## VIT_00s0868g00030  -  Unknown protein
## VIT_14s0060g01280  -  Pyrophosphate-energized vacuolar membrane proton pump
## VIT_04s0044g01700  -  RNA polymerase I specific transcription initiation factor RRN3
## VIT_01s0244g00110  -  Unknown protein
## VIT_18s0001g10830  -  Patatin
## VIT_17s0000g01470  -  SAP domain-containing protein
## VIT_14s0066g02060  -  AAA-type ATPase
## VIT_03s0017g02320  -  AAA-type ATPase
## VIT_07s0104g00720  -  Transcription termination factor mitochondrial mTERF
## VIT_04s0044g00750  -  S-receptor kinase
## VIT_13s0019g02680  -  Unknown
## VIT_06s0004g05070  -  Zinc transporter (ZIP2)
## VIT_18s0001g06950  -  Purine permease 1 (PUP1)
## VIT_19s0014g04980  -  Cinnamyl alcohol dehydrogenase
## VIT_01s0011g02080  -  Ribosomal protein L36a/L44 (RPL36aB) 60S
## VIT_04s0008g03400  -  ERF/AP2 Gene Family (VvERF035),Dehydration Responsive Element-Binding
## VIT_01s0150g00330  -  U-box domain-containing protein
## VIT_17s0000g05680  -  Band 7 family
```

```
## VIT_17s0000g01230  -  putative MADS-box TM8a (VviTM8a)
## VIT_19s0014g02240  -  Ethylene responsive element binding factor 4
## VIT_02s0087g00250  -  No hit
## VIT_05s0077g00520  -  Gibberellin 2-oxidase
## VIT_04s0044g00660  -  S-receptor kinase
## VIT_15s0048g02610  -  Ribosomal protein P1  acidic 60S
## VIT_14s0060g01300  -  Universal stress protein (USP) family protein
## VIT_19s0014g01180  -  Pathogenesis-related
## VIT_16s0013g02020  -  Ribosomal protein L15e
## VIT_03s0132g00350  -  Wall-associated receptor kinase-like 10
## VIT_00s0207g00170  -  Allergen V5/Tpx-1
## VIT_13s0067g01410  -  Anthranilate N-hydroxycinnamoyl/benzoyltransferase
## VIT_14s0081g00050  -  PATHOgenesIS-related 4
## VIT_12s0178g00020  -  ERGIC and golgi 3
## VIT_07s0005g02160  -  UBQ5 (ubiquitin 5)
## VIT_16s0050g01610  -  UDP-glycosyltransferase 88A4
## VIT_10s0003g05670  -  Pentatricopeptide (PPR) repeat-containing protein
## VIT_12s0028g01640  -  S-receptor kinase
## VIT_10s0003g01900  -  RKF1 (receptor-like kinase in flowers 1)
## VIT_15s0045g00380  -  Phosphoesterase
## VIT_17s0000g02560  -  No hit
## VIT_05s0094g00020  -  RWP-RK domain-containing protein
## VIT_18s0001g09510  -  CYP81B2v1
## VIT_13s0067g00010  -  Tafazzin
## VIT_16s0050g01650  -  Unknown
## VIT_16s0050g01900  -  Leucine-rich repeat protein kinase
## VIT_15s0021g00090  -  Protein binding
## VIT_07s0141g00640  -  ProT1 (PROLINE transporter 1)
## VIT_18s0001g11580  -  CYP82A3
## VIT_09s0002g00650  -  Auxin-induced protein
## VIT_12s0028g02340  -  Ketol-acid reductoisomerase
## VIT_16s0100g00450  -  Arginine/serine-rich splicing factor RSP41
## VIT_07s0104g01240  -  DegP2 protease
## VIT_14s0108g00100  -  Serine carboxypeptidase S10
## VIT_18s0001g12170  -  CYP72A1
## VIT_06s0004g04620  -  Unknown
## VIT_01s0150g00370  -  C2 domain-containing protein
## VIT_08s0007g02370  -  Unknown
## VIT_14s0068g00010  -  feronia receptor-like kinase
## VIT_12s0034g02230  -  UDP-glucose:glycoprotein glucosyltransferase
## VIT_18s0001g06840  -  Peroxidase GvPx2b class III
## VIT_07s0104g01360  -  Constans-like 13
## VIT_14s0066g02360  -  Seed maturation protein PM23
## VIT_13s0084g00430  -  Unknown
## VIT_00s2547g00010  -  WRKY DNA-binding protein 21
## VIT_03s0038g01780  -  Triosephosphate isomerase, chloroplast precursor
## VIT_16s0148g00330  -  Receptor kinase LRK10
## VIT_13s0067g00600  -  Arginine-tRNA-protein transferase 1
## VIT_13s0067g03140  -  WRKY Transcription Factor (VvWRKY41)
## VIT_14s0060g01320  -  Universal stress protein (USP) family protein
## VIT_08s0040g00540  -  VQ motif-containing protein
## VIT_01s0011g01110  -  Molybdenum cofactor sulfurase
## VIT_02s0087g00370  -  Anthranilate N-hydroxycinnamoyl/benzoyltransferase
## VIT_05s0049g01550  -  Peptide transporter protein 3
```

```
## VIT_12s0028g02960  -  No hit
## VIT_00s0258g00090  -  Brassinosteroid insensitive 1-associated receptor kinase 1
## VIT_02s0025g01690  -  Unknown protein
## VIT_16s0098g00950  -  Subtilase
## VIT_07s0129g00890  -  Protein kinase
## VIT_01s0127g00380  -  Pyrrolidone-carboxylate peptidase
## VIT_06s0061g00220  -  Transcription initiation factor TFIIB
## VIT_01s0026g00880  -  Transducin protein
## VIT_15s0045g00370  -  Phosphatidylglycerol specific phospholipase C
## VIT_13s0084g00130  -  CXE carboxylesterase
## VIT_06s0004g01110  -  Cation efflux family protein MTPc3
## VIT_04s0008g01480  -  NOL1/NOP2/sun family protein
## VIT_00s0225g00120  -  Unknown protein
## VIT_19s0014g01250  -  TUBBY like protein 7 TLP7
## VIT_13s0073g00680  -  Endonuclease/exonuclease/phosphatase family protein
## VIT_01s0127g00530  -  Protein disulfide isomerase
## VIT_00s0424g00020  -  Receptor serine/threonine kinase
## VIT_05s0077g01680  -  s2_Pathogenesis protein 10 [Vitis vinifera]
## VIT_01s0010g00710  -  Armadillo/beta-catenin repeat protein / U-box domain-containing protei
## VIT_04s0079g00610  -  Early nodulin ENOD18 protein
## VIT_08s0007g01360  -  Unknown protein
## VIT_08s0217g00100  -  Pyruvate decarboxylase isozyme 2
## VIT_13s0067g02130  -  Dehydration-induced protein (ERD15)
## VIT_17s0000g01460  -  Protein kinase AKIN gamma
## VIT_04s0008g04750  -  Monooxygenase
## VIT_14s0006g00640  -  DELLA protein RGL1 (RGA-like protein 1)
## VIT_08s0058g00590  -  Macrophage migration inhibitory factor
## VIT_16s0039g01310  -  Receptor serine/threonine kinase PR5K
## VIT_16s0098g00120  -  Polynucleotidyl transferase, Ribonuclease H fold
## VIT_12s0055g00200  -  UDP-glucose glucosyltransferase
## VIT_07s0129g00920  -  Receptor-like protein kinase
## VIT_06s0004g03220  -  Elongation factor 1-alpha 1
## VIT_01s0026g01040  -  Unknown protein
## VIT_18s0089g00510  -  Isopentenyltransferase 1
## VIT_07s0151g00340  -  Sulfate transporter 3.1 (AST12) (AtST1)
## VIT_00s0194g00020  -  DNA-directed RNA polymerase II subunit D
## VIT_04s0044g00720  -  S-receptor kinase
## VIT_10s0003g03150  -  Ribosomal protein L18A (RPL18aB) 60S
## VIT_12s0057g01280  -  Heterogeneous nuclear ribonucleoprotein A1/A3
## VIT_17s0000g05290  -  Pre-mRNA cleavage complex II protein Clp1
## VIT_18s0001g11470  -  CyP82A3
## VIT_08s0007g00070  -  RNA recognition motif (RRM)-containing protein
## VIT_13s0064g00270  -  Cinnamyl alcohol dehydrogenase
## VIT_07s0141g00020  -  Sensitive to proton rhizotoxicity 1
## VIT_14s0030g02220  -  Sec-independent protein translocase protein TatA
## VIT_00s0960g00010  -  Phosphosulfolactate synthase protein
## VIT_10s0092g00590  -  Leucine-rich repeat family
## VIT_08s0007g06300  -  RNA recognition motif (RRM)-containing
## VIT_06s0004g07650  -  Taxadien-5-alpha-ol-O-acetyltransferase
## VIT_07s0129g00530  -  Hydrolase, alpha/beta fold family
## VIT_08s0058g00970  -  Cationic peroxidase
## VIT_01s0137g00610  -  Sphere organelles protein
## VIT_18s0001g06650  -  basic helix-loop-helix (bHLH) family
## VIT_19s0014g02830  -  Whitefly-induced gp91-phox
## VIT_04s0043g00340  -  Myb KAN1 (KANADI 1)
```

```
## VIT_19s0014g02970  -  Choline transporter
## VIT_18s0001g01550  -  No hit
## VIT_03s0038g02740  -  Unknown protein
## VIT_08s0007g03040  -  E8 protein
## VIT_06s0004g05960  -  Unknown protein
## VIT_16s0148g00320  -  Ser/Thr receptor-like kinase1
## VIT_17s0000g05790  -  Cupin, RmlC-type
## VIT_04s0023g03450  -  AP endonuclease 2
## VIT_17s0000g02160  -  Unknown protein
## VIT_02s0025g03370  -  YDA (YODA)
## VIT_05s0020g00880  -  5-nucleotidase
## VIT_09s0002g05120  -  Zinc finger (C3HC4-type ring finger)
## VIT_04s0044g00120  -  Kinase
## VIT_14s0066g00760  -  Disease resistance protein (NBS-LRR class)
## VIT_16s0039g01330  -  Receptor kinase homolog LRK10
## VIT_03s0063g01930  -  DC1 domain-containing protein
## VIT_07s0005g04150  -  Ribosomal protein L23 (RPL23C) 60S
## VIT_07s0005g03060  -  1-aminocyclopropane-1-carboxylate oxidase homolog 1
## VIT_13s0019g05160  -  Pullulanase
## VIT_18s0001g11430  -  flavonoid 3-monooxygenase
## VIT_04s0023g01660  -  Scarecrow-like transcription factor 7 (SCL7)
## VIT_05s0124g00610  -  Ankyrin repeat protein family
## VIT_16s0100g00200  -  Unknown protein
## VIT_12s0028g03560  -  Unknown protein
## VIT_14s0006g00820  -  Ribosomal protein L13 (RPL13D) 60S
## VIT_03s0038g03410  -  NAC domain-containing protein (VvNAC41)
## VIT_02s0012g00540  -  Peroxidase
## VIT_18s0001g06940  -  Purine permease 1 (PUP1)
## VIT_06s0004g07150  -  Ribosomal protein S4 (RPS4D) 40S
## VIT_02s0025g04910  -  Growth-regulating factor 5
## VIT_04s0008g06220  -  Ribosomal protein S11 (RPS11C) 40S
## VIT_18s0001g06580  -  Plastocyanin domain-containing protein
## VIT_01s0026g01590  -  Non phototropic hypocotyl 1-like
## VIT_08s0056g00780  -  MATE efflux family protein
## VIT_04s0008g03150  -  S-adenosyl-L-methionine-dependent methyltransferase mraW
## VIT_13s0019g01200  -  Unknown
## VIT_04s0008g02090  -  Copper transporter 5
## VIT_03s0088g00340  -  No hit
## VIT_01s0011g01090  -  Unknown protein
## VIT_12s0034g01010  -  PYM (partner of Y14-MAGO)
## VIT_04s0044g00640  -  S-receptor kinase
## VIT_06s0080g00890  -  Unknown protein
## VIT_00s0294g00020  -  Kinase
```

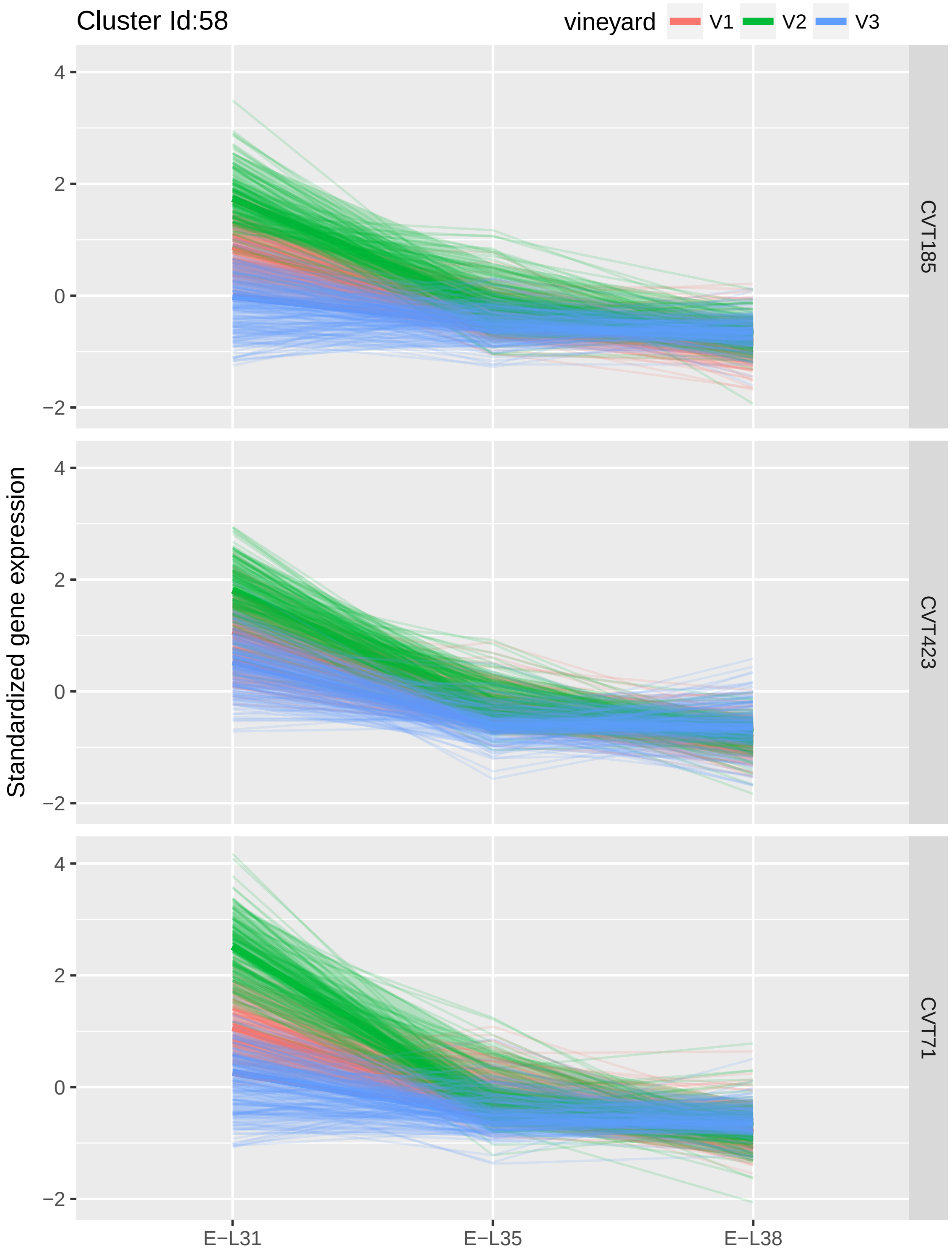

Cluster Id:58
vineyard
V1
V2
V3
Standardized gene expression
CVT185
CVT423
CVT71
4
2
0
−2
E−L31
E−L35
E−L38

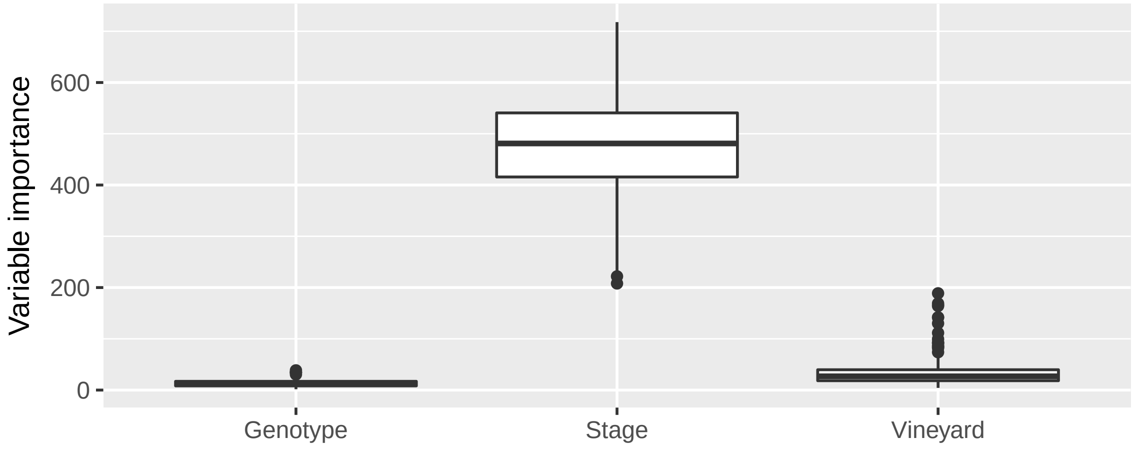

Variable importance
600
400
200
0
Genotype
Stage
Vineyard

Cluster no. 59

```
## Number of genes in the cluster: 45
## Homogeneity Index:    0.35
## Variable importance for Stage:    Median =  115.2  - Rank =  105
## Variable importance for Clone:    Median =  34.55  - Rank =  5
## Variable importance for Vineyard: Median =  135.6  - Rank =  11
##
## Gene ID                Gene Annotation
## VIT_00s1569g00010  -  Heat shock protein 81-2 (HSP81-2)
## VIT_00s0194g00030  -  Heat shock protein 81-2 (HSP81-2)
## VIT_00s2341g00010  -  Heat shock protein 81-4 (HSP81-4)
## VIT_18s0001g14500  -  SHD (shepherd)
## VIT_18s0001g11610  -  No hit
## VIT_11s0016g03070  -  Hexokinase-2
## VIT_18s0001g11600  -  Transducin protein
## VIT_13s0156g00070  -  DEAD box RNA helicase
## VIT_11s0016g02470  -  Carrier protein, Mitochondrial
## VIT_07s0129g01060  -  D-amino acid oxidase
## VIT_08s0058g01180  -  Protein kinase ATN1
## VIT_11s0016g03400  -  MSS1 (sugar transport protein 13)
## VIT_11s0016g01740  -  Zinc finger (C2H2 type) family
## VIT_01s0010g03330  -  Unknown
## VIT_18s0001g11070  -  No hit
## VIT_14s0060g00110  -  Glutamyl-tRNA synthetase, cytoplasmic
## VIT_16s0098g00660  -  Unknown protein
## VIT_09s0002g02930  -  RNA polymerase transcriptional regulation mediator
## VIT_08s0040g01870  -  Ribosomal protein L12 (RPL12C) 60S
## VIT_03s0038g01410  -  Aquaporin PIP PIP1A
## VIT_16s0022g00540  -  Glycerol 3-phosphate permease
## VIT_12s0028g00930  -  Glutathione S-transferase (VvGST3)
## VIT_01s0010g03320  -  Kelch repeat-containing F-box protein
## VIT_01s0146g00330  -  Hydroxyproline-rich glycoprotein family protein
## VIT_14s0068g00360  -  LNG1 (LONGIFOLIA1)
## VIT_12s0035g01060  -  Shaggy protein kinase kappa
## VIT_14s0006g01790  -  Beta-mannosidase
## VIT_04s0023g02880  -  GATA transcription factor 14
## VIT_15s0021g01220  -  Transcription factor
## VIT_06s0004g05030  -  FtsH protease that is localized to the mitochondrion
## VIT_01s0011g00720  -  WRKY Transcription Factor (VvWRKY01)
## VIT_17s0000g00800  -  Zinc finger (DHHC type)
## VIT_18s0001g14610  -  Clavata1 receptor kinase (CLV1)
## VIT_04s0044g00580  -  Actin 7 (ACT7) / actin 2
## VIT_14s0108g00520  -  Protease inhibitor/seed storage/lipid transfer protein (LTP)
## VIT_18s0086g00420  -  V-type H+-transporting ATPase 21kDa proteolipid subunit
## VIT_14s0066g02540  -  Acid phosphatase
## VIT_04s0008g01750  -  Ribosomal protein S3 (RPS3C) 40S
## VIT_07s0005g00700  -  Epoxide hydrolase
## VIT_04s0023g01430  -  Indeterminate(ID)-domain 2
## VIT_12s0035g01070  -  No hit
## VIT_00s0288g00060  -  No hit
## VIT_01s0127g00440  -  ADP-ribosylation factor A1B
## VIT_14s0171g00160  -  Adenylate kinase
## VIT_05s0020g01580  -  CBS
```

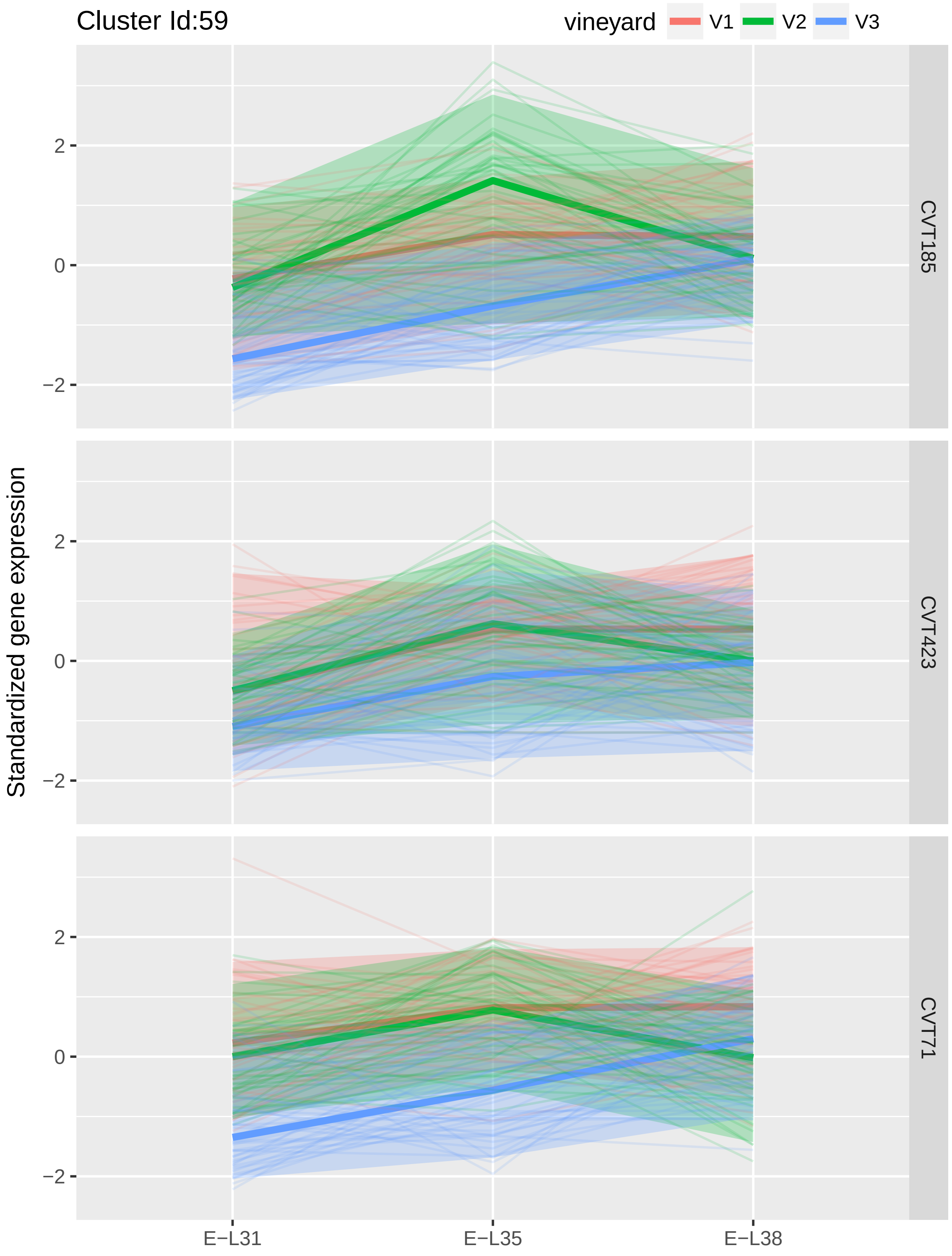
Cluster Id:59
vineyard
V1
V2
V3
Standardized gene expression
CVT185
CVT423
CVT71
2
0
−2
E−L31
E−L35
E−L38

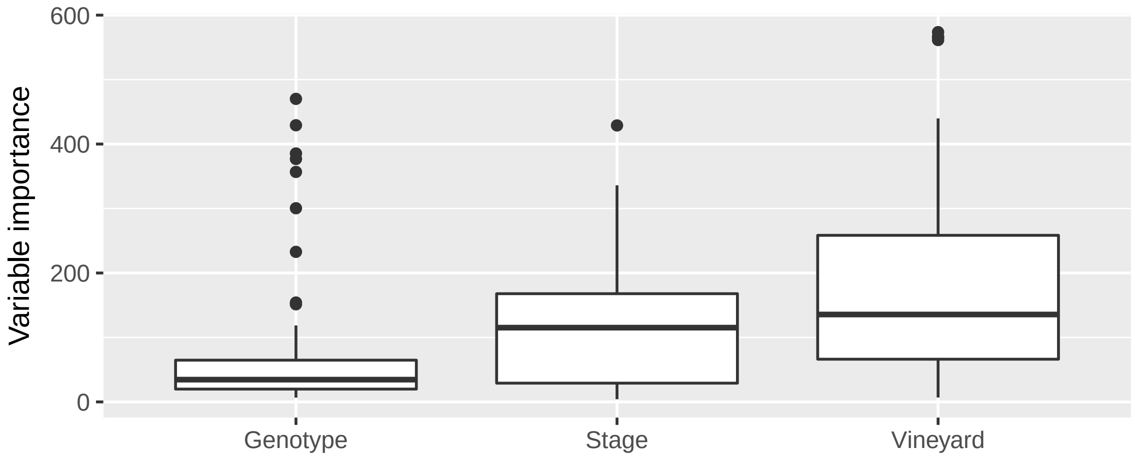
Variable importance
600
400
200
0
Genotype
Stage
Vineyard

Cluster no. 60

```
## Number of genes in the cluster: 130
## Homogeneity Index:    0.46
## Variable importance for Stage:    Median =  207.1  - Rank =  87
## Variable importance for Clone:    Median =  26.93  - Rank =  16
## Variable importance for Vineyard: Median =  67.37  - Rank =  28
##
## Gene ID               Gene Annotation
## VIT_02s0025g03670  -  Serine racemase
## VIT_18s0122g00240  -  Lys Motif-Type Receptor-Like Kinase LYK8
## VIT_14s0068g01240  -  Pentatricopeptide (PPR) repeat-containing
## VIT_07s0095g00640  -  Serine/threonine kinase BRLK
## VIT_18s0001g05400  -  Unknown
## VIT_12s0121g00060  -  R protein MLA10
## VIT_18s0001g05080  -  SEC14 cytosolic factor, putative
## VIT_19s0027g00520  -  Peptidase, trypsin serine and cysteine proteases
## VIT_13s0084g00180  -  DNA-directed RNA polymerase III subunit C2
## VIT_07s0151g00320  -  Pentatricopeptide (PPR) repeat-containing protein
## VIT_18s0122g01120  -  Pentatricopeptide repeat
## VIT_10s0003g05490  -  Toprim domain-containing protein
## VIT_14s0006g01390  -  Pentatricopeptide (PPR) repeat
## VIT_11s0016g02100  -  CRS1
## VIT_01s0011g05540  -  Unknown protein
## VIT_17s0000g04470  -  No hit
## VIT_18s0122g00670  -  Switching protein 3B ATSWI3B
## VIT_09s0002g08240  -  R protein MLA10
## VIT_04s0023g02720  -  RTE1 (reversion-to-ethylene sensitivity1)
## VIT_01s0010g02550  -  Cyclin-dependent kinase G-1
## VIT_12s0035g02090  -  Leucine-rich repeat family protein
## VIT_17s0000g04710  -  Pentatricopeptide (PPR) repeat-containing protein
## VIT_06s0004g01100  -  Embryo sac development arrest 3
## VIT_03s0091g00540  -  Prolyl 4-hydroxylase
## VIT_08s0007g01940  -  UDP-sulfoquinovose:DAG sulfoquinovosyltransferase
## VIT_14s0060g01100  -  Unknown protein
## VIT_06s0009g02170  -  CRR2 (chlororespiratory reduction 2)
## VIT_13s0019g00830  -  Armadillo repeat-containing protein
## VIT_01s0011g00630  -  Pentatricopeptide (PPR) repeat-containing
## VIT_12s0035g00420  -  Disease resistance protein
## VIT_18s0001g01350  -  Toprim domain-containing protein
## VIT_03s0038g02610  -  Monooxygenase (MO2)
## VIT_01s0010g00630  -  Pentatricopeptide (PPR) repeat-containing protein
## VIT_01s0011g06400  -  Indeterminate(ID)-domain 16
## VIT_07s0005g00570  -  Binding
## VIT_18s0001g09200  -  Ubiquitin system component Cue domain-containing protein
## VIT_13s0156g00620  -  S-receptor kinase
## VIT_06s0061g00120  -  Beta-1,3-glucanase [Vitis riparia]
## VIT_18s0001g13270  -  Papain cysteine peptidase XBCP3
## VIT_18s0001g07000  -  Pentatricopeptide (PPR) repeat-containing protein
## VIT_05s0020g02960  -  Homeotic gene regulator
## VIT_13s0084g00170  -  DNA-directed RNA polymerase III subunit C2
## VIT_16s0013g00200  -  Pentatricopeptide (PPR) repeat-containing protein
## VIT_17s0000g01150  -  DnaJ homolog, subfamily B, member 12
## VIT_15s0048g00870  -  AT-hook protein 1 (AHP1)
## VIT_13s0064g00980  -  Telomerase reverse transcriptase (TERT)
## VIT_15s0048g01830  -  Pentatricopeptide (PPR) repeat-containing
```

```
## VIT_18s0001g13220  -  Pentatricopeptide (PPR) repeat-containing protein
## VIT_18s0001g15560  -  Unknown protein
## VIT_01s0026g01130  -  Unknown protein
## VIT_07s0005g02720  -  No hit
## VIT_04s0008g03870  -  Pentatricopeptide (PPR) repeat-containing
## VIT_04s0044g00510  -  GT2-like trihelix DNA-binding protein
## VIT_05s0051g00130  -  Glutathione S-transferase 25 GSTU7
## VIT_04s0008g06470  -  Cysteinyl-tRNA synthetase
## VIT_09s0002g00080  -  Phytosulfokine receptor
## VIT_06s0061g00040  -  Hexokinase 6
## VIT_12s0057g01080  -  Kelch repeat-containing protein
## VIT_18s0001g06350  -  VQ motif-containing protein
## VIT_01s0011g03190  -  ATP-dependent Clp protease adaptor protein ClpS containing protein
## VIT_18s0041g00430  -  Patellin-1
## VIT_07s0005g01610  -  Unknown protein
## VIT_08s0007g01890  -  Unknown protein
## VIT_01s0026g01470  -  Glycine-rich protein
## VIT_15s0046g02120  -  EMB3001 (embryo defective 3001)
## VIT_12s0035g00890  -  Ion channel DMI1, chloroplast precursor
## VIT_08s0040g00920  -  Glutathione S-transferase 25 GSTU7
## VIT_15s0024g00240  -  C-5 cytosine-specific DNA methylase
## VIT_01s0010g03630  -  Zinc finger (MYND type)
## VIT_18s0122g00010  -  Peroxisomal membrane protein (PMP36)
## VIT_05s0051g00460  -  Leucine-rich repeat protein kinase
## VIT_06s0004g03410  -  Ribosomal protein S23 (RPS23B) 40S
## VIT_08s0056g01420  -  Polynucleotide phosphorylase
## VIT_05s0051g00140  -  RRS1 (resistant to ralstonia solanacearum 1)
## VIT_07s0005g00670  -  Unknown protein
## VIT_01s0011g00290  -  Unknown protein
## VIT_13s0064g00580  -  FK506-binding protein genes family (VvFKBP42)
## VIT_12s0059g01850  -  Peroxisomal membrane protein
## VIT_11s0016g00580  -  Unknown
## VIT_09s0002g04490  -  PDX1 (pyridoxine biosynthesis 1.3)
## VIT_03s0063g01430  -  Pentatricopeptide (PPR) repeat
## VIT_00s0317g00110  -  Esterase/lipase/thioesterase
## VIT_07s0031g02800  -  Transcription factor
## VIT_18s0117g00250  -  Pentatricopeptide repeat-containing protein
## VIT_11s0149g00090  -  Clavata1 receptor kinase (CLV1)
## VIT_19s0014g05220  -  Unknown protein
## VIT_14s0030g00960  -  R protein MLA10
## VIT_11s0016g00880  -  Strictosidine synthase; Soluble quinoprotein glucose dehydrogenase
## VIT_11s0016g00380  -  basic helix-loop-helix (bHLH) family
## VIT_11s0037g00860  -  Haemolysin-III related
## VIT_05s0020g01130  -  DEAD/DEAH box helicase
## VIT_08s0040g01440  -  TRNA (guanine-N(7)-)-methyltransferase
## VIT_07s0031g03010  -  Unknown protein
## VIT_02s0012g00190  -  Unknown protein
## VIT_04s0008g02390  -  Zinc finger (C3HC4-type ring finger)
## VIT_07s0151g00510  -  GCN5 N-acetyltransferase (GNAT)
## VIT_14s0060g00160  -  Nuclear pore complex protein Nup98-Nup96
## VIT_07s0151g00380  -  No hit
## VIT_14s0060g00170  -  Nuclear pore complex protein Nup98-Nup96
## VIT_14s0060g01450  -  3'-5' exoribonuclease
## VIT_14s0030g02190  -  FtsH protease
```

```
## VIT_19s0014g01260  -  Multiprotein-bridging factor 1a MBF1A
## VIT_00s0194g00070  -  Constans-like 10
## VIT_06s0004g07140  -  Maternal effect embryo arrest 67
## VIT_05s0102g00430  -  Unknown protein
## VIT_18s0001g08790  -  U-box domain-containing protein
## VIT_15s0107g00500  -  AMP-dependent synthetase and ligase
## VIT_19s0014g01270  -  Unknown
## VIT_16s0050g01140  -  Glutamine cyclotransferase
## VIT_02s0109g00370  -  RNA recognition motif (RRM)-containing protein
## VIT_05s0051g00230  -  R protein L6
## VIT_03s0063g01890  -  Ring-H2 finger protein ATL3G
## VIT_10s0003g01720  -  Nucleoside triphosphatase
## VIT_01s0146g00490  -  EMB2261 (embryo defective 2261)
## VIT_08s0040g01650  -  Protein kinase
## VIT_18s0122g01160  -  12-oxophytodienoate reductase 2
## VIT_04s0008g01290  -  CGA1 (cytokinin-responsive GATA factor 1)
## VIT_00s0291g00090  -  Cyclin-related
## VIT_18s0001g06810  -  Transcriptional regulator
## VIT_08s0032g00320  -  Pigment defective 318 (PDE318)
## VIT_09s0002g05090  -  CENP-C
## VIT_19s0090g01030  -  Aspartate carbamoyltransferase chloroplast precursor
## VIT_18s0001g07170  -  Transducin protein
## VIT_14s0060g01420  -  Unknown protein
## VIT_08s0007g01250  -  Lumen common protein family, chloroplast
## VIT_02s0012g01090  -  RNA-binding protein P67
## VIT_07s0205g00010  -  Unknown
## VIT_04s0008g05360  -  Serine hydrolase [Vitis vinifera]
## VIT_17s0000g08450  -  Carbonic anhydrase chloroplast
## VIT_07s0104g00660  -  2OG-Fe(II) oxygenase
```

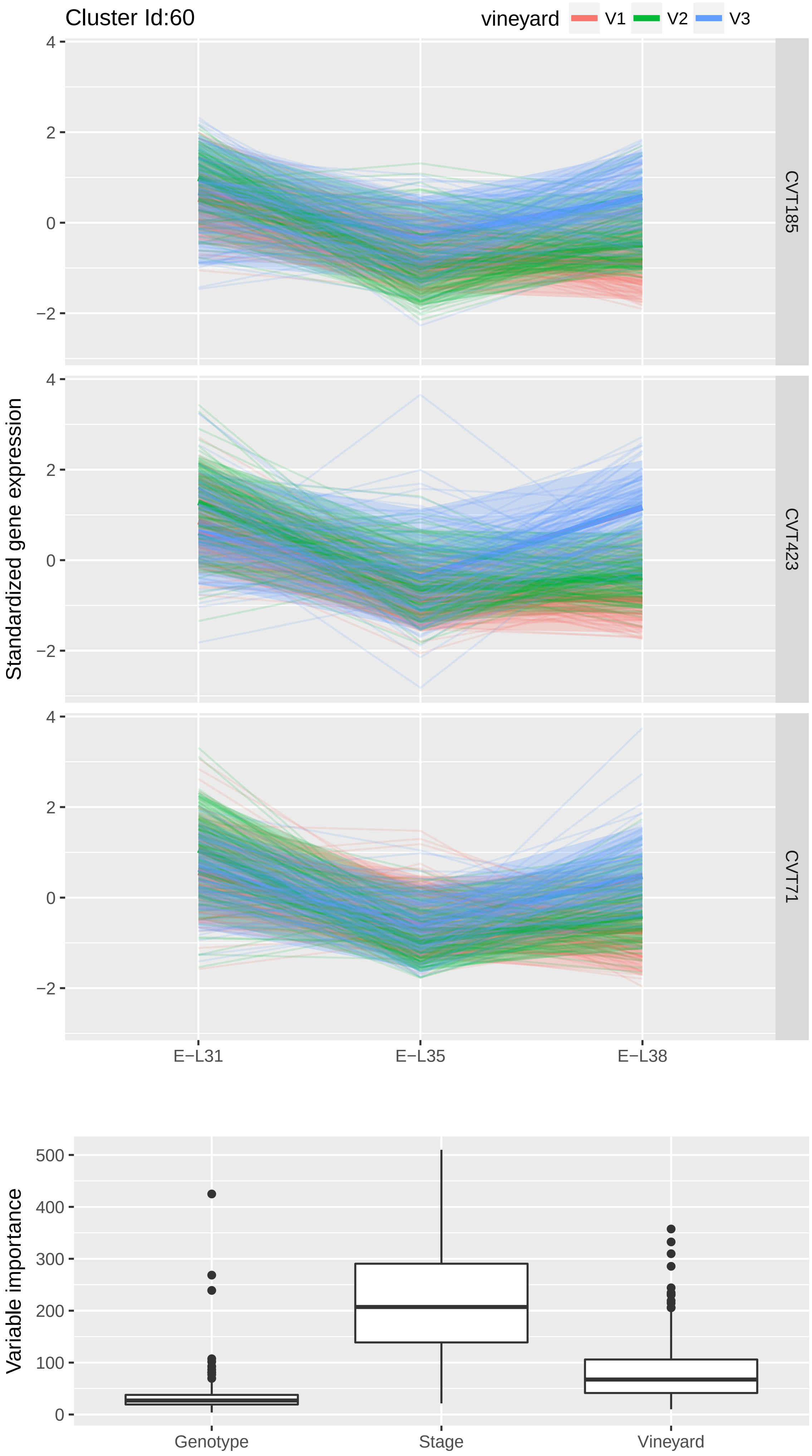

Cluster Id:60
vineyard
V1
V2
V3
Standardized gene expression
CVT185
CVT423
CVT71
4
2
0
−2
E−L31
E−L35
E−L38
Variable importance
500
400
300
200
100
0
Genotype
Stage
Vineyard

Cluster no. 61

```
## Number of genes in the cluster: 84
## Homogeneity Index:    0.63
## Variable importance for Stage:    Median =  234.2  - Rank =  82
## Variable importance for Clone:    Median =   19.8  - Rank =  50
## Variable importance for Vineyard: Median =  58.17  - Rank =  35
##
## Gene ID               Gene Annotation
## VIT_17s0000g09850  -  Unknown protein
## VIT_07s0031g02210  -  No hit
## VIT_04s0008g04300  -  DnaJ homolog, subfamily B, member 4
## VIT_18s0001g12270  -  Unknown protein
## VIT_14s0036g01030  -  Ankyrin repeat
## VIT_17s0000g02670  -  Transducin protein
## VIT_07s0005g05500  -  Unknown protein
## VIT_06s0004g00480  -  Tubulin alpha
## VIT_07s0031g02200  -  Auxin efflux carrier
## VIT_08s0007g06730  -  Spindle pole body component 98
## VIT_18s0001g12360  -  Deoxyuridine 5'-triphosphate nucleotidohydrolase
## VIT_04s0008g01370  -  Peptidyl-prolyl cis-trans isomerase SDCCAG10
## VIT_13s0019g02250  -  Ankyrin
## VIT_03s0091g00600  -  Amino acid permease
## VIT_07s0104g00670  -  BSD domain-containing protein
## VIT_15s0048g02580  -  Pyruvate dehydrogenase E1 component alpha subunit
## VIT_05s0020g00740  -  Phosphate translocator
## VIT_14s0030g01280  -  Double-stranded RNA-binding (DsRBD) domain-containing protein
## VIT_12s0035g00740  -  No hit
## VIT_07s0031g02120  -  Unknown
## VIT_16s0050g00770  -  YDA (YODA)
## VIT_19s0085g00410  -  No hit
## VIT_17s0000g05090  -  Pentatricopeptide (PPR) repeat-containing protein
## VIT_07s0031g01290  -  RNA-binding protein AKIP1
## VIT_06s0004g01360  -  Selenoprotein O
## VIT_00s0194g00090  -  forkhead-associated domain-containing protein
## VIT_08s0040g01590  -  OTU-like cysteine protease Ovarian tumour, otubain
## VIT_09s0002g04770  -  Calcineurin-like phosphoesterase family
## VIT_14s0083g00710  -  Zinc finger (C3HC4-type ring finger)
## VIT_03s0091g00710  -  Mitotic checkpoint protein BUB3
## VIT_05s0049g01240  -  Casein kinase I delta
## VIT_08s0040g01640  -  Telomere repeat binding factor like TRFL8
## VIT_01s0011g01490  -  Protein kinase
## VIT_02s0025g03970  -  Small subunit ribosomal protein S1
## VIT_01s0127g00430  -  phosphatidylinositol 3-kinase
## VIT_14s0068g01270  -  No hit
## VIT_00s0250g00020  -  Protein tyrosine phosphatase
## VIT_14s0128g00380  -  Yip1 domain , member 1
## VIT_11s0016g00240  -  Serine/threonine protein phosphatase 7
## VIT_10s0116g00670  -  Pg4
## VIT_02s0025g02090  -  Kinesin light chain
## VIT_14s0128g00740  -  Unknown protein
## VIT_07s0005g01330  -  SAR1 (secretion-associated ras)
## VIT_09s0054g01060  -  Tassel serine threonine kinase 1
## VIT_11s0016g03460  -  Casein kinase I delta
## VIT_05s0020g02520  -  BTB-POZ and math Domain 2 ATBPM2
## VIT_17s0000g05330  -  Serine/threonine protein phosphatase PP2A-5 catalytic subunit (PP2A5)
```

```
## VIT_04s0023g02740  -  ATP-binding region, ATPase
## VIT_18s0076g00260  -  Unknown protein
## VIT_02s0012g01000  -  Protein tyrosine phosphatase
## VIT_12s0035g01110  -  No hit
## VIT_00s0357g00010  -  Senescence-associated protein
## VIT_12s0028g00570  -  Vacuolar protein sorting 16 VCL1 (vacuoleless 1)
## VIT_01s0137g00280  -  Actin related protein 3
## VIT_01s0011g00870  -  Appr-1-p processing enzyme family protein
## VIT_05s0077g00020  -  Protein transporter
## VIT_03s0088g00950  -  Unknown protein
## VIT_01s0011g06070  -  Unknown protein
## VIT_00s0250g00030  -  Glycosyltransferase family protein 1
## VIT_06s0061g01170  -  Adaptor protein complex AP-2, alpha 1 subunit
## VIT_01s0137g00020  -  No hit
## VIT_16s0050g00660  -  Adaptor-related protein complex 1, sigma 2
## VIT_18s0001g15600  -  Vacuolar protein sorting 45
## VIT_12s0035g00730  -  No hit
## VIT_17s0000g00890  -  Geranylgeranylated protein ATGP4
## VIT_00s0404g00080  -  Vacuolar protein sorting 28
## VIT_06s0004g01350  -  Emsy N terminus domain-containing protein
## VIT_04s0008g00840  -  Ser/Thr specific protein phosphatase 2A B regulatory subunit beta isof
## VIT_02s0025g01700  -  RNA polymerase II transcriptional coactivator KELP
## VIT_01s0026g01970  -  RNA-binding region RNP-1 (RNA recognition motif)
## VIT_18s0001g07130  -  GEM-like 1
## VIT_14s0108g01450  -  Calmodulin-binding
## VIT_02s0025g03930  -  Transcription initiation factor TFIIE beta subunit
## VIT_13s0019g02260  -  BTB-POZ and math Domain 3 ATBPM3
## VIT_18s0001g14700  -  Unknown
## VIT_01s0011g03130  -  Unknown protein
## VIT_00s0471g00040  -  BSD domain-containing protein
## VIT_00s1003g00010  -  Senescence-associated protein
## VIT_19s0014g02660  -  SPA3 (SPA1-related 3)
## VIT_04s0023g00800  -  LUC7 N_terminus domain-containing protein
## VIT_00s0270g00100  -  Diphthamide synthesis DPH2 family
## VIT_04s0008g06500  -  Solute carrier family 35
## VIT_05s0077g00470  -  UPL6 (ubiquitin protein LIGASE 6)
## VIT_07s0031g01350  -  Unknown protein
```

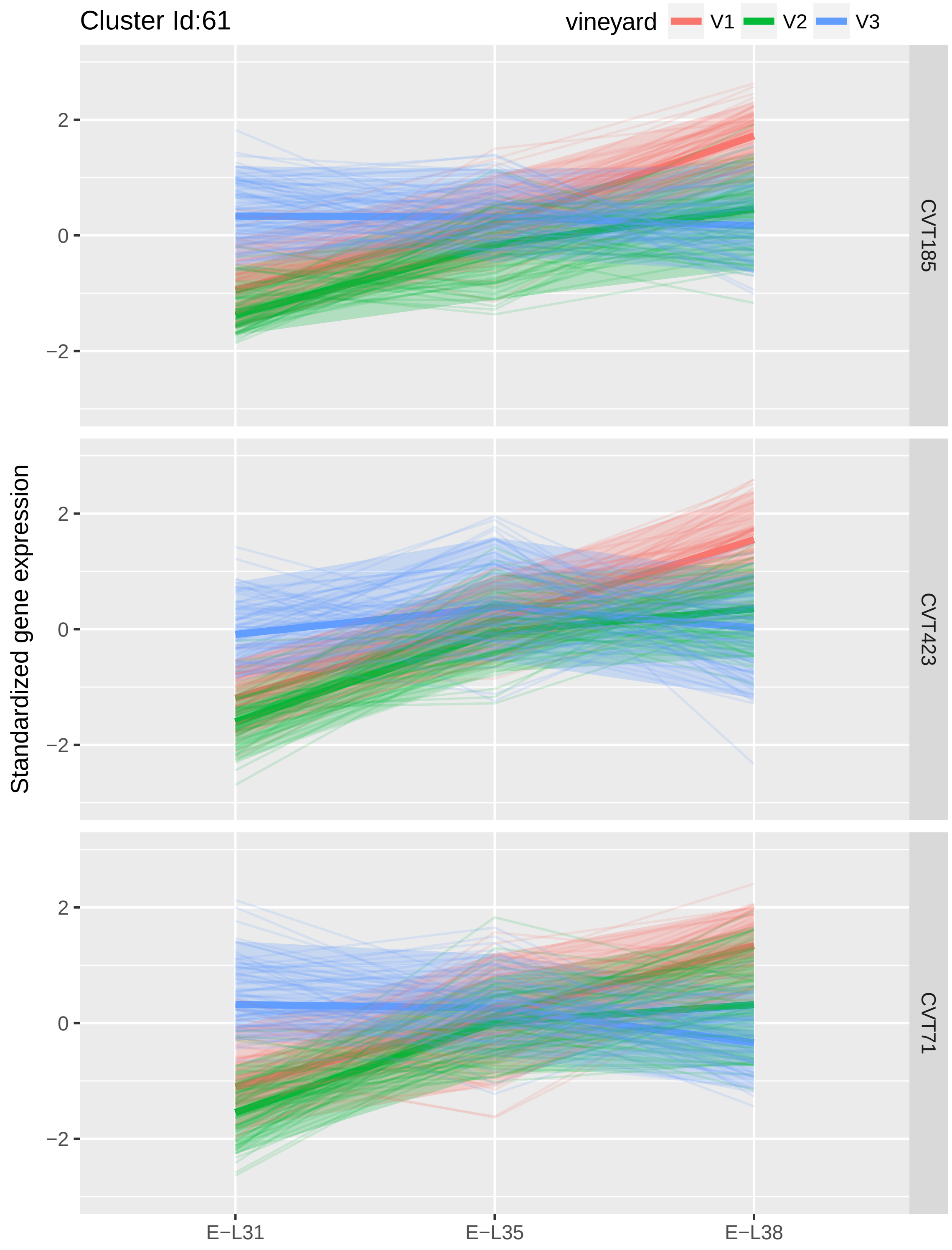
Cluster Id:61
vineyard
V1
V2
V3
Standardized gene expression
CVT185
CVT423
CVT71
2
0
−2
E−L31
E−L35
E−L38

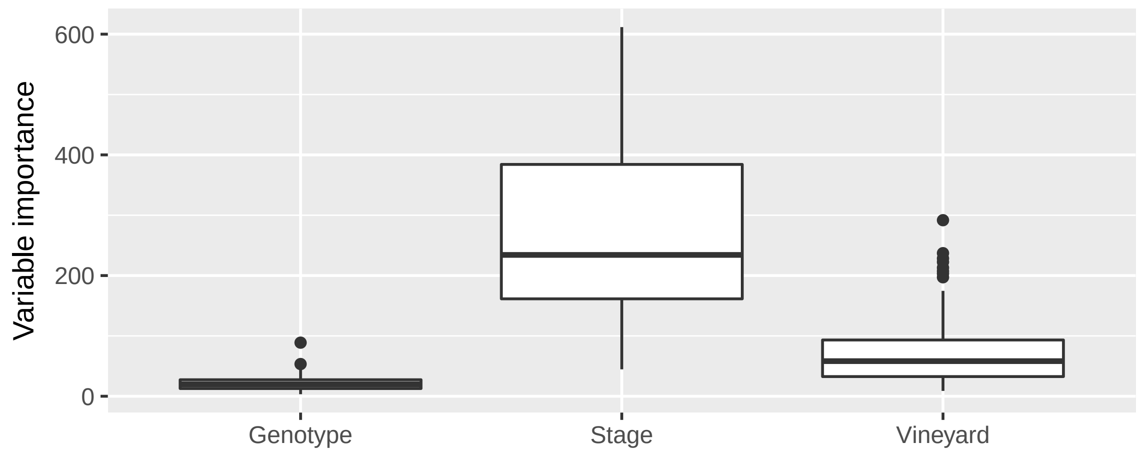
Variable importance
600
400
200
0
Genotype
Stage
Vineyard

Cluster no. 62

```
## Number of genes in the cluster: 87
## Homogeneity Index:    0.78
## Variable importance for Stage:    Median =  449.2  - Rank =  47
## Variable importance for Clone:    Median =  25.85  - Rank =  19
## Variable importance for Vineyard: Median =  36.83  - Rank =  55
##
## Gene ID               Gene Annotation
## VIT_19s0085g00480  -  No hit
## VIT_18s0001g00510  -  Prolyl endopeptidase
## VIT_05s0049g00330  -  E8 protein
## VIT_08s0007g07850  -  UDP-N-acetylmuramoylalanine-D-glutamate ligase
## VIT_01s0010g02920  -  Pre-mRNA-splicing factor ATP-dependent RNA helicase PRP16
## VIT_13s0074g00640  -  Unknown protein
## VIT_13s0067g03380  -  Regulator of chromosome condensation (RCC1)
## VIT_09s0002g03370  -  No hit
## VIT_06s0004g01930  -  Zinc finger (C3HC4-type ring finger)BIG BROTHER
## VIT_05s0077g00750  -  basic helix-loop-helix (bHLH) family
## VIT_08s0056g00080  -  Glycosyl transferase family 8 protein
## VIT_14s0068g00420  -  Alpha-amylase isozyme C2 precursor
## VIT_08s0056g00740  -  Unknown protein
## VIT_00s0265g00030  -  Deoxyoctulonosic acid synthetase
## VIT_18s0001g13880  -  Zinc finger (CCCH-type) family protein
## VIT_07s0130g00400  -  Chloride channel protein (CLC-c)
## VIT_08s0007g01790  -  Defective in exine formation protein (DEX1)
## VIT_02s0025g04640  -  Unknown protein
## VIT_09s0002g05240  -  Cyclin A2;3
## VIT_02s0025g01580  -  UDP-glucose 4-epimerase GEPI48
## VIT_08s0007g03370  -  Unknown protein
## VIT_17s0000g05140  -  Rhomboid
## VIT_08s0058g00820  -  ALPHA-SNAP2 (alpha-soluble nsf attachment protein)
## VIT_18s0122g00470  -  Spindle checkpoint protein Bub1b
## VIT_19s0014g02750  -  N-ethylmaleimide sensitive factor
## VIT_13s0106g00340  -  No hit
## VIT_17s0000g01750  -  Auxin-independent growth promoter
## VIT_06s0004g03810  -  Kinesin motor protein
## VIT_01s0127g00840  -  DNA-binding protein
## VIT_09s0070g00310  -  Glucan endo-1,3-beta-glucosidase 6 precursor
## VIT_17s0000g00240  -  Mei2 AML1
## VIT_01s0137g00420  -  Proteasome 20S beta subunit B (PBB2) (PRCFC)
## VIT_17s0000g07490  -  Unknown protein
## VIT_14s0030g01960  -  Aldo/keto reductase
## VIT_08s0032g01230  -  DEAH (Asp-Glu-Ala-His) box polypeptide 36
## VIT_11s0103g00630  -  Unknown protein
## VIT_08s0040g02500  -  Nodulin MtN21 family
## VIT_19s0015g01220  -  THO complex subunit 3
## VIT_10s0003g01800  -  Zinc finger (FYVE type)
## VIT_18s0001g12910  -  Inositol-pentakisphosphate 2-kinase 1 ATIPK1
## VIT_14s0068g01450  -  Unknown protein
## VIT_00s0265g00050  -  1,3-beta-glucan synthase
## VIT_13s0158g00360  -  Calcium-transporting ATPase 11 ACA11
## VIT_13s0320g00070  -  Myosin-like protein XIF
## VIT_11s0016g04970  -  NADH-cytochrome b5 reductase
## VIT_10s0003g03160  -  Ribosomal protein L18A 60S
## VIT_13s0067g00210  -  Unknown protein
```

```
## VIT_04s0008g02850  -  Proteasome 20S beta subunit A (PBA1) (PRCD)
## VIT_04s0044g01240  -  PRLI-interacting factor G
## VIT_13s0019g00070  -  MAP kinase phosphatase (MKP1)
## VIT_02s0025g04900  -  Phosphoglycerate mutase
## VIT_13s0067g03300  -  Leukotriene A-4 hydrolase
## VIT_10s0003g05320  -  Tubulin beta-7 chain
## VIT_01s0010g02210  -  FRK1 (FLG22-induced receptor-like kinase 1)
## VIT_08s0007g05210  -  Amino acid permease
## VIT_09s0002g04220  -  Ras-related protein Rab-7A
## VIT_04s0023g00050  -  Sterol glucosyltransferase
## VIT_17s0000g06580  -  Vesicle transport v-SNARE 11
## VIT_01s0026g01620  -  Protein kinase APK1A
## VIT_11s0016g02720  -  SNF7.1
## VIT_14s0030g02230  -  Unfertilized embryo sac 12 UNE12
## VIT_11s0016g01440  -  VPS2.1 SNF7
## VIT_04s0023g02920  -  Unknown protein
## VIT_06s0009g01060  -  Tousled-like kinase 2
## VIT_07s0005g06640  -  SEC14 cytosolic factor
## VIT_14s0060g02090  -  Copper chaperone (CCH)
## VIT_07s0005g04460  -  ABC Transporter (VvMRP22 - VvABCC22)
## VIT_04s0044g01210  -  Phosphatidylinositol 4-kinase
## VIT_06s0004g04190  -  PHD zinc finger protein
## VIT_03s0063g00530  -  Calmodulin
## VIT_00s0194g00100  -  No pollen (Osnop)
## VIT_11s0016g03430  -  Protein phosphatase 2C
## VIT_17s0000g01950  -  Dihydropyrimidine dehydrogenase
## VIT_07s0151g00350  -  MHK; kinase
## VIT_16s0013g01960  -  Chromosome condensation
## VIT_16s0098g01140  -  CRK (CDPK-related kinase)
## VIT_08s0040g00280  -  Aladin
## VIT_16s0098g00280  -  Unknown protein
## VIT_02s0025g00470  -  EMB3001 (embryo defective 3001)
## VIT_14s0081g00160  -  Myosin-like protein XIC
## VIT_14s0066g00950  -  Isocitrate dehydrogenase, chloroplast precursor
## VIT_14s0036g01460  -  SNF2 domain-containing protein helicase ATRX/CHR20
## VIT_11s0052g01030  -  Porin
## VIT_11s0052g01600  -  UDP-glucose flavonoid 3-O-glucosyltransferase 7
## VIT_15s0048g01490  -  Geraniol 10-hydroxylase
## VIT_12s0035g01920  -  Methionine S-methyltransferase
## VIT_07s0005g01070  -  No hit
```

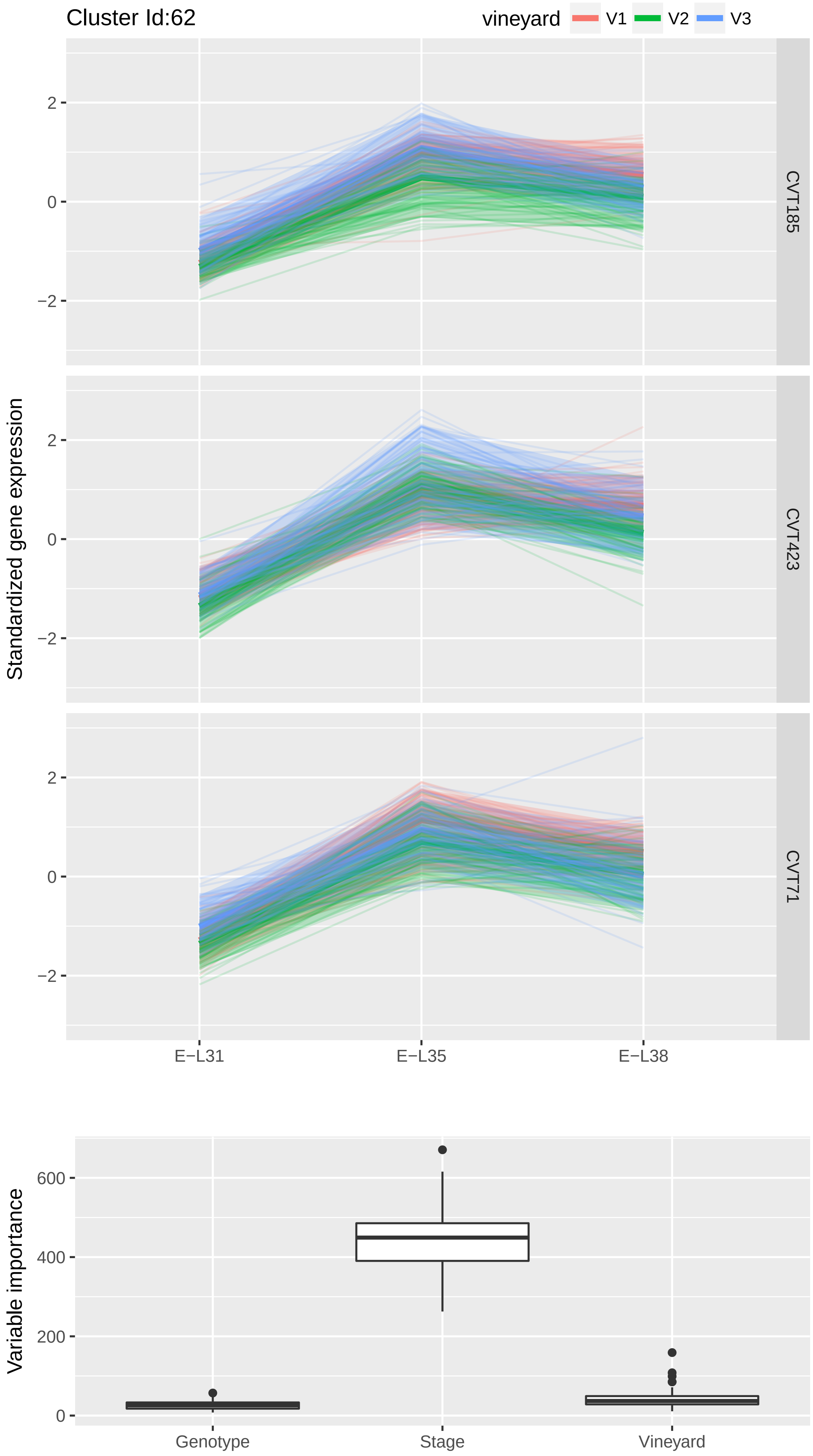

Cluster Id:62
vineyard
V1
V2
V3
Standardized gene expression
CVT185
CVT423
CVT71
2
0
−2
E−L31
E−L35
E−L38
Variable importance
600
400
200
0
Genotype
Stage
Vineyard

Cluster no. 63

```
## Number of genes in the cluster: 65
## Homogeneity Index:    0.59
## Variable importance for Stage:    Median =  43.59  - Rank =  109
## Variable importance for Clone:    Median =   23.1  - Rank =  33
## Variable importance for Vineyard: Median =  315.4  - Rank =  1
##
## Gene ID               Gene Annotation
## VIT_02s0033g01380  -  Alternative oxidase 1D
## VIT_01s0026g01730  -  WRKY Transcription Factor (VvWRKY02)
## VIT_13s0156g00580  -  S-receptor kinase
## VIT_01s0010g01840  -  GEM-like protein 5
## VIT_01s0011g00420  -  RPK1 (receptor-like protein kinase 1)
## VIT_12s0028g00610  -  Aspartic Protease (VvAP31)
## VIT_12s0035g00020  -  Leucine Rich Repeat receptor-like kinase
## VIT_01s0011g03850  -  Unknown protein
## VIT_13s0156g00570  -  S-receptor kinase
## VIT_05s0077g01580  -  s10 (VvPR10.2)_Pathogenesis protein 10 [Vitis vinifera]
## VIT_09s0018g00780  -  HcrVf1 protein
## VIT_08s0040g01710  -  Phenylalanine ammonia-lyase (PAL1)
## VIT_19s0015g01370  -  Molecular chaperone DnaJ
## VIT_11s0052g01090  -  4-coumarate-CoA ligase 2
## VIT_12s0028g00860  -  NAC domain-containing protein (VvNAC36)
## VIT_13s0156g00550  -  S-receptor kinase
## VIT_04s0023g02900  -  Ferulate-5-hydroxylase
## VIT_14s0128g00780  -  Lipoxygenase (VvLOXC)
## VIT_01s0011g02890  -  Unknown protein
## VIT_05s0077g01570  -  s11_Pathogenesis protein 10 [Vitis vinifera]
## VIT_06s0004g00020  -  NAC domain-containing protein (VvNAC44)
## VIT_07s0005g04020  -  Leucine Rich Repeat receptor-like kinase
## VIT_15s0046g00280  -  Unknown
## VIT_14s0066g02470  -  Unknown protein
## VIT_07s0104g01260  -  flavin-containing monooxygenase
## VIT_13s0156g00590  -  S-receptor kinase
## VIT_12s0028g02530  -  Zinc finger (C3HC4-type ring finger)
## VIT_17s0000g02930  -  Glutathione S-transferase 25 GSTU7
## VIT_17s0000g00640  -  No hit
## VIT_01s0011g02320  -  Unknown protein
## VIT_12s0059g01720  -  No hit
## VIT_18s0001g00180  -  Protein kinase
## VIT_09s0002g05010  -  Kelch repeat-containing F-box family protein
## VIT_04s0044g01420  -  Polygalacturonase GH28
## VIT_13s0067g02300  -  Hypoxia-responsive
## VIT_11s0078g00290  -  Cinnamate 4-hydroxylase
## VIT_14s0083g01140  -  B12D
## VIT_06s0004g01010  -  Dirigent protein pDIR9
## VIT_16s0100g01040  -  Stilbene synthase (VvSTS32)
## VIT_05s0020g01360  -  Myb family
## VIT_13s0106g00010  -  Brassinosteroid insensitive 1-associated receptor kinase 1
## VIT_15s0048g01350  -  Gibberellin receptor GID1L3
## VIT_06s0004g03100  -  MLO1
## VIT_05s0077g01560  -  s12 (VvPR10.3)_Pathogenesis protein 10 [Vitis vinifera]
## VIT_16s0050g01890  -  Unknown
## VIT_18s0001g06400  -  No hit
## VIT_15s0021g02470  -  Epoxide hydrolase-like protein
```

```
## VIT_08s0056g00300  -  Unknown
## VIT_09s0002g06900  -  NtEIG-A1
## VIT_13s0074g00350  -  Unknown
## VIT_19s0027g01330  -  R protein  PRF  disease resistance protein
## VIT_07s0130g00040  -  myb domain protein 4
## VIT_04s0008g06060  -  Unknown
## VIT_18s0166g00210  -  Regulator of chromosome condensation (RCC1)
## VIT_10s0071g01060  -  Pyruvate kinase
## VIT_03s0063g00880  -  Calmodulin-binding protein
## VIT_14s0060g02300  -  Hypoxia-responsive
## VIT_13s0084g00670  -  Unknown protein
## VIT_12s0034g00030  -  Anthocyanidin 3-O-glucosyltransferase
## VIT_18s0001g00840  -  Syringolide-induced protein 14-1-1
## VIT_00s0194g00120  -  Serine acetyltransferase
## VIT_13s0019g02080  -  DNA-binding protein
## VIT_01s0011g04040  -  No hit
## VIT_04s0044g00870  -  U-box domain containing protein
## VIT_05s0029g00350  -  Heat shock transcription factor A5
```

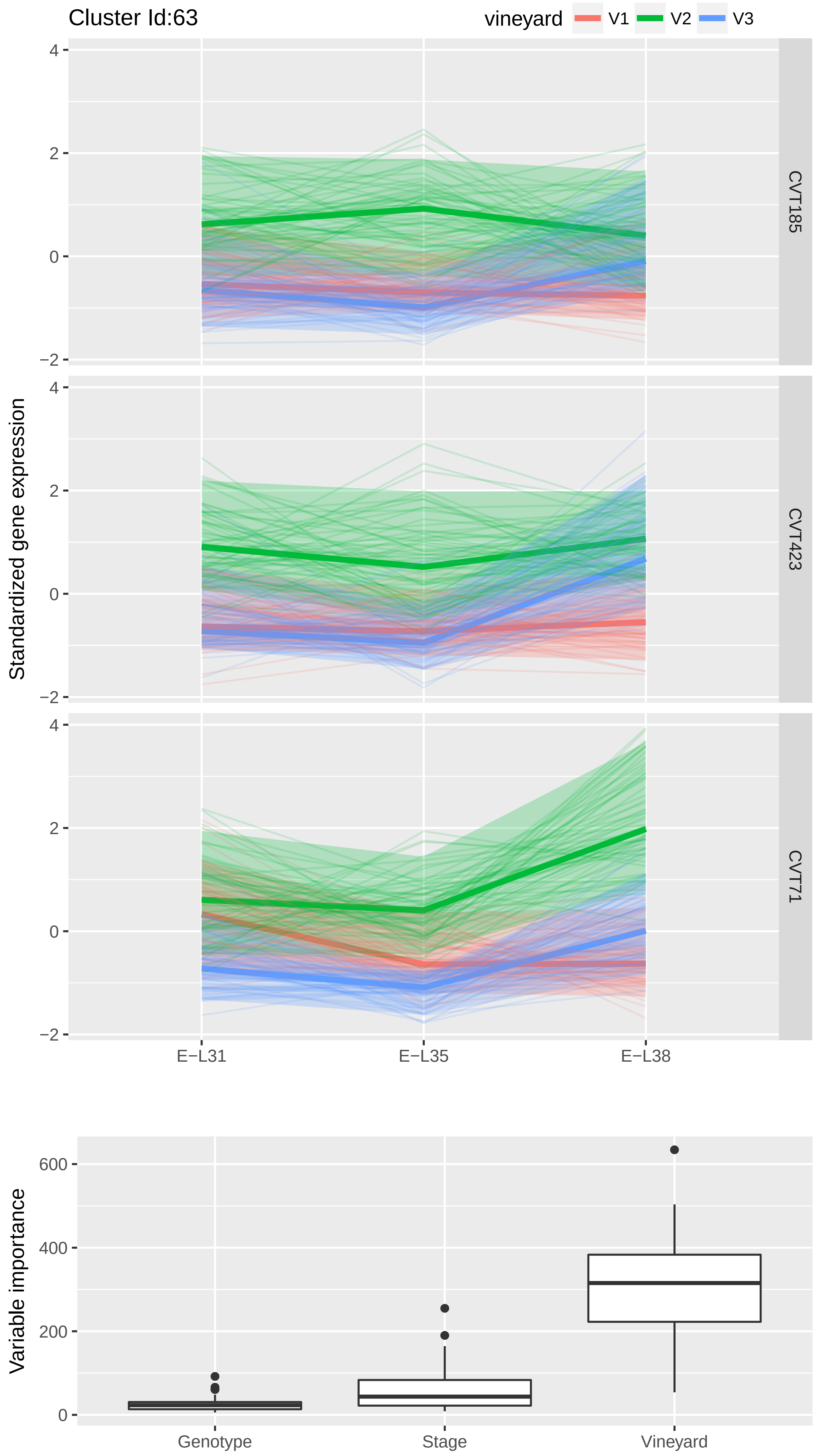

Cluster Id:63
vineyard
V1
V2
V3
Standardized gene expression
CVT185
CVT423
CVT71
E−L31
E−L35
E−L38
Variable importance
Genotype
Stage
Vineyard

Cluster no. 64

```
## Number of genes in the cluster: 110
## Homogeneity Index:    0.66
## Variable importance for Stage:    Median =  386.2  - Rank =  58
## Variable importance for Clone:    Median =  12.94  - Rank =  84
## Variable importance for Vineyard: Median =  47.83  - Rank =  41
##
## Gene ID               Gene Annotation
## VIT_01s0011g03390  -  Unknown protein
## VIT_18s0122g01290  -  RWP-RK domain-containing protein
## VIT_18s0001g01950  -  Pentatricopeptide (PPR) repeat-containing protein
## VIT_13s0067g00860  -  Early flowering 4
## VIT_13s0067g00940  -  NLI interacting factor (NIF)
## VIT_15s0048g01150  -  TCP family transcription factor TCP9
## VIT_03s0063g00840  -  Nodulation protein
## VIT_09s0002g00760  -  Unknown protein
## VIT_01s0026g00930  -  Zinc finger (MYND type)
## VIT_15s0046g01570  -  Acidic endochitinase (CHIB1)
## VIT_14s0060g00870  -  Pentatricopeptide (PPR) repeat-containing
## VIT_05s0077g01820  -  Protein phosphatase 2C
## VIT_02s0012g01900  -  3-beta hydroxysteroid dehydrogenase
## VIT_03s0038g01480  -  Beta-fructofuranosidase
## VIT_14s0066g01020  -  DEAD box RNA helicase 1
## VIT_04s0023g00150  -  Inner membrane ALBINO3-like protein 2
## VIT_03s0017g00530  -  Elongation factor 1-alpha HBS1
## VIT_06s0004g04690  -  DNA-binding protein
## VIT_15s0046g01460  -  Unknown
## VIT_09s0002g07530  -  Tetratricopeptide helical
## VIT_18s0001g08770  -  CF9
## VIT_00s0625g00040  -  Polyadenylate-binding protein 2
## VIT_13s0019g03480  -  HAD superfamily hydrolase
## VIT_19s0090g00330  -  1-acyl-sn-glycerol-3-phosphate acyltransferase
## VIT_02s0025g02310  -  LHW (LONESOME HIGHWAY)
## VIT_11s0016g03050  -  MATE efflux family protein
## VIT_01s0011g03350  -  PRP4 pre-mRNA processing factor 4 homolog B
## VIT_14s0060g02160  -  ATP-dependent Clp protease proteolytic subunit (ClpR1) (nClpP5)
## VIT_13s0019g02910  -  Cis-zeatin O-beta-D-glucosyltransferase
## VIT_04s0023g00980  -  SET domain protein
## VIT_02s0025g02960  -  Naringenin,2-oxoglutarate 3-dioxygenase
## VIT_13s0147g00110  -  ferredoxin
## VIT_11s0016g05710  -  UDP-glucuronic acid/UDP-N-acetylgalactosamine transporter
## VIT_00s0229g00050  -  C2H2 zinc-finger protein ZPT3-3
## VIT_02s0012g01440  -  Zinc finger (C3HC4-type ring finger)
## VIT_01s0150g00360  -  Unknown
## VIT_19s0014g00990  -  DNA polymerase delta subunit 3
## VIT_01s0127g00050  -  SOUL heme-binding
## VIT_08s0007g04150  -  RKL1 (Receptor-like kinase 1)
## VIT_01s0026g02080  -  Kelch repeat-containing F-box protein
## VIT_10s0003g01540  -  Pentatricopeptide (PPR) repeat-containing protein
## VIT_05s0136g00320  -  Unknown protein
## VIT_18s0001g06600  -  Unknown protein
## VIT_07s0005g04270  -  No hit
## VIT_01s0026g00940  -  ARR3 typeA
## VIT_11s0118g00630  -  Unknown protein
## VIT_15s0046g01220  -  No hit
```

```
## VIT_05s0020g01030  -  No hit
## VIT_13s0067g03430  -  ARR9 typeA
## VIT_16s0098g01090  -  PEPR1 (PEP1 receptor 1)
## VIT_10s0116g01660  -  Lactoylglutathione lyase
## VIT_08s0007g04760  -  Tankyrase 1
## VIT_03s0038g00010  -  ATP-dependent RNA helicase DDX23/PRP28
## VIT_19s0027g01090  -  Molybdenum cofactor sulfurase
## VIT_09s0002g03440  -  Protein kinase
## VIT_06s0004g05320  -  Tropinone reductase
## VIT_08s0007g00170  -  DPB-1 transcription factor (DPB)
## VIT_08s0007g03380  -  Pentatricopeptide (PPR) repeat-containing protein
## VIT_03s0088g00710  -  Pathogenesis-related protein 1 precursor (PRP 1)
## VIT_13s0019g03130  -  Glucosyltransferase-2
## VIT_01s0150g00120  -  ERF/AP2 Gene Family (VvERF112)
## VIT_16s0098g00240  -  Ser/Thr receptor-like kinase1
## VIT_18s0041g01360  -  Protein translocon component Tic40, Chloroplast
## VIT_02s0087g00390  -  Endonuclease
## VIT_06s0004g08430  -  ATP-dependent DNA helicase recQ
## VIT_14s0060g00390  -  Pectinesterase family
## VIT_02s0025g04960  -  Unknown protein
## VIT_01s0011g06460  -  Deoxymugineic acid synthase
## VIT_15s0048g00820  -  Pentatricopeptide (PPR) repeat-containing protein
## VIT_06s0004g05860  -  Unknown protein
## VIT_03s0063g00450  -  Alpha-amylase
## VIT_09s0002g06430  -  Lactoylglutathione lyase
## VIT_00s0194g00180  -  Unknown protein
## VIT_16s0022g01250  -  Unknown protein
## VIT_03s0091g00230  -  Unknown protein
## VIT_13s0158g00250  -  No hit
## VIT_18s0122g00550  -  Cyclin-dependent kinase B2;1
## VIT_06s0004g06880  -  Ran-binding protein 1 RanBP1
## VIT_05s0049g01430  -  EDA6/MEE37 (embryo sac development arrest 6)
## VIT_03s0038g04030  -  Pentatricopeptide (PPR) repeat-containing
## VIT_18s0001g07020  -  No hit
## VIT_15s0048g01430  -  24-sterol C-methyltransferase
## VIT_11s0016g04350  -  Methyltransferase Met-10+ like family protein
## VIT_06s0004g03170  -  Transcription initiation factor TFIIH subunit H2
## VIT_11s0118g00790  -  Pto-like serine/threonine kinase
## VIT_03s0038g03740  -  Pentatricopeptide (PPR) repeat-containing
## VIT_05s0077g01260  -  Acyl-activating enzyme 7
## VIT_14s0060g01230  -  Lon protease
## VIT_18s0001g09110  -  Photosystem II PsbC protein
## VIT_08s0007g05760  -  No hit
## VIT_14s0060g00400  -  Pentatricopeptide (PPR) repeat-containing
## VIT_19s0015g00270  -  No hit
## VIT_16s0013g01940  -  Kinase-like protein TMKL1
## VIT_17s0053g00980  -  DnaJ homolog, subfamily C, member 15
## VIT_13s0019g03120  -  Glucosyltransferase-2
## VIT_05s0020g00690  -  PPR2
## VIT_19s0085g00450  -  No hit
## VIT_13s0158g00320  -  FAD linked oxidase, N-terminal
## VIT_13s0067g02120  -  Arogenate dehydrogenase
## VIT_14s0030g00500  -  Copine BON3 (BONZAI 3)
## VIT_19s0015g02730  -  Glutathione S-transferase 25 GSTU25
## VIT_17s0000g07500  -  PTAC2 (plastid transcriptionally active2); binding
```

```
## VIT_05s0049g01170  -  Unknown protein
## VIT_10s0042g01050  -  Serine carboxypeptidase II
## VIT_08s0056g00770  -  Unknown protein
## VIT_19s0014g02950  -  No hit
## VIT_18s0001g00670  -  Unknown protein
## VIT_14s0108g01570  -  Zinc finger (C2H2 type) family
## VIT_04s0043g00820  -  Syntaxin 1B/2/3/4
## VIT_08s0056g01560  -  DsRNA-binding protein ODB1 [Oryza sativa Japonica Group]
```

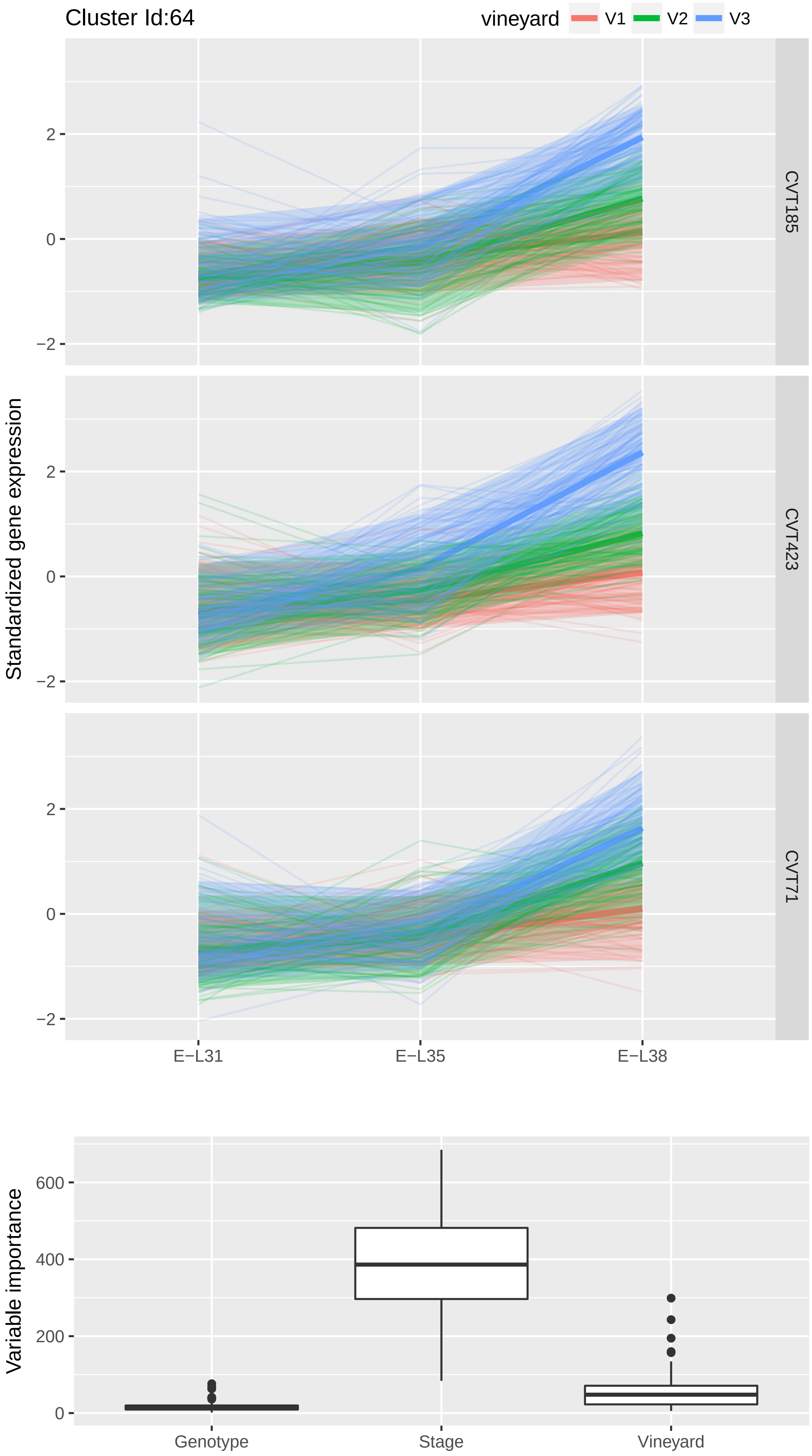
Cluster Id:64
vineyard
V1
V2
V3
CVT185
CVT423
CVT71
Standardized gene expression
2
0
−2
E−L31
E−L35
E−L38
Variable importance
600
400
200
0
Genotype
Stage
Vineyard

Cluster no. 65

```
## Number of genes in the cluster: 113
## Homogeneity Index:    0.72
## Variable importance for Stage:    Median =  545.9  - Rank =  23
## Variable importance for Clone:    Median =   9.42  - Rank =  112
## Variable importance for Vineyard: Median =  28.88  - Rank =  68
##
## Gene ID               Gene Annotation
## VIT_18s0001g13330  -  Purple acid phosphatase 32- ATPAP32/PAP32
## VIT_06s0004g01520  -  Pentatricopeptide (PPR) repeat-containing
## VIT_07s0104g01160  -  Substrate carrier , Mitochondrial
## VIT_14s0066g01160  -  Unknown protein
## VIT_13s0067g00790  -  R protein  PRF  disease resistance protein
## VIT_06s0004g08290  -  KH domain-containing protein
## VIT_09s0002g03270  -  RPS5 (resistant to p. syringae 5)
## VIT_01s0010g03770  -  DnaJ homolog, subfamily B, member 12
## VIT_18s0001g11650  -  Pentatricopeptide repeat
## VIT_17s0000g00530  -  Armadillo/beta-catenin repeat
## VIT_08s0007g00060  -  5-oxoprolinase
## VIT_07s0005g05100  -  Phytochrome  interacting  factor 3-like 5
## VIT_12s0057g01490  -  Telomere repeat binding protein 1
## VIT_17s0000g04660  -  Agmatine deiminase
## VIT_01s0010g00640  -  No hit
## VIT_07s0191g00190  -  GTP cyclohydrolase II/3,4-dihydroxy-2-butanone 4-phosphate synthase
## VIT_01s0011g04790  -  1-phosphatidylinositol-3-phosphate 5-kinase
## VIT_17s0000g10240  -  Isocitrate dehydrogenase [NADP]
## VIT_09s0002g06260  -  RPS5 (resistant to p. syringae 5)
## VIT_14s0030g00840  -  R protein disease resistance protein
## VIT_18s0117g00180  -  CYP71B10
## VIT_11s0149g00120  -  DICER-like 4
## VIT_05s0029g00040  -  Cyclin-dependent kinase inhibitor (KIP-related protein 4)
## VIT_18s0122g00920  -  Zinc finger (DHHC type)
## VIT_14s0036g00240  -  CC-NBS-LRR class
## VIT_07s0005g03070  -  LHW (LONESOME HIGHWAY)
## VIT_12s0028g00050  -  Beta-1,3 glucanase
## VIT_19s0014g01910  -  Unknown protein
## VIT_10s0042g00240  -  Rpp4 candidate 1
## VIT_00s0378g00010  -  Disease resistance protein (NBS class)
## VIT_06s0009g01200  -  Argonaute
## VIT_10s0003g01500  -  Zinc finger (C3HC4-type ring finger)
## VIT_04s0023g01780  -  phytoene dehydrogenase-related (PDH) (VvPDH1)
## VIT_06s0004g02480  -  Cysteine peptidase
## VIT_08s0007g06280  -  Unknown
## VIT_07s0104g00730  -  Unknown protein
## VIT_19s0090g00570  -  Salicylic acid-induced fragment 1 protein
## VIT_13s0139g00030  -  R protein MLA10
## VIT_12s0035g00410  -  Disease resistance protein
## VIT_13s0019g02730  -  Unknown protein
## VIT_15s0021g01130  -  Rho GTPase; Rho GTP binding protein
## VIT_17s0000g07020  -  Cis-zeatin O-beta-D-glucosyltransferase
## VIT_00s0394g00010  -  Carboxyl-terminal proteinase
## VIT_07s0031g00100  -  flavonol synthase
## VIT_09s0018g01970  -  Unknown protein
## VIT_17s0000g07950  -  Unknown protein
## VIT_12s0059g00620  -  Exosome complex exonuclease RRP6
```

```
## VIT_13s0067g00740  -  R protein  PRF  disease resistance protein
## VIT_14s0066g00610  -  Unknown protein
## VIT_16s0050g00620  -  Calmodulin-binding heat shock protein
## VIT_10s0116g00020  -  Tobamovirus multiplication 1 homolog 3
## VIT_04s0044g01450  -  Unknown protein
## VIT_15s0045g00440  -  Disease resistance protein (TIR-NBS-LRR class)
## VIT_01s0011g03310  -  Unknown protein
## VIT_05s0020g01740  -  RanGAP1 interacting protein
## VIT_08s0040g02870  -  Fe-S-cluster oxidoreductase
## VIT_12s0034g01750  -  R protein MLA10
## VIT_13s0047g01010  -  R protein MLA10
## VIT_19s0014g01330  -  Peptide-N4-asparagine amidase A
## VIT_05s0020g02460  -  ABC Transporter (VvNAP4 - VvABCI4)
## VIT_04s0023g02290  -  S-adenosyl-L-methionine:salicylic acid carboxyl methyltransferase
## VIT_05s0020g02310  -  Pyruvate,orthophosphate dikinase
## VIT_06s0061g01510  -  Histone deacetylase 9
## VIT_13s0047g00740  -  Disease resistance protein (NBS class)
## VIT_12s0034g01490  -  R protein MLA10
## VIT_07s0129g00810  -  CYP81E8
## VIT_16s0050g00250  -  Disease resistance family protein
## VIT_05s0124g00020  -  Isocitrate lyase
## VIT_14s0083g00230  -  Unknown
## VIT_13s0101g00300  -  R protein MLA10
## VIT_00s2582g00010  -  Unknown
## VIT_18s0001g12090  -  Unknown protein
## VIT_18s0001g13870  -  Calmodulin binding protein
## VIT_00s0194g00200  -  FAR1-related sequence 10
## VIT_07s0129g00970  -  Protein kinase family
## VIT_12s0034g01270  -  RPS4 (resistant to p. syringae 4)
## VIT_11s0016g05260  -  Unknown protein
## VIT_18s0072g01130  -  Unknown protein
## VIT_14s0060g00210  -  flap endonuclease 1a
## VIT_14s0060g01220  -  Pentatricopeptide repeat
## VIT_09s0002g02300  -  Indeterminate(ID)-domain 7
## VIT_18s0001g10590  -  Inner membrane import protein Tic22, Chloroplast
## VIT_18s0041g02410  -  Aldehyde oxidase 1
## VIT_14s0066g00850  -  Nitrate transporter2.5
## VIT_18s0001g07740  -  SABRE
## VIT_08s0040g03140  -  FRK1 (FLG22-induced receptor-like kinase 1)
## VIT_06s0061g00350  -  ABC Transporter (VvWBC24 - VvABCG24)
## VIT_07s0104g00030  -  No hit
## VIT_16s0050g00110  -  Unknown protein
## VIT_10s0003g01690  -  Unknown protein
## VIT_11s0016g00110  -  Potassium channel tetramerisation domain-containing protein
## VIT_14s0066g01490  -  UDP-glucoronosyl and UDP-glucosyl transferase
## VIT_08s0058g01450  -  STN8 (state transition 8)
## VIT_02s0025g00080  -  Unknown protein
## VIT_14s0006g01840  -  Seven in absentia protein
## VIT_08s0032g01190  -  Nuclear transcription factor Y subunit A-10
## VIT_16s0100g01250  -  Unknown protein
## VIT_01s0127g00010  -  Carboxyl-terminal proteinase
## VIT_07s0005g02290  -  Unknown protein
## VIT_11s0037g00280  -  Accumulation of photosystem one 2
## VIT_13s0067g01340  -  Unknown protein
## VIT_13s0067g01670  -  Ankyrin repeat protein
```

```
## VIT_14s0036g00330  -  RPS2 (resistant to p. syringae 2)
## VIT_14s0066g01500  -  UDP-glucoronosyl and UDP-glucosyl transferase
## VIT_07s0129g00960  -  Protein kinase pto
## VIT_06s0009g01360  -  R protein  PRF  disease resistance protein
## VIT_18s0001g07270  -  R protein MLA10
## VIT_12s0142g00100  -  AML5 (arabidopsis mei2-like protein 5)
## VIT_00s2512g00010  -  No hit
## VIT_03s0038g02800  -  Cyclin B2;4
## VIT_08s0040g00250  -  Tyrosine-specific protein phosphatase
## VIT_12s0034g01630  -  No hit
## VIT_06s0009g01350  -  R protein  PRF  disease resistance protein
```

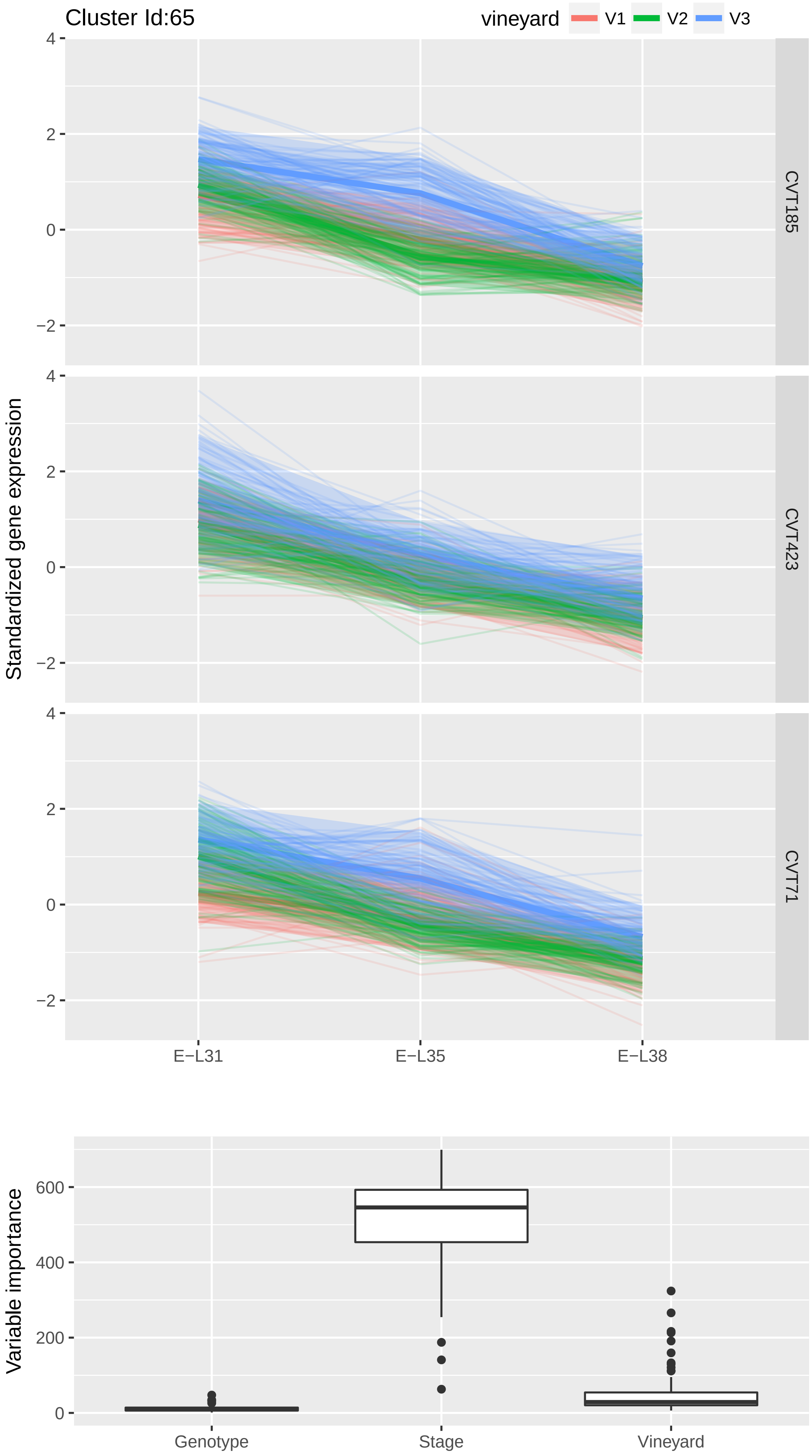

Cluster Id:65
vineyard
V1
V2
V3
Standardized gene expression
CVT185
CVT423
CVT71
E−L31
E−L35
E−L38
Variable importance
Genotype
Stage
Vineyard

Cluster no. 66

```
## Number of genes in the cluster: 230
## Homogeneity Index:    0.92
## Variable importance for Stage:    Median =  646.1  - Rank =  6
## Variable importance for Clone:    Median =  12.92  - Rank =  85
## Variable importance for Vineyard: Median =  13.47  - Rank =  104
##
## Gene ID               Gene Annotation
## VIT_05s0094g01250  -  Allyl alcohol dehydrogenase
## VIT_16s0100g01310  -  DNA mismatch repair protein MSH3
## VIT_06s0004g04550  -  Ankyrin repeat
## VIT_05s0062g00110  -  Unknown protein
## VIT_14s0006g01250  -  GTPase activating protein
## VIT_06s0061g01540  -  KCBP- interacting  protein kinase
## VIT_14s0060g00290  -  Anaphase-promoting complex component APC11
## VIT_08s0040g02360  -  Calmodulin binding protein
## VIT_09s0002g01900  -  APM1 (Aminopeptidase M1)
## VIT_12s0055g00580  -  Receptor protein kinase
## VIT_18s0041g01900  -  Elongation factor 1-gamma
## VIT_19s0014g01160  -  RAB GTPase RAB11
## VIT_00s0407g00010  -  Pleckstriny (PH) domain-containing protein
## VIT_05s0062g00150  -  Pentatricopeptide repeat protein
## VIT_12s0057g01480  -  Glucose-1-phosphate adenylyltransferase small subunit, chloroplast
## VIT_16s0039g01820  -  Unknown protein
## VIT_16s0098g00030  -  Receptor kinase LRK10
## VIT_19s0027g00540  -  No hit
## VIT_10s0116g00440  -  Protein kinase G11A
## VIT_00s0294g00100  -  Brassinosteroid insensitive 1-associated receptor kinase 1
## VIT_17s0000g08530  -  Boron transporter-like protein 1
## VIT_09s0002g07120  -  Adenine phosphoribosyltransferase
## VIT_07s0129g00310  -  Formamidase
## VIT_06s0004g02380  -  Cinnamyl alcohol dehydrogenase
## VIT_14s0066g00870  -  ATPase-like domain-containing
## VIT_07s0031g02950  -  Ammonium transporter 2
## VIT_00s0260g00080  -  Ribosomal protein L32 60S
## VIT_11s0016g05110  -  Sodium hypersensitive 1
## VIT_11s0016g02360  -  TET2 (tetraspanin2)
## VIT_02s0154g00070  -  Abnormal floral organs
## VIT_08s0007g03710  -  ABC Transporter (VvPDR23 - VvABCG53)
## VIT_12s0035g01030  -  Unknown protein
## VIT_09s0002g06210  -  RPS5 (resistant to p. syringae 5)
## VIT_14s0060g01730  -  No hit
## VIT_04s0023g03880  -  Unknown
## VIT_07s0031g00120  -  Leucine-rich repeat transmembrane protein kinase
## VIT_14s0060g00120  -  Germin-like protein 2 [Vitis vinifera]
## VIT_06s0009g02320  -  4-diphosphocytidyl-2-C-methyl-D-erythritol kinase, chloroplast precurs
## VIT_00s2705g00010  -  Unknown
## VIT_11s0016g03700  -  Pentatricopeptide (PPR) repeat-containing protein
## VIT_15s0046g01560  -  Unknown protein
## VIT_11s0016g02790  -  Zinc finger (CCCH-type) family protein
## VIT_06s0004g00880  -  Protease inhibitor/seed storage/lipid transfer protein (LTP)
## VIT_06s0004g05330  -  Tropinone reductase
## VIT_04s0044g00170  -  MUTL protein homolog 3 (ATMLH3/MLH3)
## VIT_07s0005g00100  -  LPA1 (LOW PSII accumulation1)
## VIT_18s0001g12190  -  CYP721A1
```

```
## VIT_02s0025g03030  -  Zinc finger (C3HC4-type ring finger)
## VIT_13s0064g00280  -  Cinnamyl alcohol dehydrogenase
## VIT_01s0150g00540  -  Unknown protein
## VIT_08s0007g06670  -  Homeobox-leucine zipper protein 14
## VIT_02s0025g01740  -  Auxin response factor 9
## VIT_14s0068g02110  -  Sulfite oxidase
## VIT_06s0004g03240  -  Elongation factor 1-alpha 1
## VIT_06s0004g05340  -  Tropinone reductase
## VIT_14s0060g00690  -  ADP-ribosylation factor A1B
## VIT_18s0122g00160  -  Pitrilysin
## VIT_11s0016g02530  -  Protein arginine N-methyltransferase
## VIT_01s0137g00200  -  Unknown protein
## VIT_00s0333g00040  -  Early tobacco anther 1, putative
## VIT_04s0008g04240  -  ferredoxin
## VIT_07s0031g01460  -  Nuclear transcription factor Y subunit B-3
## VIT_14s0083g01030  -  putative MADS-box Fruitfull  2 (VviFUL2)
## VIT_02s0025g02190  -  Short-chain dehydrogenase/reductase
## VIT_09s0002g00540  -  Lipase GDSL 1
## VIT_14s0060g00710  -  Synbindin TRAPPC4
## VIT_06s0004g02230  -  Unknown protein
## VIT_15s0048g00910  -  Unknown protein
## VIT_14s0030g00250  -  Sugar transporter ERD6-like 3
## VIT_00s0389g00040  -  CYP72A1
## VIT_19s0085g00870  -  Aldehyde Dehydrogenase (VvALDH5F2)
## VIT_19s0015g01200  -  Calcium-binding protein CML
## VIT_02s0025g03380  -  3-ketoacyl-CoA reductase 3
## VIT_19s0090g01570  -  Ribosomal protein S8 (RPS8A) 40S
## VIT_11s0037g00010  -  ERF/AP2 Gene Family (VvRAV6)
## VIT_06s0004g02370  -  Cinnamyl alcohol dehydrogenase
## VIT_19s0014g05370  -  No hit
## VIT_04s0008g01160  -  Indeterminate(ID)-domain 7
## VIT_14s0128g00470  -  Unknown protein
## VIT_01s0011g02940  -  basic helix-loop-helix (bHLH) family
## VIT_04s0008g01320  -  Unknown protein
## VIT_06s0009g02110  -  Kelch repeat-containing F-box family protein
## VIT_01s0026g01950  -  Homeobox-leucine zipper protein HB13
## VIT_18s0072g01110  -  GASA like
## VIT_11s0016g03640  -  Rac-like GTP-binding protein ARAC7 (GTPase protein ROP9)
## VIT_02s0025g02850  -  Subtilisin protease
## VIT_05s0102g01210  -  ABC Transporter (VvNAP1 - VvABCI1)
## VIT_18s0001g12340  -  Unknown protein
## VIT_18s0089g01420  -  Hydrolase, alpha/beta fold
## VIT_01s0150g00310  -  Ribosomal protein S27 (ARS27A) 40S
## VIT_06s0004g07310  -  Indole-3-acetate beta-glucosyltransferase
## VIT_02s0025g01870  -  NAC domain-containing protein (VvNAC72)
## VIT_04s0008g00200  -  Tryptophan-tRNA ligase
## VIT_15s0046g01780  -  Ribosomal protein L5
## VIT_08s0058g00370  -  Unknown protein
## VIT_14s0066g00560  -  Ribosomal protein L27A (RPL27aC) 60S
## VIT_17s0000g02530  -  FK506-binding protein genes family (VvFKBP17-2)
## VIT_18s0001g12180  -  CYP721A1
## VIT_03s0017g01420  -  Reticuline oxidase
## VIT_19s0014g04270  -  S-locus protein kinase
## VIT_18s0001g08240  -  Leucine-rich repeat transmembrane protein kinase
```

```
## VIT_00s0274g00060  -  Glycine-rich protein
## VIT_05s0077g01220  -  Ribosomal protein L29 (RPL29B) 60S
## VIT_00s0323g00060  -  Invertase/pectin methylesterase inhibitor
## VIT_16s0098g00560  -  SP1L2 (SPIRAL2)
## VIT_01s0011g04980  -  Sulfate transporter 91
## VIT_09s0002g00840  -  Nuclear pore complex protein Nup107
## VIT_08s0007g07210  -  Ribosomal protein S28 (RPS28C) 40S
## VIT_02s0025g01910  -  Cellulose synthase CSLG3
## VIT_04s0008g04160  -  Dehydration-responsive protein (RD22)
## VIT_10s0003g03180  -  Phospho-N-acetylmuramoyl-pentapeptide-transferase
## VIT_03s0038g04410  -  Unknown
## VIT_10s0092g00260  -  Alpha-glucosidase 1 (AGLU1)
## VIT_09s0002g09290  -  Disease resistance protein
## VIT_12s0059g02180  -  MATE efflux family protein
## VIT_15s0021g01340  -  Phagocytosis and cell motility protein ELMO1
## VIT_13s0064g01320  -  CYP712A1
## VIT_18s0001g08430  -  Branched-chain-amino-acid aminotransferase 2, chloroplast (Atbcat-2)
## VIT_04s0044g01740  -  3-hydroxy-3-methylglutaryl-coenzyme A reductase 3
## VIT_06s0004g08360  -  NADH dehydrogenase I subunit N
## VIT_11s0052g00260  -  Unknown protein
## VIT_10s0092g00040  -  Toprim domain-containing protein
## VIT_06s0004g04310  -  Alcohol dehydrogenase
## VIT_09s0002g01460  -  Ribosomal protein S3 (RPS3A) 40S
## VIT_05s0020g01840  -  3,8-divinyl protochlorophyllide a 8-vinyl reductase
## VIT_09s0002g00860  -  Unknown protein
## VIT_05s0020g04490  -  No hit
## VIT_01s0011g00480  -  Aspartate/glutamate/uridylate kinase
## VIT_00s2380g00010  -  S-locus lectin protein kinase
## VIT_15s0021g02300  -  Squamosa promoter-binding protein (VvSBP14)
## VIT_13s0084g00850  -  NADPH:quinone oxidoreductase
## VIT_10s0003g02680  -  Phytochrome E
## VIT_18s0001g08090  -  IAA9
## VIT_07s0031g00250  -  Transporter-related
## VIT_14s0068g01690  -  TCP family transcription factor TCP20
## VIT_18s0001g13460  -  putative MADS-box Apetala  3a (VviAP3a)
## VIT_02s0154g00260  -  Nitrate transporter
## VIT_17s0000g06450  -  Ribulose-phosphate 3-epimerase, chloroplast precursor
## VIT_15s0021g01880  -  Arabidopsis thaliana homeobox protein 2
## VIT_18s0001g01470  -  No hit
## VIT_17s0000g04840  -  Sterile alpha motif (SAM) domain-containing
## VIT_18s0001g08630  -  Unknown protein
## VIT_05s0049g00270  -  E8 protein
## VIT_13s0156g00190  -  No hit
## VIT_05s0049g00250  -  2-oxoglutarate-dependent dioxygenase
## VIT_06s0009g01530  -  Embryonic abundant protein
## VIT_00s0199g00070  -  Pleckstriny (PH) domain-containing protein
## VIT_00s0551g00020  -  Ribosomal protein L15e
## VIT_08s0040g03320  -  Glutamate N-acetyltransferase
## VIT_05s0020g04880  -  Seed specific protein Bn15D14A
## VIT_04s0023g02240  -  S-adenosyl-L-methionine:salicylic acid carboxyl methyltransferase
## VIT_06s0004g04430  -  Ubiquitin-conjugating enzyme E2 G1
## VIT_03s0017g02000  -  Anthocyanidin 3-O-glucosyltransferase
## VIT_18s0072g00300  -  5-formyltetrahydrofolate cycloligase
## VIT_14s0060g01720  -  Dormancy/auxin associated protein
## VIT_18s0001g01490  -  Oxidoreductase N-terminal domain-containing
```

```
## VIT_06s0004g03530  -  Nitrate excretion transporter1
## VIT_08s0007g04590  -  UGT73C2 (UDP-glucosyl transferase 73C2)
## VIT_02s0012g01380  -  No hit
## VIT_16s0050g00080  -  Remorin
## VIT_10s0003g03120  -  Urease accessory protein G
## VIT_07s0005g02460  -  No hit
## VIT_02s0087g00810  -  SWIB complex BAF60b domain-containing protein
## VIT_02s0025g01260  -  NADPH HC toxin reductase
## VIT_13s0073g00650  -  Pyrimidine 5'-nucleotidase
## VIT_13s0019g02640  -  Subtilisin protease C1
## VIT_16s0022g02110  -  DEAD box RNA helicase (PRH75)
## VIT_08s0007g03340  -  Ribosomal protein L10A (RPL10aA) 60S
## VIT_05s0020g04500  -  Unknown protein
## VIT_16s0039g02550  -  Seed specific protein Bn15D1B
## VIT_03s0038g02840  -  Unknown
## VIT_13s0073g00560  -  DnaJ homolog, subfamily A, member 2
## VIT_16s0013g00210  -  Metacaspase 1
## VIT_18s0001g14130  -  Zinc finger (C2H2 type) family
## VIT_00s0253g00060  -  OBF binding protein 2
## VIT_14s0030g01620  -  Ribosomal protein S24 (RPS24A) 40S
## VIT_17s0000g05610  -  Isopiperitenol dehydrogenase
## VIT_19s0014g04400  -  S-locus protein kinase
## VIT_06s0004g00220  -  Protein kinase APK1B
## VIT_03s0180g00130  -  D-3-phosphoglycerate dehydrogenase, chloroplast precursor
## VIT_15s0024g00390  -  Ribosomal protein L3
## VIT_08s0040g00690  -  Unknown
## VIT_00s0199g00190  -  14-3-3 protein GF14 kappa (GRF8)
## VIT_14s0108g00850  -  Ribosomal protein L34 (RPL34A) 60S
## VIT_01s0011g04410  -  APG2 (albino and pale green 2)
## VIT_18s0001g09140  -  Esterase
## VIT_06s0004g05310  -  Tropinone reductase
## VIT_19s0093g00220  -  Glutathione S-transferase 8 GSTU19
## VIT_17s0000g09400  -  Unknown protein
## VIT_19s0090g00830  -  Aspartic Protease (VvAP45)
## VIT_04s0008g01970  -  ER (ERECTA)
## VIT_05s0062g01090  -  Unknown
## VIT_03s0017g01930  -  CYP97B2
## VIT_18s0001g08100  -  Retroelement pol polyprotein
## VIT_00s0226g00170  -  No hit
## VIT_05s0020g01170  -  Translationally-controlled tumor protein
## VIT_11s0065g00150  -  Glycogen synthase
## VIT_18s0001g09040  -  LIM domain protein WLIM1
## VIT_09s0002g00100  -  phytoene desaturase (PDS) (VvPDS1)
## VIT_14s0060g01550  -  Dehydration-induced 19
## VIT_19s0090g00990  -  Acid phosphatase/vanadium-dependent haloperoxidase
## VIT_06s0009g01070  -  Unknown protein
## VIT_00s0276g00020  -  No hit
## VIT_12s0055g00250  -  UDP-glucose glucosyltransferase
## VIT_18s0122g00330  -  Unknown protein
## VIT_18s0001g12620  -  U-box domain-containing protein
## VIT_03s0038g02520  -  Aquaporin PIP3
## VIT_05s0029g01000  -  HcrVf3 protein
## VIT_00s0194g00340  -  No hit
## VIT_01s0011g05450  -  Unknown protein
## VIT_06s0004g04560  -  Unknown protein
```

```
## VIT_18s0001g15650  -  Pathogenesis related protein
## VIT_00s1553g00010  -  Subtilisin-like proteinase AIR3
## VIT_16s0039g01910  -  Vegetative storage protein win4.5
## VIT_18s0001g03190  -  Glutaredoxin 2
## VIT_08s0007g03620  -  Ribosomal protein P2 (RPP2A) acidic 60S
## VIT_04s0023g02650  -  CYP90D1 ROT3 (ROTUNDIFOLIA 3) steroid hydroxylase
## VIT_18s0001g15640  -  Pathogenesis-related
## VIT_15s0021g01870  -  Actin-related protein 2/3 complex subunit 5
## VIT_07s0005g03540  -  Plastid-specific 50S ribosomal protein 6
## VIT_15s0021g00460  -  Ribose-phosphate pyrophosphokinase 1
## VIT_02s0025g03590  -  Phospholipid hydroperoxide glutathione peroxidase
## VIT_05s0020g02480  -  Glutamine synthetase
## VIT_02s0025g01820  -  Cellulose synthase CSLG3
## VIT_11s0016g03580  -  Catalytic
## VIT_12s0059g02220  -  MATE efflux family protein
## VIT_08s0032g00050  -  Acyl-activating enzyme 15
## VIT_10s0092g00250  -  Alpha-glucosidase 1 (AGLU1)
## VIT_18s0001g05180  -  Beta-D-xylosidase
## VIT_00s0454g00030  -  Subtilisin protease
```

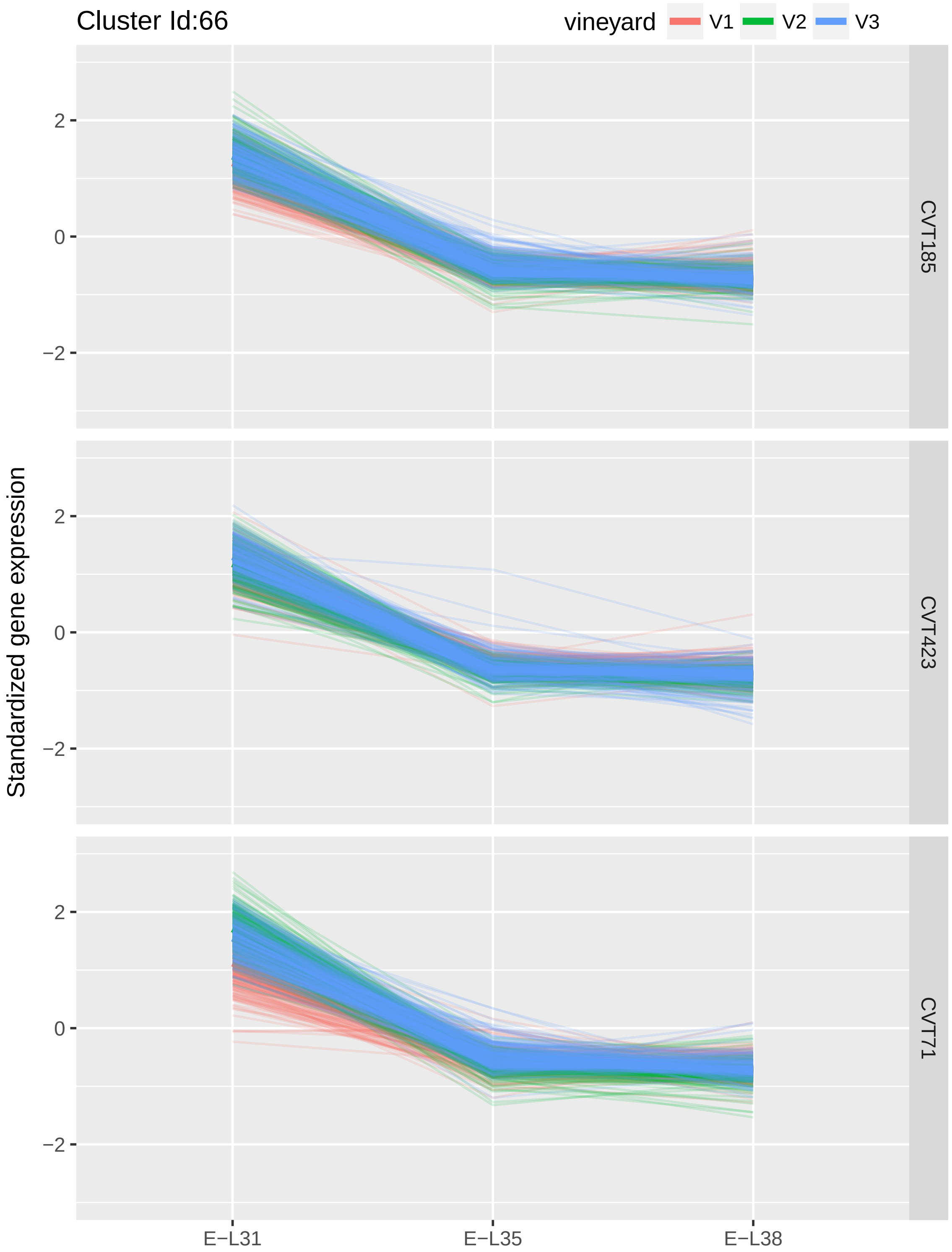

Cluster Id:66
vineyard
V1
V2
V3
Standardized gene expression
CVT185
CVT423
CVT71
2
0
−2
E−L31
E−L35
E−L38

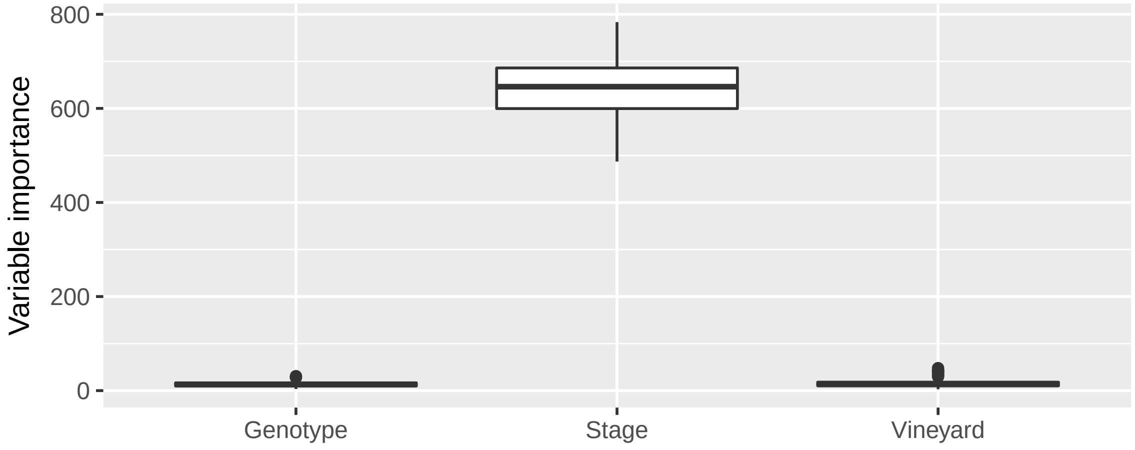

Variable importance
800
600
400
200
0
Genotype
Stage
Vineyard

Cluster no. 67

```
## Number of genes in the cluster: 122
## Homogeneity Index:    0.81
## Variable importance for Stage:    Median =  581.4  - Rank =  20
## Variable importance for Clone:    Median =  10.43  - Rank =  104
## Variable importance for Vineyard: Median =  12.47  - Rank =  106
##
## Gene ID               Gene Annotation
## VIT_18s0075g00880  -  TRNA nucleotidyltransferase
## VIT_18s0001g05830  -  Unknown
## VIT_16s0098g01580  -  Heat shock protein 70
## VIT_16s0098g01260  -  Intramitochondrial sorting protein MSP1 protein
## VIT_13s0156g00310  -  N-terminal asparagine amidohydrolase
## VIT_14s0068g00660  -  ATAN11 (ANTHOCYANIN11) (VvWDR2)
## VIT_03s0038g00970  -  Dynamin-like protein 2b
## VIT_14s0030g01610  -  Cytidine/deoxycytidylate deaminase
## VIT_10s0003g02250  -  Translation initiation factor eIF-4E
## VIT_12s0057g00550  -  WRKY Transcription Factor (VvWRKY38)
## VIT_08s0007g07020  -  CCR4-NOT transcription complex subunit 6
## VIT_16s0050g00480  -  CYP715A1
## VIT_01s0011g00160  -  Alpha-1,4-glucan-protein synthase
## VIT_00s0269g00030  -  No hit
## VIT_05s0051g00930  -  DNA primase large subunit
## VIT_09s0018g02090  -  EDA30 (embryo sac development arrest 30)
## VIT_10s0003g01220  -  Copper-binding family protein
## VIT_00s0686g00020  -  BSD domain-containing protein
## VIT_19s0015g01390  -  Transcription initiation factor TFIIH subunit H1
## VIT_05s0029g00310  -  Serine hydroxymethyltransferase 1
## VIT_08s0056g00320  -  Zinc finger (C3HC4-type ring finger)
## VIT_13s0156g00330  -  Unknown
## VIT_08s0007g03750  -  1,4-alpha-glucan branching enzyme IIB, chloroplast precursor
## VIT_16s0050g02660  -  Pyruvate kinase
## VIT_00s0665g00010  -  Cyclin dependent kinase group C 2
## VIT_00s1238g00010  -  BSD domain-containing protein
## VIT_12s0059g02450  -  Zinc finger (CCCH-type) family protein
## VIT_15s0046g00470  -  Dynamin-like protein 2b
## VIT_16s0013g01400  -  No hit
## VIT_12s0057g01530  -  Endomembrane protein 70, TM4 ;
## VIT_10s0003g04300  -  F-box/LRR-repeat protein 20
## VIT_06s0061g00900  -  Unknown protein
## VIT_13s0019g02310  -  Translation initiation factor eIF-3 subunit 4
## VIT_13s0074g00200  -  MRNA cap guanine-N7 methyltransferase 1
## VIT_14s0068g01260  -  Phosphate carrier protein
## VIT_03s0063g02280  -  Unknown protein
## VIT_04s0023g00440  -  Zinc finger (C2H2 type) family
## VIT_17s0000g09120  -  Ethylene-responsive DEAD box RNA helicase (RH30)
## VIT_03s0038g01340  -  Unknown protein
## VIT_05s0049g01940  -  Nuclear transport factor 2 (NTF2)
## VIT_00s0270g00110  -  No hit
## VIT_06s0009g02770  -  Pre-mRNA branch site protein p14
## VIT_05s0020g00560  -  WD-40 repeat
## VIT_16s0013g02140  -  DNA-directed RNA polymerase III subunit C4
## VIT_13s0019g03670  -  Unknown protein
## VIT_00s0229g00030  -  SEC16 homolog B
## VIT_17s0000g07880  -  Bromo-adjacenty (BAH) domain-containing protein
```

```
## VIT_03s0038g01490  -  Unknown protein
## VIT_01s0011g04530  -  Tetratricopeptide repeat protein 5
## VIT_08s0007g07050  -  Unknown protein
## VIT_08s0007g05910  -  folylpolyglutamate synthase
## VIT_14s0030g01800  -  TSO1 (chinese for 'ugly')
## VIT_00s0592g00010  -  Thymidine kinase
## VIT_01s0011g03620  -  Monoglyceride lipase
## VIT_13s0019g03330  -  Adenylosuccinate lyase
## VIT_05s0049g01230  -  Splicing factor 3B subunit 5
## VIT_17s0000g00850  -  FRIGIDA-like 2
## VIT_05s0077g00610  -  Desiccation protein PCC13-62 precursor
## VIT_13s0019g03600  -  Nuclear transcription factor Y subunit B-8
## VIT_02s0012g01100  -  Protein phosphatase 2C POLTERGEIST-like 1
## VIT_00s0198g00170  -  AAA-type ATPase
## VIT_19s0015g00180  -  Unknown protein
## VIT_05s0020g00760  -  Pyruvate dehydrogenase kinase, Mitochondrial
## VIT_08s0007g01150  -  Unc51-like kinase
## VIT_12s0059g01270  -  Drug/metabolite transporter DMT family transporter
## VIT_01s0011g03380  -  Dihydrolipoamide S-acetyltransferase (LTA2)
## VIT_19s0014g00550  -  Vacuolar sorting-associated protein (Vps27)
## VIT_08s0007g05880  -  Dehydration-induced protein (ERD15)
## VIT_05s0124g00040  -  Epsin protein
## VIT_11s0103g00610  -  Unknown protein
## VIT_02s0025g00110  -  DNA excision repair protein ERCC-3
## VIT_02s0109g00380  -  Stress response suppressor 1
## VIT_19s0014g01310  -  Rab3-GAP regulatory domain
## VIT_05s0020g01770  -  PBS1 (avrPphB susceptible 1)
## VIT_04s0044g00540  -  Pre-mRNA-splicing factor ISY1
## VIT_03s0038g02350  -  No hit
## VIT_09s0002g02100  -  DEAD box RNA helicase (RH20)
## VIT_18s0001g08180  -  Calcineurin phosphoesterase
## VIT_00s2313g00010  -  Coiled-coil protein
## VIT_18s0001g00320  -  LYS/HIS transporter 7 LHT7
## VIT_04s0008g06580  -  DNA-directed RNA polymerase II subunit B
## VIT_13s0019g00430  -  RNA recognition motif (RRM)-containing protein
## VIT_15s0046g00440  -  Phosphatidylinositol 3- and 4-kinase / ubiquitin
## VIT_00s0586g00020  -  Unknown protein
## VIT_14s0006g02860  -  Unknown protein
## VIT_07s0005g03980  -  RNA-binding protein Musashi
## VIT_12s0059g02740  -  SET domain protein
## VIT_00s0652g00020  -  Unknown protein
## VIT_08s0007g07960  -  DnaJ homolog, subfamily C, member 9
## VIT_10s0116g01910  -  ABC Transporter (VvNAP2 - VvABCI2)
## VIT_08s0007g05870  -  N-acetylglucosaminyl transferase
## VIT_12s0028g01260  -  V-type H+-transporting ATPase subunit D
## VIT_12s0028g03130  -  Proteasome 26S regulatory subunit S2 (RPN1)
## VIT_04s0023g02970  -  UPF0183 protein
## VIT_19s0177g00260  -  Unknown protein
## VIT_03s0180g00050  -  DNA-directed RNA polymerase II subunit C
## VIT_17s0000g01920  -  Nuclear transcription factor, X-box binding 1
## VIT_03s0038g02590  -  Proteasome 26S regulatory subunit S3 (RPN3)
## VIT_00s0483g00040  -  No hit
## VIT_07s0031g01860  -  Glucose-6-phosphate/phosphate translocator
## VIT_18s0072g00710  -  U6 snRNA-associated Sm-like protein LSm3
## VIT_08s0007g07150  -  Splicing factor 3B subunit 3
```

```
## VIT_00s0215g00050  -  Proteasome 26S regulatory subunit (RPN2)
## VIT_07s0095g00730  -  Sterile alpha motif (SAM) domain-containing protein
## VIT_02s0012g01790  -  Pattern formation protein (EMB30) (GNOM)
## VIT_16s0100g00500  -  SEU1 protein
## VIT_07s0005g06360  -  E3 ubiquitin-protein ligase PRT1
## VIT_14s0171g00420  -  Unknown protein
## VIT_05s0020g00030  -  WD-40 repeat
## VIT_18s0001g07300  -  MADS box interactor
## VIT_06s0080g00460  -  Nuclear transcription factor Y subunit B related
## VIT_03s0017g01770  -  Zinc finger (DHHC type)
## VIT_01s0010g01180  -  Unknown protein
## VIT_08s0007g01780  -  MRNA-capping enzyme
## VIT_12s0134g00420  -  Leucine-rich repeat family protein
## VIT_14s0108g01390  -  Ubiquitin conjugation factor E4 B
## VIT_06s0080g01240  -  MD-2 lipid recognition domain-containing protein
## VIT_19s0014g02380  -  No hit
## VIT_13s0074g00190  -  MRNA cap guanine-N7 methyltransferase 1
## VIT_04s0023g00010  -  Unknown protein
## VIT_17s0000g04620  -  Unknown protein
## VIT_05s0102g00770  -  EDR2 (enhanced disease resistance 2)
```

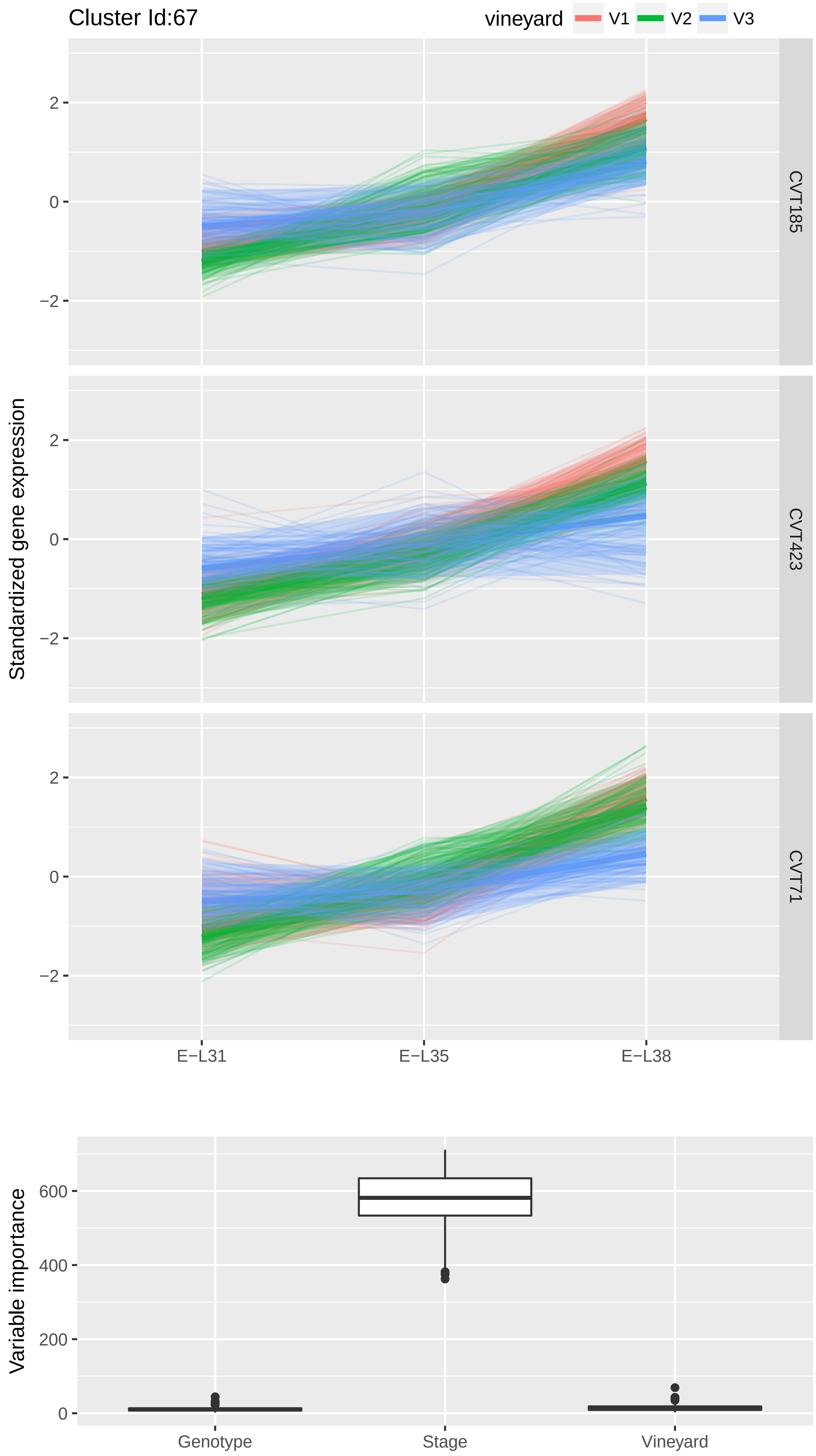
Cluster Id:67
vineyard
V1
V2
V3
Standardized gene expression
2
0
−2
CVT185
CVT423
CVT71
E−L31
E−L35
E−L38
Variable importance
600
400
200
0
Genotype
Stage
Vineyard

Cluster no. 68

```
## Number of genes in the cluster: 229
## Homogeneity Index:    0.81
## Variable importance for Stage:    Median =  334.2  - Rank =  66
## Variable importance for Clone:    Median =  23.23  - Rank =  31
## Variable importance for Vineyard: Median =  31.18  - Rank =  65
##
## Gene ID               Gene Annotation
## VIT_00s0541g00020  -  Basic Leucine Zipper Transcription Factor (VvbZIP55)
## VIT_07s0031g01160  -  Hyperosmotically inducible periplasmic protein
## VIT_06s0004g04480  -  SLT1 (sodium- and lithium-tolerant 1)
## VIT_14s0171g00180  -  Leucine-rich repeat transmembrane protein kinase
## VIT_12s0028g02210  -  Riboflavin kinase
## VIT_11s0016g02760  -  No hit
## VIT_04s0044g00030  -  ATSAC1B/IBS2 (impaired in baba-induced sterility 2)
## VIT_02s0025g03570  -  DNA binding / hydrolase, acting on ester bonds
## VIT_08s0040g00970  -  Plectin (myosin-like)
## VIT_18s0001g02250  -  Ras-related protein Rab-7A
## VIT_02s0025g04990  -  Unknown protein
## VIT_01s0010g00060  -  Unknown protein
## VIT_00s0934g00010  -  RARE-cold-inducible 2B
## VIT_18s0001g10360  -  Unknown protein
## VIT_09s0018g01980  -  Unknown
## VIT_14s0068g01860  -  Unknown protein
## VIT_07s0005g01240  -  Triacylglycerol lipase
## VIT_04s0008g01360  -  Tassel serine threonine kinase 1
## VIT_06s0009g01880  -  Vacuolar protein sorting-associated protein 26
## VIT_18s0072g00370  -  Cellulose synthase CESA2
## VIT_04s0008g02030  -  Unknown protein
## VIT_03s0088g01220  -  Unknown protein
## VIT_08s0040g02640  -  Serine/threonine-protein kinase AFC1
## VIT_06s0004g00300  -  Hcr2-p4.1
## VIT_15s0024g00820  -  Stress-induced protein
## VIT_14s0108g00900  -  Pectinesterase family
## VIT_12s0057g01370  -  Unknown protein
## VIT_17s0000g00730  -  ATP binding / DNA binding
## VIT_18s0001g09700  -  F-box protein GID2
## VIT_05s0049g01050  -  Unknown
## VIT_03s0038g04450  -  Basic Leucine Zipper Transcription Factor (VvbZIP07)
## VIT_09s0018g01540  -  Unknown protein
## VIT_07s0005g00990  -  Dehydration-induced 19
## VIT_13s0064g01250  -  Gamma response 1
## VIT_01s0150g00350  -  Zinc finger (DHHC type)
## VIT_17s0000g06200  -  Mini zinc finger 1 MIF1
## VIT_14s0083g00360  -  Unknown protein
## VIT_11s0052g00630  -  Metallothionein
## VIT_05s0062g00850  -  Auxin-responsive protein
## VIT_04s0008g00530  -  Serine/threonine-protein kinase Aurora-1
## VIT_00s0199g00050  -  2-oxoglutarate/malate carrier protein, Mitochondrial
## VIT_03s0017g01490  -  Berberine bridge enzyme
## VIT_13s0074g00550  -  Amino acid transporter family
## VIT_04s0023g03690  -  Proteasome 20S alpha subunit D2 (PAD2) (PRS1) (PRC6)
## VIT_06s0009g03550  -  Regulator of chromosome condensation (RCC1)
## VIT_10s0116g00370  -  Unknown protein
## VIT_11s0118g00350  -  Vacuolar pyrophosphatase
```

```
## VIT_16s0100g00400  -  ERF/AP2 Gene Family (VvERF019),Dehydration Responsive Element-Binding
## VIT_07s0031g01630  -  Unknown protein
## VIT_14s0066g00670  -  MAP kinase kinase 3
## VIT_10s0003g01270  -  WAVE1 (WASP (Wiskott-Aldrich syndrome protein)
## VIT_19s0138g00120  -  1,3-beta-glucan synthase
## VIT_05s0049g01410  -  Phosphate transporter 1
## VIT_07s0005g00980  -  Dehydration-induced 19
## VIT_18s0001g15730  -  Dof zinc finger protein DOF3.5
## VIT_07s0130g00140  -  Thioesterase family
## VIT_07s0005g00240  -  Transducin protein
## VIT_05s0049g00150  -  SWI/SNF matrix-associated regulator of chromatin sbfamily A mber 3 3
## VIT_18s0001g00360  -  Dehydrin (VvDHN2)
## VIT_11s0016g03880  -  Receptor protein kinase PERK1
## VIT_11s0016g05530  -  Plastocyanin domain-containing protein
## VIT_04s0008g06000  -  ERF/AP2 Gene Family (VvERF045)
## VIT_14s0060g00090  -  SNARE YKT6 1
## VIT_13s0019g02210  -  Sterol 4-alpha-methyl-oxidase 1 (SMO1)
## VIT_00s0274g00090  -  Quinone oxidoreductase
## VIT_18s0001g06340  -  RPS4 (resistant to p. syringae 4)
## VIT_18s0001g12830  -  1,4-beta-mannan endohydrolase
## VIT_07s0005g06620  -  Unknown protein
## VIT_07s0005g04510  -  DENN (AEX-3) domain-containing
## VIT_15s0021g00220  -  ABC Transporter (VvMRP10 - VvABCC10)
## VIT_03s0038g02860  -  Amino acid permease
## VIT_07s0005g03620  -  Serine/threonine kinase 19 isoform 1 STK19
## VIT_18s0001g03200  -  Emp24/gp25L/p24
## VIT_17s0000g02880  -  S-adenosyl-L-methionine:salicylic acid carboxyl methyltransferase
## VIT_05s0020g03030  -  Leucine-rich repeat family protein
## VIT_07s0005g05600  -  UDP-glucose:sterol 3-O-glucosyltransferase
## VIT_07s0005g06610  -  Unknown protein
## VIT_15s0045g00550  -  Unknown protein
## VIT_05s0062g01270  -  Cyclo-DOPA 5-O-glucosyltransferase
## VIT_03s0063g01290  -  Gibberellin 20 oxidase 2
## VIT_00s0252g00050  -  Unknown protein
## VIT_04s0044g01610  -  CYP82C1p
## VIT_05s0124g00280  -  Anthocyanin membrane protein 1
## VIT_01s0010g03750  -  Unknown protein
## VIT_13s0067g03520  -  No hit
## VIT_09s0054g00890  -  V-type H+-transporting ATPase subunit A
## VIT_00s0216g00060  -  Nuclear transport factor 2 (NTF2)
## VIT_19s0085g01100  -  ECT8 (evolutionarily conserved C-terminal region 8)
## VIT_01s0146g00410  -  GEM-like protein 5
## VIT_15s0024g00920  -  NFD4 (nuclear fusion defective 4)
## VIT_03s0180g00040  -  Cyclin D3_2
## VIT_01s0011g01510  -  KUP11 (K+ uptake permease 11)
## VIT_11s0016g05190  -  Phosphate translocator-related
## VIT_18s0001g08710  -  Basic Leucine Zipper Transcription Factor (VvbZIP44)
## VIT_18s0001g11260  -  Nucleobase-ascorbate transporter 3 (NAT3)
## VIT_11s0016g02300  -  Nucleotide pyrophosphatase
## VIT_12s0028g00960  -  phytoene synthase (PSY) (VvPSY2)
## VIT_05s0077g00810  -  Sodium-inducible calcium-binding protein (ACP1)
## VIT_08s0007g02840  -  Unknown protein
## VIT_11s0037g01230  -  basic helix-loop-helix (bHLH) family
## VIT_15s0048g02720  -  fatty acid elongase
```

```
## VIT_06s0009g00130  -  No hit
## VIT_15s0048g02730  -  Ribosome biogenesis regulatory protein (RRS1)
## VIT_04s0023g02730  -  Nodulin MtN21 family
## VIT_08s0058g01460  -  1,4-beta-mannan endohydrolase
## VIT_03s0088g01200  -  Unknown protein
## VIT_13s0067g02560  -  Unknown protein
## VIT_15s0045g00620  -  Unknown protein
## VIT_11s0016g03130  -  Ceramide glucosyltransferase
## VIT_13s0019g01100  -  Unknown protein
## VIT_08s0040g02610  -  Unknown
## VIT_15s0048g00800  -  Guanine nucleotide exchange MIN7
## VIT_18s0001g02790  -  Unknown protein
## VIT_14s0060g00720  -  ABC Transporter (VvNAP6 - VvABCI6)
## VIT_08s0007g01470  -  Copper transporter 3
## VIT_00s0662g00030  -  Dehydration Responsive Element-Binding Transcription Factor (VvDREB37)
## VIT_00s0662g00040  -  Dehydration Responsive Element-Binding Transcription Factor (VvDREB38)
## VIT_05s0077g00280  -  Beta-amylase
## VIT_02s0012g02810  -  CYP76C4
## VIT_14s0068g00370  -  No hit
## VIT_02s0109g00230  -  Early-responsive to dehydration protein / ERD protein
## VIT_12s0059g01300  -  Unknown protein
## VIT_01s0026g01020  -  Binding
## VIT_13s0074g00720  -  Neutral/alkaline invertase
## VIT_03s0063g00310  -  ABA-responsive element-binding protein ABF4 (VvABF-8), Basic Leucine Z
## VIT_17s0000g07780  -  Calmodulin-binding protein
## VIT_14s0068g00520  -  F-box domain containing protein
## VIT_13s0101g00100  -  Unknown protein
## VIT_13s0073g00670  -  Unknown protein
## VIT_18s0001g13080  -  Unknown protein
## VIT_17s0000g03200  -  Unknown protein
## VIT_01s0127g00590  -  Protein disulfide isomerase
## VIT_16s0098g00760  -  NAC domain-containing protein (VvNAC71)
## VIT_14s0068g00400  -  Hydrolase, alpha/beta fold family
## VIT_18s0001g08490  -  Early-responsive to dehydration
## VIT_05s0020g03980  -  Calmodulin-binding region IQD19
## VIT_12s0059g01060  -  Hydroperoxide lyase (HPL1)
## VIT_19s0014g01890  -  Reticulon-4-interacting protein 1
## VIT_19s0138g00110  -  1,3-beta-glucan synthase
## VIT_07s0005g04900  -  Unknown protein
## VIT_12s0059g00670  -  NADP-dependent oxidoreductase
## VIT_12s0059g01650  -  Division site determinant MinD, Plastid
## VIT_07s0031g00700  -  Caltractin / centrin
## VIT_19s0014g01780  -  Basic Leucine Zipper Transcription Factor (VvbZIP52)
## VIT_04s0069g00750  -  AHK5 (Cytokinin independent 2)
## VIT_10s0597g00020  -  UBA; HSC20, C-terminal oligomerisation
## VIT_18s0001g02780  -  Lipase GDSL
## VIT_10s0003g04050  -  Unknown protein
## VIT_18s0001g14660  -  ABC Transporter (VvMDR3 - VvABCB3)
## VIT_19s0014g05310  -  Sec23/sec24 transport
## VIT_01s0011g04250  -  Zinc finger (B-box type)
## VIT_14s0083g01160  -  COBRA protein
## VIT_04s0023g02050  -  Inorganic pyrophosphatase
## VIT_01s0026g01460  -  Thioredoxin H-type 2 (Trx-H-2)
## VIT_19s0014g01390  -  Phosphoenolpyruvate carboxylase
## VIT_08s0007g04550  -  NCS1 nucleoside transporter family protein
```

```
## VIT_05s0049g00770  -  No hit
## VIT_05s0049g00840  -  No hit
## VIT_03s0038g04600  -  Endoplasmatic reticulum retrieval protein 1B
## VIT_10s0003g01480  -  BIN2 (brassinosteroid-insensitive 2)
## VIT_00s0199g00040  -  No hit
## VIT_06s0061g00560  -  Exostosin FRA8 (Fragile fiber8)
## VIT_08s0007g04580  -  UGT73C2 (UDP-glucosyl transferase 73C2)
## VIT_03s0038g03570  -  Monocopper oxidase SKS5 (SKU5 Similar 5)
## VIT_13s0067g00660  -  Steroid 23-alpha-hydroxylase
## VIT_06s0061g00550  -  Xyloglucan endotransglucosylase/hydrolase 32
## VIT_08s0007g07330  -  No hit
## VIT_04s0023g03420  -  Calcium-dependent protein kinase (VvCPK3)
## VIT_01s0026g02430  -  Calcineurin B
## VIT_02s0025g04920  -  Phosphate translocator protein2, Plastidic
## VIT_17s0000g08210  -  Zinc finger (C3HC4-type ring finger)
## VIT_00s0274g00080  -  Benzoquinone reductase
## VIT_14s0108g00480  -  basic helix-loop-helix (bHLH) family
## VIT_14s0030g01880  -  Calmodulin-binding region IQD26
## VIT_10s0003g01410  -  CBL-interacting protein kinase 20 (CIPK20)
## VIT_12s0055g00910  -  Kelch repeat-containing F-box protein
## VIT_18s0001g13060  -  C3H2C3 ring-finger protein
## VIT_11s0052g00320  -  Ca2+-ATPase 10 ACA10, plasma membrane
## VIT_13s0074g00390  -  CYP77A2
## VIT_19s0177g00230  -  Unknown
## VIT_16s0013g02100  -  2-phosphoglycerate kinase
## VIT_19s0090g01360  -  No hit
## VIT_05s0102g01200  -  Lectin
## VIT_17s0000g06300  -  No hit
## VIT_18s0122g01200  -  Pentatricopeptide (PPR) repeat-containing protein
## VIT_15s0024g00910  -  NFD4 (nuclear fusion defective 4)
## VIT_03s0091g00260  -  Zinc finger protein 4
## VIT_14s0030g01740  -  <U+03B6>-carotene desaturase (ZDS) (VvZDS1)
## VIT_12s0057g01470  -  RAB GTPase RAB2
## VIT_17s0000g09210  -  No pollen (Osnop)
## VIT_08s0007g03560  -  Anthocyanin membrane protein 1 (Anm1)
## VIT_18s0001g02810  -  Acyl-CoA synthetase long-chain member 9
## VIT_06s0080g00560  -  Beta-alanine synthase
## VIT_05s0020g01490  -  Rapid ALkalinization Factor RALFL33
## VIT_06s0004g00590  -  Lysine decarboxylase
## VIT_01s0010g01830  -  Regulator of nonsense transcripts 1
## VIT_09s0018g01700  -  Unknown protein
## VIT_18s0001g08270  -  Amino acid permease 6
## VIT_07s0005g01950  -  myb domain protein 78
## VIT_07s0005g04410  -  Subtilase
## VIT_01s0010g02200  -  Zinc finger (C3HC4-type ring finger)
## VIT_11s0016g00330  -  Pectinesterase family
## VIT_15s0048g02390  -  Wall-associated receptor kinase-like 14
## VIT_16s0039g02210  -  ER lumen protein retaining receptor
## VIT_02s0025g03730  -  Translation initiation factor eIF-2B alpha subunit
## VIT_07s0005g01700  -  Transcription regulator
## VIT_18s0122g01440  -  AMP-dependent synthetase and ligase
## VIT_10s0003g04950  -  Esterase/lipase/thioesterase
## VIT_13s0067g00140  -  Proline-rich family protein
## VIT_07s0005g02200  -  Autophagy 8i (APG8i)
## VIT_13s0064g00030  -  Choline transporter
```

```
## VIT_13s0067g03500  -  Protein phosphatase 2C
## VIT_08s0058g01210  -  Non-specific lipid transfer protein LTP1
## VIT_17s0000g00430  -  basic helix-loop-helix (bHLH) family
## VIT_03s0088g01150  -  Squalene monooxygenase
## VIT_08s0007g08330  -  Polygalacturonase PG1
## VIT_01s0011g04240  -  Zinc finger (B-box type)
## VIT_13s0019g04610  -  Salt stress-inducible protein kinase
## VIT_04s0008g04490  -  MARD1 (mediator of ABA-regulated dormancy 1)
## VIT_17s0000g00830  -  Nodulin MtN3 family
## VIT_17s0000g00010  -  MtN19
## VIT_08s0058g01480  -  1,4-beta-mannan endohydrolase
## VIT_03s0038g03940  -  Tonoplast monosaccharide transporter2
## VIT_08s0007g00480  -  Oxysterol binding protein
## VIT_18s0041g00680  -  Endomembrane protein 70
## VIT_00s0271g00110  -  flavodoxin-like quinone reductase 1
## VIT_13s0019g04750  -  Kinesin morphogenesis of root hair 2 (MRH2)
## VIT_07s0031g00800  -  Cyclin-dependent kinase inhibitor KIP-related protein 7
## VIT_11s0016g04160  -  Sulfate transporter 3.5
```

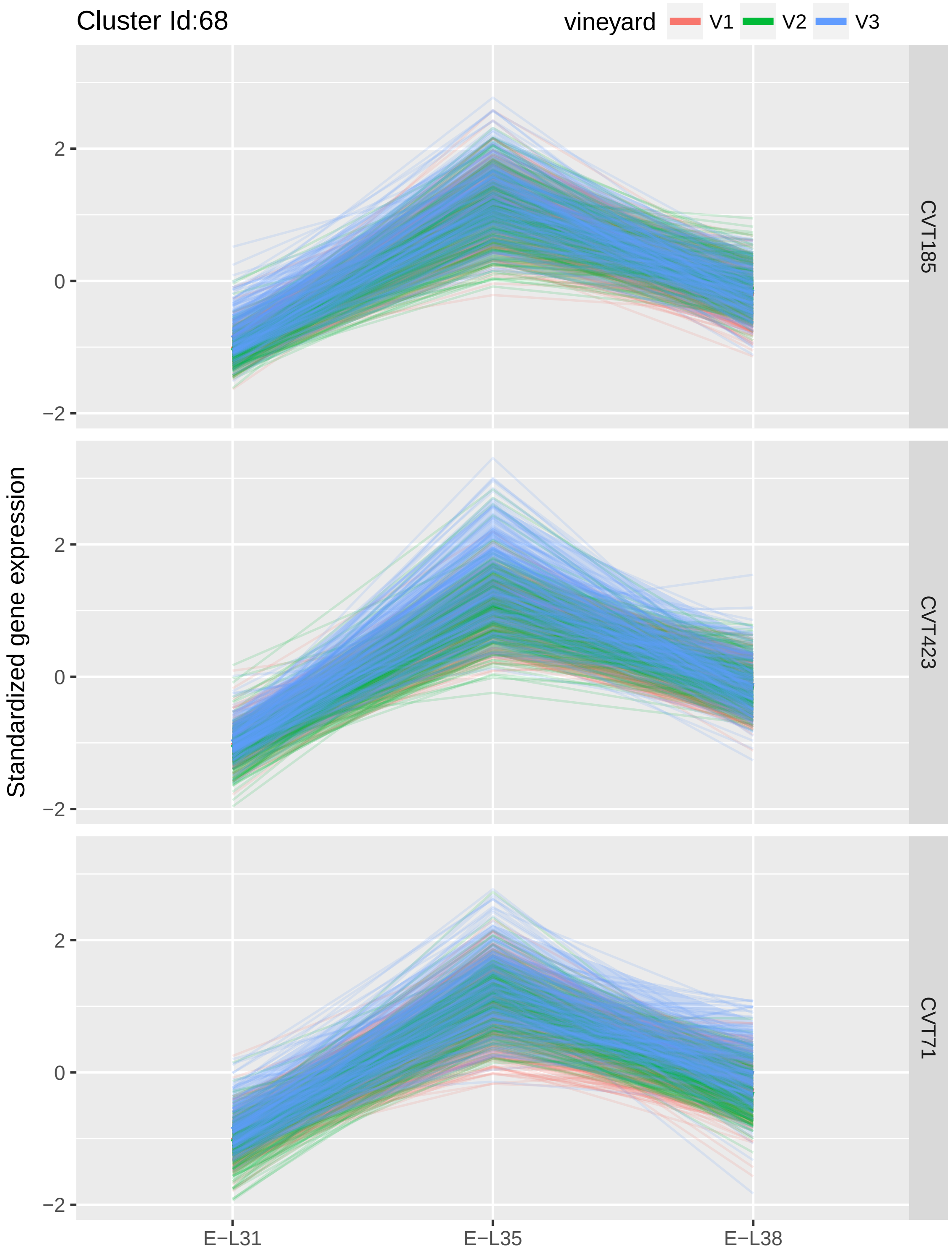
Cluster Id:68
vineyard
V1
V2
V3
Standardized gene expression
CVT185
CVT423
CVT71
2
0
−2
E−L31
E−L35
E−L38

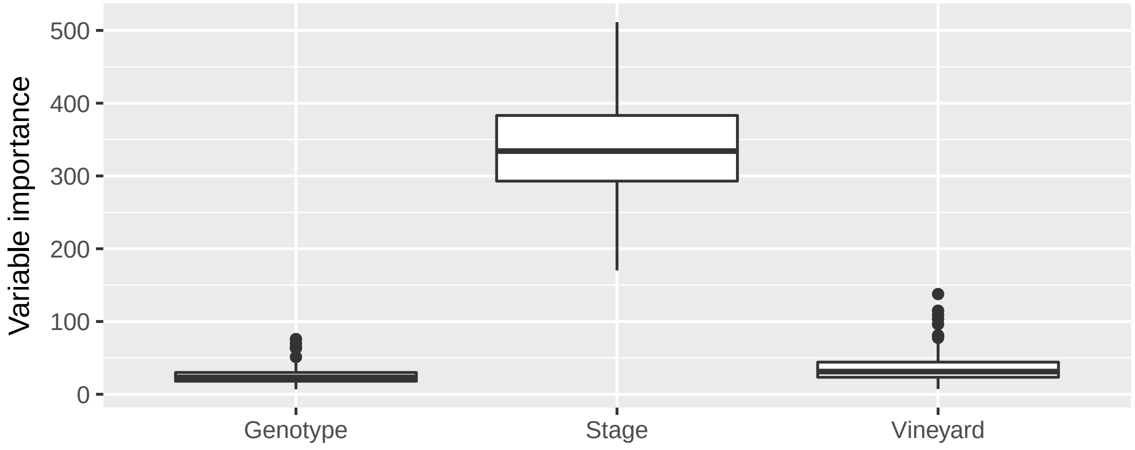
Variable importance
500
400
300
200
100
0
Genotype
Stage
Vineyard

Cluster no. 69

```
## Number of genes in the cluster: 219
## Homogeneity Index:    0.82
## Variable importance for Stage:    Median =  642.7  - Rank =  7
## Variable importance for Clone:    Median =   11.9  - Rank =  96
## Variable importance for Vineyard: Median =  11.96  - Rank =  110
##
## Gene ID               Gene Annotation
## VIT_06s0004g04020  -  Ribosomal protein L35 (RPL35D) 60S
## VIT_14s0030g02020  -  Aldo/keto reductase
## VIT_03s0063g00830  -  Carboxyesterase 5 CXE5
## VIT_09s0002g04150  -  Isochorismatase protein rutB
## VIT_15s0046g00040  -  Calcium-transporting ATPase 2, endoplasmic reticulum-type ECA2
## VIT_18s0001g10620  -  No hit
## VIT_13s0067g00120  -  GCN5 N-acetyltransferase (GNAT)
## VIT_10s0116g01800  -  Calcium Dependent Protein Kinase (VvCPK12)
## VIT_11s0103g00550  -  Subtilisin-like serine protease 3
## VIT_12s0035g01260  -  NBS-LRR type disease resistance protein
## VIT_08s0032g00440  -  Syntaxin 1B/2/3/4
## VIT_04s0008g07360  -  ADP-ribosylation factor 3 (ARF3)
## VIT_19s0135g00100  -  Ribosomal protein L18A (RPL18aB) 60S
## VIT_04s0079g00200  -  Auxin response factor 8
## VIT_03s0038g00320  -  Unknown protein
## VIT_04s0008g01960  -  Copper-transporting ATPase PAA2
## VIT_11s0052g00020  -  Balbiani ring 1
## VIT_14s0006g02840  -  Catalytic
## VIT_08s0007g08970  -  Phosphate/phosphoenolpyruvate translocator
## VIT_15s0107g00310  -  EF hand
## VIT_03s0097g00620  -  N-acylethanolamine amidohydrolase
## VIT_13s0019g02090  -  Thylakoid lumenal 15 kDa protein 1
## VIT_15s0048g02630  -  RNA binding motif protein 38
## VIT_04s0023g01170  -  Unknown protein
## VIT_06s0004g07380  -  Acyl-carrier protein S-malonyltransferase
## VIT_00s0199g00300  -  Protein transport protein SFT1
## VIT_05s0062g01020  -  No hit
## VIT_12s0121g00010  -  Ribosomal protein L29 50S
## VIT_11s0037g00820  -  WD-40 repeat protein family
## VIT_03s0063g00850  -  RNA-binding protein 47B (ATRBP47B)
## VIT_07s0005g02170  -  Unknown protein
## VIT_05s0020g00430  -  Unknown protein
## VIT_12s0057g00300  -  Unknown protein
## VIT_08s0007g01660  -  Chaperone protein dnaJ-related
## VIT_07s0031g00790  -  Steroid nuclear receptor, ligand-binding
## VIT_10s0003g01400  -  MRH1 (morphogenesis of root hair 1)
## VIT_08s0007g07700  -  Unknown protein
## VIT_00s1644g00010  -  S-locus protein kinase
## VIT_00s0598g00040  -  Unknown protein
## VIT_07s0104g00220  -  Phenazine biosynthesis PhzC/PhzF
## VIT_18s0001g06240  -  R protein L6
## VIT_03s0038g01600  -  ABA-responsive protein (HVA22a)
## VIT_08s0105g00250  -  Aluminum activated malate transporter 1
## VIT_04s0043g00330  -  Unknown protein
## VIT_19s0014g02740  -  Metallothionein
## VIT_19s0014g02230  -  Ribosomal protein S20, Chloroplast 30S
## VIT_13s0106g00380  -  Phosphoacetylglucosamine mutase
```

```
## VIT_05s0020g03590  -  Unknown protein
## VIT_19s0015g00970  -  Unknown protein
## VIT_09s0002g01550  -  Lecithine cholesterol acyltransferase
## VIT_18s0001g04760  -  Ribosomal protein L30 (RPL30B) 60S
## VIT_04s0008g01450  -  Cyclopropane-fatty-acyl-phospholipid synthase
## VIT_11s0037g00180  -  Leucine aminopeptidase 3, chloroplast precursor
## VIT_12s0028g01970  -  Serine/threonine-protein kinase NAK
## VIT_00s0341g00030  -  Unknown protein
## VIT_17s0000g08200  -  Phosphoglycerate/bisphosphoglycerate mutase
## VIT_15s0046g00110  -  GHMP kinase
## VIT_00s0218g00050  -  Kinesin motor protein
## VIT_13s0067g00730  -  Carboxylic ester hydrolase
## VIT_05s0051g00940  -  Bet1 SNARE 1-1
## VIT_01s0026g02480  -  CP12-2
## VIT_14s0066g01840  -  SAG101 (senescence-associated gene 101)
## VIT_07s0031g01150  -  FK506-binding protein genes family (VvFKBP16-2)
## VIT_01s0011g03220  -  Choline transporter
## VIT_09s0002g00210  -  Endonuclease
## VIT_02s0087g00160  -  Unknown
## VIT_12s0028g02220  -  Ubiquitin-conjugating enzyme E2 N
## VIT_11s0016g01760  -  Cleavage and polyadenylation specificity factor 5
## VIT_08s0007g01330  -  Unknown protein
## VIT_09s0002g09090  -  Ser/thr specific protein kinase
## VIT_07s0005g04610  -  Alcohol dehydrogenase class III
## VIT_06s0004g08460  -  CBS1
## VIT_04s0008g05010  -  Unknown protein
## VIT_11s0016g04860  -  Ethylene-responsive element-binding protein EREBP-4
## VIT_11s0118g00670  -  Ribosomal protein L14 (RPL14B) 60S
## VIT_03s0180g00140  -  Acetyl xylan esterase AxeA
## VIT_10s0003g05660  -  Unknown protein
## VIT_17s0000g10020  -  1,3-beta-glucan synthase
## VIT_16s0039g00300  -  Unknown protein
## VIT_02s0012g02420  -  TRNA pseudouridine synthase
## VIT_01s0010g01760  -  Remorin
## VIT_16s0098g01350  -  Oligopeptide transporter 6
## VIT_12s0028g01110  -  Phytochrome- interacting  factor 5 PIL6
## VIT_18s0001g15580  -  Glycogenin glucosyltransferase (glycogenin)
## VIT_08s0007g01500  -  DNA binding / nuclease
## VIT_13s0019g04880  -  No hit
## VIT_00s2085g00010  -  Leucine-rich repeat family protein
## VIT_00s0912g00010  -  Unknown protein
## VIT_07s0104g01390  -  Arsenate reductase (glutaredoxin)
## VIT_06s0004g00730  -  Ubiquitin-conjugating enzyme E2 D/E
## VIT_18s0001g06410  -  Ribosomal protein 60S
## VIT_10s0003g04970  -  ATPase, BadF/BadG/BcrA/BcrD-type
## VIT_11s0065g00640  -  Cycloartenol synthase
## VIT_10s0003g01280  -  Ribulose bisphosphate carboxylase, large chain
## VIT_18s0001g12370  -  Glucose-6-phosphate isomerase, cytosolic (PGIC)
## VIT_02s0012g00890  -  Unknown protein
## VIT_15s0048g00660  -  Autophagy 8f (APG8f)
## VIT_14s0068g01550  -  Unknown protein
## VIT_01s0010g02180  -  Myosin-related
## VIT_03s0063g02290  -  Unknown protein
## VIT_10s0003g02590  -  SH3 domain-containing protein 3 (SH3P3)
## VIT_09s0002g03620  -  NADP-dependent malic enzyme
```

```
## VIT_15s0046g00100  -  WNK kinase 6
## VIT_09s0002g02290  -  Beta-galactosidase
## VIT_08s0007g09010  -  Esterase
## VIT_01s0026g02070  -  Peptidyl-prolyl cis-trans isomerase cyclophilin-type
## VIT_11s0052g00290  -  Ribosomal protein L7 (RPL7D) 60S
## VIT_17s0000g07280  -  Histone deacetylase HDA05
## VIT_14s0036g00850  -  Endoribonuclease L-PSP
## VIT_09s0002g07420  -  No hit
## VIT_01s0010g02140  -  Z-ring ARC3
## VIT_05s0077g01070  -  Universal stress protein (USP) family protein
## VIT_10s0003g01640  -  Transcription repressor
## VIT_01s0011g06550  -  Na+/H+ antiporter SOS1
## VIT_07s0005g01440  -  Unknown protein
## VIT_17s0000g07790  -  N-hydroxythioamide S-beta-glucosyltransferase
## VIT_15s0045g01120  -  6-phosphofructokinase
## VIT_14s0060g02060  -  Tetratricopeptide repeat (TPR)-containing
## VIT_14s0060g02350  -  Lysyl-tRNA synthetase
## VIT_09s0002g00810  -  S-2-hydroxy-acid oxidase, peroxisomal
## VIT_13s0019g01010  -  ABA2 (ABA deficient 2)
## VIT_17s0000g04690  -  Protease SppA
## VIT_16s0039g02490  -  Phospholipid scramblase 1
## VIT_14s0108g01560  -  Alpha-1,4 glucan phosphorylase, L isozyme, chloroplast precursor
## VIT_04s0043g01040  -  No hit
## VIT_08s0007g07090  -  Sphingosine-1-phosphate phosphatase
## VIT_07s0130g00130  -  Calcium-dependent protein kinase (VvCPK7)
## VIT_11s0016g00170  -  Guanine nucleotide exchange MIN7
## VIT_19s0027g01070  -  R protein disease resistance protein
## VIT_11s0016g01910  -  THO complex subunit 6
## VIT_04s0008g05480  -  BRI1-KD interacting protein 118
## VIT_13s0064g00590  -  Cyclin-related
## VIT_07s0031g00540  -  Rapid ALkalinization Factor RALFL34
## VIT_14s0068g01870  -  Nodulin
## VIT_08s0040g01360  -  Histidyl-tRNA synthetase
## VIT_08s0007g00350  -  Import inner membrane translocase subunit Tim9, Mitochondrial
## VIT_10s0116g00160  -  Bromo-adjacenty (BAH) domain-containing protein
## VIT_19s0014g02730  -  Cytochrome c oxidase subunit XVII assembly protein
## VIT_06s0004g00040  -  Pur alpha-1 (purin-rich alpha 1)
## VIT_02s0025g02780  -  Ribosomal protein P1  acidic 60S
## VIT_14s0030g00320  -  Sugar transporter ERD6-like 5
## VIT_03s0038g04490  -  Diaminopimelate decarboxylase
## VIT_07s0031g01730  -  Iron-sulfur assembly protein IscA, chloroplast precursor
## VIT_15s0048g02550  -  Pentatricopeptide (PPR) repeat
## VIT_01s0011g03080  -  Methionine aminopeptidase 1B, chloroplast precursor
## VIT_04s0079g00160  -  Auxin response factor 8
## VIT_13s0067g02060  -  Biotin/lipoyl attachment domain-containing protein
## VIT_05s0094g00870  -  Ribosomal protein L5 (RPL5B) 60S
## VIT_00s2569g00010  -  Sialidase BNR/Asp-box repeat family protein
## VIT_02s0025g05150  -  TFIIH basal transcription factor complex TTD-A subunit
## VIT_12s0055g01100  -  Beta-hydroxyacyl-ACP dehydratase
## VIT_09s0054g00960  -  No hit
## VIT_00s0322g00020  -  HHP4 (heptahelical protein 4)
## VIT_16s0050g02330  -  Regulator of nonsense transcripts 1
## VIT_17s0000g09200  -  Unknown protein
## VIT_04s0044g00520  -  BolA
## VIT_12s0057g00940  -  Glucose-6-phosphate 1-dehydrogenase 2, chloroplast precursor
```

```
## VIT_18s0001g08020  -  Unknown protein
## VIT_08s0007g06310  -  myb family
## VIT_01s0010g02280  -  Unknown protein
## VIT_02s0012g01220  -  Unknown protein
## VIT_19s0014g02680  -  Ribosomal protein S2
## VIT_19s0014g01430  -  3-phosphoshikimate 1-carboxyvinyltransferase, chloroplast precursor
## VIT_19s0014g01350  -  Ribulose bisphosphate carboxylase, large chain
## VIT_04s0044g00950  -  ATXR5 (setdomain group 15)
## VIT_07s0031g02970  -  Unknown protein
## VIT_11s0065g00710  -  Cleavage stimulation factor subunit 3
## VIT_13s0156g00450  -  R protein disease resistance protein
## VIT_15s0021g00610  -  Histone deacetylase 9
## VIT_01s0127g00150  -  Menaquinone biosynthesis methyltransferase ubiE
## VIT_09s0002g03660  -  Retrotransposon protein, Unclassified
## VIT_05s0049g01310  -  Ribosomal protein L14 (RPL14B) 60S
## VIT_15s0021g01180  -  Tubulin-specific chaperone A
## VIT_19s0015g00390  -  Unknown protein
## VIT_16s0098g00410  -  Receptor kinase homolog LRK10
## VIT_15s0046g02830  -  Unknown
## VIT_06s0009g03810  -  ABH1 (ABA hypersensitive 1)
## VIT_14s0108g01160  -  Latex abundant protein 1
## VIT_05s0077g02060  -  Ribosomal protein L18 (RPL18B) 60S
## VIT_08s0007g08760  -  Thioredoxin reductase
## VIT_08s0007g06080  -  Beta 1-3 glucanase [Vitis vinifera]
## VIT_11s0037g00930  -  GDP-L-fucose synthase.
## VIT_17s0000g10010  -  Callose synthase catalytic subunit
## VIT_10s0003g00640  -  D-tyrosyl-tRNA(Tyr) deacylase
## VIT_00s0612g00030  -  Unknown protein
## VIT_05s0020g01470  -  Aminoacyl-tRNA synthetase
## VIT_02s0025g01080  -  No hit
## VIT_03s0038g02810  -  Unknown protein
## VIT_19s0027g01060  -  Tetratricopeptide repeat (TPR)-containing
## VIT_03s0063g01020  -  Armadillo/beta-catenin repeat
## VIT_02s0241g00140  -  Calmodulin (A)
## VIT_07s0005g04600  -  Alcohol dehydrogenase class III
## VIT_18s0001g11150  -  Acyl-peptide hydrolase
## VIT_02s0241g00160  -  Esterase/lipase/thioesterase family
## VIT_04s0023g01520  -  Unknown protein
## VIT_15s0046g03580  -  (+)-neomenthol dehydrogenase
## VIT_10s0003g01520  -  Ribosomal protein L11 (RPL11D) 60S
## VIT_14s0068g00750  -  C2 domain-containing protein
## VIT_12s0035g01330  -  RPS2 (resistant to p. syringae 2)
## VIT_18s0001g06010  -  Hydrolase, alpha/beta fold
## VIT_17s0119g00350  -  3,4-dihydroxy-2-butanone kinase
## VIT_01s0026g01770  -  Ubiquitin-conjugating enzyme E2 A
## VIT_01s0026g00340  -  Threonyl-tRNA synthetase
## VIT_17s0000g03520  -  Receptor kinase RK20-1
## VIT_06s0061g00750  -  Superoxide dismutase [Cu-Zn], chloroplast precursor
## VIT_14s0128g00220  -  Signal peptidase complex subunit 3
## VIT_11s0016g05610  -  Translation elongation factor Ts (EF-Ts)
## VIT_03s0088g00410  -  Pyruvate kinase isozyme A, chloroplast precursor
## VIT_13s0067g00400  -  Anthranilate synthase alpha 1
## VIT_12s0028g03490  -  Dihydroorotase
## VIT_06s0004g01070  -  No hit
## VIT_13s0019g04910  -  Pectate lyase
```

```
## VIT_06s0009g03240  -  Pleckstrin homology (PH) domain-containing
## VIT_00s0214g00130  -  F-box family protein
## VIT_07s0005g02410  -  Peptidyl-prolyl cis-trans isomerase, chloroplast ROC4 (rotamase CyP 4)
## VIT_18s0041g00090  -  Cation efflux MTP11
## VIT_06s0004g05530  -  Serine/threonine protein phosphatase PP1
## VIT_09s0002g05180  -  No hit
## VIT_10s0003g03070  -  Beta-ketoacyl-ACP synthase precursor
```

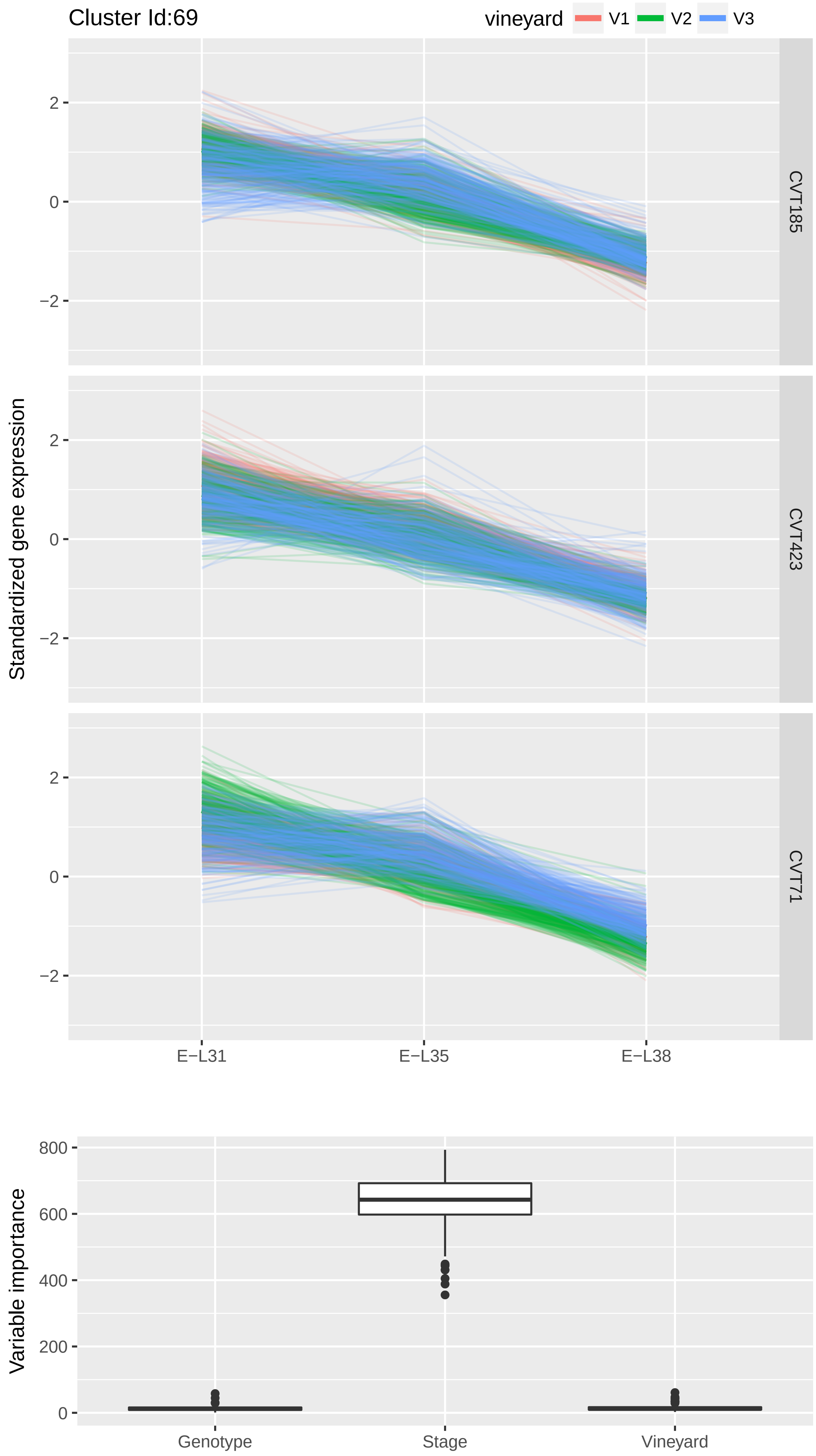
Cluster Id:69
vineyard
V1
V2
V3
Standardized gene expression
2
0
−2
CVT185
CVT423
CVT71
E−L31
E−L35
E−L38
Variable importance
800
600
400
200
0
Genotype
Stage
Vineyard

Cluster no. 70

```
## Number of genes in the cluster: 106
## Homogeneity Index:    0.63
## Variable importance for Stage:    Median =  339.8  - Rank =  64
## Variable importance for Clone:    Median =  14.94  - Rank =  73
## Variable importance for Vineyard: Median =  66.35  - Rank =  29
##
## Gene ID               Gene Annotation
## VIT_05s0020g02830  -  Unknown protein
## VIT_03s0038g00380  -  Xylulose kinase
## VIT_08s0007g08740  -  No hit
## VIT_14s0006g03120  -  HEAT repeat-containing protein
## VIT_08s0032g01140  -  Unknown protein
## VIT_00s0265g00070  -  Ubiquitin-conjugating enzyme E2 O
## VIT_11s0016g01780  -  HAB1 (homology toABI1) (VvPP2C-1)
## VIT_14s0060g01620  -  Single-strand DNA binding protein
## VIT_17s0000g08820  -  Nucleoside phosphatase GDA1/CD39
## VIT_09s0002g04690  -  Pentatricopeptide (PPR) repeat-containing protein
## VIT_17s0000g06630  -  Unknown protein
## VIT_09s0070g00580  -  Diaminopimelate decarboxylase
## VIT_07s0031g01780  -  TRNA-histidine guanylyltransferase
## VIT_14s0030g01580  -  AIN1 (ACC insensitive 1) 5'-3' exoribonuclease (XRN4)
## VIT_11s0037g00560  -  Unknown protein
## VIT_12s0035g01100  -  KH domain-containing quaking protein
## VIT_13s0106g00690  -  No hit
## VIT_17s0000g02180  -  Unknown protein
## VIT_00s1927g00020  -  Ubiquitin-conjugating enzyme E2 O
## VIT_01s0026g01280  -  EMB2745 (embryo defective 2745)
## VIT_19s0014g01010  -  NHL repeat-containing protein
## VIT_05s0077g01480  -  ARR1 typeB
## VIT_09s0002g02420  -  Dual-specific kinase DSK1
## VIT_08s0007g06410  -  FAR1-related sequence5
## VIT_07s0005g02270  -  Pto kinase interactor 1
## VIT_04s0023g00530  -  Auxin responsive SAUR protein
## VIT_17s0000g02170  -  Unknown protein
## VIT_02s0025g00020  -  No hit
## VIT_19s0085g00400  -  No hit
## VIT_03s0038g03720  -  fertility restorer
## VIT_04s0008g01460  -  Unknown protein
## VIT_18s0001g07120  -  Transport inhibitor response 1 protein
## VIT_06s0004g02800  -  Homeodomain-leucine zipper protein Revoluta (REV)
## VIT_06s0004g07410  -  NADH dehydrogenase subunit 7
## VIT_05s0049g01810  -  No hit
## VIT_18s0122g01380  -  Crp1 protein
## VIT_14s0083g00100  -  Unknown protein
## VIT_11s0016g03950  -  Dehydration-responsive protein (RD22)
## VIT_06s0009g02510  -  HEAT repeat family protein
## VIT_17s0000g03750  -  Chitin elicitor-binding CEBIP LysM domain-containing
## VIT_00s0201g00080  -  No hit
## VIT_04s0044g00470  -  No hit
## VIT_12s0134g00450  -  S-locus lectin protein kinase
## VIT_10s0092g00170  -  Subtilase
## VIT_05s0020g02580  -  Pentatricopeptide (PPR) repeat-containing
## VIT_12s0035g01190  -  AMP-dependent synthetase and ligase
## VIT_12s0057g00690  -  Histidine kinase (AHK2)
```

```
## VIT_09s0002g06530  -  fertility restorer
## VIT_17s0000g04760  -  UDP-glycosyltransferase 89B2
## VIT_08s0007g03080  -  Pentatricopeptide repeat
## VIT_11s0016g05470  -  Nuclear pore complex protein Nup98-Nup96
## VIT_14s0036g00570  -  Transcription factor jumonji (jmj) protein
## VIT_18s0122g01010  -  Protein phosphatase 2C DBP
## VIT_01s0010g02780  -  ATP-dependent RNA helicase
## VIT_13s0139g00160  -  Golgin candidate 6
## VIT_08s0040g02430  -  Unknown protein
## VIT_06s0004g03270  -  Unknown protein
## VIT_14s0060g00270  -  Pentatricopeptide (PPR) repeat-containing
## VIT_07s0031g01990  -  Ribonucleotide reductase large subunit A
## VIT_12s0035g01200  -  AMP-dependent synthetase and ligase
## VIT_06s0009g02390  -  Scarecrow transcription factor 3 (SCL3)
## VIT_10s0116g00470  -  Ubiquitin family
## VIT_11s0016g01210  -  Pentatricopeptide (PPR) repeat-containing
## VIT_08s0040g02510  -  PHD zinc finger protein
## VIT_19s0015g01870  -  WRKY DNA-binding protein 2 (WRKY-3), WRKY Transcription Factor (VvWRKY
## VIT_13s0158g00240  -  No hit
## VIT_08s0007g04890  -  ACT domain-containing protein
## VIT_01s0010g00550  -  Prolyl oligopeptidase
## VIT_01s0113g00420  -  Zinc finger (DHHC type)
## VIT_06s0061g01450  -  XS domain-containing protein
## VIT_19s0090g01420  -  Wax synthase isoform 1
## VIT_19s0014g01880  -  Homeobox protein
## VIT_04s0008g03110  -  EMB1611
## VIT_05s0062g00610  -  Xyloglucan endotransglucosylase/hydrolase 23
## VIT_13s0067g00100  -  Unknown protein
## VIT_05s0049g01490  -  Splicing factor U2AF 65 kDa subunit
## VIT_05s0077g00720  -  Peroxidase 40
## VIT_13s0073g00610  -  Ubiquitin-specific protease 12
## VIT_03s0038g03440  -  Alanyl-tRNA synthetase
## VIT_13s0074g00160  -  3-oxoacyl-[acyl-carrier-protein] synthase
## VIT_16s0050g00980  -  ferric reduction oxidase 2
## VIT_00s0287g00010  -  Histone-lysine N-methyltransferase, H3 lysine-9 specific SUVH5
## VIT_17s0000g03120  -  Poly(A) polymerase
## VIT_06s0004g03940  -  CCR4-NOT transcription complex subunit 2
## VIT_16s0039g01700  -  Inositol 1,3,4-triphosphate 5/6 kinase
## VIT_17s0000g06890  -  Unknown protein
## VIT_00s0652g00030  -  Microtubule organization 1 protein (MOR1)
## VIT_09s0002g07070  -  Pre-mRNA-processing factor 8
## VIT_16s0022g01290  -  SDG29 (SET Domain group 29)
## VIT_17s0000g07910  -  Nodulin MtN21 family
## VIT_05s0077g00250  -  Unknown protein
## VIT_07s0104g00340  -  PWWP domain-containing protein
## VIT_01s0150g00380  -  Unknown protein
## VIT_01s0010g01000  -  NIK1 (NSP- interacting  kinase 1)
## VIT_03s0017g01670  -  Pentatricopeptide (PPR) repeat-containing protein
## VIT_19s0014g01500  -  PHD finger transcription factor
## VIT_12s0028g01810  -  Shaggy protein kinase delta
## VIT_05s0077g00800  -  DNA replication protein
## VIT_04s0008g05610  -  Centromeric protein
## VIT_09s0018g01620  -  Vacuolar protein sorting 53
## VIT_01s0011g02220  -  AAA-type ATPase
## VIT_06s0009g03210  -  Pentatricopeptide (PPR) repeat-containing protein
```

```
## VIT_11s0065g00930  -  Cyclic nucleotide-gated ion channel 17
## VIT_05s0020g00460  -  Evolutionarily conserved C-terminal region 6
## VIT_14s0128g00230  -  DNA binding
## VIT_13s0074g00650  -  Unknown protein
```

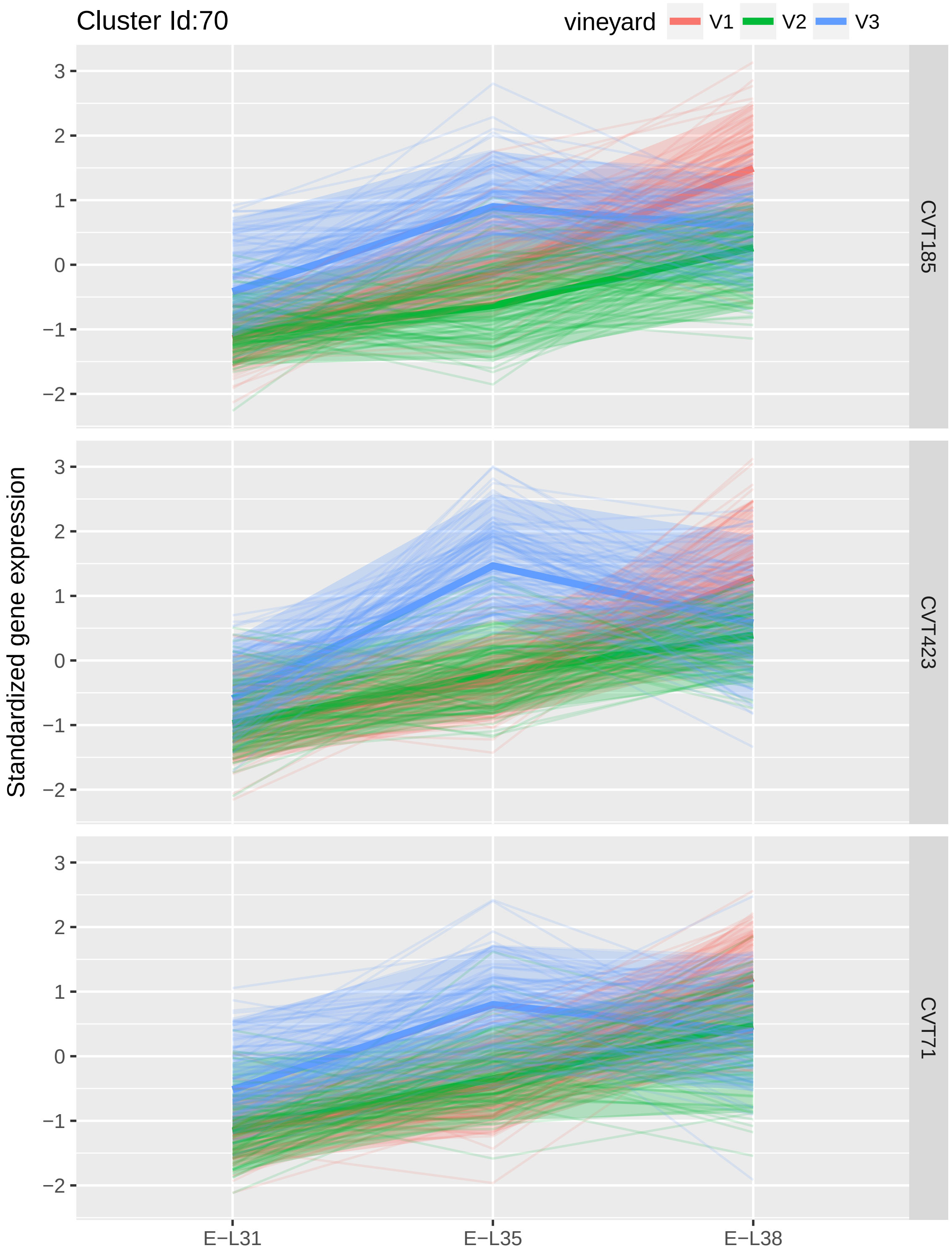

Cluster Id:70
vineyard
V1
V2
V3
CVT185
CVT423
CVT71
Standardized gene expression
3
2
1
0
−1
−2
E−L31
E−L35
E−L38

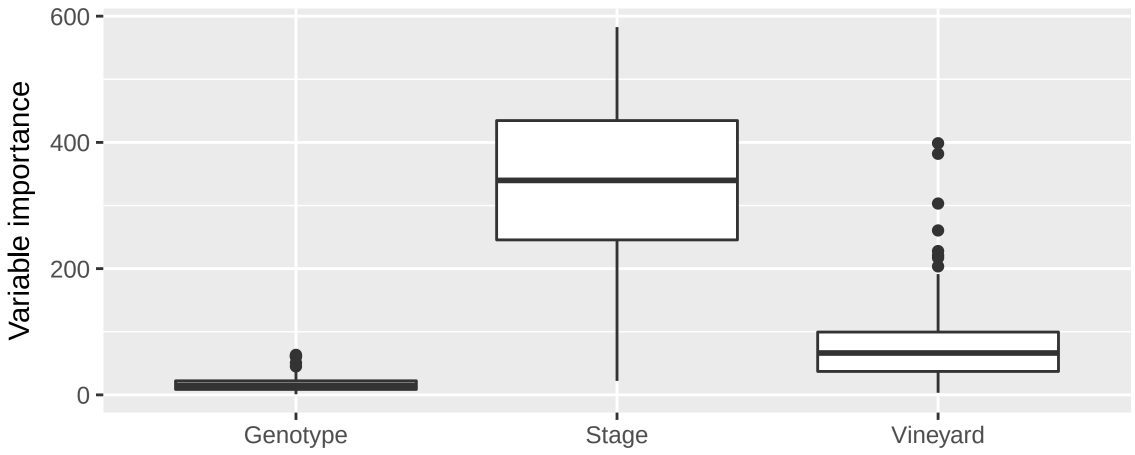

Variable importance
600
400
200
0
Genotype
Stage
Vineyard

Cluster no. 71

```
## Number of genes in the cluster: 138
## Homogeneity Index:    0.49
## Variable importance for Stage:    Median =  172.3  - Rank =  96
## Variable importance for Clone:    Median =  24.43  - Rank =  24
## Variable importance for Vineyard: Median =   91.4  - Rank =  19
##
## Gene ID               Gene Annotation
## VIT_19s0014g04130  -  Serine/threonine-protein kinase receptor ARK3
## VIT_15s0048g02540  -  Pseudo-response regulator 9 (APRR9)
## VIT_00s0316g00010  -  Disease resistance protein
## VIT_01s0026g00910  -  Unknown
## VIT_12s0059g01320  -  Glucan endo-1,3-beta-glucosidase 7 precursor
## VIT_08s0007g00930  -  Regulator of chromosome condensation (RCC1)
## VIT_16s0098g00330  -  ORG1 (OBP3-responsive gene 1)
## VIT_09s0002g04930  -  Kelch repeat-containing F-box family protein
## VIT_17s0000g02520  -  Membrane protein
## VIT_17s0000g00940  -  F-box protein
## VIT_06s0004g01220  -  PAP/fibrillin family
## VIT_14s0068g01490  -  NAC domain-containing protein (VvNAC73)
## VIT_03s0038g02310  -  myb domain protein 4 (VvMyb4a)
## VIT_11s0118g00570  -  Tristeza Virus Resistance Gene (Ctv)
## VIT_00s0291g00050  -  Octicosapeptide/Phox/Bem1p (PB1) domain-containing protein
## VIT_15s0021g01260  -  Unknown
## VIT_05s0020g04000  -  Zinc finger (C3HC4-type ring finger)
## VIT_14s0060g01940  -  Epoxide hydrolase
## VIT_13s0019g02280  -  Regulator of chromosome condensation (RCC1)
## VIT_06s0004g07270  -  UDP-glucoronosyl and UDP-glucosyl transferase
## VIT_18s0157g00020  -  GIGANTEA protein
## VIT_09s0002g05080  -  Kelch repeat-containing F-box family protein
## VIT_13s0158g00090  -  Unknown
## VIT_16s0148g00120  -  Receptor kinase homolog LRK10
## VIT_08s0040g02820  -  Unknown protein
## VIT_12s0059g00280  -  Dehydration Responsive Element-Binding Transcription Factor (VvDREB17)
## VIT_16s0100g00690  -  Ankyrin repeat
## VIT_00s0262g00090  -  Receptor kinase RK20-1
## VIT_02s0087g01010  -  CRK10 (cysteine-rich RLK10)
## VIT_17s0000g09610  -  CYP71D10
## VIT_18s0001g05640  -  Unknown protein
## VIT_13s0067g02870  -  chalcone isomerase  2 [Vitis vinifera] (VvCHI2)
## VIT_08s0040g01860  -  Unknown protein
## VIT_11s0016g00500  -  Auxin-responsive SAUR37
## VIT_02s0012g01400  -  PLATZ transcription factor
## VIT_12s0028g01150  -  Protein TRANSPARENT TESTA 12
## VIT_00s2745g00010  -  Receptor kinase RK20-1
## VIT_17s0000g08370  -  RCD one 2 (SRO2)
## VIT_09s0002g06380  -  Polygalacturonase GH28
## VIT_06s0004g07280  -  UDP-glucuronosyl/UDP-glucosyltransferase
## VIT_12s0028g03880  -  Pentatricopeptide (PPR) repeat-containing protein
## VIT_04s0008g01120  -  Glutaredoxin
## VIT_09s0002g08460  -  8-amino-7-oxononanoate synthase
## VIT_18s0072g00550  -  Unknown protein
## VIT_18s0001g14520  -  Unknown protein
## VIT_07s0005g02390  -  Protein phosphatase 2C POLTERGEIST
## VIT_14s0006g02950  -  Lateral organ boundaries protein 41
```

```
## VIT_14s0066g00810  -  Raffinose synthase
## VIT_07s0005g00590  -  Deoxyribodipyrimidine photolyase
## VIT_18s0117g00140  -  FAR1-related sequence 12
## VIT_09s0002g00640  -  Small heat stress protein class CIII
## VIT_00s2485g00010  -  CRK10 (cysteine-rich RLK10)
## VIT_02s0025g02590  -  Homeobox-leucine zipper protein ATHB-12 (VvATHB-2)
## VIT_12s0057g01410  -  Exostosin
## VIT_00s0203g00050  -  Receptor-interacting protein
## VIT_09s0002g06770  -  Unknown protein
## VIT_13s0019g04540  -  GCN5 N-acetyltransferase (GNAT)
## VIT_16s0050g02710  -  RPK1 (receptor-like protein kinase 1)
## VIT_16s0050g01830  -  AAA-type ATPase
## VIT_09s0002g00630  -  No hit
## VIT_16s0100g00220  -  No hit
## VIT_03s0017g01550  -  CRK10 (cysteine-rich RLK10)
## VIT_05s0020g00600  -  1-cys peroxiredoxin
## VIT_01s0011g04180  -  Phosphatidic acid phosphatase / PAP2
## VIT_06s0004g07500  -  WRKY Transcription Factor (VvWRKY16)
## VIT_00s0258g00050  -  Receptor serine/threonine kinase
## VIT_00s0265g00090  -  Unknown protein
## VIT_10s0003g03170  -  CYP710A1 C-22 sterol desaturase
## VIT_19s0014g02900  -  Ring finger protein 185
## VIT_19s0090g01270  -  PHD finger transcription factor
## VIT_14s0066g02180  -  myb domain protein 35
## VIT_08s0007g07690  -  Polygalacturonase inhibiting protein PGIP1
## VIT_13s0019g02300  -  Wall-associated receptor kinase-like 20
## VIT_19s0015g01740  -  Actin-related protein 8
## VIT_11s0149g00050  -  Hexose transporter 7
## VIT_04s0008g00480  -  Pentatricopeptide (PPR) repeat-containing
## VIT_16s0039g02350  -  Dihydroflavonol 4-reductase
## VIT_02s0234g00110  -  Oleosin OLE-4
## VIT_16s0098g00850  -  Caffeic acid O-methyltransferase (COMT1)
## VIT_14s0108g00270  -  Aldose 1-epimerase
## VIT_16s0039g01200  -  Receptor serine/threonine kinase
## VIT_16s0039g02280  -  EIX receptor 2
## VIT_02s0154g00310  -  Protease inhibitor/seed storage/lipid transfer protein (LTP)
## VIT_02s0012g01940  -  Myb KAN2 (KANADI 2)
## VIT_19s0014g02460  -  Plastocyanin domain-containing protein
## VIT_02s0025g01790  -  Cellulose synthase CSLG3
## VIT_14s0068g00110  -  feronia receptor-like kinase
## VIT_11s0016g02980  -  Unknown protein
## VIT_07s0005g05660  -  F-box/kelch-repeat protein
## VIT_18s0041g01860  -  Avirulence induced gene (AIG1)
## VIT_11s0016g05330  -  SPX2 (SYG1/Pho81/XPR1) domain-containing protein SPX2
## VIT_16s0148g00280  -  Receptor kinase homolog LRK10
## VIT_04s0044g01290  -  MAP3Ka
## VIT_00s0259g00200  -  Nudix hydrolase 2
## VIT_04s0008g05380  -  Serine hydrolase [Vitis vinifera]
## VIT_03s0063g02660  -  DNA-3-methyladenine glycosylase II
## VIT_08s0105g00300  -  Phospholipase C
## VIT_16s0098g00270  -  No hit
## VIT_08s0040g02260  -  Protamine P1
## VIT_11s0065g00210  -  Heat shock protein-related
## VIT_06s0004g02630  -  Zinc finger (C3HC4-type ring finger)
## VIT_18s0001g06120  -  Cis-zeatin O-beta-D-glucosyltransferase
```

```
## VIT_12s0057g01440  -  Heavy-metal-associated domain-containing protein
## VIT_16s0148g00250  -  Zinc finger (C3HC4-type ring finger)
## VIT_05s0049g00160  -  IBR10 (indole-3-butyric acid response 10); catalytic/ dodecenoyl-CoA d
## VIT_16s0022g00080  -  HcrVf2 protein
## VIT_12s0028g01030  -  Unknown
## VIT_16s0050g01300  -  C2 domain-containing protein
## VIT_19s0090g00710  -  No hit
## VIT_15s0048g02230  -  Calcineurin phosphoesterase
## VIT_15s0046g00070  -  PBP1 (pinoid-binding protein 1)
## VIT_18s0157g00050  -  Carboxypeptidase D
## VIT_18s0001g15390  -  Gaiacol peroxidase
## VIT_13s0064g00810  -  (5,6) (5`,6`) (9,10) (9`,10`) cleavage dioxygenase (CCD1) (VvCCD1.1)
## VIT_17s0000g00920  -  Glutamate decarboxylase 1 (GAD 1)
## VIT_00s0388g00050  -  Receptor kinase homolog LRK14
## VIT_06s0080g00070  -  S-receptor kinase
## VIT_14s0006g02990  -  3-ketoacyl-CoA synthase
## VIT_09s0002g02690  -  3'-5' exonuclease domain-containing protein
## VIT_11s0065g00360  -  Acetyl-CoA carboxylase, biotin carboxylase subunit (CAC2)
## VIT_11s0016g02520  -  Unknown protein
## VIT_05s0102g00090  -  Biotin synthase (BioB) (BIO2)
## VIT_16s0022g00240  -  EIX receptor 2
## VIT_16s0039g02360  -  Disease resistance protein (NBS-LRR class)
## VIT_12s0028g02350  -  basic helix-loop-helix (bHLH) family
## VIT_13s0019g01180  -  Glucan endo-1,3-beta-glucosidase precursor
## VIT_18s0001g03540  -  Auxin transporter protein 4
## VIT_19s0014g03520  -  fiber protein
## VIT_06s0009g00580  -  Receptor protein kinase
## VIT_14s0066g00400  -  Chalcone isomerase
## VIT_14s0006g02660  -  Receptor serine/threonine kinase PR5K-l
## VIT_02s0012g01660  -  ABC Transporter (VvMDR5 - VvABCB5)
## VIT_00s0388g00060  -  Receptor serine/threonine kinase
## VIT_18s0164g00100  -  Laccase
## VIT_18s0001g14950  -  EMB2170 (embryo defective 2170)
## VIT_00s2178g00010  -  R protein  PRF  disease resistance protein
## VIT_01s0011g06380  -  BPC2/BBR/BPC2/BPC2 (basic pentacysteine2)
## VIT_05s0102g00940  -  R protein  PRF  disease resistance protein
```

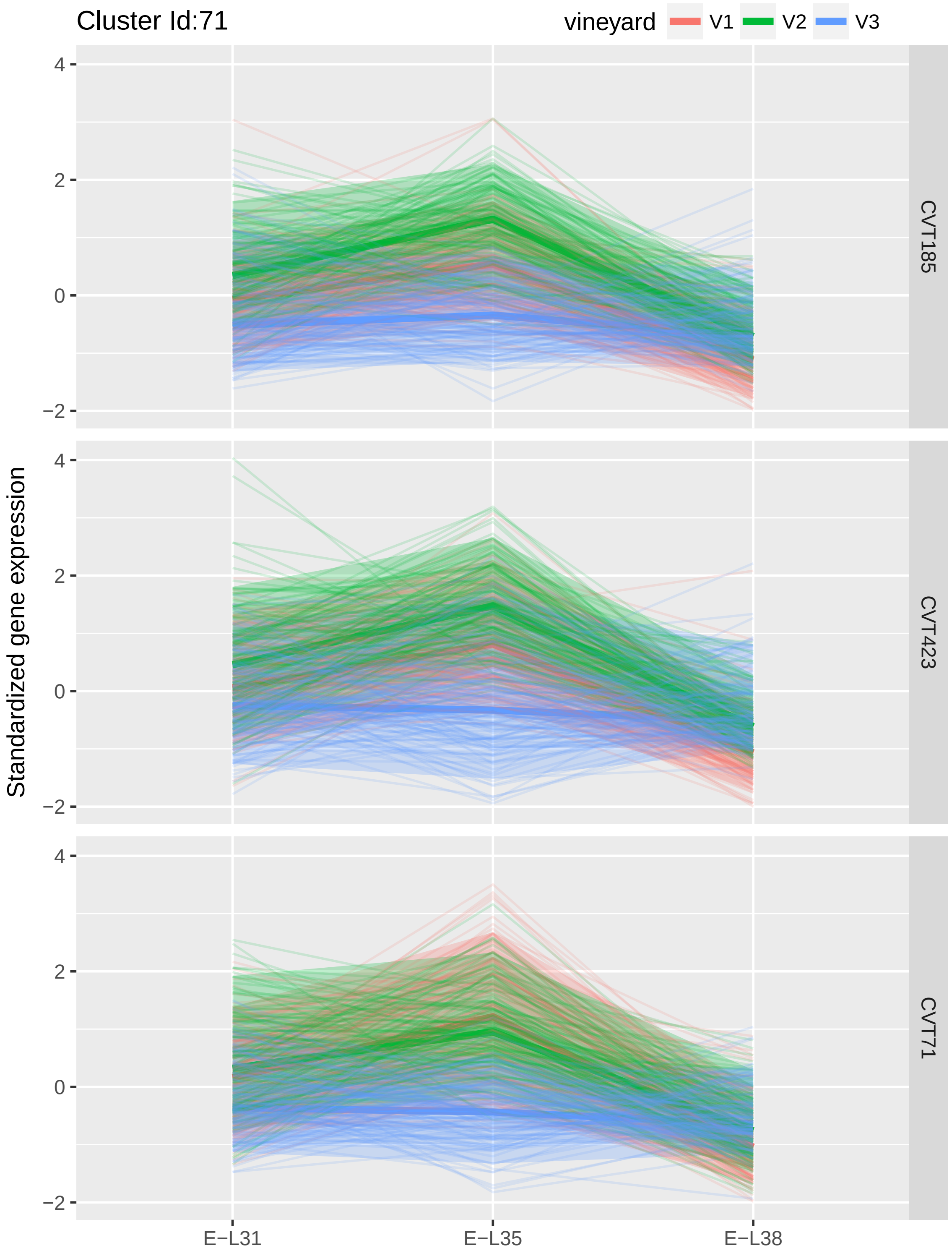

Cluster Id:71
vineyard
V1
V2
V3
Standardized gene expression
CVT185
CVT423
CVT71
4
2
0
−2
E−L31
E−L35
E−L38

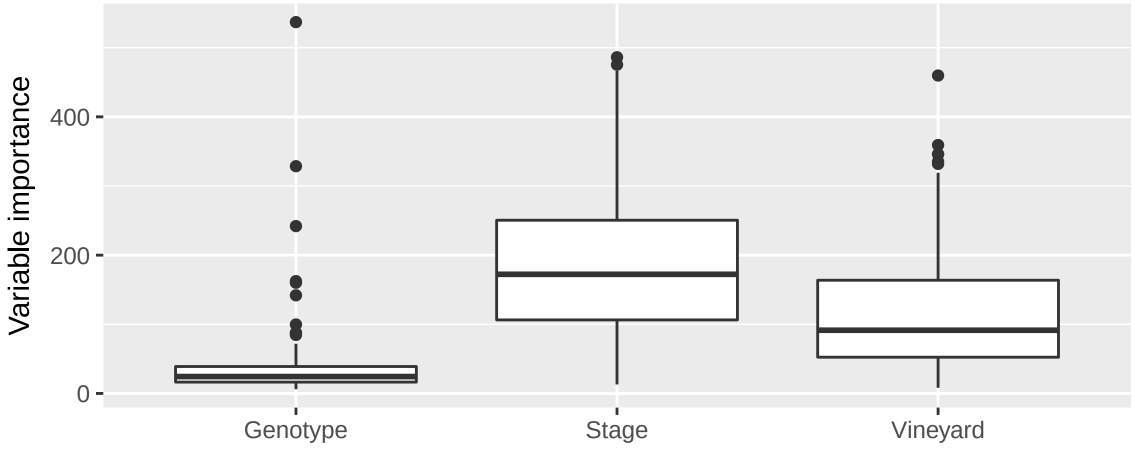

Variable importance
400
200
0
Genotype
Stage
Vineyard

Cluster no. 72

```
## Number of genes in the cluster: 288
## Homogeneity Index:    0.86
## Variable importance for Stage:    Median =  631.7  - Rank =  10
## Variable importance for Clone:    Median =  12.31  - Rank =  90
## Variable importance for Vineyard: Median =  13.88  - Rank =  102
##
## Gene ID              Gene Annotation
## VIT_17s0000g02600  -  Unknown protein
## VIT_13s0064g00150  -  No hit
## VIT_02s0033g00520  -  Disease resistance protein (NBS-LRR class)
## VIT_11s0016g01290  -  CYP72A1
## VIT_08s0007g08250  -  Nuclear transcription factor Y subunit A-9
## VIT_12s0055g00710  -  Phosphatidylserine decarboxylase
## VIT_11s0118g00330  -  Unknown protein
## VIT_19s0015g01670  -  Phenylalanine-tRNA ligase
## VIT_01s0010g02100  -  Indeterminate(ID)-domain 5
## VIT_05s0062g01170  -  Ku70
## VIT_08s0007g07890  -  Pentatricopeptide (PPR) repeat-containing
## VIT_17s0000g09060  -  Unknown
## VIT_01s0010g02870  -  AAA-type ATPase
## VIT_04s0008g05490  -  L-ascorbate peroxidase.
## VIT_13s0067g01490  -  NBS-LRR type disease resistance protein Rps1-k-1
## VIT_11s0016g05300  -  Heme oxygenase 1
## VIT_00s0480g00090  -  Catechol oxidase (EC 1.10.3.1) precursor - grape
## VIT_14s0060g01400  -  Pentatricopeptide (PPR) repeat-containing protein
## VIT_13s0067g00810  -  R protein  PRF  disease resistance protein
## VIT_06s0004g02090  -  Nucleolar GTP-binding protein NOG1
## VIT_06s0009g03630  -  CYP86A2
## VIT_17s0000g08070  -  Aldehyde Dehydrogenase (VvALDH2B4)
## VIT_12s0028g01700  -  WRKY DNA-binding protein 65
## VIT_09s0002g01380  -  ATMYB66/WER/WER1 (WEREWOLF 1)
## VIT_10s0003g02070  -  putative MADS-box Agamous 2 (VviAG2)
## VIT_14s0066g00970  -  Anthranilate phosphoribosyltransferase, chloroplast precursor
## VIT_15s0021g01060  -  CYP72A1
## VIT_18s0001g07960  -  Serine hydroxymethyltransferase
## VIT_18s0089g01050  -  PHD finger transcription factor
## VIT_14s0171g00040  -  Cf-2 gene
## VIT_17s0000g00840  -  Unknown protein
## VIT_14s0060g00650  -  No hit
## VIT_00s0361g00040  -  Anthocyanidin reductase (VvANR) [Vitis vinifera] GeneID: 100232981
## VIT_06s0004g00410  -  Carboxyl-terminal peptidase
## VIT_12s0055g00720  -  Phosphatidylserine decarboxylase
## VIT_08s0032g00670  -  Glutamate carboxypeptidase (AMP1)
## VIT_08s0040g00720  -  Snf1 protein kinase (KIN10) (SKIN10)
## VIT_19s0014g00520  -  RKF1 (receptor-like kinase in flowers 1)
## VIT_05s0020g01760  -  Glutaredoxin-like protein
## VIT_15s0046g01230  -  Leaf senescence protein-like
## VIT_14s0030g01160  -  R protein disease resistance protein
## VIT_05s0077g02100  -  Senescence-associated protein
## VIT_00s0204g00080  -  TRNA/rRNA methyltransferase (SpoU)
## VIT_15s0107g00270  -  Mechanosensitive ion channel
## VIT_16s0050g02100  -  Disease resistance
## VIT_00s0897g00010  -  Leucine-rich repeat family protein
## VIT_00s0203g00160  -  Cyclin D-type
```

```
## VIT_11s0016g01460  -  BRXL4 (Brevis radix like 4)
## VIT_07s0185g00130  -  MAP kinase phosphatase 1
## VIT_15s0046g00390  -  Seven in absentia SINA4
## VIT_02s0025g03430  -  GMP synthase
## VIT_17s0053g00970  -  No hit
## VIT_07s0005g00090  -  Auxin-responsive GH3
## VIT_04s0023g03810  -  Jasmonate O-methyltransferase
## VIT_01s0010g03850  -  Calmodulin-binding protein
## VIT_07s0141g00850  -  ATP-binding protein involved in chromosome partitioning MRP
## VIT_12s0057g00220  -  Unknown protein
## VIT_17s0000g05480  -  EMB2454 (embryo defective 2454)
## VIT_07s0104g00830  -  Sugar transporter ERD6-like 7
## VIT_00s0285g00060  -  Glutamate carboxypeptidase (AMP1)
## VIT_02s0025g01850  -  Cellulose synthase CSLG3
## VIT_14s0066g01430  -  Unknown protein
## VIT_15s0046g01430  -  Aldose 1-epimerase
## VIT_19s0014g01440  -  KH domain-containing quaking protein
## VIT_17s0000g07360  -  Transposon protein, Mutator sub-class
## VIT_18s0072g00820  -  VFB3 (VIER F- box  proteinE 3)
## VIT_18s0041g00840  -  UDP-glucose: anthocyanidin 5,3-O-glucosyltransferase
## VIT_14s0171g00380  -  DNA-binding bromodomain-containing protein
## VIT_06s0004g05790  -  Unknown protein
## VIT_10s0092g00570  -  Leucine-rich repeat family
## VIT_19s0014g01470  -  Thioredoxin TTL1 (Tetratricopetide-repeat thioredoxin-like 1)
## VIT_12s0035g00480  -  Disease resistance protein
## VIT_13s0064g00240  -  No hit
## VIT_08s0058g00510  -  MATE efflux family protein
## VIT_07s0129g00250  -  Phototropic-responsive NPH3
## VIT_01s0011g03450  -  Alpha-glucosidase
## VIT_10s0116g00500  -  Myb caprice CPC
## VIT_13s0067g02580  -  R protein MLA10
## VIT_08s0007g08810  -  Nonsense-mediated mRNA decay NMD3
## VIT_08s0007g05020  -  Dentin sialophosphoprotein
## VIT_15s0046g01650  -  Unknown
## VIT_18s0001g09740  -  S-locus receptor protein kinase
## VIT_08s0007g08140  -  Helicase
## VIT_14s0030g00990  -  R protein disease resistance protein
## VIT_15s0046g01980  -  UDP-glucosyltransferase
## VIT_07s0191g00050  -  Peroxidase 17
## VIT_11s0016g02960  -  Glucan endo-1,3-beta-glucosidase 4 precursor
## VIT_02s0025g03940  -  Unknown protein
## VIT_10s0003g05030  -  Leucine-rich repeat family protein
## VIT_14s0081g00710  -  Unknown protein
## VIT_07s0151g00570  -  OB-fold nucleic acid binding domain containing protein
## VIT_04s0008g07370  -  Glutamine dumper  2
## VIT_17s0000g03820  -  Unknown protein
## VIT_19s0014g03730  -  No hit
## VIT_19s0014g04300  -  S-locus protein kinase
## VIT_00s0975g00020  -  Cellulose synthase CSLE1
## VIT_03s0038g04330  -  Unknown
## VIT_18s0089g00400  -  Carboxyesterase18 CXE18
## VIT_11s0052g01390  -  Adenosylmethionine-8-amino-7- oxononanoate aminotransferase
## VIT_01s0026g00150  -  Methyl-CPG-binding Domain 9 MBD9
## VIT_12s0028g02000  -  Triacylglycerol lipase
```

```
## VIT_04s0008g05530  -  Unknown protein
## VIT_10s0042g01020  -  Isochorismatase
## VIT_02s0033g01410  -  Alpha-glucosidase 2
## VIT_06s0009g00410  -  BLH1 (embryo sac development arrest 29)
## VIT_19s0014g00470  -  RKF1 (receptor-like kinase in flowers 1)
## VIT_05s0094g01010  -  Indole-3-acetate beta-glucosyltransferase
## VIT_05s0077g01290  -  Unknown protein
## VIT_04s0043g00860  -  Unknown protein
## VIT_17s0000g02550  -  HMR1 protein
## VIT_16s0022g00890  -  Invertase/pectin methylesterase inhibitor
## VIT_12s0057g00210  -  No hit
## VIT_06s0004g04610  -  fasciclin arabinogalactan-protein (FLA4)
## VIT_06s0009g03640  -  Annexin 1 (ANN1)
## VIT_07s0141g00510  -  RNA polymerase Rpb7 N-terminal domain-containing
## VIT_04s0008g01800  -  myb domain protein 7
## VIT_00s0753g00010  -  Unknown
## VIT_01s0026g00690  -  RNase H domain-containing protein
## VIT_19s0014g01480  -  No hit
## VIT_02s0025g02560  -  O-succinylhomoserine sulfhydrylase
## VIT_00s0646g00010  -  Hydrolase, alpha/beta fold
## VIT_10s0003g03010  -  Unknown protein
## VIT_06s0004g06050  -  Thioesterase family
## VIT_12s0059g00230  -  Epoxide hydrolase 2
## VIT_04s0023g01460  -  Pentatricopeptide (PPR) repeat membrane-associated salt-inducible prot
## VIT_03s0167g00070  -  putative MADS-box Short Vegetal Phase 5 (VviSVP5)
## VIT_08s0007g02440  -  Aspartyl protease
## VIT_07s0129g00350  -  Unknown protein
## VIT_13s0019g04580  -  Zinc finger (DNL type)
## VIT_02s0033g00030  -  No hit
## VIT_18s0001g08920  -  No hit
## VIT_13s0067g02610  -  Unknown
## VIT_00s0480g00070  -  Polyphenol oxidase II, chloroplast precursor
## VIT_18s0001g00500  -  Coiled-coil domain-containing protein 13
## VIT_19s0014g03200  -  COP1-interacting protein 7
## VIT_08s0007g06640  -  Growth-regulating factor related
## VIT_04s0008g05410  -  Serine hydrolase [Vitis vinifera]
## VIT_07s0129g00950  -  Protein kinase
## VIT_18s0089g00280  -  TIR-NBS-LRR-TIR disease resistance protein
## VIT_18s0001g04520  -  No hit
## VIT_06s0004g00740  -  Acyl-CoA synthetases (Acyl-activating enzyme 18)
## VIT_07s0141g01030  -  R protein MLA10
## VIT_19s0014g00790  -  RKF1 (receptor-like kinase in flowers 1)
## VIT_10s0003g04450  -  ABC Transporter (VvMRP18 - VvABCC18)
## VIT_10s0003g02000  -  RKF1 (receptor-like kinase in flowers 1)
## VIT_01s0011g01790  -  Galactose-binding like
## VIT_08s0007g00570  -  WRKY Transcription Factor (VvWRKY27)
## VIT_19s0015g01250  -  No hit
## VIT_08s0032g00420  -  Exostosin
## VIT_12s0028g03210  -  Tyrosine aminotransferase
## VIT_00s1045g00020  -  Pentatricopeptide (PPR) repeat-containing
## VIT_06s0061g00440  -  Heavy-metal-associated domain-containing protein
## VIT_12s0057g00470  -  C2 domain containing protein
## VIT_08s0058g00260  -  Ethylene overproducer 1 (ETO1)
## VIT_00s0880g00010  -  basic helix-loop-helix (bHLH) family
## VIT_18s0041g02170  -  Peptidoglycan-binding LysM:Cell wall hydrolase, SleB precursor
```

```
## VIT_12s0034g01660  -  R protein MLA10
## VIT_15s0046g00230  -  Lateral organ boundaries protein 1
## VIT_04s0023g03680  -  Histidine kinase 1 (AHK1)
## VIT_05s0020g03640  -  Unknown protein
## VIT_12s0028g02420  -  1-aminocyclopropane-1-carboxylate oxidase homolog 1
## VIT_19s0015g00440  -  Nuclear transcription factor Y subunit B-3
## VIT_07s0129g00600  -  Aspartic Protease (VvAP16)
## VIT_07s0005g03390  -  Unknown protein
## VIT_13s0047g00730  -  R protein MLA10
## VIT_13s0064g00370  -  Alpha-N-acetylglucosaminidase
## VIT_08s0007g03050  -  E8 protein
## VIT_14s0108g00750  -  Zinc Finger Homeodomain Transcription Factor (VvZHD8)
## VIT_19s0014g00710  -  RKF1 (receptor-like kinase in flowers 1)
## VIT_05s0020g01860  -  Nucleolar protein 58
## VIT_14s0083g00610  -  Pentatricopeptide (PPR) repeat-containing protein
## VIT_05s0020g01640  -  Transcription termination factor mitochondrial mTERF
## VIT_17s0000g04480  -  ERF/AP2 Gene Family (VvERF048)
## VIT_13s0067g00620  -  Cinnamyl alcohol dehydrogenase
## VIT_09s0002g04240  -  Membrane protein
## VIT_16s0098g00290  -  GLT1 (NADH-dependent glutamate synthase 1 gene)
## VIT_00s0389g00020  -  Acyl-CoA oxidase (ACX1)
## VIT_10s0003g04100  -  Auxin response factor 3
## VIT_07s0031g02410  -  Unknown protein
## VIT_14s0060g01410  -  Dihydrolipoyl dehydrogenase
## VIT_18s0001g12540  -  Maf, septum formation protein
## VIT_18s0001g03730  -  SET Domain group 37
## VIT_17s0000g08900  -  LRR receptor-like kinase 2
## VIT_02s0154g00590  -  Unknown
## VIT_09s0018g00250  -  DNA-3-methyladenine glycosylase I
## VIT_18s0001g08230  -  Receptor-like protein kinase HAIKU2
## VIT_18s0001g07140  -  Zinc Finger Homeodomain Transcription Factor (VvZHD10)
## VIT_13s0067g01230  -  AP2-associated kinase
## VIT_00s0480g00100  -  Catechol oxidase (EC 1.10.3.1) precursor - grape
## VIT_03s0038g02150  -  Unknown
## VIT_18s0001g12100  -  Auxilin
## VIT_03s0063g01970  -  Non-symbiotic hemoglobin 2
## VIT_07s0005g00850  -  ERS type ethylene receptor
## VIT_04s0008g01850  -  Trihelix DNA-binding protein (GT2)
## VIT_13s0019g03060  -  Glucosyltransferase-2
## VIT_00s0817g00010  -  Hydrolase
## VIT_03s0091g01130  -  Meprin and TRAF homology domain-containing protein
## VIT_05s0077g02020  -  Epoxide hydrolase
## VIT_14s0066g00300  -  Unknown
## VIT_12s0057g00570  -  Zinc finger (CCCH-type) family protein
## VIT_12s0059g00220  -  Epoxide hydrolase
## VIT_13s0147g00150  -  TIR-NBS-LRR-TIR disease resistance protein
## VIT_12s0035g01280  -  R protein disease resistance protein
## VIT_13s0019g02130  -  Tropinone reductase
## VIT_07s0141g00480  -  No hit
## VIT_19s0090g01170  -  UPF0041
## VIT_17s0000g06750  -  Unknown protein
## VIT_00s1239g00010  -  Abhydrolase domain-containing protein
## VIT_10s0003g01290  -  No hit
## VIT_17s0053g00950  -  Cyclic nucleotide-binding transporter 1
## VIT_18s0157g00170  -  Alliin lyase precursor
```

```
## VIT_07s0104g00300  -  Hemolysin
## VIT_09s0070g00710  -  Isopentenyltransferase
## VIT_13s0064g00290  -  Cinnamyl alcohol dehydrogenase
## VIT_19s0014g03770  -  Retrotransposon
## VIT_17s0000g07980  -  RDR2 (RNA-dependent RNA polymerase 2)
## VIT_18s0117g00390  -  far-red impaired responsive family protein
## VIT_05s0094g01640  -  Pentatricopeptide (PPR) repeat
## VIT_16s0039g02130  -  Pentatricopeptide (PPR) repeat-containing
## VIT_00s0227g00090  -  No hit
## VIT_06s0004g05390  -  Tropinone reductase
## VIT_09s0002g03460  -  Taurine dioxygenase
## VIT_09s0002g03860  -  RPS5 (resistant to p. syringae 5)
## VIT_09s0070g00720  -  No hit
## VIT_00s0238g00060  -  RPS4 (resistant to p. syringae 4)
## VIT_19s0090g01230  -  Microtubule associated protein (MAP65-1a)
## VIT_07s0129g00940  -  Protein kinase
## VIT_19s0014g00700  -  Leucine-rich repeat protein kinase
## VIT_00s0226g00060  -  Unknown protein
## VIT_07s0104g01320  -  Transport inhibitor response 1
## VIT_06s0004g08190  -  ERF/AP2 Gene Family (VvERF055)
## VIT_11s0016g03030  -  Calmodulin binding protein
## VIT_02s0154g00410  -  Unknown protein
## VIT_05s0020g02760  -  Signal peptidase complex subunit 3
## VIT_19s0014g05090  -  Thioredoxin h
## VIT_13s0067g02430  -  R protein MLA10
## VIT_19s0093g00400  -  Glutathione S-transferase 25 GSTU25
## VIT_12s0035g02060  -  Methionine S-methyltransferase
## VIT_04s0023g01730  -  Unknown protein
## VIT_06s0080g00060  -  Arabidopsis histidine phosphotransfer AHP3
## VIT_10s0116g00560  -  Polyphenol oxidase II, chloroplast precursor
## VIT_01s0150g00510  -  Protein phosphatase 2C
## VIT_12s0035g00440  -  R protein disease resistance protein
## VIT_04s0044g01870  -  Auxin efflux carrier
## VIT_02s0012g02030  -  Homeobox-7
## VIT_04s0008g05830  -  Armadillo/beta-catenin repeat
## VIT_19s0014g03210  -  COP1-interacting protein 7
## VIT_00s0480g00080  -  Polyphenol oxidase II, chloroplast precursor
## VIT_18s0041g00170  -  Brassinosteroid insensitive 1-associated receptor kinase 1
## VIT_09s0002g02050  -  1-deoxy-D-xylulose 5-phosphate synthase
## VIT_17s0000g08730  -  Protein phosphatase 2 regulatory subunit B
## VIT_12s0059g01640  -  Unknown protein
## VIT_17s0053g00710  -  Calcium-dependent protein kinase
## VIT_17s0000g03130  -  Unknown protein
## VIT_16s0050g02730  -  Receptor-like kinase LRK14
## VIT_10s0003g02470  -  SRG1 (senescence-related gene 1) oxidoreductase
## VIT_13s0067g01300  -  AP2-associated kinase
## VIT_00s0261g00020  -  Unknown protein
## VIT_15s0021g01300  -  Pentatricopeptide (PPR) repeat-containing protein
## VIT_08s0007g08910  -  Cis-zeatin O-beta-D-glucosyltransferase
## VIT_05s0077g01130  -  Seed maturation protein PM36
## VIT_08s0007g00620  -  Zinc finger (DHHC type)
## VIT_11s0052g00240  -  Accelerated cell death 11 (ACD11)
## VIT_14s0060g00260  -  Phytochrome interacting factor 3 (PIF3)
## VIT_16s0098g00150  -  Receptor serine/threonine kinase PR5K
## VIT_05s0020g03670  -  MARD1 (mediator of ABA-regulated dormancy 1)
```

```
## VIT_17s0000g05750  -  Isochorismate synthase
## VIT_13s0067g00910  -  R protein MLA10
## VIT_18s0041g00930  -  UDP-glucose: anthocyanidin 5,3-O-glucosyltransferase
## VIT_18s0001g11780  -  No hit
## VIT_13s0019g03590  -  Catalytic
## VIT_08s0007g05060  -  ABC Transporter (VvMDR17 - VvABCB17)
## VIT_01s0011g06300  -  Annexin 5 (ANN5)
## VIT_18s0122g00750  -  HSP20-like chaperone
## VIT_01s0026g00260  -  Potassium transporter (KUP7)
## VIT_02s0154g00580  -  Unknown
## VIT_15s0021g00990  -  LOL3 (LSD ONE like 3)
## VIT_03s0038g02680  -  Zeta-carotene desaturase
## VIT_08s0007g01040  -  Aldo-keto reductase
## VIT_13s0067g00170  -  Autophagy 18 ATG18d
## VIT_02s0087g00030  -  Alpha-glucosidase
## VIT_08s0007g02100  -  Alpha-1,4-glycosyltransferase
## VIT_07s0104g01530  -  Transcription termination factor mitochondrial mTERF
## VIT_13s0019g04380  -  Auxin response factor 10
## VIT_18s0041g00160  -  Disease resistance protein (NBS class)
## VIT_00s0215g00070  -  ADP-ribosylation factor
## VIT_15s0048g00520  -  Receptor-like protein kinase
## VIT_17s0000g08850  -  SEC14 cytosolic factor
```

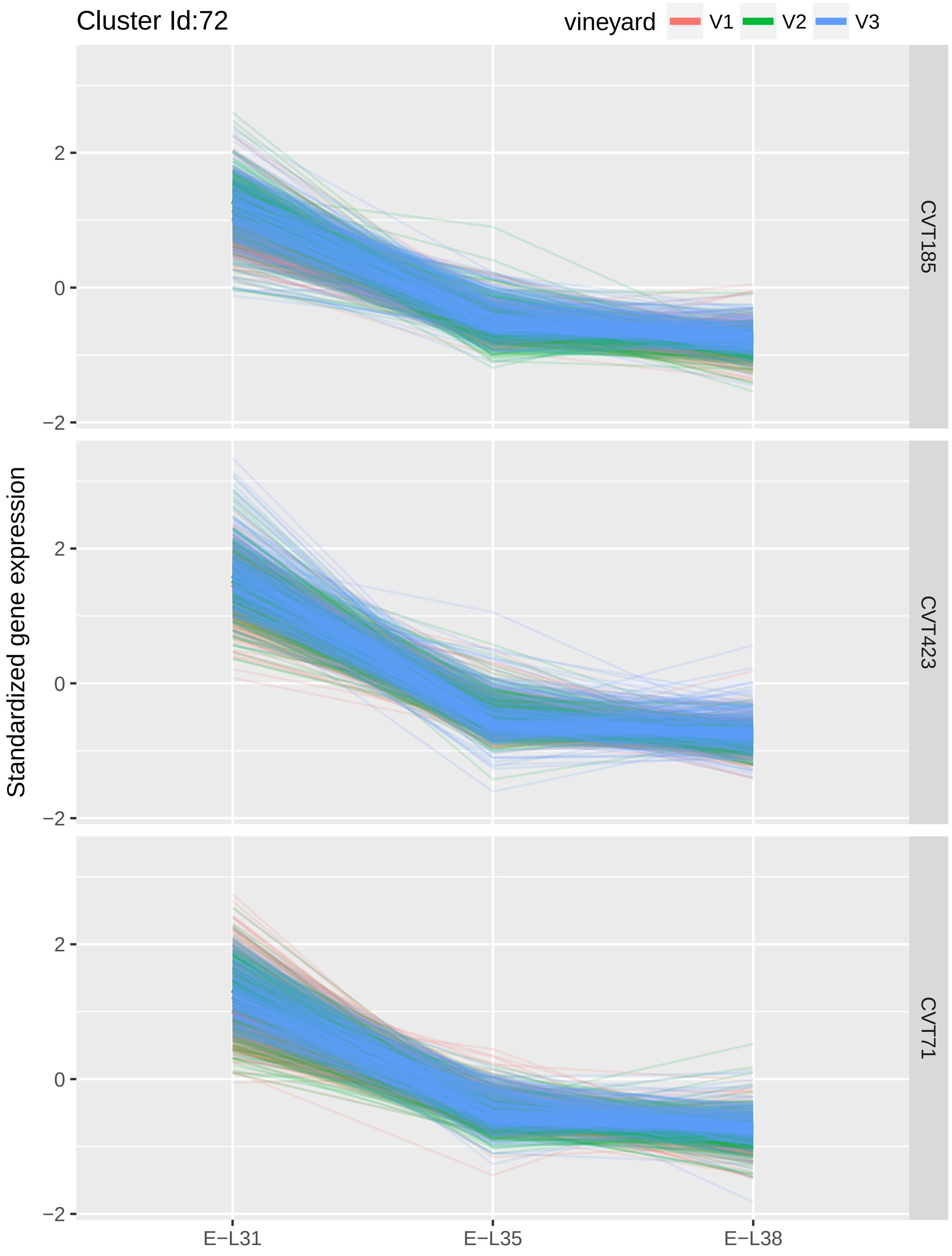
Cluster Id:72
vineyard
V1
V2
V3
Standardized gene expression
CVT185
CVT423
CVT71
2
0
−2
E−L31
E−L35
E−L38

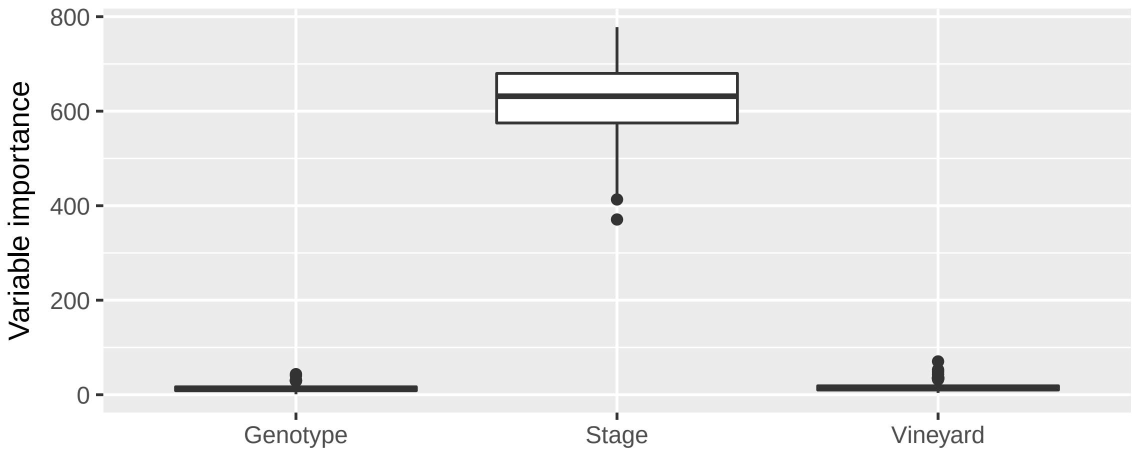
Variable importance
800
600
400
200
0
Genotype
Stage
Vineyard

Cluster no. 73

```
## Number of genes in the cluster: 197
## Homogeneity Index:    0.78
## Variable importance for Stage:    Median =  406.6  - Rank =  54
## Variable importance for Clone:    Median =  24.19  - Rank =  26
## Variable importance for Vineyard: Median =  36.76  - Rank =  56
##
## Gene ID               Gene Annotation
## VIT_11s0016g04070  -  Hydroxyproline-rich glycoprotein
## VIT_18s0001g12260  -  Incomplete root hair elongation
## VIT_04s0023g02450  -  Papain cysteine proteinase
## VIT_06s0004g02140  -  MATE efflux family protein
## VIT_08s0007g08290  -  GTP-binding protein TypA/BipA
## VIT_18s0001g10030  -  WRKY Transcription Factor (VvWRKY54)
## VIT_19s0014g03380  -  3'(2') 5'-bisphosphate nucleotidase
## VIT_09s0054g00930  -  7-dehydrocholesterol reductase
## VIT_14s0108g00740  -  GASA4
## VIT_14s0108g00490  -  Cullin 3a
## VIT_17s0000g04770  -  PHD finger transcription factor
## VIT_14s0219g00040  -  Zinc finger (C3HC4-type ring finger)
## VIT_03s0063g00820  -  Carboxyesterase 12; CXE12
## VIT_08s0105g00260  -  Digalactosyldiacylglycerol synthase 1
## VIT_06s0061g00230  -  ABC Transporter (VvWBC26 - VvABCG26)
## VIT_05s0094g00340  -  Chitinase class IV
## VIT_12s0059g02510  -  Zinc finger (B-box type)
## VIT_19s0014g03720  -  Unknown protein
## VIT_01s0011g04810  -  Unknown protein
## VIT_01s0026g00220  -  Aldehyde Dehydrogenase (VvALDH2B8)
## VIT_05s0102g00570  -  O-sialoglycoprotein endopeptidase
## VIT_07s0031g01640  -  ABC Transporter (VvGCN3 - VvABCF3)
## VIT_05s0049g01980  -  3-isopropylmalate dehydratase large subunit 2
## VIT_18s0001g04060  -  Catalytic
## VIT_11s0016g00790  -  Small nuclear ribonucleoprotein LSM1
## VIT_07s0005g04120  -  PHR1 (phosphate starvation response 1)
## VIT_17s0000g07800  -  Unknown protein
## VIT_05s0062g01190  -  Pirin
## VIT_02s0025g01610  -  Phosphatidylinositol 4-kinase type-II
## VIT_18s0001g12390  -  Glucan endo-1,3-beta-glucosidase 7 precursor
## VIT_04s0008g02680  -  No hit
## VIT_07s0005g01050  -  Unknown protein
## VIT_03s0038g00760  -  Arginine decarboxylase (Fragment)
## VIT_07s0005g04390  -  Clavata1 receptor kinase (CLV1)
## VIT_00s2349g00010  -  2-oxoglutarate/malate carrier protein, Mitochondrial
## VIT_16s0050g00140  -  Heavy-metal-associated domain-containing protein
## VIT_04s0023g00600  -  Isopentenyl diphosphate isomerase 2
## VIT_16s0022g01440  -  Transketolase, chloroplast precursor
## VIT_08s0007g07670  -  NAC domain-containing protein (VvNAC60)
## VIT_14s0030g01950  -  Transferase
## VIT_14s0066g00960  -  Glutaredoxin
## VIT_14s0128g00150  -  DNA excision repair protein ERCC-1
## VIT_04s0023g01490  -  Ovate family protein 7 OFP7
## VIT_02s0033g00380  -  VvMybA2 (C-term)
## VIT_04s0008g05210  -  Basic Leucine Zipper Transcription Factor (VvbZIP10)
## VIT_05s0051g00670  -  11,12 9-cis epoxycarotenoid dioxygenase (NCED1) (VvNCED1)
## VIT_12s0057g00710  -  DnaJ homolog, subfamily B, member 12
```

```
## VIT_17s0000g04050  -  Lipase family
## VIT_08s0058g01040  -  SOS2 (salt overly sensitive 2)
## VIT_18s0001g03520  -  Camphor resistance CrcB
## VIT_15s0048g01230  -  1-acylglycerol-3-phosphate O-acyltransferase
## VIT_08s0007g01650  -  Auxin-independent growth promoter
## VIT_15s0046g02290  -  ATBRM/CHR2 (Arabidopsis thaliana BRAHMA)
## VIT_02s0025g02140  -  Heat shock protein 70
## VIT_04s0008g06570  -  Chorismate mutase, cytosolic (CM2)
## VIT_11s0037g00470  -  No hit
## VIT_02s0025g04580  -  Hydroxymethylglutaryl-CoA synthase
## VIT_02s0025g00800  -  Cation/hydrogen exchanger (CHX18)
## VIT_03s0063g00360  -  Glycoprotein 3-alpha-L-fucosyltransferase
## VIT_08s0056g00160  -  U3 small nucleolar RNA-associated protein 11
## VIT_11s0016g05560  -  Glucan endo-1,3-beta-glucosidase 7 precursor
## VIT_13s0084g00610  -  NADH dehydrogenase (ubiquinone) 1 alpha subcomplex 6
## VIT_17s0000g08080  -  Armadillo/beta-catenin repeat protein / U-box domain-containing protei
## VIT_12s0059g02280  -  Spg1p (septum-promoting GTPase)
## VIT_00s2497g00010  -  U-box domain-containing protein 8
## VIT_08s0007g07320  -  Unknown protein
## VIT_18s0001g13370  -  Transcription initiation factor TFIIF beta subunit (TFIIF-beta)
## VIT_08s0040g01270  -  Dual specificity protein phosphatase
## VIT_14s0068g00850  -  Potassium transporter (KUP3)
## VIT_19s0015g01300  -  Amino acid permease 7
## VIT_08s0007g02570  -  CCR4-NOT transcription complex subunit 7
## VIT_12s0028g03890  -  Unknown protein
## VIT_08s0056g01070  -  MATE efflux family protein
## VIT_04s0079g00530  -  Isocitrate dehydrogenase, chloroplast precursor
## VIT_02s0033g00390  -  VvMybA2
## VIT_09s0002g04280  -  Dynein light chain LC6, flagellar outer arm
## VIT_13s0019g04730  -  Glycerol-3-phosphate dehydrogenase
## VIT_13s0047g01210  -  Phospholipid-translocating ATPase ALA1
## VIT_02s0025g04330  -  Thaumatin VVTL1 [Vitis vinifera]
## VIT_12s0034g02390  -  T-complex protein 11
## VIT_14s0066g00310  -  Unknown protein
## VIT_01s0026g02620  -  Expansin  (VvEXPA1)
## VIT_05s0049g01330  -  GLTP1 (glycolipid transfer protein 1)
## VIT_17s0000g08840  -  Trans-2-enoyl-CoA reductase
## VIT_10s0071g00890  -  Unknown protein
## VIT_08s0007g05140  -  MAP70 protein .
## VIT_06s0061g01110  -  Lecithine cholesterol acyltransferase
## VIT_15s0046g00340  -  Transcription factor jumonji (jmjC) domain-containing protein
## VIT_07s0104g00930  -  Gibberellin receptor GID1L2
## VIT_07s0255g00110  -  WD40
## VIT_15s0048g01920  -  F-box family protein
## VIT_16s0039g00720  -  Folate-biopterin transporter
## VIT_03s0038g02850  -  Protein phosphatase 2 (formerly 2A), regulatory subunit B'
## VIT_02s0025g02740  -  RNA-binding protein RBP37
## VIT_08s0007g05370  -  Strictosidine synthase
## VIT_05s0124g00270  -  Unknown protein
## VIT_14s0030g01670  -  Proteasome 26S regulatory subunit (RPN6)
## VIT_00s0207g00280  -  WAK receptor protein kinase
## VIT_05s0020g04110  -  ELIP1 (early light-inducible protein)
## VIT_01s0150g00430  -  Unknown protein
## VIT_11s0016g05770  -  Alkaline alpha galactosidase 2
## VIT_15s0046g02390  -  ANTR2 (anion transporter 2)
```

```
## VIT_00s2364g00010  -  U-box domain-containing protein 8
## VIT_06s0004g05730  -  Universal stress protein (USP) family protein
## VIT_15s0046g02300  -  Beta-cyanoalanine synthase
## VIT_19s0014g02450  -  ALF5 (Aberrant lateral root formation 5)
## VIT_11s0016g03920  -  Polyol transporter 5
## VIT_17s0000g01730  -  Adenylate kinase
## VIT_01s0011g04640  -  Auxin Efflux Carrier
## VIT_00s0499g00030  -  Stearoyl-CoA 9-desaturase
## VIT_12s0035g01940  -  Unknown protein
## VIT_05s0051g00720  -  3-ketoacyl-CoA thiolase PED1
## VIT_11s0118g00590  -  Unknown protein
## VIT_01s0011g02310  -  Unknown protein
## VIT_18s0001g09290  -  Unknown protein
## VIT_06s0004g07550  -  Wound-induced protein WI12
## VIT_07s0005g01710  -  WRKY Transcription Factor (VvWRKY19)
## VIT_14s0006g03230  -  14-3-3 protein GF14 nu (GRF7)
## VIT_01s0010g02260  -  Esterase
## VIT_10s0003g04180  -  Early-responsive to dehydration stress protein (ERD4)
## VIT_14s0036g00810  -  HMG-CoA synthase 2
## VIT_14s0171g00360  -  Unknown protein
## VIT_04s0023g02940  -  Unknown protein
## VIT_06s0004g03910  -  Unknown protein
## VIT_11s0016g05130  -  Signal peptidase, endoplasmic reticulum-type
## VIT_19s0090g00240  -  R protein disease resistance protein
## VIT_04s0008g01060  -  Substrate carrier, Mitochondrial
## VIT_07s0005g02710  -  Unknown protein
## VIT_14s0060g01980  -  Unknown protein
## VIT_14s0068g01470  -  Hydroxyproline-rich glycoprotein family protein
## VIT_02s0025g02390  -  Oligopeptide transporter OPT7
## VIT_14s0068g00300  -  ABRC5
## VIT_03s0063g01360  -  Unknown protein
## VIT_11s0118g00200  -  Sucrose-phosphate synthase
## VIT_17s0000g09350  -  Dehydration-induced 19
## VIT_10s0003g01430  -  MEE62 (maternal effect embryo arrest 62)
## VIT_05s0049g00040  -  Cellulose synthase CSLG2
## VIT_07s0191g00080  -  GCR2-like 1 ABA receptor
## VIT_03s0038g01380  -  Calcium-binding EF hand
## VIT_09s0002g01850  -  HAB1 (homology toABI1)
## VIT_01s0137g00340  -  Unknown protein
## VIT_11s0016g03560  -  Ethylene-responsive protein
## VIT_05s0020g02590  -  Pentatricopeptide (PPR) repeat-containing protein
## VIT_17s0000g07450  -  Glycerophosphoryl diester phosphodiesterase
## VIT_00s0216g00040  -  ER lumen protein retaining receptor
## VIT_13s0019g00550  -  Cofilin
## VIT_18s0001g07240  -  14-3-3 protein GF14 kappa (GRF8)
## VIT_02s0033g00180  -  Phenylpropanoid:glucosyltransferase 2
## VIT_08s0007g08280  -  Remorin
## VIT_14s0068g00640  -  Acetyl-CoA synthetase
## VIT_13s0019g05170  -  Zinc finger (C2H2 type) family
## VIT_16s0050g02520  -  RNA polymerase sigma subunit SigE (sigE)
## VIT_00s0203g00180  -  Unknown protein
## VIT_12s0057g01400  -  Steroid nuclear receptor, ligand-binding
## VIT_05s0049g01460  -  Calmodulin-binding protein
## VIT_04s0023g03100  -  3-oxo-5-alpha-steroid 4-dehydrogenase, C-terminal
## VIT_08s0040g02280  -  Phosphatidic acid phosphatase / PAP2
```

```
## VIT_17s0000g07300  -  ATP synthase gamma chain, mitochondrial (ATPC)
## VIT_15s0046g01920  -  ferric reduction oxidase 2
## VIT_07s0005g00120  -  Unknown
## VIT_00s0322g00030  -  Alpha-6-galactosyltransferase
## VIT_16s0039g02230  -  UDP glucose:flavonoid 3-o-glucosyltransferase (VvUFGT) GeneID: 1002330
## VIT_17s0000g07820  -  LIM domain protein WLIM1
## VIT_12s0028g03730  -  Hydroxyproline-rich glycoprotein
## VIT_05s0020g01980  -  Acyl carrier protein, mitochondrial 3
## VIT_14s0060g00360  -  Isopenicillin N epimerase
## VIT_17s0000g06190  -  myb domain protein 94 (VvMYB30)
## VIT_18s0001g12420  -  SEC14 cytosolic factor
## VIT_05s0124g00360  -  Protein kinase Pti1
## VIT_01s0011g06250  -  Xyloglucan endotransglycosylase
## VIT_02s0025g03310  -  Arsenite transport protein (ArsB)
## VIT_16s0039g02120  -  NADH dehydrogenase (ubiquinone) Fe-S protein 6
## VIT_19s0014g01000  -  Ubiquitin domain containing 1
## VIT_12s0059g00640  -  Beta-1,3-galactosyltransferase sqv-2
## VIT_17s0000g02140  -  3-hydroxyisobutyrate dehydrogenase
## VIT_00s0239g00110  -  Mei2 AML1
## VIT_05s0062g00760  -  Receptor kinase RHG4
## VIT_14s0006g02620  -  Cgi67 serine protease
## VIT_00s0477g00030  -  MATE efflux family protein ripening responsive
## VIT_05s0049g00760  -  No hit
## VIT_02s0025g02420  -  Major Facilitator Super
## VIT_12s0035g01140  -  RAB GTPase RAB11C
## VIT_09s0002g03690  -  Avr9/Cf-9 rapidly elicited protein 146
## VIT_02s0033g00130  -  Cis-zeatin O-beta-D-glucosyltransferase
## VIT_05s0049g00550  -  No hit
## VIT_07s0141g00110  -  Guanylate-binding
## VIT_08s0007g03760  -  Growth-regulating factor 4 AtGRF4
## VIT_08s0040g01190  -  3-oxoacyl-reductase, chloroplast precursor
## VIT_18s0001g11930  -  Thaumatin SCUTL2
## VIT_08s0056g00310  -  Auxin efflux carrier
## VIT_02s0025g00700  -  Aluminum-activated malate transporter 9
## VIT_07s0005g03670  -  CHR8 (chromatin remodeling 8)
## VIT_04s0008g03100  -  Unknown protein
## VIT_12s0028g03740  -  Hydroxyproline-rich glycoprotein
## VIT_05s0049g00050  -  Cellulose synthase CSLG2
## VIT_04s0008g00720  -  Vacuolar processing enzyme gamma
## VIT_08s0007g00540  -  Haloacid dehalogenase hydrolase
```

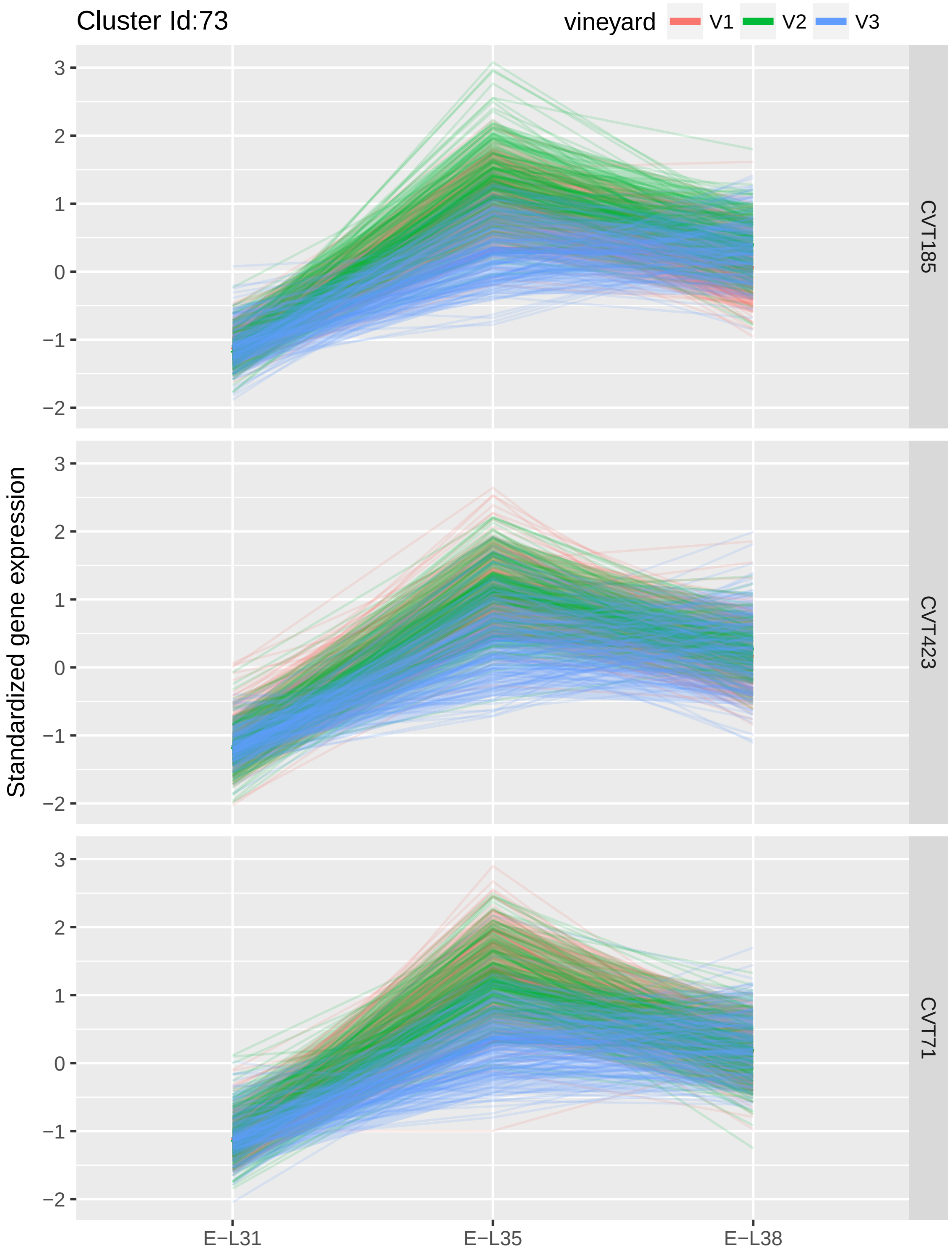
Cluster Id:73
vineyard
V1
V2
V3
Standardized gene expression
CVT185
CVT423
CVT71
3
2
1
0
−1
−2
E−L31
E−L35
E−L38
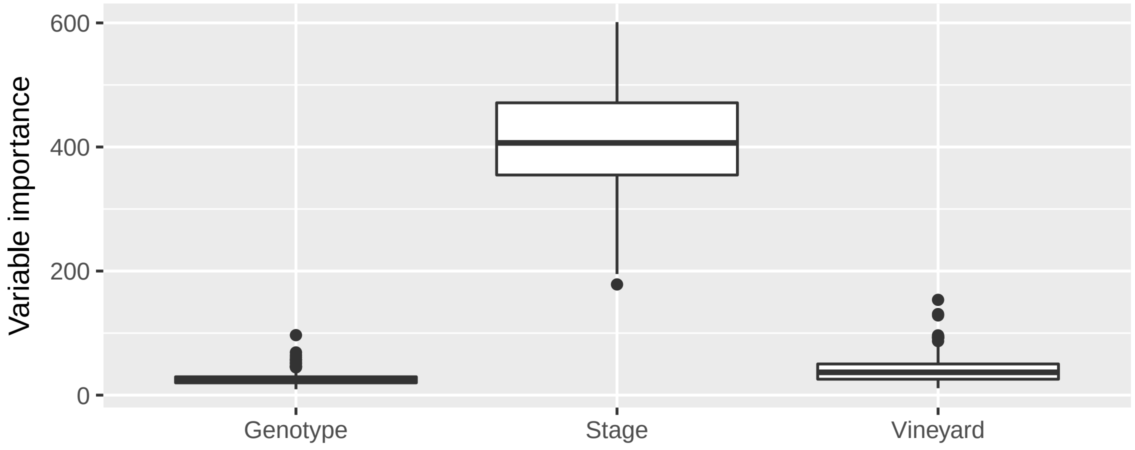
Variable importance
600
400
200
0
Genotype
Stage
Vineyard

Cluster no. 74

```
## Number of genes in the cluster: 59
## Homogeneity Index:    0.68
## Variable importance for Stage:    Median =  125.6  - Rank =  102
## Variable importance for Clone:    Median =  27.67  - Rank =  13
## Variable importance for Vineyard: Median =    124  - Rank =  12
##
## Gene ID               Gene Annotation
## VIT_03s0038g00660  -  Unknown protein
## VIT_19s0090g01090  -  No hit
## VIT_00s0203g00220  -  S-locus lectin protein kinase
## VIT_04s0008g04480  -  Zinc finger (C3HC4-type ring finger)
## VIT_11s0118g00480  -  Leucine-rich repeat
## VIT_13s0064g01280  -  SWAP (Suppressor-of-White-APricot)
## VIT_14s0030g01980  -  Auxin-induced protein PCNT115
## VIT_00s2776g00010  -  S-locus lectin protein kinase
## VIT_15s0046g03620  -  2-oxo acid dehydrogenase, lipoyl-binding site
## VIT_18s0001g15460  -  Stearyl acyl carrier protein desaturase
## VIT_01s0011g05810  -  Adagio protein 1
## VIT_09s0002g00610  -  Ribosomal protein SA (RPSaB) 40S
## VIT_03s0038g03690  -  Unknown protein
## VIT_00s0347g00050  -  Ser/Thr receptor-like kinase1
## VIT_19s0093g00060  -  Polygalacturonase PGA3
## VIT_05s0062g01010  -  Aldo/keto reductase AKR
## VIT_14s0036g00510  -  Cell division cycle 5
## VIT_03s0017g00550  -  Beta-ureidopropionase
## VIT_03s0063g00340  -  No hit
## VIT_17s0000g07900  -  Protease Do 9
## VIT_09s0002g06810  -  Binding
## VIT_08s0040g02760  -  Ankyrin repeat
## VIT_17s0000g01330  -  Unknown protein
## VIT_17s0000g06380  -  YGL010w
## VIT_14s0030g02300  -  CRSP complex subunit 2
## VIT_06s0009g03800  -  PHD finger transcription factor
## VIT_03s0038g00870  -  Dihydrouridine synthase 3
## VIT_09s0002g04180  -  Unknown protein
## VIT_04s0069g01090  -  No hit
## VIT_16s0050g00450  -  DNA-directed RNA polymerase III subunit C3
## VIT_06s0004g04600  -  Protein phosphatase 2C POLTERGEIST-like 4
## VIT_09s0002g01100  -  DAG
## VIT_03s0063g01380  -  Unknown protein
## VIT_18s0041g00630  -  Proton-dependent oligopeptide transport (POT) family protein
## VIT_11s0016g00060  -  No hit
## VIT_17s0000g02840  -  Pre-mRNA-splicing factor SPF27
## VIT_14s0128g00900  -  MORC family CW-type zinc finger 4
## VIT_07s0141g00800  -  FLK (flowering locus KH domain)
## VIT_03s0063g00350  -  Unknown
## VIT_16s0050g01780  -  Unknown protein
## VIT_04s0043g00830  -  Unknown protein
## VIT_07s0151g00360  -  Unknown protein
## VIT_08s0007g00550  -  ftsh3 (FtsH protease 3)
## VIT_19s0014g03560  -  High-level expression of sugar-inducible gene 2
## VIT_00s2623g00010  -  Nuclear transport factor 2 (NTF2)
## VIT_06s0061g01260  -  EMB1211 (embryo defective 1211)
## VIT_14s0006g01440  -  Double-stranded RNA binding protein
```

```
## VIT_03s0063g01080  -  OST1 (open stomata 1) AAPK
## VIT_08s0007g00560  -  ftsh3 (FtsH protease 3)
## VIT_01s0026g02740  -  No hit
## VIT_16s0039g02510  -  Cell division cycle 20, cofactor of APC complex
## VIT_06s0004g07930  -  Nucleotidyltransferase family
## VIT_11s0052g01400  -  Ribitol kinase
## VIT_17s0000g05500  -  Cysteine endopeptidase
## VIT_07s0104g00700  -  Exosome complex component RRP4
## VIT_07s0005g01740  -  Zinc knuckle
## VIT_05s0094g00090  -  Unknown protein
## VIT_07s0005g00180  -  TOC159 (translocon outer membrane complex 159)
## VIT_17s0000g00500  -  Serine C-palmitoyltransferase
```

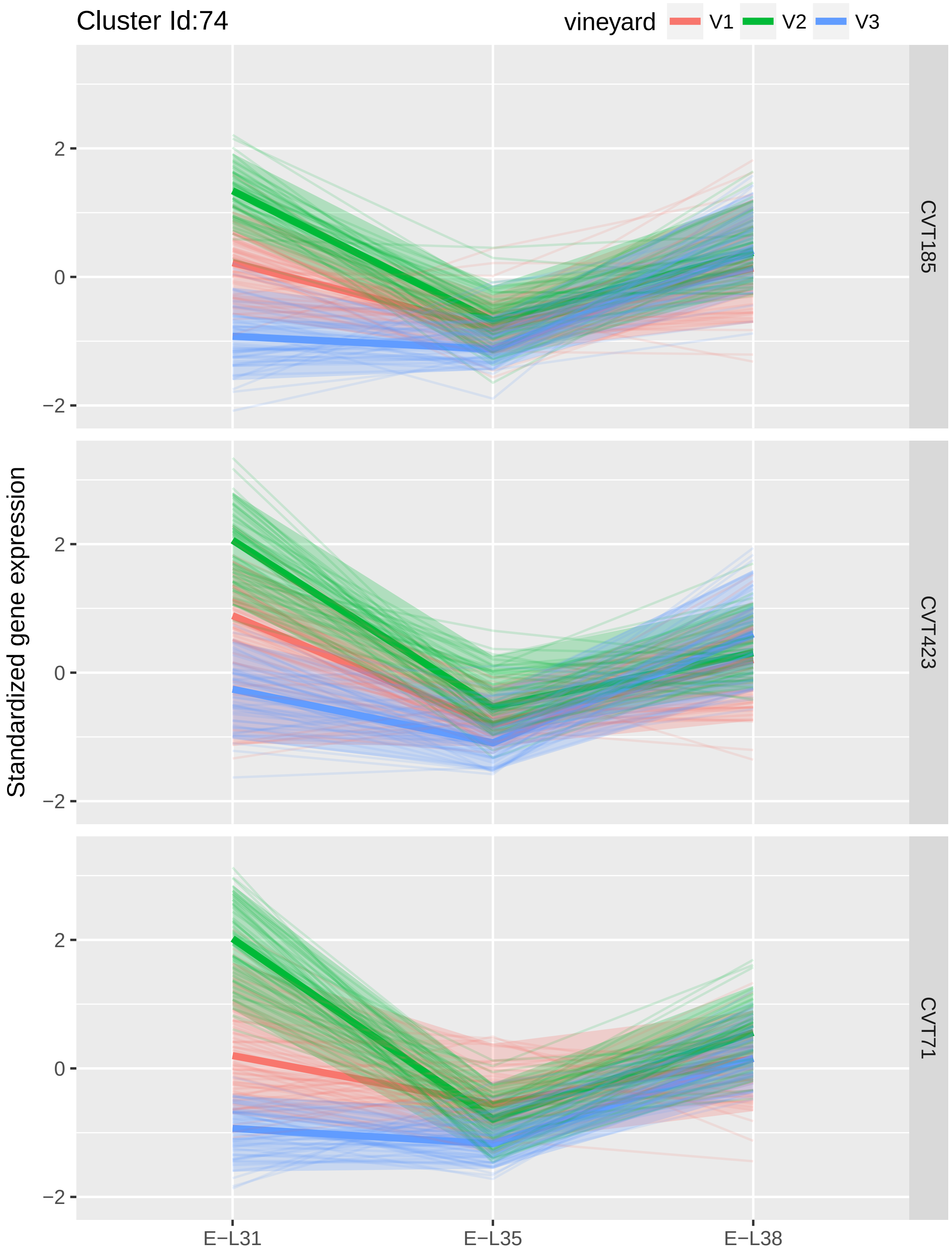
Cluster Id:74
vineyard
V1
V2
V3
Standardized gene expression
2
0
−2
CVT185
CVT423
CVT71
E−L31
E−L35
E−L38

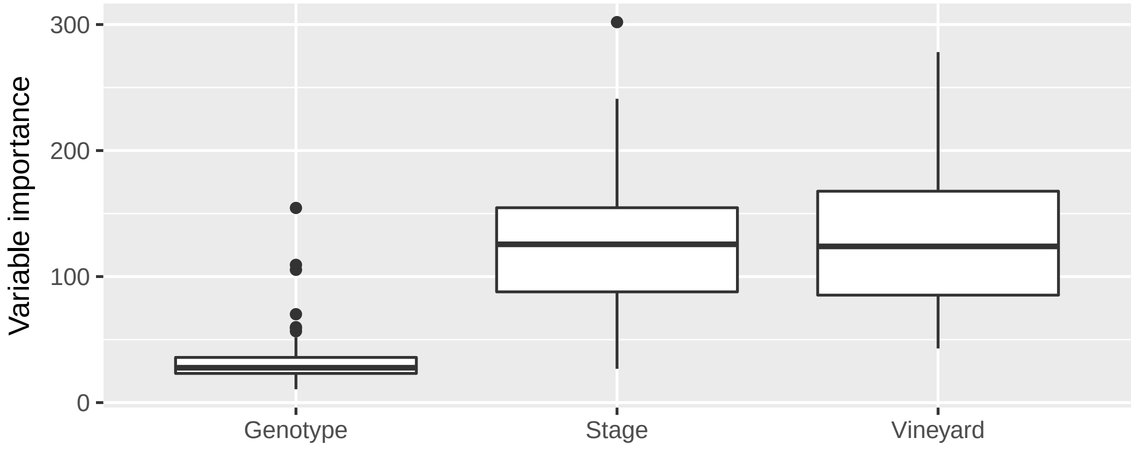
Variable importance
300
200
100
0
Genotype
Stage
Vineyard

Cluster no. 75

```
## Number of genes in the cluster: 174
## Homogeneity Index:    0.73
## Variable importance for Stage:    Median =  346.7  - Rank =  63
## Variable importance for Clone:    Median =  25.53  - Rank =  20
## Variable importance for Vineyard: Median =  37.31  - Rank =  54
##
## Gene ID               Gene Annotation
## VIT_02s0025g03020  -  NAC domain-containing protein (VvNAC25)
## VIT_09s0054g00110  -  No hit
## VIT_18s0122g00630  -  Cinnamoyl-CoA reductase
## VIT_13s0064g01470  -  Urophorphyrin III methylase
## VIT_02s0025g03260  -  Aquaporin NIP5;1
## VIT_08s0007g03530  -  Zf A20 and AN1 domain-containing stress-associated protein 4
## VIT_13s0019g04760  -  Rhamnogalacturonan xylosyltransferase 1
## VIT_17s0000g05620  -  Integral membrane HPP family protein
## VIT_04s0008g00220  -  IAA6
## VIT_08s0007g00500  -  Telomere repeat binding factor like TRFL2
## VIT_17s0053g00040  -  Metal-nicotianamine transporter YSL5
## VIT_18s0001g06260  -  R protein L6
## VIT_00s0487g00050  -  R protein disease resistance protein
## VIT_00s0283g00030  -  Calnexin 1 (CNX1)
## VIT_14s0006g00410  -  Binding
## VIT_05s0020g03470  -  Hydroxyproline-rich glycoprotein
## VIT_09s0002g05660  -  Sulfate transporter Sulp familly
## VIT_13s0106g00370  -  Myb family
## VIT_08s0040g00980  -  Tubulin beta-1 chain
## VIT_07s0197g00090  -  R protein disease resistance protein
## VIT_15s0021g00500  -  NADP-dependent malic enzyme
## VIT_09s0002g02590  -  Unknown protein
## VIT_07s0197g00150  -  Disease resistance protein (CC-NBS-LRR class)
## VIT_13s0175g00080  -  Calcium Dependent Protein Kinase (VvCPK13)
## VIT_11s0016g03230  -  Unknown protein
## VIT_03s0091g00380  -  Thioredoxin domain-containing protein 9
## VIT_18s0001g09130  -  Armadillo/beta-catenin repeat
## VIT_19s0085g00740  -  ZIP family transporter
## VIT_05s0077g00450  -  Ribosomal protein S1 30S
## VIT_18s0089g00520  -  R protein L6
## VIT_10s0116g00690  -  No hit
## VIT_04s0008g00630  -  SNF7.2
## VIT_05s0102g00610  -  No hit
## VIT_05s0049g01220  -  Protein kinase MK6
## VIT_01s0011g02360  -  Unknown
## VIT_14s0108g01180  -  Unknown protein
## VIT_03s0088g00270  -  No hit
## VIT_13s0019g02400  -  Transducin protein
## VIT_14s0006g02690  -  Delta 7-sterol-C5-desaturase (STE1)
## VIT_18s0001g07220  -  Cyclin delta-2
## VIT_14s0068g00290  -  RPK1 (receptor-like protein kinase 1)
## VIT_04s0044g01200  -  Auxin-independent growth promoter
## VIT_18s0122g00230  -  Lys Motif-Type Receptor-Like Kinase LYK8
## VIT_09s0002g00130  -  H(+)-ATPase (VvPH5)
## VIT_17s0000g04850  -  OBF binding protein 4
## VIT_06s0009g03360  -  Unknown protein
## VIT_07s0005g04340  -  Unknown protein
```

```
## VIT_01s0113g00470  -  Unknown protein
## VIT_19s0090g01440  -  Fimbrin 2
## VIT_08s0040g02190  -  Unknown
## VIT_04s0023g03060  -  Kinesin motor protein
## VIT_19s0014g04990  -  No hit
## VIT_07s0191g00220  -  Unknown protein
## VIT_08s0007g08230  -  Curculin (mannose-binding) lectin
## VIT_01s0011g01280  -  Zinc transporter ZIP5
## VIT_18s0089g00920  -  RNA binding motif protein 38
## VIT_18s0001g05700  -  Unknown protein
## VIT_16s0039g02180  -  NADH dehydrogenase subunit B17.2
## VIT_06s0009g01790  -  ABA-responsive element-binding protein 3 (AREB3) (VvABF-4), Basic Leuc
## VIT_08s0007g00150  -  Zinc finger (C3HC4-type ring finger)
## VIT_00s2871g00010  -  Yippee
## VIT_19s0014g02670  -  Protein kinase CRK1 CRK1 protein(Cdc2-related kinase 1)
## VIT_13s0158g00410  -  Nucleic acid binding
## VIT_03s0088g00380  -  Tubulin alpha
## VIT_02s0025g04770  -  Unknown protein
## VIT_08s0007g02950  -  Unknown protein
## VIT_00s1194g00020  -  No hit
## VIT_07s0185g00030  -  Ubiquitin-associated (UBA)/TS-N domain-containing protein
## VIT_05s0020g00670  -  S-adenosylmethionine synthetase 1 (SAM1)
## VIT_12s0059g00770  -  Monocopper oxidase SKS5 (SKU5 Similar 5)
## VIT_09s0002g01710  -  Zeta-cop, subunit of COP-I complex
## VIT_02s0025g01330  -  Polygalacturonase GH28
## VIT_17s0000g03500  -  Mov34 STAM-binding protein
## VIT_15s0048g02520  -  Zinc transporter (ZAT)
## VIT_07s0005g04350  -  No hit
## VIT_11s0016g04020  -  Ras-related protein Rab-5A
## VIT_19s0014g00110  -  Protein kinase
## VIT_12s0142g00360  -  putative MADS-box Agamous 1 (VviAG1)
## VIT_09s0018g02130  -  Calcium-transporting ATPase 9 ACA9
## VIT_01s0011g05790  -  Microtubule associated protein (MAP70-1)
## VIT_06s0004g01160  -  10-formyltetrahydrofolate synthetase
## VIT_18s0001g11860  -  Polygalacturonate 4-alpha-galacturonosyltransferase
## VIT_19s0014g00460  -  PMR5 (powdery mildew resistant 5)
## VIT_18s0001g08800  -  Lecithine cholesterol acyltransferase-like protein
## VIT_08s0007g06000  -  Glucan endo-1,3-beta-glucosidase GII precursor
## VIT_05s0102g01180  -  Hydroxyacylglutathione hydrolase 2
## VIT_04s0044g01620  -  RGLG1 (ring Domain LIGASE1)
## VIT_01s0127g00600  -  DC1 domain-containing protein
## VIT_08s0007g01690  -  Phosphate translocator protein2, Plastidic
## VIT_08s0217g00060  -  Importin 11
## VIT_17s0000g06310  -  Cycling DOF factor 2
## VIT_01s0150g00180  -  Exostosin
## VIT_16s0039g00390  -  Unknown protein
## VIT_17s0000g03170  -  Phosphatidic acid phosphatase
## VIT_19s0014g03000  -  Leucine-rich repeat-containing protein 40
## VIT_10s0042g00950  -  Succinyl-CoA ligase alpha 1 subunit
## VIT_18s0001g14040  -  Endo-1,4-beta-glucanase
## VIT_12s0028g01750  -  Cell wall hydrolase
## VIT_11s0016g01310  -  TT2 (transparent testa 2)
## VIT_07s0005g03630  -  Ankyrin
## VIT_06s0004g00830  -  Serine/threonine protein phosphatase PP2A-4 catalytic subunit (PP2A4)
## VIT_05s0020g03890  -  Kinase interacting
```

```
## VIT_19s0085g01030  -  Metacaspase TYPE-II
## VIT_05s0029g00420  -  Nucleotide sugar epimerase
## VIT_14s0060g00980  -  Unknown
## VIT_16s0050g00830  -  Beta-ketoacyl-CoA synthase
## VIT_05s0077g00370  -  Heat stable protein 1 HS1
## VIT_05s0051g00650  -  Potassium channel protein
## VIT_02s0012g01960  -  Transcription factor jumonji (jmj)
## VIT_02s0025g03480  -  Kinesin motor domain containing protein
## VIT_17s0000g08260  -  Kinesin light chain
## VIT_04s0023g03180  -  Zinc finger (CCCH-type) family protein
## VIT_04s0008g06290  -  Receptor Like Protein 15 RLP15
## VIT_03s0063g00810  -  Carboxyesterase 12; CXE12
## VIT_11s0016g05870  -  Unknown protein
## VIT_00s0204g00040  -  Unknown protein
## VIT_05s0020g02800  -  Carbohydrate kinase
## VIT_08s0007g04520  -  Caffeic acid 3-O-methyltransferase
## VIT_06s0004g06760  -  NDA2 (alternative NAD(P)H dehydrogenase 2)
## VIT_11s0016g00600  -  Beta-ketoacyl ACP synthase, plastidic
## VIT_15s0048g00170  -  H(+)-ATPase 4 AHA4
## VIT_11s0016g01950  -  Casein kinase I delta
## VIT_03s0132g00100  -  ATHVA22A (Arabidopsis thaliana HVA22 homologue A)
## VIT_08s0032g00150  -  F-box family protein
## VIT_13s0156g00140  -  Zinc finger (C3HC4-type ring finger)
## VIT_03s0063g00290  -  Methylcrotonoyl-CoA carboxylase beta chain
## VIT_05s0020g02030  -  Shikimate dehydrogenase
## VIT_10s0003g05470  -  FAD-binding domain-containing protein
## VIT_06s0004g07070  -  Protein kinase
## VIT_01s0011g03060  -  Symbiosis receptor-like kinase
## VIT_08s0007g00530  -  3-beta hydroxysteroid dehydrogenase/isomerase protein
## VIT_17s0000g02260  -  Aldose 1-epimerase
## VIT_19s0090g01740  -  Scarecrow transcription factor 5 (SCL5)
## VIT_12s0035g01960  -  Stichel
## VIT_02s0154g00100  -  Transaldolase totaL2
## VIT_10s0003g02060  -  Ankyrin protein kinase
## VIT_11s0052g01800  -  Constans-like 5
## VIT_17s0000g09150  -  ATP binding
## VIT_13s0073g00170  -  Exocyst subunit EXO70 A1
## VIT_12s0059g01980  -  Ultrapetala1
## VIT_05s0049g00300  -  1-aminocyclopropane-1-carboxylate oxidase homolog 1
## VIT_01s0137g00630  -  Alpha-galactosidase/ hydrolase, hydrolyzing O-glycosyl compounds
## VIT_11s0016g02280  -  Glycine dehydrogenase 2
## VIT_00s0260g00060  -  FK506-binding protein genes family (VvFKBP15)
## VIT_19s0090g00440  -  Unknown protein
## VIT_12s0057g01310  -  SUB1 (short under blue light 1)
## VIT_04s0044g01750  -  Thioredoxin reductase 2
## VIT_02s0025g01380  -  Endo-1,4-beta-glucanase
## VIT_13s0084g00830  -  Unknown protein
## VIT_18s0001g09350  -  No hit
## VIT_15s0046g02420  -  Aquaporin TMP-C
## VIT_18s0089g00600  -  R protein L6
## VIT_02s0025g02260  -  ORMDL protein
## VIT_18s0001g09080  -  No hit
## VIT_04s0008g00640  -  Epsin N-terminal homology (ENTH) domain-containing
## VIT_05s0020g02220  -  Prolyl 4-hydroxylase alpha subunit
## VIT_05s0020g00720  -  Pto kinase interactor 1
```

```
## VIT_07s0104g01370  -  Zinc finger (C3HC4-type ring finger)
## VIT_15s0024g00070  -  Methylenetetrahydrofolate reductase 1 (AtMTHFR1)
## VIT_02s0012g01410  -  TRN2 (TORNADO 2)
## VIT_03s0091g01060  -  Cyclin delta-2
## VIT_18s0001g12470  -  Dehydration-responsive protein
## VIT_08s0058g00130  -  BCL2 binding anthogene
## VIT_15s0021g01860  -  Unknown protein
## VIT_00s0710g00020  -  SNF1-related protein kinase 2.7 SNRK2.7 SRK2F
## VIT_11s0016g00820  -  Unknown
## VIT_11s0016g04540  -  ABC Transporter (VvPDR1 - VvABCG31)
## VIT_08s0056g01570  -  5-methyltetrahydropteroyltriglutamate--homocysteine S-methyltransferas
## VIT_15s0046g03520  -  Phosphomannomutase
## VIT_06s0061g00860  -  Zinc-binding protein
## VIT_00s0652g00010  -  Dof zinc finger protein DOF1.4
## VIT_18s0001g05370  -  Unknown protein
## VIT_09s0002g09150  -  RAE1 RNA export 1 homolog
## VIT_01s0127g00810  -  Adaptor-related protein complex 1, sigma 2
```

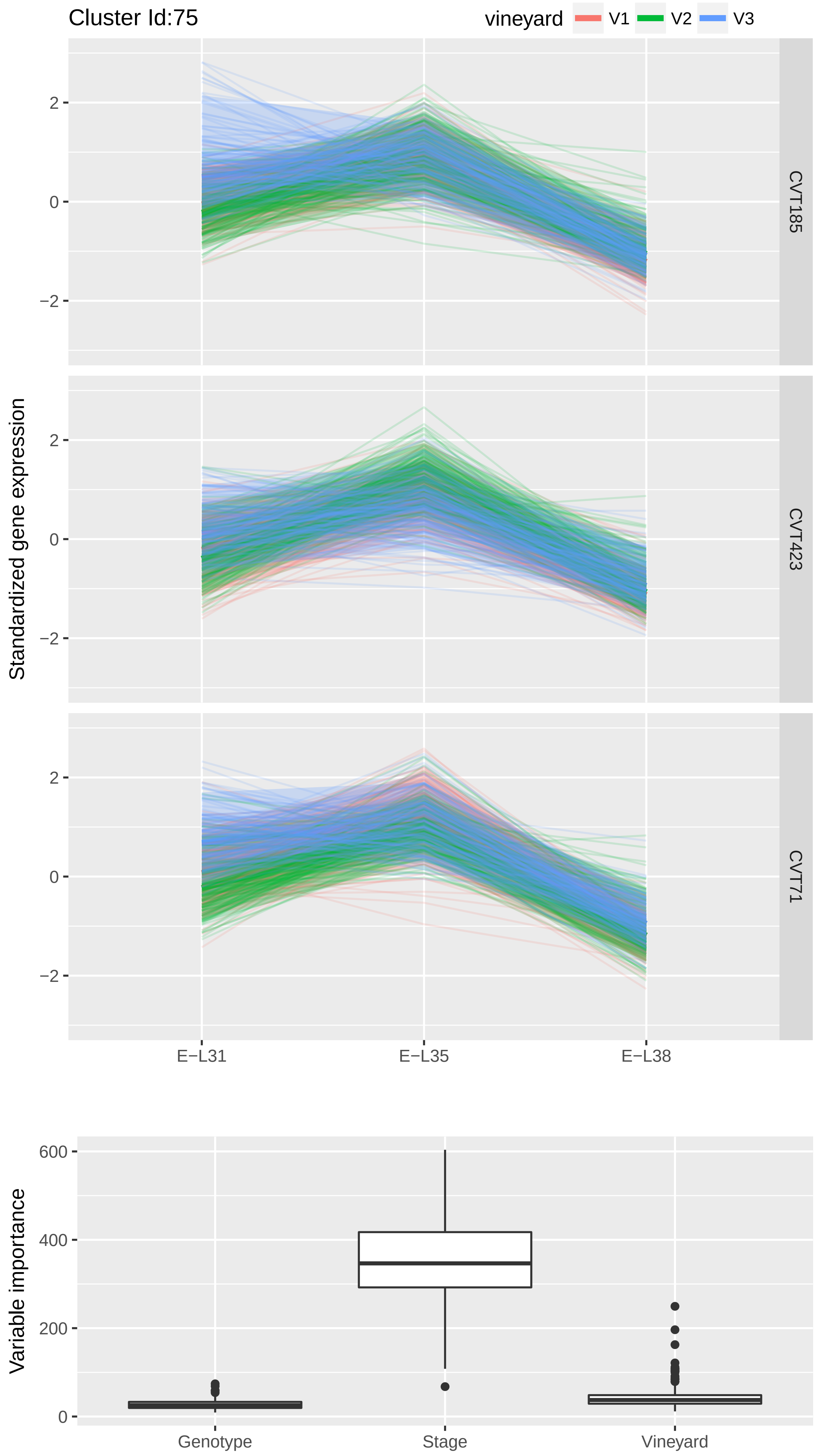

Cluster Id:75
vineyard
V1
V2
V3
Standardized gene expression
CVT185
CVT423
CVT71
E−L31
E−L35
E−L38
Variable importance
Genotype
Stage
Vineyard

Cluster no. 76

```
## Number of genes in the cluster: 48
## Homogeneity Index:    0.53
## Variable importance for Stage:    Median =  204.6  - Rank =  89
## Variable importance for Clone:    Median =  23.13  - Rank =  32
## Variable importance for Vineyard: Median =  93.63  - Rank =  17
##
## Gene ID                Gene Annotation
## VIT_09s0002g03800  -  RPS5 (resistant to p. syringae 5)
## VIT_15s0048g02020  -  EMB2744 (embryo defective 2744)
## VIT_16s0050g02440  -  HAT dimerisation domain-containing protein
## VIT_08s0007g04700  -  Phosphatidylinositol-4-phosphate 5-kinase
## VIT_13s0019g04820  -  Amine oxidase FLD (flowering locus D)
## VIT_06s0009g02740  -  Pentatricopeptide (PPR) repeat-containing protein
## VIT_18s0001g14940  -  Pentatricopeptide (PPR) repeat-containing
## VIT_06s0004g02070  -  GTP-binding protein engA
## VIT_09s0002g00250  -  Disease resistance protein (NBS-LRR class)
## VIT_18s0122g00660  -  RNA recognition motif (RRM)-containing protein
## VIT_00s0467g00010  -  RPM1 (resistance to p. syringae pv maculicola 1)
## VIT_04s0023g00390  -  Tetratricopeptide helical
## VIT_16s0039g00900  -  CYP89A28
## VIT_13s0019g03690  -  fertility restorer salt-inducible
## VIT_06s0004g03740  -  DNA-directed RNA polymerase III subunit C1
## VIT_17s0000g02320  -  Glutaredoxin
## VIT_16s0022g00970  -  Pentatricopeptide (PPR) repeat-containing protein
## VIT_06s0080g00370  -  F-box domain containing protein
## VIT_10s0042g01260  -  Transcription termination factor mitochondrial mTERF
## VIT_02s0012g01760  -  Pentatricopeptide repeat
## VIT_11s0016g04580  -  ABC Transporter (VvPDR2 - VvABCG32)
## VIT_04s0023g01060  -  Proton gradient regulation 3
## VIT_06s0004g03920  -  Pto serine/threonine kinase
## VIT_07s0005g01420  -  Transducin protein
## VIT_08s0007g08660  -  Binding
## VIT_14s0108g00130  -  GTP-binding protein LepA
## VIT_06s0004g06630  -  DTA2 (downstream target of AGL15 2)
## VIT_01s0026g01710  -  Zinc knuckle
## VIT_16s0039g00910  -  CYP89A28
## VIT_15s0048g00690  -  Pentatricopeptide (PPR) repeat-containing protein
## VIT_01s0010g03950  -  Pentatricopeptide (PPR) repeat-containing protein
## VIT_11s0016g00480  -  Pectinesterase PME2
## VIT_15s0024g01460  -  FtsH protease
## VIT_04s0008g06600  -  WRKY DNA-binding protein 32 (WRKY-15), WRKY Transcription Factor (VvWR
## VIT_03s0038g02080  -  Pentatricopeptide (PPR) repeat-containing protein
## VIT_08s0040g03240  -  Pentatricopeptide (PPR) repeat
## VIT_12s0035g01990  -  Intron maturase, type II
## VIT_03s0063g00900  -  Pentatricopeptide (PPR) repeat-containing protein
## VIT_04s0023g02340  -  Unknown protein
## VIT_02s0087g00080  -  Pentatricopeptide (PPR) repeat-containing protein
## VIT_00s0204g00120  -  Subtilisin-like serine protease 3
## VIT_14s0006g00170  -  Pentatricopeptide (PPR) repeat-containing protein
## VIT_06s0004g03730  -  DNA-directed RNA polymerase III subunit C1
## VIT_16s0039g00870  -  CYP89A5
## VIT_06s0004g07180  -  Pentatricopeptide repeat-containing
## VIT_01s0150g00620  -  Unknown protein
## VIT_19s0015g00110  -  CYP71D10
```

```
## VIT_15s0048g02830  -  Rac GTPase activating protein
```

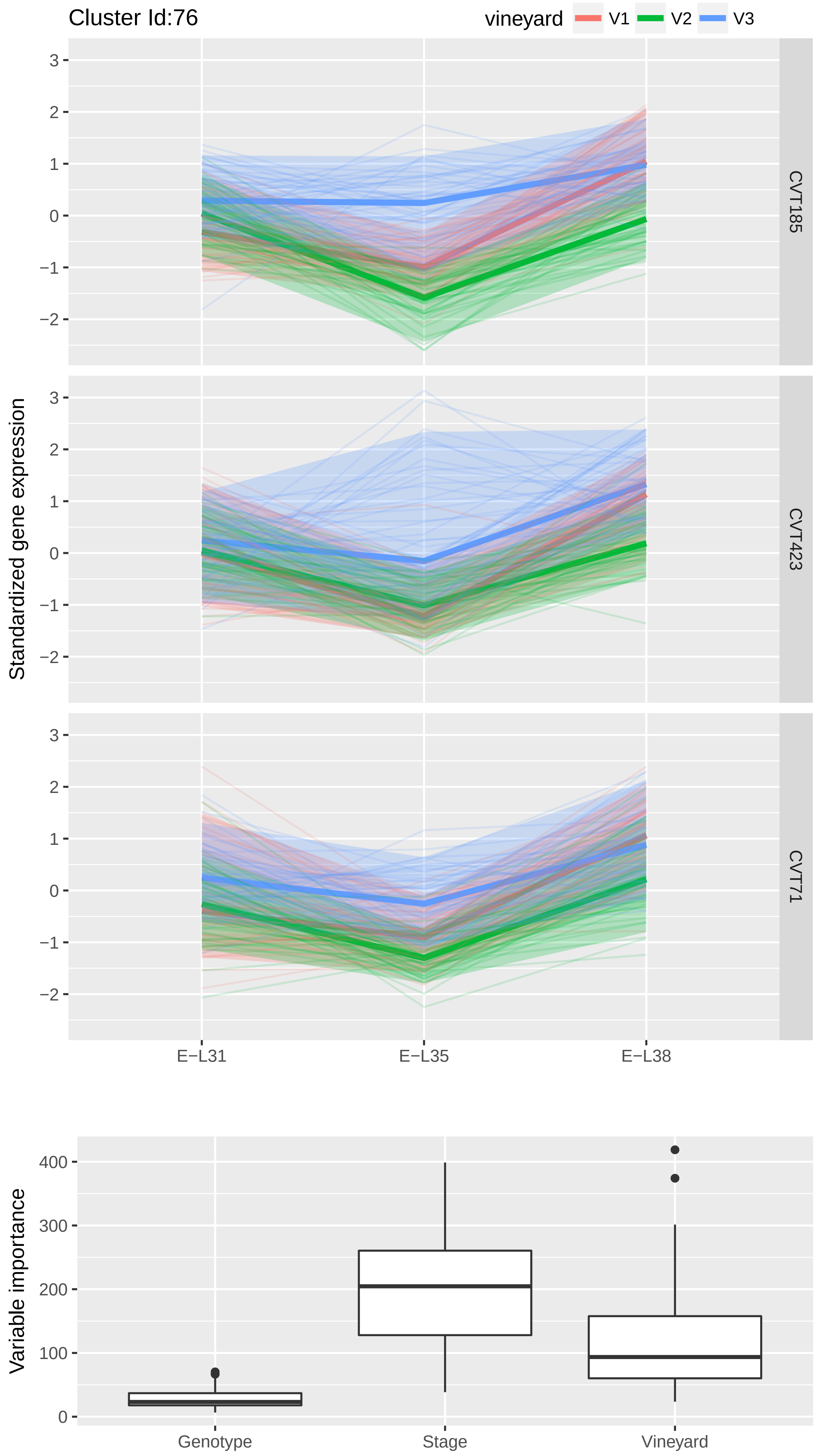

Cluster Id:76
vineyard
V1
V2
V3
CVT185
CVT423
CVT71
Standardized gene expression
E−L31
E−L35
E−L38
Variable importance
Genotype
Stage
Vineyard

Cluster no. 77

```
## Number of genes in the cluster: 218
## Homogeneity Index:    0.69
## Variable importance for Stage:    Median =  491.2  - Rank =  34
## Variable importance for Clone:    Median =  16.82  - Rank =  67
## Variable importance for Vineyard: Median =  33.45  - Rank =  62
##
## Gene ID               Gene Annotation
## VIT_00s0227g00110  -  Gamma hydroxybutyrate dehydrogenase-like protein
## VIT_13s0106g00500  -  RNA recognition motif (RRM)-containing protein
## VIT_02s0025g01590  -  Triacylglycerol lipase
## VIT_09s0002g05170  -  Unknown protein
## VIT_00s0225g00140  -  Ribose-phosphate pyrophosphokinase 3
## VIT_01s0011g01710  -  Exocyst complex component EXO84 C
## VIT_19s0015g01730  -  Uridine/cytidine kinase 1
## VIT_01s0010g03570  -  NLI interacting factor (NIF)
## VIT_00s0207g00010  -  Anthranilate N-benzoyltransferase protein 1
## VIT_03s0063g00390  -  Lag one homologue 2
## VIT_08s0058g01340  -  Leucine-rich repeat transmembrane protein kinase
## VIT_06s0004g05020  -  Haloacid dehalogenase hydrolase
## VIT_13s0064g00650  -  ATAB2
## VIT_05s0051g00440  -  Transmembrane protein FT27/PFT27
## VIT_06s0009g00880  -  Axial regulator YABBY2
## VIT_08s0007g00130  -  Heat shock protein 70
## VIT_07s0031g03200  -  ferrochelatase II
## VIT_03s0088g00400  -  Tubulin alpha-6 chain
## VIT_02s0025g00370  -  2-C-methyl-D-erythritol 2,4-cyclodiphosphate synthase, chloroplast
## VIT_18s0001g01970  -  F-actin capping protein beta subunit
## VIT_16s0050g01440  -  Unknown protein
## VIT_00s0229g00140  -  Enoyl-CoA hydratase
## VIT_12s0059g01810  -  Photosystem II psbZ
## VIT_03s0038g03910  -  ANTR2 (anion transporter 2)
## VIT_01s0011g02990  -  NAC domain-containing protein (VvNAC12)
## VIT_07s0031g01560  -  Unknown protein
## VIT_14s0108g01490  -  Pentatricopeptide (PPR) repeat-containing protein
## VIT_04s0008g03780  -  Myb-related protein 3R-1 (Plant c-MYB-like protein 1) MYB3R1
## VIT_18s0157g00200  -  Steroleosin-B
## VIT_03s0038g00740  -  GC1 (GIANT chloroplast 1)
## VIT_19s0090g01750  -  Unknown protein
## VIT_11s0118g00310  -  1,2-diacylglycerol 3-beta-galactosyltransferase
## VIT_16s0050g00960  -  Prolyl-tRNA synthetase
## VIT_18s0001g03080  -  Chitin elicitor-binding CEBIP LysM domain-containing
## VIT_15s0046g02730  -  R protein  PRF  disease resistance protein
## VIT_05s0020g04040  -  Chlorophyllase (CLH2)
## VIT_12s0057g00020  -  Protein thylakoid formation1
## VIT_10s0003g02750  -  IMP dehydrogenase/GMP reductase
## VIT_15s0046g00500  -  Wax synthase
## VIT_17s0000g06610  -  Zinc finger (C3HC4-type ring finger)
## VIT_11s0065g00180  -  Proton extrusion protein CemA family protein pcxA
## VIT_06s0004g00610  -  Accelerated cell death 1 ACD1
## VIT_17s0000g08590  -  Unknown protein
## VIT_16s0022g02350  -  ACI13
## VIT_07s0005g03740  -  RabGAP/TBC domain-containing protein
## VIT_14s0060g01580  -  Protein phosphatase 2C
## VIT_02s0025g02770  -  Glucan endo-1,3-beta-glucosidase 7 precursor
```

```
## VIT_07s0005g00460  -  Complex 1 LYR
## VIT_06s0004g08210  -  Receptor protein kinase
## VIT_05s0094g00130  -  DNA-directed RNA polymerase II subunit H
## VIT_08s0007g07970  -  Serine/threonine-protein kinase Nek4
## VIT_18s0001g05480  -  Unknown protein
## VIT_16s0039g01650  -  Transporter-related
## VIT_04s0008g05060  -  C-5 cytosine-specific DNA methylase
## VIT_18s0001g01250  -  Senescence-inducible chloroplast stay-green protein 2
## VIT_18s0001g00470  -  Monocopper oxidase SKS5 (SKU5 Similar 5)
## VIT_06s0004g08070  -  Basic Leucine Zipper Transcription Factor (VvbZIP17)
## VIT_18s0001g12900  -  S-adenosyl-L-methionine:salicylic acid carboxyl methyltransferase
## VIT_08s0007g02500  -  Transcription termination factor mitochondrial mTERF
## VIT_01s0137g00250  -  T-complex protein 1 epsilon subunit
## VIT_03s0088g00260  -  Serine carboxypeptidase S10
## VIT_11s0016g04320  -  Nudix hydrolase 20
## VIT_07s0151g00310  -  NIMA protein kinase NEK1
## VIT_01s0011g02870  -  Related to Ubiquitin 1 (RUB1)
## VIT_07s0141g00700  -  R protein MLA10
## VIT_01s0011g00850  -  Unknown protein
## VIT_01s0011g00600  -  Acclimation of photosynthesis to environment
## VIT_09s0054g00220  -  Cation efflux family protein MTPc4
## VIT_15s0046g00090  -  DNA-directed RNA polymerase III subunit C11
## VIT_11s0052g00890  -  Unknown protein
## VIT_09s0002g01150  -  RelA/SpoT protein (RSH3)
## VIT_05s0094g00390  -  Unknown protein
## VIT_12s0059g00570  -  fasciclin arabinogalactan-protein (FLA7)
## VIT_04s0043g01010  -  violaxanthin de-epoxidase (VDE1) (VvVDE1)
## VIT_15s0048g01650  -  WD-40 repeat family protein / beige-related
## VIT_05s0062g00830  -  Ribosomal protein S1
## VIT_08s0040g02210  -  Lectin protein kinase
## VIT_04s0023g01040  -  LIM domain containing protein
## VIT_18s0122g00710  -  3-beta-hydroxy-delta5-steroid dehydrogenase
## VIT_07s0031g01420  -  ATATH13
## VIT_12s0035g01430  -  Zinc finger (CCCH-type) family protein
## VIT_11s0016g03710  -  Abl interactor protein 1 (ABIL1)
## VIT_18s0001g12770  -  Trichohyalin AUL1 (auxin-like 1 protein)
## VIT_01s0127g00220  -  PMR5 (powdery mildew resistant 5)
## VIT_19s0015g02940  -  CYP72A59
## VIT_00s2507g00010  -  F-box family protein
## VIT_06s0004g07990  -  Metalloendopeptidase
## VIT_17s0000g04820  -  BSD domain-containing protein
## VIT_17s0000g07850  -  Nucleobase-ascorbate transporter 6 (NAT6)
## VIT_01s0010g02160  -  RGS1 (regulator of G-protein SIGNALING 1)
## VIT_18s0001g13050  -  Protein translocase Tic20
## VIT_05s0077g00060  -  D1 protease
## VIT_14s0128g00020  -  Na+/H+ exchanger
## VIT_14s0108g00020  -  PIN8 (PIN-formed 8); auxin:hydrogen symporter/ transporter
## VIT_14s0006g00910  -  ATA15 protein
## VIT_07s0151g01060  -  Chalcone isomerase
## VIT_15s0045g01310  -  UMP-CMP kinase
## VIT_12s0059g00520  -  RabGAP/TBC domain-containing protein
## VIT_02s0154g00140  -  3-oxoacyl-[acyl-carrier-protein] synthase 3 A, chloroplast precursor
## VIT_07s0005g00050  -  Heavy-metal-associated domain-containing protein
## VIT_18s0072g01000  -  Dihydrolipoamide S-acetyltransferase
## VIT_11s0052g00130  -  Calcineurin phosphoesterase
```

```
## VIT_11s0016g01270  -  Unknown protein
## VIT_04s0008g03850  -  Pentatricopeptide (PPR) repeat
## VIT_16s0039g00660  -  RNA polymerase nonessential primary-like sigma factor SIGA
## VIT_14s0036g00970  -  Thioredoxin domain-containing protein 9
## VIT_18s0001g14090  -  Molecular chaperone DnaJ
## VIT_00s0333g00070  -  Unknown protein
## VIT_02s0087g00630  -  Alcohol oxidase
## VIT_04s0023g02390  -  Thioredoxin domain-containing protein 9
## VIT_04s0023g03030  -  Zinc finger (B-box type)
## VIT_03s0091g00970  -  ADP-ribosylation factor-like A1D
## VIT_17s0000g04020  -  ATP-dependent Clp protease adaptor protein ClpS containing protein
## VIT_08s0040g03220  -  LCL1 (LHY/CCA1-like 1)
## VIT_16s0013g00940  -  Kinetochore protein NDC80
## VIT_13s0156g00200  -  Unknown protein
## VIT_17s0000g09310  -  Unknown protein
## VIT_12s0035g00310  -  Protein kinase SPK-3 ASK1 (SnRK-6)
## VIT_19s0014g00100  -  Chalcone isomerase 3
## VIT_17s0000g04680  -  Signal peptide peptidase SPPA
## VIT_13s0067g02570  -  Potassium transporter 2
## VIT_18s0001g09340  -  Acyl-CoA binding protein
## VIT_14s0006g00630  -  Dehydroascorbate reductase
## VIT_06s0004g00120  -  Zinc finger (C3HC4-type ring finger)
## VIT_15s0046g02020  -  BPS1 (BYPASS 1)
## VIT_07s0141g00660  -  Signal recognition particle subunit 9 SRP9
## VIT_07s0031g02510  -  Sirtuin (silent mating type information regulation 2 homolog)
## VIT_01s0011g00620  -  14-3-3 protein GF14 iota (GRF12)
## VIT_06s0004g04700  -  Outer envelope protein 16
## VIT_18s0072g00240  -  Zinc finger (DHHC type)
## VIT_17s0000g05370  -  MYC ZCW32 (bigpetal, bigpetalub)
## VIT_12s0059g00450  -  DNA topoisomerase I
## VIT_05s0020g03370  -  5-nucleotidase surE
## VIT_17s0000g02010  -  Atypical receptor kinase MARK
## VIT_17s0000g00720  -  DNA-binding protein
## VIT_17s0000g00460  -  V-type H+-transporting ATPase subunit C
## VIT_17s0000g09740  -  Esterase
## VIT_12s0059g01590  -  Lipase GDSL
## VIT_07s0031g02690  -  NADH dehydrogenase
## VIT_04s0023g01560  -  Unknown protein
## VIT_01s0011g00500  -  RUS1 (Root uvb sensitive 1)
## VIT_11s0037g01060  -  Metal-dependent phosphohydrolase HD domain-containing protein
## VIT_15s0045g01170  -  3-hydroxyisobutyryl-CoA hydrolase
## VIT_04s0008g00710  -  Unknown
## VIT_03s0038g03640  -  Phosphoglycerate mutase
## VIT_12s0034g00240  -  Mekk1
## VIT_08s0007g05420  -  Tubulin folding cofactor B
## VIT_13s0019g05290  -  Acyl-CoA binding
## VIT_00s0233g00010  -  No hit
## VIT_11s0037g01370  -  Ankyrin
## VIT_18s0122g00610  -  3-hydroxy-3-methylglutaryl-coenzyme A reductase 3
## VIT_16s0115g00160  -  LEM3 (ligand-effect modulator 3)
## VIT_08s0007g05010  -  Unknown protein
## VIT_01s0011g00640  -  Hcr2-0B
## VIT_07s0031g02010  -  LCV2 (like COV 2)
## VIT_02s0154g00090  -  Vacuolar invertase 2, GIN2
## VIT_11s0078g00420  -  Galactokinase
```

```
## VIT_16s0050g00750  -  Short-chain dehydrogenase/reductase
## VIT_02s0025g03760  -  Myosin-related
## VIT_08s0056g00240  -  Unknown protein
## VIT_04s0008g02500  -  Hydroxyproline-rich glycoprotein LKP2
## VIT_11s0103g00420  -  XH/XS domain-containing protein
## VIT_14s0030g01900  -  Ribose-5-phosphate isomerase
## VIT_17s0000g00710  -  DNA-binding protein
## VIT_11s0016g02090  -  Esterase
## VIT_17s0000g05190  -  Unknown protein
## VIT_12s0059g02660  -  No hit
## VIT_09s0054g00790  -  Brittle-1, chloroplast precursor
## VIT_18s0001g14240  -  Adaptor-related protein complex 2, sigma 1 sub
## VIT_00s2527g00010  -  Beta-fructosidase (BFRUCT3)
## VIT_00s0659g00010  -  Unknown
## VIT_12s0057g01220  -  Emp24/gp25L/p24 family protein
## VIT_18s0072g00410  -  Unknown protein
## VIT_13s0019g03280  -  LIM domain containing protein
## VIT_08s0007g01640  -  Fructokinase
## VIT_03s0038g02000  -  Amidase
## VIT_13s0067g03110  -  HEAT repeat-containing protein
## VIT_08s0007g05560  -  Protein kinase
## VIT_04s0008g04060  -  RD22
## VIT_01s0010g00950  -  Aminoalcoholphosphotransferase (AAPT1)
## VIT_00s0211g00050  -  Protein BRICK1 (AtBRK1)
## VIT_14s0006g02530  -  Non-specific lipid-transfer protein 2 (LTP 2)
## VIT_04s0044g01480  -  3'(2'),5'-bisphosphate nucleotidase
## VIT_05s0062g00720  -  UDP-glucoronosyl/UDP-glucosyl transferase UGT75C1
## VIT_00s0391g00040  -  F-box family protein
## VIT_14s0068g00090  -  feronia receptor-like kinase
## VIT_14s0081g00600  -  C-myc binding protein
## VIT_14s0060g00430  -  Plant IF (intermediate filament)
## VIT_13s0074g00110  -  Bark storage protein A precursor
## VIT_12s0059g01240  -  Nitrate transporter (NTP3)
## VIT_13s0064g00440  -  24-sterol C-methyltransferase
## VIT_01s0150g00250  -  Peptide transporter PTR2-B
## VIT_04s0008g01210  -  Delta-8 sphingolipid desaturase
## VIT_07s0104g01430  -  Phototropic-responsive NPH3
## VIT_06s0004g02890  -  Protein disulfide-isomerase A1
## VIT_06s0080g00390  -  Endomembrane protein 70 protein
## VIT_13s0320g00040  -  Glycosyl hydrolase family 20 protein
## VIT_01s0011g03610  -  MAPK activating protein
## VIT_18s0122g00780  -  Inorganic phosphate transporter
## VIT_09s0018g01240  -  Balbiani ring 1
## VIT_11s0065g00130  -  Taxane 10-beta-hydroxylase
## VIT_18s0001g12980  -  Cyclin-dependent protein kinase CYCT1;4
## VIT_08s0056g01520  -  Acetylglucosaminyltransferase
## VIT_18s0001g10170  -  PAB8 (poly(A) binding protein 8)
## VIT_12s0028g02200  -  Riboflavin kinase
## VIT_12s0034g01760  -  Unknown protein
## VIT_13s0067g02270  -  CTV.22
## VIT_01s0011g00370  -  SP1L2 (SPIRAL2)
## VIT_19s0015g01070  -  Calcineurin B (VvCBL02)
## VIT_05s0102g00120  -  Glutathione peroxidase
## VIT_10s0003g04390  -  ABC Transporter (VvMRP17 - VvABCC17)
```

```
## VIT_11s0037g01410  -  XBAT32 (XB3 ortholog 2 in Arabidopsis thaliana 32)
## VIT_08s0040g03070  -  WRKY Transcription Factor (VvWRKY26) - (VvPH3)
## VIT_18s0001g00170  -  Unknown protein
## VIT_11s0016g01280  -  DNA-directed RNA polymerase III subunit C4
## VIT_06s0061g01230  -  Cellulose synthase CSLA02
## VIT_16s0098g00870  -  Transparent Testa Glabra 1 (VvWD1)
## VIT_18s0001g10520  -  Sterol carrier protein 2
```

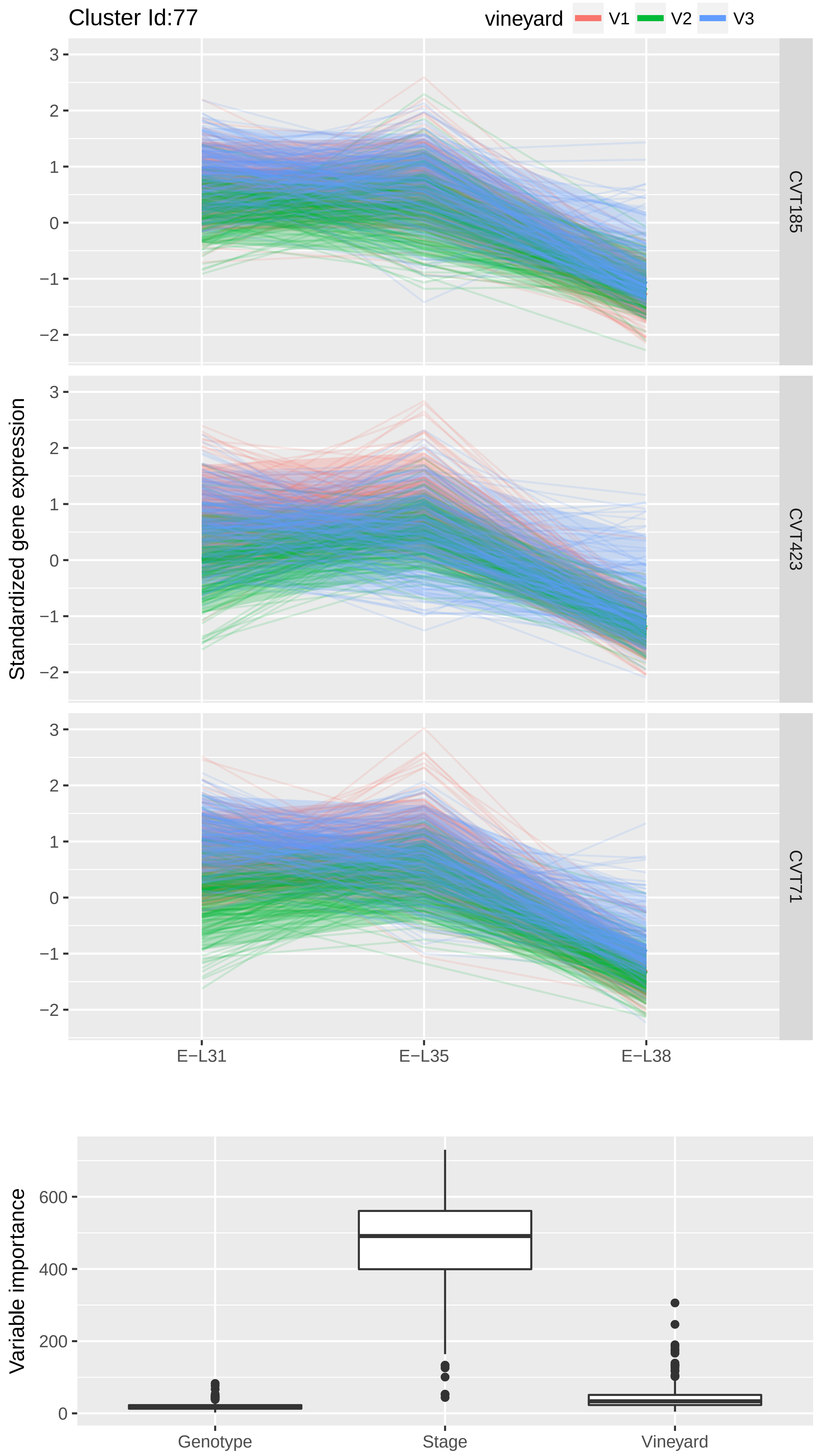

Cluster Id:77
vineyard
V1
V2
V3
Standardized gene expression
CVT185
CVT423
CVT71
E−L31
E−L35
E−L38
Variable importance
Genotype
Stage
Vineyard

Cluster no. 78

```
## Number of genes in the cluster: 237
## Homogeneity Index:    0.76
## Variable importance for Stage:    Median =  562.9  - Rank =  22
## Variable importance for Clone:    Median =  13.56  - Rank =  79
## Variable importance for Vineyard: Median =  17.73  - Rank =  90
##
## Gene ID               Gene Annotation
## VIT_14s0066g00700  -  Oleosin H-isoform
## VIT_06s0004g08100  -  VP1/ABI3 family regulatory protein
## VIT_10s0116g01720  -  Unknown protein
## VIT_12s0059g00600  -  Unknown protein
## VIT_01s0026g02460  -  Zinc finger homeobox 30
## VIT_03s0088g00240  -  Replication protein A 70b
## VIT_18s0001g07790  -  Unknown
## VIT_01s0011g04900  -  Serine carboxypeptidase-like 50 SCPL50 (
## VIT_03s0038g02500  -  SKP1
## VIT_11s0016g02020  -  EMB1789 (embryo defective 1789)
## VIT_04s0044g00860  -  Protein BRUSHY 1 (Protein TONSOKU) (Protein MGOUN 3)
## VIT_08s0040g01560  -  RNA polymerase primary sigma factor SIGF
## VIT_05s0124g00540  -  EMB1674
## VIT_13s0019g00040  -  Beta-N-acetylhexosaminidase
## VIT_08s0040g02840  -  ABC Transporter (VvWBC21 - VvABCG21)
## VIT_15s0048g02750  -  Unknown
## VIT_16s0098g01400  -  ISI1 (impaired sucrose induction 1)
## VIT_03s0038g02530  -  Pre-mRNA-splicing factor SYF2
## VIT_13s0139g00310  -  DNA-directed RNA polymerase II subunit E
## VIT_03s0132g00230  -  Aldehyde Dehydrogenase (VvALDH6B5)
## VIT_13s0074g00310  -  Translation initiation factor eIF-2 gamma subunit
## VIT_12s0028g03480  -  Wax synthase
## VIT_18s0001g14140  -  No hit
## VIT_16s0100g00010  -  Carboxyesterase 20 CXE20
## VIT_06s0009g01920  -  No hit
## VIT_17s0000g04200  -  Transcription initiation factor TFIID subunit D2
## VIT_10s0116g00680  -  PDF2 (protodermal factor2)
## VIT_04s0023g02680  -  Dimethyladenosine transferase
## VIT_01s0011g03540  -  Lateral organ boundaries protein 41
## VIT_15s0048g02120  -  Myb domain protein 3R2
## VIT_18s0076g00340  -  CYP707A3
## VIT_18s0001g01880  -  Pentatricopeptide (PPR) repeat
## VIT_00s0214g00090  -  F-box protein PP2-B10 (Protein phloem protein 2-like B10)
## VIT_16s0098g01790  -  Calmodulin-binding region IQD21
## VIT_08s0007g06800  -  Chromomethylase
## VIT_01s0011g02040  -  Splicing factor 3A subunit 1
## VIT_14s0081g00150  -  Ankyrin
## VIT_12s0134g00280  -  S-locus lectin protein kinase
## VIT_06s0009g02220  -  CRR2 (chlororespiratory reduction 2)
## VIT_18s0122g01320  -  Catalase 3
## VIT_12s0028g03870  -  Pentatricopeptide (PPR) repeat-containing protein
## VIT_08s0007g04770  -  Zinc finger (C2H2 type) family
## VIT_01s0011g04380  -  Acyl-(acyl carrier protein) thioesterase
## VIT_18s0001g09240  -  No hit
## VIT_07s0130g00380  -  DNA (cytosine-5)-methyltransferase AthI
## VIT_01s0127g00300  -  CRS2-associated factor 2, chloroplastic
## VIT_14s0060g00240  -  Myb domain protein 61
```

```
## VIT_15s0046g03360  -  Unknown protein
## VIT_03s0091g01030  -  Telomere repeat binding factor 1
## VIT_05s0020g00980  -  No hit
## VIT_03s0038g01960  -  Proline-rich family protein
## VIT_14s0060g02100  -  Pentatricopeptide (PPR) repeat-containing
## VIT_15s0048g00830  -  LOB domain-containing 18 (Asymmetric leaves 2-like protein 20)
## VIT_07s0205g00060  -  Dihydrouridine synthase 2
## VIT_01s0244g00160  -  Unknown protein
## VIT_14s0066g01450  -  MBD9 (methyl-CPG-binding Domain 9)
## VIT_18s0001g04580  -  Unknown protein
## VIT_10s0003g05690  -  Ribulose bisphosphate carboxylase, large chain
## VIT_18s0001g05550  -  Splicing factor, arginine/serine-rich 2
## VIT_11s0149g00070  -  Ribosomal protein S6
## VIT_06s0004g08330  -  No hit
## VIT_09s0002g01700  -  Epidermal patterning factor-like protein 3
## VIT_10s0071g00060  -  No hit
## VIT_09s0002g01500  -  Zinc finger (C3HC4-type ring finger) family protein
## VIT_02s0025g04830  -  Copper chaperone for superoxide dismutase
## VIT_12s0035g01050  -  Octicosapeptide/Phox/Bem1p (PB1) domain-containing protein
## VIT_15s0046g03650  -  (+)-neomenthol dehydrogenase
## VIT_03s0063g02170  -  No hit
## VIT_01s0150g00400  -  Calnexin 1 (CNX1)
## VIT_06s0004g01330  -  Pentatricopeptide (PPR) repeat-containing protein
## VIT_10s0003g00430  -  Unknown protein
## VIT_13s0067g02530  -  Unknown protein
## VIT_18s0001g08990  -  No hit
## VIT_07s0005g04480  -  Tyrosine decarboxylase
## VIT_17s0000g06430  -  Peptidyl-prolyl cis-trans isomerase NIMA-interacting 4
## VIT_09s0002g07010  -  SNF4
## VIT_12s0028g03940  -  Transcription initiation factor TFIID subunit 1-A
## VIT_01s0011g02110  -  Unknown
## VIT_17s0000g09770  -  Cysteine proteinase
## VIT_14s0171g00100  -  Blue (type 1) copper domain
## VIT_04s0008g01780  -  Transposon protein, putative, CACTA
## VIT_06s0004g03670  -  Protein kinase APK1B, chloroplast precursor
## VIT_03s0091g00770  -  No hit
## VIT_16s0098g01860  -  ClpX, ATPase regulatory subunit
## VIT_10s0003g01440  -  Transcriptional regulator family
## VIT_01s0127g00410  -  CRS2-associated factor 2
## VIT_07s0031g00600  -  RNA recognition motif (RRM)-containing protein
## VIT_07s0005g01490  -  Polycomb group protein EZA1 (SWINGER)
## VIT_01s0010g02040  -  Hydroxyproline-rich glycoprotein
## VIT_19s0014g00410  -  PMR5 (powdery mildew resistant 5)
## VIT_00s0233g00030  -  Trehalose-6-phosphate phosphatase
## VIT_00s0301g00100  -  BFN1 (bifunctional nuclease I)
## VIT_14s0030g01830  -  Protein kinase PKN/PRK1, effector
## VIT_01s0011g04370  -  Phosphatidylserine synthase 2
## VIT_19s0014g00360  -  Root initiation defective 3 RID3
## VIT_07s0005g00110  -  Rapid ALkalinization Factor RALFL11
## VIT_08s0007g01810  -  ANTR2 (anion transporter 2
## VIT_08s0007g03330  -  Iron-sulfur assembly protein IscA 2
## VIT_08s0040g02420  -  Ribosomal protein S23 (RPS23B) 40S
## VIT_18s0122g01400  -  Exostosin family protein
## VIT_14s0128g00180  -  Zinc finger (C3HC4-type ring finger)
## VIT_00s0207g00020  -  Unknown protein
```

```
## VIT_05s0049g02360  -  DEAD/DEAH box helicase (RH10)
## VIT_06s0004g00130  -  Pyruvate kinase
## VIT_05s0020g01120  -  Rhomboid
## VIT_18s0001g06070  -  Unknown protein
## VIT_05s0077g01300  -  Aldo-keto reductase
## VIT_02s0154g00110  -  Trehalose-6-phosphate phosphatase (AtTPPA)
## VIT_07s0005g00280  -  Zinc finger (c3hc4-type ring finger) nitrogen limitation adaptation
## VIT_03s0038g01460  -  Chalcone synthase
## VIT_08s0007g02910  -  Gibberellin signal transduction protein (SPINDLY)
## VIT_08s0040g02400  -  Myosin heavy chain
## VIT_07s0005g02740  -  Peptide chain release factor
## VIT_06s0009g01770  -  Lipase GDSL
## VIT_01s0011g04670  -  ABC Transporter (VvWBC7 - VvABCG7)
## VIT_18s0089g00350  -  Dynamin-like protein 6
## VIT_01s0011g02050  -  No hit
## VIT_06s0004g00960  -  Unknown
## VIT_13s0019g04370  -  Phosphoglucomutase/phosphomannomutase
## VIT_04s0023g02490  -  Pentatricopeptide (PPR) repeat-containing
## VIT_07s0031g00470  -  DNA polymerase alpha
## VIT_14s0060g02120  -  Rad9
## VIT_18s0089g00360  -  Eukaryotic translation initiation factor SUI1
## VIT_19s0015g02880  -  Glutathione S-transferase 20 GSTU20
## VIT_03s0091g00290  -  Pathogenesis-related homeodomain protein (PRHA)
## VIT_18s0041g02120  -  R protein L6
## VIT_04s0008g06680  -  Unknown protein
## VIT_18s0001g08910  -  No hit
## VIT_17s0000g04860  -  Unknown
## VIT_18s0001g14190  -  Unknown
## VIT_16s0039g00490  -  Unknown protein
## VIT_05s0077g00700  -  ATP-binding
## VIT_06s0004g01140  -  GRIK1 (geminivirus rep  interacting  kinase 1)
## VIT_07s0104g01580  -  Luminidependens
## VIT_01s0011g01130  -  EMB1025
## VIT_09s0002g05710  -  ATP:ADP antiporter
## VIT_18s0089g01110  -  Nuclear division RFT1-like protein
## VIT_08s0007g08930  -  Unknown protein
## VIT_02s0025g04290  -  Thaumatin
## VIT_00s1916g00010  -  DNA binding
## VIT_07s0005g01650  -  Nodal modulator 1 precursor
## VIT_18s0001g14340  -  No hit
## VIT_16s0098g00680  -  DNA mismatch repair protein MutS2
## VIT_15s0046g01110  -  Histone H3.2
## VIT_18s0041g00100  -  Unknown protein
## VIT_05s0020g03200  -  Spermine synthase
## VIT_01s0127g00320  -  CRS2-associated factor 2
## VIT_15s0048g01780  -  Peptidyl-prolyl cis-trans isomerase-like 3
## VIT_18s0117g00160  -  Pentatricopeptide (PPR) repeat-containing protein
## VIT_13s0019g00800  -  Histone H4
## VIT_18s0001g09630  -  No hit
## VIT_06s0061g01520  -  Beta-fructofuranosidase
## VIT_01s0137g00670  -  Quinolinate synthase
## VIT_02s0025g00830  -  F-box family protein
## VIT_01s0137g00640  -  DEAH (Asp-Glu-Ala-His) box polypeptide 33
## VIT_11s0016g03060  -  No hit
```

```
## VIT_01s0137g00070  -  B-keto acyl reductase
## VIT_03s0091g00400  -  Transducin family protein / WD-40 repeat
## VIT_15s0107g00240  -  Unknown protein
## VIT_08s0007g06470  -  VQ motif-containing protein
## VIT_05s0062g01220  -  Prolyl-4 hydroxylase
## VIT_13s0064g00400  -  Ppr2
## VIT_15s0046g01810  -  S-locus lectin protein kinase
## VIT_12s0142g00110  -  Splicing factor, arginine/serine-rich 2
## VIT_06s0004g05500  -  Chlororespiratory reduction 2 (CRR2)
## VIT_05s0020g04950  -  CwfJ
## VIT_19s0014g01640  -  Malate dehydrogenase
## VIT_08s0056g01550  -  Unknown
## VIT_11s0103g00310  -  Unknown protein
## VIT_08s0007g08700  -  DTW domain-containing protein
## VIT_08s0007g01730  -  Ataxin-3
## VIT_11s0016g03480  -  Xyloglucan endo-transglycosylase, C-terminal
## VIT_18s0001g09640  -  No hit
## VIT_18s0089g00220  -  RNA recognition motif (RRM)-containing protein
## VIT_14s0006g00780  -  No hit
## VIT_12s0035g00830  -  Group II intron splicing factor CRS1
## VIT_08s0058g01150  -  Unknown protein
## VIT_12s0035g00260  -  Mlo4
## VIT_16s0098g01230  -  Cytochrome b5
## VIT_07s0104g01040  -  Unknown protein
## VIT_09s0002g01530  -  Unknown protein
## VIT_13s0074g00470  -  OTU cysteine protease
## VIT_07s0205g00050  -  Dihydrouridine synthase 2
## VIT_11s0052g01130  -  CD2-binding protein-related
## VIT_19s0090g00120  -  Ubiquitin-conjugating enzyme E2 variant
## VIT_03s0091g00320  -  Ribosomal rna assembly protein mis3
## VIT_15s0048g00500  -  Pectinesterase family
## VIT_03s0038g02100  -  SAM-dependent methyltransferase
## VIT_03s0097g00460  -  Geraniol 10-hydroxylase
## VIT_06s0004g00560  -  Peptidyl-tRNA hydrolase
## VIT_08s0040g00140  -  Unknown protein
## VIT_12s0035g00840  -  Unknown protein
## VIT_07s0129g00270  -  Casein kinase II alpha chain
## VIT_07s0005g06720  -  PPR2
## VIT_14s0066g01880  -  Zf AN1 domain-containing stress-associated protein 12 (SAP12)
## VIT_07s0005g04590  -  Tetratricopeptide repeat domain 4
## VIT_12s0028g00110  -  Cyclin CycL1;1 RCY1
## VIT_19s0090g01200  -  Unknown protein
## VIT_02s0012g02220  -  Xyloglucan endotransglucosylase/hydrolase 30
## VIT_08s0040g00150  -  PQ-loop repeat / transmembrane
## VIT_10s0523g00020  -  Tyrosine specific protein phosphatase
## VIT_02s0012g01810  -  Heat shock transcription factor A1D
## VIT_06s0004g07790  -  Lateral organ boundaries Domain 15
## VIT_12s0028g01210  -  DNA-directed RNA Polymerase II subunit F
## VIT_01s0011g00210  -  Cytochrome b5 isoform Cb5-D
## VIT_00s2207g00010  -  UDP-N-acetylglucosamine pyrophosphorylase
## VIT_18s0001g07260  -  Chaperone protein dnaJ
## VIT_13s0019g03490  -  Zinc finger (CCCH-type) family protein
## VIT_15s0021g01500  -  RWD domain-containing protein
## VIT_19s0085g00030  -  Unknown protein
## VIT_15s0048g00890  -  Cornichon family
```

```
## VIT_18s0001g03470  -  Flavonol synthase
## VIT_02s0241g00110  -  Aminomethyltransferase
## VIT_12s0134g00380  -  S-locus lectin protein kinase family
## VIT_07s0005g03940  -  ATBARD1/BARD1 (breast cancer associated ring 1);
## VIT_06s0061g00760  -  Zinc finger (C2H2 type) family
## VIT_18s0001g09300  -  Protein Mpv17
## VIT_13s0019g03510  -  Zinc finger (CCCH-type) family protein
## VIT_09s0002g00880  -  Oligouridylate binding protein 1B UBP1B
## VIT_07s0005g02600  -  Ribosomal protein L7Ae
## VIT_15s0021g00450  -  Unknown protein
## VIT_16s0050g02300  -  Unknown
## VIT_11s0118g00320  -  Transposase
## VIT_12s0142g00430  -  Alternative oxidase 2, (AOX2)
## VIT_07s0005g03810  -  Unknown protein
## VIT_04s0023g00750  -  KRR1 family protein
## VIT_06s0004g01640  -  UDP-glucoronosyl and UDP-glucosyl transferase
## VIT_14s0108g01060  -  Pentatricopeptide (PPR) repeat-containing protein
## VIT_04s0044g02050  -  Protein serine/threonine phosphatase
## VIT_17s0000g01910  -  Glutamine synthetase cytosolic isozyme 1
## VIT_01s0127g00670  -  Proline extensin-like receptor kinase 1 (PERK1)
## VIT_06s0004g07340  -  Nucleotide binding
## VIT_12s0057g00250  -  NDB3 (alternative NAD(P)H dehydrogenase 32)
## VIT_11s0016g05620  -  TPR4/WSIP2 (topless-related 4)
## VIT_05s0062g00140  -  PRD1
## VIT_16s0039g02240  -  ADP-ribosylation factor B1C
## VIT_01s0011g01420  -  UDP-N-acetylglucosamine pyrophosphorylase
```

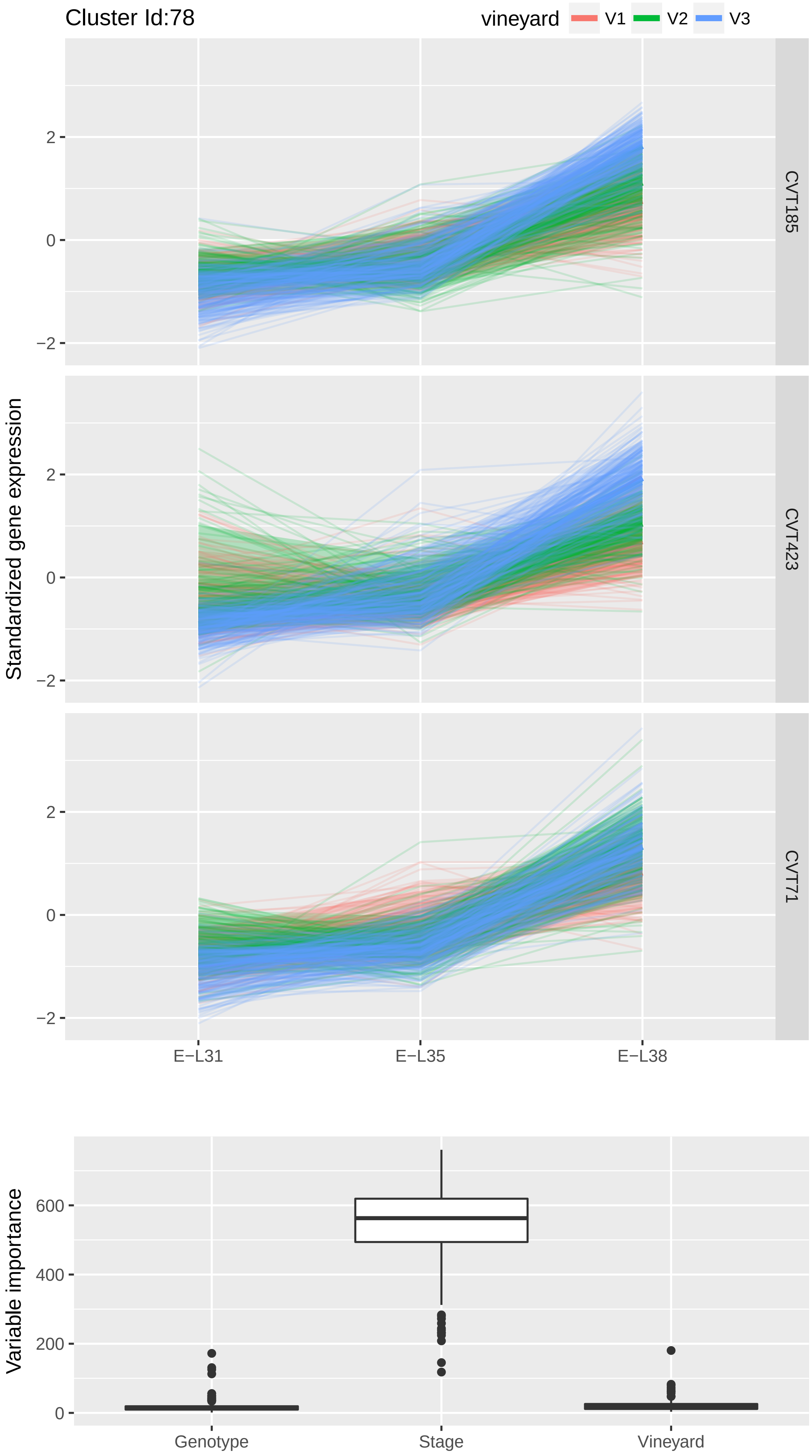

Cluster Id:78
vineyard
V1
V2
V3
Standardized gene expression
CVT185
CVT423
CVT71
E−L31
E−L35
E−L38
Variable importance
Genotype
Stage
Vineyard

Cluster no. 79

```
## Number of genes in the cluster: 115
## Homogeneity Index:    0.78
## Variable importance for Stage:    Median =  388.4  - Rank =  56
## Variable importance for Clone:    Median =  20.21  - Rank =  47
## Variable importance for Vineyard: Median =  37.46  - Rank =  53
##
## Gene ID               Gene Annotation
## VIT_04s0008g02480  -  Unknown protein
## VIT_01s0026g01660  -  Alpha-amylase isozyme C2 precursor
## VIT_14s0060g01630  -  Translation initiation factor eIF-3 subunit 10
## VIT_17s0000g05760  -  Nuclear protein ZAP
## VIT_15s0048g01040  -  DNA repair protein
## VIT_12s0034g00080  -  Flavonoid-glucosyltransferase
## VIT_10s0071g00310  -  F-box/LRR-repeat protein 2
## VIT_07s0005g00880  -  fumarate hydratase
## VIT_18s0001g03380  -  No hit
## VIT_16s0039g02640  -  AIN1 (ACC insensitive 1) 5'-3' exoribonuclease (XRN4)
## VIT_07s0129g01120  -  RNA exonuclease 1
## VIT_13s0073g00150  -  Heat shock protein-related
## VIT_16s0098g01680  -  Kinesin light chain
## VIT_15s0048g00860  -  Microtubule associated protein (MAP65/ASE1) pleiade
## VIT_07s0141g00140  -  Histone acetyltransferase GCN5
## VIT_01s0011g01970  -  Pentatricopeptide (PPR) repeat
## VIT_07s0005g04280  -  MRNA splicing protein (Prp39)
## VIT_19s0177g00310  -  Tryptophan synthase beta chain 1
## VIT_05s0102g00070  -  Unknown protein
## VIT_12s0028g02390  -  Unknown
## VIT_13s0067g01740  -  Receptor protein kinase
## VIT_10s0116g01770  -  Met-10+ like family protein
## VIT_00s0394g00020  -  Pentatricopeptide (PPR) repeat-containing protein
## VIT_07s0031g00010  -  Exonuclease
## VIT_16s0050g01690  -  Receptor kinase homolog LRK10
## VIT_19s0027g00460  -  Glutathione S-transferase 25 GSTU25
## VIT_04s0008g05860  -  Unknown
## VIT_19s0027g00370  -  CYP716A1
## VIT_14s0068g02200  -  Unknown protein
## VIT_12s0035g00990  -  Big apical meristem 1 BAM1
## VIT_11s0016g02370  -  Unknown protein
## VIT_14s0108g00070  -  Transcription initiation factor TFIID subunit D4
## VIT_14s0060g01330  -  Lipoamide dehydrogenase
## VIT_06s0009g02380  -  Indigoidine synthase A family protein
## VIT_17s0000g03440  -  EMB2454 (embryo defective 2454)
## VIT_17s0000g03380  -  Calmodulin binding protein
## VIT_13s0019g01650  -  Expansin  (VvEXPA13)
## VIT_16s0100g00740  -  Unknown
## VIT_12s0034g01120  -  UDP-glycosyltransferase 71A13
## VIT_11s0016g00030  -  Digalactosyldiacylglycerol synthase 2, chloroplastic
## VIT_07s0031g01540  -  4-alpha-glucanotransferase
## VIT_06s0004g02940  -  Zinc finger (CCCH-type) family protein
## VIT_00s0274g00050  -  Unknown protein
## VIT_11s0149g00020  -  Brix domain-containing protein
## VIT_01s0011g03980  -  Wall-associated kinase
## VIT_18s0076g00360  -  EMB1075 (embryo defective 1075) carboxy-lyase
## VIT_19s0027g01790  -  Acetylornithine aminotransferase
```

```
## VIT_14s0108g01170  -  Pectinesterase family
## VIT_00s0215g00020  -  Protease Do-like 10
## VIT_03s0038g04040  -  Aminoacylase-1
## VIT_18s0122g01210  -  Cuticular water permeability
## VIT_14s0081g00620  -  SAP1 protein
## VIT_05s0124g00250  -  Histone-lysine N-methyltransferase ATX1
## VIT_03s0063g02680  -  Radialis-like protein 5
## VIT_11s0065g01170  -  Unknown protein
## VIT_05s0020g03420  -  PHD finger transcription factor
## VIT_10s0003g02010  -  RKF1 (receptor-like kinase in flowers 1)
## VIT_03s0038g04120  -  Cellulose synthase CSLD4
## VIT_09s0002g04840  -  Unknown
## VIT_11s0037g00680  -  Leucine Rich Repeat receptor-like kinase
## VIT_10s0071g01200  -  Vacuolar pyrophosphatase [Vitis vinifera]
## VIT_03s0017g01440  -  Berberine bridge enzyme
## VIT_11s0149g00140  -  D-amino acid dehydrogenase
## VIT_04s0008g03530  -  Ankyrin repeat
## VIT_19s0027g00320  -  Unknown
## VIT_04s0008g06330  -  TPR1 (topless-related 1)
## VIT_08s0105g00330  -  Enhanced silencing phenotype 4 ESP4 symplekin
## VIT_00s0469g00020  -  Cellulose synthase CSLE1
## VIT_12s0028g00260  -  Phosphoribosylamine--glycine ligase (PUR2)
## VIT_07s0005g01340  -  Ribosomal protein L29e
## VIT_03s0063g02530  -  Unknown protein
## VIT_09s0002g02560  -  Ribosomal protein L28e
## VIT_18s0001g12680  -  Unknown protein
## VIT_14s0068g01790  -  Unknown
## VIT_10s0003g01880  -  RKF1 (receptor-like kinase in flowers 1)
## VIT_13s0067g01750  -  Receptor protein kinase
## VIT_10s0003g00470  -  Trans-resveratrol di-O-methyltransferase - VvROMT
## VIT_12s0028g01390  -  Small heat shock protein ACD31
## VIT_18s0166g00290  -  Protein kinase
## VIT_00s0207g00130  -  Pathogenesis-related protein 1 precursor (PRP 1)
## VIT_18s0089g01190  -  Stage III sporulation protein AA
## VIT_18s0001g13120  -  GTP-binding protein hflX
## VIT_03s0097g00490  -  Unknown protein
## VIT_01s0011g01870  -  Peptidase, trypsin serine and cysteine proteases
## VIT_16s0013g01520  -  U3 small nucleolar RNA-associated protein 10
## VIT_05s0020g04770  -  CRS1
## VIT_01s0150g00140  -  ORTH2/VIM1 (variant in methylation 1)
## VIT_03s0017g01430  -  Integral membrane Yip1 family protein
## VIT_03s0038g01320  -  Crp1 protein
## VIT_03s0017g01040  -  Cis-zeatin O-beta-D-glucosyltransferase
## VIT_10s0116g00900  -  Unknown protein
## VIT_15s0046g02930  -  DNA-binding protein
## VIT_18s0001g01110  -  Monooxygenase (MO2)
## VIT_08s0056g00030  -  Diadenosine 5',5'''-P1,P4-tetraphosphate hydrolase
## VIT_07s0197g00080  -  SNF1 protein kinase 2-3 AKIP OST1
## VIT_10s0003g02020  -  RKF1 (receptor-like kinase in flowers 1)
## VIT_05s0051g00830  -  Dihydroxy-acid dehydratase
## VIT_04s0044g00060  -  RWP-RK domain-containing protein
## VIT_03s0132g00340  -  Wall-associated receptor kinase-like 10
## VIT_00s0753g00020  -  Histone H2A.4 HTA12
## VIT_01s0011g02840  -  Cytochrome B561
## VIT_01s0011g05270  -  Protein BCCIP homolog
```

```
## VIT_08s0007g02780  -  Asp/Glu racemase
## VIT_06s0061g00700  -  Auxin-regulated protein
## VIT_08s0007g08900  -  Cis-zeatin O-beta-D-glucosyltransferase
## VIT_11s0065g01070  -  Unknown
## VIT_13s0067g03390  -  Pseudo-response regulator 7 (APRR7)
## VIT_19s0085g01020  -  EMB1135
## VIT_01s0127g00710  -  Mov34 STAM-binding protein
## VIT_05s0094g01650  -  Unknown
## VIT_00s0207g00160  -  Pathogenesis-related protein 1 precursor (PRP 1)
## VIT_10s0042g00160  -  Unknown
## VIT_04s0008g02370  -  Pentatricopeptide (PPR) repeat-containing protein
## VIT_05s0020g04400  -  No hit
## VIT_00s0371g00100  -  Mannitol dehydrogenase
```

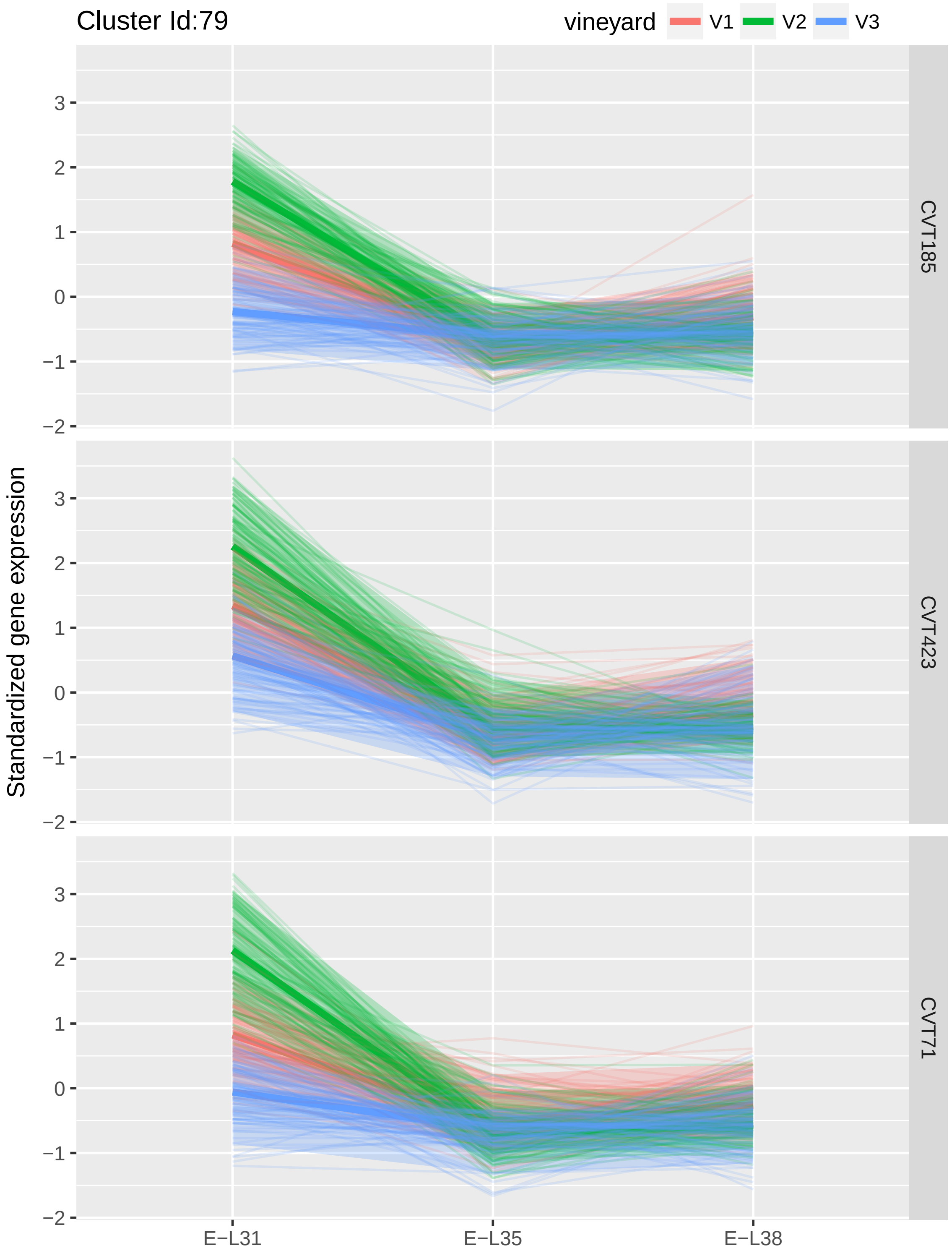

Cluster Id:79
vineyard
V1
V2
V3
Standardized gene expression
CVT185
CVT423
CVT71
3
2
1
0
−1
−2
E−L31
E−L35
E−L38

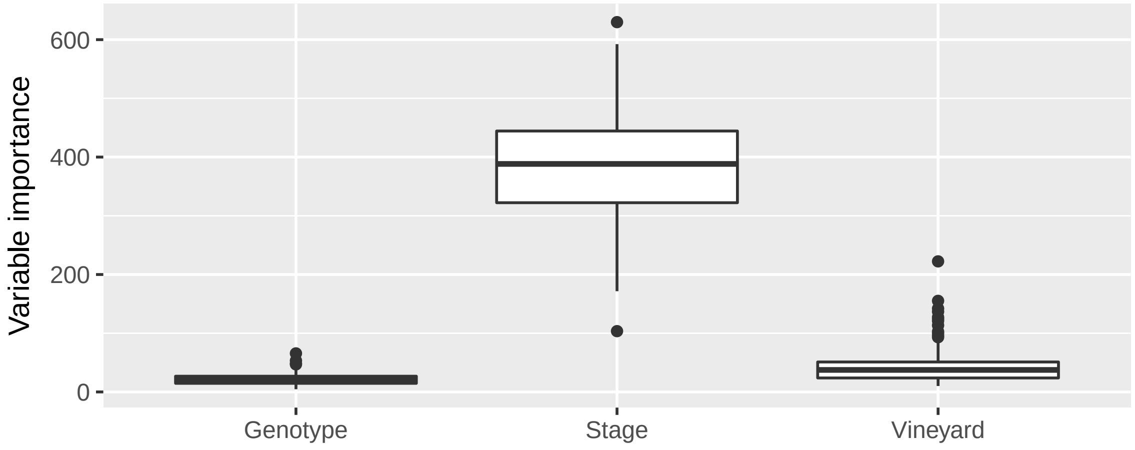

Variable importance
600
400
200
0
Genotype
Stage
Vineyard

Cluster no. 80

```
## Number of genes in the cluster: 95
## Homogeneity Index:    0.72
## Variable importance for Stage:    Median =  475.4  - Rank =  42
## Variable importance for Clone:    Median =  19.38  - Rank =  54
## Variable importance for Vineyard: Median =  37.99  - Rank =  52
##
## Gene ID               Gene Annotation
## VIT_08s0058g00670  -  Unknown protein
## VIT_06s0004g04030  -  Copper chaperone
## VIT_11s0016g00910  -  Cation exchanger (CAX9)
## VIT_02s0012g02190  -  Cellulose synthase CSLD2
## VIT_07s0129g00430  -  Acyl-CoA binding protein
## VIT_11s0016g04700  -  Beta-ketoacyl-CoA synthase
## VIT_00s0207g00190  -  Pentatricopeptide (PPR) repeat-containing protein
## VIT_18s0001g06390  -  Auxin-independent growth promoter
## VIT_15s0046g01530  -  Intracellular protein transport protein USO1
## VIT_01s0011g05030  -  Unknown protein
## VIT_11s0016g05740  -  Calmodulin-5 (CAM5)
## VIT_11s0149g00300  -  Chitinase, class V
## VIT_08s0007g00770  -  NDP1 (random potato CDNA clone);
## VIT_01s0244g00010  -  basic helix-loop-helix (bHLH) family (VvCEB1)
## VIT_04s0008g06720  -  Protein phosphatase 2C
## VIT_04s0044g00490  -  DnaJ homolog, subfamily B, member 4
## VIT_11s0118g00210  -  Nodulation receptor kinase
## VIT_02s0025g03090  -  Unknown protein
## VIT_10s0003g01460  -  Nucleobase-ascorbate transporter 2 (NAT2)
## VIT_00s0340g00060  -  Endo-1,4-beta-glucanase korrigan (KOR)
## VIT_16s0098g00540  -  Golgin candidate 5
## VIT_10s0071g00550  -  GRAM domain-containing protein / ABA-responsive
## VIT_00s2526g00010  -  Endo-1,4-beta-glucanase korrigan (KOR)
## VIT_14s0128g00160  -  Protein kinase CDG1
## VIT_19s0014g05040  -  formin homology 2 domain-containing protein 5
## VIT_14s0219g00080  -  Unknown protein
## VIT_14s0066g02190  -  CW14
## VIT_12s0028g03900  -  No hit
## VIT_00s0340g00050  -  Endo-1,4-beta-glucanase korrigan (KOR)
## VIT_19s0090g01120  -  Unknown protein
## VIT_08s0007g08300  -  Calcium-dependent protein kinase (VvCPK11)
## VIT_18s0001g06830  -  Methyltransferase type 11
## VIT_10s0003g03190  -  RNA recognition motif (RRM)-containing
## VIT_01s0244g00150  -  Auxin response factor 2
## VIT_13s0019g03400  -  Signal peptidase complex subunit 2
## VIT_18s0122g01020  -  Unknown protein
## VIT_01s0011g05000  -  Auxin-independent growth promoter
## VIT_01s0137g00690  -  Translation initiation factor eIF-4B
## VIT_08s0056g00430  -  Unknown protein
## VIT_03s0063g00160  -  Zinc finger (C3HC4-type ring finger)
## VIT_18s0072g00780  -  Ubiquitin interaction motif-containing protein
## VIT_04s0023g01800  -  Maf, septum formation protein
## VIT_03s0038g02890  -  F-box family protein
## VIT_11s0016g03760  -  Unknown protein
## VIT_05s0062g00480  -  Xyloglucan endo-transglycosylase, C-terminal
## VIT_08s0040g01820  -  No hit
## VIT_13s0067g00960  -  Ser/Thr protein kinase
```

```
## VIT_14s0068g01360  -  GEM-like protein 5
## VIT_19s0090g01650  -  LOP1 TRN1 (LOPPED 1, TORNADO 1)
## VIT_17s0000g02150  -  Ribosomal protein L19
## VIT_14s0030g00830  -  SOD Cu/Zn superoxide dismutase
## VIT_10s0003g04070  -  Metallo-beta-lactamase
## VIT_03s0063g02090  -  Unknown protein
## VIT_00s0434g00060  -  Unknown
## VIT_01s0010g03390  -  Gag-pol polyprotein
## VIT_07s0005g05160  -  Proteasome 26S AAA-ATPase subunit (RPT2)
## VIT_04s0023g03320  -  WD repeat-protein
## VIT_02s0012g00150  -  NAK-type protein kinase
## VIT_01s0011g01660  -  VAMP726 (VESICLE-associated membrane protein)
## VIT_10s0071g00300  -  Unknown protein
## VIT_01s0011g05110  -  Major latex protein 22
## VIT_08s0007g08840  -  Glycosyl transferaseHGA1
## VIT_07s0104g01350  -  Integral membrane family protein UPF0497
## VIT_15s0046g03680  -  Unknown protein
## VIT_08s0040g00470  -  Calmodulin-7 (CAM7)
## VIT_04s0210g00170  -  Squamosa promoter-binding protein (VvSBP4)
## VIT_16s0098g00800  -  Copper-binding family protein
## VIT_14s0066g01610  -  Zinc finger (C3HC4-type ring finger)
## VIT_19s0090g00960  -  Unknown
## VIT_12s0059g00590  -  Allergenic protein Pt2L4
## VIT_08s0007g06260  -  D111/G-patch domain-containing protein
## VIT_05s0051g00870  -  Glutathione reductase
## VIT_05s0102g00440  -  FAR1-related sequence 5
## VIT_17s0000g04520  -  Cell growth defect factor 1
## VIT_04s0008g03690  -  Peroxin-4
## VIT_14s0066g01340  -  DNA-binding protein
## VIT_11s0052g01630  -  Flavonoid 3-O-glucosyltransferase
## VIT_13s0067g01960  -  Dehydration Responsive Element-Binding Transcription Factor (VvDREB19)
## VIT_04s0023g00130  -  Unknown protein
## VIT_00s2082g00010  -  O-sialoglycoprotein endopeptidase
## VIT_07s0005g01250  -  Unknown protein
## VIT_16s0050g01850  -  AAA-type ATPase
## VIT_02s0025g01670  -  Unknown protein
## VIT_17s0000g02280  -  Suppressor of lin-12 protein
## VIT_18s0001g04150  -  Avr9 elicitor response protein
## VIT_08s0007g02340  -  Exocyst subunit EXO70 A2
## VIT_16s0039g00820  -  CYP89A5
## VIT_17s0000g02800  -  Hydroxymethylglutaryl coenzyme A synthase
## VIT_07s0005g00070  -  5,10-methylenetetrahydrofolate dehydrogenase-5
## VIT_06s0004g04490  -  Unknown protein
## VIT_18s0072g00810  -  Ubiquinol-cytochrome c reductase subunit 6
## VIT_14s0066g01930  -  Unknown
## VIT_00s0265g00020  -  TRNA synthetase class I (C)
## VIT_17s0000g00410  -  No hit
## VIT_07s0031g00030  -  Endoplasmatic reticulum retrieval protein 1A
```

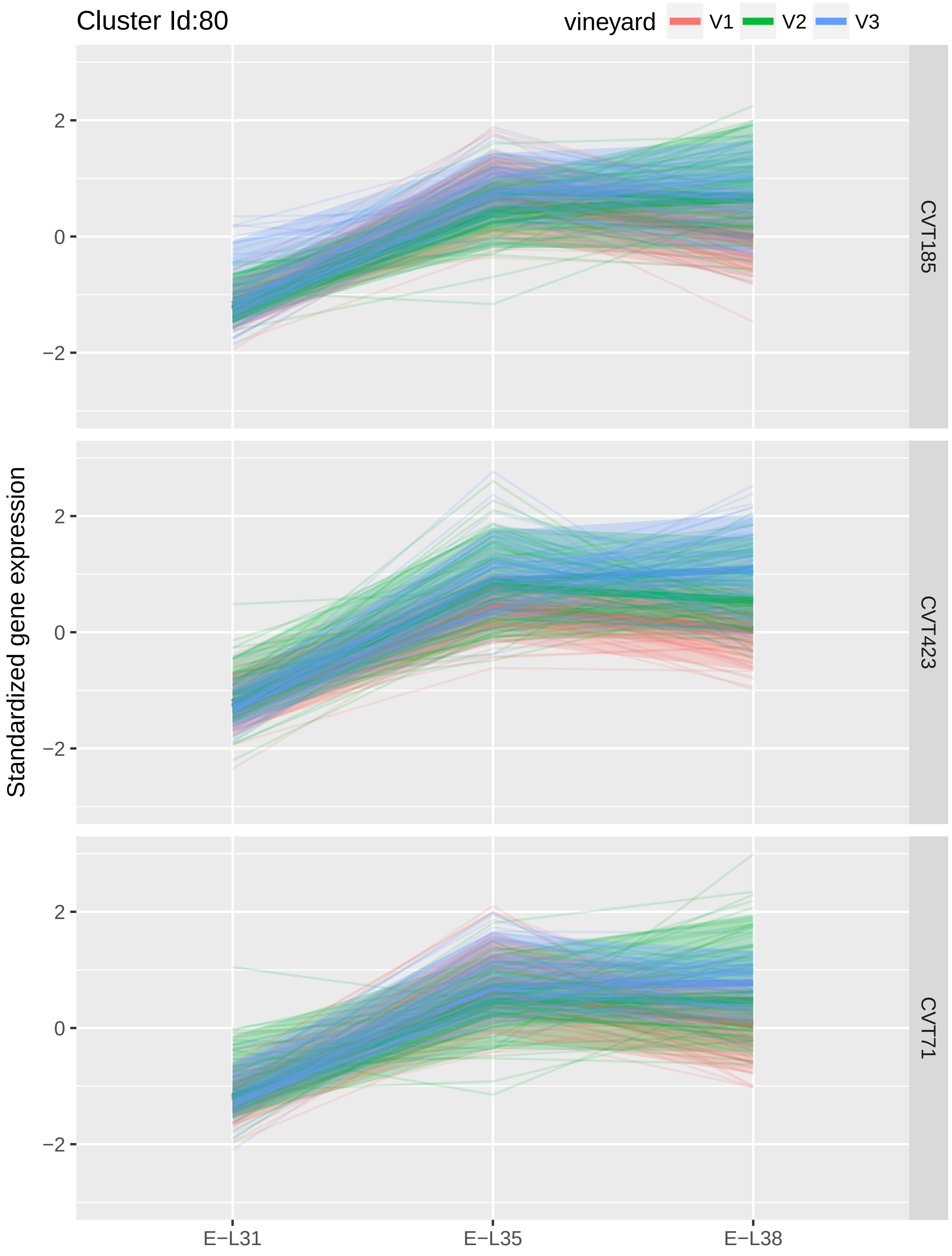

Cluster Id:80
vineyard
V1
V2
V3
Standardized gene expression
CVT185
CVT423
CVT71
2
0
−2
E−L31
E−L35
E−L38

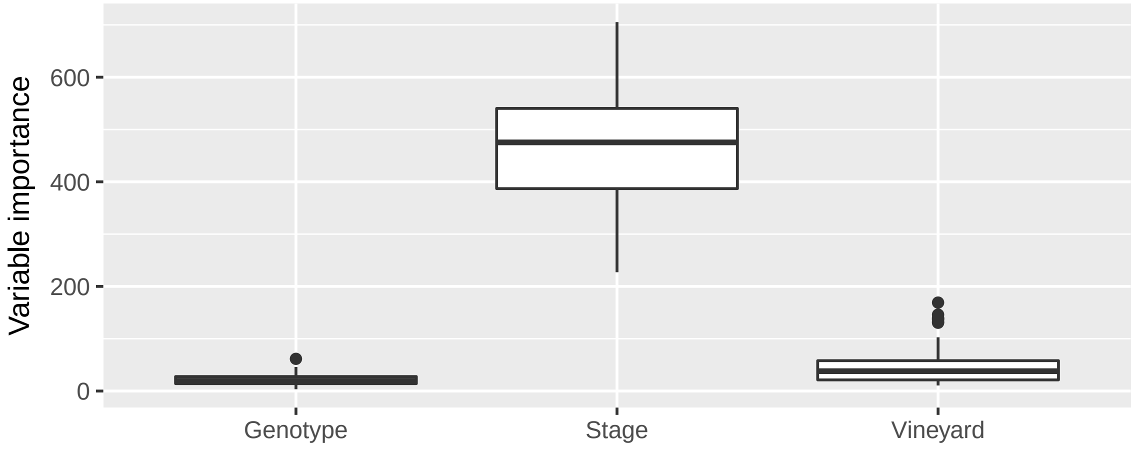

Variable importance
600
400
200
0
Genotype
Stage
Vineyard

Cluster no. 81

```
## Number of genes in the cluster: 30
## Homogeneity Index:    0.54
## Variable importance for Stage:    Median =  194.4  - Rank =  90
## Variable importance for Clone:    Median =  35.07  - Rank =  4
## Variable importance for Vineyard: Median =   52.9  - Rank =  38
##
## Gene ID               Gene Annotation
## VIT_19s0135g00200  -  CYP72A59
## VIT_13s0084g00160  -  Proline-rich family protein
## VIT_19s0135g00190  -  CYP72A59
## VIT_05s0094g00460  -  Eukaryotic translation initiation factor
## VIT_08s0007g03940  -  Histone deacetylase HD2A
## VIT_19s0014g01940  -  Unknown
## VIT_05s0094g00480  -  Ethylene-responsive protein
## VIT_01s0011g04850  -  Unknown protein
## VIT_06s0009g01940  -  GCN5 N-acetyltransferase (GNAT)
## VIT_00s0362g00010  -  DnaJ homolog, subfamily A, member 2
## VIT_14s0108g01200  -  Vesicle transport v-SNARE 13
## VIT_16s0039g01390  -  TRNA-Ala synthetase, cytosolic
## VIT_08s0040g02930  -  Unknown
## VIT_05s0020g02870  -  DEAD/DEAH box helicase
## VIT_13s0073g00330  -  VPS60.1 SNF7
## VIT_18s0089g00870  -  VAMP72A1
## VIT_17s0000g02500  -  RAB GTPase RABA6B
## VIT_04s0159g00020  -  RNA recognition motif (RRM)-containing CCCH
## VIT_09s0018g01180  -  TTL (transthyretin-like protein)
## VIT_10s0003g04260  -  Unknown protein
## VIT_19s0014g00030  -  FRIGIDA protein
## VIT_06s0004g05370  -  Tropinone reductase
## VIT_16s0098g00860  -  Flavanone 3-hydroxylase
## VIT_14s0066g00720  -  Transducin protein
## VIT_17s0000g08970  -  Proline-rich family protein
## VIT_00s0585g00030  -  BETA-1,2-N-acetylglucosaminyltransferase I
## VIT_00s0207g00220  -  Cold acclimation protein
## VIT_07s0151g00530  -  Unknown protein
## VIT_15s0046g00450  -  Phosphatidic acid Phosphatase
## VIT_19s0014g00060  -  FRIGIDA protein
```

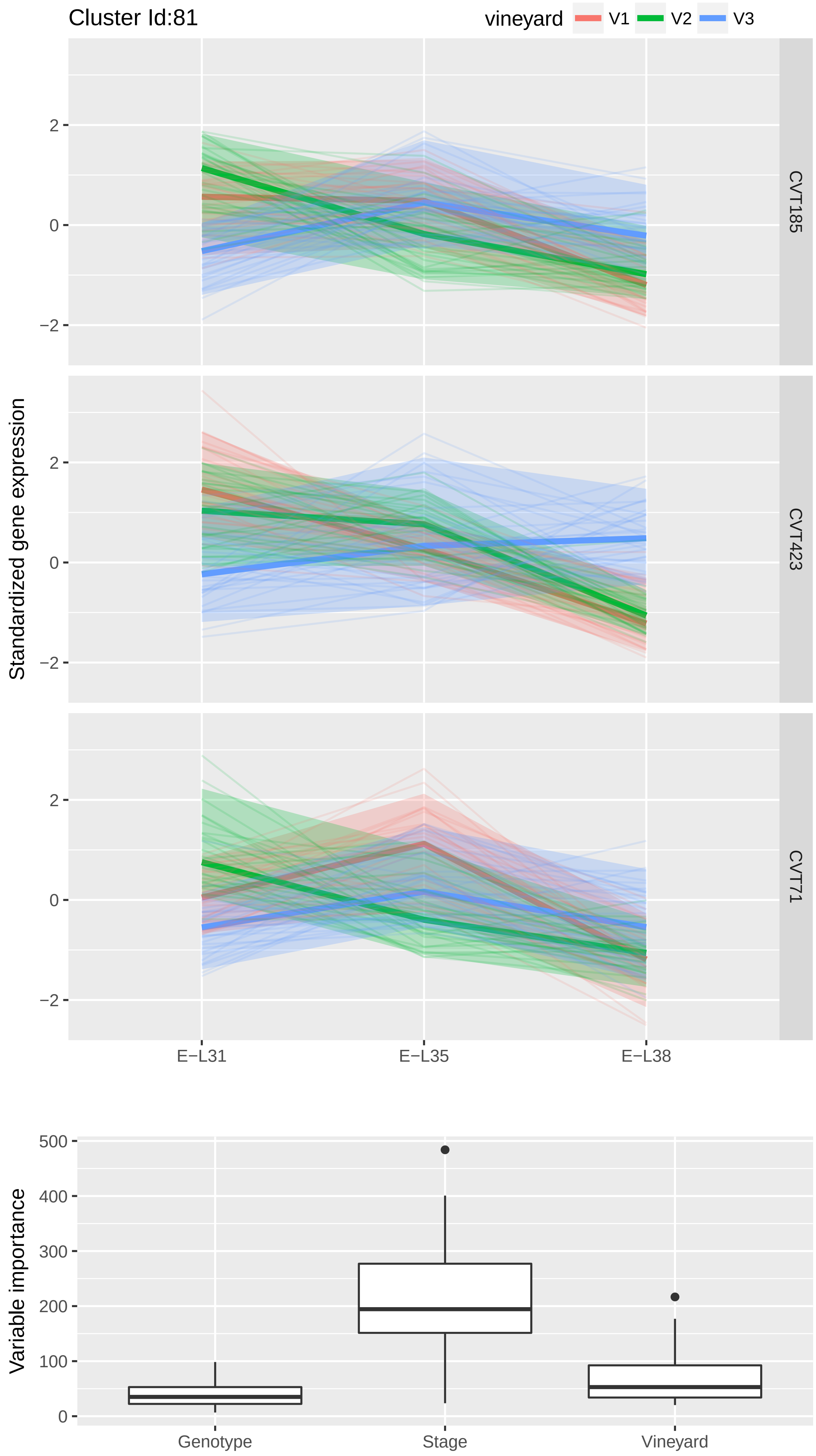

Cluster Id:81
vineyard V1 V2 V3
Standardized gene expression
CVT185
CVT423
CVT71
2
0
−2
E−L31
E−L35
E−L38
Variable importance
500
400
300
200
100
0
Genotype
Stage
Vineyard

Cluster no. 82

```
## Number of genes in the cluster: 108
## Homogeneity Index:    0.77
## Variable importance for Stage:    Median =    191  - Rank =  91
## Variable importance for Clone:    Median =  18.65  - Rank =  59
## Variable importance for Vineyard: Median =  98.28  - Rank =  16
##
## Gene ID                Gene Annotation
## VIT_06s0009g00690  -  NIMA-related kinase NEK6
## VIT_00s0691g00010  -  Calcium-transporting ATPase 13 ACA13
## VIT_09s0002g08640  -  Protein kinase Xa21
## VIT_00s0463g00030  -  Ca2+-transporting ATPase type 2 isoform 8
## VIT_01s0011g01860  -  No hit
## VIT_01s0011g00030  -  ACT domain containing protein (ACR4)
## VIT_18s0072g01020  -  Unknown protein
## VIT_04s0023g03570  -  Oligopeptide transporter 7 ATOPT7
## VIT_05s0020g02650  -  Unknown protein
## VIT_18s0117g00340  -  TCP family transcription factor TCP14
## VIT_00s0779g00010  -  Unknown protein
## VIT_01s0011g00710  -  Zinc finger (C2H2 type) family
## VIT_06s0080g00810  -  lycopene ß-cyclase (LBCY2) (VvLBCY1)
## VIT_06s0004g02060  -  Aldehyde Dehydrogenase (VvALDH3J1)
## VIT_13s0064g01510  -  Auxin-independent growth promoter
## VIT_11s0037g00040  -  basic helix-loop-helix (bHLH) family
## VIT_04s0023g02020  -  replication factor C subunit 3/5
## VIT_05s0029g00880  -  R protein L6
## VIT_18s0001g10700  -  Protein phosphatase 2C / PP2C
## VIT_12s0028g03590  -  Unknown protein
## VIT_16s0050g02310  -  No hit
## VIT_01s0011g00560  -  DREPP plasma membrane polypeptide
## VIT_13s0019g04390  -  GATA transcription factor 10
## VIT_08s0040g01920  -  Crinkly4
## VIT_02s0025g04520  -  Calcium-dependent protein kinase 1 CDPK protein kinase
## VIT_03s0038g02650  -  Protein phosphatase 2C FSPP2C2
## VIT_05s0029g00390  -  basic helix-loop-helix (bHLH) family
## VIT_12s0134g00300  -  S-locus lectin protein kinase family
## VIT_10s0003g05380  -  Cytochrome b5-like Fatty acid hydroxylase
## VIT_16s0050g00100  -  Myosin-related
## VIT_15s0045g00710  -  R protein disease resistance protein
## VIT_17s0000g05210  -  LNG1 (LONGIFOLIA1)
## VIT_00s0253g00040  -  Monocopper oxidase SKS17 (SKU5 Similar 17)
## VIT_19s0085g01110  -  Aspartic Protease (VvAP46)
## VIT_11s0052g00610  -  Homogenitisate geranylgeranyl transferase
## VIT_16s0039g02020  -  Cellulose synthase CSLD3
## VIT_07s0005g01290  -  DnaJ homolog, subfamily C, member 7
## VIT_09s0002g05190  -  No hit
## VIT_08s0040g00010  -  feronia receptor-like kinase
## VIT_15s0046g01680  -  Delta-8 sphingolipid desaturase
## VIT_01s0010g00020  -  DNA-3-methyladenine glycosidase I
## VIT_00s0945g00010  -  Unknown protein
## VIT_07s0031g01440  -  Receptor protein kinase
## VIT_02s0025g03220  -  Trihelix DNA-binding protein (GT2)
## VIT_02s0025g04120  -  Calmodulin binding protein
## VIT_14s0006g02870  -  Unknown
## VIT_16s0050g00780  -  Arsenite transport protein (ArsB)
```

```
## VIT_01s0011g04770  -  Armadillo/beta-catenin repeat / U-box domain-containing protein
## VIT_02s0025g01630  -  SOS3 (salt overly sensitive 3)
## VIT_18s0041g00660  -  Proton-dependent oligopeptide transport (POT) family protein
## VIT_08s0007g01520  -  TPR-containing protein kinase
## VIT_06s0004g02720  -  Pleckstriny (PH) domain-containing protein
## VIT_09s0002g04500  -  Carnitine/acylcarnitine carrier, Mitochondrial
## VIT_16s0013g00630  -  Cyclin-U4-1 (CycU4;1) CYCP4;1
## VIT_08s0040g01550  -  Extra sporogenous cells
## VIT_09s0054g01690  -  Transcription factor
## VIT_13s0019g03610  -  RNA recognition motif (RRM)-containing protein
## VIT_05s0020g03380  -  WNK1 (with no lysine (K) 1)
## VIT_04s0043g00160  -  Receptor protein kinase
## VIT_04s0023g00490  -  Auxin responsive SAUR protein
## VIT_14s0006g03100  -  No hit
## VIT_13s0019g02890  -  Glucosyltransferase-2
## VIT_02s0025g04130  -  Rhomboid ATRBL2
## VIT_06s0004g06820  -  Unknown protein
## VIT_02s0025g02410  -  Plastocyanin domain-containing protein
## VIT_04s0023g03170  -  GDP-D-mannose-4,6-dehydratase (MUR1)
## VIT_18s0117g00360  -  R protein L6
## VIT_10s0003g05480  -  SEC14 cytosolic factor
## VIT_12s0028g01660  -  No hit
## VIT_02s0012g00090  -  Phosphatidylinositol-4-phosphate 5-kinase
## VIT_14s0068g00510  -  Unknown
## VIT_00s0804g00020  -  Unknown protein
## VIT_15s0021g01280  -  Cyclin-U4-1 (CycU4;1) CYCP4;1
## VIT_06s0004g02530  -  Wall-associated receptor kinase 5
## VIT_03s0017g00680  -  UDP-d-apiose/udp-d-xylose synthase 1 AXS1
## VIT_03s0038g04580  -  SNF1-Related protein kinase regulatory subunit gamma 1
## VIT_08s0105g00380  -  Leucoanthocyanidin dioxygenase
## VIT_17s0000g06210  -  GASA like
## VIT_03s0038g03580  -  Lipase GDSL
## VIT_11s0016g04010  -  Unknown protein
## VIT_13s0019g04030  -  Serine carboxypeptidase SCPL27
## VIT_11s0016g03900  -  AAA-type ATPase
## VIT_04s0023g01320  -  Minor allergen Alt a 7
## VIT_04s0043g00560  -  DD1A protein
## VIT_19s0090g00360  -  Unknown
## VIT_19s0090g00510  -  Unknown protein
## VIT_17s0000g08990  -  Alliinase
## VIT_09s0002g00320  -  Pectinesterase PME3
## VIT_03s0091g01090  -  Unknown
## VIT_08s0007g00580  -  No hit
## VIT_04s0008g00700  -  No hit
## VIT_00s0207g00230  -  Phototropic-responsive NPH3
## VIT_08s0040g01540  -  Extra sporogenous cells
## VIT_11s0016g02110  -  FAD linked oxidase, N-terminal
## VIT_05s0102g00450  -  MSS3 (multicopy suppressors of snf4 deficiency in yeast 3)
## VIT_17s0000g07030  -  Cis-zeatin O-beta-D-glucosyltransferase
## VIT_10s0116g00520  -  Xyloglucan endotransglucosylase/hydrolase 8
## VIT_09s0002g04470  -  No hit
## VIT_19s0015g00710  -  Cellulose synthase CSLE1
## VIT_17s0000g07070  -  UDP-glucoronosyl and UDP-glucosyl transferase
## VIT_01s0150g00460  -  Xyloglucan endotransglucosylase/hydrolase precursor
```

```
## VIT_07s0129g00370  -  Unknown protein
## VIT_14s0066g01360  -  No hit
## VIT_05s0020g05050  -  Cellulose synthase CSLE1
## VIT_06s0061g01470  -  ABC Transporter (VvPDR22 - VvABCG52)
## VIT_15s0046g01360  -  No hit
## VIT_09s0002g05740  -  Alpha/beta hydrolase fold.
## VIT_11s0016g04240  -  Vernalization insensitive 3
```

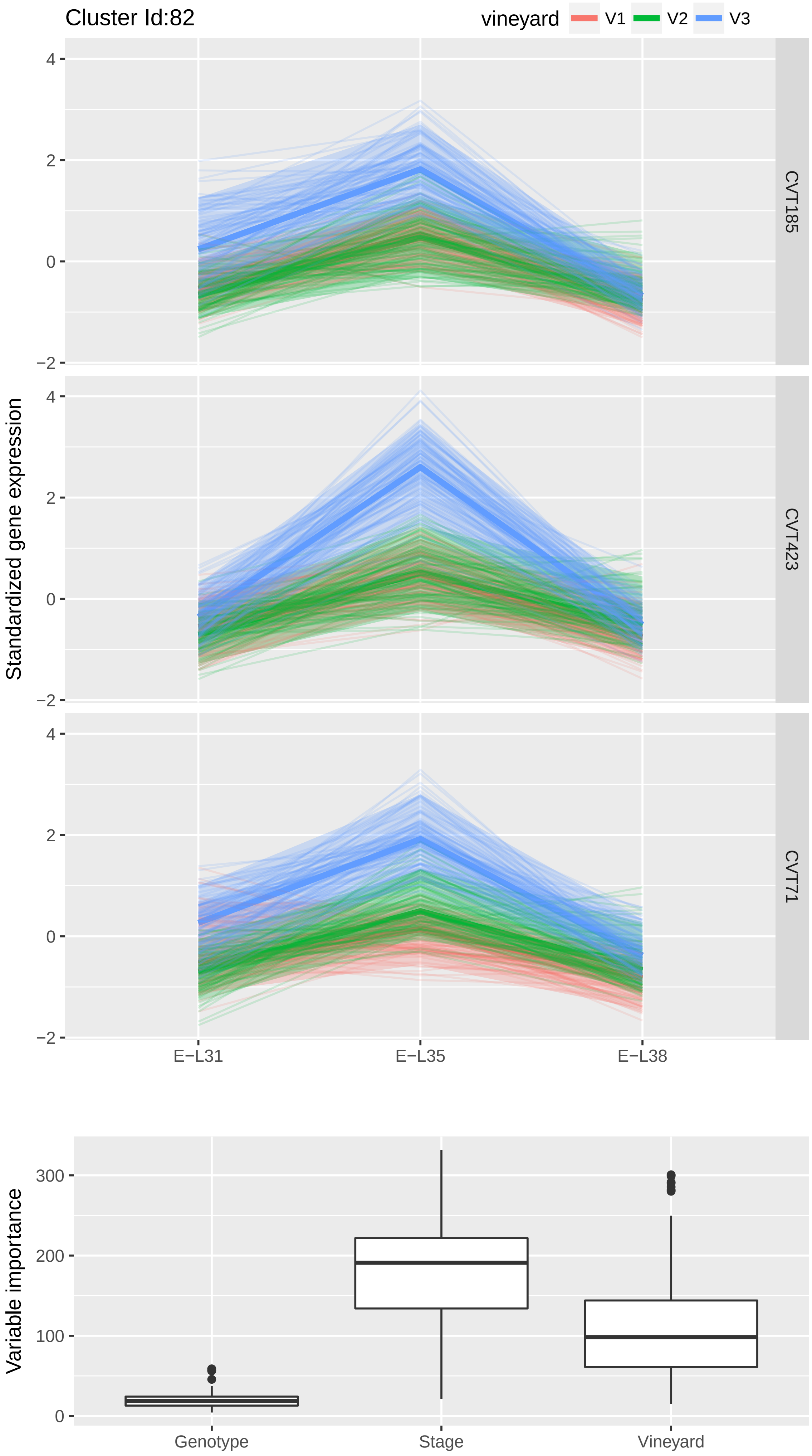
Cluster Id:82
vineyard
V1
V2
V3
Standardized gene expression
CVT185
CVT423
CVT71
E−L31
E−L35
E−L38
Variable importance
Genotype
Stage
Vineyard

Cluster no. 83

```
## Number of genes in the cluster: 55
## Homogeneity Index:    0.46
## Variable importance for Stage:    Median =  33.71  - Rank =  111
## Variable importance for Clone:    Median =  41.73  - Rank =  3
## Variable importance for Vineyard: Median =  198.9  - Rank =  3
##
## Gene ID               Gene Annotation
## VIT_17s0000g02410  -  Cold-induced wall associated kinase
## VIT_11s0016g03720  -  Aspartate aminotransferase, cytoplasmic (Transaminase A)
## VIT_02s0012g02240  -  Unknown protein
## VIT_00s0214g00150  -  F-box protein PP2-B1 (Protein phloem protein 2-like B1)
## VIT_09s0002g06360  -  Unknown protein
## VIT_08s0040g00440  -  Unknown protein
## VIT_01s0026g00900  -  Metalloendoproteinase 1 precursor
## VIT_07s0005g00750  -  Sucrose synthase
## VIT_04s0210g00080  -  Strictosidine synthase
## VIT_01s0026g01340  -  Glutathione S-transferase 29 GSTU18
## VIT_17s0000g09190  -  Octicosapeptide/Phox/Bem1p (PB1) domain-containing protein
## VIT_00s2607g00010  -  Phosphoserine aminotransferase
## VIT_14s0128g00520  -  Alpha-L-fucosidase 2 precursor
## VIT_02s0012g01590  -  Unknown protein
## VIT_00s0409g00050  -  Receptor-like serine-threonine protein kinase
## VIT_07s0205g00040  -  Unknown protein
## VIT_18s0122g01270  -  Protein kinase APK1B
## VIT_17s0000g04890  -  D-aminoacyl-tRNA deacylase GEKO1
## VIT_00s0374g00030  -  ARK3 (Arabidopsis Receptor Kinase 3)
## VIT_02s0025g01780  -  Cellulose synthase CSLG3
## VIT_18s0041g01330  -  RPS4 (resistant to p. syringae 4)
## VIT_15s0045g00330  -  Ring-H2 finger protein ATL3A
## VIT_06s0004g08250  -  Leucine-rich repeat transmembrane protein kinase, putative
## VIT_01s0011g02790  -  IDA (inflorescence deficient in abscission)
## VIT_04s0023g03370  -  flavanone-3-hydroxylase  1 (F3H1) [Vitis vinifera]
## VIT_03s0017g01410  -  No hit
## VIT_07s0005g06290  -  Cytochrome b5 isoform Cb5-C
## VIT_10s0003g00900  -  Anthranilate N-hydroxycinnamoyl/benzoyltransferase
## VIT_04s0008g04210  -  GLB3 (2-on-2 hemoglobin like gene 3)
## VIT_00s0790g00020  -  CYP81D2
## VIT_18s0001g11990  -  Methyltransferase-like protein 2, meth_7
## VIT_14s0006g02820  -  Mannose-6-phosphate isomerase
## VIT_12s0035g00620  -  Basic Leucine Zipper Transcription Factor (VvbZIP32)
## VIT_16s0098g01380  -  Yippee
## VIT_04s0023g01130  -  E3 ubiquitin-protein ligase RNF5
## VIT_14s0066g01060  -  Polygalacturonase GH28
## VIT_11s0016g02550  -  Protein phosphatase 2 regulatory subunit B
## VIT_17s0000g08770  -  Cysteine-rich receptor-like protein kinase 2
## VIT_19s0014g04960  -  Nodulation receptor kinase
## VIT_17s0000g07510  -  Myb family
## VIT_06s0004g07940  -  Ribosomal protein S26 (RPS26C) 40S
## VIT_17s0053g00780  -  Homeodomain GLABROUS11
## VIT_16s0098g01810  -  Histone H2B , member E
## VIT_13s0073g00130  -  Pentatricopeptide (PPR) repeat membrane-associated salt-inducible prot
## VIT_03s0038g00220  -  Purple acid phosphatase 10 ATPAP10/PAP10
## VIT_14s0006g02750  -  Peptidyl-tRNA hydrolase
## VIT_12s0059g01820  -  Protein phosphatase 1D
```

```
## VIT_00s0253g00150  -  Methyl jasmonate esterase
## VIT_08s0007g05770  -  Pentatricopeptide (PPR) repeat-containing protein
## VIT_01s0011g05600  -  Receptor-like protein kinase
## VIT_07s0151g01080  -  Unknown protein
## VIT_13s0067g02050  -  Unknown protein
## VIT_04s0008g01260  -  Zinc transporter ZIP4
## VIT_00s2579g00010  -  Phosphoserine aminotransferase
## VIT_18s0041g01650  -  R protein L6
```

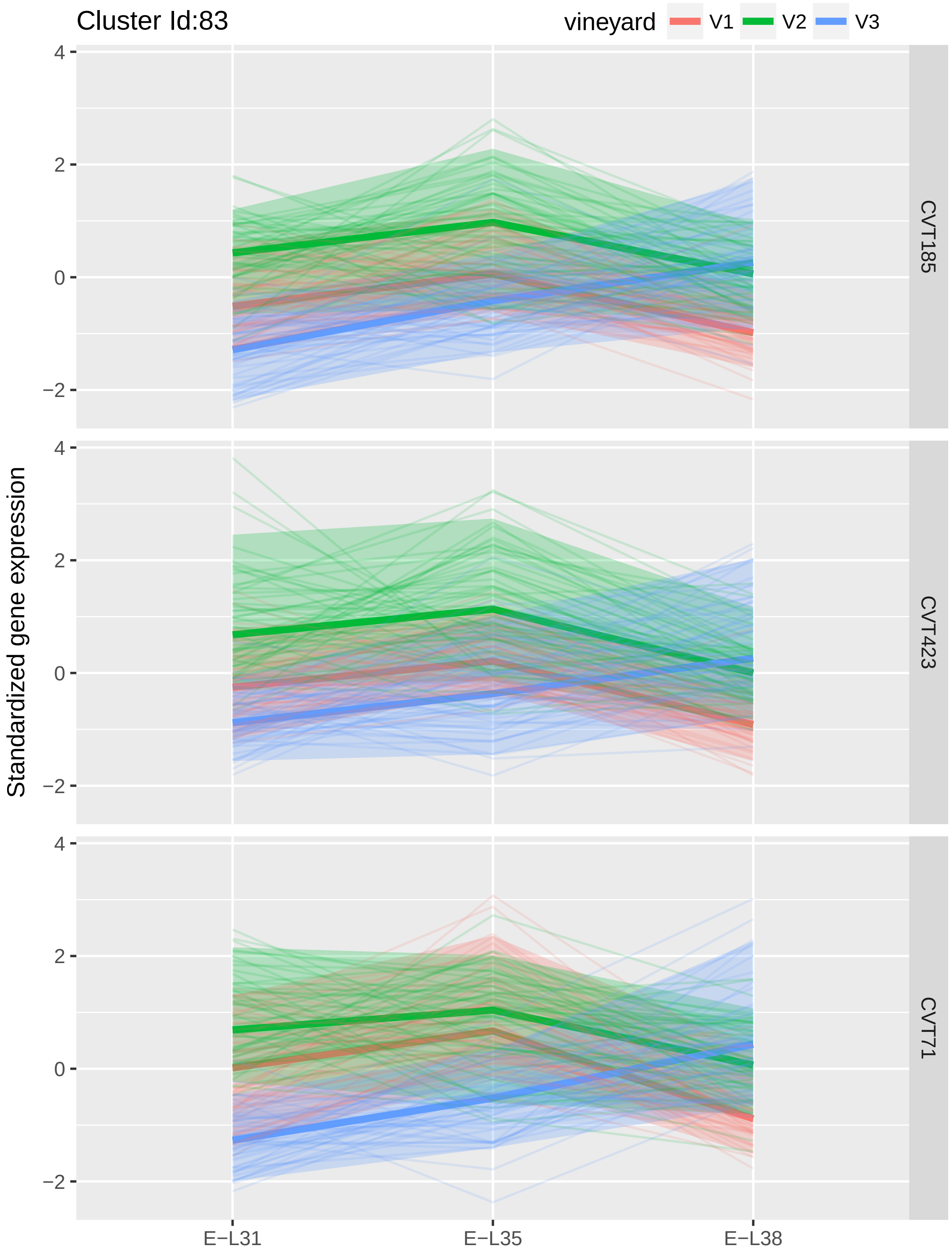
Cluster Id:83
vineyard
V1
V2
V3
Standardized gene expression
4
2
0
−2
CVT185
CVT423
CVT71
E−L31
E−L35
E−L38

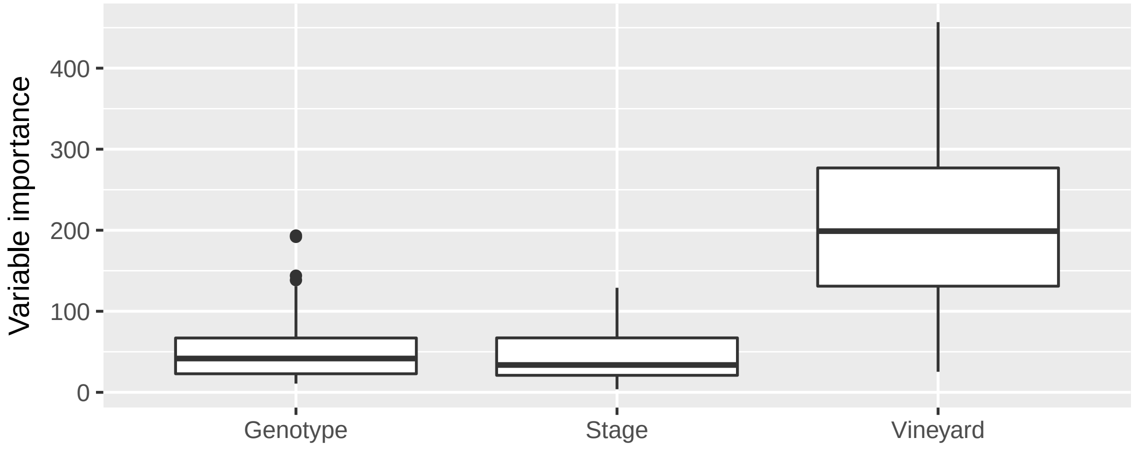
Variable importance
400
300
200
100
0
Genotype
Stage
Vineyard

Cluster no. 84

```
## Number of genes in the cluster: 111
## Homogeneity Index:    0.75
## Variable importance for Stage:    Median =    268  - Rank =  77
## Variable importance for Clone:    Median =  20.48  - Rank =  45
## Variable importance for Vineyard: Median =  74.61  - Rank =  24
##
## Gene ID               Gene Annotation
## VIT_08s0007g07420  -  MUT9-related serine/threonine protein kinase
## VIT_08s0007g02160  -  Transposon protein, CACTA, En/Spm sub-class
## VIT_01s0010g03690  -  Splicing factor YT521-B
## VIT_11s0016g00200  -  CBL-interacting protein kinase 6 (CIPK6)
## VIT_04s0044g02020  -  Kinesin ZWI (ZWICHEL)
## VIT_08s0040g01020  -  DNA helicase SNF2 domain-containing protein
## VIT_15s0046g01410  -  Unknown protein
## VIT_11s0016g00210  -  No hit
## VIT_18s0001g01870  -  SUR2
## VIT_18s0001g04180  -  Auxin response factor ARF17
## VIT_08s0007g05510  -  Unknown protein
## VIT_07s0031g01180  -  Armadillo helical
## VIT_08s0056g00100  -  Protein kinase
## VIT_07s0130g00490  -  Anaphase-promoting complex/cyclosome 8
## VIT_07s0130g00340  -  Esterase/lipase/thioesterase
## VIT_01s0011g01640  -  ARE1
## VIT_16s0050g00160  -  DTA2 (downstream target of AGL15 2)
## VIT_10s0003g01990  -  RKF1 (receptor-like kinase in flowers 1)
## VIT_19s0015g01210  -  KH domain-containing protein
## VIT_18s0001g05780  -  Leucine-rich repeat family protein
## VIT_00s0218g00090  -  Exostosin family protein
## VIT_12s0028g01170  -  Auxin response factor ARF6
## VIT_11s0052g00970  -  AAA-type ATPase
## VIT_15s0048g00320  -  UDP-glucuronic acid epimerase 1
## VIT_02s0025g00150  -  PRLI-interacting factor G
## VIT_19s0014g05330  -  Ankyrin repeat
## VIT_07s0095g00550  -  D-mannose binding lectin
## VIT_18s0001g08330  -  Erg28 like protein
## VIT_03s0063g02500  -  Adenylyl cyclase associated protein
## VIT_19s0015g01270  -  Proteasome activator subunit 4
## VIT_05s0020g00440  -  Boron transporter-like protein 1
## VIT_04s0008g01350  -  Conserved oligomeric Golgi complex component 8
## VIT_12s0028g00140  -  KCO6 (Ca2+ activated outward rectifying K+ channel 6)
## VIT_18s0001g12990  -  Anthranilate N-benzoyltransferase protein 1
## VIT_09s0002g08410  -  Amino acid permease
## VIT_07s0005g02260  -  Squamosa promoter-binding protein (VvSBP7)
## VIT_12s0059g01040  -  Unknown protein
## VIT_04s0008g02900  -  Protein disulfide isomerase
## VIT_14s0006g01970  -  Auxin efflux carrier family
## VIT_18s0041g02140  -  putative MADS-box Agamous-like 12 (VviAGL12)
## VIT_13s0067g00570  -  En/Spm transposon protein
## VIT_08s0217g00010  -  Succinate/fumarate mitochondrial transporter
## VIT_18s0001g14670  -  Rac-like GTP-binding protein RAC1
## VIT_18s0001g11220  -  EMB1427 (embryo defective 1427)
## VIT_12s0142g00130  -  Mitogen-activated Protein Kinase (VvMPK8)
## VIT_00s0313g00080  -  Kinesin motor protein
## VIT_06s0004g04850  -  XH/XS domain-containing protein
```

```
## VIT_04s0008g04350  -  Syntaxin 5
## VIT_18s0041g01850  -  Polyneuridine-aldehyde esterase precursor
## VIT_11s0016g05010  -  Lactoylglutathione lyase
## VIT_07s0005g04360  -  Unknown protein
## VIT_01s0010g02820  -  OTU cysteine protease
## VIT_07s0005g01830  -  Unknown protein
## VIT_06s0004g01750  -  Polygalacturonase inhibitor protein
## VIT_08s0007g01630  -  Tubulin beta-2 chain
## VIT_05s0020g00890  -  ABC Transporter (VvMDR10 - VvABCB10)
## VIT_06s0009g03450  -  LRP1 (lateral root primordium 1)
## VIT_08s0040g02710  -  UDP-glucuronic acid decarboxylase 1
## VIT_05s0029g00480  -  Eceriferum 2 (CER2)
## VIT_09s0002g02410  -  RNA-binding protein
## VIT_18s0075g00250  -  Unknown protein
## VIT_07s0095g00190  -  No hit
## VIT_16s0022g01640  -  Receptor serine/threonine kinase
## VIT_07s0129g00630  -  Unknown protein
## VIT_08s0056g00110  -  Emp24/gp25L/p24
## VIT_17s0000g05800  -  Aldehyde Dehydrogenase (VvALDHI2A1)
## VIT_04s0008g05650  -  Vesicle-associated membrane protein 7
## VIT_17s0000g04670  -  Serine/threonine protein kinase ATPK3
## VIT_14s0068g01150  -  Sialyltransferase
## VIT_03s0038g00460  -  SH3 domain-containing protein 2
## VIT_16s0050g01620  -  ABC Transporter (VvWBC23 - VvABCG23)
## VIT_18s0001g13260  -  ATP-dependent protease La (LON) domain-containing protein
## VIT_19s0090g00020  -  TOR1 (TORTIFOLIA 1)
## VIT_06s0009g02500  -  Plastocyanin domain-containing protein
## VIT_06s0004g06540  -  Undecaprenyl pyrophosphate synthetase
## VIT_00s0313g00040  -  PAS2 (PASTICCINO 2)
## VIT_08s0040g02470  -  Inorganic pyrophosphatase
## VIT_01s0137g00770  -  Unknown protein
## VIT_18s0001g00810  -  24-methylenesterol C-methyltransferase 2
## VIT_14s0060g00230  -  Pectinacetylesterase
## VIT_14s0060g00600  -  Shaggy protein kinase alpha (ASK-alpha)
## VIT_10s0003g02030  -  Nitrate transporter 1:2
## VIT_11s0016g03960  -  Kinesin family member 2/24
## VIT_11s0016g04190  -  Extensin-like protein
## VIT_16s0022g01650  -  Receptor serine/threonine kinase PR5K
## VIT_07s0031g00060  -  3-beta-glucuronosyltransferase
## VIT_05s0049g00600  -  No hit
## VIT_02s0241g00040  -  Cyclobutane pyrimidine dimer photolyase
## VIT_10s0116g01880  -  MATE efflux family protein
## VIT_05s0029g00410  -  Werner Syndrome exonuclease (WEX)
## VIT_07s0129g00490  -  Hydrogenobyrinic acid a,c-diamide synthase
## VIT_08s0032g00930  -  Fasciclin domain-containing protein FLA17
## VIT_10s0071g00670  -  Calcium-binding EF hand
## VIT_14s0060g02070  -  Protein phosphatase 2C
## VIT_05s0020g02890  -  GPI-anchored protein-like protein II
## VIT_13s0067g02160  -  Ca(2+)-dependent nuclease
## VIT_11s0016g04690  -  SOUL heme-binding
## VIT_04s0008g05180  -  Ankyrin
## VIT_07s0197g00250  -  Disease resistance protein (CC-NBS-LRR class)
## VIT_16s0050g00190  -  DTA2 (downstream target of AGL15 2)
## VIT_06s0004g04520  -  OBP3 (OBF-binding protein 3)
## VIT_00s0229g00170  -  Unknown protein
```

```
## VIT_07s0104g01250  -  flavin-containing monooxygenase
## VIT_18s0001g02340  -  Armadillo/beta-catenin repeat
## VIT_19s0014g02980  -  MLO11 (mildew resistance locus O 11)
## VIT_12s0028g03570  -  SPA3 (SPA1-related 3)
## VIT_08s0032g00290  -  RNA-binding region RNP-1
## VIT_01s0026g00840  -  ARL1 (ARG1-like 1)
## VIT_15s0046g01930  -  fringe protein
## VIT_06s0004g02850  -  Aquaporin PIP2B
## VIT_14s0068g01540  -  PBS1 (avrPphB susceptible 1)
```

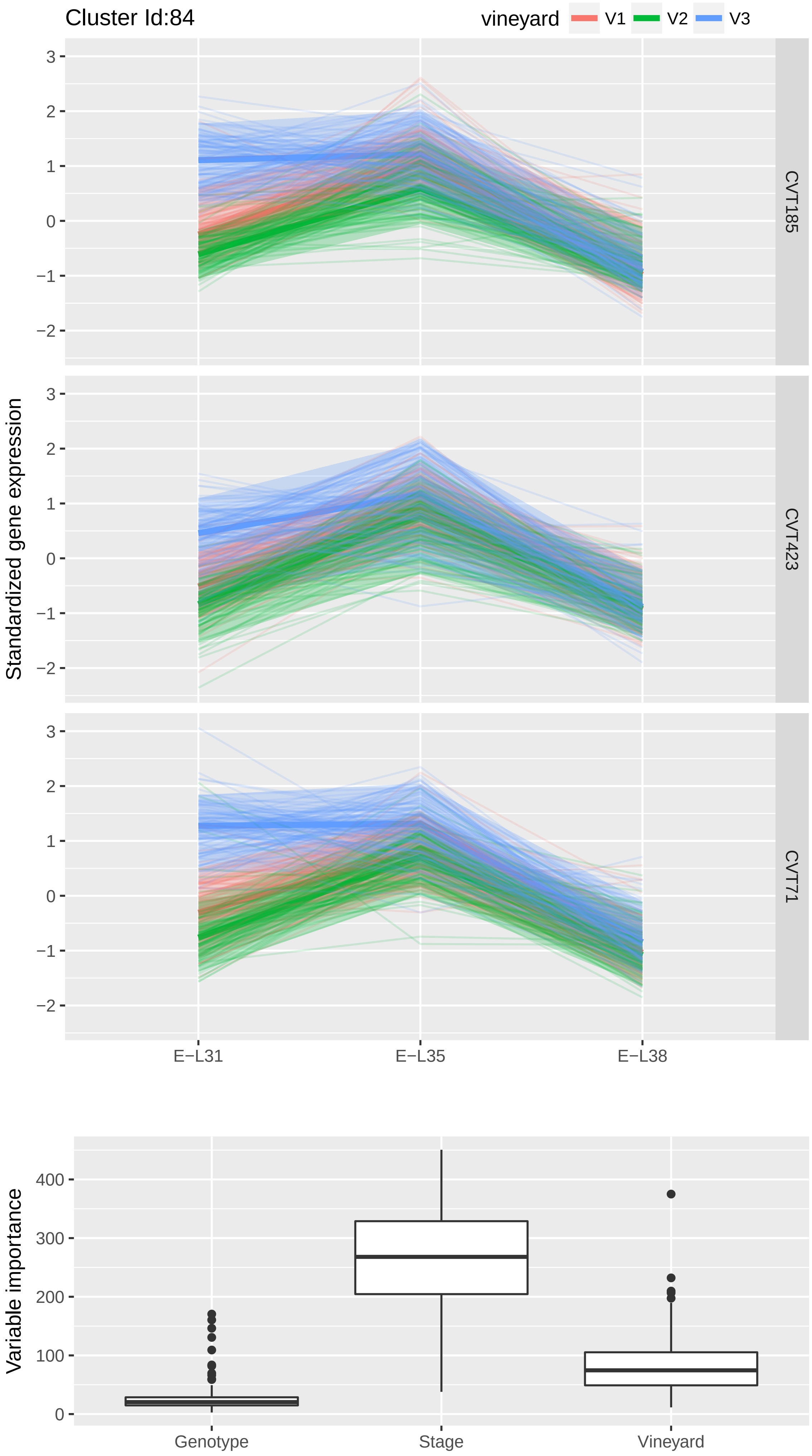

Cluster Id:84
vineyard
V1
V2
V3
CVT185
CVT423
CVT71
Standardized gene expression
3
2
1
0
−1
−2
E−L31
E−L35
E−L38
Variable importance
400
300
200
100
0
Genotype
Stage
Vineyard

Cluster no. 85

```
## Number of genes in the cluster: 121
## Homogeneity Index:    0.63
## Variable importance for Stage:    Median =  310.5  - Rank =  68
## Variable importance for Clone:    Median =  21.52  - Rank =  38
## Variable importance for Vineyard: Median =   72.1  - Rank =  27
##
## Gene ID               Gene Annotation
## VIT_05s0029g00500  -  DnaJ homolog, subfamily A, member 2
## VIT_01s0010g00560  -  Unknown protein
## VIT_02s0025g01510  -  Unknown
## VIT_05s0020g00630  -  Ethylene-induced calmodulin-binding protein 1
## VIT_15s0048g02990  -  AAA-type ATPase
## VIT_11s0118g00250  -  IMP dehydrogenase/GMP reductase
## VIT_18s0001g02300  -  NAC domain-containing protein (VvNAC08)
## VIT_15s0021g02790  -  ATELC/ELC
## VIT_15s0046g02480  -  Protein kinase
## VIT_06s0004g06940  -  Zinc transporter ZIP6
## VIT_11s0118g00760  -  Zinc finger (C3HC4-type ring finger)
## VIT_01s0011g05950  -  NSL1 (necrotic spotted lesions 1)
## VIT_04s0210g00060  -  Mucin-like protein
## VIT_13s0064g01300  -  RAB GTPase SMG1
## VIT_06s0004g08150  -  trans-cinnamate 4-monooxygenase (VvC4H)
## VIT_02s0025g04170  -  Heat shock transcription factor B2B
## VIT_14s0006g01930  -  Unknown protein
## VIT_10s0042g01150  -  ARGONAUTE 2 (AGO2)
## VIT_17s0000g00570  -  Unknown protein
## VIT_10s0071g01070  -  Unknown protein
## VIT_02s0012g01570  -  Cinnamoyl-CoA reductase
## VIT_06s0009g02640  -  Hydroxymethylglutaryl-CoA lyase
## VIT_13s0158g00150  -  Crinkly4
## VIT_03s0088g01180  -  Proline iminopeptidase
## VIT_19s0014g00180  -  Ribokinase
## VIT_12s0059g01200  -  UBX domain-containing protein 1
## VIT_13s0019g04190  -  Chorismate synthase 1, chloroplast precursor
## VIT_11s0016g01900  -  N-MYRISTOYLtransferase
## VIT_10s0042g01180  -  ARGONAUTE 2 (AGO2)
## VIT_18s0001g05030  -  Glucan 1,3-beta-glucosidase precursor
## VIT_14s0171g00400  -  Lysine histidine transporter 1
## VIT_18s0001g14840  -  Nickel ion transporter
## VIT_08s0007g01110  -  No hit
## VIT_02s0087g00330  -  Glycosyl transferase family 1 protein
## VIT_13s0019g02100  -  No hit
## VIT_13s0084g00240  -  Steroid sulfotransferase
## VIT_12s0057g00680  -  Constans interacting protein 6
## VIT_09s0002g03550  -  ABC Transporter (VvPDR3 - VvABCG33)
## VIT_11s0016g02580  -  MAPK activating protein-like
## VIT_09s0002g03560  -  ABC Transporter (VvPDR4 - VvABCG34)
## VIT_00s0324g00050  -  UDP-glucose glucosyltransferase
## VIT_13s0019g02110  -  fringe-related protein
## VIT_14s0068g00730  -  Unknown protein
## VIT_18s0001g12800  -  Dihydroflavonol 4-reductase (VvDFR)  mRNA  XM_002281822.1
## VIT_17s0000g09870  -  MA3 domain-containing protein
## VIT_15s0046g03090  -  DTA2 (downstream target of AGL15 2)
## VIT_18s0164g00040  -  Unknown protein
```

```
## VIT_13s0019g00690  -  Carnitine racemase
## VIT_08s0007g05000  -  S-adenosylmethionine synthetase
## VIT_09s0002g03640  -  ABC Transporter (VvPDR7 - VvABCG37)
## VIT_12s0035g00330  -  AAA-type ATPase
## VIT_01s0127g00260  -  ATP-citrate synthase
## VIT_03s0017g02350  -  Naringenin 3-dioxygenase
## VIT_00s0374g00050  -  U-box domain containing protein
## VIT_02s0025g04310  -  Thaumatin
## VIT_02s0154g00450  -  Zinc knuckle
## VIT_11s0118g00180  -  HHP1 (heptahelical protein 1)
## VIT_12s0035g00350  -  AAA-type ATPase
## VIT_05s0094g00330  -  Chitinase, class IV [Vitis vinifera]
## VIT_01s0150g00410  -  Unknown protein
## VIT_11s0016g00810  -  Inter-alpha-trypsin inhibitor heavy chain
## VIT_04s0008g06250  -  Serine-rich protein
## VIT_18s0001g04160  -  Translation initiation factor eIF-5
## VIT_06s0009g01420  -  Protease inhibitor/seed storage/lipid transfer protein (LTP)
## VIT_08s0007g08010  -  No hit
## VIT_19s0085g00850  -  LEM3 (ligand-effect modulator 3)
## VIT_14s0068g00930  -  chalcone synthase 1 (CHS1)
## VIT_19s0014g03510  -  ARF GTPase activator, ARF-GAP Domain 5
## VIT_05s0077g00190  -  Sodium-inducible calcium-binding protein (ACP1)
## VIT_02s0025g05120  -  ACT domain-containing protein (ACR8)
## VIT_01s0011g01290  -  N-carbamyl-L-amino acid amidohydrolase
## VIT_13s0067g02500  -  Xanthine/uracil permease
## VIT_01s0011g02430  -  Metal-dependent phosphohydrolase HD domain-containing protein
## VIT_19s0027g00230  -  NAC domain-containing protein (VvNAC33)
## VIT_02s0025g01320  -  No hit
## VIT_03s0017g01980  -  Choline-phosphate cytidylyltransferase B
## VIT_18s0001g00140  -  Unknown protein
## VIT_07s0005g00140  -  Peripheral-type benzodiazepine receptor
## VIT_03s0063g00050  -  UDP-glucuronosyl/UDP-glucosyltransferase
## VIT_18s0117g00270  -  Zinc Finger Homeodomain Transcription Factor (VvZHD12)
## VIT_14s0068g00920  -  chalcone synthase 2 (CHS2)
## VIT_06s0009g01620  -  Harpin-induced protein
## VIT_07s0005g02570  -  WRKY Transcription Factor (VvWRKY20)
## VIT_13s0067g03070  -  ARR17 typeA
## VIT_09s0002g03570  -  ABC transporter G member 15
## VIT_04s0044g01300  -  DNA cross-link repair protein
## VIT_04s0044g01230  -  Unknown
## VIT_06s0004g05170  -  Lectin protein kinase
## VIT_17s0000g05850  -  ABC Transporter (VvATH2 - VvABCA3)
## VIT_16s0013g01140  -  Phosphofructokinase
## VIT_13s0019g01280  -  DC1 domain-containing protein
## VIT_15s0048g01420  -  Plastocyanin domain-containing protein
## VIT_18s0086g00520  -  No hit
## VIT_00s0916g00010  -  Zinc finger (C3HC4-type ring finger) protein (RMA1)
## VIT_06s0009g01410  -  No hit
## VIT_02s0025g04300  -  Thaumatin
## VIT_17s0000g00580  -  Calmodulin-7 (CAM7)
## VIT_04s0008g05090  -  Peptidyl-prolyl isomerase D (cyclophilin D)
## VIT_17s0000g01080  -  HVA22E (HVA22-like protein E)
## VIT_07s0141g00090  -  Fatty acid elongase 1
## VIT_04s0008g02250  -  Beta-ketoacyl-CoA synthase
## VIT_01s0011g04140  -  Octicosapeptide/Phox/Bem1p (PB1) domain-containing protein
```

```
## VIT_05s0124g00080  -  No hit
## VIT_10s0092g00710  -  Receptor protein kinase
## VIT_08s0007g05580  -  Embryo-abundant protein
## VIT_05s0077g00730  -  WRKY Transcription Factor (VvWRKY14)
## VIT_05s0077g01990  -  Epoxide hydrolase 2
## VIT_15s0046g02400  -  Glycerol-3-phosphate acyltransferase 8
## VIT_05s0020g01960  -  Na+/H+ antiporter isoform 2
## VIT_00s0218g00170  -  UDP-rhamnose/rhamnosyltransferase
## VIT_04s0023g02480  -  Dehydrin 1b
## VIT_13s0019g01250  -  Metallophosphoesterase
## VIT_02s0012g00760  -  Haloacid dehalogenase hydrolase
## VIT_17s0000g09570  -  CYP71A26
## VIT_13s0019g01260  -  Enoyl reductase
## VIT_08s0058g00770  -  WAVE3
## VIT_03s0017g01690  -  CIP1 (COP1-interacting protein 4)
## VIT_17s0000g08220  -  Unknown protein
## VIT_16s0050g00390  -  4-coumarate-CoA ligase
## VIT_04s0008g03430  -  Exostosin family
## VIT_01s0150g00170  -  SEC14 cytosolic factor
```

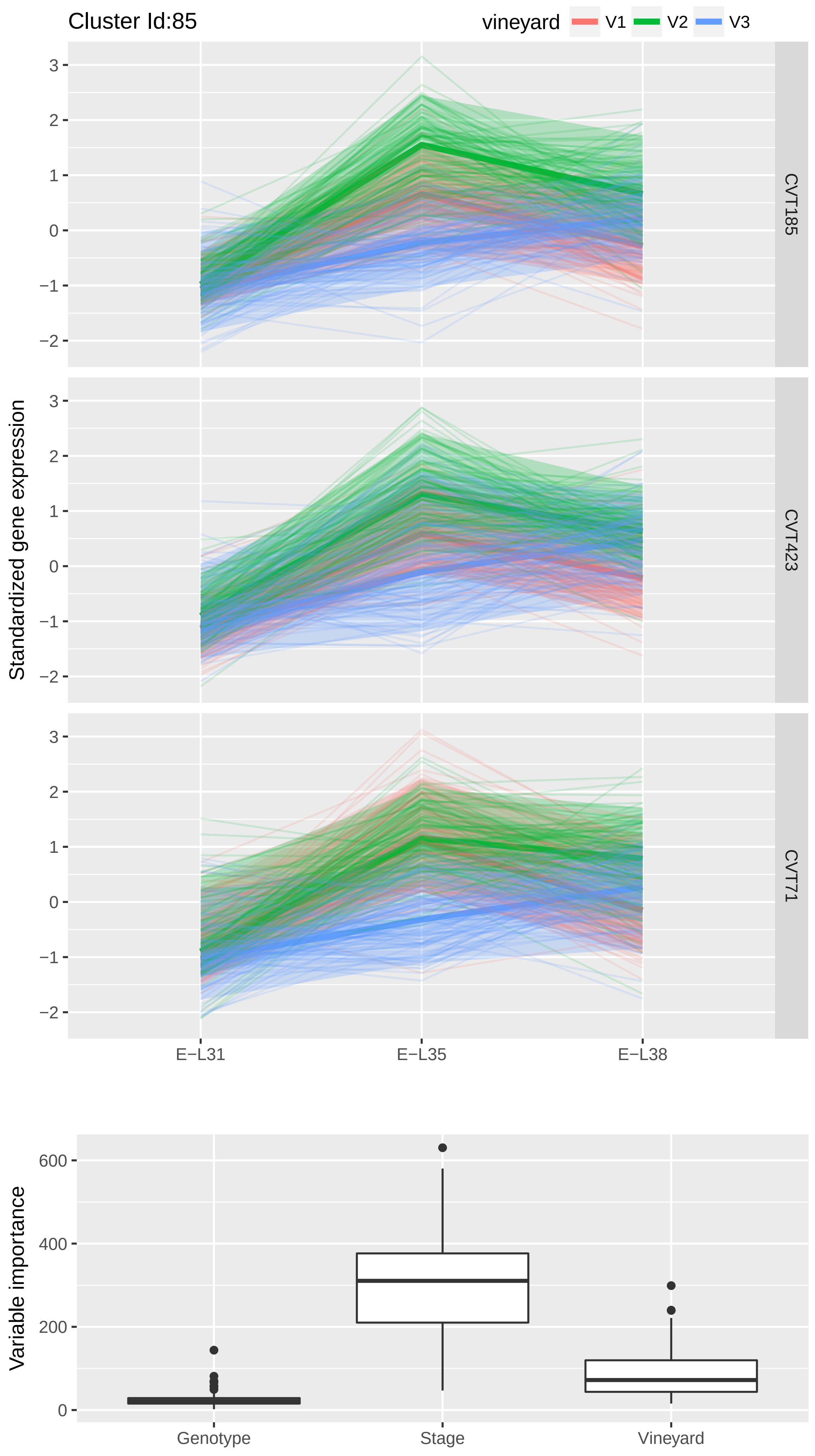

Cluster Id:85
vineyard
V1
V2
V3
Standardized gene expression
CVT185
CVT423
CVT71
3
2
1
0
−1
−2
E−L31
E−L35
E−L38
Variable importance
600
400
200
0
Genotype
Stage
Vineyard

Cluster no. 86

```
## Number of genes in the cluster: 195
## Homogeneity Index:    0.77
## Variable importance for Stage:    Median =    242  - Rank =  80
## Variable importance for Clone:    Median =  21.41  - Rank =  41
## Variable importance for Vineyard: Median =  35.78  - Rank =  57
##
## Gene ID                Gene Annotation
## VIT_14s0030g01240  -  Transport inhibitor response 1 protein
## VIT_12s0178g00040  -  Dentin sialophosphoprotein
## VIT_15s0046g00260  -  Unknown protein
## VIT_18s0001g14630  -  ATSYTB/NTMC2T1.2/NTMC2TYPE1.2/SYTB
## VIT_14s0066g00180  -  RAB GTPase RAB2C
## VIT_09s0018g01290  -  Unknown protein
## VIT_19s0014g04280  -  S-locus protein kinase
## VIT_19s0014g00960  -  Spg1p (septum-promoting GTPase)
## VIT_16s0100g00470  -  BCL2 binding anthogene
## VIT_05s0049g01450  -  No hit
## VIT_16s0022g01830  -  YLS7 (yellow-leaf-specific gene 7)
## VIT_07s0104g00350  -  Circadian clock coupling factor ZGT
## VIT_18s0001g02770  -  Alpha-L-fucosidase 2 precursor
## VIT_11s0016g02000  -  No hit
## VIT_04s0008g02560  -  AarF domain containing kinase
## VIT_01s0011g04820  -  Molecular chaperone DnaJ
## VIT_06s0080g00160  -  Vacuolar protein sorting 4
## VIT_14s0060g02000  -  ATPP2-B14
## VIT_16s0039g02750  -  NADH glutamate dehydrogenase
## VIT_18s0001g02760  -  Lipase GDSL
## VIT_13s0019g04200  -  Patatin
## VIT_19s0015g01310  -  Amino acid permease 7
## VIT_09s0002g06070  -  Late embryogenesis abundant protein
## VIT_07s0151g00800  -  Unknown
## VIT_07s0031g02250  -  Zinc finger (C3HC4-type ring finger)
## VIT_00s2419g00010  -  Heavy-metal-associated domain-containing protein
## VIT_06s0004g05550  -  RAB GTPase RABA2B
## VIT_17s0000g02020  -  Erwinia induced protein 2
## VIT_13s0139g00150  -  Seven in absentia SINA3
## VIT_02s0025g04720  -  Leucoanthocyanidin dioxygenase (VvLDOX) [Vitis vinifera]
## VIT_02s0154g00300  -  Small nuclear ribonucleoprotein Sm D3
## VIT_02s0025g00240  -  ß-carotene hydroxylase (BCH1) (VvBCH1)
## VIT_07s0005g03690  -  Protein kinase MK6
## VIT_09s0002g08540  -  Serine-rich protein
## VIT_03s0038g02880  -  Unknown
## VIT_18s0001g00210  -  Cytokinin riboside 5'-monophosphate phosphoribohydrolase LOG5
## VIT_04s0008g00690  -  Unknown protein
## VIT_07s0005g05520  -  Pectate lyase
## VIT_17s0000g06970  -  Diacylglycerol kinase 1
## VIT_17s0000g09480  -  Calcineurin phosphoesterase
## VIT_02s0033g00450  -  VvMybA3
## VIT_04s0008g02920  -  Desiccation protein PCC13-62 precursor
## VIT_08s0040g02700  -  Heavy-metal-associated domain-containing protein
## VIT_06s0004g05460  -  Protein phosphatase 2C DBP (VvPP2C-6)
## VIT_18s0122g01340  -  BTB/POZ domain-containing protein
## VIT_18s0001g05300  -  Trehalose-6-phosphate phosphatase
## VIT_14s0066g01380  -  Calcium homeostasis regulator CHoR1
```

```
## VIT_00s0264g00040  -  Prolyl 4-hydroxylase, alpha subunit
## VIT_16s0050g02680  -  Protein phosphatase 2C
## VIT_01s0010g01430  -  C2 domain-containing protein
## VIT_02s0025g04280  -  Osmotin
## VIT_04s0008g02040  -  Heterotrimeric G protein alpha subunit
## VIT_00s0441g00020  -  Squalene monooxygenase
## VIT_08s0007g06760  -  Cation efflux family protein MTPc3
## VIT_00s0404g00040  -  ARF GTPase activator, ARF-GAP Domain 5
## VIT_02s0025g04890  -  CYP76B1
## VIT_00s1683g00010  -  Wound-responsive protein
## VIT_18s0001g07370  -  Ubiquitin carboxyl-terminal hydrolase 16
## VIT_02s0025g04680  -  Cell division cycle 20-like protein 1
## VIT_08s0007g07620  -  Thioredoxin 2
## VIT_14s0006g02850  -  BEE1 (BR ENHANCED EXPRESSION 1)
## VIT_04s0008g02740  -  AarF domain containing kinase
## VIT_13s0019g02200  -  Protein phosphatase 2CA AHG3 PP2CA (VvPP2C-3)
## VIT_00s1389g00010  -  Cinnamyl alcohol dehydrogenase
## VIT_19s0093g00550  -  11,12 9-cis epoxycarotenoid dioxygenase (NCED3) (VvNCED3)
## VIT_19s0015g01290  -  Amino acid permease 7
## VIT_06s0004g02620  -  Phenylalanine ammonia-lyase
## VIT_02s0025g04870  -  No hit
## VIT_05s0051g00690  -  No hit
## VIT_08s0032g00760  -  Translation initiation factor eIF-2 beta subunit
## VIT_02s0033g00410  -  VvMybA1
## VIT_13s0106g00490  -  Vacuolar protein sorting 4
## VIT_05s0020g00840  -  No hit
## VIT_00s0225g00060  -  MATE efflux family protein
## VIT_03s0038g02280  -  ERD7 (EARLY-responsive TO dehydration 7)
## VIT_08s0007g02300  -  VPS60.2 SNF7
## VIT_01s0137g00780  -  Unknown protein
## VIT_03s0017g02360  -  AarF domain containing kinase
## VIT_14s0060g01460  -  Pentatricopeptide (PPR) repeat-containing protein
## VIT_14s0081g00030  -  Pathogenesis-related protein-4 (Chitinase )
## VIT_03s0063g01220  -  Nodulin 1A, senescence-associated
## VIT_01s0011g05570  -  Zinc finger (C2H2 type) protein pux2
## VIT_01s0011g04620  -  Unknown protein
## VIT_10s0071g00850  -  Binding
## VIT_05s0049g00480  -  Zinc finger (C3HC4-type ring finger)
## VIT_08s0007g01510  -  Phosphatidic acid phosphatase / PAP2
## VIT_08s0007g06040  -  Beta-1,3-glucanase
## VIT_18s0001g01030  -  Protein disulfide isomerase
## VIT_09s0002g01000  -  Ferredoxin 4Fe-4S, iron-sulfur binding
## VIT_02s0025g01150  -  No hit
## VIT_10s0116g00940  -  Early-responsive to dehydration protein / ERD protein
## VIT_06s0004g04350  -  Unknown protein
## VIT_00s0323g00070  -  Pectin methylesterase inhibitor
## VIT_03s0038g00860  -  Basic Leucine Zipper Transcription Factor (VvbZIP05)
## VIT_19s0014g03290  -  NAC domain-containing protein (VvNAC17)
## VIT_15s0048g01090  -  SPX (SYG1/Pho81/XPR1) domain-containing protein
## VIT_13s0064g00840  -  (5,6) (5`,6`) (9,10) (9`,10`) cleavage dioxygenase (CCD1) (VvCCD1.2)
## VIT_13s0067g00700  -  Hydrolase, alpha/beta fold
## VIT_04s0008g04410  -  Hypoxia up-regulated 1 HSP70
## VIT_06s0080g00970  -  NAC domain-containing protein (VvNAC67)
## VIT_14s0171g00510  -  Cell division cycle 48-interacting UBX-domain protein
## VIT_00s0407g00020  -  2-oxoglutarate/malate carrier protein, Mitochondrial
```

```
## VIT_02s0025g04880  -  Geraniol 10-hydroxylase
## VIT_04s0069g00860  -  Sarcosine oxidase
## VIT_03s0063g00710  -  Carboxyesterase 12; CXE12
## VIT_08s0007g04230  -  Unknown protein
## VIT_06s0004g02970  -  Zinc finger (C3HC4-type ring finger)
## VIT_00s1321g00010  -  Unknown
## VIT_05s0077g01460  -  No hit
## VIT_03s0063g00730  -  CXE carboxylesterase CXE10
## VIT_01s0011g02460  -  Receptor protein kinase PERK1
## VIT_04s0008g00650  -  3'-5' exonuclease domain-containing protein
## VIT_03s0063g00370  -  Nitrite reductase
## VIT_18s0122g00080  -  UPF0496 protein
## VIT_08s0007g05860  -  GASA like
## VIT_07s0031g02840  -  Diacylglycerol kinase 2
## VIT_15s0046g01440  -  Basic Leucine Zipper Transcription Factor (VvbZIP40)
## VIT_02s0025g04730  -  Glyoxylate reductase
## VIT_18s0001g02750  -  Unknown protein
## VIT_05s0136g00140  -  AarF domain containing kinase
## VIT_01s0010g03510  -  Anthocyanin  O-methyltransferase (VvAOMT1)
## VIT_11s0118g00420  -  Heparanase protein 3 precursor
## VIT_07s0005g01060  -  Histone H1
## VIT_08s0007g03640  -  Basic Leucine Zipper Transcription Factor (VvbZIP26)
## VIT_10s0071g00810  -  Binding
## VIT_18s0001g06270  -  Transcription factor jumonji (jmjC) domain-containing protein
## VIT_00s0346g00110  -  Mannitol dehydrogenase
## VIT_18s0001g12840  -  ADP-glucose pyrophosphorylase large subunit CagpL2
## VIT_00s0551g00030  -  Protein transport protein Sec61 subunit alpha
## VIT_18s0001g11630  -  Allene oxide synthase (jasmonates from fatty acids)
## VIT_15s0048g02870  -  Homeobox-leucine zipper protein HB-7 (VvATHB-8)
## VIT_08s0105g00230  -  BLH1 (embryo sac development arrest 29)
## VIT_10s0071g00840  -  Unknown protein
## VIT_13s0067g00720  -  AarF domain containing kinase
## VIT_08s0040g01170  -  Hydroxymethylglutaryl-CoA lyase
## VIT_00s0941g00020  -  Constans interacting protein 6
## VIT_13s0019g04260  -  Monoglyceride lipase
## VIT_16s0100g00270  -  Peptidoglycan-binding LysM domain-containing protein
## VIT_04s0023g02150  -  Phosphatidic acid phosphatase / PAP2
## VIT_15s0045g00040  -  Copper-binding family protein
## VIT_06s0004g04360  -  Amino acid binding protein
## VIT_07s0151g00850  -  Unknown
## VIT_04s0023g01110  -  PQ-loop repeat protein
## VIT_06s0004g01130  -  Seven in absentia SINA2
## VIT_11s0016g03170  -  Arabidopsis histidine phosphotransfer AHP4
## VIT_01s0011g01140  -  Cationic amino acid transporter 5
## VIT_07s0129g00510  -  Unknown
## VIT_00s2342g00020  -  No hit
## VIT_07s0104g00180  -  Nectarin IV NEC4
## VIT_03s0063g01820  -  AOS (allene oxide synthase)
## VIT_15s0048g00740  -  No hit
## VIT_17s0000g01930  -  Potassium transporter 2
## VIT_17s0000g09800  -  No hit
## VIT_04s0008g07020  -  Tic55 (translocon at the inner envelope membrane of chloroplasts 55)
## VIT_17s0000g06650  -  IBS1 (impaired in baba-induced sterility 1)
## VIT_13s0084g00310  -  ACI112
## VIT_15s0046g01610  -  Acidic endochitinase (CHIB1)
```

```
## VIT_11s0037g01210  -  Eceriferum 1 (CER1 protein) Sterol desaturase
## VIT_16s0039g00520  -  DNA helicase
## VIT_10s0003g03750  -  9-cis-epoxycarotenoid dioxygenase 2 (NCED2) (VvNCED2)
## VIT_12s0059g00490  -  Unknown
## VIT_14s0006g01660  -  Pyridoxal kinase
## VIT_01s0011g06270  -  Hydroxyproline-rich glycoprotein
## VIT_17s0000g03150  -  No hit
## VIT_11s0016g03180  -  ABI1 (ABA insensitive 1)
## VIT_08s0040g01890  -  Aquaporin PIP2;4
## VIT_07s0130g00320  -  Esterase/lipase/thioesterase
## VIT_10s0116g00700  -  DNA helicase SNF2 domain-containing protein
## VIT_04s0008g07310  -  AarF domain-containing kinase ABC1
## VIT_05s0062g01120  -  PIN1
## VIT_06s0004g03630  -  HAD superfamily hydrolase
## VIT_14s0006g01580  -  Unknown protein
## VIT_17s0000g09810  -  Pectate lyase
## VIT_10s0116g00420  -  DnaJ (Hsp40) homolog, subfamily C, member 25
## VIT_05s0051g00590  -  Pectate lyase
## VIT_15s0046g01590  -  Acidic chitinase III
## VIT_14s0066g00250  -  Lipase GDSL
## VIT_00s0220g00070  -  Latex cyanogenic beta glucosidase
## VIT_12s0059g00750  -  Prephenate dehydratase
## VIT_18s0001g01080  -  Unknown protein
## VIT_13s0067g01990  -  Unknown protein
## VIT_14s0060g00760  -  Galactinol synthase
## VIT_19s0093g00620  -  Ring-type E3 ubiquitin ligase
## VIT_12s0142g00160  -  Unknown protein
## VIT_01s0010g00240  -  CTP-synthetase
## VIT_16s0098g00980  -  Unknown
## VIT_16s0098g00690  -  Major Facilitator Superfamily
## VIT_05s0020g02860  -  Zinc finger (C3HC4-type ring finger)
## VIT_16s0098g00720  -  Lectin protein kinase
## VIT_11s0016g00760  -  Ubiquinone biosynthesis protein COQ9
## VIT_00s2574g00010  -  Protein transport protein Sec61 subunit alpha
## VIT_17s0000g07470  -  Binding
## VIT_13s0019g04280  -  Aquaporin PIP2B
## VIT_18s0001g01130  -  Expansin  (VvEXPA19)
## VIT_08s0007g03690  -  Inosine-uridine preferring nucleoside hydrolase family protein
```

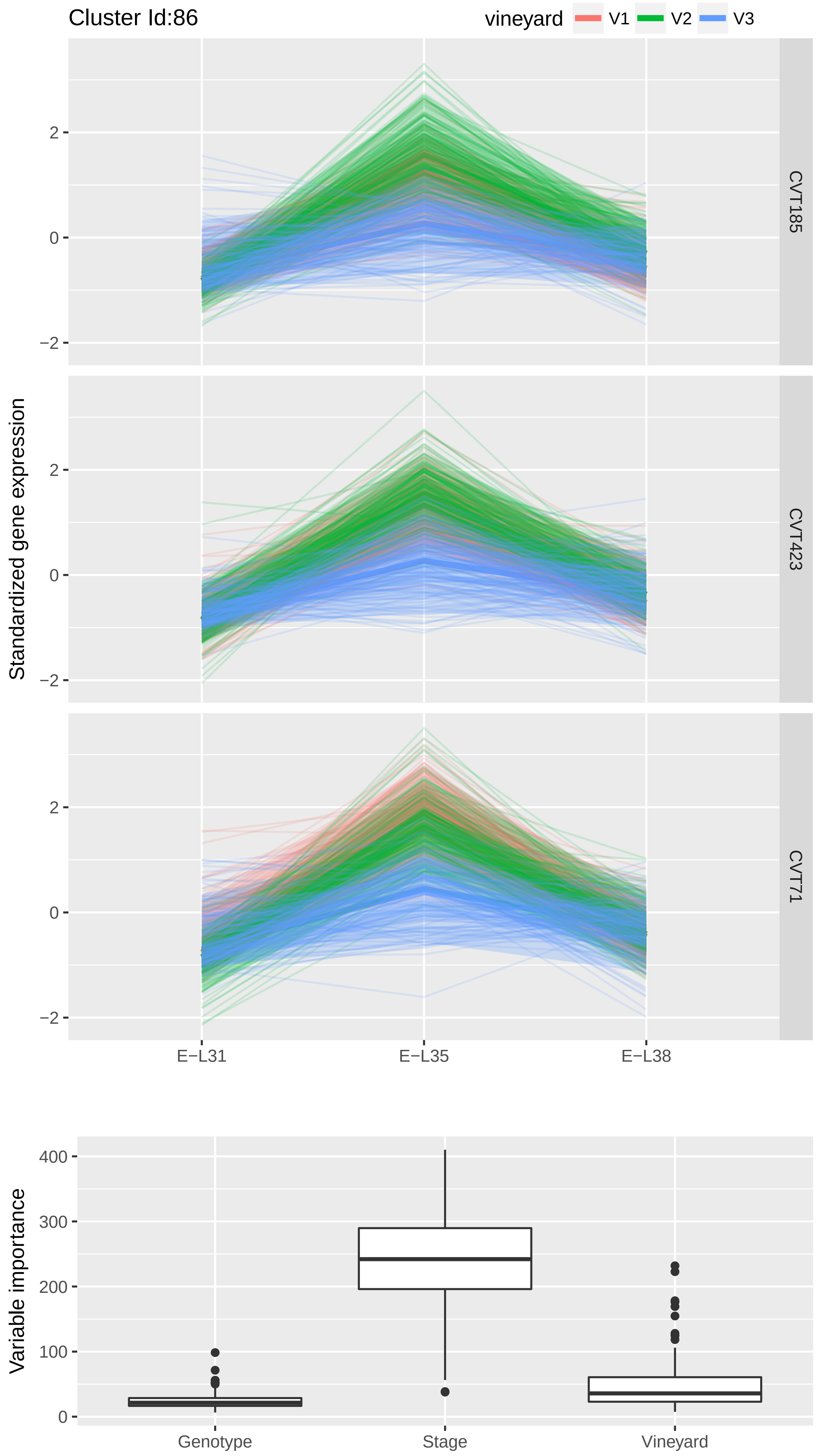

Cluster Id:86
vineyard
V1
V2
V3
Standardized gene expression
CVT185
CVT423
CVT71
2
0
−2
E−L31
E−L35
E−L38
Variable importance
400
300
200
100
0
Genotype
Stage
Vineyard

Cluster no. 87

```
## Number of genes in the cluster: 112
## Homogeneity Index:    0.75
## Variable importance for Stage:    Median =  519.9  - Rank =  29
## Variable importance for Clone:    Median =  13.99  - Rank =  78
## Variable importance for Vineyard: Median =  17.12  - Rank =  92
##
## Gene ID               Gene Annotation
## VIT_17s0000g01210  -  Unknown protein
## VIT_05s0020g04010  -  Unknown protein
## VIT_13s0067g01650  -  Glutaredoxin
## VIT_06s0061g01430  -  flavonoid 1-2 rhamnosyltransferase
## VIT_08s0040g01200  -  Short-chain type alcohol dehydrogenase
## VIT_11s0052g01840  -  Diacylglycerol kinase
## VIT_13s0067g03820  -  chalcone isomerase  1 [Vitis vinifera] (VvCHI1)
## VIT_04s0023g02360  -  Chaperone BCS1 mitochondrial
## VIT_01s0010g01680  -  Glutaredoxin
## VIT_18s0001g01650  -  Unknown
## VIT_05s0136g00260  -  chalcone synthase 3 (CHS3)
## VIT_15s0048g02970  -  Extensin
## VIT_19s0014g02650  -  Glycosyl transferase family 14 protein
## VIT_12s0028g00580  -  Unknown protein
## VIT_03s0017g00870  -  Transferase family / Anthocyanin Acyl-transferase (VvAnAT)
## VIT_01s0011g05650  -  1-aminocyclopropane-1-carboxylate oxidase
## VIT_17s0000g00620  -  Protein kinase CRK1
## VIT_17s0000g06400  -  NAC domain-containing protein (VvNAC05)
## VIT_05s0049g01020  -  VvMyb15
## VIT_18s0001g01060  -  Zinc finger (C3HC4-type ring finger)
## VIT_00s0956g00010  -  TRNA (guanine-N(1)-)-methyltransferase
## VIT_16s0039g02670  -  Translocase of chloroplast 34
## VIT_05s0020g03050  -  Unknown
## VIT_16s0022g00440  -  Dynein light chain LC8-type
## VIT_00s0768g00040  -  Receptor protein kinase PERK1
## VIT_14s0060g01390  -  Protein disulfide isomerase A6 precursor (P5)
## VIT_04s0008g02050  -  Hydrolase, alpha/beta fold
## VIT_14s0066g00360  -  NAC domain-containing protein (VvNAC59)
## VIT_16s0050g00650  -  No hit
## VIT_04s0008g06480  -  Alpha-1,3-galactosyltransferase
## VIT_03s0063g00120  -  Wax synthase
## VIT_19s0014g03390  -  Unknown protein
## VIT_00s0929g00020  -  Receptor protein kinase PERK1
## VIT_00s1291g00030  -  Receptor protein kinase PERK1
## VIT_07s0031g00990  -  Binding
## VIT_13s0019g05190  -  RAB GTPase RAB_ALPHA
## VIT_13s0074g00770  -  Copper chaperone ATX1
## VIT_07s0031g01020  -  L-ascorbate oxidase
## VIT_12s0059g01960  -  Unknown protein
## VIT_19s0014g01420  -  14-3-3 protein GF14 iota (GRF12)
## VIT_00s1227g00010  -  Unknown
## VIT_14s0060g02680  -  Cyclin H-1
## VIT_10s0003g00880  -  Nudix hydrolase 15
## VIT_05s0049g01890  -  Unknown
## VIT_18s0041g01680  -  No hit
## VIT_11s0052g01150  -  Nicotianamine synthase
## VIT_04s0008g06820  -  Unknown
```

```
## VIT_13s0067g02030  -  IMK2 (inflorescence meristem receptor-like kinase 2)
## VIT_09s0002g03510  -  ELF7 (early flowering 7)
## VIT_06s0004g07800  -  Pentatricopeptide (PPR) repeat-containing protein
## VIT_13s0156g00260  -  Homeobox-leucine zipper protein HAT14
## VIT_18s0001g01180  -  Vacuolar sorting-associated protein (Vps27)
## VIT_14s0068g01330  -  Transport inhibitor response 1 protein
## VIT_18s0001g08300  -  Tubulin alpha-6 chain
## VIT_12s0059g00880  -  WRKY Transcription Factor (VvWRKY37)
## VIT_08s0032g00920  -  Zinc finger (C3HC4-type ring finger) CIC7E11
## VIT_18s0001g01120  -  Proteasome 26S regulatory subunit S3 (RPN3)
## VIT_11s0052g00960  -  Syntaxin
## VIT_13s0067g03810  -  Cytochrome c oxidase subunit 6b (COX6b)
## VIT_06s0004g06580  -  PfkB-type carbohydrate kinase
## VIT_04s0023g00760  -  Biotin carboxyl carrier protein 2 (BCCP2)
## VIT_18s0041g01700  -  TIR-NBS-LRR-TIR disease resistance protein
## VIT_08s0007g02860  -  RNA-binding protein
## VIT_05s0136g00240  -  Ankyrin repeat protein
## VIT_13s0067g00550  -  Clathrin, light polypeptide B
## VIT_18s0041g00740  -  UDP-glucose: anthocyanidin 5,3-O-glucosyltransferase
## VIT_02s0012g02270  -  Autophagy protein ATG5
## VIT_10s0071g01030  -  Transducin family protein / WD-40 repeat
## VIT_04s0008g03810  -  Coiled-coil domain-containing protein 25
## VIT_17s0000g10000  -  Nitrate-responsive NOI protein
## VIT_07s0005g00490  -  UDP-glucuronic acid decarboxylase 1
## VIT_16s0039g01580  -  Carbohydrate kinase
## VIT_19s0014g03630  -  Unknown
## VIT_16s0039g01460  -  GPCR-type G protein12 GTG1
## VIT_16s0100g01270  -  F-box family protein
## VIT_05s0020g04730  -  Zinc finger (C3HC4-type ring finger)
## VIT_18s0001g07610  -  EMB1674 (embryo defective 1674) kinase interacting family protein
## VIT_10s0116g00820  -  Adenosine/AMP deaminase
## VIT_18s0001g08640  -  RNA-binding protein Musashi
## VIT_10s0116g00070  -  Unknown
## VIT_04s0210g00180  -  Isocitrate dehydrogenase subunit 1
## VIT_02s0025g03290  -  Hydroxyproline-rich glycoprotein
## VIT_10s0003g05020  -  GTP-binding protein obg
## VIT_15s0046g01300  -  No hit
## VIT_01s0011g01940  -  Unknown protein
## VIT_08s0032g00990  -  Expp1 protein precursor
## VIT_15s0048g00600  -  Vesicle-associated membrane protein
## VIT_18s0001g10150  -  ERF/AP2 Gene Family (VvERF006),Dehydration Responsive Element-Binding
## VIT_00s0220g00090  -  Leucine zipper-EF-hand containing transmembrane protein 1
## VIT_06s0004g02200  -  Unknown protein
## VIT_04s0044g02040  -  Ubiquitin carboxyl-terminal hydrolase L5
## VIT_18s0001g07720  -  TIFY gene family (VvZML3)
## VIT_04s0044g01060  -  Leaf senescence related protein-like
## VIT_12s0028g00980  -  myb family
## VIT_15s0021g01740  -  Unknown
## VIT_17s0000g10060  -  Lipase GDSL
## VIT_18s0122g00970  -  IRE1A (Yeast endoribonuclease/protein kinase IRE1-like gene)
## VIT_06s0004g04930  -  Oxysterol binding protein
## VIT_01s0137g00660  -  Coenzyme Q biosynthesis Coq4 protein
## VIT_18s0072g00670  -  GT2-like trihelix DNA-binding protein
## VIT_19s0015g00760  -  SEC14 cytosolic factor
```

```
## VIT_03s0091g00520  -  Prolyl 4-hydroxylase
## VIT_02s0087g00130  -  Unknown protein
## VIT_08s0040g03100  -  Glutathione S-transferase GSTO1
## VIT_06s0061g00280  -  2-phosphoglycerate kinase
## VIT_14s0066g02560  -  Thioredoxin H-type 2 (Trx-H-2)
## VIT_03s0063g00500  -  Prpk (p53-related protein kinase)
## VIT_06s0009g01610  -  Unknown protein
## VIT_16s0100g00570  -  Dehydration-responsive protein
## VIT_08s0056g00180  -  Translation initiation factor IF-2B subunit delta
## VIT_07s0031g01490  -  RKP (Related to KPC1)
## VIT_13s0067g01770  -  Steroid 5alpha-reductase
```

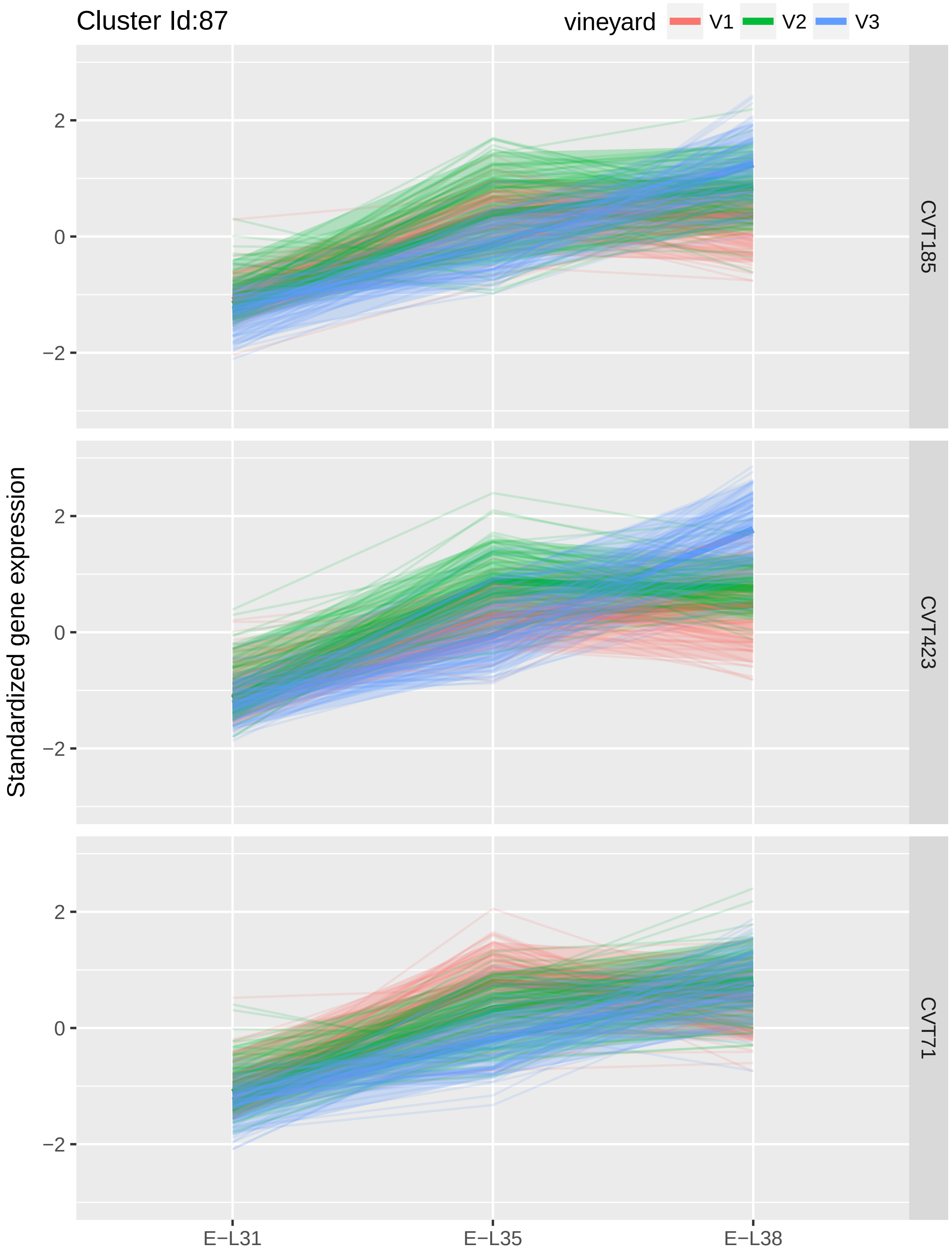
Cluster Id:87
vineyard
V1
V2
V3
Standardized gene expression
CVT185
CVT423
CVT71
2
0
−2
E−L31
E−L35
E−L38

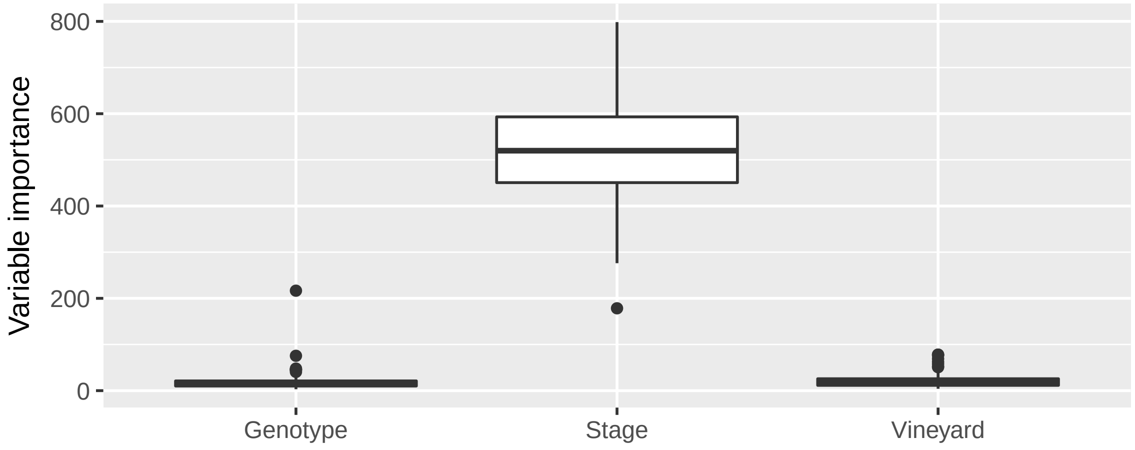
Variable importance
800
600
400
200
0
Genotype
Stage
Vineyard

Cluster no. 88

```
## Number of genes in the cluster: 78
## Homogeneity Index:    0.67
## Variable importance for Stage:    Median =  487.8  - Rank =  36
## Variable importance for Clone:    Median =  11.14  - Rank =  98
## Variable importance for Vineyard: Median =  34.88  - Rank =  59
##
## Gene ID                Gene Annotation
## VIT_05s0062g01000  -  Aldo/keto reductase
## VIT_05s0020g00810  -  Ribosomal protein L3
## VIT_01s0011g03410  -  DNA repair protein RAD23
## VIT_17s0000g05920  -  Kelch repeat-containing F-box protein
## VIT_04s0023g01530  -  Transcription initiation factor TFIIB
## VIT_14s0060g00350  -  Aspartic Protease (VvAP35)
## VIT_01s0010g03940  -  Sphingosine-1-phosphate lyase
## VIT_18s0001g13550  -  Zinc knuckle (CCHC-type) family
## VIT_14s0006g02960  -  KH domain containing protein
## VIT_05s0020g04640  -  Unknown protein
## VIT_05s0077g00900  -  No hit
## VIT_19s0014g04740  -  MRNA cap guanine-N7 methyltransferase 1
## VIT_15s0046g02870  -  Unknown protein
## VIT_14s0006g01890  -  Unknown protein
## VIT_01s0011g01180  -  DNA polymerase eta subunit
## VIT_09s0002g04680  -  Heat shock protein 91
## VIT_10s0003g03880  -  F-box domain containing protein
## VIT_15s0021g00370  -  Accumulation of photosystem one I
## VIT_00s0225g00020  -  Ribosomal protein L27A (RPL27aC) 60S
## VIT_02s0033g00060  -  DNAJ plastid division protein (ARC6)
## VIT_17s0000g03310  -  Heat shock protein 70
## VIT_11s0118g00580  -  Unknown
## VIT_02s0025g02520  -  Unknown protein
## VIT_08s0007g07010  -  Ribosomal protein L21 (RPL21A) 60S
## VIT_11s0016g03240  -  Avr9/Cf-9 rapidly elicited protein 146
## VIT_07s0104g00710  -  Auxin-binding protein 1 (ABP1)
## VIT_18s0001g11760  -  MATE efflux family protein
## VIT_01s0146g00170  -  N-ethylammeline chlorohydrolase BH0746
## VIT_13s0047g00460  -  Essential for mitotic growth 1
## VIT_15s0021g02760  -  Unknown
## VIT_14s0068g01640  -  Ribosomal protein L7A
## VIT_19s0176g00140  -  CID9 (CTC-Interacting Domain 9)
## VIT_07s0031g02740  -  Auxin-responsive SAUR12
## VIT_08s0007g06230  -  Protein translocase ATOEP16-3 Tim17
## VIT_11s0149g00400  -  Diadenosine tetraphosphatase
## VIT_09s0002g01180  -  Zinc finger (C3HC4-type ring finger)
## VIT_07s0031g01480  -  ATP/GTP binding protein
## VIT_07s0031g01190  -  Unknown protein
## VIT_03s0063g01330  -  Nodulin 1A, senescence-associated
## VIT_11s0016g04290  -  3-hydroxyisobutyryl-CoA hydrolase
## VIT_07s0130g00190  -  Suppressor of gene silencing 3 (SGS3)
## VIT_08s0007g03720  -  Calcineurin phosphoesterase
## VIT_05s0049g00120  -  Unknown protein
## VIT_04s0008g05940  -  Glycoprotein, Mitochondrial
## VIT_17s0000g06840  -  Unknown protein
## VIT_14s0006g01820  -  Histone deacetylase (RPD3A)
## VIT_05s0062g00960  -  Exostosin (Xyloglucan galactosyltransferase KATAMARI 1)
```

```
## VIT_16s0039g00930  -  CYP89A5
## VIT_13s0067g03650  -  Histone H4
## VIT_02s0012g02250  -  Basic Leucine Zipper Transcription Factor (VvbZIP04)
## VIT_14s0083g00930  -  BIM2 (BES1-interacting Myc-like protein 2)
## VIT_19s0090g01790  -  Armadillo/beta-catenin repeat
## VIT_04s0008g03770  -  Aspartate aminotransferase P1
## VIT_11s0103g00380  -  Cd2+-exporting ATPase HMA2 (Heavy metal ATPase 2)
## VIT_09s0002g02480  -  F-type H+-transporting ATPase g chain
## VIT_17s0000g06570  -  Timing of CAB expression 1 protein
## VIT_08s0217g00110  -  Basic helix-loop-helix aprataxin
## VIT_06s0009g03590  -  Unknown protein
## VIT_18s0001g09100  -  DNA excision repair protein ERCC-8
## VIT_09s0018g01490  -  Anthraniloyal-CoA: methanol anthraniloyal transferase
## VIT_18s0001g06080  -  UDP-glycosyltransferase 85A1
## VIT_12s0057g01140  -  AarF domain containing kinase
## VIT_06s0009g01800  -  CDKC;1; CDKC;1 (CYCLIN-dependent kinase C;1)
## VIT_19s0177g00070  -  Polyubiquitin (UBQ14)
## VIT_05s0020g04910  -  Double strand break repair protein (XRCC4)
## VIT_12s0055g00830  -  Methyltransferase
## VIT_08s0032g00950  -  Unknown protein
## VIT_03s0038g00950  -  Auxin-responsive SAUR9
## VIT_19s0177g00040  -  Polyubiquitin (UBQ14)
## VIT_12s0057g00590  -  Vacuolar protein sorting 11 (VPS11)
## VIT_19s0014g02440  -  MATE efflux family protein
## VIT_11s0103g00370  -  Cd2+-exporting ATPase HMA2 (Heavy metal ATPase 2)
## VIT_08s0056g00730  -  SEC14 cytosolic factor
## VIT_13s0101g00460  -  No hit
## VIT_12s0055g00390  -  COP9 signalosome complex subunit 8
## VIT_02s0025g00330  -  Unknown protein
## VIT_18s0001g11520  -  flavonoid 3-monooxygenase
## VIT_17s0000g00560  -  UPF0497 family
```

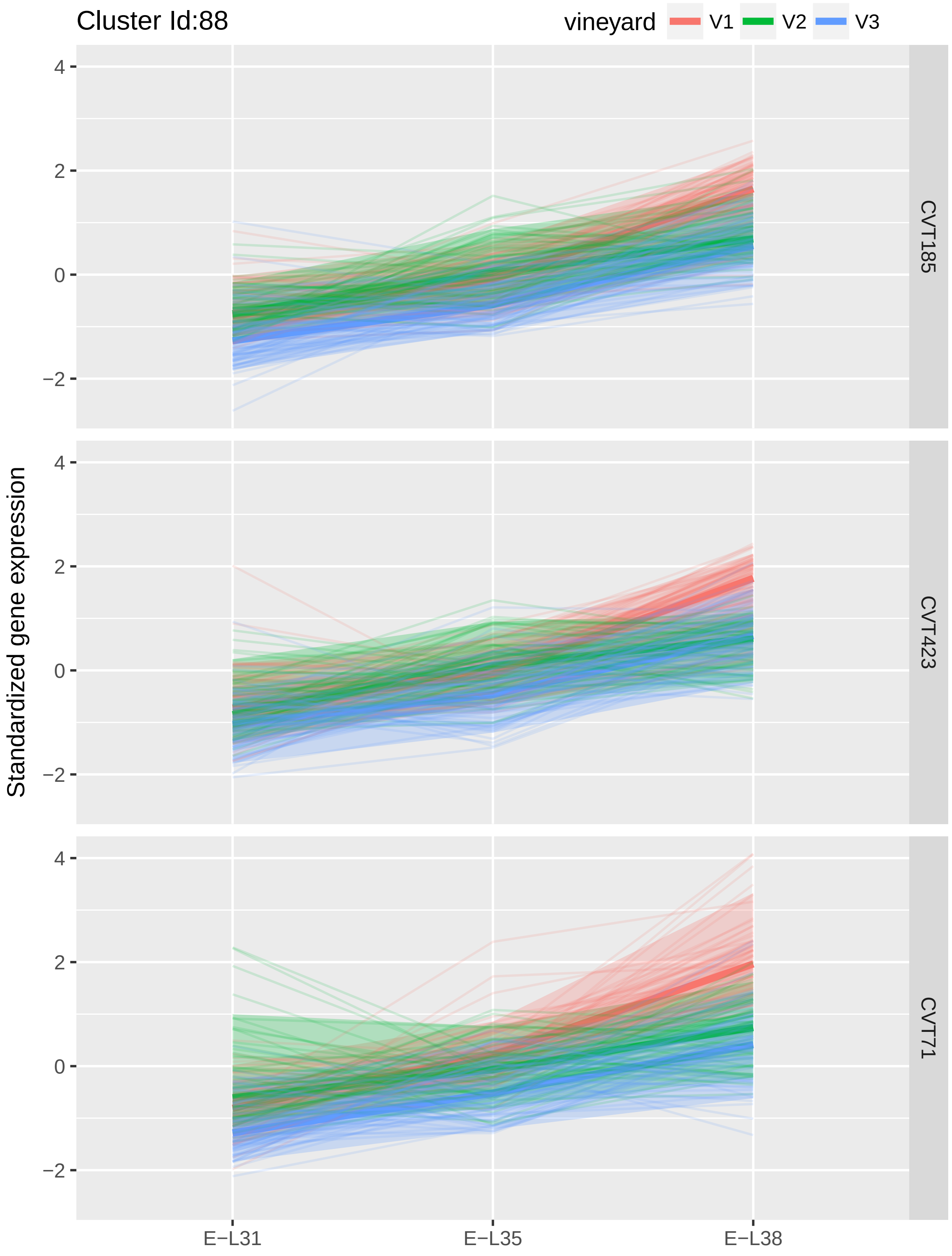

Cluster Id:88
vineyard
V1
V2
V3
4
2
0
−2
CVT185
CVT423
CVT71
Standardized gene expression
E−L31
E−L35
E−L38

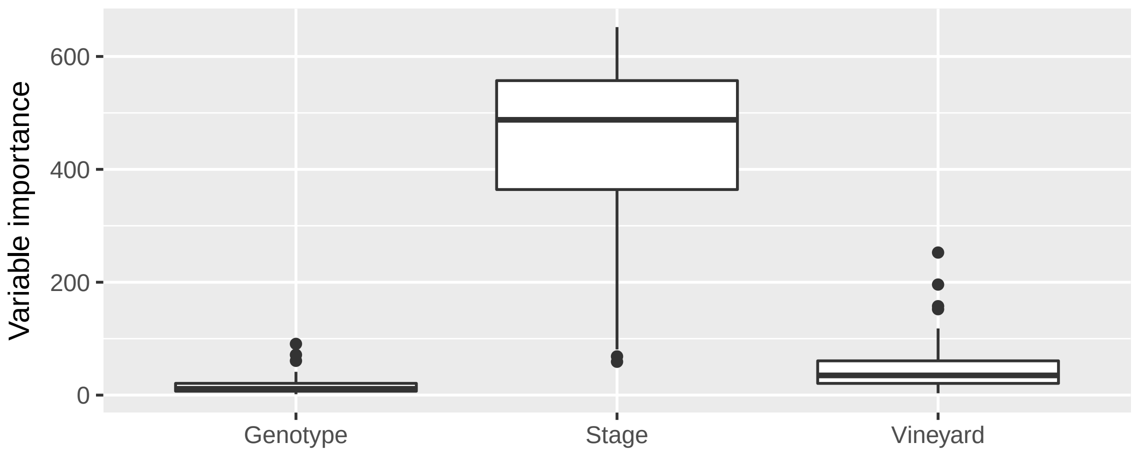

600
400
200
0
Variable importance
Genotype
Stage
Vineyard

Cluster no. 89

```
## Number of genes in the cluster: 148
## Homogeneity Index:    0.67
## Variable importance for Stage:    Median =    137  - Rank =  100
## Variable importance for Clone:    Median =  14.97  - Rank =  72
## Variable importance for Vineyard: Median =  191.2  - Rank =  5
##
## Gene ID               Gene Annotation
## VIT_17s0000g00650  -  No hit
## VIT_18s0122g00180  -  Calmodulin CML37
## VIT_07s0031g01380  -  ferulate 5-hydroxylase
## VIT_00s1352g00010  -  Myb domain protein 84
## VIT_18s0001g07320  -  2-oxoglutarate/malate carrier protein, Mitochondrial
## VIT_04s0008g02510  -  Receptor kinase homolog LRK10
## VIT_10s0003g00790  -  Glutamate receptor protein
## VIT_00s0225g00050  -  MATE efflux family protein
## VIT_00s0463g00020  -  Scarecrow transcription factor 5 (SCL5)
## VIT_17s0000g08380  -  Unknown protein
## VIT_05s0077g02350  -  No hit
## VIT_12s0057g00420  -  Auxin-responsive protein AIR12
## VIT_14s0030g02150  -  Calmodulin
## VIT_10s0003g00140  -  ERF/AP2 Gene Family (VvERF064)
## VIT_16s0098g00420  -  Ser/Thr receptor-like kinase1
## VIT_17s0000g00680  -  No hit
## VIT_09s0002g02950  -  No hit
## VIT_08s0032g01220  -  Calcium Dependent Protein Kinase (VvCPK9)
## VIT_18s0001g03620  -  Avr9/Cf-9 rapidly elicited protein 284
## VIT_18s0072g00990  -  Leucine-rich repeat protein kinase
## VIT_18s0001g09230  -  Salt tolerance zinc finger
## VIT_03s0017g02110  -  Anthocyanidin 3-O-glucosyltransferase
## VIT_18s0166g00260  -  No hit
## VIT_03s0063g00460  -  ERF/AP2 Gene Family (VvERF117)
## VIT_02s0025g04460  -  ERF/AP2 Gene Family (VvERF021)
## VIT_13s0067g01070  -  Cys-3-His zinc finger protein
## VIT_13s0067g02630  -  No hit
## VIT_16s0013g01110  -  Ethylene-responsive transcription factor 5
## VIT_13s0019g00480  -  Zinc finger (C2H2 type) family
## VIT_18s0072g00980  -  Leucine-rich repeat protein kinase
## VIT_15s0048g02070  -  BON2-associated protein (BAP2)
## VIT_11s0016g04050  -  No hit
## VIT_11s0016g01810  -  Unknown protein
## VIT_08s0058g00690  -  WRKY Transcription Factor (VvWRKY24)
## VIT_02s0025g03000  -  ERF/AP2 Gene Family (VvRAV3)
## VIT_18s0001g11680  -  Unknown protein
## VIT_07s0005g01140  -  Unknown protein
## VIT_01s0010g02940  -  Calmodulin CML37
## VIT_01s0127g00700  -  Unknown protein
## VIT_08s0007g05030  -  myb domain protein 36
## VIT_09s0002g01080  -  Lipoxygenase (VvLOXO)
## VIT_18s0166g00190  -  U-box domain-containing protein
## VIT_01s0010g02970  -  Calcium-binding protein CML
## VIT_16s0100g00380  -  ERF/AP2 Gene Family (VvERF022),Dehydration Responsive Element-Binding
## VIT_04s0023g02380  -  R protein MLA10
## VIT_11s0016g00660  -  ERF/AP2 Gene Family (VvERF009),Dehydration Responsive Element-Binding
## VIT_18s0041g02060  -  12-oxophytodienoate reductase 2
```

```
## VIT_05s0020g04390  -  Ca2+-ATPase 13 ACA13, plasma membrane
## VIT_18s0001g06030  -  Erg-1
## VIT_14s0108g01000  -  Calcium-binding EF hand
## VIT_07s0005g01760  -  Glycerol-3-phosphate acyltransferase 3 (AtGPAT3)
## VIT_08s0007g08750  -  Heat shock transcription factor B3
## VIT_02s0154g00010  -  Auxin-responsive SAUR11
## VIT_05s0049g00140  -  Unknown protein
## VIT_07s0031g00720  -  Ethylene-responsive transcription factor ERF109
## VIT_18s0001g04660  -  No hit
## VIT_15s0024g00400  -  R protein MLA10
## VIT_12s0034g00130  -  Anthocyanidin 3-O-glucosyltransferase
## VIT_01s0010g02930  -  Calmodulin
## VIT_03s0132g00080  -  ATHVA22A (Arabidopsis thaliana HVA22 homologue A)
## VIT_08s0105g00190  -  U-box domain-containing protein
## VIT_17s0000g02050  -  Nudix hydrolase 17
## VIT_15s0048g02300  -  NAC domain-containing protein (VvNAC53)
## VIT_06s0009g00370  -  U-box domain-containing protein
## VIT_05s0049g01070  -  Glutathione S-transferase 19 GSTU1
## VIT_04s0008g05760  -  WRKY Transcription Factor (VvWRKY08)
## VIT_02s0025g03300  -  Potassium channel tetramerisation domain-containing protein
## VIT_10s0003g05330  -  Ethylene-regulated nuclear protein ERN1
## VIT_15s0048g02320  -  NAC domain-containing protein (VvNAC51)
## VIT_15s0048g01390  -  Gibberellin receptor GID1L3
## VIT_14s0066g02350  -  Galactinol synthase
## VIT_01s0011g04550  -  Unknown protein
## VIT_13s0019g01980  -  Aspartic Protease (VvAP32)
## VIT_01s0011g06140  -  Avr9/Cf-9 rapidly elicited protein
## VIT_02s0025g00350  -  Heavy-metal-associated domain-containing protein
## VIT_02s0012g00690  -  No hit
## VIT_05s0077g01600  -  s8_Pathogenesis protein 10 [Vitis vinifera]
## VIT_14s0066g02610  -  Unknown
## VIT_19s0015g01440  -  Unknown protein
## VIT_01s0011g01350  -  No hit
## VIT_06s0004g05090  -  Zinc finger (C3HC4-type ring finger)
## VIT_16s0013g01080  -  ERF/AP2 Gene Family (VvERF086)
## VIT_19s0140g00230  -  No hit
## VIT_01s0011g04630  -  Ribose 5-phosphate isomerase
## VIT_06s0009g03540  -  No hit
## VIT_17s0000g05360  -  Germin
## VIT_05s0020g03240  -  S-receptor kinase
## VIT_01s0011g05580  -  Atypical receptor kinase MARK
## VIT_10s0003g00480  -  Isoflavone methyltransferase/ Orcinol O-methyltransferase 1 oomt1
## VIT_09s0002g00220  -  Avr9/Cf-9 rapidly elicited protein 132
## VIT_19s0014g04650  -  Avr9/Cf-9 rapidly elicited protein 20
## VIT_08s0058g00990  -  Peroxidase
## VIT_11s0016g01860  -  RPS2 (resistant to p. syringae 2)
## VIT_14s0006g00180  -  Aspartyl protease
## VIT_08s0007g07390  -  Unknown protein
## VIT_08s0007g00310  -  Protein phosphatase 2C POLTERGEIST-like 4
## VIT_16s0098g01000  -  No hit
## VIT_04s0008g05510  -  SYNC1 protein, related
## VIT_11s0052g00450  -  WRKY Transcription Factor (VvWRKY34)
## VIT_01s0137g00050  -  myb family
## VIT_01s0010g02950  -  Calcium-binding protein CML
## VIT_06s0004g07770  -  Peroxidase
```

```
## VIT_18s0001g03410  -  R protein L6
## VIT_00s0265g00110  -  Lectin-receptor like protein kinase 3
## VIT_14s0083g00920  -  No hit
## VIT_04s0008g03190  -  F-box family protein (FBX13)
## VIT_17s0000g07730  -  Armadillo/beta-catenin repeat / U-box domain-containing protein
## VIT_16s0050g01970  -  Serine/threonine protein kinase LRK19
## VIT_07s0031g01130  -  Ascorbate oxidase
## VIT_04s0008g02230  -  ERF/AP2 Gene Family (VvERF011),Dehydration Responsive Element-Binding
## VIT_19s0014g04080  -  Serine/threonine-protein kinase receptor ARK3
## VIT_18s0041g01290  -  Serine/threonine-protein kinase CCR4
## VIT_17s0000g01630  -  Calmodulin CML37
## VIT_18s0001g06180  -  Phosphate-induced protein 1
## VIT_17s0000g03160  -  No hit
## VIT_15s0046g01140  -  WRKY Transcription Factor (VvWRKY47)
## VIT_01s0011g03500  -  Homocysteine S-methyltransferase 1
## VIT_17s0000g09710  -  Leucine-rich repeat transmembrane protein kinase
## VIT_00s0262g00010  -  Receptor kinase RK20-1
## VIT_06s0080g01090  -  CCR4-NOT transcription complex subunit 7/8
## VIT_11s0016g00670  -  ERF/AP2 Gene Family (VvERF010),Dehydration Responsive Element-Binding
## VIT_08s0105g00180  -  U-box domain-containing protein
## VIT_02s0025g01280  -  WRKY Transcription Factor (VvWRKY05)
## VIT_05s0020g04930  -  RPM1 (resistance to p. syringae pv maculicola 1)
## VIT_13s0019g03100  -  Cis-zeatin O-beta-D-glucosyltransferase
## VIT_13s0067g00260  -  Nematode-resistance protein
## VIT_11s0016g03350  -  Dehydration Responsive Element-Binding Transcription Factor (VvDREB16)
## VIT_04s0008g00440  -  Clavata1 receptor kinase (CLV1)
## VIT_17s0000g03560  -  (myb domain protein 62
## VIT_16s0013g01000  -  ERF/AP2 Gene Family (VvERF084)
## VIT_08s0007g01420  -  Glutathione S-transferase 8 GSTU8
## VIT_14s0006g00450  -  myb domain protein 20
## VIT_16s0013g01050  -  ERF/AP2 Gene Family (VvERF107)
## VIT_16s0013g00990  -  ERF/AP2 Gene Family (VvERF105)
## VIT_07s0005g01320  -  Unknown
## VIT_03s0017g01990  -  UDP-glucose glucosyltransferase
## VIT_05s0020g04940  -  NBS-LRR type disease resistance protein
## VIT_12s0055g01280  -  Brassinosteroid insensitive 1-associated receptor kinase 1
## VIT_16s0013g00980  -  ERF/AP2 Gene Family (VvERF082)
## VIT_14s0083g01220  -  feronia receptor-like kinase
## VIT_16s0013g01070  -  ERF/AP2 Gene Family (VvERF085)
## VIT_04s0023g00580  -  Auxin-responsive SAUR32
## VIT_16s0013g00950  -  ERF/AP2 Gene Family (VvERF080)
## VIT_16s0013g00970  -  Ethylene responsive element binding factor 5
## VIT_12s0057g01430  -  Heavy-metal-associated domain-containing protein
## VIT_18s0001g15120  -  Unknown protein
## VIT_13s0067g02710  -  Unknown protein
## VIT_19s0090g00820  -  MAPKKK15
```

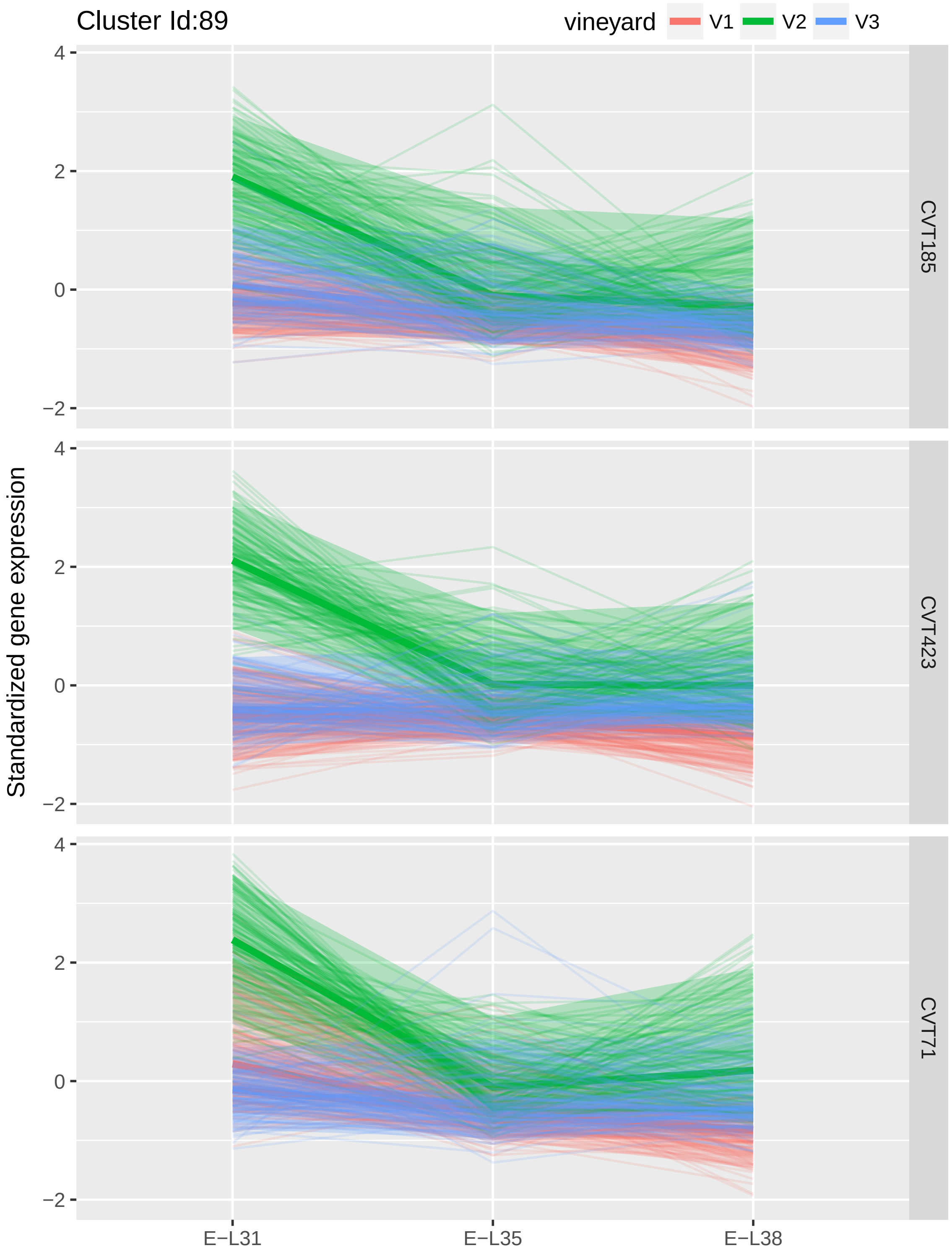
Cluster Id:89
vineyard
V1
V2
V3
Standardized gene expression
CVT185
CVT423
CVT71
4
2
0
−2
E−L31
E−L35
E−L38

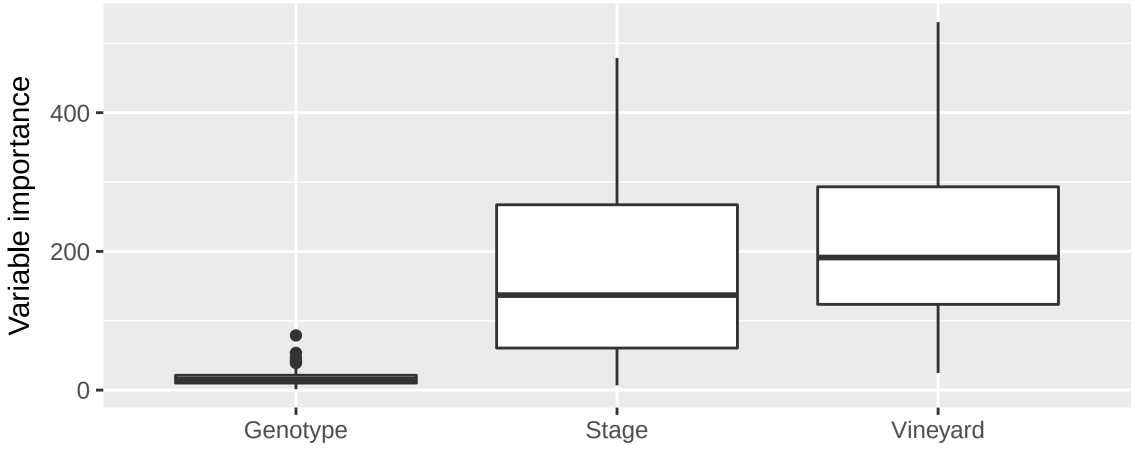
Variable importance
400
200
0
Genotype
Stage
Vineyard

Cluster no. 90

```
## Number of genes in the cluster: 166
## Homogeneity Index:    0.78
## Variable importance for Stage:    Median =  454.6  - Rank =  45
## Variable importance for Clone:    Median =  19.56  - Rank =  53
## Variable importance for Vineyard: Median =  30.45  - Rank =  67
##
## Gene ID               Gene Annotation
## VIT_11s0016g02510  -  Unknown protein
## VIT_02s0087g00690  -  Adapter-related protein complex 4 epsilon 1 sub
## VIT_16s0098g01630  -  Importin beta-2
## VIT_01s0011g04500  -  Oxidoreductase NAD-binding domain
## VIT_11s0016g02840  -  Adaptor-related protein complex 4, sigma 1 sub
## VIT_00s0340g00070  -  UDP-N-acetylglucosamine transferase subunit alg13
## VIT_07s0191g00140  -  Signal transducer
## VIT_05s0094g00070  -  RabGAP/TBC domain-containing protein
## VIT_09s0054g00440  -  TIFY gene family (VvZML2)
## VIT_18s0072g00090  -  No hit
## VIT_08s0056g00830  -  Presenilin
## VIT_15s0046g01630  -  Rhamnogalacturonan xylosyltransferase 1
## VIT_11s0016g04980  -  Zinc finger (CCCH-type) family protein
## VIT_12s0034g02240  -  Potassium channel AKT2/3
## VIT_16s0050g02570  -  Protein phosphatase 2C
## VIT_13s0064g00970  -  F-type H+-transporting ATPase oligomycin sensitivity conferral
## VIT_12s0035g00850  -  Embryogenesis-associated protein
## VIT_00s0265g00130  -  Unknown protein
## VIT_14s0068g00770  -  FAR1-related sequence 5
## VIT_04s0023g03230  -  Auxin-responsive SAUR9
## VIT_09s0002g05260  -  Microtubule-severing ATPase
## VIT_08s0056g01670  -  BTB-POZ and math Domain 4 ATBPM4
## VIT_14s0068g00890  -  Mitochondrial substrate carrier family protein
## VIT_10s0003g00190  -  DNA-binding protein
## VIT_19s0014g00450  -  PMR5 (powdery mildew resistant 5)
## VIT_11s0052g00710  -  Unknown protein
## VIT_13s0067g03040  -  Unknown
## VIT_07s0005g04430  -  No hit
## VIT_18s0001g12570  -  DNA-3-methyladenine glycosylase 1
## VIT_03s0038g03110  -  ATSLY1
## VIT_08s0040g01730  -  EIN2 (ethylene insensitive 2)
## VIT_08s0040g01260  -  Unknown protein
## VIT_04s0008g05300  -  Phosphate translocator
## VIT_08s0007g05690  -  lycopene ß-cyclase (LBCY1) (VvLBCY2)
## VIT_03s0038g01790  -  basic helix-loop-helix (bHLH) family
## VIT_04s0023g02040  -  Endoplasmatic reticulum retrieval protein 1B
## VIT_10s0003g02840  -  Purine permease 10 PUP10
## VIT_11s0016g02040  -  Ribosomal protein S16
## VIT_13s0158g00140  -  SEC31 homolog B
## VIT_19s0027g00490  -  DNA topoisomerase 2-associated protein PAT1
## VIT_04s0008g06860  -  Ubiquitin-specific protease 22 UBP22
## VIT_19s0014g02840  -  3-deoxy-manno-octulosonate cytidylyltransferase
## VIT_18s0001g03820  -  Phosphatidylinositol-4-phosphate 5-kinase 1
## VIT_11s0016g04370  -  Syntaxin 5
## VIT_02s0025g00820  -  Cation/hydrogen exchanger (CHX18)
## VIT_01s0011g05750  -  Zinc finger (C3HC4-type ring finger)
## VIT_07s0005g01120  -  FAR1-related sequence 11
```

```
## VIT_03s0110g00120  -  Dynamin-like protein 6
## VIT_01s0011g05340  -  Unknown protein
## VIT_15s0048g00540  -  Golgi SNARE 12 protein
## VIT_14s0108g00010  -  Isochorismate synthase 1, chloroplast precursor
## VIT_01s0011g05590  -  Erwinia induced protein 2
## VIT_01s0026g00310  -  Adaptor protein complex AP-1, gamma 1 subunit
## VIT_14s0083g01000  -  Zinc finger (C3HC4-type ring finger)
## VIT_15s0048g01050  -  Unknown
## VIT_15s0046g01900  -  ferric reduction oxidase 2
## VIT_18s0001g13280  -  RanBPM
## VIT_14s0066g01620  -  Hypoxia-responsive
## VIT_18s0001g15090  -  RAB GTPase RAB18
## VIT_02s0025g01570  -  Dynamin-like protein 2b
## VIT_06s0004g04410  -  SEC14 cytosolic factor
## VIT_12s0035g01740  -  Dynamin-like protein 2b
## VIT_19s0014g00860  -  Zinc finger (FYVE type)
## VIT_14s0066g01130  -  Universal stress protein A
## VIT_14s0108g00810  -  Mini zinc finger 2 MIF2
## VIT_19s0015g01850  -  CLC-F (chloride channel F)
## VIT_02s0025g03960  -  TET10 (tetraspanin10)
## VIT_19s0014g04540  -  S-locus protein kinase
## VIT_14s0066g00290  -  RGLG2 (ring Domain LIGASE2)
## VIT_16s0039g02010  -  Vacuolar sorting-associated protein (Vps27)
## VIT_03s0038g01310  -  Auxin responsive SAUR protein
## VIT_02s0025g00100  -  Adaptor-related protein complex 2, beta 1 subunit
## VIT_05s0124g00560  -  UDP-galactose transporter 6
## VIT_19s0014g00320  -  Transposon protein
## VIT_01s0011g01300  -  Polygalacturonase QRT3
## VIT_14s0171g00280  -  Unknown protein
## VIT_08s0007g06770  -  Unknown protein
## VIT_15s0021g01020  -  Casein kinase II subunit beta-2
## VIT_14s0081g00320  -  IFA-binding protein
## VIT_04s0008g01200  -  Eukaryotic translation initiation factor SUI1
## VIT_12s0034g01790  -  Phosphatidylinositol-4-phosphate 5-kinase 8
## VIT_17s0000g07920  -  Hypoxia-responsive
## VIT_08s0007g04220  -  SER/ARG-rich protein kinase 4 SRPK4
## VIT_01s0011g06280  -  Oxidoreductase
## VIT_17s0000g02510  -  Golgin candidate 2
## VIT_08s0040g01250  -  CRK (CDPK-related kinase) CRK1
## VIT_11s0052g00460  -  Cation exchanger (CAX10)
## VIT_00s1397g00010  -  Katanin p80 (WD repeat containing) subunit B 1
## VIT_13s0156g00080  -  Lactose permease
## VIT_18s0001g08720  -  CW7 protein
## VIT_08s0040g00050  -  Unknown protein
## VIT_01s0011g05250  -  Senescence-associated protein
## VIT_19s0090g01130  -  Calcium-binding EF hand
## VIT_14s0108g00140  -  Ubiquitin-conjugating enzyme E2 A
## VIT_04s0023g03300  -  2OG-Fe(II) oxygenase
## VIT_07s0151g00970  -  F-box protein
## VIT_19s0090g01720  -  WRKY Transcription Factor (VvWRKY56)
## VIT_09s0002g02470  -  Protein phosphatase 2 regulatory subunit B
## VIT_08s0007g06510  -  Protein phosphatase 2C
## VIT_04s0079g00440  -  Unknown protein
## VIT_08s0007g01030  -  Aldo/keto reductase
## VIT_06s0009g01020  -  FK506-binding protein 4/5 TWD1 (twisted dwarf 1)
```

```
## VIT_06s0004g02120  -  Oxoglutarate/malate translocator
## VIT_08s0007g02830  -  Unknown protein
## VIT_18s0001g06200  -  Phosphotyrosyl phosphatase activator (PTPA)
## VIT_00s0370g00020  -  Clathrin, light polypeptide B
## VIT_10s0071g00950  -  Serine/threonine protein phosphatase 2A
## VIT_08s0007g07570  -  CCR4-NOT transcription complex subunit 3
## VIT_18s0117g00150  -  Heterogeneous nuclear ribonucleoprotein A1/A3
## VIT_06s0004g05800  -  Phosphatidic acid phosphatase / PAP2
## VIT_01s0010g02610  -  Unknown protein
## VIT_05s0077g00620  -  O-acetyltransferase
## VIT_03s0063g02260  -  Phosphatidylinositol 4-kinase
## VIT_19s0015g00770  -  Protein kinase G11A
## VIT_13s0019g02410  -  Serine/threonine protein phosphatase PP1
## VIT_15s0048g02030  -  Zinc finger (C3HC4-type ring finger)
## VIT_10s0003g02770  -  Pterin-4-alpha-carbinolamine dehydratase
## VIT_11s0016g03660  -  Glucose transporter 2 plastidic
## VIT_07s0151g00290  -  Unknown protein
## VIT_16s0039g00700  -  RCP1 (root cap 1)
## VIT_17s0000g08570  -  S-ribonuclease binding protein SBP1
## VIT_07s0255g00100  -  Extra-large G-protein (XLG1)
## VIT_14s0036g00730  -  Protein phosphatase 2 (formerly 2A), regulatory subunit B''
## VIT_06s0004g04570  -  Actin beta/gamma 1
## VIT_11s0118g00300  -  ARP2/3 complex 21 kDa subunit
## VIT_02s0012g01680  -  Trehalose phosphatase 5
## VIT_01s0010g03900  -  putative MADS-box sepallata 3 (VviSEP3)
## VIT_04s0023g01270  -  Transporter-related
## VIT_14s0030g00700  -  Transport protein particle (TRAPP) component Bet3
## VIT_17s0000g07150  -  Dentin sialophosphoprotein
## VIT_11s0118g00440  -  Adenylate cyclase
## VIT_08s0040g02130  -  Syntaxin of plants SYP7
## VIT_08s0040g01430  -  Unknown protein
## VIT_17s0000g09090  -  Armadillo/beta-catenin repeat
## VIT_00s0225g00220  -  Adaptor-related protein complex 1, mu 1 subunit
## VIT_00s1525g00010  -  GAE3 (UDP-D- glcucuronate  4-epimerase 3)
## VIT_12s0028g02070  -  Cupin, RmlC-type
## VIT_17s0000g08230  -  Unknown protein
## VIT_01s0011g03920  -  Carrier protein, Mitochondrial
## VIT_06s0004g06020  -  Alpha-glucan phosphorylase, H isozyme
## VIT_13s0067g03320  -  Tonneau 1b TON1B
## VIT_18s0001g08060  -  Unknown
## VIT_04s0008g01030  -  Unknown protein
## VIT_18s0001g08210  -  SUT4 (sucrose transporter 4) (SUT2-1)
## VIT_11s0016g04710  -  Serine-threonine kinase receptor-associated protein STRAP
## VIT_02s0012g02670  -  Unknown protein
## VIT_02s0033g00080  -  Pleckstriny (PH) domain-containing protein
## VIT_15s0048g02590  -  Vacuolar protein sorting 8 (VPS8)
## VIT_00s0776g00020  -  Katanin p80 (WD repeat containing) subunit B 1
## VIT_03s0038g02760  -  V-type H+-transporting ATPase subunit B
## VIT_15s0048g00460  -  Unknown protein
## VIT_10s0003g04360  -  DDB1A (UV-damaged DNA-binding protein 1A)
## VIT_02s0012g00170  -  1,4-alpha-D-glucan maltohydrolase
## VIT_02s0033g01310  -  Amino acid permease
## VIT_04s0023g03250  -  Kinectin
## VIT_04s0008g05920  -  Golgi SNARE 11 protein
```

```
## VIT_03s0038g04010  -  Pantothenate kinase
## VIT_04s0023g03830  -  HECT ubiquitin-protein ligase 3 (KAKTUS protein)
## VIT_13s0019g00420  -  F-box family protein
## VIT_19s0015g00670  -  E3 ubiquitin-protein ligase HUWE1
## VIT_11s0016g03650  -  CDKF;1 (CDK-activating kinase 1A
## VIT_18s0001g12760  -  Proteasome 20S alpha subunit F2 (PAF2) (PRC2B) (PRS1)
## VIT_11s0016g01180  -  F-box/LRR-repeat protein 20
## VIT_05s0020g02240  -  Unknown protein
## VIT_14s0068g01280  -  V-type H+-transporting ATPase subunit C
## VIT_05s0077g00140  -  Transducin protein
```

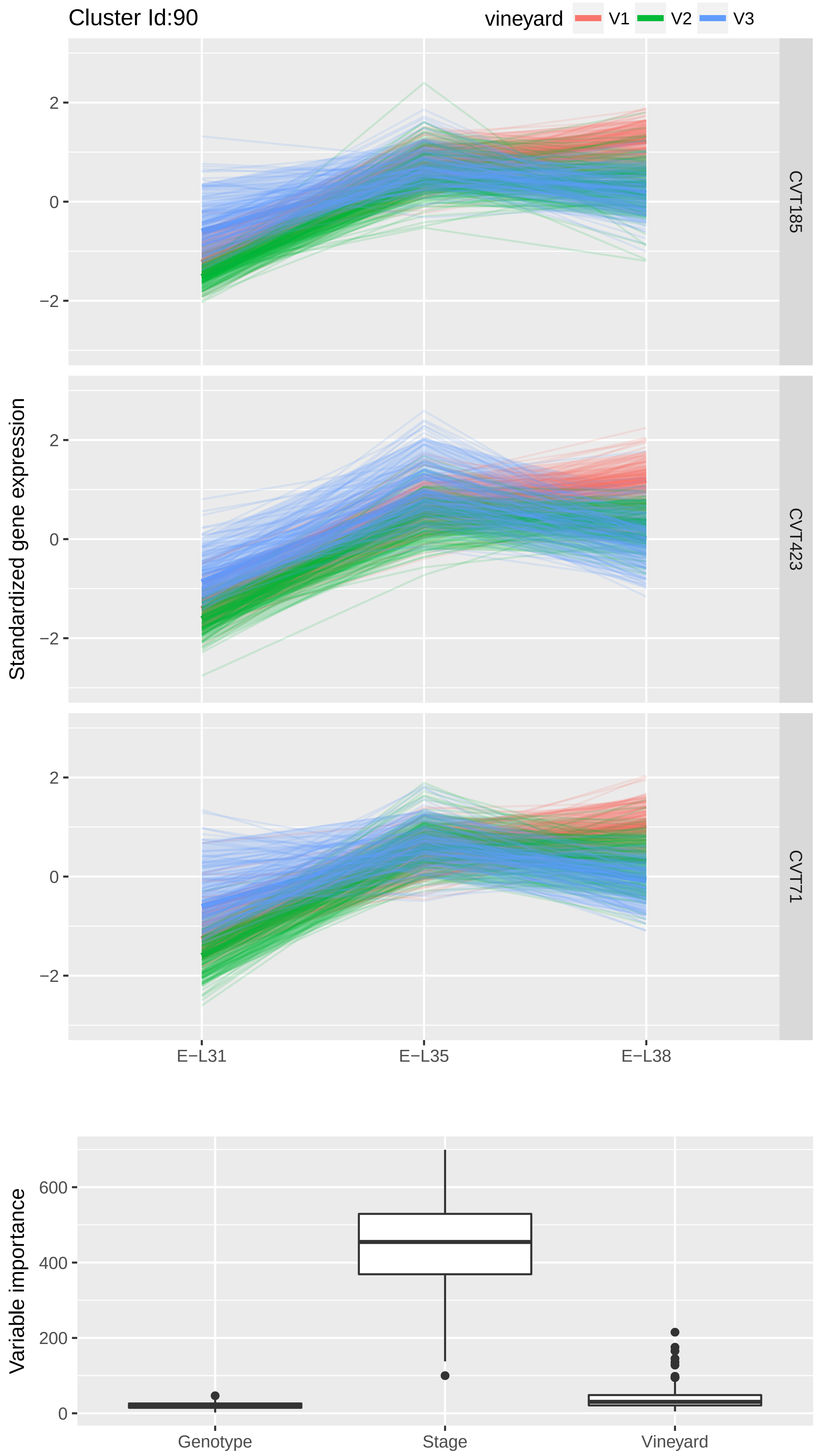

Cluster Id:90
vineyard
V1
V2
V3
Standardized gene expression
CVT185
CVT423
CVT71
2
0
−2
E−L31
E−L35
E−L38
Variable importance
600
400
200
0
Genotype
Stage
Vineyard

Cluster no. 91

```
## Number of genes in the cluster: 40
## Homogeneity Index:    0.56
## Variable importance for Stage:    Median =  105.8  - Rank =  106
## Variable importance for Clone:    Median =  47.19  - Rank =  2
## Variable importance for Vineyard: Median =  79.36  - Rank =  23
##
## Gene ID               Gene Annotation
## VIT_03s0017g01460  -  No hit
## VIT_06s0004g03620  -  Mitogen-activated Protein Kinase (VvMPK7)
## VIT_08s0007g02360  -  Harpin inducing protein 1-like 9
## VIT_17s0000g07670  -  Betaine-aldehyde dehydrogenase
## VIT_01s0010g03930  -  WRKY Transcription Factor (VvWRKY03)
## VIT_06s0004g03330  -  Lateral organ boundaries protein 11
## VIT_06s0009g03670  -  F-box family protein
## VIT_15s0046g02700  -  Acyl-CoA thioesterase
## VIT_02s0025g00480  -  Serine/threonine-protein phosphatase PP1
## VIT_00s0615g00020  -  Cinnamyl alcohol dehydrogenase
## VIT_18s0001g04640  -  No hit
## VIT_01s0010g03640  -  DnaJ homolog, subfamily A, member 3
## VIT_05s0102g00210  -  Unknown protein
## VIT_00s0226g00100  -  Regulator of chromosome condensation (RCC1)
## VIT_10s0116g00960  -  Transcription factor jumonji (jmjC) domain-containing protein
## VIT_19s0014g04940  -  Chitin-inducible gibberellin-responsive protein 1
## VIT_02s0241g00020  -  Unknown
## VIT_14s0006g02610  -  Unknown protein
## VIT_04s0023g00290  -  ABC Transporter (VvNAP5 - VvABCI5)
## VIT_05s0062g00270  -  UDP-glucose:flavonoid 7-O-glucosyltransferase
## VIT_09s0002g00410  -  No hit
## VIT_08s0040g03040  -  Glutathione S-transferase GSTO1
## VIT_06s0061g01560  -  PINOID
## VIT_18s0001g11080  -  RNA-binding protein 47C' ATRBP47C'
## VIT_09s0002g05980  -  CYP734A7 castasterone 26-hydroxylase
## VIT_10s0003g05640  -  UDP-arabinose 4-epimerase 3
## VIT_19s0015g00380  -  Cystathionine beta-synthase
## VIT_19s0090g01530  -  Pirin
## VIT_07s0005g01130  -  No hit
## VIT_17s0000g00200  -  Ethylene-responsive transcription factor ERF114
## VIT_08s0058g01120  -  Unknown
## VIT_09s0002g00420  -  Senescence-associated protein
## VIT_01s0137g00220  -  ALB4 (ALBINA 4)
## VIT_17s0000g06710  -  Leucine-rich repeat transmembrane protein kinase
## VIT_05s0020g04280  -  Ca2+-ATPase 13 ACA13, plasma membrane
## VIT_10s0003g00150  -  NSL1 (necrotic spotted lesions 1)
## VIT_08s0007g08590  -  Pentatricopeptide (PPR) repeat-containing protein
## VIT_05s0062g00310  -  UDP-glucoronosyl/UDP-glucosyl transferase UGT75C1
## VIT_14s0006g01610  -  PMI2 (plastid movement impaired 2)
## VIT_14s0128g00390  -  Mitochondrial FAD carrier
```

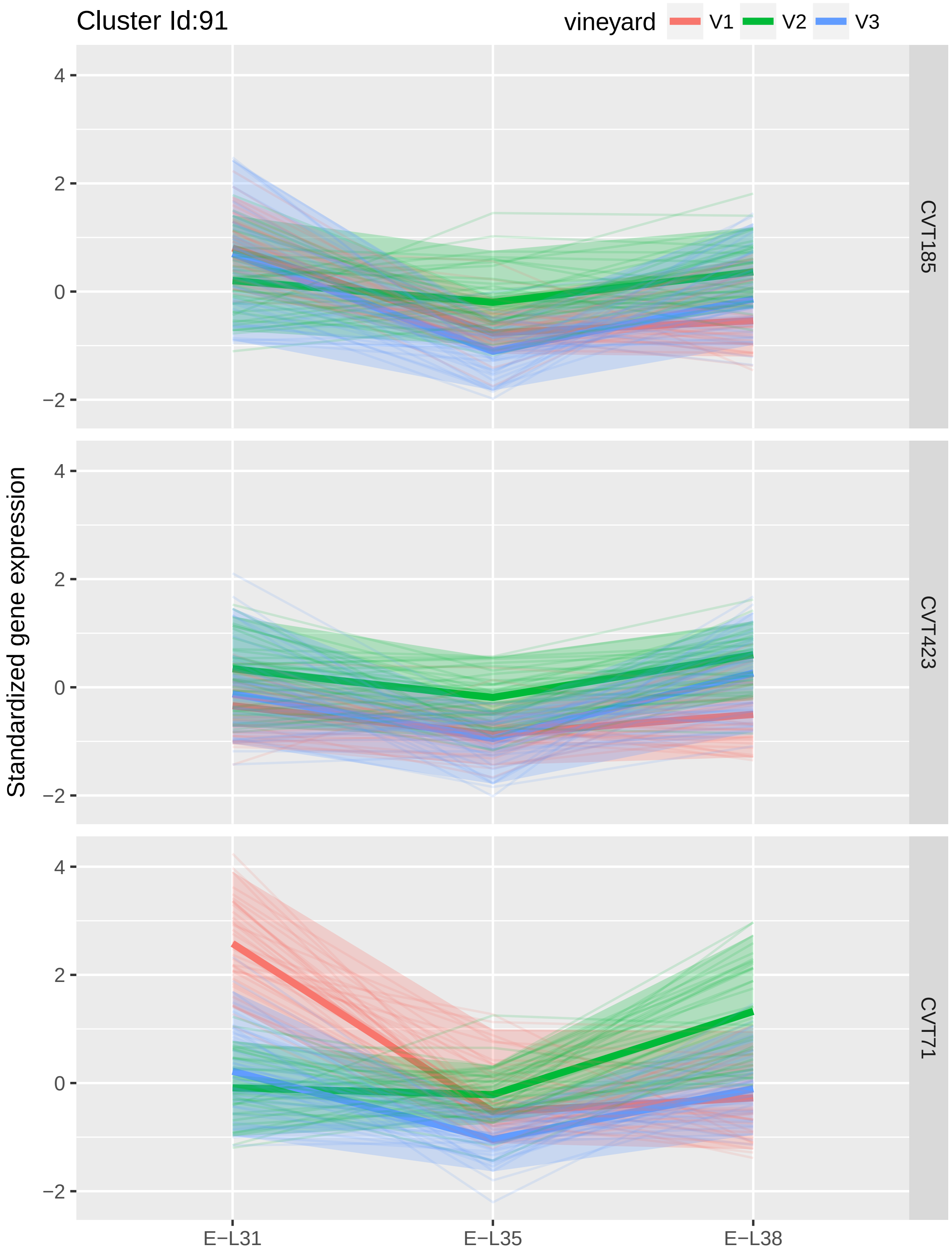
Cluster Id:91
vineyard
V1
V2
V3
Standardized gene expression
CVT185
CVT423
CVT71
4
2
0
−2
E−L31
E−L35
E−L38

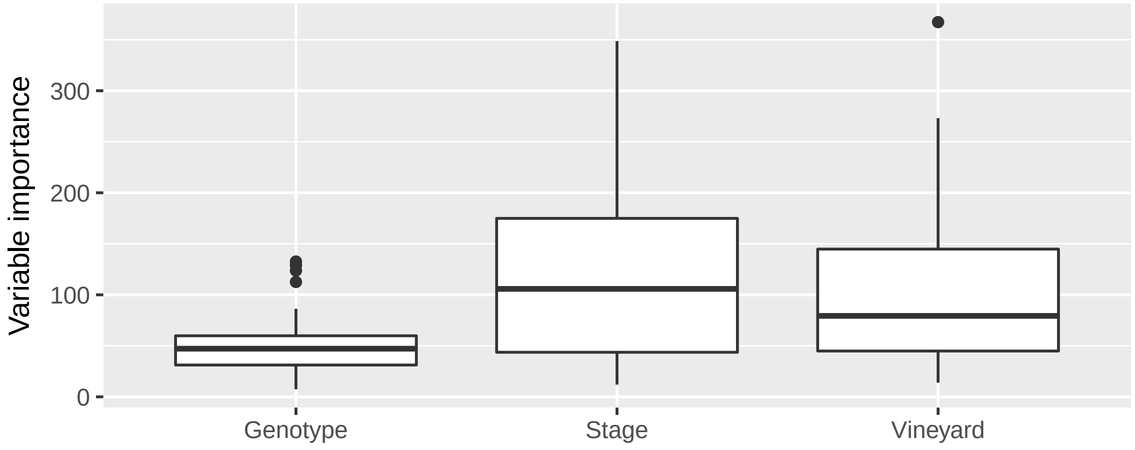
Variable importance
300
200
100
0
Genotype
Stage
Vineyard

Cluster no. 92

```
## Number of genes in the cluster: 130
## Homogeneity Index:    0.69
## Variable importance for Stage:    Median =  327.9  - Rank =  67
## Variable importance for Clone:    Median =   23.6  - Rank =  30
## Variable importance for Vineyard: Median =  49.58  - Rank =  40
##
## Gene ID               Gene Annotation
## VIT_12s0035g00010  -  DNA (cytosine-5)-methyltransferase (ATHIM)
## VIT_12s0059g02310  -  PDF2 (protodermal factor2)
## VIT_08s0058g00860  -  Unknown protein
## VIT_11s0052g00350  -  Receptor kinase TRKe
## VIT_12s0034g02560  -  DNA (cytosine-5)-methyltransferase (ATHIM)
## VIT_18s0001g15220  -  Unknown protein
## VIT_03s0063g00140  -  Caffeoyl-CoA O-methyltransferase (CCoAOMT2)
## VIT_18s0001g15130  -  WD40 repeat protein
## VIT_07s0031g02540  -  Emb2170 (embryo defective 2170)
## VIT_01s0011g04700  -  Heavy-metal-associated domain-containing protein
## VIT_01s0010g02770  -  Adenylate kinase-b
## VIT_08s0040g01520  -  Indole-3-acetate beta-glucosyltransferase
## VIT_00s0640g00020  -  Cig3
## VIT_15s0046g02380  -  CYP86A8
## VIT_12s0055g00160  -  UDP-glycosyltransferase 71E1
## VIT_13s0175g00120  -  ABA-responsive element-binding protein 3 (AREB3) (VvABF-3), Basic Leuc
## VIT_12s0034g02570  -  Leucine Rich Repeat receptor-like kinase
## VIT_14s0006g02910  -  Unknown protein
## VIT_18s0122g00480  -  U2 snRNP auxiliary factor small subunit
## VIT_00s0527g00010  -  Cig3
## VIT_14s0083g00460  -  Tryptophan synthase beta chain 2
## VIT_04s0044g00160  -  Wuschel-related homeobox 13
## VIT_07s0129g00930  -  Unknown protein
## VIT_04s0044g00980  -  Ribosomal protein L17
## VIT_05s0020g02770  -  Unknown protein
## VIT_07s0031g00740  -  Centrin
## VIT_05s0102g00080  -  U1 small nuclear ribonucleoprotein 70 kDa,
## VIT_12s0057g01450  -  Subtilisin protease
## VIT_13s0019g01490  -  C2 domain-containing protein
## VIT_17s0000g04250  -  HAT transposase
## VIT_18s0001g06670  -  Ring-H2 finger protein ATL1N
## VIT_16s0022g00450  -  Unknown protein
## VIT_19s0014g03490  -  No hit
## VIT_05s0102g00330  -  On-specific serine/threonine protein kinase
## VIT_11s0016g04600  -  myb family
## VIT_15s0048g01670  -  CYP704G7
## VIT_14s0060g01690  -  fumarase
## VIT_06s0004g00170  -  Antitermination NusB domain-containing
## VIT_09s0070g00240  -  Cinnamoyl-CoA reductase
## VIT_14s0068g00430  -  Dentin sialophosphoprotein
## VIT_18s0001g13150  -  BEL1 homeotic protein 3
## VIT_08s0007g02260  -  Carboxylic ester hydrolase
## VIT_04s0069g01060  -  DNA-binding protein PD3, chloroplast
## VIT_04s0008g03160  -  G protein protein gamma subunit (AGG2)
## VIT_07s0031g00340  -  F-box domain containing protein
## VIT_15s0046g01390  -  Ethylene-responsive transcription factor cytokinin response factor 4
## VIT_10s0116g00430  -  Unknown protein
```

```
## VIT_06s0004g02360  -  Myosin-related
## VIT_04s0023g01020  -  BEL1 homeobox 2 protein (BLH2) (SAWTOOTH 1)
## VIT_15s0048g00030  -  Unknown protein
## VIT_19s0014g00810  -  RKF1 (receptor-like kinase in flowers 1)
## VIT_10s0003g04030  -  Zinc-binding protein
## VIT_10s0003g00080  -  Ribosomal protein L11 methyltransferase
## VIT_09s0002g04510  -  Unknown protein
## VIT_01s0150g00550  -  Histone-lysine N-methyltransferase ASHR2
## VIT_04s0069g01070  -  DNA-binding protein PD3, chloroplast
## VIT_16s0098g01250  -  Metal-nicotianamine transporter YSL3
## VIT_11s0037g00410  -  Zinc finger (C2H2 type) family
## VIT_12s0028g02680  -  Unknown protein
## VIT_17s0000g03830  -  Protease inhibitor/seed storage/lipid transfer protein (LTP)
## VIT_18s0001g04140  -  Vacuolar protein sorting 9 (VPS9)
## VIT_18s0001g09420  -  Progesterone 5-beta-reductase
## VIT_11s0052g00880  -  NADPH-cytochrome P450 oxydoreductase isoform 3
## VIT_10s0597g00030  -  Monothiol glutaredoxin-S17 (AtGrxS17)
## VIT_13s0175g00200  -  V-type H+-transporting ATPase subunit I
## VIT_19s0014g01630  -  Isopentenyltransferase 9
## VIT_07s0031g01500  -  Unknown protein
## VIT_02s0012g00380  -  Norcoclaurine synthase
## VIT_02s0025g01340  -  Abscisic acid receptor PYL8  RCAR3
## VIT_15s0048g00020  -  Thaumatin SE39b
## VIT_03s0038g00360  -  Isochorismatase hydrolase
## VIT_05s0102g00650  -  Peptidyl-prolyl isomerase E (cyclophilin E)
## VIT_16s0050g01910  -  Unknown
## VIT_14s0030g00740  -  ACP2 (Acyl carrier protein 2)
## VIT_09s0018g01880  -  RNA-binding protein
## VIT_02s0025g04030  -  Methyl-CpG-binding domain 13 MBD13
## VIT_19s0085g00550  -  Ser/Thr protein kinase
## VIT_00s0324g00040  -  Molecular chaperone DnaJ
## VIT_01s0026g02280  -  Binding
## VIT_08s0007g07370  -  Adenylyl-sulfate kinase, chloroplast precursor
## VIT_10s0071g00750  -  Protein arginine N-methyltransferase
## VIT_11s0037g00400  -  Zinc finger (C2H2 type) family
## VIT_05s0051g00180  -  Glutathione S-transferase 8 GSTU8
## VIT_08s0040g02670  -  Lumen common protein , Chloroplast
## VIT_07s0129g00130  -  Unknown
## VIT_06s0004g01790  -  Zf A20 and AN1 domain-containing stress-associated protein 5
## VIT_09s0018g01890  -  No hit
## VIT_05s0020g00210  -  No hit
## VIT_00s0227g00160  -  Iron-stress related protein
## VIT_19s0014g00620  -  Receptor kinase 2
## VIT_14s0066g02630  -  Polyribonucleotide 5'-hydroxyl-kinase
## VIT_14s0060g01680  -  TPR3 (topless-related 3)
## VIT_19s0014g03500  -  Unknown protein
## VIT_13s0019g01410  -  OBP3 (OBF-binding protein 3)
## VIT_03s0063g02700  -  CPL4 (C-Terminal Domain Phosphatase-like 4)
## VIT_11s0118g00450  -  No hit
## VIT_05s0077g01210  -  Dihydrolipoyl dehydrogenase
## VIT_14s0030g01720  -  DNA-damage-repair/toleration protein (DRT102)
## VIT_16s0050g01010  -  ATP binding protein
## VIT_19s0027g01150  -  Phenylalanine-tRNA synthetase
## VIT_12s0028g03370  -  Tryptophan-tRNA ligase
## VIT_13s0019g04630  -  Unknown protein
```

```
## VIT_07s0129g00050  -  Unknown protein
## VIT_13s0067g02550  -  Transcription factor related
## VIT_14s0060g01700  -  fumarase
## VIT_16s0098g01610  -  Unknown
## VIT_19s0027g00750  -  DNA-binding HORMA
## VIT_13s0101g00510  -  Ribonuclease H2 subunit A
## VIT_12s0034g01650  -  Glutathione S-transferase Z2 GSTZ2
## VIT_01s0011g05490  -  F-box domain containing protein
## VIT_17s0000g04000  -  Actin-related protein 4
## VIT_06s0061g01330  -  Ribosomal protein L24 60S
## VIT_05s0020g00200  -  No hit
## VIT_05s0020g04840  -  H1flk, putative
## VIT_07s0005g04680  -  ABC transporter C member 9
## VIT_14s0066g01400  -  MLK/Raf-related protein kinase 1
## VIT_14s0128g00260  -  Unknown
## VIT_00s1247g00020  -  Ribosomal protein L25
## VIT_14s0036g01520  -  MEE49 (maternal effect embryo arrest 49)
## VIT_02s0012g00290  -  S-ribonuclease binding protein SBP1
## VIT_04s0008g05460  -  Unknown protein
## VIT_13s0073g00620  -  MAM33 family
## VIT_15s0021g01230  -  Glutaminyl-tRNA synthetase
## VIT_05s0020g00080  -  Unknown
## VIT_14s0068g00870  -  Nudix hydrolase 13
## VIT_05s0020g04820  -  H1flk
## VIT_04s0008g02120  -  EMB2758 (embryo defective 2758)
## VIT_07s0197g00010  -  Ribosomal protein L25
## VIT_17s0000g01440  -  Unknown protein
## VIT_04s0210g00030  -  Strictosidine synthase
```

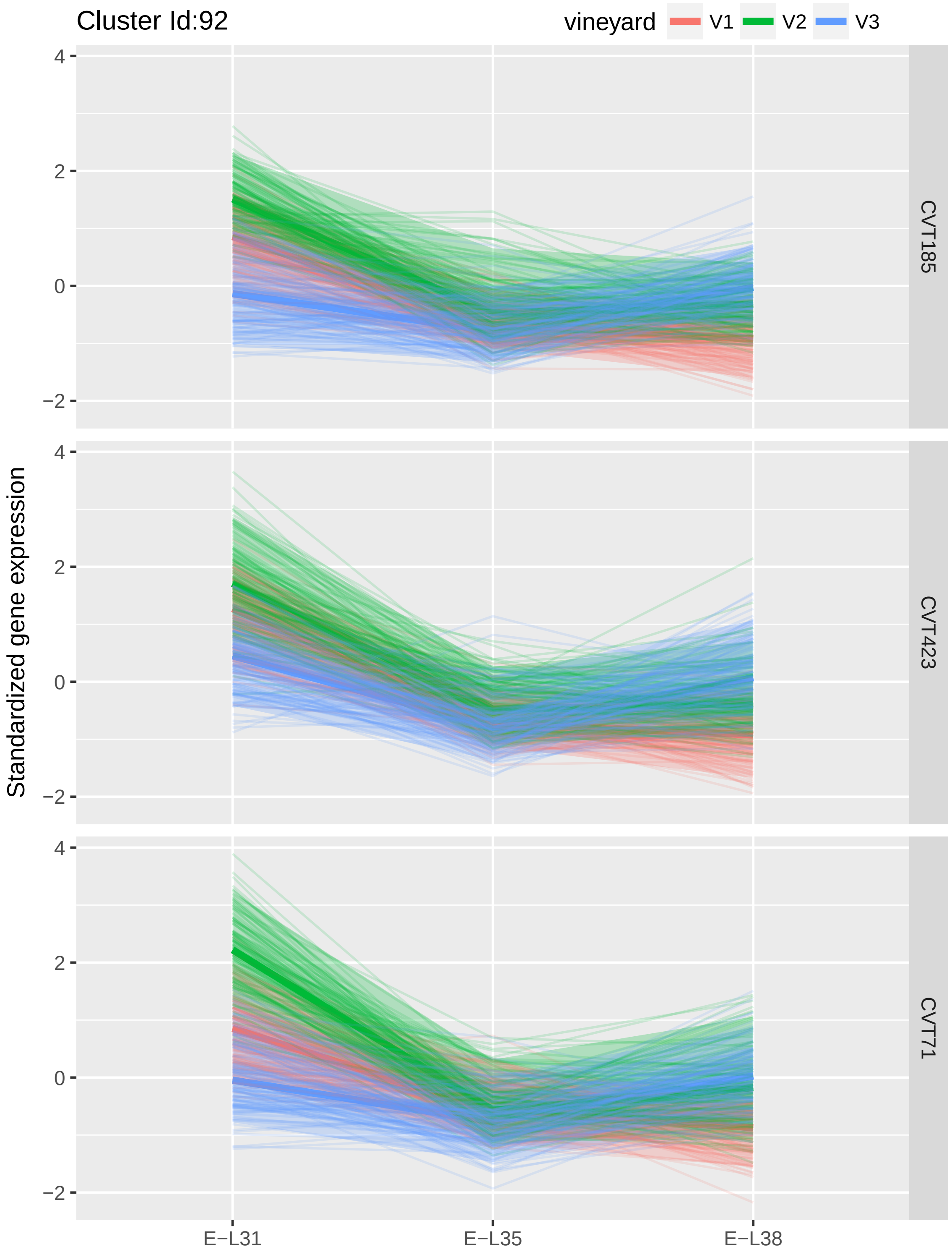

Cluster Id:92
vineyard
V1
V2
V3
Standardized gene expression
CVT185
CVT423
CVT71
4
2
0
−2
E−L31
E−L35
E−L38

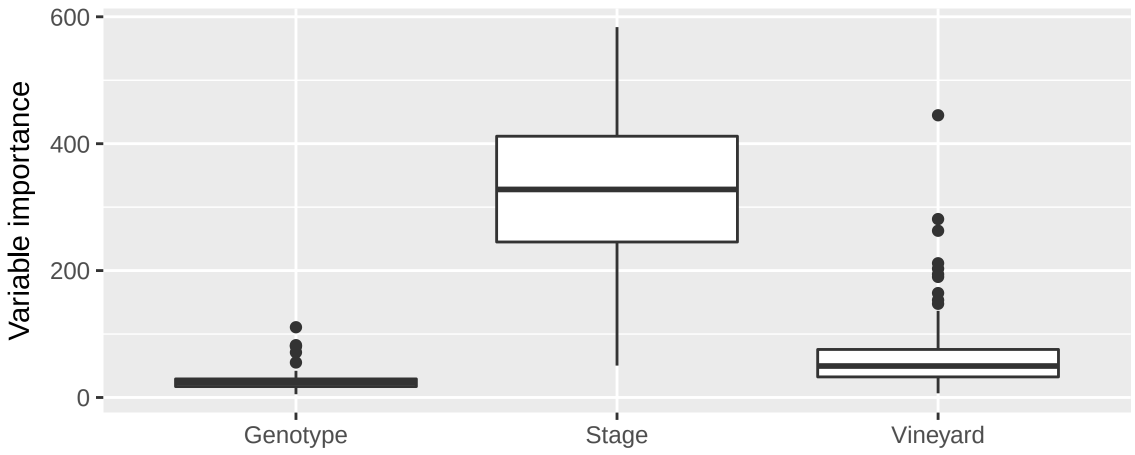

Variable importance
600
400
200
0
Genotype
Stage
Vineyard

Cluster no. 93

```
## Number of genes in the cluster: 62
## Homogeneity Index:    0.54
## Variable importance for Stage:    Median =  206.9  - Rank =  88
## Variable importance for Clone:    Median =  31.49  - Rank =  7
## Variable importance for Vineyard: Median =  61.12  - Rank =  34
##
## Gene ID                Gene Annotation
## VIT_01s0011g01900  -  Glutathione S-transferase, C-terminal
## VIT_09s0054g00980  -  Zinc finger (C3HC4-type ring finger)
## VIT_01s0026g01700  -  Unknown protein
## VIT_05s0020g01250  -  BTB/POZ domain-containing protein
## VIT_09s0002g06090  -  Mov34 family protein
## VIT_14s0128g00430  -  Protein kinase NPK1 (ANP1)
## VIT_16s0022g02120  -  Metalloendopeptidase OMA1
## VIT_05s0020g01790  -  Triacylglycerol lipase.
## VIT_03s0038g03620  -  Rho guanyl-nucleotide exchange factor ROPGEF1
## VIT_08s0040g01410  -  Unknown protein
## VIT_01s0026g02650  -  KH domain-containing protein
## VIT_18s0089g01220  -  Cupin super
## VIT_04s0023g03430  -  basic helix-loop-helix (bHLH) family
## VIT_01s0010g03400  -  Exostosin
## VIT_06s0004g02870  -  Unknown protein
## VIT_10s0116g00830  -  Patatin
## VIT_14s0066g00450  -  Sucrase
## VIT_07s0031g01650  -  Programmed cell death 2 C-terminal domain-containing protein
## VIT_04s0044g00100  -  Unknown protein
## VIT_12s0055g00960  -  Unknown
## VIT_13s0019g00350  -  ARF GTPase activator, ARF-GAP Domain 13
## VIT_13s0019g01860  -  Actin-related protein 5
## VIT_12s0028g03630  -  Ubiquitin-conjugating enzyme E2 O
## VIT_02s0012g00180  -  Serine protease
## VIT_08s0056g01620  -  FH protein interacting protein FIP2
## VIT_02s0033g01150  -  Unknown protein
## VIT_06s0080g00310  -  Esterase/lipase/thioesterase family protein
## VIT_17s0000g01060  -  No hit
## VIT_08s0040g02290  -  Armadillo/beta-catenin repeat
## VIT_19s0090g00350  -  Unknown protein
## VIT_08s0007g03300  -  Unknown protein
## VIT_16s0039g02720  -  Glutamate dehydrogenase 2 (GDH2)
## VIT_14s0128g00770  -  F-box protein
## VIT_14s0060g00060  -  EMB1138 (embryo defective 1138)
## VIT_05s0102g00270  -  Cyclin-related
## VIT_02s0025g03270  -  Aminopeptidase N
## VIT_05s0029g00060  -  Myb UNE16 (unfertilized embryo sac 16)
## VIT_06s0004g02030  -  Xanthine dehydrogenase/oxidase
## VIT_06s0004g07760  -  GCN5L1
## VIT_11s0016g01690  -  Unknown protein
## VIT_07s0005g02500  -  SNF7 family protein
## VIT_02s0025g03580  -  DNA-binding family protein
## VIT_06s0004g02280  -  Translocation protein SEC62
## VIT_18s0001g13340  -  Purple acid phosphatase 32- ATPAP32/PAP32
## VIT_14s0060g00770  -  Glycosyl transferase family 8 protein
## VIT_00s0531g00050  -  Acetyl-CoA acetyltransferase, cytosolic 1
## VIT_13s0084g00080  -  MAP65/ASE1; t-snare
```

```
## VIT_06s0004g08310  -  Unknown protein
## VIT_12s0059g01690  -  BZIP transcription factor
## VIT_11s0052g00660  -  Aldose 1-epimerase
## VIT_14s0060g00810  -  Galactinol synthase
## VIT_08s0040g01680  -  Unknown protein
## VIT_11s0016g05050  -  Kelch repeat-containing F-box protein
## VIT_10s0116g00530  -  Thiazole biosynthetic enzyme, chloroplast (ARA6)
## VIT_14s0128g00080  -  Lipase GDSL
## VIT_10s0116g00390  -  Unknown protein
## VIT_12s0055g00810  -  Cationic peroxidase
## VIT_00s0252g00060  -  DnaJ homolog, subfamily C, member 7
## VIT_16s0098g00810  -  Unknown protein
## VIT_05s0020g01080  -  Unknown
## VIT_16s0039g00080  -  Unknown protein
## VIT_01s0010g01080  -  Phosphoribulokinase/uridine kinase
```

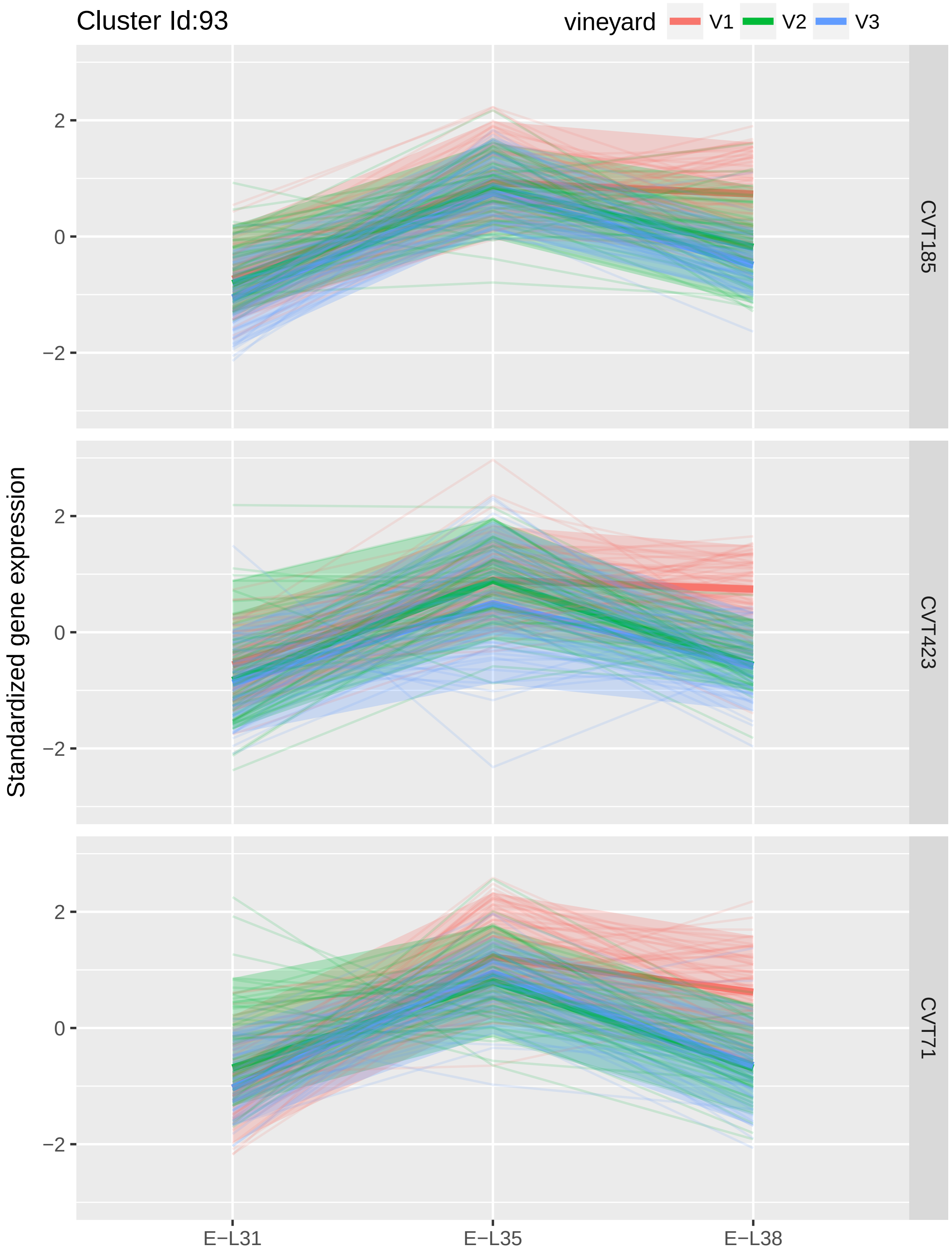

Cluster Id:93
vineyard
V1
V2
V3
Standardized gene expression
CVT185
CVT423
CVT71
2
0
−2
E−L31
E−L35
E−L38

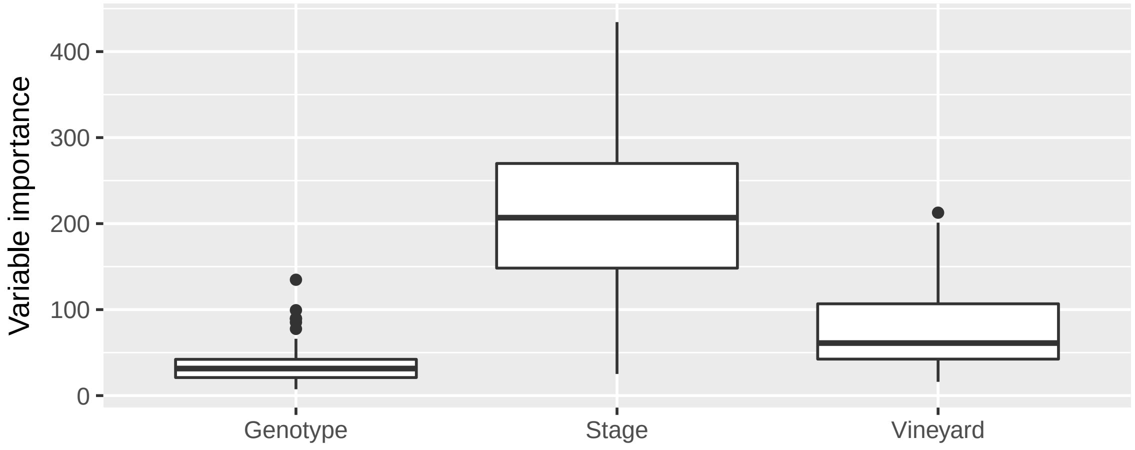

Variable importance
400
300
200
100
0
Genotype
Stage
Vineyard

Cluster no. 94

```
## Number of genes in the cluster: 164
## Homogeneity Index:    0.84
## Variable importance for Stage:    Median =  487.2  - Rank =  37
## Variable importance for Clone:    Median =   14.3  - Rank =  76
## Variable importance for Vineyard: Median =   22.1  - Rank =  83
##
## Gene ID               Gene Annotation
## VIT_09s0002g08260  -  PRLI-interacting factor L
## VIT_00s0323g00030  -  HSP associated protein
## VIT_08s0007g03020  -  Ribosomal protein S14 (RPS14C) 40S
## VIT_04s0008g04930  -  Paramyosin
## VIT_13s0019g01850  -  Transposon protein, putative, CACTA
## VIT_04s0008g05580  -  Unknown protein
## VIT_00s0499g00010  -  RNA recognition motif (RRM)-containing protein
## VIT_04s0044g00890  -  Uridylate kinase
## VIT_01s0010g01810  -  Tetratricopeptide repeat (TPR)-containing
## VIT_05s0077g01740  -  Polygalacturonase GH28
## VIT_08s0007g01400  -  Glutathione S-transferase 8 GSTU8
## VIT_03s0038g02450  -  Constans interacting protein 4
## VIT_19s0015g01750  -  WD-40 repeat
## VIT_10s0092g00090  -  Ribose-5-phosphate isomerase
## VIT_12s0028g01240  -  fanconi anemia, complementation group D2
## VIT_03s0038g03560  -  CAO (CHAOS); chromatin binding
## VIT_02s0025g01250  -  SEH1H (SEH1H)
## VIT_17s0000g06350  -  LHCA4 (Photosystem I light harvesting complex gene 4)
## VIT_14s0083g01130  -  MAP3K-like protein kinase, putative, expressed
## VIT_09s0002g06980  -  Chloroplast post-illumination chlorophyll fluorescence increase protei
## VIT_11s0016g02650  -  Ribosomal protein L31 (RPL31C) 60S
## VIT_04s0008g00950  -  Calcineurin B protein 10
## VIT_07s0185g00040  -  Unknown protein
## VIT_03s0038g00340  -  Salt tolerance homolog2
## VIT_04s0008g00600  -  RNA polymerase sigma factor SIGC
## VIT_06s0004g02270  -  Nuclear transcription factor Y subunit A-1
## VIT_13s0019g04140  -  LHCA1
## VIT_15s0021g00830  -  Chaperonin 10, Chloroplast
## VIT_19s0014g01920  -  Unknown protein
## VIT_19s0093g00150  -  Glutathione S-transferase 25 GSTU25
## VIT_18s0001g08110  -  Glutamyl-tRNA reductase, chloroplast precursor
## VIT_17s0000g02300  -  POT1 (protection of telomeres 1)
## VIT_19s0093g00310  -  Glutathione S-transferase 8 GSTU19
## VIT_00s2739g00010  -  basic helix-loop-helix (bHLH) family
## VIT_14s0060g02420  -  Transcription factor jumonji (jmjC) domain-containing protein
## VIT_07s0031g01270  -  Ring-H2 finger A2A
## VIT_10s0003g02900  -  LHCII type I CAB-1
## VIT_07s0130g00260  -  SET domain-containing protein
## VIT_19s0027g00350  -  Protoporphyrinogen IX oxidase
## VIT_18s0001g11570  -  CYP82C1p
## VIT_12s0028g01140  -  Pentatricopeptide (PPR) repeat-containing protein
## VIT_13s0175g00190  -  V-type H+-transporting ATPase subunit I
## VIT_13s0019g04130  -  AarF domain containing kinase
## VIT_00s1312g00010  -  basic helix-loop-helix (bHLH) family
## VIT_13s0019g03170  -  Heat shock protein 16.9 kDa class I
## VIT_13s0064g01390  -  Ribosomal protein L6 (RPL6C) 60S
## VIT_08s0007g07720  -  CYP93A1 2-hydroxyisoflavanone synthase
```

```
## VIT_18s0075g00270  -  Unknown
## VIT_18s0041g01870  -  4-Phosphopantetheine adenylyltransferase
## VIT_06s0009g01450  -  Salutaridine reductase
## VIT_01s0244g00170  -  Cytokinin-repressed protein CR9
## VIT_01s0011g00730  -  Alpha-1,4-glucan phosphorylase type H
## VIT_03s0038g02480  -  SKP1
## VIT_08s0007g00710  -  Unknown
## VIT_12s0055g01110  -  LHCB6 (light harvesting complex PSII)
## VIT_04s0008g06610  -  3-hydroxyisobutyryl-CoA hydrolase
## VIT_07s0005g03370  -  Unknown
## VIT_14s0108g00390  -  Homeobox-leucine zipper protein HB13
## VIT_17s0000g08860  -  UBX domain-containing protein
## VIT_14s0066g00470  -  Unknown protein
## VIT_02s0025g02210  -  MYB DOMAIN PROTEIN 6
## VIT_12s0028g01230  -  fanconi anemia, complementation group D2
## VIT_09s0002g05820  -  Unknown
## VIT_17s0000g04560  -  Kinetochore protein
## VIT_01s0011g03700  -  Acyl-CoA thioesterase
## VIT_14s0068g00800  -  Syntaxin 7
## VIT_17s0000g01690  -  Argininosuccinate synthase, chloroplast precursor
## VIT_19s0015g02700  -  Glutathione S-transferase 25 GSTU25
## VIT_06s0004g08080  -  Zinc finger (C3HC4-type ring finger)
## VIT_00s0510g00010  -  Unknown protein
## VIT_03s0063g02040  -  Caffeine synthase
## VIT_09s0002g03540  -  MYB divaricata
## VIT_09s0002g08500  -  No hit
## VIT_07s0031g01760  -  Chloroplast lumen common family protein
## VIT_06s0061g01420  -  Unknown
## VIT_05s0020g03900  -  Ribosomal protein S3A (RPS3aA) 40S
## VIT_07s0031g02460  -  Unknown protein
## VIT_11s0016g02910  -  Acid phosphatase class B
## VIT_18s0001g02530  -  DEAD/DEAH box helicase
## VIT_02s0488g00020  -  Palmitoyl-protein thioesterase 1 precursor
## VIT_06s0080g00920  -  Photosystem I subunit O (PSAO)
## VIT_16s0022g01330  -  R protein  PRF  disease resistance protein
## VIT_04s0008g05900  -  ARR12
## VIT_18s0001g12880  -  Jasmonate O-methyltransferase
## VIT_10s0042g00200  -  Thioredoxin X
## VIT_18s0001g06430  -  Homeobox-leucine zipper protein ATHB-6
## VIT_06s0080g00150  -  Subtilisin-like proteinase AIR3
## VIT_19s0014g05050  -  Heat shock  HSP20 family protein
## VIT_09s0002g06780  -  Unknown protein
## VIT_01s0137g00790  -  No hit
## VIT_16s0050g01260  -  Unknown protein
## VIT_17s0000g02220  -  No hit
## VIT_08s0040g01930  -  Unknown protein
## VIT_01s0011g03400  -  Proton-dependent oligopeptide transport (POT) family protein
## VIT_11s0016g00650  -  Chaperonin 21, Chloroplast
## VIT_10s0003g02890  -  LHCII type I CAB-1
## VIT_14s0068g00880  -  Ribosomal protein S27
## VIT_09s0002g03610  -  MYB divaricata
## VIT_03s0063g02240  -  PLATZ transcription factor
## VIT_11s0016g02250  -  Unknown protein
## VIT_06s0004g06780  -  PHD finger-like domain-containing protein 5A
## VIT_07s0104g00400  -  Exonuclease C2H2
```

```
## VIT_08s0040g00800  -  Nodulin MtN21
## VIT_02s0025g00720  -  Transmembrane protein FT27/PFT27
## VIT_04s0023g02840  -  Leucine-rich repeat disease resistance protein
## VIT_19s0014g01560  -  Endonuclease
## VIT_03s0063g00110  -  Cyclin delta-3
## VIT_19s0014g01930  -  Unknown protein
## VIT_09s0002g07160  -  5-hydroxyisobutyryl-coenzyme A hydrolase
## VIT_00s0264g00050  -  Unknown protein
## VIT_14s0066g02640  -  No hit
## VIT_01s0011g05310  -  CRR3 (chlororespiratory reduction 3)
## VIT_01s0011g03690  -  ER6 protein universal stress protein (USP) family
## VIT_03s0038g02090  -  Phospholipase C.
## VIT_07s0005g03520  -  Armadillo/beta-catenin repeat family protein
## VIT_04s0069g01020  -  NBS1 (nijmegen breakage syndrome 1)
## VIT_19s0014g03660  -  LHCII type III, chloroplast precursor
## VIT_19s0014g03250  -  Beta-mannosidase 4
## VIT_05s0020g02130  -  1-deoxy-D-xylulose-5-phosphate synthase, chloroplast precursor (VvDXS)
## VIT_18s0001g10610  -  No hit
## VIT_08s0032g00330  -  Pigment defective 318 (PDE318)
## VIT_05s0077g01670  -  s3_Pathogenesis protein 10 [Vitis vinifera]
## VIT_19s0015g01100  -  FK506-binding protein genes family (VvFKBP16-1)
## VIT_16s0039g02570  -  EMB1270
## VIT_01s0011g05170  -  Major Latex Protein Family
## VIT_08s0007g07270  -  Dicarboxylate/tricarboxylate carrier (DTC)
## VIT_16s0098g01820  -  Unknown protein
## VIT_18s0122g00460  -  CP12-3
## VIT_17s0000g04270  -  Peptidyl-prolyl cis-trans isomerase B
## VIT_01s0026g00670  -  Unknown protein
## VIT_13s0019g04950  -  KOW domain-containing transcription factor family protein
## VIT_06s0061g00510  -  Unknown protein
## VIT_03s0063g00890  -  formin-binding protein
## VIT_13s0067g03470  -  Glutathione S-transferase GSTO1
## VIT_18s0072g00880  -  Exosome complex component RRP45
## VIT_17s0053g00630  -  Rhodanese domain containing protein
## VIT_17s0000g02210  -  Hsp70 interacting protein
## VIT_15s0046g00170  -  VvMybPA1
## VIT_00s0701g00010  -  Somatic embryogenesis receptor kinase 2 SERK2
## VIT_07s0151g01000  -  Photosystem I reaction center subunit II (PSAD)
## VIT_01s0011g05190  -  Bet v I allergen
## VIT_04s0044g01260  -  SEU3B protein
## VIT_05s0102g00310  -  GUN4
## VIT_14s0006g01740  -  AAR2 protein
## VIT_01s0026g00070  -  No hit
## VIT_07s0151g00520  -  Homocysteine S-methyltransferase 3
## VIT_09s0002g00870  -  FLU (fluorescent in blue light)
## VIT_13s0073g00240  -  Glycosyl transferase family 1
## VIT_00s0641g00010  -  Protein serine/threonine kinase
## VIT_08s0007g07480  -  Aldose 1-epimerase
## VIT_12s0142g00010  -  No hit
## VIT_01s0010g02620  -  Unknown protein
## VIT_01s0026g00360  -  Nodulin MtN21 family
## VIT_16s0022g02040  -  PBS2 (PPHB susceptible 2)
## VIT_00s1274g00010  -  Phosphosulfolactate synthase protein
## VIT_18s0001g03150  -  Cation transport protein chaC
## VIT_01s0010g00920  -  Chaperonin GroEL
```

```
## VIT_02s0025g04760  -  Splicing factor YT521-B
## VIT_01s0011g01080  -  ribulose-1,5 bisphosphate carboxylase oxygenase large subunit N-methyl
## VIT_18s0122g00900  -  FK506-binding protein genes family (VvFKBP18)
## VIT_17s0000g06690  -  Unknown
## VIT_07s0031g02720  -  Myosin heavy chain
## VIT_13s0084g00820  -  Aspartic Protease (VvAP33)
## VIT_00s0324g00020  -  Hydrolase, alpha/beta fold
```

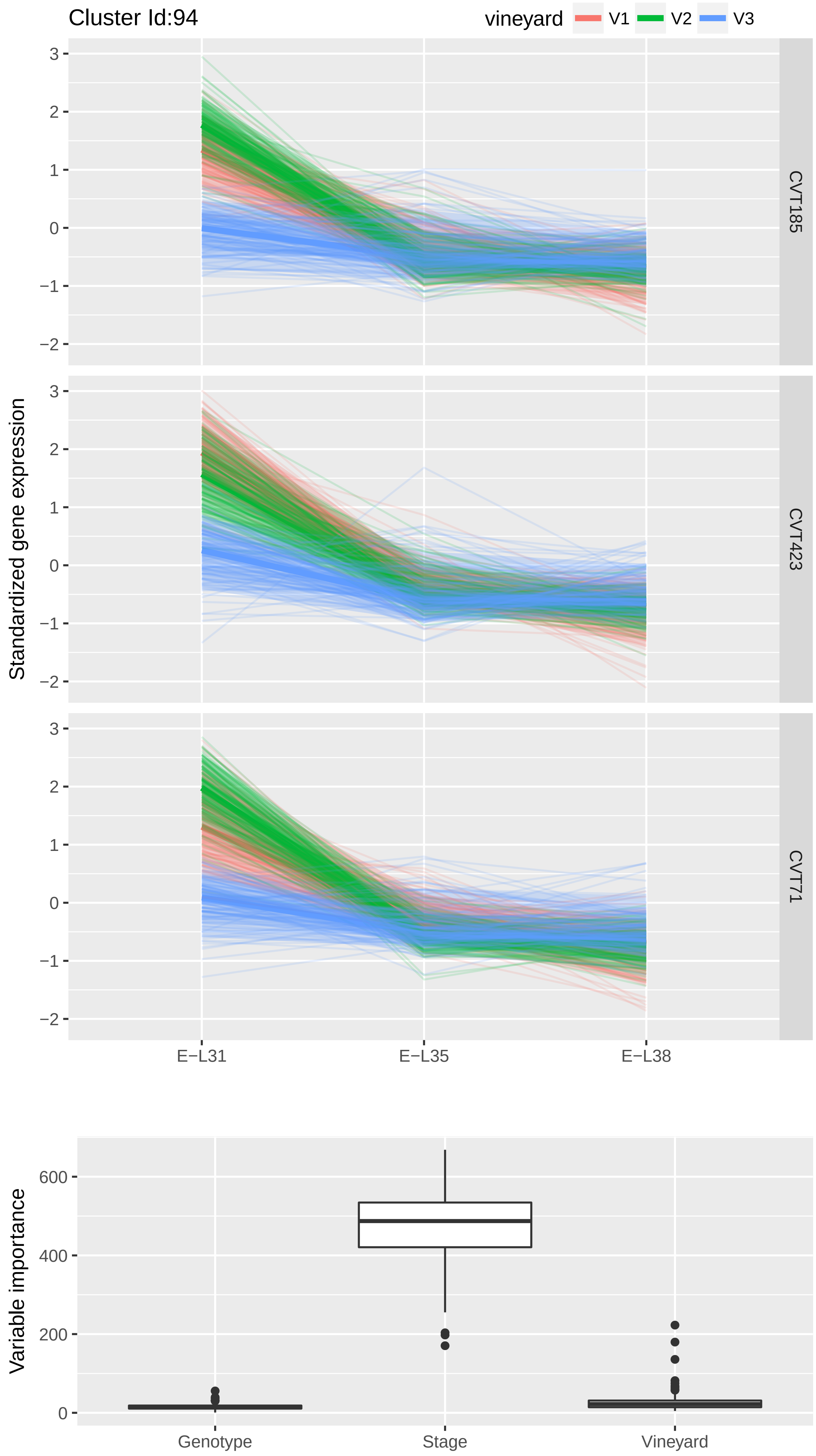
Cluster Id:94
vineyard
V1
V2
V3
Standardized gene expression
CVT185
CVT423
CVT71
3
2
1
0
−1
−2
E−L31
E−L35
E−L38
Variable importance
600
400
200
0
Genotype
Stage
Vineyard

Cluster no. 95

```
## Number of genes in the cluster: 87
## Homogeneity Index:    0.77
## Variable importance for Stage:    Median =    525  - Rank =  27
## Variable importance for Clone:    Median =  10.48  - Rank =  103
## Variable importance for Vineyard: Median =  22.86  - Rank =  81
##
## Gene ID               Gene Annotation
## VIT_08s0040g00240  -  Zinc finger (C2H2 type) family
## VIT_10s0003g00010  -  Ribosomal protein S5
## VIT_00s0838g00020  -  Ribosomal protein S5
## VIT_04s0044g00320  -  Unknown protein
## VIT_06s0004g01370  -  Lipoyltransferase
## VIT_17s0000g09970  -  Endonuclease
## VIT_15s0046g01090  -  Hydroxyproline-rich glycoprotein
## VIT_04s0008g01420  -  HAB2 (Homology to ABI2) (VvPP2C-7)
## VIT_09s0018g01270  -  Ribulose bisphosphate carboxylase/oxygenase activase 2
## VIT_14s0108g01290  -  Unknown protein
## VIT_00s1674g00010  -  4-nitrophenylphosphatase
## VIT_13s0067g03020  -  Unknown protein
## VIT_14s0066g01300  -  Unknown protein
## VIT_05s0049g02140  -  Glutamyl-tRNA(Gln) amidotransferase B
## VIT_01s0011g00270  -  Division regulator MinE, Plastid
## VIT_16s0050g01070  -  MIND (accumulation and replication of chloroplast 11)
## VIT_19s0090g01210  -  FK506-binding protein genes family (VvTIG)
## VIT_14s0066g01110  -  Ribosomal protein L12-1, chloroplast (CL12-A) 50S
## VIT_01s0011g00670  -  SNAP25A protein
## VIT_16s0022g00470  -  Peroxisomal biogenesis factor 11 (PEX11)
## VIT_04s0008g01300  -  Glycine dehydrogenase
## VIT_01s0010g01900  -  Pentatricopeptide (PPR) repeat-containing protein
## VIT_10s0116g00970  -  Leucyl tRNA synthetase
## VIT_01s0011g04730  -  Unknown protein
## VIT_13s0064g00800  -  No hit
## VIT_04s0008g06510  -  Unknown
## VIT_10s0003g03310  -  EMB2756 (embryo defective 2756)
## VIT_14s0068g01980  -  No hit
## VIT_07s0031g02320  -  Leaf senescence protein
## VIT_11s0016g04560  -  Bile acid sodium symporter
## VIT_14s0081g00720  -  basic helix-loop-helix (bHLH) family
## VIT_11s0065g01100  -  Pentatricopeptide (PPR) repeat-containing protein
## VIT_19s0014g00260  -  Protein kinase PKN/PRK1
## VIT_06s0004g00980  -  Dirigent protein pDIR9
## VIT_18s0001g11710  -  Photosystem II PsbO protein
## VIT_13s0067g03370  -  Unknown protein
## VIT_08s0058g00170  -  Nudix hydrolase 15, mitochondrial precursor (AtNUDT15)
## VIT_14s0030g00470  -  Stress regulated protein isoform 3
## VIT_19s0015g01580  -  Phenylalanine-tRNA ligase
## VIT_12s0028g00320  -  LHB1B1
## VIT_14s0006g02290  -  Dehydration Responsive Element-Binding Transcription Factor (VvDREB20)
## VIT_00s0265g00010  -  Phosphoglycerate mutase
## VIT_00s0282g00020  -  K+ efflux antiporter (KEA3)
## VIT_07s0031g02240  -  MLO-like protein 8
## VIT_12s0059g01900  -  Unknown protein
## VIT_15s0045g01340  -  Ribosomal protein L18 50S
## VIT_08s0040g02250  -  No hit
```

```
## VIT_09s0070g00260  -  No hit
## VIT_00s1034g00010  -  E3 ubiquitin-protein ligase CHIP
## VIT_18s0041g00330  -  ATP-dependent protease La (LON) domain-containing protein
## VIT_02s0025g01470  -  Unknown protein
## VIT_17s0000g08680  -  IMP dehydrogenase/GMP reductase
## VIT_18s0001g10510  -  Thioredoxin family
## VIT_07s0129g00770  -  CYP81D2
## VIT_18s0072g00970  -  DegP protease,
## VIT_19s0014g03460  -  Unknown protein
## VIT_04s0023g01910  -  Myb family
## VIT_15s0024g00640  -  SIGB (SIGMA factor B)
## VIT_08s0040g01400  -  Thylakoid lumenal 15.0 kDa protein 2
## VIT_18s0001g15310  -  Thioredoxin M-type
## VIT_14s0060g01030  -  Haloacid dehalogenase hydrolase
## VIT_01s0011g02150  -  Photosystem II stability/assembly factor, chloroplast (HCF136)
## VIT_12s0034g01210  -  PAP/fibrillin family
## VIT_05s0020g02710  -  Slingshot
## VIT_18s0001g15710  -  Electron carrier/ oxidoreductase
## VIT_19s0014g03330  -  Peptidyl-prolyl cis-trans isomerase TLP38, chloroplast
## VIT_05s0020g02410  -  Mo25
## VIT_16s0100g00330  -  Unknown protein
## VIT_09s0002g02280  -  No hit
## VIT_18s0089g01390  -  Unknown protein
## VIT_17s0000g08480  -  ATMYB66/WER/WER1 (WEREWOLF 1)
## VIT_17s0000g02910  -  Unknown protein
## VIT_04s0008g04310  -  Oxygen-evolving complex PsbP
## VIT_12s0059g01830  -  Unknown
## VIT_14s0066g01410  -  Peptidylprolyl isomerase
## VIT_01s0244g00030  -  ACP4 (Acyl carrier protein 4)
## VIT_08s0007g00320  -  GTP binding protein
## VIT_00s0386g00050  -  Xyloglucan endotransglucosylase/hydrolase precursor
## VIT_15s0048g02980  -  Unknown protein
## VIT_00s0533g00020  -  Zeaxanthin epoxidase, chloroplast precursor
## VIT_01s0011g05290  -  Inorganic carbon transport protein
## VIT_03s0038g01830  -  Proline-rich protein 4
## VIT_14s0060g00820  -  3-beta hydroxysteroid dehydrogenase
## VIT_12s0059g02340  -  Syntaxin 1B/2/3/4
## VIT_14s0066g01860  -  Zinc finger protein 5
## VIT_15s0046g01340  -  Unknown protein
## VIT_14s0066g01100  -  Monodehydroascorbate reductase, cytoplasmic isoform 2
```

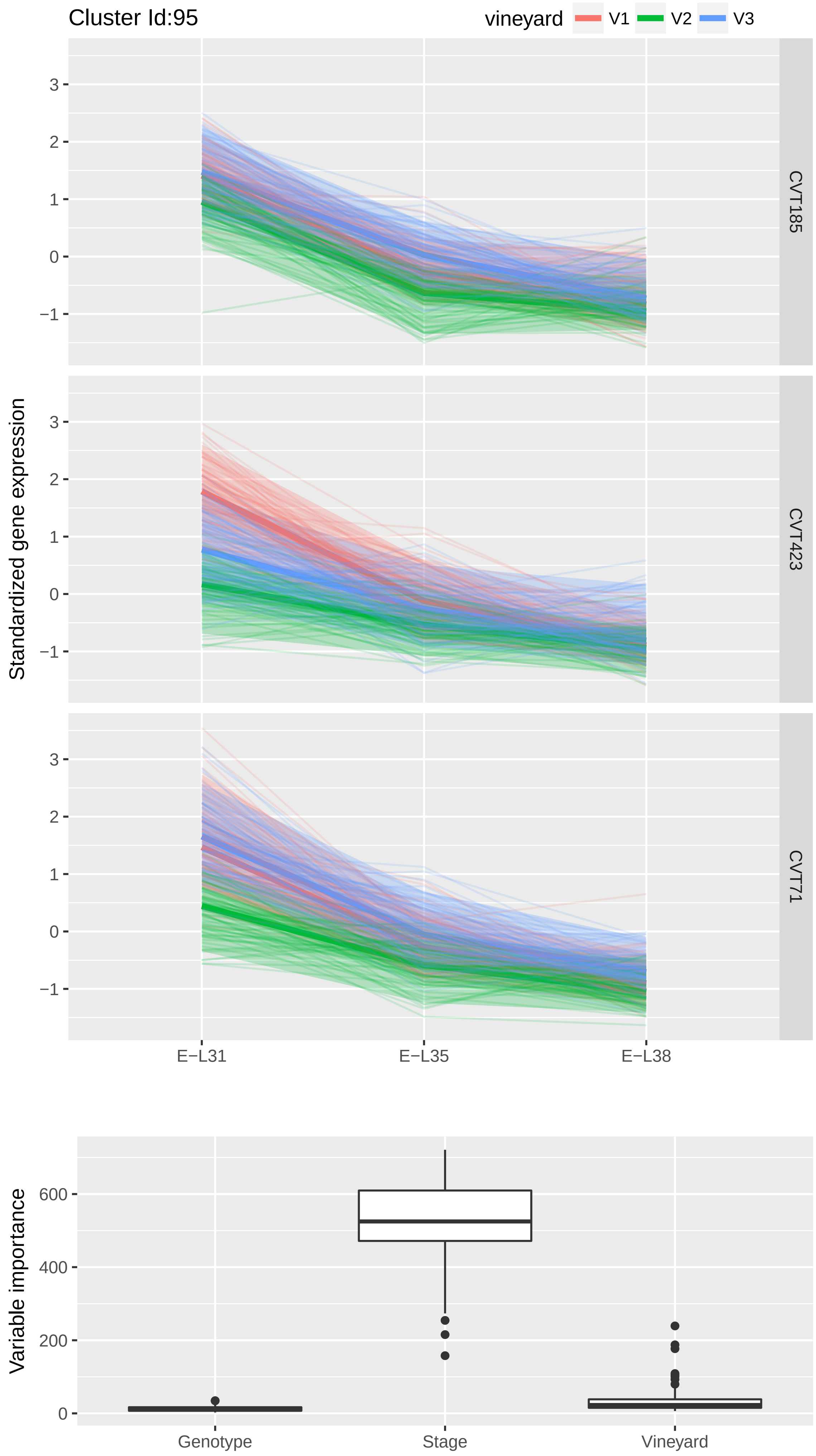

Cluster Id:95
vineyard
V1
V2
V3
Standardized gene expression
CVT185
CVT423
CVT71
E−L31
E−L35
E−L38
Variable importance
Genotype
Stage
Vineyard

Cluster no. 96

```
## Number of genes in the cluster: 98
## Homogeneity Index:    0.57
## Variable importance for Stage:    Median =  137.7  - Rank =  99
## Variable importance for Clone:    Median =  20.08  - Rank =  48
## Variable importance for Vineyard: Median =  168.9  - Rank =  6
##
## Gene ID               Gene Annotation
## VIT_14s0030g00190  -  Unknown protein
## VIT_03s0088g00620  -  Stress-induced
## VIT_08s0007g01450  -  IMP dehydrogenase
## VIT_11s0052g01200  -  Xyloglucan endotransglucosylase/hydrolase 23
## VIT_11s0052g01270  -  Xyloglucan endotransglycosylase 6
## VIT_11s0052g01320  -  Xyloglucan endotransglycosylase 6
## VIT_12s0134g00170  -  No hit
## VIT_05s0020g02390  -  Clathrin-adaptor medium chain apm3
## VIT_04s0008g01220  -  RKL1 (Receptor-like kinase 1)
## VIT_00s0455g00050  -  R protein  PRF  disease resistance protein
## VIT_18s0001g06170  -  Phosphate-induced protein 1
## VIT_14s0108g01150  -  Pentatricopeptide repeat containing protein
## VIT_07s0005g00150  -  Galactosyl transferase GMA12/MNN10
## VIT_05s0049g01750  -  Unknown protein
## VIT_12s0059g01350  -  Unknown protein
## VIT_17s0000g06590  -  Galactosyl transferase GMA12/MNN10
## VIT_14s0006g01080  -  Atypical receptor kinase MARK
## VIT_18s0001g06020  -  Phosphate-induced protein 1
## VIT_12s0057g00560  -  PAT1 (Phytochrome a signal transduction 1)
## VIT_11s0052g01280  -  Xyloglucan endotransglucosylase/hydrolase 23
## VIT_14s0108g00980  -  Cycling DOF factor 1
## VIT_11s0052g01260  -  Xyloglucan endotransglucosylase/hydrolase 23
## VIT_14s0081g00070  -  Zinc finger (C3HC4-type ring finger)
## VIT_01s0026g01030  -  Zinc finger (C3HC4-type ring finger)
## VIT_14s0066g01700  -  Calmodulin binding protein
## VIT_03s0038g03860  -  Phosphate-induced protein 1
## VIT_01s0026g00090  -  Ulp1 protease family
## VIT_04s0079g00410  -  CIR1/RVE2 (circadian 1)
## VIT_01s0010g01200  -  Cell elongation protein / DWARF1 / DIMINUTO (DIM)
## VIT_14s0060g00080  -  Unknown protein
## VIT_08s0007g08020  -  No hit
## VIT_04s0008g01020  -  Unknown protein
## VIT_07s0005g01200  -  Unknown
## VIT_11s0016g05210  -  Acetylglucosaminyltransferase
## VIT_11s0016g03330  -  Unknown protein
## VIT_08s0007g05790  -  Calmodulin
## VIT_12s0057g00530  -  DGCR14
## VIT_18s0001g06110  -  R protein L6
## VIT_19s0090g00370  -  Unknown protein
## VIT_08s0007g01620  -  Lectin
## VIT_14s0066g00460  -  Serine/threonine protein kinase
## VIT_01s0026g02530  -  Beta-1,3-galactosyltransferase
## VIT_16s0013g01430  -  F-box family protein
## VIT_11s0016g02920  -  Unknown protein
## VIT_06s0009g03420  -  C2 domain-containing protein
## VIT_09s0002g02090  -  Unknown protein
## VIT_18s0072g01090  -  TIR-NBS-LRR type R protein 7
```

```
## VIT_01s0150g00020  -  Receptor protein kinase
## VIT_18s0089g00100  -  R protein L6
## VIT_05s0077g00540  -  No hit
## VIT_15s0046g01620  -  Glycosyl transferase family 1 protein
## VIT_08s0058g00140  -  Ammonium transporter 2
## VIT_18s0001g08590  -  No hit
## VIT_00s0265g00060  -  Unknown
## VIT_02s0025g00840  -  Intracellular protein transport protein USO1
## VIT_18s0001g03260  -  R protein L6
## VIT_17s0000g05540  -  H(+)-ATPase 11 AHA11
## VIT_01s0026g00980  -  Glycosyl transferase family 8 protein
## VIT_14s0066g01870  -  Armadillo/beta-catenin repeat / U-box domain-containing protein
## VIT_14s0128g00240  -  Cation exchanger (CAX11)
## VIT_00s0270g00130  -  RKF2 (receptor-like serine/threonine kinase 2)
## VIT_11s0016g05480  -  Clathrin assembly protein 2
## VIT_18s0001g08580  -  Cellulose synthase CSLC12
## VIT_06s0009g02700  -  F-box family protein
## VIT_18s0001g14800  -  Lipase 3 (EXL3) family II extracellular
## VIT_08s0058g01440  -  Ribosomal S6 kinase p90
## VIT_14s0060g00410  -  EDA32 (embryo sac development arrest 32)
## VIT_05s0077g00920  -  MAP3K delta-1 protein kinase
## VIT_18s0001g07350  -  No hit
## VIT_12s0057g01360  -  Unknown protein
## VIT_09s0002g02660  -  Unknown protein
## VIT_05s0062g00860  -  DNA topoisomerase III alpha
## VIT_08s0056g01230  -  Cycling DOF factor 2
## VIT_13s0019g00680  -  Unknown protein
## VIT_06s0004g02950  -  Helicase conserved C-terminal domain containing protein
## VIT_04s0008g06310  -  Octicosapeptide/Phox/Bem1p (PB1) domain-containing protein
## VIT_13s0067g01290  -  Clathrin heavy chain
## VIT_13s0019g01890  -  DVL11/RTFL8 (ROTUNDIFOLIA like 8)
## VIT_14s0083g00330  -  Receptor protein kinase PERK1
## VIT_14s0171g00410  -  Ankyrin repeat
## VIT_00s1927g00010  -  Unknown
## VIT_08s0007g08040  -  No hit
## VIT_11s0118g00700  -  Protein phosphatase 2C DBP
## VIT_02s0025g02250  -  Dof zinc finger protein DOF4.6
## VIT_13s0067g01460  -  Avr9/Cf-9 rapidly elicited protein
## VIT_05s0049g01820  -  Unknown protein
## VIT_09s0002g01890  -  Hydroxyproline-rich glycoprotein
## VIT_01s0137g00700  -  Lipase GDSL
## VIT_17s0000g01830  -  Global transcription factor group E8
## VIT_16s0013g00490  -  MIM (hypersensitive to MMS, irradiation and MMC)
## VIT_03s0038g01940  -  Permease nonimprinted in Prader-Willi/Angelman
## VIT_03s0038g03260  -  Unknown protein
## VIT_06s0061g01140  -  Phototropic-responsive NPH3
## VIT_04s0044g00140  -  Unknown protein
## VIT_15s0048g00340  -  GAE3 (UDP-D- glcucuronate  4-epimerase 3)
## VIT_12s0059g02240  -  Unknown
## VIT_14s0068g01210  -  Sex determination protein tasselseed-2
## VIT_07s0031g02960  -  Permease nonimprinted in Prader-Willi/Angelman
```

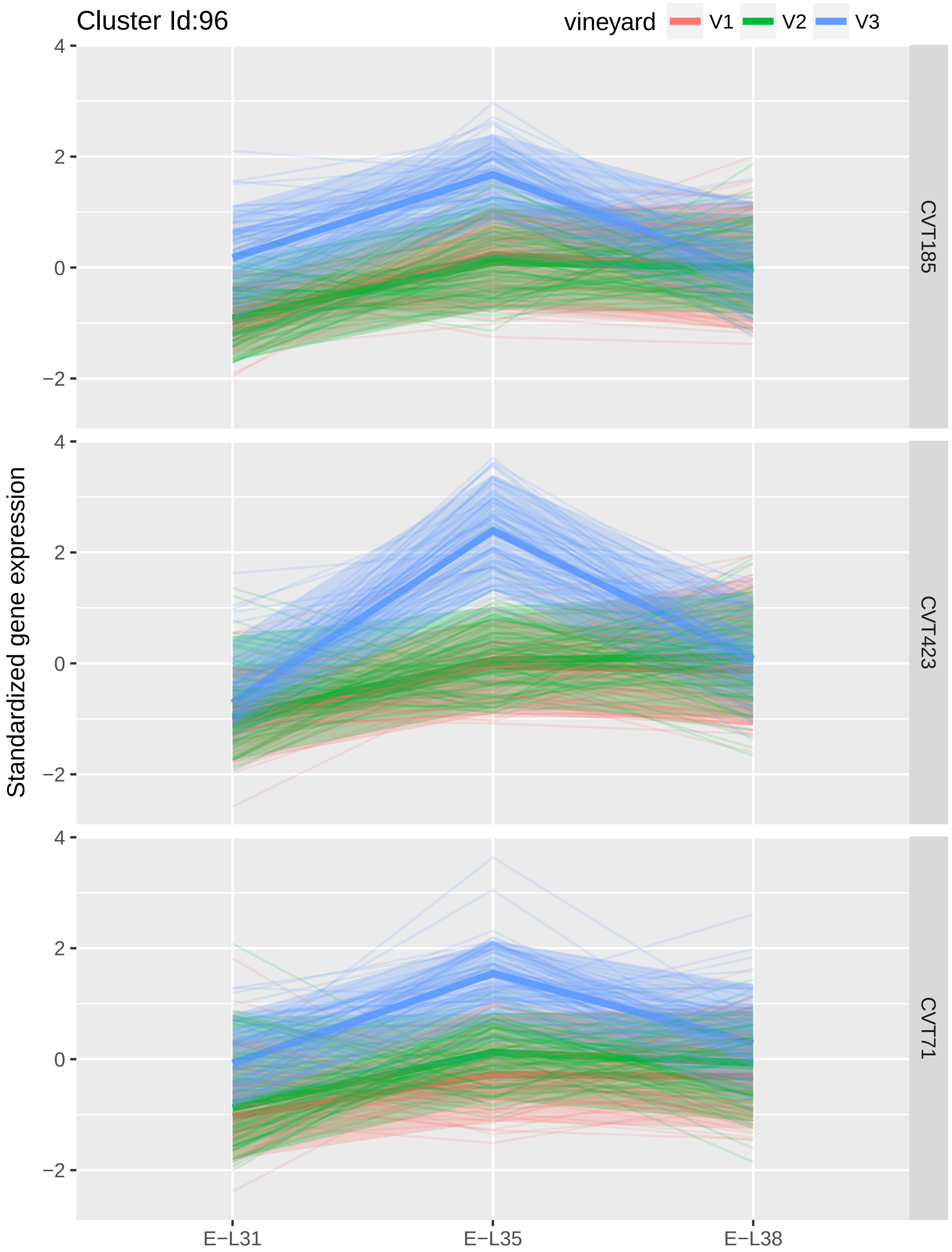
Cluster Id:96
vineyard
V1
V2
V3
Standardized gene expression
4
2
0
−2
CVT185
CVT423
CVT71
E−L31
E−L35
E−L38

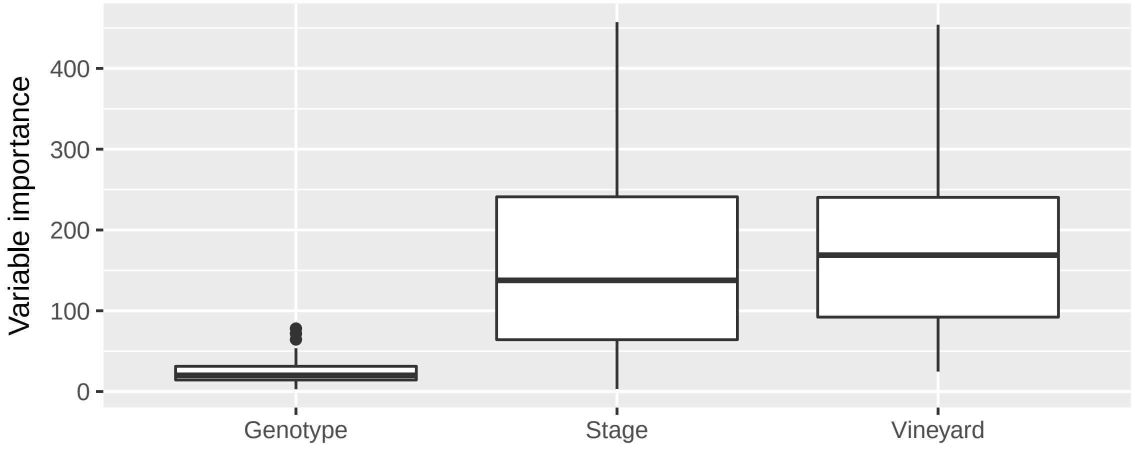
Variable importance
400
300
200
100
0
Genotype
Stage
Vineyard

Cluster no. 97

```
## Number of genes in the cluster: 68
## Homogeneity Index:    0.76
## Variable importance for Stage:    Median =  442.7  - Rank =  51
## Variable importance for Clone:    Median =  19.03  - Rank =  56
## Variable importance for Vineyard: Median =  17.07  - Rank =  93
##
## Gene ID               Gene Annotation
## VIT_07s0104g00310  -  CUE domain containing protein
## VIT_00s0669g00010  -  E3 ubiquitin-protein ligase CHIP
## VIT_17s0000g06550  -  Exonuclease
## VIT_01s0011g03090  -  Allene oxide cyclase (jasmonates from fatty acids)
## VIT_11s0052g01730  -  1-deoxy-D-xylulose-5-phosphate synthase
## VIT_07s0151g00610  -  No hit
## VIT_18s0001g13700  -  Cell growth defect factor -2
## VIT_00s0265g00120  -  EMB2756 (embryo defective 2756)
## VIT_02s0025g01000  -  V-type H+-transporting ATPase subunit E
## VIT_02s0033g01030  -  Anthraniloyal-CoA: methanol anthraniloyal transferase
## VIT_02s0012g01130  -  No hit
## VIT_17s0000g06540  -  Exonuclease
## VIT_16s0098g01240  -  Unknown protein
## VIT_14s0081g00190  -  No hit
## VIT_08s0007g03780  -  Unknown protein
## VIT_17s0000g06530  -  No hit
## VIT_08s0007g01310  -  Proton-dependent oligopeptide transport (POT) family protein
## VIT_15s0048g03000  -  GTPase RABA3
## VIT_01s0011g03760  -  Unknown
## VIT_13s0156g00170  -  Molybdopterin synthase (CNX2)
## VIT_06s0004g01910  -  ADP,ATP carrier protein
## VIT_04s0008g02180  -  BRCT domain-containing protein
## VIT_13s0019g03230  -  Unknown protein
## VIT_19s0014g04220  -  S-locus protein kinase
## VIT_00s0301g00070  -  No hit
## VIT_05s0020g03190  -  Haloacid dehalogenase hydrolase
## VIT_06s0004g06480  -  CYP71E
## VIT_18s0001g14480  -  Thaumatin SCUTL1
## VIT_13s0067g01120  -  Omega-3 fatty acid desaturase, chloroplast, temperature-sensitive (FAD
## VIT_18s0001g06610  -  Plastid-targeted protein 2
## VIT_14s0066g01240  -  L-aspartate oxidase
## VIT_07s0141g00250  -  Ethylene-induced calmodulin-binding protein 1
## VIT_18s0001g04910  -  Sulfate transporter 1.3
## VIT_13s0067g01020  -  Leucoanthocyanidin dioxygenase
## VIT_14s0006g02140  -  Cis-zeatin O-beta-D-glucosyltransferase
## VIT_07s0005g02080  -  No hit
## VIT_09s0002g00960  -  Inter-alpha-trypsin inhibitor heavy chain
## VIT_08s0056g00580  -  Nuclear shuttle  interacting  (ATNSI1)
## VIT_13s0084g00810  -  Armadillo
## VIT_05s0049g01400  -  Alcohol oxidase
## VIT_09s0054g00620  -  Kelch repeat-containing protein
## VIT_06s0080g00090  -  CYP94A1
## VIT_01s0011g03890  -  Receptor serine/threonine kinase PR5K
## VIT_00s0370g00010  -  LRR receptor-like serine/threonine-protein kinase
## VIT_09s0002g00890  -  TIFY gene family (VvJAZ4)
## VIT_07s0031g00040  -  Unknown protein
## VIT_03s0088g00960  -  Unknown protein
```

```
## VIT_08s0007g01320  -  Proton-dependent oligopeptide transport (POT) family protein
## VIT_04s0008g05630  -  Phox
## VIT_12s0059g02710  -  Myrcene synthase (VvTPS32)
## VIT_01s0146g00360  -  Constans-like 14
## VIT_17s0000g09080  -  myb domain protein 4
## VIT_18s0001g10210  -  PAB8 (poly(A) binding protein 8)
## VIT_09s0002g05400  -  ABC Transporter (VvPDR10 - VvABCG40)
## VIT_05s0020g04100  -  Dymeclin
## VIT_04s0023g01880  -  Zinc Finger Homeodomain Transcription Factor (VvZHD3)
## VIT_08s0007g00030  -  Cation/hydrogen exchanger 20 (CHX20)
## VIT_00s0301g00060  -  No hit
## VIT_19s0014g04900  -  (-)-germacrene D synthase (VvTPS27)
## VIT_18s0001g11290  -  Signal peptidase 1
## VIT_14s0066g01460  -  HEAT repeat-containing protein
## VIT_02s0012g00410  -  Naringenin,2-oxoglutarate 3-dioxygenase
## VIT_02s0012g01060  -  ABC Transporter (VvNAP3 - VvABCI3)
## VIT_11s0016g04760  -  Unknown protein
## VIT_01s0011g06050  -  RNA recognition motif (RRM)-containing protein
## VIT_14s0068g02050  -  No hit
## VIT_12s0028g01720  -  Unknown
## VIT_00s0805g00010  -  Ethylene-induced calmodulin-binding protein 1
```

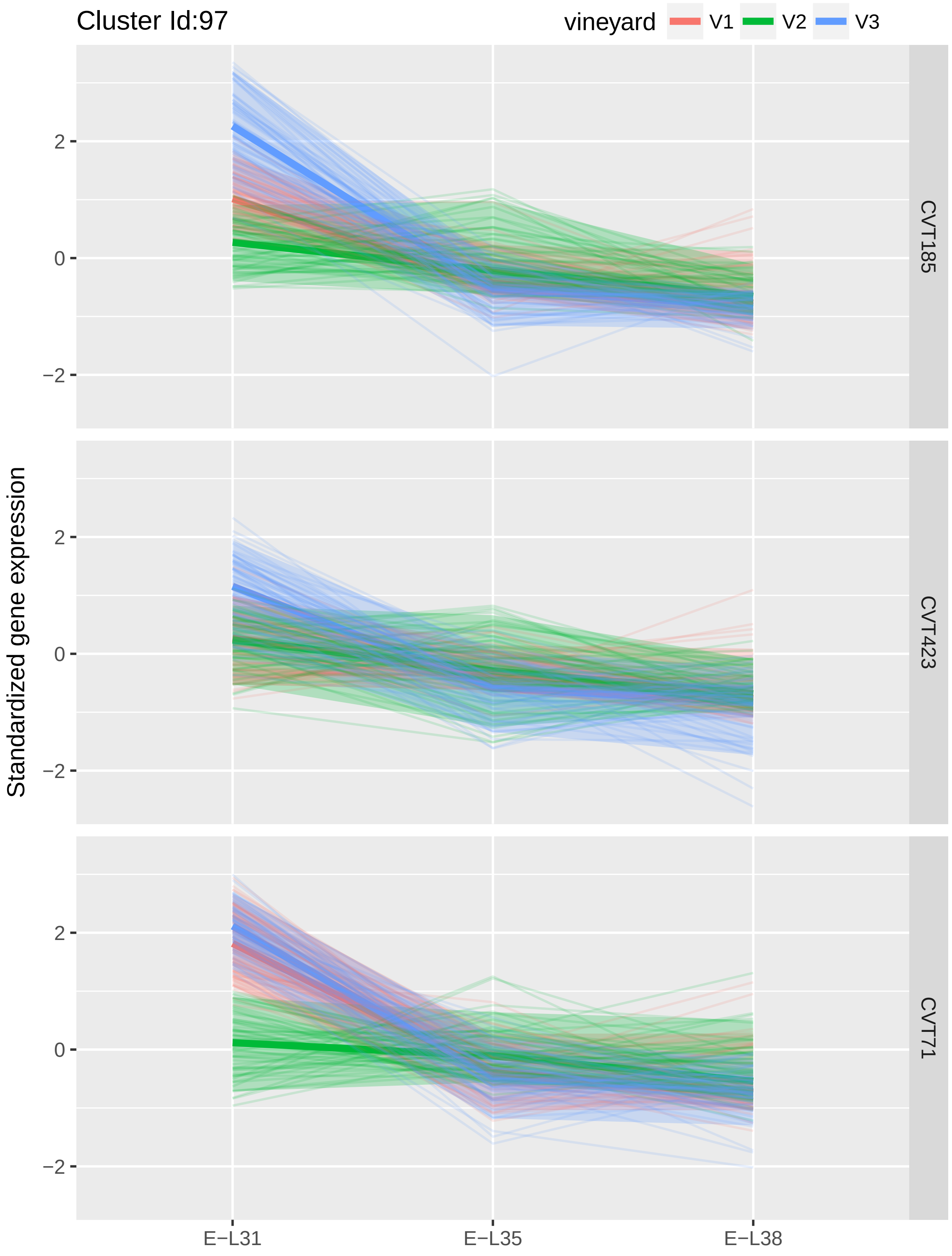

Cluster Id:97
vineyard
V1
V2
V3
Standardized gene expression
2
0
−2
CVT185
CVT423
CVT71
E−L31
E−L35
E−L38

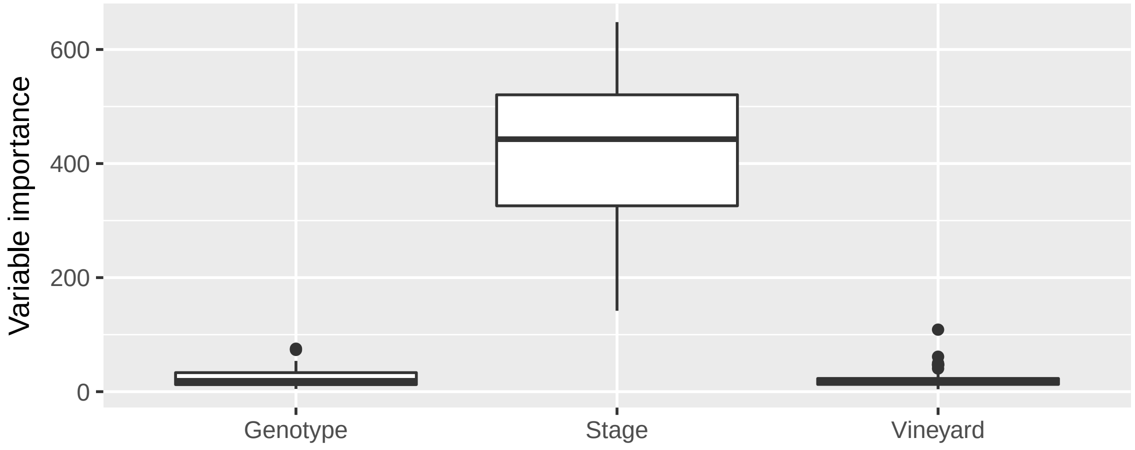

Variable importance
600
400
200
0
Genotype
Stage
Vineyard

Cluster no. 98

```
## Number of genes in the cluster: 113
## Homogeneity Index:     0.6
## Variable importance for Stage:    Median =    301  - Rank =  70
## Variable importance for Clone:    Median =  18.54  - Rank =  60
## Variable importance for Vineyard: Median =   55.4  - Rank =  37
##
## Gene ID               Gene Annotation
## VIT_12s0055g00020  -  UDP-glucose glucosyltransferase
## VIT_12s0055g00460  -  TUBBY like protein 1 TLP10
## VIT_06s0009g00320  -  U-box protein
## VIT_19s0090g00280  -  RNA-binding protein 45 (RBP45)
## VIT_17s0000g05520  -  Calcium Dependent Protein Kinase (VvCPK14)
## VIT_01s0011g02880  -  Cationic amino acid transporter 8
## VIT_06s0004g04010  -  Exocyst subunit EXO70 H7
## VIT_18s0001g04860  -  Aspartate transaminase.
## VIT_09s0054g01000  -  Unknown
## VIT_02s0033g00800  -  Nitrilase 4 (NIT4)
## VIT_02s0033g00870  -  Nitrilase 4B
## VIT_12s0028g02150  -  Transcription factor
## VIT_02s0025g02800  -  No hit
## VIT_12s0034g00270  -  R protein L6
## VIT_12s0034g00850  -  R protein L6
## VIT_17s0000g03600  -  ATSYTE/NTMC2T3.1/NTMC2TYPE3.1/SYTE
## VIT_03s0063g02250  -  Polyol transporter 5
## VIT_07s0005g04880  -  Glutathione S-transferase 25 GSTU7
## VIT_17s0000g02950  -  Glutathione S-transferase 25 GSTU7
## VIT_05s0077g01530  -  s16 (VvPR10.1)_Pathogenesis protein 10 [Vitis vinifera]
## VIT_08s0032g00240  -  CYP71A22
## VIT_06s0004g01030  -  Dirigent protein
## VIT_04s0008g06930  -  Transposase, IS4
## VIT_16s0013g00440  -  No hit
## VIT_07s0141g00890  -  CYP94A1
## VIT_10s0092g00210  -  Transcription factor jumonji (jmj) protein
## VIT_12s0028g00590  -  3-hydroxyisobutyryl-CoA hydrolase
## VIT_09s0002g08370  -  TPR1 (topless-related 1)
## VIT_14s0068g01250  -  Mitochondrial phosphate transporter
## VIT_12s0034g00370  -  R protein L6
## VIT_04s0008g01280  -  No hit
## VIT_06s0004g02010  -  No hit
## VIT_04s0008g04920  -  2-oxoglutarate-dependent dioxygenase
## VIT_14s0006g00350  -  Glutamine synthetase cytosolic isozyme 2
## VIT_18s0117g00550  -  Laccase
## VIT_08s0007g07120  -  Kelch-like protein 18
## VIT_17s0000g08150  -  basic helix-loop-helix (bHLH) family
## VIT_01s0137g00600  -  MAP kinase phosphatase (MKP1)
## VIT_06s0004g01020  -  Dirigent protein
## VIT_05s0062g00020  -  F-box family protein
## VIT_01s0146g00320  -  No hit
## VIT_07s0031g02630  -  Aldehyde Dehydrogenase (VvALDH3H5)
## VIT_05s0094g00350  -  Chitinase class IV
## VIT_05s0077g02210  -  Calcineurin phosphoesterase
## VIT_08s0007g04020  -  AT-hook protein 1 (AHP1)
## VIT_12s0057g01060  -  Jasmonate O-methyltransferase
## VIT_07s0129g01010  -  Unknown
```

```
## VIT_02s0025g04260  -  Osmotin [Vitis vinifera]
## VIT_19s0090g01870  -  ABC Transporter (VvMRP26 - VvABCC26)
## VIT_09s0002g05560  -  ABC Transporter (VvPDR14 - VvABCG44)
## VIT_15s0048g00280  -  TRNA 2'phosphotransferase
## VIT_11s0016g03190  -  Unknown
## VIT_18s0122g00380  -  Squamosa promoter-binding protein (VvSBP17)
## VIT_06s0080g00360  -  Basic Leucine Zipper Transcription Factor (VvbZIP20)
## VIT_07s0005g01350  -  Universal stress protein (USP) family protein
## VIT_08s0058g00700  -  F-box protein (FBL3)
## VIT_03s0038g00130  -  No hit
## VIT_16s0098g01210  -  No hit
## VIT_07s0005g00870  -  Erg-1
## VIT_13s0064g01110  -  No hit
## VIT_02s0025g04250  -  Osmotin
## VIT_07s0005g04890  -  Glutathione S-transferase 25 GSTU7
## VIT_05s0020g04380  -  Calcium-transporting ATPase 13 ACA13
## VIT_18s0001g09550  -  Receptor kinase TRKe
## VIT_02s0033g00700  -  Nitrilase
## VIT_18s0001g09000  -  No hit
## VIT_05s0077g01540  -  s15_Pathogenesis protein 10 [Vitis vinifera]
## VIT_18s0157g00210  -  Inositol-1,4,5-trisphosphate 5-Phosphatase
## VIT_04s0023g01290  -  Anthocyanidin 3-O-glucosyltransferase
## VIT_15s0048g01080  -  Unknown
## VIT_19s0015g00210  -  Zinc transporter ZIP11
## VIT_07s0141g00380  -  U3 small nucleolar RNA-associated protein 18
## VIT_04s0069g01010  -  Harpin-induced protein
## VIT_13s0067g02090  -  Unknown protein
## VIT_08s0032g00360  -  N-6 Adenine-specific DNA methylase
## VIT_08s0058g01110  -  Ovate family protein 4 OFP4
## VIT_12s0035g01000  -  Serine protease inhibitor, serine-type
## VIT_05s0020g05040  -  Proteinase inhibitor 1 PPI3B2
## VIT_05s0077g01550  -  s14_Pathogenesis protein 10 [Vitis vinifera]
## VIT_16s0050g00030  -  Cleavage and polyadenylation specificity factor 5
## VIT_14s0060g01210  -  Basic Leucine Zipper Transcription Factor (VvbZIP37)
## VIT_00s0347g00040  -  Avr9/Cf-9 rapidly elicited protein 20
## VIT_03s0097g00570  -  Bromodomain containing protein
## VIT_12s0035g00920  -  Unknown protein
## VIT_18s0001g02480  -  Unknown protein
## VIT_00s0270g00120  -  Alpha-amylase/subtilisin inhibitor
## VIT_12s0134g00030  -  E-beta-ocimene synthase
## VIT_15s0048g02640  -  No hit
## VIT_05s0020g05000  -  Inhibitor of trypsin and hageman factor (CMTI-V)
## VIT_04s0008g01990  -  Myosin-like protein XI-I
## VIT_09s0002g00390  -  Translation initiation factor eIF-2B alpha subunit
## VIT_07s0031g01260  -  No hit
## VIT_13s0019g05070  -  Nodulin family protein
## VIT_06s0004g04590  -  Epsin N-terminal homology (ENTH) domain-containing
## VIT_18s0001g14920  -  Unknown protein
## VIT_12s0057g01100  -  Nudix hydrolase 2
## VIT_14s0060g01560  -  PHD finger transcription factor
## VIT_14s0068g01760  -  Alcohol dehydrogenase
## VIT_18s0075g00480  -  R protein L6
## VIT_12s0034g01930  -  Globulin-like protein
## VIT_12s0034g01870  -  Cupin
```

```
## VIT_13s0067g02240  -  Armadillo helical
## VIT_16s0098g01220  -  RabGAP/TBC domain-containing protein
## VIT_18s0001g11880  -  No hit
## VIT_01s0011g04690  -  Pentatricopeptide (PPR) repeat-containing protein
## VIT_19s0014g02170  -  4-amino-4-deoxychorismate lyase
## VIT_12s0035g01360  -  FAR1-related sequence 11
## VIT_00s0270g00010  -  No hit
## VIT_09s0002g02570  -  No hit
## VIT_05s0020g02170  -  Sugar transporter ERD6-like 16
## VIT_02s0025g04340  -  Osmotin
## VIT_09s0054g00580  -  No hit
## VIT_13s0067g00890  -  Phosphoinositide-specific phospholipase C
```

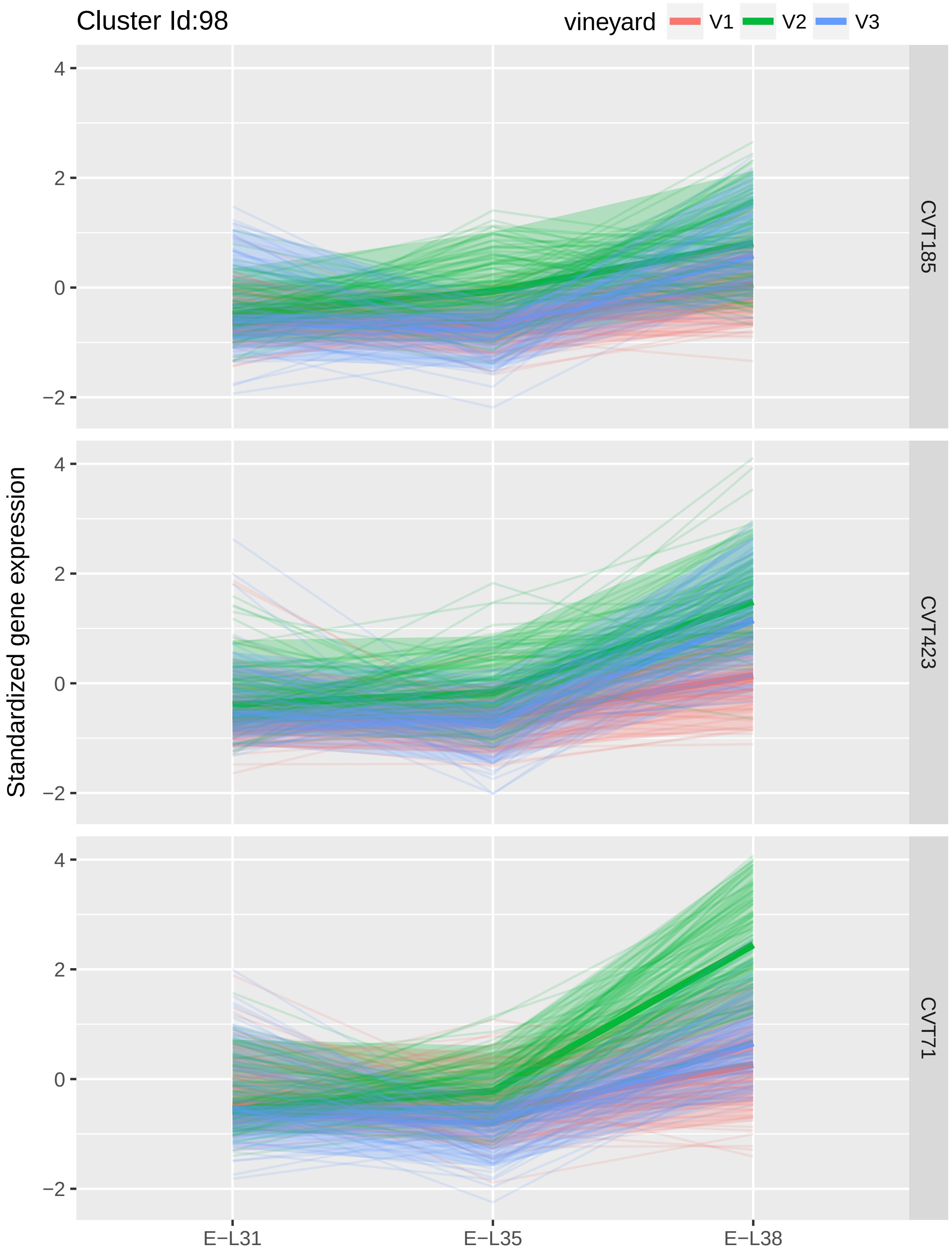
Cluster Id:98
vineyard
V1
V2
V3
Standardized gene expression
4
2
0
−2
CVT185
CVT423
CVT71
E−L31
E−L35
E−L38

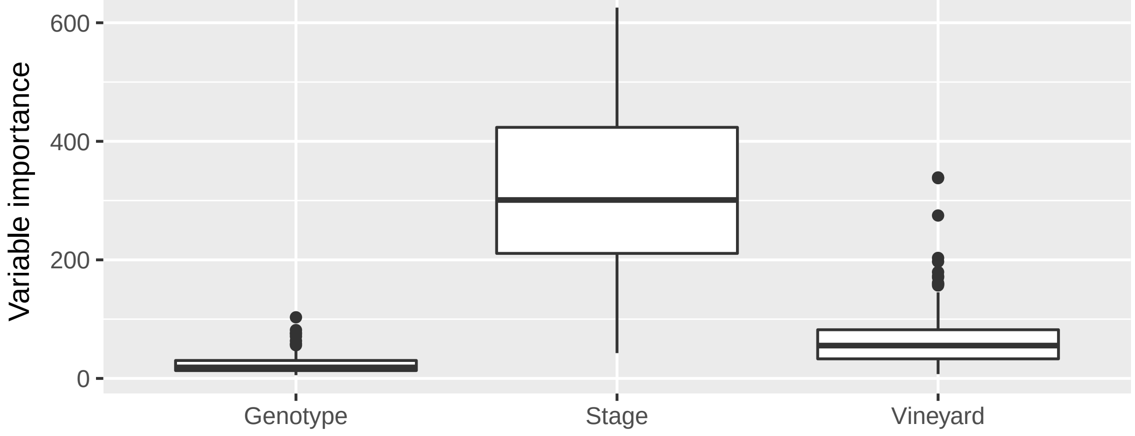
Variable importance
600
400
200
0
Genotype
Stage
Vineyard

Cluster no. 99

```
## Number of genes in the cluster: 119
## Homogeneity Index:    0.82
## Variable importance for Stage:    Median =  545.3  - Rank =  25
## Variable importance for Clone:    Median =  17.08  - Rank =  66
## Variable importance for Vineyard: Median =  26.98  - Rank =  71
##
## Gene ID               Gene Annotation
## VIT_06s0009g03000  -  Flavonoid 3',5'-hydroxylase
## VIT_06s0009g03010  -  Flavonoid 3',5'-hydroxylase (F3'5'H)
## VIT_05s0020g02360  -  Protein phosphatase 2C
## VIT_16s0050g02360  -  Carrier protein CGI-69, Mitochondrial
## VIT_02s0025g02620  -  BDG1 (BODYGUARD1) hydrolase
## VIT_11s0037g00810  -  Unknown protein
## VIT_00s0301g00040  -  Glycine-rich protein
## VIT_05s0020g01370  -  Unknown
## VIT_12s0028g01600  -  FtsH protease (VAR2)
## VIT_14s0060g00860  -  Beta-fructofuranosidase
## VIT_19s0093g00190  -  Glutathione S-transferase 25 GSTU25
## VIT_16s0039g00010  -  ABC Transporter (VvWBC19 - VvABCG19)
## VIT_17s0000g01310  -  Acetohydroxyacid synthase 3
## VIT_18s0001g07620  -  Myb family
## VIT_17s0053g00140  -  Serine/threonine-protein kinase Chk1
## VIT_17s0000g05940  -  Chromosome structural maintenance protein MAG2
## VIT_19s0090g00460  -  Aconitate hydratase, cytoplasmic
## VIT_04s0008g07320  -  Protein phosphatase 2C
## VIT_04s0008g03930  -  RD22
## VIT_13s0019g04640  -  PUMILIO 4
## VIT_03s0038g03130  -  Flavin containing monooxygenase 3
## VIT_06s0004g00060  -  Unknown protein
## VIT_00s2576g00010  -  Phosphoenolpyruvate carboxykinase
## VIT_17s0000g02370  -  Receptor protein kinase
## VIT_02s0025g03530  -  Gamma-glutamylcysteine synthetase
## VIT_01s0137g00010  -  NADH dehydrogenase (ubiquinone) 1 beta subcomplex 7
## VIT_08s0058g01190  -  MAC/Perforin domain-containing protein
## VIT_02s0012g02820  -  Geraniol 10-hydroxylase
## VIT_08s0007g01460  -  FK506-binding protein genes family (VvFKBP72)
## VIT_04s0008g00800  -  Ferrochelatase-2
## VIT_11s0016g00720  -  Polygalacturonase GH28
## VIT_14s0006g01530  -  Armadillo/beta-catenin repeat
## VIT_12s0028g01120  -  Amine oxidase
## VIT_08s0007g05800  -  Patatin
## VIT_18s0001g12730  -  Carbonate dehydratase
## VIT_11s0016g05630  -  Acetylglucosaminyltransferase
## VIT_05s0062g00210  -  Vacuolar ATP synthase subunit F
## VIT_06s0004g03840  -  Chitinase 2
## VIT_07s0151g00430  -  Kinase interacting family protein
## VIT_08s0058g01270  -  Zinc finger (C3HC4-type ring finger)
## VIT_18s0001g15380  -  No hit
## VIT_14s0060g01440  -  DNA helicase RECQI1
## VIT_11s0016g02870  -  Peptidase alpha subunit, Mitochondrial
## VIT_08s0040g01770  -  Cyclic nucleotide-gated ion channel 15
## VIT_16s0022g00960  -  Invertase/pectin methylesterase inhibitor
## VIT_17s0000g09500  -  Serine/threonine-protein kinase AFC2
## VIT_06s0061g00940  -  Vacuolar protein sorting 41 (VPS41)
```

```
## VIT_09s0002g07110  -  KEG (keep on going)
## VIT_08s0040g02680  -  Unknown protein
## VIT_13s0073g00440  -  BSU1-like protein 3 BSL3
## VIT_06s0009g03430  -  Zinc finger (C3HC4-type ring finger)
## VIT_01s0011g03480  -  Cinnamoyl CoA reductase
## VIT_06s0004g03900  -  Calcium-transporting ATPase 1, endoplasmic reticulum-type ECA1
## VIT_13s0073g00490  -  Serine/threonine-protein phosphatase BSL3
## VIT_16s0100g00350  -  ABC Transporter (VvTAP3 - VvABCB23)
## VIT_18s0001g08280  -  HEN4 (HUA enhancer 4)
## VIT_11s0118g00220  -  Chloride channel protein CLC
## VIT_16s0098g00250  -  Zinc finger (C3HC4-type ring finger)
## VIT_06s0004g07560  -  Ribosomal protein S29 28S
## VIT_11s0052g00980  -  Yip1 domain , member 6
## VIT_17s0000g06180  -  Hydrolase, alpha/beta fold
## VIT_17s0000g02900  -  ATP synthase beta chain 2, mitochondrial
## VIT_07s0151g00370  -  Kinase interacting
## VIT_18s0041g02110  -  Unknown protein
## VIT_19s0014g03700  -  Cyclic nucleotide-regulated ion channel 4
## VIT_03s0038g04350  -  Proteasome 26S AAA-ATPase subunit (RPT4)
## VIT_06s0009g03170  -  Peptidyl-prolyl cis-trans isomerase, parvulin-type
## VIT_08s0040g01950  -  Zinc finger (C3HC4-type ring finger)
## VIT_00s2840g00010  -  Phosphoenolpyruvate carboxykinase
## VIT_09s0002g02600  -  VPS46.2 SNF7
## VIT_01s0011g05240  -  No hit
## VIT_17s0000g02960  -  Cyclin-dependent kinase D1;3
## VIT_14s0060g01530  -  DNA polymerase III subunit epsilon
## VIT_15s0021g02070  -  Unknown protein
## VIT_03s0088g01170  -  Proline iminopeptidase
## VIT_07s0129g00620  -  Metal transporter Nramp3
## VIT_18s0001g03950  -  Pm27 protein
## VIT_06s0004g02680  -  Lysine decarboxylase
## VIT_13s0067g00870  -  Peptidyl-prolyl cis-trans isomerase ROC7 (rotamase CyP 7)
## VIT_07s0031g03060  -  NSL1 (necrotic spotted lesions 1)
## VIT_07s0031g01740  -  ATP synthase 24 kDa subunit
## VIT_16s0098g01130  -  Unknown protein
## VIT_11s0016g03100  -  6-phosphogluconate dehydrogenase
## VIT_14s0060g00840  -  Adaptor-related protein complex 3, mu 1 sub
## VIT_05s0077g00550  -  SFR2 (sensitive TO FREEZING 2)
## VIT_08s0032g00280  -  Regulator of chromosome condensation (RCC1)
## VIT_01s0026g01480  -  Adenosine/AMP deaminase active site
## VIT_05s0020g02430  -  Protein FBL4
## VIT_05s0020g03080  -  Acyl-CoA synthetase long-chain member 6
## VIT_13s0064g00570  -  Constans interacting protein 6
## VIT_00s0299g00110  -  Glycoprotein homolog, related
## VIT_15s0024g00260  -  Na+/H+ antiporter isoform 6
## VIT_00s2240g00010  -  GAE3 (UDP-D- glcucuronate  4-epimerase 3)
## VIT_09s0002g06370  -  Trafficking protein particle complex protein 2
## VIT_17s0000g09490  -  Quinolinate synthase
## VIT_00s0270g00050  -  Unknown protein
## VIT_13s0084g00050  -  Cysteine proteinase inhibitor
## VIT_12s0057g00480  -  RNA-binding protein Musashi
## VIT_08s0040g00680  -  Phosphatidylinositol 4-kinase type-II
## VIT_00s1995g00010  -  Phosphoenolpyruvate carboxykinase
## VIT_17s0000g08140  -  MLK/Raf-related protein kinase 1
```

```
## VIT_11s0065g00320  -  Proteasome 20S beta subunit A (PBA1) (PRCD)
## VIT_04s0069g00790  -  Cyclic nucleotide-regulated ion channel (CNGC14)
## VIT_04s0008g06320  -  Unknown protein
## VIT_14s0036g00050  -  Aluminum-induced protein
## VIT_05s0077g01100  -  Ubiquitin-conjugating enzyme E2 J1
## VIT_04s0023g01650  -  Succinate dehydrogenase flavoprotein subunit,precursor
## VIT_03s0063g02030  -  4-hydroxy-3-methylbut-2-enyl diphosphate reductase
## VIT_05s0077g02320  -  Hypoxia-responsive
## VIT_16s0039g02700  -  Unknown protein
## VIT_05s0077g02380  -  DnaJ homolog, subfamily B, member 12
## VIT_18s0001g00900  -  EMB1793
## VIT_03s0180g00080  -  Cyclase
## VIT_18s0122g00110  -  Unknown protein
## VIT_18s0001g01020  -  V-type H+-transporting ATPase subunit B
## VIT_15s0021g02460  -  Unknown protein
## VIT_14s0108g00080  -  V-type H+-transporting ATPase subunit AC39
## VIT_13s0074g00710  -  Unknown protein
## VIT_19s0014g01850  -  Zinc finger (C3HC4-type ring finger)
```

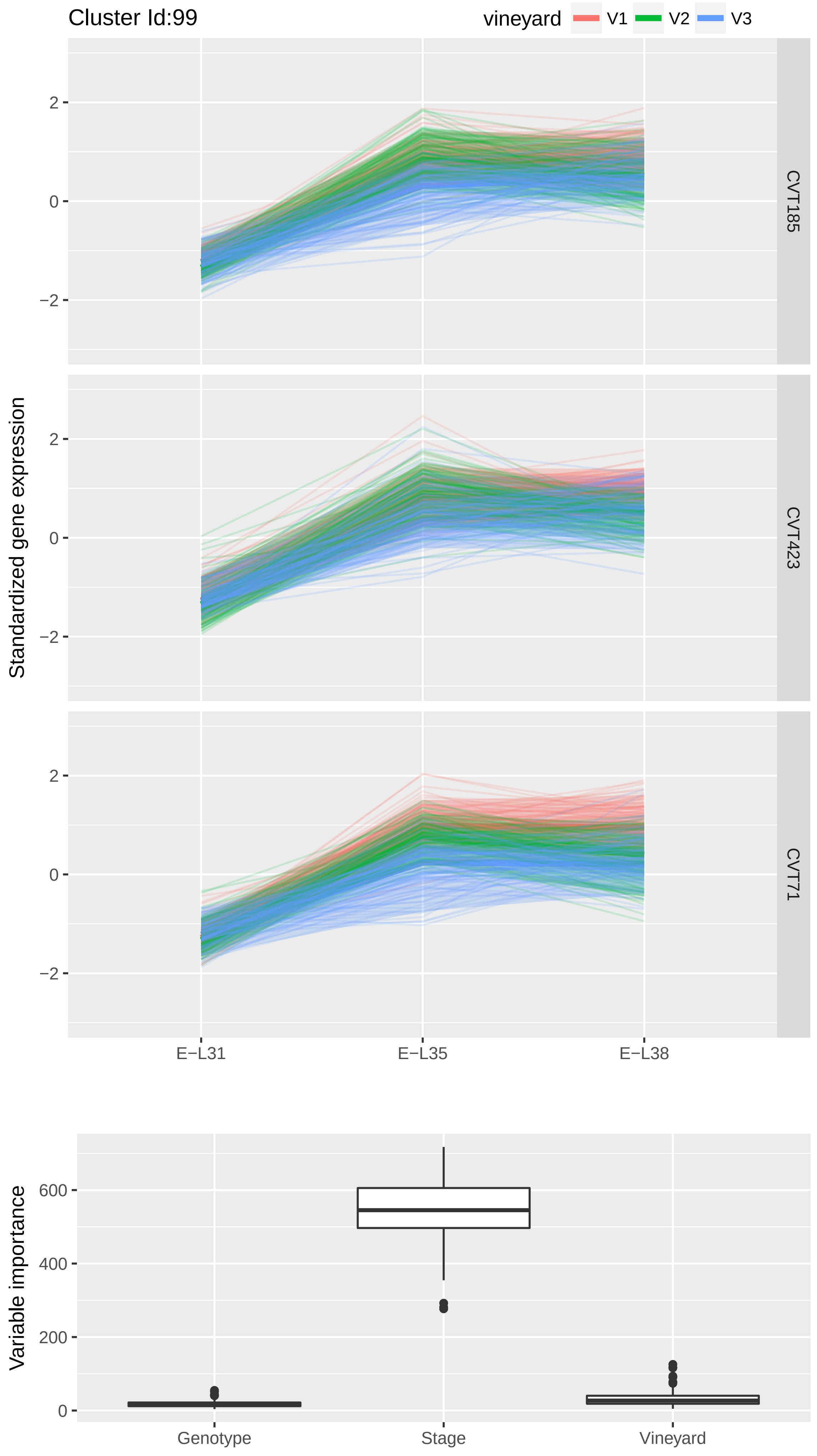
Cluster Id:99
vineyard
V1
V2
V3
CVT185
CVT423
CVT71
Standardized gene expression
2
0
−2
E−L31
E−L35
E−L38
Variable importance
600
400
200
0
Genotype
Stage
Vineyard

Cluster no. 100

```
## Number of genes in the cluster: 63
## Homogeneity Index:    0.69
## Variable importance for Stage:    Median =  440.8  - Rank =  52
## Variable importance for Clone:    Median =   19.7  - Rank =  51
## Variable importance for Vineyard: Median =  28.09  - Rank =  69
##
## Gene ID               Gene Annotation
## VIT_02s0025g01400  -  Leucine-rich repeat family protein stress
## VIT_00s0287g00080  -  Vesicle-associated membrane protein
## VIT_07s0130g00210  -  PHD finger transcription factor
## VIT_06s0009g01000  -  Zinc finger (C3HC4-type ring finger) CIC7E11
## VIT_13s0019g03470  -  Translation initiation factor eIF-3 subunit 5
## VIT_02s0025g03280  -  Aminopeptidase N
## VIT_01s0011g05760  -  Zinc finger (C3HC4-type ring finger)
## VIT_11s0016g02120  -  Density-regulated protein DRP1
## VIT_14s0030g00950  -  SOD Cu/Zn superoxide dismutase
## VIT_16s0050g00590  -  Cleavage and polyadenylation specificity factor subunit 2
## VIT_10s0071g00200  -  Disease resistance protein (TIR-NBS-LRR class)
## VIT_13s0067g03160  -  No hit
## VIT_09s0002g06000  -  Cytochrome c oxidase subunit Vb
## VIT_04s0008g04890  -  WD-40 repeat protein (LEUNIG)
## VIT_13s0047g01100  -  Vacuolar protein sorting 36
## VIT_17s0000g04960  -  Polygalacturonate 4-alpha-galacturonosyltransferase GAUT11
## VIT_06s0004g05480  -  Translation initiation factor eIF-3 subunit 3
## VIT_14s0066g01600  -  NHL repeat-containing protein
## VIT_18s0001g08220  -  SUT4 (sucrose transporter 4)
## VIT_18s0001g03220  -  Translation initiation factor eIF-1A
## VIT_02s0154g00330  -  Unknown protein
## VIT_04s0023g00820  -  Unknown protein
## VIT_08s0058g00570  -  Macrophage migration inhibitory factor
## VIT_06s0004g08280  -  Ubiquinol-cytochrome c reductase subunit 8
## VIT_01s0011g06030  -  Myrosinase precursor
## VIT_19s0090g00390  -  Endoribonuclease L-PSP
## VIT_11s0016g04520  -  Transmembrane protein 19
## VIT_11s0118g00660  -  Unknown protein
## VIT_01s0146g00290  -  Unknown protein
## VIT_06s0061g01500  -  No hit
## VIT_05s0077g01980  -  Unknown protein
## VIT_15s0048g02570  -  Component of oligomeric golgi complex 4
## VIT_15s0021g01210  -  Peptidyl-prolyl cis-trans isomerase, cyclophilin type
## VIT_01s0011g06130  -  Erwinia induced protein 2
## VIT_05s0029g00330  -  Adenosylhomocysteinase (S-adenosyl-L-homocysteine hydrolase)
## VIT_17s0000g01860  -  BPC2/BBR/BPC2/BPC2 (basic pentacysteine2)
## VIT_07s0289g00020  -  ferredoxin
## VIT_09s0002g04620  -  Unknown protein
## VIT_03s0063g00070  -  PAB8 (poly(A) binding protein 8)
## VIT_06s0004g04910  -  Unknown protein
## VIT_18s0001g02730  -  Unknown
## VIT_13s0019g03860  -  F-type H+-transporting ATPase d chain
## VIT_03s0017g00710  -  flavonol synthase
## VIT_16s0098g01100  -  Ribosomal protein L24 60S
## VIT_05s0049g00510  -  Ethylene response factor ERF1
## VIT_15s0046g00270  -  fiber protein Fb11
## VIT_14s0066g01350  -  No hit
```

```
## VIT_04s0008g00830  -  NADH dehydrogenase (ubiquinone) Fe-S protein 7
## VIT_18s0117g00320  -  Unknown protein
## VIT_12s0059g02470  -  Unknown protein
## VIT_13s0019g04270  -  No hit
## VIT_19s0090g01330  -  V-type H+-transporting ATPase subunit H
## VIT_04s0069g01180  -  NC domain-containing protein
## VIT_03s0132g00200  -  Aldehyde Dehydrogenase (VvALDH6B3)
## VIT_14s0108g01370  -  Saccharopine dehydrogenase
## VIT_17s0000g01700  -  Leucine-rich repeat transmembrane protein kinase
## VIT_05s0077g02340  -  NADH dehydrogenase (ubiquinone) Fe-S protein 5
## VIT_14s0219g00120  -  PQ-loop repeat / transmembrane
## VIT_05s0020g04250  -  U5 snRNP protein, DIM1 family
## VIT_14s0060g00890  -  Agenet domain-containing protein
## VIT_06s0009g01590  -  DNA repair protein Nse1
## VIT_03s0038g01440  -  Adenosylhomocysteine nucleosidase.
## VIT_07s0005g02990  -  Bet1 SNARE 1-1
```

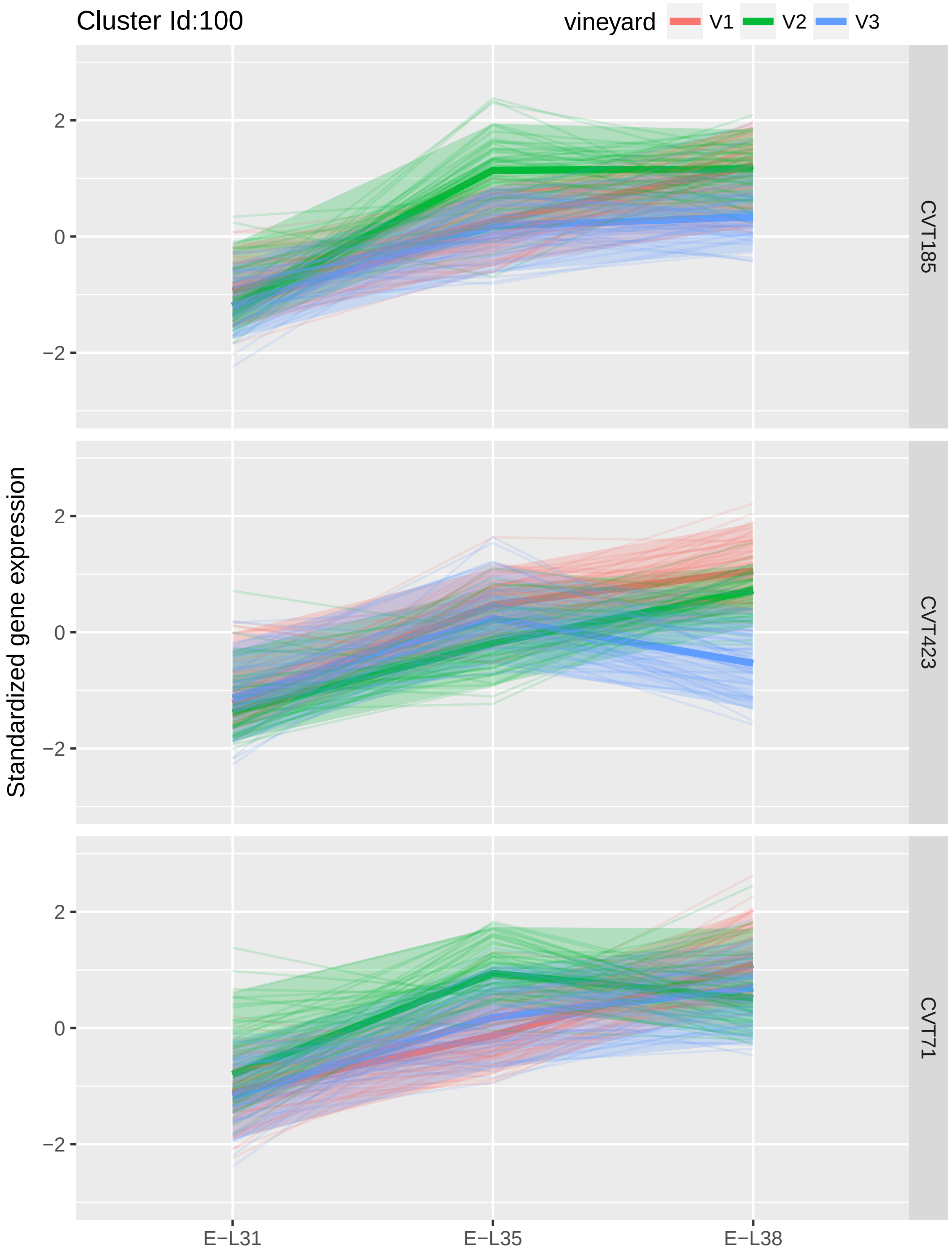
Cluster Id:100
vineyard
V1
V2
V3
Standardized gene expression
2
0
−2
CVT185
CVT423
CVT71
E−L31
E−L35
E−L38

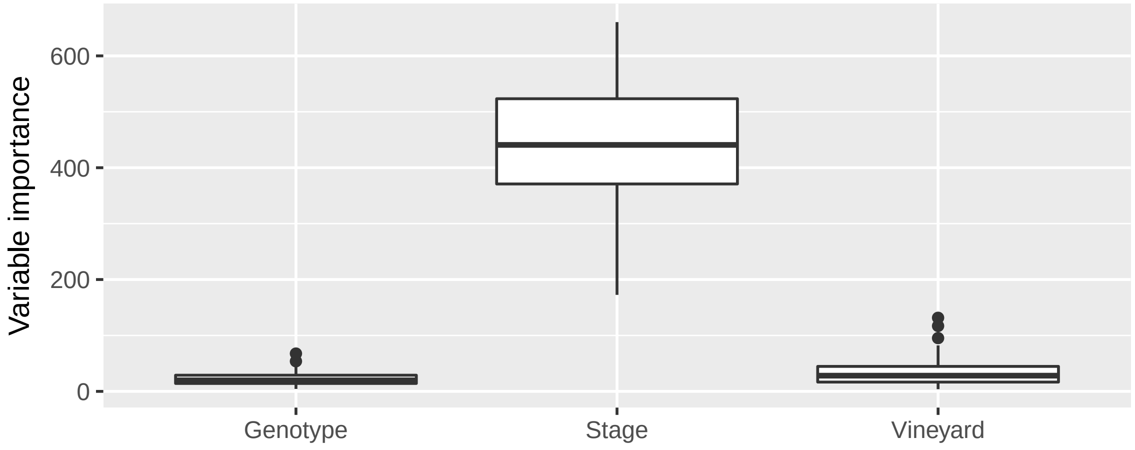
Variable importance
600
400
200
0
Genotype
Stage
Vineyard

Cluster no. 101

```
## Number of genes in the cluster: 98
## Homogeneity Index:    0.83
## Variable importance for Stage:    Median =  483.6  - Rank =  39
## Variable importance for Clone:    Median =  10.81  - Rank =  100
## Variable importance for Vineyard: Median =  18.42  - Rank =  86
##
## Gene ID               Gene Annotation
## VIT_10s0071g01020  -  Lipoic acid synthase
## VIT_14s0108g00570  -  RNA helicase protein, mitochondrial
## VIT_00s1232g00010  -  Double-stranded telomeric DNA binding
## VIT_06s0004g04780  -  Methyl-CpG-binding domain-containing protein
## VIT_18s0001g01820  -  NAC domain-containing protein (VvNAC15)
## VIT_15s0048g01160  -  Phospholipase D beta 1
## VIT_05s0020g01320  -  Cullin 1C
## VIT_13s0019g01430  -  Heat shock protein 70
## VIT_16s0098g00350  -  SDG29 (SET Domain group 29)
## VIT_07s0005g01370  -  DNA mismatch repair protein MSH6
## VIT_00s0309g00020  -  Histone-lysine N-methyltransferase, H3 lysine-9 specific SUVH5
## VIT_04s0008g02780  -  RBR1 (retinoblastoma-related 1)
## VIT_09s0002g00770  -  2OG-Fe(II) oxygenase
## VIT_18s0122g00890  -  Oligosaccharyltransferase complex subunit alpha (ribophorin I)
## VIT_11s0016g04120  -  No hit
## VIT_06s0004g06120  -  Cell division protein FtsZ
## VIT_09s0002g04760  -  SNF1-related protein kinase regulatory subunit beta-2 (AKIN beta-2)
## VIT_12s0028g03080  -  Homeobox transcription factor
## VIT_06s0061g01610  -  DNA polymerase epsilon, subunit A
## VIT_13s0047g00490  -  No hit
## VIT_01s0011g02230  -  Para-hydroxybenzoate--polyprenyltransferase
## VIT_06s0004g01290  -  KH domain and CCCH containing protein
## VIT_18s0001g07210  -  Unknown
## VIT_11s0016g00360  -  1-phosphatidylinositol-3-phosphate 5-kinase
## VIT_19s0014g01580  -  No hit
## VIT_13s0019g01440  -  No hit
## VIT_04s0008g06080  -  Unknown protein
## VIT_11s0016g04790  -  No hit
## VIT_14s0219g00210  -  Ubiquitin family
## VIT_04s0023g02600  -  folate transporter 1
## VIT_10s0042g00580  -  Zinc finger (C3HC4-type ring finger)
## VIT_05s0049g02220  -  Histone-lysine N-methyltransferase, H3 lysine-9 specific SUVH9
## VIT_14s0060g01140  -  PAPA-1
## VIT_06s0004g00110  -  Unknown protein
## VIT_05s0020g00580  -  Protein transporter
## VIT_01s0150g00030  -  ARF GTPase activator SCARFACE
## VIT_05s0077g00010  -  Protein transporter
## VIT_00s0404g00070  -  No hit
## VIT_06s0004g05570  -  Subtilase
## VIT_06s0009g01760  -  F-box protein / WD-40 repeat
## VIT_07s0005g01680  -  Stachyose synthase
## VIT_02s0025g01650  -  PHD finger transcription factor
## VIT_03s0017g00300  -  DnaJ homolog, subfamily B, member 12
## VIT_14s0060g01260  -  Peptide chain release factor 2
## VIT_18s0001g00960  -  Pseudouridine synthase
## VIT_14s0066g00820  -  Unknown protein
## VIT_03s0038g04510  -  Chaperone BCS1 mitochondrial
```

```
## VIT_15s0046g00760  -  Histone-lysine N-methyltransferase ATX5
## VIT_06s0061g01080  -  Binding
## VIT_15s0107g00410  -  Pleckstriny (PH) domain-containing protein
## VIT_05s0020g00010  -  Protein transporter
## VIT_16s0050g00690  -  Protein kinase
## VIT_00s0313g00070  -  putative MADS-box Short Vegetal Phase 1 (VviSVP1)
## VIT_18s0001g13530  -  SH3 domain-containing protein 2
## VIT_14s0036g00430  -  Glycogenin glucosyltransferase (glycogenin)
## VIT_06s0004g00910  -  Protease inhibitor/seed storage/lipid transfer protein (LTP)
## VIT_05s0020g00850  -  Transposon protein, Mutator sub-class
## VIT_09s0002g00780  -  Vesicle transport protein (AtSEC22)
## VIT_14s0060g02240  -  U6 snRNA-associated Sm-like protein LSm4
## VIT_09s0070g00370  -  Unknown protein
## VIT_01s0011g02120  -  HUA2 (enhancer of AG-4 2)
## VIT_18s0001g11270  -  SERine Proteinase Inhibitor Serpin P
## VIT_06s0004g00920  -  Phosphatidylinositol glycan, class V
## VIT_19s0014g01570  -  RNA-binding protein AKIP1
## VIT_02s0025g03400  -  Growth-on protein GRO10
## VIT_14s0171g00530  -  Protein phosphatase 2 (formerly 2A), regulatory subunit B'
## VIT_01s0011g05400  -  Actin related protein 2/3 complex, subunit 2
## VIT_02s0025g02580  -  RNA polymerase II mediator complex
## VIT_06s0009g03330  -  Cig3
## VIT_13s0067g03720  -  SAC3/GANP
## VIT_18s0001g03290  -  1-phosphatidylinositol-4-phosphate 5-kinase
## VIT_18s0001g09260  -  Unknown protein
## VIT_08s0007g05250  -  Cig3
## VIT_10s0003g00240  -  Unknown protein
## VIT_19s0014g01490  -  Galactosyltransferase family protein
## VIT_07s0130g00170  -  Transmembrane CLPTM1 Cleft lip and palate transmembrane protein 1
## VIT_16s0098g01360  -  No hit
## VIT_05s0020g01220  -  ESD4 (early in short days 4)
## VIT_01s0011g01930  -  Alpha-1,4-glycosyltransferase
## VIT_04s0044g01930  -  FAR1-related sequence 5
## VIT_14s0068g00630  -  Importin
## VIT_10s0003g00180  -  Drought induced 19 protein
## VIT_18s0001g01810  -  MEI1 (meiosis defective 1)
## VIT_01s0010g01450  -  Unknown protein
## VIT_13s0019g01230  -  Dolichyl-phosphate beta-glucosyltransferase
## VIT_07s0005g05320  -  Calcium-dependent protein kinase 13 CPK13
## VIT_07s0151g00300  -  Unknown protein
## VIT_06s0009g01050  -  Zinc knuckle
## VIT_14s0219g00010  -  Protein phosphatase 2 (formerly 2A), regulatory subunit B'
## VIT_00s0317g00100  -  Splicing factor 3B subunit 3
## VIT_13s0019g01240  -  Actin beta/gamma 1
## VIT_19s0014g02080  -  AAA-type ATPase
## VIT_06s0061g00840  -  PHD finger transcription factor
## VIT_12s0028g03090  -  Homeobox transcription factor
## VIT_07s0005g00610  -  Tubulin gamma complex component 4
## VIT_04s0044g01920  -  FAR1-related sequence 5
## VIT_06s0004g07570  -  Zinc finger (C3HC4-type ring finger)
## VIT_16s0050g01460  -  Translocation protein SEC63
```

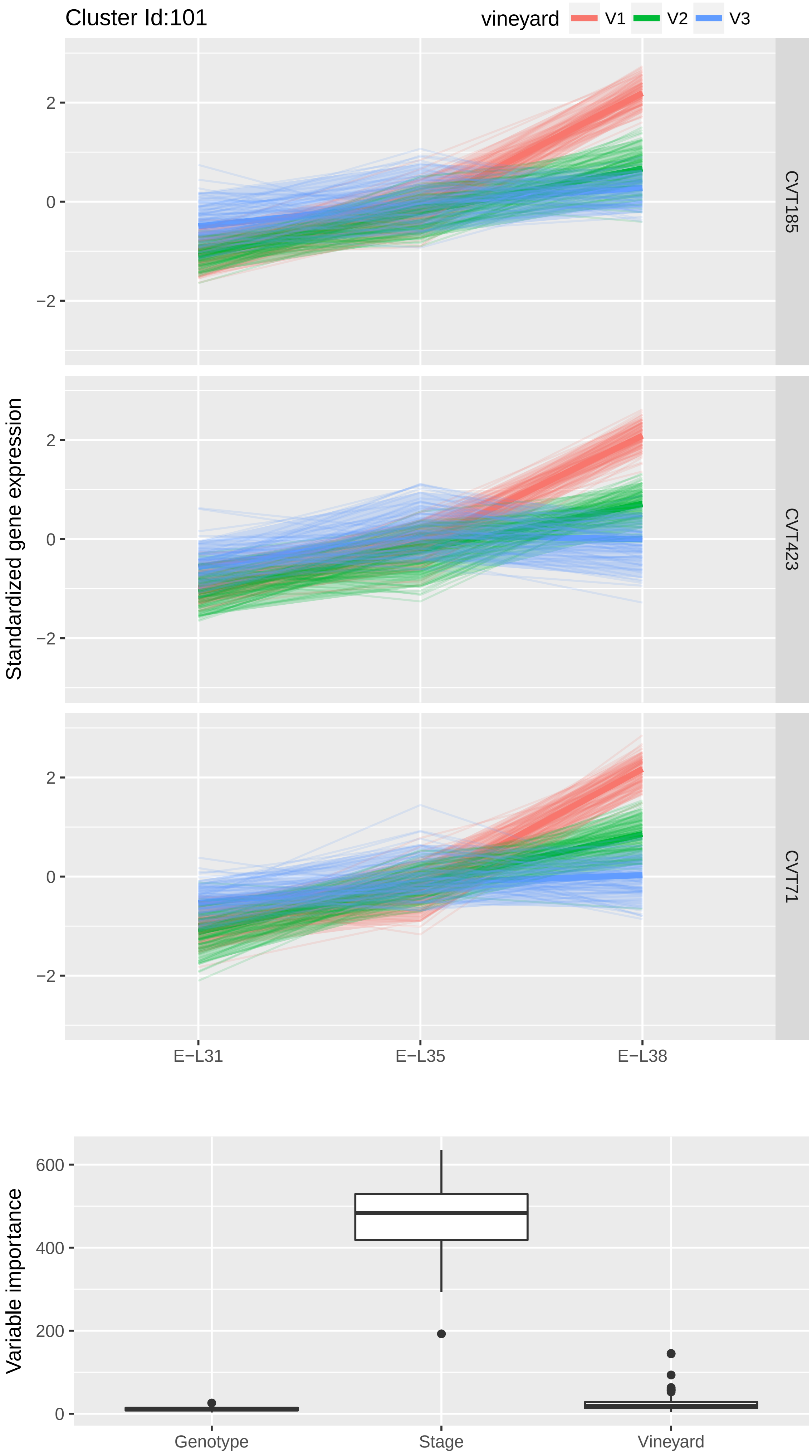
Cluster Id:101
vineyard
V1
V2
V3
Standardized gene expression
CVT185
CVT423
CVT71
2
0
−2
E−L31
E−L35
E−L38
Variable importance
600
400
200
0
Genotype
Stage
Vineyard

Cluster no. 102

```
## Number of genes in the cluster: 69
## Homogeneity Index:    0.67
## Variable importance for Stage:    Median =  305.7  - Rank =  69
## Variable importance for Clone:    Median =  26.95  - Rank =  15
## Variable importance for Vineyard: Median =  61.77  - Rank =  32
##
## Gene ID               Gene Annotation
## VIT_08s0007g07220  -  Ribosomal protein S5 (RPS5B) 40S
## VIT_13s0156g00320  -  KH domain and CCCH containing protein
## VIT_10s0071g00770  -  Aspartic Protease (VvAP29)
## VIT_17s0000g03780  -  No hit
## VIT_19s0090g01080  -  ERF/AP2 transcription factor sub B-1(ATERF-3)
## VIT_14s0068g01370  -  Translocon-associated protein beta TRAP complex
## VIT_18s0001g14400  -  Peptidyl-prolyl cis-trans isomerase ROC5 (rotamase CYP 5)
## VIT_14s0060g00550  -  ATP-citrate synthase
## VIT_09s0002g01620  -  Ribosomal protein L9 (RPL90A/C) 60S
## VIT_05s0020g04790  -  Ribosomal protein S15 (RPS15D) 40S
## VIT_11s0016g04770  -  Unknown
## VIT_03s0063g00130  -  Ribosomal protein S16 (RPS16A) 40S
## VIT_03s0038g03230  -  1-acyl-sn-glycerol-3-phosphate acyltransferase zeta precursor
## VIT_08s0040g02540  -  Diaminopimelate epimerase
## VIT_08s0040g03200  -  Ribosomal protein L4/L1 (RPL4A) 60S
## VIT_03s0038g00160  -  Bromo-adjacenty (BAH) domain-containing protein
## VIT_09s0002g03960  -  Cytochrome c oxidase subunit 19
## VIT_19s0014g01520  -  4-methyl-5-thiazole monophosphate biosynthesis protein
## VIT_18s0001g02630  -  F-box only protein 9
## VIT_00s0313g00030  -  Auxin-independent growth promoter
## VIT_06s0004g02330  -  Sulfite reductase
## VIT_13s0067g02170  -  Unknown protein
## VIT_07s0005g03930  -  Developmental protein DAG, plastid
## VIT_16s0022g01560  -  Nucleotide-sensitive chloride conductance regulator (ICln)
## VIT_00s0516g00030  -  Dehydrogenase/reductase SDR family member 4
## VIT_16s0039g01490  -  Unknown protein
## VIT_09s0002g08480  -  NADP-dependent oxidoreductase
## VIT_17s0000g05160  -  SP1L2 (SPIRAL2)
## VIT_18s0089g01180  -  No hit
## VIT_12s0055g01090  -  Unknown protein
## VIT_10s0116g01400  -  Peroxisomal membrane protein PMP22
## VIT_04s0023g01970  -  Aldose 1-epimerase
## VIT_16s0039g00350  -  Unknown protein
## VIT_13s0019g00530  -  Ribosomal protein S15A (RPS15aF) 40S
## VIT_18s0089g00020  -  Phosphatidylinositol N-acetylglucosaminyltransferase subunit P
## VIT_09s0070g00050  -  Ribosomal protein S12 (RPS12C) 40S
## VIT_07s0005g00330  -  No hit
## VIT_05s0102g00260  -  Cell division cycle protein 48
## VIT_07s0005g02760  -  Unknown
## VIT_04s0008g03320  -  UVB-resistance protein
## VIT_02s0025g04620  -  Phospholipase D beta 1
## VIT_03s0063g00280  -  Thioredoxin M-type 3
## VIT_10s0116g00180  -  Ribosomal protein L4/L1
## VIT_04s0008g02100  -  CID7 damaged DNA binding
## VIT_03s0038g04320  -  SUMO activating enzyme 2 (SAE2)
## VIT_08s0040g01380  -  Acylphosphatase family
## VIT_01s0011g02180  -  Nucleolin
```

```
## VIT_12s0059g00030  -  Unknown protein
## VIT_09s0002g05950  -  Ubiquinol-cytochrome c reductase cytochrome c1
## VIT_13s0019g01040  -  Unknown protein
## VIT_07s0031g00200  -  IMP dehydrogenase
## VIT_06s0009g03080  -  Histone deacetylase (HD2A)
## VIT_02s0012g02460  -  NADP-malic enzyme
## VIT_00s0477g00040  -  Clathrin assembly protein 16
## VIT_12s0028g01800  -  Ribosomal protein L11 (RPL11D) 60S
## VIT_05s0020g01880  -  Unknown protein
## VIT_08s0217g00120  -  Ribosomal protein L41 60S
## VIT_13s0019g02170  -  Laccase
## VIT_07s0104g00150  -  Outer envelope membrane protein
## VIT_19s0015g01770  -  High mobility group (HMG1/2)
## VIT_02s0025g00090  -  Arginine/serine-rich splicing factor 10
## VIT_01s0150g00580  -  Ribosomal protein L10 (RPL10B) 60S
## VIT_06s0080g00740  -  Ribosomal protein S14 (RPS14C) 40S
## VIT_12s0059g01480  -  Lipase family
## VIT_11s0016g03630  -  Peroxiredoxin-5
## VIT_14s0036g00520  -  L-allo-threonine aldolase
## VIT_16s0098g01710  -  Unknown protein
## VIT_19s0027g00710  -  Ribosomal protein L27 (RPL27C) 60S
## VIT_19s0090g00490  -  Unknown protein
```

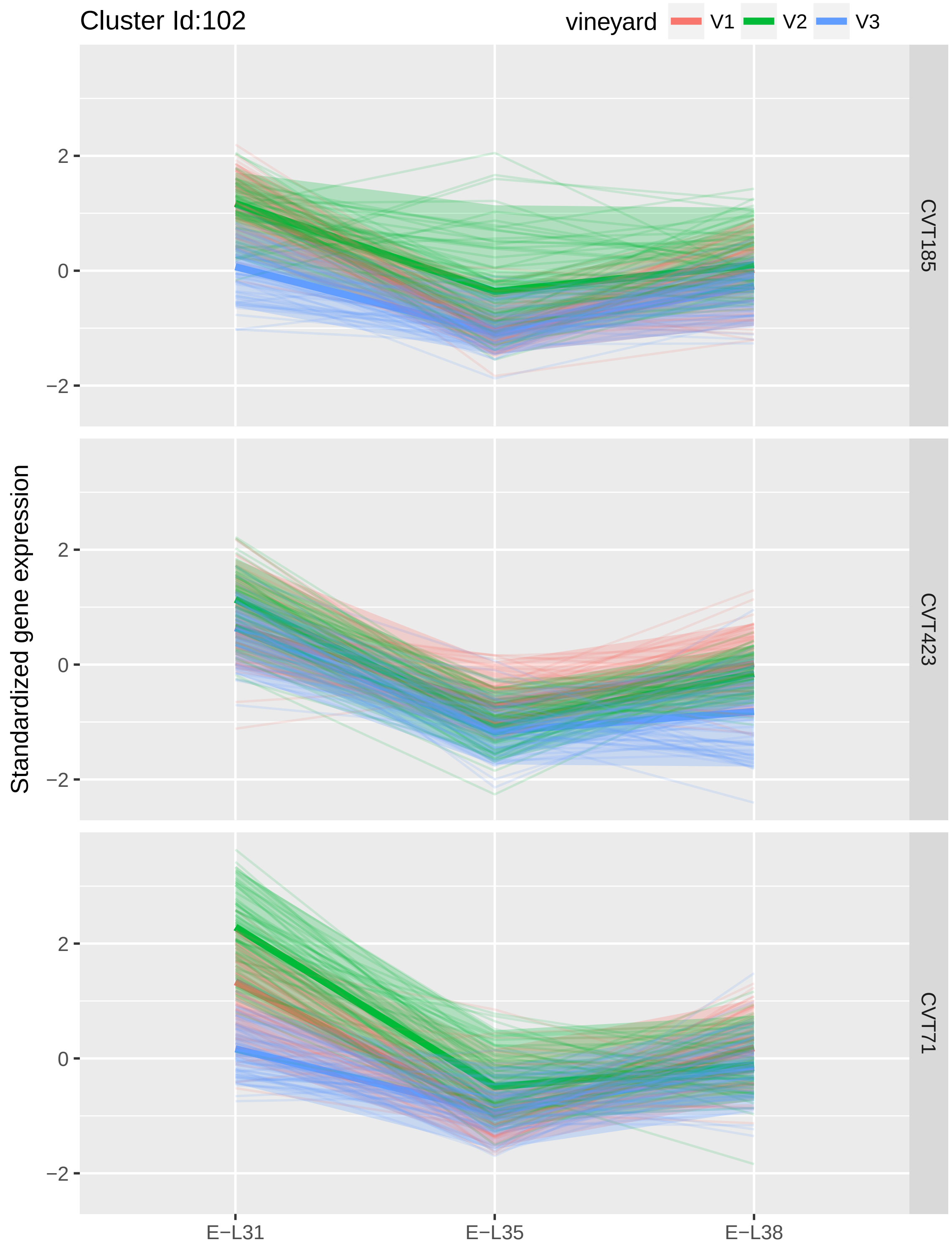
Cluster Id:102
vineyard
V1
V2
V3
Standardized gene expression
CVT185
CVT423
CVT71
2
0
−2
E−L31
E−L35
E−L38

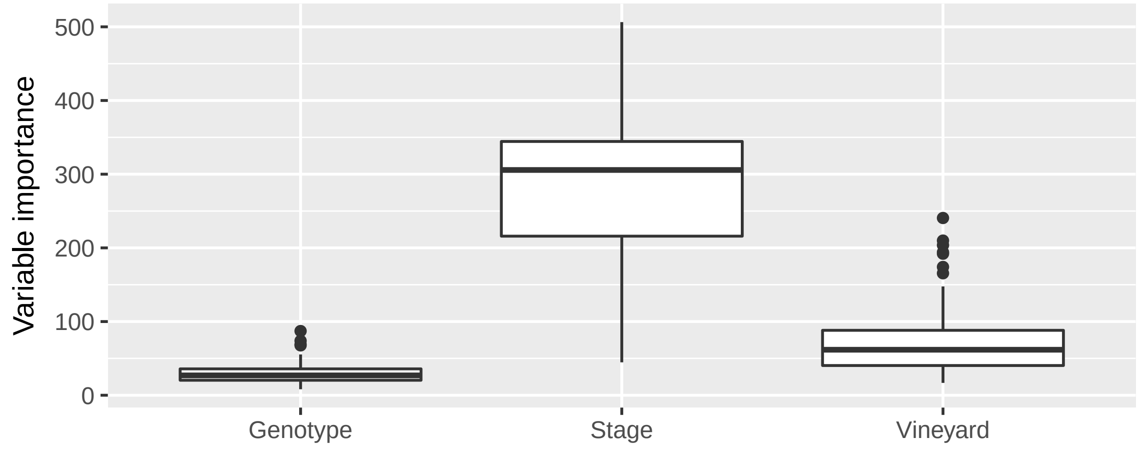
Variable importance
500
400
300
200
100
0
Genotype
Stage
Vineyard

Cluster no. 103

```
## Number of genes in the cluster: 38
## Homogeneity Index:    0.77
## Variable importance for Stage:    Median =  385.1  - Rank =  59
## Variable importance for Clone:    Median =  22.49  - Rank =  35
## Variable importance for Vineyard: Median =  18.09  - Rank =  89
##
## Gene ID                Gene Annotation
## VIT_05s0049g01740  -  Actin related protein 2/3 complex, subunit 2
## VIT_00s0561g00020  -  Stem-specific protein TSJT1
## VIT_07s0141g00620  -  Bile acid:sodium symporter
## VIT_14s0171g00240  -  SYN2 (Sister chromatid cohesion 1 (SCC1) protein homolog 2) cohesin
## VIT_14s0068g01200  -  Inducer of CBF expression 1 ICE1
## VIT_18s0089g01410  -  Purine permease 4 PUP4
## VIT_08s0007g04740  -  RAB GTPase RAB_ALPHA
## VIT_11s0103g00190  -  Phospholipase A2 alpha
## VIT_19s0014g04690  -  Indole-3-acetic acid-amido synthetase
## VIT_19s0014g01820  -  RAB GTPase RABA1F
## VIT_11s0037g00290  -  Plastocyanin domain-containing protein
## VIT_14s0006g02550  -  Non-specific lipid-transfer protein 2 (LTP 2)
## VIT_17s0000g09980  -  REF6 (relative of early flowering 6)
## VIT_03s0063g00910  -  No hit
## VIT_08s0007g04620  -  No hit
## VIT_08s0040g02340  -  Xylan synthase
## VIT_19s0015g01020  -  ABA-responsive element-binding protein ABF2, Basic Leucine Zipper Tran
## VIT_00s0494g00010  -  Growth-regulating factor 1
## VIT_00s0333g00050  -  DIR1 (defective IN induced resistance 1)
## VIT_17s0000g01670  -  WD40
## VIT_09s0018g00720  -  No hit
## VIT_01s0137g00410  -  CYP86A2
## VIT_08s0040g02350  -  Xylan synthase
## VIT_10s0003g00030  -  Dof zinc finger protein DOF5.3
## VIT_03s0097g00380  -  Monofunctional aspartokinase
## VIT_03s0088g00570  -  Glutamine synthetase B1 GLB1
## VIT_14s0219g00280  -  Glycerol-3-phosphate dehydrogenase (NAD+)
## VIT_06s0004g06960  -  BCL2 binding anthogene
## VIT_01s0146g00420  -  No hit
## VIT_06s0004g03420  -  Dof zinc finger protein DOF5.3
## VIT_15s0021g02510  -  GATA transcription factor 2
## VIT_07s0031g02600  -  No hit
## VIT_08s0007g01370  -  Protease inhibitor/seed storage/lipid transfer protein (LTP)
## VIT_08s0056g01190  -  myb domain protein 105
## VIT_04s0008g00560  -  Tetratricopeptide repeat (TPR)-containing
## VIT_12s0059g01750  -  Caffeic acid O-methyltransferase (VvOMT2)
## VIT_17s0000g08460  -  Carbonic anhydrase, chloroplast precursor
## VIT_07s0005g02010  -  Nitrogen regulatory protein PII 1
```

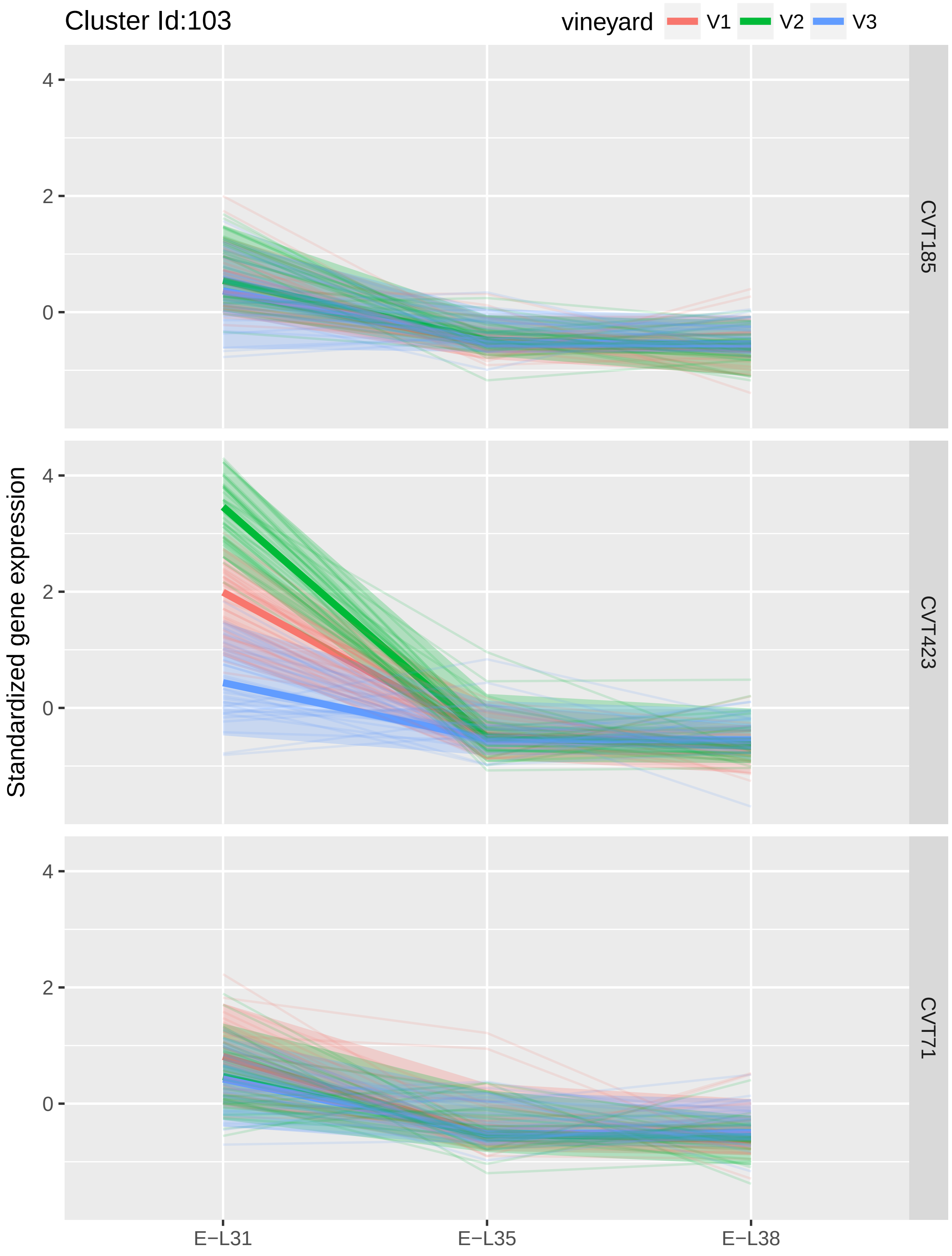

Cluster Id:103
vineyard
V1
V2
V3
Standardized gene expression
4
2
0
CVT185
CVT423
CVT71
E−L31
E−L35
E−L38

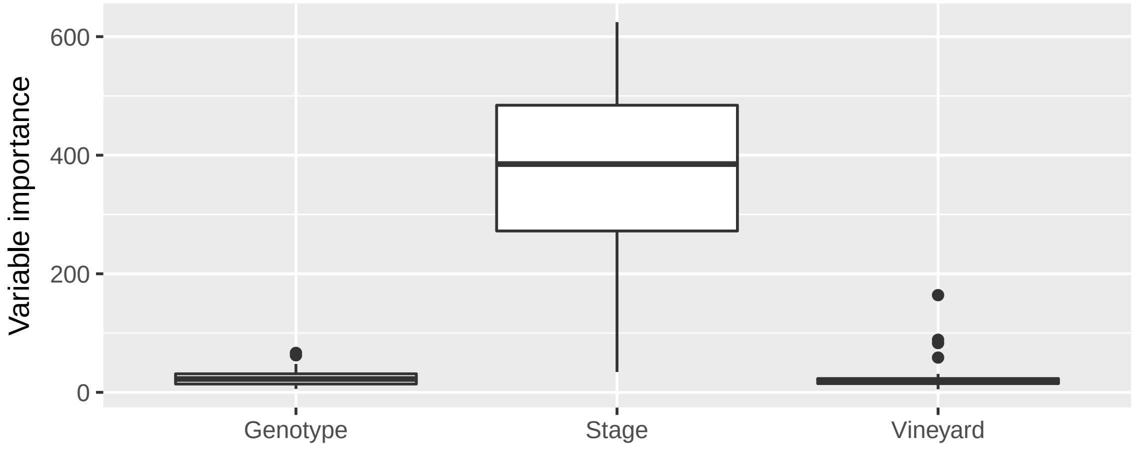

Variable importance
600
400
200
0
Genotype
Stage
Vineyard

Cluster no. 104

```
## Number of genes in the cluster: 63
## Homogeneity Index:    0.82
## Variable importance for Stage:    Median =  485.6  - Rank =  38
## Variable importance for Clone:    Median =  26.89  - Rank =  17
## Variable importance for Vineyard: Median =  26.64  - Rank =  73
##
## Gene ID               Gene Annotation
## VIT_10s0003g01490  -  NIK3 (NSP- interacting  kinase 3)
## VIT_07s0005g03710  -  Casein Kinase I-like 10
## VIT_17s0000g04120  -  Histone deacetylase HDA15
## VIT_18s0072g00170  -  BRI1 (brassinosteroid insensitive 1)
## VIT_14s0066g02020  -  Proton-dependent oligopeptide transport (POT) family protein
## VIT_06s0061g01380  -  Pop3 peptide
## VIT_19s0014g04860  -  ATP synthase g subunit , Mitochondrial
## VIT_16s0022g02070  -  Unknown protein
## VIT_09s0054g01250  -  Cycloartenol synthase
## VIT_05s0020g01820  -  Integral membrane family protein UPF0497
## VIT_02s0241g00120  -  Receptor protein kinase
## VIT_08s0007g05970  -  Purple acid phosphatase 16 ATPAP16/PAP16
## VIT_07s0005g00420  -  Zinc finger (C3HC4-type ring finger)
## VIT_15s0021g01550  -  Unknown protein
## VIT_06s0004g04860  -  Expansin  (VvEXPA6)
## VIT_00s0532g00070  -  CRK10 (cysteine-rich RLK10); kinase
## VIT_06s0009g01080  -  5-methyltetrahydropteroyltriglutamate--homocysteine S-methyltransferas
## VIT_11s0016g01320  -  VvMybPA2
## VIT_02s0025g03390  -  Aquaporin TMP-C
## VIT_09s0070g00500  -  Ubiquitin-specific protease 3 (UBP3)
## VIT_01s0011g02470  -  Calcium-binding protein CML
## VIT_03s0132g00050  -  ATHVA22A (Arabidopsis thaliana HVA22 homologue A)
## VIT_04s0008g06140  -  No hit
## VIT_06s0009g01930  -  5'-AMP-activated protein kinase beta-2 subunit
## VIT_04s0008g06150  -  No hit
## VIT_09s0054g01090  -  Cycloartenol synthase
## VIT_00s0434g00090  -  Aspartic Protease (VvAP49)
## VIT_11s0016g05350  -  RabGAP/TBC domain-containing protein
## VIT_00s0357g00020  -  Beta-hydroxyacyl-ACP dehydratase
## VIT_14s0128g00100  -  Aminotransferase class IV
## VIT_15s0048g01850  -  Xyloglucan endotransglucosylase/hydrolase XTH19
## VIT_06s0004g05080  -  Zinc finger (C3HC4-type ring finger)
## VIT_07s0129g00410  -  Casein kinase II alpha chain 2
## VIT_04s0023g03610  -  MLO4 (mildew resistance locus O 4)
## VIT_06s0004g05400  -  Tropinone reductase
## VIT_16s0022g00640  -  BCL-2-associated athanogene 7 ATBAG7
## VIT_14s0060g00440  -  Glucan endo-1,3-beta-glucosidase-related
## VIT_12s0028g00310  -  Protein kinase family
## VIT_06s0004g08410  -  Bis(5'-adenosyl)-triphosphatase
## VIT_12s0034g01350  -  R protein L6
## VIT_14s0060g02150  -  Rapid ALkalinization Factor RALFL33
## VIT_19s0014g04630  -  S-locus protein kinase
## VIT_07s0005g05610  -  3-ketoacyl-CoA thiolase PED1
## VIT_13s0019g05230  -  NAC domain-containing protein (VvNAC21)
## VIT_16s0050g02540  -  Nodulin MtN3 family
## VIT_18s0001g12500  -  Unknown protein
## VIT_12s0142g00190  -  No hit
```

```
## VIT_04s0008g00030  -  Unknown
## VIT_18s0122g00410  -  PPI1 (proton pump interactor 1
## VIT_13s0019g04230  -  V-type H+-transporting ATPase subunit G
## VIT_05s0094g00300  -  Chitinase class IV
## VIT_01s0011g00830  -  Nodulin
## VIT_05s0020g00640  -  Methyltransferase
## VIT_14s0068g01630  -  Actin related protein 2
## VIT_00s2125g00010  -  C-4 sterol methyl oxidase
## VIT_18s0001g11120  -  Ternary complex factor MIP1
## VIT_02s0012g01210  -  Acetylornithine deacetylase
## VIT_08s0007g03110  -  Transposon protein, Mutator sub-class
## VIT_15s0024g01010  -  Amidase
## VIT_07s0005g03760  -  Phytochelatin synthase 1 (PCS1)
## VIT_07s0141g00240  -  NADH dehydrogenase (ubiquinone) flavoprotein 2
## VIT_08s0058g00740  -  Iron-responsive transporter
## VIT_11s0016g02950  -  Profilin 1 (PRO1) (PFN1) (PRF1) / allergen Ara t 8
```

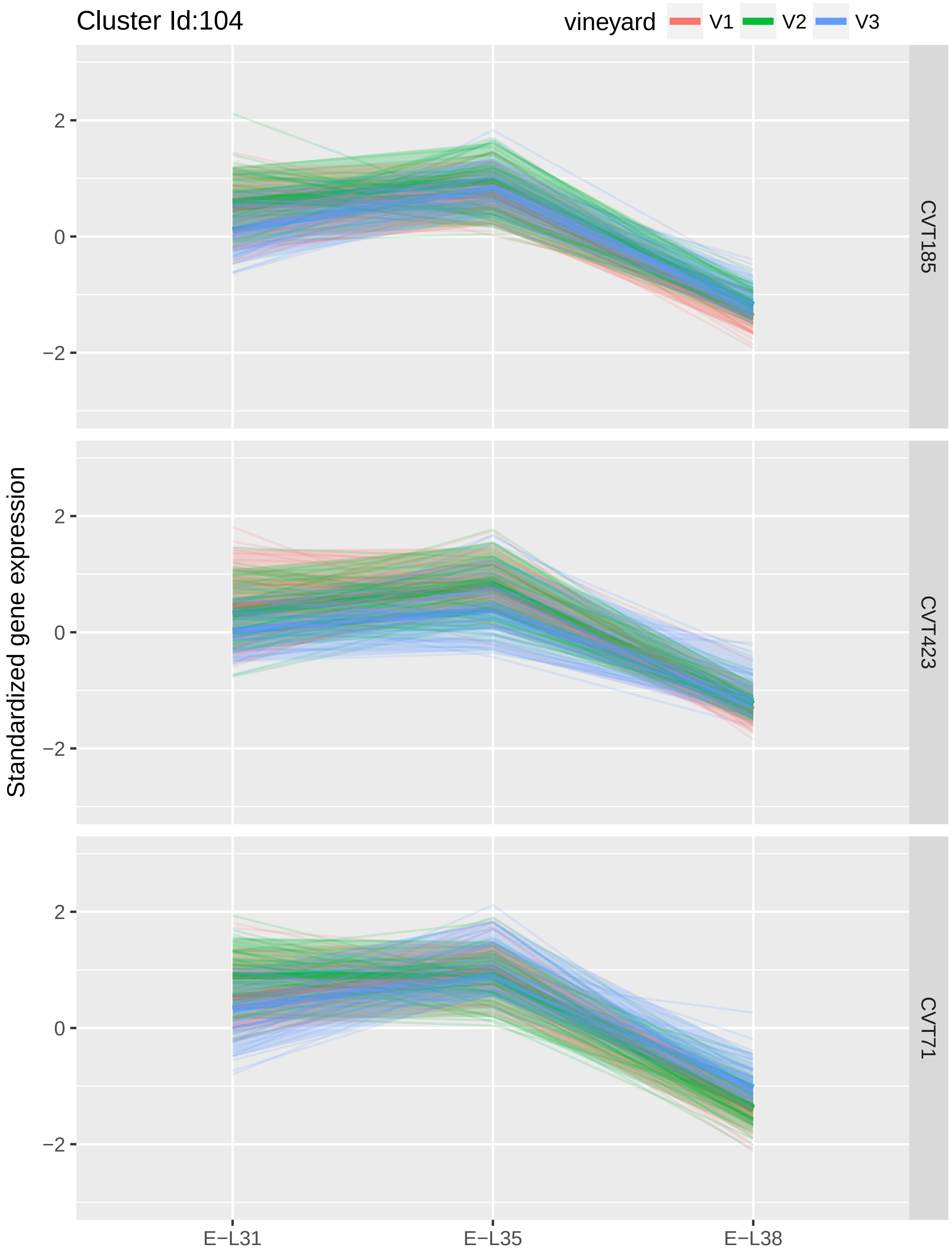

Cluster Id:104
vineyard
V1
V2
V3
Standardized gene expression
2
0
−2
CVT185
CVT423
CVT71
E−L31
E−L35
E−L38

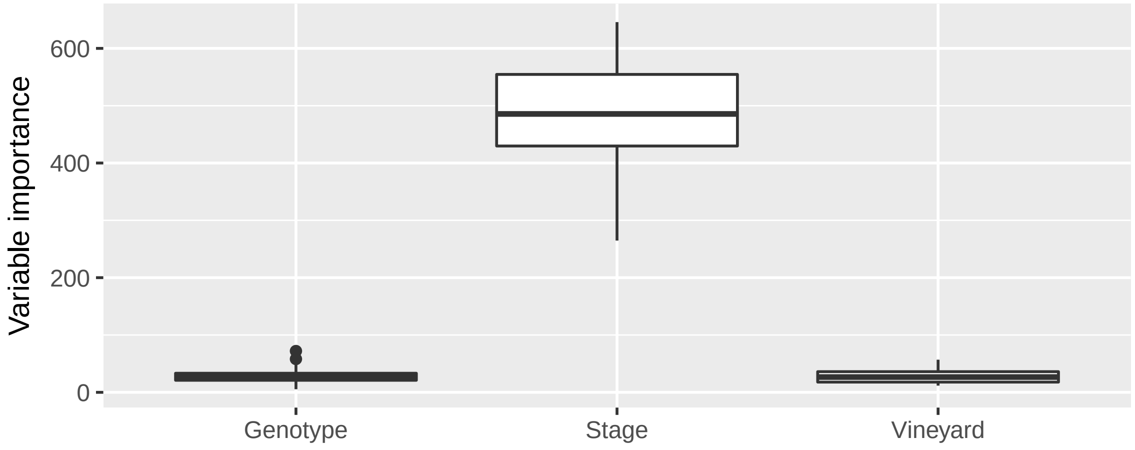

Variable importance
600
400
200
0
Genotype
Stage
Vineyard

Cluster no. 105

```
## Number of genes in the cluster: 81
## Homogeneity Index:     0.6
## Variable importance for Stage:    Median =  228.2  - Rank =  83
## Variable importance for Clone:    Median =  18.74  - Rank =  58
## Variable importance for Vineyard: Median =  73.59  - Rank =  25
##
## Gene ID               Gene Annotation
## VIT_13s0158g00180  -  Hydrolase, alpha/beta fold
## VIT_06s0061g00090  -  DnaJ homolog, subfamily C, member 7
## VIT_10s0116g01550  -  Unknown protein
## VIT_19s0085g01150  -  Isocitrate dehydrogenase, chloroplast precursor
## VIT_18s0122g01350  -  Small nuclear ribonucleoprotein D3
## VIT_01s0182g00050  -  ribulose-1,5 bisphosphate carboxylase oxygenase large subunit N-methyl
## VIT_04s0023g00920  -  DCL2 (DICER 2)
## VIT_12s0034g01230  -  Thaumatin ATLP-1
## VIT_04s0008g02830  -  Galactokinase like protein
## VIT_11s0016g00610  -  Unknown
## VIT_04s0008g06040  -  Aspartate aminotransferase, chloroplast precursor
## VIT_05s0029g01200  -  Methionyl-tRNA synthetase
## VIT_06s0004g05470  -  Sterol 4-alpha-methyl-oxidase 2 (SMO2)
## VIT_09s0002g06100  -  Mov34
## VIT_12s0057g01330  -  Zinc finger (C3HC4-type ring finger)
## VIT_09s0002g04600  -  Ras-related protein Rab-5A
## VIT_17s0053g00680  -  Argonaute protein (AGO1)
## VIT_08s0007g05700  -  Cellular retinaldehyde-binding/triple function, C-terminal
## VIT_14s0108g01460  -  Circadian clock coupling factor
## VIT_05s0062g01110  -  15-cis-<U+03B6>-carotene isomerase (Z-ISO) (VvZISO1)
## VIT_08s0058g01250  -  Unknown protein
## VIT_05s0020g03840  -  Unknown protein
## VIT_06s0004g03250  -  Protein disulfide isomerase
## VIT_15s0046g01540  -  F-box family protein
## VIT_16s0022g02100  -  Embryo-specific 3
## VIT_17s0000g00250  -  Unknown protein
## VIT_15s0046g00140  -  ORMDL protein
## VIT_07s0031g00180  -  RNA recognition motif (RRM)-containing protein
## VIT_18s0001g11820  -  LIM domain containing protein
## VIT_08s0007g02150  -  Receptor ser/thr protein kinase
## VIT_14s0030g02310  -  Auxin-induced protein 22D
## VIT_08s0007g06390  -  Enoyl-[acyl-carrier-protein] reductase [NADH], chloroplast precursor
## VIT_14s0060g00920  -  Phospholipase/Carboxylesterase
## VIT_03s0063g02190  -  HVA22K (HVA22-like protein K)
## VIT_05s0020g02840  -  Short-chain dehydrogenase/reductase
## VIT_14s0171g00310  -  SET domain-containing protein
## VIT_04s0008g02930  -  Oligouridylate binding protein 1B UBP1B
## VIT_01s0137g00400  -  Unknown protein
## VIT_05s0094g01450  -  Unknown
## VIT_19s0014g02720  -  Unknown protein
## VIT_02s0025g04530  -  DNA-directed RNA polymerase II subunit A
## VIT_16s0039g00260  -  Pectate lyase
## VIT_18s0122g00870  -  Zinc finger (C3HC4-type ring finger)
## VIT_15s0021g01540  -  AvrPto-dependent Pto-interacting protein 3
## VIT_12s0035g01220  -  Unknown protein
## VIT_09s0054g01590  -  Membrane related protein CP5
## VIT_06s0004g01780  -  Serine/threonine-protein kinase OXI1
```

```
## VIT_11s0016g02890  -  Dual specificity protein phosphatase
## VIT_02s0087g00910  -  (9,10) (9`,10`) cleavage dioxygenase (CCD4) (VvCCD4a)
## VIT_13s0064g01090  -  Zinc finger (CCCH-type) family protein
## VIT_03s0017g01020  -  Methyl-CpG-binding domain 8 MBD08
## VIT_07s0141g00740  -  fimbrin 1
## VIT_10s0042g00140  -  Dihydroorotate dehydrogenase
## VIT_13s0067g03510  -  ARR9 typeA
## VIT_06s0080g00470  -  RNA-binding region RNP-1
## VIT_10s0003g02620  -  AWPM19
## VIT_02s0025g00260  -  Polygalacturonase GH28
## VIT_01s0011g00470  -  Metallophosphatase
## VIT_07s0031g02420  -  F-box domain containing protein
## VIT_04s0044g00250  -  Beta-cyanoalanine synthase 1
## VIT_04s0023g01480  -  Dimethylaniline monooxygenase, N-oxide-forming
## VIT_06s0004g03340  -  Ribose-5-phosphate isomerase
## VIT_18s0089g01240  -  ATP-dependent Clp protease ATP-binding subunit ClpX1 (CLPX)
## VIT_17s0000g04310  -  DEAD (Asp-Glu-Ala-Asp) box polypeptide 18
## VIT_02s0025g01010  -  Beta-1,3-galactosyltransferase sqv-2
## VIT_05s0049g00960  -  Cryptochrome 2
## VIT_08s0007g05810  -  Calmodulin-binding, plant
## VIT_09s0002g02200  -  Root hair defective 3 GTP-binding (RHD3)
## VIT_12s0034g00970  -  Peptidase C50, separase
## VIT_08s0040g01940  -  Zinc finger (C3HC4-type ring finger)
## VIT_10s0003g03080  -  Minichromosome maintenance protein 3
## VIT_08s0007g05830  -  No hit
## VIT_00s0357g00130  -  Annexin ANN6
## VIT_16s0039g00610  -  Serine/threonine-protein kinase ppk15
## VIT_07s0141g00720  -  fimbrin 1
## VIT_08s0007g03140  -  Rac GTPase activating protein 1
## VIT_07s0005g03780  -  Alcohol oxidase
## VIT_00s1365g00010  -  fimbrin 1
## VIT_19s0014g03300  -  NAC domain-containing protein (VvNAC18)
## VIT_05s0020g01540  -  Protein arginine N-methyltransferase
## VIT_19s0015g00860  -  Oligopeptide transporter 1
```

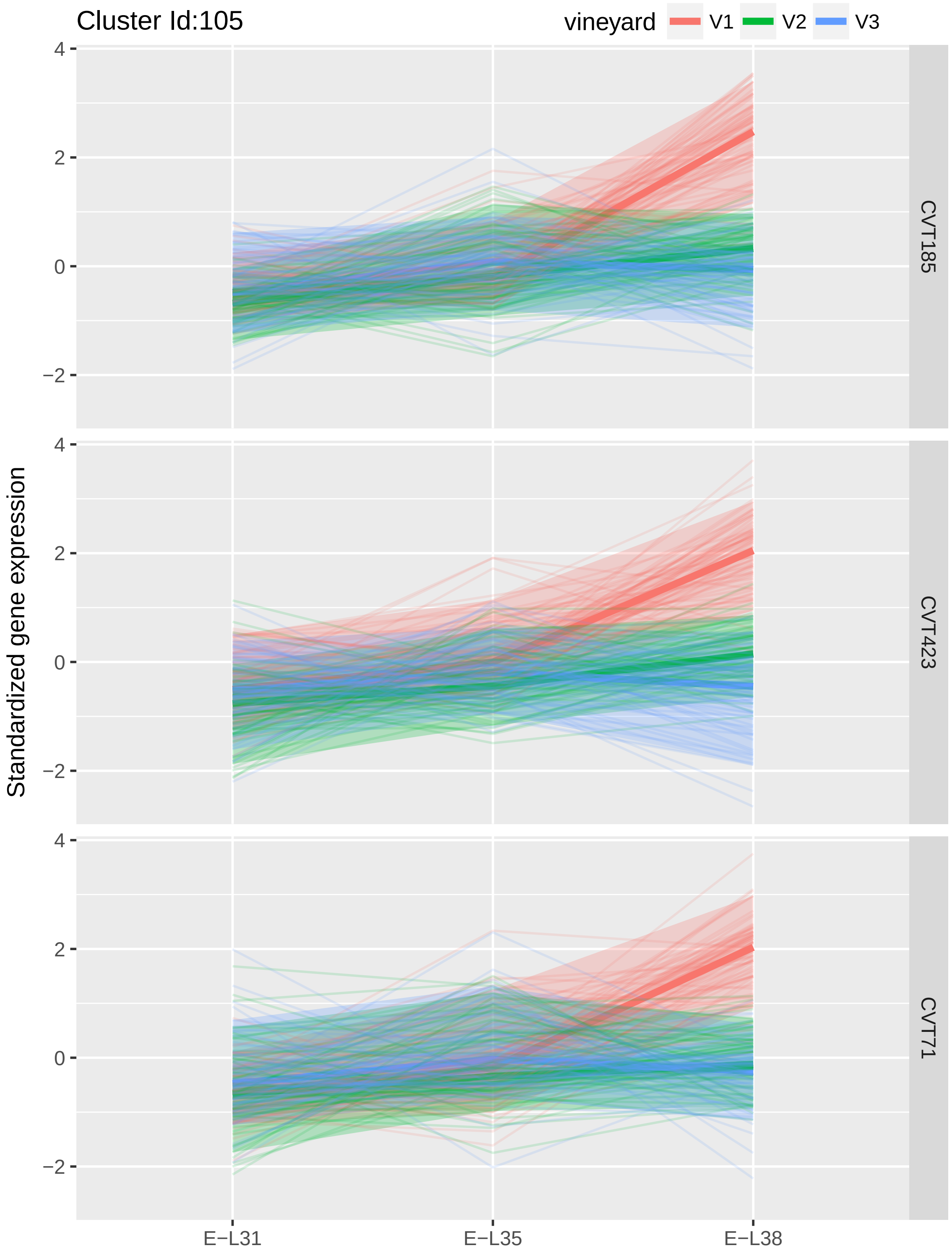
Cluster Id:105
vineyard
V1
V2
V3
Standardized gene expression
CVT185
CVT423
CVT71
4
2
0
−2
E−L31
E−L35
E−L38

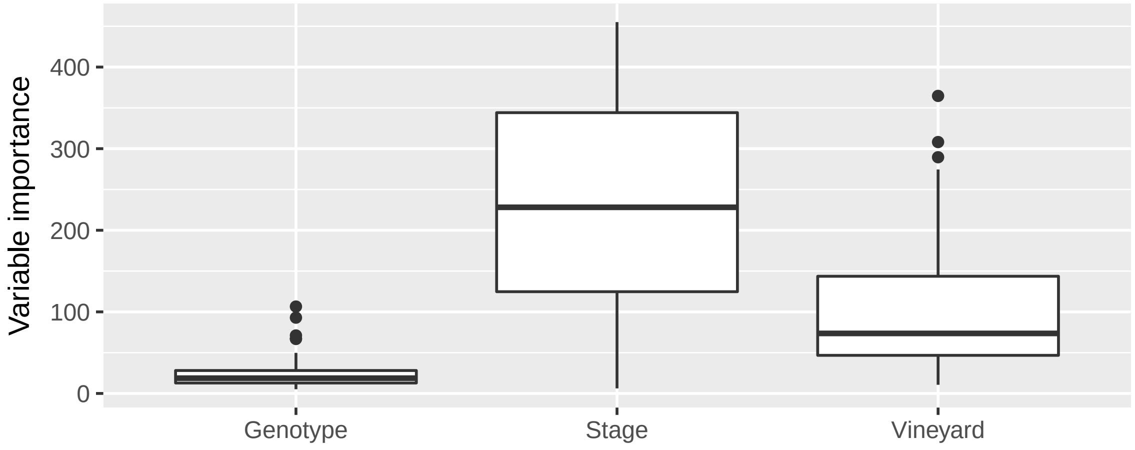
Variable importance
400
300
200
100
0
Genotype
Stage
Vineyard

Cluster no. 106

```
## Number of genes in the cluster: 71
## Homogeneity Index:    0.72
## Variable importance for Stage:    Median =  216.2  - Rank =  84
## Variable importance for Clone:    Median =  15.19  - Rank =  71
## Variable importance for Vineyard: Median =  112.7  - Rank =  15
##
## Gene ID              Gene Annotation
## VIT_05s0062g01070  -  Receptor protein kinase
## VIT_07s0104g01560  -  Transcription termination factor mitochondrial mTERF
## VIT_07s0141g00780  -  Pentatricopeptide (PPR) repeat-containing protein
## VIT_02s0025g03540  -  Tubulin beta-6 chain
## VIT_06s0061g01340  -  Phosphoglycerate mutase
## VIT_03s0038g00910  -  Ribosome-binding factor A, chloroplast precursor
## VIT_10s0042g01070  -  Zinc finger (C3HC4-type ring finger)
## VIT_04s0023g02640  -  CDK5RAP1
## VIT_17s0000g07760  -  ER degradation enhancer, mannosidase alpha-like 1
## VIT_14s0083g00620  -  NIK1 (NSP- interacting  kinase 1)
## VIT_08s0056g00510  -  KH domain-containing protein
## VIT_01s0011g05880  -  RDR1 (RNA-dependent RNA polymerase 1)
## VIT_05s0077g01030  -  Unknown
## VIT_02s0012g01250  -  Carnitine/acylcarnitine carrier, Mitochondrial
## VIT_11s0118g00290  -  Ubiquitin-specific protease 22 (UBP22)
## VIT_05s0102g00730  -  Integral membrane HRF1
## VIT_10s0116g00100  -  Strubbelig receptor family 8
## VIT_17s0000g08240  -  HT1 (high leaf temperature 1)
## VIT_11s0016g04650  -  Drug/metabolite transporter DMT family transporter
## VIT_02s0025g01130  -  Phytochrome A supressor spa1 (SPA1)
## VIT_06s0004g03700  -  Phototropin-1
## VIT_09s0002g03870  -  RPS5 (resistant to p. syringae 5)
## VIT_13s0067g02420  -  No hit
## VIT_18s0001g14680  -  Vacuolar protein sorting 35
## VIT_12s0028g00040  -  Unknown protein
## VIT_13s0019g04090  -  Ubiquitin-conjugating enzyme E2 C
## VIT_10s0003g01680  -  Trehalose synthase
## VIT_12s0059g01150  -  Dehydrogenase
## VIT_14s0066g01560  -  Cyclic nucleotide-gated ion channel 18
## VIT_17s0000g08600  -  Signal recognition particle receptor beta subunit
## VIT_01s0026g00300  -  Zinc finger (C3HC4-type ring finger)
## VIT_06s0080g01110  -  Transcription factor (E2F) E2F1
## VIT_05s0049g00890  -  Serine/threonine-protein phosphatase PP1
## VIT_10s0116g00050  -  Receptor protein kinase
## VIT_14s0060g01160  -  Cyclin-related
## VIT_10s0116g00910  -  CERK1 (chitin elicitor receptor kinase 1)
## VIT_11s0016g05000  -  Dynein light chain LC8-type
## VIT_05s0020g01530  -  Alpha-1,6-xylosyltransferase
## VIT_05s0029g01540  -  Ankyrin repeat
## VIT_18s0001g11140  -  Peptidase S9, prolyl oligopeptidase
## VIT_17s0000g03070  -  R protein MLA10
## VIT_18s0072g00690  -  DNA-binding protein GT-1-related
## VIT_15s0048g00490  -  Methionine aminopeptidase
## VIT_02s0012g01640  -  Inositol 1,3,4-trisphosphate 5/6-kinase.
## VIT_08s0007g03600  -  KCBP- interacting  protein kinase
## VIT_14s0081g00540  -  No hit
## VIT_01s0026g01510  -  Protein kinase 5'-AMP-activated
```

```
## VIT_13s0064g00380  -  Unknown protein
## VIT_05s0029g01500  -  Ankyrin repeat protein
## VIT_18s0001g09090  -  NPGR1 (no pollen germination related 1)
## VIT_01s0011g03210  -  Aspartic Protease (VvAP1)
## VIT_14s0068g00690  -  OTU cysteine protease
## VIT_11s0016g01220  -  Auxin-binding protein ABP19a precursor
## VIT_18s0001g09360  -  Hydrogenobyrinic acid a,c-diamide synthase
## VIT_01s0010g01850  -  Regulator of nonsense transcripts 1
## VIT_18s0001g10090  -  Kinesin protein FRA1 (fragile fiber 1)
## VIT_08s0007g04670  -  Glutamine synthetase
## VIT_08s0007g01920  -  MYB divaricata
## VIT_15s0024g01540  -  Glutathione S-transferase 8 GSTU8
## VIT_12s0035g00750  -  Disease resistance protein
## VIT_06s0004g05910  -  Organic anion transmembrane transporter
## VIT_03s0063g00100  -  Unknown protein
## VIT_15s0046g01940  -  Mechanosensitive ion channel
## VIT_11s0016g00050  -  Unknown protein
## VIT_17s0000g06440  -  Galactoside 2-alpha-L-fucosyltransferase
## VIT_18s0001g10250  -  NAC domain containing protein 19
## VIT_03s0038g04250  -  Cellulose synthase CESA2
## VIT_07s0005g02060  -  Boron transporter-like protein 1
## VIT_13s0067g02480  -  SOS2 (salt overly sensitive 2)
## VIT_00s0370g00060  -  Epoxide hydrolase 2
## VIT_14s0108g00760  -  Mini zinc finger 2 MIF2
```

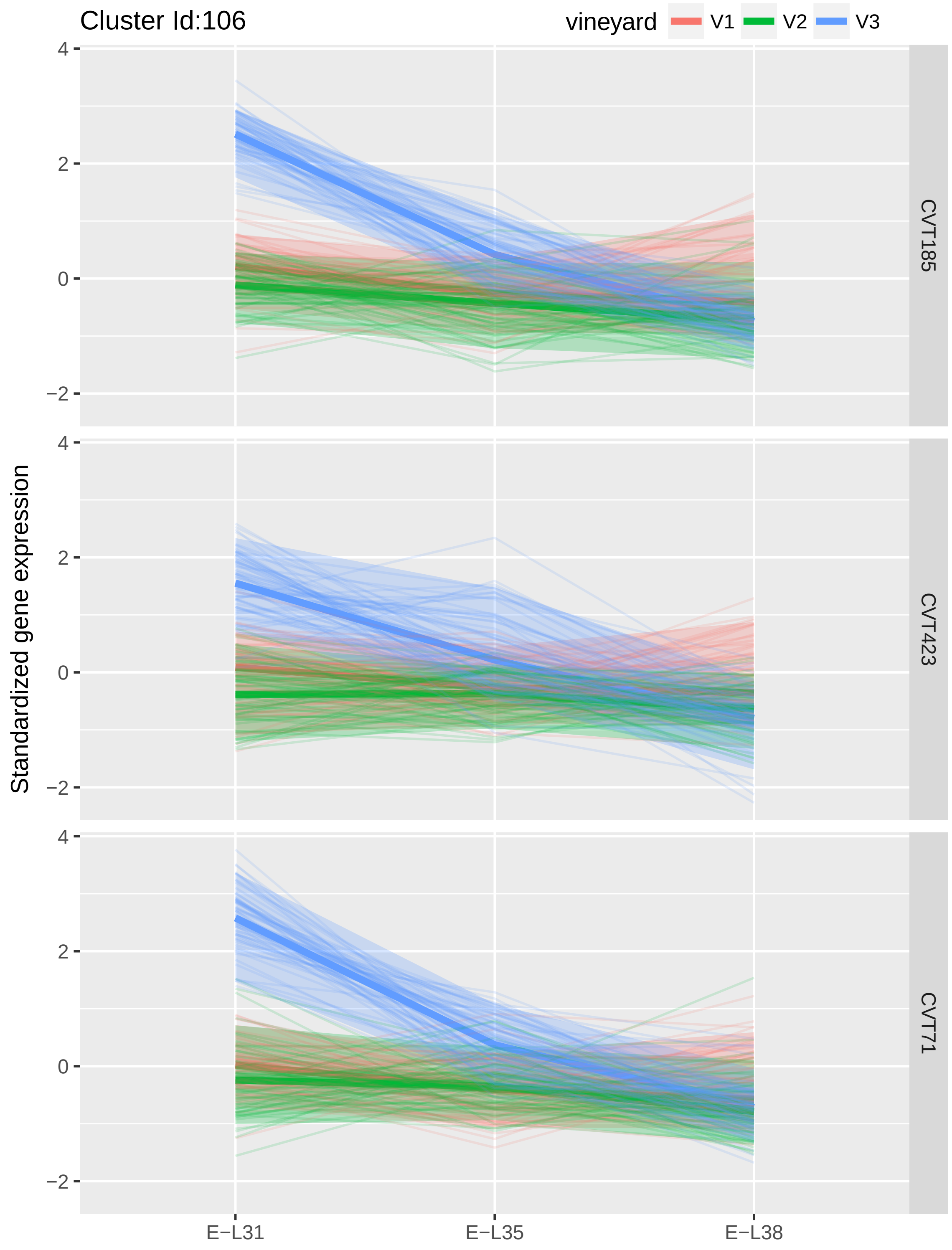

Cluster Id:106
vineyard
V1
V2
V3
Standardized gene expression
CVT185
CVT423
CVT71
4
2
0
−2
E−L31
E−L35
E−L38

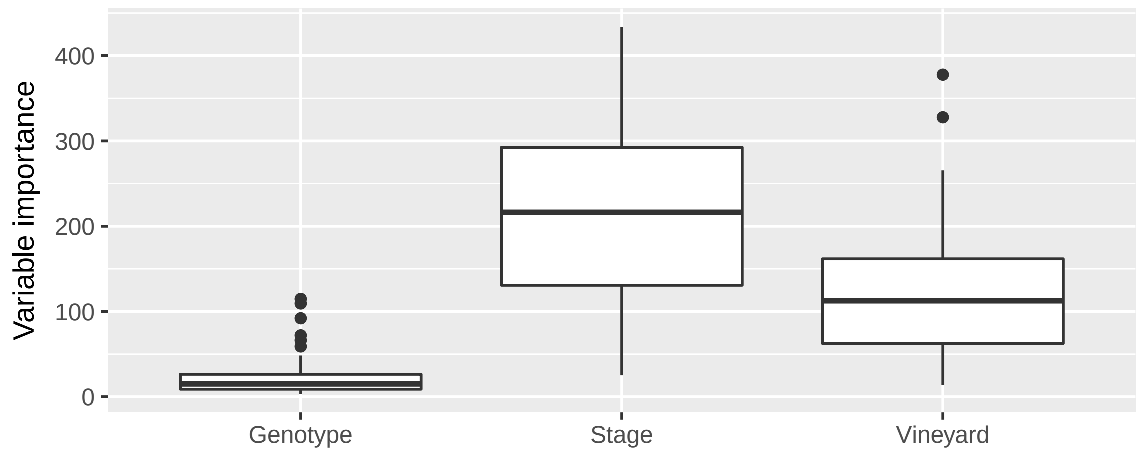

Variable importance
400
300
200
100
0
Genotype
Stage
Vineyard

Cluster no. 107

```
## Number of genes in the cluster: 107
## Homogeneity Index:    0.74
## Variable importance for Stage:    Median =  249.9  - Rank =  78
## Variable importance for Clone:    Median =  21.42  - Rank =  40
## Variable importance for Vineyard: Median =  45.05  - Rank =  42
##
## Gene ID               Gene Annotation
## VIT_11s0016g02080  -  Esterase
## VIT_07s0005g05510  -  Kinase-like protein TMKL1
## VIT_03s0038g00960  -  Transcription factor TFIIF
## VIT_15s0046g03200  -  ATP-dependent RNA helicase DDX6/DHH1
## VIT_00s0483g00010  -  Nucleobase-ascorbate transporter 11 (NAT11)
## VIT_14s0083g00990  -  IMP dehydrogenase
## VIT_15s0048g01260  -  fructokinase 1
## VIT_16s0050g01020  -  Protein kinase family
## VIT_11s0016g00280  -  Bifunctional lysine-ketoglutarate reductase/saccharopine dehydrogenase
## VIT_03s0063g00860  -  Serine carboxypeptidase
## VIT_01s0026g00130  -  Ulp1 protease family
## VIT_00s0813g00010  -  Nucleobase-ascorbate transporter 11 (NAT11)
## VIT_18s0001g01990  -  Pseudouridylate synthase
## VIT_18s0075g00330  -  Sucrose-phosphate synthase
## VIT_08s0007g02790  -  Unknown protein
## VIT_07s0005g03830  -  Calmodulin-binding protein
## VIT_02s0012g01930  -  RCD1 (radical-induced cell death1)
## VIT_13s0084g00340  -  Unknown protein
## VIT_14s0006g00520  -  Glucan endo-1,3-beta-glucosidase 3 precursor
## VIT_12s0055g00440  -  No hit
## VIT_19s0014g03080  -  Regulator of chromosome condensation (RCC1)
## VIT_08s0040g00820  -  CYP94A1
## VIT_12s0034g01170  -  Patatin
## VIT_14s0083g00910  -  Auxin-independent growth promoter
## VIT_00s1251g00010  -  UDP-glucosyl transferase 85A2
## VIT_04s0008g03350  -  Unknown protein
## VIT_04s0043g00720  -  Transketolase
## VIT_13s0067g01800  -  Histidine acid phosphatase
## VIT_14s0066g01180  -  Phospholipid-transporting ATPase
## VIT_01s0026g00290  -  No hit
## VIT_18s0001g12560  -  Sugar transporter ERD6-like 6
## VIT_01s0026g01090  -  Unknown
## VIT_14s0128g00330  -  Mevalonate kinase (MK)
## VIT_04s0023g02280  -  S-adenosyl-L-methionine:salicylic acid carboxyl methyltransferase
## VIT_12s0035g00800  -  DEAH (Asp-Glu-Ala-His) box polypeptide 36
## VIT_17s0000g08660  -  Anthranilate phosphoribosyltransferase, chloroplast precursor
## VIT_19s0085g00720  -  3-deoxy-8-phosphooctulonate synthase.
## VIT_07s0104g01300  -  No hit
## VIT_02s0025g04210  -  UDP-glucose 4-epimerase
## VIT_13s0067g01000  -  PHD finger protein alfin
## VIT_08s0058g00890  -  WD40
## VIT_04s0008g06280  -  Calcium-binding EF hand
## VIT_03s0038g00480  -  TIFY gene family (VvZMLI)
## VIT_07s0129g01110  -  Avr9/Cf-9 rapidly elicited protein 189
## VIT_00s0391g00010  -  Pentatricopeptide (PPR) repeat-containing
## VIT_05s0049g00970  -  No hit
## VIT_03s0038g01050  -  Phenylalanine-tRNA ligase
```

```
## VIT_07s0130g00310  -  Unknown protein
## VIT_18s0001g09430  -  Tetratricopeptide repeat (TPR)-containing
## VIT_12s0055g00930  -  Heavy-metal-associated domain-containing protein
## VIT_05s0049g00980  -  Cofilin
## VIT_11s0016g04220  -  Copper-binding family protein
## VIT_07s0130g00470  -  Ankyrin repeat
## VIT_13s0019g00520  -  RAB GTPase ARA3
## VIT_05s0020g02290  -  Endonuclease
## VIT_17s0000g08290  -  Dof zinc finger protein DOF5.6
## VIT_17s0000g02270  -  Vacuolar protein sorting-associated protein 26
## VIT_02s0087g00190  -  SH3 domain-containing protein 2
## VIT_06s0004g00440  -  No hit
## VIT_18s0001g09370  -  Arginine N-methyltransferase 2
## VIT_14s0108g01020  -  Expansin  (VvEXPA16)
## VIT_18s0001g13000  -  Adaptor-related protein complex AP-4, mu 1
## VIT_04s0023g03240  -  Transcription factor TFIIF
## VIT_10s0003g00330  -  HSL1 (HAESA-like 1)
## VIT_06s0009g01320  -  L-ascorbate oxidase
## VIT_08s0007g06520  -  Actin beta/gamma 1
## VIT_13s0156g00480  -  R protein disease resistance protein
## VIT_13s0067g01980  -  Proteinase inhibitor (LUTI)
## VIT_11s0037g00110  -  Transport protein particle (TRAPP) component Bet3
## VIT_04s0043g00740  -  Cytoplasmic FMR1 interacting protein
## VIT_10s0003g01070  -  Unknown protein
## VIT_18s0001g08870  -  Unknown
## VIT_13s0019g04120  -  Rhomboid
## VIT_01s0011g00120  -  Unknown protein
## VIT_14s0066g01170  -  MAP4K alpha1
## VIT_05s0062g01280  -  Cyclo-DOPA 5-O-glucosyltransferase
## VIT_01s0011g01780  -  F-box protein PP2-B12 (Protein phloem protein 2-like B12)
## VIT_12s0059g01030  -  Dentin sialophosphoprotein
## VIT_07s0031g02310  -  Leucine-rich repeat-containing protein 40
## VIT_02s0012g00600  -  Vacuolar protein sorting 55
## VIT_12s0059g00550  -  Ankyrin repeat and BTB (POZ) domain containing
## VIT_15s0046g01450  -  Alpha-L-fucosidase
## VIT_01s0011g05850  -  SEC14 cytosolic factor
## VIT_18s0001g00370  -  Ceramidase
## VIT_00s0394g00030  -  Triacylglycerol lipase
## VIT_06s0004g07910  -  Ovate family protein 3 OFP3
## VIT_03s0063g02480  -  Guanine nucleotide-binding protein beta subunit
## VIT_10s0003g00620  -  Lipase GDSL
## VIT_18s0001g05660  -  Tubulin folding cofactor E
## VIT_18s0001g07290  -  Unknown protein
## VIT_16s0098g00750  -  Calcium ion binding
## VIT_09s0002g02170  -  No hit
## VIT_04s0079g00520  -  Armadillo/beta-catenin repeat
## VIT_02s0087g00240  -  Unknown protein
## VIT_18s0001g12480  -  Metal transporter CNNM4 (Cyclin-M4)
## VIT_04s0023g00940  -  Phospholipase A1
## VIT_00s2604g00010  -  ADP-ribosylation factor
## VIT_14s0108g01130  -  EXS family protein / ERD1/XPR1/SYG1
## VIT_14s0066g02590  -  Nitrate transporter NRT1-5
## VIT_03s0038g03430  -  Expansin  (VvEXLA1)
## VIT_05s0020g03320  -  Copper amine oxidase
## VIT_08s0007g08200  -  MATE efflux family protein
```

```
## VIT_13s0067g00990  -  Heat shock protein-related
## VIT_06s0004g01270  -  Callose synthase catalytic subunit
## VIT_02s0025g03620  -  Glutathione S-transferase 1 GSTT1
## VIT_18s0001g10160  -  Wuschel homeobox 4
## VIT_18s0072g00660  -  Nodulin MtN21 family
```

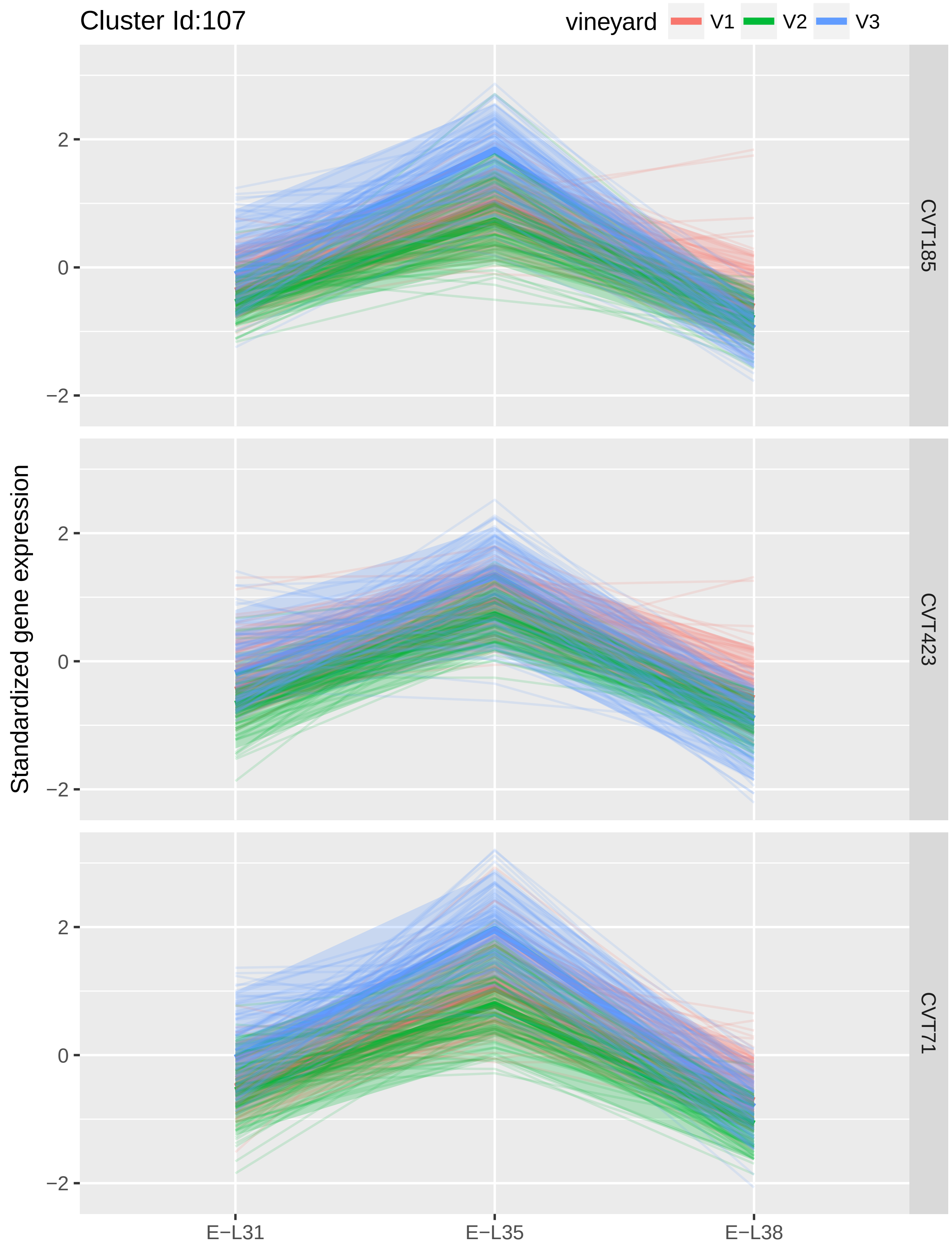
Cluster Id:107
vineyard
V1
V2
V3
Standardized gene expression
2
0
−2
CVT185
CVT423
CVT71
E−L31
E−L35
E−L38

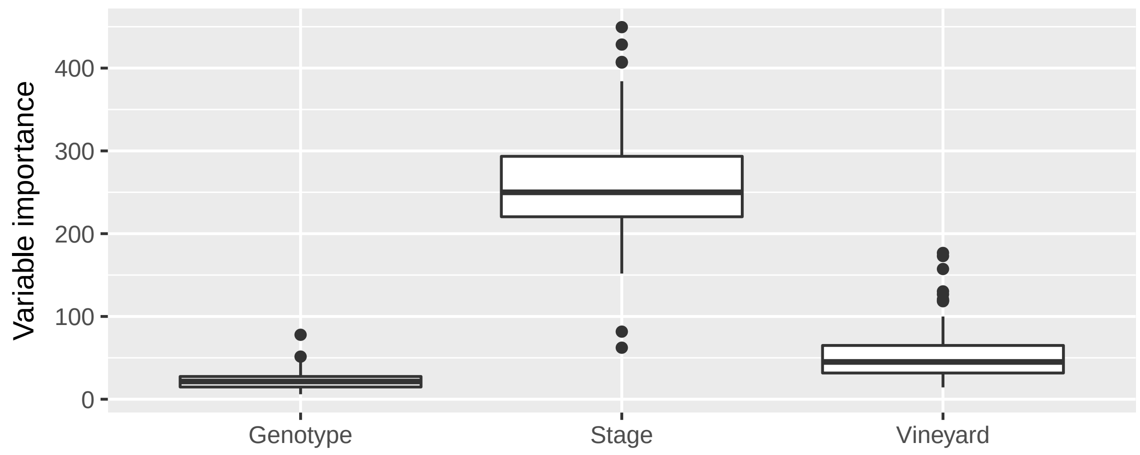
Variable importance
400
300
200
100
0
Genotype
Stage
Vineyard

Cluster no. 108

```
## Number of genes in the cluster: 64
## Homogeneity Index:    0.76
## Variable importance for Stage:    Median =   29.9  - Rank =  112
## Variable importance for Clone:    Median =  18.38  - Rank =  61
## Variable importance for Vineyard: Median =  272.5  - Rank =  2
##
## Gene ID               Gene Annotation
## VIT_16s0100g00780  -  Stilbene synthase (VvSTS10)
## VIT_11s0016g03080  -  Clavata1 receptor kinase (CLV1)
## VIT_16s0100g01160  -  Stilbene synthase (VvSTS45)
## VIT_10s0042g00700  -  Leucine-rich repeat receptor-like protein kinase 1
## VIT_16s0100g00770  -  Stilbene synthase (VvSTS9)
## VIT_16s0039g01100  -  Phenylalanin ammonia-lyase [Vitis vinifera]
## VIT_16s0100g00750  -  Stilbene synthase (VvSTS7)
## VIT_04s0044g00220  -  Monooxygenase
## VIT_16s0100g00990  -  Stilbene synthase (VvSTS27)
## VIT_16s0100g00950  -  Stilbene synthase (VvSTS25)
## VIT_05s0094g00260  -  Chitinase, class IV [Vitis vinifera]
## VIT_10s0042g00600  -  Leucine Rich Repeat receptor-like kinase
## VIT_00s0391g00070  -  3-deoxy-D-arabino-heptulosonate 7-phosphate synthase
## VIT_16s0100g00760  -  Stilbene synthase (VvSTS8)
## VIT_16s0100g00910  -  Stilbene synthase (VvSTS21)
## VIT_16s0100g00830  -  Stilbene synthase (VvSTS15)
## VIT_16s0100g01170  -  Stilbene synthase (VvSTS46)
## VIT_16s0100g01030  -  Stilbene synthase (VvSTS31)
## VIT_16s0100g01120  -  Stilbene synthase (VvSTS39)
## VIT_11s0016g02070  -  basic helix-loop-helix (bHLH) family
## VIT_16s0100g01200  -  Stilbene synthase (VvSTS48)
## VIT_16s0100g01150  -  Stilbene synthase (VvSTS43)
## VIT_18s0001g05310  -  F-box domain containing protein
## VIT_16s0039g01130  -  Phenylalanin ammonia-lyase [Vitis vinifera]
## VIT_16s0039g01170  -  Phenylalanine ammonium lyase
## VIT_16s0100g01140  -  Stilbene synthase (VvSTS42)
## VIT_11s0065g00350  -  Trans-cinnamate 4-monooxygenase
## VIT_16s0100g01130  -  Stilbene synthase (VvSTS41)
## VIT_16s0100g00880  -  Stilbene synthase (VvSTS19)
## VIT_02s0033g00270  -  Pinoresinol-lariciresinol reductase
## VIT_10s0042g00870  -  Stilbene synthase (VvSTS2)
## VIT_16s0039g01110  -  Phenylalanin ammonia-lyase [Vitis vinifera]
## VIT_16s0100g00860  -  Stilbene synthase (VvSTS18)
## VIT_16s0100g00930  -  Stilbene synthase (VvSTS23)
## VIT_09s0002g06890  -  Dicyanin blue copper protein
## VIT_16s0100g00810  -  Stilbene synthase (VvSTS13)
## VIT_16s0100g01010  -  Stilbene synthase (VvSTS29)
## VIT_16s0100g00940  -  Stilbene synthase (VvSTS24)
## VIT_08s0007g05490  -  Pyruvate kinase
## VIT_08s0105g00200  -  U-box domain-containing protein
## VIT_10s0042g00860  -  Stilbene Synthase (VvSTS1)
## VIT_16s0100g00900  -  Stilbene synthase (VvSTS20)
## VIT_07s0031g01710  -  WRKY Transcription Factor (VvWRKY22)
## VIT_16s0039g01120  -  Phenylalanin ammonia-lyase [Vitis vinifera]
## VIT_10s0042g00850  -  Stilbene Synthase (VvSTS1)
## VIT_16s0100g01070  -  Stilbene synthase (VvSTS35)
## VIT_16s0100g00840  -  Stilbene synthase (VvSTS16)
```

```
## VIT_10s0042g00840  -  Stilbene Synthase (VvSTS1)
## VIT_18s0166g00250  -  No hit
## VIT_12s0028g00270  -  WRKY Transcription Factor (VvWRKY35)
## VIT_10s0003g04080  -  Unknown
## VIT_16s0100g01020  -  Stilbene synthase (VvSTS30)
## VIT_16s0100g01000  -  Stilbene synthase (VvSTS28)
## VIT_16s0039g01320  -  Phenylalanin ammonia-lyase [Vitis vinifera]
## VIT_05s0094g00220  -  Chitinase class IV
## VIT_06s0004g01000  -  Dirigent protein
## VIT_16s0100g00800  -  Stilbene synthase (VvSTS12)
## VIT_16s0100g00920  -  Stilbene synthase (VvSTS22)
## VIT_18s0001g05060  -  2,3-bisphosphoglycerate-dependent phosphoglycerate mutase
## VIT_16s0039g02380  -  Disease resistance family protein
## VIT_02s0087g00710  -  Abscisic acid 8` hydroxylase (CYP707A1) (VvA8H-CYP707A1)
## VIT_15s0046g00360  -  Aspartic Protease (VvAP37)
## VIT_01s0127g00070  -  Nitrate transporter2.5
## VIT_18s0001g11620  -  WAK receptor protein kinase
```

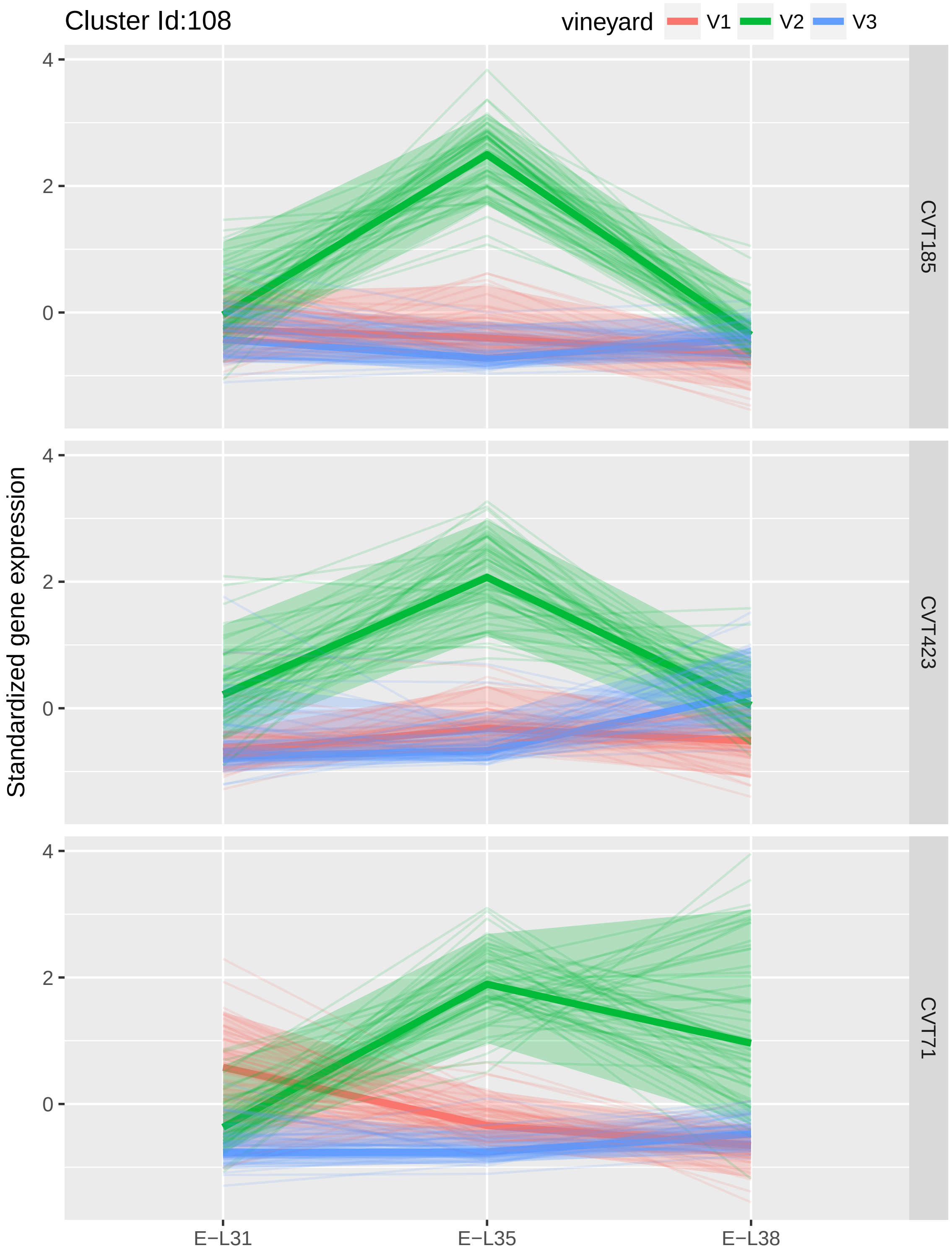
Cluster Id:108
vineyard
V1
V2
V3
CVT185
CVT423
CVT71
Standardized gene expression
4
2
0
E−L31
E−L35
E−L38

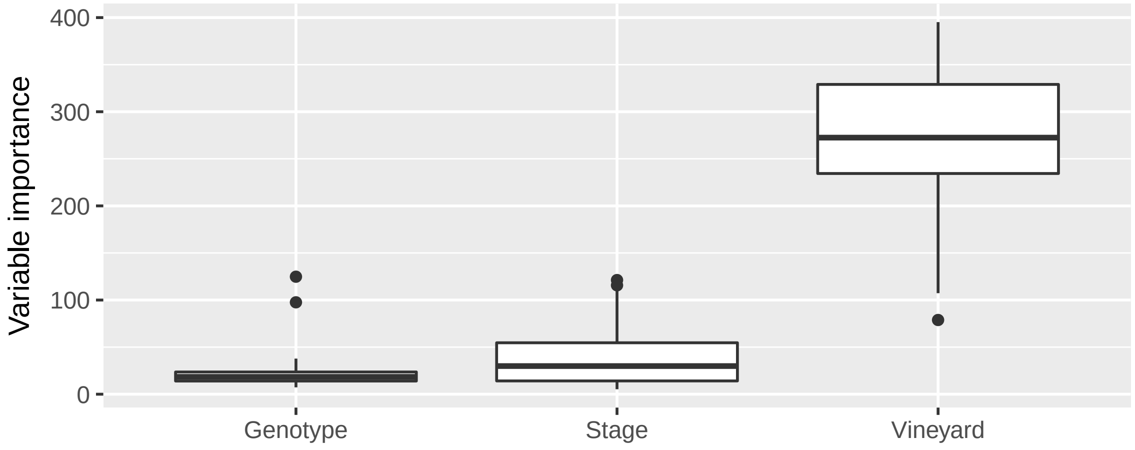
Variable importance
400
300
200
100
0
Genotype
Stage
Vineyard

Cluster no. 109

```
## Number of genes in the cluster: 30
## Homogeneity Index:    0.68
## Variable importance for Stage:    Median =  176.3  - Rank =  94
## Variable importance for Clone:    Median =  12.68  - Rank =  86
## Variable importance for Vineyard: Median =  198.3  - Rank =  4
##
## Gene ID                Gene Annotation
## VIT_05s0077g01970  -  Zinc finger (C3HC4-type ring finger)
## VIT_09s0054g00530  -  Zinc finger (B-box type)
## VIT_10s0003g01730  -  PUB17 (plant U- box 17)
## VIT_12s0028g03270  -  Ethylene-responsive transcription factor 9
## VIT_10s0003g03810  -  Jasmonate ZIM domain-containing protein (VvJAZ7)
## VIT_07s0191g00230  -  Ring-H2 finger protein ATL5F
## VIT_00s0487g00010  -  Disease resistance protein (TIR-NBS-LRR class)
## VIT_07s0197g00240  -  R protein disease resistance protein
## VIT_08s0032g01150  -  Syntaxin 1B/2/3/4
## VIT_12s0134g00240  -  Avr9/Cf-9 rapidly elicited protein 20
## VIT_11s0052g01300  -  Xyloglucan endotransglycosylase 6
## VIT_06s0004g04180  -  Zinc finger (C2H2 type) protein (ZAT11)
## VIT_07s0005g02020  -  Sulfate adenylyltransferase
## VIT_06s0004g06840  -  Protein phosphatase 2C (VvPP2C-5)
## VIT_11s0052g01330  -  Xyloglucan endotransglucosylase/hydrolase 23
## VIT_11s0052g01190  -  Xyloglucan endotransglucosylase-hydrolase XTH3
## VIT_11s0052g01180  -  Xyloglucan endotransglucosylase/hydrolase 23
## VIT_01s0011g04660  -  Unknown protein
## VIT_01s0244g00100  -  Serine carboxypeptidase S10
## VIT_12s0028g03010  -  Glutaredoxin
## VIT_18s0001g03240  -  ERF/AP2 Gene Family (VvERF008),Dehydration Responsive Element-Binding
## VIT_07s0104g00120  -  Unknown protein
## VIT_18s0001g06150  -  Phosphate-induced protein 1
## VIT_03s0091g00690  -  Salt tolerance zinc finger
## VIT_01s0011g04420  -  Unknown protein
## VIT_17s0000g02100  -  Wall-associated kinase 2 (WAK2)
## VIT_09s0002g04580  -  Unknown protein
## VIT_00s1361g00010  -  Unknown
## VIT_05s0102g00360  -  On-specific serine/threonine protein kinase
## VIT_04s0023g01080  -  Lys Motif-Type Receptor-Like Kinase LYK4
```

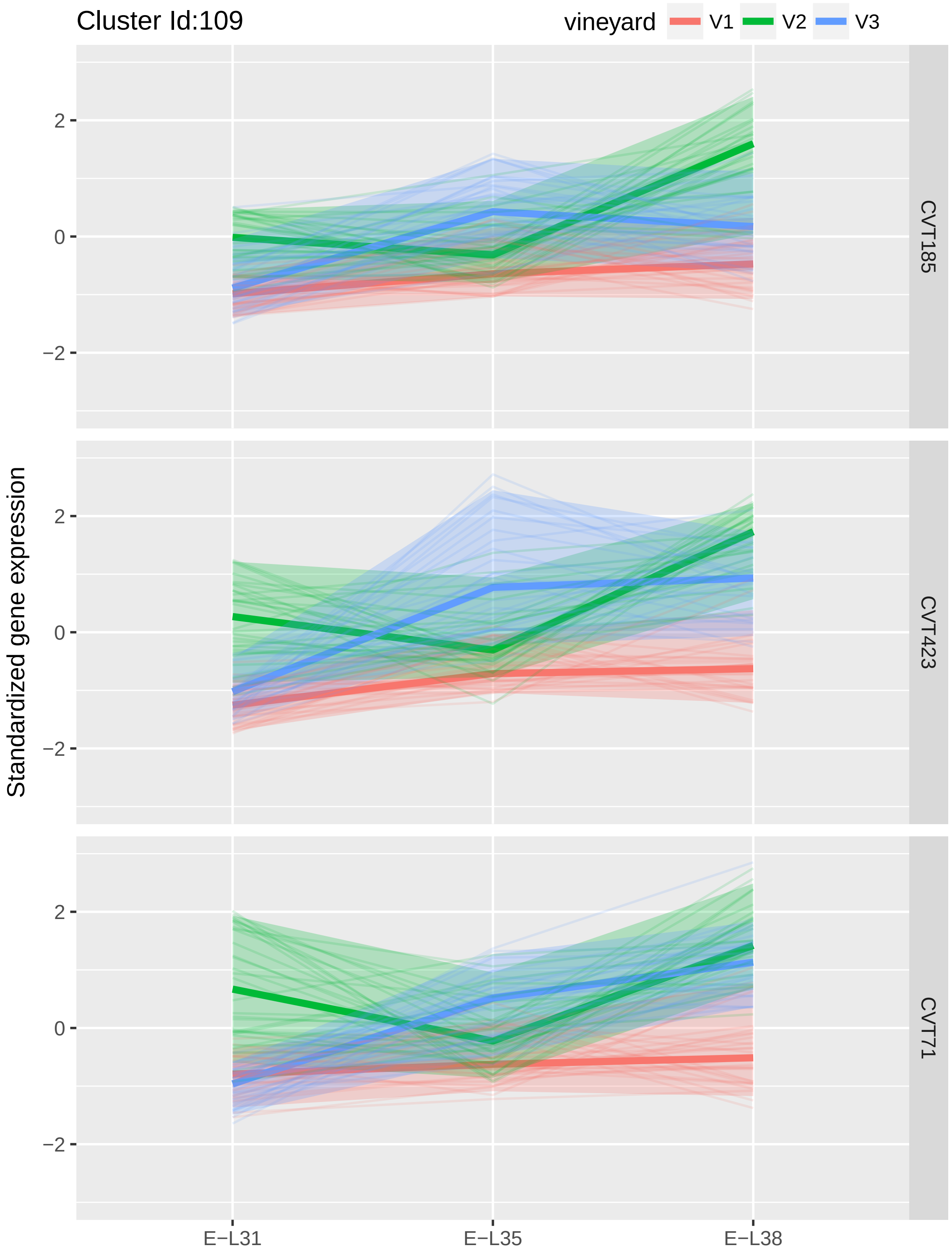
Cluster Id:109
vineyard
V1
V2
V3
Standardized gene expression
2
0
−2
CVT185
CVT423
CVT71
E−L31
E−L35
E−L38

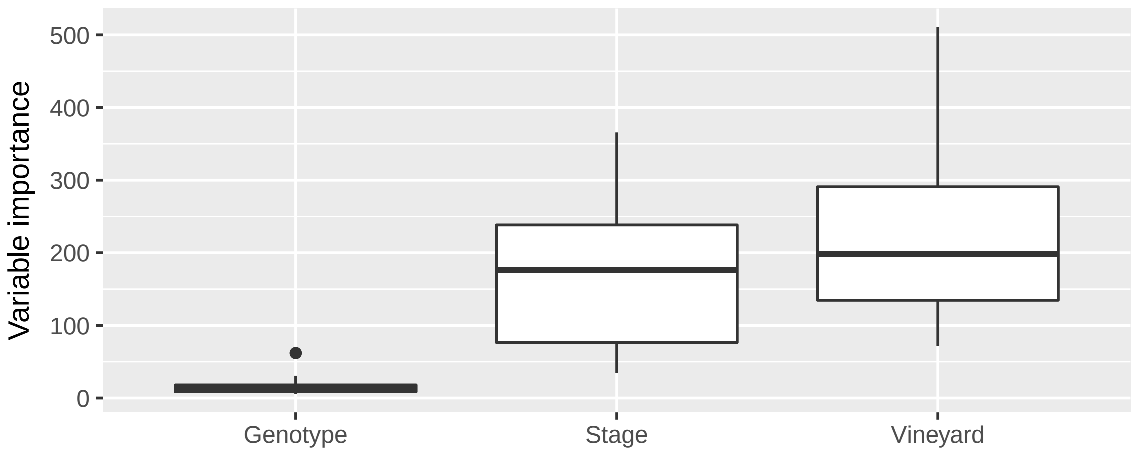
Variable importance
500
400
300
200
100
0
Genotype
Stage
Vineyard

Cluster no. 110

```
## Number of genes in the cluster: 89
## Homogeneity Index:    0.63
## Variable importance for Stage:    Median =  293.3  - Rank =  71
## Variable importance for Clone:    Median =  20.76  - Rank =  43
## Variable importance for Vineyard: Median =  80.16  - Rank =  22
##
## Gene ID               Gene Annotation
## VIT_13s0139g00290  -  R protein disease resistance protein
## VIT_00s0531g00060  -  Cellulose synthase CSLE1
## VIT_18s0001g08600  -  basic helix-loop-helix (bHLH) family
## VIT_09s0002g05920  -  R protein MLA10
## VIT_09s0070g00350  -  R protein disease resistance protein
## VIT_18s0001g01140  -  Peroxidase 64
## VIT_08s0007g08270  -  Pentatricopeptide (PPR) repeat
## VIT_13s0158g00210  -  RPM1 (resistance to p. syringae pv maculicola 1)
## VIT_11s0118g00280  -  Ubiquitin-specific protease 22 (UBP22)
## VIT_18s0001g15720  -  Leucine Rich Repeat receptor-like kinase
## VIT_12s0028g00400  -  1,3-beta-glucan synthase
## VIT_17s0000g08870  -  Carboxyl-terminal proteinase
## VIT_09s0002g03980  -  RPS5 (resistant to p. syringae 5)
## VIT_17s0000g08760  -  Receptor kinase TRKa
## VIT_02s0012g00840  -  Pentatricopeptide (PPR) repeat-containing protein
## VIT_19s0090g00940  -  RNASE THREE-like protein 2 (RTL2)
## VIT_12s0028g02110  -  Unknown protein
## VIT_05s0077g01760  -  Polygalacturonase GH28
## VIT_18s0001g08320  -  IMP dehydrogenase/GMP reductase
## VIT_05s0020g03310  -  Diamine oxidase
## VIT_09s0002g05000  -  R protein disease resistance protein
## VIT_01s0011g02190  -  Lectin
## VIT_19s0014g00900  -  Brassinosteroid Signaling positive regulator (BZR1)
## VIT_03s0038g02960  -  Pentatricopeptide (PPR) repeat-containing protein
## VIT_13s0158g00260  -  R protein MLA10
## VIT_18s0001g05250  -  Dehydration Responsive Element-Binding Transcription Factor (VvDREB27)
## VIT_18s0117g00070  -  R protein L6
## VIT_08s0007g01380  -  Rho guanyl-nucleotide exchange factor ROPGEF7
## VIT_05s0020g02270  -  Unknown
## VIT_09s0002g00240  -  R protein disease resistance protein
## VIT_13s0156g00630  -  S-receptor kinase
## VIT_01s0137g00740  -  Unknown protein
## VIT_18s0001g10680  -  Lipin, N-terminal conserved region family protein, expressed
## VIT_18s0117g00060  -  R protein L6
## VIT_18s0001g05840  -  RNA recognition motif (RRM)-containing protein
## VIT_17s0000g03490  -  Cysteine desulfurase, (NIFS)
## VIT_06s0009g00450  -  Aluminium-tolerance ALMT1
## VIT_08s0007g02380  -  Heat shock protein-related
## VIT_11s0103g00340  -  Pentatricopeptide (PPR) repeat-containing
## VIT_17s0119g00030  -  Guanine nucleotide-binding protein alpha-1 subunit
## VIT_06s0004g05970  -  Antiviral helicase SKI2
## VIT_14s0030g01730  -  No hit
## VIT_13s0139g00010  -  R protein MLA10
## VIT_05s0051g00450  -  GATA transcription factor 1
## VIT_10s0042g00460  -  RPS2 (resistant to p. syringae 2)
## VIT_16s0098g01750  -  Unknown protein
## VIT_14s0036g00540  -  Ribulose bisphosphate carboxylase, large chain
```

```
## VIT_00s1403g00010  -  Unknown
## VIT_13s0019g04890  -  Shugoshin-1
## VIT_14s0108g01260  -  Unknown protein
## VIT_18s0041g01620  -  R protein L6
## VIT_12s0034g01320  -  TIR-NBS-LRR disease resistance
## VIT_11s0078g00430  -  L-fucokinase
## VIT_13s0156g00500  -  R protein disease resistance protein
## VIT_13s0067g03000  -  Beta Galactosidase
## VIT_13s0019g00650  -  No hit
## VIT_09s0002g03300  -  disease resistance protein (NBS-LRR class)
## VIT_11s0052g00210  -  R protein disease resistance protein
## VIT_08s0040g01420  -  Bromodomain associated
## VIT_12s0028g03060  -  HSL1 (HAESA-like 1)
## VIT_19s0015g00300  -  KCO1 (two pore K channel)
## VIT_15s0046g00250  -  Transducin protein
## VIT_01s0127g00160  -  Cysteine-rich receptor-like protein kinase 2
## VIT_19s0090g00260  -  R protein disease resistance protein
## VIT_08s0007g02520  -  Unknown
## VIT_16s0050g02320  -  Regulator of nonsense transcripts 1
## VIT_18s0041g01630  -  No hit
## VIT_08s0007g08960  -  Ca2+-transporting ATPase type 2 isoform 4
## VIT_13s0156g00440  -  R protein disease resistance protein
## VIT_17s0000g04790  -  BIM1 (BES1-interacting Myc-like protein 1)
## VIT_11s0052g00200  -  R protein  PRF  disease resistance protein
## VIT_14s0108g00430  -  ABC Transporter (VvMDR4 - VvABCB4)
## VIT_10s0003g04490  -  Phototropic-responsive NPH3
## VIT_00s2390g00010  -  R protein MLA10
## VIT_13s0019g03370  -  Myb family
## VIT_18s0001g07380  -  DNA-binding protein
## VIT_01s0011g01850  -  Cytochrome c6 (ATC6)
## VIT_01s0011g00130  -  Squamosa promoter-binding protein (VvSBP1)
## VIT_15s0046g03590  -  (+)-neomenthol dehydrogenase
## VIT_07s0005g02810  -  2Fe-2S-binding
## VIT_09s0018g01920  -  Exocyst complex component EXO84 B
## VIT_16s0050g02490  -  Tetrahydrofolylpolyglutamate synthase
## VIT_14s0171g00450  -  DNA-binding protein GT-1-related
## VIT_13s0047g00940  -  Cinnamyl alcohol dehydrogenase
## VIT_14s0083g00340  -  Beta-glucan-binding protein 4
## VIT_13s0064g01850  -  R protein MLA10
## VIT_14s0068g01530  -  Zinc finger (DHHC type)
## VIT_18s0089g00590  -  Glyceraldehyde-3-phosphate dehydrogenase, cytosolic
## VIT_17s0000g00120  -  Exostosin
```

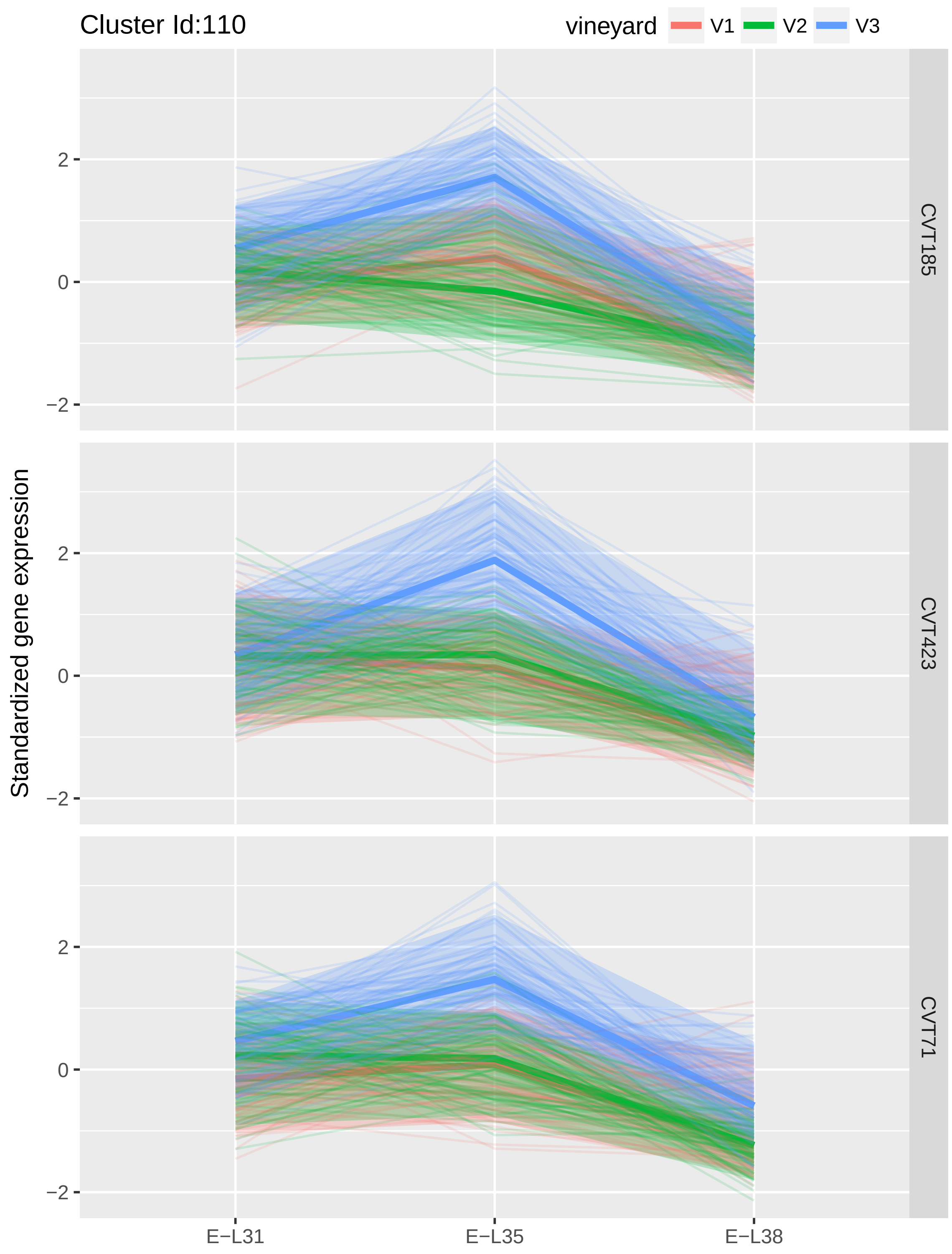

Cluster Id:110
vineyard
V1
V2
V3
CVT185
CVT423
CVT71
Standardized gene expression
2
0
−2
E−L31
E−L35
E−L38

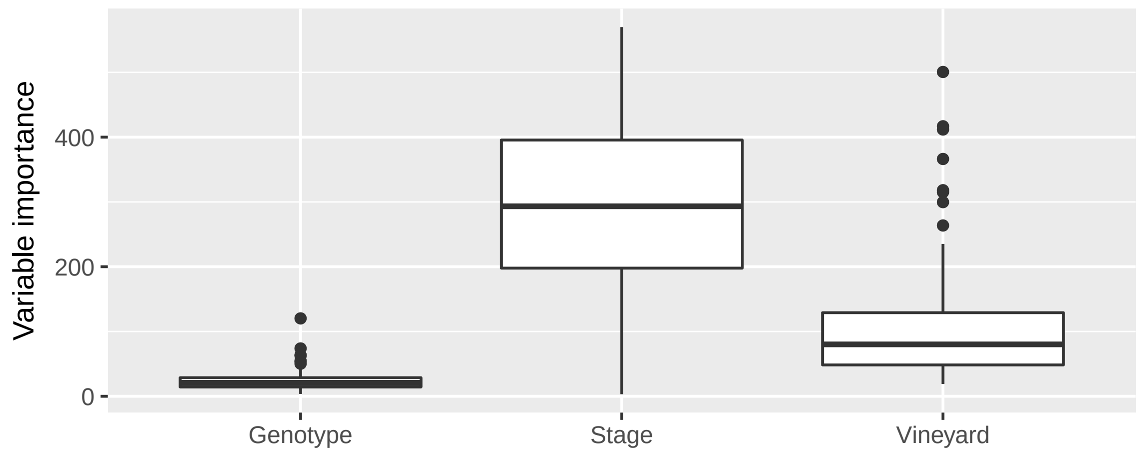

Variable importance
400
200
0
Genotype
Stage
Vineyard

Cluster no. 111

```
## Number of genes in the cluster: 65
## Homogeneity Index:    0.65
## Variable importance for Stage:    Median =  72.64  - Rank =  107
## Variable importance for Clone:    Median =  22.17  - Rank =  37
## Variable importance for Vineyard: Median =  157.7  - Rank =  7
##
## Gene ID                Gene Annotation
## VIT_08s0056g00290  -  Calcium-binding protein CML
## VIT_03s0038g03530  -  LYS/HIS transporter 7
## VIT_18s0122g01260  -  Protein kinase 1B
## VIT_10s0003g01650  -  Unknown
## VIT_03s0097g00710  -  Glutamyl-tRNA reductase 1
## VIT_17s0000g03340  -  Wall-associated kinase 4
## VIT_05s0094g00160  -  No hit
## VIT_13s0067g00930  -  ACR4 (Arabidopsis CRINKLY4)
## VIT_19s0027g00020  -  WAG1
## VIT_09s0054g00910  -  Chaperone BCS1 mitochondrial
## VIT_10s0116g01200  -  WRKY Transcription Factor (VvWRKY29)
## VIT_03s0017g01400  -  Adhesive/proline-rich protein
## VIT_07s0005g02110  -  Protein phosphatase 2C DBP
## VIT_03s0017g01450  -  No hit
## VIT_16s0039g01870  -  Protein kinase
## VIT_01s0010g02450  -  B12D
## VIT_02s0025g00420  -  WRKY Transcription Factor (VvWRKY04)
## VIT_19s0014g01360  -  Curculin (mannose-binding) lectin
## VIT_18s0001g02020  -  Amino acid permease 3
## VIT_01s0011g00380  -  SP1L1 (SPIRAL1-like1)
## VIT_12s0059g00370  -  EF hand
## VIT_05s0020g03680  -  GCN5 N-acetyltransferase (GNAT)
## VIT_01s0011g06600  -  Glutamate decarboxylase
## VIT_10s0042g00920  -  Stilbene synthase (VvSTS5)
## VIT_06s0009g01810  -  APK2A (protein kinase 2A)
## VIT_10s0042g00890  -  Stilbene Synthase (VvSTS3)
## VIT_12s0059g00310  -  Unknown
## VIT_03s0091g00810  -  Beta-1,4-xylosidase
## VIT_06s0009g03680  -  F-box family protein
## VIT_13s0156g00150  -  Unknown
## VIT_05s0077g01690  -  s1_Pathogenesis protein 10 [Vitis vinifera]
## VIT_10s0003g05430  -  Reticuline oxidase precursor
## VIT_04s0044g00040  -  Dirigent
## VIT_05s0077g00500  -  myb domain protein 108
## VIT_06s0004g01670  -  UDP-glucoronosyl and UDP-glucosyl transferase
## VIT_19s0014g01370  -  Curculin (mannose-binding) lectin
## VIT_07s0031g01070  -  Ascorbate oxidase
## VIT_03s0091g00160  -  NtPRp27 secretory protein
## VIT_18s0041g02010  -  12-oxophytodienoate reductase 1
## VIT_04s0044g01120  -  Alcohol dehydrogenase 2 [Vitis vinifera]
## VIT_05s0049g00730  -  No hit
## VIT_04s0008g00180  -  NADP-dependent malic enzyme
## VIT_19s0014g02120  -  Metallothionein
## VIT_05s0020g03710  -  GCN5 N-acetyltransferase (GNAT)
## VIT_03s0017g01280  -  ABC Transporter (VvWBC12 - VvABCG12)
## VIT_12s0059g00350  -  EF hand
## VIT_10s0003g05450  -  Reticuline oxidase precursor
```

```
## VIT_04s0008g05970  -  Unknown protein
## VIT_06s0004g04150  -  Auxin-regulated protein
## VIT_16s0050g02510  -  WRKY Transcription Factor (VvWRKY51)
## VIT_08s0058g01160  -  No hit
## VIT_18s0001g08820  -  Unknown protein
## VIT_12s0059g01580  -  Unknown
## VIT_07s0005g01360  -  Zinc finger (C3HC4-type ring finger)
## VIT_11s0103g00490  -  Unknown protein
## VIT_05s0049g00720  -  No hit
## VIT_10s0003g02810  -  WRKY Transcription Factor (VvWRKY31)
## VIT_07s0005g03220  -  ERF/AP2 Gene Family (VvERF098)
## VIT_01s0011g03650  -  Map kinase substrate 1 MKS1
## VIT_14s0060g01180  -  KNAT2 (knotted1-like homeobox gene 6)
## VIT_05s0020g03690  -  GCN5 N-acetyltransferase (GNAT)
## VIT_06s0004g01570  -  No hit
## VIT_08s0058g00150  -  Ammonium transporter 2
## VIT_05s0049g00800  -  Unknown protein
## VIT_17s0119g00120  -  Alpha-amylase/subtilisin inhibitor
```

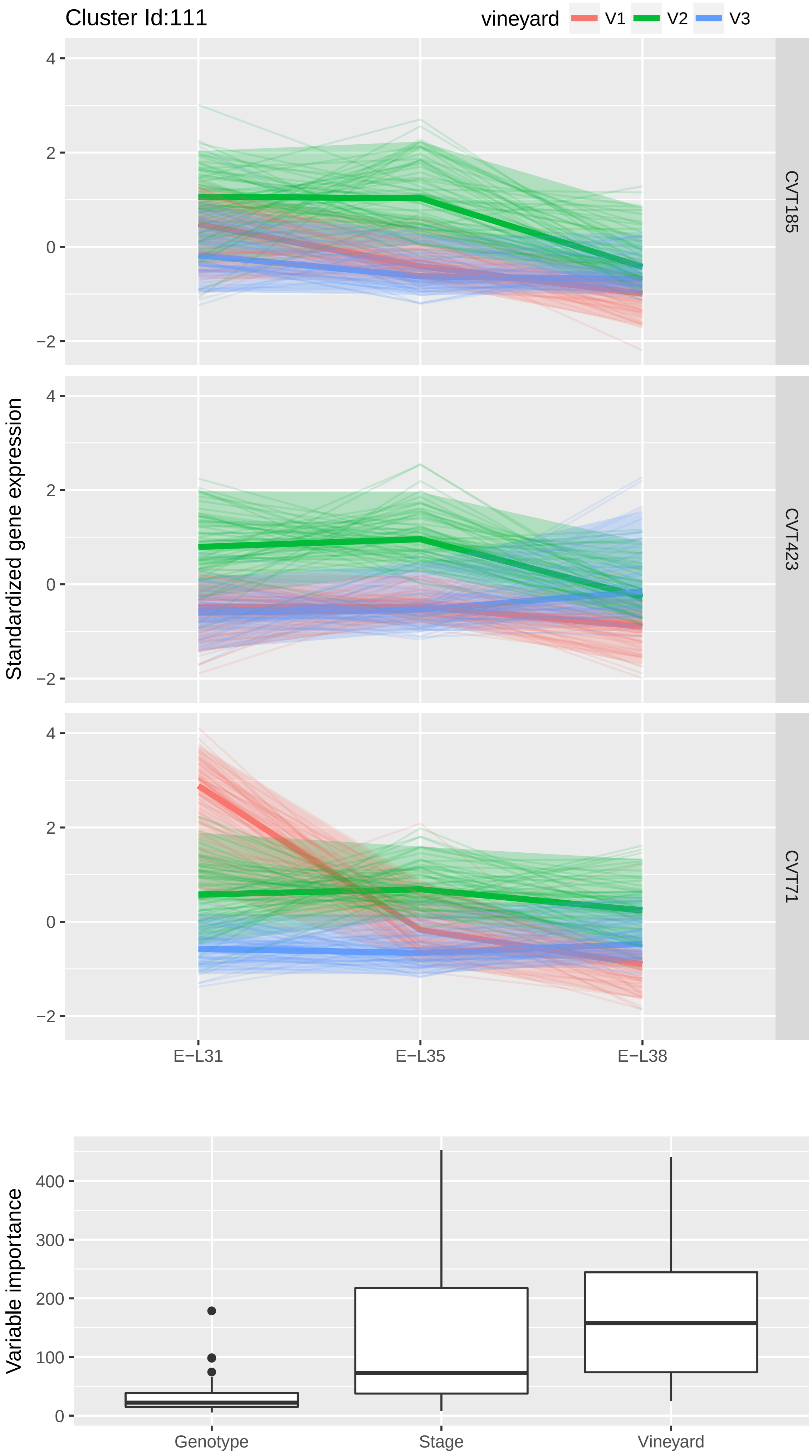
Cluster Id:111
vineyard
V1
V2
V3
CVT185
CVT423
CVT71
Standardized gene expression
E−L31
E−L35
E−L38
Variable importance
Genotype
Stage
Vineyard

Cluster no. 112

```
## Number of genes in the cluster: 36
## Homogeneity Index:    0.56
## Variable importance for Stage:    Median =  43.41  - Rank =  110
## Variable importance for Clone:    Median =  30.47  - Rank =  8
## Variable importance for Vineyard: Median =  139.2  - Rank =  9
##
## Gene ID                Gene Annotation
## VIT_01s0127g00230  -  NIK3 (NSP- interacting  kinase 3)
## VIT_01s0011g06260  -  Anthranilate synthase beta subunit
## VIT_10s0092g00200  -  Drug/metabolite transporter DMT family transporter
## VIT_05s0029g00400  -  Kinesin PAKRP1L
## VIT_14s0066g00640  -  Pescadillo
## VIT_01s0011g00490  -  Pentatricopeptide (PPR) repeat-containing protein
## VIT_12s0034g01220  -  Unknown protein
## VIT_16s0050g01050  -  Unknown protein
## VIT_06s0004g05540  -  Metal tolerance protein B MTPB1
## VIT_04s0008g05050  -  Pentatricopeptide (PPR) repeat-containing protein
## VIT_16s0039g00280  -  Unknown protein
## VIT_06s0004g03710  -  PRLI-interacting factor G
## VIT_05s0049g01850  -  Unknown protein
## VIT_15s0046g02270  -  Unknown protein
## VIT_19s0027g00740  -  Inhibitor of apoptosis
## VIT_12s0059g01130  -  CERK1 (chitin elicitor receptor kinase 1)
## VIT_11s0016g03690  -  Peroxisomal fatty acid beta-oxidation multifunctional protein (aim1)
## VIT_08s0007g08690  -  Villin-3
## VIT_12s0059g02000  -  Glycosyl transferase family 14 protein
## VIT_15s0046g01890  -  WD40
## VIT_09s0002g00950  -  Nucleoside diphosphate kinase family protein
## VIT_11s0016g03280  -  Protein phosphatases PP1 regulatory subunit SDS22
## VIT_10s0003g01180  -  Ketol-acid reductoisomerase
## VIT_05s0020g01460  -  Protein phosphatase 2C
## VIT_17s0000g03190  -  Glycosyl transferase family 28 protein
## VIT_03s0091g01190  -  Magnesium transporter CorA
## VIT_17s0000g08360  -  Ataxia-telangiectasia mutated protein (Atm)
## VIT_04s0023g03160  -  Unknown protein
## VIT_01s0011g01470  -  far-red impaired response 1
## VIT_03s0180g00020  -  Phosphoric monoester hydrolase
## VIT_10s0003g00520  -  Unknown protein
## VIT_03s0063g01980  -  PDV2 (plastid division2)
## VIT_18s0001g13430  -  Coatomer alpha subunit
## VIT_02s0087g00420  -  Zinc finger (C3HC4-type ring finger)
## VIT_10s0003g01190  -  Ketol-acid reductoisomerase
## VIT_08s0007g01680  -  Ceramidase
```

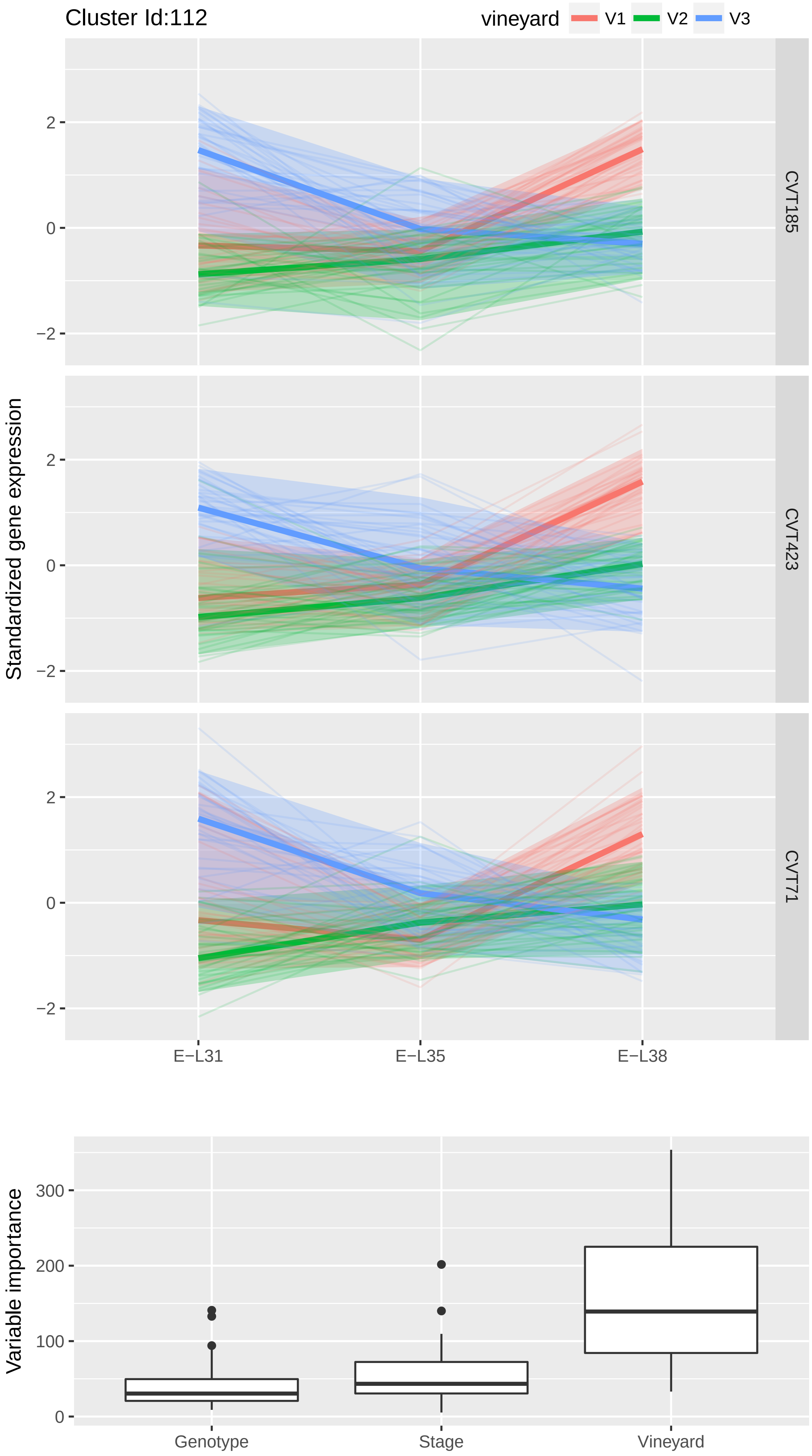

Cluster Id:112
vineyard
V1
V2
V3
Standardized gene expression
CVT185
CVT423
CVT71
E−L31
E−L35
E−L38
Variable importance
Genotype
Stage
Vineyard
